# Child and Adolescent Health From 1990 to 2015: Findings From the Global Burden of Diseases, Injuries, and Risk Factors 2015 Study

**DOI:** 10.1001/jamapediatrics.2017.0250

**Published:** 2017-04-03

**Authors:** The Global Burden of Disease Child and Adolescent Health Collaboration, Nicholas Kassebaum, Hmwe Hmwe Kyu, Leo Zoeckler, Helen Elizabeth Olsen, Katie Thomas, Christine Pinho, Zulfiqar A. Bhutta, Lalit Dandona, Alize Ferrari, Tsegaye Tewelde Ghiwot, Simon I. Hay, Yohannes Kinfu, Xiaofeng Liang, Alan Lopez, Deborah Carvalho Malta, Ali H. Mokdad, Mohsen Naghavi, George C. Patton, Joshua Salomon, Benn Sartorius, Roman Topor-Madry, Stein Emil Vollset, Andrea Werdecker, Harvey A. Whiteford, Kalkidan Hasen Abate, Kaja Abbas, Solomon Abrha Damtew, Muktar Beshir Ahmed, Nadia Akseer, Rajaa Al-Raddadi, Mulubirhan Assefa Alemayohu, Khalid Altirkawi, Amanuel Alemu Abajobir, Azmeraw T. Amare, Carl A. T. Antonio, Johan Arnlov, Al Artaman, Hamid Asayesh, Euripide Frinel G. Arthur Avokpaho, Ashish Awasthi, Beatriz Paulina Ayala Quintanilla, Umar Bacha, Balem Demtsu Betsu, Aleksandra Barac, Till Winfried Bärnighausen, Estifanos Baye, Neeraj Bedi, Isabela M. Bensenor, Adugnaw Berhane, Eduardo Bernabe, Oscar Alberto Bernal, Addisu Shunu Beyene, Sibhatu Biadgilign, Boris Bikbov, Cheryl Anne Boyce, Alexandra Brazinova, Gessessew Bugssa Hailu, Austin Carter, Carlos A. Castañeda-Orjuela, Ferrán Catalá-López, Fiona J. Charlson, Abdulaal A. Chitheer, Jee-Young Jasmine Choi, Liliana G. Ciobanu, John Crump, Rakhi Dandona, Robert P. Dellavalle, Amare Deribew, Gabrielle deVeber, Daniel Dicker, Eric L. Ding, Manisha Dubey, Amanuel Yesuf Endries, Holly E. Erskine, Emerito Jose Aquino Faraon, Andre Faro, Farshad Farzadfar, Joao C. Fernandes, Daniel Obadare Fijabi, Christina Fitzmaurice, Thomas D. Fleming, Luisa Sorio Flor, Kyle J. Foreman, Richard C. Franklin, Maya S. Fraser, Joseph J. Frostad, Nancy Fullman, Gebremedhin Berhe Gebregergs, Alemseged Aregay Gebru, Johanna M. Geleijnse, Katherine B. Gibney, Mahari Gidey Yihdego, Ibrahim Abdelmageem Mohamed Ginawi, Melkamu Dedefo Gishu, Tessema Assefa Gizachew, Elizabeth Glaser, Audra L. Gold, Ellen Goldberg, Philimon Gona, Atsushi Goto, Harish Chander Gugnani, Guohong Jiang, Rajeev Gupta, Fisaha Haile Tesfay, Graeme J. Hankey, Rasmus Havmoeller, Martha Hijar, Masako Horino, H. Dean Hosgood, Guoqing Hu, Kathryn H. Jacobsen, Mihajlo B. Jakovljevic, Sudha P. Jayaraman, Vivekanand Jha, Tariku Jibat, Catherine O. Johnson, Jost Jonas, Amir Kasaeian, Norito Kawakami, Peter N. Keiyoro, Ibrahim Khalil, Young-Ho Khang, Jagdish Khubchandani, Aliasghar A. Ahmad Kiadaliri, Christian Kieling, Daniel Kim, Niranjan Kissoon, Luke D. Knibbs, Ai Koyanagi, Kristopher J. Krohn, Barthelemy Kuate Defo, Burcu Kucuk Bicer, Rachel Kulikoff, G. Anil Kumar, Dharmesh Kumar Lal, Hilton Y. Lam, Heidi J. Larson, Anders Larsson, Dennis Odai Laryea, Janni Leung, Stephen S. Lim, Loon-Tzian Lo, Warren D. Lo, Katharine J. Looker, Paulo A. Lotufo, Hassan Magdy Abd El Razek, Reza Malekzadeh, Desalegn Markos Shifti, Mohsen Mazidi, Peter A. Meaney, Kidanu Gebremariam Meles, Peter Memiah, Walter Mendoza, Mubarek Abera Mengistie, Gebremichael Welday Mengistu, George A. Mensah, Ted R. Miller, Charles Mock, Alireza Mohammadi, Shafiu Mohammed, Lorenzo Monasta, Ulrich Mueller, Chie Nagata, Aliya Naheed, Grant Nguyen, Quyen Le Nguyen, Elaine Nsoesie, In-Hwan Oh, Anselm Okoro, Jacob Olusegun Olusanya, Bolajoko O. Olusanya, Alberto Ortiz, Deepak Paudel, David M. Pereira, Norberto Perico, Max Petzold, Michael Robert Phillips, Guilherme V. Polanczyk, Farshad Pourmalek, Mostafa Qorbani, Anwar Rafay, Vafa Rahimi-Movaghar, Mahfuzar Rahman, Rajesh Kumar Rai, Usha Ram, Zane Rankin, Giuseppe Remuzzi, Andre M. N. Renzaho, Hirbo Shore Roba, David Rojas-Rueda, Luca Ronfani, Rajesh Sagar, Juan Ramon Sanabria, Muktar Sano Kedir Mohammed, Itamar S. Santos, Maheswar Satpathy, Monika Sawhney, Ben Schöttker, David C. Schwebel, James G. Scott, Sadaf G. Sepanlou, Amira Shaheen, Masood Ali Shaikh, June She, Rahman Shiri, Ivy Shiue, Inga Dora Sigfusdottir, Jasvinder Singh, Naris Silpakit, Alison Smith, Chandrashekhar Sreeramareddy, Jeffrey D. Stanaway, Dan J. Stein, Caitlyn Steiner, Muawiyyah Babale Sufiyan, Soumya Swaminathan, Rafael Tabarés-Seisdedos, Karen M. Tabb, Fentaw Tadese, Mohammad Tavakkoli, Bineyam Taye, Stephanie Teeple, Teketo Kassaw Tegegne, Girma Temam Shifa, Abdullah Sulieman Terkawi, Bernadette Thomas, Alan J. Thomson, Ruoyan Tobe-Gai, Marcello Tonelli, Bach Xuan Tran, Christopher Troeger, Kingsley N. Ukwaja, Olalekan Uthman, Tommi Vasankari, Narayanaswamy Venketasubramanian, Vasiliy Victorovich Vlassov, Elisabete Weiderpass, Robert Weintraub, Solomon Weldemariam Gebrehiwot, Ronny Westerman, Hywel C. Williams, Charles D. A. Wolfe, Rachel Woodbrook, Yuichiro Yano, Naohiro Yonemoto, Seok-Jun Yoon, Mustafa Z. Younis, Chuanhua Yu, Maysaa El Sayed Zaki, Elias Asfaw Zegeye, Liesl Joanna Zuhlke, Christopher J. L. Murray, Theo Vos

**Affiliations:** 1 Institute for Health Metrics and Evaluation, University of Washington, Seattle; 2 Centre of Excellence in Women and Child Health, Aga Khan University, Karachi, Pakistan; 3 Public Health Foundation of India, Gurgaon–122002, National Capital Region, India; 4 School of Public Health, University of Queensland, Brisbane, Queensland, Australia; 5 Jimma University, Jimma, Ethiopia; 6 Oxford Big Data Institute, Li Ka Shing Centre for Health Information and Discovery, University of Oxford, Oxford, United Kingdom; 7 Centre for Research & Action in Public Health, University of Canberra, Canberra, Australia; 8 Chinese Center for Disease Control and Prevention, Beijing, China; 9 Melbourne School of Population and Global Health, University of Melbourne, Melbourne, Victoria, Australia; 10 Universidade Federal de Minas Gerais, Belo Horizonte, Brazil; 11 Murdoch Childrens Research Institute, University of Melbourne, Victoria, Australia; 12 Harvard T. H. Chan School of Public Health, Harvard University, Boston, Massachusetts; 13 School of Nursing and Public Health, University of KwaZulu-Natal, South African Medical Research Council/University of KwaZulu-Natal Gastrointestinal Cancer Research Center, Durban, South Africa; 14 Institute of Public Health, Faculty of Health Sciences, Jagiellonian University Medical College, Kraków, Poland; 15 Center for Disease Burden, Norwegian Institute of Public Health, Bergen, Norway; 16 Federal Institute for Population Research, Wiesbaden, Germany; 17 Department of Population Health, Virginia Tech, Blacksburg; 18 Wolaita Soda University, Wolaita Soda, Ethiopia; 19 The Hospital for Sick Children, Centre for Child Health, Toronto, Ontario, Canada; 20 Ministry of Health, Jeddah, Saudi Arabia; 21 Mekelle University, Mekelle, Ethiopia; 22 King Saud University, Riyadh, Saudi Arabia; 23 University of Adelaide, Adelaide, Australia; 24 Department of Health Policy and Administration, University of Philippines–Manila, Manila, Philippines; 25 Department of Medical Services, Uppsala University, Uppsala, Sweden; 26 Dalarna University, Uppsala, Sweden; 27 University of Manitoba, Winnipeg, Manitoba, Canada; 28 Qom University of Medical Sciences, Qom, Iran; 29 Institute de Recherche Clinique du Bénin, Cotonou, Benin; 30 Sanjay Gandhi Postgraduate Institute of Medical Sciences, Lucknow, India; 31 The Judith Lumley Centre for Mother, Infant, and Family Health Research, La Trobe University, Melbourne, Victoria, Australia; 32 School of Health Sciences, University of Management and Technology, Lahore, Pakistan; 33 Faculty of Medicine, University of Belgrade, Belgrade, Serbia; 34 Monash University, Melbourne, Victoria, Australia; 35 College of Public Health and Tropical Medicine, Jazan, Saudi Arabia; 36 University of Sao Paulo, Sao Paulo, Brazil; 37 College of Health Sciences, Debre Berhan University, Debre Berhan, Ethiopia; 38 King’s College London, London, United Kingdom; 39 University Andes, Bogota, Columbia; 40 Haramaya University, Dire Dawa, Ethiopia; 41 Independent Public Health Consultants, Addis Ababa, Ethiopia; 42 Department of Nephrology Issues of Transplanted Kidney, V. I. Shumakov Federal Research Center of Transplantology and Artificial Organs, Moscow, Russia; 43 National Heart, Lung, and Blood Institute, National Institutes of Health, Bethesda, Maryland; 44 Faculty of Health Sciences and Social Work, Department of Public Health, Trnava University, Trnava, Slovakia; 45 Instituto Nacional de Salud, Bogotá, Colombia; 46 University of Valencia, Valencia, Spain; 47 Health Research Institute and CIBERSAM, Valencia, Spain; 48 Ministry of Health, Baghdad, Iraq; 49 Seoul National University, Seoul, South Korea; 50 Departmentà Centre for International Health, University of Otago, Dunedin, New Zealand; 51 Public Health Foundation of India, New Delhi, India; 52 School of Medicine, School of Public Health, University of Colorado, Aurora; 53 Nuffield Department of Medicine, University of Oxford, Oxford, United Kingdom; 54 International Institute for Population Sciences, Mumbai, India; 55 Arba Minch University, Arba Minch, Ethiopia; 56 Queensland Centre for Mental Health Research, Brisbane, Queensland, Australia; 57 Federal University of Sergipe, Aracaju, Brazil; 58 Non-Communicable Diseases Research Center, Tehran University of Medical Sciences, Tehran, Iran; 59 Center for Biotechnology and Fine Chemistry, Catholic University of Portugal, Porto, Portugal; 60 Heller School for Social Policy and Management, Brandeis University, Waltham, Massachusetts; 61 Escola Nacional de Saúde Pública Sergio Arouca/Fiocruz, Rio De Janeiro, Brazil; 62 James Cook University, Townsville, Queensland, Australia; 63 Wageningen University, Wageningen, Netherlands; 64 The Peter Doherty Institute for Infection and Immunity, University of Melbourne, Melbourne, Victoria, Australia; 65 Addis Ababa University, Addis Ababa, Ethiopia; 66 Department of Public Health, Mizan-Tepi University, Ethiopia; 67 College of Medicine, University of Hail, Hail, Saudi Arabia; 68 University of Massachusetts–Boston; 69 National Cancer Center, Tokyo, Japan; 70 Department of Microbiology, Departments of Epidemiology and Biostatistics, St James School of Medicine, the Quarter, Anguilla; 71 School of Public Health, Tianjin Medical University, Tianjin, China; 72 Eternal Heart Care Centre and Research Institute, Jaipur, India; 73 School of Medicine and Pharmacology, University of Western Australia, Perth, Australia; 74 Karolinska Institutet, Stockholm, Sweden; 75 Fundacion Entornos, Cuernavaca, Morelos, Mexico; 76 Nevada Division of Public and Behavioral Health, Carson City, Nevada; 77 Albert Einstein College of Medicine, Bronx, New York; 78 Department of Epidemiology and Health Statistics, School of Public Health, Central South University, Changsha, Hunan, China; 79 Department of Global and Community Health, George Mason University, Fairfax, Virginia; 80 University of Kragujevac, Kragujevac, Serbia; 81 Virginia Commonwealth University, Richmond; 82 George Institute for Global Health, New Delhi, India; 83 University of Oxford, Oxford, United Kingdom; 84 Department of Ophthalmology, Medical Faculty Mannheim, Ruprecht-Karlas University, Heidelberg, Germany; 85 School of Public Health, University of Tokyo, Tokyo, Japan; 86 University of Nairobi, Nairobi, Kenya; 87 Ball State University, Muncie, Indiana; 88 Department of Clinical Sciences, Lund University, Lund, Sweden; 89 Federal University of Rio Grande de Sul, Porto Alegre, Brazil; 90 Hospital de Clinicas de Porto Alegre, Porto Alegre, Brazil; 91 Department of Health Sciences, Northeastern University, Boston, Massachusetts; 92 University of British Columbia, Vancouver, British Columbia, Canada; 93 Research and Development Unit, Parc Sanitari Sant Joan de Deu, Barcelona, Spain; 94 University of Montreal, Montreal, Quebec, Canada; 95 Institute of Public Health, Hacettepe University, Ankara, Turkey; 96 Institute of Health Policy and Development Studies, National Institutes of Health, Manila, Philippines; 97 Department of Infectious Disease Epidemiology, London School of Hygiene and Tropical Medicine, London, United Kingdom; 98 Komfo Anokye Teaching Hospital, Kumasi, Ghana; 99 UnionHealth Associates LLC, St Louis, Missouri; 100 Alton Mental Health Center, Alton, Illinois; 101 Department of Pediatrics, Department of Neurology, The Ohio State University, Columbus; 102 University of Bristol, Bristol, United Kingdom; 103 Faculty of Medicine, Mansoura University, Mansoura, Egypt; 104 Madda Walabu University, Robe, Ethiopia; 105 Institute of Genetics and Developmental Biology, Key State Laboratory of Molecular Developmental Biology, Chinese Academy of Sciences, Beijing, China; 106 Perelman School of Medicine, University of Pennsylvania, Philadelphia; 107 University of West Florida, Pensacola; 108 United Nations Population Fund, Lima, Peru; 109 Pacific Institute for Research and Evaluation, Calverton, Maryland; 110 School of Medicine, School of Global Health, University of Washington, Seattle; 111 Baqiyatallah University of Medical Sciences, Tehran, Iran; 112 Ahmadu Bello University, Zaria, Kaduna, Nigeria; 113 Institute for Maternal and Child Health IRCCS Burlo Garofolo, Trieste, Italy; 114 National Center for Child Health and Development, Tokyo, Japan; 115 International Centre for Diarrheal Disease Research, Dhaka, Bangladesh; 116 Institute for Global Health, Duy Tan University, Da Nang, Vietnam; 117 Department of Preventive Medicine, College of Medicine, Kyung Hee University, Seoul, South Korea; 118 Society for Family Health, Abuja, Nigeria; 119 Center for Healthy Start Initiative, Lagos, Nigeria; 120 IIS-Fundacion Jimenez Diaz-UAM, Madrid, Spain; 121 UK Department for International Development, Lalitpur, Nepal; 122 Universidade do Porto, Porto, Portugal; 123 Istituto di Richerche Farmacologiche Mario Negri, Bergamo, Italy; 124 Health Metrics Unit, University of Gothenburg, Gothenburg, Sweden; 125 School of Medicine, Shanghai Jiao Tong University, Shanghai, China; 126 School of Medicine, Alborz University of Medical Sciences, Karaj, Iran; 127 Contect International Health Consultants, Lahore, Punjab, Pakistan; 128 Research and Evaluation Division, Building Resources Access Communities, Dhaka, Bangladesh; 129 Society for Health and Demographic Surveillance, Suri, India; 130 International Society of Nephrology, Brussels, Belgium; 131 Western Sydney University, Penrith, Australia; 132 ISGlobal Instituto de Salud Global de Barcelona, Barcelona, Spain; 133 All India Institute of Medical Sciences, New Delhi, India; 134 Marshall University, Huntington, West Virginia; 135 Division of Clinical Epidemiology and Aging Research, German Cancer Research Center, Heidelberg, Germany; 136 Institute of Health Care and Social Sciences, FOM University, Essen, Germany; 137 University of Alabama at Birmingham; 138 Centre for Clinical Research, University of Queensland, Brisbane, Queensland, Australia; 139 Department of Public Health, An-Najah University, Nablus, Palestine; 140 Independent Consultant, Karachi, Pakistan; 141 Department of Pulmonary Medicine, Zhongshan Hospital, Fudan University, Shanghai, China; 142 Finnish Institute of Occupational Health, Work Organizations, Disability Program, University of Helsinki, Helsinki, Finland; 143 Faculty of Health and Life Sciences, Northumbria University, Newcastle Upon Tyne, United Kingdom; 144 Reykjavik University, Reykjavik, Iceland; 145 Department of Community Medicine, International Medical University, Kuala Lumpur, Selangor, Malaysia; 146 Department of Psychiatry, University of Cape Town, Cape Town, South Africa; 147 Indian Council of Medical Research, Chennai, India; 148 University of Illinois at Urbana-Champaign, Champaign; 149 Debre Markos University, Debre Markos, Ethiopia; 150 New York Medical Center, Valhalla; 151 Department of Biology, Colgate University, Hamilton, New York; 152 University of Virginia, Charlottesville; 153 Adaptive Knowledge Management, Victoria, British Columbia, Canada; 154 University of Calgary, Calgary, Alberta, Canada; 155 The Johns Hopkins University, Baltimore, Maryland; 156 Federal Teaching Hospital, Abakaliki, Nigeria; 157 University of Warwick, Coventry, United Kingdom; 158 Institute for Health Promotion Research, Tampere, Finland; 159 Raffles Neuroscience Centre, Raffles Hospital, Singapore, Singapore; 160 National Research University Higher School of Economics, Moscow, Russia; 161 Department of Medical Epidemiology and Biostatistics, Karolinska Insitutet, Stockholm, Sweden; 162 Institute of Population-based Cancer Research, Cancer Registry of Norway, Oslo, Norway; 163 Royal Children’s Hospital, Melbourne, Victoria, Australia; 164 University of Nottingham, Nottingham, United Kingdom; 165 Department of Preventive Medicine, Northwestern University, Chicago, Illinois; 166 Kyoto University, Kyoto, Japan; 167 Department of Preventive Medicine, School of Medicine, Korea University, Seoul, South Korea; 168 Jackson State University, Jackson, Missouri; 169 Wuhan University, Wuhan, China; 170 University of KwaZulu-Natal, Durban, South Africa; 171 Red Cross War Memorial Children’s Hospital, Cape Town, South Africa

## Abstract

**Importance:**

Comprehensive and timely monitoring of disease burden in all age groups, including children and adolescents, is essential for improving population health.

**Objective:**

To quantify and describe levels and trends of mortality and nonfatal health outcomes among children and adolescents from 1990 to 2015 to provide a framework for policy discussion.

**Evidence Review:**

Cause-specific mortality and nonfatal health outcomes were analyzed for 195 countries and territories by age group, sex, and year from 1990 to 2015 using standardized approaches for data processing and statistical modeling, with subsequent analysis of the findings to describe levels and trends across geography and time among children and adolescents 19 years or younger. A composite indicator of income, education, and fertility was developed (Socio-demographic Index [SDI]) for each geographic unit and year, which evaluates the historical association between SDI and health loss.

**Findings:**

Global child and adolescent mortality decreased from 14.18 million (95% uncertainty interval [UI], 14.09 million to 14.28 million) deaths in 1990 to 7.26 million (95% UI, 7.14 million to 7.39 million) deaths in 2015, but progress has been unevenly distributed. Countries with a lower SDI had a larger proportion of mortality burden (75%) in 2015 than was the case in 1990 (61%). Most deaths in 2015 occurred in South Asia and sub-Saharan Africa. Global trends were driven by reductions in mortality owing to infectious, nutritional, and neonatal disorders, which in the aggregate led to a relative increase in the importance of noncommunicable diseases and injuries in explaining global disease burden. The absolute burden of disability in children and adolescents increased 4.3% (95% UI, 3.1%-5.6%) from 1990 to 2015, with much of the increase owing to population growth and improved survival for children and adolescents to older ages. Other than infectious conditions, many top causes of disability are associated with long-term sequelae of conditions present at birth (eg, neonatal disorders, congenital birth defects, and hemoglobinopathies) and complications of a variety of infections and nutritional deficiencies. Anemia, developmental intellectual disability, hearing loss, epilepsy, and vision loss are important contributors to childhood disability that can arise from multiple causes. Maternal and reproductive health remains a key cause of disease burden in adolescent females, especially in lower-SDI countries. In low-SDI countries, mortality is the primary driver of health loss for children and adolescents, whereas disability predominates in higher-SDI locations; the specific pattern of epidemiological transition varies across diseases and injuries.

**Conclusions and Relevance:**

Consistent international attention and investment have led to sustained improvements in causes of health loss among children and adolescents in many countries, although progress has been uneven. The persistence of infectious diseases in some countries, coupled with ongoing epidemiologic transition to injuries and noncommunicable diseases, require all countries to carefully evaluate and implement appropriate strategies to maximize the health of their children and adolescents and for the international community to carefully consider which elements of child and adolescent health should be monitored.

## Key Points

**Question:**

What are the levels and trends of mortality and nonfatal health loss among children and adolescents from 1990 to 2015?

**Findings:**

This study found significant global decreases in all-cause child and adolescent mortality from 1990 to 2015, but with increasing global inequality. In countries with a low Socio-demographic Index (SDI), mortality is the primary driver of health loss in children and adolescents, largely owing to infectious, nutritional, maternal, and neonatal causes, while nonfatal health loss prevails in locations with a higher SDI.

**Meaning:**

Nations should evaluate drivers of disease burden among children and adolescents to aid implementation of appropriate strategies to maximize the health of populations.

## Teaser

This study quantifies and describes levels and trends of mortality and nonfatal health outcomes among children and adolescents from 1990 to 2015 to provide a framework for policy discussion.

## Introduction

Reducing mortality among children younger than 5 years has been a focus of significant international attention for several decades, beginning with the Convention on the Rights of the Child, accelerating during the Millennium Development Goal era, and continuing with the Sustainable Development Goals (SDGs).^1,2,3^ Global progress in reducing death in children younger than 5 years has been substantial,^4^ but much less attention has been focused on quantifying and minimizing mortality burden among older children and adolescents.^5^ Likewise, nonfatal health outcomes have received comparatively little attention despite the fact that injuries, noncommunicable diseases (NCDs), and acquired chronic conditions with childhood onset profoundly affect long-term health trajectories, future health care needs, intellectual development, and economic and productivity prospects.^6,7,8^

High return on investment is expected when evidence-based interventions are implemented to address the health and well-being of children and adolescents.^9^ During the past decades, the world experienced rapid economic changes along with declines in fertility and greater longevity in many countries, collectively leading to marked changes in global demographics.^10,11^ The identification of successes, unmet needs, and emerging challenges must therefore consider sociodemographic information to contextualize levels and trends of disease burden.^5,12^ This information can guide prevention and intervention efforts, tracking and allocation of resources for health and other youth-centric services (eg, education), and monitoring progress for countries at all points on the spectrum of economic development.

Two comprehensive reports on the burden of diseases and injuries in young persons were published following the Global Burden of Diseases, Injuries, and Risk Factors (GBD) 2013 Study.^13,14^ The first report covered children and adolescents 19 years or younger; the second described disease burden in young persons aged 10 to 24 years.^15^ In the present study—an extension of GBD 2015—we again focus on children and adolescents 19 years or younger, extending the data to 2015 and to 195 countries and territories. We present results separately by sex, describe the epidemiologic factors of several highly disabling conditions that arise from multiple GBD causes, report levels and trends in pregnancy complications among adolescents, and evaluate the association between metrics of disease burden and the Socio-demographic Index (SDI), a composite indicator of development status generated for GBD 2015.

## Methods

Detailed methods for each analytic step in GBD 2015 are described elsewhere and are compliant with the Guidelines for Accurate and Transparent Health Estimates Reporting (GATHER).^4,16,17,18,19,20,21^ Data are available online at the Global Health Data Exchange (http://ghdx.healthdata.org).

Briefly, we quantified an extensive set of health loss metrics—with corresponding uncertainty intervals (UIs)—from 1990 to 2015 for 20 age groups and both sexes in 195 countries and territories. For the present study, we further analyzed levels and trends for children and adolescents 19 years or younger, which includes the first 7 age groups of the GBD 2015 analyses. Health loss metrics in this analysis include all-cause mortality, cause-specific mortality (deaths and years of life lost [YLLs]), nonfatal health outcomes (prevalence and years lived with disability [YLDs]), and total disease burden (disability-adjusted life years [DALYs]). Countries and territories were hierarchically organized into 21 regions and 7 super-regions, which are aggregates of the 21 regions in the GBD location hierarchy. The GBD cause list organizes all diseases and injuries into a 4-level hierarchy. The first level has 3 categories: (1) communicable, maternal, neonatal, and nutritional disorders (group I conditions); (2) NCDs; and (3) injuries. Level 2 of the hierarchy has 21 cause groups, while levels 3 (166 causes) and 4 (261 causes) contain more disaggregated causes and cause groups. The full GBD cause list with corresponding *International Classification of Diseases* (*ICD)-9* and *ICD-10* codes is available in previous publications on cause-specific mortality and nonfatal health outcomes.^16,17^

Our all-cause and cause-specific mortality analyses used systematic approaches to address data challenges such as variation in both death certification practices and coding schemes, inconsistent age group reporting, and misclassification of human immunodeficiency virus (HIV) or AIDS. Each death was assigned to a single underlying cause. Cause-of-death ensemble modeling was the most widely used statistical tool for estimating cause-specific mortality across GBD 2015. Cause-of-death ensemble modeling uses a train-test-test approach to evaluate a wide range of families of statistical models, maximizing out-of-sample predictive validity of final models. Years of life lost were calculated by multiplying counts of age-specific death and normative life expectancy at the age of death.^16^

Analyses of nonfatal health outcomes used detailed epidemiologic data from systematic reviews of the literature, hospital and claims databases, health surveys, case notification systems, cohort studies, and disease-specific registries. DisMod-MR 2.1, a statistical modeling method developed in-house, was the most widely used statistical method in GBD 2015; it is a Bayesian meta-regression tool that synthesizes all available data, adjusting for different case definitions or sampling strategies, to generate internally consistent results for prevalence, incidence, remission, and excess mortality in each population.^22^ Each most-specific cause was paired with a variable number of mutually exclusive and collectively exhaustive sequelae, which quantify the main outcomes (including asymptomatic states) of diseases and injuries and are the units of analysis for nonfatal health outcomes. Years lived with disability were calculated as the product of sequela-specific prevalence and corresponding GBD disability weights derived from population surveys with more than 60 000 respondents.^23,24^ Disability weights were assumed to be invariant by geography, but the distribution of sequelae—and therefore cumulative disability per case—varies by geography, year, sex, and age. Finally, we adjusted for comorbid illness using a microsimulation framework within each population and proportionally adjusting YLDs for each comorbid condition. Disability-adjusted life years are the sum of YLLs and YLDs.^17^

We developed the SDI for GBD 2015, as described previously, to characterize epidemiologic transitions more robustly than is possible with analyses based only on income.^4,16,17,18,19^ The SDI is a composite measure of developmental status as it is associated with health, calculated as the geometric mean of the following 3 indicators: total rate of fertility, log income per capita, and mean years of education among those 15 years or older. Socio-demographic Index scores were scaled from 0 (highest fertility, lowest income, and lowest education) to 1 (highest income, highest education, and lowest fertility), and each geographical unit was assigned an SDI score for each year. We analyzed the average association between SDI score and all-ages rates of YLLs, YLDs, and DALYs for all level 2 and level 3 causes. For comparisons across SDI quintiles, each geographical unit was assigned to a single quintile according to its SDI in 2015 (eFigure 1 in the Supplement).

For all results, 95% UIs were derived from 1000 draws of the posterior distribution at each analytic step and represent the ordinal 25th and 975th draws. Unlike confidence intervals, which capture only sampling error, UIs provide a means of also capturing other sources of uncertainty owing to model specification (eg, parameter selection) and estimation (eg, data adjustments from nonreference categories and β values for covariates). Cumulative and annualized rates of change were calculated on point estimates, and corresponding UIs were derived from the same calculations performed at the draw level.

We present results as both total numbers to illustrate the absolute magnitude of burden, and all-age rates, to compare across geographical areas with differently sized populations. We completed age standardization for ages 19 years or younger for the 10 highest-ranked global causes of death and disability to help compare across populations with different age structures; all other results are presented as total number and all-ages rates only. Results for the global level, along with SDI quintile and region in order of decreasing SDI, are presented in the main article. Results for each country and territory are contained in the Supplement and are available online at http://vizhub.healthdata.org/gbd-compare by age group and sex.

## Results

### All-Cause Mortality and Cause-Specific Mortality in Children and Adolescents

Total deaths and the age-standardized mortality rate (per 100 000 population) for all causes combined, as well as the 10 largest level 3 causes of death globally, are shown for children and adolescents 19 years or younger in 1990 and 2015 in Table 1. Corresponding country-level results, with uncertainty and cumulative percent change, are in eTable 1 in the Supplement for children and adolescents 19 years or younger and eTable 2 in the Supplement for children and adolescents 5 years or younger. In 2015, there were 7.26 million (95% UI, 7.14 million to 7.39 million) deaths among children and adolescents globally, of which 5.82 million (95% UI, 5.69 million to 5.95 million) occurred among children younger than 5 years, 463 000 (95% UI, 453 000-473 000) among those aged 5 to 9 years, 391 000 (95% UI, 383 000-402 000) among children aged 10 to 14 years, and 588 000 (95% UI, 567 000-610 000) among those aged 15 to 19 years.

**Table 1.** Top 10 Global Causes of Death in Children and Adolescents 19 Years or Younger, Both Sexes, 1990 and 2015

GBD Location	No. of Deaths (Death Rate) per 100 000 Population										
	All Causes	Neonatal Preterm Birth Complications	Lower Respiratory Tract Infections	Neonatal Encephalopathy Due to Birth Asphyxia or Trauma	Diarrheal Diseases	Congenital Anomalies	Malaria	Neonatal Sepsis and Other Neonatal Infections	Meningitis	Other Neonatal Disorders	HIV and AIDS
**2015**											
Global	7 263 484 (285.4)	805 778 (31.4)	792 992 (31.1)	740 424 (28.8)	569 737 (22.4)	543 314 (21.3)	534 007 (21.0)	351 667 (13.7)	220 530 (8.7)	220 247 (8.6)	202 929 (8.1)
SDI											
High	118 122 (43.9)	13 493 (5.2)	4399 (1.7)	5509 (2.1)	832 (0.3)	23 775 (9.1)	1 (0.0)	2920 (1.1)	958 (0.4)	5802 (2.2)	699 (0.3)
High-middle	536 318 (118.3)	75 776 (17.4)	43 653 (9.7)	49 568 (11.4)	11 265 (2.5)	79 782 (18.0)	428 (0.1)	18 756 (4.3)	7066 (1.5)	21 014 (4.8)	21 175 (4.6)
Middle	1 191 374 (174.8)	178 438 (26.2)	108 851 (16.0)	133 759 (19.7)	49 594 (7.3)	132 103 (19.4)	6396 (0.9)	44 875 (6.6)	23 862 (3.5)	36 854 (5.4)	21 159 (3.1)
Low-middle	3 418 022 (425.4)	410 824 (50.0)	382 444 (47.5)	432 718 (52.6)	322 586 (40.2)	193 453 (23.8)	252 862 (31.7)	173 049 (21.1)	99 627 (12.5)	99 004 (12.1)	91 953 (11.7)
Low	1 996 606 (581.6)	126 934 (34.2)	253 251 (72.3)	118 676 (32.0)	185 251 (53.7)	113 896 (32.0)	274 248 (80.4)	111 929 (30.2)	88 928 (26.2)	57 449 (15.5)	67 860 (22.1)
GBD region											
High-income North America	42 322 (48.5)	5914 (7.3)	826 (1.0)	1683 (2.1)	328 (0.4)	7144 (8.6)	0 (0.0)	730 (0.9)	252 (0.3)	2382 (2.9)	95 (0.1)
Australasia	2582 (35.4)	205 (3.0)	55 (0.8)	150 (2.2)	16 (0.2)	513 (7.3)	0 (0.0)	36 (0.5)	18 (0.3)	141 (2.0)	2 (0.0)
High-income Asia Pacific	8211 (26.0)	519 (1.8)	325 (1.1)	205 (0.7)	54 (0.2)	1718 (5.9)	0 (0.0)	172 (0.6)	39 (0.1)	303 (1.1)	14 (0.0)
Western Europe	25 449 (29.6)	3090 (3.8)	594 (0.7)	1247 (1.5)	176 (0.2)	5728 (6.9)	0 (0.0)	514 (0.6)	232 (0.3)	1205 (1.5)	59 (0.1)
Southern Latin America	16 800 (85.5)	2582 (13.6)	892 (4.6)	579 (3.0)	173 (0.9)	3821 (19.9)	0 (0.0)	658 (3.5)	186 (1.0)	677 (3.6)	77 (0.4)
Eastern Europe	32 817 (76.0)	2390 (5.1)	2112 (4.7)	2082 (4.5)	199 (0.4)	6781 (14.8)	0 (0.0)	1318 (2.8)	429 (0.9)	1584 (3.4)	532 (1.2)
Central Europe	10 849 (48.1)	1330 (6.3)	955 (4.3)	423 (2.0)	71 (0.3)	2323 (10.8)	0 (0.0)	164 (0.8)	98 (0.4)	532 (2.5)	25 (0.1)
Central Asia	61 200 (180.5)	7663 (21.8)	14 789 (42.6)	8096 (23.0)	1777 (5.1)	6966 (20.0)	3 (0.0)	1242 (3.5)	793 (2.4)	2421 (6.9)	48 (0.2)
Central Latin America	114 654 (128.2)	13 254 (15.5)	11 000 (12.5)	5894 (6.9)	4339 (4.9)	20 035 (23.0)	20 (0.0)	6190 (7.2)	1039 (1.2)	3064 (3.6)	596 (0.7)
Andean Latin America	30 164 (135.9)	3123 (14.2)	4242 (19.1)	2508 (11.4)	1012 (4.6)	3657 (16.5)	2 (0.0)	2472 (11.2)	413 (1.9)	642 (2.9)	22 (0.1)
Caribbean	32 608 (222.5)	3476 (24.3)	3652 (25.2)	2384 (16.7)	2913 (20.2)	3669 (25.3)	36 (0.2)	1959 (13.7)	932 (6.4)	1544 (10.8)	933 (6.1)
Tropical Latin America	83 965 (133.3)	10 140 (17.5)	6045 (10.0)	6083 (10.5)	1978 (3.3)	10 729 (18.1)	20 (0.0)	5271 (9.1)	1446 (2.3)	4525 (7.8)	710 (1.1)
East Asia	309 899 (95.6)	39 620 (12.5)	28 066 (8.7)	27 558 (8.7)	2230 (0.7)	53 615 (16.7)	27 (0.0)	2766 (0.9)	3041 (0.9)	7121 (2.2)	1655 (0.5)
Southeast Asia	365 942 (162.2)	47 066 (21.2)	46 590 (20.8)	31 717 (14.3)	17 561 (7.8)	39 415 (17.6)	3273 (1.4)	18 379 (8.3)	8862 (3.9)	10 376 (4.7)	2295 (1.0)
Oceania	15 005 (290.4)	1244 (23.5)	2778 (53.1)	738 (14.0)	617 (12.0)	851 (16.2)	413 (8.3)	314 (5.9)	371 (7.2)	671 (12.7)	64 (1.3)
North Africa and Middle East	529 160 (222.5)	83 998 (34.3)	55 221 (22.8)	19 930 (8.1)	24 608 (10.1)	81 812 (33.6)	4371 (1.8)	17 737 (7.2)	11 040 (4.6)	19 646 (8.0)	494 (0.2)
South Asia	2 205 667 (343.6)	379 162 (59.8)	235 756 (37.0)	413 928 (65.2)	175 213 (27.4)	111 162 (17.4)	21 434 (3.3)	61 781 (9.8)	55 233 (8.6)	71 394 (11.3)	15 984 (2.4)
Southern sub-Saharan Africa	126 790 (386.8)	10 049 (29.4)	9265 (27.8)	9157 (26.8)	11 466 (34.3)	4269 (12.7)	873 (2.6)	4274 (12.5)	1932 (5.9)	6571 (19.2)	40 778 (128.4)
Western sub-Saharan Africa	1 680 122 (665.5)	93 613 (34.1)	170 118 (66.7)	105 859 (38.6)	197 475 (77.6)	72 544 (27.8)	353 769 (141.8)	141 738 (51.7)	71 368 (28.9)	29 955 (11.0)	47 729 (21.4)
Eastern sub-Saharan Africa	1 106 529 (476.2)	70 810 (28.4)	140 010 (59.0)	74 910 (30.1)	96 769 (41.8)	81 468 (33.9)	73 950 (31.9)	57 493 (23.1)	44 598 (19.4)	39 987 (16.1)	78 604 (37.0)
Central sub-Saharan Africa	462 738 (591.5)	26 521 (30.7)	59 690 (74.4)	25 282 (29.3)	30 751 (38.8)	25 084 (30.4)	75 807 (98.2)	26 449 (30.7)	18 196 (23.7)	15 497 (18.0)	12 202 (18.2)
**1990**											
Global	14 182 624 (584.6)	1 795 211 (71.5)	2 241 773 (91.6)	915 323 (36.4)	1 536 806 (63.3)	696 037 (28.3)	791 867 (32.9)	329 296 (13.1)	376 652 (15.6)	351 304 (14.0)	39 363 (1.6)
SDI											
High	295 736 (100.5)	42 760 (15.5)	17 451 (6.1)	17 881 (6.5)	3350 (1.2)	50 953 (18.1)	9 (0.0)	4644 (1.7)	4573 (1.6)	9008 (3.3)	927 (0.3)
High-middle	1 666 079 (319.0)	286 715 (54.8)	248 629 (47.4)	96 928 (18.5)	107 312 (20.5)	161 837 (30.9)	918 (0.2)	25 555 (4.9)	32 409 (6.2)	54 939 (10.5)	1269 (0.2)
Middle	3 608 743 (473.5)	575 921 (73.6)	622 238 (80.8)	240 833 (30.8)	317 411 (41.5)	219 456 (28.5)	12 876 (1.7)	62 606 (8.0)	84 860 (11.2)	87 112 (11.2)	1502 (0.2)
Low-middle	6 148 482 (934.4)	765 273 (107.7)	1 023 454 (153.8)	459 777 (64.6)	784 970 (120.3)	186 302 (27.4)	366 926 (57.3)	149 124 (21.0)	156 226 (24.2)	137 559 (19.5)	17 592 (2.7)
Low	2 457 431 (1297.4)	123 952 (56.3)	328 981 (168.1)	99 603 (45.2)	322 951 (170.3)	77 154 (38.1)	410 936 (224.0)	87 218 (39.8)	98 394 (52.6)	62 494 (28.6)	18 035 (9.6)
GBD region											
High-income North America	74 124 (91.8)	12 644 (15.7)	1903 (2.4)	3113 (3.9)	250 (0.3)	11 613 (14.4)	0 (0.0)	963 (1.2)	891 (1.1)	3264 (4.1)	487 (0.6)
Australasia	4856 (80.1)	658 (11.5)	120 (2.1)	277 (4.8)	10 (0.2)	829 (14.3)	0 (0.0)	60 (1.1)	57 (1.0)	77 (1.3)	7 (0.1)
High-income Asia Pacific	30 483 (69.1)	2632 (7.1)	1550 (3.7)	886 (2.4)	213 (0.5)	5776 (15.0)	5 (0.0)	489 (1.3)	373 (0.9)	635 (1.7)	29 (0.1)
Western Europe	67 742 (74.7)	10 249 (12.4)	1934 (2.2)	4157 (5.0)	261 (0.3)	12 813 (15.1)	0 (0.0)	1041 (1.3)	1073 (1.2)	1225 (1.5)	212 (0.2)
Southern Latin America	33 064 (172.3)	7143 (37.0)	3231 (16.7)	1866 (9.7)	981 (5.1)	5072 (26.3)	2 (0.0)	1171 (6.1)	668 (3.5)	1078 (5.6)	94 (0.5)
Eastern Europe	97 965 (164.7)	9661 (17.7)	9579 (16.5)	8884 (16.3)	1901 (3.3)	16 902 (29.6)	0 (0.0)	1805 (3.3)	2274 (3.8)	3293 (6.0)	199 (0.3)
Central Europe	51 452 (150.4)	8394 (26.4)	8268 (24.8)	2840 (8.9)	856 (2.6)	7929 (24.2)	0 (0.0)	574 (1.8)	929 (2.7)	2772 (8.7)	136 (0.4)
Central Asia	136 834 (390.1)	13 318 (36.7)	51 286 (143.6)	12 803 (35.3)	13 444 (37.4)	8107 (22.7)	12 (0.0)	1524 (4.2)	2341 (6.8)	3213 (8.9)	18 (0.1)
Central Latin America	262 420 (297.0)	31 927 (35.4)	34 869 (39.1)	18 532 (20.5)	40 522 (45.4)	22 447 (25.1)	233 (0.3)	8584 (9.5)	3995 (4.5)	6459 (7.2)	364 (0.4)
Andean Latin America	107 369 (508.1)	8778 (39.8)	24 661 (115.2)	4969 (22.6)	13 822 (65.0)	3676 (17.0)	50 (0.3)	4127 (18.8)	1412 (6.8)	1557 (7.1)	25 (0.1)
Caribbean	73 033 (455.0)	7303 (44.7)	8756 (54.3)	4272 (26.1)	14 855 (92.1)	4551 (28.2)	160 (1.0)	2378 (14.6)	2687 (16.7)	2538 (15.5)	775 (4.8)
Tropical Latin America	238 315 (343.6)	42 309 (61.3)	32 888 (47.3)	14 189 (20.5)	41 650 (60.2)	13 525 (19.5)	485 (0.7)	9689 (14.0)	6676 (9.6)	4045 (5.9)	392 (0.6)
East Asia	1 864 295 (383.0)	333 663 (67.9)	409 264 (83.1)	72 246 (14.7)	64 343 (13.2)	165 662 (33.8)	76 (0.0)	7371 (1.5)	27 587 (5.7)	38 942 (7.9)	55 (0.0)
Southeast Asia	1 068 595 (480.1)	122 834 (54.4)	211 599 (94.8)	62 520 (27.7)	102 556 (46.1)	48 562 (21.7)	15 537 (7.1)	25 592 (11.3)	26 666 (12.0)	29 145 (12.9)	733 (0.3)
Oceania	19 733 (517.9)	1410 (34.8)	4610 (118.0)	700 (17.3)	1526 (40.3)	647 (16.5)	592 (16.1)	231 (5.7)	546 (14.4)	592 (14.7)	13 (0.4)
North Africa and Middle East	1 045 563 (531.5)	153 951 (75.1)	168 843 (84.4)	28 635 (14.0)	123 274 (61.4)	105 344 (52.3)	4665 (2.4)	17 328 (8.5)	23 954 (12.1)	37 480 (18.3)	91 (0.0)
South Asia	4 939 233 (808.2)	831 361 (127.9)	741 686 (120.5)	497 476 (76.5)	586 134 (97.0)	136 714 (21.8)	56 232 (9.7)	79 611 (12.3)	122 461 (20.5)	111 976 (17.4)	537 (0.1)
Southern sub-Saharan Africa	144 842 (497.4)	11 693 (38.6)	20 799 (70.5)	7794 (25.7)	29 998 (101.6)	4607 (15.5)	831 (2.9)	3686 (12.2)	2585 (9.0)	12 584 (41.6)	3422 (11.7)
Western sub-Saharan Africa	1 853 426 (1333.6)	84 305 (52.7)	226 611 (159.6)	81 707 (51.0)	246 187 (177.6)	53 610 (36.3)	368 547 (275.3)	96 745 (60.6)	76 499 (56.0)	32 635 (20.5)	5590 (4.1)
Eastern sub-Saharan Africa	1 612 637 (1193.5)	80 419 (52.0)	222 288 (159.4)	72 023 (46.5)	214 691 (159.5)	53 005 (37.0)	249 176 (189.0)	48 757 (31.7)	58 781 (44.1)	45 654 (29.6)	23 340 (17.3)
Central sub-Saharan Africa	456 634 (1160.0)	20 547 (44.8)	57 016 (139.7)	15 424 (33.7)	39 323 (98.5)	14 638 (34.4)	95 256 (248.6)	17 562 (38.4)	14 185 (36.8)	12 130 (26.6)	2833 (7.4)

Abbreviations: GBD, Global Burden of Diseases, Injuries, and Risk Factors Study; HIV, human immunodeficiency virus; SDI, Socio-demographic Index.

As can be seen in Table 1, mortality in children and adolescents 19 years or younger decreased in all SDI quintiles, but inequality increased. Nearly 75% of all deaths among children and adolescents in 2015 occurred in the 2 lowest SDI quintiles (compared with 61% in 1990), while only 1.6% occurred in the highest SDI quintile (compared with 2.1% in 2015). Age-standardized rates of death declined from 1990 to 2015 at similar rates of 55% and 56% in the 2 lowest and highest SDI quintiles, respectively, while they declined by 63% in middle and high-middle SDI quintiles. South Asia accounted for 2.21 million (95% UI, 2.15 million to 2.27 million) child and adolescent deaths, 30.4% of the global total and the most of any region. Next were Western sub-Saharan Africa (1.68 million; 95% UI, 1.61 million to 1.76 million [23.1%]), Eastern sub-Saharan Africa (1.11 million; 95% UI, 1.07 million to 1.14 million [15.3%]), North Africa and the Middle East (529 000; 95% UI, 499 000-562 000 [7.3%]), and central sub-Saharan Africa (463 000; 95% UI, 408 000-524 000 [6.4%]). Geographical patterns of mortality in children younger than 5 years were similar to those in children and adolescents 19 years or younger but with a slightly greater concentration of mortality burden in the 2 lowest SDI quintiles (77% of total). Mortality rates (per 100 000 population) varied from a low of 26.0 (95% UI, 25.1-26.8) in the high-income Asia Pacific region to a high of 666 (95% UI, 638-696) in Western sub-Saharan Africa among all children and adolescents 19 years or younger and from 58.8 (95% UI, 55.8-61.8) in the high-income Asia Pacific region to 2133 (95% UI, 2029-2245) in Western sub-Saharan Africa for children 5 years or younger in 2015.

### Cause-Specific Mortality

As seen in Table 1 across the entire age range, rankings were dominated by those affecting the youngest children. Globally, the most common causes of death were neonatal preterm birth complications (mortality rate, 31.4 per 100 000 population; 95% UI, 29.1-34.2 deaths per 100 000 population), lower respiratory tract infections (LRIs) (31.1; 95% UI, 29.2-33.0), neonatal encephalopathy owing to birth asphyxia and trauma (28.8; 95% UI, 26.5-31.5), diarrheal diseases (22.4; 95% UI, 20.5-24.2), congenital anomalies (21.3; 95% UI, 19.7-23.1), malaria (21.0; 95% UI, 16.2-25.6), neonatal sepsis (13.7; 95% UI, 10.7-16.7), other neonatal disorders (8.6; 95% UI, 7.4-10.3), meningitis (8.7; 95% UI, 6.8-10.4), and HIV and AIDS (8.1; 95% UI, 7.8-8.3). With the exception of the infectious causes (malaria, diarrheal diseases, and meningitis) each cause was highly ranked in all regions.

**Figure 1.**
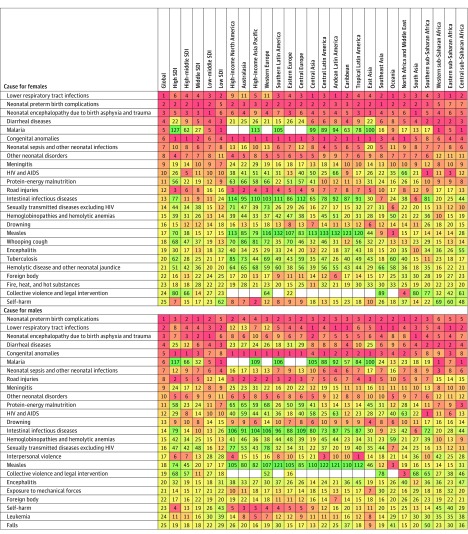
Ranking of the Top 25 Global Causes of Death in 2015 by 5 Socio-demographic Index (SDI) Quintiles and 21 Regions in the Global Burden of Diseases, Injuries, and Risk Factors Study (GBD) Ranking of causes of death in females and males. Global causes, SDI quintiles, and GBD regions appear in columns, sorted in order of decreasing SDI status. The causes are sorted according to their ranking at the global level. The color coding indicates the relative ranking of each cause, with red the highest and green the lowest. The numbers appearing in each column indicate the geography-specific ranking of that cause in 2015. Blanks indicate causes that were not contracted in that geographical area. HIV indicates human immunodeficiency virus.

Rankings of the 25 leading level 3 causes of death among children and adolescents 19 years or younger, disaggregated by sex, are shown in Figure 1. Besides the causes listed above, others ranking in the top 10 in specific regions included hemoglobinopathies and hemolytic anemias (in Western sub-Saharan Africa, where sickle cell disease is the largest level 4 cause of hemoglobinopathies), as well as selected infections (measles, HIV and AIDS, whooping cough, intestinal infectious disease, sexually transmitted infections excluding HIV [ie, congenital syphilis], and encephalitis) and injuries (drowning, road injuries, direct effects of war [ie, collective violence] and natural disasters, exposure to mechanical forces, aspiration of a foreign body, and fire).

### Differences in Causes of Death by Geography, Age, and Sex

We found important differences in mortality patterns for each of the 7 component age groups 19 years or younger in 2015 (eFigure 2A-G in the Supplement). During the neonatal period (ie, 6 days or less and 7-27 days), rankings across SDI quintiles and regions were broadly similar; mortality was dominated by neonatal complications, congenital anomalies, and LRIs. Divergence began to appear during the postneonatal period (ie, 28-364 days), when acquired infections such as LRIs, diarrhea, malaria, and meningitis predominated in lower-SDI geographical areas and congenital anomalies and sudden infant death syndrome predominated in higher-SDI geographical areas. Protein-energy malnutrition also emerged as an important cause of death in the postneonatal period in several regions, especially in males, a trend that continued into children aged 1 to 4 years, where it ranked fourth globally in both sexes. Malaria, LRIs, and diarrhea were the 3 highest-ranked causes of death in children aged 1 to 4 years; because protein-energy malnutrition and other forms of malnutrition raise the mortality risk for each, the effect of malnutrition is even higher than that reflected in results for protein-energy malnutrition alone. Geographic heterogeneity was also observed in other causes of death in children aged 1 to 4 years for both females and males at the global level, including measles (concentrated in the lowest 3 SDI quintiles, particularly Oceania and Southeast Asia), leukemia, road injuries, and drowning (all concentrated in the 3 highest SDI quintiles).

Geographical differences in causes of death in 2015 were more pronounced with increasing age (ie, 5-9 years, 10-14 years, and 15-19 years). Congenital anomalies and cancers (leukemia, brain cancer, and other neoplasms [eg, sarcomas]) were highly ranked in high-SDI regions in all age groups, simultaneously reflecting continued risk of mortality beyond the time of initial diagnosis and lower overall risk of mortality in the population. Intestinal infectious disease was highly ranked globally (second in children aged 5-9 years for both males and females), driven primarily by very large mortality numbers in South Asia and Southeast Asia. Human immunodeficiency virus and AIDS rose to be ranked first globally among children aged 10 to 14 years, driven almost entirely by epidemics in the Caribbean and sub-Saharan Africa. Diarrhea, LRIs, malaria, and protein-energy malnutrition remained important causes of death throughout all age groups but were largely limited except in geographical areas with lower SDIs. Five level 3 causes of maternal mortality—hemorrhage, hypertensive disorders, indirect causes, other direct causes, and the combined category of abortion, ectopic pregnancy, and miscarriage—were in the top 25 causes of maternal mortality globally in females aged 15 to 19 years, reflecting the high burden of maternal mortality among adolescents in the 2 lowest SDI quintiles.

The ranking of injuries as causes of death increased consistently with age and with increasing SDI; all injuries except self-harm ranked higher in males than females. Road injuries were the leading injury-associated cause of death in all age groups, rising to first globally among all causes for both sexes in adolescents aged 15 to 19 years. Drowning was the next highest-ranked cause of injury-associated death in children aged 5 to 9 years (ninth overall among females and sixth among males) and 10 to 14 years (eighth overall in females and third in males), while self-harm (second overall in females and third in males) and interpersonal violence (14th overall in females and second in males) were the next most common injury-associated causes of death among adolescents aged 15 to 19 years. The direct mortality burden of war was extremely large in North Africa and the Middle East, where it ranked second for each sex among children aged 1 to 4 years and first in all subsequent age groups in 2015.

### Leading Causes of Nonfatal Health Outcomes in Children and Adolescents

Total prevalent cases and the age-standardized prevalence rate (per 100 000 population) for all causes combined, as well as the 10 leading level 3 causes with the most YLDs globally, are shown for children and adolescents 19 years or younger in Table 2. Corresponding country-level results for 1990 and 2015, with uncertainty and mean annualized rates of change, are in eTable 3 in the Supplement for children and adolescents 19 years or younger and eTable 4 in the Supplement for children 5 years or younger. In 2015, nonfatal health outcomes caused 154 million (95% UI, 117 million to 196 million) YLDs among children and adolescents, of which 33.3 million (95% UI, 23.5 million to 45.3 million) were in children 5 years or younger, 35.0 million (95% UI, 24.9 million to 47.4 million) in those aged 5 to 9 years, 40.9 million (95% UI, 29.8 million to 54.9 million) in those aged 10 to 14 years, and 44.4 million (95% UI, 32.9 million to 58.0 million) in those aged 15 to 19 years.

**Table 2.** Top 10 Global Causes of Years Lived With Disability (YLDs) in Children and Adolescents 19 Years or Younger, Both Sexes, 1990 and 2015

GBD Location	No. (Rate) of Prevalent Cases and YLDs per 100 000 Population										
	All Causes	Iron-Deficiency Anemia	Skin and Subcutaneous Diseases	Asthma	Hemoglobinopathies and Hemolytic Anemias	Diarrheal Diseases	Congenital Anomalies	Protein-Energy Malnutrition	Epilepsy	Malaria	Neonatal Preterm Birth Complications
**Prevalence**											
Global	2 289 784 742 (91 528)	713 016 539 (28 435)	841 794 320 (33 722)	158 151 385 (6340)	590 315 873 (23 585)	23 261 098 (920)	33 930 983 (1355)	22 448 815 (881)	8 507 896 (340)	185 157 379 (7397)	19 663 514 (780)
SDI											
High	226 113 174 (82 348)	61 970 977 (22 899)	81 551 525 (29 298)	16 969 001 (6194)	33 076 375 (12 086)	208 573 (78)	4 816 429 (1759)	233 687 (88)	797 827 (291)	173 (0)	2 609 151 (968)
High-middle	426 126 367 (89 979)	116 899 236 (24 950)	161 438 549 (33 674)	31 001 285 (6567)	78 113 169 (16 524)	2 383 153 (519)	7 606 302 (1609)	1 996 215 (439)	1 685 760 (357)	1 903 940 (407)	4 007 704 (866)
Middle	620 437 513 (91 245)	183 177 818 (26 995)	235 523 024 (34 526)	40 051 924 (5918)	147 366 794 (21 680)	5 365 803 (790)	9 523 656 (1401)	5 312 581 (780)	2 279 318 (335)	8 147 684 (1204)	6 206 477 (912)
Low-middle	728 493 883 (94 109)	264 078 445 (33 931)	254 287 797 (33 195)	47 579 774 (6136)	242 252 419 (31 257)	10 271 205 (1300)	8 677 889 (1118)	10 895 631 (1359)	3 043 617 (394)	96 717 358 (12 427)	5 147 836 (654)
Low	287 519 634 (95 950)	86 753 142 (27 923)	108 638 223 (37 243)	22 328 931 (7575)	89 910 958 (29 897)	5059 868 (1574)	3 262 823 (1078)	4 053 749 (1174)	692 913 (230)	78 540 101 (26 024)	1 678 626 (511)
GBD region											
High-income North America	73 330 440 (79 912)	21 068 515 (23 475)	24 019 562 (25 692)	5 707 411 (6278)	14 931 029 (16 373)	4563 (5)	1 711 199 (1877)	57 (0)	307 601 (335)	0 (0)	1 139 482 (1295)
Australasia	5 588 277 (77 136)	1 640 974 (23 319)	2 146 577 (29 085)	832 472 (11 552)	283 121 (3945)	1639 (23)	115 479 (1603)	3 (0)	15 614 (216)	0 (0)	64 327 (900)
High-income Asia Pacific	26 481 561 (79 059)	6 465 007 (19 259)	10 625 742 (30 803)	1 693 356 (5094)	1 375 105 (4121)	6409 (21)	617 514 (1861)	22 (0)	86 975 (262)	170 (1)	221 380 (702)
Western Europe	74 207 193 (82 398)	20 289 274 (23 000)	25 919 813 (28 333)	6 752 467 (7434)	11 491 219 (12 828)	38 753 (45)	1 320 347 (1474)	43 (0)	271 816 (303)	0 (0)	737 461 (848)
Southern Latin America	17 481 603 (85 921)	5 289 625 (26 396)	5 828 636 (28 374)	1 743 887 (8559)	1 305 899 (6438)	14 815 (75)	317 631 (1565)	325 (2)	81 606 (401)	0 (0)	163 368 (817)
Eastern Europe	35 014 905 (87 740)	10 661 588 (26 359)	13 133 232 (33 901)	1 682 897 (4390)	2 945 210 (7331)	109 724 (245)	710 175 (1762)	143 535 (304)	74 876 (189)	0 (0)	364 503 (860)
Central Europe	20 577 466 (88 408)	5 438 029 (24 068)	7 978 808 (33 409)	1 063 290 (4589)	1 860 190 (8037)	24 565 (110)	426 788 (1840)	86 646 (391)	64 416 (276)	0 (0)	188 351 (833)
Central Asia	27 637 487 (90 263)	8 895 722 (28 648)	9 860 451 (32 898)	1 287 972 (4347)	2 758 875 (8982)	191 465 (572)	430 890 (1398)	200 781 (567)	162 189 (541)	9 (0)	492 888 (1523)
Central Latin America	83 994 289 (89 808)	20 274 661 (21 845)	30 778 797 (32 582)	9 302 241 (9967)	10 040 751 (10 760)	548 915 (616)	1 353 276 (1452)	189 979 (216)	431 697 (462)	144 994 (161)	539 148 (591)
Andean Latin America	20 988 527 (93 924)	7 087 131 (31 757)	7 825 043 (34 967)	2 708 694 (12 148)	2 196 360 (9831)	242 067 (1085)	267 125 (1196)	29 637 (133)	86 487 (387)	56 971 (256)	228 882 (1028)
Caribbean	14 098 610 (91 995)	4 867 798 (32 025)	5 415 326 (35 027)	2 437 520 (15 925)	1 639 505 (10 711)	161 040 (1090)	191 664 (1253)	61 637 (423)	47 701 (310)	31 611 (211)	298 919 (2000)
Tropical Latin America	62 922 628 (92 101)	18 749 359 (28 291)	24 326 804 (34 888)	9 541 805 (13 939)	11 600 835 (17 051)	588 023 (943)	951 436 (1401)	113 333 (190)	201 109 (296)	155 544 (247)	781 985 (1202)
East Asia	291 154 416 (88 314)	70 013 496 (21 384)	123 484 074 (37 206)	9 882 773 (3008)	60 640 901 (18 421)	599 691 (184)	5 838 748 (1773)	1 016 159 (312)	706 018 (215)	71 784 (22)	2 584 742 (791)
Southeast Asia	212 766 895 (92 671)	54 989 778 (24 111)	87 545 667 (37 908)	17 619 693 (7689)	45 115 712 (19 666)	2 162 600 (953)	3 206 636 (1398)	1 908 798 (846)	833 383 (364)	6 782 640 (2958)	2 874 530 (1265)
Oceania	4 801 545 (95 827)	1 171 545 (23 282)	2 038 890 (40 964)	533 158 (10 659)	964 886 (19 230)	43 411 (852)	64 763 (1290)	23 563 (453)	13 568 (270)	448 392 (8959)	86 629 (1689)
North Africa and Middle East	203 284 245 (90 320)	54 678 673 (24 033)	63 541 624 (28 678)	15 382 502 (6882)	47 859 607 (21 205)	2 159 395 (902)	2 804 537 (1241)	2 057 860 (843)	1 161 561 (514)	2 712 071 (1201)	1 604 151 (690)
South Asia	620 847 125 (93 528)	248 419 970 (37 653)	214 109 490 (32 007)	31 947 985 (4798)	199 986 527 (30 145)	8 261 636 (1274)	7 813 202 (1179)	10 067 488 (1587)	2 884 194 (434)	15 450 728 (2344)	4 572 823 (699)
Southern sub-Saharan Africa	29 695 592 (94 467)	8 012 341 (25 410)	11 355 636 (36 285)	3 433 792 (11 094)	6 406 425 (20 356)	396 166 (1220)	429 423 (1362)	160 371 (478)	66 419 (212)	650 182 (2065)	192 464 (597)
Western sub-Saharan Africa	206 177 716 (97 079)	71 758 741 (32 759)	71 801 710 (35 351)	12 737 815 (6197)	86 474 216 (40 608)	3 127 410 (1350)	2 318 812 (1079)	3 389 017 (1340)	435 017 (204)	95 826 414 (44 837)	996 271 (422)
Eastern sub-Saharan Africa	195 800 822 (95 264)	55 706 419 (26 096)	76 085 688 (37 921)	14 829 604 (7311)	56 946 975 (27 597)	3 235 815 (1488)	2 311 022 (1113)	2 290 920 (974)	393 064 (189)	40 319 184 (19 621)	1 143 654 (513)
Central sub-Saharan Africa	62 933 394 (97 393)	17 537 885 (26 368)	23 972 738 (38 407)	7 030 042 (11 074)	23 492 513 (36 235)	1 342 987 (1851)	730 306 (1114)	708 630 (896)	182 575 (284)	22 506 678 (35 097)	387 546 (525)
**YLDs**											
Global	153 738 779 (6151)	28 929 775 (1154)	18 299 658 (732)	7 170 928 (288)	4 676 015 (187)	3 780 968 (150)	3 169 555 (127)	2 779 412 (109)	2 512 221 (100)	2 471 320 (99)	2 222 098 (89)
SDI											
High	13 873 053 (5004)	2 268 673 (844)	1 903 844 (690)	773 452 (282)	333 309 (124)	34 413 (13)	525 720 (192)	29 393 (11)	142 020 (52)	5 (0)	234 807 (86)
High-middle	26 454 141 (5549)	4 564 413 (981)	3 547 547 (745)	1 445 951 (306)	717 114 (154)	402 587 (88)	684 409 (145)	257 535 (57)	444 219 (94)	53 564 (12)	489 408 (104)
Middle	38 514 608 (5656)	7 075 700 (1047)	4 977 816 (731)	1 785 676 (264)	1 328 362 (197)	856 900 (126)	847 432 (125)	645 602 (95)	618 917 (91)	193 942 (29)	765 470 (113)
Low-middle	53 056 669 (6878)	11 300 366 (1445)	5 505 932 (715)	2 153 411 (278)	1 798 392 (230)	1 668 008 (211)	840 951 (108)	1 348 008 (168)	1 042 262 (135)	1 097 615 (140)	634 062 (82)
Low	21 751 788 (7341)	3 706 095 (1179)	2 352 608 (795)	1 005 581 (341)	497 047 (162)	816 972 (254)	269 420 (89)	497 910 (144)	263 420 (87)	1 125 089 (368)	96 395 (31)
GBD region											
High-income North America	4 837 450 (5182)	768 122 (858)	638 731 (694)	260 458 (287)	129 016 (142)	768 (1)	215 027 (236)	7 (0)	50 603 (55)	0 (0)	100 598 (110)
Australasia	390 234 (5303)	59 268 (858)	49 823 (686)	37 895 (526)	4278 (62)	271 (4)	15 090 (210)	0 (0)	2535 (35)	0 (0)	6068 (84)
High-income Asia Pacific	1 520 962 (4430)	251 785 (760)	262 974 (782)	77 277 (233)	20 599 (63)	1068 (4)	64 847 (196)	2 (0)	14 260 (43)	5 (0)	16 610 (50)
Western Europe	4 510 597 (4953)	737 447 (837)	551 452 (607)	307 706 (339)	126 465 (142)	6406 (8)	141 916 (158)	5 (0)	44 328 (49)	0 (0)	65 889 (73)
Southern Latin America	1 077 954 (5267)	200 602 (1002)	140 337 (687)	79 492 (390)	12 957 (64)	2432 (12)	32 320 (159)	39 (0)	16 539 (81)	0 (0)	17 326 (85)
Eastern Europe	2 064 354 (5313)	396 743 (992)	276 676 (703)	76 460 (199)	35 684 (90)	18 078 (40)	58 397 (145)	18 042 (38)	17 728 (45)	0 (0)	34 663 (86)
Central Europe	1 161 550 (4923)	191 996 (863)	165 603 (702)	48 427 (209)	18 791 (84)	4049 (18)	40 841 (176)	10 900 (49)	14 567 (63)	0 (0)	18 275 (79)
Central Asia	1 682 951 (5565)	336 361 (1095)	204 701 (676)	58 557 (198)	37 625 (124)	31 413 (94)	32 170 (104)	25 109 (71)	41 611 (139)	0 (0)	48 759 (158)
Central Latin America	4 890 942 (5182)	755 908 (814)	706 785 (753)	423 348 (454)	91 052 (97)	90 295 (101)	130 554 (140)	23 811 (27)	113 023 (121)	4853 (5)	61 239 (66)
Andean Latin America	1 350 888 (6042)	273 511 (1228)	181 745 (813)	122 875 (551)	13 153 (59)	39 559 (177)	24 264 (109)	3687 (17)	21 852 (98)	1476 (7)	21 706 (97)
Caribbean	1 011 612 (6566)	189 319 (1248)	134 395 (875)	110 321 (721)	10 623 (70)	26 205 (177)	17 342 (113)	7640 (53)	13 827 (90)	545 (4)	30 241 (198)
Tropical Latin America	4 242 662 (6106)	718 422 (1091)	537 515 (781)	433 087 (633)	51 193 (76)	95 950 (154)	91 862 (135)	14 096 (24)	50 513 (74)	5329 (8)	92 446 (136)
East Asia	15 445 604 (4670)	2 583 236 (796)	2 497 965 (755)	449 977 (137)	851 152 (262)	98 975 (30)	398 918 (121)	127 719 (39)	186 159 (57)	1426 (0)	315 729 (96)
Southeast Asia	13 292 217 (5774)	2 030 377 (893)	1 954 826 (849)	800 218 (349)	359 444 (158)	353 453 (156)	254 392 (111)	238 088 (106)	236 992 (103)	140 513 (62)	343 313 (150)
Oceania	344 463 (6914)	49 509 (987)	57 805 (1154)	24 064 (481)	6559 (130)	7041 (138)	5006 (100)	2933 (56)	4823 (96)	13 309 (265)	7384 (147)
North Africa and Middle East	13 596 683 (6087)	2 051 891 (903)	1 462 893 (653)	698 541 (313)	499 378 (221)	353 666 (148)	265 386 (117)	256 463 (105)	377 985 (167)	42 659 (19)	183 712 (81)
South Asia	45 458 863 (6839)	10 764 532 (1635)	4 597 805 (690)	1 445 214 (217)	1 485 374 (225)	1 338 785 (207)	898 954 (136)	1 243 899 (196)	907 416 (136)	328 591 (50)	689 132 (104)
Southern sub-Saharan Africa	2 006 303 (6418)	302 072 (967)	248 647 (793)	155 539 (503)	19 923 (64)	64 622 (199)	42 545 (135)	19 996 (60)	20 611 (66)	13 685 (44)	20 124 (64)
Western sub-Saharan Africa	15 184 327 (7224)	3 262 703 (1463)	1 433 936 (694)	574 266 (279)	530 793 (242)	505 014 (218)	162 495 (75)	416 459 (165)	167 049 (78)	1 031 751 (475)	55 031 (24)
Eastern sub-Saharan Africa	13 844 433 (6824)	2 250 727 (1048)	1 589 604 (783)	670 667 (331)	268 953 (128)	525 699 (242)	212 384 (102)	283 413 (121)	145 305 (70)	564 410 (271)	76 371 (36)
Central sub-Saharan Africa	5 823 721 (9255)	755 235 (1114)	605 429 (944)	316 530 (498)	102 992 (154)	217 210 (299)	64 833 (98)	87 093 (110)	64 486 (100)	322 762 (494)	17 470 (25)

Abbreviations: GBD, Global Burden of Diseases, Injuries, and Risk Factors Study; SDI, Socio-demographic Index.

Iron-deficiency anemia was the highest-ranking level 3 cause of YLDs in children and adolescents, followed by skin and subcutaneous diseases, asthma, hemoglobinopathies and hemolytic anemias, diarrheal diseases, congenital anomalies, protein-energy malnutrition, epilepsy, malaria, and neonatal complications of preterm birth. Among children 5 years or younger, there was higher relative importance of disability owing to protein-energy malnutrition (third highest-ranking cause) and diarrheal diseases (fourth highest-ranking cause), as well as neonatal encephalopathy (ninth highest-ranking cause) and other neonatal disorders (tenth highest-ranking cause). Although the age-standardized prevalence and rate of YLDs decreased for most conditions, it increased for malaria and congenital anomalies. The burden of most conditions either decreased with increasing SDI or was relatively constant across different SDI quintiles. Two exceptions were congenital anomalies, which increased with increasing SDI, and hemoglobinopathies, which were highest in low- to middle-SDI geographical areas.

### Disability Burden From Conditions With Multiple Causes

Many clinical conditions cause significant disease burden in children and adolescents, but because they can arise from multiple causes, their effect is not obvious when examining causes of GBD. Examples that would be in the top 10 global causes of YLDs if considered alone are anemia, developmental intellectual disability, epilepsy, hearing loss, and vision loss. For example, while iron-deficiency anemia was the leading level 3 cause of disability, it accounted for only about two-thirds of total anemia in children and adolescents 19 years or younger in 2015 (eTable 5 in the Supplement), and each case tended to be less severe than other etiologic causes of anemia. Infectious diseases, hemoglobinopathies, malaria, hookworm, gynecologic conditions, and gastritis and duodenitis were other important causes of anemia in children and adolescents. Neonatal disorders were the most common nonidiopathic cause of both developmental intellectual disability (eTable 6 in the Supplement) and epilepsy (eTable 7 in the Supplement). Autism, iodine deficiency, and congenital disorders were important causes of intellectual disability, while much of the rest of intellectual disability and much of nonidiopathic epilepsy were secondary to infectious causes, especially malaria and meningitis. Hearing and vision loss also contributed to the disease burden within children and adolescents 19 years or younger, with age-associated and other hearing loss accounting for most hearing loss burden (eTable 8 in the Supplement). For vision loss, a range of causes contributed to the burden among children and adolescents 19 years or younger, including neonatal disorders and nutritional deficiencies (eTable 9 in the Supplement).

### Pregnancy Complications in Adolescents

Mortality was the primary driver of health loss owing to maternal disorders in adolescents. The global maternal mortality ratio per 100 000 live births was 278 (95% UI, 229-339) and 142 (95% UI, 123-166) in 2015 for children and adolescents aged 10 to 14 and 15 to 19 years, respectively, causing 1343 (95% UI, 1105-1640) and 26 855 (95% UI, 23 254-31 521) maternal deaths. Both age groups had a maternal mortality ratio higher than the global aggregate of 132 (95% UI, 117-153) seen in women aged 25 to 29 years (eTable 10 in the Supplement and GBD 2015 maternal mortality publication^18^). The mean annualized decline in the maternal mortality ratio among adolescents aged 10 to 19 years was only 1.4% (95% UI, 0.8%-2.0%), which was slower than the global improvement rate of 2.6% for overall maternal mortality. Maternal hemorrhage was the highest-ranked level 3 cause of maternal mortality globally, driven largely by its prominence in low-SDI geographical areas where teenage pregnancy and the burden of maternal mortality are the highest (eFigures 3 and 4 in the Supplement). Other top-ranked causes of maternal mortality included maternal hypertensive disorders, other direct maternal disorders (eg, pulmonary embolism, cardiomyopathy, and surgical and anesthetic complications), and the combined category of abortion, ectopic pregnancy, and/or miscarriage. The risk of nonfatal complications during pregnancy is also higher in adolescents than in women in their 20s (eFigure 5 in the Supplement). Abortion, ectopic pregnancy, and/or miscarriage is the most common disabling outcome of pregnancy among adolescents, followed by maternal hemorrhage, maternal hypertensive disorders, maternal sepsis and other maternal infections, and obstructed labor.

### Ranking and Trends of DALYs in Children and Adolescents

**Figure 2.**
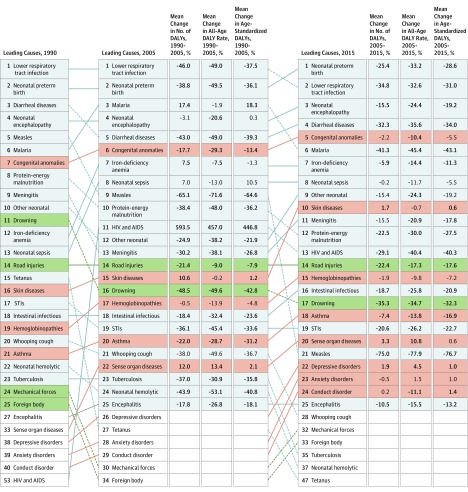
Leading Level 3 Causes of Global Disability-Adjusted Life Years (DALYs) in the Global Burden of Diseases, Injuries, and Risk Factors Study This figure shows the rankings for the top 25 causes of global disability-adjusted life years among children and adolescents 19 years or younger at the global level in 1990, 2005, and 2015. Lines connecting the boxes illustrate changes in ranking. Any cause that appears in the top 25 in any year is listed, along with its ranking during each year. Group I causes (infectious, neonatal, nutritional, and maternal) are shown in gray, noncommunicable diseases in red, and injuries in green. Changes in total DALYs are in the first column next to 2005, followed by changes in all-ages rates of DALYs, and age-standardized rates of DALYs. Statistically significant differences appear in bold. HIV indicates human immunodeficiency virus, and STI, sexually transmitted infection.

Ranking of the 25 leading level 3 causes of DALYs in 1990, 2005, and 2015, along with the changes in total number, all-ages rate, and age-standardized rate, are shown in Figure 2 for children and adolescents 19 years or younger. Corresponding DALY rankings disaggregated by SDI quintile are in eFigure 6A-E in the Supplement. Between 1990 and 2005, more than 40% declines in DALYs in children and adolescents 19 years or younger were seen for LRIs, diarrheal diseases, measles, tetanus, drowning, and neonatal hemolytic disease and other neonatal jaundice; similar declines between 2005 and 2015 were seen for malaria, measles, tetanus, and neonatal hemolytic disease and other neonatal jaundice. The most significant increase was for HIV and AIDS, which increased by close to 600% to rank 11th globally in 2005, a ranking that stayed largely static through 2015 despite a nearly 30% drop in DALYs from 2005 to 2015. Malaria and iron-deficiency anemia were the other group I conditions with significantly increased DALYs between 1990 and 2005; DALYs for both conditions also subsequently decreased significantly by 2015. Several NCDs increased in ranking from 1990 to 2015 for children and adolescents 19 years or younger. Some diseases—including congenital anomalies, asthma, and hemoglobinopathies and hemolytic anemias—increased in ranking despite registering decreased age-standardized DALY rates for each time period. In contrast, other causes—including sense organ diseases, skin diseases, and mental and substance abuse disorders such as depression, anxiety, and conduct disorder—increased in ranking with largely unchanged or slightly increased global age-standardized DALY rates from 1990 to 2015. Road injuries and drowning were the 2 highest-ranking injuries in terms of DALYs despite significant decreases from 1990 to 2015.

### SDI and Epidemiologic Transition in Children and Adolescents

**Figure 3.**
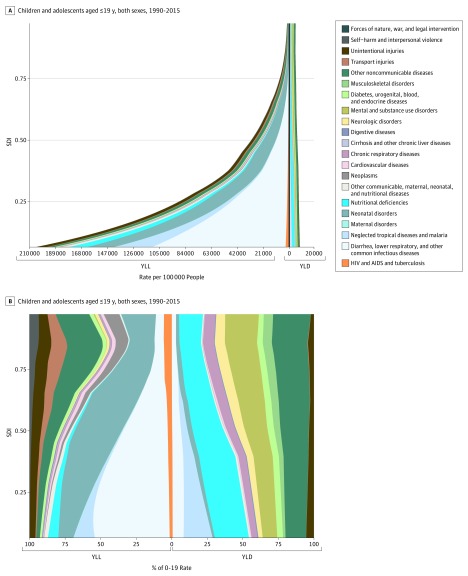
Expected Association Between Rates of Years of Life Lost (YLL) and Years Lived With Disability (YLD) Rates With Socio-demographic Index (SDI) for 21 Level 2 Causes in the Global Burden of Diseases, Injuries, and Risk Factors Study (GBD) A, Expected association between rates of YLL and YLD with SDI for the 21 GBD level 2 causes in children and adolescents 19 years or younger, both sexes, 1990-2015. Each geography is assigned an SDI value for each year, and nonlinear spline regressions are used to find the average relationship between SDI and cause-specific burden rates. B, Expected association between rates of YLL and YLD and SDI for the 21 GBD level 2 causes as a proportion of total rates of YLL and YLD in children and adolescents 19 years or younger, both sexes, 1990-2015. Each geographical area is assigned an SDI value for each year, and nonlinear spline regressions are used to find the mean association between SDI and cause-specific rates of disease burden. HIV indicates human immunodeficiency virus.

Figure 3A shows the mean association between SDI and cause-specific YLLs and YLDs from 1990 to 2015 for all level 2 causes. Nonlinearity of associations at times followed the nonlinearity of the SDI itself. There is a clear and substantial downward gradient in child and adolescent health loss with increasing SDI. Years of life lost are the dominant component of DALYs in the geographical areas with the lowest SDI, a trend that continues until an SDI of roughly 0.80, after which YLDs become responsible for a larger proportion of DALYs. There is also a clear increase in the all-ages rate of YLDs in the geographical areas with the highest SDI to the point where, at the highest SDI, 67% of all DALYs are owing to nonfatal health outcomes. Figure 3B shows the corresponding information displayed as a proportion of total rates of YLL and YLD owing to each level 2 cause at each SDI level. For most level 2 causes, the proportion of all YLLs owing to group I causes decreases with increasing SDI. The exceptions are neonatal disorders and HIV and AIDS and tuberculosis, which increased in relative importance with increasing SDI. In the geographical areas with the highest SDI, self-harm and interpersonal violence, other NCDs, and neoplasms were responsible for an increasing proportion of YLLs. The proportion of YLDs owing to group I causes similarly decreased with increasing SDI, while the proportion owing to NCDs generally increased. Most level 3 causes followed this same pattern, with 2 notable exceptions among the top causes of child and adolescent DALYs: congenital anomalies (eFigure 7A in the Supplement) and neonatal disorders (eFigure 7B in the Supplement). For congenital anomalies, there was a consistent decrease in the rate of YLLs, with increasing SDI for all causes, but increases in the rate of YLDs for most level 3 congenital anomalies, especially congenital heart anomalies and other congenital anomalies. For neonatal disorders, increasing SDI was associated with consistent improvements in neonatal sepsis, hemolytic disease of the newborn, and other neonatal disorders but not for preterm birth complications. Rates of DALYs for preterm birth complications increased initially, and there was little suggestion of further improvement beyond an SDI of 0.85 for any neonatal disorder, especially preterm birth complications.

The mean association between SDI and sex-specific rates of DALYs of level 3 causes also showed broad consistency in that the burden owing to most causes decreased with increasing SDI (eFigure 8A-V in the Supplement). Exceptions were the level 3 causes that consistently increased with increasing SDI—musculoskeletal and mental and substance abuse disorders—as well as those that either peaked in high-middle and middle SDI or improved only marginally until the highest SDI levels—asthma, epilepsy, migraine, road injuries, self-harm, interpersonal violence, and neoplasms such as leukemia, lymphoma, brain cancer, and other neoplasms. Sex differences were notable in that some conditions, such as neonatal disorders, neoplasms, nutritional deficiencies, hemoglobinopathies and hemolytic anemias, epilepsy, and all categories of injuries, had higher all-ages rates of DALYs in males, while others, such as migraine, gastrointestinal disorders, musculoskeletal disorders, and congenital anomalies, were higher in females.

## Discussion

We found widespread reductions in total disease burden among children and adolescents, but progress has been unequal. In 2015, an even greater share of global mortality burden was concentrated in the lowest-SDI countries than it was in 1990, and there has been a significant increase in the global proportion of nonfatal disease burden. Global reductions in disease burden from infectious, neonatal, maternal, and nutrition-associated causes have been accompanied by a growing importance of NCDs and injuries. Neonatal disorders and congenital anomalies remain large and, in some cases, growing problems in many countries. Nutritional deficiencies, along with infections such as HIV and AIDS, diarrhea, LRIs, malaria, intestinal infectious diseases, and vaccine-preventable diseases, also still cause enormous health loss in some countries. Some populations of children and adolescents have been struck by the massive effect of war on health, while others are struggling with the detrimental effects of road injuries, drowning, self-harm, and interpersonal violence, and the growing importance of NCDs such as mental and substance use disorders, cancer, congenital anomalies, and hemoglobinopathies. Leading causes of death and disability vary as a function of age, sex, and SDI status, so the precise challenges for each country may be very different. The SDGs set a series of absolute time-bound targets for improving health, the environment, and societal development. By design, the SDG agenda is broader than the Millennium Development Goal framework, in which there was only one child target (Millennium Development Goal 4: reduce mortality by two-thirds in children younger than 5 years). By broadening the agenda and shifting to absolute targets, the SDGs provide an excellent starting point to judge progress at the country level and should rightfully focus attention on the countries with the most progress yet to achieve.^25^ Tracking the entire spectrum of disease in children and adolescents facilitates monitoring of the SDGs but can also highlight non-SDG health challenges, and it should be used to inform final decision making with respect to SDG indicators.

Besides neonatal mortality and mortality in children younger than 5 years,^3^ other health-associated SDGs are important for understanding disease burden in children and adolescents given the age pattern of these conditions. Examples include infection-associated SDG targets addressing malaria, HIV and tuberculosis, neglected tropical diseases, severe malnutrition, and access to safe water, sanitation, and hygiene. Our analysis shows that these SDG indicators are improving for children and adolescents in most geographical areas. Sustainable Development Goals focusing on reproductive health targets such as maternal mortality, adolescent fertility, universal access to modern contraception, skilled birth attendance, and neonatal support services are also an important part of the discussion about child and adolescent health loss given the continued importance of teenage pregnancy, pregnancy-associated complications, and maternal death in many settings as well as the intricate links between maternal and child health. Many of the injury-associated SDG targets also are relevant to tracking child health progress, including those on disaster preparedness, road injuries, poisoning, self-harm, interpersonal violence, and war.

Most of the SDG targets addressing NCDs, such as those associated with mental health conditions and cardiovascular diseases, not only focus on NCDs that are primarily problems in adults but also specifically exclude children and adolescents. This exclusion is problematic, especially because of the growing importance found in our study of mental health, substance abuse, and self-harm among children and adolescents. Half of all mental illnesses begin by age 14 years and three-quarters begin by the mid-20s. If untreated, these conditions can predispose to self-harm and severely influence children’s development, educational attainment, and long-term fulfillment and economic potential.^26^ Other SDGs, such as those addressing education and sex equality, are not specifically associated with health but can have a significant effect on the health of children and adolescents.

One possible explanation for growing inequality in disease burden among children and adolescents is that many of the geographical areas with the lowest SDIs have not historically been significant recipients of development assistance for health (DAH).^27,28^ Although development assistance for child and newborn health has been among the fastest-growing focus areas of DAH since 1990 and is one of the few areas in which funding has continued to increase since 2010, it has been uneven. For example, most countries in Central and Western sub-Saharan Africa received less than half the DAH per all-cause DALY in 2010 to 2012 received by countries in Southern sub-Saharan Africa, Eastern sub-Saharan Africa, and Central Latin America.^29^ Many DAH programs have concentrated on funding widespread delivery of proven preventive measures, including vaccines; nutritional support; maternal education; improved water, sanitation, and hygiene; and treatment of diarrhea, LRIs, and HIV and AIDS. Synergistic maternal health programs have also enjoyed strong support during this period. These programs must continue and be expanded, especially in low-SDI settings in which progress in child and adolescent health has been comparatively slow, but focus must also be turned to strengthening health systems, especially in geographical areas in which such systems are underdeveloped or have been weakened by conflict.

This finding leads to another possible explanation for growing inequality; namely, that there has been inadequate focus on increasing local health system capacity and capabilities. High-SDI settings have seen improved prevention and better outcomes for many childhood illnesses, such as neonatal disorders, as well as many congenital birth defects, injuries, and cancers, such as leukemia. Improved treatment has led to increasing numbers of children now reaching adulthood with ongoing medical needs.^30,31,32,33,34^ These medical advances have not been as readily realized in geographical areas with lower SDIs or in older children and adolescents.^35,36^ Sustained investment is needed to improve prevention, diagnosis, and treatment for causes not traditionally targeted by DAH, including strengthening of workforces and facilities,^37,38,39^ cooperation between health centers in the same region,^40^ clinician specialization,^41,42,43,44^ improved surgical and anesthetic care,^34^ evidence-based case management for life-threatening complications,^45,46^ injury prevention, and active screening to identify and treat high-risk children (eg, those with congenital heart disease^47,48,49^ and hemoglobinopathies^50,51^). Integration of health care services across facilities and specialties to meet the varied needs of children and adolescents is paramount to successful intervention programs, especially in geographical areas with middle SDIs where there is a high likelihood of comorbid NCDs, injuries, and group I conditions.

Indeed, the World Health Organization Global Strategy for Women’s, Children’s, and Adolescents’ Health (2016-2030)^9^ addresses both explanations for growing inequality. In addition to advocating for programs supported by DAH, it recommends specific actions to address the interplay between environment, economics, and health through multisector action including investments and policy implementation, leadership engagement at regional and country levels, and building resilience of health systems through workforce development, human capital investment, sex equality, and youth empowerment.

One aspect of child and adolescent health that is not comprehensively addressed by the World Health Organization Global Strategy for Women’s, Children’s, and Adolescents’ Health (2016-2030)^9^ is injuries. Minimizing the burden of injuries in children and adolescents requires the implementation of specific prevention policies where they do not exist and support for treatment and rehabilitation of injured youth. First and foremost, this implementation necessitates strict limitations on child labor and elimination of child slavery.^52^ To reduce suicide, Sri Lanka and South Korea have both restricted the availability of pesticides with good effect,^53,54^ while Australia has taken the step of implementing comprehensive mental health screening and treatment.^55^ Along with suicide, interpersonal violence may be partially addressed via implementation of more stringent gun regulations,^56^ although many other social, demographic, and societal factors, such as poverty, education, and drug use, also must be addressed in policies aimed at reducing homicide.^57,58^ Road injuries, falls, and interpersonal violence are common etiologic causes of traumatic brain injury, a condition that can have significant mortality and morbidity.^59,60^ Policies requiring seat belts, appropriate use of car seats, and helmets for cyclists can greatly reduce the risk of traumatic brain injury following road injuries.^61^ Timely hospital care and ongoing rehabilitation services are necessary to minimize the long-term health burden of traumatic brain injury,^62,63^ although optimal approaches are still in development.^64^

In addition to effective medical programs, comprehensive community-based approaches are needed to maximize the development and empowerment of children and adolescents at the population level. One approach that could serve as a model—and may be possible in many high-SDI countries—is the Healthy Child, Healthy Future campaign in Northern Ireland.^65^ The central tenet of the program is to promote a shift from an approach to child health that relies on medical screening to identify children with treatable conditions to an approach that also emphasizes promotion of general health, primary prevention of diseases and injuries, and active intervention for children at risk. Involving parents, schools, and communities in this effort is seen as a crucial part because they are the entities who have the most influence on the physical and social environment of children, and their own personal behaviors are likely to have a direct effect on children in their care.

### Limitations

This analysis has several limitations. First, it is based on the GBD 2015 study results and is therefore subject to the same potential problems as the overall study, including variations in availability and quality of data, variations in coding practices, delays in release of data after their collection, paucity of data following conflict and natural disasters, and incomplete information or potential underreporting on nonfatal health outcomes in many geographical areas. Despite potential limitations, drawing conclusions on levels of and trends in child and adolescent health loss from this central study at least ensures that comparisons are all internally consistent with one another. Second, in this study we concentrated on only the leading global causes of fatal and nonfatal health loss in the aggregate age groups of children and adolescents 19 years or younger. This approach allows reporting on general patterns of health loss in children and adolescents, but given that childhood is a period of rapid development and change, it does not highlight less common conditions and may obscure some age-specific and geography-specific subtleties of epidemiologic factors of disease and injury. Third, data availability for children aged 5 to 14 years is not as robust as that for those 5 years or younger and individuals aged 15 to 19 years. Estimates of all-cause mortality were thus based primarily on extensive data sources in children 5 years or younger and individuals aged 15 to 19 years, with estimates for intervening age groups derived from a large collection of empirical life tables and use of survival history data from surveys and censuses.^16^ We found broad agreement between our all-cause mortality estimates and primary data sources from the Sample Registration System and Demographic and Health Surveys in India, which supports the GBD findings. Hill and colleagues^66^ have argued against this approach on the basis that it could underestimate mortality in populations in which interventions targeted at children 5 years or younger have not also led to improved survival in children aged 5 to 14 years. The implication is that such underestimation would dilute the perceived importance of causes that disproportionately affect children older than 5 years (eg, injuries, NCDs, and maternal disorders), but even if that were the case, it should have minimal effect on the interpretation of levels and trends within each age group or country. Fourth, given the geography-centric approach to GBD analyses, we have limited ability to evaluate disease burden in subpopulations that are not geographically based, such as refugees and many indigenous peoples. Fifth, while GBD 2015 has analyzed the total burden and underlying etiologic causes of several broad conditions, such as anemia and developmental intellectual disability, there are others that may be particularly important in children and adolescents that have not received the same level of scrutiny, including, for example, traumatic brain injury, hydrocephalus, and sepsis. Sixth, our quantification of disease burden focuses only on affected individuals and therefore does not capture indirect effects, including effects on education and development of child labor,^52^ the long-term effects of war,^67^ or the burden on parents, siblings, and communities of caring for ill or injured children and adolescents.^68^

## Conclusions

Timely, robust, and comprehensive assessment of disease burden among children and adolescents provides information that is essential to health policy decision making in countries at all points along the spectrum of economic development. Understanding the burden of disease and how it is changing helps identify context-specific successes, unmet needs, and future challenges. Child and adolescent health has dramatically improved from 1990 to 2015 throughout the world. International attention and investment have led to improvements in many infectious and nutritional diseases, but progress has been uneven. The burden of disease during childhood and adolescence is now even more concentrated in lower-SDI countries and territories than it was a generation ago, and there is an ongoing epidemiologic transition with a growing relative burden of NCDs and injuries. If we are going to continue the current pace of improvement in child and adolescent health, we must invest in better data collection, continue to monitor trends in population disease burden, and adapt health systems to meet the ongoing and changing needs of children and adolescents so that all can have a chance to grow up to be healthy.

## Notes

Supplement. **eFigure 1.** Socio-Demographic Index quintiles by GBD subnational level 1 geography, 2015 **eFigure 2a.** Top 25 Global Causes of Death, by 5 SDI quintiles and 21 GBD regions, Aged 0-6 days, Females & Males, 2015 **eFigure 2b.** Top 25 Global Causes of Death, by 5 SDI quintiles and 21 GBD regions, Aged 7-27 days, Females & Males, 2015 **eFigure 2c.** Top 25 Global Causes of Death, by 5 SDI quintiles and 21 GBD regions, Aged 28-364 days, Females & Males, 2015 **eFigure 2d.** Top 25 Global Causes of Death, by 5 SDI quintiles and 21 GBD regions, Aged 1-4 years, Females & Males, 2015 **eFigure 2e.** Top 25 Global Causes of Death, by 5 SDI quintiles and 21 GBD regions, Aged 5-9 years, Females & Males, 2015 **eFigure 2f.** Top 25 Global Causes of Death, by 5 SDI quintiles and 21 GBD regions, Aged 10-14 years, Females & Males, 2015 **eFigure 2g.** Top 25 Global Causes of Death, by 5 SDI quintiles and 21 GBD regions, Aged 15-19 years, Females & Males, 2015 **eFigure 3.** Top Global Causes of Maternal Mortality, by 5 SDI quintiles and 21 GBD regions, Aged 10 to 19 Years, Female, 2015 **eFigure 4.** The expected relationship between age, cause-specific YLLs and YLDs, and population with the Socio-Demographic Index (SDI) for the level 3 maternal causes, Females, 1990 to 2015 **eFigure 5.** Global pregnancy complication ratio (events per 100 live births) by type of complication and age group in 2015 **eFigure 6a.** Leading 25 GBD cause hierarchy level 3 causes of low SDI DALYs for both sexes combined for 1990, 2005 and 2015, 0-19 years **eFigure 6b.** Leading 25 GBD cause hierarchy level 3 causes of low-middle SDI DALYs for both sexes combined for 1990, 2005 and 2015, 0-19 years **eFigure 6c.** Leading 25 GBD cause hierarchy level 3 causes of middle SDI DALYs for both sexes combined for 1990, 2005 and 2015, 0-19 years **eFigure 6d.** Leading 25 GBD cause hierarchy level 3 causes of middle-high SDI DALYs for both sexes combined for 1990, 2005 and 2015, 0-19 years **eFigure 6e.** Leading 25 GBD cause hierarchy level 3 causes of high SDI DALYs for both sexes combined for 1990, 2005 and 2015, 0-19 years **eFigure 7a.** The expected relationship between YLL and YLD rates with SDI for GBD level 3 congenital causes, aged 0 to 19 years, both sexes, 1990 to 2015 **eFigure 7b.** The expected relationship between YLL and YLD rates with SDI for GBD level 3 neonatal causes, aged 0 to 19 years, both sexes, 1990 to 2015 **eFigure 8.** The expected relationship between cause-specific all-ages DALY rates for 0- 19 years, and Sociodemographic Index (SDI) for males (left) and females (right) **eTable 1.** Number of Deaths, Death Rates (per 100,000 population), and Cumulative Percent Change with 95% Uncertainty Intervals (UI) for the Top 10 Global Causes of Death in 195 Countries and Territories, Aged 0 to 19, Both Sexes, 1990 and 2015 **eTable 2.** Number of Deaths, Death Rates (per 100,000 population), and Cumulative Percent Change with 95% Uncertainty Intervals (UI) for the Top 10 Global Causes of Death in 195 Countries and Territories, Aged Under 5 Years, Both Sexes, 1990 and 2015 **eTable 3.** Prevalent Cases, Rates (per 100,000 population), Years Lived with Disability (YLDs), and Cumulative Percent Change with 95% Uncertainty Interval (UI) for the Top 10 Global Causes of YLDs in Children and Adolescents in 195 Countries and Territories, Aged 0 to 19 Years, Both Sexes, 1990 and 2015 **eTable 4.** Prevalent Cases, Rates (per 100,000 population), Years Lived with Disability (YLDs), and Cumulative Percent Change with 95% Uncertainty Interval (UI) for the Top 10 Global Causes of YLDs in Children and Adolescents in 195 Countries and Territories, Aged Under 5 Years, Both Sexes, 1990 and 2015 **eTable 5.** Prevalent Cases and Years Lived with Disability (YLDs), Percent Change, and Percent Change in Age-standardised Rates between 2005 and 2015 of Anemia by Cause, Aged 0 to 19 Years, Both Sexes, 2015 **eTable 6.** Prevalent Cases and Years Lived with Disability (YLDs), Percent Change, and Percent Change in Age-standardised Rates between 2005 and 2015 of Developmental Intellectual Disability by Cause, Aged 0 to 19 Years, Both Sexes, 2015 **eTable 7.** Prevalent Cases and Years Lived with Disability (YLDs), Percent Change, and Percent Change in Age-standardised Rates between 2005 and 2015 of Epilepsy by Cause, Aged 0 to 19, Both Sexes, 2015 **eTable 8.** Prevalent Cases and Years Lived with Disability (YLDs), Percent Change, and Percent Change in Age-standardised Rates between 2005 and 2015 of Hearing Loss by Cause, Aged 0 to 19, Both Sexes, 2015 **eTable 9.** Prevalent Cases and Years Lived with Disability (YLDs), Percent Change, and Percent Change in Age-standardised Rates between 2005 and 2015 of Vision Loss by Cause, Aged 0 to 19, Both Sexes, 2015 **eTable 10.** Number of Maternal Deaths, Maternal Mortality Ratio (MMR, number of deaths per 100,000 live births), and Annualized Rate of Change (ARC), in percent, for 195 Countries and Territories, Aged 10 to 19 Years, Females, 1990 to 2015
