# Supplementary material for: Child and Adolescent Health From 1990 to 2015: Findings From the Global Burden of Diseases, Injuries, and Risk Factors 2015 Study
Source: JAMA Pediatr. 2017 Apr 3;171(6):573–92. doi: 10.1001/jamapediatrics.2017.0250 (PMC5540012; doi:10.1001/jamapediatrics.2017.0250)
Supplement: Supplement. — eFigure 1. Socio-Demographic Index quintiles by GBD subnational level 1 geography, 2015 eFigure 2a. Top 25 Global Causes of Death, by 5 SDI quintiles and 21 GBD regions, Aged 0-6 days, Females & Males, 2015 eFigure 2b. Top 25 Global Causes of Death, by 5 SDI quintiles and 21 GBD regions, Aged 7-27 days, Females & Males, 2015 eFigure 2c. Top 25 Global Causes of Death, by 5 SDI quintiles and 21 GBD regions, Aged 28-364 days, Females & Males, 2015 eFigure 2d. Top 25 Global Causes of Death, by 5 SDI quintiles and 21 GBD regions, Aged 1-4 years, Females & Males, 2015 eFigure 2e. Top 25 Global Causes of Death, by 5 SDI quintiles and 21 GBD regions, Aged 5-9 years, Females & Males, 2015 eFigure 2f. Top 25 Global Causes of Death, by 5 SDI quintiles and 21 GBD regions, Aged 10-14 years, Females & Males, 2015 eFigure 2g. Top 25 Global Causes of Death, by 5 SDI quintiles and 21 GBD regions, Aged 15-19 years, Females & Males, 2015 eFigure 3. Top Global Causes of Maternal Mortality, by 5 SDI quintiles and 21 GBD regions, Aged 10 to 19 Years, Female, 2015 eFigure 4. The expected relationship between age, cause-specific YLLs and YLDs, and population with the Socio-Demographic Index (SDI) for the level 3 maternal causes, Females, 1990 to 2015 eFigure 5. Global pregnancy complication ratio (events per 100 live births) by type of complication and age group in 2015 eFigure 6a. Leading 25 GBD cause hierarchy level 3 causes of low SDI DALYs for both sexes combined for 1990, 2005 and 2015, 0-19 years eFigure 6b. Leading 25 GBD cause hierarchy level 3 causes of low-middle SDI DALYs for both sexes combined for 1990, 2005 and 2015, 0-19 years eFigure 6c. Leading 25 GBD cause hierarchy level 3 causes of middle SDI DALYs for both sexes combined for 1990, 2005 and 2015, 0-19 years eFigure 6d. Leading 25 GBD cause hierarchy level 3 causes of middle-high SDI DALYs for both sexes combined for 1990, 2005 and 2015, 0-19 years eFigure 6e. Leading 25 GBD cause hierarchy level 3 causes of high SDI DAL [file jamapediatr-171-573-s001.pdf]

Supplementary Online Content

The Global Burden of Disease Child and Adolescent Health Collaboration. Child and adolescent health from 1990 to 2015: findings from the Global Burden of Diseases, Injuries, and Risk Factors 2015 Study. *JAMA Pediatr*. Published April 3, 2017. doi:10.1001/jamapediatrics.2017.0250

eFigure 1. Socio-Demographic Index quintiles by GBD subnational level 1 geography, 2015

eFigure 2a. Top 25 Global Causes of Death, by 5 SDI quintiles and 21 GBD regions, Aged 0-6 days, Females & Males, 2015

eFigure 2b. Top 25 Global Causes of Death, by 5 SDI quintiles and 21 GBD regions, Aged 7-27 days, Females & Males, 2015

eFigure 2c. Top 25 Global Causes of Death, by 5 SDI quintiles and 21 GBD regions, Aged 28-364 days, Females & Males, 2015

eFigure 2d. Top 25 Global Causes of Death, by 5 SDI quintiles and 21 GBD regions, Aged 1-4 years, Females & Males, 2015

eFigure 2e. Top 25 Global Causes of Death, by 5 SDI quintiles and 21 GBD regions, Aged 5-9 years, Females & Males, 2015

eFigure 2f. Top 25 Global Causes of Death, by 5 SDI quintiles and 21 GBD regions, Aged 10-14 years, Females & Males, 2015

eFigure 2g. Top 25 Global Causes of Death, by 5 SDI quintiles and 21 GBD regions, Aged 15-19 years, Females & Males, 2015

eFigure 3. Top Global Causes of Maternal Mortality, by 5 SDI quintiles and 21 GBD regions, Aged 10 to 19 Years, Female, 2015

eFigure 4. The expected relationship between age, cause-specific YLLs and YLDs, and population with the Socio-Demographic Index (SDI) for the level 3 maternal causes, Females, 1990 to 2015

eFigure 5. Global pregnancy complication ratio (events per 100 live births) by type of complication and age group in 2015

eFigure 6a. Leading 25 GBD cause hierarchy level 3 causes of low SDI DALYs for both sexes combined for 1990, 2005 and 2015, 0-19 years

eFigure 6b. Leading 25 GBD cause hierarchy level 3 causes of low-middle SDI DALYs for both sexes combined for 1990, 2005 and 2015, 0-19 years

© 2017 Global Burden of Disease Child and Adolescent Health Collaboration. *JAMA Pediatrics.*

eFigure 6c. Leading 25 GBD cause hierarchy level 3 causes of middle SDI DALYs for both sexes combined for 1990, 2005 and 2015, 0-19 years

eFigure 6d. Leading 25 GBD cause hierarchy level 3 causes of middle-high SDI DALYs for both sexes combined for 1990, 2005 and 2015, 0-19 years

eFigure 6e. Leading 25 GBD cause hierarchy level 3 causes of high SDI DALYs for both sexes combined for 1990, 2005 and 2015, 0-19 years

eFigure 7a. The expected relationship between YLL and YLD rates with SDI for GBD level 3 congenital causes, aged 0 to 19 years, both sexes, 1990 to 2015

eFigure 7b. The expected relationship between YLL and YLD rates with SDI for GBD level 3 neonatal causes, aged 0 to 19 years, both sexes, 1990 to 2015

eFigure 8. The expected relationship between cause-specific all-ages DALY rates for 0- 19 years, and Sociodemographic Index (SDI) for males (left) and females (right)

eTable 1. Number of Deaths, Death Rates (per 100,000 population), and Cumulative Percent Change with 95% Uncertainty Intervals (UI) for the Top 10 Global Causes of Death in 195 Countries and Territories, Aged 0 to 19, Both Sexes, 1990 and 2015

eTable 2. Number of Deaths, Death Rates (per 100,000 population), and Cumulative Percent Change with 95% Uncertainty Intervals (UI) for the Top 10 Global Causes of Death in 195 Countries and Territories, Aged Under 5 Years, Both Sexes, 1990 and 2015

eTable 3. Prevalent Cases, Rates (per 100,000 population), Years Lived with Disability (YLDs), and Cumulative Percent Change with 95% Uncertainty Interval (UI) for the Top 10 Global Causes of YLDs in Children and Adolescents in 195 Countries and Territories, Aged 0 to 19 Years, Both Sexes, 1990 and 2015

eTable 4. Prevalent Cases, Rates (per 100,000 population), Years Lived with Disability (YLDs), and Cumulative Percent Change with 95% Uncertainty Interval (UI) for the Top 10 Global Causes of YLDs in Children and Adolescents in 195 Countries and Territories, Aged Under 5 Years, Both Sexes, 1990 and 2015

eTable 5. Prevalent Cases and Years Lived with Disability (YLDs), Percent Change, and Percent Change in Age-standardised Rates between 2005 and 2015 of Anemia by Cause, Aged 0 to 19 Years, Both Sexes, 2015

eTable 6. Prevalent Cases and Years Lived with Disability (YLDs), Percent Change, and Percent Change in Age-standardised Rates between 2005 and 2015 of Developmental Intellectual Disability by Cause, Aged 0 to 19 Years, Both Sexes, 2015

© 2017 Global Burden of Disease Child and Adolescent Health Collaboration. *JAMA Pediatrics.*

eTable 7. Prevalent Cases and Years Lived with Disability (YLDs), Percent Change, and Percent Change in Age-standardised Rates between 2005 and 2015 of Epilepsy by Cause, Aged 0 to 19, Both Sexes, 2015

eTable 8. Prevalent Cases and Years Lived with Disability (YLDs), Percent Change, and Percent Change in Age-standardised Rates between 2005 and 2015 of Hearing Loss by Cause, Aged 0 to 19, Both Sexes, 2015

eTable 9. Prevalent Cases and Years Lived with Disability (YLDs), Percent Change, and Percent Change in Age-standardised Rates between 2005 and 2015 of Vision Loss by Cause, Aged 0 to 19, Both Sexes, 2015

eTable 10. Number of Maternal Deaths, Maternal Mortality Ratio (MMR, number of deaths per 100,000 live births), and Annualized Rate of Change (ARC), in percent, for 195 Countries and Territories, Aged 10 to 19 Years, Females, 1990 to 2015

This supplementary material has been provided by the authors to give readers additional information about their work.

© 2017 Global Burden of Disease Child and Adolescent Health Collaboration. JAMA Pediatrics.

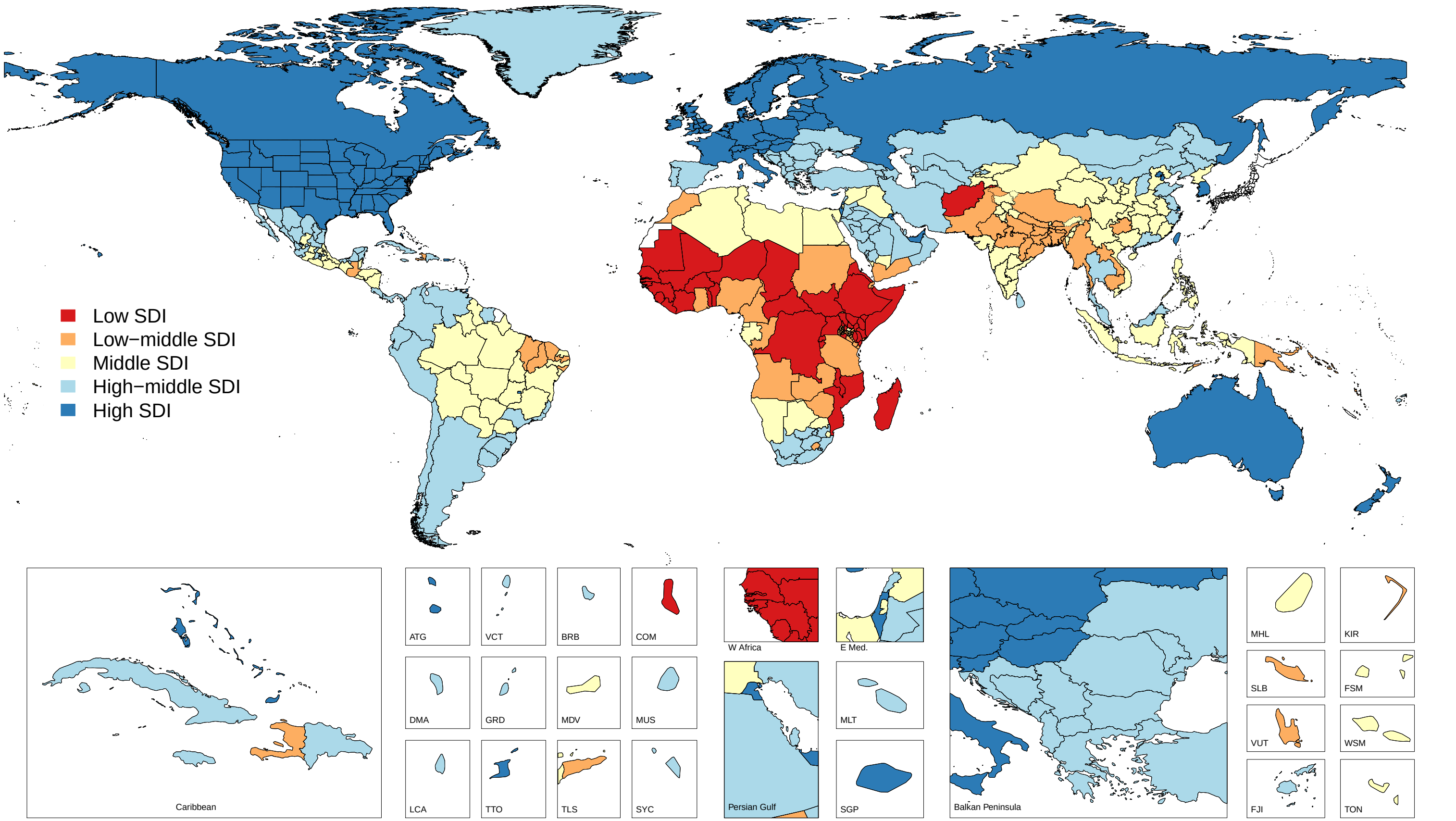

eFigure 1. Socio-Demographic Index quintiles by GBD subnational level 1 geography, 2015. SDI is calculated for each geography as a function of lag dependent income per capita, average educational attainment in the population over age 15, and the total fertility rate. SDI units are interpretable; a zero represents the lowest level of income per capita, educational attainment, and highest TFR observed 1980-2015 and a one represents the highest income per capita, educational attainment and lowest TFR observed in the same period. Cut-offs on the SDI scale for the quintiles have been selected based on examining the entire distribution of geographies 1980-2015. GBD = Global Burden of Disease. SDI = Socio-Demographic Index. TFR = total fertility rate. ATG = Antigua and Barbuda. VCT = Saint Vincent and the Grenadines. BRB = Barbados. COM = Comoros. DMA = Dominica. GRD = Grenada. MDV = Maldives. MUS = Mauritius. LCA = Saint Lucia. TTO = Trinidad and Tobago. SYC = Seychelles. MLT = Malta. SGP = Singapore. MHL = Marshall Islands. KIR = Kiribati. SLB = Solomon Islands. FSM = Federated States of Micronesia. VUT = Vanuatu. WSM = Samoa. FJI = Fiji. TON = Tonga.

© 2017 Global Burden of Disease Child and Adolescent Health Collaboration. JAMA Pediatrics.

eFigure 2a. Top 25 Global Causes of Death, by 5 SDI quintiles and 21 GBD regions, Aged 0-6 days, Females & Males, 2015

(A) Females

Cause	Global	High SDI	High-middle SDI	Middle SDI	Low-middle SDI	Low SDI	High-income North America	Australasia	High-income Asia Pacific	Western Europe	Southern Latin America	Eastern Europe	Central Europe	Central Asia	Central Latin America	Andean Latin America	Caribbean	Tropical Latin America	East Asia	Southeast Asia	Oceania	North Africa and Middle East	South Asia	Southern Sub-Saharan Africa	Western Sub-Saharan Africa	Eastern Sub-Saharan Africa	Central Sub-Saharan Africa
Neonatal preterm birth complications	1	1	1	1	2	1	1	2	2	1	1	2	1	1	1	1	1	1	1	1	1	1	2	1	2	2	2
Neonatal encephalopathy due to birth asphyxia and trauma	2	3	2	2	1	2	4	3	4	3	3	3	4	2	3	2	2	2	2	2	2	3	1	2	1	1	1
Congenital anomalies	3	2	3	3	4	5	2	1	1	2	2	1	2	3	2	4	4	3	3	3	5	2	3	5	5	6	5
Neonatal sepsis and other neonatal infections	4	5	5	4	3	3	5	5	5	5	5	5	5	6	4	3	3	5	6	4	7	5	5	4	3	3	3
Other neonatal disorders	5	4	4	5	5	4	3	4	3	4	4	4	3	4	5	5	5	4	4	5	3	4	4	3	4	4	4
Lower respiratory infections	6	6	6	6	6	6	7	6	7	6	6	6	6	5	6	6	6	6	5	6	4	6	6	6	6	5	6
Hemolytic disease and other neonatal jaundice	7	9	7	7	7	8	13	10	12	10	8	8	9	8	7	11	9	7	8	10	9	8	7	9	7	8	8
Sexually transmitted diseases excluding HIV	8	13	8	9	8	7	24	13	11	21	7	10	8	7	9	7	8	8	9	7	6	10	9	8	8	7	7
Diarrheal diseases	9	17	13	8	9	9	16	15	19	13	11	24	17	10	12	12	7	17	22	11	11	9	8	10	9	10	10
Meningitis	10	15	12	14	11	10	18	12	16	14	12	12	15	11	16	15	11	15	20	12	13	13	11	11	10	11	9
Tetanus	11	38	25	11	10	11	39	35	31	37	36	39	38	39	33	34	12	29	18	8	24	12	10	33	13	9	12
Other infectious diseases	12	7	9	13	12	12	14	9	14	11	22	7	13	9	19	27	26	10	27	9	14	23	12	7	12	12	11
Hemoglobinopathies and hemolytic anemias	13	24	26	24	13	13	22	16	29	28	27	29	22	12	30	33	25	31	17	24	35	17	22	21	11	13	14
Exposure to mechanical forces	14	14	14	12	18	17	12	14	13	18	16	17	25	19	22	13	22	23	7	25	19	25	16	17	18	16	22
Falls	15	26	24	26	15	14	25	29	26	29	26	18	29	13	28	26	36	22	30	16	31	21	19	30	14	14	27
Collective violence and legal intervention	16	40	36	15	14	16				34		13								39		7	35	37	21	29	31
Interpersonal violence	17	10	11	18	17	20	8	11	6	9	9	9	7	18	8	22	13	9	13	21	22	19	15	14	28	18	20
Cerebrovascular disease	18	22	17	17	16	19	26	37	25	20	19	30	12	23	11	20	10	20	12	22	10	16	18	12	15	28	23
Other cardiovascular and circulatory diseases	19	20	16	16	23	15	17	26	22	17	17	31	19	29	21	14	15	18	10	29	15	14	39	16	16	15	15
Foreign body	20	12	10	19	19	22	21	17	8	12	10	11	11	20	10	8	17	11	11	14	20	29	17	18	17	23	16
Cardiomyopathy and myocarditis	21	16	20	10	29	29	10	19	18	15	21	25	16	24	15	19	14	13	16	28	12	11	27	24	25	24	26
Road injuries	22	21	18	21	22	26	20	23	15	23	18	26	20	27	18	9	21	16	23	19	29	15	20	13	22	25	17
Other chronic respiratory diseases	23	34	21	23	20	24	38	34	28	22	23	34	30	26	20	32	18	32	41	13	8	27	13	36	34	21	33
Encephalitis	24	28	22	22	21	25	27	28	23	24	31	20	24	17	23	24	23	25	24	18	28	30	14	26	26	17	32
Endocrine, metabolic, blood, and immune disorders	25	8	15	20	28	30	6	7	9	7	13	14	14	15	14	25	19	12	14	15	21	18	30	15	20	27	24

(B) Males

Cause	Global	High SDI	High-middle SDI	Middle SDI	Low-middle SDI	Low SDI	High-income North America	Australasia	High-income Asia Pacific	Western Europe	Southern Latin America	Eastern Europe	Central Europe	Central Asia	Central Latin America	Andean Latin America	Caribbean	Tropical Latin America	East Asia	Southeast Asia	Oceania	North Africa and Middle East	South Asia	Southern Sub-Saharan Africa	Western Sub-Saharan Africa	Eastern Sub-Saharan Africa	Central Sub-Saharan Africa
Neonatal preterm birth complications	1	1	1	1	2	1	1	2	2	1	1	2	1	2	1	1	1	1	1	1	1	1	2	2	3	2	1
Neonatal encephalopathy due to birth asphyxia and trauma	2	4	2	2	1	2	4	3	4	3	4	3	4	1	3	2	2	2	2	2	2	3	1	1	2	1	2
Neonatal sepsis and other neonatal infections	3	5	5	4	3	3	5	5	5	5	5	5	5	6	4	3	3	5	6	4	7	5	5	4	1	3	3
Congenital anomalies	4	2	3	3	4	6	2	1	1	2	2	1	2	3	2	4	5	3	3	3	5	2	3	5	5	6	5
Other neonatal disorders	5	3	4	5	5	4	3	4	3	4	3	4	3	4	5	5	4	4	5	5	3	4	4	3	6	4	4
Lower respiratory infections	6	6	6	6	6	5	7	7	6	7	6	6	6	5	6	6	6	6	4	6	4	6	6	6	4	5	6
Hemolytic disease and other neonatal jaundice	7	9	7	7	7	8	11	12	10	9	8	8	8	8	7	10	7	7	7	12	8	7	7	9	10	8	8
Sexually transmitted diseases excluding HIV	8	11	8	9	8	7	23	14	9	22	7	9	7	7	9	7	9	8	10	8	6	10	9	7	8	7	7
Diarrheal diseases	9	17	13	8	9	10	14	18	19	12	13	24	16	10	12	13	8	16	24	9	10	8	8	10	7	10	10
Meningitis	10	15	10	13	10	9	19	13	14	13	12	12	15	11	16	17	10	15	20	10	11	13	11	12	9	9	9
Tetanus	11	37	21	12	11	11	40	35	33	39	35	41	37	40	31	31	14	29	16	13	26	12	10	32	12	11	12
Other infectious diseases	12	7	9	10	13	12	15	9	18	11	25	7	18	9	21	28	21	11	23	7	13	19	12	8	13	12	13
Hemoglobinopathies and hemolytic anemias	13	28	22	18	12	13	22	16	30	29	30	30	25	14	30	34	25	30	18	14	34	17	23	23	11	13	11
Cerebrovascular disease	14	23	18	14	15	17	27	34	24	19	20	29	12	22	10	23	11	22	13	11	9	16	16	15	14	28	23
Road injuries	15	21	17	17	14	16	20	24	15	23	18	27	21	27	19	8	19	13	19	21	29	15	15	11	16	17	16
Exposure to mechanical forces	16	18	11	16	21	15	16	11	13	18	16	14	22	18	20	15	23	17	8	27	22	27	24	17	15	16	21
Other cardiovascular and circulatory diseases	17	19	16	15	26	14	17	29	23	17	19	31	20	28	18	14	15	20	9	17	21	14	40	22	19	15	22
Foreign body	18	13	12	19	16	24	21	21	11	16	9	11	14	20	11	11	16	10	12	16	16	29	19	18	18	23	18
Interpersonal violence	19	10	14	20	18	23	8	10	7	10	11	10	9	21	8	22	13	9	17	28	18	18	17	13	29	19	24
Cardiomyopathy and myocarditis	20	14	20	11	30	31	9	19	16	14	17	17	13	25	14	19	17	14	15	19	27	11	29	26	31	27	26
Endocrine, metabolic, blood, and immune disorders	21	8	15	23	27	22	6	6	8	6	10	13	10	13	13	24	18	12	11	22	14	20	26	14	23	25	14
Collective violence and legal intervention	22	41	36	22	19	21				33		15								40		9	37	38	27	29	33
Falls	23	24	24	21	20	30	25	23	25	27	27	16	28	12	28	21	30	21	22	25	28	24	14	31	26	30	29
Encephalitis	24	27	25	24	25	18	29	30	22	25	28	21	19	16	23	27	27	28	26	24	20	25	18	28	32	14	34
Other chronic respiratory diseases	25	34	23	26	17	28	39	36	28	28	22	34	31	26	22	33	20	33	42	20	17	31	13	34	36	24	36

© 2017 Global Burden of Disease Child and Adolescent Health Collaboration. JAMA Pediatrics.

eFigure 2b. Ranking of the Top 25 Global Causes of Death for Global, by 5 SDI quintiles and 21 GBD regions, Aged 7-27 days, 2015

(A) Females

Cause	Global	High SDI	High-middle SDI	Middle SDI	Low-middle SDI	Low SDI	High-income North America	Australasia	High-income Asia Pacific	Western Europe	Southern Latin America	Eastern Europe	Central Europe	Central Asia	Central Latin America	Andean Latin America	Caribbean	Tropical Latin America	East Asia	Southeast Asia	Oceania	North Africa and Middle East	South Asia	Southern Sub-Saharan Africa	Western Sub-Saharan Africa	Eastern Sub-Saharan Africa	Central Sub-Saharan Africa
Neonatal preterm birth complications	1	2	1	1	1	4	2	3	2	2	2	3	2	3	3	2	2	3	2	1	1	1	1	3	4	5	6
Neonatal sepsis and other neonatal infections	2	3	3	4	2	1	4	5	3	5	3	2	5	5	2	1	1	1	6	2	3	3	3	1	1	1	1
Neonatal encephalopathy due to birth asphyxia and trauma	3	5	4	3	3	7	5	4	5	4	5	5	6	4	5	4	6	5	3	5	5	6	2	5	8	7	5
Congenital anomalies	4	1	2	2	5	3	1	1	1	1	1	1	1	2	1	3	3	2	1	3	6	2	5	7	6	3	3
Lower respiratory infections	5	6	6	5	4	2	8	8	6	7	6	6	4	1	4	5	5	6	4	4	2	4	4	4	2	2	2
Other neonatal disorders	6	4	5	6	6	5	3	2	4	3	4	4	3	6	6	6	4	4	5	6	4	5	6	2	7	4	4
Diarrheal diseases	7	13	7	7	7	6	11	10	13	9	8	12	10	7	7	7	7	7	15	7	8	7	7	6	5	6	9
Malaria	8	46	43	29	9	8			44		45			44	44	44	38	44	44	19	13	15	24	12	3	11	7
Hemolytic disease and other neonatal jaundice	9	14	8	8	8	11	20	21	21	16	14	11	15	14	10	13	11	8	8	14	16	11	8	11	11	13	11
Meningitis	10	12	9	10	10	9	13	11	10	10	10	10	8	10	11	10	8	10	14	10	11	10	10	9	9	8	10
Tetanus	11	41	18	9	11	12	41	39	36	41	35	42	41	42	28	35	9	27	13	8	28	9	9	36	13	10	13
Sexually transmitted diseases excluding HIV	12	20	14	16	13	10	31	19	19	34	11	15	11	9	12	9	10	13	10	13	7	13	11	10	12	9	8
Hemoglobinopathies and hemolytic anemias	13	30	23	18	12	14	34	36	27	35	32	32	26	19	35	36	32	35	17	9	21	18	17	28	10	15	15
Other infectious diseases	14	8	13	14	14	13	9	12	11	11	23	7	17	8	20	28	26	11	22	20	14	21	14	8	14	12	12
Exposure to mechanical forces	15	10	12	11	20	15	7	9	15	22	15	13	20	17	24	15	21	21	7	26	25	25	27	22	15	14	19
Sudden infant death syndrome	16	7	11	17	17	16	6	6	7	6	7	8	7	11	8	18	14	12	21	12	12	17	15	14	20	16	16
Encephalitis	17	19	16	12	15	21	24	25	22	19	27	14	14	12	17	14	29	22	24	18	22	22	12	25	25	17	29
Other chronic respiratory diseases	18	36	17	15	16	20	39	35	35	21	16	38	32	25	25	29	15	39	41	11	9	33	13	38	34	18	34
Foreign body	19	11	10	19	19	19	14	15	8	12	9	9	9	13	9	8	17	9	9	15	19	30	16	20	16	21	17
Collective violence and legal intervention	20	42	37	20	18	17				39		21								43		8	37	42	23	27	32
Cerebrovascular disease	21	26	22	21	21	24	28	28	16	25	22	31	19	24	13	19	12	23	19	25	10	16	18	15	17	35	25
Other cardiovascular and circulatory diseases	22	21	19	22	28	18	16	16	20	20	17	30	25	28	23	17	16	24	11	29	17	14	42	17	18	19	20
Cardiomyopathy and myocarditis	23	15	24	13	31	33	12	20	17	14	25	23	16	22	18	20	19	17	20	28	20	12	30	26	32	33	30
Road injuries	24	22	21	25	22	22	18	17	24	23	18	25	23	27	21	11	20	15	26	21	34	20	26	16	19	20	22
Endocrine, metabolic, blood, and immune disorders	25	9	15	23	29	29	10	7	9	8	12	16	12	18	14	25	18	14	12	16	26	19	32	13	24	28	21

(B) Males

Cause	Global	High SDI	High-middle SDI	Middle SDI	Low-middle SDI	Low SDI	High-income North America	Australasia	High-income Asia Pacific	Western Europe	Southern Latin America	Eastern Europe	Central Europe	Central Asia	Central Latin America	Andean Latin America	Caribbean	Tropical Latin America	East Asia	Southeast Asia	Oceania	North Africa and Middle East	South Asia	Southern Sub-Saharan Africa	Western Sub-Saharan Africa	Eastern Sub-Saharan Africa	Central Sub-Saharan Africa
Neonatal sepsis and other neonatal infections	1	3	3	3	1	1	4	5	3	4	3	2	6	5	2	1	1	1	6	2	3	3	3	1	1	1	1
Neonatal preterm birth complications	2	2	1	1	2	4	2	3	2	2	2	4	2	4	3	2	2	3	2	1	2	1	2	3	3	6	7
Neonatal encephalopathy due to birth asphyxia and trauma	3	5	4	4	3	6	5	4	5	5	5	5	5	2	5	4	6	5	4	5	7	6	1	5	6	7	5
Lower respiratory infections	4	6	6	5	4	2	7	9	6	8	6	6	4	1	4	5	4	6	3	4	1	4	4	4	2	2	2
Congenital anomalies	5	1	2	2	5	3	1	1	1	1	1	1	1	3	1	3	3	2	1	3	6	2	5	7	7	3	3
Other neonatal disorders	6	4	5	6	7	7	3	2	4	3	4	3	3	6	6	6	5	4	5	6	4	5	6	2	8	5	6
Diarrheal diseases	7	12	7	7	6	5	11	12	11	9	8	12	9	7	7	8	7	8	16	7	9	7	7	6	4	4	8
Meningitis	8	11	9	10	9	9	13	10	10	10	10	10	8	10	11	11	8	9	14	9	10	10	10	8	9	8	10
Malaria	9	46	43	28	10	8			44		45			45	44	45	34	44	45	22	14	16	27	13	5	11	4
Hemolytic disease and other neonatal jaundice	10	14	8	8	8	12	20	22	20	16	15	11	13	11	9	10	9	7	8	16	8	11	8	11	10	13	11
Tetanus	11	40	18	9	11	11	40	41	36	40	32	42	38	41	25	28	11	25	10	10	29	8	9	31	13	10	14
Sexually transmitted diseases excluding HIV	12	20	13	15	12	10	34	20	17	34	12	15	11	9	12	9	10	12	11	13	5	13	11	9	12	9	9
Hemoglobinopathies and hemolytic anemias	13	31	23	18	13	14	33	34	30	31	31	35	31	16	33	35	29	35	15	11	33	18	21	29	11	14	12
Other infectious diseases	14	8	14	11	14	13	9	11	12	11	23	7	16	8	19	30	22	13	21	8	13	22	14	10	14	12	13
Exposure to mechanical forces	15	10	10	12	16	15	8	8	15	17	14	14	20	15	23	14	19	18	7	26	21	25	17	17	15	15	16
Sudden infant death syndrome	16	7	12	17	17	17	6	6	7	6	7	8	7	14	8	15	16	11	19	12	11	20	15	16	18	17	17
Other chronic respiratory diseases	17	36	17	21	15	22	41	36	32	23	19	36	33	26	26	32	15	37	42	17	15	35	12	38	34	18	36
Encephalitis	18	19	16	14	19	16	24	23	21	19	26	13	15	12	17	20	27	24	24	18	16	21	13	27	28	16	26
Foreign body	19	13	11	20	18	19	14	17	9	13	9	9	10	13	10	7	14	10	9	14	19	27	16	19	17	20	18
Cerebrovascular disease	20	26	22	16	21	25	30	37	16	27	21	30	18	25	13	21	12	22	20	15	12	15	18	15	16	34	24
Road injuries	21	22	20	24	20	23	19	18	23	24	18	25	24	29	21	12	21	16	27	25	38	17	19	12	19	22	23
Endocrine, metabolic, blood, and immune disorders	22	9	15	23	26	18	10	7	8	7	11	16	12	17	14	19	17	14	13	23	17	19	29	14	25	21	15
Adverse effects of medical treatment	23	18	21	25	23	24	17	16	18	18	13	19	19	20	16	13	13	19	17	20	18	23	20	23	20	23	20
Collective violence and legal intervention	24	42	39	22	22	20				42		22								44		9	39	43	27	31	34
Other cardiovascular and circulatory diseases	25	21	19	19	28	21	16	26	22	22	17	29	28	27	24	17	18	23	12	19	22	14	42	22	22	19	22

© 2017 Global Burden of Disease Child and Adolescent Health Collaboration. JAMA Pediatrics.

eFigure 2c. Ranking of the Top 25 Global Causes of Death for Global, by 5 SDI quintiles and 21 GBD regions, Aged 28-364 days, 2015

(A) Females

Cause	Global	High SDI	High-middle SDI	Middle SDI	Low-middle SDI	Low SDI	High-income North America	Australasia	High-income Asia Pacific	Western Europe	Southern Latin America	Eastern Europe	Central Europe	Central Asia	Central Latin America	Andean Latin America	Caribbean	Tropical Latin America	East Asia	Southeast Asia	Oceania	North Africa and Middle East	South Asia	Southern Sub-Saharan Africa	Western Sub-Saharan Africa	Eastern Sub-Saharan Africa	Central Sub-Saharan Africa
Lower respiratory infections	1	3	2	1	1	1	5	3	4	4	2	2	2	1	2	1	2	2	2	1	1	2	1	3	3	1	1
Diarrheal diseases	2	7	4	3	2	3	7	10	11	10	6	10	7	3	3	4	1	3	5	3	5	3	2	2	2	3	3
Congenital anomalies	3	1	1	2	4	4	1	1	1	1	1	1	1	2	1	2	3	1	1	2	2	1	3	5	4	2	5
Malaria	4	72	58	27	3	2			67		65			63	62	64	43	57	70	19	15	14	20	9	1	4	2
Meningitis	5	13	9	5	5	6	14	16	17	11	7	7	8	9	9	10	4	7	10	4	9	6	5	6	5	7	6
Protein-energy malnutrition	6	33	10	10	6	5	39	38	44	38	10	28	33	20	4	7	5	5	8	18	10	11	9	4	6	6	4
HIV/AIDS	7	10	3	8	7	8	28	28	31	26	21	4	31	41	17	54	6	28	33	27	25	48	14	1	8	5	8
Other neonatal disorders	8	8	7	6	8	11	16	9	7	6	8	9	6	6	12	14	11	8	7	11	7	7	4	11	13	16	10
Neonatal preterm birth complications	9	4	5	4	10	16	4	4	3	3	4	6	3	5	6	5	10	4	4	6	11	4	7	7	28	18	15
Hemoglobinopathies and hemolytic anemias	10	41	29	18	9	10	40	46	38	40	38	42	28	10	46	39	28	38	28	21	32	19	10	25	7	10	16
Sexually transmitted diseases excluding HIV	11	32	22	28	13	7	49	35	30	48	23	18	15	14	20	15	14	27	17	22	4	18	13	8	10	8	7
Neonatal encephalopathy due to birth asphyxia and trauma	12	11	8	7	11	15	13	7	8	9	9	8	9	4	10	8	9	6	9	9	13	8	6	15	22	22	12
Whooping cough	13	38	24	17	12	9	34	43	47	34	15	34	24	19	22	22	7	32	16	15	8	9	11	17	9	9	9
Foreign body	14	5	6	13	16	17	8	13	5	8	3	5	5	7	5	3	8	10	6	8	18	31	17	22	12	17	17
Encephalitis	15	20	12	9	14	20	29	26	19	23	26	12	11	8	16	13	26	31	12	10	16	23	8	27	20	15	38
Measles	16	49	33	12	15	12	64	54	55	64	73	63	53	66	72	73	66	64	38	5	3	17	16	10	11	13	31
Sudden infant death syndrome	17	2	11	14	17	21	2	2	2	2	5	3	4	13	11	16	16	16	19	14	19	16	18	14	15	20	21
Neonatal sepsis and other neonatal infections	18	17	13	15	25	14	22	22	9	12	11	15	13	12	8	6	12	9	35	12	23	10	55	16	16	14	11
Other infectious diseases	19	18	27	31	18	13	15	11	24	17	32	16	18	11	30	33	36	22	36	32	30	30	28	19	14	11	13
Exposure to mechanical forces	20	6	14	11	24	26	3	5	6	18	13	11	19	16	28	12	24	21	3	24	28	29	29	29	17	27	22
Road injuries	21	15	15	16	20	23	11	14	15	20	19	19	17	25	18	9	19	12	13	29	38	12	21	13	18	24	20
Chronic obstructive pulmonary disease	22	35	19	21	19	33	30	23	36	27	35	55	38	36	23	34	15	39	43	17	12	24	15	39	38	35	24
Tuberculosis	23	54	38	36	27	19	55	56	42	55	42	38	48	37	50	35	35	43	53	37	51	43	24	23	33	19	19
Iron-deficiency anemia	24	59	47	46	28	18	56	55	57	57	58	58	59	52	67	67	65	66	58	64	29	58	33	46	47	12	14
Intestinal infectious diseases	25	57	21	20	21	46	61	57	61	58	61	54	64	46	56	62	57	55	47	16	41	52	12	66	35	40	52

(B) Males

Cause	Global	High SDI	High-middle SDI	Middle SDI	Low-middle SDI	Low SDI	High-income North America	Australasia	High-income Asia Pacific	Western Europe	Southern Latin America	Eastern Europe	Central Europe	Central Asia	Central Latin America	Andean Latin America	Caribbean	Tropical Latin America	East Asia	Southeast Asia	Oceania	North Africa and Middle East	South Asia	Southern Sub-Saharan Africa	Western Sub-Saharan Africa	Eastern Sub-Saharan Africa	Central Sub-Saharan Africa
Lower respiratory infections	1	3	2	1	1	1	5	3	4	4	2	2	2	1	2	1	1	1	2	1	1	2	1	3	3	1	1
Diarrheal diseases	2	7	4	3	2	2	7	9	9	10	6	10	7	3	3	4	2	3	5	3	5	3	2	2	1	3	3
Congenital anomalies	3	1	1	2	4	5	1	1	1	1	1	1	1	2	1	2	3	2	1	2	2	1	3	5	7	2	5
Malaria	4	72	64	34	3	3			67		68			67	64	67	43	61	70	29	16	12	29	12	2	4	2
Protein-energy malnutrition	5	34	11	12	6	4	40	36	43	39	13	28	32	18	4	7	4	4	22	22	6	13	8	4	4	5	4
Meningitis	6	13	9	4	5	6	15	12	19	11	7	7	9	8	9	11	5	8	9	4	8	5	4	6	5	7	6
HIV/AIDS	7	11	3	6	7	7	27	43	28	31	22	4	30	44	17	58	7	31	33	32	31	48	11	1	8	6	9
Hemoglobinopathies and hemolytic anemias	8	43	28	21	8	8	41	48	39	40	38	42	34	11	44	41	27	37	30	21	52	20	14	33	6	9	7
Neonatal preterm birth complications	9	4	5	5	10	15	4	4	3	3	4	6	3	6	6	6	11	5	4	6	13	4	7	8	26	19	17
Other neonatal disorders	10	9	7	8	9	13	14	10	7	6	9	9	6	5	12	12	10	7	6	12	10	7	5	10	16	16	11
Sexually transmitted diseases excluding HIV	11	33	25	30	12	9	51	34	31	49	23	20	16	15	22	14	16	28	20	25	7	18	17	9	9	8	8
Neonatal encephalopathy due to birth asphyxia and trauma	12	10	8	7	11	21	11	7	8	8	8	8	8	4	8	8	13	6	7	11	14	10	6	14	28	23	18
Whooping cough	13	41	31	23	13	10	36	39	44	37	19	39	28	22	25	28	9	42	21	17	11	9	15	21	10	10	10
Foreign body	14	6	6	11	14	16	8	16	5	9	3	5	5	7	5	3	6	10	8	7	12	30	13	18	12	17	13
Measles	15	51	34	13	15	12	64	51	55	63	73	63	53	66	72	73	66	66	39	5	3	14	16	11	11	15	28
Encephalitis	16	20	14	9	16	17	29	27	24	22	26	13	11	9	16	15	29	30	11	8	20	25	9	31	24	13	34
Sudden infant death syndrome	17	2	10	15	17	23	2	2	2	2	5	3	4	12	10	16	15	14	17	13	21	16	18	13	18	21	24
Neonatal sepsis and other neonatal infections	18	15	13	14	24	11	23	21	12	12	11	12	14	10	7	5	12	9	29	9	18	8	56	15	14	12	14
Exposure to mechanical forces	19	5	12	10	23	25	3	5	6	15	14	11	15	14	24	13	21	21	3	26	29	27	28	22	13	39	20
Road injuries	20	16	15	16	18	22	12	17	15	20	21	21	19	27	18	9	22	11	10	27	37	11	19	7	21	22	22
Other chronic respiratory diseases	21	19	17	20	21	27	10	22	16	21	12	51	29	36	13	21	8	18	41	14	4	34	20	29	29	25	21
Other infectious diseases	22	18	27	29	26	18	16	11	23	16	33	16	20	13	30	34	31	25	26	19	27	36	32	24	19	18	12
Drowning	23	27	38	28	20	24	21	19	22	30	36	25	36	24	36	17	19	35	28	15	30	22	23	32	20	27	19
Chronic obstructive pulmonary disease	24	35	26	26	19	30	31	26	34	29	35	54	39	35	26	35	14	41	51	16	9	26	12	39	40	31	26
Poisonings	25	40	40	44	31	14	32	40	42	45	31	33	43	31	43	19	30	49	31	47	28	28	39	26	15	14	23

© 2017 Global Burden of Disease Child and Adolescent Health Collaboration. JAMA Pediatrics.

eFigure 2d. Top 25 Global Causes of Death by 5 SDI, quintiles and 21 GBD regions, Aged 1-4 Years, Female & Male, 2015

(A) Females

Cause	Global	High SDI	High-middle SDI	Middle SDI	Low-middle SDI	Low SDI	High-income North America	Australasia	High-income Asia Pacific	Western Europe	Southern Latin America	Eastern Europe	Central Europe	Central Asia	Central Latin America	Andean Latin America	Caribbean	Tropical Latin America	East Asia	Southeast Asia	Oceania	North Africa and Middle East	South Asia	Southern Sub-Saharan Africa	Western Sub-Saharan Africa	Eastern Sub-Saharan Africa	Central Sub-Saharan Africa
Malaria	1	89	64	17	1	1			78		77			78	72	75	54	63	86	18	10	11	10	9	1	1	1
Lower respiratory infections	2	3	2	1	3	2	5	8	2	7	2	2	2	1	2	1	1	1	4	1	2	1	1	3	3	2	2
Diarrheal diseases	3	20	5	3	2	3	30	16	18	17	11	22	23	5	3	5	2	6	24	4	4	4	2	2	2	3	4
Protein-energy malnutrition	4	50	12	12	4	4	53	52	47	55	9	42	39	29	4	7	7	9	30	14	8	26	9	4	4	4	3
Congenital anomalies	5	1	1	2	6	6	1	1	1	1	1	1	1	2	1	2	3	2	1	3	6	3	8	8	7	7	7
Meningitis	6	13	11	9	5	5	14	15	22	11	10	9	11	11	12	10	4	8	11	8	9	6	4	10	5	5	5
Measles	7	45	15	5	7	9	72	53	52	74	89	69	52	72	90	90	80	70	22	2	1	8	7	5	8	9	19
Drowning	8	4	4	4	10	11	4	3	6	8	4	5	7	3	6	6	8	5	2	6	7	10	6	17	12	14	8
Intestinal infectious diseases	9	62	6	6	9	23	76	66	72	73	74	63	74	50	62	71	70	62	26	5	15	35	3	70	17	20	42
Whooping cough	10	58	24	15	12	8	71	71	64	68	38	56	28	19	38	17	5	23	14	13	5	9	11	14	10	10	10
Hemoglobinopathies and hemolytic anemias	11	33	27	21	8	12	32	41	36	29	27	37	31	13	47	50	17	30	20	22	58	19	13	31	6	11	15
Sexually transmitted diseases excluding HIV	12	31	22	28	13	7	56	40	29	58	15	19	13	12	18	12	11	17	19	21	3	14	15	11	11	6	6
Road injuries	13	2	3	7	15	13	2	2	3	3	3	3	4	7	5	4	6	3	3	15	19	5	12	7	13	17	9
HIV/AIDS	14	23	7	11	11	10	31	36	43	25	25	17	34	41	20	58	13	4	12	12	32	57	32	1	9	8	14
Encephalitis	15	18	9	8	14	32	28	27	16	20	21	15	10	6	13	13	27	28	8	9	11	30	5	28	29	22	44
Collective violence and legal intervention	16	60	56	10	16	19				47		13								68		2	66	63	28	35	52
Fire, heat, and hot substances	17	9	13	22	17	15	9	14	14	14	7	4	9	4	19	11	10	18	18	20	27	17	20	15	15	12	11
Tuberculosis	18	55	26	26	18	14	64	56	42	56	42	35	47	36	48	26	37	46	54	27	63	44	14	6	20	16	13
Falls	19	15	17	14	19	18	22	21	10	16	28	11	20	10	31	31	45	19	9	19	38	7	16	48	24	27	28
Foreign body	20	11	10	18	20	20	12	11	7	9	5	12	8	9	7	3	14	13	13	17	16	23	22	26	14	19	23
Other infectious diseases	21	21	33	34	22	16	16	17	19	21	31	24	27	21	34	27	41	42	38	41	34	49	18	18	27	15	16
Leukemia	22	5	8	13	30	35	7	7	5	2	6	7	5	8	8	8	16	7	5	11	24	15	28	29	48	26	40
Asthma	23	35	25	19	25	22	24	29	31	41	44	71	59	54	10	35	9	14	46	10	12	27	23	16	32	21	17
Paralytic ileus and intestinal obstruction	24	36	34	25	23	26	38	35	37	37	33	34	32	26	17	18	12	36	44	16	45	38	21	25	16	32	22
Cirrhosis and other chronic liver diseases due to other causes	25	28	28	23	21	33	27	38	26	27	30	29	26	25	24	24	24	27	40	25	41	21	17	35	21	34	37

(B) Males

Cause	Global	High SDI	High-middle SDI	Middle SDI	Low-middle SDI	Low SDI	High-income North America	Australasia	High-income Asia Pacific	Western Europe	Southern Latin America	Eastern Europe	Central Europe	Central Asia	Central Latin America	Andean Latin America	Caribbean	Tropical Latin America	East Asia	Southeast Asia	Oceania	North Africa and Middle East	South Asia	Southern Sub-Saharan Africa	Western Sub-Saharan Africa	Eastern Sub-Saharan Africa	Central Sub-Saharan Africa
Malaria	1	89	68	25	1	1			82		82			84	74	78	40	72	87	25	10	12	16	10	1	3	1
Lower respiratory infections	2	4	2	1	3	3	8	8	3	8	3	3	2	1	1	1	1	1	4	1	2	1	1	3	3	2	2
Diarrheal diseases	3	20	5	4	2	2	32	17	18	17	13	23	26	4	3	4	2	8	24	2	4	5	2	2	2	1	4
Protein-energy malnutrition	4	53	12	12	4	4	54	54	45	58	14	42	43	29	5	7	5	9	45	15	5	20	8	4	4	4	3
Meningitis	5	14	11	9	5	5	14	14	22	10	10	10	11	10	12	10	4	6	11	8	9	7	4	9	5	5	7
Congenital anomalies	6	1	1	2	10	9	1	1	1	1	1	1	1	2	2	2	3	2	2	5	8	3	10	8	9	6	11
Drowning	7	3	4	3	6	10	3	2	4	7	2	2	6	3	6	6	6	4	1	3	3	6	5	13	11	13	6
Measles	8	46	19	7	9	8	72	53	53	74	90	72	56	77	90	91	80	75	29	4	1	8	7	7	7	9	22
Road injuries	9	2	3	5	11	12	2	3	2	2	4	5	3	7	4	3	7	3	3	11	14	4	9	5	15	15	9
Hemoglobinopathies and hemolytic anemias	10	37	25	19	7	6	31	41	40	37	36	41	34	12	49	44	13	28	20	19	57	21	14	33	6	10	5
Intestinal infectious diseases	11	61	6	6	8	25	74	68	72	75	68	67	75	58	62	70	70	64	25	6	17	34	3	68	18	23	43
HIV/AIDS	12	25	8	13	12	13	35	49	38	33	29	20	35	46	23	64	14	5	14	13	36	59	35	1	8	7	14
Sexually transmitted diseases excluding HIV	13	40	29	34	15	7	60	43	34	61	19	22	20	14	25	13	12	27	28	29	6	24	17	12	12	8	8
Whooping cough	14	59	33	20	13	11	66	71	59	63	46	60	33	25	45	23	8	40	22	17	7	11	11	19	10	11	13
Encephalitis	15	17	9	8	14	29	27	22	14	18	20	14	10	6	16	12	26	31	9	9	16	32	6	27	30	22	42
Foreign body	16	11	10	15	16	16	12	12	8	9	5	11	9	11	8	5	11	13	10	14	13	17	20	16	14	16	17
Collective violence and legal intervention	17	64	58	10	17	23				50		15								73		2	64	64	35	40	53
Fire, heat, and hot substances	18	8	15	24	20	15	5	11	12	12	7	4	8	5	19	11	10	17	16	28	15	15	27	15	16	18	10
Falls	19	12	13	14	18	21	15	16	7	13	25	9	12	9	21	21	38	16	7	18	37	9	15	44	20	24	30
Tuberculosis	20	55	23	23	19	14	65	56	48	59	44	34	45	37	47	26	37	45	39	22	48	45	12	6	25	14	12
Exposure to mechanical forces	21	13	14	16	22	17	11	10	17	14	12	16	13	13	18	9	18	20	6	26	31	13	37	14	13	29	16
Leukemia	22	5	7	11	27	31	10	5	5	4	6	7	5	8	7	8	16	7	5	10	20	16	22	29	28	25	37
Poisonings	23	15	21	33	26	18	18	23	36	31	16	6	21	15	32	17	17	43	13	55	32	23	33	26	19	21	15
Epilepsy	24	19	22	31	25	22	26	15	15	16	31	31	15	16	22	36	30	24	19	33	43	26	29	20	22	19	20
Other infectious diseases	25	24	37	37	24	19	22	19	23	21	38	26	36	26	38	32	27	46	38	37	23	41	21	24	31	17	18

© 2017 Global Burden of Disease Child and Adolescent Health Collaboration. JAMA Pediatrics.

eFigure 2e. Ranking of the Top 25 Global Causes of Death for Global, by 5 SDI quintiles and 21 GBD regions, Aged 5-9 years, 2015

(A) Females

Cause	Global	High SDI	High-middle SDI	Middle SDI	Low-middle SDI	Low SDI	High-income North America	Australasia	High-income Asia Pacific	Western Europe	Southern Latin America	Eastern Europe	Central Europe	Central Asia	Central Latin America	Andean Latin America	Caribbean	Tropical Latin America	East Asia	Southeast Asia	Oceania	North Africa and Middle East	South Asia	Southern Sub-Saharan Africa	Western Sub-Saharan Africa	Eastern Sub-Saharan Africa	Central Sub-Saharan Africa
Lower respiratory infections	1	7	4	3	2	2	10	11	6	7	5	6	5	1	4	1	3	3	5	3	1	4	3	4	2	2	1
Intestinal infectious diseases	2	57	5	1	1	9	76	71	78	73	78	68	76	56	60	71	72	69	13	1	8	17	1	57	8	10	21
HIV/AIDS	3	26	2	6	4	1	31	28	49	27	29	15	32	50	21	60	4	12	19	19	24	55	12	1	3	1	3
Diarrheal diseases	4	31	14	7	3	4	34	27	30	26	35	40	36	20	7	12	5	15	29	8	3	16	2	3	4	3	7
Malaria	5	85	49	17	5	3			75		76			71	71	70	70	59	75	12	4	38	5	29	1	6	2
Road injuries	6	1	1	2	9	7	1	1	1	3	1	1	1	2	1	2	1	1	1	6	9	2	9	2	11	8	5
Congenital anomalies	7	2	3	5	6	8	2	2	3	1	2	2	2	5	2	4	2	2	3	5	6	3	7	9	7	7	8
Meningitis	8	20	12	11	7	5	21	22	26	17	12	16	21	11	15	9	9	9	12	10	11	12	6	7	5	5	4
Drowning	9	6	7	4	8	11	9	7	8	10	7	3	7	3	9	6	6	5	2	4	10	8	8	14	13	11	9
Leukemia	10	4	6	8	15	23	5	4	4	2	3	5	4	4	3	3	7	4	4	7	12	5	17	20	19	19	23
Sexually transmitted diseases excluding HIV	11	41	37	39	12	6	69	46	39	69	27	25	26	17	31	18	18	40	28	34	5	23	21	12	9	4	6
Encephalitis	12	19	9	9	10	38	27	21	17	19	17	14	11	7	18	13	26	30	18	11	13	29	4	25	34	33	41
Hemoglobinopathies and hemolytic anemias	13	28	24	22	11	13	24	29	23	23	33	35	28	14	27	43	10	17	16	13	36	14	27	32	6	21	13
Measles	14	69	38	15	13	12	77	69	67	79	91	78	70	84	92	91	87	84	41	9	2	13	15	8	10	12	27
Protein-energy malnutrition	15	56	29	29	19	10	65	65	52	63	21	52	51	58	10	17	16	20	51	17	17	39	24	10	12	9	14
Falls	16	15	15	14	18	16	26	20	12	18	24	13	19	12	22	20	44	14	7	20	41	6	13	43	16	14	32
Tuberculosis	17	53	23	25	14	14	60	49	31	54	45	42	49	32	46	22	37	47	37	28	37	33	11	5	20	13	11
Fire, heat, and hot substances	18	10	13	24	17	18	8	13	9	14	8	8	14	8	20	10	8	16	20	24	18	19	14	13	17	15	12
Collective violence and legal intervention	19	67	57	10	33	15				53		11								70		1	60	64	23	31	43
Animal contact	20	49	33	16	16	19	43	40	44	52	53	45	42	41	42	31	39	37	38	32	30	31	10	30	21	18	17
Brain and nervous system cancer	21	3	8	13	29	36	3	3	5	4	6	4	3	6	6	8	12	6	6	14	33	10	29	22	30	28	37
Other neoplasms	22	5	10	20	25	21	4	6	2	5	4	9	6	16	8	16	11	8	10	15	28	24	31	17	24	16	20
Foreign body	23	14	18	28	21	24	14	17	11	13	10	17	18	10	12	5	29	18	31	16	23	22	28	19	14	25	29
Exposure to mechanical forces	24	11	16	21	27	25	11	12	18	12	11	12	12	18	13	7	17	13	8	27	19	11	38	15	18	27	18
Epilepsy	25	12	17	23	24	34	20	9	10	8	16	20	8	9	16	23	24	23	15	29	25	15	20	16	45	40	19

(B) Males

Cause	Global	High SDI	High-middle SDI	Middle SDI	Low-middle SDI	Low SDI	High-income North America	Australasia	High-income Asia Pacific	Western Europe	Southern Latin America	Eastern Europe	Central Europe	Central Asia	Central Latin America	Andean Latin America	Caribbean	Tropical Latin America	East Asia	Southeast Asia	Oceania	North Africa and Middle East	South Asia	Southern Sub-Saharan Africa	Western Sub-Saharan Africa	Eastern Sub-Saharan Africa	Central Sub-Saharan Africa
Lower respiratory infections	1	7	7	4	2	3	11	14	7	8	7	5	6	1	4	2	4	5	6	4	1	4	3	4	2	3	2
Intestinal infectious diseases	2	57	5	3	1	12	77	69	78	76	76	68	77	64	60	72	65	73	13	2	9	15	1	57	6	11	19
Road injuries	3	1	1	1	6	6	1	1	1	3	1	2	1	3	1	1	1	1	2	3	5	1	4	2	8	5	5
Diarrheal diseases	4	35	15	7	3	4	38	34	31	27	39	42	38	21	7	9	7	19	31	6	4	20	2	3	4	2	6
HIV/AIDS	5	30	3	8	4	1	30	40	36	26	26	25	33	50	23	60	5	17	21	23	29	57	14	1	3	1	3
Drowning	6	2	2	2	7	7	4	5	3	6	5	1	4	2	5	4	3	2	1	1	2	5	7	7	7	6	7
Malaria	7	85	50	26	5	2			80		81			73	71	75	68	64	79	17	6	41	9	29	1	8	1
Congenital anomalies	8	3	4	5	9	11	2	3	5	2	3	3	2	5	3	5	2	3	4	7	10	3	5	10	11	7	11
Meningitis	9	21	13	11	8	5	19	22	25	18	16	21	23	9	17	8	10	8	14	9	8	11	8	8	5	4	8
Leukemia	10	4	6	6	14	24	5	2	2	1	2	4	3	4	2	3	6	4	3	8	11	6	12	20	19	22	18
Encephalitis	11	18	11	10	10	28	25	19	19	19	20	14	14	8	18	18	24	30	17	11	22	27	6	24	29	28	34
Hemoglobinopathies and hemolytic anemias	12	28	27	21	11	10	24	30	27	24	33	39	28	14	30	39	8	20	23	19	50	13	34	32	9	12	4
Falls	13	12	9	12	12	16	22	16	9	14	18	11	11	7	15	15	26	10	5	12	26	9	13	35	15	13	27
Exposure to mechanical forces	14	11	10	14	15	15	10	8	16	11	9	9	9	13	12	7	15	11	8	24	18	10	16	9	13	18	14
Protein-energy malnutrition	15	59	34	34	18	8	68	65	56	64	31	50	51	60	11	19	14	24	45	25	36	48	37	11	10	9	9
Sexually transmitted diseases excluding HIV	16	50	44	50	20	9	70	50	44	70	34	29	29	26	43	28	23	44	35	45	7	29	31	19	14	10	10
Measles	17	70	39	19	16	13	78	68	67	79	91	81	71	86	92	91	87	87	43	10	3	17	19	13	12	14	32
Animal contact	18	39	33	17	13	20	39	28	45	46	44	31	36	36	32	37	44	29	34	28	14	37	10	26	23	19	15
Collective violence and legal intervention	19	62	59	9	38	14				49		15								73		2	58	62	27	33	42
Tuberculosis	20	54	24	20	17	19	62	54	34	56	45	43	47	35	47	23	37	46	32	13	55	45	11	5	38	15	12
Other unintentional injuries	21	22	22	27	19	17	37	29	39	33	15	13	19	12	31	22	20	12	15	31	37	7	15	33	24	26	30
Foreign body	22	14	16	22	21	21	17	17	14	16	8	16	13	16	10	6	21	15	27	14	24	14	24	14	16	21	21
Brain and nervous system cancer	23	5	8	15	30	37	3	4	6	4	6	6	5	6	8	10	11	7	7	18	28	12	33	28	33	32	37
Fire, heat, and hot substances	24	9	17	31	23	18	9	15	11	15	10	7	16	11	21	16	12	18	24	35	16	18	25	15	18	17	13
Other neoplasms	25	6	14	23	29	22	6	7	4	5	4	10	7	18	9	17	13	9	10	21	25	23	35	18	32	16	22

© 2017 Global Burden of Disease Child and Adolescent Health Collaboration. JAMA Pediatrics.

eFigure 2f. Ranking of the Top 25 Global Causes of Death for Global, by 5 SDI quintiles and 21 GBD regions, Aged 10-14 years, 2015

(A) Females

Cause	Global	High SDI	High-middle SDI	Middle SDI	Low-middle SDI	Low SDI	High-income North America	Australasia	High-income Asia Pacific	Western Europe	Southern Latin America	Eastern Europe	Central Europe	Central Asia	Central Latin America	Andean Latin America	Caribbean	Tropical Latin America	East Asia	Southeast Asia	Oceania	North Africa and Middle East	South Asia	Southern Sub-Saharan Africa	Western Sub-Saharan Africa	Eastern Sub-Saharan Africa	Central Sub-Saharan Africa
HIV/AIDS	1	33	1	3	1	1	35	37	54	39	33	19	31	45	24	58	1	12	19	22	25	52	10	1	1	1	1
Intestinal infectious diseases	2	61	6	1	2	8	86	75	86	83	87	72	82	63	60	80	79	82	15	1	12	22	1	64	7	7	20
Lower respiratory infections	3	9	5	6	3	2	9	14	7	8	6	8	5	1	6	2	4	6	9	3	1	4	2	3	3	2	3
Road injuries	4	1	2	2	8	6	1	1	4	1	1	1	1	4	1	1	2	1	1	5	10	2	6	2	8	5	5
Diarrheal diseases	5	43	20	11	4	5	45	44	41	36	46	49	53	31	16	24	13	34	37	8	3	27	3	4	5	3	6
Congenital anomalies	6	2	4	5	7	7	2	2	5	2	2	3	2	6	4	5	3	5	4	4	7	3	4	9	9	6	9
Meningitis	7	24	13	10	6	3	25	23	32	21	15	22	24	11	19	14	16	10	18	9	5	16	7	7	4	4	4
Drowning	8	8	7	4	9	9	10	11	9	12	8	2	7	2	8	6	5	3	2	2	9	8	9	11	10	8	8
Malaria	9	92	47	25	5	4			80		83			72	66	75	66	68	76	10	6	40	8	34	2	11	2
Leukemia	10	3	3	7	14	21	5	3	2	3	3	6	4	3	2	3	6	4	3	6	13	6	17	13	16	18	18
Tuberculosis	11	53	19	20	10	10	67	57	36	56	42	43	50	26	37	15	35	40	24	16	48	26	11	6	12	9	7
Hemoglobinopathies and hemolytic anemias	12	27	26	24	12	11	27	34	22	24	34	36	26	16	26	34	12	18	20	15	47	13	28	31	6	13	12
Self-harm	13	5	8	12	13	29	3	7	1	9	4	4	8	8	5	10	11	9	5	30	11	30	12	36	34	20	22
Encephalitis	14	26	14	9	11	41	30	33	23	23	32	21	15	13	22	21	30	42	27	13	37	29	5	25	47	30	46
Other neoplasms	15	4	10	14	17	14	4	5	3	4	5	9	6	12	7	13	7	7	8	12	18	14	25	14	14	10	11
Interpersonal violence	16	7	9	13	29	26	6	9	8	10	9	5	11	20	3	7	8	2	11	20	19	18	29	5	43	17	29
Epilepsy	17	11	12	18	16	31	19	10	10	7	19	18	9	5	13	17	21	20	12	18	56	12	14	8	51	40	13
Collective violence and legal intervention	18	72	57	8	39	12				58		11								77		1	63	70	26	35	43
Cirrhosis and other chronic liver diseases due to other causes	19	31	21	23	15	23	32	28	27	31	18	26	20	9	20	20	17	17	32	24	24	17	13	30	17	23	26
Cerebrovascular disease	20	17	16	17	22	18	15	21	16	13	11	27	10	29	11	9	9	11	13	21	4	7	26	10	15	28	19
Chronic kidney disease	21	25	15	19	18	25	31	36	19	28	17	28	17	10	9	11	14	15	21	14	16	9	20	18	23	26	28
Falls	22	18	24	21	21	16	28	24	18	19	30	10	22	19	29	22	47	27	10	25	57	20	18	48	21	15	38
Brain and nervous system cancer	23	6	11	15	31	37	7	4	6	5	7	7	3	7	10	12	18	8	6	17	38	10	31	28	32	31	41
Rheumatic heart disease	24	36	25	16	20	35	39	29	31	37	29	39	35	15	46	25	10	22	25	11	2	23	16	17	44	38	25
Exposure to mechanical forces	25	16	17	26	23	28	16	15	25	14	10	13	19	25	15	8	19	21	14	27	14	11	24	12	18	39	21

(B) Males

Cause	Global	High SDI	High-middle SDI	Middle SDI	Low-middle SDI	Low SDI	High-income North America	Australasia	High-income Asia Pacific	Western Europe	Southern Latin America	Eastern Europe	Central Europe	Central Asia	Central Latin America	Andean Latin America	Caribbean	Tropical Latin America	East Asia	Southeast Asia	Oceania	North Africa and Middle East	South Asia	Southern Sub-Saharan Africa	Western Sub-Saharan Africa	Eastern Sub-Saharan Africa	Central Sub-Saharan Africa
HIV/AIDS	1	34	2	4	1	1	34	51	45	33	29	30	31	50	27	59	2	15	22	26	27	52	8	1	1	1	1
Road injuries	2	1	1	1	3	4	1	1	2	1	1	1	1	3	1	1	1	1	2	1	2	2	2	2	8	4	3
Drowning	3	3	3	2	6	7	8	9	4	8	3	2	2	2	4	4	3	3	1	2	1	3	5	9	6	6	5
Intestinal infectious diseases	4	58	6	3	2	11	79	72	79	78	80	71	80	73	60	76	73	80	16	3	8	21	1	58	7	11	23
Lower respiratory infections	5	11	8	6	4	3	12	18	8	13	9	10	7	1	6	2	6	5	8	4	3	5	4	5	3	2	4
Diarrheal diseases	6	43	23	13	5	6	46	46	41	41	50	51	54	31	14	21	20	31	38	8	5	30	3	4	5	3	7
Meningitis	7	25	14	11	8	5	23	27	31	24	22	24	25	13	19	14	16	11	19	9	9	14	7	7	4	5	8
Malaria	8	87	51	30	7	2			77		79			77	75	77	68	66	79	25	6	46	9	30	2	9	2
Congenital anomalies	9	5	5	7	9	9	4	3	6	3	5	4	5	8	5	7	4	6	4	5	12	4	13	11	10	7	10
Leukemia	10	4	4	5	13	23	5	2	3	2	2	6	4	5	3	3	7	4	3	6	16	8	16	16	18	20	18
Falls	11	14	13	10	10	12	21	17	12	12	19	11	11	7	16	15	24	10	5	10	28	11	14	37	11	8	28
Self-harm	12	2	9	14	14	16	2	4	1	6	4	3	3	4	7	10	11	13	6	33	4	23	11	36	14	13	15
Interpersonal violence	13	7	7	9	19	18	3	11	10	15	7	5	16	18	2	5	5	2	12	17	15	10	26	3	37	16	24
Encephalitis	14	24	17	12	11	33	25	22	25	23	30	23	21	17	23	28	31	42	28	18	19	31	6	28	36	25	40
Hemoglobinopathies and hemolytic anemias	15	28	26	19	17	8	29	33	24	26	33	35	28	15	29	23	12	23	25	12	52	12	36	31	9	10	6
Collective violence and legal intervention	16	60	56	8	34	10				49		14								71		1	59	59	20	32	34
Other unintentional injuries	17	20	19	25	12	14	30	21	38	27	12	9	15	10	22	18	14	12	15	19	17	6	12	32	23	23	26
Tuberculosis	18	56	27	22	16	13	65	55	42	58	49	47	50	38	43	20	40	45	29	11	36	43	15	6	15	12	9
Exposure to mechanical forces	19	9	11	16	21	17	9	8	19	9	8	8	9	12	9	8	8	9	9	24	13	7	38	8	12	24	13
Other neoplasms	20	8	12	18	27	19	7	7	5	4	6	12	8	20	8	11	10	7	10	14	25	17	29	14	31	15	19
Cerebrovascular disease	21	22	15	15	22	24	18	26	17	19	13	29	14	33	12	9	9	14	14	23	7	9	24	13	19	33	27
Animal contact	22	53	36	26	15	20	53	38	55	56	45	39	39	43	35	40	43	37	39	30	21	36	10	24	25	18	16
Epilepsy	23	17	16	20	18	30	22	13	11	11	23	21	10	6	15	19	15	20	13	20	24	15	17	10	38	38	12
Brain and nervous system cancer	24	6	10	17	30	39	6	5	7	5	10	7	6	11	10	12	19	8	7	22	34	13	33	25	32	34	38
Foreign body	25	19	18	29	23	21	19	15	18	17	11	17	20	19	13	6	22	18	35	21	30	16	28	12	13	22	25

© 2017 Global Burden of Disease Child and Adolescent Health Collaboration. JAMA Pediatrics.

eFigure 2g. Ranking of the Top 25 Global Causes of Death for Global, by 5 SDI quintiles and 21 GBD regions, Aged 15-19 years, 2015

(A) Females

Cause	Global	High SDI	High-middle SDI	Middle SDI	Low-middle SDI	Low SDI	High-income North America	Australasia	High-income Asia Pacific	Western Europe	Southern Latin America	Eastern Europe	Central Europe	Central Asia	Central Latin America	Andean Latin America	Caribbean	Tropical Latin America	East Asia	Southeast Asia	Oceania	North Africa and Middle East	South Asia	Southern Sub-Saharan Africa	Western Sub-Saharan Africa	Eastern Sub-Saharan Africa	Central Sub-Saharan Africa
Road injuries	1	1	1	1	5	7	1	1	2	1	2	1	1	2	2	2	1	1	1	1	3	2	3	2	10	8	4
Self-harm	2	2	2	2	1	19	2	2	1	2	1	2	2	1	3	1	3	3	2	3	2	4	1	6	19	21	10
HIV/AIDS	3	36	3	7	2	1	34	52	66	46	23	24	31	39	17	38	2	11	21	28	32	59	15	1	1	1	2
Malaria	4	117	57	22	6	2			104		112			108	92	91	44	79	88	6	1	8	10	12	2	4	1
Lower respiratory infections	5	10	6	4	4	6	10	14	7	11	5	10	6	3	7	3	6	4	16	7	4	6	5	5	6	5	6
Diarrheal diseases	6	63	23	16	3	4	59	64	55	54	63	82	74	51	25	42	27	51	59	18	7	41	4	7	5	2	11
Intestinal infectious diseases	7	100	9	3	7	18	112	103	109	111	115	108	113	98	82	110	106	111	24	2	18	38	2	87	13	17	38
Tuberculosis	8	53	10	6	8	5	91	79	37	68	38	30	47	16	29	9	22	32	19	4	30	20	7	3	11	6	7
Maternal hemorrhage	9	79	43	31	12	3	68	89	97	100	59	69	85	38	23	22	31	34	44	17	5	10	21	13	3	3	3
Maternal hypertensive disorders	10	62	31	20	9	9	55	67	96	92	24	61	76	37	15	13	16	14	54	25	37	19	9	10	4	9	5
Drowning	11	12	16	10	10	24	15	13	10	15	9	5	9	8	13	11	9	9	4	8	10	15	8	31	32	32	16
Fire, heat, and hot substances	12	22	7	14	11	32	21	31	18	30	18	19	28	13	43	23	26	41	35	34	17	18	6	20	39	30	22
Meningitis	13	31	19	17	13	8	32	27	46	22	34	37	34	22	33	25	29	19	30	16	16	36	12	8	7	7	8
Interpersonal violence	14	3	4	9	23	20	3	8	9	10	3	3	8	12	1	4	4	2	12	14	13	11	24	4	38	14	18
Congenital anomalies	15	4	8	12	20	21	6	6	5	3	7	6	5	14	6	14	5	10	5	9	41	5	19	24	25	18	21
Cerebrovascular disease	16	13	14	11	17	16	13	18	11	12	8	15	11	20	10	6	7	7	6	11	8	3	18	17	15	20	17
Leukemia	17	5	5	8	28	33	7	7	3	4	4	7	4	7	4	5	13	5	3	5	20	12	31	25	34	35	32
Ischemic heart disease	18	18	12	15	19	28	20	33	13	20	10	17	14	9	11	7	14	15	10	22	12	7	14	36	33	25	36
Chronic kidney disease	19	23	11	13	22	29	28	25	20	26	12	23	17	6	5	8	8	13	17	10	9	9	17	21	21	38	35
Collective violence and legal intervention	20	92	63	5	41	10				69		11								86		1	75	74	18	26	33
Indirect maternal deaths	21	56	39	39	14	11	43	39	95	72	35	57	80	65	16	48	24	17	73	53	47	28	27	11	8	12	9
Maternal abortion, miscarriage, and ectopic pregnancy	22	60	41	26	16	13	48	82	92	78	25	54	56	48	27	26	11	31	46	15	14	21	22	29	12	16	13
Other maternal disorders	23	43	42	28	15	12	29	45	93	58	52	65	66	58	34	50	30	27	37	20	11	24	23	14	14	13	12
Other neoplasms	24	6	15	24	32	17	5	4	4	5	6	9	3	11	9	17	10	6	9	12	22	23	35	16	37	11	19
Cirrhosis and other chronic liver diseases due to other causes	25	30	20	23	24	23	33	35	33	40	28	20	16	5	22	19	18	21	32	24	23	22	20	34	17	27	29

(B) Males

Cause	Global	High SDI	High-middle SDI	Middle SDI	Low-middle SDI	Low SDI	High-income North America	Australasia	High-income Asia Pacific	Western Europe	Southern Latin America	Eastern Europe	Central Europe	Central Asia	Central Latin America	Andean Latin America	Caribbean	Tropical Latin America	East Asia	Southeast Asia	Oceania	North Africa and Middle East	South Asia	Southern Sub-Saharan Africa	Western Sub-Saharan Africa	Eastern Sub-Saharan Africa	Central Sub-Saharan Africa
Road injuries	1	1	1	1	1	1	1	1	2	1	2	2	1	2	2	1	1	2	1	1	2	2	1	3	3	2	1
Interpersonal violence	2	3	2	3	7	5	3	6	13	9	3	3	8	4	1	2	2	1	8	4	6	3	10	2	12	4	7
Self-harm	3	2	3	4	2	11	2	2	1	2	1	1	2	1	3	3	4	4	3	3	1	4	2	4	9	13	4
Collective violence and legal intervention	4	51	36	2	13	2				38		6								66		1	44	49	7	12	14
Drowning	5	5	4	5	3	10	5	7	4	7	4	4	3	3	4	5	3	3	2	2	3	5	4	10	13	11	6
HIV/AIDS	6	40	8	17	5	3	37	56	54	43	33	37	41	47	19	38	5	15	32	28	34	56	23	1	2	1	3
Lower respiratory infections	7	15	6	7	4	9	17	21	10	16	8	11	6	5	8	4	8	5	15	7	5	11	6	6	4	8	5
Diarrheal diseases	8	62	33	18	6	6	57	65	53	57	59	70	76	49	23	33	30	43	55	17	12	38	7	7	6	3	8
Malaria	9	111	56	28	9	4			98		104			106	87	94	49	79	96	10	4	27	15	13	1	6	2
Intestinal infectious diseases	10	87	14	6	8	19	104	97	102	102	105	101	105	94	74	99	101	99	30	5	29	36	3	83	10	20	34
Tuberculosis	11	55	20	11	11	7	79	73	50	72	36	28	55	18	34	10	34	33	24	6	35	37	8	5	11	5	10
Other unintentional injuries	12	21	13	14	10	14	29	26	31	23	11	9	15	10	15	14	13	7	11	14	30	6	5	37	31	25	26
Meningitis	13	32	24	19	12	8	28	30	41	25	31	32	33	17	28	26	20	18	26	23	14	26	12	8	5	7	11
Exposure to mechanical forces	14	12	7	10	14	15	10	10	18	15	5	10	14	14	6	8	7	9	7	25	8	8	9	12	14	17	13
Leukemia	15	6	5	8	19	24	9	4	3	3	6	12	4	8	5	6	9	6	4	8	13	10	21	19	20	28	21
Cerebrovascular disease	16	19	10	9	17	20	19	22	17	19	13	24	17	21	12	7	11	11	6	13	7	7	18	11	21	19	20
Ischemic heart disease	17	17	9	12	16	22	18	27	11	20	15	17	18	11	11	9	14	10	10	18	18	9	11	40	24	18	35
Congenital anomalies	18	8	12	16	22	16	7	8	7	6	10	13	9	19	9	15	6	14	9	12	28	14	28	20	15	14	15
Falls	19	10	11	13	20	31	16	13	9	11	16	7	11	9	13	16	16	12	5	9	23	13	19	42	35	34	28
Chronic kidney disease	20	27	18	15	15	18	31	28	23	28	23	29	24	12	7	12	12	20	19	11	17	19	14	14	19	16	18
Epilepsy	21	18	19	22	18	13	23	14	12	13	22	22	16	7	16	19	15	23	14	19	20	20	26	9	18	9	12
Other neoplasms	22	7	16	21	25	17	6	5	5	4	7	15	7	16	10	13	10	8	12	16	19	18	30	16	22	10	17
Drug use disorders	23	4	15	26	29	39	4	3	26	5	17	5	13	20	21	20	36	19	17	38	55	15	29	17	17	71	40
Hemoglobinopathies and hemolytic anemias	24	33	34	29	27	12	30	46	28	34	38	48	36	24	35	34	17	28	29	26	51	16	36	33	8	15	9
Cirrhosis and other chronic liver diseases due to other causes	25	35	25	25	28	21	38	45	44	50	35	27	25	6	25	21	29	26	33	21	26	24	27	32	16	21	24

© 2017 Global Burden of Disease Child and Adolescent Health Collaboration. JAMA Pediatrics.

eFigure 3. Top Global Causes of Maternal Mortality, by 5 SDI quintiles and 21 GBD regions, Aged 10 to 19 Years, Females, 2015

Cause	Global	High SDI	High-middle SDI	Middle SDI	Low-middle SDI	Low SDI	High-income North America	Australasia	High-income Asia Pacific	Western Europe	Southern Latin America	Eastern Europe	Central Europe	Central Asia	Central Latin America	Andean Latin America	Caribbean	Tropical Latin America	East Asia	Southeast Asia	Oceania	North Africa and Middle East	South Asia	Southern Sub-Saharan Africa	Western Sub-Saharan Africa	Eastern Sub-Saharan Africa	Central Sub-Saharan Africa
Maternal hemorrhage	1	6	5	4	2	1	6	5	5	6	7	5	6	2	3	3	5	6	2	2	1	1	2	3	1	1	1
Maternal hypertensive disorders	2	4	1	1	1	2	5	3	4	4	1	3	3	1	1	1	2	1	4	4	4	3	1	1	2	2	2
Indirect maternal deaths	3	2	2	5	3	3	2	1	3	2	4	2	4	5	2	5	3	2	5	5	5	5	6	2	3	3	3
Maternal abortion, miscarriage, and ectopic pregnancy	4	3	3	2	4	4	3	4	1	3	2	1	1	3	4	4	1	5	3	1	3	2	3	5	4	5	5
Other maternal disorders	5	1	4	3	5	5	1	2	2	1	5	4	2	4	5	6	4	4	1	3	2	4	4	4	5	4	4
Maternal obstructed labor and uterine rupture	6	8	7	6	6	8	8	8	7	8	8	8	7	8	8	8	8	8	6	7	7	7	5	9	7	8	8
Maternal sepsis and other maternal infections	7	7	6	7	7	6	7	7	6	7	3	6	5	6	6	2	6	3	7	6	8	6	7	6	6	6	7
Late maternal deaths	8	5	8	8	8	7	4	6	8	5	6	7	8	7	7	7	7	7	8	8	6	8	8	7	8	7	6
Maternal deaths aggravated by HIV/AIDS	9	9	9	9	9	9	9	9	9	9	9	9	9	9	9	9	9	9	9	9	9	9	9	8	9	9	9

Note: Redder cells indicate lower numbers, and therefore higher rankings. Greener cells indicate higher numbers, and therfore lower rankings

© 2017 Global Burden of Disease Child and Adolescent Health Collaboration. JAMA Pediatrics.

eFigure 4. The expected relationship between YLLs and YLDs and Socio-Demographic Index (SDI) for level 3 maternal causes in 10-19 years from 1990 to 2015.

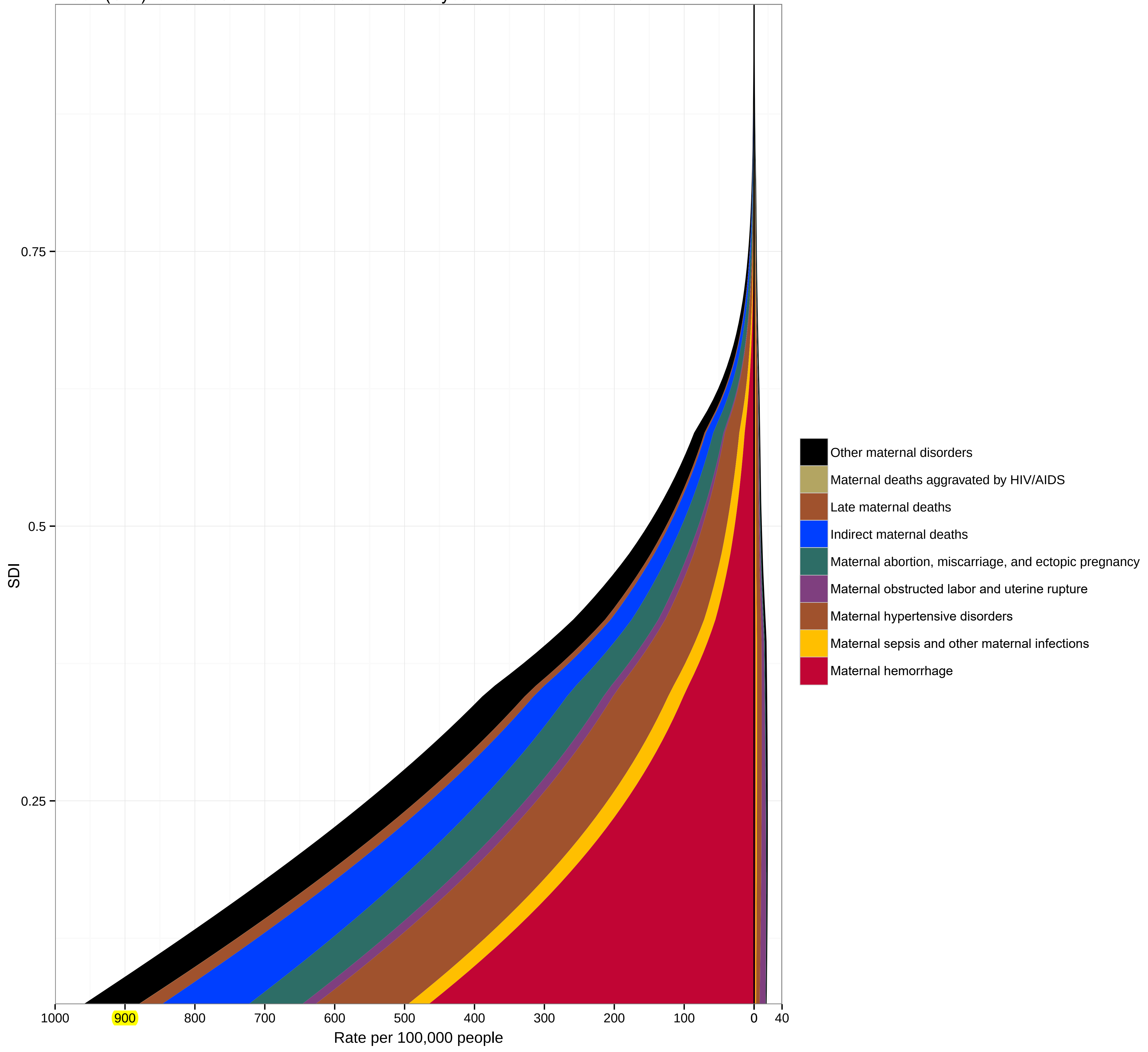

© 2017 Global Burden of Disease Child and Adolescent Health Collaboration. JAMA Pediatrics.

eFigure 5. Global pregnancy complication ratio (events per 100 live births) by type of complication and age group in 2015

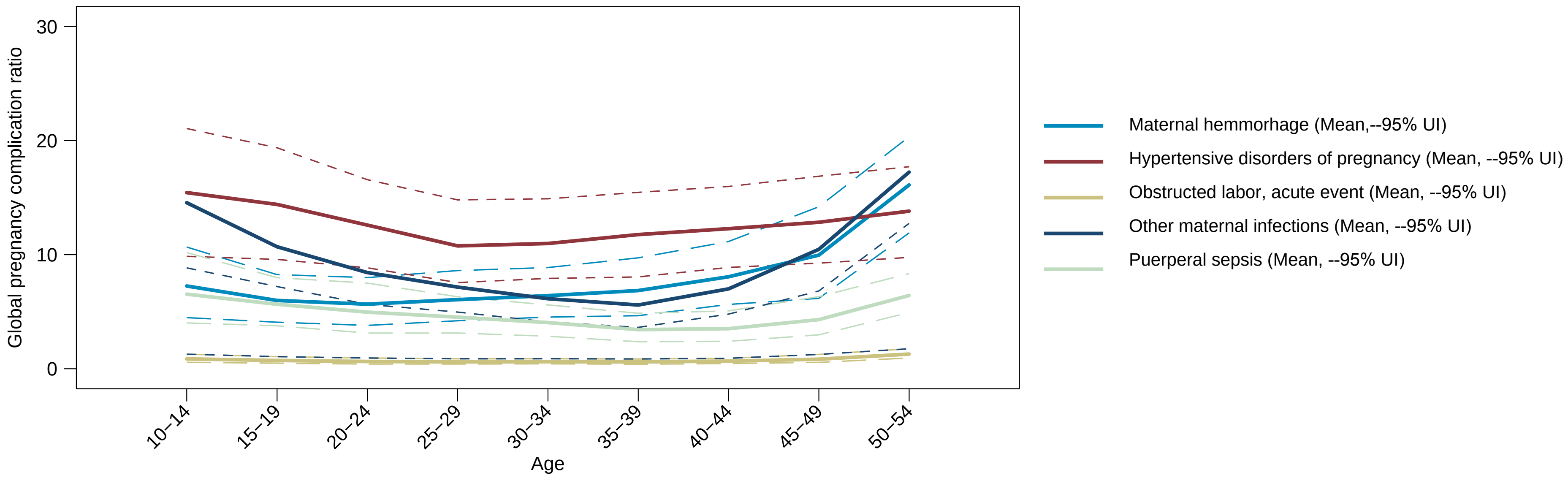

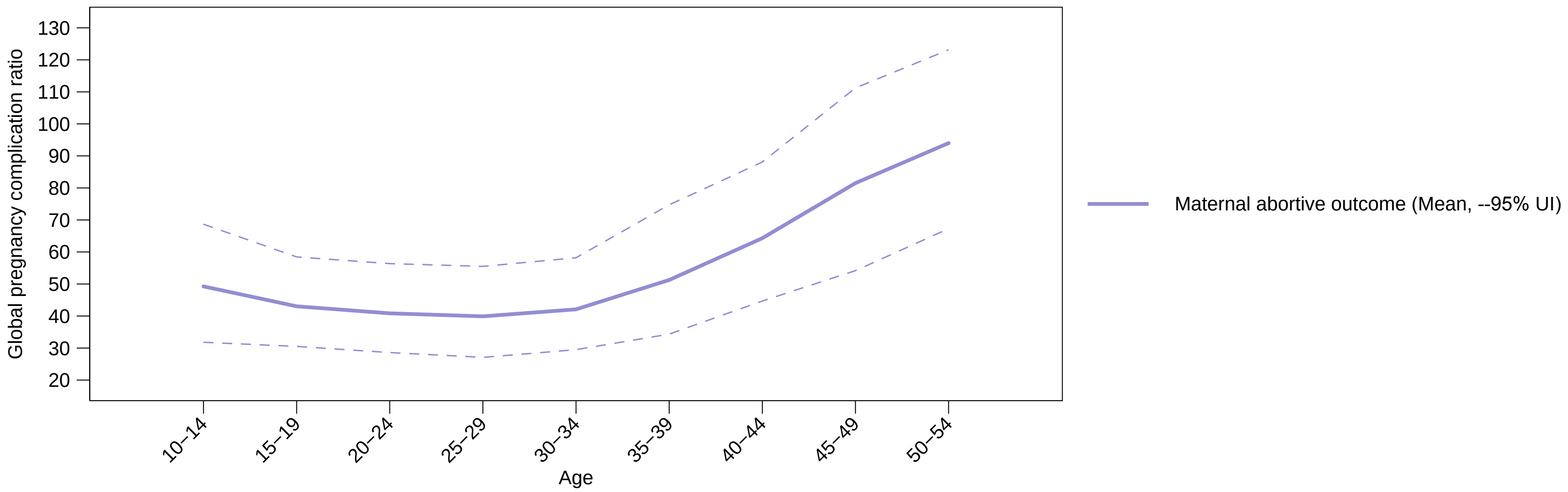

© 2017 Global Burden of Disease Child and Adolescent Health Collaboration. JAMA Pediatrics.

eFigure 6a. Leading 25 GBD cause hierarchy level 3 causes of low SDI DALYs for both sexes combined for 1990, 2005 and 2015, 0-19 years.

Causes are connected by arrows between time periods. Communicable maternal, neonatal and nutritional disorders are shown in red, non-communicable causes in blue and injuries in green. Statistically significant changes have been bolded.

Leading causes 1990	Leading causes 2005	Mean % change number of 0-19 DALYs 1990-2005	Mean % change all-age DALY rate 1990-2005	Mean % change age-standardized DALY rate 1990-2005	Leading causes 2015	Mean % change number of 0-19 DALYs 2005-2015	Mean % change all-age DALY rate 2005-2015	Mean % change age-standardized DALY rate 2005-2015
1 Malaria	1 Malaria	15.8	**-24.0**	**-17.6**	1 Malaria	**-40.6**	**-53.9**	**-48.3**
2 Lower respiratory infect	2 Lower respiratory infect	-4.6	**-37.0**	**-27.8**	2 Lower respiratory infect	**-19.6**	**-36.1**	**-24.3**
3 Diarrheal diseases	3 Diarrheal diseases	**-21.9**	**-48.1**	**-41.4**	3 Diarrheal diseases	**-24.8**	**-40.6**	**-32.8**
4 Measles	4 Neonatal preterm birth	10.1	**-29.5**	**-22.2**	4 Neonatal preterm birth	-6.5	**-30.2**	**-21.2**
5 Protein-energy malnutrition	5 Protein-energy malnutrition	-22.5	**-49.9**	**-45.7**	5 Neonatal encephalopathy	5.9	**-20.7**	-10.4
6 Neonatal preterm birth	6 Neonatal encephalopathy	14.2	**-26.7**	**-18.7**	6 Congenital anomalies	19.5	-10.1	-0.6
7 Neonatal encephalopathy	7 Neonatal sepsis	16.7	**-25.3**	-17.8	7 Neonatal sepsis	9.9	-18.0	-7.7
8 Meningitis	8 Congenital anomalies	**24.6**	**-19.7**	-12.8	8 Protein-energy malnutrition	-13.4	**-34.8**	**-28.6**
9 Neonatal sepsis	9 HIV/AIDS	**414.4**	**213.9**	**227.4**	9 Meningitis	-4.5	**-26.1**	**-18.9**
10 Congenital anomalies	10 Meningitis	-5.2	**-37.4**	**-30.9**	10 HIV/AIDS	**-30.8**	**-52.7**	**-54.8**
11 Other neonatal	11 Other neonatal	-1.8	**-37.1**	-30.5	11 Other neonatal	-5.6	-29.4	-20.4
12 STDs	12 Measles	**-69.3**	**-80.4**	**-79.5**	12 STDs	12.0	**-16.0**	-8.2
13 Iron-deficiency anemia	13 STDs	-0.9	**-36.1**	**-31.4**	13 Iron-deficiency anemia	**9.8**	**-17.4**	**-15.9**
14 Tetanus	14 Iron-deficiency anemia	**34.7**	**-15.5**	**-15.9**	14 Hemoglobinopathies	11.8	-14.1	-8.4
15 Hemoglobinopathies	15 Whooping cough	14.7	-26.7	-22.5	15 Skin diseases	**31.7**	0.6	1.6
16 Whooping cough	16 Hemoglobinopathies	11.2	-26.4	**-20.3**	16 Whooping cough	-22.3	-42.0	-36.9
17 Drowning	17 Road injuries	11.7	**-20.3**	**-15.3**	17 Road injuries	12.3	-8.3	-6.3
18 Neonatal hemolytic	18 Drowning	-4.3	**-36.4**	**-31.8**	18 Measles	-62.7	**-72.2**	**-70.0**
19 War & legal intervention	19 Skin diseases	**53.9**	-1.2	-0.6	19 Drowning	-7.0	**-27.3**	**-22.1**
20 Road injuries	20 Tuberculosis	0.4	**-29.1**	**-24.3**	20 Other NTDs	18.8	-8.5	-6.8
21 Tuberculosis	21 Neonatal hemolytic	-16.0	**-46.1**	**-40.3**	21 Tuberculosis	-14.3	**-25.4**	**-25.0**
22 HIV/AIDS	22 Tetanus	**-49.7**	**-65.4**	**-57.5**	22 Other infectious	3.7	-21.7	-15.7
23 Other infectious	23 Other NTDs	**72.6**	23.0	39.1	23 Asthma	**22.5**	-7.9	**-10.3**
24 Skin diseases	24 Other infectious	-1.5	**-36.1**	**-30.1**	24 War & legal intervention	**512.2**	**233.4**	**176.8**
25 Foreign body	25 Asthma	**21.9**	**-24.5**	**-23.5**	25 Neonatal hemolytic	-23.8	**-42.9**	**-35.3**
27 Asthma	26 Foreign body				26 Foreign body			
29 Other NTDs	66 War & legal intervention				31 Tetanus			

Legend:

Communicable, maternal, neonatal and nutritional

Non-communicable

Injuries

© 2017 Global Burden of Disease Child and Adolescent Health Collaboration. JAMA Pediatrics.

eFigure 6b. Leading 25 GBD cause hierarchy level 3 causes of low-middle SDI DALYs for both sexes combined for 1990, 2005 and 2015, 0-19 years.

Causes are connected by arrows between time periods. Communicable maternal, neonatal and nutritional disorders are shown in red, non-communicable causes in blue and injuries in green. Statistically significant changes have been bolded.

Leading causes 1990	Leading causes 2005	Mean % change number of 0-19 DALYs 1990-2005	Mean % change all-age DALY rate 1990-2005	Mean % change age-standardized DALY rate 1990-2005	Leading causes 2015	Mean % change number of 0-19 DALYs 2005-2015	Mean % change all-age DALY rate 2005-2015	Mean % change age-standardized DALY rate 2005-2015
1 Lower respiratory infect	1 Lower respiratory infect	**-41.2**	**-52.3**	**-40.7**	1 Neonatal encephalopathy	-11.1	**-23.9**	**-14.0**
2 Diarrheal diseases	2 Neonatal preterm birth	**-29.0**	**-47.2**	**-35.7**	2 Neonatal preterm birth	**-23.4**	**-34.6**	**-26.0**
3 Neonatal preterm birth	3 Neonatal encephalopathy	6.9	**-20.4**	-3.4	3 Lower respiratory infect	**-36.7**	**-39.6**	**-31.3**
4 Neonatal encephalopathy	4 Diarrheal diseases	**-38.2**	**-50.0**	**-41.5**	4 Diarrheal diseases	**-31.2**	**-38.0**	**-33.9**
5 Measles	5 Malaria	**20.7**	-8.5	3.1	5 Malaria	**-42.0**	**-48.1**	**-42.9**
6 Malaria	6 Congenital anomalies	0.4	**-23.1**	-9.1	6 Congenital anomalies	5.6	-8.4	0.9
7 Protein-energy malnutrition	7 Measles	**-55.2**	**-66.7**	**-61.4**	7 Neonatal sepsis	0.6	-14.5	-3.5
8 Congenital anomalies	8 Neonatal sepsis	15.4	-14.5	3.8	8 Iron-deficiency anemia	**-5.3**	**-18.9**	**-18.0**
9 Tetanus	9 Iron-deficiency anemia	**17.3**	**-10.1**	**-7.5**	9 Other neonatal	-13.2	-25.9	-16.5
10 Meningitis	10 Protein-energy malnutrition	**-37.4**	**-52.0**	**-43.1**	10 Meningitis	-14.9	**-23.9**	**-18.1**
11 Neonatal sepsis	11 HIV/AIDS	**596.7**	**486.9**	**488.8**	11 Protein-energy malnutrition	-26.5	**-36.6**	**-31.3**
12 Other neonatal	12 Meningitis	**-24.6**	**-39.4**	**-29.3**	12 HIV/AIDS	**-28.5**	**-43.3**	**-45.6**
13 Iron-deficiency anemia	13 Other neonatal	-16.4	**-37.8**	-24.7	13 Hemoglobinopathies	-1.1	-14.1	-9.9
14 Drowning	14 Intestinal infectious	**-15.3**	**-36.3**	**-31.7**	14 Intestinal infectious	**-17.8**	**-28.3**	**-23.9**
15 Intestinal infectious	15 Hemoglobinopathies	14.3	-13.1	-6.4	15 Skin diseases	**10.7**	0.3	**1.4**
16 STDs	16 Drowning	**-35.2**	**-45.9**	**-38.5**	16 Road injuries	-7.4	-6.7	-6.9
17 Whooping cough	17 Skin diseases	**26.6**	0.3	**1.2**	17 Drowning	**-34.2**	**-33.7**	**-28.0**
18 Neonatal hemolytic	18 STDs	**-31.1**	**-48.0**	**-40.3**	18 STDs	**-36.3**	**-42.8**	**-36.2**
19 Hemoglobinopathies	19 Road injuries	-4.5	-6.0	-1.1	19 Measles	**-81.5**	**-84.3**	**-82.7**
20 Road injuries	20 Whooping cough	-38.3	-54.2	-46.4	20 Asthma	**-7.8**	**-21.8**	**-28.2**
21 Tuberculosis	21 Neonatal hemolytic	**-45.5**	**-59.0**	**-50.0**	21 Encephalitis	-8.1	**-17.4**	**-14.0**
22 Asthma	22 Tetanus	**-77.4**	**-81.4**	**-76.3**	22 Sense organ diseases	**9.4**	**6.2**	**-1.7**
23 Skin diseases	23 Asthma	**-26.7**	**-41.5**	**-45.4**	23 Neonatal hemolytic	**-34.9**	**-43.5**	**-36.0**
24 Encephalitis	24 Tuberculosis	**-39.5**	**-37.0**	**-39.3**	24 Whooping cough	-46.3	-54.4	-49.6
25 Foreign body	25 Encephalitis	**-16.2**	**-33.0**	**-28.7**	25 Falls	1.0	-2.0	-3.7
32 Falls	29 Foreign body				27 Foreign body			
38 Sense organ diseases	30 Sense organ diseases				28 Tuberculosis			
40 HIV/AIDS	31 Falls				40 Tetanus			

Legend:

Communicable, maternal, neonatal and nutritional

Non-communicable

Injuries

© 2017 Global Burden of Disease Child and Adolescent Health Collaboration. JAMA Pediatrics.

eFigure 6c. Leading 25 GBD cause hierarchy level 3 causes of middle SDI DALYs for both sexes combined for 1990, 2005 and 2015, 0-19 years.

Causes are connected by arrows between time periods. Communicable maternal, neonatal and nutritional disorders are shown in red, non-communicable causes in blue and injuries in green. Statistically significant changes have been bolded.

Leading causes 1990
1 Lower respiratory infect
2 Neonatal preterm birth
3 Diarrheal diseases
4 Neonatal encephalopathy
5 Congenital anomalies
6 Measles
7 Drowning
8 Iron-deficiency anemia
9 Road injuries
10 Other neonatal
11 Meningitis
12 Protein-energy malnutrition
13 Neonatal sepsis
14 Skin diseases
15 Intestinal infectious
16 Tetanus
17 Whooping cough
18 Asthma
19 STDs
20 Hemoglobinopathies
21 Mechanical forces
22 Tuberculosis
23 Cerebrovascular disease
24 Encephalitis
25 COPD
27 Leukemia
33 Sense organ diseases
37 Anxiety disorders
41 Conduct disorder
43 Depressive disorders
44 Low back & neck pain
45 Migraine
80 War & legal intervention
100 HIV/AIDS

Leading causes 2005	Mean % change number of 0-19 DALYs 1990-2005	Mean % change all-age DALY rate 1990-2005	Mean % change age-standardized DALY rate 1990-2005
1 Neonatal preterm birth	**-53.8**	**-60.5**	**-43.0**
2 Neonatal encephalopathy	**-18.2**	**-31.0**	-0.3
3 Lower respiratory infect	**-67.6**	**-66.1**	**-52.3**
4 Congenital anomalies	**-29.6**	**-37.5**	-14.0
5 Diarrheal diseases	**-65.7**	**-64.7**	**-54.0**
6 Iron-deficiency anemia	**-3.0**	**-12.4**	**-4.8**
7 Road injuries	**-28.0**	-3.6	-2.2
8 Skin diseases	**1.8**	**-1.5**	**1.1**
9 Drowning	**-61.1**	**-60.2**	**-52.5**
10 Neonatal sepsis	-15.9	**-29.9**	2.4
11 Other neonatal	**-44.4**	**-52.9**	**-31.9**
12 Intestinal infectious	**-27.6**	**-37.6**	**-26.2**
13 Meningitis	**-56.3**	**-55.6**	**-44.1**
14 Measles	**-80.1**	**-83.4**	**-76.7**
15 Asthma	**-29.0**	**-25.1**	**-28.9**
16 Hemoglobinopathies	**-24.7**	**-23.6**	**-16.5**
17 Protein-energy malnutrition	**-62.7**	**-63.7**	**-51.1**
18 HIV/AIDS	**1,594.5**	**1,521.6**	**1,439.3**
19 Encephalitis	**-22.8**	**-26.8**	**-14.2**
20 Sense organ diseases	1.8	**17.6**	0.3
21 Anxiety disorders	**8.5**	**4.8**	-0.6
22 Conduct disorder	**11.7**	**-6.9**	**2.6**
23 Mechanical forces	**-45.7**	**-33.0**	**-26.2**
24 Depressive disorders	**13.0**	**13.6**	**1.7**
25 Leukemia	**-28.3**	**-19.7**	**-14.4**
27 Low back & neck pain			
28 Cerebrovascular disease			
29 Migraine			
31 Tuberculosis			
35 Whooping cough			
37 STDs			
39 Tetanus			
43 COPD			
73 War & legal intervention			

Leading causes 2015	Mean % change number of 0-19 DALYs 2005-2015	Mean % change all-age DALY rate 2005-2015	Mean % change age-standardized DALY rate 2005-2015
1 Neonatal preterm birth	**-30.7**	**-35.2**	**-33.2**
2 Congenital anomalies	-11.0	**-14.6**	**-12.0**
3 Neonatal encephalopathy	**-31.0**	**-35.5**	**-33.6**
4 Lower respiratory infect	**-46.3**	**-36.2**	**-36.6**
5 Iron-deficiency anemia	**-12.8**	**-17.6**	**-13.6**
6 Skin diseases	**-6.6**	**-1.7**	0.5
7 Diarrheal diseases	**-47.9**	**-43.3**	**-44.1**
8 Road injuries	**-28.5**	**-14.8**	**-15.6**
9 Neonatal sepsis	-14.8	**-22.0**	**-19.6**
10 Other neonatal	**-22.3**	**-27.6**	**-25.4**
11 Drowning	**-42.6**	**-38.9**	**-35.5**
12 Intestinal infectious	**-26.1**	**-29.0**	**-23.4**
13 Asthma	**-11.0**	**-10.9**	**-16.5**
14 War & legal intervention	**582.8**	**405.1**	**415.2**
15 Meningitis	**-34.4**	**-29.5**	**-29.0**
16 Hemoglobinopathies	**-13.9**	**-12.3**	**-11.3**
17 Sense organ diseases	**-3.8**	**14.5**	-0.3
18 HIV/AIDS	**-18.6**	**-25.2**	**-26.6**
19 Encephalitis	**-15.9**	**-15.8**	**-15.0**
20 Anxiety disorders	**-9.2**	**3.7**	**3.8**
21 Conduct disorder	**-7.3**	**-14.8**	**3.9**
22 Depressive disorders	**-8.7**	**6.2**	0.7
23 Protein-energy malnutrition	**-38.5**	**-36.5**	**-36.6**
24 Migraine	**-6.6**	**4.2**	**2.9**
25 Low back & neck pain	**-12.5**	**8.7**	**-1.2**
26 Leukemia			
27 Mechanical forces			
29 Measles			
33 Cerebrovascular disease			
39 Tuberculosis			
46 COPD			
58 Whooping cough			
59 STDs			
65 Tetanus			

Legend:

Communicable, maternal, neonatal and nutritional

Non-communicable

Injuries

© 2017 Global Burden of Disease Child and Adolescent Health Collaboration. JAMA Pediatrics.

eFigure 6d. Leading 25 GBD cause hierarchy level 3 causes of high-middle SDI DALYs for both sexes combined for 1990, 2005 and 2015, 0-19 years.

Causes are connected by arrows between time periods. Communicable maternal, neonatal and nutritional disorders are shown in red, non-communicable causes in blue and injuries in green. Statistically significant changes have been bolded.

Leading causes 1990	Leading causes 2005	Mean % change number of 0-19 DALYs 1990-2005	Mean % change all-age DALY rate 1990-2005	Mean % change age-standardized DALY rate 1990-2005	Leading causes 2015	Mean % change number of 0-19 DALYs 2005-2015	Mean % change all-age DALY rate 2005-2015	Mean % change age-standardized DALY rate 2005-2015
1 Neonatal preterm birth	1 Neonatal preterm birth	**-53.4**	**-60.1**	**-43.7**	1 Congenital anomalies	**-19.7**	**-21.4**	**-15.5**
2 Lower respiratory infect	2 Congenital anomalies	**-35.0**	**-41.7**	**-21.1**	2 Neonatal preterm birth	**-40.7**	**-44.1**	**-38.5**
3 Congenital anomalies	3 Lower respiratory infect	**-65.3**	**-56.5**	**-44.6**	3 Iron-deficiency anemia	**-9.5**	**-12.7**	**-6.5**
4 Diarrheal diseases	4 Neonatal encephalopathy	**-20.9**	**-33.6**	-5.7	4 Neonatal encephalopathy	**-33.6**	**-37.9**	**-31.4**
5 Neonatal encephalopathy	5 Iron-deficiency anemia	**-4.8**	**-11.3**	-1.0	5 Lower respiratory infect	**-49.7**	**-30.3**	**-33.9**
6 Road injuries	6 Road injuries	**-29.4**	**-5.9**	**-7.1**	6 Skin diseases	**-9.4**	**-2.3**	-0.2
7 Iron-deficiency anemia	7 Skin diseases	0.8	-1.2	**1.1**	7 Road injuries	**-38.1**	**-22.3**	**-23.2**
8 Drowning	8 Diarrheal diseases	**-71.0**	**-70.6**	**-61.5**	8 Other neonatal	**-31.2**	**-35.6**	**-29.1**
9 Other neonatal	9 Other neonatal	**-42.4**	**-51.2**	**-30.9**	9 HIV/AIDS	**-35.1**	**-44.5**	**-44.8**
10 Skin diseases	10 HIV/AIDS	**2,383.9**	**1,447.5**	**1,384.0**	10 Neonatal sepsis	**-15.1**	**-23.9**	**-14.3**
11 Protein-energy malnutrition	11 Drowning	**-60.7**	**-54.6**	**-48.8**	11 Asthma	**-15.4**	**-12.8**	**-13.9**
12 Meningitis	12 Neonatal sepsis	**-13.6**	**-29.1**	2.8	12 Diarrheal diseases	**-52.4**	**-45.5**	**-45.4**
13 Measles	13 Asthma	**-20.0**	**-18.8**	**-19.1**	13 Sense organ diseases	**-7.6**	**13.9**	-0.1
14 Asthma	14 Interpersonal violence	**-21.4**	**-15.8**	**-17.4**	14 Anxiety disorders	**-12.7**	2.3	**1.9**
15 Neonatal sepsis	15 Anxiety disorders	**8.2**	**4.2**	-1.3	15 Depressive disorders	**-14.2**	**4.8**	-0.5
16 Interpersonal violence	16 Depressive disorders	**12.3**	**11.4**	0.3	16 Conduct disorder	**-10.8**	**-19.5**	**0.9**
17 STDs	17 Sense organ diseases	-0.5	**17.0**	**1.2**	17 Drowning	**-47.7**	**-41.3**	**-39.0**
18 Nature disaster	18 Conduct disorder	**7.6**	**-11.4**	**0.9**	18 Interpersonal violence	**-25.2**	**-17.7**	**-16.0**
19 Mechanical forces	19 Low back & neck pain	**10.3**	**14.8**	**-1.9**	19 Migraine	**-10.6**	**2.5**	1.0
20 Leukemia	20 Migraine	**10.3**	**8.0**	-0.5	20 Low back & neck pain	**-17.0**	**7.5**	**-2.2**
21 Foreign body	21 Meningitis	**-60.0**	**-55.6**	**-46.1**	21 Hemoglobinopathies	**-18.1**	**-14.7**	**-12.3**
22 Fire & heat	22 Hemoglobinopathies	**-24.6**	**-22.1**	**-15.3**	22 Leukemia	**-22.3**	**-10.6**	**-12.2**
23 Other cardiovascular	23 Leukemia	**-29.5**	**-19.0**	**-16.6**	23 Intestinal infectious	**-26.9**	**-29.4**	**-22.7**
24 Hemoglobinopathies	24 Intestinal infectious	**-20.4**	**-32.4**	**-17.2**	24 Epilepsy	**-29.9**	**-24.2**	**-23.3**
25 Cerebrovascular disease	25 Epilepsy	**-26.2**	**-20.1**	**-17.5**	25 Autistic spectrum	**-8.4**	**-1.4**	0.3
26 Epilepsy	27 Protein-energy malnutrition				26 Foreign body			
28 Intestinal infectious	28 Mechanical forces				27 Meningitis			
29 Sense organ diseases	29 Foreign body				29 Other cardiovascular			
30 Anxiety disorders	30 Other cardiovascular				30 Protein-energy malnutrition			
31 Depressive disorders	31 Autistic spectrum				31 Mechanical forces			
32 Conduct disorder	33 Fire & heat				36 Fire & heat			
37 Low back & neck pain	34 Cerebrovascular disease				39 Cerebrovascular disease			
38 Migraine	39 Measles				58 STDs			
45 Autistic spectrum	46 STDs				65 Measles			
90 HIV/AIDS	87 Nature disaster				99 Nature disaster			

Legend:
Communicable, maternal, neonatal and nutritional
Non-communicable
Injuries

© 2017 Global Burden of Disease Child and Adolescent Health Collaboration. JAMA Pediatrics.

eFigure 6e. Leading 25 GBD cause hierarchy level 3 causes of high SDI DALYs for both sexes combined for 1990, 2005 and 2015, 0-19 years.

Causes are connected by arrows between time periods. Communicable maternal, neonatal and nutritional disorders are shown in red, non-communicable causes in blue and injuries in green. Statistically significant changes have been bolded.

Leading causes 1990	Leading causes 2005	Mean % change number of 0-19 DALYs 1990-2005	Mean % change all-age DALY rate 1990-2005	Mean % change age-standardized DALY rate 1990-2005
1 Congenital anomalies	1 Congenital anomalies	**-39.6**	**-30.0**	**-25.2**
2 Neonatal preterm birth	2 Iron-deficiency anemia	**-5.8**	**-3.8**	**3.9**
3 Road injuries	3 Neonatal preterm birth	**-49.1**	**-48.0**	**-41.2**
4 Iron-deficiency anemia	4 Skin diseases	**-6.0**	**1.1**	**1.3**
5 Skin diseases	5 Road injuries	**-38.6**	**-22.5**	**-23.3**
6 Neonatal encephalopathy	6 Asthma	**-23.5**	**-26.9**	**-26.2**
7 Lower respiratory infect	7 Depressive disorders	1.5	**3.1**	**-0.8**
8 Asthma	8 Anxiety disorders	-2.7	2.2	0.3
9 Drowning	9 Low back & neck pain	**-2.6**	**7.4**	**-1.5**
10 SIDS	10 Neonatal encephalopathy	**-53.0**	**-51.8**	**-45.3**
11 Other neonatal	11 Conduct disorder	**-4.4**	**-12.1**	-0.4
12 Depressive disorders	12 Migraine	**-5.0**	0.2	-1.7
13 Anxiety disorders	13 Other neonatal	**-16.0**	**-14.8**	-3.2
14 Conduct disorder	14 Self-harm	5.4	**12.7**	**10.2**
15 Low back & neck pain	15 Lower respiratory infect	**-59.8**	1.7	**-15.0**
16 Migraine	16 Interpersonal violence	**-13.3**	**5.0**	**5.9**
17 Interpersonal violence	17 Sense organ diseases	**-10.4**	**14.8**	0.0
18 Falls	18 Drowning	**-52.0**	**-26.5**	**-27.6**
19 Self-harm	19 Autistic spectrum	**-6.7**	**-1.2**	0.4
20 Sense organ diseases	20 SIDS	**-55.4**	**-59.1**	**-50.1**
21 Leukemia	21 Falls	**-35.6**	-0.3	**-11.3**
22 Hemoglobinopathies	22 Hemoglobinopathies	**-24.4**	**-21.0**	**-18.6**
23 Autistic spectrum	23 Other musculoskeletal	**28.4**	**18.4**	**10.6**
24 Fire & heat	24 Neonatal sepsis	**-24.6**	**-30.8**	**-16.3**
25 Neonatal sepsis	25 Endo/metab/blood/immune	**-12.3**	**22.3**	**15.3**
30 Endo/metab/blood/immune	26 Leukemia			
32 Other musculoskeletal	28 Drug use disorders			
42 Drug use disorders	31 Fire & heat			

Leading causes 2015	Mean % change number of 0-19 DALYs 2005-2015	Mean % change all-age DALY rate 2005-2015	Mean % change age-standardized DALY rate 2005-2015
1 Congenital anomalies	**-11.6**	**-8.5**	**-9.0**
2 Iron-deficiency anemia	**-4.8**	**-4.3**	-0.4
3 Skin diseases	**-6.8**	-0.3	0.1
4 Neonatal preterm birth	**-31.8**	**-30.2**	**-30.3**
5 Road injuries	**-47.7**	**-32.4**	**-33.4**
6 Depressive disorders	-0.9	**3.2**	**2.6**
7 Asthma	**-16.3**	**-13.5**	**-13.1**
8 Anxiety disorders	**-6.5**	**-3.0**	-1.5
9 Conduct disorder	**-9.1**	**-15.4**	0.5
10 Low back & neck pain	**-13.6**	**2.7**	**-1.8**
11 Migraine	**-10.8**	**-1.8**	-0.5
12 Other neonatal	**-16.8**	**-15.9**	**-15.7**
13 Neonatal encephalopathy	**-29.3**	**-27.9**	**-28.1**
14 Sense organ diseases	**-7.6**	**11.7**	0.5
15 Self-harm	**-35.2**	**-17.0**	**-17.4**
16 Autistic spectrum	**-5.4**	-0.5	**1.1**
17 Lower respiratory infect	**-37.1**	**-8.0**	**-20.7**
18 Hemoglobinopathies	**-7.3**	**-7.5**	**-5.6**
19 Interpersonal violence	**-39.3**	**-36.7**	**-35.2**
20 Other musculoskeletal	**-12.3**	**4.6**	0.3
21 SIDS	**-24.9**	**-30.5**	**-28.3**
22 Falls	**-28.3**	**-10.2**	**-18.2**
23 Endo/metab/blood/immune	**-10.3**	4.1	-2.5
24 Drug use disorders	**-8.5**	**9.8**	**11.3**
25 Neonatal sepsis	**-16.6**	**-22.9**	**-19.9**
26 Drowning			
27 Leukemia			
38 Fire & heat			

Legend:

Communicable, maternal, neonatal and nutritional

Non-communicable

Injuries

© 2017 Global Burden of Disease Child and Adolescent Health Collaboration. JAMA Pediatrics.

eFigure 7a. The expected relationship between YLL and YLD rates with SDI for GBD level 3 congenital causes, aged 0 to 19 years, both sexes, 1990 to 2015

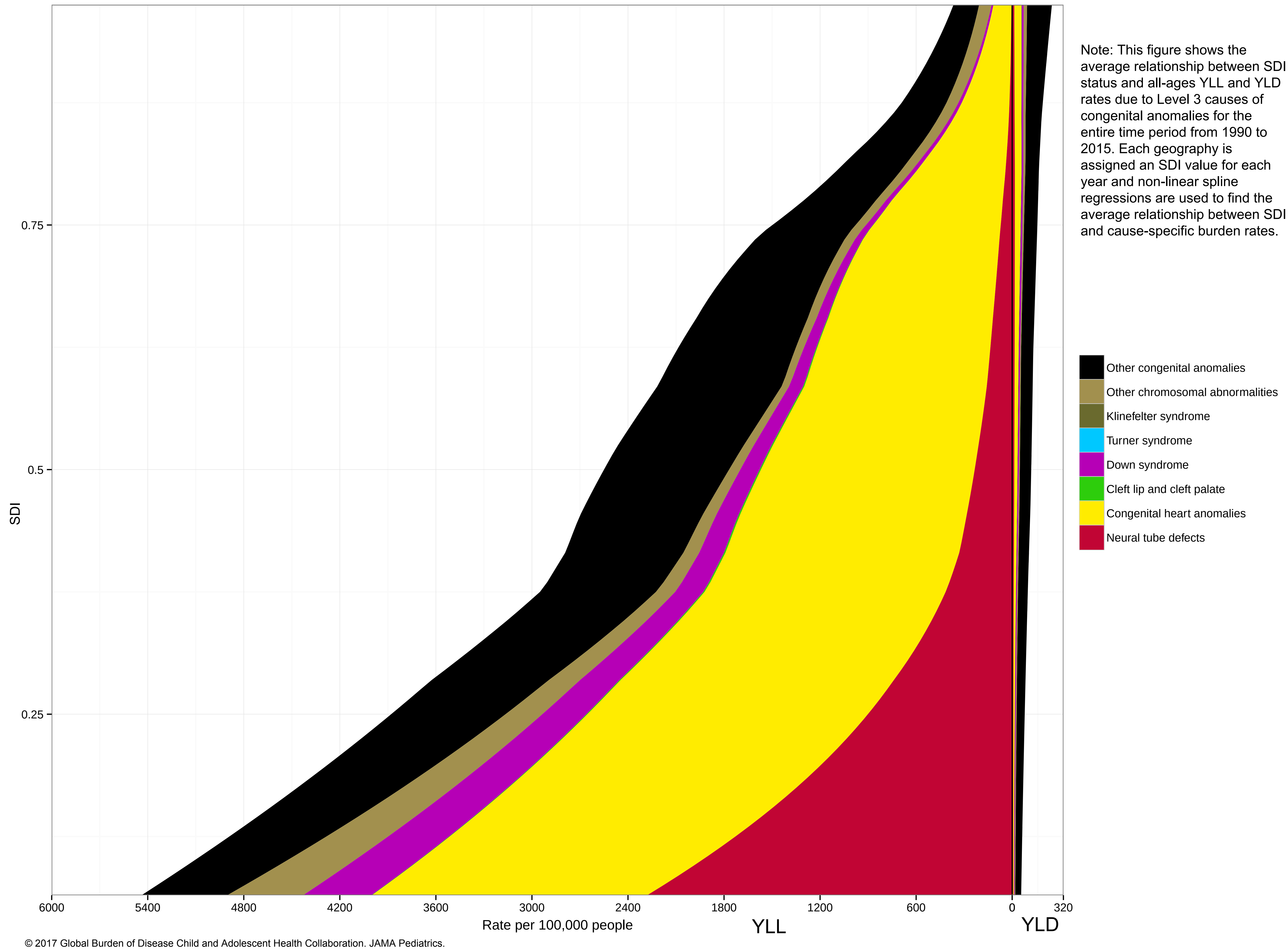

Note: This figure shows the average relationship between SDI status and all-ages YLL and YLD rates due to Level 3 causes of congenital anomalies for the entire time period from 1990 to 2015. Each geography is assigned an SDI value for each year and non-linear spline regressions are used to find the average relationship between SDI and cause-specific burden rates.

© 2017 Global Burden of Disease Child and Adolescent Health Collaboration. JAMA Pediatrics.

eFigure 7b. The expected relationship between YLL and YLD rates with SDI for GBD level 3 neonatal causes, aged 0 to 19 years, both sexes, 1990 to 2015

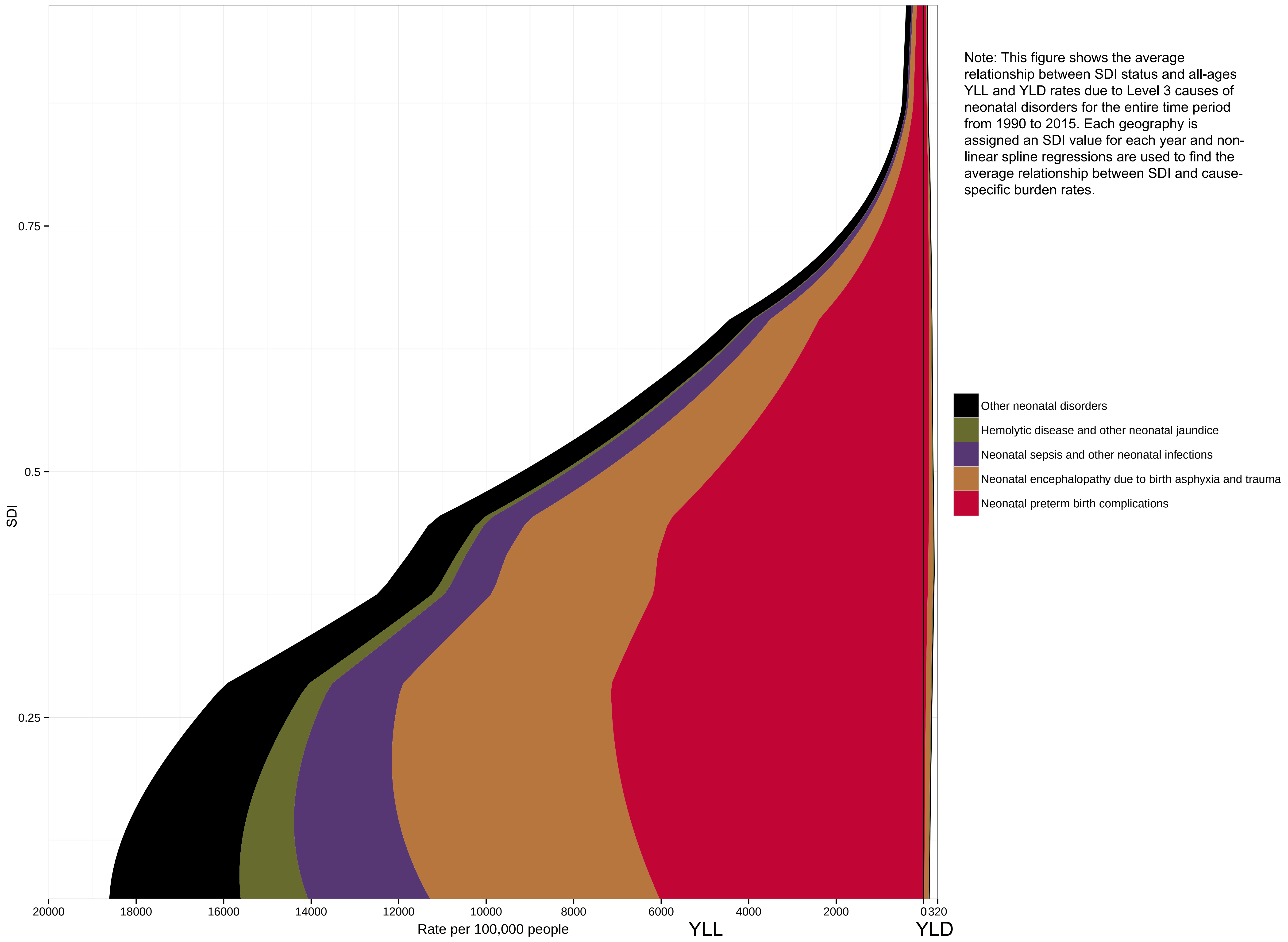

Note: This figure shows the average relationship between SDI status and all-ages YLL and YLD rates due to Level 3 causes of neonatal disorders for the entire time period from 1990 to 2015. Each geography is assigned an SDI value for each year and non-linear spline regressions are used to find the average relationship between SDI and cause-specific burden rates.

© 2017 Global Burden of Disease Child and Adolescent Health Collaboration. JAMA Pediatrics.

eFigure 8a. The expected relationship between cause-specific all-ages DALY rates for 0-19 years, and Sociodemographic Index (*SDI*) for males (*left*) and females (*right*) *All level 2 GBD cause groups*

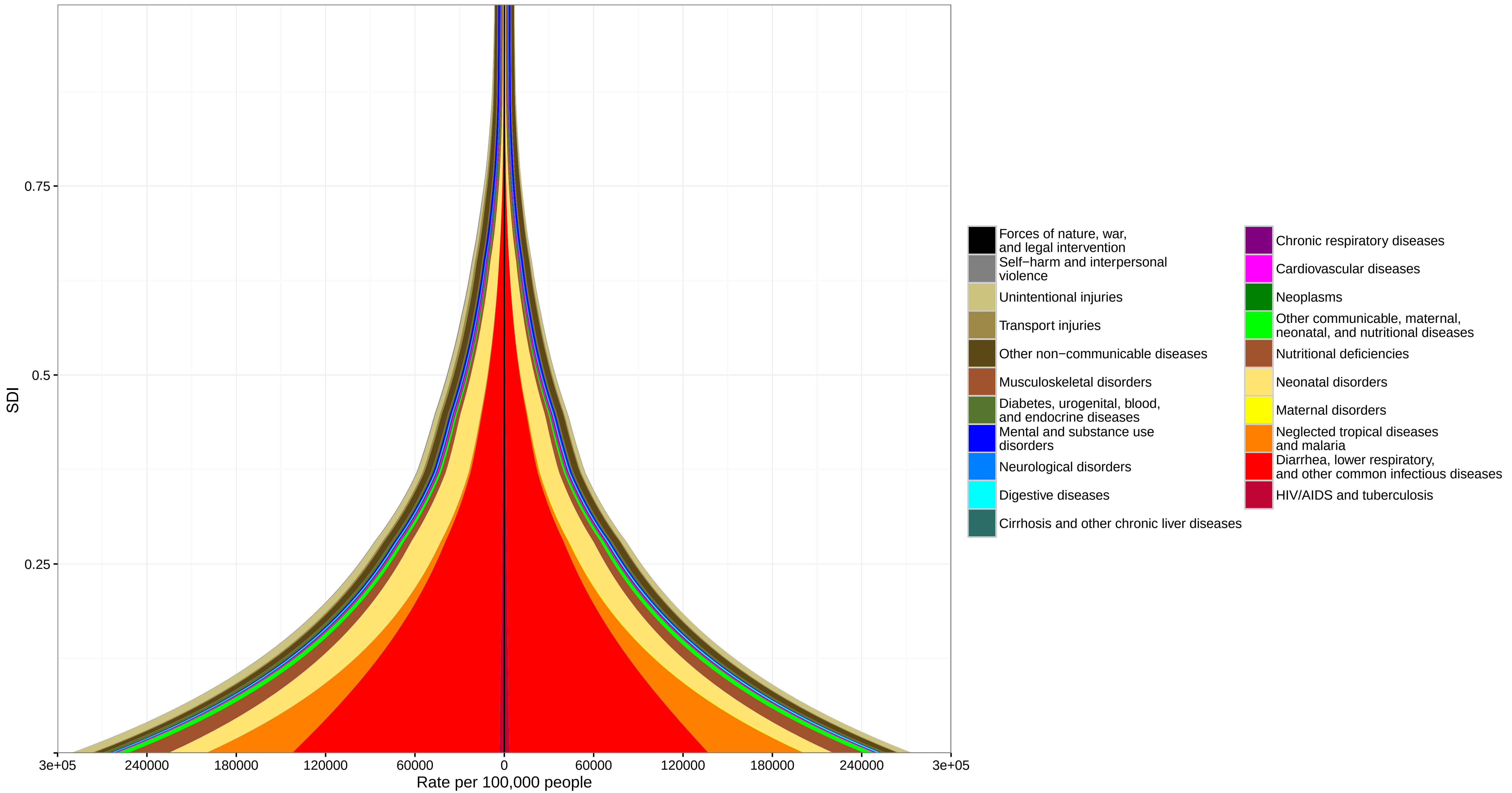

© 2017 Global Burden of Disease Child and Adolescent Health Collaboration. JAMA Pediatrics.

eFigure 8b. The expected relationship between cause-specific all-ages DALY rates for 0-19 years, and Sociodemographic Index (SDI) for males (*left*) and females (*left*) *HIV/AIDS and Tuberculosis*

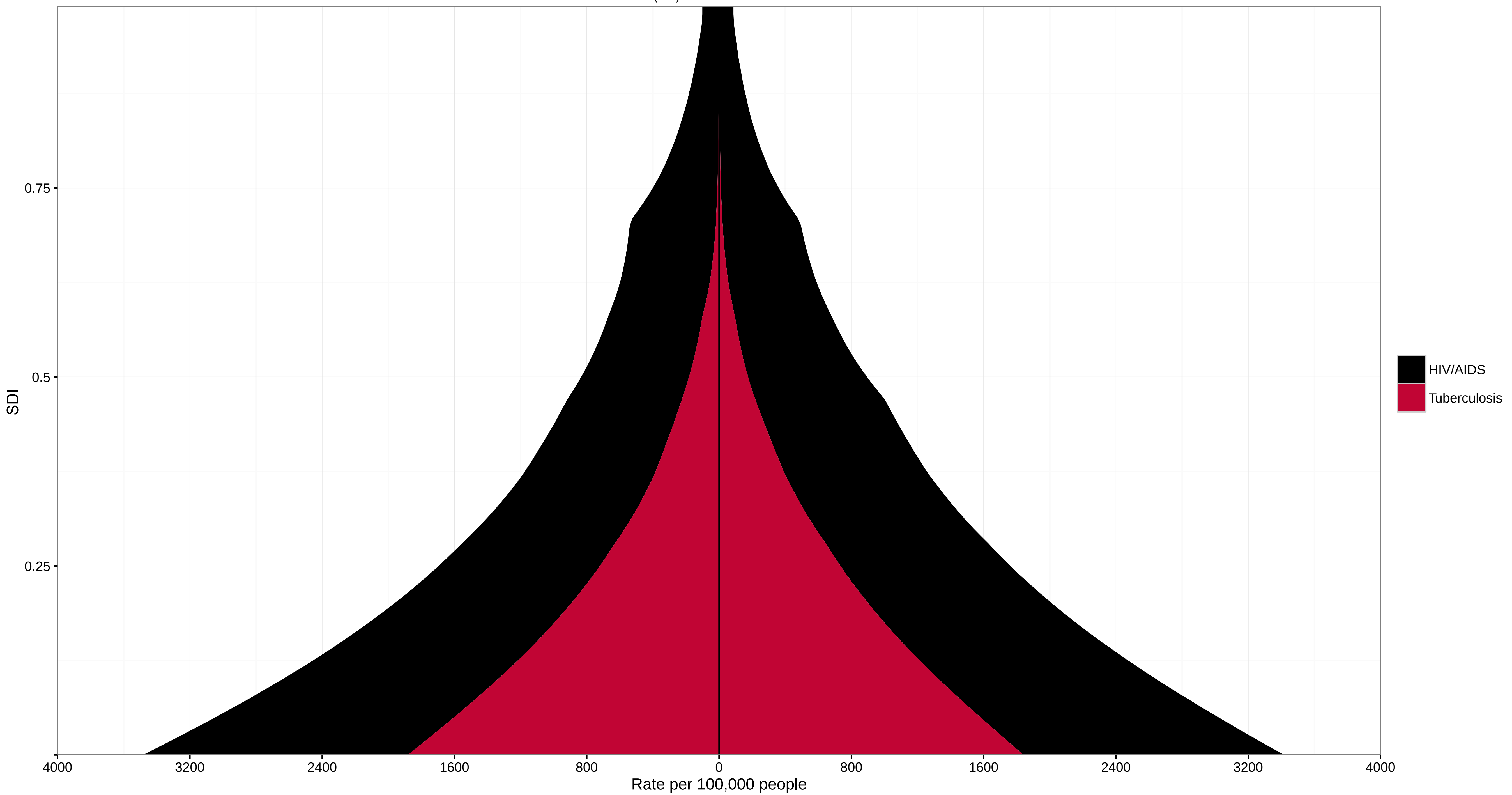

© 2017 Global Burden of Disease Child and Adolescent Health Collaboration. JAMA Pediatrics.

eFigure 8c. The expected relationship between cause-specific all-ages DALY rates for 0-19 years, and Sociodemographic Index (SDI) for males (left) and females (left) *Diarrhea, lower respiratory, and other common infectious diseases*

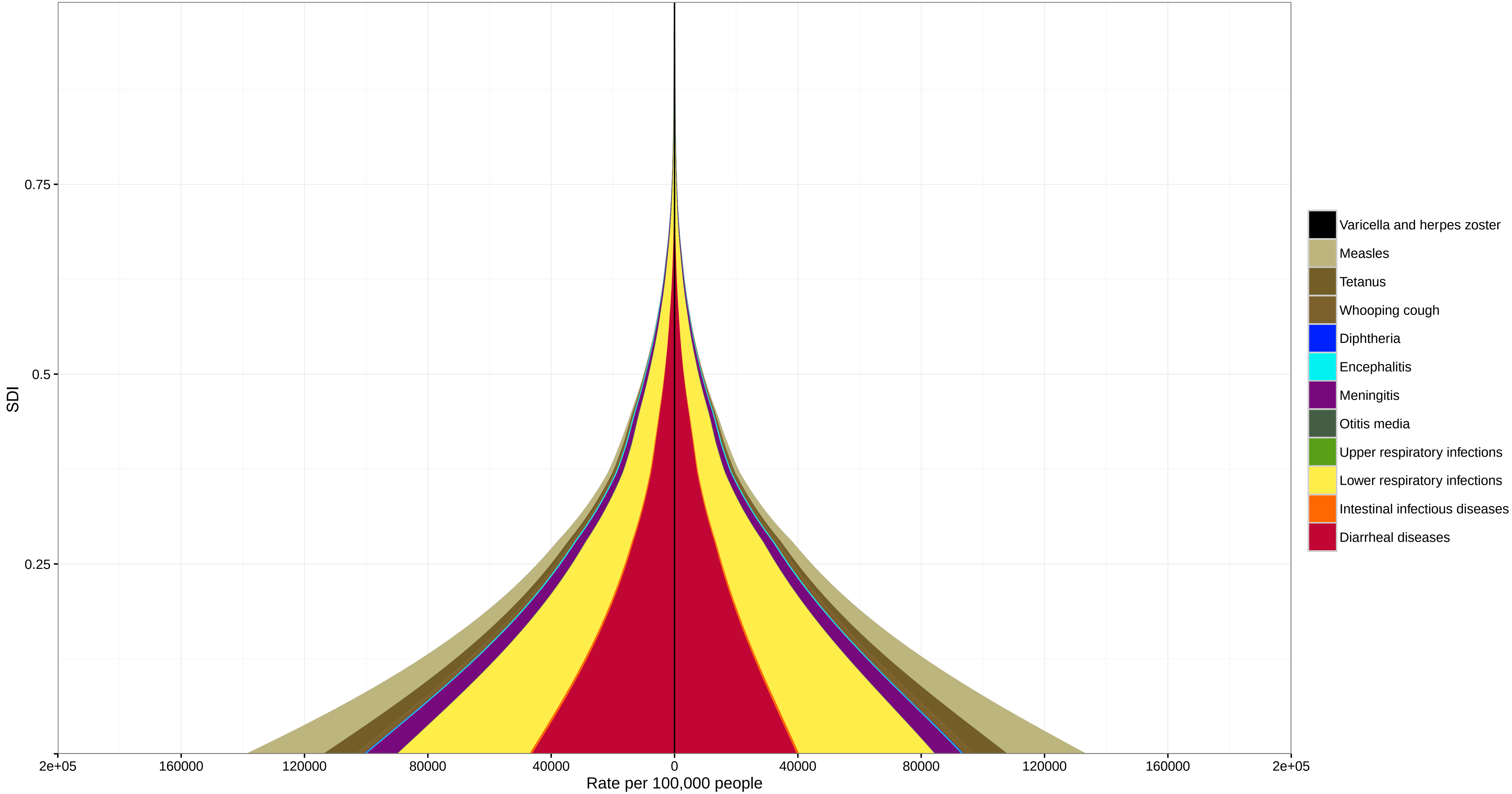

© 2017 Global Burden of Disease Child and Adolescent Health Collaboration. JAMA Pediatrics.

eFigure 8d. The expected relationship between cause-specific all-ages DALY rates for 0-19 years, and Sociodemographic Index (SDI) for males (left) and females (right) *Negelcted tropical diseases and malaria*

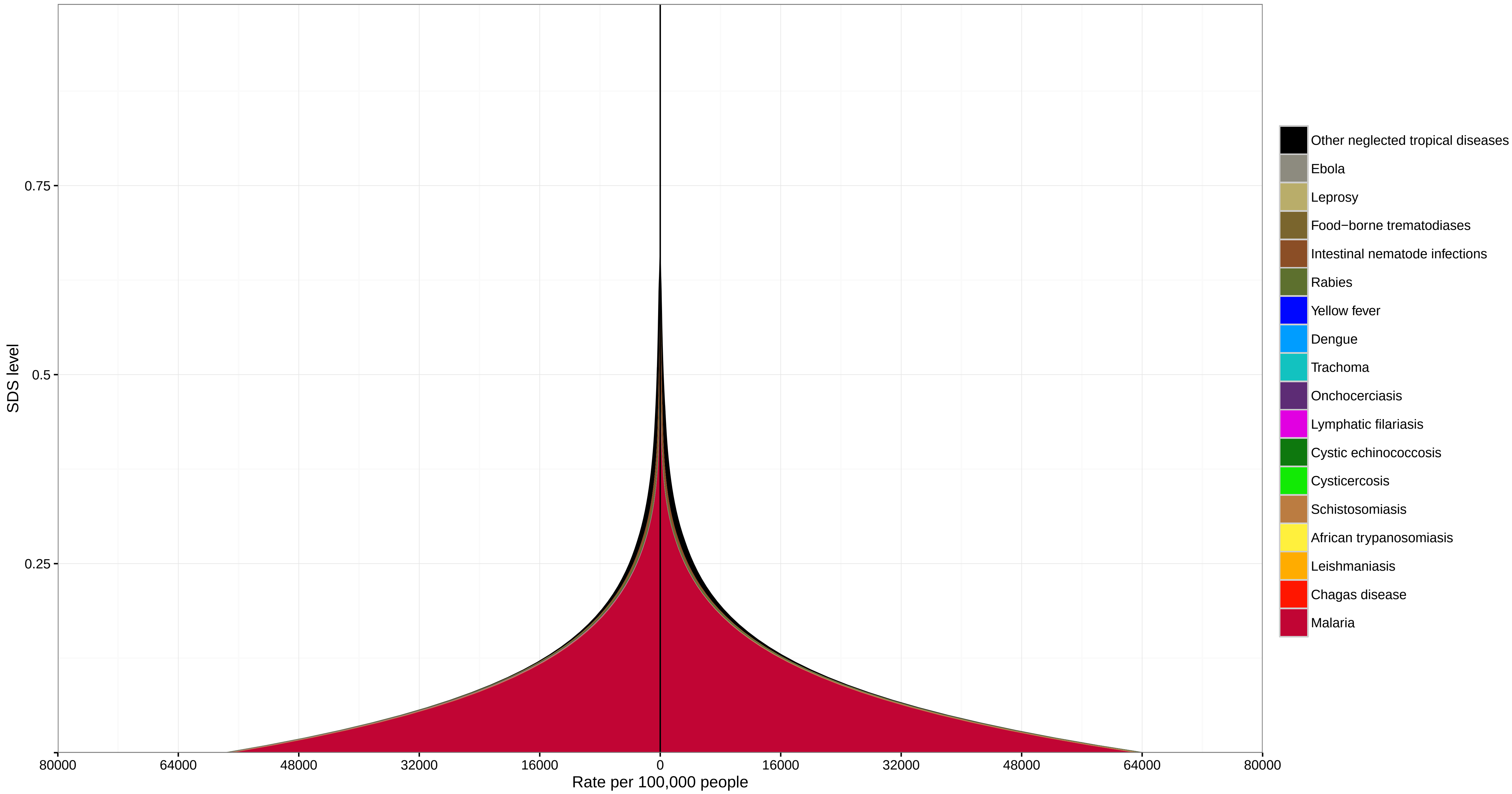

© 2017 Global Burden of Disease Child and Adolescent Health Collaboration. JAMA Pediatrics.

eFigure 8e. The expected relationship between cause-specific all-ages DALY rates for 0-19 years, and Sociodemographic Index (SDI) for females (right)

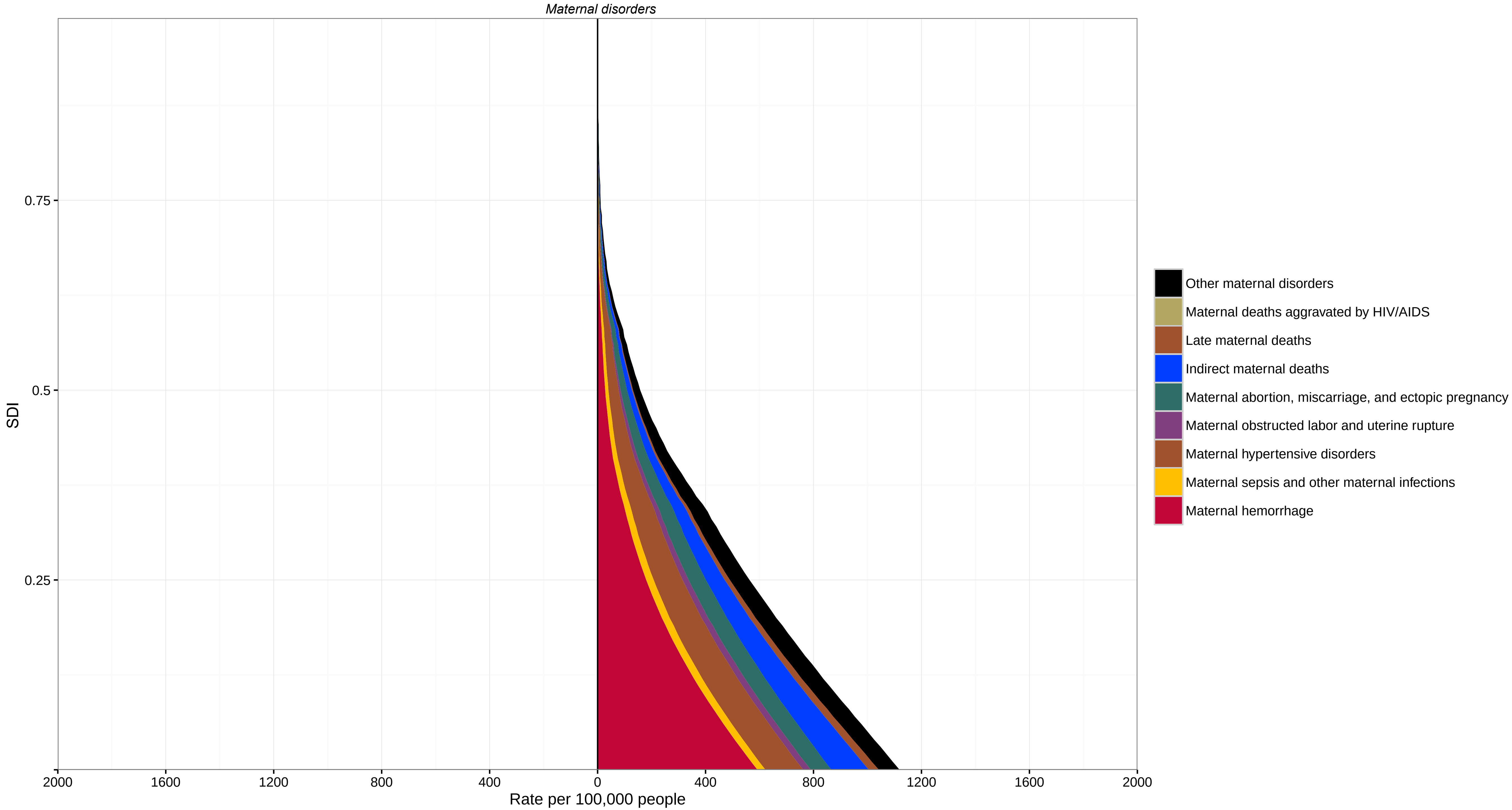

© 2017 Global Burden of Disease Child and Adolescent Health Collaboration. JAMA Pediatrics.

eFigure 8f. The expected relationship between cause-specific all-ages DALY rates for 0-19 years, and Sociodemographic Index (SDI) for males (left) and females (right)

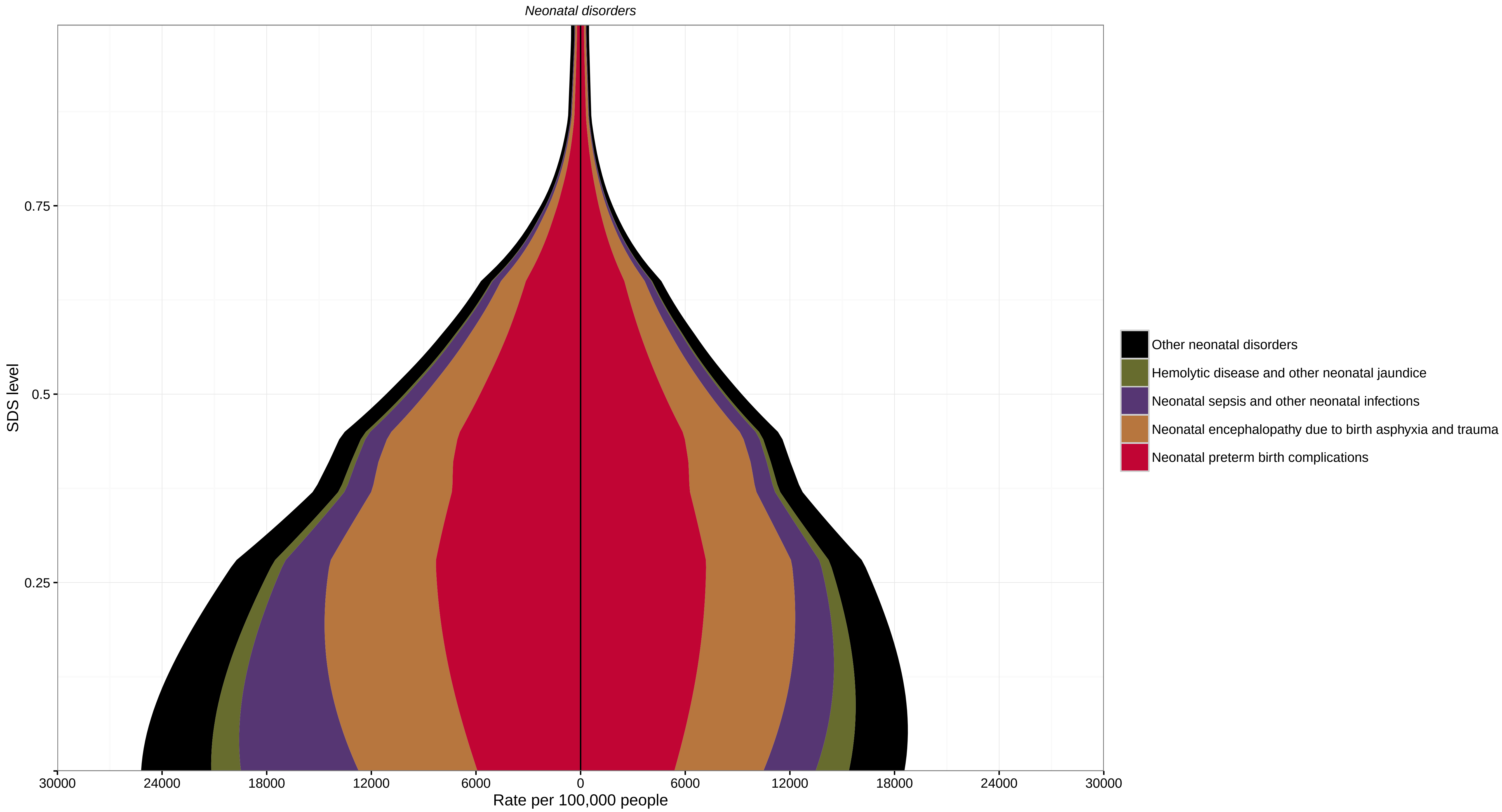

© 2017 Global Burden of Disease Child and Adolescent Health Collaboration. JAMA Pediatrics.

eFigure 8g. The expected relationship between cause-specific all-ages DALY rates for 0-19 years, and Sociodemographic Index (SDI) for males (left) and females (right) *Nutritional deficiencies*

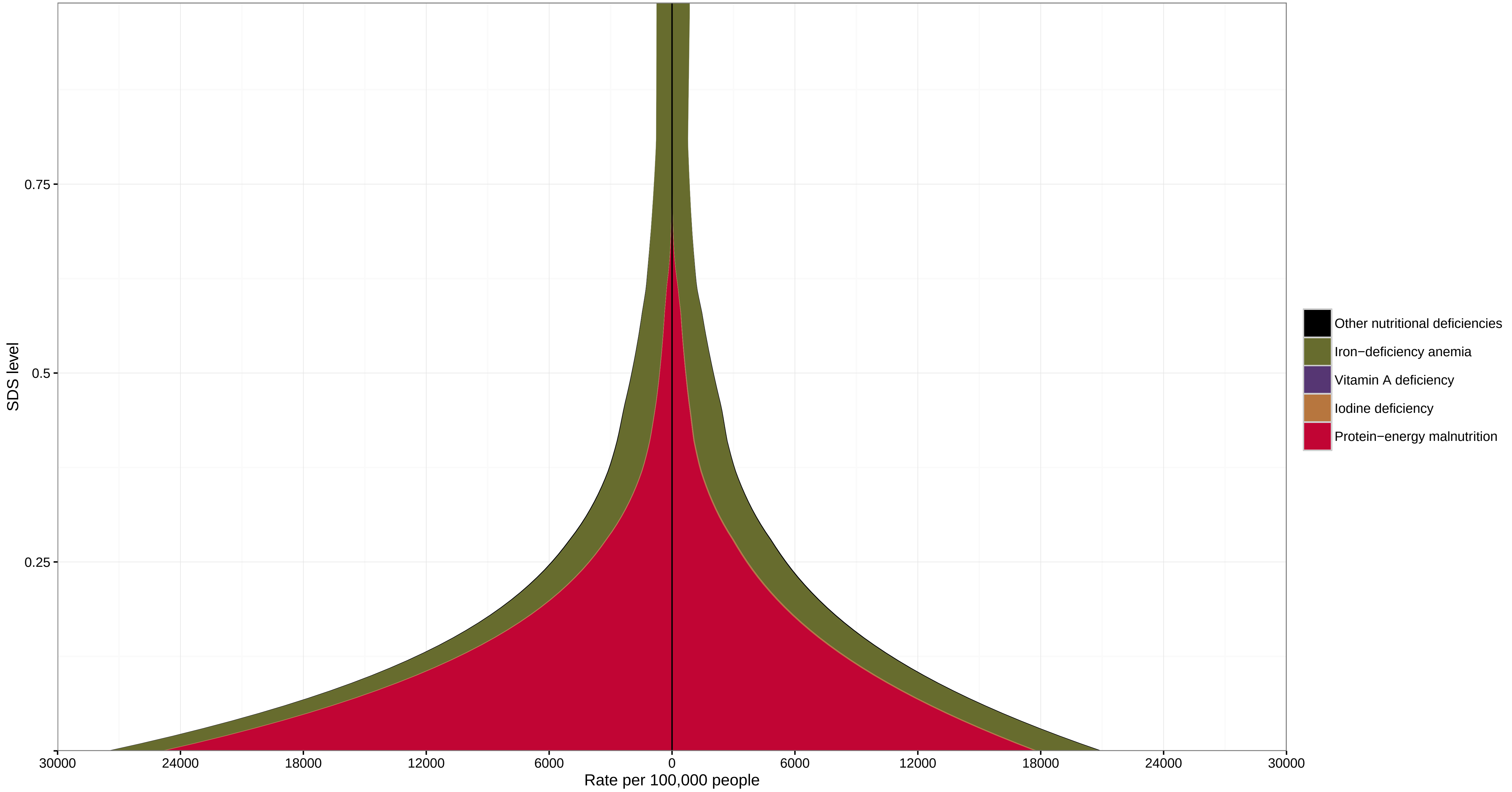

© 2017 Global Burden of Disease Child and Adolescent Health Collaboration. JAMA Pediatrics.

eFigure 8h: The expected relationship between cause-specific all ages DALY rates for 0-19 years, and Sociodemographic Index (SDI) for males (left) and females (left) *Other communicable, maternal, neonatal, and nutritional diseases*

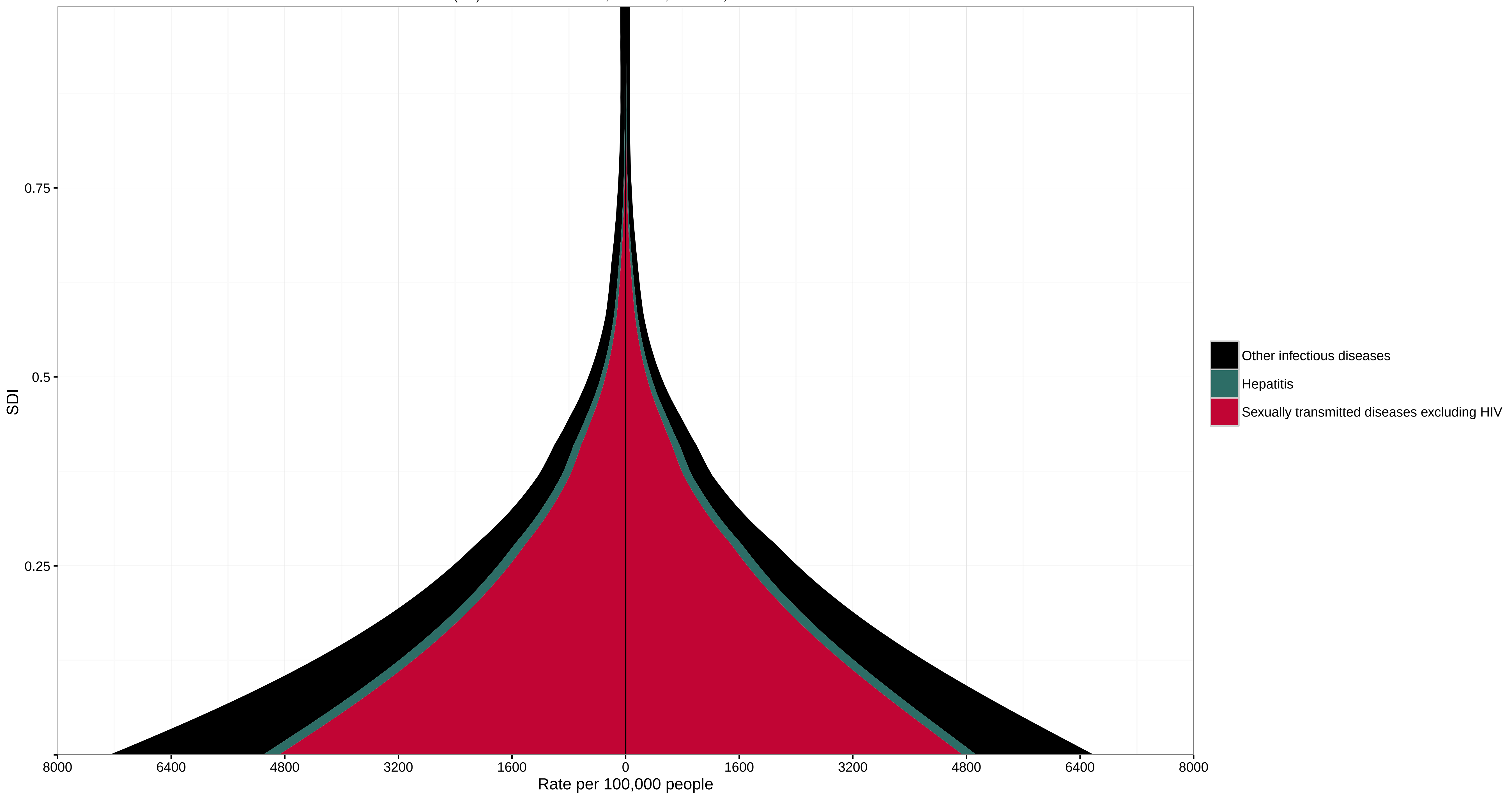

© 2017 Global Burden of Disease Child and Adolescent Health Collaboration. JAMA Pediatrics.

eFigure 8i. The expected relationship between cause-specific all ages DALY rates for 0-19 years, and Sociodemographic Index (SDI) for males (left) and females (left) *Neoplasms*

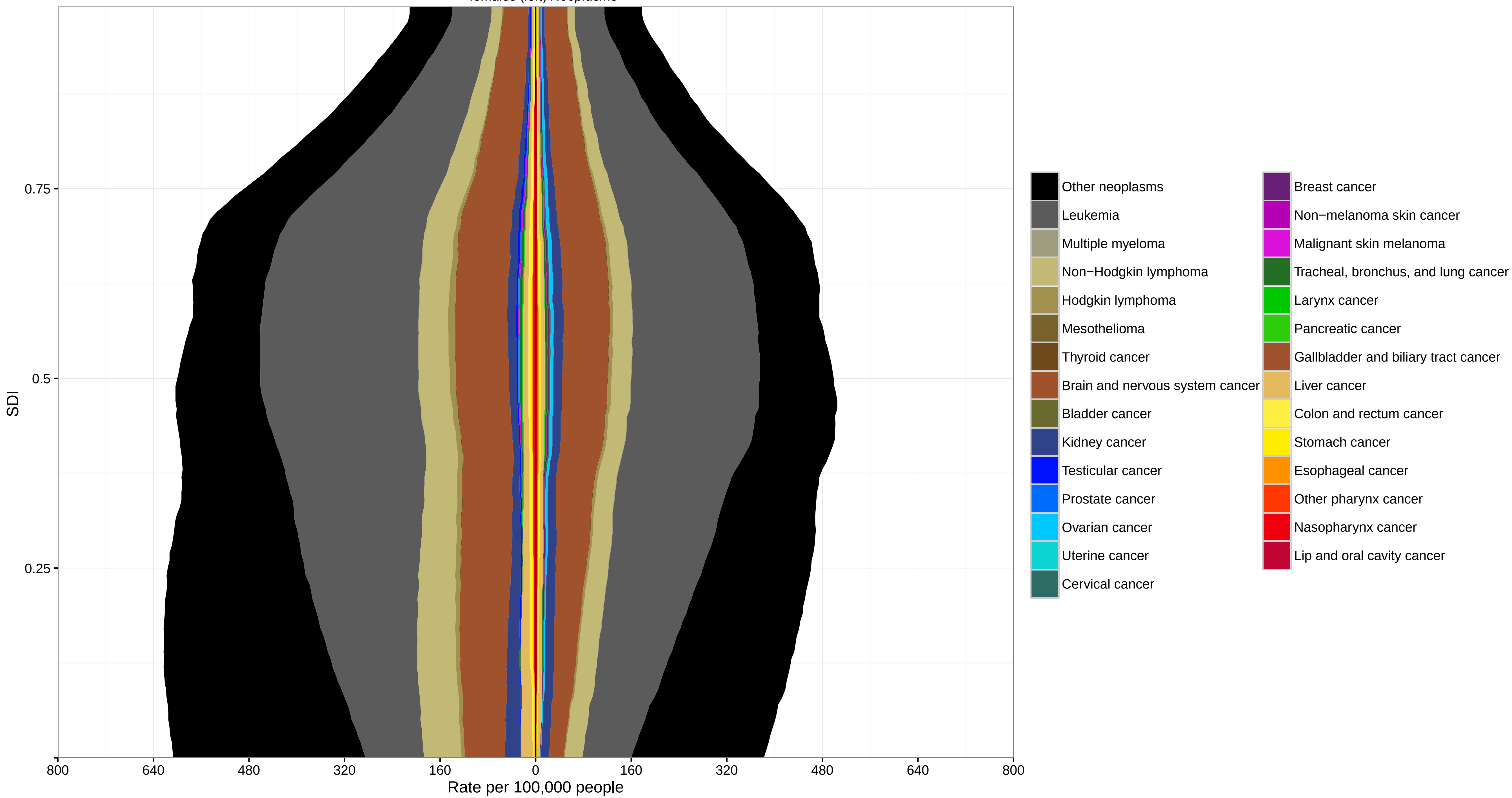

© 2017 Global Burden of Disease Child and Adolescent Health Collaboration. JAMA Pediatrics.

eFigure 8j. The expected relationship between cause-specific all ages DALY rates for 0-19 years, and Sociodemographic Index (SDI) for males (left) and females (left) *Cardiovascular diseases*

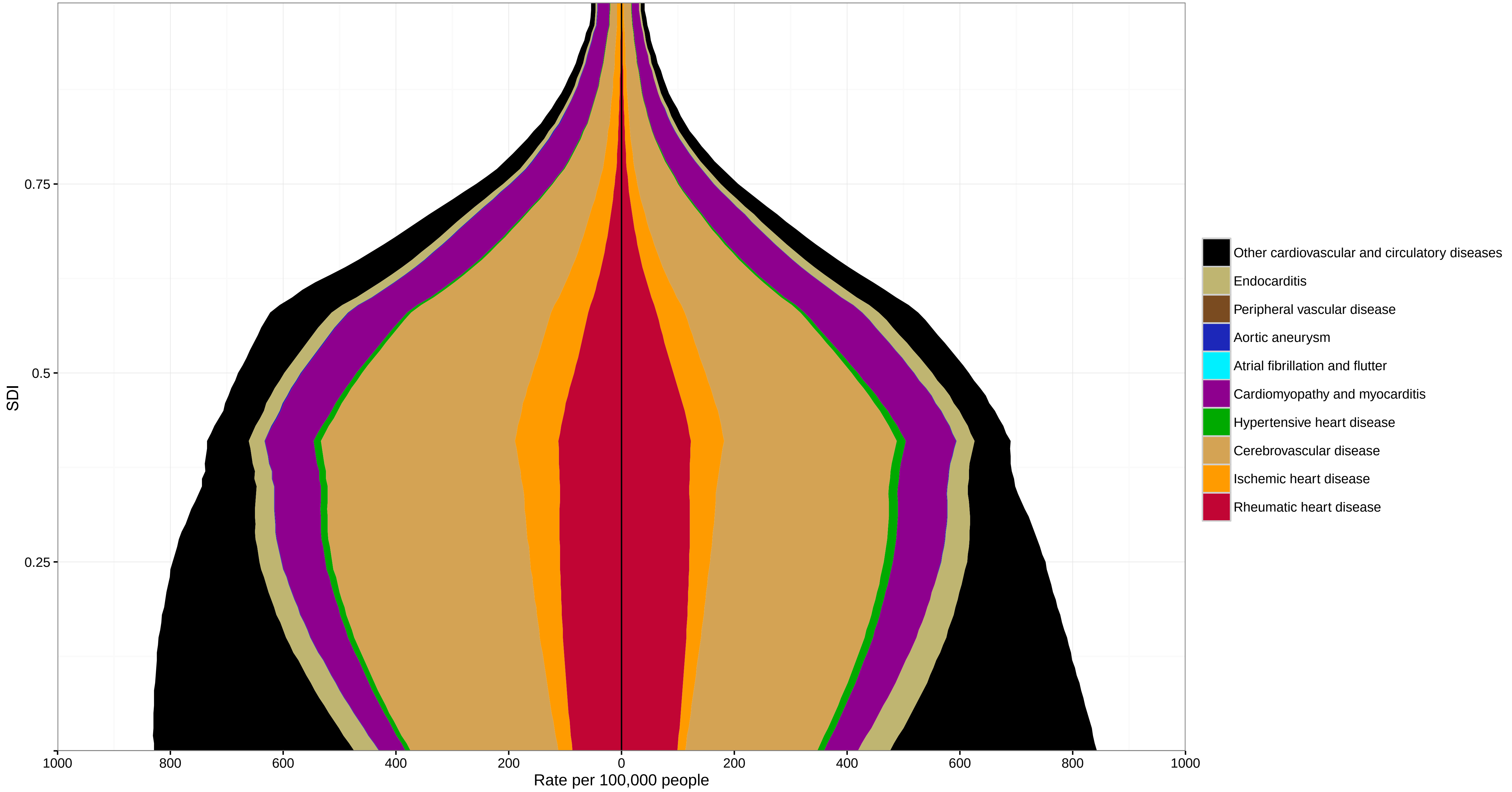

© 2017 Global Burden of Disease Child and Adolescent Health Collaboration. JAMA Pediatrics.

eFigure 8k. The expected relationship between cause-specific all ages DALY rates for 0-19 years, and Sociodemographic Index (SDI) for males (left) and females (left) *Chronic respiratory diseases*

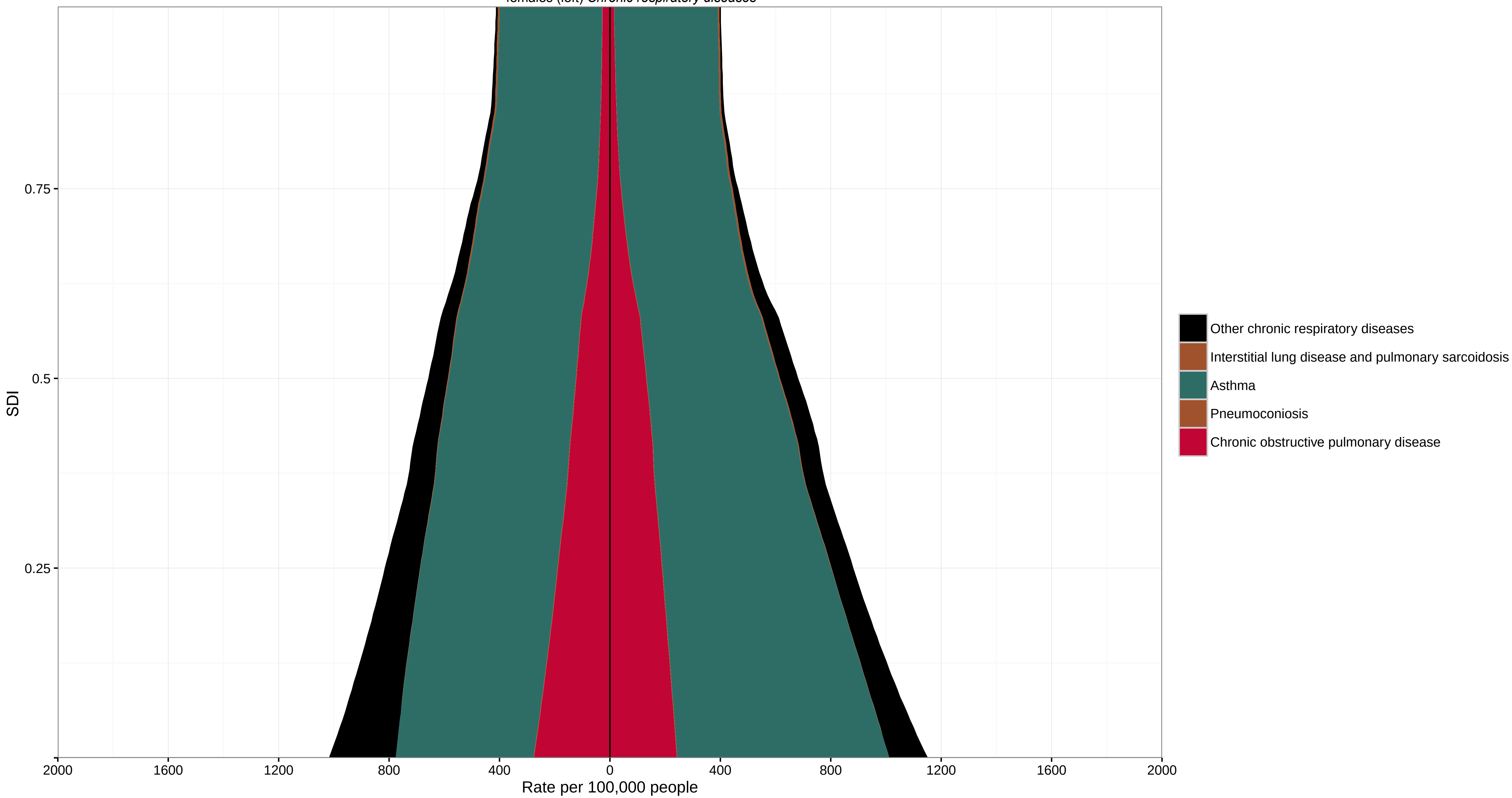

© 2017 Global Burden of Disease Child and Adolescent Health Collaboration. JAMA Pediatrics.

eFigure 8l. The expected relationship between cause-specific all ages DALY rates for 0-19 years, and Sociodemographic Index (SDI) for males (left) and females (left) *Cirrhosis and other chronic liver diseases*

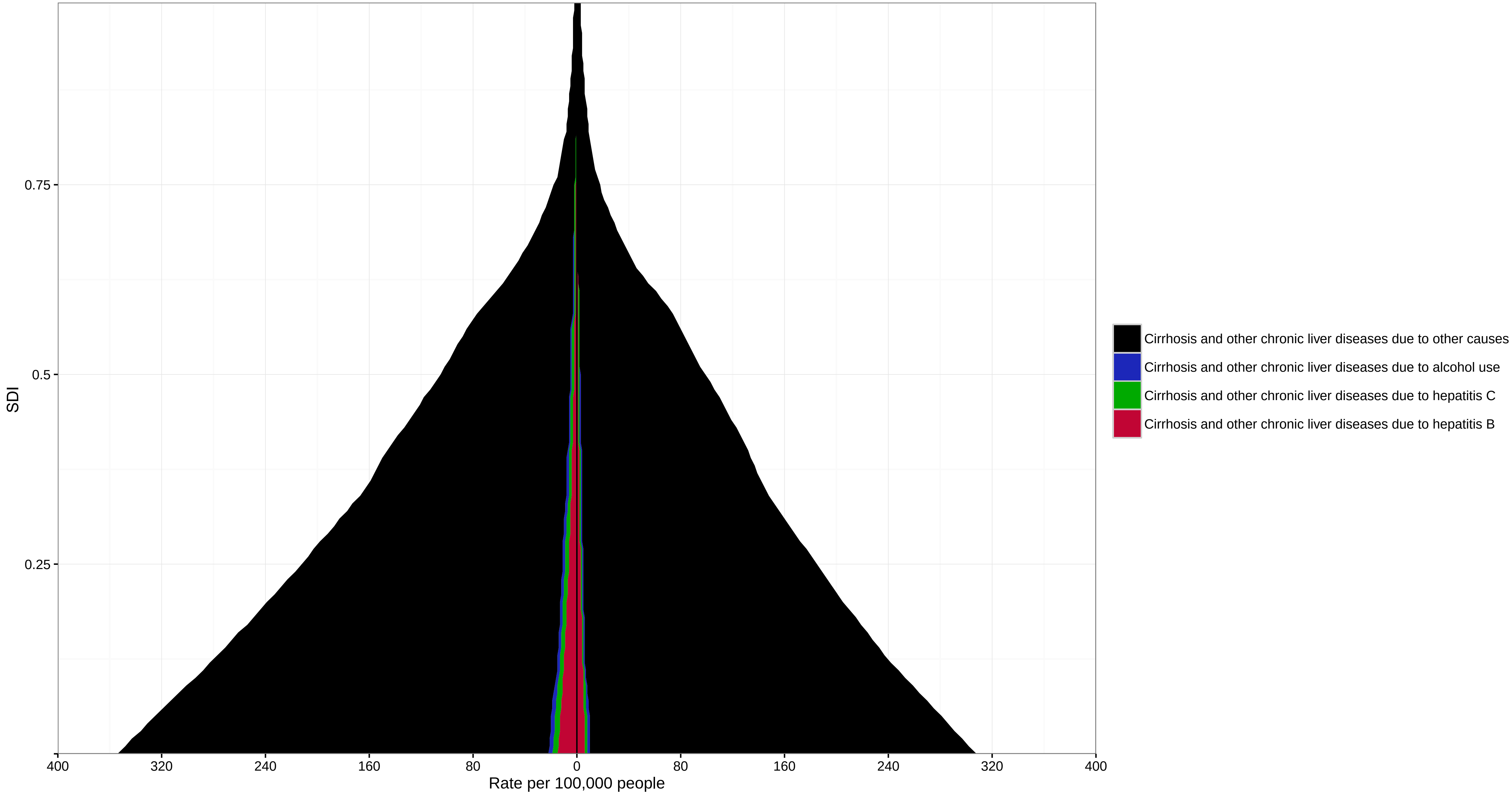

© 2017 Global Burden of Disease Child and Adolescent Health Collaboration. JAMA Pediatrics.

eFigure 8m. The expected relationship between cause-specific all ages DALY rates for 0-19 years, and Sociodemographic Index (SDI) for males (left) and females *Digestive diseases*

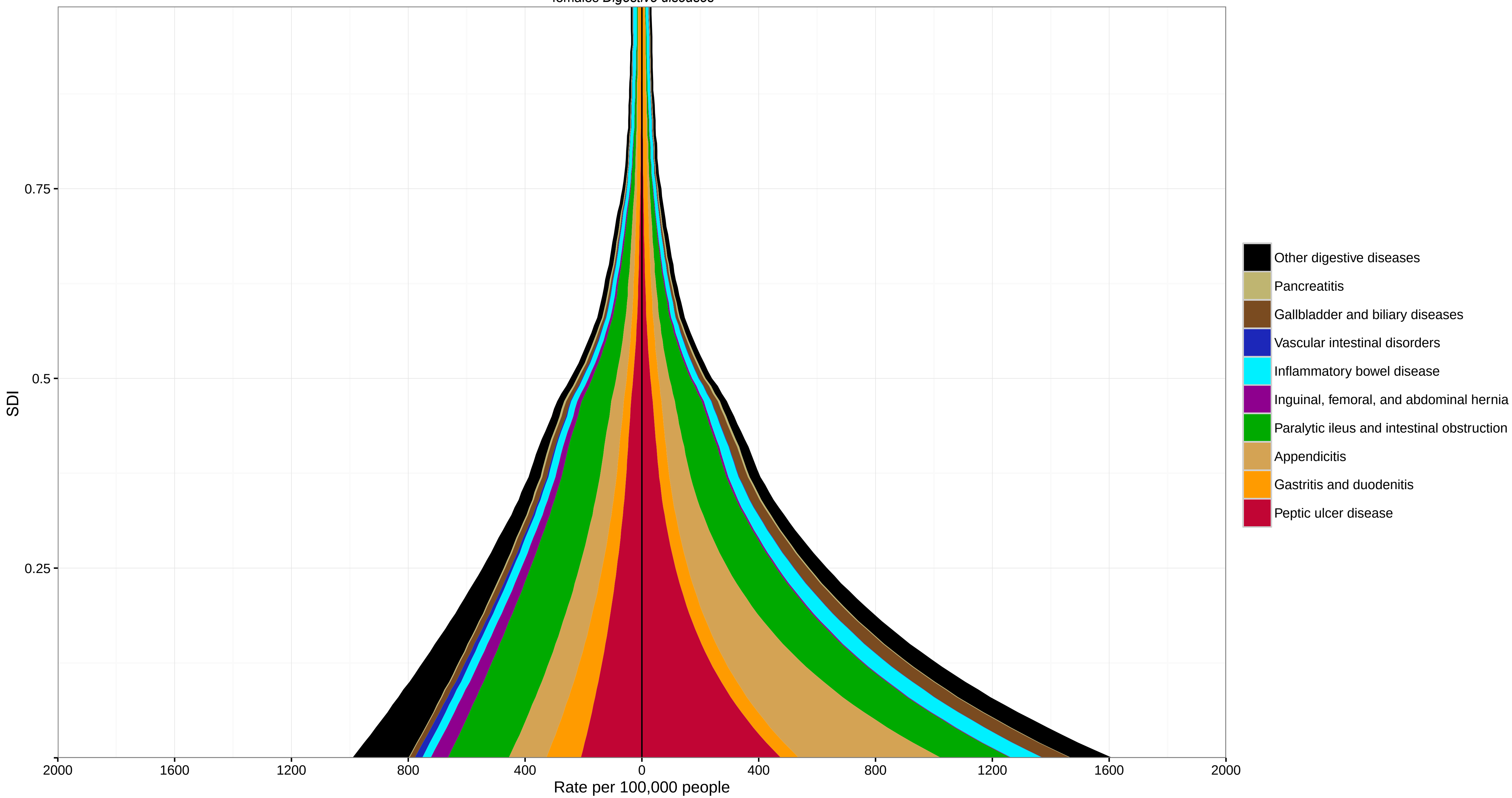

© 2017 Global Burden of Disease Child and Adolescent Health Collaboration. JAMA Pediatrics.

eFigure 8n. The expected relationship between cause-specific all-ages DALY rates for 0-19 years, and Sociodemographic Index (SDI) for males (left) and females (right) *Neurological disorders*

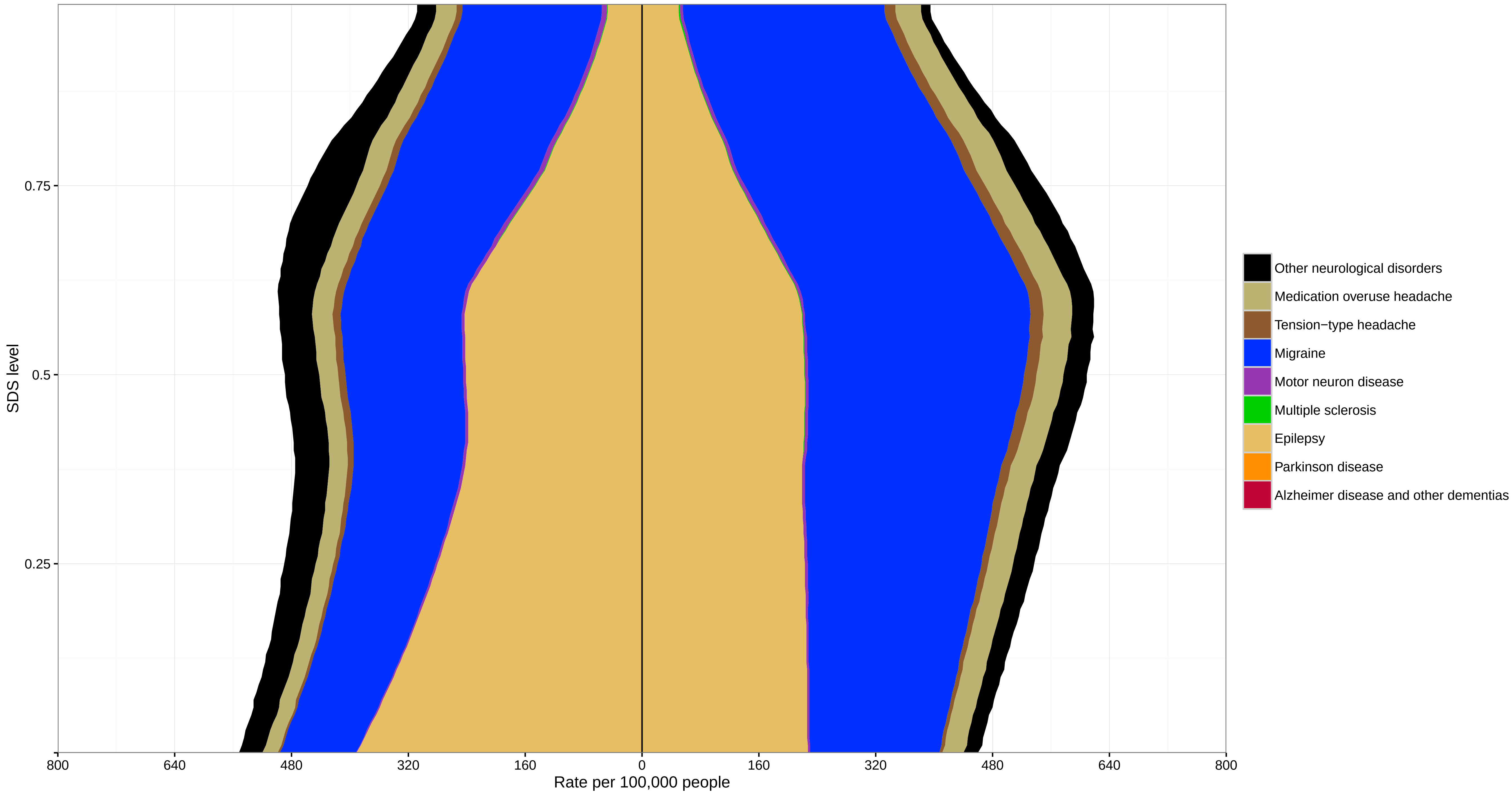

© 2017 Global Burden of Disease Child and Adolescent Health Collaboration. JAMA Pediatrics.

eFigure 8o. The expected relationship between cause-specific all-ages DALY rates for 0-19 years, and Sociodemographic Index (SDI) for males (left) and females *Mental and substance use disorders*

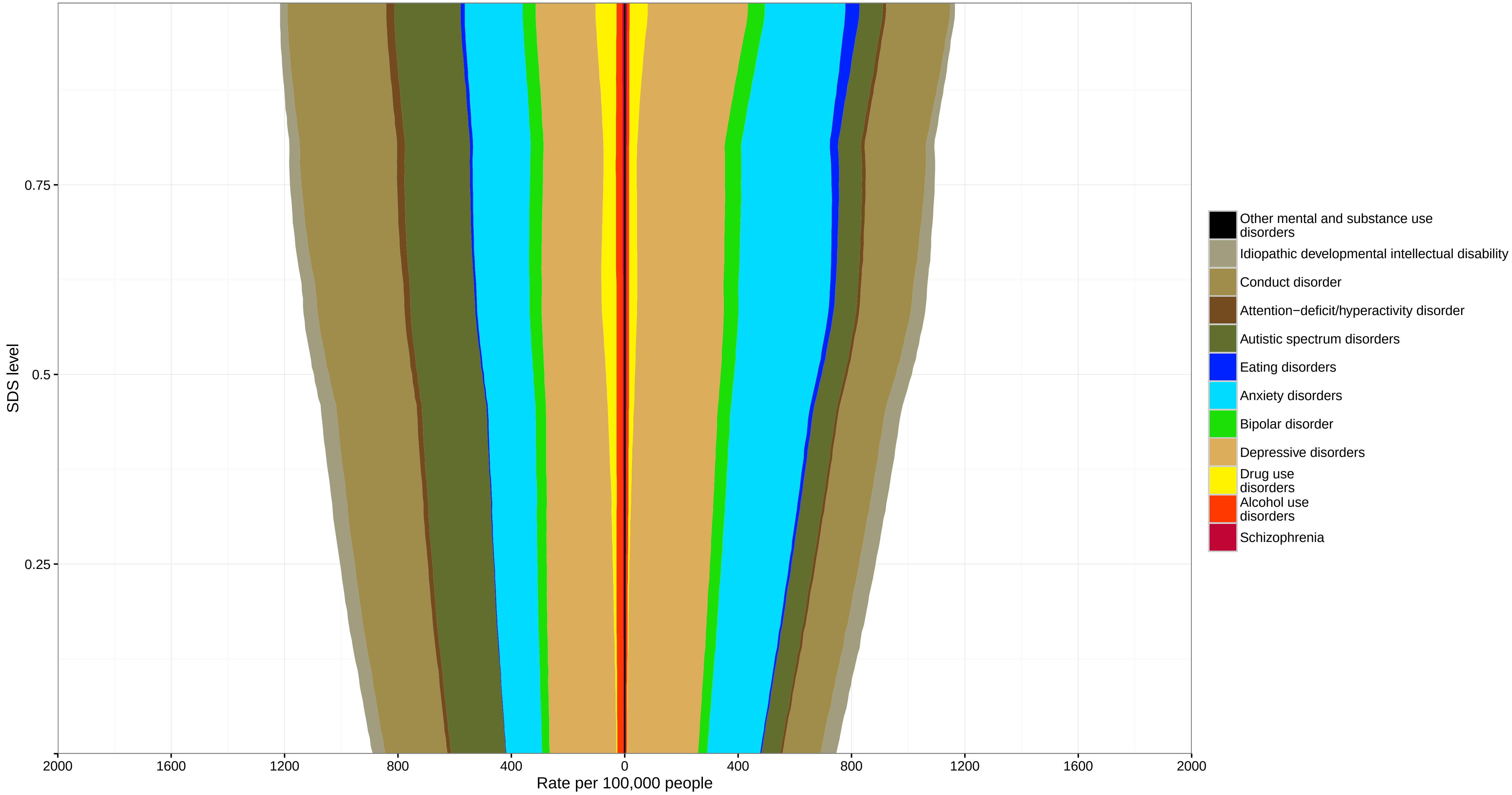

© 2017 Global Burden of Disease Child and Adolescent Health Collaboration. JAMA Pediatrics.

eFigure 8p: The expected relationship between cause-specific all ages DALY rates for 0-19 years, and Sociodemographic Index (SDI) for males (left) and females *Diabetes, urogenital, blood and endocrine disorders*

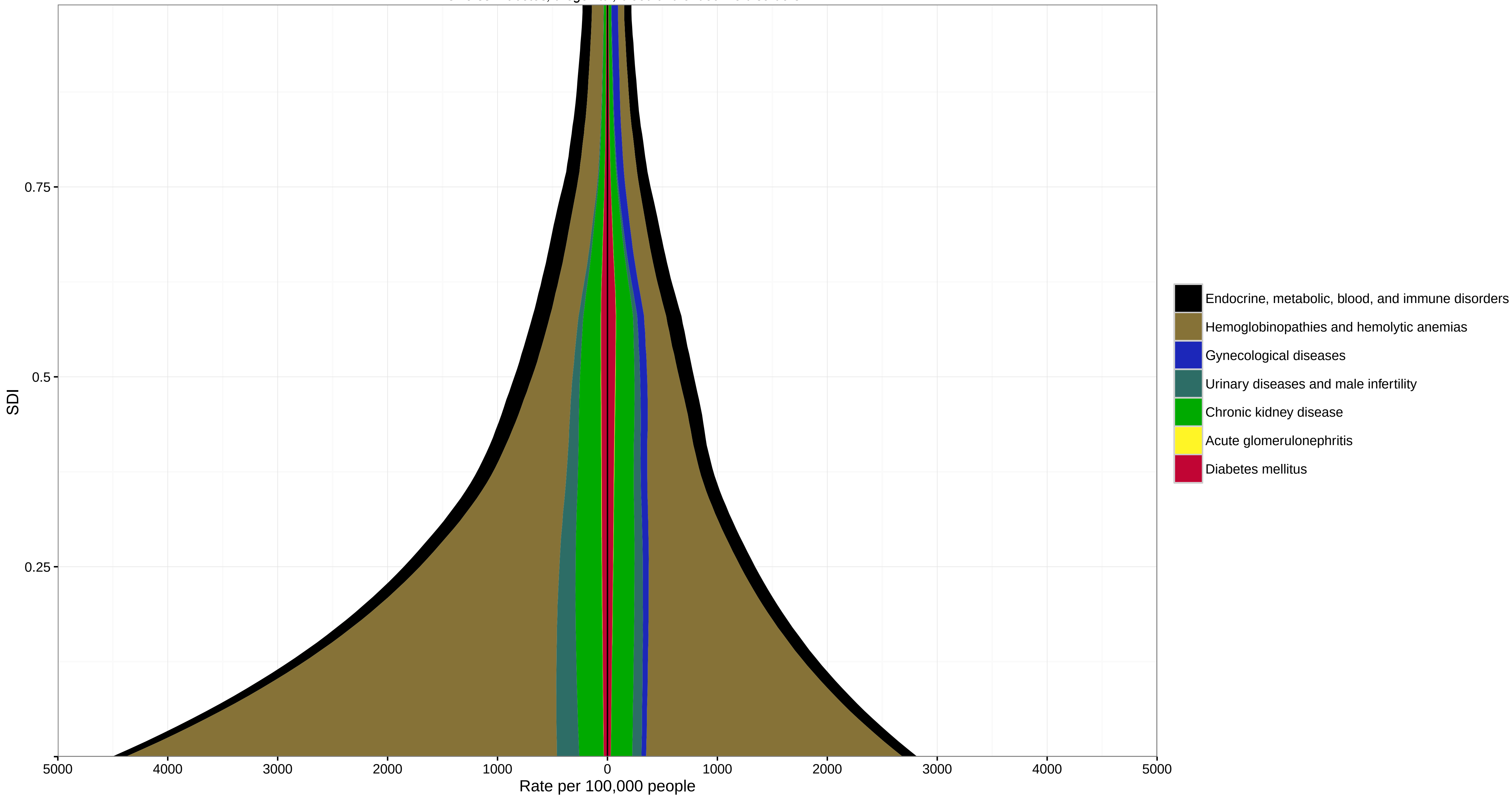

© 2017 Global Burden of Disease Child and Adolescent Health Collaboration. JAMA Pediatrics.

eFigure 8q: The expected relationship between cause-specific all ages DALY rates for 0-19 years, and Sociodemographic Index (SDI) for males (left) and females (right) *Musculoskeletal disorders*

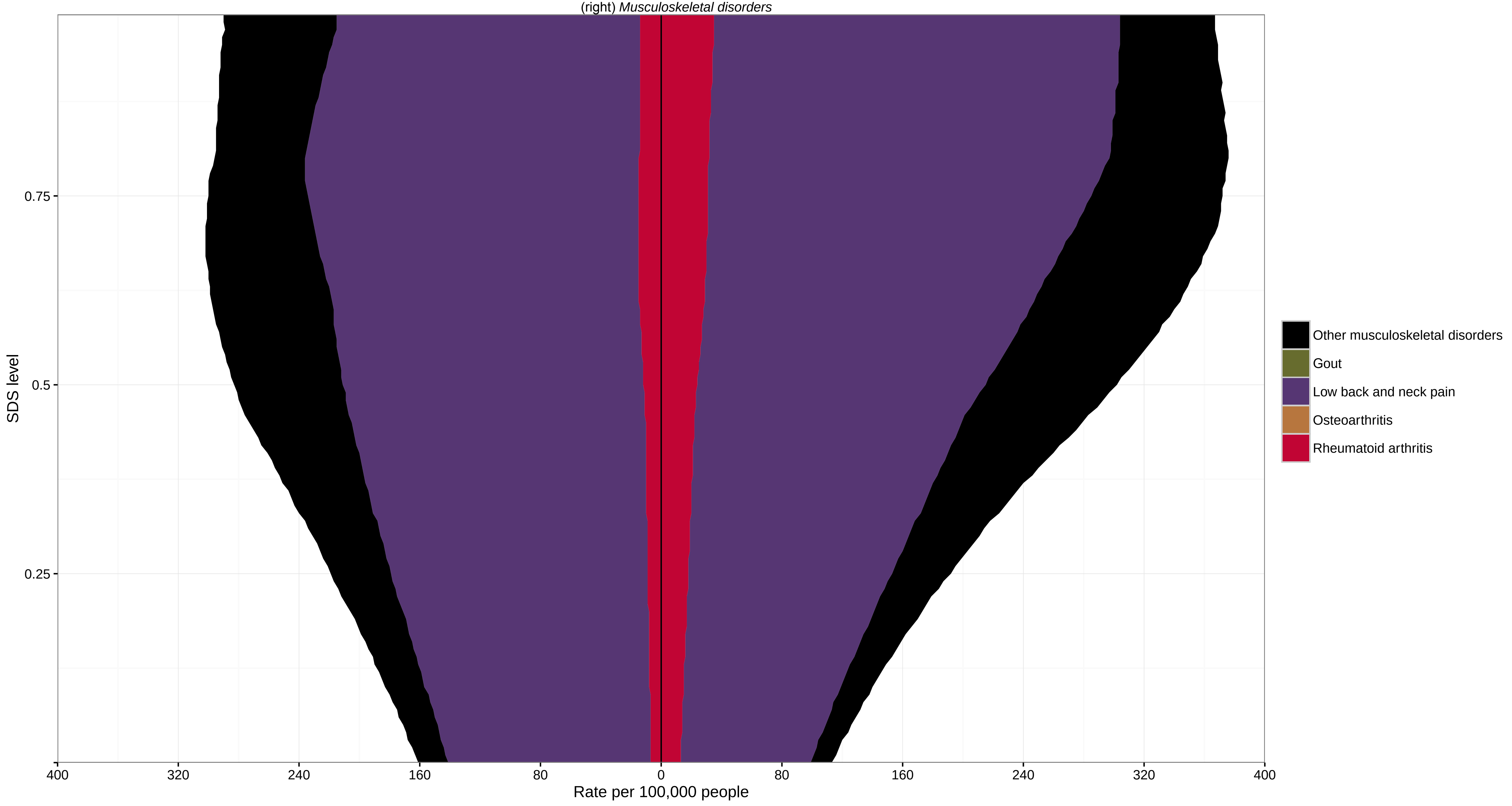

© 2017 Global Burden of Disease Child and Adolescent Health Collaboration. JAMA Pediatrics.

eFigure 8r: The expected relationship between cause-specific all ages DALY rates for 0-19 years, and Sociodemographic Index (SDI) for males (left) and females *Other non−communicable diseases*

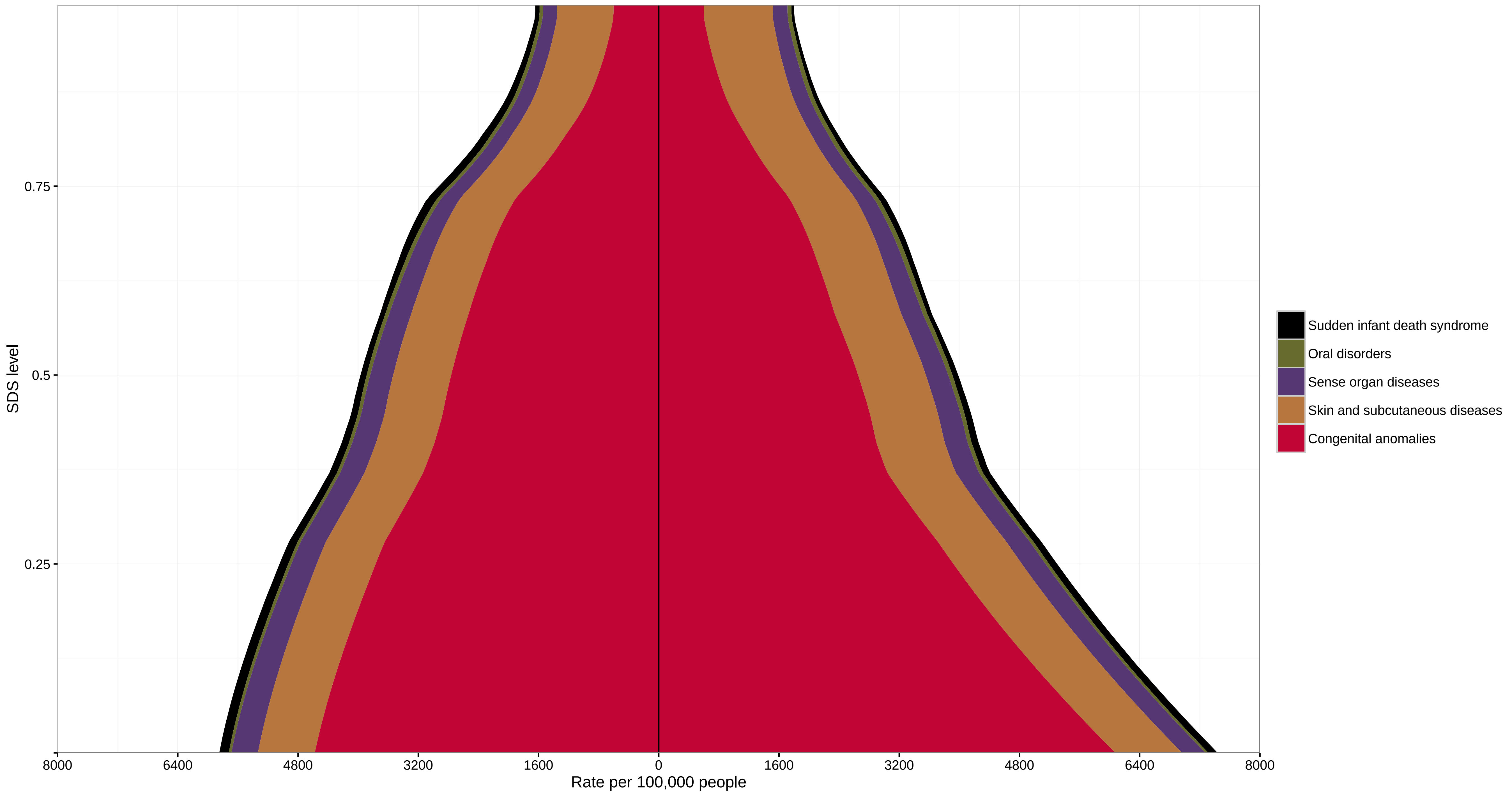

© 2017 Global Burden of Disease Child and Adolescent Health Collaboration. JAMA Pediatrics.

eFigure 8s: The expected relationship between cause-specific all ages DALY rates for 0-19 years, and Sociodemographic Index (SDI) for males (left) and females *Transport injuries*

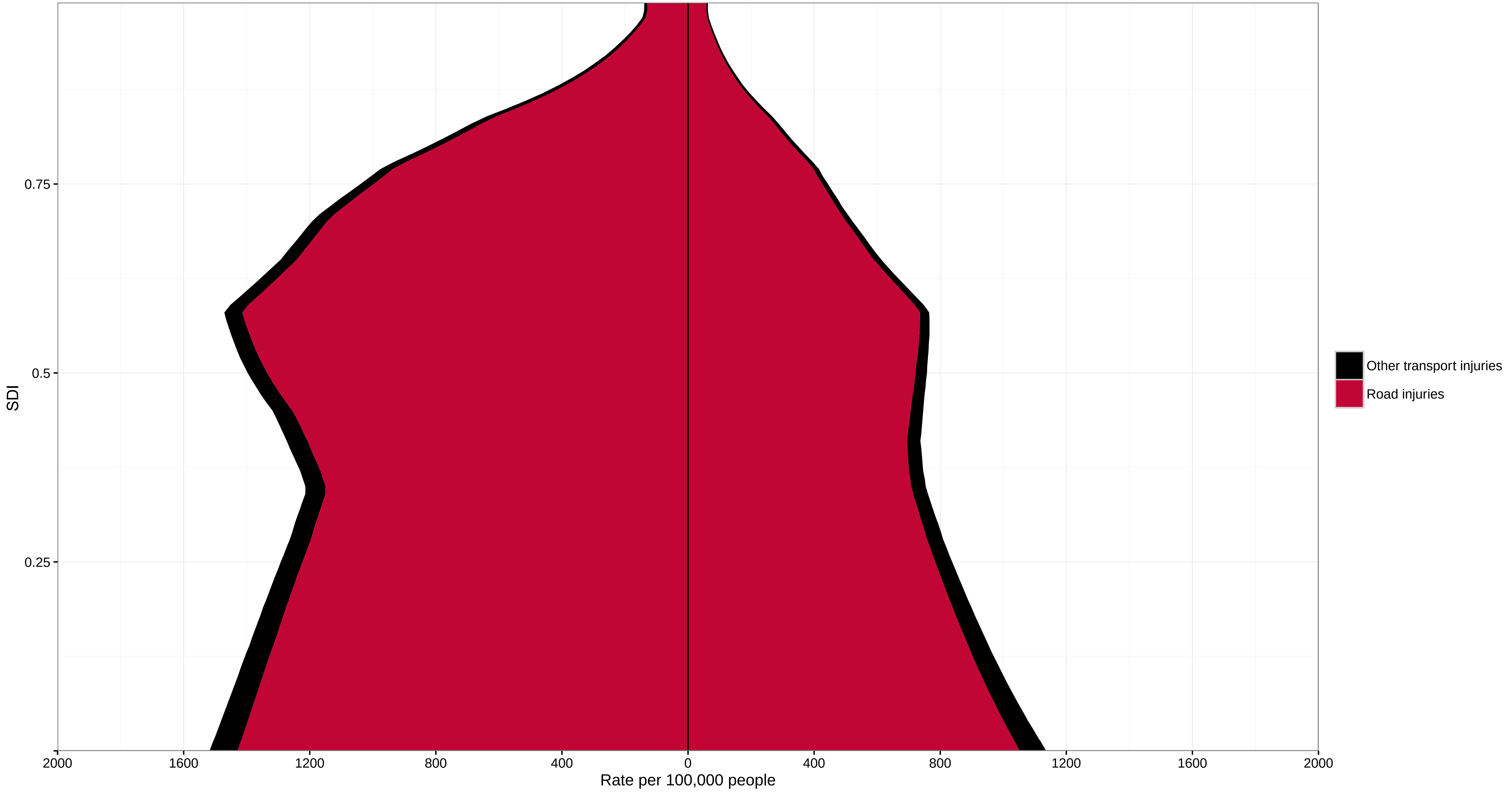

© 2017 Global Burden of Disease Child and Adolescent Health Collaboration. JAMA Pediatrics.

eFigure 8t: The expected relationship between cause-specific all ages DALY rates for 0-19 years, and Sociodemographic Index (SDI) for males (left) and females *Unintentional injuries*

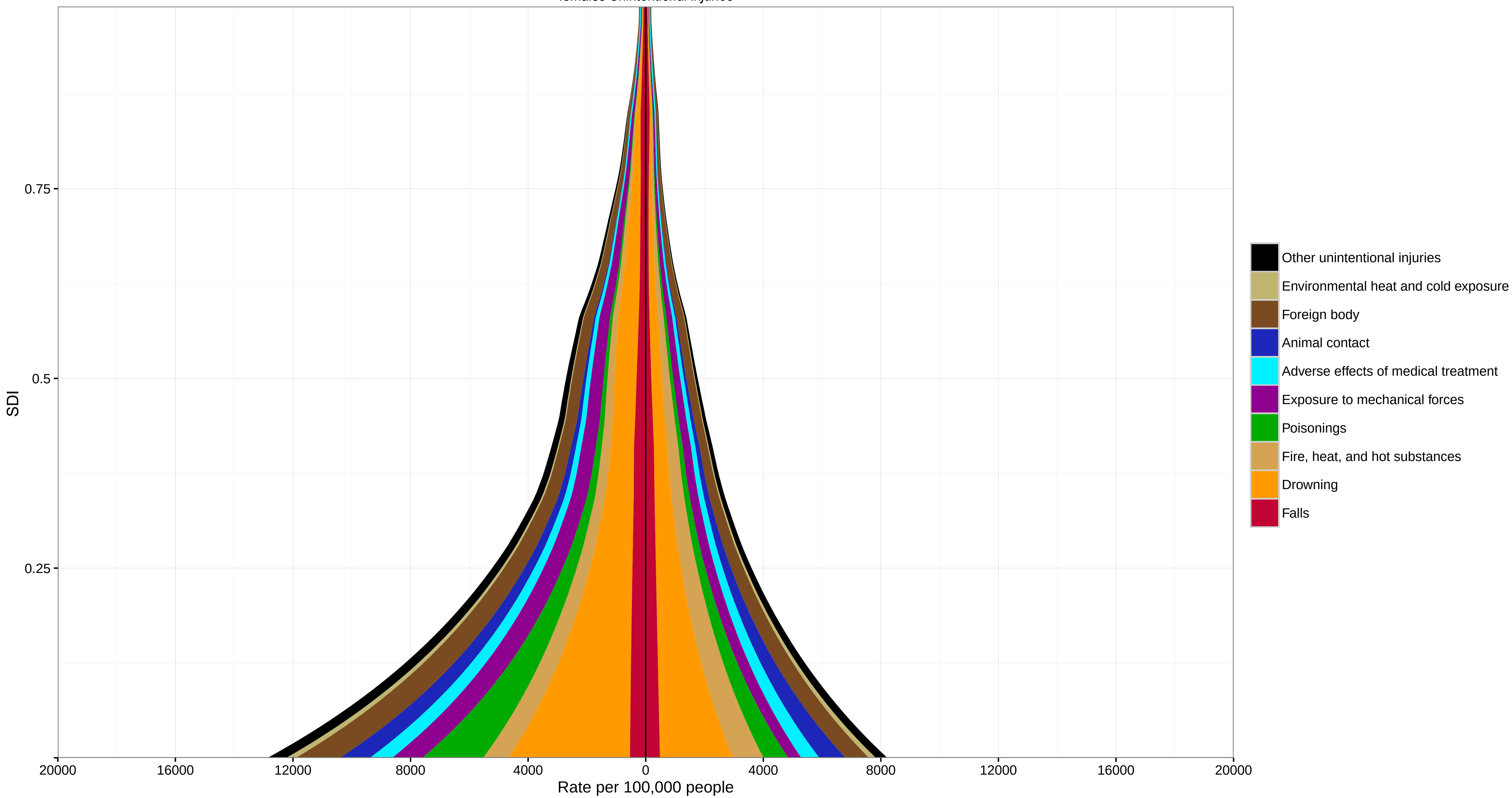

© 2017 Global Burden of Disease Child and Adolescent Health Collaboration. JAMA Pediatrics.

eFigure 8u: The expected relationship between cause-specific all ages DALY rates for 0-19 years, and Sociodemographic Index (SDI) for males (left) and females *Self-harm and interpersonal violence*

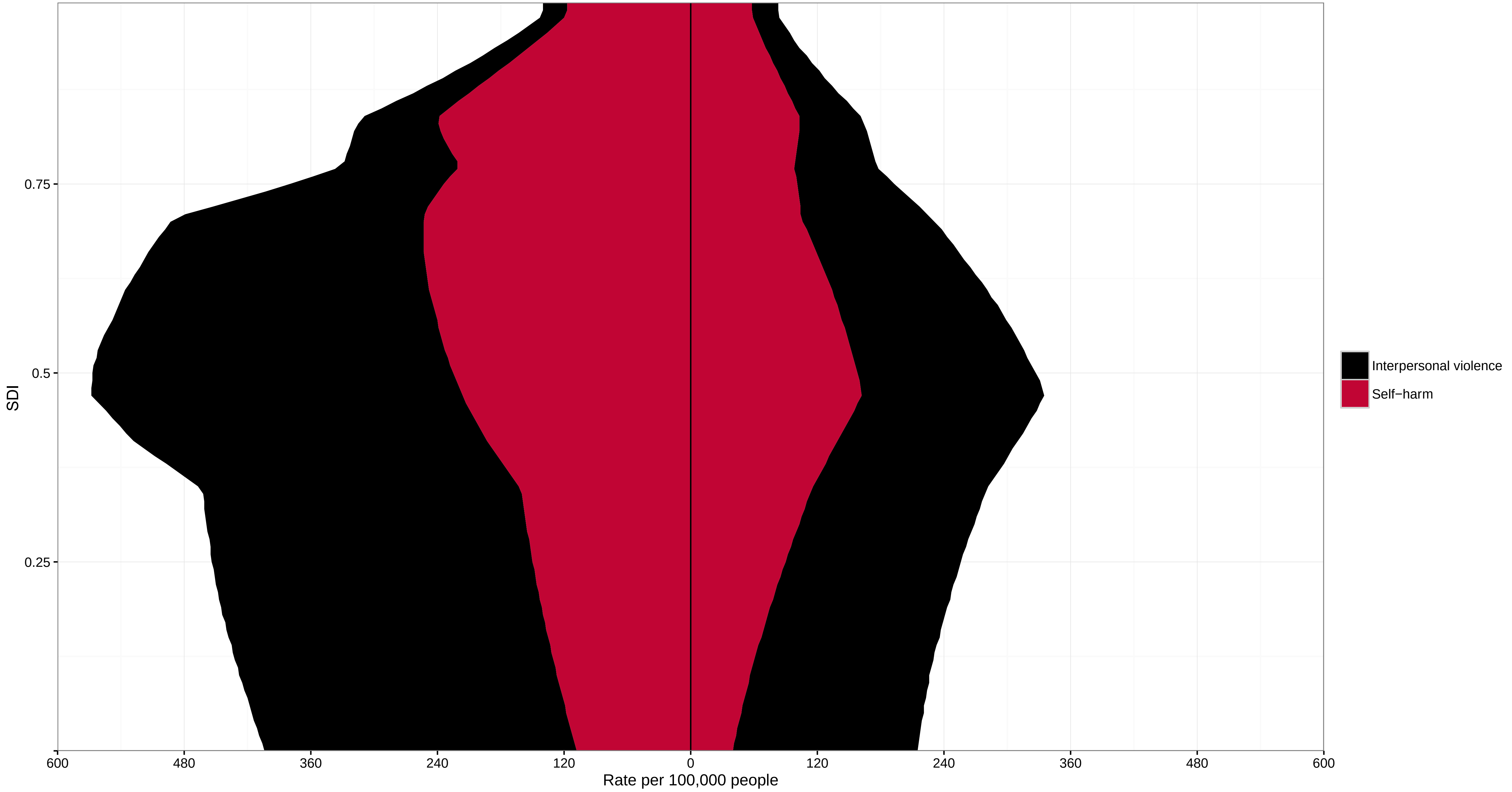

© 2017 Global Burden of Disease Child and Adolescent Health Collaboration. JAMA Pediatrics.

eFigure 8v: The expected relationship between cause-specific all ages DALY rates for 0-19 years, and Sociodemographic Index (SDI) for males (left) and females *Forces of nature, war, and legal intervention*

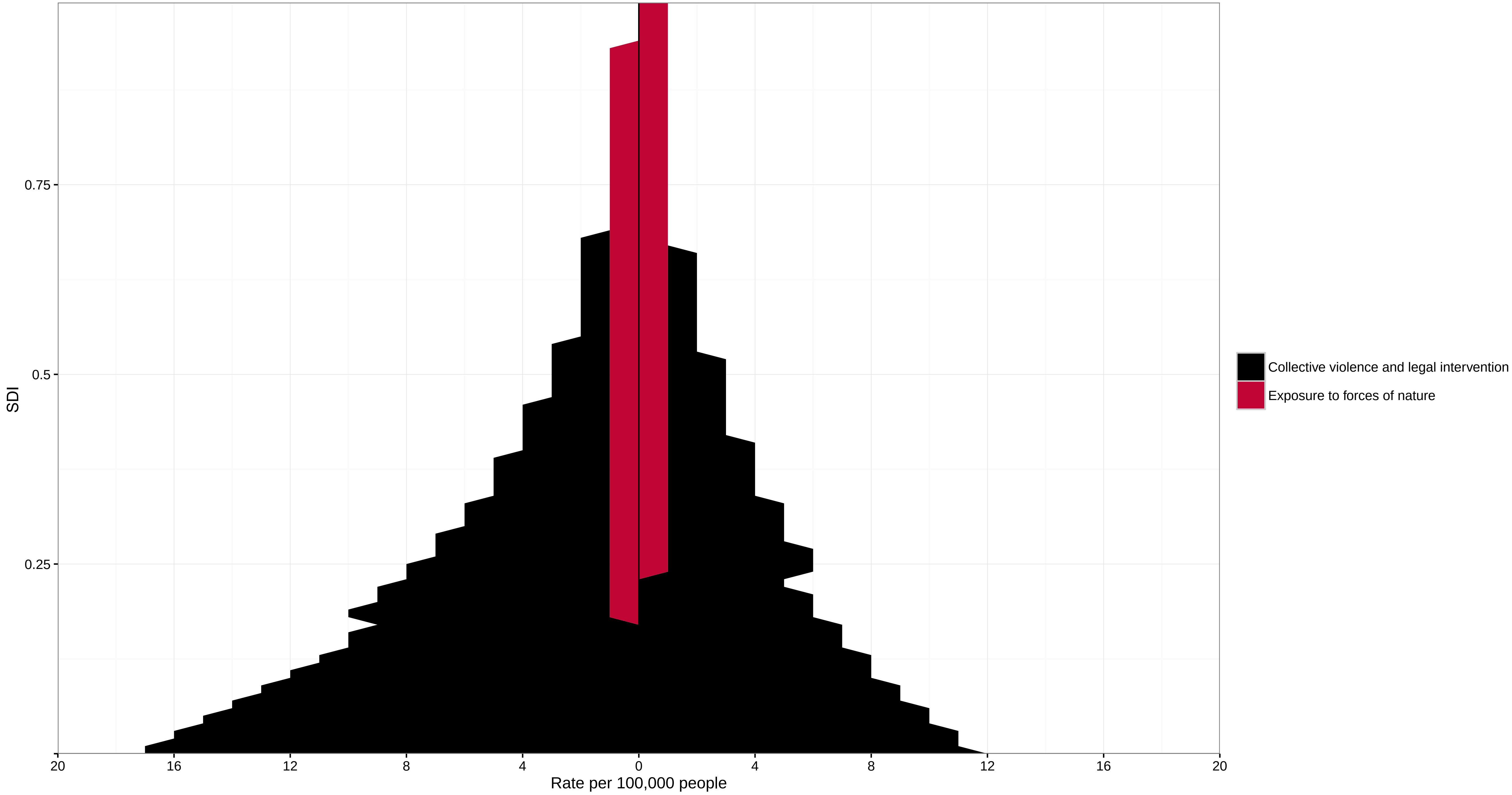

© 2017 Global Burden of Disease Child and Adolescent Health Collaboration. JAMA Pediatrics.

eTable 1. Number of Deaths, Death Rates (per 100,000 population), and Cumulative Percent Change with 95% Uncertainty Intervals (UI) for the Top 10 Global Causes of Death in 195 Countries and Territories, Aged 0 to 19, Both Sexes, 1990 and 2015 *Note: best viewed by enlarging in browser.*

Location	Measure	All causes	Neonatal preterm birth complications	Lower respiratory infections	Neonatal encephalopathy due to birth asphyxia and trauma	Diarrheal diseases	Congenital anomalies	Malaria	Neonatal sepsis and other neonatal infections	Meningitis	Other neonatal disorders	HIV/AIDS
Global	Number: 1990	14,182,624 (14,091,033–14,283,791)	1,795,211 (1,685,077–1,918,767)	2,241,773 (2,051,590–2,406,937)	915,323 (810,730–1,021,011)	1,536,806 (1,374,949–1,681,056)	696,037 (530,479–963,077)	791,867 (596,008–1,002,740)	329,296 (247,585–418,271)	376,652 (310,915–433,223)	351,304 (265,375–451,582)	39,363 (36,752–42,612)
Global	Number: 2015	7,263,484 (7,140,895–7,391,912)	805,778 (747,783–876,860)	792,992 (744,203–842,524)	740,424 (680,024–809,507)	569,737 (522,612–616,479)	543,314 (502,636–590,592)	534,007 (412,686–651,639)	351,667 (275,540–428,214)	220,530 (186,969–261,596)	220,247 (174,247–266,264)	202,929 (196,121–209,978)
Global	Number: Cumulative change (%)	-48.8 (-49.8 to -47.8)	-55.1 (-58.3 to -51.7)	-64.6 (-67.4 to -61.3)	-18.8 (-27.1 to -9.3)	-62.8 (-66.9 to -57.8)	-20.0 (-40.0 to -2.0)	-31.6 (-47.4 to -12.2)	8.0 (-11.9 to 32.5)	-41.0 (-50.4 to -25.8)	-36.2 (-50.6 to -19.3)	416.5 (378.4–450.5)
Global	Rate: 1990	584.6 (580.8–588.8)	71.5 (67.1–76.4)	91.6 (83.8–98.4)	36.4 (32.3–40.6)	63.3 (56.7–69.3)	28.3 (21.5–39.1)	32.9 (24.9–41.6)	13.1 (9.9–16.7)	15.6 (13.0–18.0)	14.0 (10.6–18.0)	1.6 (1.5–1.8)
Global	Rate: 2015	285.4 (280.6–290.5)	31.4 (29.1–34.2)	31.1 (29.2–33.0)	28.8 (26.5–31.5)	22.4 (20.5–24.2)	21.3 (19.7–23.1)	21.0 (16.2–25.6)	13.7 (10.7–16.7)	8.7 (7.4–10.3)	8.6 (6.8–10.4)	8.1 (7.8–8.3)
Global	Rate: Cumulative change (%)	-51.2 (-52.1 to -50.3)	-56.0 (-59.2 to -52.7)	-66.0 (-68.8 to -62.8)	-20.5 (-28.6 to -11.2)	-64.6 (-68.5 to -59.8)	-22.9 (-42.2 to -5.5)	-35.3 (-50.1 to -17.4)	5.5 (-14.0 to 29.6)	-44.0 (-52.9 to -29.6)	-37.7 (-51.7 to -21.2)	397.4 (360.5–430.1)
High SDI	Number: 1990	295,736 (293,186–298,672)	42,760 (41,201–45,248)	17,451 (16,746–18,329)	17,881 (16,834–18,986)	3,350 (3,117–3,591)	50,953 (42,397–53,094)	9 (6–12)	4,644 (3,765–7,181)	4,573 (4,387–4,794)	9,008 (8,499–9,583)	927 (912–943)
High SDI	Number: 2015	118,122 (116,761–119,589)	13,493 (12,118–14,234)	4,399 (4,151–4,654)	5,509 (4,985–5,922)	832 (776–880)	23,775 (22,232–27,540)	1 (1–1)	2,920 (2,334–4,355)	958 (911–1,014)	5,802 (5,173–6,245)	699 (646–754)
High SDI	Number: Cumulative change (%)	-60.1 (-60.7 to -59.5)	-68.4 (-72.9 to -66.3)	-74.8 (-76.9 to -73.0)	-69.2 (-72.6 to -66.5)	-75.1 (-77.6 to -72.6)	-53.0 (-57.7 to -34.9)	-83.2 (-88.1 to -78.6)	-36.6 (-45.6 to -29.6)	-79.0 (-80.5 to -77.5)	-35.5 (-44.5 to -29.6)	-24.6 (-30.5 to -18.5)
High SDI	Rate: 1990	100.5 (99.6–101.6)	15.5 (15.0–16.4)	6.1 (5.8–6.4)	6.5 (6.1–6.9)	1.2 (1.1–1.3)	18.1 (15.0–18.9)	0.0 (0.0–0.0)	1.7 (1.4–2.6)	1.6 (1.5–1.7)	3.3 (3.1–3.5)	0.3 (0.3–0.3)
High SDI	Rate: 2015	43.9 (43.4–44.5)	5.2 (4.7–5.5)	1.7 (1.6–1.8)	2.1 (1.9–2.3)	0.3 (0.3–0.3)	9.1 (8.5–10.5)	0.0 (0.0–0.0)	1.1 (0.9–1.7)	0.4 (0.3–0.4)	2.2 (2.0–2.4)	0.3 (0.2–0.3)
High SDI	Rate: Cumulative change (%)	-56.3 (-57.0 to -55.7)	-66.3 (-71.1 to -64.1)	-72.8 (-75.1 to -70.8)	-67.1 (-70.8 to -64.3)	-73.2 (-75.9 to -70.5)	-49.4 (-54.5 to -29.9)	-81.1 (-86.6 to -75.8)	-32.4 (-42.1 to -25.0)	-77.2 (-78.8 to -75.4)	-31.2 (-40.8 to -25.0)	-15.7 (-22.4 to -8.9)
High-middle SDI	Number: 1990	1,666,079 (1,635,236–1,700,432)	286,715 (272,767–303,522)	248,629 (234,416–262,892)	96,928 (89,645–106,292)	107,312 (99,844–115,205)	161,837 (125,384–190,683)	918 (772–1,099)	25,555 (21,607–32,750)	32,409 (24,155–38,518)	54,939 (46,951–63,782)	1,269 (1,176–1,387)
High-middle SDI	Number: 2015	536,318 (522,370–551,127)	75,776 (70,021–81,741)	43,653 (40,843–47,174)	49,568 (45,191–54,954)	11,265 (10,184–12,498)	79,782 (67,333–87,094)	428 (294–605)	18,756 (14,773–21,786)	7,066 (6,264–7,956)	21,014 (18,635–23,402)	21,175 (19,517–23,025)
High-middle SDI	Number: Cumulative change (%)	-67.8 (-68.9 to -66.7)	-73.6 (-75.7 to -71.2)	-82.4 (-83.9 to -80.7)	-48.8 (-53.6 to -43.6)	-89.5 (-90.8 to -88.1)	-49.9 (-63.3 to -36.8)	-52.8 (-69.3 to -31.5)	-26.1 (-36.0 to -15.6)	-77.8 (-82.0 to -71.9)	-61.4 (-68.0 to -53.8)	1,573.0 (1,392.7–1,761.4)
High-middle SDI	Rate: 1990	319.0 (313.1–325.5)	54.8 (52.1–58.0)	47.4 (44.7–50.2)	18.5 (17.1–20.3)	20.5 (19.0–22.0)	30.9 (24.0–36.4)	0.2 (0.1–0.2)	4.9 (4.1–6.3)	6.2 (4.6–7.4)	10.5 (9.0–12.2)	0.2 (0.2–0.3)
High-middle SDI	Rate: 2015	118.3 (115.1–121.7)	17.4 (16.1–18.8)	9.7 (9.1–10.5)	11.4 (10.4–12.6)	2.5 (2.3–2.8)	18.0 (15.2–19.7)	0.1 (0.1–0.1)	4.3 (3.4–5.0)	1.5 (1.4–1.7)	4.8 (4.3–5.4)	4.6 (4.2–5.0)
High-middle SDI	Rate: Cumulative change (%)	-62.9 (-64.2 to -61.6)	-68.2 (-70.8 to -65.4)	-79.5 (-81.3 to -77.4)	-38.4 (-44.2 to -32.2)	-87.7 (-89.3 to -86.1)	-40.9 (-56.7 to -25.4)	-48.8 (-66.8 to -25.8)	-11.3 (-23.1 to 1.3)	-74.6 (-79.4 to -67.9)	-53.7 (-61.6 to -44.6)	1,804.9 (1,598.6–2,018.8)
Middle SDI	Number: 1990	3,608,743 (3,540,237–3,683,186)	575,921 (543,773–609,110)	622,238 (569,232–675,618)	240,833 (219,106–265,021)	317,411 (281,156–347,086)	219,456 (166,488–285,691)	12,876 (10,027–16,515)	62,606 (45,982–76,729)	84,860 (63,928–102,564)	87,112 (70,977–107,110)	1,502 (1,425–1,590)
Middle SDI	Number: 2015	1,191,374 (1,155,514–1,227,971)	178,438 (164,777–194,477)	108,851 (100,741–117,441)	133,759 (123,079–145,797)	49,594 (44,578–55,256)	132,103 (115,720–145,432)	6,396 (4,992–8,097)	44,875 (32,922–54,463)	23,862 (19,668–27,831)	36,854 (31,258–43,092)	21,159 (19,597–23,345)
Middle SDI	Number: Cumulative change (%)	-67.0 (-68.2 to -65.8)	-69.0 (-71.5 to -66.3)	-82.5 (-84.3 to -80.3)	-44.3 (-49.9 to -37.5)	-84.3 (-86.3 to -82.1)	-37.8 (-57.5 to -18.1)	-49.2 (-64.9 to -31.2)	-27.8 (-39.2 to -14.3)	-71.5 (-76.7 to -64.8)	-57.1 (-66.9 to -46.8)	1,309.8 (1,186.6–1,462.4)
Middle SDI	Rate: 1990	473.5 (464.8–483.3)	73.6 (69.5–77.9)	80.8 (73.9–87.7)	30.8 (28.0–33.9)	41.5 (36.7–45.4)	28.5 (21.6–37.0)	1.7 (1.4–2.2)	8.0 (5.9–9.8)	11.2 (8.5–13.5)	11.2 (9.1–13.7)	0.2 (0.2–0.2)
Middle SDI	Rate: 2015	174.8 (169.5–180.2)	26.2 (24.2–28.6)	16.0 (14.8–17.2)	19.7 (18.1–21.4)	7.3 (6.5–8.1)	19.4 (17.0–21.4)	0.9 (0.7–1.2)	6.6 (4.8–8.0)	3.5 (2.9–4.1)	5.4 (4.6–6.3)	3.1 (2.9–3.4)
Middle SDI	Rate: Cumulative change (%)	-63.1 (-64.4 to -61.8)	-64.3 (-67.2 to -61.2)	-80.2 (-82.3 to -77.7)	-35.9 (-42.4 to -28.2)	-82.4 (-84.7 to -79.9)	-29.6 (-51.8 to -7.2)	-44.8 (-61.8 to -25.4)	-17.2 (-30.2 to -1.6)	-68.3 (-74.0 to -61.0)	-50.8 (-62.0 to -39.0)	1,463.3 (1,327.6–1,632.0)
Low-middle SDI	Number: 1990	6,148,482 (6,098,450–6,198,338)	765,273 (687,681–838,028)	1,023,454 (920,418–1,110,183)	459,777 (387,377–528,140)	784,970 (694,291–867,787)	186,302 (131,330–297,666)	366,926 (271,686–465,469)	149,124 (103,802–204,753)	156,226 (129,857–182,216)	137,559 (81,589–202,262)	17,592 (15,787–20,019)
Low-middle SDI	Number: 2015	3,418,022 (3,320,918–3,518,248)	410,824 (367,295–458,314)	382,444 (352,694–414,482)	432,718 (386,238–481,556)	322,586 (287,528–357,623)	193,453 (170,784–218,904)	252,862 (188,945–316,852)	173,049 (126,369–221,638)	99,627 (83,715–125,111)	99,004 (74,395–129,011)	91,953 (86,892–97,123)
Low-middle SDI	Number: Cumulative change (%)	-44.4 (-46.0 to -42.7)	-46.2 (-52.2 to -39.7)	-62.5 (-66.5 to -58.5)	-5.2 (-18.6 to 12.1)	-58.7 (-64.7 to -51.8)	9.0 (-27.6 to 33.7)	-30.0 (-48.5 to -8.2)	19.3 (-16.1 to 62.2)	-35.6 (-48.7 to -16.1)	-23.4 (-52.2 to 14.7)	425.0 (363.4–481.9)
Low-middle SDI	Rate: 1990	934.4 (926.7–942.3)	107.7 (96.7–118.0)	153.8 (137.9–167.0)	64.6 (54.5–74.2)	120.3 (106.4–132.9)	27.4 (19.3–43.9)	57.3 (42.8–72.6)	21.0 (14.7–28.9)	24.2 (20.2–28.1)	19.5 (11.7–28.7)	2.7 (2.4–3.1)
Low-middle SDI	Rate: 2015	425.4 (413.5–437.8)	50.0 (44.7–55.8)	47.5 (43.8–51.5)	52.6 (47.0–58.6)	40.2 (35.8–44.6)	23.8 (21.1–27.0)	31.7 (23.7–39.7)	21.1 (15.4–27.0)	12.5 (10.5–15.6)	12.1 (9.1–15.7)	11.7 (11.1–12.4)
Low-middle SDI	Rate: Cumulative change (%)	-54.5 (-55.8 to -53.1)	-53.5 (-58.7 to -47.8)	-69.0 (-72.3 to -65.7)	-17.9 (-29.5 to -3.0)	-66.4 (-71.3 to -60.9)	-8.6 (-39.6 to 12.3)	-43.9 (-58.5 to -26.9)	2.9 (-27.6 to 39.8)	-48.0 (-58.6 to -32.3)	-34.3 (-58.9 to -1.5)	335.6 (284.4–382.7)
Low SDI	Number: 1990	2,457,431 (2,430,603–2,485,072)	123,952 (104,152–145,779)	328,981 (281,373–377,974)	99,603 (77,284–120,973)	322,951 (266,111–379,227)	77,154 (52,917–130,168)	410,936 (303,421–528,235)	87,218 (64,660–117,569)	98,394 (73,880–122,856)	62,494 (40,922–88,562)	18,035 (16,141–20,307)
Low SDI	Number: 2015	1,996,606 (1,932,206–2,067,138)	126,934 (111,052–144,139)	253,251 (229,901–278,154)	118,676 (99,577–140,545)	185,251 (165,894–204,634)	113,896 (95,537–143,284)	274,248 (211,890–340,624)	111,929 (88,170–142,382)	88,928 (71,885–107,526)	57,449 (37,636–77,990)	67,860 (64,014–72,088)
Low SDI	Number: Cumulative change (%)	-18.7 (-21.7 to -15.8)	3.2 (-12.6 to 24.3)	-22.4 (-34.4 to -9.0)	20.4 (-0.2 to 46.5)	-42.0 (-51.8 to -29.1)	56.1 (9.4–100.1)	-32.1 (-48.1 to -12.0)	30.4 (0.4–67.7)	-7.8 (-28.8 to 21.8)	-5.0 (-36.4 to 36.3)	277.8 (240.2–316.8)
Low SDI	Rate: 1990	1,297.4 (1,281.5–1,315.2)	56.3 (47.3–66.2)	168.1 (143.4–193.7)	45.2 (35.2–54.9)	170.3 (140.1–200.2)	38.1 (25.9–64.9)	224.0 (166.6–286.1)	39.8 (29.5–53.5)	52.6 (39.7–65.4)	28.6 (18.8–40.4)	9.6 (8.6–10.9)
Low SDI	Rate: 2015	581.6 (563.0–601.9)	34.2 (29.9–38.9)	72.3 (65.6–79.4)	32.0 (26.8–37.9)	53.7 (48.1–59.1)	32.0 (26.9–40.2)	80.4 (62.1–99.6)	30.2 (23.8–38.4)	26.2 (21.3–31.5)	15.5 (10.2–21.0)	22.1 (20.8–23.5)
Low SDI	Rate: Cumulative change (%)	-55.2 (-56.8 to -53.5)	-38.8 (-48.1 to -26.3)	-56.7 (-63.5 to -49.0)	-28.6 (-40.8 to -13.2)	-68.2 (-73.5 to -61.1)	-10.9 (-38.2 to 14.9)	-63.5 (-71.8 to -53.5)	-22.9 (-40.5 to -1.0)	-49.3 (-60.7 to -33.1)	-43.9 (-62.4 to -19.5)	129.8 (107.5–153.8)
High-income	Number: 1990	210,272 (208,409–212,611)	33,329 (32,138–35,689)	8,740 (8,393–9,146)	10,301 (9,815–11,041)	1,717 (1,602–1,844)	36,105 (29,607–37,779)	8 (6–11)	3,725 (2,658–5,020)	3,065 (2,957–3,192)	6,281 (5,942–6,731)	832 (822–842)
High-income	Number: 2015	95,366 (94,489–96,266)	12,312 (11,195–12,959)	2,694 (2,558–2,842)	3,866 (3,530–4,163)	749 (702–799)	18,926 (17,746–21,436)	1 (1–1)	2,111 (1,705–3,239)	729 (696–767)	4,709 (4,238–5,062)	249 (244–254)
High-income	Number: Cumulative change (%)	-54.6 (-55.3 to -54.0)	-63.0 (-68.2 to -60.5)	-69.1 (-71.3 to -67.1)	-62.4 (-67.2 to -58.9)	-56.3 (-60.9 to -52.1)	-47.2 (-52.7 to -27.2)	-85.0 (-89.6 to -80.4)	-43.1 (-52.2 to -26.7)	-76.2 (-77.6 to -74.7)	-24.9 (-34.6 to -17.3)	-70.0 (-70.7 to -69.3)
High-income	Rate: 1990	87.8 (86.9–88.8)	14.8 (14.3–15.9)	3.8 (3.6–3.9)	4.6 (4.4–4.9)	0.7 (0.7–0.8)	15.8 (13.0–16.5)	0.0 (0.0–0.0)	1.7 (1.2–2.2)	1.3 (1.3–1.4)	2.8 (2.6–3.0)	0.3 (0.3–0.4)
High-income	Rate: 2015	41.3 (40.9–41.7)	5.7 (5.2–6.0)	1.2 (1.1–1.3)	1.8 (1.6–1.9)	0.3 (0.3–0.4)	8.6 (8.0–9.7)	0.0 (0.0–0.0)	1.0 (0.8–1.5)	0.3 (0.3–0.3)	2.2 (2.0–2.3)	0.1 (0.1–0.1)
High-income	Rate: Cumulative change (%)	-53.0 (-53.6 to -52.3)	-61.6 (-66.9 to -59.0)	-68.4 (-70.7 to -66.2)	-61.0 (-66.0 to -57.3)	-54.9 (-59.6 to -50.5)	-45.3 (-51.1 to -24.7)	-84.6 (-89.5 to -79.7)	-41.0 (-50.3 to -23.9)	-75.5 (-76.9 to -73.9)	-22.0 (-32.1 to -14.1)	-69.1 (-69.9 to -68.4)
High-income North America	Number: 1990	74,124 (73,922–74,327)	12,644 (12,248–13,190)	1,903 (1,798–2,015)	3,113 (2,956–3,310)	250 (231–270)	11,613 (10,173–12,056)	0 (0–2)	963 (682–1,341)	891 (844–938)	3,264 (3,083–3,485)	487 (480–494)
High-income North America	Number: 2015	42,322 (42,078–42,549)	5,914 (5,442–6,243)	826 (774–880)	1,683 (1,552–1,813)	328 (298–359)	7,144 (6,640–8,375)	0 (0–0)	730 (562–1,105)	252 (237–268)	2,382 (2,147–2,579)	95 (94–97)
High-income North America	Number: Cumulative change (%)	-42.9 (-43.3 to -42.6)	-53.2 (-58.8 to -50.1)	-56.5 (-60.3 to -52.6)	-45.8 (-52.4 to -40.9)	31.3 (15.0–48.3)	-38.2 (-44.3 to -16.9)	-100.0 (-100.0 to -100.0)	-24.1 (-33.8 to -10.3)	-71.7 (-73.9 to -69.1)	-26.9 (-35.6 to -18.8)	-80.3 (-80.7 to -79.9)
High-income North America	Rate: 1990	91.8 (91.6–92.1)	15.7 (15.2–16.4)	2.4 (2.2–2.5)	3.9 (3.7–4.1)	0.3 (0.3–0.3)	14.4 (12.7–15.0)	0.0 (0.0–0.0)	1.2 (0.8–1.7)	1.1 (1.1–1.2)	4.1 (3.8–4.3)	0.6 (0.6–0.6)
High-income North America	Rate: 2015	48.5 (48.2–48.8)	7.3 (6.7–7.7)	1.0 (0.9–1.0)	2.1 (1.9–2.2)	0.4 (0.4–0.4)	8.6 (8.0–10.1)	0.0 (0.0–0.0)	0.9 (0.7–1.4)	0.3 (0.3–0.3)	2.9 (2.6–3.2)	0.1 (0.1–0.1)
High-income North America	Rate: Cumulative change (%)	-47.2 (-47.5 to -46.8)	-53.7 (-59.2 to -50.6)	-59.1 (-62.7 to -55.4)	-46.4 (-52.9 to -41.5)	28.0 (11.9–44.6)	-40.0 (-46.0 to -19.3)	-100.0 (-100.0 to -100.0)	-25.0 (-34.5 to -11.3)	-73.4 (-75.5 to -70.9)	-27.7 (-36.3 to -19.7)	-82.1 (-82.5 to -81.7)
Canada	Number: 1990	5,302 (5,244–5,354)	726 (657–818)	123 (111–136)	285 (244–328)	7 (6–9)	1,068 (813–1,163)	0 (0–0)	47 (32–65)	59 (53–65)	144 (119–172)	13 (12–13)
Canada	Number: 2015	3,127 (3,021–3,241)	397 (349–448)	61 (53–69)	195 (163–231)	17 (14–19)	565 (508–632)	0 (0–0)	40 (25–55)	24 (21–27)	208 (173–248)	4 (4–5)
Canada	Number: Cumulative change (%)	-41.0 (-43.2 to -38.8)	-45.0 (-54.6 to -36.4)	-50.2 (-57.8 to -41.4)	-30.8 (-45.2 to -15.1)	125.8 (72.9–185.8)	-46.4 (-54.3 to -24.1)	nan (nan–nan)	-14.7 (-33.8 to 7.4)	-59.0 (-64.6 to -53.0)	46.3 (10.8–86.8)	-63.6 (-66.4 to -60.7)
Canada	Rate: 1990	71.4 (70.6–72.1)	10.2 (9.2–11.5)	1.7 (1.5–1.9)	4.0 (3.4–4.6)	0.1 (0.1–0.1)	14.9 (11.3–16.2)	0.0 (0.0–0.0)	0.7 (0.5–0.9)	0.8 (0.7–0.9)	2.0 (1.7–2.4)	0.2 (0.2–0.2)
Canada	Rate: 2015	40.6 (39.2–42.2)	5.6 (4.9–6.3)	0.8 (0.7–0.9)	2.8 (2.3–3.3)	0.2 (0.2–0.3)	7.8 (7.0–8.8)	0.0 (0.0–0.0)	0.6 (0.4–0.8)	0.3 (0.3–0.4)	2.9 (2.4–3.5)	0.1 (0.1–0.1)
Canada	Rate: Cumulative change (%)	-43.1 (-45.3 to -40.8)	-45.1 (-54.6 to -36.4)	-51.0 (-58.5 to -42.2)	-30.8 (-45.3 to -15.1)	123.2 (70.6–182.7)	-46.8 (-54.6 to -24.6)	nan (nan–nan)	-14.8 (-33.8 to 7.3)	-59.5 (-65.1 to -53.4)	46.2 (10.6–86.7)	-64.5 (-67.2 to -61.7)
Greenland	Number: 1990	99 (93–104)	27 (23–31)	5 (3–6)	9 (7–11)	0 (0–0)	13 (6–17)	0 (0–0)	4 (2–7)	2 (1–3)	7 (4–10)	0 (0–0)
Greenland	Number: 2015	29 (27–32)	7 (5–8)	0 (0–1)	2 (2–3)	0 (0–0)	4 (3–5)	0 (0–0)	1 (0–2)	0 (0–0)	1 (1–2)	0 (0–0)
Greenland	Number: Cumulative change (%)	-70.0 (-72.6 to -67.2)	-74.4 (-79.4 to -68.9)	-82.1 (-87.6 to -75.4)	-66.8 (-75.7 to -55.4)	-25.8 (-63.2 to 27.5)	-66.4 (-76.4 to -47.0)	nan (nan–nan)	-64.8 (-85.0 to -42.5)	-82.2 (-88.1 to -75.1)	-71.4 (-82.5 to -59.2)	inf (-98.3 to -79.2)
Greenland	Rate: 1990	268.6 (256.8–280.4)	61.1 (52.7–70.8)	11.9 (8.5–15.6)	20.2 (16.0–24.9)	0.5 (0.3–0.8)	31.2 (16.8–40.5)	0.0 (0.0–0.0)	9.4 (4.9–15.6)	5.6 (4.0–7.6)	15.9 (11.1–24.5)	0.5 (0.2–1.0)
Greenland	Rate: 2015	120.3 (112.6–128.7)	24.0 (19.9–28.5)	3.1 (2.4–3.8)	10.0 (7.9–12.5)	0.5 (0.3–0.7)	14.8 (11.9–17.7)	0.0 (0.0–0.0)	4.6 (2.5–7.7)	1.4 (1.1–1.7)	6.6 (5.0–8.7)	0.1 (0.0–0.2)
Greenland	Rate: Cumulative change (%)	-55.2 (-58.6 to -51.5)	-60.5 (-68.3 to -52.0)	-73.5 (-81.5 to -63.7)	-49.4 (-63.0 to -32.3)	9.2 (-45.2 to 87.1)	-49.8 (-64.5 to -20.7)	nan (nan–nan)	-46.1 (-77.1 to -12.2)	-74.3 (-82.9 to -64.5)	-56.2 (-73.3 to -37.4)	inf (-96.4 to -69.9)
United States	Number: 1990	68,688 (68,489–68,893)	11,884 (11,499–12,374)	1,773 (1,669–1,884)	2,817 (2,663–3,001)	242 (224–262)	10,526 (9,333–10,924)	0 (0–2)	911 (646–1,271)	829 (784–875)	3,111 (2,930–3,325)	474 (466–481)
United States	Number: 2015	39,151 (38,944–39,359)	5,508 (5,046–5,823)	764 (713–818)	1,484 (1,363–1,612)	310 (281–341)	6,572 (6,088–7,743)	0 (0–0)	688 (532–1,049)	227 (213–243)	2,170 (1,954–2,359)	91 (89–92)
United States	Number: Cumulative change (%)	-43.0 (-43.3 to -42.7)	-53.6 (-59.1 to -50.6)	-56.8 (-60.8 to -52.8)	-47.2 (-53.7 to -42.0)	28.5 (11.8–45.6)	-37.3 (-43.9 to -16.3)	-100.0 (-100.0 to -100.0)	-24.4 (-34.6 to -9.8)	-72.5 (-74.8 to -69.9)	-30.1 (-38.6 to -22.4)	-80.8 (-81.2 to -80.4)
United States	Rate: 1990	93.8 (93.5–94.1)	16.2 (15.7–16.9)	2.4 (2.3–2.6)	3.9 (3.6–4.1)	0.3 (0.3–0.4)	14.4 (12.8–14.9)	0.0 (0.0–0.0)	1.2 (0.9–1.7)	1.1 (1.1–1.2)	4.3 (4.0–4.5)	0.7 (0.6–0.7)
United States	Rate: 2015	49.3 (49.0–49.6)	7.4 (6.8–7.9)	1.0 (0.9–1.0)	2.0 (1.8–2.2)	0.4 (0.4–0.5)	8.7 (8.1–10.3)	0.0 (0.0–0.0)	0.9 (0.7–1.4)	0.3 (0.3–0.3)	2.9 (2.6–3.2)	0.1 (0.1–0.1)
United States	Rate: Cumulative change (%)	-47.5 (-47.8 to -47.1)	-54.2 (-59.6 to -51.2)	-59.6 (-63.4 to -55.7)	-47.9 (-54.3 to -42.7)	25.1 (8.8–41.9)	-39.2 (-45.7 to -19.0)	-100.0 (-100.0 to -100.0)	-25.3 (-35.4 to -10.9)	-74.3 (-76.4 to -71.8)	-31.0 (-39.3 to -23.4)	-82.6 (-83.0 to -82.2)
Australasia	Number: 1990	4,856 (4,799–4,912)	658 (605–719)	120 (110–130)	277 (247–313)	10 (9–11)	829 (703–893)	0 (0–0)	60 (45–93)	57 (52–62)	77 (66–89)	7 (7–7)
Australasia	Number: 2015	2,582 (2,521–2,647)	205 (172–237)	55 (49–62)	150 (123–176)	16 (13–19)	513 (453–608)	0 (0–0)	36 (22–70)	18 (17–20)	141 (116–166)	2 (2–3)
Australasia	Number: Cumulative change (%)	-46.8 (-48.3 to -45.3)	-68.7 (-75.4 to -63.1)	-53.7 (-59.6 to -46.7)	-45.2 (-56.8 to -34.0)	55.0 (28.7–87.4)	-37.6 (-47.4 to -12.9)	nan (nan–nan)	-41.1 (-60.2 to -12.9)	-67.2 (-70.7 to -63.3)	84.1 (39.6–133.1)	-62.4 (-65.9 to -58.5)
Australasia	Rate: 1990	80.1 (79.1–81.1)	11.5 (10.6–12.5)	2.1 (1.9–2.2)	4.8 (4.3–5.5)	0.2 (0.2–0.2)	14.3 (12.1–15.4)	0.0 (0.0–0.0)	1.1 (0.8–1.6)	1.0 (0.9–1.1)	1.3 (1.2–1.6)	0.1 (0.1–0.1)
Australasia	Rate: 2015	35.4 (34.5–36.4)	3.0 (2.5–3.4)	0.8 (0.7–0.9)	2.2 (1.8–2.5)	0.2 (0.2–0.3)	7.3 (6.5–8.7)	0.0 (0.0–0.0)	0.5 (0.3–1.0)	0.3 (0.2–0.3)	2.0 (1.7–2.4)	0.0 (0.0–0.0)
Australasia	Rate: Cumulative change (%)	-55.8 (-57.0 to -54.5)	-74.1 (-79.7 to -69.5)	-61.8 (-66.7 to -56.0)	-54.8 (-64.4 to -45.5)	27.3 (5.7–53.9)	-48.4 (-56.6 to -28.0)	nan (nan–nan)	-51.4 (-67.2 to -28.1)	-72.9 (-75.9 to -69.7)	52.0 (15.2–92.5)	-67.9 (-70.8 to -64.6)
Australia	Number: 1990	3,820 (3,766–3,872)	554 (502–613)	80 (72–89)	230 (201–265)	7 (6–9)	659 (541–718)	0 (0–0)	56 (41–88)	44 (40–49)	69 (57–81)	6 (5–6)
Australia	Number: 2015	2,023 (1,966–2,086)	152 (123–182)	42 (36–48)	135 (109–160)	12 (10–15)	412 (360–479)	0 (0–0)	30 (16–66)	12 (11–14)	124 (99–148)	2 (2–2)
Australia	Number: Cumulative change (%)	-47.0 (-48.8 to -45.2)	-72.4 (-78.7 to -66.3)	-47.3 (-56.2 to -37.1)	-40.8 (-54.7 to -27.3)	60.0 (25.2–101.7)	-36.8 (-47.9 to -10.7)	nan (nan–nan)	-46.9 (-68.7 to -13.2)	-71.5 (-75.4 to -67.1)	82.6 (34.7–136.1)	-59.4 (-63.6 to -54.6)
Australia	Rate: 1990	76.9 (75.8–78.0)	11.8 (10.7–13.1)	1.7 (1.5–1.9)	4.9 (4.3–5.7)	0.2 (0.1–0.2)	13.9 (11.4–15.2)	0.0 (0.0–0.0)	1.2 (0.9–1.9)	0.9 (0.8–1.0)	1.5 (1.2–1.7)	0.1 (0.1–0.1)
Australia	Rate: 2015	33.3 (32.3–34.4)	2.6 (2.1–3.1)	0.7 (0.6–0.8)	2.3 (1.9–2.7)	0.2 (0.2–0.3)	7.0 (6.1–8.2)	0.0 (0.0–0.0)	0.5 (0.3–1.1)	0.2 (0.2–0.2)	2.1 (1.7–2.5)	0.0 (0.0–0.0)
Australia	Rate: Cumulative change (%)	-56.7 (-58.2 to -55.1)	-77.9 (-83.0 to -73.1)	-57.5 (-64.6 to -49.4)	-52.6 (-63.7 to -41.7)	28.2 (0.6–61.6)	-49.2 (-58.1 to -28.0)	nan (nan–nan)	-57.5 (-74.9 to -30.5)	-76.9 (-80.1 to -73.2)	46.2 (7.8–89.1)	-65.7 (-69.2 to -61.6)
New Zealand	Number: 1990	1,036 (1,020–1,053)	104 (94–115)	40 (36–45)	46 (40–53)	2 (2–3)	170 (156–183)	0 (0–0)	4 (2–5)	13 (11–14)	8 (6–10)	1 (1–1)
New Zealand	Number: 2015	558 (540–578)	53 (44–61)	13 (11–16)	15 (12–18)	3 (3–4)	101 (89–127)	0 (0–0)	5 (1–7)	6 (5–6)	16 (13–20)	0 (0–0)
New Zealand	Number: Cumulative change (%)	-46.1 (-48.1 to -44.0)	-48.6 (-58.1 to -40.0)	-65.9 (-72.4 to -58.9)	-66.5 (-74.3 to -57.9)	42.8 (11.1–78.9)	-40.2 (-49.0 to -20.2)	nan (nan–nan)	23.8 (-27.2 to 77.2)	-52.4 (-58.9 to -44.5)	101.1 (49.5–163.4)	-74.6 (-77.4 to -71.7)
New Zealand	Rate: 1990	95.1 (93.5–96.7)	9.9 (8.9–10.9)	3.8 (3.4–4.3)	4.4 (3.8–5.1)	0.3 (0.2–0.3)	16.1 (14.9–17.3)	0.0 (0.0–0.0)	0.4 (0.2–0.6)	1.2 (1.1–1.4)	0.8 (0.6–1.0)	0.1 (0.1–0.1)
New Zealand	Rate: 2015	45.9 (44.3–47.6)	4.8 (4.1–5.5)	1.2 (1.0–1.4)	1.4 (1.1–1.7)	0.3 (0.3–0.4)	9.0 (7.9–11.3)	0.0 (0.0–0.0)	0.5 (0.2–0.7)	0.5 (0.5–0.6)	1.5 (1.2–1.8)	0.0 (0.0–0.0)
New Zealand	Rate: Cumulative change (%)	-51.7 (-53.6 to -49.7)	-50.8 (-60.0 to -42.7)	-68.4 (-74.4 to -61.7)	-68.0 (-75.5 to -59.8)	32.5 (3.1–66.3)	-44.2 (-52.4 to -25.3)	nan (nan–nan)	18.4 (-30.3 to 69.7)	-56.7 (-62.7 to -49.3)	92.0 (42.6–151.4)	-77.4 (-79.9 to -74.9)
High-income Asia Pacific	Number: 1990	30,483 (28,681–32,746)	2,632 (2,234–3,277)	1,550 (1,400–1,727)	886 (775–1,048)	213 (185–247)	5,776 (3,930–6,666)	5 (3–7)	489 (347–783)	373 (334–416)	635 (551–759)	29 (28–30)
High-income Asia Pacific	Number: 2015	8,211 (7,986–8,460)	519 (403–609)	325 (298–346)	205 (174–232)	54 (49–59)	1,718 (1,589–2,035)	0 (0–0)	172 (116–298)	39 (36–41)	303 (246–347)	14 (14–14)
High-income Asia Pacific	Number: Cumulative change (%)	-73.0 (-75.0 to -71.1)	-79.9 (-86.5 to -74.7)	-78.9 (-82.0 to -76.3)	-76.6 (-82.7 to -71.6)	-74.3 (-78.6 to -69.6)	-69.4 (-75.1 to -48.2)	-94.0 (-96.3 to -91.5)	-64.4 (-75.3 to -46.8)	-89.5 (-90.8 to -88.2)	-51.8 (-64.6 to -41.6)	-51.0 (-52.6 to -49.6)
High-income Asia Pacific	Rate: 1990	69.1 (64.4–75.1)	7.1 (6.0–8.9)	3.7 (3.3–4.2)	2.4 (2.1–2.8)	0.5 (0.5–0.6)	15.0 (10.2–17.4)	0.0 (0.0–0.0)	1.3 (0.9–2.1)	0.9 (0.8–1.0)	1.7 (1.5–2.0)	0.1 (0.1–0.1)
High-income Asia Pacific	Rate: 2015	26.0 (25.2–26.8)	1.8 (1.4–2.1)	1.1 (1.0–1.1)	0.7 (0.6–0.8)	0.2 (0.2–0.2)	5.9 (5.5–7.0)	0.0 (0.0–0.0)	0.6 (0.4–1.1)	0.1 (0.1–0.1)	1.1 (0.9–1.2)	0.0 (0.0–0.0)
High-income Asia Pacific	Rate: Cumulative change (%)	-62.4 (-65.5 to -59.4)	-73.8 (-82.3 to -66.9)	-71.5 (-75.8 to -67.7)	-69.4 (-77.4 to -62.9)	-65.2 (-71.4 to -58.6)	-59.5 (-67.1 to -31.3)	-91.8 (-95.2 to -88.1)	-53.5 (-67.7 to -30.4)	-85.5 (-87.4 to -83.5)	-37.0 (-53.7 to -23.6)	-31.2 (-33.5 to -29.0)
Brunei	Number: 1990	111 (106–116)	11 (8–14)	8 (7–9)	3 (1–5)	0 (0–1)	23 (19–26)	0 (0–0)	2 (0–3)	1 (1–2)	3 (1–6)	0 (0–0)
Brunei	Number: 2015	91 (84–99)	8 (6–10)	6 (5–7)	2 (1–3)	0 (0–0)	17 (14–20)	0 (0–0)	2 (1–3)	0 (0–1)	3 (2–5)	0 (0–0)
Brunei	Number: Cumulative change (%)	-17.6 (-25.3 to -9.5)	-23.0 (-48.8 to 6.7)	-24.9 (-39.8 to -8.7)	-6.6 (-53.2 to 63.3)	-39.7 (-58.5 to -17.3)	-24.3 (-35.7 to -11.2)	nan (nan–nan)	31.4 (-38.5 to 145.5)	-45.6 (-58.2 to -28.2)	7.7 (-51.2 to 103.2)	inf (-27.5 to inf)
Brunei	Rate: 1990	85.5 (81.9–89.4)	7.4 (5.5–9.5)	6.0 (5.2–7.0)	2.2 (1.3–3.5)	0.6 (0.5–0.7)	16.1 (13.5–18.2)	0.0 (0.0–0.0)	1.4 (0.6–2.5)	1.3 (1.1–1.5)	2.6 (1.3–4.3)	0.1 (0.0–0.1)
Brunei	Rate: 2015	70.7 (65.0–76.6)	6.8 (5.1–8.5)	4.9 (4.2–5.7)	2.3 (1.5–3.3)	0.4 (0.3–0.5)	14.1 (11.6–16.4)	0.0 (0.0–0.0)	2.0 (1.2–3.1)	0.7 (0.6–0.9)	3.0 (1.9–4.4)	0.1 (0.0–0.1)

© 2017 Global Burden of Disease Child and Adolescent Health Collaboration. JAMA Pediatrics.

eTable 1. Number of Deaths, Death Rates (per 100,000 population), and Cumulative Percent Change with 95% Uncertainty Intervals (UI) for the Top 10 Global Causes of Death in 195 Countries and Territories, Aged 0 to 19, Both Sexes, 1990 and 2015 *Note: best viewed by enlarging in browser.*

Location	Measure	All causes	Neonatal preterm birth complications	Lower respiratory infections	Neonatal encephalopathy due to birth asphyxia and trauma	Diarrheal diseases	Congenital anomalies	Malaria	Neonatal sepsis and other neonatal infections	Meningitis	Other neonatal disorders	HIV/AIDS
Brunei	Rate: Cumulative change (%)	-17.3 (-25.0 to -9.2)	-5.9 (-37.5 to 30.4)	-17.8 (-34.2 to -0.5)	14.0 (-42.9 to 99.1)	-30.2 (-51.8 to -4.1)	-11.9 (-25.2 to 3.2)	nan (nan–nan)	60.4 (-24.9 to 199.5)	-42.2 (-55.7 to -23.2)	31.4 (-40.5 to 148.2)	inf (-23.6 to inf)
Japan	Number: 1990	14,867 (14,776–14,957)	1,079 (1,023–1,159)	620 (593–654)	522 (489–567)	56 (52–62)	3,135 (2,772–3,250)	2 (0–4)	159 (104–197)	128 (122–135)	365 (339–393)	13 (13–13)
Japan	Number: 2015	5,126 (5,057–5,192)	227 (186–253)	242 (219–258)	136 (115–152)	41 (37–45)	1,263 (1,180–1,521)	0 (0–0)	62 (48–97)	27 (25–29)	183 (152–205)	9 (9–9)
Japan	Number: Cumulative change (%)	-65.5 (-66.0 to -65.0)	-78.9 (-83.6 to -76.1)	-60.9 (-66.1 to -57.6)	-73.9 (-79.2 to -70.4)	-26.0 (-40.3 to -16.8)	-59.5 (-62.8 to -45.0)	-100.0 (-100.0 to -100.0)	-60.0 (-69.0 to -42.3)	-78.6 (-81.1 to -76.7)	-49.6 (-60.8 to -42.2)	-27.5 (-28.5 to -26.4)
Japan	Rate: 1990	51.2 (50.8–51.5)	4.6 (4.3–4.9)	2.3 (2.2–2.4)	2.2 (2.1–2.4)	0.2 (0.2–0.2)	12.6 (11.1–13.0)	0.0 (0.0–0.0)	0.7 (0.4–0.8)	0.5 (0.5–0.5)	1.5 (1.4–1.7)	0.0 (0.0–0.0)
Japan	Rate: 2015	24.6 (24.3–25.0)	1.2 (1.0–1.3)	1.2 (1.1–1.3)	0.7 (0.6–0.8)	0.2 (0.2–0.2)	6.5 (6.0–7.7)	0.0 (0.0–0.0)	0.3 (0.3–0.5)	0.1 (0.1–0.1)	1.0 (0.8–1.1)	0.0 (0.0–0.0)
Japan	Rate: Cumulative change (%)	-51.9 (-52.6 to -51.2)	-73.8 (-79.7 to -70.4)	-48.0 (-55.3 to -43.5)	-67.7 (-74.3 to -63.3)	-4.2 (-22.0 to 8.1)	-48.4 (-52.5 to -30.2)	-100.0 (-100.0 to -100.0)	-50.4 (-61.6 to -28.5)	-71.6 (-75.1 to -69.1)	-37.5 (-51.4 to -28.4)	1.7 (0.2–3.3)
Singapore	Number: 1990	578 (562–594)	75 (67–90)	63 (58–71)	25 (21–31)	7 (6–8)	155 (109–169)	0 (0–0)	2 (1–3)	7 (6–8)	10 (8–13)	0 (0–0)
Singapore	Number: 2015	169 (161–177)	10 (8–12)	19 (16–21)	3 (2–4)	0 (0–1)	33 (29–44)	0 (0–0)	1 (0–2)	0 (0–0)	8 (6–10)	0 (0–0)
Singapore	Number: Cumulative change (%)	-70.8 (-72.4 to -69.1)	-86.1 (-90.4 to -83.0)	-69.5 (-76.4 to -65.1)	-86.0 (-90.5 to -81.7)	-86.5 (-90.0 to -83.2)	-77.6 (-81.9 to -59.5)	nan (nan–nan)	-35.0 (-66.0 to -7.1)	-89.8 (-91.8 to -88.1)	-18.4 (-41.7 to 9.6)	-39.9 (-43.8 to -35.8)
Singapore	Rate: 1990	64.4 (62.6–66.1)	8.1 (7.2–9.6)	7.2 (6.6–8.1)	2.7 (2.3–3.4)	0.8 (0.7–0.9)	17.0 (12.0–18.6)	0.0 (0.0–0.0)	0.3 (0.2–0.4)	0.8 (0.7–0.9)	1.1 (0.9–1.4)	0.0 (0.0–0.1)
Singapore	Rate: 2015	20.4 (19.4–21.6)	1.5 (1.2–1.8)	2.4 (2.1–2.7)	0.5 (0.4–0.6)	0.1 (0.1–0.2)	4.6 (4.0–6.1)	0.0 (0.0–0.0)	0.2 (0.1–0.3)	0.1 (0.1–0.1)	1.2 (1.0–1.5)	0.0 (0.0–0.0)
Singapore	Rate: Cumulative change (%)	-68.3 (-70.2 to -66.2)	-81.3 (-87.1 to -77.1)	-66.1 (-74.0 to -61.2)	-81.2 (-87.2 to -75.5)	-83.8 (-88.1 to -79.6)	-72.2 (-77.7 to -49.3)	nan (nan–nan)	-12.7 (-54.3 to 24.7)	-88.4 (-90.8 to -86.4)	9.5 (-21.7 to 47.2)	-34.8 (-39.2 to -30.3)
South Korea	Number: 1990	14,925 (13,130–17,165)	1,466 (1,075–2,069)	857 (718–1,028)	335 (237–477)	149 (121–180)	2,463 (1,023–3,288)	3 (2–3)	324 (184–594)	236 (199–275)	255 (177–364)	15 (15–16)
South Korea	Number: 2015	2,823 (2,618–3,061)	272 (190–346)	58 (50–66)	63 (47–80)	11 (9–13)	402 (340–477)	0 (0–0)	105 (56–200)	9 (8–11)	107 (77–140)	4 (4–4)
South Korea	Number: Cumulative change (%)	-81.0 (-83.7 to -77.9)	-80.6 (-88.8 to -71.9)	-93.2 (-94.5 to -91.7)	-80.2 (-88.2 to -70.7)	-92.3 (-94.2 to -89.8)	-81.7 (-88.6 to -56.6)	-90.7 (-92.1 to -89.2)	-66.9 (-80.5 to -47.5)	-95.7 (-96.6 to -94.8)	-56.1 (-73.9 to -34.3)	-71.2 (-73.2 to -69.3)
South Korea	Rate: 1990	105.3 (91.1–123.1)	11.9 (8.7–16.8)	6.4 (5.3–7.8)	2.7 (1.9–3.9)	1.1 (0.9–1.4)	19.5 (8.1–26.1)	0.0 (0.0–0.0)	2.6 (1.5–4.8)	1.7 (1.4–2.0)	2.1 (1.4–3.0)	0.1 (0.1–0.1)
South Korea	Rate: 2015	28.8 (26.4–31.5)	3.2 (2.3–4.1)	0.6 (0.5–0.7)	0.7 (0.6–1.0)	0.1 (0.1–0.2)	4.6 (3.9–5.5)	0.0 (0.0–0.0)	1.2 (0.7–2.4)	0.1 (0.1–0.1)	1.3 (0.9–1.7)	0.0 (0.0–0.1)
South Korea	Rate: Cumulative change (%)	-72.5 (-77.0 to -67.4)	-71.7 (-83.7 to -58.9)	-90.0 (-92.2 to -87.8)	-71.0 (-82.8 to -57.1)	-88.8 (-91.7 to -85.0)	-73.3 (-83.5 to -36.5)	-86.2 (-88.4 to -84.0)	-51.6 (-71.6 to -23.4)	-93.7 (-94.9 to -92.2)	-35.8 (-61.9 to -3.9)	-57.7 (-60.6 to -54.6)
Western Europe	Number: 1990	67,742 (67,468–68,013)	10,249 (9,821–11,191)	1,934 (1,847–2,092)	4,157 (3,943–4,452)	261 (244–290)	12,813 (10,051–13,444)	0 (0–0)	1,041 (781–1,522)	1,073 (1,023–1,140)	1,225 (1,139–1,329)	212 (207–216)
Western Europe	Number: 2015	25,449 (24,722–26,242)	3,090 (2,686–3,336)	594 (558–631)	1,247 (1,092–1,385)	176 (160–192)	5,728 (5,209–6,699)	0 (0–0)	514 (383–887)	232 (219–247)	1,205 (1,030–1,358)	59 (58–60)
Western Europe	Number: Cumulative change (%)	-62.4 (-63.5 to -61.3)	-69.8 (-76.0 to -66.8)	-69.2 (-72.8 to -66.6)	-69.9 (-75.1 to -66.1)	-32.1 (-43.2 to -24.0)	-54.8 (-60.7 to -33.2)	nan (nan–nan)	-50.9 (-60.6 to -31.7)	-78.3 (-80.1 to -76.5)	-1.4 (-19.7 to 12.7)	-72.0 (-72.8 to -71.2)
Western Europe	Rate: 1990	74.7 (74.4–75.0)	12.4 (11.9–13.5)	2.2 (2.1–2.4)	5.0 (4.8–5.4)	0.3 (0.3–0.3)	15.1 (11.9–15.9)	0.0 (0.0–0.0)	1.3 (0.9–1.8)	1.2 (1.2–1.3)	1.5 (1.4–1.6)	0.2 (0.2–0.2)
Western Europe	Rate: 2015	29.6 (28.7–30.6)	3.8 (3.3–4.1)	0.7 (0.7–0.7)	1.5 (1.3–1.7)	0.2 (0.2–0.2)	6.9 (6.3–8.1)	0.0 (0.0–0.0)	0.6 (0.5–1.1)	0.3 (0.3–0.3)	1.5 (1.3–1.7)	0.1 (0.1–0.1)
Western Europe	Rate: Cumulative change (%)	-60.4 (-61.5 to -59.1)	-69.3 (-75.5 to -66.2)	-68.3 (-72.0 to -65.5)	-69.4 (-74.7 to -65.5)	-30.9 (-41.8 to -22.6)	-53.8 (-59.9 to -31.6)	nan (nan–nan)	-50.0 (-60.0 to -30.5)	-77.5 (-79.4 to -75.6)	0.4 (-18.3 to 14.7)	-70.0 (-70.8 to -69.0)
Andorra	Number: 1990	5 (5–6)	0 (0–1)	0 (0–0)	0 (0–0)	0 (0–0)	1 (1–1)	0 (0–0)	0 (0–0)	0 (0–0)	0 (0–0)	0 (0–0)
Andorra	Number: 2015	2 (2–2)	0 (0–0)	0 (0–0)	0 (0–0)	0 (0–0)	0 (0–0)	0 (0–0)	0 (0–0)	0 (0–0)	0 (0–0)	0 (0–0)
Andorra	Number: Cumulative change (%)	-54.8 (-60.0 to -48.7)	-75.9 (-85.1 to -64.8)	-67.3 (-77.1 to -54.9)	-70.5 (-84.2 to -49.5)	-48.6 (-73.6 to -12.0)	-50.7 (-63.7 to -34.3)	nan (nan–nan)	-49.6 (-81.8 to 2.1)	-69.2 (-84.2 to -49.5)	-47.0 (-84.5 to 21.2)	441.5 (-97.9 to 122.1)
Andorra	Rate: 1990	47.0 (41.7–53.1)	7.0 (5.1–9.3)	1.3 (1.0–1.7)	3.3 (2.1–4.9)	0.2 (0.1–0.2)	11.3 (9.1–13.8)	0.0 (0.0–0.0)	0.8 (0.4–1.4)	0.7 (0.5–1.1)	1.3 (0.6–2.3)	0.4 (0.0–2.1)
Andorra	Rate: 2015	17.6 (16.0–19.7)	1.5 (1.0–2.0)	0.3 (0.3–0.4)	0.8 (0.5–1.3)	0.1 (0.0–0.1)	4.7 (3.5–6.3)	0.0 (0.0–0.0)	0.3 (0.1–0.6)	0.2 (0.1–0.3)	0.5 (0.2–0.9)	0.0 (0.0–0.1)
Andorra	Rate: Cumulative change (%)	-62.4 (-67.1 to -57.1)	-78.4 (-86.6 to -68.5)	-73.6 (-81.8 to -62.8)	-73.6 (-85.9 to -54.8)	-57.1 (-78.3 to -25.5)	-57.7 (-69.1 to -43.4)	nan (nan–nan)	-54.9 (-83.7 to -8.3)	-75.1 (-87.5 to -58.5)	-52.7 (-86.3 to 8.6)	287.6 (-98.5 to 65.4)
Austria	Number: 1990	1,363 (1,330–1,399)	184 (164–216)	36 (32–41)	123 (107–141)	1 (1–2)	263 (172–293)	0 (0–0)	7 (4–10)	14 (12–15)	20 (17–25)	1 (1–2)
Austria	Number: 2015	518 (489–552)	53 (44–62)	8 (7–10)	23 (19–28)	4 (3–5)	118 (105–132)	0 (0–0)	3 (2–6)	3 (3–4)	19 (15–23)	0 (0–0)
Austria	Number: Cumulative change (%)	-61.9 (-64.2 to -59.3)	-71.0 (-76.8 to -65.0)	-75.8 (-79.9 to -71.4)	-81.1 (-85.2 to -76.2)	174.3 (111.0–248.5)	-54.2 (-61.4 to -34.4)	nan (nan–nan)	-48.9 (-64.4 to -28.3)	-75.1 (-78.8 to -71.1)	-6.1 (-30.2 to 20.1)	-58.8 (-62.7 to -54.7)
Austria	Rate: 1990	77.2 (75.1–79.3)	11.3 (10.1–13.3)	2.2 (1.9–2.4)	7.6 (6.6–8.7)	0.1 (0.1–0.1)	15.9 (10.4–17.8)	0.0 (0.0–0.0)	0.5 (0.3–0.7)	0.8 (0.7–0.9)	1.3 (1.1–1.6)	0.1 (0.1–0.1)
Austria	Rate: 2015	31.1 (29.2–33.3)	3.5 (3.0–4.1)	0.6 (0.5–0.6)	1.5 (1.3–1.9)	0.3 (0.2–0.4)	7.7 (6.8–8.6)	0.0 (0.0–0.0)	0.3 (0.2–0.4)	0.2 (0.2–0.3)	1.3 (1.0–1.6)	0.0 (0.0–0.1)
Austria	Rate: Cumulative change (%)	-59.7 (-62.3 to -56.7)	-68.7 (-74.9 to -62.2)	-74.2 (-78.6 to -69.3)	-79.6 (-84.0 to -74.3)	197.6 (127.9–278.8)	-50.9 (-58.8 to -29.2)	nan (nan–nan)	-44.8 (-61.6 to -22.5)	-73.3 (-77.4 to -69.0)	1.4 (-24.6 to 29.7)	-55.6 (-59.7 to -51.2)
Belgium	Number: 1990	1,842 (1,820–1,866)	254 (229–284)	38 (34–43)	127 (108–150)	6 (5–7)	311 (192–346)	0 (0–0)	29 (18–59)	19 (17–22)	40 (33–49)	6 (6–6)
Belgium	Number: 2015	814 (771–860)	54 (43–65)	18 (16–20)	52 (42–64)	6 (5–8)	165 (144–187)	0 (0–0)	16 (8–35)	7 (6–8)	44 (35–53)	2 (1–2)
Belgium	Number: Cumulative change (%)	-55.8 (-58.2 to -53.3)	-78.7 (-83.2 to -73.8)	-52.7 (-60.3 to -44.0)	-58.2 (-67.7 to -47.5)	6.1 (-16.7 to 33.5)	-45.6 (-55.7 to -16.8)	nan (nan–nan)	-44.8 (-60.2 to -25.6)	-61.8 (-67.6 to -55.2)	10.9 (-15.7 to 41.7)	-67.7 (-70.4 to -64.9)
Belgium	Rate: 1990	78.8 (77.8–79.8)	11.6 (10.4–13.0)	1.7 (1.5–1.9)	5.8 (5.0–6.9)	0.3 (0.3–0.4)	14.0 (8.6–15.6)	0.0 (0.0–0.0)	1.3 (0.8–2.7)	0.9 (0.8–1.0)	1.8 (1.5–2.3)	0.3 (0.3–0.3)
Belgium	Rate: 2015	32.4 (30.7–34.3)	2.3 (1.8–2.7)	0.7 (0.7–0.8)	2.2 (1.8–2.7)	0.3 (0.2–0.3)	6.8 (5.9–7.7)	0.0 (0.0–0.0)	0.7 (0.4–1.5)	0.3 (0.3–0.4)	1.8 (1.5–2.2)	0.1 (0.1–0.1)
Belgium	Rate: Cumulative change (%)	-58.9 (-61.2 to -56.4)	-80.5 (-84.6 to -76.0)	-56.3 (-63.4 to -48.2)	-61.7 (-70.4 to -51.9)	-2.6 (-23.7 to 22.7)	-50.1 (-59.4 to -23.4)	nan (nan–nan)	-49.5 (-63.6 to -31.9)	-64.6 (-70.0 to -58.5)	1.6 (-22.8 to 29.7)	-69.3 (-71.9 to -66.6)
Cyprus	Number: 1990	211 (205–218)	33 (23–43)	13 (10–16)	11 (7–17)	3 (2–4)	28 (19–36)	0 (0–0)	7 (3–12)	2 (2–3)	19 (10–30)	0 (0–0)
Cyprus	Number: 2015	60 (56–65)	5 (3–6)	1 (1–2)	2 (1–3)	0 (0–1)	9 (8–10)	0 (0–0)	1 (1–3)	0 (0–0)	4 (2–6)	0 (0–0)
Cyprus	Number: Cumulative change (%)	-71.4 (-73.4 to -69.3)	-83.9 (-89.6 to -76.0)	-85.7 (-89.5 to -81.1)	-77.7 (-88.7 to -61.7)	-70.5 (-81.1 to -57.5)	-66.1 (-76.2 to -53.3)	nan (nan–nan)	-68.0 (-87.9 to -40.6)	-83.6 (-88.2 to -77.7)	-75.6 (-87.9 to -55.3)	inf (-88.4 to -27.3)
Cyprus	Rate: 1990	96.1 (93.1–99.0)	16.0 (11.2–20.7)	6.2 (4.8–7.7)	5.7 (3.4–8.4)	1.5 (1.1–2.1)	13.4 (9.3–17.1)	0.0 (0.0–0.0)	3.3 (1.6–6.1)	1.2 (1.0–1.5)	9.4 (5.2–14.6)	0.0 (0.0–0.0)
Cyprus	Rate: 2015	38.0 (35.2–41.4)	4.2 (3.0–5.5)	1.3 (1.1–1.6)	2.0 (1.2–2.9)	0.7 (0.6–0.9)	7.1 (6.1–8.3)	0.0 (0.0–0.0)	1.6 (0.9–2.8)	0.3 (0.2–0.3)	3.5 (2.2–5.1)	0.0 (0.0–0.0)
Cyprus	Rate: Cumulative change (%)	-60.5 (-63.6 to -56.9)	-72.8 (-82.4 to -59.3)	-77.9 (-84.1 to -70.2)	-62.3 (-80.8 to -35.1)	-51.7 (-69.1 to -30.4)	-45.4 (-61.8 to -24.5)	nan (nan–nan)	-45.9 (-79.7 to 0.6)	-76.1 (-83.1 to -67.2)	-58.7 (-79.5 to -24.2)	inf (-87.0 to -3.0)
Denmark	Number: 1990	845 (822–870)	140 (123–160)	16 (14–18)	56 (47–67)	2 (2–3)	225 (184–246)	0 (0–0)	2 (1–3)	25 (22–28)	23 (19–29)	1 (1–1)
Denmark	Number: 2015	355 (327–387)	57 (44–69)	7 (5–8)	17 (12–21)	4 (3–5)	79 (66–107)	0 (0–0)	1 (1–2)	4 (3–4)	8 (6–11)	0 (0–0)
Denmark	Number: Cumulative change (%)	-58.0 (-61.5 to -54.0)	-59.0 (-71.6 to -48.9)	-55.9 (-65.4 to -45.0)	-69.1 (-78.4 to -59.4)	53.6 (13.4–91.9)	-64.1 (-71.7 to -41.0)	nan (nan–nan)	-27.5 (-51.5 to -0.9)	-82.9 (-86.0 to -79.9)	-61.9 (-74.1 to -47.6)	-68.2 (-71.0 to -65.2)
Denmark	Rate: 1990	70.4 (68.5–72.6)	12.3 (10.8–14.0)	1.4 (1.2–1.6)	4.9 (4.1–5.9)	0.2 (0.2–0.3)	19.7 (16.1–21.5)	0.0 (0.0–0.0)	0.2 (0.1–0.3)	2.2 (1.9–2.5)	2.1 (1.7–2.6)	0.1 (0.1–0.1)
Denmark	Rate: 2015	28.8 (26.4–31.8)	5.2 (4.1–6.4)	0.6 (0.5–0.7)	1.6 (1.2–2.0)	0.4 (0.3–0.5)	7.0 (5.9–9.4)	0.0 (0.0–0.0)	0.2 (0.1–0.2)	0.4 (0.3–0.4)	0.8 (0.6–1.1)	0.0 (0.0–0.0)
Denmark	Rate: Cumulative change (%)	-59.0 (-62.8 to -54.7)	-56.8 (-70.1 to -46.2)	-57.2 (-66.7 to -46.2)	-67.5 (-77.2 to -57.3)	54.5 (14.0–93.0)	-63.7 (-71.4 to -40.5)	nan (nan–nan)	-23.6 (-49.0 to 4.5)	-83.6 (-86.6 to -80.6)	-59.9 (-72.7 to -44.8)	-69.7 (-72.5 to -66.7)
Finland	Number: 1990	781 (756–807)	99 (85–115)	20 (18–22)	27 (22–34)	2 (2–2)	181 (166–197)	0 (0–0)	4 (2–6)	9 (7–10)	36 (28–44)	0 (0–0)
Finland	Number: 2015	306 (278–338)	19 (13–25)	3 (2–3)	9 (6–12)	0 (0–0)	69 (56–88)	0 (0–0)	2 (1–3)	1 (1–2)	12 (8–16)	0 (0–0)
Finland	Number: Cumulative change (%)	-60.8 (-64.6 to -56.4)	-79.8 (-87.1 to -72.9)	-85.2 (-88.3 to -81.6)	-65.6 (-77.4 to -50.9)	-69.8 (-77.5 to -60.6)	-61.8 (-69.5 to -48.9)	nan (nan–nan)	-39.2 (-64.0 to -10.4)	-80.6 (-84.4 to -76.0)	-65.6 (-77.1 to -51.6)	-67.1 (-70.1 to -64.1)
Finland	Rate: 1990	63.9 (61.8–66.1)	8.5 (7.3–9.8)	1.7 (1.5–1.9)	2.4 (1.9–2.9)	0.2 (0.2–0.2)	15.3 (14.0–16.7)	0.0 (0.0–0.0)	0.4 (0.2–0.5)	0.7 (0.7–0.8)	3.1 (2.4–3.8)	0.1 (0.1–0.1)
Finland	Rate: 2015	25.2 (22.7–28.1)	1.8 (1.3–2.4)	0.3 (0.2–0.3)	0.9 (0.6–1.2)	0.1 (0.0–0.1)	6.2 (5.0–8.0)	0.0 (0.0–0.0)	0.2 (0.1–0.3)	0.1 (0.1–0.2)	1.1 (0.8–1.5)	0.0 (0.0–0.0)
Finland	Rate: Cumulative change (%)	-60.6 (-64.8 to -55.8)	-78.1 (-86.0 to -70.6)	-84.5 (-87.8 to -80.7)	-62.6 (-75.4 to -46.5)	-67.7 (-76.0 to -57.6)	-59.1 (-67.5 to -45.1)	nan (nan–nan)	-33.9 (-60.9 to -2.5)	-79.9 (-83.9 to -74.8)	-62.6 (-75.1 to -47.4)	-64.4 (-67.6 to -61.2)
France	Number: 1990	10,727 (10,604–10,855)	964 (864–1,078)	188 (167–207)	754 (662–848)	80 (69–91)	1,733 (1,497–1,864)	0 (0–0)	259 (195–399)	140 (128–154)	175 (148–205)	44 (42–47)
France	Number: 2015	4,191 (3,714–4,766)	217 (150–289)	66 (55–79)	260 (186–350)	40 (31–51)	839 (664–1,072)	0 (0–0)	112 (59–232)	29 (24–35)	284 (202–373)	8 (8–9)
France	Number: Cumulative change (%)	-60.9 (-65.5 to -55.6)	-77.3 (-84.7 to -69.6)	-64.5 (-71.5 to -56.8)	-65.2 (-76.2 to -52.3)	-48.8 (-62.1 to -32.6)	-51.2 (-63.1 to -28.8)	nan (nan–nan)	-57.8 (-74.8 to -29.4)	-79.2 (-83.2 to -74.5)	63.7 (12.5–121.8)	-80.7 (-82.3 to -79.1)
France	Rate: 1990	72.1 (71.2–73.0)	7.0 (6.3–7.9)	1.3 (1.2–1.4)	5.5 (4.8–6.2)	0.6 (0.5–0.7)	12.4 (10.7–13.4)	0.0 (0.0–0.0)	1.9 (1.4–2.9)	1.0 (0.9–1.1)	1.3 (1.1–1.5)	0.3 (0.3–0.3)
France	Rate: 2015	28.0 (24.7–31.9)	1.5 (1.0–2.0)	0.4 (0.4–0.5)	1.8 (1.3–2.4)	0.3 (0.2–0.3)	5.7 (4.5–7.3)	0.0 (0.0–0.0)	0.8 (0.4–1.6)	0.2 (0.2–0.2)	2.0 (1.4–2.6)	0.1 (0.1–0.1)
France	Rate: Cumulative change (%)	-61.1 (-65.8 to -55.7)	-78.5 (-85.5 to -71.1)	-65.6 (-72.4 to -57.8)	-67.0 (-77.4 to -54.7)	-51.1 (-63.9 to -35.5)	-53.4 (-64.9 to -31.9)	nan (nan–nan)	-59.9 (-76.2 to -33.0)	-79.8 (-83.7 to -75.3)	55.5 (6.8–110.7)	-80.8 (-82.4 to -79.1)
Germany	Number: 1990	11,929 (11,791–12,071)	2,134 (1,941–2,380)	226 (205–251)	453 (378–539)	24 (20–28)	2,310 (1,597–2,536)	0 (0–0)	199 (122–399)	159 (143–177)	206 (164–255)	37 (34–39)
Germany	Number: 2015	3,942 (3,647–4,319)	532 (422–651)	69 (60–79)	178 (140–224)	22 (18–26)	914 (790–1,062)	0 (0–0)	87 (46–194)	31 (27–35)	129 (100–162)	7 (7–7)
Germany	Number: Cumulative change (%)	-66.9 (-69.6 to -63.8)	-75.0 (-81.0 to -69.2)	-69.2 (-73.9 to -63.9)	-60.1 (-70.7 to -48.0)	-7.2 (-28.1 to 14.8)	-59.7 (-67.5 to -40.6)	nan (nan–nan)	-56.9 (-70.4 to -40.0)	-80.3 (-83.2 to -77.0)	-36.0 (-54.8 to -14.2)	-79.8 (-81.5 to -78.1)
Germany	Rate: 1990	73.5 (72.6–74.4)	14.0 (12.8–15.6)	1.4 (1.3–1.6)	3.0 (2.5–3.5)	0.2 (0.1–0.2)	14.9 (10.3–16.3)	0.0 (0.0–0.0)	1.3 (0.8–2.6)	1.0 (0.9–1.1)	1.4 (1.1–1.7)	0.2 (0.2–0.2)
Germany	Rate: 2015	29.2 (26.9–32.2)	4.2 (3.3–5.2)	0.5 (0.4–0.6)	1.4 (1.1–1.8)	0.2 (0.1–0.2)	7.1 (6.1–8.3)	0.0 (0.0–0.0)	0.7 (0.4–1.5)	0.2 (0.2–0.3)	1.0 (0.8–1.3)	0.1 (0.1–0.1)
Germany	Rate: Cumulative change (%)	-60.3 (-63.6 to -56.3)	-69.8 (-77.2 to -62.9)	-63.1 (-68.9 to -56.5)	-51.8 (-64.7 to -37.2)	12.1 (-13.1 to 39.0)	-51.4 (-60.9 to -28.2)	nan (nan–nan)	-48.1 (-64.3 to -27.6)	-76.1 (-79.7 to -72.1)	-22.8 (-45.5 to 3.5)	-75.4 (-77.4 to -73.4)
Greece	Number: 1990	1,764 (1,735–1,793)	348 (312–448)	56 (47–90)	84 (70–112)	1 (1–2)	470 (179–526)	0 (0–0)	21 (5–33)	11 (10–17)	9 (7–12)	2 (2–2)
Greece	Number: 2015	562 (539–588)	79 (69–92)	18 (15–24)	18 (14–23)	0 (0–0)	167 (106–190)	0 (0–0)	3 (2–6)	3 (2–4)	7 (5–9)	0 (0–0)
Greece	Number: Cumulative change (%)	-68.1 (-69.6 to -66.6)	-76.9 (-80.6 to -73.3)	-67.1 (-73.5 to -60.4)	-78.3 (-83.3 to -72.9)	-60.3 (-69.2 to -51.5)	-63.0 (-69.7 to -40.4)	nan (nan–nan)	-74.7 (-91.6 to -37.4)	-71.1 (-76.3 to -65.2)	-24.6 (-44.3 to -0.7)	-75.8 (-77.8 to -73.6)
Greece	Rate: 1990	78.4 (76.9–79.8)	18.1 (16.3–23.3)	2.6 (2.2–4.3)	4.4 (3.7–5.8)	0.1 (0.1–0.1)	23.1 (8.9–25.8)	0.0 (0.0–0.0)	1.1 (0.3–1.7)	0.5 (0.4–0.8)	0.5 (0.4–0.7)	0.1 (0.1–0.1)
Greece	Rate: 2015	29.5 (28.2–31.0)	4.7 (4.1–5.4)	1.0 (0.8–1.3)	1.1 (0.8–1.4)	0.0 (0.0–0.0)	9.3 (6.0–10.6)	0.0 (0.0–0.0)	0.2 (0.1–0.4)	0.2 (0.1–0.2)	0.4 (0.3–0.5)	0.0 (0.0–0.0)
Greece	Rate: Cumulative change (%)	-62.3 (-64.2 to -60.4)	-73.9 (-78.0 to -69.8)	-62.0 (-69.8 to -54.1)	-75.4 (-81.1 to -69.3)	-55.4 (-65.9 to -44.6)	-58.0 (-65.7 to -32.1)	nan (nan–nan)	-71.4 (-90.5 to -29.3)	-66.6 (-73.0 to -59.3)	-14.7 (-37.1 to 12.3)	-70.9 (-73.6 to -68.0)
Iceland	Number: 1990	45 (42–47)	6 (5–7)	2 (2–3)	2 (1–2)	0 (0–0)	8 (8–9)	0 (0–0)	0 (0–1)	0 (0–0)	0 (0–0)	0 (0–0)
Iceland	Number: 2015	16 (15–18)	1 (0–1)	0 (0–1)	0 (0–0)	0 (0–0)	3 (3–4)	0 (0–0)	0 (0–0)	0 (0–0)	0 (0–0)	0 (0–0)
Iceland	Number: Cumulative change (%)	-62.9 (-66.0 to -59.3)	-80.7 (-86.4 to -75.7)	-68.3 (-73.9 to -61.7)	-73.5 (-80.3 to -65.3)	-25.4 (-45.1 to -1.9)	-55.1 (-63.1 to -44.1)	nan (nan–nan)	-51.8 (-67.2 to -30.7)	-80.3 (-83.7 to -76.1)	-6.3 (-39.6 to 29.8)	-65.0 (-68.0 to -62.0)
Iceland	Rate: 1990	54.4 (51.4–57.5)	7.9 (6.8–9.0)	3.6 (3.2–4.0)	2.5 (2.1–3.0)	0.2 (0.1–0.2)	10.9 (9.8–12.1)	0.0 (0.0–0.0)	0.8 (0.5–1.4)	1.0 (0.9–1.2)	0.4 (0.3–0.5)	0.1 (0.1–0.1)
Iceland	Rate: 2015	19.4 (17.9–21.0)	1.5 (1.0–1.9)	1.1 (0.9–1.3)	0.7 (0.5–0.8)	0.1 (0.1–0.2)	4.8 (4.0–5.8)	0.0 (0.0–0.0)	0.4 (0.2–0.8)	0.2 (0.2–0.2)	0.4 (0.3–0.5)	0.0 (0.0–0.0)
Iceland	Rate: Cumulative change (%)	-64.4 (-67.5 to -60.9)	-80.6 (-86.3 to -75.5)	-69.2 (-74.7 to -62.7)	-73.3 (-80.1 to -65.0)	-26.3 (-45.9 to -2.9)	-55.6 (-63.6 to -44.7)	nan (nan–nan)	-51.5 (-67.0 to -30.2)	-81.1 (-84.4 to -77.0)	-5.6 (-39.2 to 30.8)	-66.3 (-69.2 to -63.4)
Ireland	Number: 1990	870 (828–914)	104 (92–120)	33 (30–37)	35 (29–42)	1 (1–2)	191 (154–209)	0 (0–0)	4 (2–8)	13 (12–15)	8 (6–10)	0 (0–0)
Ireland	Number: 2015	378 (355–403)	35 (29–42)	12 (10–14)	13 (10–16)	2 (2–3)	112 (99–127)	0 (0–0)	4 (3–7)	3 (3–4)	11 (9–14)	0 (0–0)
Ireland	Number: Cumulative change (%)	-56.5 (-60.0 to -52.8)	-65.9 (-73.6 to -58.2)	-63.4 (-68.8 to -56.7)	-61.0 (-70.9 to -48.8)	63.2 (23.7–108.2)	-40.5 (-49.9 to -22.4)	nan (nan–nan)	4.1 (-31.3 to 47.6)	-71.4 (-75.8 to -66.5)	37.8 (-0.1 to 83.0)	-52.7 (-57.0 to -48.2)
Ireland	Rate: 1990	78.3 (74.9–81.9)	11.1 (9.8–12.8)	3.1 (2.8–3.4)	3.7 (3.1–4.5)	0.2 (0.1–0.2)	19.2 (15.5–21.0)	0.0 (0.0–0.0)	0.5 (0.3–0.9)	1.2 (1.1–1.4)	0.9 (0.7–1.1)	0.1 (0.1–0.1)
Ireland	Rate: 2015	30.3 (28.5–32.3)	2.8 (2.3–3.3)	1.0 (0.8–1.1)	1.1 (0.9–1.3)	0.2 (0.2–0.3)	8.9 (7.8–10.0)	0.0 (0.0–0.0)	0.4 (0.2–0.6)	0.3 (0.3–0.3)	0.9 (0.7–1.1)	0.0 (0.0–0.0)
Ireland	Rate: Cumulative change (%)	-61.3 (-64.2 to -58.2)	-74.5 (-80.2 to -68.7)	-68.7 (-73.5 to -63.0)	-70.8 (-78.2 to -61.6)	27.6 (-3.6 to 63.0)	-53.4 (-60.7 to -39.1)	nan (nan–nan)	-22.2 (-48.6 to 10.4)	-74.9 (-78.8 to -70.6)	3.5 (-25.2 to 38.0)	-55.3 (-59.2 to -51.1)
Israel	Number: 1990	1,605 (1,565–1,645)	325 (292–369)	72 (64–83)	70 (57–85)	9 (7–11)	356 (239–392)	0 (0–0)	26 (16–53)	20 (18–24)	46 (38–57)	4 (4–4)
Israel	Number: 2015	939 (890–994)	88 (68–106)	26 (23–30)	32 (25–39)	10 (8–11)	264 (228–296)	0 (0–0)	20 (11–45)	8 (7–9)	56 (45–68)	2 (2–3)
Israel	Number: Cumulative change (%)	-41.5 (-45.0 to -38.0)	-72.6 (-79.5 to -66.4)	-63.1 (-68.8 to -56.4)	-53.4 (-65.8 to -39.8)	8.8 (-15.0 to 36.9)	-24.4 (-39.1 to 11.6)	nan (nan–nan)	-22.0 (-44.8 to 7.0)	-60.7 (-66.7 to -53.3)	22.2 (-11.0 to 58.1)	-35.0 (-40.9 to -28.9)
Israel	Rate: 1990	87.4 (85.2–89.6)	17.6 (15.9–20.1)	3.9 (3.5–4.5)	3.8 (3.1–4.6)	0.5 (0.4–0.6)	19.3 (13.0–21.3)	0.0 (0.0–0.0)	1.4 (0.9–2.9)	1.1 (1.0–1.3)	2.5 (2.1–3.1)	0.2 (0.2–0.3)

© 2017 Global Burden of Disease Child and Adolescent Health Collaboration. JAMA Pediatrics.

eTable 1. Number of Deaths, Death Rates (per 100,000 population), and Cumulative Percent Change with 95% Uncertainty Intervals (UI) for the Top 10 Global Causes of Death in 195 Countries and Territories, Aged 0 to 19, Both Sexes, 1990 and 2015*Note: best viewed by enlarging in browser.*

Location	Measure	All causes	Neonatal preterm birth complications	Lower respiratory infections	Neonatal encephalopathy due to birth asphyxia and trauma	Diarrheal diseases	Congenital anomalies	Malaria	Neonatal sepsis and other neonatal infections	Meningitis	Other neonatal disorders	HIV/AIDS
Israel	Rate: 2015	31.7 (30.1–33.5)	2.9 (2.2–3.5)	0.9 (0.8–1.0)	1.1 (0.8–1.3)	0.3 (0.3–0.4)	8.6 (7.5–9.7)	0.0 (0.0–0.0)	0.7 (0.4–1.5)	0.3 (0.2–0.3)	1.8 (1.5–2.2)	0.1 (0.1–0.1)
Israel	Rate: Cumulative change (%)	-63.7 (-65.8 to -61.6)	-83.6 (-87.7 to -79.9)	-77.3 (-80.8 to -73.1)	-72.1 (-79.5 to -63.9)	-34.4 (-48.8 to -17.5)	-54.6 (-63.4 to -32.8)	nan (nan–nan)	-53.3 (-67.0 to -35.9)	-76.1 (-79.7 to -71.7)	-26.8 (-46.7 to -5.2)	-59.6 (-63.2 to -55.8)
Italy	Number: 1990	8,980 (8,865–9,088)	1,737 (1,605–1,889)	282 (252–326)	682 (599–771)	17 (14–20)	1,640 (1,301–1,760)	0 (0–0)	91 (40–122)	74 (67–85)	212 (181–249)	32 (31–34)
Italy	Number: 2015	2,816 (2,554–3,116)	331 (259–413)	48 (40–55)	133 (102–169)	12 (9–15)	607 (496–747)	0 (0–0)	46 (25–65)	15 (13–18)	198 (154–255)	8 (8–9)
Italy	Number: Cumulative change (%)	-68.6 (-71.6 to -65.1)	-80.9 (-85.5 to -75.7)	-82.8 (-86.2 to -79.3)	-80.4 (-85.6 to -74.1)	-28.2 (-47.6 to -5.8)	-62.4 (-70.6 to -42.3)	nan (nan–nan)	-47.1 (-63.0 to -26.9)	-79.2 (-82.7 to -75.4)	-5.4 (-30.7 to 29.5)	-73.3 (-75.5 to -71.0)
Italy	Rate: 1990	73.7 (72.6–74.8)	16.9 (15.6–18.4)	2.5 (2.2–2.9)	6.7 (5.8–7.5)	0.2 (0.1–0.2)	15.4 (12.1–16.5)	0.0 (0.0–0.0)	0.9 (0.4–1.2)	0.6 (0.6–0.7)	2.1 (1.8–2.4)	0.3 (0.3–0.3)
Italy	Rate: 2015	26.9 (24.2–30.1)	3.6 (2.8–4.5)	0.5 (0.4–0.5)	1.4 (1.1–1.8)	0.1 (0.1–0.2)	6.3 (5.1–7.8)	0.0 (0.0–0.0)	0.5 (0.3–0.7)	0.1 (0.1–0.2)	2.2 (1.7–2.8)	0.1 (0.1–0.1)
Italy	Rate: Cumulative change (%)	-63.5 (-67.3 to -59.0)	-78.7 (-83.9 to -73.1)	-81.4 (-85.2 to -77.3)	-78.2 (-84.0 to -71.2)	-20.5 (-42.0 to 5.0)	-58.4 (-67.6 to -35.6)	nan (nan–nan)	-41.2 (-58.9 to -18.8)	-77.0 (-81.1 to -72.5)	5.2 (-23.0 to 44.0)	-69.3 (-71.9 to -66.5)
Luxembourg	Number: 1990	67 (64–70)	6 (5–7)	2 (2–2)	5 (4–6)	0 (0–0)	8 (7–9)	0 (0–0)	0 (0–1)	0 (0–0)	3 (2–3)	0 (0–0)
Luxembourg	Number: 2015	28 (27–31)	1 (1–1)	0 (0–1)	1 (0–1)	0 (0–0)	5 (4–6)	0 (0–0)	0 (0–0)	0 (0–0)	2 (1–2)	0 (0–0)
Luxembourg	Number: Cumulative change (%)	-57.2 (-60.7 to -53.5)	-76.4 (-82.8 to -70.0)	-61.6 (-69.3 to -53.2)	-79.5 (-84.5 to -73.6)	42.0 (6.9–84.4)	-42.1 (-54.4 to -15.4)	nan (nan–nan)	-44.8 (-60.6 to -24.4)	-77.0 (-80.9 to -72.7)	-34.3 (-51.2 to -15.3)	-65.6 (-68.6 to -62.7)
Luxembourg	Rate: 1990	76.7 (73.1–80.3)	7.1 (6.1–8.1)	2.8 (2.4–3.2)	6.2 (5.3–7.1)	0.5 (0.4–0.6)	10.0 (8.7–11.1)	0.0 (0.0–0.0)	0.9 (0.6–1.6)	0.8 (0.7–0.9)	3.8 (3.2–4.6)	0.1 (0.1–0.1)
Luxembourg	Rate: 2015	23.1 (21.5–25.0)	1.2 (0.9–1.5)	0.8 (0.6–0.9)	0.9 (0.7–1.1)	0.5 (0.4–0.7)	4.2 (3.5–5.4)	0.0 (0.0–0.0)	0.4 (0.2–0.7)	0.1 (0.1–0.2)	1.8 (1.4–2.3)	0.0 (0.0–0.0)
Luxembourg	Rate: Cumulative change (%)	-69.8 (-72.3 to -67.1)	-82.5 (-87.2 to -77.8)	-72.3 (-77.9 to -66.1)	-84.9 (-88.5 to -80.4)	3.9 (-21.7 to 35.0)	-57.8 (-66.8 to -38.2)	nan (nan–nan)	-59.1 (-70.9 to -44.0)	-83.6 (-86.5 to -80.5)	-51.4 (-63.9 to -37.3)	-76.0 (-78.0 to -73.9)
Malta	Number: 1990	78 (75–82)	19 (17–21)	3 (3–4)	4 (3–4)	0 (0–0)	20 (17–22)	0 (0–0)	0 (0–1)	0 (0–0)	0 (0–0)	0 (0–0)
Malta	Number: 2015	35 (32–37)	6 (5–7)	1 (1–1)	1 (1–1)	0 (0–0)	10 (8–12)	0 (0–0)	0 (0–0)	0 (0–0)	1 (0–1)	0 (0–0)
Malta	Number: Cumulative change (%)	-55.4 (-59.2 to -51.2)	-65.5 (-71.4 to -59.4)	-69.6 (-74.9 to -64.0)	-64.5 (-73.0 to -54.3)	26.2 (-2.8 to 58.9)	-46.4 (-53.3 to -37.8)	nan (nan–nan)	-47.8 (-59.9 to -33.6)	-81.7 (-84.4 to -78.6)	35.9 (-0.3 to 82.2)	-65.5 (-68.3 to -62.6)
Malta	Rate: 1990	73.8 (70.3–77.5)	18.4 (16.7–20.3)	3.7 (3.3–4.2)	3.8 (3.2–4.5)	0.3 (0.3–0.4)	19.1 (16.3–21.1)	0.0 (0.0–0.0)	0.6 (0.4–1.1)	0.8 (0.7–0.9)	0.8 (0.6–0.9)	0.1 (0.1–0.1)
Malta	Rate: 2015	46.0 (42.4–50.1)	9.6 (8.1–11.5)	1.6 (1.4–1.9)	2.1 (1.6–2.5)	0.6 (0.5–0.7)	15.2 (12.4–17.5)	0.0 (0.0–0.0)	0.5 (0.3–0.9)	0.2 (0.2–0.2)	1.6 (1.2–1.9)	0.0 (0.0–0.0)
Malta	Rate: Cumulative change (%)	-37.7 (-43.3 to -31.4)	-47.6 (-56.5 to -38.3)	-55.8 (-63.7 to -47.4)	-46.0 (-59.0 to -30.4)	90.9 (46.6–141.5)	-20.2 (-30.8 to -7.2)	nan (nan–nan)	-20.6 (-38.9 to 1.0)	-74.2 (-78.1 to -69.8)	106.8 (51.7–177.3)	-53.5 (-57.4 to -49.4)
Netherlands	Number: 1990	2,432 (2,386–2,482)	351 (313–397)	52 (46–58)	187 (162–215)	4 (4–5)	587 (498–634)	0 (0–0)	39 (17–53)	58 (52–65)	51 (42–60)	7 (6–7)
Netherlands	Number: 2015	1,078 (1,012–1,155)	108 (91–127)	27 (24–31)	64 (52–78)	7 (6–8)	259 (225–326)	0 (0–0)	28 (13–38)	15 (13–17)	70 (56–85)	1 (1–1)
Netherlands	Number: Cumulative change (%)	-55.6 (-58.6 to -52.5)	-69.1 (-75.4 to -62.8)	-46.3 (-54.7 to -37.0)	-65.4 (-73.3 to -56.1)	56.9 (19.4–96.2)	-55.5 (-63.2 to -34.2)	nan (nan–nan)	-28.0 (-46.1 to -9.1)	-73.0 (-77.1 to -68.9)	40.1 (3.5–81.0)	-77.9 (-79.7 to -76.0)
Netherlands	Rate: 1990	66.2 (64.9–67.6)	10.0 (8.9–11.3)	1.5 (1.3–1.6)	5.3 (4.6–6.1)	0.1 (0.1–0.2)	16.5 (14.0–17.9)	0.0 (0.0–0.0)	1.1 (0.5–1.5)	1.6 (1.5–1.8)	1.5 (1.2–1.7)	0.2 (0.2–0.2)
Netherlands	Rate: 2015	30.2 (28.1–32.5)	3.3 (2.8–3.9)	0.8 (0.7–0.9)	2.0 (1.6–2.4)	0.2 (0.2–0.3)	7.7 (6.7–9.7)	0.0 (0.0–0.0)	0.9 (0.4–1.2)	0.5 (0.4–0.5)	2.2 (1.7–2.6)	0.0 (0.0–0.0)
Netherlands	Rate: Cumulative change (%)	-54.4 (-57.7 to -50.9)	-66.7 (-73.6 to -60.0)	-46.2 (-54.9 to -36.7)	-62.7 (-71.3 to -52.7)	63.5 (24.5–104.8)	-53.1 (-61.3 to -30.9)	nan (nan–nan)	-22.5 (-42.0 to -2.2)	-72.4 (-76.8 to -68.1)	50.8 (11.4–94.9)	-77.9 (-79.8 to -75.9)
Norway	Number: 1990	810 (788–833)	66 (56–76)	23 (20–25)	95 (84–107)	2 (1–2)	155 (142–168)	0 (0–0)	4 (2–6)	17 (15–20)	30 (25–35)	0 (0–0)
Norway	Number: 2015	297 (281–316)	19 (14–23)	5 (4–6)	22 (16–27)	3 (2–3)	68 (57–93)	0 (0–0)	2 (1–3)	3 (2–3)	11 (8–14)	0 (0–0)
Norway	Number: Cumulative change (%)	-63.2 (-65.4 to -60.6)	-70.1 (-79.8 to -62.5)	-74.5 (-79.7 to -69.3)	-76.4 (-82.8 to -70.9)	52.0 (12.2–90.8)	-56.0 (-64.0 to -36.2)	nan (nan–nan)	-45.9 (-63.3 to -22.0)	-82.2 (-85.1 to -78.7)	-61.2 (-72.9 to -48.6)	-62.8 (-66.5 to -59.1)
Norway	Rate: 1990	72.7 (70.7–74.8)	6.2 (5.3–7.2)	2.2 (1.9–2.4)	9.0 (7.9–10.0)	0.2 (0.2–0.2)	14.5 (13.3–15.7)	0.0 (0.0–0.0)	0.4 (0.2–0.6)	1.6 (1.4–1.9)	2.8 (2.3–3.3)	0.1 (0.1–0.1)
Norway	Rate: 2015	24.1 (22.7–25.7)	1.7 (1.3–2.1)	0.5 (0.4–0.6)	2.0 (1.5–2.4)	0.3 (0.2–0.3)	5.9 (5.0–8.1)	0.0 (0.0–0.0)	0.2 (0.1–0.3)	0.3 (0.2–0.3)	1.0 (0.7–1.3)	0.0 (0.0–0.0)
Norway	Rate: Cumulative change (%)	-66.8 (-69.0 to -64.4)	-71.6 (-80.8 to -64.5)	-76.7 (-81.5 to -71.8)	-77.6 (-83.7 to -72.4)	39.9 (3.9–75.8)	-59.2 (-66.7 to -40.6)	nan (nan–nan)	-48.8 (-65.2 to -26.0)	-84.0 (-86.7 to -80.9)	-63.2 (-74.3 to -51.3)	-66.7 (-70.1 to -63.2)
Portugal	Number: 1990	3,006 (2,982–3,030)	367 (324–446)	180 (163–216)	137 (116–168)	29 (24–36)	479 (250–533)	0 (0–0)	42 (13–60)	58 (52–68)	26 (21–34)	3 (3–4)
Portugal	Number: 2015	510 (496–526)	25 (21–29)	20 (18–23)	14 (11–17)	4 (4–5)	103 (93–112)	0 (0–0)	9 (5–12)	5 (4–6)	23 (19–28)	3 (3–4)
Portugal	Number: Cumulative change (%)	-83.0 (-83.5 to -82.5)	-93.0 (-94.9 to -91.7)	-88.3 (-90.4 to -86.5)	-89.6 (-92.2 to -86.9)	-83.4 (-87.6 to -79.3)	-77.4 (-81.7 to -57.3)	nan (nan–nan)	-74.4 (-84.2 to -55.6)	-90.7 (-92.0 to -89.3)	-9.8 (-34.2 to 16.8)	-3.4 (-11.9 to 5.3)
Portugal	Rate: 1990	118.7 (117.6–119.8)	17.7 (15.6–21.5)	7.8 (7.0–9.4)	6.6 (5.6–8.1)	1.4 (1.1–1.7)	22.0 (11.5–24.6)	0.0 (0.0–0.0)	2.0 (0.7–2.9)	2.5 (2.2–2.9)	1.3 (1.0–1.7)	0.1 (0.1–0.2)
Portugal	Rate: 2015	27.4 (26.5–28.4)	1.7 (1.4–2.0)	1.1 (1.0–1.3)	0.9 (0.8–1.1)	0.3 (0.2–0.3)	6.3 (5.7–6.9)	0.0 (0.0–0.0)	0.6 (0.4–0.8)	0.3 (0.3–0.3)	1.6 (1.3–1.9)	0.2 (0.2–0.2)
Portugal	Rate: Cumulative change (%)	-76.9 (-77.7 to -76.1)	-90.5 (-93.0 to -88.7)	-85.3 (-87.9 to -82.8)	-85.8 (-89.4 to -82.1)	-78.5 (-84.0 to -73.0)	-69.9 (-75.8 to -42.7)	nan (nan–nan)	-65.2 (-78.5 to -39.5)	-87.9 (-89.6 to -86.0)	22.9 (-10.4 to 58.9)	36.8 (24.7–49.3)
Spain	Number: 1990	7,267 (7,174–7,354)	839 (755–921)	215 (194–248)	333 (285–383)	32 (28–40)	1,318 (867–1,444)	0 (0–0)	160 (113–281)	160 (144–181)	101 (83–121)	50 (48–53)
Spain	Number: 2015	2,133 (2,003–2,277)	154 (116–187)	44 (39–50)	94 (72–115)	11 (9–13)	478 (414–556)	0 (0–0)	94 (64–162)	26 (22–29)	137 (104–170)	9 (9–10)
Spain	Number: Cumulative change (%)	-70.6 (-72.5 to -68.6)	-81.6 (-86.5 to -77.3)	-79.1 (-82.6 to -75.5)	-71.5 (-79.1 to -64.2)	-65.6 (-73.6 to -57.0)	-62.7 (-70.2 to -37.7)	nan (nan–nan)	-39.9 (-54.6 to -24.6)	-83.6 (-86.0 to -80.9)	37.3 (-1.6 to 77.9)	-81.0 (-82.6 to -79.4)
Spain	Rate: 1990	77.3 (76.1–78.4)	11.3 (10.2–12.4)	2.5 (2.2–2.9)	4.5 (3.8–5.2)	0.4 (0.4–0.5)	16.7 (11.0–18.4)	0.0 (0.0–0.0)	2.2 (1.5–3.8)	1.8 (1.6–2.1)	1.4 (1.1–1.6)	0.5 (0.5–0.5)
Spain	Rate: 2015	25.5 (23.9–27.4)	2.0 (1.5–2.5)	0.5 (0.5–0.6)	1.2 (0.9–1.5)	0.1 (0.1–0.2)	6.0 (5.2–7.0)	0.0 (0.0–0.0)	1.2 (0.8–2.1)	0.3 (0.3–0.4)	1.8 (1.4–2.2)	0.1 (0.1–0.1)
Spain	Rate: Cumulative change (%)	-67.0 (-69.2 to -64.4)	-82.1 (-86.9 to -78.0)	-78.4 (-82.2 to -74.5)	-72.3 (-79.7 to -65.2)	-66.4 (-74.3 to -57.8)	-63.1 (-70.7 to -38.2)	nan (nan–nan)	-41.7 (-55.9 to -26.7)	-82.9 (-85.5 to -80.0)	33.5 (-4.4 to 73.0)	-78.7 (-80.4 to -76.9)
Sweden	Number: 1990	1,226 (1,193–1,259)	141 (123–159)	29 (25–33)	116 (102–131)	1 (1–1)	311 (283–365)	0 (0–0)	17 (6–23)	14 (12–15)	24 (20–29)	1 (1–1)
Sweden	Number: 2015	515 (487–547)	37 (24–45)	9 (6–11)	33 (23–41)	3 (2–3)	127 (107–205)	0 (0–0)	9 (4–13)	3 (2–3)	28 (19–35)	0 (0–0)
Sweden	Number: Cumulative change (%)	-58.0 (-60.6 to -55.1)	-73.6 (-82.4 to -67.1)	-66.9 (-73.4 to -60.6)	-70.8 (-79.5 to -63.6)	81.8 (24.2–123.0)	-59.2 (-66.1 to -43.9)	nan (nan–nan)	-42.7 (-57.9 to -21.5)	-77.6 (-80.8 to -73.4)	15.8 (-16.8 to 52.8)	-66.9 (-69.2 to -64.6)
Sweden	Rate: 1990	56.9 (55.4–58.4)	6.6 (5.8–7.5)	1.4 (1.2–1.6)	5.4 (4.8–6.1)	0.1 (0.1–0.1)	14.6 (13.3–17.1)	0.0 (0.0–0.0)	0.8 (0.3–1.1)	0.7 (0.6–0.7)	1.2 (1.0–1.4)	0.1 (0.1–0.1)
Sweden	Rate: 2015	23.2 (21.9–24.7)	1.7 (1.1–2.1)	0.4 (0.3–0.5)	1.5 (1.1–1.9)	0.1 (0.1–0.2)	5.8 (4.9–9.4)	0.0 (0.0–0.0)	0.4 (0.2–0.6)	0.1 (0.1–0.2)	1.3 (0.9–1.6)	0.0 (0.0–0.0)
Sweden	Rate: Cumulative change (%)	-59.2 (-61.8 to -56.3)	-74.1 (-82.8 to -67.7)	-68.2 (-74.5 to -62.2)	-71.4 (-79.9 to -64.3)	74.0 (18.1–113.9)	-60.3 (-66.9 to -45.4)	nan (nan–nan)	-43.8 (-58.7 to -23.0)	-78.5 (-81.6 to -74.4)	13.5 (-18.5 to 49.7)	-68.3 (-70.5 to -66.1)
Switzerland	Number: 1990	1,122 (1,097–1,148)	150 (129–186)	28 (24–32)	53 (43–65)	7 (6–8)	280 (197–310)	0 (0–0)	4 (3–7)	21 (19–23)	34 (27–44)	3 (3–3)
Switzerland	Number: 2015	529 (485–581)	58 (48–72)	11 (9–13)	37 (29–47)	5 (4–6)	139 (120–159)	0 (0–0)	6 (2–8)	2 (2–2)	24 (19–31)	0 (0–0)
Switzerland	Number: Cumulative change (%)	-52.8 (-56.8 to -48.2)	-60.4 (-69.7 to -50.6)	-58.9 (-66.6 to -50.2)	-28.9 (-47.5 to -5.2)	-28.4 (-44.5 to -9.9)	-49.4 (-58.7 to -25.7)	nan (nan–nan)	51.1 (-34.5 to 144.9)	-88.5 (-90.4 to -86.3)	-27.1 (-49.2 to -0.9)	-72.4 (-74.8 to -69.8)
Switzerland	Rate: 1990	72.2 (70.5–74.0)	10.1 (8.8–12.6)	1.9 (1.7–2.1)	3.6 (3.0–4.4)	0.5 (0.4–0.5)	18.8 (13.2–20.8)	0.0 (0.0–0.0)	0.3 (0.2–0.5)	1.4 (1.3–1.6)	2.4 (1.9–3.0)	0.2 (0.2–0.2)
Switzerland	Rate: 2015	32.3 (29.5–35.5)	3.7 (3.1–4.6)	0.7 (0.6–0.8)	2.4 (1.9–3.0)	0.3 (0.3–0.4)	8.7 (7.6–10.1)	0.0 (0.0–0.0)	0.4 (0.2–0.5)	0.2 (0.1–0.2)	1.6 (1.2–2.0)	0.1 (0.1–0.1)
Switzerland	Rate: Cumulative change (%)	-55.3 (-59.3 to -50.7)	-62.9 (-71.6 to -53.8)	-61.4 (-68.7 to -53.3)	-33.4 (-50.8 to -11.2)	-32.8 (-48.0 to -15.4)	-52.7 (-61.4 to -30.4)	nan (nan–nan)	41.5 (-38.6 to 129.3)	-89.1 (-90.9 to -87.0)	-31.8 (-52.5 to -7.2)	-73.7 (-76.1 to -71.3)
United Kingdom	Number: 1990	10,689 (10,600–10,774)	1,962 (1,891–2,040)	409 (388–430)	789 (745–836)	31 (29–34)	1,914 (1,847–1,992)	0 (0–0)	116 (61–140)	246 (231–261)	150 (139–161)	11 (10–11)
United Kingdom	Number: 2015	5,387 (5,289–5,485)	1,198 (1,053–1,273)	189 (172–203)	233 (207–257)	34 (30–37)	1,178 (1,086–1,523)	0 (0–0)	60 (45–102)	67 (62–71)	126 (110–140)	10 (9–10)
United Kingdom	Number: Cumulative change (%)	-49.6 (-50.6 to -48.6)	-38.9 (-47.4 to -34.1)	-53.8 (-58.0 to -49.3)	-70.4 (-73.8 to -67.0)	10.1 (-4.5 to 23.2)	-38.4 (-43.8 to -23.6)	nan (nan–nan)	-45.0 (-64.0 to -9.6)	-72.7 (-74.9 to -70.2)	-15.8 (-27.5 to -3.9)	-9.2 (-12.7 to -5.5)
United Kingdom	Rate: 1990	72.8 (72.2–73.4)	13.7 (13.2–14.3)	2.8 (2.7–3.0)	5.5 (5.2–5.8)	0.2 (0.2–0.2)	13.3 (12.8–13.8)	0.0 (0.0–0.0)	0.8 (0.4–1.0)	1.7 (1.6–1.8)	1.0 (1.0–1.1)	0.1 (0.1–0.1)
United Kingdom	Rate: 2015	35.8 (35.2–36.5)	8.0 (7.0–8.5)	1.3 (1.2–1.4)	1.6 (1.4–1.7)	0.2 (0.2–0.3)	7.9 (7.3–10.2)	0.0 (0.0–0.0)	0.4 (0.3–0.7)	0.4 (0.4–0.5)	0.8 (0.7–0.9)	0.1 (0.1–0.1)
United Kingdom	Rate: Cumulative change (%)	-50.8 (-51.8 to -49.7)	-41.6 (-49.7 to -37.0)	-55.1 (-59.2 to -50.8)	-71.8 (-75.0 to -68.4)	6.4 (-7.7 to 19.0)	-40.7 (-45.9 to -26.4)	nan (nan–nan)	-47.5 (-65.6 to -13.6)	-73.4 (-75.5 to -71.0)	-19.6 (-30.8 to -8.2)	-10.6 (-14.1 to -7.0)
England	Number: 1990	8,883 (8,808–8,964)	1,667 (1,605–1,736)	331 (311–350)	668 (626–711)	26 (24–28)	1,570 (1,509–1,649)	0 (0–0)	95 (51–117)	219 (205–233)	120 (109–130)	9 (9–9)
England	Number: 2015	4,567 (4,484–4,654)	1,062 (927–1,133)	155 (140–168)	197 (174–218)	25 (21–27)	998 (912–1,300)	0 (0–0)	48 (35–87)	60 (55–64)	103 (89–117)	9 (8–9)
England	Number: Cumulative change (%)	-48.6 (-49.7 to -47.5)	-36.2 (-45.2 to -31.0)	-53.0 (-57.7 to -47.9)	-70.4 (-74.0 to -66.6)	-4.4 (-18.2 to 8.8)	-36.4 (-42.3 to -20.7)	nan (nan–nan)	-46.1 (-66.3 to -8.4)	-72.4 (-74.7 to -69.8)	-13.5 (-26.5 to -0.0)	-5.1 (-9.1 to -0.9)
England	Rate: 1990	72.7 (72.1–73.4)	13.9 (13.4–14.5)	2.7 (2.6–2.9)	5.6 (5.2–6.0)	0.2 (0.2–0.2)	13.1 (12.5–13.7)	0.0 (0.0–0.0)	0.8 (0.4–1.0)	1.8 (1.7–1.9)	1.0 (0.9–1.1)	0.1 (0.1–0.1)
England	Rate: 2015	35.6 (34.9–36.3)	8.3 (7.2–8.8)	1.2 (1.1–1.3)	1.5 (1.4–1.7)	0.2 (0.2–0.2)	7.8 (7.1–10.1)	0.0 (0.0–0.0)	0.4 (0.3–0.7)	0.5 (0.4–0.5)	0.8 (0.7–0.9)	0.1 (0.1–0.1)
England	Rate: Cumulative change (%)	-51.1 (-52.1 to -50.0)	-40.8 (-49.1 to -35.9)	-55.5 (-60.0 to -50.7)	-72.5 (-75.8 to -69.0)	-9.9 (-23.0 to 2.6)	-40.5 (-46.0 to -25.6)	nan (nan–nan)	-50.0 (-68.8 to -14.9)	-73.8 (-76.1 to -71.4)	-19.7 (-31.8 to -7.2)	-9.2 (-13.0 to -5.2)
Northern Ireland	Number: 1990	360 (342–378)	65 (58–74)	23 (21–26)	20 (17–24)	0 (0–1)	85 (76–94)	0 (0–0)	5 (2–7)	3 (3–4)	3 (2–3)	0 (0–0)
Northern Ireland	Number: 2015	190 (165–219)	37 (28–47)	10 (9–12)	6 (4–8)	2 (2–3)	49 (40–61)	0 (0–0)	3 (1–4)	1 (0–1)	3 (2–4)	0 (0–0)
Northern Ireland	Number: Cumulative change (%)	-47.2 (-54.5 to -38.4)	-43.4 (-57.4 to -27.2)	-53.9 (-62.8 to -43.5)	-67.5 (-77.1 to -55.6)	190.4 (111.6–285.2)	-41.5 (-53.7 to -26.4)	nan (nan–nan)	-35.1 (-59.8 to -0.7)	-71.9 (-77.2 to -66.2)	13.6 (-22.8 to 60.6)	-26.5 (-33.5 to -19.5)
Northern Ireland	Rate: 1990	72.5 (68.8–76.4)	14.0 (12.4–15.7)	4.8 (4.3–5.4)	4.4 (3.7–5.2)	0.2 (0.2–0.2)	17.8 (15.8–19.5)	0.0 (0.0–0.0)	1.1 (0.4–1.5)	0.8 (0.7–0.9)	0.7 (0.6–0.8)	0.0 (0.0–0.0)
Northern Ireland	Rate: 2015	41.2 (35.8–47.6)	8.1 (6.2–10.4)	2.4 (2.0–2.8)	1.5 (1.1–2.0)	0.6 (0.5–0.8)	10.9 (8.8–13.4)	0.0 (0.0–0.0)	0.7 (0.4–0.9)	0.2 (0.2–0.3)	0.8 (0.6–1.1)	0.0 (0.0–0.0)
Northern Ireland	Rate: Cumulative change (%)	-43.1 (-51.0 to -33.6)	-41.6 (-56.0 to -24.8)	-50.6 (-60.3 to -39.4)	-66.4 (-76.3 to -54.1)	207.5 (123.8–308.3)	-38.5 (-51.4 to -22.6)	nan (nan–nan)	-33.0 (-58.5 to 2.5)	-69.7 (-75.4 to -63.4)	17.5 (-20.2 to 66.3)	-18.8 (-26.4 to -11.0)
Scotland	Number: 1990	944 (918–968)	137 (123–152)	36 (32–39)	66 (58–75)	2 (2–3)	170 (157–182)	0 (0–0)	11 (4–15)	11 (10–13)	19 (16–23)	1 (1–1)
Scotland	Number: 2015	398 (363–439)	54 (43–66)	14 (12–17)	20 (15–25)	5 (4–6)	81 (69–104)	0 (0–0)	6 (3–9)	2 (2–3)	14 (10–18)	0 (0–0)
Scotland	Number: Cumulative change (%)	-57.8 (-61.6 to -53.5)	-60.6 (-69.2 to -51.3)	-59.3 (-66.1 to -51.4)	-69.7 (-77.1 to -60.9)	92.3 (45.5–145.9)	-51.7 (-60.2 to -38.7)	nan (nan–nan)	-39.0 (-60.2 to -7.6)	-77.1 (-80.8 to -73.2)	-26.6 (-48.3 to -2.1)	-38.8 (-44.5 to -33.0)
Scotland	Rate: 1990	75.1 (73.0–77.2)	11.7 (10.5–12.9)	2.9 (2.6–3.2)	5.7 (5.0–6.4)	0.2 (0.2–0.3)	14.1 (13.0–15.2)	0.0 (0.0–0.0)	1.0 (0.4–1.3)	0.9 (0.8–1.1)	1.7 (1.4–2.0)	0.1 (0.1–0.1)
Scotland	Rate: 2015	36.5 (33.1–40.5)	5.3 (4.3–6.5)	1.4 (1.2–1.6)	2.0 (1.5–2.5)	0.5 (0.4–0.6)	7.9 (6.6–10.0)	0.0 (0.0–0.0)	0.6 (0.4–0.9)	0.2 (0.2–0.3)	1.4 (1.0–1.8)	0.1 (0.1–0.1)
Scotland	Rate: Cumulative change (%)	-51.5 (-56.1 to -46.0)	-54.4 (-64.3 to -43.6)	-52.7 (-60.8 to -43.4)	-64.8 (-73.4 to -54.7)	121.7 (67.3–183.7)	-44.2 (-54.1 to -29.0)	nan (nan–nan)	-29.3 (-53.8 to 7.1)	-73.6 (-78.0 to -69.0)	-14.9 (-40.0 to 13.6)	-29.7 (-36.2 to -22.7)
Wales	Number: 1990	500 (485–516)	90 (83–99)	18 (16–20)	33 (29–38)	1 (1–1)	87 (80–96)	0 (0–0)	4 (2–5)	11 (9–13)	7 (6–8)	0 (0–0)
Wales	Number: 2015	232 (221–243)	44 (37–50)	8 (6–9)	8 (7–10)	1 (1–1)	48 (42–62)	0 (0–0)	2 (1–4)	2 (2–3)	4 (3–5)	0 (0–0)
Wales	Number: Cumulative change (%)	-53.6 (-56.0 to -50.9)	-50.8 (-58.2 to -43.5)	-56.1 (-63.2 to -48.1)	-74.0 (-79.1 to -67.3)	4.2 (-22.3 to 34.9)	-45.3 (-52.7 to -34.0)	nan (nan–nan)	-48.1 (-68.0 to -17.6)	-74.6 (-78.1 to -70.8)	-34.5 (-51.0 to -15.9)	-11.1 (-19.1 to -3.0)
Wales	Rate: 1990	69.1 (66.9–71.3)	13.0 (11.9–14.2)	2.6 (2.3–2.9)	4.8 (4.2–5.5)	0.2 (0.2–0.2)	12.4 (11.4–13.7)	0.0 (0.0–0.0)	0.6 (0.4–0.8)	1.6 (1.4–1.8)	1.0 (0.9–1.3)	0.1 (0.0–0.1)
Wales	Rate: 2015	35.1 (33.3–36.9)	7.2 (6.0–8.2)	1.2 (1.1–1.4)	1.4 (1.1–1.7)	0.2 (0.2–0.3)	7.6 (6.7–9.9)	0.0 (0.0–0.0)	0.3 (0.2–0.6)	0.4 (0.4–0.5)	0.8 (0.6–0.9)	0.0 (0.0–0.1)
Wales	Rate: Cumulative change (%)	-49.2 (-52.0 to -46.1)	-44.5 (-52.9 to -36.3)	-51.5 (-59.3 to -42.7)	-70.7 (-76.5 to -63.1)	16.0 (-13.9 to 50.5)	-38.8 (-47.3 to -26.1)	nan (nan–nan)	-41.5 (-64.0 to -7.1)	-72.2 (-76.0 to -67.9)	-26.3 (-44.8 to -5.3)	-2.5 (-11.2 to 6.5)
Southern Latin America	Number: 1990	33,064 (32,921–33,203)	7,143 (6,659–7,655)	3,231 (3,021–3,451)	1,866 (1,619–2,156)	981 (886–1,082)	5,072 (4,598–5,380)	2 (2–2)	1,171 (711–1,487)	668 (622–716)	1,078 (893–1,272)	94 (89–100)
Southern Latin America	Number: 2015	16,800 (16,447–17,165)	2,582 (2,331–2,837)	892 (803–986)	579 (499–660)	173 (149–199)	3,821 (3,469–4,121)	0 (0–1)	658 (493–903)	186 (169–208)	677 (582–778)	77 (72–81)
Southern Latin America	Number: Cumulative change (%)	-49.2 (-50.3 to -48.0)	-63.8 (-67.1 to -60.3)	-72.3 (-75.8 to -68.7)	-68.8 (-74.4 to -62.6)	-82.3 (-85.3 to -79.0)	-24.5 (-31.3 to -17.5)	-60.7 (-64.8 to -56.4)	-42.4 (-57.3 to -23.8)	-72.0 (-75.2 to -68.3)	-36.5 (-49.5 to -21.6)	-18.6 (-25.2 to -11.6)
Southern Latin America	Rate: 1990	172.3 (171.5–173.0)	37.0 (34.5–39.6)	16.7 (15.7–17.9)	9.7 (8.4–11.2)	5.1 (4.6–5.6)	26.3 (23.8–27.9)	0.0 (0.0–0.0)	6.1 (3.7–7.7)	3.5 (3.2–3.7)	5.6 (4.6–6.6)	0.5 (0.5–0.5)
Southern Latin America	Rate: 2015	85.5 (83.7–87.4)	13.6 (12.2–14.9)	4.6 (4.1–5.1)	3.0 (2.6–3.5)	0.9 (0.8–1.0)	19.9 (18.0–21.4)	0.0 (0.0–0.0)	3.5 (2.6–4.7)	1.0 (0.9–1.1)	3.6 (3.1–4.1)	0.4 (0.4–0.4)
Southern Latin America	Rate: Cumulative change (%)	-50.4 (-51.5 to -49.2)	-63.3 (-66.7 to -59.8)	-72.7 (-76.1 to -69.1)	-68.3 (-74.0 to -62.1)	-82.3 (-85.3 to -79.0)	-24.3 (-31.1 to -17.2)	-63.4 (-67.2 to -59.5)	-41.6 (-56.7 to -22.8)	-72.5 (-75.7 to -68.8)	-35.6 (-48.8 to -20.6)	-21.5 (-27.9 to -14.7)
Argentina	Number: 1990	24,416 (24,289–24,546)	5,536 (5,087–5,985)	2,064 (1,877–2,257)	1,389 (1,156–1,665)	794 (702–896)	3,401 (2,990–3,682)	2 (2–2)	952 (541–1,249)	482 (439–525)	921 (744–1,104)	83 (78–88)
Argentina	Number: 2015	13,297 (12,975–13,644)	2,174 (1,957–2,415)	736 (654–824)	478 (402–560)	149 (127–174)	2,883 (2,564–3,169)	0 (0–1)	556 (402–748)	153 (136–174)	548 (458–643)	64 (60–69)
Argentina	Number: Cumulative change (%)	-45.5 (-46.9 to -44.1)	-60.7 (-64.6 to -56.2)	-64.2 (-69.3 to -58.3)	-65.2 (-72.8 to -56.2)	-81.0 (-84.6 to -77.0)	-15.0 (-24.5 to -5.5)	-60.7 (-64.8 to -56.4)	-39.6 (-57.2 to -18.7)	-68.1 (-72.4 to -62.8)	-39.7 (-53.8 to -23.1)	-22.3 (-29.4 to -14.7)

© 2017 Global Burden of Disease Child and Adolescent Health Collaboration. JAMA Pediatrics.

eTable 1. Number of Deaths, Death Rates (per 100,000 population), and Cumulative Percent Change with 95% Uncertainty Intervals (UI) for the Top 10 Global Causes of Death in 195 Countries and Territories, Aged 0 to 19, Both Sexes, 1990 and 2015*Note: best viewed by enlarging in browser.*

Location	Measure	All causes	Neonatal preterm birth complications	Lower respiratory infections	Neonatal encephalopathy due to birth asphyxia and trauma	Diarrheal diseases	Congenital anomalies	Malaria	Neonatal sepsis and other neonatal infections	Meningitis	Other neonatal disorders	HIV/AIDS
Argentina	Rate: 1990	188.7 (187.8–189.7)	42.4 (39.0–45.9)	15.9 (14.4–17.4)	10.7 (8.9–12.8)	6.1 (5.4–6.9)	26.1 (23.0–28.3)	0.0 (0.0–0.0)	7.3 (4.2–9.6)	3.7 (3.4–4.1)	7.1 (5.7–8.5)	0.6 (0.6–0.7)
Argentina	Rate: 2015	94.4 (92.1–96.9)	15.7 (14.1–17.4)	5.2 (4.6–5.9)	3.5 (2.9–4.0)	1.1 (0.9–1.2)	20.7 (18.4–22.7)	0.0 (0.0–0.0)	4.0 (2.9–5.4)	1.1 (1.0–1.2)	4.0 (3.3–4.6)	0.5 (0.4–0.5)
Argentina	Rate: Cumulative change (%)	-50.0 (-51.2 to -48.6)	-62.9 (-66.6 to -58.8)	-67.0 (-71.7 to -61.5)	-67.2 (-74.4 to -58.8)	-82.4 (-85.8 to -78.7)	-20.6 (-29.6 to -11.7)	-65.0 (-68.7 to -61.2)	-43.2 (-59.7 to -23.5)	-70.6 (-74.6 to -65.8)	-43.2 (-56.5 to -27.6)	-29.3 (-35.8 to -22.4)
Chile	Number: 1990	6,978 (6,920–7,034)	1,318 (1,202–1,448)	1,015 (946–1,086)	335 (270–402)	126 (108–145)	1,367 (1,240–1,470)	0 (0–0)	157 (114–231)	160 (144–175)	100 (80–125)	9 (9–10)
Chile	Number: 2015	2,782 (2,689–2,884)	337 (294–379)	111 (97–128)	71 (58–86)	12 (10–14)	772 (703–837)	0 (0–0)	68 (44–120)	30 (26–34)	97 (78–115)	10 (9–10)
Chile	Number: Cumulative change (%)	-60.1 (-61.6 to -58.6)	-74.3 (-78.2 to -70.8)	-89.0 (-90.6 to -87.1)	-78.4 (-83.5 to -72.3)	-90.1 (-92.0 to -87.9)	-43.4 (-49.2 to -36.9)	nan (nan–nan)	-56.7 (-70.7 to -38.6)	-81.0 (-83.6 to -77.9)	-2.1 (-27.9 to 27.3)	1.8 (-9.6 to 13.5)
Chile	Rate: 1990	134.1 (133.0–135.2)	25.2 (23.0–27.7)	19.4 (18.0–20.7)	6.4 (5.2–7.7)	2.4 (2.1–2.8)	26.1 (23.7–28.1)	0.0 (0.0–0.0)	3.0 (2.2–4.4)	3.1 (2.8–3.4)	1.9 (1.5–2.4)	0.2 (0.2–0.2)
Chile	Rate: 2015	59.9 (57.7–62.1)	7.8 (6.8–8.8)	2.5 (2.2–2.9)	1.7 (1.4–2.0)	0.3 (0.2–0.3)	17.6 (16.0–19.1)	0.0 (0.0–0.0)	1.6 (1.0–2.8)	0.7 (0.6–0.7)	2.2 (1.8–2.7)	0.2 (0.2–0.2)
Chile	Rate: Cumulative change (%)	-55.4 (-57.0 to -53.6)	-68.9 (-73.5 to -64.6)	-87.1 (-89.0 to -84.8)	-73.8 (-80.1 to -66.4)	-88.4 (-90.6 to -85.8)	-32.4 (-39.5 to -24.7)	nan (nan–nan)	-47.5 (-64.5 to -25.5)	-78.3 (-81.4 to -74.7)	18.7 (-12.7 to 54.4)	13.4 (0.8–27.2)
Uruguay	Number: 1990	1,668 (1,623–1,716)	288 (260–316)	151 (135–167)	141 (120–162)	60 (51–70)	302 (278–337)	0 (0–0)	61 (39–78)	25 (22–28)	55 (45–66)	1 (1–1)
Uruguay	Number: 2015	720 (625–836)	70 (53–90)	43 (34–53)	29 (21–38)	11 (8–14)	164 (130–211)	0 (0–0)	33 (22–45)	2 (2–3)	32 (23–42)	2 (2–2)
Uruguay	Number: Cumulative change (%)	-56.8 (-62.7 to -49.8)	-75.6 (-81.5 to -68.4)	-71.2 (-77.6 to -63.4)	-79.1 (-84.8 to -71.6)	-81.5 (-86.7 to -75.1)	-45.6 (-57.6 to -31.7)	nan (nan–nan)	-43.8 (-58.7 to -25.9)	-89.0 (-91.6 to -86.2)	-41.0 (-58.7 to -19.3)	51.9 (33.7–70.4)
Uruguay	Rate: 1990	158.6 (154.3–163.2)	27.7 (25.0–30.3)	14.5 (13.0–16.1)	13.5 (11.6–15.6)	5.8 (4.9–6.7)	29.0 (26.7–32.3)	0.0 (0.0–0.0)	5.9 (3.8–7.5)	2.4 (2.2–2.7)	5.4 (4.3–6.4)	0.1 (0.1–0.2)
Uruguay	Rate: 2015	75.9 (65.5–88.7)	7.9 (6.0–10.1)	4.7 (3.7–5.8)	3.3 (2.4–4.4)	1.2 (0.9–1.6)	18.1 (14.3–23.3)	0.0 (0.0–0.0)	3.8 (2.5–5.1)	0.3 (0.2–0.4)	3.6 (2.7–4.8)	0.2 (0.2–0.3)
Uruguay	Rate: Cumulative change (%)	-52.1 (-59.0 to -44.0)	-71.5 (-78.4 to -63.1)	-67.5 (-74.9 to -58.5)	-75.6 (-82.3 to -66.9)	-78.8 (-84.8 to -71.4)	-37.5 (-51.4 to -21.4)	nan (nan–nan)	-34.4 (-51.8 to -13.6)	-87.7 (-90.6 to -84.6)	-31.2 (-51.8 to -5.9)	65.7 (45.6–86.0)
Central Europe, Eastern Europe, and Central Asia	Number: 1990	286,252 (281,846–290,586)	31,374 (29,169–33,573)	69,134 (66,795–71,460)	24,528 (21,903–27,024)	16,202 (15,345–17,140)	32,939 (28,987–34,647)	12 (9–15)	3,904 (3,145–5,600)	5,545 (5,159–5,893)	9,279 (8,143–10,642)	354 (338–372)
Central Europe, Eastern Europe, and Central Asia	Number: 2015	104,867 (100,423–109,703)	11,384 (10,175–12,661)	17,857 (16,116–19,690)	10,602 (9,246–12,012)	2,048 (1,704–2,431)	16,070 (14,766–17,325)	3 (1–5)	2,725 (2,167–3,470)	1,322 (1,183–1,542)	4,538 (3,856–5,380)	606 (551–665)
Central Europe, Eastern Europe, and Central Asia	Number: Cumulative change (%)	-63.4 (-65.0 to -61.6)	-63.7 (-67.7 to -59.4)	-74.2 (-77.0 to -71.4)	-56.7 (-62.7 to -49.9)	-87.3 (-89.6 to -84.8)	-51.1 (-56.1 to -41.9)	-73.4 (-85.4 to -55.5)	-29.2 (-43.7 to -12.9)	-76.1 (-79.1 to -71.1)	-50.8 (-60.2 to -39.2)	71.1 (54.2–89.5)
Central Europe, Eastern Europe, and Central Asia	Rate: 1990	224.0 (220.4–227.5)	25.6 (23.8–27.4)	54.3 (52.5–56.1)	20.0 (17.9–22.1)	12.8 (12.1–13.5)	26.2 (23.1–27.6)	0.0 (0.0–0.0)	3.2 (2.6–4.6)	4.3 (4.0–4.6)	7.6 (6.6–8.7)	0.3 (0.3–0.3)
Central Europe, Eastern Europe, and Central Asia	Rate: 2015	104.8 (100.5–109.5)	11.1 (9.9–12.3)	17.4 (15.8–19.2)	10.3 (9.0–11.7)	2.0 (1.6–2.3)	15.7 (14.4–16.9)	0.0 (0.0–0.0)	2.6 (2.1–3.4)	1.3 (1.2–1.5)	4.4 (3.7–5.2)	0.6 (0.5–0.7)
Central Europe, Eastern Europe, and Central Asia	Rate: Cumulative change (%)	-53.2 (-55.3 to -51.0)	-56.7 (-61.5 to -51.6)	-67.9 (-71.3 to -64.5)	-48.3 (-55.5 to -40.2)	-84.5 (-87.2 to -81.4)	-40.0 (-46.2 to -28.8)	-62.3 (-79.5 to -37.1)	-15.5 (-32.8 to 3.8)	-69.3 (-73.1 to -62.8)	-41.3 (-52.5 to -27.5)	118.1 (96.6–141.3)
Eastern Europe	Number: 1990	97,965 (95,787–100,083)	9,661 (8,581–10,595)	9,579 (8,978–10,295)	8,884 (8,003–9,783)	1,901 (1,704–2,124)	16,902 (13,578–18,162)	0 (0–0)	1,805 (1,274–3,380)	2,274 (2,107–2,470)	3,293 (2,901–3,738)	199 (188–211)
Eastern Europe	Number: 2015	32,817 (31,264–34,668)	2,390 (1,909–2,803)	2,112 (1,906–2,328)	2,082 (1,729–2,442)	199 (171–231)	6,781 (6,002–8,238)	0 (0–0)	1,318 (983–1,889)	429 (384–481)	1,584 (1,283–1,862)	532 (481–587)
Eastern Europe	Number: Cumulative change (%)	-66.5 (-68.3 to -64.6)	-75.2 (-79.9 to -71.1)	-77.9 (-80.8 to -75.2)	-76.5 (-80.6 to -71.9)	-89.4 (-91.3 to -87.5)	-59.4 (-65.3 to -38.9)	nan (nan–nan)	-22.4 (-48.7 to 0.3)	-81.1 (-83.4 to -78.4)	-51.7 (-61.1 to -42.5)	166.7 (137.1–199.1)
Eastern Europe	Rate: 1990	164.7 (160.9–168.5)	17.7 (15.7–19.4)	16.5 (15.4–17.7)	16.3 (14.7–18.0)	3.3 (3.0–3.7)	29.6 (23.7–31.9)	0.0 (0.0–0.0)	3.3 (2.3–6.2)	3.8 (3.5–4.2)	6.0 (5.3–6.8)	0.3 (0.3–0.4)
Eastern Europe	Rate: 2015	76.0 (72.6–80.0)	5.1 (4.1–6.0)	4.7 (4.2–5.1)	4.5 (3.7–5.3)	0.4 (0.4–0.5)	14.8 (13.1–17.9)	0.0 (0.0–0.0)	2.8 (2.1–4.1)	0.9 (0.8–1.1)	3.4 (2.8–4.0)	1.2 (1.1–1.3)
Eastern Europe	Rate: Cumulative change (%)	-53.8 (-56.1 to -51.3)	-70.9 (-76.5 to -66.0)	-71.7 (-75.4 to -68.3)	-72.4 (-77.2 to -67.0)	-87.0 (-89.2 to -84.6)	-49.5 (-56.9 to -23.8)	nan (nan–nan)	-9.0 (-39.9 to 17.6)	-75.3 (-78.3 to -71.8)	-43.3 (-54.4 to -32.4)	244.1 (206.1–285.0)
Belarus	Number: 1990	4,372 (3,965–4,840)	359 (246–494)	449 (376–531)	358 (239–493)	53 (42–67)	845 (614–1,022)	0 (0–0)	86 (41–160)	91 (77–109)	198 (100–333)	0 (0–1)
Belarus	Number: 2015	1,073 (943–1,249)	48 (29–73)	32 (25–41)	68 (40–103)	1 (1–2)	266 (194–358)	0 (0–0)	34 (17–58)	13 (10–17)	40 (19–67)	1 (0–4)
Belarus	Number: Cumulative change (%)	-75.4 (-79.0 to -71.0)	-85.8 (-92.9 to -75.7)	-92.7 (-94.8 to -90.2)	-79.9 (-89.6 to -66.7)	-96.9 (-98.0 to -95.4)	-67.4 (-78.6 to -46.2)	nan (nan–nan)	-53.8 (-82.2 to -6.0)	-85.2 (-89.6 to -79.5)	-76.4 (-92.0 to -50.3)	130.6 (13.4–267.5)
Belarus	Rate: 1990	157.2 (141.9–174.5)	13.9 (9.5–19.1)	16.6 (13.9–19.7)	13.9 (9.3–19.1)	2.0 (1.6–2.5)	31.6 (22.8–38.3)	0.0 (0.0–0.0)	3.3 (1.6–6.2)	3.3 (2.8–4.0)	7.7 (3.9–12.9)	0.0 (0.0–0.1)
Belarus	Rate: 2015	54.9 (48.5–63.4)	2.4 (1.4–3.6)	1.6 (1.3–2.1)	3.3 (2.0–5.0)	0.1 (0.1–0.1)	13.1 (9.7–17.6)	0.0 (0.0–0.0)	1.7 (0.8–2.9)	0.7 (0.5–0.9)	1.9 (1.0–3.3)	0.1 (0.0–0.2)
Belarus	Rate: Cumulative change (%)	-65.0 (-70.2 to -58.7)	-82.2 (-91.1 to -69.4)	-90.1 (-92.9 to -86.8)	-74.8 (-87.0 to -58.1)	-95.9 (-97.4 to -94.0)	-56.9 (-71.7 to -28.9)	nan (nan–nan)	-42.0 (-77.7 to 18.0)	-79.8 (-85.7 to -72.1)	-70.3 (-89.9 to -37.4)	254.5 (71.7–469.6)
Estonia	Number: 1990	607 (586–627)	27 (21–34)	39 (35–44)	31 (25–38)	4 (4–5)	99 (90–108)	0 (0–0)	14 (7–19)	10 (9–12)	58 (48–68)	0 (0–0)
Estonia	Number: 2015	81 (71–93)	3 (2–5)	3 (2–4)	3 (2–5)	0 (0–0)	19 (14–29)	0 (0–0)	3 (2–4)	0 (0–0)	2 (1–3)	0 (0–0)
Estonia	Number: Cumulative change (%)	-86.5 (-88.3 to -84.5)	-86.7 (-92.7 to -79.6)	-90.5 (-92.9 to -88.0)	-87.6 (-92.3 to -82.6)	-95.7 (-97.0 to -94.1)	-80.5 (-85.6 to -71.3)	nan (nan–nan)	-77.4 (-86.6 to -64.4)	-93.5 (-95.0 to -91.6)	-95.9 (-97.4 to -94.0)	931.2 (743.1–1,137.9)
Estonia	Rate: 1990	144.6 (139.5–149.8)	7.2 (5.7–9.0)	9.8 (8.7–10.9)	8.3 (6.7–10.0)	1.2 (1.0–1.5)	24.6 (22.3–26.9)	0.0 (0.0–0.0)	3.8 (2.0–5.2)	2.6 (2.3–2.9)	15.2 (12.8–18.0)	0.0 (0.0–0.0)
Estonia	Rate: 2015	32.5 (28.6–37.0)	1.4 (0.8–1.9)	1.4 (1.1–1.8)	1.5 (1.0–2.0)	0.1 (0.1–0.1)	7.4 (5.5–11.1)	0.0 (0.0–0.0)	1.2 (0.8–1.7)	0.3 (0.2–0.3)	0.9 (0.6–1.3)	0.2 (0.2–0.2)
Estonia	Rate: Cumulative change (%)	-77.5 (-80.4 to -74.3)	-80.4 (-89.3 to -70.0)	-85.2 (-88.8 to -81.3)	-81.7 (-88.6 to -74.3)	-93.4 (-95.4 to -91.0)	-70.0 (-77.9 to -56.1)	nan (nan–nan)	-66.8 (-80.2 to -47.6)	-89.7 (-92.0 to -86.8)	-93.9 (-96.1 to -91.2)	1,758.6 (1,409.9–2,140.7)
Latvia	Number: 1990	1,109 (1,075–1,145)	41 (32–50)	54 (48–61)	86 (72–100)	7 (6–8)	219 (202–237)	0 (0–0)	10 (6–14)	24 (21–26)	78 (65–92)	0 (0–0)
Latvia	Number: 2015	172 (142–208)	4 (2–6)	6 (5–8)	16 (10–23)	0 (0–0)	42 (29–63)	0 (0–0)	3 (1–5)	1 (0–1)	6 (3–9)	0 (0–0)
Latvia	Number: Cumulative change (%)	-84.5 (-87.1 to -81.2)	-89.7 (-94.5 to -84.2)	-87.6 (-91.0 to -83.5)	-81.0 (-88.1 to -71.8)	-94.7 (-96.4 to -92.5)	-80.7 (-86.8 to -70.5)	nan (nan–nan)	-66.6 (-80.0 to -49.7)	-95.2 (-96.5 to -93.4)	-91.9 (-95.1 to -87.9)	21.5 (-2.5 to 48.8)
Latvia	Rate: 1990	156.4 (151.5–161.6)	6.3 (5.0–7.7)	7.9 (7.0–8.8)	13.1 (11.0–15.2)	1.1 (0.9–1.3)	32.0 (29.5–34.7)	0.0 (0.0–0.0)	1.6 (1.0–2.2)	3.5 (3.1–3.8)	11.9 (10.0–14.0)	0.1 (0.1–0.1)
Latvia	Rate: 2015	45.4 (37.5–55.1)	1.1 (0.7–1.7)	1.8 (1.3–2.3)	4.5 (2.8–6.4)	0.1 (0.1–0.1)	11.3 (7.9–16.9)	0.0 (0.0–0.0)	0.9 (0.5–1.4)	0.3 (0.2–0.4)	1.7 (1.0–2.5)	0.2 (0.2–0.2)
Latvia	Rate: Cumulative change (%)	-71.0 (-76.0 to -64.8)	-81.5 (-90.1 to -71.6)	-77.1 (-83.4 to -69.6)	-65.7 (-78.7 to -49.2)	-90.2 (-93.3 to -86.2)	-64.7 (-75.9 to -46.2)	nan (nan–nan)	-39.9 (-64.0 to -9.4)	-91.1 (-93.5 to -87.8)	-85.4 (-91.3 to -78.3)	127.9 (81.5–180.1)
Lithuania	Number: 1990	1,260 (1,230–1,289)	98 (84–112)	58 (51–65)	84 (71–97)	9 (7–11)	304 (271–328)	0 (0–0)	9 (5–19)	23 (20–25)	36 (29–43)	0 (0–0)
Lithuania	Number: 2015	263 (247–284)	10 (7–13)	12 (9–14)	9 (6–11)	0 (0–0)	56 (45–80)	0 (0–0)	6 (3–8)	2 (1–2)	5 (3–6)	0 (0–0)
Lithuania	Number: Cumulative change (%)	-79.1 (-80.5 to -77.4)	-89.4 (-93.0 to -86.2)	-78.8 (-84.3 to -74.3)	-89.0 (-92.2 to -85.6)	-92.4 (-94.8 to -89.8)	-81.4 (-85.1 to -70.4)	nan (nan–nan)	-22.1 (-75.4 to 32.4)	-90.6 (-92.7 to -88.4)	-85.6 (-90.2 to -80.2)	32.7 (8.3–59.0)
Lithuania	Rate: 1990	118.8 (115.9–121.8)	9.8 (8.4–11.2)	5.6 (5.0–6.3)	8.4 (7.2–9.7)	0.9 (0.7–1.1)	29.7 (26.4–32.1)	0.0 (0.0–0.0)	0.9 (0.6–1.9)	2.2 (2.0–2.5)	3.6 (2.9–4.3)	0.0 (0.0–0.1)
Lithuania	Rate: 2015	42.0 (39.1–45.6)	1.9 (1.3–2.4)	2.1 (1.6–2.5)	1.7 (1.2–2.1)	0.1 (0.1–0.2)	9.9 (8.0–14.2)	0.0 (0.0–0.0)	1.1 (0.6–1.5)	0.4 (0.3–0.4)	0.9 (0.7–1.2)	0.1 (0.1–0.1)
Lithuania	Rate: Cumulative change (%)	-64.7 (-67.3 to -61.6)	-80.7 (-87.2 to -74.8)	-62.5 (-72.6 to -54.5)	-80.0 (-85.8 to -73.7)	-86.2 (-90.6 to -81.4)	-66.3 (-73.1 to -46.4)	nan (nan–nan)	41.7 (-55.3 to 141.0)	-83.2 (-87.1 to -79.4)	-73.8 (-82.2 to -63.9)	124.6 (82.7–167.8)
Moldova	Number: 1990	3,306 (2,984–3,666)	212 (159–279)	541 (457–633)	406 (319–520)	91 (72–113)	541 (291–650)	0 (0–0)	119 (37–193)	72 (61–85)	49 (34–70)	0 (0–1)
Moldova	Number: 2015	657 (519–828)	45 (30–65)	79 (56–108)	43 (28–67)	6 (4–8)	154 (106–213)	0 (0–0)	24 (11–40)	6 (5–9)	23 (15–35)	0 (0–1)
Moldova	Number: Cumulative change (%)	-80.0 (-84.7 to -74.2)	-78.1 (-86.8 to -66.9)	-85.1 (-89.9 to -79.2)	-89.0 (-93.1 to -83.2)	-93.0 (-95.7 to -89.6)	-70.6 (-80.5 to -55.4)	nan (nan–nan)	-76.6 (-87.4 to -59.4)	-90.4 (-93.2 to -86.8)	-50.0 (-71.2 to -20.0)	5.7 (-13.0 to 26.4)
Moldova	Rate: 1990	217.4 (195.7–241.2)	14.4 (10.8–18.9)	35.4 (29.8–41.5)	27.6 (21.7–35.3)	6.0 (4.8–7.4)	35.9 (19.4–43.1)	0.0 (0.0–0.0)	8.1 (2.5–13.2)	4.7 (3.9–5.5)	3.4 (2.4–4.8)	0.1 (0.1–0.1)
Moldova	Rate: 2015	78.9 (61.8–100.3)	5.7 (3.8–8.3)	9.8 (6.9–13.4)	5.6 (3.6–8.5)	0.8 (0.5–1.1)	19.1 (13.2–26.5)	0.0 (0.0–0.0)	3.1 (1.5–5.1)	0.8 (0.6–1.1)	3.0 (2.0–4.5)	0.1 (0.1–0.1)
Moldova	Rate: Cumulative change (%)	-63.5 (-72.2 to -52.5)	-59.0 (-75.3 to -38.1)	-72.0 (-81.1 to -60.7)	-79.5 (-87.1 to -68.6)	-86.8 (-92.0 to -80.3)	-45.1 (-63.7 to -16.5)	nan (nan–nan)	-56.3 (-76.5 to -24.2)	-82.0 (-87.4 to -75.2)	-6.5 (-46.1 to 49.6)	90.5 (56.1–128.8)
Russia	Number: 1990	66,315 (66,015–66,649)	7,070 (6,141–7,920)	6,518 (6,057–7,051)	6,129 (5,364–6,925)	1,426 (1,243–1,622)	10,802 (8,811–11,649)	0 (0–0)	1,219 (722–2,679)	1,523 (1,379–1,687)	2,160 (1,827–2,526)	168 (157–180)
Russia	Number: 2015	23,666 (23,346–24,011)	1,721 (1,369–1,984)	1,736 (1,558–1,922)	1,409 (1,150–1,645)	161 (136–190)	4,669 (4,227–5,974)	0 (0–0)	995 (738–1,535)	303 (271–339)	1,196 (962–1,416)	476 (426–530)
Russia	Number: Cumulative change (%)	-64.3 (-64.8 to -63.8)	-75.6 (-79.8 to -71.5)	-73.3 (-77.0 to -69.8)	-76.9 (-81.2 to -72.9)	-88.6 (-90.8 to -86.2)	-56.3 (-62.1 to -32.4)	nan (nan–nan)	-6.8 (-47.8 to 29.7)	-80.0 (-82.8 to -77.1)	-44.2 (-56.2 to -32.3)	183.6 (148.3–221.2)
Russia	Rate: 1990	165.3 (164.8–165.9)	19.2 (16.7–21.5)	16.5 (15.4–17.9)	16.7 (14.6–18.8)	3.6 (3.2–4.2)	27.9 (22.8–30.2)	0.0 (0.0–0.0)	3.3 (2.0–7.3)	3.8 (3.4–4.2)	5.9 (5.0–6.9)	0.4 (0.4–0.5)
Russia	Rate: 2015	78.1 (77.0–79.1)	5.1 (4.1–5.9)	5.3 (4.8–5.9)	4.2 (3.4–4.9)	0.5 (0.4–0.6)	14.1 (12.8–18.1)	0.0 (0.0–0.0)	3.0 (2.2–4.6)	0.9 (0.8–1.0)	3.6 (2.9–4.2)	1.4 (1.3–1.6)
Russia	Rate: Cumulative change (%)	-52.8 (-53.4 to -52.1)	-73.2 (-77.8 to -68.7)	-67.6 (-72.0 to -63.4)	-74.7 (-79.4 to -70.3)	-86.7 (-89.3 to -83.9)	-48.8 (-55.7 to -20.9)	nan (nan–nan)	2.2 (-42.7 to 42.3)	-75.2 (-78.6 to -71.6)	-38.8 (-51.9 to -25.6)	242.3 (200.4–286.6)
Ukraine	Number: 1990	20,995 (18,993–23,104)	1,851 (1,493–2,225)	1,916 (1,619–2,264)	1,787 (1,435–2,189)	308 (249–382)	4,088 (3,070–4,805)	0 (0–0)	346 (239–494)	528 (451–618)	712 (559–880)	28 (25–31)
Ukraine	Number: 2015	6,901 (5,458–8,704)	557 (357–826)	241 (174–321)	531 (327–798)	29 (18–42)	1,574 (1,106–2,156)	0 (0–0)	251 (143–377)	102 (73–139)	310 (190–465)	50 (44–57)
Ukraine	Number: Cumulative change (%)	-67.0 (-74.4 to -58.1)	-69.5 (-81.1 to -54.2)	-87.3 (-91.2 to -82.5)	-69.9 (-82.2 to -54.0)	-90.4 (-94.1 to -85.2)	-60.7 (-74.1 to -40.3)	nan (nan–nan)	-26.1 (-54.0 to 11.8)	-80.5 (-87.0 to -72.7)	-55.7 (-72.9 to -33.4)	78.4 (50.6–108.2)
Ukraine	Rate: 1990	163.3 (146.5–181.1)	15.9 (12.8–19.1)	15.6 (13.1–18.4)	15.4 (12.3–18.8)	2.5 (2.1–3.2)	33.8 (25.3–39.9)	0.0 (0.0–0.0)	3.0 (2.1–4.2)	4.2 (3.6–4.9)	6.1 (4.8–7.6)	0.2 (0.2–0.2)
Ukraine	Rate: 2015	79.5 (63.3–99.7)	6.2 (4.0–9.3)	2.7 (2.0–3.6)	6.0 (3.7–9.0)	0.3 (0.2–0.5)	17.8 (12.6–24.2)	0.0 (0.0–0.0)	2.8 (1.6–4.2)	1.1 (0.8–1.6)	3.5 (2.1–5.2)	0.6 (0.5–0.7)
Ukraine	Rate: Cumulative change (%)	-51.1 (-62.0 to -37.6)	-60.2 (-75.4 to -40.3)	-82.2 (-87.5 to -75.4)	-60.7 (-76.8 to -39.9)	-87.0 (-92.1 to -80.0)	-46.3 (-64.6 to -18.6)	nan (nan–nan)	-3.6 (-40.0 to 45.9)	-72.6 (-81.5 to -61.6)	-42.2 (-64.7 to -13.0)	162.5 (121.8–207.1)
Central Europe	Number: 1990	51,452 (50,848–52,074)	8,394 (7,789–9,148)	8,268 (7,832–8,677)	2,840 (2,595–3,088)	856 (779–939)	7,929 (6,887–8,565)	0 (0–0)	574 (357–724)	929 (851–996)	2,772 (2,476–3,182)	136 (127–147)
Central Europe	Number: 2015	10,849 (10,225–11,589)	1,330 (1,131–1,528)	955 (845–1,086)	423 (373–475)	71 (61–82)	2,323 (2,083–2,664)	0 (0–0)	164 (128–232)	98 (89–110)	532 (462–605)	25 (23–28)
Central Europe	Number: Cumulative change (%)	-78.9 (-80.1 to -77.4)	-84.1 (-87.2 to -81.0)	-88.4 (-90.0 to -86.7)	-85.1 (-87.2 to -82.7)	-91.7 (-93.1 to -90.1)	-70.5 (-75.0 to -62.1)	nan (nan–nan)	-70.4 (-79.3 to -56.3)	-89.3 (-90.7 to -87.4)	-80.7 (-84.5 to -77.1)	-81.1 (-83.1 to -78.6)
Central Europe	Rate: 1990	150.4 (148.5–152.3)	26.4 (24.5–28.8)	24.8 (23.4–26.0)	8.9 (8.2–9.7)	2.6 (2.4–2.9)	24.2 (20.9–26.1)	0.0 (0.0–0.0)	1.8 (1.1–2.3)	2.7 (2.5–2.9)	8.7 (7.8–10.0)	0.4 (0.4–0.4)
Central Europe	Rate: 2015	48.1 (45.1–51.5)	6.3 (5.4–7.2)	4.3 (3.8–5.0)	2.0 (1.8–2.3)	0.3 (0.3–0.4)	10.8 (9.6–12.4)	0.0 (0.0–0.0)	0.8 (0.6–1.1)	0.4 (0.4–0.5)	2.5 (2.2–2.9)	0.1 (0.1–0.1)
Central Europe	Rate: Cumulative change (%)	-68.0 (-70.0 to -65.7)	-76.0 (-80.7 to -71.3)	-82.4 (-84.8 to -79.8)	-77.5 (-80.7 to -73.9)	-87.4 (-89.5 to -84.9)	-55.1 (-61.9 to -42.0)	nan (nan–nan)	-55.4 (-68.9 to -34.2)	-83.7 (-85.8 to -80.7)	-70.9 (-76.7 to -65.6)	-71.8 (-74.7 to -68.1)
Albania	Number: 1990	3,899 (3,610–4,218)	171 (137–202)	1,544 (1,336–1,742)	69 (54–84)	90 (70–112)	244 (176–408)	0 (0–0)	5 (3–9)	93 (74–111)	156 (132–183)	0 (0–0)
Albania	Number: 2015	688 (561–850)	23 (15–35)	94 (69–124)	11 (7–18)	2 (1–3)	127 (83–179)	0 (0–0)	2 (1–3)	11 (7–19)	56 (38–81)	0 (0–0)
Albania	Number: Cumulative change (%)	-82.3 (-85.9 to -77.9)	-86.1 (-91.3 to -78.4)	-93.9 (-95.6 to -91.7)	-82.8 (-89.9 to -72.3)	-97.2 (-98.4 to -95.7)	-44.6 (-74.7 to -19.9)	nan (nan–nan)	-57.2 (-74.1 to -32.7)	-87.4 (-92.5 to -78.1)	-63.9 (-76.5 to -46.5)	inf (-77.5 to 346.3)
Albania	Rate: 1990	270.0 (250.3–292.0)	11.8 (9.5–13.9)	105.9 (91.7–119.4)	4.8 (3.8–5.9)	6.2 (4.8–7.7)	16.9 (12.2–28.1)	0.0 (0.0–0.0)	0.4 (0.2–0.6)	6.4 (5.1–7.7)	10.8 (9.1–12.6)	0.0 (0.0–0.0)
Albania	Rate: 2015	89.9 (72.3–112.7)	3.2 (2.1–4.9)	13.0 (9.5–17.1)	1.6 (1.0–2.5)	0.3 (0.2–0.5)	17.6 (11.5–24.8)	0.0 (0.0–0.0)	0.3 (0.2–0.5)	1.6 (1.0–2.7)	7.7 (5.3–11.2)	0.0 (0.0–0.0)
Albania	Rate: Cumulative change (%)	-66.6 (-73.7 to -57.9)	-72.3 (-82.7 to -56.9)	-87.7 (-91.3 to -83.3)	-65.6 (-79.7 to -44.7)	-94.4 (-96.7 to -91.4)	10.6 (-49.1 to 59.6)	nan (nan–nan)	-14.8 (-48.4 to 34.0)	-75.2 (-85.3 to -56.2)	-28.0 (-53.2 to 6.6)	inf (-59.3 to 709.3)
Bosnia and Herzegovina	Number: 1990	1,883 (1,804–1,957)	360 (301–417)	118 (92–141)	95 (57–145)	42 (29–57)	215 (171–252)	0 (0–0)	29 (14–50)	28 (20–38)	55 (27–95)	0 (0–0)
Bosnia and Herzegovina	Number: 2015	320 (295–347)	39 (30–49)	11 (9–14)	16 (11–23)	2 (1–3)	64 (52–76)	0 (0–0)	8 (4–13)	3 (1–4)	9 (5–15)	0 (0–0)
Bosnia and Herzegovina	Number: Cumulative change (%)	-83.0 (-84.6 to -81.4)	-88.9 (-91.8 to -85.3)	-90.1 (-92.7 to -86.5)	-81.2 (-89.8 to -68.6)	-94.8 (-97.4 to -91.4)	-69.7 (-77.8 to -59.6)	nan (nan–nan)	-68.2 (-87.5 to -40.8)	-88.8 (-94.2 to -81.5)	-79.2 (-92.0 to -58.2)	inf (-0.1 to 219.9)
Bosnia and Herzegovina	Rate: 1990	142.2 (136.7–147.5)	30.6 (25.6–35.4)	9.1 (7.1–11.0)	8.1 (4.8–12.3)	3.5 (2.4–4.7)	17.4 (13.8–20.4)	0.0 (0.0–0.0)	2.5 (1.2–4.3)	2.2 (1.5–3.0)	4.7 (2.3–8.1)	0.0 (0.0–0.0)
Bosnia and Herzegovina	Rate: 2015	45.7 (41.8–49.8)	6.4 (4.9–8.0)	1.7 (1.3–2.1)	2.7 (1.8–3.7)	0.3 (0.2–0.5)	10.0 (8.1–12.0)	0.0 (0.0–0.0)	1.3 (0.7–2.2)	0.5 (0.3–0.7)	1.6 (0.9–2.5)	0.0 (0.0–0.0)
Bosnia and Herzegovina	Rate: Cumulative change (%)	-67.9 (-71.0 to -64.7)	-78.8 (-84.4 to -72.1)	-81.1 (-86.3 to -74.0)	-64.2 (-80.6 to -40.2)	-90.0 (-94.9 to -83.3)	-41.5 (-57.2 to -21.6)	nan (nan–nan)	-39.5 (-76.1 to 12.7)	-78.2 (-89.0 to -63.1)	-60.4 (-84.7 to -20.4)	inf (80.3–491.7)
Bulgaria	Number: 1990	2,908 (2,845–2,973)	255 (222–292)	535 (496–607)	96 (78–117)	29 (24–35)	543 (328–605)	0 (0–0)	13 (9–23)	45 (41–51)	231 (197–276)	2 (1–2)
Bulgaria	Number: 2015	858 (707–1,054)	98 (71–128)	98 (73–129)	45 (31–62)	8 (5–11)	208 (155–273)	0 (0–0)	10 (5–15)	10 (8–13)	30 (21–43)	2 (1–2)
Bulgaria	Number: Cumulative change (%)	-70.5 (-75.8 to -63.9)	-61.3 (-72.8 to -48.4)	-81.6 (-86.2 to -75.6)	-52.6 (-68.5 to -31.4)	-71.7 (-80.7 to -59.0)	-60.8 (-71.8 to -44.8)	nan (nan–nan)	-17.2 (-58.1 to 34.1)	-76.3 (-82.0 to -69.2)	-86.6 (-91.1 to -80.8)	-6.8 (-20.6 to 8.3)
Bulgaria	Rate: 1990	135.5 (132.3–138.9)	13.3 (11.5–15.2)	25.8 (23.9–29.4)	5.0 (4.1–6.1)	1.5 (1.2–1.8)	27.1 (16.3–30.2)	0.0 (0.0–0.0)	0.7 (0.5–1.2)	2.2 (1.9–2.4)	12.1 (10.3–14.4)	0.1 (0.1–0.1)
Bulgaria	Rate: 2015	68.6 (56.7–84.1)	7.9 (5.7–10.3)	7.8 (5.8–10.2)	3.6 (2.6–5.0)	0.7 (0.5–0.9)	16.6 (12.3–21.6)	0.0 (0.0–0.0)	0.9 (0.5–1.2)	0.9 (0.7–1.1)	2.5 (1.7–3.5)	0.2 (0.1–0.2)
Bulgaria	Rate: Cumulative change (%)	-49.4 (-58.5 to -38.0)	-40.2 (-57.9 to -20.2)	-69.9 (-77.4 to -60.1)	-26.7 (-51.3 to 6.1)	-54.8 (-69.2 to -34.4)	-37.6 (-55.0 to -12.1)	nan (nan–nan)	28.3 (-35.0 to 107.5)	-60.3 (-69.8 to -48.3)	-79.3 (-86.3 to -70.4)	71.1 (45.5–99.0)
Croatia	Number: 1990	1,023 (998–1,047)	143 (128–159)	39 (35–43)	70 (61–81)	6 (5–7)	187 (168–201)	0 (0–0)	5 (2–13)	12 (10–13)	94 (82–108)	0 (0–0)
Croatia	Number: 2015	304 (284–324)	24 (19–29)	6 (5–7)	20 (16–24)	1 (0–1)	69 (60–81)	0 (0–0)	8 (4–11)	2 (1–2)	21 (17–26)	0 (0–0)

© 2017 Global Burden of Disease Child and Adolescent Health Collaboration. JAMA Pediatrics.

eTable 1. Number of Deaths, Death Rates (per 100,000 population), and Cumulative Percent Change with 95% Uncertainty Intervals (UI) for the Top 10 Global Causes of Death in 195 Countries and Territories, Aged 0 to 19, Both Sexes, 1990 and 2015*Note: best viewed by enlarging in browser.*

Location	Measure	All causes	Neonatal preterm birth complications	Lower respiratory infections	Neonatal encephalopathy due to birth asphyxia and trauma	Diarrheal diseases	Congenital anomalies	Malaria	Neonatal sepsis and other neonatal infections	Meningitis	Other neonatal disorders	HIV/AIDS
Croatia	Number: Cumulative change (%)	-70.3 (-72.3 to -68.2)	-83.2 (-86.6 to -79.1)	-83.5 (-86.6 to -80.3)	-71.3 (-78.0 to -63.4)	-81.9 (-86.5 to -76.6)	-62.9 (-68.4 to -53.3)	nan (nan–nan)	128.0 (-48.4 to 309.5)	-82.6 (-85.7 to -79.2)	-77.0 (-82.1 to -71.2)	-33.0 (-44.2 to -20.8)
Croatia	Rate: 1990	91.8 (89.3–94.2)	14.4 (12.9–16.0)	3.6 (3.2–4.1)	7.1 (6.2–8.3)	0.6 (0.5–0.7)	18.0 (16.2–19.5)	0.0 (0.0–0.0)	0.6 (0.3–1.4)	1.1 (1.0–1.2)	9.6 (8.3–10.9)	0.0 (0.0–0.0)
Croatia	Rate: 2015	37.0 (34.4–39.7)	3.2 (2.6–3.9)	0.8 (0.7–0.9)	2.7 (2.2–3.3)	0.1 (0.1–0.2)	9.0 (7.8–10.7)	0.0 (0.0–0.0)	1.1 (0.6–1.6)	0.3 (0.2–0.3)	2.9 (2.4–3.6)	0.0 (0.0–0.0)
Croatia	Rate: Cumulative change (%)	-59.7 (-62.6 to -56.6)	-77.5 (-82.0 to -72.0)	-77.6 (-82.0 to -73.0)	-61.5 (-70.5 to -50.9)	-75.3 (-81.7 to -67.8)	-49.6 (-57.4 to -36.4)	nan (nan–nan)	205.9 (-30.7 to 448.9)	-76.0 (-80.4 to -71.3)	-69.2 (-76.0 to -61.4)	-7.3 (-23.7 to 9.7)
Czech Republic	Number: 1990	2,373 (2,319–2,428)	434 (398–486)	113 (101–128)	164 (144–187)	5 (4–6)	430 (274–480)	0 (0–0)	11 (7–23)	33 (29–38)	55 (47–65)	1 (1–1)
Czech Republic	Number: 2015	498 (462–537)	35 (26–43)	20 (17–24)	28 (22–35)	2 (2–3)	86 (71–119)	0 (0–0)	10 (5–14)	4 (3–5)	32 (26–40)	0 (0–0)
Czech Republic	Number: Cumulative change (%)	-79.0 (-80.6 to -77.2)	-91.8 (-94.3 to -89.8)	-81.7 (-85.8 to -77.9)	-82.3 (-87.7 to -77.3)	-48.7 (-64.6 to -32.7)	-79.0 (-84.5 to -55.9)	nan (nan–nan)	9.9 (-63.4 to 81.7)	-86.1 (-89.1 to -82.7)	-40.1 (-57.4 to -22.1)	-33.4 (-43.5 to -22.4)
Czech Republic	Rate: 1990	90.0 (87.7–92.3)	18.7 (17.1–20.8)	4.5 (4.0–5.1)	7.1 (6.2–8.0)	0.2 (0.2–0.3)	17.8 (11.4–19.9)	0.0 (0.0–0.0)	0.5 (0.3–1.0)	1.3 (1.2–1.5)	2.4 (2.0–2.8)	0.0 (0.0–0.1)
Czech Republic	Rate: 2015	25.1 (23.3–27.1)	1.8 (1.4–2.2)	1.0 (0.9–1.2)	1.5 (1.1–1.8)	0.1 (0.1–0.2)	4.3 (3.6–6.0)	0.0 (0.0–0.0)	0.5 (0.3–0.7)	0.2 (0.2–0.3)	1.7 (1.3–2.0)	0.0 (0.0–0.0)
Czech Republic	Rate: Cumulative change (%)	-72.1 (-74.2 to -69.7)	-90.3 (-93.3 to -88.0)	-76.8 (-82.1 to -71.9)	-79.1 (-85.5 to -73.2)	-36.9 (-56.7 to -17.0)	-74.6 (-81.2 to -46.6)	nan (nan–nan)	29.5 (-56.9 to 114.5)	-82.4 (-86.2 to -78.1)	-29.3 (-49.7 to -8.0)	-10.8 (-25.2 to 4.6)
Hungary	Number: 1990	3,069 (3,000–3,135)	598 (549–650)	180 (164–199)	456 (409–504)	8 (6–9)	575 (535–615)	0 (0–0)	18 (11–24)	42 (38–46)	44 (37–51)	2 (2–3)
Hungary	Number: 2015	713 (595–863)	110 (75–155)	25 (18–31)	23 (15–33)	4 (3–5)	171 (128–244)	0 (0–0)	9 (5–13)	5 (4–6)	69 (46–97)	0 (0–0)
Hungary	Number: Cumulative change (%)	-76.8 (-80.7 to -71.8)	-81.6 (-87.7 to -73.8)	-86.0 (-89.6 to -82.0)	-94.9 (-96.7 to -92.7)	-43.0 (-62.9 to -23.1)	-70.2 (-78.2 to -57.2)	nan (nan–nan)	-49.4 (-66.5 to -28.7)	-87.7 (-90.4 to -84.5)	58.4 (-0.2 to 129.4)	-85.4 (-87.5 to -83.2)
Hungary	Rate: 1990	121.6 (118.6–124.5)	26.0 (23.8–28.2)	7.5 (6.8–8.3)	19.8 (17.8–21.9)	0.3 (0.3–0.4)	24.1 (22.4–25.8)	0.0 (0.0–0.0)	0.8 (0.5–1.1)	1.7 (1.6–1.9)	1.9 (1.6–2.2)	0.1 (0.1–0.1)
Hungary	Rate: 2015	38.6 (31.8–47.4)	6.5 (4.4–9.2)	1.4 (1.1–1.8)	1.4 (0.9–2.0)	0.3 (0.2–0.3)	9.8 (7.3–14.0)	0.0 (0.0–0.0)	0.5 (0.3–0.8)	0.3 (0.2–0.4)	4.1 (2.8–5.7)	0.0 (0.0–0.0)
Hungary	Rate: Cumulative change (%)	-68.2 (-74.0 to -60.9)	-75.0 (-83.3 to -64.3)	-81.2 (-86.2 to -75.6)	-93.1 (-95.6 to -90.0)	-23.4 (-49.6 to 3.7)	-59.5 (-70.6 to -41.7)	nan (nan–nan)	-31.2 (-54.6 to -3.2)	-83.2 (-87.1 to -78.8)	115.5 (35.7–211.9)	-79.4 (-82.4 to -76.2)
Macedonia	Number: 1990	1,462 (1,415–1,513)	371 (328–419)	158 (134–182)	63 (50–77)	173 (147–201)	150 (90–191)	0 (0–0)	3 (2–6)	24 (18–29)	170 (136–221)	0 (0–0)
Macedonia	Number: 2015	327 (257–411)	75 (51–106)	18 (13–25)	13 (8–20)	4 (3–6)	56 (40–77)	0 (0–0)	3 (1–5)	4 (3–6)	21 (14–31)	0 (0–0)
Macedonia	Number: Cumulative change (%)	-77.6 (-82.4 to -71.5)	-79.6 (-86.3 to -70.8)	-87.9 (-91.6 to -83.3)	-78.6 (-86.8 to -65.7)	-97.3 (-98.3 to -96.0)	-60.4 (-75.3 to -36.2)	nan (nan–nan)	1.3 (-52.1 to 64.4)	-80.9 (-87.3 to -71.9)	-87.1 (-91.9 to -80.3)	inf (-53.8 to 1,658.5)
Macedonia	Rate: 1990	221.7 (214.5–229.6)	57.5 (50.9–65.0)	24.0 (20.3–27.7)	9.8 (7.8–12.1)	26.5 (22.5–30.7)	22.9 (13.8–29.3)	0.0 (0.0–0.0)	0.5 (0.4–1.0)	3.6 (2.8–4.4)	26.5 (21.1–34.3)	0.0 (0.0–0.0)
Macedonia	Rate: 2015	71.4 (55.5–90.9)	17.6 (12.1–24.7)	4.3 (3.0–5.8)	3.1 (2.0–4.7)	1.1 (0.7–1.6)	13.0 (9.1–17.7)	0.0 (0.0–0.0)	0.8 (0.4–1.2)	1.0 (0.7–1.4)	5.0 (3.3–7.4)	0.0 (0.0–0.0)
Macedonia	Rate: Cumulative change (%)	-67.8 (-75.1 to -58.5)	-69.3 (-79.4 to -56.1)	-82.1 (-87.6 to -75.1)	-67.8 (-80.1 to -48.4)	-95.9 (-97.5 to -93.9)	-40.7 (-63.1 to -3.9)	nan (nan–nan)	52.0 (-28.2 to 146.9)	-71.6 (-81.1 to -58.2)	-80.7 (-87.8 to -70.4)	inf (-34.3 to 2,350.6)
Montenegro	Number: 1990	256 (207–309)	47 (30–67)	22 (15–30)	37 (23–53)	2 (1–3)	22 (15–30)	0 (0–0)	6 (2–11)	2 (1–4)	16 (8–28)	0 (0–0)
Montenegro	Number: 2015	67 (57–79)	7 (4–9)	3 (2–4)	7 (4–10)	0 (0–0)	7 (5–10)	0 (0–0)	1 (0–2)	0 (0–0)	2 (1–4)	0 (0–0)
Montenegro	Number: Cumulative change (%)	-73.4 (-79.7 to -65.7)	-84.3 (-91.4 to -74.5)	-84.5 (-90.8 to -76.6)	-78.5 (-88.7 to -64.6)	-90.1 (-96.1 to -80.3)	-63.5 (-79.1 to -41.6)	nan (nan–nan)	-68.6 (-89.6 to -37.5)	-81.0 (-91.7 to -64.1)	-81.2 (-93.8 to -59.9)	inf (54.4–535.9)
Montenegro	Rate: 1990	132.2 (106.4–159.9)	25.4 (16.5–36.0)	11.6 (8.0–15.8)	20.1 (12.7–28.9)	1.1 (0.6–1.9)	11.6 (8.2–16.0)	0.0 (0.0–0.0)	3.3 (1.5–6.0)	1.2 (0.7–2.2)	8.9 (4.5–15.4)	0.0 (0.0–0.0)
Montenegro	Rate: 2015	45.3 (38.2–54.0)	5.3 (3.5–7.4)	2.3 (1.7–3.2)	5.7 (3.8–8.3)	0.1 (0.1–0.2)	5.6 (4.0–7.9)	0.0 (0.0–0.0)	1.2 (0.6–2.2)	0.3 (0.2–0.5)	2.0 (0.9–3.5)	0.0 (0.0–0.0)
Montenegro	Rate: Cumulative change (%)	-65.2 (-73.9 to -54.4)	-77.8 (-87.9 to -64.0)	-78.9 (-87.5 to -67.7)	-69.6 (-84.0 to -49.9)	-86.2 (-94.6 to -72.5)	-49.1 (-71.1 to -18.3)	nan (nan–nan)	-55.6 (-85.4 to -11.5)	-74.3 (-89.2 to -50.5)	-73.4 (-91.2 to -42.9)	inf (92.6–718.1)
Poland	Number: 1990	13,944 (13,783–14,099)	3,320 (2,985–3,814)	866 (782–970)	789 (641–964)	106 (91–126)	3,140 (2,342–3,411)	0 (0–0)	321 (162–445)	230 (207–259)	334 (262–426)	33 (30–36)
Poland	Number: 2015	2,944 (2,482–3,532)	456 (315–637)	107 (87–128)	96 (65–135)	7 (5–9)	768 (580–1,015)	0 (0–0)	74 (45–125)	18 (14–22)	64 (42–92)	8 (7–8)
Poland	Number: Cumulative change (%)	-78.9 (-82.2 to -74.7)	-86.2 (-90.9 to -80.2)	-87.5 (-90.3 to -84.7)	-87.5 (-92.3 to -81.4)	-92.8 (-95.0 to -90.2)	-75.1 (-82.2 to -61.6)	nan (nan–nan)	-74.9 (-87.5 to -51.4)	-92.1 (-93.9 to -90.0)	-80.3 (-87.6 to -69.7)	-76.0 (-79.5 to -72.2)
Poland	Rate: 1990	125.5 (123.9–126.9)	31.7 (28.5–36.5)	7.9 (7.1–8.8)	7.5 (6.1–9.2)	1.0 (0.8–1.2)	29.2 (21.6–31.7)	0.0 (0.0–0.0)	3.1 (1.6–4.3)	2.1 (1.9–2.3)	3.2 (2.5–4.1)	0.3 (0.3–0.3)
Poland	Rate: 2015	39.0 (32.7–47.2)	6.4 (4.4–8.9)	1.4 (1.2–1.7)	1.4 (0.9–1.9)	0.1 (0.1–0.1)	10.6 (8.0–14.1)	0.0 (0.0–0.0)	1.0 (0.6–1.8)	0.2 (0.2–0.3)	0.9 (0.6–1.3)	0.1 (0.1–0.1)
Poland	Rate: Cumulative change (%)	-68.9 (-74.1 to -62.4)	-79.7 (-86.6 to -70.9)	-81.5 (-85.7 to -77.2)	-81.7 (-88.8 to -72.7)	-89.3 (-92.6 to -85.4)	-62.8 (-73.4 to -42.4)	nan (nan–nan)	-63.1 (-81.6 to -28.8)	-88.1 (-90.9 to -84.9)	-71.1 (-81.9 to -55.5)	-63.3 (-68.7 to -57.6)
Romania	Number: 1990	14,167 (14,007–14,334)	1,208 (1,012–1,414)	4,095 (3,873–4,326)	605 (506–732)	349 (291–415)	1,411 (1,169–1,514)	0 (0–0)	45 (28–63)	306 (265–340)	1,299 (1,085–1,507)	95 (86–104)
Romania	Number: 2015	2,414 (2,088–2,814)	158 (113–208)	485 (389–601)	59 (43–81)	29 (21–40)	406 (312–522)	0 (0–0)	10 (6–17)	21 (17–27)	160 (118–206)	12 (11–14)
Romania	Number: Cumulative change (%)	-83.0 (-85.3 to -80.1)	-86.7 (-90.8 to -81.7)	-88.1 (-90.6 to -85.1)	-90.0 (-93.3 to -86.2)	-91.4 (-93.9 to -87.9)	-70.3 (-79.1 to -58.9)	nan (nan–nan)	-76.2 (-85.5 to -64.1)	-92.8 (-94.4 to -90.8)	-87.5 (-91.4 to -82.8)	-86.5 (-88.6 to -84.3)
Romania	Rate: 1990	222.5 (219.8–225.2)	20.7 (17.4–24.3)	66.2 (62.5–70.0)	10.4 (8.7–12.6)	5.8 (4.8–6.9)	23.2 (19.2–24.9)	0.0 (0.0–0.0)	0.8 (0.5–1.1)	4.9 (4.2–5.4)	22.3 (18.6–25.9)	1.4 (1.3–1.6)
Romania	Rate: 2015	65.6 (56.1–77.5)	4.8 (3.5–6.3)	13.9 (11.1–17.4)	1.8 (1.3–2.5)	0.9 (0.6–1.2)	11.8 (9.1–15.2)	0.0 (0.0–0.0)	0.3 (0.2–0.5)	0.6 (0.5–0.8)	4.9 (3.6–6.3)	0.3 (0.3–0.4)
Romania	Rate: Cumulative change (%)	-70.5 (-74.9 to -65.1)	-76.5 (-83.7 to -67.6)	-79.0 (-83.6 to -73.5)	-82.3 (-88.2 to -75.5)	-84.7 (-89.3 to -78.4)	-47.4 (-63.3 to -26.8)	nan (nan–nan)	-58.0 (-74.4 to -36.5)	-87.4 (-90.4 to -83.8)	-77.9 (-84.7 to -69.5)	-78.2 (-81.4 to -74.7)
Serbia	Number: 1990	4,438 (4,003–4,933)	1,084 (792–1,435)	366 (242–513)	331 (214–466)	30 (19–45)	635 (353–940)	0 (0–0)	105 (50–151)	82 (50–119)	220 (116–479)	0 (0–1)
Serbia	Number: 2015	1,107 (1,048–1,172)	197 (162–229)	44 (36–53)	76 (62–92)	4 (3–5)	209 (176–263)	0 (0–0)	19 (13–34)	9 (7–10)	46 (36–56)	1 (0–2)
Serbia	Number: Cumulative change (%)	-74.9 (-77.9 to -71.9)	-81.1 (-87.4 to -73.4)	-87.3 (-91.5 to -80.7)	-75.5 (-84.5 to -63.6)	-84.5 (-90.9 to -74.8)	-63.6 (-79.4 to -28.2)	nan (nan–nan)	-78.5 (-89.5 to -51.5)	-88.2 (-92.8 to -81.6)	-74.6 (-91.5 to -58.7)	inf (-57.6 to inf)
Serbia	Rate: 1990	163.4 (146.8–182.2)	41.8 (30.5–55.3)	13.7 (9.0–19.3)	12.8 (8.3–18.0)	1.2 (0.7–1.7)	23.9 (13.2–35.3)	0.0 (0.0–0.0)	4.0 (1.9–5.8)	3.1 (1.9–4.5)	8.5 (4.5–18.4)	0.0 (0.0–0.0)
Serbia	Rate: 2015	60.1 (56.6–64.0)	11.8 (9.8–13.7)	2.5 (2.0–3.0)	4.6 (3.7–5.5)	0.3 (0.2–0.3)	12.2 (10.2–15.4)	0.0 (0.0–0.0)	1.2 (0.8–2.1)	0.5 (0.4–0.6)	2.8 (2.2–3.4)	0.1 (0.0–0.2)
Serbia	Rate: Cumulative change (%)	-63.0 (-67.6 to -58.3)	-70.5 (-80.3 to -58.6)	-80.8 (-87.3 to -70.7)	-61.9 (-75.8 to -43.2)	-76.1 (-85.9 to -60.7)	-43.6 (-68.3 to 11.9)	nan (nan–nan)	-66.6 (-83.7 to -24.5)	-82.0 (-89.1 to -71.7)	-60.5 (-86.8 to -35.6)	inf (-35.8 to inf)
Slovakia	Number: 1990	1,633 (1,583–1,682)	333 (301–365)	214 (191–237)	42 (33–52)	9 (7–12)	286 (239–323)	0 (0–0)	5 (3–8)	24 (21–29)	86 (70–105)	0 (0–0)
Slovakia	Number: 2015	508 (460–565)	94 (76–113)	36 (31–42)	19 (15–25)	2 (2–3)	120 (100–144)	0 (0–0)	3 (2–5)	7 (5–8)	12 (9–15)	0 (0–0)
Slovakia	Number: Cumulative change (%)	-68.9 (-72.1 to -65.1)	-71.6 (-77.3 to -65.1)	-82.7 (-85.9 to -79.2)	-52.5 (-66.0 to -35.8)	-69.3 (-79.4 to -57.4)	-57.7 (-66.7 to -46.2)	nan (nan–nan)	-33.5 (-52.5 to -6.9)	-70.9 (-78.9 to -62.0)	-85.6 (-90.2 to -80.2)	inf (-86.9 to inf)
Slovakia	Rate: 1990	103.3 (99.9–106.5)	22.7 (20.6–24.9)	13.7 (12.2–15.3)	2.9 (2.3–3.6)	0.6 (0.5–0.8)	18.9 (15.8–21.4)	0.0 (0.0–0.0)	0.4 (0.2–0.6)	1.6 (1.3–1.9)	5.9 (4.8–7.2)	0.0 (0.0–0.0)
Slovakia	Rate: 2015	46.5 (41.9–51.9)	9.0 (7.3–10.8)	3.4 (2.9–4.0)	1.9 (1.5–2.4)	0.3 (0.2–0.4)	11.4 (9.5–13.6)	0.0 (0.0–0.0)	0.3 (0.2–0.5)	0.7 (0.5–0.8)	1.2 (0.9–1.5)	0.0 (0.0–0.0)
Slovakia	Rate: Cumulative change (%)	-54.9 (-59.8 to -49.3)	-60.4 (-68.3 to -51.3)	-74.9 (-79.5 to -69.8)	-33.5 (-52.5 to -10.2)	-56.2 (-70.6 to -39.1)	-39.4 (-52.2 to -22.9)	nan (nan–nan)	-7.2 (-33.6 to 30.2)	-57.4 (-69.3 to -44.2)	-79.9 (-86.3 to -72.3)	inf (-83.8 to inf)
Slovenia	Number: 1990	390 (378–403)	64 (59–70)	13 (11–15)	16 (14–19)	1 (0–1)	84 (77–92)	0 (0–0)	2 (1–4)	4 (3–4)	6 (4–7)	0 (0–0)
Slovenia	Number: 2015	96 (89–104)	10 (7–13)	2 (1–2)	3 (2–4)	0 (0–0)	26 (20–35)	0 (0–0)	1 (1–2)	0 (0–0)	3 (2–4)	0 (0–0)
Slovenia	Number: Cumulative change (%)	-75.2 (-77.1 to -73.1)	-83.5 (-88.1 to -79.8)	-81.5 (-85.4 to -77.0)	-81.1 (-86.5 to -74.7)	-79.9 (-85.0 to -73.7)	-69.1 (-75.3 to -61.2)	nan (nan–nan)	-32.0 (-54.4 to -7.6)	-86.0 (-88.5 to -82.9)	-37.2 (-54.7 to -14.7)	-81.9 (-85.6 to -77.7)
Slovenia	Rate: 1990	80.7 (77.9–83.7)	15.7 (14.3–17.1)	2.9 (2.5–3.3)	4.0 (3.4–4.6)	0.3 (0.2–0.3)	19.3 (17.8–21.2)	0.0 (0.0–0.0)	0.7 (0.5–1.0)	0.9 (0.8–1.0)	1.5 (1.2–1.8)	0.1 (0.1–0.1)
Slovenia	Rate: 2015	24.4 (22.7–26.3)	2.7 (1.9–3.3)	0.6 (0.5–0.7)	0.8 (0.6–1.0)	0.1 (0.0–0.1)	6.5 (5.2–8.9)	0.0 (0.0–0.0)	0.5 (0.3–0.6)	0.1 (0.1–0.2)	0.9 (0.7–1.2)	0.0 (0.0–0.0)
Slovenia	Rate: Cumulative change (%)	-69.7 (-72.1 to -67.2)	-82.8 (-87.6 to -78.9)	-79.0 (-83.5 to -73.8)	-80.2 (-85.9 to -73.6)	-77.5 (-83.2 to -70.6)	-66.1 (-72.9 to -57.3)	nan (nan–nan)	-29.0 (-52.4 to -3.7)	-83.2 (-86.2 to -79.4)	-34.4 (-52.8 to -10.9)	-78.6 (-83.2 to -73.6)
Central Asia	Number: 1990	136,834 (132,758–140,779)	13,318 (11,503–15,341)	51,286 (48,975–53,423)	12,803 (10,557–15,006)	13,444 (12,620–14,331)	8,107 (7,315–8,789)	12 (9–15)	1,524 (928–2,157)	2,341 (1,993–2,624)	3,213 (2,323–4,297)	18 (12–27)
Central Asia	Number: 2015	61,200 (57,193–65,723)	7,663 (6,564–8,837)	14,789 (13,127–16,605)	8,096 (6,823–9,506)	1,777 (1,436–2,164)	6,966 (5,478–8,145)	3 (1–5)	1,242 (759–1,736)	793 (676–989)	2,421 (1,827–3,207)	48 (30–74)
Central Asia	Number: Cumulative change (%)	-55.3 (-58.4 to -51.8)	-42.1 (-51.7 to -31.6)	-71.1 (-74.8 to -67.4)	-36.2 (-48.1 to -21.2)	-86.8 (-89.4 to -83.7)	-13.8 (-33.6 to 0.3)	-73.4 (-85.4 to -55.5)	-16.5 (-43.0 to 14.1)	-65.7 (-72.6 to -54.6)	-22.0 (-49.7 to 12.1)	175.6 (68.0–300.6)
Central Asia	Rate: 1990	390.1 (378.7–401.1)	36.7 (31.7–42.3)	143.6 (137.2–149.5)	35.3 (29.1–41.4)	37.4 (35.2–39.9)	22.7 (20.5–24.6)	0.0 (0.0–0.0)	4.2 (2.6–6.0)	6.8 (5.8–7.6)	8.9 (6.4–11.9)	0.1 (0.0–0.1)
Central Asia	Rate: 2015	180.5 (169.1–193.4)	21.8 (18.7–25.1)	42.6 (37.9–47.8)	23.0 (19.4–27.0)	5.1 (4.1–6.2)	20.0 (15.7–23.3)	0.0 (0.0–0.0)	3.5 (2.2–4.9)	2.4 (2.1–3.0)	6.9 (5.2–9.1)	0.2 (0.1–0.2)
Central Asia	Rate: Cumulative change (%)	-53.7 (-56.9 to -50.2)	-40.4 (-50.3 to -29.5)	-70.3 (-74.0 to -66.5)	-34.3 (-46.5 to -18.9)	-86.5 (-89.2 to -83.3)	-11.7 (-31.8 to 2.6)	-71.9 (-84.9 to -53.0)	-14.2 (-41.4 to 17.2)	-64.4 (-71.3 to -52.6)	-19.8 (-48.3 to 15.3)	200.1 (80.2–341.9)
Armenia	Number: 1990	4,238 (3,974–4,516)	616 (470–762)	1,115 (1,002–1,227)	121 (70–190)	375 (324–435)	477 (406–593)	0 (0–0)	182 (85–315)	18 (15–21)	229 (115–389)	0 (0–0)
Armenia	Number: 2015	820 (702–956)	91 (61–130)	116 (95–142)	24 (13–39)	10 (8–14)	199 (150–252)	0 (0–0)	45 (23–71)	3 (2–4)	47 (25–78)	0 (0–0)
Armenia	Number: Cumulative change (%)	-80.6 (-83.6 to -77.0)	-84.8 (-90.6 to -77.1)	-89.5 (-91.7 to -86.6)	-77.7 (-90.2 to -59.9)	-97.1 (-97.9 to -96.0)	-57.6 (-70.8 to -45.2)	-100.0 (-100.0 to -100.0)	-72.5 (-87.2 to -52.5)	-80.7 (-84.9 to -75.4)	-76.4 (-90.5 to -51.0)	inf (107.8–inf)
Armenia	Rate: 1990	309.6 (290.6–329.5)	45.4 (34.6–56.1)	80.3 (72.2–88.4)	8.9 (5.2–14.0)	27.1 (23.4–31.4)	34.8 (29.6–43.2)	0.0 (0.0–0.0)	13.4 (6.3–23.2)	1.3 (1.1–1.5)	16.8 (8.5–28.6)	0.0 (0.0–0.0)
Armenia	Rate: 2015	112.7 (96.6–131.5)	12.8 (8.5–18.2)	15.9 (13.0–19.5)	3.5 (1.9–5.5)	1.5 (1.1–1.9)	27.5 (20.7–34.8)	0.0 (0.0–0.0)	6.3 (3.3–10.0)	0.5 (0.4–0.6)	6.6 (3.6–10.9)	0.0 (0.0–0.0)
Armenia	Rate: Cumulative change (%)	-63.5 (-69.2 to -56.8)	-71.2 (-82.3 to -56.6)	-80.1 (-84.2 to -74.7)	-57.8 (-81.5 to -24.0)	-94.5 (-96.0 to -92.5)	-20.0 (-44.8 to 3.4)	-100.0 (-100.0 to -100.0)	-48.0 (-75.8 to -10.5)	-63.6 (-71.4 to -53.7)	-55.3 (-81.9 to -7.5)	inf (289.0–inf)
Azerbaijan	Number: 1990	17,992 (16,625–19,417)	1,571 (1,101–2,023)	8,107 (7,324–8,953)	1,581 (1,118–2,095)	1,532 (1,308–1,792)	1,055 (621–1,318)	1 (1–1)	65 (24–128)	132 (109–171)	126 (49–245)	0 (0–1)
Azerbaijan	Number: 2015	6,975 (5,841–8,286)	921 (597–1,273)	1,791 (1,400–2,281)	1,209 (845–1,593)	181 (120–254)	774 (498–1,059)	0 (0–0)	52 (19–104)	65 (50–82)	82 (34–166)	1 (0–3)
Azerbaijan	Number: Cumulative change (%)	-61.1 (-67.9 to -53.4)	-39.9 (-62.8 to -10.6)	-77.8 (-83.2 to -71.5)	-21.0 (-49.8 to 14.0)	-88.0 (-92.3 to -82.6)	-24.0 (-52.0 to 16.9)	-75.9 (-80.3 to -71.3)	-1.1 (-74.2 to 139.7)	-50.2 (-65.9 to -33.7)	-16.8 (-78.1 to 102.9)	62.6 (-22.5 to 195.9)
Azerbaijan	Rate: 1990	524.1 (485.1–564.5)	43.8 (30.7–56.4)	234.6 (212.2–258.7)	44.1 (31.2–58.5)	44.1 (37.8–51.6)	30.2 (17.8–37.7)	0.1 (0.0–0.1)	1.9 (0.7–3.6)	4.0 (3.3–5.1)	3.6 (1.4–6.9)	0.0 (0.0–0.0)
Azerbaijan	Rate: 2015	207.7 (176.0–245.1)	26.0 (16.9–36.0)	51.7 (40.9–65.4)	34.2 (23.9–45.0)	5.1 (3.4–7.2)	22.1 (14.3–30.1)	0.0 (0.0–0.0)	1.5 (0.5–2.9)	2.0 (1.6–2.5)	2.3 (1.0–4.7)	0.0 (0.0–0.1)
Azerbaijan	Rate: Cumulative change (%)	-60.3 (-66.9 to -52.8)	-39.1 (-62.3 to -9.4)	-77.9 (-83.1 to -71.8)	-20.1 (-49.2 to 15.2)	-88.3 (-92.4 to -83.0)	-24.1 (-51.7 to 16.4)	-73.2 (-77.8 to -68.3)	-0.8 (-74.0 to 139.1)	-47.7 (-64.4 to -30.6)	-16.4 (-78.1 to 104.5)	76.1 (-17.7 to 225.7)
Georgia	Number: 1990	4,695 (4,400–5,021)	583 (442–728)	1,476 (1,320–1,636)	831 (651–987)	173 (143–206)	229 (193–297)	0 (0–0)	91 (62–134)	54 (44–64)	52 (35–75)	0 (0–0)
Georgia	Number: 2015	1,272 (1,104–1,465)	194 (146–251)	92 (74–113)	143 (108–185)	13 (10–16)	154 (111–194)	0 (0–0)	64 (44–88)	13 (11–17)	143 (104–188)	0 (0–0)
Georgia	Number: Cumulative change (%)	-72.9 (-76.8 to -68.5)	-66.0 (-76.4 to -53.5)	-93.7 (-95.1 to -91.9)	-82.5 (-87.3 to -76.4)	-92.3 (-94.4 to -89.4)	-31.0 (-58.7 to -7.9)	-100.0 (-100.0 to -100.0)	-28.0 (-52.0 to 0.7)	-74.4 (-80.2 to -67.0)	191.9 (63.0–336.8)	402.4 (46.2–1,183.0)
Georgia	Rate: 1990	276.6 (259.2–296.1)	35.2 (26.7–43.9)	86.8 (77.6–96.2)	50.2 (39.3–59.6)	10.2 (8.5–12.2)	13.6 (11.5–17.7)	0.0 (0.0–0.0)	5.5 (3.8–8.1)	3.1 (2.6–3.7)	3.1 (2.1–4.5)	0.0 (0.0–0.0)
Georgia	Rate: 2015	128.5 (111.7–147.6)	19.6 (14.8–25.3)	9.2 (7.5–11.3)	14.4 (10.9–18.6)	1.3 (1.0–1.7)	15.4 (11.1–19.3)	0.0 (0.0–0.0)	6.4 (4.4–8.9)	1.4 (1.2–1.7)	14.4 (10.5–19.0)	0.0 (0.0–0.0)
Georgia	Rate: Cumulative change (%)	-53.5 (-60.1 to -45.9)	-43.3 (-60.5 to -22.3)	-89.3 (-91.6 to -86.3)	-70.9 (-78.8 to -60.5)	-87.1 (-90.7 to -82.2)	15.6 (-30.6 to 54.1)	-100.0 (-100.0 to -100.0)	20.0 (-19.9 to 67.9)	-55.2 (-65.2 to -42.3)	387.0 (171.8–628.5)	759.3 (148.8–2,090.6)
Kazakhstan	Number: 1990	19,042 (17,945–20,130)	3,445 (2,972–3,851)	4,848 (4,411–5,318)	661 (483–1,003)	1,423 (1,236–1,610)	2,002 (1,809–2,226)	0 (0–3)	271 (117–437)	310 (275–347)	405 (322–512)	6 (5–7)
Kazakhstan	Number: 2015	8,492 (7,189–10,018)	1,884 (1,360–2,460)	880 (726–1,076)	427 (287–617)	96 (73–123)	1,469 (1,094–1,918)	0 (0–0)	282 (129–478)	85 (69–105)	310 (217–421)	11 (9–14)
Kazakhstan	Number: Cumulative change (%)	-55.4 (-62.3 to -47.4)	-45.2 (-59.3 to -29.4)	-81.8 (-85.4 to -77.0)	-34.1 (-55.5 to -7.6)	-93.2 (-95.0 to -91.0)	-26.5 (-45.6 to -4.2)	-100.0 (-100.0 to -100.0)	11.1 (-44.3 to 84.8)	-72.4 (-78.1 to -65.1)	-22.6 (-47.1 to 4.7)	79.8 (42.9–121.5)
Kazakhstan	Rate: 1990	272.7 (257.3–287.8)	49.2 (42.5–55.0)	67.8 (61.7–74.4)	9.4 (6.9–14.3)	19.8 (17.2–22.5)	28.4 (25.6–31.6)	0.0 (0.0–0.0)	3.9 (1.7–6.2)	4.4 (3.9–4.9)	5.8 (4.6–7.3)	0.1 (0.1–0.1)
Kazakhstan	Rate: 2015	131.6 (112.6–154.0)	27.3 (19.7–35.6)	12.9 (10.7–15.7)	6.2 (4.2–8.9)	1.4 (1.1–1.8)	21.4 (16.0–27.9)	0.0 (0.0–0.0)	4.1 (1.9–6.9)	1.3 (1.0–1.6)	4.5 (3.1–6.1)	0.2 (0.2–0.2)
Kazakhstan	Rate: Cumulative change (%)	-51.7 (-58.9 to -43.5)	-44.5 (-58.7 to -28.5)	-81.0 (-84.7 to -76.1)	-33.3 (-55.0 to -6.4)	-93.0 (-94.8 to -90.7)	-24.4 (-44.1 to -1.5)	-100.0 (-100.0 to -100.0)	12.5 (-43.6 to 87.2)	-70.7 (-76.6 to -63.2)	-21.7 (-46.5 to 5.9)	91.3 (52.8–134.0)
Kyrgyzstan	Number: 1990	10,106 (9,761–10,452)	1,020 (797–1,269)	3,857 (3,616–4,089)	1,167 (884–1,412)	821 (721–925)	644 (560–795)	0 (0–1)	95 (57–131)	197 (140–233)	303 (209–416)	1 (0–2)
Kyrgyzstan	Number: 2015	5,143 (4,709–5,607)	982 (822–1,148)	1,034 (905–1,180)	813 (663–971)	180 (141–221)	689 (556–827)	0 (0–0)	96 (67–128)	50 (37–76)	204 (158–262)	2 (0–5)
Kyrgyzstan	Number: Cumulative change (%)	-49.1 (-53.7 to -44.2)	-1.9 (-26.3 to 27.0)	-73.1 (-76.9 to -68.9)	-29.1 (-47.0 to -4.8)	-78.0 (-83.2 to -71.8)	8.1 (-22.0 to 32.3)	-100.0 (-100.0 to -100.0)	4.9 (-27.4 to 41.5)	-72.6 (-82.6 to -49.4)	-29.6 (-54.9 to 4.8)	54.7 (-38.2 to 206.8)
Kyrgyzstan	Rate: 1990	425.7 (411.5–439.6)	41.3 (32.3–51.4)	159.6 (149.6–169.2)	47.3 (35.9–57.2)	33.9 (29.8–38.1)	26.6 (23.1–32.9)	0.0 (0.0–0.0)	3.9 (2.3–5.4)	8.4 (6.0–9.9)	12.3 (8.5–16.9)	0.1 (0.0–0.1)
Kyrgyzstan	Rate: 2015	190.0 (174.5–206.4)	34.9 (29.2–40.8)	37.4 (32.8–42.6)	28.9 (23.6–34.5)	6.4 (5.1–7.9)	24.8 (20.1–29.9)	0.0 (0.0–0.0)	3.4 (2.4–4.5)	1.9 (1.4–2.8)	7.3 (5.6–9.3)	0.1 (0.0–0.2)
Kyrgyzstan	Rate: Cumulative change (%)	-55.3 (-59.3 to -51.2)	-13.9 (-35.4 to 11.4)	-76.5 (-79.7 to -72.8)	-37.8 (-53.5 to -16.5)	-80.9 (-85.4 to -75.5)	-5.6 (-31.8 to 15.4)	-100.0 (-100.0 to -100.0)	-8.4 (-36.7 to 23.6)	-75.9 (-84.5 to -55.7)	-38.4 (-60.6 to -8.3)	48.4 (-38.0 to 191.1)
Mongolia	Number: 1990	7,223 (6,861–7,608)	591 (420–786)	3,026 (2,697–3,361)	749 (559–955)	89 (57–133)	370 (245–630)	0 (0–0)	73 (33–128)	341 (112–555)	103 (46–185)	0 (0–0)

© 2017 Global Burden of Disease Child and Adolescent Health Collaboration. JAMA Pediatrics.

eTable 1. Number of Deaths, Death Rates (per 100,000 population), and Cumulative Percent Change with 95% Uncertainty Intervals (UI) for the Top 10 Global Causes of Death in 195 Countries and Territories, Aged 0 to 19, Both Sexes, 1990 and 2015 *Note: best viewed by enlarging in browser.*

Location	Measure	All causes	Neonatal preterm birth complications	Lower respiratory infections	Neonatal encephalopathy due to birth asphyxia and trauma	Diarrheal diseases	Congenital anomalies	Malaria	Neonatal sepsis and other neonatal infections	Meningitis	Other neonatal disorders	HIV/AIDS
Mongolia	Number: 2015	2,423 (2,099–2,828)	259 (173–356)	491 (374–639)	432 (326–555)	2 (1–3)	339 (247–471)	0 (0–0)	48 (22–90)	22 (12–33)	54 (25–95)	0 (0–0)
Mongolia	Number: Cumulative change (%)	-66.4 (-71.3 to -60.6)	-54.7 (-71.4 to -32.4)	-83.7 (-87.7 to -78.4)	-41.0 (-59.5 to -16.6)	-97.4 (-98.7 to -95.5)	-1.1 (-49.1 to 44.7)	nan (nan–nan)	-24.8 (-72.4 to 49.6)	-90.8 (-97.3 to -72.6)	-37.3 (-79.0 to 42.4)	inf (-5.3 to 624.6)
Mongolia	Rate: 1990	583.4 (554.4–614.1)	46.9 (33.4–62.4)	240.5 (214.5–266.9)	59.5 (44.4–75.8)	7.0 (4.6–10.6)	29.7 (19.7–50.4)	0.0 (0.0–0.0)	5.8 (2.6–10.2)	27.9 (9.1–44.9)	8.2 (3.7–14.7)	0.0 (0.0–0.0)
Mongolia	Rate: 2015	202.7 (177.1–234.7)	20.6 (13.8–28.2)	39.4 (30.2–51.2)	34.2 (25.9–44.0)	0.2 (0.1–0.3)	27.3 (20.0–37.8)	0.0 (0.0–0.0)	3.8 (1.8–7.1)	1.9 (1.1–2.9)	4.3 (2.0–7.6)	0.0 (0.0–0.0)
Mongolia	Rate: Cumulative change (%)	-65.2 (-70.0 to -59.5)	-54.8 (-71.5 to -32.5)	-83.5 (-87.5 to -78.3)	-41.1 (-59.6 to -16.8)	-97.3 (-98.7 to -95.5)	-0.8 (-48.7 to 44.9)	nan (nan–nan)	-25.3 (-72.4 to 49.0)	-90.3 (-97.1 to -70.7)	-37.6 (-79.1 to 41.3)	inf (8.5–753.4)
Tajikistan	Number: 1990	20,799 (19,411–22,249)	2,010 (1,422–2,582)	7,558 (6,804–8,361)	1,301 (771–1,922)	4,148 (3,617–4,709)	706 (597–866)	2 (1–5)	230 (97–437)	426 (358–524)	519 (244–878)	3 (1–7)
Tajikistan	Number: 2015	9,946 (8,460–11,610)	1,381 (1,017–1,804)	2,333 (1,805–2,950)	906 (569–1,335)	909 (608–1,278)	909 (611–1,206)	2 (1–4)	194 (90–359)	236 (183–326)	467 (221–810)	17 (3–38)
Tajikistan	Number: Cumulative change (%)	-52.1 (-59.8 to -43.2)	-29.4 (-53.5 to 0.0)	-69.0 (-76.7 to -59.9)	-26.2 (-59.4 to 25.7)	-77.9 (-85.4 to -68.8)	31.1 (-22.2 to 80.7)	37.9 (-57.8 to 204.0)	-2.4 (-64.4 to 91.3)	-43.7 (-58.7 to -21.0)	5.2 (-64.2 to 126.0)	inf (73.7–815.9)
Tajikistan	Rate: 1990	579.9 (542.4–620.3)	52.3 (37.0–67.2)	208.6 (188.4–230.9)	33.9 (20.1–50.0)	112.8 (98.5–127.8)	19.1 (16.1–23.4)	0.1 (0.0–0.2)	6.0 (2.5–11.4)	12.4 (10.5–15.2)	13.6 (6.4–23.0)	0.1 (0.0–0.2)
Tajikistan	Rate: 2015	224.2 (192.2–260.2)	29.5 (21.7–38.5)	52.3 (40.9–66.0)	19.4 (12.2–28.5)	19.8 (13.4–27.8)	19.8 (13.4–26.3)	0.1 (0.0–0.1)	4.2 (1.9–7.7)	5.6 (4.4–7.5)	10.0 (4.7–17.3)	0.4 (0.1–1.0)
Tajikistan	Rate: Cumulative change (%)	-61.3 (-67.3 to -54.3)	-42.1 (-61.9 to -17.9)	-74.8 (-80.9 to -67.7)	-39.5 (-66.7 to 3.2)	-82.3 (-88.2 to -75.1)	6.1 (-36.9 to 45.9)	-1.5 (-69.8 to 112.9)	-20.4 (-70.9 to 55.5)	-54.7 (-66.3 to -37.7)	-14.0 (-70.7 to 84.6)	inf (60.0–728.3)
Turkmenistan	Number: 1990	13,263 (11,820–14,784)	1,089 (716–1,478)	5,821 (5,105–6,608)	1,025 (629–1,409)	1,610 (1,350–1,894)	685 (574–818)	1 (1–1)	106 (43–199)	194 (165–225)	265 (109–522)	0 (0–2)
Turkmenistan	Number: 2015	5,457 (4,442–6,653)	645 (412–911)	1,552 (1,160–2,004)	532 (335–762)	169 (111–238)	750 (437–1,046)	0 (0–0)	116 (52–228)	64 (46–92)	255 (106–519)	11 (5–18)
Turkmenistan	Number: Cumulative change (%)	-58.7 (-67.7 to -48.1)	-38.7 (-62.9 to -6.9)	-73.2 (-80.7 to -64.0)	-45.5 (-69.4 to -12.1)	-89.3 (-93.2 to -84.6)	10.9 (-39.0 to 57.0)	-100.0 (-100.0 to -100.0)	25.7 (-52.4 to 174.5)	-66.8 (-76.7 to -54.0)	13.0 (-62.3 to 140.8)	inf (547.5–inf)
Turkmenistan	Rate: 1990	599.3 (535.2–666.1)	46.1 (30.3–62.6)	260.0 (227.5–295.1)	43.4 (26.7–59.7)	71.3 (59.7–84.1)	30.0 (25.2–35.9)	0.1 (0.1–0.1)	4.6 (1.8–8.5)	9.1 (7.7–10.5)	11.3 (4.6–22.2)	0.0 (0.0–0.1)
Turkmenistan	Rate: 2015	269.2 (219.5–327.9)	31.6 (20.2–44.6)	76.4 (57.2–98.6)	26.1 (16.4–37.3)	8.3 (5.5–11.7)	36.9 (21.5–51.4)	0.0 (0.0–0.0)	5.7 (2.6–11.2)	3.2 (2.3–4.5)	12.5 (5.2–25.4)	0.6 (0.3–0.9)
Turkmenistan	Rate: Cumulative change (%)	-54.9 (-64.7 to -43.5)	-29.1 (-57.0 to 7.6)	-70.4 (-78.7 to -60.2)	-37.0 (-64.6 to 1.6)	-88.2 (-92.4 to -83.0)	24.4 (-32.2 to 76.3)	-100.0 (-100.0 to -100.0)	44.5 (-45.1 to 214.6)	-64.7 (-75.1 to -51.5)	30.1 (-56.5 to 177.1)	inf (583.6–inf)
Uzbekistan	Number: 1990	39,471 (36,500–42,754)	2,389 (1,521–3,433)	15,475 (13,874–17,100)	5,362 (4,033–6,680)	3,269 (2,804–3,786)	1,934 (1,554–2,256)	3 (3–4)	407 (165–766)	665 (555–774)	1,208 (591–2,105)	5 (1–13)
Uzbekistan	Number: 2015	20,669 (17,430–24,470)	1,301 (809–1,962)	6,497 (5,148–7,981)	3,605 (2,593–4,694)	213 (150–291)	1,678 (1,109–2,300)	0 (0–0)	341 (141–675)	251 (195–339)	856 (411–1,506)	4 (1–11)
Uzbekistan	Number: Cumulative change (%)	-47.5 (-56.1 to -37.5)	-42.4 (-69.4 to -5.0)	-57.9 (-67.0 to -47.1)	-31.1 (-54.4 to -2.3)	-93.4 (-95.5 to -90.8)	-12.9 (-39.7 to 16.5)	-100.0 (-100.0 to -100.0)	-0.5 (-68.6 to 118.8)	-61.7 (-72.2 to -45.3)	-17.7 (-72.2 to 78.2)	inf (-71.9 to 229.1)
Uzbekistan	Rate: 1990	324.9 (301.5–351.2)	18.8 (12.0–27.0)	124.3 (111.5–137.3)	42.2 (31.8–52.6)	26.1 (22.4–30.2)	15.5 (12.5–18.1)	0.0 (0.0–0.0)	3.2 (1.3–6.0)	5.7 (4.8–6.6)	9.5 (4.7–16.6)	0.0 (0.0–0.1)
Uzbekistan	Rate: 2015	173.1 (146.8–204.1)	10.7 (6.6–16.1)	53.5 (42.5–65.6)	29.5 (21.2–38.4)	1.7 (1.2–2.4)	13.8 (9.1–18.9)	0.0 (0.0–0.0)	2.8 (1.2–5.5)	2.2 (1.7–2.9)	7.0 (3.4–12.3)	0.0 (0.0–0.1)
Uzbekistan	Rate: Cumulative change (%)	-46.6 (-55.1 to -36.6)	-40.1 (-68.2 to -1.1)	-56.8 (-66.1 to -45.9)	-28.4 (-52.6 to 1.6)	-93.2 (-95.4 to -90.6)	-10.7 (-37.9 to 19.4)	-100.0 (-100.0 to -100.0)	3.3 (-67.4 to 127.2)	-61.6 (-71.9 to -45.3)	-14.5 (-71.1 to 85.1)	inf (-69.5 to 260.4)
Latin America and Caribbean	Number: 1990	681,138 (674,537–687,480)	90,318 (86,083–93,981)	101,176 (97,656–104,946)	41,963 (39,678–44,452)	110,851 (106,597–115,329)	44,200 (40,781–52,145)	930 (779–1,110)	24,779 (21,590–31,430)	14,772 (12,761–16,263)	14,601 (13,358–15,941)	1,557 (1,351–1,798)
Latin America and Caribbean	Number: 2015	261,392 (254,498–268,810)	29,994 (27,919–32,149)	24,940 (23,288–26,876)	16,870 (15,560–18,294)	10,244 (9,182–11,567)	38,092 (33,116–40,971)	80 (56–113)	15,892 (13,827–19,108)	3,832 (3,364–4,488)	9,775 (8,538–10,927)	2,263 (2,083–2,445)
Latin America and Caribbean	Number: Cumulative change (%)	-61.6 (-62.7 to -60.5)	-66.8 (-68.8 to -64.4)	-75.3 (-77.4 to -73.2)	-59.8 (-63.0 to -56.3)	-90.8 (-91.8 to -89.5)	-13.0 (-32.2 to -2.2)	-91.3 (-93.9 to -87.8)	-35.6 (-41.6 to -29.5)	-73.9 (-78.4 to -67.5)	-32.9 (-41.5 to -24.0)	46.1 (27.1–64.9)
Latin America and Caribbean	Rate: 1990	348.8 (345.4–352.0)	45.7 (43.5–47.5)	51.5 (49.7–53.4)	21.2 (20.1–22.5)	56.4 (54.2–58.7)	22.5 (20.7–26.5)	0.5 (0.4–0.6)	12.5 (10.9–15.9)	7.6 (6.5–8.3)	7.4 (6.8–8.1)	0.8 (0.7–0.9)
Latin America and Caribbean	Rate: 2015	138.4 (134.6–142.5)	16.7 (15.5–17.9)	13.5 (12.6–14.6)	9.4 (8.6–10.2)	5.6 (5.0–6.3)	20.8 (18.1–22.4)	0.0 (0.0–0.1)	8.8 (7.7–10.6)	2.0 (1.8–2.4)	5.4 (4.7–6.1)	1.2 (1.1–1.3)
Latin America and Caribbean	Rate: Cumulative change (%)	-60.3 (-61.5 to -59.1)	-63.5 (-65.8 to -60.9)	-73.8 (-76.0 to -71.5)	-55.8 (-59.3 to -52.0)	-90.1 (-91.2 to -88.8)	-6.4 (-27.0 to 5.2)	-91.3 (-94.0 to -87.9)	-29.4 (-36.0 to -22.7)	-72.7 (-77.5 to -65.9)	-26.4 (-35.9 to -16.7)	45.3 (26.6–63.7)
Central Latin America	Number: 1990	262,420 (259,202–265,744)	31,927 (30,164–33,460)	34,869 (33,601–36,151)	18,532 (17,435–19,554)	40,522 (39,012–41,907)	22,447 (21,425–23,838)	233 (199–273)	8,584 (7,042–11,489)	3,995 (3,815–4,214)	6,459 (5,934–6,968)	364 (353–376)
Central Latin America	Number: 2015	114,654 (110,482–119,116)	13,254 (11,982–14,477)	11,000 (10,185–11,869)	5,894 (5,310–6,495)	4,339 (3,971–4,768)	20,035 (18,164–21,537)	20 (15–26)	6,190 (4,975–7,866)	1,039 (960–1,156)	3,064 (2,656–3,477)	596 (569–628)
Central Latin America	Number: Cumulative change (%)	-56.3 (-58.1 to -54.6)	-58.5 (-61.8 to -54.9)	-68.4 (-70.9 to -65.9)	-68.2 (-71.2 to -65.1)	-89.3 (-90.3 to -88.1)	-10.6 (-19.6 to -3.0)	-91.3 (-93.6 to -88.4)	-27.5 (-36.2 to -18.8)	-74.0 (-76.0 to -71.6)	-52.5 (-58.1 to -46.6)	63.8 (57.5–70.1)
Central Latin America	Rate: 1990	297.0 (293.4–300.7)	35.4 (33.4–37.1)	39.1 (37.7–40.5)	20.5 (19.3–21.7)	45.4 (43.7–47.0)	25.1 (23.9–26.6)	0.3 (0.2–0.3)	9.5 (7.8–12.7)	4.5 (4.3–4.8)	7.2 (6.6–7.7)	0.4 (0.4–0.4)
Central Latin America	Rate: 2015	128.2 (123.3–133.2)	15.5 (14.0–16.9)	12.5 (11.6–13.5)	6.9 (6.2–7.6)	4.9 (4.5–5.4)	23.0 (20.9–24.8)	0.0 (0.0–0.0)	7.2 (5.8–9.2)	1.2 (1.1–1.3)	3.6 (3.1–4.1)	0.7 (0.6–0.7)
Central Latin America	Rate: Cumulative change (%)	-56.8 (-58.7 to -55.0)	-56.3 (-59.8 to -52.5)	-67.9 (-70.4 to -65.3)	-66.5 (-69.7 to -63.3)	-89.1 (-90.1 to -88.0)	-8.0 (-17.2 to -0.2)	-91.9 (-94.1 to -89.2)	-23.8 (-33.0 to -14.7)	-74.2 (-76.2 to -71.8)	-50.1 (-56.0 to -43.9)	56.0 (49.9–62.0)
Colombia	Number: 1990	40,098 (38,447–41,699)	4,225 (3,426–4,817)	4,547 (4,098–4,992)	2,806 (2,285–3,258)	4,267 (3,842–4,729)	3,446 (2,621–3,812)	102 (79–131)	1,242 (700–2,618)	1,134 (1,023–1,245)	781 (625–932)	29 (27–31)
Colombia	Number: 2015	18,348 (15,687–21,602)	1,616 (1,073–2,219)	1,445 (1,153–1,805)	681 (450–947)	371 (289–472)	3,127 (2,121–4,097)	9 (5–14)	1,047 (663–1,760)	238 (195–293)	643 (427–896)	91 (85–97)
Colombia	Number: Cumulative change (%)	-54.2 (-61.2 to -45.7)	-61.7 (-73.2 to -47.1)	-68.1 (-75.0 to -59.8)	-75.6 (-83.6 to -66.1)	-91.3 (-93.3 to -88.5)	-9.3 (-32.0 to 18.0)	-90.5 (-94.6 to -85.0)	-5.7 (-46.5 to 39.1)	-79.0 (-83.3 to -73.8)	-17.3 (-43.1 to 15.3)	212.3 (185.1–240.4)
Colombia	Rate: 1990	245.8 (235.9–255.5)	25.5 (20.7–29.1)	27.4 (24.7–30.1)	16.9 (13.8–19.7)	25.6 (23.1–28.4)	20.8 (15.8–23.0)	0.6 (0.5–0.8)	7.5 (4.2–15.8)	6.9 (6.2–7.5)	4.7 (3.8–5.6)	0.2 (0.2–0.2)
Colombia	Rate: 2015	124.1 (104.9–147.7)	11.8 (7.8–16.2)	10.1 (8.0–12.7)	5.0 (3.3–6.9)	2.6 (2.0–3.3)	22.3 (15.1–29.3)	0.1 (0.0–0.1)	7.6 (4.8–12.8)	1.6 (1.3–2.0)	4.7 (3.1–6.5)	0.6 (0.6–0.6)
Colombia	Rate: Cumulative change (%)	-49.5 (-57.6 to -39.4)	-53.7 (-67.6 to -36.1)	-63.0 (-71.2 to -53.2)	-70.6 (-80.2 to -59.1)	-89.8 (-92.2 to -86.5)	7.3 (-19.8 to 40.0)	-90.1 (-94.5 to -84.4)	13.8 (-35.4 to 67.9)	-76.2 (-81.1 to -70.2)	-0.2 (-31.3 to 39.3)	235.0 (205.5–265.8)
Costa Rica	Number: 1990	2,064 (1,883–2,269)	429 (365–503)	170 (145–201)	136 (107–167)	104 (88–124)	409 (240–494)	0 (0–0)	46 (28–63)	33 (28–38)	82 (64–103)	5 (5–5)
Costa Rica	Number: 2015	1,044 (882–1,251)	121 (89–163)	42 (31–56)	43 (30–60)	18 (13–24)	284 (191–375)	0 (0–0)	17 (9–33)	11 (8–14)	46 (33–62)	8 (8–9)
Costa Rica	Number: Cumulative change (%)	-49.2 (-58.7 to -38.4)	-71.4 (-79.6 to -61.1)	-74.8 (-81.9 to -66.6)	-67.5 (-78.7 to -53.0)	-82.4 (-87.6 to -76.2)	-29.5 (-49.7 to -3.4)	-64.6 (-69.5 to -59.3)	-60.9 (-80.4 to -27.4)	-66.3 (-74.2 to -57.2)	-42.2 (-61.5 to -17.9)	59.3 (44.5–74.2)
Costa Rica	Rate: 1990	140.9 (129.0–154.6)	28.3 (24.2–33.2)	11.3 (9.7–13.4)	9.0 (7.1–11.0)	6.9 (5.9–8.2)	27.2 (16.0–32.7)	0.0 (0.0–0.0)	3.1 (1.9–4.2)	2.3 (2.0–2.6)	5.4 (4.3–6.8)	0.4 (0.4–0.4)
Costa Rica	Rate: 2015	75.6 (63.1–91.4)	9.5 (7.0–12.8)	3.2 (2.4–4.3)	3.4 (2.4–4.7)	1.4 (1.0–1.9)	21.7 (14.6–28.7)	0.0 (0.0–0.0)	1.4 (0.8–2.6)	0.8 (0.6–1.0)	3.6 (2.6–4.9)	0.6 (0.5–0.6)
Costa Rica	Rate: Cumulative change (%)	-46.2 (-56.7 to -34.1)	-66.3 (-75.9 to -54.1)	-71.6 (-79.7 to -62.1)	-61.8 (-74.9 to -44.7)	-80.0 (-85.9 to -72.8)	-18.9 (-42.4 to 11.3)	-67.4 (-72.1 to -62.3)	-53.9 (-76.9 to -14.5)	-63.9 (-72.7 to -53.9)	-31.9 (-54.7 to -3.3)	47.7 (34.3–61.5)
El Salvador	Number: 1990	11,452 (10,707–12,253)	1,475 (1,267–1,680)	1,039 (883–1,204)	509 (400–621)	1,904 (1,613–2,206)	859 (647–1,174)	1 (1–1)	431 (321–629)	170 (144–199)	270 (213–337)	7 (4–10)
El Salvador	Number: 2015	2,621 (2,281–3,025)	193 (127–278)	215 (166–272)	61 (39–91)	61 (44–80)	426 (286–577)	0 (0–0)	73 (40–157)	28 (22–39)	33 (19–49)	15 (5–28)
El Salvador	Number: Cumulative change (%)	-77.1 (-80.5 to -72.9)	-86.8 (-91.2 to -80.8)	-79.1 (-84.5 to -72.8)	-87.8 (-92.5 to -81.2)	-96.8 (-97.7 to -95.6)	-48.2 (-72.5 to -22.7)	-80.7 (-84.1 to -76.8)	-83.1 (-90.8 to -70.8)	-82.9 (-87.6 to -76.4)	-87.4 (-93.2 to -79.5)	106.4 (4.3–199.9)
El Salvador	Rate: 1990	401.8 (376.5–428.6)	49.7 (42.7–56.6)	35.6 (30.2–41.3)	17.2 (13.5–21.0)	64.8 (55.0–75.0)	29.2 (22.0–40.0)	0.1 (0.1–0.1)	14.6 (10.8–21.2)	6.0 (5.0–6.9)	9.1 (7.2–11.4)	0.2 (0.2–0.4)
El Salvador	Rate: 2015	116.9 (100.0–137.9)	10.0 (6.6–14.4)	10.4 (7.9–13.1)	3.2 (2.1–4.7)	3.0 (2.2–4.0)	21.4 (14.3–29.1)	0.0 (0.0–0.0)	3.8 (2.1–8.1)	1.3 (1.0–1.8)	1.7 (1.0–2.5)	0.7 (0.2–1.3)
El Salvador	Rate: Cumulative change (%)	-70.9 (-75.5 to -65.1)	-79.8 (-86.5 to -70.5)	-70.6 (-78.4 to -61.6)	-81.2 (-88.5 to -71.2)	-95.3 (-96.7 to -93.6)	-23.6 (-59.3 to 14.4)	-78.3 (-82.1 to -73.9)	-74.1 (-85.9 to -55.3)	-77.5 (-83.6 to -68.9)	-80.8 (-89.6 to -68.7)	160.3 (16.6–289.5)
Guatemala	Number: 1990	32,865 (31,915–33,831)	2,422 (1,929–2,838)	6,616 (5,992–7,264)	841 (644–1,110)	6,927 (6,227–7,609)	804 (649–1,255)	57 (42–75)	1,093 (766–1,703)	225 (199–252)	916 (678–1,150)	68 (64–71)
Guatemala	Number: 2015	15,425 (13,605–17,590)	1,281 (1,002–1,573)	3,386 (2,819–4,045)	728 (553–906)	1,500 (1,229–1,831)	1,490 (1,228–1,794)	1 (0–1)	630 (441–1,025)	168 (140–200)	373 (285–465)	95 (89–102)
Guatemala	Number: Cumulative change (%)	-53.0 (-58.9 to -46.2)	-46.7 (-57.8 to -34.9)	-48.6 (-58.5 to -36.9)	-11.1 (-38.7 to 23.4)	-78.3 (-82.8 to -72.5)	93.0 (15.9–149.1)	-97.7 (-98.5 to -96.7)	-41.7 (-58.1 to -22.6)	-24.9 (-40.3 to -6.8)	-58.4 (-69.7 to -45.0)	40.2 (28.2–52.0)
Guatemala	Rate: 1990	551.5 (535.7–567.0)	37.6 (30.0–44.1)	109.6 (99.4–120.2)	13.1 (10.0–17.3)	115.5 (103.8–126.9)	12.9 (10.4–20.1)	1.0 (0.8–1.4)	17.0 (11.9–26.5)	3.8 (3.4–4.3)	14.3 (10.6–17.9)	1.2 (1.2–1.3)
Guatemala	Rate: 2015	194.7 (171.7–221.6)	16.0 (12.5–19.6)	42.4 (35.4–50.6)	9.1 (6.9–11.3)	18.8 (15.4–23.0)	18.6 (15.4–22.4)	0.0 (0.0–0.0)	7.8 (5.5–12.8)	2.1 (1.8–2.5)	4.7 (3.6–5.8)	1.2 (1.1–1.3)
Guatemala	Rate: Cumulative change (%)	-64.7 (-69.0 to -59.6)	-57.3 (-66.1 to -47.8)	-61.1 (-68.5 to -52.3)	-28.8 (-50.9 to -1.1)	-83.6 (-87.0 to -79.4)	50.4 (-9.5 to 93.4)	-98.4 (-98.9 to -97.7)	-53.3 (-66.4 to -38.0)	-44.2 (-55.5 to -31.0)	-66.8 (-75.8 to -56.0)	-2.7 (-10.8 to 5.4)
Honduras	Number: 1990	12,936 (12,549–13,319)	1,221 (920–1,511)	1,018 (873–1,168)	538 (330–805)	2,390 (2,069–2,732)	1,228 (859–1,901)	8 (5–12)	505 (258–855)	136 (95–217)	201 (103–349)	26 (19–36)
Honduras	Number: 2015	5,282 (4,779–5,904)	577 (435–724)	379 (300–470)	268 (177–387)	302 (233–383)	913 (721–1,164)	1 (0–1)	317 (177–483)	53 (35–75)	71 (40–111)	74 (54–98)
Honduras	Number: Cumulative change (%)	-59.2 (-63.3 to -54.2)	-51.8 (-65.7 to -34.2)	-62.4 (-71.5 to -52.0)	-46.6 (-71.6 to -9.5)	-87.3 (-90.4 to -83.2)	-21.6 (-52.9 to 9.2)	-86.9 (-93.1 to -77.8)	-31.2 (-67.5 to 17.2)	-59.1 (-73.6 to -35.4)	-59.6 (-84.2 to -20.3)	185.6 (130.6–247.2)
Honduras	Rate: 1990	406.9 (395.4–418.5)	35.6 (26.8–44.0)	31.1 (26.8–35.6)	15.7 (9.6–23.5)	73.9 (64.1–84.4)	37.1 (25.9–57.6)	0.3 (0.2–0.4)	14.7 (7.5–24.9)	4.4 (3.1–7.1)	5.9 (3.0–10.2)	0.8 (0.6–1.1)
Honduras	Rate: 2015	162.3 (146.4–181.5)	18.7 (14.1–23.4)	12.0 (9.4–14.9)	8.7 (5.7–12.5)	9.5 (7.3–12.1)	29.0 (22.9–37.0)	0.0 (0.0–0.0)	10.2 (5.7–15.6)	1.6 (1.1–2.3)	2.3 (1.3–3.6)	2.2 (1.6–2.9)
Honduras	Rate: Cumulative change (%)	-60.1 (-64.1 to -55.1)	-46.5 (-61.9 to -27.0)	-61.1 (-70.6 to -50.4)	-40.8 (-68.5 to 0.4)	-87.1 (-90.4 to -83.0)	-17.4 (-50.7 to 15.3)	-88.7 (-94.1 to -80.5)	-23.8 (-63.9 to 29.8)	-61.1 (-74.9 to -38.5)	-55.3 (-82.5 to -12.0)	167.5 (114.8–224.5)
Mexico	Number: 1990	129,510 (127,132–131,900)	16,938 (16,054–17,900)	17,879 (16,976–18,777)	11,476 (10,820–12,177)	19,379 (18,483–20,247)	12,593 (12,078–13,079)	14 (13–15)	3,826 (2,682–5,043)	1,531 (1,452–1,611)	3,597 (3,321–3,883)	140 (138–142)
Mexico	Number: 2015	51,389 (49,094–53,917)	6,719 (5,981–7,490)	3,766 (3,441–4,142)	3,010 (2,656–3,400)	1,246 (1,132–1,384)	10,653 (9,689–11,550)	4 (3–4)	2,998 (2,157–3,678)	288 (268–309)	1,540 (1,324–1,761)	219 (216–222)
Mexico	Number: Cumulative change (%)	-60.3 (-62.3 to -58.1)	-60.3 (-64.6 to -56.0)	-78.9 (-80.9 to -76.5)	-73.8 (-76.8 to -70.5)	-93.6 (-94.2 to -92.8)	-15.3 (-22.7 to -7.9)	-71.8 (-74.6 to -68.8)	-21.2 (-30.2 to -10.5)	-81.2 (-82.8 to -79.4)	-57.1 (-62.8 to -50.8)	56.6 (53.3–60.0)
Mexico	Rate: 1990	291.3 (285.9–296.6)	37.7 (35.8–39.9)	40.2 (38.2–42.2)	25.6 (24.1–27.1)	43.6 (41.6–45.6)	28.2 (27.1–29.3)	0.0 (0.0–0.0)	8.5 (6.0–11.2)	3.5 (3.3–3.6)	8.0 (7.4–8.7)	0.3 (0.3–0.3)
Mexico	Rate: 2015	114.5 (109.2–120.3)	15.6 (13.9–17.4)	8.6 (7.8–9.4)	7.0 (6.2–7.9)	2.8 (2.6–3.1)	24.4 (22.2–26.4)	0.0 (0.0–0.0)	7.0 (5.0–8.5)	0.6 (0.6–0.7)	3.6 (3.1–4.1)	0.5 (0.5–0.5)
Mexico	Rate: Cumulative change (%)	-60.7 (-62.7 to -58.4)	-58.7 (-63.1 to -54.2)	-78.7 (-80.7 to -76.2)	-72.7 (-75.8 to -69.3)	-93.5 (-94.2 to -92.8)	-13.5 (-21.1 to -5.9)	-73.7 (-76.3 to -71.0)	-18.0 (-27.4 to -6.9)	-81.4 (-83.0 to -79.6)	-55.4 (-61.3 to -48.9)	50.0 (46.8–53.3)
Nicaragua	Number: 1990	10,883 (10,574–11,222)	1,303 (1,128–1,495)	1,419 (1,222–1,630)	717 (567–899)	2,852 (2,557–3,153)	766 (414–1,101)	36 (25–51)	421 (270–549)	236 (199–285)	138 (102–188)	1 (0–1)
Nicaragua	Number: 2015	2,583 (2,287–2,936)	295 (228–371)	364 (299–441)	177 (128–238)	105 (75–140)	424 (285–558)	0 (0–0)	157 (113–206)	43 (34–60)	32 (23–41)	4 (1–9)
Nicaragua	Number: Cumulative change (%)	-76.3 (-79.1 to -73.0)	-77.2 (-82.9 to -70.6)	-74.1 (-80.0 to -66.5)	-75.0 (-82.7 to -65.8)	-96.3 (-97.4 to -95.0)	-41.1 (-68.3 to -13.6)	-98.9 (-99.3 to -98.4)	-61.8 (-73.1 to -47.2)	-81.4 (-85.8 to -75.4)	-76.2 (-84.7 to -65.5)	229.0 (44.5–520.6)
Nicaragua	Rate: 1990	415.0 (403.4–427.8)	48.1 (41.6–55.1)	53.3 (45.9–61.1)	26.5 (20.9–33.2)	106.5 (95.6–117.8)	28.6 (15.5–41.1)	1.4 (1.0–2.0)	15.6 (10.0–20.3)	9.0 (7.6–10.8)	5.1 (3.8–7.0)	0.0 (0.0–0.1)
Nicaragua	Rate: 2015	111.5 (98.3–127.1)	13.3 (10.3–16.7)	16.0 (13.0–19.4)	7.9 (5.8–10.7)	4.6 (3.3–6.2)	18.8 (12.6–24.7)	0.0 (0.0–0.0)	7.1 (5.1–9.3)	1.9 (1.5–2.6)	1.4 (1.1–1.9)	0.2 (0.1–0.4)
Nicaragua	Rate: Cumulative change (%)	-73.1 (-76.4 to -69.3)	-72.2 (-79.2 to -64.2)	-69.7 (-76.6 to -60.9)	-69.6 (-78.9 to -58.4)	-95.7 (-97.0 to -94.2)	-30.3 (-62.5 to 1.8)	-98.8 (-99.3 to -98.2)	-53.5 (-67.3 to -35.8)	-79.1 (-84.1 to -72.3)	-71.2 (-81.5 to -58.1)	249.5 (45.7–577.4)
Panama	Number: 1990	2,272 (2,075–2,485)	303 (254–355)	175 (143–217)	132 (98–177)	174 (140–216)	339 (170–459)	0 (0–0)	78 (56–112)	57 (48–68)	60 (44–81)	3 (2–6)
Panama	Number: 2015	1,830 (1,539–2,183)	177 (118–249)	186 (147–238)	109 (70–159)	99 (75–127)	321 (173–466)	0 (0–0)	87 (57–122)	31 (23–44)	54 (35–79)	7 (3–14)
Panama	Number: Cumulative change (%)	-19.2 (-33.5 to -1.4)	-41.4 (-61.2 to -18.5)	7.2 (-18.1 to 39.5)	-16.2 (-46.0 to 22.0)	-42.3 (-57.6 to -23.4)	-2.9 (-29.7 to 32.2)	18.9 (-8.2 to 46.9)	13.3 (-24.4 to 63.5)	-44.5 (-61.3 to -20.0)	-8.5 (-42.1 to 36.6)	104.7 (32.1–191.2)
Panama	Rate: 1990	193.1 (176.5–211.2)	25.6 (21.5–30.0)	14.9 (12.2–18.4)	11.2 (8.3–15.0)	14.8 (11.9–18.3)	28.7 (14.4–38.8)	0.0 (0.0–0.0)	6.6 (4.8–9.5)	4.9 (4.1–5.8)	5.1 (3.8–6.9)	0.3 (0.2–0.5)
Panama	Rate: 2015	131.0 (110.0–156.4)	12.8 (8.6–18.1)	13.4 (10.6–17.1)	7.9 (5.1–11.6)	7.1 (5.4–9.2)	23.2 (12.5–33.7)	0.0 (0.0–0.0)	6.3 (4.1–8.9)	2.3 (1.7–3.2)	3.9 (2.6–5.8)	0.6 (0.3–1.0)
Panama	Rate: Cumulative change (%)	-31.9 (-44.0 to -17.0)	-49.6 (-66.6 to -29.9)	-9.1 (-30.6 to 18.3)	-28.0 (-53.6 to 4.9)	-51.1 (-64.1 to -35.1)	-17.2 (-40.0 to 12.9)	-2.5 (-24.6 to 20.3)	-2.6 (-35.0 to 40.6)	-53.1 (-67.3 to -32.3)	-21.3 (-50.3 to 17.4)	68.5 (8.1–139.9)
Venezuela	Number: 1990	20,334 (20,217–20,450)	3,607 (3,230–3,983)	2,002 (1,851–2,168)	1,373 (1,128–1,648)	2,519 (2,338–2,711)	1,998 (1,770–2,389)	12 (9–16)	937 (536–1,223)	468 (426–516)	408 (320–507)	82 (77–87)
Venezuela	Number: 2015	16,127 (15,346–16,885)	2,271 (1,951–2,647)	1,213 (1,086–1,364)	814 (678–972)	633 (547–733)	2,395 (2,153–2,656)	3 (2–4)	860 (537–1,091)	176 (147–202)	269 (217–328)	79 (74–84)
Venezuela	Number: Cumulative change (%)	-20.7 (-24.6 to -17.0)	-36.9 (-45.8 to -27.6)	-39.3 (-47.4 to -30.3)	-40.0 (-53.2 to -24.7)	-74.8 (-78.7 to -70.4)	20.7 (1.0–40.0)	-71.6 (-83.4 to -55.1)	-6.6 (-27.2 to 14.0)	-62.3 (-69.2 to -55.4)	-33.1 (-50.9 to -13.4)	-3.8 (-12.2 to 5.2)
Venezuela	Rate: 1990	199.9 (198.8–201.0)	34.2 (30.6–37.8)	19.3 (17.8–20.9)	13.0 (10.7–15.6)	24.0 (22.3–25.8)	19.2 (17.0–22.9)	0.1 (0.1–0.2)	8.9 (5.1–11.6)	4.5 (4.1–5.0)	3.9 (3.0–4.8)	0.8 (0.8–0.9)
Venezuela	Rate: 2015	142.8 (135.9–149.5)	20.7 (17.7–24.1)	10.9 (9.7–12.2)	7.4 (6.2–8.8)	5.7 (4.9–6.6)	21.5 (19.3–23.9)	0.0 (0.0–0.0)	7.8 (4.9–9.9)	1.6 (1.3–1.8)	2.4 (2.0–3.0)	0.7 (0.7–0.7)
Venezuela	Rate: Cumulative change (%)	-28.6 (-32.1 to -25.2)	-39.5 (-48.0 to -30.6)	-43.5 (-51.1 to -35.1)	-42.5 (-55.1 to -27.8)	-76.3 (-80.0 to -72.1)	13.1 (-5.3 to 31.1)	-75.7 (-85.8 to -62.1)	-10.6 (-30.2 to 9.2)	-65.4 (-71.7 to -59.1)	-35.9 (-53.0 to -17.0)	-13.1 (-20.6 to -5.0)
Andean Latin America	Number: 1990	107,369 (105,596–109,094)	8,778 (7,436–10,263)	24,661 (22,766–26,749)	4,969 (3,995–6,032)	13,822 (12,412–15,286)	3,676 (3,028–5,795)	50 (41–62)	4,127 (2,962–6,560)	1,412 (1,108–1,777)	1,557 (1,152–2,064)	25 (19–33)
Andean Latin America	Number: 2015	30,164 (28,206–32,153)	3,123 (2,542–3,663)	4,242 (3,791–4,766)	2,508 (1,960–3,073)	1,012 (815–1,233)	3,657 (2,904–4,313)	2 (2–3)	2,472 (1,874–3,481)	413 (345–511)	642 (448–886)	22 (13–37)
Andean Latin America	Number: Cumulative change (%)	-71.9 (-73.8 to -70.0)	-64.2 (-70.9 to -55.8)	-82.7 (-85.3 to -80.0)	-49.1 (-59.8 to -36.5)	-92.6 (-94.3 to -90.7)	4.0 (-41.1 to 28.8)	-94.0 (-95.5 to -92.2)	-38.3 (-53.7 to -16.6)	-70.0 (-78.2 to -57.7)	-57.5 (-73.0 to -36.8)	-13.3 (-39.2 to 18.8)
Andean Latin America	Rate: 1990	508.1 (499.7–516.1)	39.8 (33.7–46.6)	115.2 (106.4–124.8)	22.6 (18.1–27.4)	65.0 (58.4–71.8)	17.0 (14.0–26.9)	0.3 (0.2–0.3)	18.8 (13.5–29.8)	6.8 (5.3–8.5)	7.1 (5.2–9.4)	0.1 (0.1–0.2)
Andean Latin America	Rate: 2015	135.9 (127.1–145.0)	14.2 (11.6–16.7)	19.1 (17.1–21.5)	11.4 (8.9–14.0)	4.6 (3.7–5.5)	16.5 (13.1–19.5)	0.0 (0.0–0.0)	11.2 (8.5–15.8)	1.9 (1.6–2.3)	2.9 (2.0–4.0)	0.1 (0.1–0.2)
Andean Latin America	Rate: Cumulative change (%)	-73.2 (-75.1 to -71.4)	-64.1 (-70.9 to -55.7)	-83.4 (-85.8 to -80.7)	-49.0 (-59.8 to -36.4)	-93.0 (-94.5 to -91.1)	1.4 (-42.6 to 25.7)	-94.6 (-96.0 to -93.0)	-38.3 (-53.8 to -16.6)	-71.8 (-79.4 to -60.5)	-57.6 (-73.1 to -36.8)	-20.1 (-44.2 to 9.9)

© 2017 Global Burden of Disease Child and Adolescent Health Collaboration. JAMA Pediatrics.

eTable 1. Number of Deaths, Death Rates (per 100,000 population), and Cumulative Percent Change with 95% Uncertainty Intervals (UI) for the Top 10 Global Causes of Death in 195 Countries and Territories, Aged 0 to 19, Both Sexes, 1990 and 2015*Note: best viewed by enlarging in browser.*

Location	Measure	All causes	Neonatal preterm birth complications	Lower respiratory infections	Neonatal encephalopathy due to birth asphyxia and trauma	Diarrheal diseases	Congenital anomalies	Malaria	Neonatal sepsis and other neonatal infections	Meningitis	Other neonatal disorders	HIV/AIDS
Bolivia	Number: 1990	30,529 (29,837–31,272)	2,400 (1,689–3,128)	5,946 (4,986–6,960)	1,980 (1,279–2,763)	5,882 (4,705–7,103)	851 (476–1,695)	0 (0–1)	1,770 (968–2,982)	705 (445–1,113)	516 (229–929)	0 (0–1)
Bolivia	Number: 2015	9,415 (8,259–10,758)	971 (631–1,386)	1,116 (862–1,435)	1,092 (676–1,557)	510 (338–726)	718 (444–1,253)	0 (0–0)	987 (537–1,617)	171 (121–251)	282 (119–498)	1 (0–1)
Bolivia	Number: Cumulative change (%)	-69.2 (-73.1 to -64.8)	-58.6 (-74.4 to -38.9)	-81.0 (-86.4 to -74.3)	-42.6 (-66.2 to -9.3)	-91.2 (-94.5 to -86.8)	-7.6 (-58.1 to 36.7)	-24.6 (-53.7 to 6.7)	-40.3 (-69.1 to 3.7)	-73.5 (-86.5 to -54.6)	-35.8 (-79.5 to 38.4)	58.7 (-11.2 to 165.9)
Bolivia	Rate: 1990	740.9 (723.8–758.8)	54.7 (38.5–71.1)	142.3 (119.4–166.6)	45.1 (29.2–63.1)	141.8 (113.5–171.2)	20.1 (11.3–39.9)	0.0 (0.0–0.0)	40.5 (22.2–68.0)	17.5 (11.2–27.3)	11.8 (5.2–21.1)	0.0 (0.0–0.0)
Bolivia	Rate: 2015	203.6 (178.7–232.6)	21.0 (13.6–29.9)	24.1 (18.6–31.0)	23.6 (14.6–33.7)	11.0 (7.3–15.6)	15.5 (9.6–27.1)	0.0 (0.0–0.0)	21.3 (11.6–34.9)	3.7 (2.6–5.4)	6.1 (2.6–10.8)	0.0 (0.0–0.0)
Bolivia	Rate: Cumulative change (%)	-72.5 (-76.0 to -68.6)	-60.7 (-75.7 to -42.0)	-82.9 (-87.7 to -76.8)	-45.5 (-67.9 to -14.1)	-92.1 (-95.1 to -88.2)	-15.3 (-61.5 to 25.1)	-41.3 (-64.7 to -16.5)	-43.6 (-70.7 to -2.2)	-76.9 (-88.1 to -60.6)	-39.3 (-80.7 to 31.1)	37.3 (-23.0 to 124.9)
Ecuador	Number: 1990	18,147 (17,576–18,723)	1,659 (1,461–1,879)	3,270 (2,975–3,558)	645 (524–781)	2,786 (2,508–3,086)	979 (862–1,117)	37 (28–48)	431 (203–575)	251 (221–287)	409 (328–500)	3 (1–7)
Ecuador	Number: 2015	7,929 (7,198–8,749)	989 (802–1,200)	1,240 (1,062–1,454)	376 (289–476)	246 (197–307)	1,205 (790–1,499)	1 (0–1)	303 (197–396)	92 (71–127)	239 (183–303)	6 (4–11)
Ecuador	Number: Cumulative change (%)	-56.3 (-60.8 to -51.2)	-40.1 (-51.2 to -27.1)	-62.0 (-68.0 to -54.7)	-41.0 (-56.3 to -22.6)	-91.1 (-93.0 to -88.7)	24.0 (-25.1 to 57.1)	-96.9 (-98.0 to -95.5)	-26.2 (-49.7 to 8.3)	-63.0 (-71.8 to -49.8)	-40.8 (-57.1 to -20.4)	97.8 (-5.9 to 257.8)
Ecuador	Rate: 1990	335.2 (324.9–345.3)	29.5 (26.0–33.4)	59.4 (54.1–64.6)	11.5 (9.3–13.9)	50.7 (45.7–56.1)	17.7 (15.6–20.2)	0.7 (0.6–0.9)	7.7 (3.6–10.2)	4.7 (4.1–5.3)	7.3 (5.8–8.9)	0.1 (0.0–0.1)
Ecuador	Rate: 2015	129.1 (117.1–142.5)	16.3 (13.2–19.7)	20.2 (17.3–23.7)	6.2 (4.8–7.8)	4.0 (3.2–5.0)	19.7 (12.9–24.5)	0.0 (0.0–0.0)	5.0 (3.2–6.5)	1.5 (1.2–2.1)	3.9 (3.0–5.0)	0.1 (0.1–0.2)
Ecuador	Rate: Cumulative change (%)	-61.5 (-65.5 to -56.9)	-44.6 (-54.8 to -32.5)	-65.9 (-71.3 to -59.4)	-45.4 (-59.6 to -28.4)	-92.0 (-93.8 to -89.9)	12.3 (-32.1 to 42.0)	-97.5 (-98.3 to -96.3)	-31.7 (-53.5 to 0.1)	-67.4 (-75.1 to -56.0)	-45.3 (-60.4 to -26.4)	70.1 (-19.8 to 205.5)
Peru	Number: 1990	58,691 (57,220–60,220)	4,718 (3,724–5,929)	15,444 (14,029–17,002)	2,343 (1,833–2,961)	5,153 (4,491–5,821)	1,844 (1,465–2,898)	12 (8–16)	1,925 (1,290–3,416)	455 (379–591)	631 (444–921)	21 (15–28)
Peru	Number: 2015	12,819 (11,620–14,273)	1,161 (881–1,470)	1,885 (1,643–2,146)	1,038 (780–1,337)	255 (207–316)	1,734 (1,277–2,123)	1 (1–1)	1,181 (890–1,639)	148 (107–182)	120 (87–157)	14 (6–27)
Peru	Number: Cumulative change (%)	-78.2 (-80.2 to -75.6)	-74.9 (-81.8 to -66.3)	-87.8 (-89.7 to -85.6)	-55.1 (-67.1 to -40.4)	-95.0 (-96.1 to -93.6)	-1.0 (-51.1 to 28.3)	-88.4 (-92.2 to -83.9)	-35.2 (-56.4 to -8.4)	-66.4 (-80.6 to -55.9)	-79.9 (-88.2 to -70.1)	-33.3 (-61.4 to 6.7)
Peru	Rate: 1990	506.8 (494.3–519.8)	39.3 (31.0–49.4)	131.9 (119.9–144.8)	19.5 (15.3–24.6)	44.4 (38.7–50.1)	15.6 (12.4–24.7)	0.1 (0.1–0.1)	16.0 (10.8–28.5)	4.0 (3.3–5.2)	5.3 (3.7–7.7)	0.2 (0.1–0.3)
Peru	Rate: 2015	112.2 (101.5–125.0)	10.3 (7.8–13.0)	16.5 (14.4–18.7)	9.2 (6.9–11.9)	2.2 (1.8–2.8)	15.2 (11.2–18.7)	0.0 (0.0–0.0)	10.5 (7.9–14.5)	1.3 (0.9–1.6)	1.1 (0.8–1.4)	0.1 (0.1–0.2)
Peru	Rate: Cumulative change (%)	-77.9 (-79.9 to -75.3)	-73.3 (-80.6 to -64.1)	-87.5 (-89.4 to -85.2)	-52.2 (-64.9 to -36.5)	-94.9 (-96.1 to -93.6)	2.6 (-49.4 to 33.0)	-88.9 (-92.5 to -84.7)	-31.2 (-53.7 to -2.8)	-66.5 (-80.6 to -56.1)	-78.7 (-87.4 to -68.2)	-34.9 (-62.7 to 4.8)
Caribbean	Number: 1990	73,033 (70,688–75,462)	7,303 (6,464–8,178)	8,756 (7,505–10,086)	4,272 (3,400–5,195)	14,855 (12,884–17,115)	4,551 (3,586–5,624)	160 (70–284)	2,378 (1,713–3,360)	2,687 (2,001–3,737)	2,538 (1,686–3,613)	775 (588–997)
Caribbean	Number: 2015	32,608 (28,458–37,518)	3,476 (2,893–4,172)	3,652 (2,783–4,690)	2,384 (1,758–3,120)	2,913 (2,031–4,128)	3,669 (2,860–4,547)	36 (14–67)	1,959 (1,399–2,631)	932 (600–1,434)	1,544 (1,002–2,281)	933 (793–1,075)
Caribbean	Number: Cumulative change (%)	-55.3 (-61.4 to -48.4)	-52.2 (-61.3 to -42.3)	-58.0 (-69.6 to -44.3)	-43.4 (-60.2 to -23.4)	-80.2 (-86.5 to -71.7)	-18.0 (-45.9 to 4.4)	-74.3 (-90.4 to -48.7)	-15.6 (-40.5 to 19.8)	-64.0 (-80.1 to -42.6)	-36.6 (-62.9 to 2.0)	22.9 (-6.2 to 57.0)
Caribbean	Rate: 1990	455.0 (440.5–470.1)	44.7 (39.5–50.0)	54.3 (46.6–62.6)	26.1 (20.8–31.8)	92.1 (79.9–106.1)	28.2 (22.2–34.8)	1.0 (0.4–1.8)	14.6 (10.5–20.6)	16.7 (12.5–23.3)	15.5 (10.3–22.1)	4.8 (3.7–6.2)
Caribbean	Rate: 2015	222.5 (193.9–256.6)	24.3 (20.2–29.2)	25.2 (19.2–32.4)	16.7 (12.3–21.8)	20.2 (14.1–28.6)	25.3 (19.8–31.3)	0.2 (0.1–0.5)	13.7 (9.8–18.4)	6.4 (4.1–9.9)	10.8 (7.0–16.0)	6.1 (5.2–7.0)
Caribbean	Rate: Cumulative change (%)	-51.1 (-57.8 to -43.4)	-45.4 (-55.8 to -34.0)	-53.3 (-66.3 to -37.9)	-35.3 (-54.5 to -12.4)	-77.9 (-85.0 to -68.3)	-8.9 (-39.7 to 15.7)	-72.6 (-89.8 to -44.5)	-3.6 (-32.1 to 36.8)	-60.3 (-78.0 to -37.0)	-27.7 (-57.6 to 16.6)	29.2 (-1.4 to 64.9)
Antigua and Barbuda	Number: 1990	31 (26–36)	6 (5–8)	3 (2–3)	1 (1–2)	1 (1–1)	3 (2–4)	0 (0–0)	0 (0–2)	0 (0–0)	0 (0–1)	0 (0–0)
Antigua and Barbuda	Number: 2015	27 (22–34)	4 (3–7)	2 (1–3)	1 (0–2)	0 (0–0)	3 (2–5)	0 (0–0)	1 (0–2)	0 (0–0)	1 (0–1)	0 (0–0)
Antigua and Barbuda	Number: Cumulative change (%)	-10.2 (-30.3 to 16.1)	-27.9 (-53.6 to 11.5)	-14.6 (-38.2 to 14.2)	-3.2 (-41.5 to 50.8)	-56.6 (-69.4 to -41.0)	18.8 (-15.6 to 60.3)	nan (nan–nan)	21.8 (-24.6 to 90.2)	-53.0 (-64.2 to -40.0)	45.4 (-13.0 to 133.0)	164.8 (141.2–190.2)
Antigua and Barbuda	Rate: 1990	139.6 (117.9–164.7)	31.2 (23.2–39.8)	13.6 (11.2–16.5)	7.1 (5.0–9.4)	7.1 (5.7–8.9)	15.3 (12.1–18.8)	0.0 (0.0–0.0)	4.4 (2.3–9.4)	2.7 (2.3–3.3)	4.0 (2.9–5.4)	0.6 (0.6–0.7)
Antigua and Barbuda	Rate: 2015	96.9 (77.7–121.4)	17.7 (11.7–26.0)	9.1 (7.0–11.6)	5.3 (3.4–7.9)	2.5 (1.8–3.1)	14.4 (10.6–18.9)	0.0 (0.0–0.0)	4.1 (2.1–8.3)	1.0 (0.8–1.3)	4.6 (3.0–6.7)	1.3 (1.2–1.4)
Antigua and Barbuda	Rate: Cumulative change (%)	-30.0 (-46.2 to -9.0)	-42.3 (-62.9 to -10.8)	-32.1 (-51.2 to -9.0)	-22.5 (-53.2 to 20.7)	-65.1 (-75.5 to -52.4)	-5.1 (-32.9 to 28.9)	nan (nan–nan)	-2.6 (-39.7 to 52.1)	-63.0 (-71.9 to -52.5)	16.4 (-30.4 to 86.6)	106.4 (88.0–126.5)
The Bahamas	Number: 1990	261 (206–327)	53 (38–71)	24 (16–32)	26 (17–38)	5 (3–7)	21 (14–30)	0 (0–0)	15 (9–22)	3 (2–5)	19 (11–29)	2 (1–3)
The Bahamas	Number: 2015	136 (100–190)	21 (11–33)	10 (7–15)	13 (7–21)	1 (0–1)	16 (10–24)	0 (0–0)	9 (5–16)	1 (0–1)	10 (5–17)	0 (0–1)
The Bahamas	Number: Cumulative change (%)	-46.7 (-64.6 to -21.9)	-59.5 (-78.8 to -30.6)	-53.9 (-71.8 to -29.0)	-48.6 (-75.7 to -7.3)	-77.9 (-88.0 to -63.5)	-20.4 (-55.9 to 31.6)	nan (nan–nan)	-34.3 (-66.4 to 13.5)	-65.4 (-79.2 to -43.4)	-40.6 (-73.0 to 7.4)	-75.7 (-83.6 to -68.2)
The Bahamas	Rate: 1990	234.1 (184.8–293.4)	48.0 (34.1–64.0)	21.6 (15.2–29.5)	23.9 (15.8–34.0)	4.9 (3.2–7.2)	19.3 (13.0–27.3)	0.0 (0.0–0.0)	13.5 (8.3–20.3)	3.4 (2.3–4.6)	17.4 (10.7–26.6)	2.2 (1.4–3.5)
The Bahamas	Rate: 2015	124.3 (90.7–174.9)	19.8 (11.2–31.8)	9.9 (6.8–14.4)	12.3 (6.8–20.2)	1.1 (0.7–1.6)	15.2 (10.0–22.9)	0.0 (0.0–0.0)	8.9 (4.8–15.4)	1.1 (0.8–1.6)	10.2 (5.6–16.7)	0.5 (0.2–1.0)
The Bahamas	Rate: Cumulative change (%)	-45.9 (-64.5 to -20.1)	-57.5 (-77.7 to -27.1)	-52.5 (-71.0 to -26.6)	-46.0 (-74.5 to -2.7)	-77.1 (-87.6 to -62.2)	-17.3 (-54.3 to 37.0)	nan (nan–nan)	-31.1 (-64.8 to 19.0)	-64.8 (-78.8 to -42.1)	-37.7 (-71.7 to 12.7)	-76.7 (-84.8 to -69.7)
Barbados	Number: 1990	151 (118–193)	25 (17–34)	13 (10–19)	18 (12–25)	3 (2–4)	21 (15–28)	0 (0–0)	8 (4–16)	2 (2–3)	5 (3–7)	0 (0–0)
Barbados	Number: 2015	78 (56–110)	10 (5–17)	6 (4–8)	7 (4–12)	0 (0–1)	15 (9–22)	0 (0–0)	6 (3–12)	0 (0–0)	3 (1–6)	0 (0–0)
Barbados	Number: Cumulative change (%)	-46.8 (-64.6 to -21.7)	-57.6 (-78.4 to -28.2)	-54.9 (-73.0 to -29.3)	-55.4 (-77.8 to -21.8)	-78.3 (-88.1 to -63.5)	-26.5 (-56.9 to 17.9)	nan (nan–nan)	-23.1 (-59.0 to 33.3)	-74.7 (-83.4 to -63.1)	-25.8 (-64.2 to 38.6)	38.3 (26.5–50.6)
Barbados	Rate: 1990	188.5 (145.2–242.4)	33.9 (23.5–45.8)	17.5 (12.6–24.2)	24.1 (16.3–33.2)	4.3 (2.9–6.4)	27.2 (19.6–37.1)	0.0 (0.0–0.0)	11.4 (6.5–21.0)	3.3 (2.5–4.4)	6.8 (4.5–9.7)	0.8 (0.8–0.9)
Barbados	Rate: 2015	118.8 (83.9–168.7)	16.8 (9.2–27.6)	9.2 (6.3–13.5)	12.5 (6.9–20.4)	1.1 (0.7–1.6)	23.5 (15.0–35.4)	0.0 (0.0–0.0)	10.1 (5.2–19.2)	1.0 (0.7–1.3)	5.8 (3.1–9.6)	1.4 (1.3–1.4)
Barbados	Rate: Cumulative change (%)	-35.4 (-57.9 to -3.3)	-48.3 (-73.7 to -12.4)	-45.4 (-68.0 to -13.3)	-45.6 (-72.9 to -4.7)	-73.6 (-85.7 to -55.4)	-10.4 (-48.0 to 44.8)	nan (nan–nan)	-6.3 (-50.1 to 62.4)	-69.6 (-80.2 to -55.4)	-9.5 (-56.4 to 69.1)	66.4 (52.0–81.9)
Belize	Number: 1990	309 (274–349)	57 (46–66)	42 (35–50)	22 (17–28)	22 (17–28)	22 (17–31)	0 (0–0)	14 (9–23)	5 (4–7)	6 (5–8)	0 (0–0)
Belize	Number: 2015	202 (158–264)	24 (15–38)	19 (14–26)	15 (8–23)	5 (3–7)	22 (15–32)	0 (0–0)	12 (7–20)	2 (1–3)	6 (3–10)	6 (1–12)
Belize	Number: Cumulative change (%)	-34.1 (-50.9 to -12.5)	-56.3 (-73.7 to -32.3)	-53.6 (-67.6 to -34.3)	-31.8 (-62.6 to 7.0)	-75.6 (-84.5 to -63.0)	1.5 (-44.5 to 54.5)	-74.6 (-85.8 to -57.9)	-5.5 (-43.3 to 42.5)	-61.9 (-75.4 to -41.2)	-1.9 (-46.4 to 59.7)	2,627.7 (1,466.7–4,193.6)
Belize	Rate: 1990	266.0 (237.0–298.9)	46.8 (38.2–54.9)	36.0 (29.7–42.4)	18.7 (14.5–23.5)	18.9 (14.9–23.5)	19.3 (14.9–26.2)	0.2 (0.1–0.2)	11.5 (8.1–19.1)	4.9 (4.0–6.0)	5.6 (4.1–7.3)	0.2 (0.1–0.4)
Belize	Rate: 2015	132.9 (103.7–173.9)	16.5 (10.1–25.4)	13.0 (9.6–17.8)	10.2 (5.9–15.6)	3.6 (2.5–5.1)	15.0 (10.1–21.5)	0.0 (0.0–0.0)	8.5 (5.1–13.7)	1.4 (1.0–2.0)	4.4 (2.5–6.8)	3.9 (0.9–8.0)
Belize	Rate: Cumulative change (%)	-49.7 (-62.5 to -33.2)	-64.5 (-78.6 to -45.0)	-63.5 (-74.5 to -48.2)	-44.7 (-69.6 to -13.1)	-80.5 (-87.6 to -70.5)	-19.7 (-55.8 to 22.5)	-82.6 (-90.2 to -71.6)	-23.4 (-54.0 to 15.5)	-70.8 (-81.1 to -54.8)	-20.4 (-56.5 to 29.5)	1,960.5 (1,050.3–3,174.5)
Bermuda	Number: 1990	18 (16–20)	2 (1–2)	1 (0–1)	1 (1–1)	0 (0–0)	1 (1–2)	0 (0–0)	0 (0–0)	0 (0–0)	1 (0–1)	0 (0–0)
Bermuda	Number: 2015	7 (6–8)	0 (0–0)	0 (0–0)	0 (0–0)	0 (0–0)	1 (0–1)	0 (0–0)	0 (0–0)	0 (0–0)	0 (0–0)	0 (0–0)
Bermuda	Number: Cumulative change (%)	-59.1 (-67.0 to -49.4)	-85.0 (-91.5 to -76.4)	-70.9 (-78.1 to -61.9)	-63.9 (-79.4 to -41.5)	-86.3 (-90.9 to -80.3)	-26.1 (-51.2 to 1.9)	-37.0 (-55.7 to -17.1)	-59.0 (-74.8 to -38.2)	-83.0 (-86.8 to -78.6)	-50.6 (-70.8 to -23.5)	29.3 (17.9–41.0)
Bermuda	Rate: 1990	96.0 (84.0–109.6)	13.6 (10.4–17.6)	6.4 (5.1–7.6)	8.9 (6.6–11.6)	1.4 (1.1–1.8)	10.4 (8.0–16.4)	0.0 (0.0–0.0)	1.7 (1.1–3.1)	1.5 (1.3–1.8)	8.1 (5.9–10.7)	0.8 (0.7–0.8)
Bermuda	Rate: 2015	39.9 (32.4–49.4)	2.2 (1.3–3.2)	1.9 (1.4–2.5)	3.4 (2.1–5.1)	0.2 (0.1–0.3)	8.1 (5.8–11.3)	0.0 (0.0–0.0)	0.7 (0.4–1.3)	0.3 (0.2–0.3)	4.2 (2.6–6.2)	1.0 (1.0–1.1)
Bermuda	Rate: Cumulative change (%)	-58.2 (-67.6 to -46.9)	-83.8 (-90.9 to -74.6)	-69.4 (-77.7 to -58.8)	-61.1 (-77.9 to -37.0)	-85.2 (-90.4 to -78.3)	-19.8 (-48.4 to 12.3)	-36.1 (-55.1 to -15.8)	-55.8 (-72.8 to -33.5)	-82.4 (-86.6 to -77.2)	-46.8 (-68.6 to -17.5)	35.5 (24.2–47.4)
Cuba	Number: 1990	4,030 (3,999–4,061)	371 (306–440)	311 (284–340)	329 (275–389)	126 (109–145)	580 (515–658)	0 (0–0)	83 (22–135)	197 (180–214)	73 (57–92)	6 (6–7)
Cuba	Number: 2015	1,223 (1,191–1,259)	77 (64–92)	80 (71–91)	66 (55–79)	15 (12–18)	238 (211–296)	0 (0–0)	31 (10–46)	19 (17–21)	84 (71–101)	10 (10–11)
Cuba	Number: Cumulative change (%)	-69.6 (-70.5 to -68.7)	-79.0 (-83.1 to -74.3)	-73.9 (-77.7 to -69.8)	-79.5 (-84.0 to -74.3)	-87.8 (-90.3 to -84.9)	-58.7 (-64.9 to -49.5)	nan (nan–nan)	-60.2 (-71.7 to -47.7)	-90.0 (-91.2 to -88.6)	17.3 (-12.6 to 52.0)	63.7 (49.0–78.6)
Cuba	Rate: 1990	114.9 (113.9–115.8)	11.4 (9.4–13.6)	9.3 (8.5–10.2)	10.1 (8.5–12.0)	3.8 (3.3–4.4)	17.5 (15.5–19.8)	0.0 (0.0–0.0)	2.6 (0.7–4.2)	5.9 (5.4–6.4)	2.3 (1.8–2.8)	0.2 (0.2–0.2)
Cuba	Rate: 2015	51.7 (50.3–53.4)	3.7 (3.1–4.4)	3.6 (3.2–4.1)	3.2 (2.6–3.8)	0.7 (0.6–0.8)	10.8 (9.5–13.4)	0.0 (0.0–0.0)	1.5 (0.5–2.2)	0.9 (0.8–1.0)	4.0 (3.4–4.8)	0.5 (0.4–0.5)
Cuba	Rate: Cumulative change (%)	-55.0 (-56.2 to -53.5)	-67.8 (-74.1 to -60.5)	-61.0 (-66.9 to -54.7)	-68.5 (-75.4 to -60.5)	-81.7 (-85.4 to -77.4)	-38.2 (-47.4 to -24.3)	nan (nan–nan)	-38.9 (-56.6 to -19.6)	-85.4 (-87.3 to -83.3)	80.2 (34.3–133.4)	141.5 (119.5–164.0)
Dominica	Number: 1990	53 (44–64)	8 (5–11)	4 (3–5)	5 (3–6)	3 (2–4)	5 (4–7)	0 (0–0)	3 (2–7)	0 (0–1)	1 (0–1)	0 (0–0)
Dominica	Number: 2015	41 (31–53)	5 (3–7)	3 (2–5)	4 (2–5)	1 (0–1)	5 (3–7)	0 (0–0)	3 (2–6)	0 (0–0)	1 (0–1)	0 (0–0)
Dominica	Number: Cumulative change (%)	-23.0 (-43.9 to 5.7)	-30.9 (-61.0 to 12.8)	-21.7 (-49.5 to 19.4)	-15.8 (-50.3 to 31.6)	-63.7 (-78.6 to -42.1)	-11.4 (-45.1 to 34.2)	nan (nan–nan)	7.8 (-39.0 to 71.6)	-46.6 (-66.0 to -15.3)	2.3 (-45.3 to 74.4)	212.0 (3.0–872.7)
Dominica	Rate: 1990	178.1 (148.0–212.4)	28.1 (18.4–38.5)	15.2 (11.9–19.0)	16.7 (11.8–22.4)	12.1 (9.2–15.6)	19.0 (14.5–24.5)	0.0 (0.0–0.0)	12.1 (6.8–24.1)	2.8 (2.2–3.5)	4.6 (2.9–6.6)	0.3 (0.1–0.6)
Dominica	Rate: 2015	187.6 (143.3–247.5)	27.0 (17.5–38.5)	16.6 (11.5–23.6)	19.9 (12.7–27.6)	6.2 (4.0–9.3)	23.6 (14.3–35.0)	0.0 (0.0–0.0)	17.7 (10.6–29.3)	2.0 (1.4–2.9)	6.4 (4.0–9.6)	0.9 (0.5–1.4)
Dominica	Rate: Cumulative change (%)	6.6 (-23.0 to 47.2)	1.0 (-42.9 to 64.8)	11.2 (-28.7 to 70.1)	23.1 (-27.4 to 92.5)	-47.6 (-69.3 to -16.0)	26.6 (-22.1 to 92.1)	nan (nan–nan)	57.6 (-10.9 to 150.7)	-26.2 (-53.4 to 16.8)	49.7 (-20.1 to 155.1)	305.8 (34.5–1,165.3)
Dominican Republic	Number: 1990	14,881 (14,232–15,586)	2,374 (2,043–2,709)	1,206 (1,059–1,366)	1,026 (821–1,256)	2,539 (2,250–2,822)	996 (587–1,335)	2 (1–3)	887 (677–1,286)	515 (393–604)	491 (372–627)	31 (23–39)
Dominican Republic	Number: 2015	7,041 (6,182–8,044)	1,202 (953–1,483)	615 (502–753)	446 (326–602)	231 (174–304)	916 (606–1,184)	0 (0–0)	841 (625–1,117)	158 (124–204)	220 (159–303)	107 (73–145)
Dominican Republic	Number: Cumulative change (%)	-52.6 (-58.8 to -45.8)	-49.1 (-60.3 to -37.1)	-48.7 (-59.7 to -35.3)	-56.0 (-69.1 to -39.8)	-90.8 (-93.2 to -87.8)	-4.9 (-43.7 to 23.7)	-74.1 (-82.9 to -63.0)	-3.1 (-29.4 to 31.3)	-68.5 (-77.8 to -53.6)	-54.3 (-69.4 to -35.1)	246.8 (167.1–339.8)
Dominican Republic	Rate: 1990	391.5 (374.5–409.8)	60.9 (52.5–69.5)	31.6 (27.8–35.8)	26.3 (21.1–32.2)	66.4 (58.8–73.7)	26.0 (15.4–34.9)	0.1 (0.1–0.1)	22.8 (17.4–33.1)	13.6 (10.4–15.9)	12.6 (9.6–16.1)	0.8 (0.6–1.1)
Dominican Republic	Rate: 2015	175.6 (154.1–200.9)	30.5 (24.2–37.6)	15.4 (12.5–18.8)	11.3 (8.3–15.3)	5.8 (4.3–7.6)	23.0 (15.2–29.8)	0.0 (0.0–0.0)	21.3 (15.8–28.3)	3.9 (3.1–5.1)	5.6 (4.0–7.7)	2.6 (1.8–3.6)
Dominican Republic	Rate: Cumulative change (%)	-55.1 (-61.0 to -48.6)	-49.8 (-60.8 to -37.9)	-51.2 (-61.7 to -38.4)	-56.6 (-69.5 to -40.6)	-91.3 (-93.5 to -88.3)	-8.5 (-46.1 to 19.1)	-77.4 (-85.0 to -67.7)	-4.5 (-30.4 to 29.4)	-70.3 (-79.1 to -56.2)	-54.9 (-69.8 to -35.9)	218.5 (144.5–304.7)
Grenada	Number: 1990	99 (72–133)	16 (10–23)	13 (9–20)	9 (6–14)	4 (2–6)	10 (6–15)	0 (0–0)	4 (2–8)	1 (1–2)	2 (1–4)	0 (0–0)
Grenada	Number: 2015	48 (35–68)	5 (2–9)	5 (3–8)	4 (2–7)	0 (0–1)	6 (4–9)	0 (0–0)	2 (1–5)	0 (0–0)	1 (0–2)	0 (0–0)
Grenada	Number: Cumulative change (%)	-49.4 (-68.8 to -24.5)	-62.0 (-82.5 to -30.2)	-56.4 (-76.2 to -30.7)	-51.9 (-78.7 to -11.9)	-80.8 (-90.3 to -66.8)	-33.6 (-66.1 to 10.3)	nan (nan–nan)	-32.2 (-69.2 to 24.8)	-69.5 (-83.1 to -49.2)	-40.5 (-75.3 to 16.3)	189.1 (3.1–740.8)
Grenada	Rate: 1990	197.6 (146.5–262.8)	31.5 (19.6–44.9)	26.6 (18.1–38.2)	19.2 (11.8–27.7)	8.0 (5.1–12.1)	19.9 (12.9–29.5)	0.0 (0.0–0.0)	9.2 (5.1–16.1)	3.5 (2.4–4.8)	5.8 (3.2–8.9)	0.4 (0.1–0.7)
Grenada	Rate: 2015	128.0 (91.8–179.9)	15.5 (8.0–26.5)	15.1 (10.2–22.3)	11.9 (6.2–19.9)	2.0 (1.3–3.1)	16.9 (10.9–25.6)	0.0 (0.0–0.0)	7.9 (3.9–13.7)	1.3 (0.9–1.9)	4.4 (2.2–7.6)	1.0 (0.6–1.7)
Grenada	Rate: Cumulative change (%)	-33.2 (-58.4 to -0.3)	-47.8 (-76.0 to -4.1)	-40.1 (-67.3 to -5.4)	-33.9 (-70.7 to 21.1)	-73.1 (-86.3 to -53.4)	-9.4 (-53.6 to 49.5)	nan (nan–nan)	-6.9 (-57.8 to 71.3)	-59.1 (-76.8 to -32.5)	-18.1 (-66.1 to 60.0)	309.5 (45.3–1,091.2)
Guyana	Number: 1990	1,510 (1,437–1,582)	279 (212–322)	140 (123–158)	103 (81–124)	225 (197–254)	93 (78–117)	11 (8–14)	71 (45–148)	15 (13–18)	44 (32–57)	3 (1–7)
Guyana	Number: 2015	624 (546–721)	82 (62–101)	42 (33–54)	51 (37–65)	31 (22–42)	56 (43–70)	2 (1–3)	45 (31–70)	4 (3–5)	29 (20–40)	30 (13–53)
Guyana	Number: Cumulative change (%)	-58.6 (-64.4 to -52.3)	-70.3 (-77.4 to -62.0)	-69.4 (-76.5 to -61.3)	-49.8 (-64.2 to -32.5)	-86.1 (-90.1 to -81.0)	-38.2 (-58.1 to -17.7)	-79.2 (-89.1 to -65.1)	-30.4 (-57.4 to 3.6)	-72.4 (-80.3 to -60.7)	-32.5 (-56.3 to -2.8)	869.2 (407.7–1,384.9)
Guyana	Rate: 1990	365.3 (348.1–381.9)	64.3 (48.9–74.4)	33.5 (29.5–37.7)	23.8 (18.9–28.7)	52.9 (46.4–59.7)	22.2 (18.7–27.9)	2.9 (2.2–3.8)	16.6 (10.5–34.2)	3.9 (3.3–4.5)	10.3 (7.5–13.3)	0.8 (0.3–1.7)
Guyana	Rate: 2015	212.3 (183.5–247.4)	30.3 (23.1–37.4)	15.3 (12.0–19.5)	18.8 (13.9–24.3)	11.5 (8.4–15.5)	20.6 (15.9–25.8)	0.7 (0.4–1.1)	16.9 (11.7–26.1)	1.5 (1.1–2.0)	10.9 (7.7–14.9)	9.7 (4.0–16.9)
Guyana	Rate: Cumulative change (%)	-41.8 (-50.4 to -32.4)	-52.5 (-63.8 to -39.3)	-54.1 (-65.0 to -41.6)	-19.7 (-42.7 to 8.1)	-78.2 (-84.5 to -70.2)	-5.5 (-35.9 to 26.4)	-75.7 (-87.1 to -60.7)	11.1 (-32.1 to 65.3)	-61.3 (-72.3 to -45.3)	8.1 (-30.1 to 55.6)	1,196.0 (582.1–1,908.8)
Haiti	Number: 1990	44,097 (41,898–46,416)	2,645 (1,971–3,415)	6,310 (5,108–7,603)	2,228 (1,433–3,071)	11,190 (9,282–13,307)	2,038 (1,565–2,686)	138 (51–262)	1,042 (531–1,743)	1,773 (1,120–2,794)	1,643 (835–2,673)	682 (503–899)
Haiti	Number: 2015	19,374 (15,604–24,101)	1,418 (919–2,045)	2,590 (1,770–3,578)	1,504 (940–2,199)	2,488 (1,647–3,645)	1,844 (1,221–2,555)	32 (10–61)	788 (389–1,299)	689 (375–1,201)	1,002 (466–1,703)	728 (600–861)
Haiti	Number: Cumulative change (%)	-56.0 (-65.0 to -44.8)	-45.1 (-66.2 to -17.4)	-58.4 (-73.2 to -40.1)	-29.3 (-60.6 to 11.2)	-77.5 (-85.7 to -65.9)	-6.2 (-50.3 to 37.4)	-72.9 (-91.4 to -39.9)	-17.0 (-64.0 to 53.9)	-58.4 (-81.2 to -21.7)	-32.7 (-72.2 to 32.6)	9.4 (-18.7 to 43.8)
Haiti	Rate: 1990	998.2 (949.0–1,049.8)	55.5 (41.4–71.5)	140.7 (114.2–169.2)	46.8 (30.2–64.4)	249.0 (206.9–296.6)	45.6 (34.9–59.9)	3.3 (1.3–6.1)	22.0 (11.2–36.8)	40.1 (25.3–62.6)	34.6 (17.5–56.3)	15.6 (11.6–20.5)
Haiti	Rate: 2015	407.0 (327.9–506.6)	29.6 (19.2–42.7)	54.5 (37.2–75.3)	31.4 (19.7–45.9)	52.4 (34.7–76.8)	38.7 (25.6–53.7)	0.7 (0.2–1.3)	16.5 (8.1–27.2)	14.5 (7.9–25.3)	20.9 (9.7–35.6)	15.4 (12.7–18.2)
Haiti	Rate: Cumulative change (%)	-59.2 (-67.5 to -48.7)	-45.4 (-66.3 to -17.9)	-60.7 (-74.7 to -43.5)	-29.7 (-60.8 to 10.5)	-78.7 (-86.5 to -67.7)	-12.2 (-52.8 to 27.7)	-76.0 (-92.3 to -46.8)	-17.7 (-64.4 to 52.9)	-61.2 (-82.5 to -27.5)	-33.4 (-72.4 to 30.5)	0.7 (-24.8 to 31.8)
Jamaica	Number: 1990	2,142 (1,792–2,545)	482 (378–599)	174 (138–220)	127 (91–172)	196 (149–257)	229 (160–300)	0 (0–0)	81 (47–163)	42 (34–52)	54 (39–73)	5 (2–8)
Jamaica	Number: 2015	1,219 (936–1,597)	258 (159–380)	69 (51–93)	91 (55–138)	18 (12–26)	181 (112–268)	0 (0–0)	77 (44–133)	15 (10–24)	32 (20–49)	2 (1–3)
Jamaica	Number: Cumulative change (%)	-42.4 (-58.8 to -22.6)	-45.6 (-68.1 to -17.4)	-59.2 (-72.1 to -40.6)	-25.3 (-60.3 to 20.7)	-90.4 (-93.9 to -85.5)	-19.3 (-50.1 to 19.1)	nan (nan–nan)	3.2 (-41.9 to 69.9)	-63.7 (-77.9 to -38.1)	-37.3 (-65.9 to 1.6)	-53.0 (-69.9 to -28.7)
Jamaica	Rate: 1990	190.5 (159.6–226.1)	43.0 (33.7–53.4)	15.4 (12.2–19.5)	11.3 (8.2–15.3)	17.3 (13.1–22.6)	20.4 (14.2–26.6)	0.0 (0.0–0.0)	7.2 (4.2–14.6)	3.8 (3.0–4.6)	4.8 (3.5–6.6)	0.5 (0.2–0.7)
Jamaica	Rate: 2015	132.0 (100.2–174.5)	29.3 (18.1–43.1)	7.7 (5.7–10.4)	10.4 (6.3–15.7)	2.0 (1.4–2.9)	20.2 (12.5–29.9)	0.0 (0.0–0.0)	8.8 (5.0–15.2)	1.6 (1.1–2.6)	3.7 (2.3–5.6)	0.3 (0.1–0.3)

© 2017 Global Burden of Disease Child and Adolescent Health Collaboration. JAMA Pediatrics.

eTable 1. Number of Deaths, Death Rates (per 100,000 population), and Cumulative Percent Change with 95% Uncertainty Intervals (UI) for the Top 10 Global Causes of Death in 195 Countries and Territories, Aged 0 to 19, Both Sexes, 1990 and 2015*Note: best viewed by enlarging in browser.*

Location	Measure	All causes	Neonatal preterm birth complications	Lower respiratory infections	Neonatal encephalopathy due to birth asphyxia and trauma	Diarrheal diseases	Congenital anomalies	Malaria	Neonatal sepsis and other neonatal infections	Meningitis	Other neonatal disorders	HIV/AIDS
Jamaica	Rate: Cumulative change (%)	-29.9 (-50.3 to -5.1)	-30.8 (-59.5 to 5.0)	-49.2 (-65.4 to -25.7)	-5.0 (-49.5 to 53.4)	-87.9 (-92.3 to -81.7)	1.5 (-37.4 to 49.9)	nan (nan–nan)	31.2 (-26.2 to 116.0)	-55.3 (-72.8 to -22.8)	-20.3 (-56.7 to 29.1)	-50.3 (-68.5 to -24.7)
Puerto Rico	Number: 1990	1,408 (1,386–1,429)	377 (349–410)	86 (77–94)	60 (50–72)	5 (4–6)	186 (163–206)	0 (0–0)	27 (9–41)	19 (17–20)	65 (53–80)	11 (10–12)
Puerto Rico	Number: 2015	511 (479–546)	78 (66–93)	20 (17–24)	17 (13–22)	4 (3–5)	81 (70–94)	0 (0–0)	21 (5–34)	3 (2–3)	36 (28–45)	3 (3–3)
Puerto Rico	Number: Cumulative change (%)	-63.7 (-65.9 to -61.2)	-79.1 (-82.3 to -75.5)	-75.6 (-79.7 to -70.6)	-70.8 (-78.1 to -62.4)	-20.7 (-39.3 to 2.0)	-55.9 (-64.1 to -44.2)	-29.1 (-40.6 to -17.9)	-25.0 (-47.1 to 5.4)	-83.6 (-86.0 to -80.8)	-43.6 (-58.7 to -26.9)	-69.6 (-72.0 to -67.1)
Puerto Rico	Rate: 1990	115.2 (113.3–117.0)	32.4 (30.0–35.2)	7.2 (6.5–7.9)	5.2 (4.3–6.2)	0.5 (0.4–0.6)	15.7 (13.7–17.5)	0.0 (0.0–0.0)	2.4 (0.8–3.5)	1.6 (1.4–1.7)	5.6 (4.6–6.9)	0.9 (0.9–1.0)
Puerto Rico	Rate: 2015	56.6 (52.8–60.8)	9.9 (8.4–11.7)	2.4 (2.1–2.9)	2.2 (1.7–2.8)	0.5 (0.4–0.6)	9.9 (8.5–11.4)	0.0 (0.0–0.0)	2.7 (0.7–4.3)	0.4 (0.3–0.4)	4.6 (3.6–5.7)	0.4 (0.4–0.4)
Puerto Rico	Rate: Cumulative change (%)	-50.9 (-54.2 to -47.2)	-69.4 (-74.1 to -64.1)	-65.8 (-71.6 to -58.4)	-57.3 (-67.9 to -44.8)	14.5 (-12.6 to 47.6)	-36.5 (-48.4 to -19.6)	-7.9 (-22.3 to 6.2)	9.9 (-22.5 to 54.5)	-77.1 (-80.6 to -73.1)	-17.3 (-39.4 to 7.1)	-58.3 (-61.7 to -54.8)
Saint Lucia	Number: 1990	154 (115–203)	31 (20–42)	17 (12–25)	11 (7–15)	9 (6–14)	12 (8–17)	0 (0–0)	7 (3–14)	3 (2–4)	4 (3–6)	0 (0–0)
Saint Lucia	Number: 2015	71 (50–103)	10 (5–16)	6 (4–9)	5 (2–9)	1 (0–1)	10 (6–16)	0 (0–0)	4 (1–9)	0 (0–1)	2 (1–4)	0 (0–0)
Saint Lucia	Number: Cumulative change (%)	-52.2 (-69.5 to -26.0)	-66.2 (-83.4 to -40.8)	-63.2 (-79.4 to -38.9)	-49.9 (-76.0 to -10.5)	-87.0 (-93.2 to -77.4)	-15.2 (-54.7 to 48.1)	nan (nan–nan)	-38.3 (-69.8 to 8.8)	-77.3 (-85.9 to -64.9)	-37.2 (-71.4 to 17.2)	37.2 (25.4–50.3)
Saint Lucia	Rate: 1990	219.8 (165.6–287.7)	44.1 (29.5–59.5)	24.7 (17.2–35.1)	15.9 (10.5–22.4)	13.8 (8.9–20.8)	17.7 (12.3–24.5)	0.0 (0.0–0.0)	10.4 (5.2–20.9)	4.7 (3.4–6.5)	6.6 (4.3–9.2)	0.5 (0.4–0.5)
Saint Lucia	Rate: 2015	131.4 (89.8–193.1)	20.2 (10.5–33.2)	11.6 (7.6–17.9)	10.8 (5.4–17.9)	2.3 (1.5–3.7)	19.4 (11.8–30.9)	0.0 (0.0–0.0)	8.5 (3.6–18.2)	1.3 (0.9–1.9)	5.5 (2.8–9.3)	0.8 (0.7–0.8)
Saint Lucia	Rate: Cumulative change (%)	-38.6 (-61.5 to -2.7)	-52.6 (-76.8 to -17.1)	-50.7 (-72.5 to -17.5)	-29.7 (-66.3 to 25.4)	-82.2 (-90.7 to -68.9)	14.8 (-39.0 to 101.3)	nan (nan–nan)	-13.7 (-57.7 to 52.2)	-70.7 (-82.1 to -54.2)	-12.0 (-60.0 to 64.2)	69.6 (55.0–85.5)
Saint Vincent and the Grenadines	Number: 1990	134 (108–165)	32 (23–41)	12 (9–16)	13 (9–17)	10 (7–14)	10 (8–14)	0 (0–0)	3 (0–13)	2 (2–3)	0 (0–0)	0 (0–0)
Saint Vincent and the Grenadines	Number: 2015	56 (43–73)	9 (6–14)	4 (2–5)	3 (1–4)	1 (1–2)	6 (4–9)	0 (0–0)	4 (2–7)	0 (0–0)	2 (1–3)	0 (0–0)
Saint Vincent and the Grenadines	Number: Cumulative change (%)	-57.4 (-69.8 to -40.9)	-69.1 (-81.3 to -53.4)	-66.7 (-79.1 to -48.0)	-74.7 (-85.5 to -60.7)	-84.4 (-90.9 to -74.7)	-39.8 (-62.4 to -6.5)	nan (nan–nan)	1,037.1 (-57.6 to 2,766.4)	-79.7 (-86.5 to -70.9)	746.9 (368.8–1,262.3)	23.0 (12.4–34.6)
Saint Vincent and the Grenadines	Rate: 1990	270.9 (216.0–332.9)	67.1 (48.5–86.1)	25.8 (18.6–33.7)	26.9 (19.1–35.1)	20.7 (14.5–28.4)	22.2 (16.6–28.7)	0.0 (0.0–0.0)	8.0 (0.3–27.6)	5.4 (4.1–6.9)	0.6 (0.4–0.8)	1.4 (1.3–1.5)
Saint Vincent and the Grenadines	Rate: 2015	165.8 (126.0–219.6)	31.0 (19.7–45.2)	12.4 (8.9–17.7)	10.1 (6.2–14.7)	4.7 (3.2–7.1)	19.7 (13.1–28.2)	0.0 (0.0–0.0)	14.9 (8.9–25.2)	1.6 (1.2–2.1)	7.5 (4.5–11.2)	2.5 (2.3–2.6)
Saint Vincent and the Grenadines	Rate: Cumulative change (%)	-37.7 (-56.5 to -12.3)	-52.7 (-71.3 to -28.5)	-50.2 (-69.2 to -21.6)	-61.3 (-77.8 to -39.8)	-76.5 (-86.3 to -61.6)	-9.1 (-43.9 to 41.7)	nan (nan–nan)	1,639.5 (-35.0 to 4,283.8)	-70.2 (-80.5 to -56.8)	1,196.0 (617.5–1,984.1)	76.5 (61.2–93.7)
Suriname	Number: 1990	681 (630–737)	137 (112–158)	65 (56–76)	48 (37–61)	75 (64–88)	58 (43–72)	2 (2–3)	34 (23–65)	13 (10–15)	13 (9–17)	1 (0–2)
Suriname	Number: 2015	396 (344–458)	74 (59–91)	30 (24–38)	34 (26–44)	12 (9–17)	50 (35–65)	0 (0–0)	31 (22–50)	4 (3–6)	9 (6–11)	2 (0–6)
Suriname	Number: Cumulative change (%)	-41.7 (-50.0 to -32.4)	-45.5 (-57.4 to -32.5)	-53.2 (-64.0 to -39.5)	-27.5 (-48.1 to -1.4)	-82.8 (-87.8 to -76.8)	-13.3 (-38.6 to 13.5)	-87.1 (-92.6 to -79.3)	-5.1 (-33.1 to 32.0)	-65.5 (-76.4 to -47.9)	-29.2 (-52.0 to -2.0)	138.8 (-16.3 to 340.8)
Suriname	Rate: 1990	343.8 (318.7–371.7)	67.0 (55.1–77.3)	32.9 (28.5–38.0)	23.8 (18.1–29.8)	37.7 (32.1–43.8)	29.1 (21.6–36.2)	1.6 (1.1–2.1)	17.0 (11.4–31.9)	6.7 (5.5–7.8)	6.5 (4.9–8.3)	0.6 (0.2–1.1)
Suriname	Rate: 2015	216.8 (188.0–251.7)	41.7 (33.4–51.4)	16.8 (13.3–21.3)	19.5 (14.8–24.8)	7.2 (5.3–9.7)	27.8 (19.9–36.6)	0.2 (0.1–0.3)	17.7 (12.4–28.7)	2.4 (1.8–3.5)	5.1 (3.8–6.7)	1.5 (0.3–3.4)
Suriname	Rate: Cumulative change (%)	-36.9 (-45.9 to -26.5)	-37.3 (-50.9 to -22.3)	-48.5 (-60.4 to -33.3)	-16.7 (-40.3 to 13.4)	-80.8 (-86.5 to -74.1)	-3.3 (-31.6 to 26.7)	-87.3 (-92.7 to -79.7)	9.0 (-23.1 to 51.4)	-62.9 (-74.7 to -44.0)	-18.7 (-44.9 to 12.5)	145.2 (-15.4 to 358.7)
Trinidad and Tobago	Number: 1990	1,016 (925–1,120)	193 (164–226)	89 (77–103)	119 (93–147)	34 (28–40)	126 (108–148)	0 (0–0)	24 (7–38)	16 (14–18)	37 (27–47)	6 (6–6)
Trinidad and Tobago	Number: 2015	519 (392–697)	83 (51–124)	29 (21–41)	42 (25–64)	9 (6–13)	95 (63–137)	0 (0–0)	14 (5–24)	3 (2–4)	49 (30–72)	6 (6–7)
Trinidad and Tobago	Number: Cumulative change (%)	-48.7 (-62.5 to -30.8)	-56.5 (-74.7 to -32.5)	-67.1 (-77.4 to -52.0)	-63.7 (-79.7 to -42.6)	-72.2 (-82.3 to -57.8)	-23.8 (-51.2 to 12.3)	nan (nan–nan)	-36.2 (-64.8 to 1.8)	-80.4 (-85.6 to -73.9)	36.6 (-22.1 to 112.3)	3.0 (-5.2 to 11.5)
Trinidad and Tobago	Rate: 1990	208.3 (189.2–229.8)	41.9 (35.5–48.9)	18.2 (15.6–21.1)	25.8 (20.2–31.8)	6.9 (5.8–8.3)	26.4 (22.4–30.8)	0.0 (0.0–0.0)	5.4 (1.7–8.4)	3.1 (2.7–3.5)	8.0 (6.0–10.3)	1.2 (1.2–1.3)
Trinidad and Tobago	Rate: 2015	145.9 (109.8–196.9)	24.0 (14.8–35.9)	8.2 (5.9–11.6)	12.2 (7.4–18.4)	2.6 (1.8–3.9)	27.1 (17.9–39.0)	0.0 (0.0–0.0)	4.3 (1.6–7.1)	0.9 (0.7–1.1)	14.2 (8.7–20.9)	1.8 (1.7–1.9)
Trinidad and Tobago	Rate: Cumulative change (%)	-29.6 (-48.9 to -4.6)	-42.1 (-66.4 to -10.2)	-54.7 (-69.0 to -33.8)	-51.7 (-73.0 to -23.6)	-61.6 (-75.7 to -41.6)	3.7 (-33.8 to 53.6)	nan (nan–nan)	-15.2 (-53.2 to 35.3)	-72.3 (-79.8 to -62.9)	81.8 (3.7–182.3)	46.3 (34.9–58.6)
Virgin Islands, U.S.	Number: 1990	54 (51–57)	10 (8–12)	2 (2–3)	3 (2–4)	0 (0–0)	6 (5–8)	0 (0–0)	1 (0–2)	0 (0–0)	3 (2–4)	0 (0–0)
Virgin Islands, U.S.	Number: 2015	21 (18–23)	2 (2–3)	0 (0–0)	1 (0–1)	0 (0–0)	3 (2–4)	0 (0–0)	0 (0–1)	0 (0–0)	0 (0–1)	0 (0–0)
Virgin Islands, U.S.	Number: Cumulative change (%)	-61.2 (-65.5 to -56.1)	-76.0 (-81.8 to -69.5)	-70.9 (-76.9 to -63.8)	-64.2 (-75.5 to -50.0)	-86.0 (-90.2 to -81.2)	-48.9 (-61.4 to -36.0)	-43.1 (-54.8 to -32.4)	-21.6 (-57.4 to 32.6)	-75.8 (-82.6 to -64.2)	-68.9 (-78.8 to -56.8)	91.4 (-19.2 to 439.8)
Virgin Islands, U.S.	Rate: 1990	124.4 (117.8–131.4)	24.3 (20.3–28.4)	6.5 (5.6–7.5)	8.2 (6.4–10.3)	1.7 (1.4–2.1)	15.2 (12.8–18.7)	0.0 (0.0–0.0)	2.6 (1.5–5.2)	0.6 (0.5–0.7)	7.2 (5.6–9.1)	0.3 (0.1–0.5)
Virgin Islands, U.S.	Rate: 2015	76.1 (68.5–84.8)	9.7 (7.7–11.9)	3.0 (2.5–3.6)	4.8 (3.7–6.2)	0.4 (0.3–0.5)	12.5 (10.0–15.6)	0.0 (0.0–0.0)	3.0 (2.0–4.4)	0.2 (0.2–0.3)	3.7 (2.7–4.8)	0.6 (0.4–1.0)
Virgin Islands, U.S.	Rate: Cumulative change (%)	-38.8 (-45.6 to -30.6)	-59.7 (-69.5 to -48.7)	-53.2 (-62.9 to -41.6)	-39.8 (-58.8 to -15.9)	-77.2 (-83.9 to -69.2)	-16.7 (-37.0 to 4.6)	-19.4 (-35.8 to -4.2)	31.6 (-28.4 to 122.6)	-62.3 (-72.9 to -44.3)	-47.7 (-64.5 to -27.5)	201.3 (27.5–750.0)
Tropical Latin America	Number: 1990	238,315 (233,807–242,979)	42,309 (39,986–44,222)	32,888 (31,414–34,255)	14,189 (13,336–15,270)	41,650 (39,552–43,717)	13,525 (11,536–17,972)	485 (399–589)	9,689 (6,997–11,769)	6,676 (5,402–7,344)	4,045 (3,684–4,535)	392 (328–484)
Tropical Latin America	Number: 2015	83,965 (80,712–87,490)	10,140 (9,048–11,500)	6,045 (5,538–6,658)	6,083 (5,446–6,858)	1,978 (1,756–2,202)	10,729 (8,286–12,326)	20 (16–27)	5,271 (4,024–6,561)	1,446 (1,269–1,765)	4,525 (3,779–5,185)	710 (616–822)
Tropical Latin America	Number: Cumulative change (%)	-64.8 (-66.3 to -63.2)	-76.0 (-78.6 to -72.7)	-81.6 (-83.4 to -79.4)	-57.1 (-62.0 to -51.8)	-95.2 (-95.8 to -94.6)	-18.3 (-49.0 to 2.3)	-95.6 (-96.9 to -94.0)	-45.4 (-52.3 to -38.7)	-78.0 (-82.1 to -69.7)	12.5 (-12.2 to 33.2)	82.0 (65.5–94.5)
Tropical Latin America	Rate: 1990	343.6 (337.1–350.3)	61.3 (57.9–64.0)	47.3 (45.2–49.3)	20.5 (19.3–22.1)	60.2 (57.1–63.2)	19.5 (16.6–25.9)	0.7 (0.6–0.8)	14.0 (10.1–17.1)	9.6 (7.7–10.5)	5.9 (5.3–6.6)	0.6 (0.5–0.7)
Tropical Latin America	Rate: 2015	133.3 (127.7–139.2)	17.5 (15.6–19.8)	10.0 (9.1–11.0)	10.5 (9.4–11.8)	3.3 (3.0–3.7)	18.1 (14.0–20.9)	0.0 (0.0–0.0)	9.1 (6.9–11.3)	2.3 (2.0–2.9)	7.8 (6.5–8.9)	1.1 (0.9–1.3)
Tropical Latin America	Rate: Cumulative change (%)	-61.2 (-63.0 to -59.4)	-71.4 (-74.5 to -67.5)	-78.9 (-81.1 to -76.4)	-48.9 (-54.7 to -42.7)	-94.4 (-95.1 to -93.6)	-4.1 (-40.3 to 20.1)	-95.5 (-96.8 to -93.7)	-35.1 (-43.3 to -27.1)	-75.1 (-79.9 to -65.7)	33.8 (4.4–58.5)	97.0 (79.3–110.6)
Brazil	Number: 1990	232,881 (228,398–237,534)	41,385 (39,036–43,235)	32,079 (30,627–33,443)	13,783 (12,919–14,881)	41,021 (38,938–43,097)	13,060 (11,097–17,497)	484 (399–588)	9,420 (6,857–11,602)	6,505 (5,219–7,187)	3,916 (3,568–4,397)	390 (325–482)
Brazil	Number: 2015	80,450 (77,218–83,751)	9,587 (8,502–10,920)	5,774 (5,288–6,359)	5,833 (5,199–6,618)	1,876 (1,670–2,086)	10,162 (7,907–11,690)	20 (15–26)	5,111 (3,954–6,454)	1,368 (1,206–1,643)	4,405 (3,643–5,072)	699 (606–807)
Brazil	Number: Cumulative change (%)	-65.4 (-67.0 to -63.8)	-76.8 (-79.4 to -73.6)	-82.0 (-83.8 to -79.8)	-57.6 (-62.6 to -52.1)	-95.4 (-96.0 to -94.8)	-19.7 (-49.9 to 0.8)	-95.7 (-97.0 to -94.0)	-45.5 (-52.5 to -38.6)	-78.6 (-82.5 to -70.6)	13.1 (-12.3 to 34.9)	80.0 (64.7–91.9)
Brazil	Rate: 1990	348.2 (341.5–355.1)	62.3 (58.7–65.0)	47.9 (45.8–50.0)	20.7 (19.4–22.4)	61.5 (58.4–64.7)	19.5 (16.6–26.2)	0.7 (0.6–0.9)	14.2 (10.3–17.5)	9.7 (7.7–10.7)	5.9 (5.4–6.6)	0.6 (0.5–0.7)
Brazil	Rate: 2015	133.2 (127.5–139.1)	17.3 (15.4–19.7)	9.9 (9.1–11.0)	10.5 (9.4–11.9)	3.3 (2.9–3.7)	18.0 (14.0–20.7)	0.0 (0.0–0.0)	9.2 (7.1–11.6)	2.3 (2.0–2.8)	7.9 (6.6–9.1)	1.1 (1.0–1.3)
Brazil	Rate: Cumulative change (%)	-61.7 (-63.6 to -59.8)	-72.2 (-75.3 to -68.3)	-79.2 (-81.4 to -76.7)	-49.2 (-55.2 to -42.5)	-94.6 (-95.3 to -93.8)	-5.1 (-40.8 to 19.3)	-95.5 (-96.9 to -93.7)	-34.8 (-43.1 to -26.5)	-75.7 (-80.2 to -66.5)	35.6 (5.1–61.7)	96.0 (79.1–109.2)
Paraguay	Number: 1990	5,433 (5,118–5,733)	924 (786–1,091)	808 (692–929)	405 (318–494)	629 (520–744)	464 (398–543)	0 (0–0)	268 (75–381)	170 (131–211)	129 (99–163)	2 (1–3)
Paraguay	Number: 2015	3,514 (3,048–4,073)	552 (412–717)	271 (217–334)	250 (182–335)	102 (73–139)	566 (365–744)	0 (0–0)	159 (68–227)	77 (48–122)	119 (86–159)	11 (2–25)
Paraguay	Number: Cumulative change (%)	-35.2 (-44.7 to -24.7)	-39.9 (-55.1 to -21.2)	-66.2 (-73.9 to -56.3)	-37.6 (-57.1 to -12.8)	-83.5 (-89.0 to -76.3)	23.7 (-27.9 to 69.6)	-51.6 (-58.6 to -43.3)	-35.2 (-58.0 to -2.1)	-54.1 (-72.1 to -31.5)	-5.3 (-37.1 to 34.5)	404.8 (39.6–779.6)
Paraguay	Rate: 1990	220.2 (208.1–231.9)	35.8 (30.4–42.2)	32.1 (27.5–36.8)	15.7 (12.3–19.1)	24.9 (20.6–29.3)	18.3 (15.7–21.4)	0.0 (0.0–0.0)	10.4 (2.9–14.8)	6.9 (5.4–8.5)	5.0 (3.9–6.3)	0.1 (0.1–0.1)
Paraguay	Rate: 2015	133.2 (115.3–154.9)	21.4 (16.0–27.8)	10.4 (8.3–12.8)	9.7 (7.1–13.0)	3.9 (2.8–5.4)	21.8 (14.1–28.7)	0.0 (0.0–0.0)	6.2 (2.6–8.8)	3.0 (1.8–4.7)	4.7 (3.3–6.2)	0.4 (0.1–0.9)
Paraguay	Rate: Cumulative change (%)	-39.4 (-48.4 to -29.4)	-39.8 (-54.9 to -21.0)	-67.5 (-75.0 to -57.9)	-37.4 (-57.0 to -12.5)	-84.0 (-89.3 to -77.0)	20.9 (-29.4 to 65.7)	-60.5 (-66.1 to -54.0)	-35.1 (-57.9 to -1.9)	-56.9 (-73.8 to -35.7)	-5.1 (-37.0 to 34.8)	363.2 (20.0–716.3)
Southeast Asia, East Asia, and Oceania	Number: 1990	2,952,623 (2,879,653–3,028,330)	457,908 (431,414–484,352)	625,474 (568,666–679,691)	135,467 (118,453–153,693)	168,426 (138,028–194,286)	214,872 (160,970–283,468)	16,206 (11,297–22,422)	33,195 (25,846–43,003)	54,800 (38,543–71,060)	68,679 (55,107–84,266)	801 (671–1,127)
Southeast Asia, East Asia, and Oceania	Number: 2015	690,847 (665,692–717,597)	87,932 (80,019–96,902)	77,435 (70,698–84,534)	60,014 (53,801–67,352)	20,409 (17,125–23,954)	93,882 (84,284–105,088)	3,714 (2,733–4,879)	21,461 (16,733–27,894)	12,275 (10,015–14,872)	18,170 (14,811–22,519)	4,015 (2,888–6,167)
Southeast Asia, East Asia, and Oceania	Number: Cumulative change (%)	-76.6 (-77.6 to -75.5)	-80.8 (-82.8 to -78.6)	-87.6 (-89.1 to -85.9)	-55.4 (-62.3 to -47.6)	-87.8 (-90.3 to -84.6)	-54.8 (-68.7 to -38.8)	-76.1 (-84.8 to -63.7)	-34.4 (-49.6 to -16.2)	-77.0 (-83.2 to -69.3)	-73.1 (-80.0 to -65.1)	417.7 (268.1–691.5)
Southeast Asia, East Asia, and Oceania	Rate: 1990	414.0 (403.8–424.5)	63.4 (59.8–67.1)	87.0 (79.1–94.5)	18.8 (16.4–21.3)	23.6 (19.4–27.3)	30.0 (22.4–39.5)	2.3 (1.6–3.2)	4.6 (3.6–6.0)	7.7 (5.4–10.0)	9.5 (7.6–11.7)	0.1 (0.1–0.2)
Southeast Asia, East Asia, and Oceania	Rate: 2015	124.5 (119.9–129.4)	16.2 (14.7–17.8)	14.0 (12.8–15.3)	11.0 (9.9–12.4)	3.7 (3.1–4.3)	17.1 (15.3–19.1)	0.7 (0.5–0.9)	3.9 (3.1–5.1)	2.2 (1.8–2.7)	3.3 (2.7–4.1)	0.7 (0.5–1.1)
Southeast Asia, East Asia, and Oceania	Rate: Cumulative change (%)	-69.9 (-71.2 to -68.5)	-74.5 (-77.1 to -71.6)	-83.8 (-85.7 to -81.6)	-40.9 (-50.0 to -30.5)	-84.2 (-87.5 to -80.2)	-41.1 (-59.1 to -20.1)	-70.3 (-81.0 to -54.8)	-13.2 (-33.3 to 10.9)	-70.6 (-78.5 to -60.8)	-64.4 (-73.5 to -53.7)	550.5 (361.2–895.8)
East Asia	Number: 1990	1,864,295 (1,794,268–1,938,981)	333,663 (309,790–356,855)	409,264 (374,213–444,441)	72,246 (59,924–88,163)	64,343 (56,000–72,918)	165,662 (126,107–214,692)	76 (65–89)	7,371 (4,462–10,090)	27,587 (16,627–35,503)	38,942 (30,212–47,860)	55 (40–74)
East Asia	Number: 2015	309,899 (294,947–325,501)	39,620 (34,934–44,569)	28,066 (25,444–31,792)	27,558 (23,991–31,311)	2,230 (1,914–2,590)	53,615 (46,003–65,020)	27 (24–29)	2,766 (1,645–3,572)	3,041 (2,058–3,673)	7,121 (5,878–8,534)	1,655 (1,334–2,007)
East Asia	Number: Cumulative change (%)	-83.4 (-84.3 to -82.2)	-88.1 (-89.5 to -86.5)	-93.1 (-94.0 to -92.0)	-61.4 (-69.4 to -53.4)	-96.5 (-97.2 to -95.7)	-66.5 (-76.7 to -51.1)	-64.2 (-69.6 to -58.6)	-61.8 (-71.0 to -50.6)	-88.7 (-91.2 to -85.9)	-81.4 (-86.1 to -75.3)	2,941.5 (2,470.0–3,531.4)
East Asia	Rate: 1990	383.0 (368.7–398.3)	67.9 (63.0–72.6)	83.1 (76.0–90.2)	14.7 (12.2–17.9)	13.2 (11.5–14.9)	33.8 (25.8–43.8)	0.0 (0.0–0.0)	1.5 (0.9–2.1)	5.7 (3.5–7.3)	7.9 (6.1–9.7)	0.0 (0.0–0.0)
East Asia	Rate: 2015	95.6 (91.0–100.5)	12.5 (11.0–14.1)	8.7 (7.9–9.9)	8.7 (7.6–9.9)	0.7 (0.6–0.8)	16.7 (14.3–20.3)	0.0 (0.0–0.0)	0.9 (0.5–1.1)	0.9 (0.6–1.1)	2.2 (1.8–2.7)	0.5 (0.4–0.6)
East Asia	Rate: Cumulative change (%)	-75.0 (-76.5 to -73.3)	-81.6 (-83.7 to -79.0)	-89.5 (-90.8 to -87.7)	-40.1 (-52.5 to -27.7)	-94.7 (-95.8 to -93.5)	-49.0 (-64.4 to -25.5)	-49.2 (-56.7 to -41.3)	-40.9 (-55.1 to -23.6)	-83.2 (-86.8 to -79.2)	-71.2 (-78.5 to -61.7)	4,492.3 (3,768.7–5,391.7)
China	Number: 1990	1,829,548 (1,757,168–1,905,739)	328,530 (304,277–351,973)	403,054 (368,156–438,856)	70,391 (58,142–86,168)	63,739 (55,434–72,536)	161,671 (123,436–208,892)	71 (60–85)	7,166 (4,336–9,812)	27,175 (16,247–35,140)	38,126 (29,730–46,961)	54 (40–72)
China	Number: 2015	297,444 (283,048–312,371)	38,083 (33,565–42,783)	26,806 (24,233–30,339)	26,479 (22,922–30,161)	2,150 (1,836–2,509)	51,615 (44,340–63,033)	26 (23–29)	2,637 (1,576–3,422)	2,935 (1,960–3,549)	6,726 (5,545–8,083)	1,623 (1,310–1,978)
China	Number: Cumulative change (%)	-83.7 (-84.7 to -82.7)	-88.4 (-89.7 to -86.8)	-93.3 (-94.2 to -92.2)	-61.9 (-69.6 to -53.7)	-96.6 (-97.3 to -95.8)	-67.0 (-77.3 to -51.8)	-63.2 (-69.0 to -57.2)	-62.5 (-71.5 to -51.1)	-88.9 (-91.3 to -86.2)	-82.0 (-86.6 to -76.2)	2,929.8 (2,471.8–3,548.6)
China	Rate: 1990	387.1 (372.0–402.9)	68.7 (63.7–73.6)	84.2 (76.9–91.7)	14.7 (12.2–18.0)	13.4 (11.7–15.2)	34.0 (25.9–43.9)	0.0 (0.0–0.0)	1.5 (0.9–2.1)	5.8 (3.5–7.5)	8.0 (6.2–9.8)	0.0 (0.0–0.0)
China	Rate: 2015	95.1 (90.5–99.9)	12.4 (10.9–14.0)	8.6 (7.8–9.8)	8.6 (7.5–9.8)	0.7 (0.6–0.8)	16.6 (14.3–20.3)	0.0 (0.0–0.0)	0.9 (0.5–1.1)	0.9 (0.6–1.1)	2.2 (1.8–2.6)	0.5 (0.4–0.6)
China	Rate: Cumulative change (%)	-75.4 (-76.9 to -73.8)	-81.9 (-84.0 to -79.4)	-89.7 (-91.1 to -88.1)	-40.6 (-52.6 to -27.8)	-94.8 (-95.9 to -93.7)	-49.4 (-65.2 to -26.2)	-47.5 (-55.4 to -39.0)	-41.7 (-55.6 to -24.0)	-83.5 (-86.9 to -79.4)	-72.0 (-79.1 to -63.0)	4,509.5 (3,802.7–5,463.1)
North Korea	Number: 1990	28,651 (16,564–45,772)	4,695 (2,529–7,649)	6,070 (2,706–11,423)	1,758 (872–2,996)	580 (206–1,162)	3,078 (1,332–5,567)	4 (2–7)	146 (52–320)	356 (166–695)	614 (225–1,245)	0 (0–3)
North Korea	Number: 2015	10,098 (6,638–15,098)	1,368 (588–2,457)	1,206 (662–2,079)	1,017 (420–1,902)	72 (34–147)	1,506 (806–2,581)	0 (0–1)	76 (25–161)	89 (51–150)	237 (73–519)	27 (6–79)
North Korea	Number: Cumulative change (%)	-61.3 (-81.4 to -31.2)	-67.9 (-88.1 to -32.8)	-76.0 (-91.6 to -45.3)	-33.6 (-78.6 to 40.9)	-83.8 (-95.5 to -61.7)	-42.1 (-79.3 to 19.9)	-77.7 (-87.7 to -64.9)	-33.8 (-83.9 to 60.0)	-69.4 (-89.4 to -33.7)	-48.2 (-90.1 to 37.3)	inf (1,105.8–87,727.5)
North Korea	Rate: 1990	375.9 (214.9–604.8)	61.3 (33.0–100.0)	80.6 (35.8–151.7)	23.0 (11.4–39.1)	7.7 (2.7–15.5)	40.7 (17.5–74.0)	0.1 (0.0–0.1)	1.9 (0.7–4.2)	4.8 (2.2–9.3)	8.0 (3.0–16.3)	0.0 (0.0–0.0)
North Korea	Rate: 2015	145.5 (93.1–221.3)	20.7 (8.9–37.2)	18.0 (9.9–31.2)	15.4 (6.4–28.8)	1.1 (0.5–2.2)	22.5 (11.9–38.6)	0.0 (0.0–0.0)	1.2 (0.4–2.4)	1.3 (0.7–2.2)	3.6 (1.1–7.9)	0.4 (0.1–1.1)
North Korea	Rate: Cumulative change (%)	-57.4 (-79.7 to -23.3)	-62.8 (-86.3 to -22.0)	-72.9 (-90.6 to -38.2)	-23.0 (-75.2 to 63.4)	-81.9 (-95.1 to -57.0)	-34.5 (-76.7 to 35.5)	-77.3 (-87.6 to -64.0)	-23.4 (-81.4 to 85.2)	-66.9 (-88.7 to -27.4)	-40.1 (-88.5 to 59.2)	inf (1,160.2–97,457.3)
Taiwan	Number: 1990	6,094 (5,999–6,191)	437 (312–545)	139 (103–211)	96 (54–149)	22 (14–33)	913 (755–1,084)	0 (0–0)	59 (24–116)	55 (34–82)	200 (110–319)	0 (0–0)
Taiwan	Number: 2015	2,356 (1,922–2,959)	169 (95–264)	53 (41–72)	61 (34–102)	7 (4–12)	493 (317–747)	0 (0–0)	52 (22–93)	16 (10–25)	158 (82–253)	3 (2–6)
Taiwan	Number: Cumulative change (%)	-61.3 (-68.5 to -51.3)	-60.4 (-78.6 to -35.4)	-59.9 (-72.8 to -43.1)	-29.8 (-68.5 to 30.9)	-64.2 (-82.3 to -34.8)	-45.9 (-64.8 to -19.8)	nan (nan–nan)	3.3 (-61.5 to 101.7)	-68.1 (-83.6 to -45.8)	-13.0 (-61.0 to 65.6)	inf (2,408.6–5,979.0)
Taiwan	Rate: 1990	88.4 (87.0–90.0)	7.1 (5.1–8.8)	2.0 (1.5–3.0)	1.6 (0.9–2.4)	0.4 (0.2–0.5)	14.6 (12.0–17.4)	0.0 (0.0–0.0)	1.0 (0.4–1.9)	0.9 (0.5–1.3)	3.2 (1.8–5.2)	0.0 (0.0–0.0)
Taiwan	Rate: 2015	51.8 (40.7–67.2)	4.3 (2.4–6.7)	1.2 (0.9–1.6)	1.6 (0.9–2.6)	0.2 (0.1–0.3)	12.3 (7.9–18.7)	0.0 (0.0–0.0)	1.3 (0.6–2.4)	0.4 (0.2–0.6)	4.0 (2.1–6.5)	0.1 (0.0–0.1)
Taiwan	Rate: Cumulative change (%)	-41.4 (-54.0 to -23.9)	-37.9 (-66.4 to 1.4)	-38.1 (-58.6 to -10.4)	10.2 (-50.5 to 105.5)	-44.1 (-73.0 to 3.3)	-15.6 (-45.6 to 25.6)	nan (nan–nan)	62.0 (-39.6 to 215.9)	-49.6 (-75.2 to -11.8)	36.6 (-38.7 to 159.8)	inf (3,444.8–8,651.8)
Southeast Asia	Number: 1990	1,068,595 (1,049,222–1,088,565)	122,834 (107,894–140,671)	211,599 (173,334–245,478)	62,520 (50,203–75,168)	102,556 (72,441–127,029)	48,562 (34,276–73,158)	15,537 (10,665–21,783)	25,592 (19,423–34,184)	26,666 (17,633–38,388)	29,145 (20,472–40,974)	733 (607–1,046)
Southeast Asia	Number: 2015	365,942 (346,792–386,347)	47,066 (40,823–54,048)	46,590 (40,843–52,186)	31,717 (26,406–37,667)	17,561 (14,378–21,136)	39,415 (34,904–43,893)	3,273 (2,303–4,379)	18,379 (14,296–24,176)	8,862 (7,167–11,068)	10,376 (7,292–14,396)	2,295 (1,291–4,429)
Southeast Asia	Number: Cumulative change (%)	-65.7 (-67.6 to -63.7)	-61.5 (-67.7 to -54.9)	-77.7 (-81.8 to -72.3)	-48.6 (-60.9 to -33.9)	-82.4 (-87.2 to -75.4)	-14.3 (-48.6 to 11.7)	-78.0 (-86.3 to -65.9)	-26.7 (-46.9 to -1.5)	-64.9 (-77.1 to -45.9)	-62.8 (-78.0 to -44.4)	222.1 (82.8–516.8)
Southeast Asia	Rate: 1990	480.1 (471.3–489.1)	54.4 (47.7–62.3)	94.8 (77.7–110.0)	27.7 (22.2–33.3)	46.1 (32.6–57.0)	21.7 (15.3–32.6)	7.1 (4.9–9.9)	11.3 (8.6–15.1)	12.0 (7.9–17.3)	12.9 (9.1–18.2)	0.3 (0.3–0.5)
Southeast Asia	Rate: 2015	162.2 (153.6–171.3)	21.2 (18.4–24.4)	20.8 (18.2–23.3)	14.3 (11.9–17.0)	7.8 (6.4–9.4)	17.6 (15.6–19.6)	1.4 (1.0–1.9)	8.3 (6.4–10.9)	3.9 (3.2–4.9)	4.7 (3.3–6.5)	1.0 (0.6–2.0)
Southeast Asia	Rate: Cumulative change (%)	-66.2 (-68.1 to -64.1)	-60.7 (-67.1 to -54.0)	-77.9 (-81.9 to -72.5)	-47.5 (-60.1 to -32.5)	-82.6 (-87.4 to -75.7)	-14.0 (-48.5 to 12.0)	-79.1 (-87.0 to -67.7)	-25.4 (-45.9 to 0.2)	-65.5 (-77.4 to -46.7)	-62.1 (-77.6 to -43.4)	209.3 (75.1–495.2)
Cambodia	Number: 1990	51,084 (49,652–52,601)	6,492 (5,081–7,887)	13,029 (10,498–15,490)	1,398 (840–2,097)	5,519 (3,902–7,316)	1,980 (999–4,344)	1,548 (926–2,350)	784 (335–1,474)	1,502 (821–2,548)	1,563 (612–2,899)	24 (13–36)
Cambodia	Number: 2015	15,098 (13,702–16,571)	2,610 (1,963–3,274)	2,327 (1,855–2,842)	1,036 (662–1,481)	530 (347–790)	1,486 (1,065–2,015)	393 (222–628)	592 (299–1,009)	395 (243–618)	638 (303–1,111)	139 (59–236)
Cambodia	Number: Cumulative change (%)	-70.4 (-73.3 to -67.5)	-59.1 (-71.2 to -45.5)	-81.9 (-86.2 to -76.7)	-19.7 (-59.7 to 39.6)	-90.0 (-94.4 to -83.5)	-8.6 (-58.1 to 37.6)	-72.6 (-87.1 to -51.0)	-8.0 (-67.1 to 102.9)	-70.6 (-86.8 to -46.2)	-50.2 (-83.5 to 13.0)	487.7 (214.9–860.8)
Cambodia	Rate: 1990	801.8 (780.2–825.1)	93.5 (73.2–113.6)	196.2 (158.3–232.8)	20.2 (12.2–30.2)	85.1 (60.9–112.1)	29.8 (15.1–64.8)	28.2 (17.7–41.1)	11.3 (4.9–21.3)	23.3 (13.1–38.9)	22.6 (8.8–41.9)	0.4 (0.2–0.6)

© 2017 Global Burden of Disease Child and Adolescent Health Collaboration. JAMA Pediatrics.

eTable 1. Number of Deaths, Death Rates (per 100,000 population), and Cumulative Percent Change with 95% Uncertainty Intervals (UI) for the Top 10 Global Causes of Death in 195 Countries and Territories, Aged 0 to 19, Both Sexes, 1990 and 2015*Note: best viewed by enlarging in browser.*

Location	Measure	All causes	Neonatal preterm birth complications	Lower respiratory infections	Neonatal encephalopathy due to birth asphyxia and trauma	Diarrheal diseases	Congenital anomalies	Malaria	Neonatal sepsis and other neonatal infections	Meningitis	Other neonatal disorders	HIV/AIDS
Cambodia	Rate: 2015	225.4 (204.7–247.4)	38.6 (29.0–48.4)	34.5 (27.6–42.1)	15.3 (9.8–21.9)	7.9 (5.2–11.7)	22.1 (15.8–29.9)	6.0 (3.4–9.5)	8.8 (4.4–14.9)	5.9 (3.7–9.2)	9.4 (4.5–16.4)	2.1 (0.9–3.6)
Cambodia	Rate: Cumulative change (%)	-71.9 (-74.6 to -69.1)	-58.1 (-70.5 to -44.0)	-82.2 (-86.4 to -77.1)	-17.7 (-58.7 to 42.7)	-90.4 (-94.6 to -84.2)	-10.1 (-58.5 to 34.8)	-77.4 (-89.2 to -60.3)	-6.0 (-66.3 to 107.7)	-72.0 (-87.2 to -49.9)	-49.2 (-83.1 to 15.0)	445.1 (177.6–790.6)
Indonesia	Number: 1990	478,709 (470,466–487,091)	50,390 (39,029–62,691)	80,626 (47,723–105,351)	36,577 (25,406–48,589)	59,099 (31,410–80,278)	14,732 (8,735–27,155)	4,160 (2,082–6,888)	8,387 (4,262–14,532)	13,935 (6,034–23,699)	12,074 (5,585–21,260)	0 (0–1)
Indonesia	Number: 2015	162,905 (147,794–179,823)	23,223 (18,149–29,136)	16,404 (12,082–20,434)	18,168 (13,116–23,619)	10,498 (7,407–13,712)	14,371 (11,584–17,396)	871 (539–1,279)	5,805 (2,883–10,298)	4,254 (2,812–6,319)	5,118 (2,524–8,974)	976 (129–3,043)
Indonesia	Number: Cumulative change (%)	-66.0 (-69.1 to -62.3)	-53.0 (-67.3 to -36.5)	-78.2 (-86.2 to -64.2)	-48.5 (-67.0 to -22.3)	-80.4 (-88.9 to -65.6)	10.9 (-49.5 to 74.6)	-76.0 (-90.0 to -57.0)	-22.4 (-68.9 to 51.3)	-63.6 (-84.3 to -22.7)	-50.7 (-82.5 to -3.0)	inf (101,485.7–inf)
Indonesia	Rate: 1990	565.6 (555.8–575.3)	59.2 (45.8–73.6)	95.9 (56.7–125.4)	42.9 (29.8–57.0)	70.0 (37.1–95.2)	17.4 (10.3–32.1)	4.9 (2.5–8.1)	9.9 (5.0–17.1)	16.6 (7.1–28.2)	14.2 (6.6–25.0)	0.0 (0.0–0.0)
Indonesia	Rate: 2015	174.3 (158.1–192.5)	25.2 (19.7–31.6)	17.6 (13.0–21.9)	19.7 (14.2–25.6)	11.2 (7.9–14.7)	15.5 (12.5–18.7)	0.9 (0.6–1.3)	6.3 (3.1–11.2)	4.5 (3.0–6.7)	5.5 (2.7–9.7)	1.0 (0.1–3.2)
Indonesia	Rate: Cumulative change (%)	-69.2 (-72.1 to -65.8)	-56.7 (-69.9 to -41.5)	-80.3 (-87.5 to -67.7)	-52.5 (-69.6 to -28.3)	-82.3 (-90.0 to -68.9)	1.2 (-54.0 to 59.8)	-78.6 (-91.1 to -61.9)	-28.6 (-71.4 to 39.2)	-67.2 (-85.9 to -30.4)	-54.7 (-83.9 to -11.0)	inf (90,402.6–inf)
Laos	Number: 1990	36,871 (34,793–39,093)	5,352 (3,902–6,587)	8,560 (6,437–10,953)	972 (533–1,574)	5,441 (3,569–7,414)	1,219 (528–3,208)	66 (22–122)	559 (237–1,101)	1,014 (456–1,782)	1,201 (488–2,246)	0 (0–0)
Laos	Number: 2015	12,955 (10,716–15,600)	2,301 (1,669–3,026)	2,527 (1,728–3,495)	771 (452–1,203)	875 (546–1,334)	1,066 (650–1,818)	12 (6–20)	462 (215–866)	397 (205–678)	539 (254–993)	11 (0–48)
Laos	Number: Cumulative change (%)	-64.8 (-71.1 to -57.1)	-56.4 (-68.5 to -40.4)	-69.9 (-80.1 to -56.4)	-11.5 (-62.5 to 68.1)	-83.2 (-90.7 to -72.7)	9.8 (-51.5 to 67.8)	-77.8 (-92.0 to -52.7)	1.8 (-67.1 to 147.9)	-55.6 (-81.5 to -14.7)	-44.9 (-82.4 to 26.2)	331,496.7 (19,036.8–1,005,573.6)
Laos	Rate: 1990	1,275.4 (1,206.3–1,349.4)	164.2 (119.8–202.7)	290.6 (216.6–372.5)	29.8 (16.4–48.2)	188.3 (123.9–256.3)	40.2 (17.2–105.3)	2.5 (0.9–4.6)	17.2 (7.3–34.0)	35.2 (15.9–61.2)	37.1 (15.2–69.0)	0.0 (0.0–0.0)
Laos	Rate: 2015	404.9 (334.9–487.8)	70.8 (51.4–93.2)	79.0 (54.1–109.3)	23.7 (13.9–37.0)	27.4 (17.2–41.8)	33.2 (20.3–56.6)	0.4 (0.2–0.6)	14.3 (6.7–26.7)	12.5 (6.5–21.3)	16.6 (7.9–30.6)	0.4 (0.0–1.6)
Laos	Rate: Cumulative change (%)	-68.2 (-74.0 to -61.3)	-56.2 (-68.4 to -40.6)	-72.3 (-81.8 to -59.6)	-11.2 (-62.2 to 69.0)	-84.8 (-91.5 to -75.4)	4.3 (-54.3 to 58.7)	-81.5 (-93.2 to -61.5)	1.7 (-67.1 to 147.3)	-60.1 (-83.3 to -24.5)	-45.1 (-82.3 to 25.2)	287,626.7 (16,422.8–872,944.0)
Malaysia	Number: 1990	12,041 (11,903–12,189)	1,480 (1,226–1,724)	984 (632–1,280)	575 (441–731)	237 (135–354)	1,282 (967–1,679)	7 (4–12)	461 (248–798)	321 (180–515)	827 (633–1,056)	3 (1–6)
Malaysia	Number: 2015	5,939 (5,462–6,511)	374 (276–493)	387 (316–478)	210 (153–282)	60 (41–86)	1,009 (757–1,239)	1 (1–3)	249 (145–384)	130 (88–196)	206 (149–275)	39 (20–68)
Malaysia	Number: Cumulative change (%)	-50.7 (-54.8 to -46.0)	-74.5 (-81.7 to -65.9)	-59.0 (-71.2 to -40.0)	-62.5 (-75.9 to -46.4)	-72.5 (-85.4 to -51.0)	-18.9 (-48.5 to 12.9)	-70.8 (-87.4 to -45.7)	-40.2 (-73.9 to 6.2)	-55.8 (-78.2 to -24.9)	-74.5 (-83.1 to -62.1)	1,142.3 (771.9–1,564.0)
Malaysia	Rate: 1990	134.3 (132.9–135.9)	15.7 (13.0–18.3)	10.8 (7.0–14.1)	6.1 (4.7–7.8)	2.6 (1.5–3.9)	13.8 (10.4–18.0)	0.1 (0.0–0.1)	4.9 (2.6–8.5)	3.5 (2.0–5.6)	8.8 (6.7–11.2)	0.0 (0.0–0.1)
Malaysia	Rate: 2015	59.2 (54.2–65.1)	4.0 (2.9–5.3)	3.9 (3.2–4.9)	2.2 (1.6–3.0)	0.6 (0.4–0.9)	10.7 (8.0–13.1)	0.0 (0.0–0.0)	2.7 (1.6–4.1)	1.3 (0.9–2.1)	2.2 (1.6–2.9)	0.4 (0.2–0.7)
Malaysia	Rate: Cumulative change (%)	-55.9 (-59.8 to -51.5)	-74.3 (-81.6 to -65.7)	-62.3 (-73.5 to -44.8)	-62.3 (-75.8 to -46.1)	-73.6 (-86.1 to -53.3)	-20.1 (-49.4 to 11.4)	-75.7 (-89.4 to -55.5)	-39.9 (-73.8 to 6.8)	-58.7 (-79.6 to -30.5)	-74.3 (-83.0 to -61.9)	933.0 (632.0–1,278.4)
Maldives	Number: 1990	706 (675–739)	45 (32–62)	75 (38–110)	43 (32–56)	45 (20–72)	78 (46–126)	0 (0–0)	10 (6–16)	10 (4–17)	47 (34–62)	0 (0–0)
Maldives	Number: 2015	121 (108–137)	9 (7–13)	6 (4–8)	10 (7–13)	2 (1–3)	23 (18–30)	0 (0–0)	3 (1–5)	1 (0–1)	7 (4–9)	0 (0–0)
Maldives	Number: Cumulative change (%)	-82.7 (-84.8 to -80.4)	-77.1 (-86.6 to -66.4)	-90.4 (-94.8 to -83.0)	-75.8 (-83.6 to -65.5)	-94.2 (-97.3 to -88.1)	-66.0 (-83.4 to -41.8)	nan (nan–nan)	-68.4 (-81.3 to -52.6)	-84.8 (-93.6 to -67.5)	-84.5 (-90.2 to -77.2)	97.8 (62.3–136.3)
Maldives	Rate: 1990	450.3 (430.7–470.3)	27.1 (19.5–37.7)	46.2 (23.4–67.5)	26.2 (19.5–34.0)	28.1 (13.0–44.4)	48.1 (28.7–77.6)	0.0 (0.0–0.0)	6.1 (3.9–10.0)	6.5 (2.8–10.9)	28.8 (20.8–37.9)	0.1 (0.0–0.1)
Maldives	Rate: 2015	88.9 (79.2–100.3)	7.1 (5.1–9.6)	4.7 (3.6–5.8)	7.4 (5.6–9.9)	1.6 (1.1–2.3)	17.2 (13.4–21.8)	0.0 (0.0–0.0)	2.2 (1.4–3.9)	1.0 (0.7–1.4)	5.2 (3.5–7.1)	0.1 (0.1–0.2)
Maldives	Rate: Cumulative change (%)	-80.2 (-82.6 to -77.6)	-72.6 (-83.9 to -59.7)	-88.7 (-93.8 to -79.9)	-71.0 (-80.3 to -58.7)	-93.2 (-96.9 to -86.3)	-59.9 (-80.4 to -31.3)	nan (nan–nan)	-62.2 (-77.6 to -43.4)	-82.5 (-92.5 to -63.0)	-81.5 (-88.2 to -72.7)	134.8 (91.2–181.3)
Mauritius	Number: 1990	639 (626–652)	172 (158–185)	42 (23–54)	57 (48–67)	29 (14–39)	66 (44–80)	0 (0–0)	21 (12–28)	8 (4–10)	8 (6–9)	0 (0–1)
Mauritius	Number: 2015	272 (258–288)	48 (41–57)	14 (11–16)	14 (12–18)	4 (3–5)	51 (34–59)	0 (0–0)	10 (6–13)	2 (1–2)	12 (9–14)	2 (2–2)
Mauritius	Number: Cumulative change (%)	-57.3 (-59.8 to -54.7)	-72.1 (-76.0 to -66.9)	-64.2 (-75.9 to -38.6)	-73.6 (-79.4 to -66.8)	-82.2 (-89.2 to -67.1)	-20.7 (-39.1 to 10.2)	-19.4 (-41.2 to 10.4)	-51.1 (-64.8 to -36.8)	-68.9 (-78.6 to -50.2)	49.5 (12.5–96.2)	131.8 (98.3–171.5)
Mauritius	Rate: 1990	154.7 (151.5–158.0)	42.3 (39.0–45.6)	10.3 (5.7–13.3)	14.0 (11.9–16.5)	7.3 (3.5–9.8)	16.2 (10.8–19.8)	0.0 (0.0–0.0)	5.3 (3.0–7.0)	2.0 (1.2–2.6)	2.0 (1.6–2.4)	0.2 (0.2–0.3)
Mauritius	Rate: 2015	94.4 (89.0–100.6)	18.8 (16.1–22.6)	5.1 (4.0–6.1)	5.8 (4.8–7.1)	1.8 (1.3–2.2)	19.1 (12.7–22.3)	0.0 (0.0–0.0)	4.0 (2.7–5.4)	0.9 (0.7–1.0)	4.7 (3.9–5.7)	0.8 (0.7–0.9)
Mauritius	Rate: Cumulative change (%)	-39.0 (-42.8 to -34.9)	-55.6 (-61.8 to -47.3)	-47.0 (-64.7 to -8.4)	-58.0 (-67.3 to -47.2)	-73.2 (-83.8 to -49.8)	20.9 (-7.6 to 68.2)	-5.0 (-30.2 to 31.0)	-22.3 (-44.1 to 0.4)	-55.4 (-69.9 to -28.1)	137.5 (78.6–211.7)	218.1 (171.4–275.2)
Myanmar	Number: 1990	151,024 (133,646–169,048)	14,363 (10,837–18,478)	43,432 (34,979–52,507)	11,193 (7,699–15,030)	17,138 (12,516–22,291)	5,555 (3,880–8,738)	7,906 (4,874–11,637)	1,652 (763–3,003)	3,575 (2,083–5,986)	2,751 (1,296–4,932)	89 (3–405)
Myanmar	Number: 2015	42,453 (35,983–50,680)	4,394 (3,003–6,068)	8,336 (6,341–11,179)	5,519 (3,884–7,393)	1,675 (1,152–2,380)	4,058 (2,967–5,312)	1,797 (1,103–2,654)	1,223 (543–2,374)	947 (610–1,497)	1,194 (590–2,103)	246 (18–1,158)
Myanmar	Number: Cumulative change (%)	-71.7 (-76.9 to -65.3)	-68.6 (-80.8 to -53.1)	-80.5 (-86.3 to -72.6)	-48.5 (-68.4 to -19.7)	-89.9 (-94.0 to -84.5)	-21.3 (-61.7 to 17.4)	-75.7 (-87.6 to -58.1)	-13.7 (-68.8 to 95.1)	-70.7 (-86.4 to -44.2)	-49.2 (-81.9 to 4.6)	503.3 (16.9–1,247.6)
Myanmar	Rate: 1990	745.0 (658.6–834.3)	70.2 (53.0–90.3)	215.1 (173.3–260.2)	54.7 (37.6–73.4)	84.6 (61.8–110.2)	27.3 (19.1–43.0)	39.3 (24.3–57.6)	8.1 (3.7–14.7)	17.7 (10.2–29.6)	13.5 (6.3–24.1)	0.4 (0.0–2.0)
Myanmar	Rate: 2015	233.1 (197.0–279.9)	25.5 (17.4–35.2)	47.3 (35.8–63.5)	32.0 (22.5–42.9)	9.2 (6.3–13.3)	22.9 (16.8–30.0)	9.0 (5.5–13.4)	7.1 (3.2–13.8)	5.2 (3.3–8.4)	6.9 (3.4–12.2)	1.2 (0.1–5.7)
Myanmar	Rate: Cumulative change (%)	-68.5 (-74.4 to -61.1)	-62.8 (-77.3 to -44.4)	-77.7 (-84.3 to -68.5)	-38.9 (-62.5 to -4.8)	-88.7 (-93.3 to -82.6)	-9.6 (-55.9 to 35.2)	-75.4 (-87.5 to -57.6)	2.1 (-63.0 to 130.8)	-67.6 (-85.2 to -37.8)	-40.0 (-78.5 to 23.4)	506.1 (14.2–1,253.0)
Philippines	Number: 1990	144,759 (141,390–148,351)	13,064 (10,429–15,380)	38,489 (28,583–44,442)	6,591 (5,225–8,019)	9,425 (7,004–11,149)	6,972 (5,846–8,252)	374 (264–499)	9,609 (7,387–13,515)	3,590 (2,721–4,186)	3,371 (2,499–4,252)	491 (469–516)
Philippines	Number: 2015	80,159 (74,567–86,458)	8,576 (6,717–10,243)	11,997 (10,481–13,528)	4,360 (3,153–5,432)	3,514 (2,928–4,100)	9,320 (8,006–10,860)	19 (11–28)	8,083 (6,290–11,057)	2,179 (1,877–2,499)	1,219 (898–1,554)	765 (697–839)
Philippines	Number: Cumulative change (%)	-44.6 (-48.8 to -40.0)	-34.1 (-46.1 to -21.5)	-68.3 (-74.2 to -58.1)	-33.2 (-50.6 to -12.5)	-61.9 (-71.1 to -49.0)	35.2 (8.4–67.6)	-94.7 (-97.0 to -91.3)	-15.1 (-31.4 to 3.8)	-38.3 (-50.7 to -19.1)	-63.2 (-73.6 to -51.0)	55.8 (40.1–72.2)
Philippines	Rate: 1990	411.9 (402.6–421.8)	35.0 (28.0–41.2)	108.4 (80.5–125.1)	17.7 (14.0–21.5)	26.6 (19.7–31.3)	19.2 (16.1–22.8)	1.2 (0.8–1.5)	25.8 (19.8–36.3)	10.5 (7.9–12.2)	9.0 (6.7–11.4)	1.5 (1.4–1.6)
Philippines	Rate: 2015	187.5 (174.5–202.2)	19.9 (15.6–23.8)	28.0 (24.5–31.5)	10.1 (7.3–12.6)	8.2 (6.8–9.6)	21.7 (18.7–25.3)	0.0 (0.0–0.1)	18.8 (14.6–25.7)	5.1 (4.4–5.9)	2.8 (2.1–3.6)	1.8 (1.6–2.0)
Philippines	Rate: Cumulative change (%)	-54.5 (-57.9 to -50.8)	-42.8 (-53.2 to -32.0)	-73.7 (-78.6 to -65.2)	-42.1 (-57.2 to -24.2)	-68.5 (-76.0 to -58.0)	14.5 (-8.8 to 42.1)	-95.9 (-97.7 to -93.5)	-26.5 (-40.6 to -10.1)	-50.3 (-60.2 to -35.2)	-68.2 (-77.1 to -57.6)	19.6 (7.5–32.0)
Sri Lanka	Number: 1990	20,674 (20,211–21,153)	3,222 (2,838–3,673)	1,981 (1,706–2,227)	1,439 (1,230–1,699)	1,308 (1,112–1,510)	786 (661–1,140)	5 (3–8)	329 (193–423)	317 (267–362)	1,767 (1,529–2,001)	5 (5–6)
Sri Lanka	Number: 2015	4,524 (3,926–5,225)	597 (460–757)	225 (183–276)	245 (181–318)	25 (19–34)	744 (584–924)	1 (0–2)	207 (134–279)	42 (34–51)	134 (99–171)	3 (3–3)
Sri Lanka	Number: Cumulative change (%)	-78.1 (-81.0 to -74.7)	-81.4 (-85.9 to -76.0)	-88.5 (-91.0 to -85.6)	-82.8 (-87.8 to -76.9)	-98.0 (-98.5 to -97.3)	-3.3 (-34.3 to 24.9)	-72.5 (-87.7 to -51.0)	-35.9 (-53.2 to -17.0)	-86.6 (-89.5 to -82.4)	-92.4 (-94.4 to -90.0)	-35.6 (-43.3 to -27.8)
Sri Lanka	Rate: 1990	303.9 (297.0–311.1)	49.5 (43.6–56.4)	29.4 (25.4–33.1)	22.1 (18.9–26.1)	19.4 (16.5–22.4)	11.8 (9.9–17.0)	0.1 (0.0–0.1)	5.1 (3.0–6.5)	4.7 (4.0–5.4)	27.1 (23.5–30.7)	0.1 (0.1–0.1)
Sri Lanka	Rate: 2015	71.1 (61.5–82.3)	10.0 (7.7–12.7)	3.5 (2.9–4.3)	4.1 (3.0–5.3)	0.4 (0.3–0.5)	11.9 (9.3–14.9)	0.0 (0.0–0.0)	3.5 (2.3–4.7)	0.7 (0.5–0.8)	2.2 (1.7–2.9)	0.1 (0.1–0.1)
Sri Lanka	Rate: Cumulative change (%)	-76.6 (-79.8 to -72.9)	-79.6 (-84.6 to -73.7)	-87.9 (-90.5 to -84.7)	-81.2 (-86.6 to -74.7)	-97.9 (-98.5 to -97.2)	3.8 (-29.1 to 34.8)	-70.1 (-86.4 to -46.9)	-30.0 (-48.9 to -9.3)	-85.9 (-89.0 to -81.5)	-91.7 (-93.9 to -89.1)	-30.5 (-38.8 to -21.9)
Seychelles	Number: 1990	44 (42–47)	5 (4–7)	7 (6–8)	2 (1–3)	0 (0–0)	5 (3–8)	0 (0–0)	1 (0–2)	1 (1–1)	1 (0–2)	0 (0–0)
Seychelles	Number: 2015	26 (23–30)	2 (1–3)	3 (3–4)	1 (0–2)	0 (0–0)	4 (3–6)	0 (0–0)	1 (0–1)	0 (0–1)	0 (0–1)	0 (0–0)
Seychelles	Number: Cumulative change (%)	-40.9 (-48.5 to -32.2)	-61.2 (-75.1 to -43.9)	-48.0 (-58.1 to -36.3)	-31.2 (-65.1 to 15.3)	-78.0 (-83.9 to -70.4)	-6.9 (-32.8 to 22.1)	nan (nan–nan)	-7.3 (-59.7 to 84.1)	-43.1 (-55.0 to -28.9)	-28.5 (-72.9 to 41.2)	inf (inf–inf)
Seychelles	Rate: 1990	141.3 (133.8–150.0)	19.2 (14.1–24.7)	23.8 (19.8–27.5)	7.1 (4.3–10.5)	1.8 (1.5–2.3)	16.9 (12.0–26.8)	0.0 (0.0–0.0)	4.6 (2.4–8.2)	4.9 (4.2–5.8)	4.4 (2.2–7.7)	0.0 (0.0–0.0)
Seychelles	Rate: 2015	88.8 (78.8–100.5)	7.5 (5.2–10.3)	12.8 (10.1–15.2)	4.7 (2.8–7.1)	0.4 (0.3–0.5)	15.7 (11.8–22.8)	0.0 (0.0–0.0)	3.9 (2.0–6.4)	3.0 (2.5–3.5)	2.8 (1.4–5.0)	0.8 (0.4–1.4)
Seychelles	Rate: Cumulative change (%)	-37.1 (-45.1 to -27.9)	-60.4 (-74.5 to -42.6)	-45.8 (-56.2 to -33.6)	-29.7 (-64.3 to 18.2)	-77.0 (-83.1 to -69.1)	-3.8 (-30.5 to 26.0)	nan (nan–nan)	-5.3 (-58.9 to 88.4)	-39.1 (-51.7 to -24.0)	-27.0 (-72.3 to 44.2)	inf (inf–inf)
Thailand	Number: 1990	52,227 (49,137–55,650)	9,418 (7,880–11,258)	5,199 (4,181–6,133)	1,306 (889–1,829)	1,571 (1,193–1,937)	4,773 (2,477–6,266)	377 (248–561)	847 (602–1,229)	644 (487–905)	1,355 (1,015–1,798)	113 (75–172)
Thailand	Number: 2015	8,965 (8,136–9,904)	752 (550–997)	543 (450–653)	368 (218–560)	70 (52–93)	1,168 (795–1,514)	6 (3–10)	385 (180–562)	108 (82–142)	153 (103–220)	42 (24–75)
Thailand	Number: Cumulative change (%)	-82.8 (-84.6 to -80.7)	-91.9 (-94.2 to -89.2)	-89.4 (-91.7 to -86.5)	-70.8 (-83.9 to -51.8)	-95.4 (-96.9 to -93.6)	-73.0 (-84.3 to -45.7)	-98.2 (-99.1 to -96.8)	-52.9 (-75.9 to -26.5)	-82.7 (-88.1 to -75.7)	-88.3 (-93.1 to -81.9)	-63.3 (-75.4 to -46.3)
Thailand	Rate: 1990	242.3 (227.3–259.3)	47.7 (39.9–57.0)	24.9 (19.9–29.5)	6.6 (4.5–9.3)	7.5 (5.6–9.3)	23.6 (12.2–31.1)	1.6 (1.1–2.4)	4.3 (3.1–6.2)	2.9 (2.2–4.1)	6.9 (5.1–9.1)	0.5 (0.3–0.8)
Thailand	Rate: 2015	57.9 (52.2–64.4)	5.7 (4.2–7.6)	3.6 (2.9–4.3)	2.8 (1.7–4.3)	0.5 (0.4–0.7)	8.5 (5.8–11.1)	0.0 (0.0–0.1)	2.9 (1.4–4.3)	0.7 (0.5–0.9)	1.2 (0.8–1.7)	0.2 (0.1–0.5)
Thailand	Rate: Cumulative change (%)	-76.1 (-78.9 to -72.9)	-87.9 (-91.3 to -83.8)	-85.4 (-88.7 to -81.2)	-56.2 (-75.9 to -27.8)	-93.4 (-95.5 to -90.6)	-60.1 (-76.8 to -20.2)	-97.6 (-98.8 to -95.7)	-29.5 (-63.9 to 10.0)	-75.7 (-83.6 to -65.6)	-82.5 (-89.6 to -72.9)	-54.7 (-70.7 to -31.3)
Timor-Leste	Number: 1990	5,241 (4,888–5,604)	601 (472–741)	1,141 (544–1,603)	132 (78–210)	734 (319–1,111)	160 (69–390)	15 (2–33)	76 (31–140)	129 (40–245)	160 (60–303)	1 (0–5)
Timor-Leste	Number: 2015	1,962 (1,380–2,704)	315 (205–436)	321 (169–522)	132 (69–218)	118 (51–220)	183 (101–306)	1 (0–3)	75 (33–135)	56 (25–104)	90 (42–163)	8 (0–30)
Timor-Leste	Number: Cumulative change (%)	-62.5 (-73.8 to -47.8)	-46.6 (-66.1 to -21.5)	-68.4 (-85.6 to -37.2)	7.3 (-51.8 to 90.1)	-80.8 (-93.3 to -57.4)	44.7 (-46.3 to 152.2)	-81.3 (-96.2 to -42.7)	21.1 (-63.9 to 184.4)	-44.6 (-84.5 to 37.4)	-30.6 (-76.8 to 59.7)	inf (-29.2 to 1,359,754.8)
Timor-Leste	Rate: 1990	1,016.4 (949.6–1,084.4)	102.5 (80.3–126.3)	214.5 (100.3–303.4)	22.6 (13.3–35.8)	140.5 (59.7–212.5)	29.4 (12.4–71.4)	3.1 (0.6–6.8)	13.0 (5.4–24.0)	25.1 (7.8–46.9)	27.5 (10.4–51.7)	0.3 (0.0–1.1)
Timor-Leste	Rate: 2015	258.7 (186.7–353.1)	39.4 (25.7–54.4)	41.0 (21.9–66.1)	16.5 (8.6–27.3)	15.1 (6.7–27.8)	23.4 (13.2–38.9)	0.2 (0.1–0.5)	9.4 (4.2–16.9)	7.3 (3.4–13.4)	11.2 (5.3–20.4)	1.3 (0.0–4.7)
Timor-Leste	Rate: Cumulative change (%)	-74.5 (-81.8 to -65.0)	-60.9 (-75.2 to -42.4)	-78.4 (-90.2 to -57.5)	-21.5 (-64.7 to 38.8)	-87.1 (-95.5 to -71.0)	1.5 (-62.2 to 79.6)	-87.3 (-97.3 to -63.3)	-11.6 (-73.7 to 106.6)	-62.8 (-89.1 to -8.5)	-49.5 (-83.0 to 17.2)	inf (-48.1 to 915,311.9)
Vietnam	Number: 1990	113,305 (109,481–116,970)	18,075 (13,842–23,080)	17,777 (9,480–24,948)	2,153 (1,229–3,480)	1,881 (748–3,175)	10,890 (5,239–16,541)	1,057 (431–1,866)	2,819 (1,268–5,186)	1,582 (624–2,676)	3,978 (1,700–7,399)	0 (0–3)
Vietnam	Number: 2015	30,148 (27,234–33,188)	3,808 (2,668–5,178)	3,443 (2,786–4,201)	843 (469–1,374)	166 (112–241)	5,882 (3,989–7,624)	165 (96–258)	1,260 (656–2,128)	336 (192–492)	1,050 (528–1,822)	56 (6–296)
Vietnam	Number: Cumulative change (%)	-73.4 (-76.1 to -70.6)	-78.7 (-85.0 to -70.8)	-78.8 (-87.1 to -63.0)	-57.0 (-81.2 to -20.6)	-89.1 (-95.5 to -75.9)	-40.7 (-69.5 to -6.4)	-80.3 (-93.2 to -57.8)	-47.8 (-81.7 to 5.2)	-73.4 (-90.6 to -41.0)	-68.7 (-89.4 to -32.0)	22,358.6 (1,359.1–58,364.2)
Vietnam	Rate: 1990	325.5 (314.6–336.0)	50.6 (38.7–64.6)	50.1 (26.7–70.4)	6.0 (3.4–9.7)	5.4 (2.1–9.0)	30.7 (14.8–46.7)	3.1 (1.3–5.4)	7.9 (3.6–14.5)	4.5 (1.8–7.6)	11.1 (4.8–20.7)	0.0 (0.0–0.0)
Vietnam	Rate: 2015	103.8 (93.8–114.2)	13.1 (9.2–17.8)	11.8 (9.5–14.4)	2.9 (1.6–4.7)	0.6 (0.4–0.8)	20.2 (13.7–26.2)	0.6 (0.3–0.9)	4.3 (2.3–7.3)	1.2 (0.7–1.7)	3.6 (1.8–6.3)	0.2 (0.0–1.0)
Vietnam	Rate: Cumulative change (%)	-68.1 (-71.4 to -64.7)	-73.8 (-81.5 to -64.0)	-74.3 (-84.4 to -55.0)	-47.1 (-76.8 to -2.2)	-87.0 (-94.6 to -71.3)	-27.9 (-62.9 to 14.1)	-77.0 (-91.9 to -50.8)	-35.9 (-77.5 to 29.2)	-68.1 (-88.6 to -29.3)	-61.5 (-87.0 to -16.4)	25,971.3 (1,583.9–67,835.4)
Oceania	Number: 1990	19,733 (17,937–21,672)	1,410 (1,113–1,726)	4,610 (3,466–5,691)	700 (514–918)	1,526 (1,034–2,047)	647 (366–1,300)	592 (321–928)	231 (133–376)	546 (357–819)	592 (321–910)	13 (5–30)
Oceania	Number: 2015	15,005 (11,457–19,946)	1,244 (856–1,688)	2,778 (1,852–3,984)	738 (468–1,084)	617 (412–921)	851 (503–1,471)	413 (245–658)	314 (155–558)	371 (224–588)	671 (340–1,092)	64 (43–88)
Oceania	Number: Cumulative change (%)	-23.7 (-43.4 to 2.7)	-10.7 (-40.6 to 23.7)	-38.4 (-61.0 to -6.1)	8.9 (-37.8 to 72.9)	-57.8 (-75.4 to -31.2)	42.6 (-15.9 to 110.9)	-22.9 (-64.1 to 45.2)	45.0 (-30.1 to 168.5)	-27.9 (-63.8 to 23.0)	21.9 (-43.7 to 130.5)	506.6 (170.6–905.1)
Oceania	Rate: 1990	517.9 (471.7–567.6)	34.8 (27.5–42.6)	118.0 (89.1–146.0)	17.3 (12.7–22.7)	40.3 (27.4–54.0)	16.5 (9.3–33.0)	16.1 (9.1–25.1)	5.7 (3.3–9.3)	14.4 (9.5–21.7)	14.7 (8.0–22.5)	0.4 (0.2–0.8)
Oceania	Rate: 2015	290.4 (222.3–385.3)	23.5 (16.2–31.9)	53.1 (35.6–76.0)	14.0 (8.9–20.5)	12.0 (8.0–17.8)	16.2 (9.6–28.0)	8.3 (4.9–13.2)	5.9 (2.9–10.6)	7.2 (4.4–11.3)	12.7 (6.4–20.7)	1.3 (0.8–1.8)
Oceania	Rate: Cumulative change (%)	-43.7 (-58.2 to -24.7)	-31.7 (-54.5 to -5.4)	-54.0 (-70.8 to -29.8)	-16.8 (-52.4 to 32.0)	-69.0 (-81.8 to -49.6)	6.9 (-36.9 to 57.3)	-43.8 (-73.4 to 4.3)	10.8 (-46.6 to 104.6)	-47.2 (-73.1 to -10.3)	-7.0 (-57.1 to 75.7)	352.9 (102.9–649.6)
American Samoa	Number: 1990	41 (38–44)	6 (5–7)	4 (3–5)	3 (2–4)	0 (0–0)	3 (2–4)	0 (0–0)	0 (0–1)	0 (0–1)	1 (1–2)	0 (0–0)
American Samoa	Number: 2015	26 (23–30)	2 (1–3)	2 (1–2)	1 (1–2)	0 (0–0)	3 (2–4)	0 (0–0)	0 (0–0)	0 (0–0)	1 (0–1)	0 (0–0)
American Samoa	Number: Cumulative change (%)	-36.0 (-45.0 to -25.8)	-59.1 (-70.4 to -44.7)	-51.6 (-64.2 to -33.9)	-52.6 (-67.5 to -33.2)	-65.8 (-76.6 to -48.5)	3.1 (-29.3 to 40.4)	2.3 (-60.8 to 87.2)	-40.7 (-70.9 to 1.3)	-60.0 (-75.2 to -28.8)	-32.9 (-60.4 to 4.5)	825.8 (427.5–1,647.7)
American Samoa	Rate: 1990	142.5 (132.8–151.9)	18.1 (14.9–21.5)	13.9 (10.4–16.7)	10.8 (8.4–13.6)	2.1 (1.4–2.7)	10.9 (8.5–13.5)	0.0 (0.0–0.0)	2.1 (1.2–3.2)	3.2 (2.0–4.1)	5.7 (4.1–7.5)	0.0 (0.0–0.0)
American Samoa	Rate: 2015	67.2 (60.1–75.7)	5.5 (4.2–7.0)	4.8 (3.9–5.8)	3.7 (2.8–4.9)	0.5 (0.4–0.6)	7.9 (6.1–10.0)	0.0 (0.0–0.0)	0.9 (0.5–1.5)	0.8 (0.6–1.1)	2.7 (1.8–3.7)	0.1 (0.1–0.2)
American Samoa	Rate: Cumulative change (%)	-52.7 (-58.9 to -45.9)	-69.5 (-78.0 to -58.8)	-64.5 (-73.4 to -52.2)	-64.7 (-75.8 to -50.5)	-75.8 (-83.3 to -64.2)	-26.0 (-48.9 to 0.3)	-39.8 (-77.6 to 14.5)	-56.0 (-78.4 to -24.9)	-72.5 (-82.5 to -52.0)	-50.3 (-70.6 to -22.9)	599.8 (290.9–1,219.5)
Federated States of Micronesia	Number: 1990	168 (142–198)	24 (18–30)	28 (20–37)	10 (6–15)	5 (3–8)	9 (6–15)	0 (0–0)	3 (1–6)	5 (3–8)	8 (3–13)	0 (0–1)
Federated States of Micronesia	Number: 2015	48 (39–59)	4 (2–6)	4 (3–6)	3 (1–5)	0 (0–0)	4 (3–6)	0 (0–0)	1 (0–2)	0 (0–1)	2 (0–3)	0 (0–2)
Federated States of Micronesia	Number: Cumulative change (%)	-71.0 (-78.0 to -62.0)	-81.4 (-89.5 to -70.5)	-82.9 (-88.9 to -74.5)	-69.8 (-84.9 to -47.1)	-90.4 (-94.8 to -83.5)	-47.6 (-75.4 to -16.6)	nan (nan–nan)	-58.4 (-85.3 to -12.3)	-80.7 (-91.1 to -66.6)	-71.0 (-90.7 to -34.8)	948.8 (-5.4 to 3,337.7)
Federated States of Micronesia	Rate: 1990	292.3 (246.8–342.8)	40.1 (30.4–50.8)	47.9 (35.2–62.8)	17.9 (11.4–26.6)	9.4 (5.7–14.0)	16.4 (10.4–26.4)	0.0 (0.0–0.0)	5.7 (2.6–10.4)	9.5 (6.1–15.2)	13.4 (6.6–22.4)	0.7 (0.0–2.7)
Federated States of Micronesia	Rate: 2015	100.8 (80.7–125.3)	9.7 (5.8–14.9)	10.0 (7.1–13.5)	6.8 (3.8–11.4)	1.1 (0.6–1.7)	10.2 (6.9–14.0)	0.0 (0.0–0.0)	2.7 (1.3–5.2)	2.1 (1.2–3.2)	4.6 (1.7–8.7)	1.4 (0.1–5.3)
Federated States of Micronesia	Rate: Cumulative change (%)	-65.2 (-73.8 to -54.4)	-75.3 (-86.1 to -60.9)	-78.5 (-86.2 to -67.9)	-59.9 (-79.9 to -30.0)	-88.0 (-93.6 to -79.4)	-32.7 (-68.2 to 7.7)	nan (nan–nan)	-45.0 (-80.5 to 16.0)	-76.7 (-89.2 to -59.4)	-61.6 (-87.7 to -13.6)	1,162.5 (8.8–4,082.3)
Fiji	Number: 1990	773 (629–955)	93 (66–126)	135 (93–185)	71 (51–94)	27 (18–38)	56 (39–78)	0 (0–0)	23 (15–35)	21 (15–28)	25 (16–36)	0 (0–0)
Fiji	Number: 2015	735 (541–1,003)	94 (65–129)	108 (68–166)	55 (35–78)	31 (19–49)	85 (48–133)	0 (0–0)	22 (13–36)	18 (12–26)	20 (12–29)	1 (0–3)
Fiji	Number: Cumulative change (%)	-3.2 (-34.5 to 41.7)	4.8 (-39.1 to 65.3)	-16.2 (-55.1 to 43.2)	-19.5 (-53.6 to 27.3)	21.2 (-35.2 to 109.0)	60.5 (-24.5 to 190.3)	nan (nan–nan)	-1.2 (-46.6 to 60.1)	-10.3 (-49.7 to 42.7)	-14.5 (-57.4 to 53.1)	365.0 (194.7–640.0)

© 2017 Global Burden of Disease Child and Adolescent Health Collaboration. JAMA Pediatrics.

eTable 1. Number of Deaths, Death Rates (per 100,000 population), and Cumulative Percent Change with 95% Uncertainty Intervals (UI) for the Top 10 Global Causes of Death in 195 Countries and Territories, Aged 0 to 19, Both Sexes, 1990 and 2015*Note: best viewed by enlarging in browser.*

Location	Measure	All causes	Neonatal preterm birth complications	Lower respiratory infections	Neonatal encephalopathy due to birth asphyxia and trauma	Diarrheal diseases	Congenital anomalies	Malaria	Neonatal sepsis and other neonatal infections	Meningitis	Other neonatal disorders	HIV/AIDS
Fiji	Rate: 1990	205.8 (169.3–252.4)	24.0 (17.1–32.4)	35.2 (24.4–47.9)	18.2 (13.3–24.2)	7.1 (4.8–9.8)	14.5 (10.3–20.0)	0.0 (0.0–0.0)	6.1 (3.9–9.0)	5.7 (4.1–7.6)	6.5 (4.1–9.3)	0.1 (0.1–0.2)
Fiji	Rate: 2015	225.6 (165.9–307.5)	29.4 (20.3–40.1)	33.3 (20.9–51.1)	17.2 (11.2–24.4)	9.5 (6.0–15.1)	26.2 (14.8–40.9)	0.0 (0.0–0.0)	7.0 (4.1–11.3)	5.6 (3.7–8.2)	6.3 (3.9–9.3)	0.6 (0.3–1.0)
Fiji	Rate: Cumulative change (%)	11.5 (-24.3 to 62.3)	27.4 (-26.0 to 100.8)	-1.1 (-46.6 to 68.8)	-2.2 (-43.7 to 54.6)	41.8 (-24.0 to 144.7)	91.4 (-10.2 to 247.3)	nan (nan–nan)	20.1 (-35.1 to 94.5)	2.7 (-42.3 to 62.5)	3.8 (-48.2 to 85.8)	453.4 (249.8–790.3)
Guam	Number: 1990	63 (59–67)	7 (6–8)	6 (5–7)	4 (3–5)	0 (0–0)	8 (7–9)	0 (0–0)	1 (0–2)	1 (0–1)	2 (1–2)	0 (0–0)
Guam	Number: 2015	75 (65–87)	8 (5–11)	6 (5–8)	3 (2–5)	0 (0–0)	11 (7–14)	0 (0–0)	2 (1–3)	0 (0–0)	5 (3–7)	0 (0–0)
Guam	Number: Cumulative change (%)	19.7 (2.8–39.2)	17.6 (-24.1 to 68.1)	4.2 (-21.8 to 35.5)	-7.4 (-40.1 to 31.9)	-42.4 (-59.8 to -21.5)	33.1 (-7.5 to 80.4)	-3.2 (-68.0 to 111.8)	64.2 (-4.6 to 148.8)	-40.0 (-65.4 to 24.8)	131.5 (43.4–258.6)	242.0 (128.2–451.3)
Guam	Rate: 1990	110.4 (103.8–117.2)	12.2 (10.1–14.3)	10.5 (8.8–12.2)	6.8 (5.4–8.4)	0.7 (0.5–0.8)	13.9 (11.6–16.3)	0.0 (0.0–0.0)	2.4 (1.6–4.1)	2.2 (1.3–2.8)	3.7 (2.8–4.8)	0.1 (0.0–0.2)
Guam	Rate: 2015	136.0 (117.8–158.5)	16.2 (11.2–22.1)	11.8 (9.4–14.8)	7.1 (5.0–9.5)	0.4 (0.3–0.5)	20.5 (14.8–26.4)	0.0 (0.0–0.0)	4.3 (2.7–6.1)	1.2 (0.9–1.8)	9.5 (6.6–13.5)	0.3 (0.2–0.5)
Guam	Rate: Cumulative change (%)	23.3 (5.2–44.0)	35.2 (-12.7 to 93.3)	14.2 (-14.6 to 48.8)	6.5 (-31.1 to 51.8)	-37.6 (-56.4 to -14.6)	48.9 (3.3–101.0)	-20.9 (-73.7 to 72.4)	89.0 (9.7–186.3)	-38.0 (-64.0 to 29.7)	166.4 (65.1–312.7)	255.5 (137.5–473.6)
Kiribati	Number: 1990	253 (216–294)	29 (22–37)	30 (21–39)	18 (12–25)	41 (29–54)	2 (1–4)	0 (0–0)	5 (2–8)	18 (11–27)	10 (4–17)	0 (0–0)
Kiribati	Number: 2015	160 (109–235)	19 (11–28)	18 (10–31)	15 (8–24)	8 (4–14)	3 (1–5)	0 (0–0)	7 (3–13)	7 (4–13)	10 (4–19)	0 (0–0)
Kiribati	Number: Cumulative change (%)	-36.1 (-57.5 to -4.5)	-34.7 (-62.8 to 3.1)	-36.1 (-67.0 to 9.4)	-12.6 (-58.8 to 53.9)	-78.1 (-89.1 to -59.7)	22.8 (-40.5 to 115.9)	nan (nan–nan)	68.8 (-36.2 to 274.9)	-52.2 (-79.6 to -6.2)	20.0 (-62.7 to 162.1)	147.7 (76.4–272.5)
Kiribati	Rate: 1990	582.0 (501.3–672.9)	62.3 (48.0–77.6)	67.7 (48.9–87.4)	39.0 (26.7–54.4)	93.6 (66.0–123.7)	6.3 (3.5–9.1)	0.0 (0.0–0.0)	10.8 (5.5–18.8)	41.9 (27.3–62.0)	21.3 (10.3–37.0)	0.0 (0.0–0.1)
Kiribati	Rate: 2015	288.0 (199.2–415.2)	32.6 (19.3–49.2)	33.3 (18.8–55.0)	26.5 (14.3–42.5)	15.6 (8.7–26.3)	5.8 (3.1–9.5)	0.0 (0.0–0.0)	13.0 (5.8–23.7)	14.4 (7.8–24.7)	17.8 (7.5–33.3)	0.1 (0.0–0.2)
Kiribati	Rate: Cumulative change (%)	-50.2 (-66.6 to -26.4)	-46.8 (-69.6 to -16.1)	-49.3 (-73.5 to -14.0)	-29.0 (-66.6 to 24.7)	-82.7 (-91.2 to -68.7)	-2.3 (-52.5 to 70.7)	nan (nan–nan)	37.2 (-48.0 to 204.8)	-62.9 (-83.6 to -28.6)	-2.6 (-69.7 to 113.8)	104.1 (46.9–205.8)
Marshall Islands	Number: 1990	95 (76–115)	14 (10–18)	16 (11–22)	5 (3–8)	4 (2–7)	5 (2–9)	0 (0–0)	1 (0–3)	3 (1–5)	4 (2–7)	0 (0–1)
Marshall Islands	Number: 2015	50 (36–70)	6 (3–10)	6 (3–9)	3 (1–5)	0 (0–1)	4 (2–6)	0 (0–0)	1 (0–2)	1 (0–1)	2 (0–4)	0 (0–2)
Marshall Islands	Number: Cumulative change (%)	-46.0 (-63.2 to -23.9)	-54.4 (-77.2 to -23.7)	-63.0 (-78.8 to -40.2)	-34.0 (-71.2 to 25.5)	-80.4 (-90.6 to -65.5)	-8.4 (-56.2 to 56.5)	nan (nan–nan)	-10.6 (-71.7 to 103.8)	-62.7 (-83.9 to -32.8)	-38.0 (-81.4 to 34.1)	1,223.9 (6.1–4,256.9)
Marshall Islands	Rate: 1990	305.6 (248.2–364.8)	41.5 (30.5–53.4)	51.7 (36.5–68.9)	16.5 (9.5–24.7)	14.0 (8.5–21.8)	15.3 (9.1–28.4)	0.0 (0.0–0.0)	5.1 (2.4–9.2)	10.1 (6.2–16.0)	13.5 (6.5–22.7)	1.1 (0.0–4.0)
Marshall Islands	Rate: 2015	151.0 (109.1–206.8)	18.6 (9.5–29.8)	17.7 (11.5–26.5)	10.2 (4.8–17.7)	2.4 (1.4–3.8)	12.2 (7.3–19.0)	0.0 (0.0–0.0)	3.9 (1.5–7.5)	3.1 (1.9–4.9)	7.3 (2.8–14.1)	1.5 (0.1–6.5)
Marshall Islands	Rate: Cumulative change (%)	-49.9 (-65.5 to -29.9)	-54.2 (-77.0 to -23.4)	-64.7 (-79.6 to -43.4)	-33.9 (-71.2 to 25.6)	-81.9 (-91.1 to -68.6)	-12.4 (-57.7 to 48.3)	nan (nan–nan)	-10.7 (-71.6 to 102.8)	-66.3 (-84.8 to -41.0)	-38.0 (-81.4 to 34.4)	1,134.7 (0.6–3,972.2)
Northern Mariana Islands	Number: 1990	19 (13–28)	2 (1–4)	2 (1–3)	1 (0–1)	0 (0–0)	1 (0–2)	0 (0–0)	0 (0–0)	0 (0–0)	1 (0–2)	0 (0–0)
Northern Mariana Islands	Number: 2015	16 (11–21)	1 (0–2)	1 (0–1)	0 (0–1)	0 (0–0)	2 (1–3)	0 (0–0)	0 (0–0)	0 (0–0)	0 (0–1)	0 (0–0)
Northern Mariana Islands	Number: Cumulative change (%)	-14.2 (-50.8 to 33.6)	-44.8 (-79.1 to 12.7)	-41.7 (-72.6 to 2.7)	-31.0 (-74.1 to 47.0)	-39.0 (-72.2 to 14.4)	57.4 (-32.4 to 194.7)	18.5 (-60.6 to 141.8)	9.9 (-74.0 to 150.2)	-40.5 (-73.1 to 13.3)	-26.5 (-79.3 to 66.3)	1,629.3 (986.7–2,851.0)
Northern Mariana Islands	Rate: 1990	133.0 (91.3–187.6)	17.8 (8.8–29.4)	14.2 (8.3–22.0)	7.0 (3.3–11.7)	0.9 (0.5–1.4)	10.6 (6.0–16.9)	0.0 (0.0–0.1)	2.1 (0.8–4.4)	1.1 (0.6–1.7)	6.5 (2.6–12.5)	0.0 (0.0–0.0)
Northern Mariana Islands	Rate: 2015	53.7 (39.8–71.7)	4.6 (2.4–7.5)	3.8 (2.5–5.6)	2.2 (1.2–3.8)	0.2 (0.1–0.3)	7.6 (4.8–11.5)	0.0 (0.0–0.0)	1.0 (0.3–2.0)	0.3 (0.2–0.4)	2.0 (1.0–3.6)	0.1 (0.1–0.2)
Northern Mariana Islands	Rate: Cumulative change (%)	-57.6 (-74.9 to -35.6)	-70.5 (-88.9 to -39.8)	-70.5 (-85.8 to -49.1)	-63.2 (-86.2 to -21.6)	-70.2 (-86.3 to -44.3)	-19.4 (-65.1 to 50.0)	-51.5 (-84.0 to -0.1)	-41.5 (-86.1 to 33.4)	-71.3 (-86.5 to -46.7)	-60.9 (-89.0 to -11.5)	739.7 (429.7–1,342.0)
Papua New Guinea	Number: 1990	15,751 (14,062–17,574)	981 (713–1,272)	3,898 (2,868–4,915)	477 (309–678)	1,278 (825–1,765)	459 (216–1,019)	526 (284–828)	157 (74–282)	420 (248–670)	450 (215–738)	7 (3–12)
Papua New Guinea	Number: 2015	12,399 (8,999–17,074)	948 (578–1,368)	2,410 (1,545–3,568)	569 (321–889)	528 (332–818)	634 (326–1,225)	387 (230–622)	244 (103–466)	304 (165–511)	561 (248–963)	50 (34–66)
Papua New Guinea	Number: Cumulative change (%)	-20.9 (-45.0 to 10.1)	-1.2 (-41.6 to 45.5)	-36.5 (-62.3 to 0.6)	26.6 (-39.0 to 124.7)	-56.4 (-76.4 to -25.9)	55.4 (-18.7 to 148.6)	-17.9 (-62.4 to 57.6)	74.9 (-35.0 to 263.6)	-21.3 (-65.6 to 47.5)	39.7 (-46.0 to 180.6)	662.3 (339.6–1,206.4)
Papua New Guinea	Rate: 1990	636.6 (569.7–709.7)	37.0 (26.9–47.9)	153.7 (112.9–193.8)	18.0 (11.7–25.6)	52.3 (33.8–72.3)	17.9 (8.4–39.7)	22.1 (12.2–34.8)	6.0 (2.8–10.6)	17.1 (10.1–27.4)	17.1 (8.1–27.8)	0.3 (0.2–0.5)
Papua New Guinea	Rate: 2015	326.2 (237.7–447.4)	24.2 (14.8–34.9)	62.5 (40.2–92.7)	14.5 (8.2–22.7)	14.0 (8.9–21.5)	16.4 (8.4–31.6)	10.7 (6.4–17.0)	6.2 (2.6–11.9)	8.0 (4.4–13.3)	14.4 (6.4–24.6)	1.3 (0.9–1.8)
Papua New Guinea	Rate: Cumulative change (%)	-48.5 (-63.9 to -28.7)	-33.1 (-60.4 to -1.5)	-58.2 (-75.1 to -33.6)	-14.3 (-58.7 to 51.8)	-71.8 (-84.6 to -51.8)	2.9 (-46.4 to 64.8)	-46.7 (-75.5 to 1.5)	18.2 (-56.0 to 146.7)	-49.2 (-77.5 to -5.1)	-5.7 (-63.4 to 89.6)	402.7 (188.2–760.1)
Samoa	Number: 1990	153 (107–217)	19 (9–31)	19 (11–30)	10 (4–19)	2 (1–4)	11 (6–17)	0 (0–0)	3 (1–7)	4 (2–7)	7 (2–14)	0 (0–2)
Samoa	Number: 2015	69 (48–97)	5 (2–9)	5 (3–9)	4 (1–8)	0 (0–0)	8 (4–13)	0 (0–0)	1 (0–3)	1 (0–1)	2 (0–5)	1 (0–4)
Samoa	Number: Cumulative change (%)	-53.2 (-71.0 to -29.7)	-71.1 (-87.2 to -42.2)	-66.9 (-83.1 to -43.3)	-54.4 (-82.7 to -5.2)	-82.5 (-91.8 to -68.9)	-23.1 (-65.1 to 38.1)	nan (nan–nan)	-39.1 (-82.2 to 37.7)	-68.2 (-85.7 to -42.5)	-54.5 (-86.5 to 5.1)	1,126.5 (2.0–3,918.5)
Samoa	Rate: 1990	158.1 (113.3–223.0)	19.2 (9.8–32.0)	19.3 (11.4–30.2)	10.6 (4.8–19.1)	2.7 (1.3–4.9)	11.5 (6.5–17.9)	0.0 (0.0–0.0)	3.7 (1.5–7.5)	4.3 (2.5–7.2)	7.2 (2.5–14.7)	0.7 (0.0–2.4)
Samoa	Rate: 2015	76.1 (53.3–107.2)	5.7 (2.5–10.8)	6.4 (3.8–10.1)	4.8 (1.9–9.1)	0.5 (0.2–0.9)	8.8 (4.7–15.3)	0.0 (0.0–0.0)	2.1 (0.8–4.3)	1.3 (0.7–2.1)	3.1 (0.8–6.6)	1.4 (0.1–4.9)
Samoa	Rate: Cumulative change (%)	-50.3 (-68.8 to -25.7)	-67.8 (-85.7 to -35.4)	-64.4 (-81.7 to -39.6)	-49.1 (-80.7 to 6.1)	-81.6 (-91.3 to -67.5)	-16.7 (-61.9 to 49.9)	nan (nan–nan)	-32.1 (-80.3 to 53.4)	-67.1 (-85.0 to -41.3)	-49.2 (-85.1 to 17.3)	1,238.6 (13.9–4,260.0)
Solomon Islands	Number: 1990	694 (582–817)	85 (56–119)	106 (70–144)	33 (19–51)	44 (25–68)	27 (15–50)	18 (7–36)	8 (3–16)	19 (10–30)	24 (10–43)	1 (0–5)
Solomon Islands	Number: 2015	498 (395–627)	61 (34–94)	61 (42–86)	33 (17–52)	14 (9–22)	37 (24–54)	5 (1–11)	10 (4–19)	11 (6–17)	21 (7–39)	4 (0–14)
Solomon Islands	Number: Cumulative change (%)	-27.4 (-46.5 to -4.3)	-25.9 (-57.6 to 11.2)	-39.2 (-62.8 to -4.2)	4.8 (-51.0 to 82.9)	-64.5 (-80.5 to -35.8)	52.6 (-18.5 to 141.6)	-68.1 (-89.3 to -31.2)	38.6 (-51.9 to 199.6)	-35.5 (-71.9 to 17.5)	0.9 (-64.6 to 116.3)	1,716.9 (39.1–5,495.1)
Solomon Islands	Rate: 1990	332.3 (280.1–389.2)	37.5 (24.8–52.4)	48.6 (32.5–66.2)	14.9 (8.7–22.7)	21.0 (12.2–31.7)	12.3 (7.1–22.7)	9.0 (4.0–17.1)	3.8 (1.7–7.1)	9.3 (5.4–14.4)	10.6 (4.4–19.2)	0.9 (0.0–2.7)
Solomon Islands	Rate: 2015	164.6 (131.5–206.4)	19.7 (11.1–30.2)	19.8 (13.9–27.7)	10.6 (5.5–16.6)	4.7 (2.9–7.2)	12.0 (8.0–17.5)	1.7 (0.6–3.5)	3.3 (1.4–6.1)	3.7 (2.2–5.6)	6.7 (2.5–12.7)	1.5 (0.1–4.9)
Solomon Islands	Rate: Cumulative change (%)	-50.0 (-62.6 to -34.5)	-46.1 (-69.2 to -19.0)	-57.4 (-73.8 to -33.5)	-23.9 (-64.4 to 33.1)	-75.8 (-86.6 to -57.4)	7.0 (-42.1 to 68.9)	-79.0 (-92.7 to -55.1)	0.4 (-65.0 to 116.0)	-56.9 (-80.5 to -22.9)	-26.9 (-74.4 to 57.0)	1,175.7 (2.3–3,755.3)
Tonga	Number: 1990	101 (77–131)	15 (9–22)	10 (6–15)	2 (1–4)	1 (0–2)	6 (4–10)	0 (0–0)	4 (1–8)	7 (4–11)	6 (3–11)	0 (0–0)
Tonga	Number: 2015	67 (50–91)	8 (4–14)	5 (3–8)	1 (0–3)	0 (0–0)	5 (3–9)	0 (0–0)	3 (1–6)	4 (3–6)	4 (2–8)	0 (0–0)
Tonga	Number: Cumulative change (%)	-32.1 (-53.2 to -3.6)	-40.8 (-71.2 to 3.6)	-40.5 (-65.7 to -3.3)	-25.6 (-72.0 to 54.8)	-57.9 (-78.6 to -26.3)	-9.5 (-48.4 to 49.0)	nan (nan–nan)	-11.3 (-70.1 to 87.5)	-32.5 (-63.1 to 10.4)	-19.8 (-71.8 to 67.9)	1,272.7 (714.7–2,307.4)
Tonga	Rate: 1990	193.2 (148.1–247.4)	28.9 (18.1–41.1)	19.5 (12.7–29.1)	4.9 (2.5–8.3)	2.9 (1.7–4.6)	12.9 (8.4–18.7)	0.0 (0.0–0.0)	8.2 (3.7–15.1)	13.9 (8.4–21.3)	12.5 (5.9–21.7)	0.0 (0.0–0.0)
Tonga	Rate: 2015	138.1 (101.9–188.1)	18.5 (9.7–30.2)	12.1 (8.1–17.5)	3.7 (1.7–6.5)	1.2 (0.8–1.9)	12.3 (7.8–18.6)	0.0 (0.0–0.0)	7.2 (3.0–13.5)	9.3 (6.1–14.0)	10.0 (4.3–18.5)	0.3 (0.2–0.5)
Tonga	Rate: Cumulative change (%)	-27.2 (-49.5 to 2.6)	-32.7 (-67.3 to 17.8)	-34.8 (-62.3 to 4.4)	-15.6 (-68.3 to 75.6)	-54.3 (-76.7 to -19.4)	0.2 (-42.8 to 64.1)	nan (nan–nan)	0.7 (-66.2 to 112.6)	-29.1 (-61.2 to 15.8)	-9.0 (-67.9 to 90.0)	1,403.9 (789.7–2,514.7)
Vanuatu	Number: 1990	259 (219–303)	35 (25–47)	39 (24–54)	14 (8–21)	13 (7–21)	11 (6–20)	6 (2–12)	4 (2–7)	7 (3–11)	10 (5–19)	1 (0–2)
Vanuatu	Number: 2015	259 (209–327)	34 (23–47)	39 (27–55)	17 (9–27)	7 (4–11)	18 (11–28)	2 (0–5)	6 (2–12)	6 (3–9)	12 (6–21)	1 (0–6)
Vanuatu	Number: Cumulative change (%)	0.8 (-23.0 to 32.3)	-1.5 (-40.4 to 44.4)	7.4 (-37.1 to 80.7)	31.6 (-36.1 to 128.8)	-39.7 (-71.1 to 11.8)	70.6 (-4.0 to 171.6)	-53.8 (-87.4 to 4.7)	72.9 (-39.5 to 287.4)	-3.3 (-58.4 to 81.7)	32.0 (-49.6 to 172.8)	1,577.4 (24.6–5,158.7)
Vanuatu	Rate: 1990	290.1 (247.2–337.6)	37.0 (26.1–48.8)	42.5 (26.1–58.7)	14.7 (8.7–22.0)	15.0 (8.0–22.7)	12.3 (7.0–21.7)	7.1 (2.9–13.7)	4.6 (2.1–8.2)	7.9 (4.4–12.9)	11.3 (5.3–19.9)	1.3 (0.0–3.0)
Vanuatu	Rate: 2015	210.6 (171.3–266.0)	27.2 (18.3–37.8)	31.7 (21.9–44.4)	13.9 (7.8–21.7)	6.0 (3.6–9.2)	14.5 (9.0–22.4)	1.9 (0.7–4.2)	5.2 (2.2–9.8)	4.9 (2.9–7.6)	9.9 (4.8–16.8)	1.6 (0.1–5.4)
Vanuatu	Rate: Cumulative change (%)	-26.8 (-44.0 to -4.5)	-24.4 (-54.3 to 10.8)	-20.1 (-52.7 to 33.6)	0.9 (-50.9 to 75.5)	-55.8 (-78.6 to -18.9)	27.6 (-27.9 to 103.9)	-68.2 (-90.9 to -27.8)	32.5 (-54.2 to 197.0)	-31.6 (-69.5 to 25.5)	1.1 (-61.3 to 109.2)	1,163.9 (-3.3 to 3,822.8)
North Africa and Middle East	Number: 1990	1,045,563 (1,024,123–1,069,193)	153,951 (140,401–171,700)	168,843 (145,136–192,499)	28,635 (23,501–34,111)	123,274 (105,424–141,064)	105,344 (69,284–174,792)	4,665 (2,290–7,695)	17,328 (11,731–22,308)	23,954 (15,673–31,998)	37,480 (28,604–47,079)	91 (45–172)
North Africa and Middle East	Number: 2015	529,160 (499,142–562,252)	83,998 (75,004–93,033)	55,221 (47,534–63,265)	19,930 (16,625–23,789)	24,608 (19,776–30,375)	81,812 (69,600–99,956)	4,371 (2,282–7,413)	17,737 (10,289–22,632)	11,040 (8,413–14,933)	19,646 (15,164–24,838)	494 (396–611)
North Africa and Middle East	Number: Cumulative change (%)	-49.4 (-52.3 to -46.1)	-45.3 (-51.5 to -39.0)	-67.1 (-71.8 to -61.7)	-29.7 (-43.8 to -12.2)	-79.9 (-84.1 to -75.2)	-18.6 (-41.5 to 11.3)	-0.0 (-47.6 to 69.2)	3.4 (-21.7 to 36.9)	-52.3 (-67.0 to -33.1)	-46.7 (-61.8 to -28.7)	540.1 (199.2–945.9)
North Africa and Middle East	Rate: 1990	531.5 (520.4–543.4)	75.1 (68.5–83.8)	84.4 (72.5–96.1)	14.0 (11.5–16.6)	61.4 (52.5–70.3)	52.3 (34.4–86.7)	2.4 (1.2–3.9)	8.5 (5.7–10.9)	12.1 (7.9–16.1)	18.3 (14.0–23.0)	0.0 (0.0–0.1)
North Africa and Middle East	Rate: 2015	222.5 (209.8–236.6)	34.3 (30.6–37.9)	22.8 (19.6–26.0)	8.1 (6.8–9.7)	10.1 (8.1–12.4)	33.6 (28.6–41.0)	1.8 (1.0–3.1)	7.2 (4.2–9.2)	4.6 (3.5–6.2)	8.0 (6.2–10.1)	0.2 (0.2–0.3)
North Africa and Middle East	Rate: Cumulative change (%)	-58.1 (-60.4 to -55.5)	-54.3 (-59.5 to -49.0)	-72.9 (-76.7 to -68.4)	-41.2 (-53.0 to -26.6)	-83.5 (-86.9 to -79.6)	-32.6 (-51.6 to -8.0)	-18.1 (-56.7 to 37.5)	-13.8 (-34.8 to 14.1)	-61.0 (-72.9 to -45.6)	-55.5 (-68.1 to -40.5)	435.1 (154.3–769.2)
North Africa and Middle East	Number: 1990	1,045,563 (1,024,123–1,069,193)	153,951 (140,401–171,700)	168,843 (145,136–192,499)	28,635 (23,501–34,111)	123,274 (105,424–141,064)	105,344 (69,284–174,792)	4,665 (2,290–7,695)	17,328 (11,731–22,308)	23,954 (15,673–31,998)	37,480 (28,604–47,079)	91 (45–172)
North Africa and Middle East	Number: 2015	529,160 (499,142–562,252)	83,998 (75,004–93,033)	55,221 (47,534–63,265)	19,930 (16,625–23,789)	24,608 (19,776–30,375)	81,812 (69,600–99,956)	4,371 (2,282–7,413)	17,737 (10,289–22,632)	11,040 (8,413–14,933)	19,646 (15,164–24,838)	494 (396–611)
North Africa and Middle East	Number: Cumulative change (%)	-49.4 (-52.3 to -46.1)	-45.3 (-51.5 to -39.0)	-67.1 (-71.8 to -61.7)	-29.7 (-43.8 to -12.2)	-79.9 (-84.1 to -75.2)	-18.6 (-41.5 to 11.3)	-0.0 (-47.6 to 69.2)	3.4 (-21.7 to 36.9)	-52.3 (-67.0 to -33.1)	-46.7 (-61.8 to -28.7)	540.1 (199.2–945.9)
North Africa and Middle East	Rate: 1990	531.5 (520.4–543.4)	75.1 (68.5–83.8)	84.4 (72.5–96.1)	14.0 (11.5–16.6)	61.4 (52.5–70.3)	52.3 (34.4–86.7)	2.4 (1.2–3.9)	8.5 (5.7–10.9)	12.1 (7.9–16.1)	18.3 (14.0–23.0)	0.0 (0.0–0.1)
North Africa and Middle East	Rate: 2015	222.5 (209.8–236.6)	34.3 (30.6–37.9)	22.8 (19.6–26.0)	8.1 (6.8–9.7)	10.1 (8.1–12.4)	33.6 (28.6–41.0)	1.8 (1.0–3.1)	7.2 (4.2–9.2)	4.6 (3.5–6.2)	8.0 (6.2–10.1)	0.2 (0.2–0.3)
North Africa and Middle East	Rate: Cumulative change (%)	-58.1 (-60.4 to -55.5)	-54.3 (-59.5 to -49.0)	-72.9 (-76.7 to -68.4)	-41.2 (-53.0 to -26.6)	-83.5 (-86.9 to -79.6)	-32.6 (-51.6 to -8.0)	-18.1 (-56.7 to 37.5)	-13.8 (-34.8 to 14.1)	-61.0 (-72.9 to -45.6)	-55.5 (-68.1 to -40.5)	435.1 (154.3–769.2)
Afghanistan	Number: 1990	109,314 (103,391–115,412)	7,548 (4,789–11,141)	21,897 (10,722–30,057)	3,342 (1,812–5,036)	10,065 (4,463–15,308)	6,929 (2,713–15,977)	2,079 (453–4,551)	925 (376–1,764)	5,170 (2,248–8,705)	4,132 (1,557–7,378)	5 (0–24)
Afghanistan	Number: 2015	117,891 (104,488–132,494)	10,547 (7,409–14,335)	20,502 (15,164–26,231)	5,200 (3,262–7,661)	6,225 (3,949–9,025)	10,784 (5,810–21,271)	2,473 (796–5,102)	2,308 (1,104–4,105)	5,826 (3,559–9,402)	4,683 (1,919–8,012)	22 (5–63)
Afghanistan	Number: Cumulative change (%)	8.0 (-6.0 to 23.0)	46.4 (-6.0 to 122.5)	2.9 (-35.8 to 84.6)	68.1 (-11.7 to 185.0)	-28.7 (-65.8 to 36.7)	79.6 (10.0–176.6)	61.8 (-51.2 to 286.8)	200.7 (13.3–560.8)	33.9 (-43.8 to 179.6)	26.9 (-44.0 to 136.7)	1,444.4 (40.0–4,140.2)
Afghanistan	Rate: 1990	1,119.4 (1,060.6–1,177.1)	67.2 (42.6–99.5)	214.9 (102.1–297.0)	29.9 (16.3–44.7)	98.1 (42.6–149.5)	66.3 (25.5–153.6)	22.2 (5.6–47.9)	8.3 (3.4–15.8)	51.9 (22.7–87.9)	37.0 (14.0–65.9)	0.1 (0.0–0.3)
Afghanistan	Rate: 2015	626.7 (554.3–705.1)	53.9 (37.9–73.3)	106.9 (79.0–136.7)	26.6 (16.7–39.2)	32.5 (20.6–47.0)	56.0 (30.1–110.6)	13.4 (4.5–27.3)	11.8 (5.7–21.1)	30.5 (18.7–49.1)	24.0 (9.8–41.0)	0.1 (0.0–0.3)
Afghanistan	Rate: Cumulative change (%)	-43.9 (-51.2 to -36.2)	-16.0 (-45.9 to 27.6)	-45.0 (-66.2 to 0.4)	-3.8 (-49.1 to 63.1)	-61.7 (-81.6 to -25.4)	-1.6 (-41.1 to 53.5)	-22.4 (-74.8 to 75.4)	72.1 (-34.9 to 279.0)	-30.0 (-70.3 to 47.9)	-27.6 (-67.8 to 34.5)	657.1 (-28.8 to 1,902.0)
Algeria	Number: 1990	54,358 (50,890–57,946)	11,081 (9,017–13,155)	4,053 (3,030–5,356)	2,745 (1,651–4,058)	4,023 (2,668–5,525)	5,853 (4,220–8,468)	1 (1–2)	2,790 (1,497–4,520)	1,427 (843–2,380)	2,043 (971–3,560)	2 (1–4)
Algeria	Number: 2015	25,745 (21,882–30,616)	5,561 (4,106–7,233)	1,226 (925–1,573)	1,826 (1,103–2,712)	644 (413–952)	4,363 (3,381–5,670)	1 (0–1)	2,103 (1,156–3,324)	498 (320–780)	1,202 (580–2,074)	7 (1–21)
Algeria	Number: Cumulative change (%)	-52.6 (-60.4 to -43.4)	-49.2 (-63.5 to -31.2)	-68.8 (-79.6 to -54.5)	-28.8 (-62.5 to 22.0)	-83.1 (-90.9 to -72.0)	-22.2 (-50.4 to 9.6)	-19.8 (-49.2 to 13.0)	-17.8 (-59.7 to 56.9)	-61.9 (-81.5 to -31.2)	-31.0 (-77.0 to 47.9)	inf (-48.0 to 596.3)
Algeria	Rate: 1990	363.6 (340.7–387.2)	73.1 (59.5–86.8)	26.9 (20.2–35.5)	18.1 (10.9–26.7)	26.5 (17.6–36.4)	38.7 (27.9–56.0)	0.0 (0.0–0.0)	18.4 (9.9–29.8)	9.5 (5.6–15.7)	13.5 (6.4–23.5)	0.0 (0.0–0.0)
Algeria	Rate: 2015	157.4 (134.8–185.7)	32.4 (23.9–42.1)	7.6 (5.8–9.6)	10.6 (6.4–15.8)	3.8 (2.5–5.6)	25.7 (20.0–33.3)	0.0 (0.0–0.0)	12.3 (6.7–19.4)	3.1 (2.0–4.7)	7.0 (3.4–12.1)	0.0 (0.0–0.1)
Algeria	Rate: Cumulative change (%)	-56.7 (-63.5 to -48.7)	-55.1 (-67.7 to -39.3)	-71.0 (-80.9 to -58.4)	-37.1 (-66.9 to 8.0)	-85.0 (-91.8 to -75.1)	-30.8 (-55.9 to -2.7)	-17.9 (-50.0 to 17.4)	-27.5 (-64.4 to 38.5)	-64.8 (-82.3 to -38.2)	-39.1 (-79.7 to 30.6)	inf (-42.5 to 565.7)
Bahrain	Number: 1990	376 (359–394)	87 (76–99)	26 (20–31)	17 (12–22)	16 (12–20)	84 (71–97)	0 (0–0)	3 (2–8)	3 (3–4)	14 (10–19)	0 (0–0)
Bahrain	Number: 2015	193 (174–214)	25 (20–30)	11 (8–13)	6 (4–7)	3 (2–4)	58 (46–72)	0 (0–0)	5 (3–7)	1 (0–1)	6 (4–7)	0 (0–0)
Bahrain	Number: Cumulative change (%)	-48.7 (-54.0 to -42.8)	-70.9 (-77.6 to -63.4)	-56.1 (-66.7 to -43.4)	-63.2 (-75.6 to -47.4)	-80.3 (-86.6 to -72.1)	-29.9 (-47.5 to -7.5)	nan (nan–nan)	60.2 (-34.1 to 160.5)	-73.2 (-81.8 to -57.5)	-55.1 (-74.7 to -32.3)	inf (184.2–598.8)
Bahrain	Rate: 1990	156.3 (149.5–163.0)	33.0 (29.1–37.6)	10.5 (8.2–12.6)	6.5 (4.8–8.4)	6.1 (4.6–7.8)	32.4 (27.6–37.7)	0.0 (0.0–0.0)	1.5 (0.8–3.3)	1.6 (1.2–2.0)	5.4 (3.9–7.5)	0.0 (0.0–0.1)
Bahrain	Rate: 2015	52.3 (47.1–57.9)	6.9 (5.6–8.5)	3.1 (2.2–3.7)	1.7 (1.3–2.2)	0.8 (0.6–1.1)	15.9 (12.7–19.8)	0.0 (0.0–0.0)	1.5 (0.9–2.0)	0.3 (0.2–0.4)	1.7 (1.2–2.2)	0.1 (0.1–0.2)
Bahrain	Rate: Cumulative change (%)	-66.5 (-70.1 to -62.7)	-78.9 (-83.8 to -73.5)	-70.4 (-77.4 to -62.2)	-73.4 (-82.3 to -61.8)	-86.0 (-90.5 to -80.3)	-50.5 (-62.9 to -34.7)	nan (nan–nan)	16.0 (-52.3 to 88.7)	-82.3 (-87.9 to -71.8)	-67.5 (-81.7 to -50.9)	inf (84.8–370.5)
Egypt	Number: 1990	181,585 (174,118–188,900)	17,401 (14,761–20,041)	49,761 (40,676–56,493)	2,143 (1,459–2,916)	38,014 (31,550–43,798)	15,304 (12,015–25,333)	3 (3–4)	1,224 (728–1,835)	1,589 (1,343–1,842)	4,769 (3,345–6,355)	2 (1–3)
Egypt	Number: 2015	67,639 (56,061–81,880)	8,517 (6,286–10,886)	10,543 (8,136–13,607)	803 (498–1,225)	4,175 (3,025–5,829)	16,376 (12,303–20,822)	3 (3–4)	1,475 (760–2,192)	390 (308–498)	2,584 (1,755–3,607)	25 (13–39)
Egypt	Number: Cumulative change (%)	-62.7 (-69.3 to -54.7)	-50.7 (-64.8 to -34.0)	-78.6 (-84.3 to -71.1)	-61.2 (-77.9 to -38.8)	-88.9 (-92.4 to -83.7)	13.3 (-44.4 to 55.7)	3.9 (-17.3 to 28.7)	26.0 (-28.2 to 102.4)	-75.1 (-81.5 to -65.9)	-43.9 (-65.3 to -12.3)	1,001.2 (765.5–1,284.9)
Egypt	Rate: 1990	559.3 (536.9–581.2)	51.1 (43.4–58.9)	150.3 (122.1–170.7)	6.3 (4.3–8.6)	114.0 (94.6–131.2)	46.0 (36.1–76.2)	0.0 (0.0–0.0)	3.6 (2.1–5.4)	5.0 (4.2–5.8)	14.0 (9.8–18.7)	0.0 (0.0–0.0)
Egypt	Rate: 2015	158.1 (132.7–189.7)	18.7 (13.8–23.9)	23.6 (18.3–30.4)	1.8 (1.1–2.7)	9.3 (6.7–12.9)	36.6 (27.6–46.3)	0.0 (0.0–0.0)	3.2 (1.7–4.8)	0.9 (0.7–1.2)	5.7 (3.8–7.9)	0.1 (0.0–0.1)
Egypt	Rate: Cumulative change (%)	-71.7 (-76.5 to -66.0)	-63.2 (-73.7 to -50.8)	-84.1 (-88.3 to -78.6)	-71.1 (-83.5 to -54.3)	-91.8 (-94.3 to -88.0)	-15.8 (-58.7 to 15.1)	-23.5 (-40.1 to -5.1)	-6.2 (-46.5 to 50.6)	-81.5 (-86.0 to -75.0)	-58.2 (-74.2 to -34.7)	755.4 (574.6–971.6)
Iran	Number: 1990	175,966 (159,228–194,184)	26,289 (20,449–32,907)	16,938 (11,696–22,230)	2,090 (1,106–3,430)	7,699 (4,880–11,424)	26,358 (10,399–38,407)	13 (7–22)	251 (69–709)	1,969 (1,105–2,985)	9,315 (5,146–14,399)	0 (0–1)
Iran	Number: 2015	27,962 (23,178–33,548)	4,958 (3,296–6,930)	1,403 (1,050–1,852)	915 (509–1,477)	270 (172–402)	6,404 (4,298–8,485)	2 (2–3)	415 (177–752)	231 (158–327)	2,026 (1,166–3,211)	1 (0–1)

© 2017 Global Burden of Disease Child and Adolescent Health Collaboration. JAMA Pediatrics.

eTable 1. Number of Deaths, Death Rates (per 100,000 population), and Cumulative Percent Change with 95% Uncertainty Intervals (UI) for the Top 10 Global Causes of Death in 195 Countries and Territories, Aged 0 to 19, Both Sexes, 1990 and 2015*Note: best viewed by enlarging in browser.*

Location	Measure	All causes	Neonatal preterm birth complications	Lower respiratory infections	Neonatal encephalopathy due to birth asphyxia and trauma	Diarrheal diseases	Congenital anomalies	Malaria	Neonatal sepsis and other neonatal infections	Meningitis	Other neonatal disorders	HIV/AIDS
Iran	Number: Cumulative change (%)	-84.1 (-87.2 to -80.5)	-80.8 (-87.8 to -72.1)	-91.4 (-94.5 to -87.1)	-51.2 (-79.1 to -7.7)	-96.2 (-98.1 to -93.7)	-72.6 (-85.3 to -46.0)	-76.3 (-87.9 to -59.6)	191.6 (-57.6 to 651.8)	-86.9 (-93.6 to -75.9)	-75.9 (-89.4 to -54.4)	48.8 (-29.3 to 149.7)
Iran	Rate: 1990	535.3 (483.9–590.9)	78.7 (61.3–98.6)	50.2 (34.9–65.9)	6.3 (3.3–10.3)	22.8 (14.5–33.7)	78.7 (31.1–114.5)	0.0 (0.0–0.1)	0.8 (0.2–2.1)	5.9 (3.3–8.9)	27.9 (15.4–43.1)	0.0 (0.0–0.0)
Iran	Rate: 2015	113.9 (94.6–136.3)	20.0 (13.3–28.0)	5.6 (4.2–7.4)	3.7 (2.1–6.0)	1.1 (0.7–1.6)	25.7 (17.2–34.0)	0.0 (0.0–0.0)	1.7 (0.7–3.0)	0.9 (0.6–1.3)	8.2 (4.7–13.0)	0.0 (0.0–0.0)
Iran	Rate: Cumulative change (%)	-78.6 (-82.9 to -74.0)	-74.2 (-83.6 to -62.5)	-88.4 (-92.6 to -82.6)	-34.3 (-71.9 to 24.5)	-94.9 (-97.4 to -91.5)	-63.2 (-80.3 to -27.7)	-68.5 (-83.6 to -46.1)	290.4 (-43.2 to 903.6)	-82.3 (-91.3 to -67.5)	-67.6 (-85.8 to -38.7)	108.1 (-0.3 to 251.0)
Iraq	Number: 1990	42,005 (40,523–43,512)	7,790 (6,165–9,379)	4,913 (3,663–6,265)	1,402 (805–2,217)	3,569 (2,593–4,770)	5,555 (3,360–9,559)	4 (3–5)	3,398 (1,473–5,473)	1,155 (668–1,986)	1,373 (641–2,437)	0 (0–0)
Iraq	Number: 2015	46,643 (41,572–52,284)	7,310 (5,285–9,276)	3,018 (2,295–3,817)	1,597 (939–2,476)	1,332 (879–1,860)	7,593 (5,224–12,131)	0 (0–0)	4,669 (2,125–7,215)	799 (457–1,330)	1,340 (617–2,397)	14 (8–23)
Iraq	Number: Cumulative change (%)	11.1 (-1.9 to 25.4)	-4.7 (-33.3 to 28.0)	-37.1 (-55.9 to -13.5)	24.7 (-46.4 to 122.7)	-61.3 (-76.4 to -41.2)	44.2 (-10.0 to 103.2)	-100.0 (-100.0 to -100.0)	45.9 (-21.0 to 140.3)	-24.9 (-62.5 to 33.9)	15.1 (-60.6 to 158.5)	inf (1,272.7–4,179.7)
Iraq	Rate: 1990	369.7 (356.6–382.8)	64.7 (51.2–78.0)	42.3 (31.5–53.8)	11.7 (6.7–18.5)	30.6 (22.2–40.7)	47.6 (28.9–81.8)	0.0 (0.0–0.1)	28.3 (12.3–45.4)	10.1 (5.9–17.3)	11.5 (5.4–20.3)	0.0 (0.0–0.0)
Iraq	Rate: 2015	226.0 (200.8–252.7)	32.2 (23.3–40.9)	13.9 (10.6–17.4)	7.0 (4.1–10.9)	6.0 (4.0–8.4)	34.4 (23.9–54.8)	0.0 (0.0–0.0)	20.6 (9.4–31.8)	3.8 (2.2–6.1)	5.9 (2.7–10.6)	0.1 (0.0–0.1)
Iraq	Rate: Cumulative change (%)	-38.8 (-46.0 to -30.8)	-49.5 (-64.6 to -32.1)	-66.5 (-76.3 to -54.3)	-33.9 (-71.6 to 17.8)	-79.5 (-87.4 to -69.1)	-23.7 (-52.4 to 7.3)	-100.0 (-100.0 to -100.0)	-22.7 (-58.2 to 27.3)	-59.9 (-79.4 to -29.1)	-39.2 (-79.2 to 36.6)	inf (650.5–2,215.4)
Jordan	Number: 1990	4,626 (4,500–4,755)	944 (768–1,120)	503 (397–623)	196 (121–302)	111 (73–161)	913 (739–1,140)	0 (0–0)	196 (107–324)	60 (36–102)	174 (93–280)	0 (0–0)
Jordan	Number: 2015	3,782 (3,468–4,159)	630 (492–785)	301 (248–363)	189 (126–262)	27 (19–36)	996 (854–1,156)	0 (0–0)	228 (135–359)	34 (24–51)	86 (46–141)	1 (0–2)
Jordan	Number: Cumulative change (%)	-18.2 (-25.1 to -10.0)	-32.5 (-48.9 to -12.3)	-38.9 (-55.9 to -18.2)	3.7 (-45.5 to 66.3)	-74.4 (-85.6 to -59.5)	11.2 (-19.1 to 42.0)	nan (nan–nan)	28.7 (-40.6 to 121.8)	-35.7 (-69.7 to 12.5)	-44.0 (-77.1 to 16.7)	inf (370.2–1,421.4)
Jordan	Rate: 1990	223.4 (217.3–229.5)	43.6 (35.5–51.7)	24.2 (19.1–29.8)	9.1 (5.6–14.0)	5.4 (3.5–7.7)	43.3 (34.9–54.0)	0.0 (0.0–0.0)	9.1 (5.0–15.0)	2.9 (1.8–5.0)	8.1 (4.3–13.0)	0.0 (0.0–0.0)
Jordan	Rate: 2015	105.8 (97.1–116.1)	17.2 (13.5–21.5)	8.3 (6.9–10.0)	5.2 (3.4–7.2)	0.7 (0.5–1.0)	27.4 (23.5–31.8)	0.0 (0.0–0.0)	6.2 (3.7–9.8)	1.0 (0.7–1.4)	2.4 (1.3–3.9)	0.0 (0.0–0.1)
Jordan	Rate: Cumulative change (%)	-52.6 (-56.6 to -47.9)	-60.0 (-69.7 to -48.0)	-64.9 (-74.5 to -53.2)	-38.8 (-67.8 to -1.5)	-85.3 (-91.7 to -76.9)	-35.4 (-52.9 to -17.4)	nan (nan–nan)	-24.0 (-65.0 to 30.8)	-63.3 (-82.5 to -36.2)	-67.0 (-86.5 to -31.2)	inf (173.4–775.4)
Kuwait	Number: 1990	1,038 (912–1,175)	89 (72–108)	73 (61–84)	64 (51–79)	13 (10–15)	244 (206–277)	0 (0–0)	15 (7–23)	7 (6–9)	20 (14–26)	2 (2–3)
Kuwait	Number: 2015	757 (644–885)	151 (118–186)	44 (36–54)	16 (12–21)	4 (3–5)	289 (231–354)	0 (0–0)	8 (5–14)	5 (4–6)	9 (6–11)	1 (1–2)
Kuwait	Number: Cumulative change (%)	-26.6 (-40.4 to -10.1)	71.9 (26.5–122.0)	-38.3 (-52.8 to -18.2)	-73.4 (-81.5 to -63.2)	-66.1 (-75.6 to -53.7)	19.5 (-7.5 to 52.4)	nan (nan–nan)	-37.4 (-70.8 to 20.1)	-30.9 (-47.8 to -9.8)	-52.6 (-69.4 to -30.6)	-38.1 (-50.8 to -22.9)
Kuwait	Rate: 1990	124.0 (109.8–139.2)	11.8 (9.5–14.4)	8.8 (7.4–10.3)	8.5 (6.8–10.5)	1.6 (1.3–2.0)	30.7 (26.0–35.0)	0.0 (0.0–0.0)	2.0 (0.9–3.0)	0.9 (0.7–1.1)	2.6 (1.9–3.5)	0.3 (0.3–0.4)
Kuwait	Rate: 2015	59.2 (51.0–68.5)	11.0 (8.6–13.6)	3.4 (2.8–4.2)	1.2 (0.9–1.6)	0.3 (0.2–0.4)	21.4 (17.1–26.2)	0.0 (0.0–0.0)	0.6 (0.4–1.1)	0.4 (0.3–0.5)	0.7 (0.5–0.9)	0.1 (0.1–0.2)
Kuwait	Rate: Cumulative change (%)	-52.0 (-60.7 to -41.6)	-5.4 (-30.4 to 22.2)	-60.5 (-69.5 to -47.9)	-85.3 (-89.8 to -79.7)	-79.6 (-85.3 to -72.1)	-29.6 (-45.5 to -10.4)	nan (nan–nan)	-65.5 (-83.9 to -33.7)	-54.9 (-66.0 to -41.2)	-73.8 (-83.1 to -61.7)	-58.7 (-67.0 to -48.9)
Lebanon	Number: 1990	3,327 (3,036–3,659)	635 (532–752)	252 (183–328)	125 (73–190)	99 (58–153)	604 (472–745)	0 (0–0)	40 (17–77)	39 (23–62)	90 (44–154)	3 (0–17)
Lebanon	Number: 2015	1,079 (900–1,305)	149 (100–207)	32 (23–44)	42 (22–68)	7 (4–12)	316 (220–425)	0 (0–0)	32 (16–56)	8 (5–13)	31 (13–57)	4 (0–18)
Lebanon	Number: Cumulative change (%)	-67.4 (-73.5 to -59.9)	-76.1 (-84.9 to -65.3)	-86.7 (-91.7 to -80.0)	-63.9 (-83.0 to -33.8)	-91.3 (-95.8 to -84.1)	-46.3 (-67.9 to -20.6)	nan (nan–nan)	0.0 (-60.7 to 123.9)	-77.3 (-89.2 to -59.5)	-59.9 (-86.2 to -10.7)	120.8 (-27.6 to 338.9)
Lebanon	Rate: 1990	268.8 (245.6–295.1)	51.1 (42.8–60.5)	20.0 (14.6–26.0)	10.1 (5.9–15.4)	7.9 (4.6–12.2)	48.4 (37.9–59.7)	0.0 (0.0–0.0)	3.2 (1.4–6.3)	3.2 (1.9–5.0)	7.3 (3.6–12.4)	0.3 (0.0–1.4)
Lebanon	Rate: 2015	62.9 (52.3–76.9)	9.5 (6.4–13.2)	2.0 (1.4–2.8)	2.7 (1.5–4.3)	0.5 (0.3–0.8)	20.0 (13.8–27.0)	0.0 (0.0–0.0)	2.1 (1.0–3.6)	0.5 (0.3–0.9)	2.0 (0.9–3.7)	0.2 (0.0–0.9)
Lebanon	Rate: Cumulative change (%)	-76.5 (-80.9 to -70.9)	-81.1 (-88.1 to -72.5)	-89.4 (-93.5 to -83.7)	-71.3 (-86.5 to -47.5)	-93.1 (-96.7 to -87.3)	-57.6 (-74.7 to -37.0)	nan (nan–nan)	-20.9 (-69.0 to 76.7)	-82.8 (-92.0 to -68.4)	-68.1 (-89.0 to -29.1)	51.9 (-55.4 to 210.7)
Libya	Number: 1990	6,385 (5,952–6,849)	1,234 (1,008–1,481)	601 (456–768)	211 (117–340)	277 (172–411)	1,163 (893–1,558)	0 (0–0)	72 (32–137)	88 (49–141)	190 (93–335)	4 (0–13)
Libya	Number: 2015	3,512 (2,898–4,178)	517 (354–712)	135 (102–173)	100 (57–156)	35 (22–53)	700 (547–881)	0 (0–0)	71 (33–129)	24 (14–36)	83 (38–147)	9 (0–26)
Libya	Number: Cumulative change (%)	-44.9 (-55.3 to -33.4)	-57.7 (-71.6 to -40.5)	-76.9 (-84.6 to -66.7)	-48.6 (-75.6 to -7.7)	-86.2 (-92.9 to -76.8)	-37.9 (-59.1 to -12.3)	nan (nan–nan)	19.1 (-56.7 to 169.8)	-70.1 (-86.9 to -48.1)	-49.7 (-82.7 to 3.7)	inf (-16.8 to 4,906.1)
Libya	Rate: 1990	274.0 (255.4–293.7)	53.6 (43.8–64.3)	25.6 (19.4–32.8)	9.2 (5.1–14.8)	11.9 (7.4–17.6)	50.0 (38.4–66.9)	0.0 (0.0–0.0)	3.1 (1.4–6.0)	3.7 (2.1–6.1)	8.3 (4.1–14.5)	0.2 (0.0–0.6)
Libya	Rate: 2015	147.3 (121.4–175.0)	21.7 (14.9–29.9)	5.5 (4.2–7.1)	4.2 (2.4–6.6)	1.5 (0.9–2.2)	28.9 (22.5–36.4)	0.0 (0.0–0.0)	3.0 (1.4–5.4)	1.0 (0.6–1.5)	3.5 (1.6–6.2)	0.4 (0.0–1.1)
Libya	Rate: Cumulative change (%)	-46.1 (-56.3 to -35.0)	-59.1 (-72.5 to -42.4)	-77.8 (-85.1 to -68.1)	-50.3 (-76.5 to -10.9)	-86.8 (-93.2 to -77.7)	-40.3 (-60.7 to -15.6)	nan (nan–nan)	15.1 (-58.2 to 161.8)	-71.4 (-87.2 to -51.1)	-51.4 (-83.3 to 0.2)	inf (-17.2 to 4,701.5)
Morocco	Number: 1990	60,831 (58,893–62,779)	9,450 (7,498–11,558)	7,094 (5,759–8,535)	3,292 (2,162–4,696)	8,593 (6,740–10,443)	4,260 (3,031–8,118)	1 (0–1)	3,089 (1,730–4,832)	2,198 (1,016–3,531)	1,652 (817–2,937)	1 (1–3)
Morocco	Number: 2015	20,186 (17,387–23,543)	4,263 (3,059–5,498)	1,182 (913–1,521)	1,717 (1,150–2,416)	335 (233–475)	2,822 (1,816–4,809)	0 (0–1)	1,596 (978–2,385)	373 (262–529)	364 (182–628)	17 (3–35)
Morocco	Number: Cumulative change (%)	-66.8 (-71.5 to -61.0)	-54.0 (-68.8 to -32.7)	-83.1 (-87.9 to -77.1)	-44.9 (-69.1 to -9.5)	-96.0 (-97.4 to -94.1)	-31.9 (-54.8 to -1.8)	-12.8 (-42.5 to 18.6)	-43.9 (-72.3 to -5.9)	-80.8 (-90.1 to -68.0)	-74.2 (-90.8 to -44.9)	721.2 (218.1–1,097.8)
Morocco	Rate: 1990	461.9 (447.4–476.8)	70.9 (56.3–86.8)	53.6 (43.5–64.5)	24.7 (16.2–35.2)	64.9 (50.9–78.8)	32.2 (22.9–61.3)	0.0 (0.0–0.0)	23.2 (13.0–36.3)	16.6 (7.7–26.7)	12.4 (6.1–22.1)	0.0 (0.0–0.0)
Morocco	Rate: 2015	158.9 (137.1–185.0)	33.2 (23.9–42.9)	9.3 (7.2–11.8)	13.4 (9.0–18.9)	2.6 (1.8–3.7)	22.0 (14.2–37.5)	0.0 (0.0–0.0)	12.4 (7.6–18.6)	2.9 (2.1–4.2)	2.8 (1.4–4.9)	0.1 (0.0–0.3)
Morocco	Rate: Cumulative change (%)	-65.6 (-70.4 to -59.7)	-52.2 (-67.5 to -30.1)	-82.5 (-87.5 to -76.4)	-42.8 (-67.9 to -5.9)	-95.9 (-97.3 to -93.9)	-29.6 (-53.1 to 1.6)	-11.5 (-43.1 to 21.3)	-41.8 (-71.3 to -2.1)	-80.0 (-89.7 to -66.7)	-73.2 (-90.5 to -42.8)	768.4 (232.3–1,166.8)
Palestine	Number: 1990	4,321 (4,002–4,669)	1,366 (1,141–1,599)	388 (285–506)	236 (140–357)	71 (46–108)	687 (469–1,092)	0 (0–0)	251 (45–479)	53 (30–92)	77 (27–179)	0 (0–0)
Palestine	Number: 2015	3,384 (2,870–3,970)	893 (668–1,134)	186 (140–231)	202 (136–289)	19 (14–26)	654 (464–1,009)	0 (0–0)	223 (44–373)	21 (14–29)	100 (56–163)	1 (1–3)
Palestine	Number: Cumulative change (%)	-21.5 (-35.5 to -6.6)	-33.9 (-53.0 to -12.8)	-50.7 (-65.4 to -32.7)	-7.1 (-53.2 to 61.4)	-70.9 (-83.0 to -55.5)	-1.8 (-35.4 to 38.6)	nan (nan–nan)	-1.3 (-49.2 to 67.8)	-54.6 (-78.8 to -23.6)	77.1 (-53.8 to 284.0)	inf (885.3–2,878.1)
Palestine	Rate: 1990	268.8 (249.7–289.5)	77.2 (64.4–90.4)	25.0 (18.7–32.2)	13.5 (8.0–20.4)	4.3 (2.8–6.4)	40.7 (28.0–64.6)	0.0 (0.0–0.0)	14.3 (2.5–27.2)	3.3 (1.9–5.5)	4.5 (1.6–10.3)	0.0 (0.0–0.0)
Palestine	Rate: 2015	128.7 (109.7–150.3)	32.2 (24.1–40.9)	7.4 (5.6–9.2)	7.3 (4.9–10.5)	0.7 (0.5–1.0)	24.1 (17.2–37.0)	0.0 (0.0–0.0)	8.1 (1.6–13.5)	0.8 (0.6–1.1)	3.6 (2.0–5.9)	0.1 (0.0–0.1)
Palestine	Rate: Cumulative change (%)	-52.0 (-60.2 to -43.3)	-57.8 (-70.1 to -44.2)	-69.7 (-78.1 to -59.6)	-41.2 (-70.3 to 2.0)	-81.8 (-89.3 to -72.6)	-39.0 (-59.5 to -14.0)	nan (nan–nan)	-37.2 (-67.7 to 6.3)	-72.2 (-86.6 to -54.3)	12.1 (-70.9 to 144.3)	inf (489.5–1,702.1)
Oman	Number: 1990	3,388 (2,968–3,848)	563 (394–752)	333 (219–476)	92 (47–156)	58 (32–94)	402 (264–681)	0 (0–0)	8 (2–19)	33 (16–62)	458 (271–686)	0 (0–0)
Oman	Number: 2015	995 (906–1,087)	135 (100–171)	47 (38–58)	37 (24–54)	4 (2–5)	224 (181–281)	0 (0–0)	9 (2–19)	6 (3–9)	99 (64–137)	1 (1–1)
Oman	Number: Cumulative change (%)	-70.4 (-75.1 to -65.7)	-75.0 (-84.3 to -63.4)	-84.8 (-90.7 to -76.3)	-53.7 (-79.2 to -16.5)	-91.9 (-96.2 to -85.4)	-38.9 (-71.2 to -10.6)	10.7 (-23.0 to 44.9)	34.8 (-64.0 to 248.1)	-78.0 (-91.7 to -56.3)	-76.3 (-87.7 to -57.9)	581.2 (352.3–810.0)
Oman	Rate: 1990	281.1 (248.3–316.9)	44.1 (30.9–58.9)	26.8 (18.0–37.8)	7.3 (3.7–12.3)	4.6 (2.6–7.5)	31.8 (20.9–54.0)	0.0 (0.0–0.0)	0.7 (0.2–1.6)	2.7 (1.4–4.9)	35.9 (21.2–53.7)	0.0 (0.0–0.0)
Oman	Rate: 2015	74.0 (67.6–80.8)	9.1 (6.7–11.4)	3.5 (2.9–4.3)	2.5 (1.6–3.6)	0.3 (0.2–0.4)	15.4 (12.4–19.2)	0.0 (0.0–0.0)	0.6 (0.2–1.3)	0.4 (0.3–0.7)	6.7 (4.3–9.2)	0.1 (0.1–0.2)
Oman	Rate: Cumulative change (%)	-73.5 (-77.5 to -69.5)	-78.6 (-86.6 to -68.8)	-86.0 (-91.3 to -78.5)	-60.5 (-82.2 to -28.5)	-92.8 (-96.5 to -87.1)	-47.2 (-75.0 to -23.0)	-10.0 (-38.9 to 19.7)	14.9 (-69.3 to 196.7)	-80.5 (-92.4 to -61.7)	-79.8 (-89.5 to -64.1)	659.8 (402.1–909.0)
Qatar	Number: 1990	283 (232–342)	83 (62–108)	8 (5–11)	7 (4–12)	1 (0–2)	68 (48–91)	0 (0–0)	1 (0–3)	2 (1–3)	7 (3–13)	0 (0–0)
Qatar	Number: 2015	302 (240–379)	62 (43–88)	6 (4–8)	10 (5–15)	1 (0–1)	93 (67–126)	0 (0–0)	1 (0–2)	2 (1–3)	11 (6–18)	0 (0–0)
Qatar	Number: Cumulative change (%)	7.9 (-20.7 to 42.4)	-23.1 (-51.5 to 13.2)	-16.3 (-50.1 to 27.6)	41.0 (-36.1 to 163.5)	-22.5 (-65.2 to 42.8)	41.2 (-10.2 to 112.2)	nan (nan–nan)	10.9 (-69.4 to 139.6)	8.0 (-51.6 to 108.1)	76.7 (-34.1 to 259.2)	inf (81.8–326.1)
Qatar	Rate: 1990	154.8 (128.4–185.4)	43.5 (32.5–56.3)	4.3 (3.0–6.1)	4.1 (2.3–6.5)	0.8 (0.4–1.3)	35.8 (25.4–47.7)	0.0 (0.0–0.0)	0.9 (0.2–1.9)	1.2 (0.6–1.9)	4.1 (1.9–7.1)	0.0 (0.0–0.1)
Qatar	Rate: 2015	64.9 (51.7–81.1)	13.1 (9.1–18.3)	1.4 (1.1–1.9)	2.1 (1.2–3.3)	0.2 (0.1–0.3)	19.8 (14.3–26.7)	0.0 (0.0–0.0)	0.3 (0.1–0.5)	0.5 (0.3–0.7)	2.5 (1.4–3.9)	0.0 (0.0–0.1)
Qatar	Rate: Cumulative change (%)	-57.6 (-68.5 to -44.5)	-69.2 (-80.6 to -54.6)	-65.8 (-79.3 to -48.5)	-43.0 (-74.2 to 6.7)	-68.3 (-85.5 to -41.9)	-42.9 (-63.4 to -14.2)	nan (nan–nan)	-55.6 (-87.8 to -3.9)	-56.0 (-80.0 to -16.2)	-28.8 (-73.6 to 44.6)	inf (-22.5 to 82.6)
Saudi Arabia	Number: 1990	30,601 (28,374–33,236)	8,612 (7,353–9,687)	1,233 (882–1,657)	1,304 (968–1,679)	1,310 (833–1,909)	5,874 (4,568–8,542)	1 (0–2)	2,186 (1,610–2,919)	223 (111–359)	1,114 (716–1,675)	11 (2–34)
Saudi Arabia	Number: 2015	9,549 (8,831–10,357)	1,777 (1,299–2,121)	246 (211–304)	390 (295–479)	118 (97–142)	2,860 (2,435–3,531)	1 (0–1)	842 (659–1,137)	37 (26–47)	262 (167–357)	32 (16–63)
Saudi Arabia	Number: Cumulative change (%)	-68.7 (-71.9 to -65.6)	-79.3 (-84.9 to -74.5)	-79.1 (-86.3 to -70.2)	-69.4 (-79.0 to -59.3)	-90.3 (-94.0 to -85.2)	-49.5 (-67.4 to -31.4)	-26.3 (-58.3 to 8.1)	-60.7 (-71.3 to -48.1)	-81.5 (-89.1 to -71.3)	-74.5 (-88.5 to -59.2)	471.2 (27.9–1,034.1)
Saudi Arabia	Rate: 1990	306.4 (283.6–337.4)	81.7 (69.8–91.9)	12.5 (9.1–16.5)	12.4 (9.2–16.0)	12.7 (8.1–18.5)	57.0 (44.3–82.8)	0.0 (0.0–0.0)	20.8 (15.3–27.7)	2.2 (1.1–3.6)	10.6 (6.8–16.0)	0.1 (0.0–0.3)
Saudi Arabia	Rate: 2015	83.7 (77.4–90.8)	15.6 (11.4–18.6)	2.1 (1.8–2.6)	3.4 (2.6–4.2)	1.0 (0.8–1.2)	24.9 (21.2–30.8)	0.0 (0.0–0.0)	7.4 (5.8–10.0)	0.3 (0.2–0.4)	2.3 (1.5–3.1)	0.3 (0.1–0.6)
Saudi Arabia	Rate: Cumulative change (%)	-72.6 (-75.5 to -69.9)	-80.8 (-86.0 to -76.4)	-82.2 (-88.0 to -75.1)	-71.8 (-80.7 to -62.5)	-91.4 (-94.7 to -86.8)	-54.7 (-70.6 to -38.4)	-46.6 (-67.7 to -23.6)	-63.7 (-73.6 to -52.1)	-84.1 (-90.5 to -75.8)	-76.5 (-89.4 to -62.4)	385.1 (9.0–875.3)
Sudan	Number: 1990	124,735 (118,168–131,547)	18,827 (15,025–22,783)	20,169 (15,366–25,753)	2,610 (1,444–4,174)	18,602 (13,327–24,661)	9,649 (4,762–21,483)	2,482 (1,134–4,426)	762 (308–1,507)	2,574 (1,398–4,099)	2,687 (1,153–4,861)	41 (11–79)
Sudan	Number: 2015	86,737 (73,033–104,361)	20,156 (15,890–24,814)	9,375 (6,573–13,100)	2,803 (1,584–4,421)	7,905 (4,854–12,032)	11,572 (7,291–19,025)	1,851 (852–3,288)	1,500 (718–2,840)	1,673 (865–2,647)	2,576 (1,192–4,804)	313 (243–396)
Sudan	Number: Cumulative change (%)	-30.4 (-42.1 to -16.0)	8.1 (-14.7 to 34.2)	-52.5 (-68.2 to -30.2)	16.1 (-42.7 to 106.0)	-56.6 (-73.3 to -32.4)	38.0 (-19.7 to 101.6)	-20.0 (-60.0 to 36.1)	144.1 (-10.1 to 470.6)	-28.3 (-69.5 to 36.3)	13.4 (-61.8 to 134.7)	961.9 (295.1–2,444.7)
Sudan	Rate: 1990	915.6 (866.2–965.5)	123.0 (98.0–148.9)	146.8 (111.8–187.4)	17.1 (9.4–27.4)	133.3 (95.3–176.1)	66.8 (32.8–149.4)	18.7 (8.8–33.0)	5.0 (2.0–9.9)	19.0 (10.3–30.2)	17.7 (7.6–31.8)	0.3 (0.1–0.6)
Sudan	Rate: 2015	378.1 (319.1–453.1)	84.1 (66.3–103.5)	40.5 (28.5–56.3)	11.7 (6.6–18.5)	33.8 (20.8–51.5)	49.3 (31.1–80.8)	8.2 (3.9–14.4)	6.3 (3.0–11.9)	7.3 (3.8–11.4)	10.8 (5.0–20.1)	1.5 (1.1–1.8)
Sudan	Rate: Cumulative change (%)	-58.7 (-65.5 to -50.3)	-30.9 (-45.5 to -14.2)	-71.8 (-81.2 to -58.6)	-25.9 (-63.3 to 31.6)	-74.1 (-84.1 to -59.5)	-14.6 (-50.8 to 25.5)	-53.2 (-76.2 to -20.6)	55.1 (-42.7 to 261.2)	-57.8 (-82.0 to -20.1)	-28.0 (-75.6 to 48.9)	542.0 (146.9–1,396.4)
Syria	Number: 1990	22,558 (21,177–24,092)	2,887 (2,233–3,543)	3,049 (1,944–3,922)	1,645 (1,053–2,327)	1,620 (994–2,252)	2,663 (1,828–3,486)	0 (0–1)	372 (167–694)	686 (394–1,061)	1,011 (480–1,679)	2 (1–3)
Syria	Number: 2015	23,934 (15,564–32,397)	714 (494–962)	992 (800–1,193)	635 (368–972)	55 (37–79)	1,835 (1,426–2,170)	1 (0–1)	297 (161–489)	213 (152–301)	355 (157–603)	3 (1–5)
Syria	Number: Cumulative change (%)	6.3 (-31.3 to 44.4)	-74.8 (-83.2 to -64.5)	-65.6 (-76.4 to -46.3)	-59.8 (-78.3 to -34.6)	-96.3 (-98.0 to -93.7)	-28.3 (-51.7 to 0.5)	40.2 (-1.4 to 93.5)	-4.9 (-58.1 to 96.3)	-66.0 (-81.6 to -40.6)	-61.2 (-84.5 to -26.3)	19.6 (-39.6 to 79.3)
Syria	Rate: 1990	286.6 (269.4–305.5)	35.5 (27.5–43.6)	38.2 (24.4–49.0)	20.2 (13.0–28.6)	20.0 (12.3–27.8)	33.0 (22.8–43.2)	0.0 (0.0–0.0)	4.6 (2.1–8.6)	8.6 (5.0–13.3)	12.5 (5.9–20.7)	0.0 (0.0–0.0)
Syria	Rate: 2015	270.8 (177.8–365.3)	8.9 (6.2–12.0)	11.4 (9.1–13.7)	7.9 (4.6–12.1)	0.7 (0.4–0.9)	22.2 (17.1–26.3)	0.0 (0.0–0.0)	3.7 (2.0–6.1)	2.4 (1.7–3.5)	4.4 (1.9–7.5)	0.0 (0.0–0.1)
Syria	Rate: Cumulative change (%)	-5.4 (-38.5 to 28.0)	-74.6 (-83.0 to -64.1)	-68.6 (-78.4 to -50.9)	-59.4 (-78.1 to -33.9)	-96.5 (-98.1 to -94.1)	-30.2 (-52.8 to -3.0)	3.9 (-28.2 to 43.3)	-4.5 (-57.9 to 97.1)	-69.2 (-83.2 to -46.4)	-61.1 (-84.4 to -25.8)	3.2 (-49.6 to 55.4)
Tunisia	Number: 1990	12,894 (12,369–13,449)	2,692 (2,174–3,214)	1,120 (840–1,427)	711 (423–1,085)	849 (559–1,201)	1,491 (1,000–2,458)	0 (0–0)	570 (303–942)	300 (155–509)	591 (285–983)	0 (0–0)
Tunisia	Number: 2015	3,701 (3,294–4,182)	752 (571–961)	168 (137–200)	253 (171–356)	44 (31–59)	755 (600–984)	0 (0–0)	249 (154–366)	45 (29–67)	116 (63–192)	2 (1–4)
Tunisia	Number: Cumulative change (%)	-71.3 (-74.6 to -67.3)	-71.7 (-79.7 to -61.0)	-84.6 (-88.8 to -78.8)	-61.5 (-79.2 to -36.6)	-94.5 (-96.9 to -91.3)	-45.8 (-69.8 to -23.7)	nan (nan–nan)	-52.0 (-77.4 to -19.0)	-83.0 (-92.3 to -68.0)	-77.2 (-91.2 to -53.2)	714.7 (318.7–1,067.0)
Tunisia	Rate: 1990	327.5 (314.2–341.4)	69.0 (55.7–82.3)	28.2 (21.1–35.9)	18.2 (10.8–27.7)	21.4 (14.1–30.4)	37.9 (25.4–62.4)	0.0 (0.0–0.0)	14.6 (7.8–24.1)	7.5 (3.9–12.8)	15.1 (7.3–25.1)	0.0 (0.0–0.0)
Tunisia	Rate: 2015	102.1 (91.1–115.2)	20.3 (15.4–26.0)	4.7 (3.8–5.6)	6.8 (4.6–9.6)	1.2 (0.9–1.6)	20.5 (16.3–26.7)	0.0 (0.0–0.0)	6.7 (4.1–9.9)	1.2 (0.8–1.8)	3.1 (1.7–5.2)	0.1 (0.0–0.1)
Tunisia	Rate: Cumulative change (%)	-68.8 (-72.5 to -64.6)	-70.2 (-78.6 to -59.0)	-82.9 (-87.6 to -76.6)	-59.4 (-78.0 to -33.0)	-94.0 (-96.6 to -90.6)	-42.0 (-67.7 to -18.4)	nan (nan–nan)	-49.5 (-76.3 to -14.7)	-81.5 (-91.7 to -65.7)	-76.0 (-90.7 to -50.9)	811.3 (367.2–1,206.9)
Turkey	Number: 1990	117,730 (111,390–124,300)	21,086 (16,854–25,482)	21,290 (16,823–26,001)	4,311 (2,635–6,309)	13,542 (9,704–17,919)	9,590 (3,767–15,189)	15 (12–18)	613 (242–1,146)	4,936 (1,597–8,767)	5,509 (2,644–9,388)	0 (0–0)
Turkey	Number: 2015	28,872 (23,996–35,096)	5,443 (3,595–7,806)	1,273 (970–1,677)	1,597 (959–2,444)	277 (180–423)	6,286 (3,329–9,025)	2 (1–2)	922 (188–1,693)	243 (149–358)	2,121 (1,211–3,255)	15 (8–22)
Turkey	Number: Cumulative change (%)	-75.4 (-79.8 to -70.0)	-74.0 (-83.2 to -63.4)	-93.9 (-95.8 to -91.3)	-60.2 (-80.9 to -31.4)	-97.9 (-98.8 to -96.5)	-29.3 (-66.7 to 6.0)	-86.0 (-89.6 to -82.3)	61.9 (-38.8 to 236.6)	-93.0 (-97.8 to -81.3)	-56.1 (-81.3 to -16.3)	inf (2,085.6–inf)
Turkey	Rate: 1990	462.6 (437.7–488.5)	82.6 (66.0–99.8)	83.7 (66.1–102.3)	16.9 (10.3–24.7)	53.3 (38.1–70.6)	37.7 (14.8–59.8)	0.1 (0.0–0.1)	2.4 (1.0–4.5)	19.4 (6.3–34.4)	21.6 (10.4–36.8)	0.0 (0.0–0.0)
Turkey	Rate: 2015	117.1 (96.5–143.2)	23.0 (15.2–32.9)	5.2 (3.9–6.9)	6.7 (4.1–10.3)	1.1 (0.7–1.8)	26.2 (13.8–37.7)	0.0 (0.0–0.0)	3.9 (0.8–7.1)	1.0 (0.6–1.5)	9.0 (5.1–13.7)	0.1 (0.0–0.1)
Turkey	Rate: Cumulative change (%)	-74.7 (-79.3 to -68.9)	-71.9 (-81.8 to -60.6)	-93.7 (-95.7 to -91.0)	-57.2 (-79.4 to -26.0)	-97.8 (-98.8 to -96.3)	-25.0 (-64.8 to 12.7)	-86.5 (-90.0 to -82.9)	74.0 (-34.2 to 261.6)	-92.8 (-97.7 to -80.5)	-52.8 (-79.8 to -9.7)	inf (2,143.5–inf)
United Arab Emirates	Number: 1990	1,398 (987–1,922)	314 (179–478)	43 (21–74)	64 (30–112)	19 (8–35)	263 (128–441)	0 (0–0)	31 (12–63)	21 (8–40)	68 (26–130)	1 (0–2)
United Arab Emirates	Number: 2015	892 (667–1,194)	100 (54–166)	17 (11–25)	32 (14–57)	2 (1–4)	222 (135–343)	0 (0–0)	34 (15–63)	10 (5–16)	29 (12–54)	4 (0–15)
United Arab Emirates	Number: Cumulative change (%)	-33.5 (-57.2 to 0.6)	-65.3 (-84.1 to -33.7)	-53.2 (-79.2 to -11.4)	-40.2 (-82.3 to 36.8)	-82.9 (-94.0 to -64.1)	-3.3 (-57.8 to 89.3)	132.8 (41.2–277.3)	38.2 (-59.1 to 208.5)	-40.0 (-79.6 to 31.3)	-45.4 (-85.9 to 34.8)	inf (92.0–9,348.0)
United Arab Emirates	Rate: 1990	177.7 (128.1–239.6)	36.8 (21.1–56.1)	5.4 (2.8–9.1)	7.6 (3.6–13.2)	2.4 (1.0–4.2)	31.4 (15.4–52.6)	0.0 (0.0–0.0)	3.7 (1.4–7.5)	2.7 (1.2–4.9)	8.0 (3.1–15.3)	0.1 (0.0–0.4)
United Arab Emirates	Rate: 2015	50.3 (37.7–66.9)	5.6 (3.0–9.2)	1.0 (0.7–1.4)	1.8 (0.8–3.2)	0.2 (0.1–0.3)	12.3 (7.5–19.0)	0.0 (0.0–0.0)	1.9 (0.8–3.5)	0.6 (0.3–0.9)	1.6 (0.7–3.0)	0.3 (0.0–0.9)
United Arab Emirates	Rate: Cumulative change (%)	-70.7 (-80.8 to -56.6)	-83.7 (-92.5 to -68.8)	-78.9 (-90.3 to -61.3)	-71.9 (-91.7 to -35.7)	-92.1 (-97.2 to -83.5)	-55.1 (-80.3 to -11.9)	-13.8 (-47.8 to 36.4)	-35.0 (-80.8 to 45.0)	-73.5 (-90.5 to -43.1)	-74.3 (-93.4 to -36.8)	inf (-13.8 to 4,183.9)
Yemen	Number: 1990	87,235 (84,573–89,919)	14,873 (11,479–17,805)	14,991 (8,878–20,244)	1,999 (1,039–3,338)	14,642 (8,567–20,300)	7,321 (3,364–17,977)	58 (13–140)	510 (208–1,053)	1,397 (567–2,461)	2,155 (823–4,087)	8 (0–35)

© 2017 Global Burden of Disease Child and Adolescent Health Collaboration. JAMA Pediatrics.

eTable 1. Number of Deaths, Death Rates (per 100,000 population), and Cumulative Percent Change with 95% Uncertainty Intervals (UI) for the Top 10 Global Causes of Death in 195 Countries and Territories, Aged 0 to 19, Both Sexes, 1990 and 2015*Note: best viewed by enlarging in browser.*

Location	Measure	All causes	Neonatal preterm birth complications	Lower respiratory infections	Neonatal encephalopathy due to birth asphyxia and trauma	Diarrheal diseases	Congenital anomalies	Malaria	Neonatal sepsis and other neonatal infections	Meningitis	Other neonatal disorders	HIV/AIDS
Yemen	Number: 2015	55,018 (50,033–60,780)	11,266 (8,669–13,782)	4,461 (3,362–5,772)	1,538 (861–2,426)	3,098 (1,846–4,693)	6,538 (4,360–10,058)	30 (9–66)	727 (324–1,334)	583 (300–898)	1,538 (623–2,868)	13 (1–54)
Yemen	Number: Cumulative change (%)	-36.9 (-42.9 to -30.7)	-23.8 (-38.2 to -7.1)	-68.1 (-80.3 to -49.3)	-15.9 (-59.6 to 50.1)	-77.1 (-88.6 to -60.3)	8.0 (-44.2 to 68.9)	-29.4 (-82.6 to 69.7)	76.2 (-37.1 to 307.0)	-50.0 (-81.3 to 2.4)	-14.2 (-72.3 to 85.2)	345.8 (-58.5 to 1,462.1)
Yemen	Rate: 1990	865.7 (835.3–893.7)	130.3 (100.6–156.2)	145.5 (84.4–196.4)	17.6 (9.2–29.2)	139.7 (81.0–193.0)	68.5 (31.0–168.8)	0.6 (0.2–1.4)	4.5 (1.8–9.2)	13.9 (5.7–24.1)	19.0 (7.3–36.1)	0.1 (0.0–0.4)
Yemen	Rate: 2015	366.6 (332.9–404.5)	72.3 (55.6–88.4)	29.4 (22.3–37.9)	9.9 (5.5–15.6)	20.2 (12.0–30.5)	42.6 (28.5–65.4)	0.2 (0.1–0.4)	4.7 (2.1–8.6)	3.9 (2.0–6.0)	9.9 (4.0–18.4)	0.1 (0.0–0.4)
Yemen	Rate: Cumulative change (%)	-57.6 (-61.8 to -53.3)	-44.2 (-54.8 to -32.0)	-78.3 (-86.5 to -65.4)	-38.5 (-70.5 to 9.2)	-84.3 (-92.3 to -73.0)	-24.3 (-61.3 to 19.1)	-54.8 (-87.9 to 7.3)	28.4 (-54.1 to 195.7)	-66.8 (-87.4 to -33.4)	-37.6 (-79.9 to 34.9)	180.9 (-72.1 to 876.1)
South Asia	Number: 1990	4,939,233 (4,898,664–4,979,693)	831,361 (757,132–909,394)	741,686 (670,241–801,207)	497,476 (427,049–565,767)	586,134 (523,495–635,184)	136,714 (95,509–172,176)	56,232 (47,055–66,284)	79,611 (48,880–116,828)	122,461 (103,610–142,281)	111,976 (53,420–170,094)	537 (467–617)
South Asia	Number: 2015	2,205,667 (2,147,004–2,268,067)	379,162 (339,652–428,597)	235,756 (214,894–257,976)	413,928 (376,848–455,579)	175,213 (155,858–196,689)	111,162 (84,287–129,827)	21,434 (16,548–27,403)	61,781 (37,086–84,394)	55,233 (46,485–66,357)	71,394 (50,245–93,100)	15,984 (14,800–17,272)
South Asia	Number: Cumulative change (%)	-55.3 (-56.6 to -54.1)	-54.3 (-59.5 to -48.8)	-68.1 (-71.6 to -64.5)	-16.3 (-27.3 to -3.2)	-70.0 (-74.0 to -65.2)	-16.5 (-50.0 to 3.0)	-61.5 (-71.9 to -48.2)	-19.3 (-50.6 to 19.5)	-54.5 (-62.9 to -42.1)	-30.3 (-59.3 to 14.0)	2,890.7 (2,484.7–3,334.1)
South Asia	Rate: 1990	808.2 (801.5–815.0)	127.9 (116.5–140.0)	120.5 (108.8–130.3)	76.5 (65.7–87.0)	97.0 (86.6–105.1)	21.8 (15.2–27.5)	9.7 (8.1–11.4)	12.3 (7.6–18.1)	20.5 (17.4–23.8)	17.4 (8.3–26.5)	0.1 (0.1–0.1)
South Asia	Rate: 2015	343.6 (334.4–353.5)	59.8 (53.5–67.5)	37.0 (33.7–40.5)	65.2 (59.4–71.8)	27.4 (24.4–30.8)	17.4 (13.2–20.4)	3.3 (2.5–4.2)	9.8 (5.9–13.3)	8.6 (7.2–10.4)	11.3 (7.9–14.7)	2.4 (2.3–2.6)
South Asia	Rate: Cumulative change (%)	-57.5 (-58.7 to -56.3)	-53.2 (-58.5 to -47.5)	-69.2 (-72.6 to -65.7)	-14.2 (-25.5 to -0.9)	-71.6 (-75.5 to -67.1)	-17.7 (-50.9 to 1.6)	-66.1 (-75.2 to -54.4)	-17.5 (-49.5 to 22.0)	-57.8 (-65.6 to -46.2)	-29.4 (-58.6 to 15.3)	2,469.9 (2,113.4–2,864.5)
South Asia	Number: 1990	4,939,233 (4,898,664–4,979,693)	831,361 (757,132–909,394)	741,686 (670,241–801,207)	497,476 (427,049–565,767)	586,134 (523,495–635,184)	136,714 (95,509–172,176)	56,232 (47,055–66,284)	79,611 (48,880–116,828)	122,461 (103,610–142,281)	111,976 (53,420–170,094)	537 (467–617)
South Asia	Number: 2015	2,205,667 (2,147,004–2,268,067)	379,162 (339,652–428,597)	235,756 (214,894–257,976)	413,928 (376,848–455,579)	175,213 (155,858–196,689)	111,162 (84,287–129,827)	21,434 (16,548–27,403)	61,781 (37,086–84,394)	55,233 (46,485–66,357)	71,394 (50,245–93,100)	15,984 (14,800–17,272)
South Asia	Number: Cumulative change (%)	-55.3 (-56.6 to -54.1)	-54.3 (-59.5 to -48.8)	-68.1 (-71.6 to -64.5)	-16.3 (-27.3 to -3.2)	-70.0 (-74.0 to -65.2)	-16.5 (-50.0 to 3.0)	-61.5 (-71.9 to -48.2)	-19.3 (-50.6 to 19.5)	-54.5 (-62.9 to -42.1)	-30.3 (-59.3 to 14.0)	2,890.7 (2,484.7–3,334.1)
South Asia	Rate: 1990	808.2 (801.5–815.0)	127.9 (116.5–140.0)	120.5 (108.8–130.3)	76.5 (65.7–87.0)	97.0 (86.6–105.1)	21.8 (15.2–27.5)	9.7 (8.1–11.4)	12.3 (7.6–18.1)	20.5 (17.4–23.8)	17.4 (8.3–26.5)	0.1 (0.1–0.1)
South Asia	Rate: 2015	343.6 (334.4–353.5)	59.8 (53.5–67.5)	37.0 (33.7–40.5)	65.2 (59.4–71.8)	27.4 (24.4–30.8)	17.4 (13.2–20.4)	3.3 (2.5–4.2)	9.8 (5.9–13.3)	8.6 (7.2–10.4)	11.3 (7.9–14.7)	2.4 (2.3–2.6)
South Asia	Rate: Cumulative change (%)	-57.5 (-58.7 to -56.3)	-53.2 (-58.5 to -47.5)	-69.2 (-72.6 to -65.7)	-14.2 (-25.5 to -0.9)	-71.6 (-75.5 to -67.1)	-17.7 (-50.9 to 1.6)	-66.1 (-75.2 to -54.4)	-17.5 (-49.5 to 22.0)	-57.8 (-65.6 to -46.2)	-29.4 (-58.6 to 15.3)	2,469.9 (2,113.4–2,864.5)
Bangladesh	Number: 1990	586,506 (576,437–596,924)	82,399 (64,204–97,741)	112,345 (99,449–126,176)	30,550 (21,688–39,997)	87,575 (74,867–101,095)	13,656 (7,558–31,696)	2,445 (1,071–4,536)	9,467 (3,857–17,715)	1,011 (527–2,153)	11,777 (3,387–25,599)	0 (0–0)
Bangladesh	Number: 2015	157,987 (150,046–167,278)	14,544 (10,102–20,303)	22,317 (18,770–26,019)	30,689 (24,480–37,189)	4,349 (3,199–5,839)	9,932 (6,010–17,244)	478 (226–859)	5,542 (2,713–9,372)	1,210 (376–2,066)	4,739 (1,603–9,215)	16 (2–53)
Bangladesh	Number: Cumulative change (%)	-73.1 (-74.5 to -71.5)	-82.2 (-88.1 to -75.6)	-80.1 (-83.6 to -76.3)	4.1 (-28.5 to 49.2)	-95.0 (-96.5 to -93.0)	-18.7 (-64.6 to 15.8)	-76.5 (-92.2 to -46.0)	-28.7 (-73.8 to 62.8)	61.3 (-81.7 to 212.7)	-49.9 (-83.8 to 7.7)	inf (inf–inf)
Bangladesh	Rate: 1990	928.3 (912.4–944.5)	122.6 (95.6–145.4)	175.4 (155.1–196.8)	45.3 (32.2–59.3)	140.1 (119.9–162.1)	21.1 (11.6–49.2)	4.4 (2.0–8.0)	14.2 (5.8–26.5)	1.6 (0.9–3.4)	17.6 (5.1–38.2)	0.0 (0.0–0.0)
Bangladesh	Rate: 2015	267.8 (254.0–283.8)	25.5 (17.7–35.6)	38.8 (32.6–45.2)	53.7 (42.9–65.1)	7.5 (5.5–10.1)	17.1 (10.4–29.8)	0.7 (0.3–1.3)	9.7 (4.8–16.4)	2.0 (0.6–3.5)	8.3 (2.8–16.1)	0.0 (0.0–0.1)
Bangladesh	Rate: Cumulative change (%)	-71.2 (-72.7 to -69.4)	-79.1 (-85.9 to -71.3)	-77.8 (-81.8 to -73.6)	22.8 (-15.5 to 75.9)	-94.6 (-96.2 to -92.4)	-8.7 (-60.3 to 29.9)	-80.3 (-93.3 to -55.3)	-16.5 (-69.3 to 89.5)	68.8 (-80.7 to 229.2)	-41.4 (-80.9 to 25.4)	inf (inf–inf)
Bhutan	Number: 1990	2,719 (2,572–2,876)	544 (420–672)	289 (185–395)	278 (172–404)	264 (126–448)	75 (51–111)	28 (12–51)	38 (17–70)	89 (42–159)	51 (21–89)	0 (0–4)
Bhutan	Number: 2015	752 (684–836)	164 (119–214)	54 (40–70)	129 (82–180)	32 (16–53)	41 (33–51)	8 (4–14)	18 (8–34)	22 (11–36)	22 (9–38)	3 (0–9)
Bhutan	Number: Cumulative change (%)	-72.3 (-75.3 to -69.0)	-69.4 (-78.2 to -59.2)	-80.3 (-87.2 to -70.5)	-51.3 (-70.9 to -25.1)	-86.9 (-92.2 to -79.4)	-42.2 (-66.5 to -15.4)	-64.3 (-86.2 to -28.5)	-44.0 (-81.4 to 33.3)	-71.7 (-85.8 to -48.2)	-48.9 (-83.9 to 14.3)	1,344.5 (26.9–3,922.5)
Bhutan	Rate: 1990	815.6 (771.5–861.9)	155.4 (119.8–192.0)	86.3 (55.4–117.7)	79.4 (49.3–115.2)	79.6 (39.1–133.7)	22.3 (15.4–32.9)	8.8 (4.2–15.7)	11.0 (5.0–20.4)	27.2 (13.1–47.7)	15.0 (6.3–26.0)	0.3 (0.0–1.5)
Bhutan	Rate: 2015	295.6 (267.7–329.2)	67.2 (48.8–87.6)	21.3 (15.6–27.9)	52.8 (33.6–73.8)	12.9 (6.4–21.3)	16.5 (13.2–20.5)	3.2 (1.6–5.5)	7.5 (3.4–13.9)	8.9 (4.5–14.4)	9.1 (3.9–15.7)	1.2 (0.2–3.4)
Bhutan	Rate: Cumulative change (%)	-63.7 (-67.7 to -59.4)	-56.3 (-68.9 to -41.7)	-74.0 (-83.1 to -61.0)	-30.3 (-58.4 to 7.1)	-82.9 (-89.9 to -73.2)	-21.8 (-54.5 to 15.0)	-58.5 (-83.8 to -17.8)	-20.2 (-73.4 to 90.0)	-63.9 (-81.9 to -33.8)	-27.6 (-77.3 to 61.8)	1,684.0 (48.5–4,910.3)
India	Number: 1990	3,708,158 (3,670,701–3,747,173)	670,447 (611,230–732,588)	529,408 (468,469–577,353)	363,134 (309,873–413,775)	402,457 (351,691–441,347)	108,861 (66,159–140,447)	49,852 (41,654–59,267)	57,409 (35,454–85,192)	97,062 (81,407–113,088)	83,001 (39,542–128,360)	530 (464–596)
India	Number: 2015	1,609,284 (1,554,454–1,665,347)	307,619 (275,366–345,257)	166,408 (149,061–185,963)	272,346 (245,766–303,641)	131,008 (116,989–145,906)	83,819 (56,901–102,059)	17,214 (12,799–22,501)	42,759 (25,309–59,305)	35,887 (30,723–43,011)	48,785 (34,339–65,375)	15,726 (14,614–16,947)
India	Number: Cumulative change (%)	-56.6 (-58.1 to -55.0)	-54.0 (-59.5 to -48.4)	-68.5 (-72.5 to -64.2)	-24.5 (-35.1 to -11.6)	-67.3 (-71.8 to -61.9)	-20.9 (-50.3 to 0.1)	-65.1 (-75.3 to -51.9)	-22.3 (-54.6 to 16.3)	-62.8 (-69.1 to -52.9)	-35.3 (-64.6 to 7.1)	2,883.4 (2,522.6–3,323.4)
India	Rate: 1990	798.5 (790.4–807.1)	136.4 (124.3–149.1)	113.6 (100.4–124.1)	73.8 (63.1–84.1)	87.6 (76.6–96.0)	22.9 (13.9–29.5)	11.2 (9.3–13.3)	11.7 (7.3–17.4)	21.4 (18.0–24.9)	17.1 (8.2–26.4)	0.1 (0.1–0.1)
India	Rate: 2015	337.1 (325.5–349.0)	65.7 (58.8–73.7)	35.1 (31.4–39.2)	58.2 (52.5–64.8)	27.5 (24.5–30.7)	17.8 (12.1–21.6)	3.5 (2.6–4.6)	9.2 (5.4–12.7)	7.5 (6.4–8.9)	10.4 (7.3–14.0)	3.2 (3.0–3.4)
India	Rate: Cumulative change (%)	-57.8 (-59.3 to -56.2)	-51.7 (-57.5 to -45.8)	-69.0 (-72.9 to -64.7)	-20.7 (-31.8 to -7.2)	-68.5 (-72.8 to -63.2)	-20.1 (-50.1 to 1.2)	-68.4 (-77.8 to -56.2)	-18.6 (-52.2 to 21.9)	-64.9 (-70.9 to -55.7)	-32.9 (-63.1 to 10.8)	2,519.2 (2,194.5–2,912.3)
Nepal	Number: 1990	109,823 (107,351–112,264)	5,701 (3,512–8,274)	26,832 (23,237–30,779)	12,736 (8,762–16,355)	15,580 (12,353–18,727)	1,470 (1,064–2,110)	351 (178–648)	1,518 (633–2,763)	2,738 (1,442–4,610)	2,092 (603–4,375)	0 (0–0)
Nepal	Number: 2015	30,824 (28,568–33,068)	1,810 (1,037–2,833)	4,815 (4,032–5,613)	5,605 (4,275–6,908)	1,707 (1,283–2,183)	1,095 (713–1,442)	179 (97–302)	900 (416–1,653)	740 (334–1,162)	864 (304–1,669)	104 (7–372)
Nepal	Number: Cumulative change (%)	-71.9 (-74.0 to -69.7)	-67.1 (-81.8 to -48.0)	-81.9 (-85.5 to -77.9)	-54.7 (-68.2 to -36.2)	-88.9 (-92.2 to -84.4)	-20.8 (-65.4 to 14.9)	-41.0 (-76.3 to 16.0)	-30.8 (-74.8 to 50.2)	-71.0 (-83.8 to -50.0)	-46.3 (-85.0 to 24.7)	819,899.8 (72,874.1–1,862,866.5)
Nepal	Rate: 1990	924.1 (903.2–944.9)	43.8 (27.0–63.6)	222.0 (191.0–255.3)	97.6 (67.3–125.3)	134.3 (106.5–161.1)	11.8 (8.5–17.3)	3.3 (1.7–6.1)	11.7 (4.9–21.3)	23.8 (12.6–40.0)	16.2 (4.7–33.7)	0.0 (0.0–0.0)
Nepal	Rate: 2015	273.1 (253.4–293.0)	17.2 (9.9–26.9)	44.7 (37.3–52.1)	53.3 (40.7–65.7)	15.3 (11.3–19.6)	10.2 (6.6–13.4)	1.4 (0.8–2.4)	8.6 (4.0–15.7)	6.5 (2.9–10.4)	8.2 (2.9–15.9)	0.8 (0.1–3.0)
Nepal	Rate: Cumulative change (%)	-70.4 (-72.6 to -68.1)	-59.2 (-77.4 to -35.6)	-79.7 (-83.9 to -75.3)	-43.8 (-60.6 to -20.9)	-88.4 (-91.9 to -83.8)	-8.1 (-60.8 to 34.7)	-50.3 (-79.1 to -4.4)	-14.7 (-68.9 to 84.0)	-71.0 (-83.7 to -50.2)	-34.1 (-81.6 to 51.6)	780,320.2 (68,467.8–1,813,204.8)
Pakistan	Number: 1990	532,026 (520,000–544,108)	72,269 (55,168–90,074)	72,810 (59,846–85,934)	90,776 (69,788–112,167)	80,256 (64,513–94,403)	12,649 (10,106–16,006)	3,554 (2,045–5,687)	11,177 (5,244–19,837)	21,558 (14,258–32,371)	15,052 (6,348–26,915)	6 (0–32)
Pakistan	Number: 2015	406,817 (380,095–436,449)	55,023 (38,213–73,846)	42,161 (33,911–51,229)	105,157 (83,448–128,075)	38,116 (29,113–48,639)	16,273 (12,324–19,969)	3,553 (2,051–5,557)	12,561 (5,883–22,117)	17,372 (11,794–26,162)	16,982 (8,294–28,089)	133 (24–351)
Pakistan	Number: Cumulative change (%)	-23.5 (-29.0 to -17.8)	-22.2 (-49.3 to 10.9)	-41.4 (-55.7 to -24.5)	18.1 (-13.1 to 55.7)	-51.9 (-65.5 to -35.7)	31.4 (-13.4 to 75.4)	9.4 (-50.7 to 98.2)	29.1 (-53.3 to 177.0)	-15.3 (-52.0 to 36.8)	32.8 (-50.9 to 173.5)	8,294.8 (418.2–24,568.0)
Pakistan	Rate: 1990	740.4 (723.6–756.6)	93.0 (71.1–115.9)	99.5 (81.8–117.5)	116.8 (89.8–144.4)	112.9 (91.0–132.6)	17.3 (13.8–21.9)	5.8 (3.4–9.2)	14.5 (6.8–25.7)	30.4 (20.4–45.6)	19.6 (8.3–35.2)	0.0 (0.0–0.0)
Pakistan	Rate: 2015	430.2 (402.3–460.6)	55.9 (38.9–75.0)	44.3 (35.7–53.7)	106.8 (84.8–130.2)	40.5 (31.1–51.3)	17.1 (12.9–20.9)	4.2 (2.4–6.6)	12.8 (6.0–22.6)	18.5 (12.6–27.6)	17.4 (8.5–28.7)	0.1 (0.0–0.4)
Pakistan	Rate: Cumulative change (%)	-41.9 (-46.0 to -37.7)	-38.6 (-59.9 to -12.6)	-54.9 (-65.9 to -41.9)	-6.8 (-31.4 to 22.9)	-63.7 (-73.7 to -51.8)	0.9 (-33.2 to 34.3)	-20.9 (-64.5 to 42.5)	1.6 (-63.3 to 117.7)	-36.1 (-63.4 to 2.2)	4.1 (-61.3 to 114.5)	6,032.9 (293.0–17,745.8)
Sub-Saharan Africa	Number: 1990	4,067,541 (4,027,451–4,106,894)	196,966 (170,440–226,421)	526,716 (451,223–599,277)	176,950 (145,437–209,038)	530,201 (443,588–617,201)	125,861 (89,416–201,588)	713,812 (517,854–924,083)	166,751 (126,304–217,525)	152,052 (117,510–187,335)	103,005 (74,810–138,772)	35,187 (32,575–38,490)
Sub-Saharan Africa	Number: 2015	3,376,180 (3,278,905–3,476,219)	200,994 (175,652–229,643)	379,085 (345,501–416,777)	215,210 (179,820–256,225)	336,463 (299,026–376,161)	183,367 (157,352–223,408)	504,400 (385,529–622,144)	229,956 (180,234–292,799)	136,096 (110,133–169,618)	92,012 (68,864–120,339)	179,315 (172,692–185,867)
Sub-Saharan Africa	Number: Cumulative change (%)	-17.0 (-19.5 to -14.5)	2.5 (-11.7 to 19.0)	-27.6 (-37.5 to -16.2)	22.5 (2.0–47.8)	-36.0 (-47.0 to -22.8)	52.9 (8.4–92.5)	-28.0 (-46.2 to -4.7)	39.8 (9.6–79.0)	-9.2 (-28.9 to 18.5)	-8.6 (-35.1 to 26.5)	410.7 (368.7–448.5)
Sub-Saharan Africa	Rate: 1990	1,185.6 (1,172.9–1,198.5)	50.4 (43.6–57.9)	149.6 (128.0–170.4)	45.2 (37.1–53.4)	154.5 (129.0–180.5)	34.6 (24.3–55.8)	214.1 (156.5–276.0)	42.8 (32.4–55.7)	45.0 (35.1–55.2)	26.5 (19.3–35.7)	10.3 (9.5–11.3)
Sub-Saharan Africa	Rate: 2015	566.8 (550.6–583.7)	31.2 (27.3–35.6)	62.6 (57.0–68.8)	33.4 (27.9–39.8)	56.1 (49.9–62.6)	29.7 (25.5–36.1)	85.1 (65.5–104.8)	35.8 (28.0–45.5)	23.2 (18.9–28.6)	14.3 (10.7–18.7)	33.5 (32.3–34.8)
Sub-Saharan Africa	Rate: Cumulative change (%)	-52.2 (-53.6 to -50.7)	-37.8 (-46.4 to -27.8)	-57.9 (-63.7 to -51.2)	-25.6 (-38.1 to -10.3)	-63.4 (-69.6 to -55.9)	-9.8 (-36.7 to 13.8)	-59.5 (-69.5 to -47.0)	-15.3 (-33.6 to 8.3)	-47.8 (-58.8 to -32.2)	-44.7 (-60.7 to -23.4)	225.9 (199.3–249.6)
Southern Sub-Saharan Africa	Number: 1990	144,842 (141,135–149,018)	11,693 (10,224–13,256)	20,799 (19,404–22,225)	7,794 (6,684–8,986)	29,998 (28,141–31,984)	4,607 (4,083–5,421)	831 (508–1,220)	3,686 (2,662–5,010)	2,585 (2,258–2,953)	12,584 (11,251–13,764)	3,422 (2,983–3,894)
Southern Sub-Saharan Africa	Number: 2015	126,790 (121,509–132,122)	10,049 (8,763–11,333)	9,265 (8,216–10,386)	9,157 (7,684–10,766)	11,466 (9,950–13,112)	4,269 (3,680–4,825)	873 (395–1,624)	4,274 (3,019–5,932)	1,932 (1,587–2,430)	6,571 (5,670–7,470)	40,778 (38,455–43,279)
Southern Sub-Saharan Africa	Number: Cumulative change (%)	-12.4 (-16.7 to -8.0)	-13.6 (-27.0 to 1.8)	-55.4 (-61.3 to -48.9)	18.3 (-5.6 to 43.7)	-61.7 (-67.2 to -55.7)	-6.5 (-27.1 to 9.7)	4.8 (-41.9 to 86.3)	18.0 (-16.1 to 61.5)	-24.8 (-40.4 to -1.6)	-47.6 (-55.0 to -39.1)	1,097.7 (954.0–1,247.7)
Southern Sub-Saharan Africa	Rate: 1990	497.4 (484.9–511.5)	38.6 (33.8–43.8)	70.5 (65.8–75.3)	25.7 (22.1–29.7)	101.6 (95.3–108.3)	15.5 (13.7–18.2)	2.9 (1.8–4.3)	12.2 (8.8–16.6)	9.0 (7.9–10.3)	41.6 (37.2–45.5)	11.7 (10.2–13.3)
Southern Sub-Saharan Africa	Rate: 2015	386.8 (371.3–402.7)	29.4 (25.6–33.1)	27.8 (24.6–31.1)	26.8 (22.5–31.5)	34.3 (29.9–39.1)	12.7 (10.9–14.3)	2.6 (1.2–4.9)	12.5 (8.8–17.3)	5.9 (4.9–7.4)	19.2 (16.6–21.8)	128.4 (121.1–136.3)
Southern Sub-Saharan Africa	Rate: Cumulative change (%)	-22.2 (-26.0 to -18.4)	-23.5 (-35.4 to -9.9)	-60.5 (-65.8 to -54.9)	4.7 (-16.4 to 27.2)	-66.2 (-71.0 to -60.9)	-17.7 (-35.8 to -3.6)	-10.6 (-50.1 to 57.7)	4.3 (-25.8 to 42.6)	-33.6 (-47.1 to -13.8)	-53.7 (-60.2 to -46.1)	1,005.7 (872.0–1,147.9)
Botswana	Number: 1990	3,118 (2,837–3,478)	275 (175–389)	419 (334–508)	166 (94–257)	682 (541–812)	98 (77–125)	15 (6–28)	72 (29–134)	50 (29–73)	279 (160–405)	151 (120–186)
Botswana	Number: 2015	2,321 (1,894–2,945)	209 (132–301)	160 (105–229)	152 (87–236)	175 (107–259)	92 (64–131)	9 (3–21)	77 (36–140)	29 (17–49)	203 (111–314)	623 (513–732)
Botswana	Number: Cumulative change (%)	-25.4 (-39.7 to -5.2)	-20.9 (-53.1 to 21.4)	-61.3 (-75.3 to -42.5)	-0.7 (-50.8 to 75.9)	-73.9 (-84.2 to -59.9)	-4.5 (-36.5 to 35.4)	-38.2 (-76.5 to 31.7)	27.0 (-55.7 to 184.0)	-37.7 (-67.7 to 9.6)	-22.3 (-62.3 to 34.5)	317.0 (232.9–416.4)
Botswana	Rate: 1990	373.4 (335.4–424.8)	31.7 (20.2–44.9)	49.4 (39.5–60.3)	19.1 (10.9–29.6)	79.9 (63.7–95.2)	11.5 (9.0–14.8)	2.0 (0.9–3.5)	8.3 (3.4–15.5)	6.2 (3.6–9.1)	32.2 (18.5–46.7)	17.8 (14.1–22.0)
Botswana	Rate: 2015	236.9 (193.0–300.8)	20.6 (13.1–29.8)	16.3 (10.7–23.3)	15.0 (8.6–23.3)	17.7 (11.0–26.0)	9.2 (6.5–13.1)	1.0 (0.3–2.2)	7.7 (3.6–13.8)	3.1 (1.8–5.2)	20.1 (11.0–31.0)	64.3 (52.9–75.6)
Botswana	Rate: Cumulative change (%)	-36.3 (-48.8 to -18.4)	-32.3 (-59.8 to 3.9)	-66.6 (-78.6 to -50.5)	-15.0 (-57.8 to 50.6)	-77.5 (-86.3 to -65.4)	-18.6 (-45.6 to 15.2)	-48.8 (-80.6 to 8.0)	8.6 (-62.1 to 142.6)	-47.2 (-72.3 to -7.2)	-33.5 (-67.7 to 15.0)	264.7 (192.0–350.4)
Lesotho	Number: 1990	6,052 (5,816–6,288)	629 (413–862)	995 (815–1,168)	328 (181–515)	1,469 (1,194–1,688)	129 (87–200)	0 (0–0)	117 (48–230)	92 (55–131)	626 (342–938)	53 (38–73)
Lesotho	Number: 2015	7,462 (6,679–8,332)	660 (419–905)	770 (585–984)	405 (225–622)	923 (703–1,174)	156 (117–216)	0 (0–0)	160 (70–296)	94 (63–139)	571 (317–884)	2,196 (1,936–2,447)
Lesotho	Number: Cumulative change (%)	23.4 (9.5–38.9)	9.6 (-37.8 to 69.6)	-21.7 (-42.7 to 5.2)	34.7 (-36.7 to 143.4)	-36.4 (-53.4 to -15.0)	26.5 (-13.5 to 77.6)	nan (nan–nan)	60.8 (-44.9 to 258.4)	9.2 (-38.1 to 78.4)	-0.9 (-54.5 to 79.6)	4,143.6 (2,948.7–5,540.0)
Lesotho	Rate: 1990	620.2 (595.8–644.6)	61.6 (40.5–84.4)	100.7 (82.5–117.5)	32.2 (17.8–50.4)	149.7 (122.1–172.4)	13.0 (8.8–20.1)	0.0 (0.0–0.0)	11.6 (4.8–22.5)	9.7 (5.9–13.9)	61.4 (33.5–91.9)	5.5 (3.9–7.5)
Lesotho	Rate: 2015	707.6 (633.0–790.0)	60.1 (38.2–82.4)	72.3 (55.0–92.0)	36.9 (20.6–56.6)	87.6 (66.9–111.1)	14.5 (10.9–20.1)	0.0 (0.0–0.0)	14.6 (6.4–27.0)	9.1 (6.2–13.3)	52.0 (29.0–80.5)	213.4 (188.5–237.7)
Lesotho	Rate: Cumulative change (%)	14.2 (1.3–28.1)	1.9 (-42.1 to 57.8)	-27.4 (-46.7 to -2.6)	25.2 (-41.1 to 125.5)	-40.8 (-56.6 to -21.1)	16.9 (-19.7 to 63.9)	nan (nan–nan)	49.2 (-48.7 to 231.5)	-0.7 (-43.0 to 60.7)	-7.9 (-57.6 to 66.5)	3,938.2 (2,815.7–5,232.4)
Namibia	Number: 1990	4,404 (4,196–4,623)	407 (268–567)	577 (448–693)	231 (126–365)	964 (756–1,146)	110 (83–151)	196 (78–356)	101 (44–191)	66 (41–93)	431 (243–629)	41 (35–48)
Namibia	Number: 2015	3,991 (3,397–4,658)	410 (268–577)	357 (252–478)	285 (162–439)	453 (306–629)	137 (101–181)	86 (26–182)	131 (58–251)	53 (34–79)	388 (206–628)	812 (668–953)
Namibia	Number: Cumulative change (%)	-9.3 (-23.3 to 6.4)	4.6 (-38.0 to 63.3)	-36.9 (-58.3 to -10.2)	34.6 (-35.9 to 141.0)	-52.1 (-69.3 to -29.1)	27.1 (-15.0 to 77.1)	-49.9 (-84.1 to 9.8)	50.4 (-47.1 to 234.5)	-14.6 (-53.6 to 42.2)	-3.6 (-56.1 to 68.1)	1,842.0 (1,582.4–2,112.6)
Namibia	Rate: 1990	490.0 (468.1–513.4)	42.0 (27.6–58.4)	63.1 (49.0–75.9)	23.9 (13.1–37.6)	106.3 (83.6–126.6)	11.9 (8.9–16.2)	22.8 (9.5–40.9)	10.5 (4.6–19.7)	7.7 (4.9–10.8)	44.5 (25.1–64.8)	4.6 (3.9–5.4)
Namibia	Rate: 2015	320.1 (273.9–372.2)	31.1 (20.4–43.8)	28.3 (20.1–37.6)	21.7 (12.3–33.4)	35.8 (24.3–49.5)	10.6 (7.9–14.1)	7.1 (2.2–14.8)	10.0 (4.4–19.1)	4.4 (2.9–6.4)	29.5 (15.7–47.7)	68.4 (56.5–80.2)
Namibia	Rate: Cumulative change (%)	-34.6 (-44.5 to -23.4)	-22.8 (-54.2 to 20.5)	-54.4 (-69.7 to -34.9)	-0.7 (-52.6 to 77.7)	-65.7 (-77.9 to -49.3)	-8.0 (-38.4 to 28.2)	-65.1 (-88.9 to -24.2)	10.7 (-61.0 to 146.2)	-40.0 (-66.4 to -2.2)	-28.9 (-67.6 to 23.8)	1,390.4 (1,199.1–1,595.0)
South Africa	Number: 1990	98,702 (94,926–102,562)	7,490 (6,707–8,310)	14,947 (13,806–16,079)	4,343 (3,771–4,922)	21,136 (19,721–22,788)	3,040 (2,686–3,584)	132 (89–193)	1,893 (1,376–2,540)	1,524 (1,306–1,731)	10,301 (9,272–11,236)	129 (113–145)
South Africa	Number: 2015	69,092 (65,522–72,771)	5,483 (4,912–6,052)	4,249 (3,659–4,939)	3,899 (3,479–4,384)	3,712 (3,057–4,458)	2,300 (1,778–2,717)	21 (15–29)	1,720 (1,274–2,197)	666 (544–784)	4,578 (3,955–5,114)	27,072 (25,182–29,181)
South Africa	Number: Cumulative change (%)	-30.0 (-34.3 to -25.0)	-26.5 (-36.1 to -16.0)	-71.5 (-76.2 to -66.5)	-9.6 (-24.7 to 9.5)	-82.4 (-85.6 to -78.3)	-23.4 (-46.8 to -6.0)	-82.7 (-90.5 to -72.6)	-8.5 (-22.8 to 5.7)	-55.8 (-66.4 to -42.6)	-55.4 (-61.8 to -48.6)	20,915.8 (18,920.5–23,000.3)
South Africa	Rate: 1990	517.9 (498.1–538.0)	38.1 (34.1–42.2)	77.6 (71.7–83.4)	22.1 (19.2–25.0)	109.7 (102.4–118.4)	15.7 (13.9–18.5)	0.7 (0.5–1.0)	9.6 (7.0–12.9)	8.0 (6.9–9.1)	52.4 (47.2–57.2)	0.7 (0.6–0.8)
South Africa	Rate: 2015	347.4 (329.7–365.5)	27.0 (24.2–29.8)	21.2 (18.3–24.6)	19.2 (17.2–21.6)	18.5 (15.3–22.2)	11.4 (8.8–13.5)	0.1 (0.1–0.2)	8.5 (6.3–10.8)	3.4 (2.7–3.9)	22.6 (19.5–25.2)	138.9 (129.2–149.7)
South Africa	Rate: Cumulative change (%)	-32.9 (-37.0 to -28.3)	-28.8 (-38.0 to -18.6)	-72.7 (-77.1 to -67.9)	-12.3 (-27.0 to 6.2)	-83.1 (-86.2 to -79.2)	-26.5 (-48.9 to -9.8)	-83.5 (-90.9 to -73.8)	-11.4 (-25.3 to 2.3)	-57.8 (-67.9 to -45.2)	-56.8 (-63.0 to -50.2)	20,582.2 (18,630.6–22,623.3)
Swaziland	Number: 1990	2,981 (2,845–3,129)	214 (139–293)	508 (414–608)	129 (76–195)	754 (611–889)	79 (61–108)	0 (0–0)	62 (28–116)	54 (31–80)	231 (138–330)	109 (81–142)
Swaziland	Number: 2015	3,218 (2,853–3,622)	202 (140–270)	350 (262–459)	140 (82–211)	399 (276–525)	77 (58–99)	0 (0–0)	76 (35–137)	48 (30–68)	204 (118–304)	1,006 (871–1,148)
Swaziland	Number: Cumulative change (%)	8.0 (-5.6 to 22.6)	-1.5 (-38.9 to 52.7)	-30.0 (-51.5 to -4.4)	17.0 (-41.8 to 108.6)	-46.3 (-64.2 to -26.0)	-0.9 (-26.8 to 28.6)	-35.3 (-87.2 to 67.3)	41.4 (-45.5 to 193.5)	-4.6 (-48.7 to 63.5)	-6.6 (-52.1 to 52.1)	842.3 (622.7–1,103.0)
Swaziland	Rate: 1990	482.5 (459.7–506.8)	32.1 (20.9–43.8)	79.5 (64.9–94.9)	19.4 (11.4–29.1)	117.9 (95.8–138.6)	12.4 (9.6–16.8)	0.0 (0.0–0.1)	9.3 (4.3–17.4)	9.1 (5.5–13.3)	34.6 (20.7–49.4)	17.2 (12.9–22.4)
Swaziland	Rate: 2015	491.1 (435.9–553.0)	29.5 (20.5–39.3)	52.4 (39.2–68.4)	20.5 (12.0–30.7)	59.6 (41.4–78.3)	11.5 (8.8–14.8)	0.0 (0.0–0.1)	11.2 (5.1–20.0)	7.4 (4.7–10.5)	29.7 (17.2–44.3)	158.1 (137.1–180.5)
Swaziland	Rate: Cumulative change (%)	1.9 (-10.7 to 15.6)	-4.0 (-40.5 to 48.8)	-33.2 (-53.8 to -9.1)	13.9 (-43.3 to 102.9)	-48.8 (-65.7 to -29.5)	-5.5 (-30.2 to 22.4)	-42.0 (-88.6 to 49.8)	37.7 (-46.8 to 185.9)	-13.6 (-51.8 to 45.0)	-9.0 (-53.3 to 48.1)	842.7 (625.0–1,101.4)
Zimbabwe	Number: 1990	29,582 (28,704–30,431)	2,675 (1,845–3,642)	3,351 (2,812–4,009)	2,593 (1,775–3,504)	4,991 (4,108–5,874)	1,148 (946–1,396)	486 (259–777)	1,439 (811–2,276)	797 (589–1,057)	712 (372–1,207)	2,937 (2,503–3,399)
Zimbabwe	Number: 2015	40,704 (37,291–44,510)	3,083 (2,142–4,107)	3,376 (2,693–4,143)	4,272 (3,027–5,643)	5,801 (4,580–7,215)	1,506 (1,220–1,836)	755 (317–1,463)	2,107 (1,226–3,389)	1,040 (720–1,502)	625 (260–1,168)	9,067 (7,828–10,372)
Zimbabwe	Number: Cumulative change (%)	37.6 (25.8–50.8)	20.5 (-27.7 to 84.5)	1.9 (-24.2 to 32.2)	70.6 (7.3–147.4)	17.5 (-12.2 to 54.0)	32.3 (5.2–63.9)	55.5 (-20.9 to 178.1)	58.9 (-25.3 to 186.8)	34.1 (-17.2 to 107.2)	-0.7 (-67.1 to 111.0)	209.8 (172.7–251.2)
Zimbabwe	Rate: 1990	439.0 (425.9–452.0)	37.8 (26.1–51.5)	49.1 (41.2–58.5)	36.7 (25.1–49.5)	73.1 (60.3–85.8)	16.7 (13.8–20.2)	7.5 (4.2–11.8)	20.4 (11.5–32.2)	12.2 (9.1–15.9)	10.1 (5.3–17.1)	43.2 (36.7–50.0)
Zimbabwe	Rate: 2015	453.2 (416.3–496.9)	31.4 (21.8–41.8)	36.3 (29.1–44.4)	43.5 (30.9–57.5)	61.8 (49.0–76.8)	15.9 (12.9–19.2)	8.6 (3.8–16.5)	21.5 (12.5–34.6)	11.7 (8.2–16.5)	6.4 (2.7–11.9)	111.1 (96.2–127.1)
Zimbabwe	Rate: Cumulative change (%)	3.3 (-5.7 to 13.1)	-13.1 (-47.9 to 33.0)	-25.3 (-44.0 to -3.7)	23.0 (-22.7 to 78.3)	-14.6 (-36.0 to 11.5)	-4.3 (-23.6 to 18.1)	14.0 (-42.0 to 104.5)	14.5 (-46.0 to 106.6)	-1.7 (-37.5 to 49.6)	-28.4 (-76.2 to 52.1)	158.2 (127.2–193.3)

© 2017 Global Burden of Disease Child and Adolescent Health Collaboration. JAMA Pediatrics.

eTable 1. Number of Deaths, Death Rates (per 100,000 population), and Cumulative Percent Change with 95% Uncertainty Intervals (UI) for the Top 10 Global Causes of Death in 195 Countries and Territories, Aged 0 to 19, Both Sexes, 1990 and 2015*Note: best viewed by enlarging in browser.*

Location	Measure	All causes	Neonatal preterm birth complications	Lower respiratory infections	Neonatal encephalopathy due to birth asphyxia and trauma	Diarrheal diseases	Congenital anomalies	Malaria	Neonatal sepsis and other neonatal infections	Meningitis	Other neonatal disorders	HIV/AIDS
Western Sub-Saharan Africa	Number: 1990	1,853,426 (1,827,608–1,878,503)	84,305 (66,917–103,056)	226,611 (187,439–266,044)	81,707 (62,235–103,618)	246,187 (198,450–295,273)	53,610 (31,942–102,712)	368,547 (260,659–487,929)	96,745 (68,550–130,656)	76,499 (57,010–98,926)	32,635 (20,348–48,474)	5,590 (4,077–7,776)
Western Sub-Saharan Africa	Number: 2015	1,680,122 (1,610,324–1,759,179)	93,613 (74,484–114,561)	170,118 (149,354–193,360)	105,859 (80,488–135,229)	197,475 (167,873–231,666)	72,544 (50,731–115,684)	353,769 (278,800–430,240)	141,738 (104,869–184,083)	71,368 (54,264–95,030)	29,955 (19,426–45,545)	47,729 (43,776–51,851)
Western Sub-Saharan Africa	Number: Cumulative change (%)	-9.3 (-13.3 to -5.1)	12.2 (-13.5 to 39.3)	-24.3 (-36.9 to -9.1)	31.6 (-3.3 to 72.7)	-18.7 (-35.7 to 2.3)	42.7 (6.5–88.9)	-1.5 (-26.7 to 31.1)	49.8 (7.6–106.9)	-5.0 (-31.0 to 30.8)	-3.4 (-47.0 to 60.2)	786.3 (508.7–1,065.1)
Western Sub-Saharan Africa	Rate: 1990	1,333.6 (1,312.8–1,355.0)	52.7 (41.8–64.4)	159.6 (131.3–188.2)	51.0 (38.9–64.6)	177.6 (143.2–213.5)	36.3 (21.3–70.5)	275.3 (196.8–362.4)	60.6 (42.9–81.7)	56.0 (42.0–72.2)	20.5 (12.9–30.5)	4.1 (3.0–5.7)
Western Sub-Saharan Africa	Rate: 2015	665.5 (637.8–695.7)	34.1 (27.1–41.7)	66.7 (58.5–75.9)	38.6 (29.3–49.3)	77.6 (66.1–90.9)	27.8 (19.4–44.3)	141.8 (111.8–172.2)	51.7 (38.3–67.1)	28.9 (22.1–38.1)	11.0 (7.1–16.6)	21.4 (19.6–23.3)
Western Sub-Saharan Africa	Rate: Cumulative change (%)	-50.1 (-52.3 to -47.8)	-34.6 (-49.5 to -18.8)	-57.8 (-64.9 to -49.1)	-23.2 (-43.5 to 0.8)	-55.8 (-65.0 to -44.2)	-19.0 (-40.1 to 7.8)	-47.2 (-60.6 to -30.3)	-12.8 (-37.3 to 20.0)	-47.5 (-61.8 to -28.4)	-43.9 (-69.1 to -7.2)	443.8 (274.9–614.7)
Benin	Number: 1990	44,308 (43,244–45,338)	2,499 (1,691–3,440)	7,283 (4,793–9,380)	2,055 (1,243–3,003)	5,790 (3,525–7,796)	1,455 (788–2,770)	5,193 (2,231–8,454)	2,301 (1,288–3,414)	2,076 (1,151–3,225)	723 (356–1,312)	66 (26–133)
Benin	Number: 2015	34,862 (32,136–38,095)	2,560 (1,651–3,556)	4,450 (3,455–5,563)	2,359 (1,458–3,421)	2,570 (1,757–3,472)	1,746 (1,214–2,646)	6,913 (4,665–9,343)	2,784 (1,600–4,131)	1,680 (1,065–2,534)	580 (256–1,153)	501 (415–592)
Benin	Number: Cumulative change (%)	-21.3 (-27.8 to -13.9)	5.9 (-35.5 to 61.8)	-35.8 (-56.2 to -6.6)	21.6 (-35.0 to 99.4)	-52.1 (-72.9 to -24.7)	31.3 (-18.9 to 95.1)	57.6 (-23.8 to 189.6)	30.6 (-35.0 to 133.1)	-10.2 (-55.1 to 64.0)	-7.3 (-69.1 to 110.2)	837.0 (282.6–1,706.5)
Benin	Rate: 1990	1,219.4 (1,188.1–1,250.7)	59.5 (40.3–81.8)	195.9 (128.1–252.9)	49.0 (29.6–71.4)	160.1 (97.3–216.7)	37.9 (20.2–72.8)	149.5 (65.8–242.3)	55.0 (30.8–81.3)	58.3 (32.8–89.7)	17.4 (8.7–31.2)	1.9 (0.8–3.7)
Benin	Rate: 2015	529.9 (487.7–580.5)	36.4 (23.5–50.5)	66.6 (52.0–83.0)	33.5 (20.7–48.6)	38.9 (26.9–52.3)	25.8 (18.0–38.9)	106.3 (72.1–143.2)	39.6 (22.8–58.8)	26.0 (16.8–38.8)	8.3 (3.7–16.4)	8.4 (7.0–9.9)
Benin	Rate: Cumulative change (%)	-56.5 (-60.2 to -52.3)	-36.9 (-61.5 to -3.2)	-64.1 (-75.7 to -47.5)	-27.5 (-61.1 to 18.4)	-73.7 (-85.0 to -58.3)	-25.0 (-54.5 to 11.8)	-15.8 (-59.1 to 53.6)	-22.3 (-61.3 to 38.0)	-50.4 (-74.9 to -10.3)	-45.2 (-81.6 to 24.2)	461.7 (130.2–981.7)
Burkina Faso	Number: 1990	92,522 (90,638–94,405)	2,021 (1,317–2,934)	13,961 (10,942–17,347)	3,387 (2,049–4,947)	14,822 (10,823–19,181)	2,766 (1,611–5,406)	24,373 (16,593–32,375)	3,485 (2,030–5,222)	4,104 (2,526–6,149)	1,231 (568–2,227)	746 (562–978)
Burkina Faso	Number: 2015	88,247 (82,053–95,313)	3,138 (2,039–4,499)	10,900 (8,530–13,536)	4,552 (2,804–6,552)	8,484 (6,180–11,342)	3,942 (2,482–6,590)	27,041 (20,250–34,152)	5,161 (3,017–7,637)	4,547 (2,996–6,647)	1,114 (528–2,062)	846 (671–1,028)
Burkina Faso	Number: Cumulative change (%)	-4.6 (-11.5 to 3.1)	63.5 (-3.0 to 163.2)	-20.7 (-42.3 to 5.5)	41.6 (-20.2 to 127.4)	-41.2 (-61.4 to -16.1)	49.2 (6.3–102.2)	14.2 (-20.2 to 56.0)	57.7 (-15.2 to 167.2)	17.8 (-35.4 to 94.3)	4.8 (-64.1 to 120.1)	15.3 (-12.5 to 43.2)
Burkina Faso	Rate: 1990	1,437.2 (1,404.9–1,471.5)	26.8 (17.6–38.9)	209.1 (164.3–259.4)	44.9 (27.3–65.4)	229.4 (167.5–298.1)	39.9 (23.0–79.1)	392.8 (270.3–521.5)	46.3 (27.0–69.3)	64.3 (39.9–96.2)	16.5 (7.6–29.5)	11.8 (9.0–15.6)
Burkina Faso	Rate: 2015	740.8 (686.9–801.2)	24.1 (15.7–34.6)	89.6 (70.1–111.2)	35.0 (21.6–50.3)	70.6 (51.6–93.7)	31.8 (19.9–53.4)	229.5 (172.1–289.7)	39.7 (23.3–58.8)	38.6 (25.8–55.8)	8.6 (4.1–15.9)	8.5 (6.8–10.3)
Burkina Faso	Rate: Cumulative change (%)	-48.4 (-52.2 to -44.1)	-5.3 (-43.7 to 52.6)	-56.4 (-68.4 to -41.7)	-17.8 (-53.6 to 31.7)	-68.4 (-79.2 to -54.9)	-16.2 (-41.0 to 14.3)	-40.0 (-57.9 to -18.4)	-8.7 (-50.7 to 53.8)	-36.3 (-63.9 to 4.5)	-39.5 (-79.2 to 26.9)	-27.1 (-44.3 to -9.6)
Cameroon	Number: 1990	79,996 (78,007–81,994)	3,867 (2,538–5,490)	10,235 (7,233–13,188)	3,736 (2,263–5,559)	11,850 (7,623–16,110)	3,067 (1,958–4,971)	10,753 (5,985–15,681)	5,056 (2,949–7,341)	2,807 (1,699–4,358)	1,473 (705–2,580)	585 (306–1,000)
Cameroon	Number: 2015	88,802 (79,663–98,407)	5,397 (3,376–7,671)	10,472 (7,905–13,538)	5,413 (3,290–7,899)	6,847 (4,610–9,579)	4,723 (3,175–7,292)	16,604 (10,342–23,071)	7,948 (4,708–11,458)	3,235 (2,111–4,967)	1,576 (679–2,925)	4,218 (3,531–4,958)
Cameroon	Number: Cumulative change (%)	11.0 (-0.6 to 24.0)	45.4 (-16.5 to 126.4)	5.9 (-28.5 to 51.6)	54.7 (-19.2 to 162.6)	-39.1 (-63.7 to -1.7)	58.9 (12.7–119.8)	65.9 (1.5–174.0)	65.4 (-7.5 to 165.6)	24.5 (-34.6 to 116.2)	21.9 (-60.3 to 153.1)	712.1 (312.1–1,217.6)
Cameroon	Rate: 1990	920.8 (898.1–944.4)	39.7 (26.0–56.3)	115.0 (81.5–148.2)	38.3 (23.3–57.0)	135.6 (87.8–184.0)	33.6 (21.4–54.5)	127.3 (71.7–185.1)	52.0 (30.4–75.5)	33.4 (20.5–50.6)	15.2 (7.3–26.6)	6.8 (3.6–11.6)
Cameroon	Rate: 2015	622.0 (559.5–688.4)	35.2 (22.0–50.0)	71.9 (54.5–92.7)	35.3 (21.4–51.4)	47.4 (32.1–65.7)	31.9 (21.5–49.5)	117.1 (73.1–162.6)	51.9 (30.8–74.7)	23.0 (15.2–34.5)	10.3 (4.5–19.1)	33.4 (27.8–39.2)
Cameroon	Rate: Cumulative change (%)	-32.4 (-39.5 to -24.7)	-7.7 (-46.9 to 43.9)	-35.3 (-56.2 to -7.6)	-1.7 (-48.6 to 66.8)	-63.2 (-77.8 to -41.3)	-1.7 (-30.1 to 36.3)	-1.4 (-39.0 to 60.2)	5.0 (-41.2 to 67.9)	-25.9 (-60.1 to 24.9)	-22.8 (-74.7 to 61.1)	454.8 (186.3–787.5)
Cape Verde	Number: 1990	901 (872–932)	96 (69–120)	92 (70–115)	56 (34–83)	174 (125–215)	76 (58–98)	0 (0–0)	55 (28–83)	29 (16–48)	7 (3–15)	3 (2–5)
Cape Verde	Number: 2015	406 (376–437)	51 (38–66)	36 (29–44)	43 (29–58)	10 (7–15)	46 (37–60)	0 (0–0)	45 (27–64)	7 (5–10)	7 (3–13)	4 (3–6)
Cape Verde	Number: Cumulative change (%)	-54.9 (-58.5 to -51.2)	-45.0 (-62.8 to -21.4)	-59.5 (-70.8 to -45.7)	-18.2 (-53.0 to 40.7)	-93.6 (-95.9 to -90.4)	-36.8 (-55.0 to -11.3)	121.9 (15.9–290.6)	-10.1 (-55.4 to 59.8)	-70.8 (-87.1 to -48.7)	17.7 (-62.8 to 165.7)	35.6 (-5.0 to 92.9)
Cape Verde	Rate: 1990	380.4 (368.4–393.2)	38.3 (27.6–48.0)	38.1 (29.3–47.7)	22.4 (13.8–33.0)	71.4 (51.4–88.3)	31.1 (23.9–40.0)	0.0 (0.0–0.0)	22.0 (11.4–33.2)	12.8 (7.4–20.5)	3.1 (1.3–6.0)	1.5 (1.0–2.2)
Cape Verde	Rate: 2015	198.9 (184.3–214.2)	25.8 (19.0–33.2)	17.9 (14.4–21.9)	21.5 (14.8–28.9)	5.3 (3.7–7.5)	23.2 (18.7–29.7)	0.0 (0.0–0.0)	22.5 (13.5–31.9)	3.7 (2.5–5.0)	3.7 (1.9–6.6)	2.3 (1.6–3.2)
Cape Verde	Rate: Cumulative change (%)	-47.7 (-51.8 to -43.5)	-31.0 (-53.3 to -1.4)	-51.9 (-65.2 to -35.8)	2.5 (-41.0 to 76.2)	-92.3 (-95.0 to -88.5)	-23.4 (-45.5 to 7.6)	79.9 (-6.0 to 219.4)	12.7 (-44.0 to 100.3)	-67.8 (-85.3 to -44.6)	47.3 (-53.4 to 232.8)	54.0 (8.2–120.7)
Chad	Number: 1990	63,982 (62,306–65,753)	2,736 (1,803–3,725)	9,548 (7,224–11,843)	2,052 (1,081–3,217)	12,239 (8,829–15,760)	1,496 (830–2,957)	5,365 (2,142–10,607)	2,738 (1,441–4,142)	2,497 (1,491–3,925)	1,255 (416–2,454)	142 (76–235)
Chad	Number: 2015	90,220 (82,852–99,065)	5,116 (3,465–7,174)	14,367 (10,908–18,307)	4,073 (2,421–6,105)	16,762 (12,092–21,782)	3,369 (1,941–6,214)	8,634 (2,813–17,266)	5,705 (3,442–8,266)	4,646 (2,866–7,138)	1,466 (667–2,714)	1,856 (1,354–2,436)
Chad	Number: Cumulative change (%)	41.0 (28.8–55.5)	94.0 (22.2–195.4)	53.3 (9.8–106.3)	115.3 (13.7–278.9)	40.4 (-7.3 to 99.7)	131.6 (64.7–215.7)	66.1 (-17.4 to 185.2)	127.2 (20.7–306.2)	99.7 (2.0–243.2)	44.2 (-50.9 to 198.3)	1,346.4 (663.4–2,280.7)
Chad	Rate: 1990	1,390.5 (1,347.9–1,431.9)	50.0 (33.1–68.0)	200.4 (151.1–251.6)	37.5 (19.8–58.8)	265.2 (191.0–343.1)	30.2 (16.6–60.3)	123.5 (50.6–241.5)	50.2 (26.4–75.7)	55.0 (33.0–84.9)	23.1 (7.7–45.2)	3.2 (1.8–5.2)
Chad	Rate: 2015	886.7 (812.5–975.0)	45.0 (30.4–63.0)	138.2 (105.0–176.7)	35.8 (21.3–53.5)	163.6 (118.1–212.1)	31.8 (18.3–58.8)	87.2 (29.0–173.4)	50.2 (30.4–72.7)	46.4 (29.1–70.6)	13.0 (5.9–23.9)	21.5 (16.0–28.0)
Chad	Rate: Cumulative change (%)	-36.2 (-41.8 to -29.4)	-6.8 (-41.2 to 41.4)	-29.7 (-49.8 to -5.3)	3.5 (-45.1 to 81.8)	-36.8 (-58.1 to -9.7)	8.5 (-22.5 to 47.4)	-27.3 (-63.9 to 24.6)	9.0 (-42.1 to 93.9)	-9.6 (-52.9 to 55.3)	-31.0 (-76.4 to 41.9)	644.7 (300.3–1,111.5)
Cote d'Ivoire	Number: 1990	84,666 (82,611–86,904)	5,507 (3,681–7,580)	10,265 (7,401–12,997)	4,478 (2,565–6,759)	9,823 (6,874–12,696)	2,994 (1,766–5,298)	18,684 (13,074–24,010)	5,868 (3,328–8,615)	2,402 (1,530–3,639)	1,858 (890–3,301)	680 (457–969)
Cote d'Ivoire	Number: 2015	90,624 (83,453–98,565)	7,038 (4,685–9,624)	10,301 (8,023–12,992)	6,127 (3,585–8,859)	6,591 (4,446–9,035)	4,185 (2,789–7,026)	22,514 (17,242–27,837)	8,380 (5,087–12,234)	2,638 (1,804–3,995)	1,710 (781–3,170)	3,848 (3,173–4,491)
Cote d'Ivoire	Number: Cumulative change (%)	7.1 (-1.5 to 16.6)	32.7 (-17.4 to 100.2)	3.1 (-26.4 to 48.2)	47.2 (-26.8 to 149.5)	-30.5 (-55.7 to 3.4)	45.2 (4.5–94.9)	25.0 (-9.1 to 72.7)	52.8 (-18.8 to 165.4)	16.8 (-35.8 to 97.7)	4.9 (-61.9 to 125.0)	490.5 (300.1–713.5)
Cote d'Ivoire	Rate: 1990	1,013.0 (986.6–1,040.0)	58.5 (39.1–80.3)	119.3 (86.4–150.1)	47.5 (27.3–71.7)	116.9 (82.0–151.6)	34.0 (19.9–60.1)	233.1 (163.5–298.1)	62.5 (35.6–91.5)	29.8 (19.6–44.1)	19.9 (9.6–35.2)	8.3 (5.6–11.8)
Cote d'Ivoire	Rate: 2015	647.1 (595.7–703.6)	46.4 (30.9–63.5)	72.1 (56.5–90.6)	40.4 (23.7–58.4)	46.6 (31.8–63.5)	28.8 (19.2–48.7)	164.1 (125.6–202.5)	55.4 (33.6–80.8)	19.4 (13.5–28.8)	11.3 (5.2–20.9)	30.8 (25.5–35.9)
Cote d'Ivoire	Rate: Cumulative change (%)	-36.1 (-41.1 to -30.3)	-17.5 (-48.6 to 24.5)	-37.9 (-55.5 to -11.0)	-8.6 (-54.5 to 55.1)	-58.7 (-73.5 to -39.1)	-11.7 (-36.7 to 18.4)	-27.0 (-46.6 to 0.4)	-5.3 (-49.4 to 64.1)	-31.2 (-60.7 to 14.0)	-35.1 (-76.2 to 39.2)	289.2 (163.3–433.1)
The Gambia	Number: 1990	5,165 (4,942–5,393)	369 (249–506)	885 (728–1,042)	249 (144–376)	509 (387–641)	204 (141–304)	491 (291–745)	422 (240–619)	216 (154–290)	128 (60–234)	3 (1–4)
The Gambia	Number: 2015	4,366 (4,043–4,715)	397 (252–563)	521 (419–634)	341 (198–515)	421 (307–555)	307 (234–390)	63 (22–126)	550 (331–775)	213 (143–313)	117 (40–233)	152 (107–204)
The Gambia	Number: Cumulative change (%)	-15.4 (-22.5 to -7.8)	11.6 (-34.4 to 76.4)	-40.4 (-55.3 to -22.5)	48.0 (-27.0 to 159.2)	-15.2 (-44.1 to 21.0)	56.7 (5.4–124.0)	-86.7 (-94.8 to -73.7)	38.8 (-24.9 to 132.7)	1.1 (-36.7 to 56.4)	5.3 (-67.6 to 143.9)	5,211.2 (3,234.6–7,997.5)
The Gambia	Rate: 1990	754.1 (717.5–788.5)	47.5 (32.1–65.0)	125.9 (103.5–148.6)	32.1 (18.8–48.4)	74.8 (57.3–94.2)	28.4 (19.6–42.4)	74.3 (44.9–112.1)	54.4 (31.0–79.6)	33.4 (24.4–44.0)	16.6 (7.8–30.2)	0.4 (0.3–0.7)
The Gambia	Rate: 2015	315.4 (293.0–340.0)	26.3 (16.7–37.3)	37.2 (30.1–44.9)	22.6 (13.2–34.0)	29.7 (21.8–38.9)	21.4 (16.3–27.0)	4.7 (1.8–9.2)	36.4 (22.0–51.3)	16.2 (11.2–23.4)	7.8 (2.7–15.5)	12.4 (8.9–16.4)
The Gambia	Rate: Cumulative change (%)	-58.1 (-61.7 to -54.2)	-42.6 (-66.2 to -9.2)	-70.1 (-77.4 to -61.4)	-23.9 (-62.3 to 33.1)	-59.5 (-73.1 to -42.2)	-21.7 (-47.5 to 12.2)	-93.4 (-97.4 to -87.3)	-28.7 (-61.4 to 18.8)	-50.3 (-67.8 to -25.4)	-46.1 (-83.5 to 24.3)	2,862.8 (1,785.1–4,363.9)
Ghana	Number: 1990	77,930 (76,107–79,895)	6,126 (4,360–8,106)	7,744 (6,198–9,461)	4,406 (2,713–6,519)	10,437 (8,095–12,975)	2,430 (1,840–3,236)	10,413 (7,453–13,414)	5,885 (3,287–9,004)	2,261 (1,651–3,069)	1,581 (782–2,775)	312 (203–457)
Ghana	Number: 2015	58,562 (54,034–64,305)	4,470 (2,802–6,337)	5,481 (4,225–6,875)	3,788 (2,197–5,657)	1,907 (1,267–2,662)	3,719 (2,619–4,890)	9,084 (5,217–13,514)	8,649 (5,617–11,678)	2,214 (1,595–3,234)	2,239 (1,060–4,094)	1,764 (1,424–2,127)
Ghana	Number: Cumulative change (%)	-24.8 (-31.0 to -17.7)	-24.6 (-57.1 to 15.6)	-28.0 (-48.9 to -0.6)	-8.0 (-54.9 to 61.6)	-81.3 (-88.7 to -71.8)	57.1 (-2.6 to 115.9)	-11.6 (-46.5 to 30.7)	59.2 (-7.8 to 176.2)	1.3 (-37.3 to 57.1)	64.1 (-41.5 to 249.3)	492.2 (295.4–742.5)
Ghana	Rate: 1990	827.6 (806.4–849.3)	59.4 (42.3–78.6)	81.5 (65.1–99.9)	42.7 (26.3–63.2)	110.4 (86.3–137.5)	24.8 (18.8–32.9)	113.6 (81.7–145.8)	57.2 (32.0–87.4)	25.6 (19.1–34.2)	15.4 (7.6–27.0)	3.3 (2.2–4.9)
Ghana	Rate: 2015	386.8 (356.7–424.3)	27.8 (17.4–39.4)	36.0 (27.8–45.1)	23.6 (13.7–35.2)	12.5 (8.4–17.4)	23.8 (16.8–31.4)	60.1 (34.7–88.9)	53.9 (35.0–72.7)	15.4 (11.2–21.9)	14.0 (6.6–25.5)	13.1 (10.7–15.7)
Ghana	Rate: Cumulative change (%)	-53.3 (-57.1 to -48.8)	-51.6 (-72.4 to -25.8)	-55.2 (-67.8 to -38.5)	-41.0 (-71.0 to 3.6)	-88.5 (-92.9 to -82.6)	-1.3 (-38.8 to 35.1)	-46.4 (-67.4 to -21.4)	2.0 (-40.8 to 77.3)	-38.0 (-60.9 to -5.1)	4.8 (-62.5 to 122.7)	312.5 (177.5–482.4)
Guinea	Number: 1990	68,336 (66,580–70,261)	3,376 (2,229–4,628)	12,318 (9,225–15,438)	2,843 (1,677–4,258)	7,515 (5,096–10,288)	1,857 (924–3,960)	11,037 (5,967–16,603)	3,147 (1,777–4,835)	3,026 (1,819–4,634)	1,087 (508–1,982)	84 (35–153)
Guinea	Number: 2015	56,663 (51,878–61,436)	3,596 (2,447–5,008)	7,668 (5,991–9,498)	3,459 (2,130–4,987)	3,411 (2,435–4,637)	2,117 (1,254–3,697)	14,503 (11,105–18,032)	3,664 (2,056–5,380)	2,260 (1,525–3,251)	780 (346–1,450)	722 (583–878)
Guinea	Number: Cumulative change (%)	-17.1 (-24.0 to -9.6)	11.0 (-31.5 to 71.8)	-36.0 (-55.4 to -10.2)	31.0 (-32.7 to 125.7)	-52.5 (-71.3 to -25.1)	24.0 (-20.7 to 82.9)	42.4 (-13.0 to 131.4)	24.7 (-35.5 to 112.4)	-19.0 (-57.2 to 43.1)	-16.2 (-73.1 to 75.4)	935.5 (373.0–1,835.3)
Guinea	Rate: 1990	1,599.6 (1,557.0–1,645.2)	65.5 (43.3–89.7)	281.2 (206.6–355.3)	55.1 (32.6–82.5)	178.0 (120.6–243.2)	40.4 (19.8–88.0)	276.8 (152.8–415.6)	61.3 (34.6–93.9)	72.3 (43.1–110.5)	21.3 (10.0–38.6)	2.0 (0.9–3.7)
Guinea	Rate: 2015	738.6 (676.7–801.6)	43.3 (29.5–60.2)	98.1 (77.1–121.3)	41.6 (25.7–59.9)	44.5 (31.9–59.9)	26.7 (15.9–46.7)	192.3 (148.2–238.9)	44.2 (24.8–64.7)	30.1 (20.7–43.0)	9.4 (4.2–17.4)	10.8 (8.8–13.1)
Guinea	Rate: Cumulative change (%)	-53.8 (-57.6 to -49.7)	-31.2 (-57.5 to 6.6)	-64.0 (-75.1 to -49.1)	-18.7 (-58.2 to 40.4)	-73.8 (-83.9 to -58.5)	-27.5 (-54.8 to 7.4)	-24.9 (-53.9 to 22.6)	-22.9 (-60.0 to 31.0)	-55.0 (-76.0 to -22.2)	-48.5 (-83.5 to 7.4)	543.9 (198.4–1,089.9)
Guinea-Bissau	Number: 1990	10,713 (10,163–11,275)	534 (362–727)	1,430 (1,093–1,798)	419 (254–617)	2,000 (1,500–2,564)	290 (152–583)	1,449 (726–2,353)	510 (297–763)	488 (303–719)	172 (80–321)	5 (3–9)
Guinea-Bissau	Number: 2015	9,500 (8,490–10,707)	608 (414–815)	1,249 (956–1,618)	498 (312–719)	1,275 (889–1,727)	424 (238–793)	542 (211–1,098)	646 (361–955)	589 (355–914)	144 (68–262)	274 (225–328)
Guinea-Bissau	Number: Cumulative change (%)	-11.3 (-20.9 to 0.2)	18.0 (-24.1 to 75.4)	-10.9 (-38.3 to 24.6)	26.3 (-31.5 to 107.0)	-34.9 (-57.7 to -7.4)	52.9 (4.9–113.5)	-58.6 (-86.4 to -10.4)	35.0 (-29.9 to 132.5)	28.7 (-35.9 to 128.6)	-1.3 (-67.6 to 106.9)	5,528.3 (3,037.0–8,555.1)
Guinea-Bissau	Rate: 1990	1,441.6 (1,356.9–1,532.4)	62.0 (42.1–84.4)	188.6 (144.2–237.4)	48.6 (29.6–71.6)	269.9 (201.3–345.1)	36.9 (19.3–75.0)	204.6 (105.1–328.5)	59.4 (34.7–88.8)	67.4 (42.6–98.4)	20.2 (9.5–37.5)	0.7 (0.4–1.3)
Guinea-Bissau	Rate: 2015	853.8 (758.6–973.2)	49.8 (34.0–66.8)	111.1 (85.1–143.9)	40.8 (25.6–58.9)	113.6 (78.8–154.8)	36.9 (20.7–69.2)	50.1 (20.0–100.6)	53.1 (29.8–78.4)	53.8 (33.0–81.9)	11.9 (5.6–21.6)	28.3 (23.4–33.7)
Guinea-Bissau	Rate: Cumulative change (%)	-40.7 (-47.6 to -32.3)	-16.8 (-46.3 to 23.7)	-39.8 (-58.6 to -16.3)	-10.9 (-51.5 to 45.7)	-56.9 (-72.2 to -38.9)	5.0 (-28.3 to 46.3)	-73.1 (-91.1 to -42.0)	-4.9 (-50.4 to 62.3)	-14.8 (-57.2 to 48.0)	-30.7 (-77.0 to 44.9)	4,144.3 (2,292.6–6,379.2)
Liberia	Number: 1990	24,621 (23,385–25,919)	1,178 (818–1,566)	3,696 (2,736–4,602)	959 (595–1,392)	4,159 (2,925–5,339)	693 (362–1,481)	3,402 (1,747–5,237)	1,160 (668–1,764)	1,077 (684–1,652)	384 (189–671)	34 (19–55)
Liberia	Number: 2015	13,224 (12,313–14,259)	952 (616–1,346)	1,672 (1,279–2,099)	929 (591–1,359)	1,363 (942–1,833)	685 (458–1,026)	1,720 (934–2,566)	1,085 (659–1,581)	621 (384–1,046)	218 (91–412)	303 (250–363)
Liberia	Number: Cumulative change (%)	-46.2 (-50.6 to -41.5)	-16.3 (-50.0 to 31.1)	-53.6 (-67.8 to -34.0)	2.6 (-42.9 to 71.1)	-66.1 (-78.9 to -50.3)	12.1 (-40.4 to 76.0)	-44.5 (-74.4 to -0.9)	0.1 (-47.6 to 68.4)	-39.0 (-68.1 to 7.7)	-34.0 (-79.5 to 46.7)	858.2 (470.6–1,447.8)
Liberia	Rate: 1990	1,620.9 (1,517.3–1,739.3)	68.9 (47.9–91.4)	234.5 (173.5–293.0)	56.1 (34.9–81.3)	266.8 (187.8–341.6)	43.2 (22.6–92.1)	226.7 (117.2–347.0)	68.1 (39.5–103.3)	71.0 (45.6–106.7)	22.7 (11.2–39.7)	2.3 (1.3–3.7)
Liberia	Rate: 2015	495.2 (460.5–533.8)	33.5 (21.7–47.4)	61.5 (47.2–76.9)	32.7 (20.8–47.8)	50.4 (35.2–67.4)	24.9 (16.6–37.2)	64.9 (35.7–96.7)	38.3 (23.3–55.7)	23.6 (14.9–38.8)	7.7 (3.2–14.6)	12.8 (10.6–15.5)
Liberia	Rate: Cumulative change (%)	-69.4 (-72.4 to -66.4)	-49.7 (-69.9 to -21.3)	-73.1 (-81.3 to -61.9)	-38.3 (-65.6 to 2.8)	-80.4 (-87.8 to -71.4)	-34.6 (-65.5 to 3.2)	-68.7 (-85.4 to -44.5)	-39.9 (-68.4 to 0.9)	-65.0 (-81.3 to -39.3)	-60.6 (-87.7 to -12.2)	517.1 (271.6–888.9)
Mali	Number: 1990	101,705 (99,563–103,983)	6,068 (4,131–8,147)	8,321 (6,264–10,696)	4,402 (2,560–6,614)	13,391 (9,837–17,556)	2,743 (1,358–5,890)	25,216 (16,299–34,603)	5,364 (2,997–8,291)	4,449 (2,835–6,511)	1,983 (863–3,742)	153 (72–256)
Mali	Number: 2015	108,131 (98,692–118,868)	7,886 (5,277–11,115)	6,627 (4,963–8,637)	6,214 (3,581–9,282)	7,878 (5,500–10,653)	3,852 (2,199–7,381)	34,083 (26,755–41,387)	7,577 (4,279–11,331)	4,444 (2,895–6,598)	1,656 (770–3,034)	1,466 (1,187–1,758)
Mali	Number: Cumulative change (%)	6.3 (-3.2 to 17.0)	34.0 (-12.5 to 98.6)	-18.7 (-43.7 to 12.1)	50.2 (-21.7 to 154.7)	-39.5 (-61.4 to -12.6)	48.7 (5.1–105.7)	40.3 (1.2–94.5)	51.7 (-23.1 to 169.3)	4.5 (-41.7 to 65.4)	-2.4 (-67.1 to 100.5)	996.5 (472.7–1,838.7)
Mali	Rate: 1990	1,621.2 (1,583.5–1,662.1)	81.6 (55.6–109.6)	128.8 (96.3–165.5)	59.2 (34.8–88.7)	211.9 (155.7–281.3)	40.9 (19.9–89.0)	423.8 (280.1–576.9)	72.4 (40.7–111.8)	72.3 (47.0–103.9)	26.9 (11.9–50.8)	2.5 (1.2–4.2)
Mali	Rate: 2015	873.0 (795.5–961.1)	57.8 (38.7–81.4)	52.4 (39.4–68.3)	45.5 (26.2–67.9)	63.0 (44.4–84.6)	29.9 (17.0–57.3)	280.2 (219.9–341.0)	55.6 (31.5–83.1)	37.0 (24.7–54.0)	12.2 (5.7–22.3)	13.6 (11.1–16.2)
Mali	Rate: Cumulative change (%)	-46.1 (-51.1 to -40.8)	-27.1 (-52.2 to 8.2)	-58.3 (-71.4 to -42.6)	-18.2 (-57.3 to 38.4)	-69.4 (-80.5 to -55.6)	-22.0 (-45.1 to 8.6)	-31.5 (-50.2 to -5.7)	-17.6 (-58.2 to 45.8)	-46.6 (-69.6 to -17.2)	-47.2 (-82.0 to 8.4)	518.9 (226.8–983.4)
Mauritania	Number: 1990	10,017 (9,700–10,328)	698 (451–970)	1,195 (676–1,606)	563 (328–852)	1,367 (631–1,959)	398 (234–629)	183 (51–401)	803 (453–1,173)	298 (151–482)	257 (114–475)	12 (0–63)
Mauritania	Number: 2015	8,496 (7,837–9,255)	837 (559–1,147)	1,007 (797–1,215)	764 (471–1,103)	779 (554–1,045)	644 (477–886)	257 (93–505)	1,031 (627–1,521)	311 (212–466)	204 (95–402)	76 (12–301)
Mauritania	Number: Cumulative change (%)	-15.2 (-22.1 to -6.7)	25.7 (-26.0 to 101.5)	-10.0 (-41.4 to 48.0)	45.4 (-22.9 to 147.8)	-35.6 (-65.4 to 24.0)	71.4 (14.4–155.1)	75.0 (-40.1 to 286.6)	37.3 (-25.1 to 129.7)	16.5 (-42.3 to 106.0)	-6.4 (-68.4 to 108.6)	1,864.9 (161.0–4,602.6)
Mauritania	Rate: 1990	740.5 (716.0–764.3)	46.8 (30.3–65.1)	86.7 (48.1–117.9)	37.8 (22.0–57.1)	101.3 (46.4–144.9)	28.2 (16.3–44.9)	14.1 (4.2–30.7)	54.0 (30.6–78.8)	23.1 (11.8–36.8)	17.3 (7.7–32.0)	0.9 (0.0–4.9)
Mauritania	Rate: 2015	364.6 (336.9–397.8)	34.3 (22.9–47.0)	43.1 (34.1–51.8)	31.3 (19.4–45.2)	33.4 (24.0–44.6)	27.2 (20.1–37.4)	11.2 (4.2–21.9)	42.3 (25.8–62.4)	13.8 (9.5–20.4)	8.4 (4.0–16.5)	3.9 (0.6–15.3)
Mauritania	Rate: Cumulative change (%)	-50.7 (-54.8 to -45.8)	-23.2 (-54.7 to 23.0)	-46.8 (-65.4 to -11.6)	-11.2 (-52.8 to 50.6)	-62.7 (-80.0 to -28.2)	2.2 (-32.1 to 53.6)	-1.8 (-66.1 to 115.3)	-16.3 (-54.1 to 39.9)	-33.6 (-66.0 to 15.3)	-43.0 (-80.6 to 26.7)	1,193.0 (78.6–3,002.1)
Niger	Number: 1990	132,348 (129,747–135,282)	3,983 (2,686–5,567)	20,675 (15,306–26,490)	2,960 (1,584–4,507)	29,974 (22,171–38,246)	2,441 (1,129–5,665)	17,531 (6,805–31,800)	3,843 (2,134–5,696)	10,294 (5,573–17,223)	1,685 (677–3,190)	117 (79–166)
Niger	Number: 2015	117,898 (108,729–128,400)	5,310 (3,345–7,771)	15,071 (11,566–19,230)	4,653 (2,924–6,526)	20,964 (15,489–27,721)	3,817 (2,239–7,039)	25,275 (15,013–35,791)	5,130 (3,027–7,273)	9,515 (5,808–14,797)	1,329 (468–2,432)	905 (768–1,056)
Niger	Number: Cumulative change (%)	-10.9 (-17.8 to -2.9)	38.0 (-15.3 to 121.6)	-25.3 (-49.1 to 3.7)	68.2 (-4.3 to 169.6)	-28.3 (-52.1 to 1.7)	78.3 (6.4–164.3)	65.2 (-13.3 to 197.1)	42.7 (-19.9 to 136.9)	2.2 (-49.4 to 73.9)	-7.2 (-68.8 to 107.0)	704.9 (451.5–1,031.7)
Niger	Rate: 1990	2,156.7 (2,105.3–2,211.9)	50.7 (34.3–70.7)	329.4 (240.8–428.9)	37.6 (20.1–57.1)	491.5 (361.7–630.8)	36.1 (15.8–85.6)	301.9 (119.0–545.2)	49.0 (27.6–72.3)	168.1 (89.5–283.9)	21.7 (8.7–40.6)	2.0 (1.3–2.8)
Niger	Rate: 2015	763.2 (704.5–829.7)	29.8 (18.7–43.6)	95.2 (73.5–122.0)	26.1 (16.4–36.6)	133.7 (99.3–176.9)	23.3 (13.6–43.1)	166.0 (100.1–234.6)	28.8 (17.0–40.7)	61.6 (38.4–95.4)	7.5 (2.6–13.6)	7.8 (6.6–9.2)
Niger	Rate: Cumulative change (%)	-64.6 (-67.4 to -61.5)	-39.2 (-62.7 to -2.1)	-70.3 (-79.9 to -58.5)	-25.8 (-57.5 to 19.1)	-72.1 (-81.5 to -60.3)	-25.0 (-56.9 to 12.8)	-37.4 (-66.6 to 11.0)	-37.2 (-64.9 to 3.9)	-59.2 (-80.1 to -30.0)	-59.4 (-86.1 to -8.9)	316.1 (182.8–486.3)
Nigeria	Number: 1990	932,600 (908,736–957,337)	38,660 (25,644–54,336)	102,246 (77,167–130,462)	43,596 (27,343–61,598)	102,580 (72,153–138,118)	26,662 (14,875–53,486)	211,450 (141,702–284,848)	49,473 (27,891–73,664)	35,409 (23,604–53,048)	16,673 (7,995–29,117)	2,511 (1,081–4,749)
Nigeria	Number: 2015	819,035 (748,194–894,404)	39,839 (25,561–57,272)	69,769 (55,378–88,745)	56,403 (34,647–82,330)	109,381 (83,645–139,221)	34,074 (21,233–56,890)	172,203 (124,073–220,703)	76,019 (44,170–107,827)	30,327 (19,844–49,171)	15,309 (6,655–28,550)	29,010 (25,198–32,979)
Nigeria	Number: Cumulative change (%)	-12.2 (-19.8 to -3.9)	7.0 (-39.1 to 61.8)	-30.3 (-49.9 to -6.3)	35.6 (-24.5 to 117.7)	10.8 (-26.7 to 60.0)	36.2 (-7.2 to 85.9)	-16.0 (-42.1 to 18.2)	64.7 (-12.9 to 178.3)	-9.9 (-51.0 to 52.5)	5.6 (-65.6 to 118.9)	1,296.2 (522.1–2,543.8)
Nigeria	Rate: 1990	1,418.2 (1,374.5–1,461.1)	51.0 (33.8–71.7)	152.6 (115.2–195.9)	57.5 (36.2–81.2)	157.0 (109.8–211.2)	38.1 (20.9–77.1)	333.0 (224.1–446.1)	65.5 (37.0–97.3)	54.5 (36.1–81.0)	22.2 (10.6–38.5)	3.9 (1.7–7.3)
Nigeria	Rate: 2015	687.8 (628.6–750.7)	30.8 (19.8–44.3)	58.7 (46.9–74.8)	43.6 (26.8–63.7)	91.2 (70.0–115.8)	27.7 (17.3–46.3)	146.3 (105.7–187.4)	58.9 (34.4–83.5)	26.0 (17.3–41.1)	11.9 (5.2–22.2)	27.2 (23.7–30.9)

© 2017 Global Burden of Disease Child and Adolescent Health Collaboration. JAMA Pediatrics.

eTable 1. Number of Deaths, Death Rates (per 100,000 population), and Cumulative Percent Change with 95% Uncertainty Intervals (UI) for the Top 10 Global Causes of Death in 195 Countries and Territories, Aged 0 to 19, Both Sexes, 1990 and 2015 *Note: best viewed by enlarging in browser.*

Location	Measure	All causes	Neonatal preterm birth complications	Lower respiratory infections	Neonatal encephalopathy due to birth asphyxia and trauma	Diarrheal diseases	Congenital anomalies	Malaria	Neonatal sepsis and other neonatal infections	Meningitis	Other neonatal disorders	HIV/AIDS
Nigeria	Rate: Cumulative change (%)	-51.5 (-55.6 to -47.1)	-37.2 (-64.3 to -5.3)	-60.7 (-71.7 to -46.7)	-20.5 (-55.7 to 27.5)	-39.6 (-60.1 to -13.1)	-21.9 (-46.9 to 6.8)	-54.7 (-68.8 to -36.5)	-3.6 (-48.9 to 62.7)	-49.9 (-72.2 to -17.0)	-38.6 (-79.8 to 26.3)	746.9 (282.5–1,501.2)
Sao Tome and Principe	Number: 1990	532 (514–551)	27 (17–38)	105 (75–128)	26 (16–38)	81 (56–104)	26 (18–39)	0 (0–2)	33 (19–48)	9 (5–14)	9 (4–16)	0 (0–0)
Sao Tome and Principe	Number: 2015	297 (267–331)	21 (13–30)	42 (32–53)	25 (15–36)	21 (14–29)	28 (22–35)	0 (0–0)	31 (18–44)	5 (3–8)	5 (2–11)	0 (0–0)
Sao Tome and Principe	Number: Cumulative change (%)	-44.1 (-49.8 to -37.7)	-18.8 (-51.8 to 31.1)	-58.5 (-71.5 to -42.4)	0.9 (-45.7 to 65.7)	-72.7 (-83.7 to -57.8)	9.9 (-28.1 to 60.2)	-53.1 (-94.2 to 29.6)	1.5 (-45.6 to 68.5)	-42.5 (-71.3 to 3.3)	-28.7 (-76.1 to 58.6)	807.8 (302.7–1,492.7)
Sao Tome and Principe	Rate: 1990	703.4 (678.2–728.2)	33.4 (21.6–46.9)	137.4 (99.3–168.8)	32.7 (20.4–46.7)	106.2 (73.2–136.8)	34.4 (23.2–51.2)	1.2 (0.0–3.5)	40.7 (23.7–59.1)	12.9 (7.6–19.1)	11.5 (5.4–20.7)	0.0 (0.0–0.0)
Sao Tome and Principe	Rate: 2015	266.9 (239.5–296.7)	18.2 (11.6–26.0)	37.7 (29.4–47.1)	21.8 (13.4–31.7)	18.6 (13.1–25.7)	24.5 (19.3–30.7)	0.2 (0.0–0.8)	27.1 (16.1–38.3)	4.7 (3.0–7.2)	4.9 (2.0–9.6)	0.0 (0.0–0.0)
Sao Tome and Principe	Rate: Cumulative change (%)	-62.0 (-66.0 to -57.7)	-43.1 (-66.2 to -8.1)	-71.8 (-80.5 to -61.1)	-29.3 (-61.9 to 16.1)	-81.7 (-89.0 to -71.9)	-25.1 (-51.4 to 9.7)	-67.5 (-95.9 to -13.0)	-29.1 (-61.9 to 17.0)	-60.9 (-80.2 to -31.0)	-50.3 (-83.3 to 10.3)	518.9 (177.7–982.7)
Senegal	Number: 1990	51,415 (50,478–52,462)	2,730 (1,805–3,764)	5,977 (4,816–7,237)	2,217 (1,273–3,327)	9,908 (7,802–11,944)	1,740 (1,051–3,018)	8,524 (5,582–11,921)	3,014 (1,782–4,491)	2,152 (1,528–2,996)	945 (443–1,659)	36 (26–49)
Senegal	Number: 2015	34,572 (32,407–36,920)	2,752 (1,741–3,908)	4,028 (3,274–4,838)	2,622 (1,666–3,770)	4,656 (3,537–5,958)	2,440 (1,771–3,393)	1,510 (718–2,517)	3,294 (2,114–4,633)	1,943 (1,295–2,891)	666 (270–1,274)	448 (363–549)
Senegal	Number: Cumulative change (%)	-32.8 (-37.1 to -28.0)	4.0 (-35.7 to 60.8)	-31.5 (-49.1 to -9.9)	25.8 (-28.0 to 99.9)	-52.3 (-66.6 to -35.3)	50.7 (-4.0 to 120.8)	-81.8 (-90.9 to -69.0)	15.8 (-34.3 to 86.2)	-6.7 (-43.7 to 51.0)	-18.8 (-71.9 to 72.5)	1,139.3 (844.2–1,477.8)
Senegal	Rate: 1990	974.6 (954.2–994.7)	46.1 (30.6–63.7)	111.4 (89.5–134.8)	37.5 (21.6–56.2)	188.6 (148.5–227.7)	31.5 (19.0–55.3)	166.2 (109.4–231.6)	51.0 (30.2–76.0)	42.0 (30.3–58.0)	16.1 (7.5–28.1)	0.7 (0.5–0.9)
Senegal	Rate: 2015	361.8 (337.3–387.8)	26.6 (16.8–37.8)	41.4 (33.8–49.8)	25.4 (16.1–36.4)	48.0 (36.6–61.3)	24.6 (17.9–34.1)	16.2 (7.9–26.5)	31.9 (20.5–44.8)	21.1 (14.3–30.9)	6.5 (2.6–12.3)	5.2 (4.2–6.4)
Senegal	Rate: Cumulative change (%)	-62.9 (-65.4 to -60.1)	-40.4 (-63.2 to -7.8)	-62.2 (-71.9 to -50.5)	-28.0 (-58.7 to 14.5)	-74.1 (-81.7 to -65.0)	-15.9 (-47.0 to 23.4)	-90.0 (-94.9 to -83.3)	-33.8 (-62.4 to 6.2)	-48.1 (-68.1 to -17.4)	-53.7 (-84.0 to -2.0)	652.4 (478.0–853.7)
Sierra Leone	Number: 1990	46,196 (45,030–47,369)	2,356 (1,605–3,151)	7,362 (5,597–9,235)	2,037 (1,327–2,922)	6,061 (4,260–8,174)	1,371 (657–3,114)	9,304 (4,717–14,508)	2,025 (1,115–3,118)	2,155 (1,384–3,338)	711 (335–1,238)	21 (6–42)
Sierra Leone	Number: 2015	32,557 (30,666–34,582)	1,901 (1,276–2,578)	3,957 (2,962–5,123)	1,900 (1,189–2,698)	2,410 (1,606–3,423)	1,307 (692–2,513)	7,463 (4,893–9,877)	2,001 (1,119–3,063)	1,516 (983–2,388)	422 (194–785)	349 (285–419)
Sierra Leone	Number: Cumulative change (%)	-29.5 (-34.0 to -24.9)	-16.2 (-49.1 to 28.9)	-45.2 (-61.3 to -24.3)	-2.1 (-46.5 to 53.9)	-58.9 (-75.9 to -37.2)	4.3 (-33.4 to 55.3)	-12.5 (-48.0 to 40.4)	6.5 (-48.5 to 90.8)	-25.3 (-60.2 to 26.0)	-31.2 (-76.0 to 46.3)	2,175.1 (737.8–4,933.5)
Sierra Leone	Rate: 1990	1,691.0 (1,644.4–1,736.0)	74.4 (50.9–99.5)	264.0 (200.3–331.3)	64.3 (41.9–92.0)	222.1 (156.2–297.7)	47.5 (22.6–108.0)	359.5 (183.2–556.5)	64.2 (35.5–98.5)	80.7 (52.8–123.9)	22.8 (10.8–39.5)	0.8 (0.2–1.6)
Sierra Leone	Rate: 2015	843.7 (795.0–896.7)	45.9 (30.8–62.2)	101.0 (75.7–130.7)	45.9 (28.8–65.1)	62.0 (41.9–87.7)	32.9 (17.4–63.1)	195.5 (128.9–258.6)	48.4 (27.1–74.0)	39.9 (26.2–62.1)	10.3 (4.7–19.0)	10.0 (8.2–12.0)
Sierra Leone	Rate: Cumulative change (%)	-50.1 (-53.2 to -46.8)	-36.0 (-60.9 to -1.4)	-61.0 (-72.2 to -46.3)	-25.1 (-59.0 to 17.6)	-71.1 (-83.0 to -55.9)	-24.0 (-51.8 to 13.5)	-40.9 (-64.4 to -5.4)	-18.8 (-60.7 to 44.9)	-47.7 (-71.8 to -13.4)	-47.8 (-81.7 to 10.6)	1,627.6 (547.7–3,716.8)
Togo	Number: 1990	25,437 (24,764–26,127)	1,466 (985–1,983)	3,261 (2,626–3,995)	1,255 (764–1,870)	3,494 (2,616–4,480)	890 (544–1,543)	5,165 (3,754–6,568)	1,554 (873–2,300)	740 (504–1,154)	464 (215–853)	71 (49–96)
Togo	Number: 2015	23,648 (22,119–25,370)	1,736 (1,182–2,395)	2,491 (1,994–3,081)	1,688 (1,047–2,441)	1,737 (1,230–2,325)	1,111 (760–1,708)	5,350 (4,038–6,763)	2,028 (1,124–3,059)	649 (451–930)	403 (170–830)	980 (800–1,180)
Togo	Number: Cumulative change (%)	-7.0 (-13.6 to 0.6)	22.8 (-26.6 to 83.0)	-22.4 (-43.1 to 3.7)	43.8 (-24.5 to 139.6)	-49.0 (-66.7 to -27.2)	30.4 (-7.3 to 75.2)	5.9 (-23.2 to 43.9)	39.7 (-32.4 to 131.5)	-7.1 (-49.1 to 52.6)	1.9 (-67.4 to 131.7)	1,316.2 (911.3–1,804.9)
Togo	Rate: 1990	974.0 (946.9–1,000.3)	50.6 (34.0–68.3)	122.7 (99.0–150.6)	43.3 (26.4–64.5)	134.0 (100.3–171.4)	32.7 (20.0–57.0)	203.2 (147.8–258.0)	53.7 (30.2–79.3)	29.6 (20.7–45.2)	16.1 (7.5–29.6)	2.8 (1.9–3.7)
Togo	Rate: 2015	544.7 (508.5–584.4)	37.3 (25.4–51.5)	56.4 (45.4–69.5)	36.3 (22.5–52.5)	39.6 (28.3–53.0)	24.8 (17.0–38.0)	123.8 (93.5–156.5)	43.7 (24.2–65.9)	15.5 (11.0–21.9)	8.7 (3.7–17.9)	25.9 (21.2–31.1)
Togo	Rate: Cumulative change (%)	-44.1 (-48.1 to -39.5)	-23.5 (-54.2 to 14.0)	-53.3 (-65.7 to -37.6)	-10.4 (-52.9 to 49.1)	-69.7 (-80.1 to -57.1)	-20.8 (-43.7 to 6.8)	-37.7 (-54.8 to -16.2)	-13.0 (-57.9 to 44.1)	-44.9 (-69.1 to -12.5)	-36.8 (-79.7 to 43.9)	871.5 (592.0–1,199.6)
Eastern Sub-Saharan Africa	Number: 1990	1,612,637 (1,593,639–1,633,091)	80,419 (66,899–93,795)	222,288 (187,735–255,741)	72,023 (56,989–87,551)	214,691 (170,638–256,056)	53,005 (42,841–67,290)	249,176 (181,802–326,831)	48,757 (35,291–67,232)	58,781 (42,949–76,849)	45,654 (31,074–63,743)	23,340 (21,544–25,585)
Eastern Sub-Saharan Africa	Number: 2015	1,106,529 (1,073,687–1,142,605)	70,810 (60,850–80,898)	140,010 (125,526–155,760)	74,910 (63,630–87,232)	96,769 (85,115–110,049)	81,468 (55,376–98,349)	73,950 (44,425–105,555)	57,493 (43,328–75,528)	44,598 (36,498–54,432)	39,987 (28,955–52,229)	78,604 (74,196–83,119)
Eastern Sub-Saharan Africa	Number: Cumulative change (%)	-31.4 (-33.7 to -29.1)	-11.3 (-25.9 to 5.6)	-36.5 (-47.2 to -23.6)	5.3 (-15.1 to 31.0)	-54.3 (-64.0 to -42.3)	58.0 (-11.4 to 105.7)	-69.8 (-81.2 to -54.9)	20.3 (-11.8 to 57.2)	-21.9 (-43.5 to 8.8)	-9.3 (-39.5 to 29.7)	237.5 (210.9–264.8)
Eastern Sub-Saharan Africa	Rate: 1990	1,193.5 (1,175.6–1,213.5)	52.0 (43.3–60.6)	159.4 (133.7–183.8)	46.5 (36.8–56.5)	159.5 (126.7–190.6)	37.0 (29.8–47.0)	189.0 (138.8–245.4)	31.7 (23.0–43.6)	44.1 (32.4–57.8)	29.6 (20.2–41.4)	17.3 (16.0–19.0)
Eastern Sub-Saharan Africa	Rate: 2015	476.2 (462.2–491.7)	28.4 (24.4–32.5)	59.0 (53.0–65.7)	30.1 (25.5–35.0)	41.8 (36.9–47.4)	33.9 (23.0–40.9)	31.9 (19.4–45.3)	23.1 (17.4–30.4)	19.4 (16.0–23.6)	16.1 (11.6–21.0)	37.0 (34.9–39.1)
Eastern Sub-Saharan Africa	Rate: Cumulative change (%)	-60.1 (-61.4 to -58.7)	-44.9 (-53.9 to -34.4)	-62.6 (-69.0 to -54.9)	-34.6 (-47.2 to -18.6)	-73.4 (-79.0 to -66.5)	-5.7 (-47.7 to 23.7)	-82.8 (-89.1 to -74.8)	-25.6 (-45.4 to -2.7)	-54.7 (-67.1 to -37.1)	-43.9 (-62.5 to -19.8)	114.0 (97.2–131.7)
Burundi	Number: 1990	52,779 (49,838–55,603)	2,830 (2,015–3,673)	6,354 (4,830–8,131)	1,866 (1,127–2,754)	6,123 (4,411–8,097)	1,699 (1,188–2,388)	13,999 (9,019–18,829)	1,545 (854–2,541)	1,397 (817–2,130)	1,355 (692–2,258)	557 (310–1,006)
Burundi	Number: 2015	42,095 (34,158–51,944)	3,193 (2,008–4,522)	5,749 (4,088–7,917)	2,652 (1,722–3,814)	4,609 (3,105–6,608)	3,063 (2,077–4,325)	3,632 (1,674–6,003)	2,042 (1,117–3,364)	1,682 (1,084–2,563)	1,379 (660–2,502)	918 (732–1,113)
Burundi	Number: Cumulative change (%)	-20.2 (-35.3 to -0.6)	15.4 (-27.6 to 72.0)	-7.6 (-39.4 to 32.5)	51.3 (-18.3 to 150.8)	-22.1 (-53.3 to 21.6)	88.1 (4.2–181.1)	-73.2 (-87.6 to -53.7)	43.5 (-35.7 to 159.0)	31.9 (-34.8 to 149.8)	13.5 (-55.3 to 132.8)	86.6 (-17.3 to 208.3)
Burundi	Rate: 1990	1,232.2 (1,160.1–1,301.1)	56.5 (40.3–73.2)	142.1 (108.2–181.6)	37.2 (22.5–54.9)	144.2 (105.4–189.9)	36.7 (25.7–52.0)	332.7 (216.1–444.5)	31.0 (17.2–51.0)	33.5 (20.0–50.2)	27.2 (13.9–45.1)	13.0 (7.2–23.7)
Burundi	Rate: 2015	534.6 (437.4–653.2)	36.1 (22.7–51.1)	71.0 (51.0–96.7)	30.0 (19.5–43.0)	58.6 (40.2–82.6)	36.7 (25.2–51.4)	46.4 (22.5–75.5)	23.1 (12.7–38.1)	22.2 (14.6–33.1)	15.6 (7.5–28.4)	15.4 (12.1–18.8)
Burundi	Rate: Cumulative change (%)	-56.6 (-64.7 to -46.3)	-34.7 (-59.0 to -2.8)	-49.0 (-66.3 to -26.9)	-14.4 (-53.7 to 41.8)	-58.0 (-74.1 to -35.5)	4.5 (-42.4 to 55.8)	-85.6 (-93.1 to -75.3)	-19.0 (-63.6 to 46.1)	-28.2 (-63.6 to 30.9)	-35.9 (-74.7 to 31.6)	34.5 (-42.4 to 124.0)
Comoros	Number: 1990	2,370 (2,245–2,503)	191 (137–253)	413 (288–522)	122 (76–181)	289 (178–406)	122 (90–153)	12 (4–23)	112 (63–178)	75 (43–109)	92 (44–160)	0 (0–0)
Comoros	Number: 2015	1,381 (1,078–1,775)	140 (92–199)	187 (127–264)	124 (74–183)	106 (66–162)	136 (80–205)	3 (1–8)	100 (52–164)	45 (29–69)	65 (28–123)	1 (0–6)
Comoros	Number: Cumulative change (%)	-41.7 (-55.5 to -24.9)	-24.6 (-53.5 to 15.2)	-53.1 (-71.6 to -27.3)	7.2 (-44.4 to 79.2)	-61.2 (-78.4 to -33.7)	14.1 (-39.4 to 82.9)	-64.6 (-88.2 to -19.7)	-3.2 (-54.5 to 75.6)	-33.8 (-66.2 to 22.5)	-20.3 (-71.9 to 75.2)	28,994.3 (1,070.6–106,262.2)
Comoros	Rate: 1990	817.7 (772.4–866.3)	60.1 (43.2–79.5)	139.5 (96.9–176.7)	38.6 (24.0–56.9)	100.9 (61.9–141.2)	40.9 (30.3–51.1)	4.9 (1.9–8.9)	35.6 (20.2–56.5)	26.6 (15.9–38.6)	29.3 (14.4–50.3)	0.0 (0.0–0.1)
Comoros	Rate: 2015	302.8 (238.7–386.4)	29.2 (19.4–41.5)	40.5 (28.0–56.8)	25.9 (15.6–38.2)	23.6 (15.0–35.5)	29.1 (17.4–43.7)	1.0 (0.3–2.0)	21.1 (10.9–34.3)	10.4 (6.8–15.4)	13.5 (6.0–25.7)	0.4 (0.0–1.5)
Comoros	Rate: Cumulative change (%)	-62.9 (-71.4 to -52.4)	-50.0 (-69.2 to -23.8)	-69.9 (-81.5 to -53.3)	-29.0 (-63.0 to 18.9)	-75.2 (-86.1 to -57.6)	-26.8 (-61.3 to 17.1)	-78.1 (-92.4 to -52.2)	-36.2 (-70.0 to 14.9)	-57.6 (-77.7 to -23.4)	-47.4 (-81.5 to 14.8)	18,499.6 (689.6–66,061.1)
Djibouti	Number: 1990	2,767 (2,615–2,919)	201 (133–272)	465 (337–585)	126 (72–194)	387 (262–527)	166 (128–207)	0 (0–1)	117 (64–191)	101 (59–148)	93 (42–164)	8 (2–18)
Djibouti	Number: 2015	1,764 (1,557–2,006)	135 (90–184)	253 (175–330)	103 (59–157)	149 (91–209)	152 (104–201)	1 (0–4)	93 (52–154)	67 (41–99)	63 (29–112)	53 (39–71)
Djibouti	Number: Cumulative change (%)	-36.2 (-44.1 to -27.2)	-30.4 (-55.8 to 2.0)	-44.1 (-65.0 to -20.0)	-12.8 (-55.2 to 48.5)	-59.5 (-77.8 to -33.7)	-6.6 (-38.1 to 30.2)	169.0 (-72.0 to 715.8)	-13.3 (-60.5 to 56.6)	-28.2 (-63.7 to 27.2)	-23.6 (-71.4 to 55.3)	792.5 (191.8–1,874.0)
Djibouti	Rate: 1990	753.4 (711.9–795.3)	49.0 (32.5–66.2)	125.5 (90.1–157.7)	30.8 (17.6–47.3)	106.8 (72.0–145.0)	43.4 (33.5–54.1)	0.2 (0.0–0.4)	28.8 (15.8–46.7)	28.2 (17.0–41.4)	22.9 (10.4–39.9)	2.3 (0.8–5.2)
Djibouti	Rate: 2015	448.5 (396.3–510.0)	34.0 (22.5–46.2)	64.3 (44.5–83.6)	25.8 (14.8–39.3)	38.0 (23.3–53.2)	38.6 (26.4–50.9)	0.4 (0.1–1.0)	23.5 (13.1–38.7)	17.3 (10.7–25.3)	15.8 (7.4–28.0)	13.8 (10.3–18.5)
Djibouti	Rate: Cumulative change (%)	-40.4 (-47.7 to -31.8)	-28.4 (-54.5 to 5.0)	-47.3 (-66.8 to -24.6)	-10.3 (-53.8 to 52.9)	-62.7 (-79.5 to -39.3)	-9.3 (-39.6 to 26.6)	142.1 (-74.8 to 634.4)	-11.1 (-59.5 to 60.8)	-34.3 (-66.5 to 16.0)	-21.8 (-70.6 to 59.7)	743.9 (176.8–1,755.3)
Eritrea	Number: 1990	21,871 (21,057–22,704)	960 (640–1,356)	3,417 (1,945–4,516)	619 (351–943)	3,951 (2,104–5,436)	650 (423–881)	586 (220–1,038)	546 (282–891)	637 (314–992)	540 (244–965)	35 (17–60)
Eritrea	Number: 2015	14,169 (12,228–16,487)	949 (624–1,308)	2,215 (1,612–2,869)	782 (477–1,156)	2,098 (1,486–2,834)	1,009 (700–1,313)	209 (59–574)	649 (341–1,038)	594 (387–886)	454 (209–800)	196 (136–270)
Eritrea	Number: Cumulative change (%)	-35.2 (-44.6 to -24.4)	2.5 (-35.3 to 55.0)	-30.1 (-57.6 to 16.5)	36.2 (-31.0 to 132.3)	-40.5 (-67.2 to 6.3)	63.4 (-5.7 to 151.1)	-60.3 (-89.3 to 4.6)	30.1 (-40.6 to 133.3)	7.0 (-48.4 to 108.7)	-4.7 (-63.1 to 82.2)	528.8 (223.8–999.7)
Eritrea	Rate: 1990	1,006.8 (968.5–1,044.3)	40.3 (26.8–56.9)	153.5 (86.9–202.9)	26.0 (14.7–39.5)	182.4 (97.2–250.2)	28.7 (18.5–38.9)	27.7 (10.9–48.5)	23.0 (11.9–37.5)	29.9 (15.3–45.7)	22.7 (10.3–40.4)	1.6 (0.8–2.7)
Eritrea	Rate: 2015	465.1 (401.3–542.2)	29.7 (19.6–41.0)	71.3 (52.0–92.0)	24.5 (15.0–36.2)	68.5 (48.6–91.8)	32.3 (22.5–42.2)	7.0 (2.0–19.1)	20.4 (10.7–32.6)	19.8 (13.1–29.3)	14.2 (6.6–25.1)	7.0 (4.9–9.6)
Eritrea	Rate: Cumulative change (%)	-53.8 (-60.6 to -46.2)	-23.4 (-51.6 to 15.6)	-49.8 (-69.6 to -16.4)	1.8 (-48.4 to 73.6)	-58.0 (-76.5 to -25.2)	18.8 (-31.5 to 84.4)	-72.2 (-92.4 to -28.0)	-2.9 (-55.6 to 72.1)	-24.4 (-62.9 to 46.7)	-28.9 (-72.5 to 35.9)	386.4 (152.7–749.3)
Ethiopia	Number: 1990	508,156 (491,836–526,303)	25,712 (16,032–35,375)	66,713 (40,412–88,573)	26,536 (16,282–38,292)	66,317 (32,620–94,203)	16,258 (9,902–24,015)	22,552 (7,686–43,457)	16,282 (8,188–28,693)	22,792 (11,555–36,707)	17,883 (7,563–32,810)	2,432 (1,366–3,799)
Ethiopia	Number: 2015	232,932 (214,087–253,948)	14,640 (9,114–20,518)	29,079 (21,750–36,476)	18,540 (11,553–26,368)	18,189 (12,534–24,617)	18,745 (11,267–24,585)	761 (333–1,350)	14,591 (7,732–23,794)	10,636 (7,249–14,895)	11,647 (5,082–20,367)	6,849 (5,695–8,105)
Ethiopia	Number: Cumulative change (%)	-54.1 (-58.1 to -49.8)	-40.9 (-63.9 to -9.7)	-53.8 (-70.7 to -27.9)	-26.7 (-57.0 to 16.4)	-69.4 (-83.6 to -43.0)	25.0 (-44.8 to 102.6)	-95.8 (-98.5 to -91.0)	-0.8 (-54.3 to 77.3)	-47.4 (-73.8 to -2.9)	-25.0 (-73.4 to 49.5)	207.9 (81.2–406.9)
Ethiopia	Rate: 1990	1,469.2 (1,408.0–1,546.7)	62.6 (39.1–86.0)	184.8 (108.7–245.3)	64.6 (39.7–93.0)	191.9 (93.8–270.9)	43.5 (26.1–64.4)	68.7 (24.8–131.6)	40.0 (20.2–69.9)	66.8 (33.7–107.2)	43.8 (18.6–79.8)	7.0 (4.0–11.0)
Ethiopia	Rate: 2015	417.3 (383.3–455.1)	25.3 (15.8–35.5)	51.8 (38.9–64.9)	32.1 (20.0–45.6)	32.8 (22.8–44.2)	33.1 (19.9–43.4)	1.4 (0.6–2.4)	25.3 (13.4–41.2)	19.3 (13.2–26.9)	20.2 (8.8–35.3)	13.0 (10.8–15.4)
Ethiopia	Rate: Cumulative change (%)	-71.6 (-74.1 to -68.7)	-58.0 (-74.3 to -35.9)	-70.1 (-81.3 to -52.6)	-47.9 (-69.4 to -17.3)	-80.8 (-89.8 to -64.7)	-17.0 (-63.8 to 35.3)	-97.5 (-99.1 to -94.7)	-30.0 (-67.5 to 24.7)	-67.5 (-83.7 to -39.4)	-47.0 (-81.1 to 5.6)	102.3 (19.6–232.3)
Kenya	Number: 1990	102,361 (100,309–104,382)	4,530 (3,875–5,241)	18,175 (16,412–20,097)	7,009 (5,645–8,126)	14,296 (12,748–16,011)	4,826 (4,149–5,507)	9,759 (6,160–13,048)	2,725 (1,955–3,685)	3,705 (2,686–4,819)	2,177 (1,615–2,870)	4,297 (4,052–4,560)
Kenya	Number: 2015	98,707 (94,289–103,344)	7,646 (5,875–9,500)	13,087 (11,352–14,977)	8,678 (7,377–9,997)	10,824 (9,108–12,715)	6,897 (4,133–8,743)	3,693 (1,565–6,669)	3,970 (2,807–5,210)	3,296 (2,352–5,122)	2,416 (1,727–3,265)	14,112 (13,061–15,200)
Kenya	Number: Cumulative change (%)	-3.6 (-8.1 to 1.2)	70.2 (26.1–123.8)	-27.9 (-37.7 to -17.1)	24.8 (2.7–50.0)	-24.1 (-35.9 to -10.7)	42.3 (2.2–67.2)	-62.4 (-80.5 to -36.8)	47.9 (11.2–95.3)	-10.9 (-30.8 to 18.3)	13.4 (-24.2 to 57.4)	228.6 (204.3–255.4)
Kenya	Rate: 1990	611.6 (599.0–623.4)	25.2 (21.6–29.2)	105.9 (95.7–117.0)	39.1 (31.5–45.3)	86.5 (77.3–96.5)	28.0 (24.0–32.0)	58.9 (37.7–78.0)	15.2 (10.9–20.6)	22.5 (16.5–29.1)	12.2 (9.0–16.0)	25.4 (24.0–27.0)
Kenya	Rate: 2015	366.8 (350.8–383.5)	26.7 (20.5–33.2)	47.1 (40.9–53.8)	30.3 (25.8–34.9)	40.1 (34.0–46.8)	24.7 (14.7–31.3)	13.7 (6.0–24.3)	13.9 (9.8–18.2)	12.3 (8.9–19.0)	8.4 (6.0–11.4)	56.1 (52.2–60.2)
Kenya	Rate: Cumulative change (%)	-40.0 (-42.7 to -37.1)	6.7 (-21.0 to 40.3)	-55.5 (-61.5 to -48.8)	-21.8 (-35.6 to -6.0)	-53.6 (-60.6 to -45.7)	-12.1 (-37.0 to 3.3)	-76.8 (-87.7 to -61.5)	-7.4 (-30.4 to 22.2)	-45.0 (-56.9 to -27.7)	-29.0 (-52.6 to -1.5)	120.8 (105.6–137.3)
Madagascar	Number: 1990	80,316 (76,688–83,804)	5,998 (4,653–7,370)	11,534 (8,019–13,915)	2,394 (1,734–3,152)	14,645 (9,385–18,089)	2,790 (2,096–3,443)	4,901 (2,231–8,158)	3,024 (1,712–4,690)	2,112 (1,232–3,287)	2,561 (1,214–4,099)	227 (9–1,200)
Madagascar	Number: 2015	62,331 (53,561–72,960)	5,333 (3,806–7,066)	8,841 (6,576–11,545)	2,770 (1,657–4,177)	7,704 (5,444–10,373)	3,926 (2,655–5,273)	2,628 (1,322–4,292)	3,569 (2,032–5,557)	1,868 (1,227–2,869)	2,389 (1,203–4,035)	859 (159–3,192)
Madagascar	Number: Cumulative change (%)	-22.3 (-33.9 to -9.0)	-9.9 (-37.2 to 21.5)	-20.8 (-46.0 to 18.0)	19.3 (-34.3 to 90.7)	-44.2 (-64.5 to -10.1)	44.5 (-13.3 to 110.9)	-40.0 (-71.5 to 11.1)	27.0 (-38.8 to 123.5)	-3.3 (-50.0 to 80.1)	7.2 (-59.8 to 121.6)	1,047.5 (103.6–2,358.2)
Madagascar	Rate: 1990	975.9 (933.9–1,018.9)	63.7 (49.5–78.4)	135.9 (94.2–163.9)	25.4 (18.4–33.5)	179.8 (113.7–222.6)	31.8 (23.6–39.3)	61.5 (28.7–101.0)	32.4 (18.2–49.9)	26.2 (15.5–40.4)	27.5 (13.1–44.4)	2.9 (0.1–15.3)
Madagascar	Rate: 2015	438.2 (377.7–512.3)	35.3 (25.2–46.7)	61.4 (45.9–80.1)	18.3 (11.0–27.6)	54.4 (38.6–72.9)	26.8 (18.1–36.0)	18.9 (9.6–30.6)	23.7 (13.5–36.8)	13.4 (8.9–20.4)	15.9 (8.0–26.8)	6.8 (1.2–25.9)
Madagascar	Rate: Cumulative change (%)	-55.1 (-61.6 to -47.5)	-43.8 (-60.8 to -24.2)	-53.2 (-68.1 to -29.5)	-25.5 (-59.0 to 19.4)	-67.8 (-79.6 to -47.6)	-13.1 (-48.5 to 29.1)	-65.7 (-83.5 to -36.6)	-21.3 (-62.4 to 38.9)	-44.1 (-70.5 to 2.8)	-33.6 (-75.1 to 37.6)	590.7 (29.3–1,352.1)
Malawi	Number: 1990	106,354 (102,388–110,257)	5,886 (4,284–7,659)	10,618 (7,928–13,728)	4,038 (2,602–5,660)	17,875 (13,000–23,083)	2,961 (2,073–4,456)	34,043 (22,902–45,359)	2,488 (1,292–4,104)	4,140 (2,466–6,672)	2,079 (1,012–3,619)	2,278 (1,799–2,839)
Malawi	Number: 2015	67,204 (59,441–75,749)	4,129 (2,829–5,624)	8,809 (6,796–11,194)	4,082 (2,540–5,733)	6,140 (4,388–8,283)	3,967 (2,910–5,035)	9,270 (5,588–13,534)	2,827 (1,550–4,701)	2,897 (1,966–4,359)	1,807 (809–3,401)	7,586 (6,106–9,109)
Malawi	Number: Cumulative change (%)	-36.8 (-44.2 to -28.3)	-28.2 (-53.2 to 3.3)	-14.9 (-42.8 to 19.1)	6.9 (-41.9 to 76.8)	-64.7 (-77.1 to -48.7)	42.5 (-24.7 to 108.0)	-71.9 (-83.7 to -56.2)	25.8 (-44.9 to 142.6)	-23.8 (-62.3 to 27.9)	0.6 (-68.1 to 104.7)	237.7 (157.8–312.5)
Malawi	Rate: 1990	1,559.1 (1,499.4–1,619.4)	73.4 (53.8–95.7)	152.3 (114.0–196.6)	50.3 (32.6–70.4)	262.4 (190.2–340.8)	40.1 (28.0–61.3)	515.8 (354.2–682.1)	31.2 (16.3–51.4)	61.8 (37.5–97.7)	26.1 (12.8–45.2)	34.0 (26.8–42.3)
Malawi	Rate: 2015	608.2 (539.8–685.1)	34.2 (23.4–46.5)	77.3 (59.9–97.8)	33.8 (21.1–47.4)	55.0 (39.6–73.8)	34.3 (25.3–43.4)	83.3 (51.0–121.5)	23.5 (12.9–39.0)	26.5 (18.3–39.7)	15.0 (6.7–28.2)	77.6 (63.0–92.5)
Malawi	Rate: Cumulative change (%)	-61.0 (-65.4 to -55.7)	-52.4 (-69.0 to -31.5)	-47.9 (-65.3 to -27.1)	-29.0 (-61.3 to 17.5)	-78.4 (-85.9 to -68.8)	-8.9 (-52.4 to 33.4)	-83.3 (-90.1 to -74.3)	-16.9 (-63.4 to 59.5)	-53.6 (-76.4 to -22.8)	-33.5 (-78.8 to 34.3)	131.9 (78.0–184.3)
Mozambique	Number: 1990	145,309 (142,178–148,675)	6,583 (4,221–9,356)	15,379 (11,665–20,055)	6,134 (3,786–8,980)	14,989 (10,508–20,521)	3,974 (2,864–5,477)	49,129 (35,521–61,514)	4,406 (2,277–7,211)	3,694 (2,185–5,841)	4,159 (2,042–6,893)	507 (394–657)
Mozambique	Number: 2015	104,241 (96,495–112,572)	5,131 (3,257–7,120)	7,514 (5,811–9,583)	6,906 (4,502–9,730)	5,453 (3,824–7,467)	6,526 (4,588–8,412)	18,158 (11,997–23,929)	5,228 (2,823–8,329)	2,565 (1,675–3,832)	3,861 (1,808–6,763)	19,055 (17,111–21,222)
Mozambique	Number: Cumulative change (%)	-28.2 (-33.8 to -22.4)	-18.5 (-52.6 to 28.2)	-49.7 (-66.7 to -27.5)	20.9 (-37.0 to 99.3)	-62.0 (-77.8 to -40.4)	72.6 (-9.2 to 155.1)	-61.9 (-77.2 to -41.9)	32.0 (-40.5 to 163.2)	-23.5 (-64.1 to 43.4)	6.3 (-62.2 to 121.8)	3,727.5 (2,923.0–4,650.1)
Mozambique	Rate: 1990	1,480.4 (1,444.8–1,518.0)	58.3 (37.4–83.0)	151.6 (115.4–197.4)	54.3 (33.5–79.5)	154.3 (108.8–211.0)	38.3 (27.5–53.3)	516.0 (377.0–642.4)	39.3 (20.4–64.3)	37.9 (22.9–60.2)	37.0 (18.3–61.3)	5.2 (4.0–6.8)
Mozambique	Rate: 2015	577.2 (534.4–623.9)	26.0 (16.5–36.0)	40.5 (31.5–51.7)	35.0 (22.8–49.2)	30.4 (21.6–41.0)	34.5 (24.3–44.4)	100.0 (67.1–131.8)	26.5 (14.3–42.2)	14.3 (9.5–20.8)	19.6 (9.2–34.3)	111.9 (100.4–124.6)
Mozambique	Rate: Cumulative change (%)	-61.0 (-64.1 to -57.8)	-53.5 (-73.0 to -26.6)	-72.5 (-81.7 to -60.5)	-30.9 (-64.0 to 13.9)	-79.5 (-87.8 to -68.4)	-5.2 (-50.7 to 39.9)	-80.1 (-87.9 to -69.9)	-25.1 (-66.1 to 49.0)	-58.6 (-80.1 to -23.7)	-39.6 (-78.5 to 26.8)	2,076.4 (1,612.0–2,605.4)
Rwanda	Number: 1990	57,219 (54,912–59,704)	3,369 (2,366–4,455)	9,473 (7,035–12,048)	2,185 (1,361–3,160)	5,224 (3,102–7,940)	2,284 (1,499–3,137)	7,464 (2,425–14,276)	1,917 (1,114–3,105)	1,909 (1,142–2,901)	1,600 (832–2,688)	1,130 (881–1,403)
Rwanda	Number: 2015	28,923 (25,093–33,501)	2,234 (1,494–3,040)	4,547 (3,495–5,811)	1,996 (1,209–2,876)	1,834 (1,154–2,644)	2,553 (1,694–3,556)	912 (332–1,708)	1,741 (980–2,821)	1,243 (819–1,849)	1,029 (507–1,755)	1,257 (1,016–1,546)
Rwanda	Number: Cumulative change (%)	-49.4 (-56.6 to -41.0)	-31.7 (-56.3 to 3.3)	-50.6 (-67.1 to -29.2)	-2.8 (-48.4 to 64.9)	-62.6 (-79.4 to -38.0)	16.0 (-32.7 to 72.4)	-84.8 (-95.4 to -63.9)	-2.2 (-53.9 to 80.6)	-29.3 (-64.2 to 22.8)	-27.6 (-73.1 to 48.3)	12.5 (-12.3 to 40.4)
Rwanda	Rate: 1990	1,071.4 (1,026.5–1,120.6)	58.1 (40.8–76.8)	172.2 (128.3–219.2)	37.7 (23.4–54.6)	97.0 (58.5–146.0)	41.1 (27.0–56.3)	140.3 (46.0–266.0)	33.2 (19.3–53.6)	36.3 (22.6–54.6)	27.7 (14.4–46.5)	21.1 (16.5–26.2)
Rwanda	Rate: 2015	452.0 (392.6–522.5)	33.9 (22.7–46.1)	70.2 (54.1–89.6)	30.3 (18.4–43.6)	28.5 (18.1–41.0)	39.3 (26.1–54.8)	14.3 (5.3–26.6)	26.5 (14.9–42.9)	19.5 (13.0–28.8)	15.6 (7.7–26.7)	21.3 (17.2–26.5)
Rwanda	Rate: Cumulative change (%)	-57.8 (-63.8 to -50.8)	-39.9 (-61.6 to -9.1)	-58.1 (-71.9 to -40.0)	-14.5 (-54.6 to 44.8)	-68.8 (-82.7 to -48.7)	-0.8 (-42.7 to 47.2)	-87.5 (-96.1 to -70.4)	-14.2 (-59.5 to 59.0)	-41.9 (-69.7 to -1.2)	-36.4 (-76.4 to 30.1)	1.9 (-21.2 to 27.2)
Somalia	Number: 1990	62,124 (58,731–65,736)	2,835 (1,850–3,898)	12,520 (9,869–15,277)	1,583 (809–2,462)	11,188 (7,892–14,649)	1,854 (1,302–2,774)	3,316 (1,465–5,992)	1,596 (797–2,691)	1,866 (1,111–2,775)	1,550 (673–2,836)	18 (8–35)
Somalia	Number: 2015	60,181 (53,853–67,496)	3,212 (2,086–4,568)	11,749 (9,310–14,629)	2,164 (1,252–3,258)	10,366 (7,520–13,588)	2,490 (1,904–3,160)	2,300 (945–3,986)	1,854 (954–2,993)	2,104 (1,369–3,101)	1,586 (618–2,990)	450 (338–578)
Somalia	Number: Cumulative change (%)	-3.0 (-14.0 to 10.2)	16.7 (-25.6 to 70.7)	-4.7 (-30.4 to 26.2)	47.0 (-22.2 to 144.9)	-4.9 (-35.6 to 33.5)	40.3 (-12.0 to 93.5)	-24.9 (-66.4 to 36.1)	25.0 (-39.5 to 113.5)	21.6 (-36.8 to 118.0)	13.4 (-54.9 to 124.4)	2,733.6 (1,138.6–4,928.3)
Somalia	Rate: 1990	1,314.5 (1,224.9–1,414.9)	52.2 (34.2–71.9)	254.7 (201.1–311.3)	29.1 (14.9–45.3)	236.8 (168.7–308.9)	37.1 (26.1–55.1)	73.1 (33.7–130.0)	29.6 (14.8–49.6)	40.4 (25.0–58.6)	28.7 (12.4–52.3)	0.4 (0.2–0.8)

© 2017 Global Burden of Disease Child and Adolescent Health Collaboration. JAMA Pediatrics.

eTable 1. Number of Deaths, Death Rates (per 100,000 population), and Cumulative Percent Change with 95% Uncertainty Intervals (UI) for the Top 10 Global Causes of Death in 195 Countries and Territories, Aged 0 to 19, Both Sexes, 1990 and 2015*Note: best viewed by enlarging in browser.*

Location	Measure	All causes	Neonatal preterm birth complications	Lower respiratory infections	Neonatal encephalopathy due to birth asphyxia and trauma	Diarrheal diseases	Congenital anomalies	Malaria	Neonatal sepsis and other neonatal infections	Meningitis	Other neonatal disorders	HIV/AIDS
Somalia	Rate: 2015	790.2 (698.0–894.3)	37.7 (24.5–53.6)	149.0 (117.9–185.1)	25.4 (14.7–38.1)	136.3 (99.2–177.6)	31.2 (23.8–39.5)	31.0 (12.9–54.0)	21.9 (11.3–35.3)	28.5 (18.8–41.6)	18.7 (7.3–35.2)	6.8 (5.1–8.6)
Somalia	Rate: Cumulative change (%)	-39.8 (-47.3 to -30.9)	-25.6 (-52.5 to 8.9)	-40.5 (-56.7 to -21.1)	-6.3 (-50.3 to 55.9)	-41.0 (-59.4 to -17.7)	-12.2 (-45.2 to 21.0)	-54.3 (-79.6 to -17.5)	-20.5 (-61.4 to 36.6)	-24.4 (-59.9 to 33.1)	-27.9 (-71.2 to 42.1)	1,863.9 (796.0–3,272.3)
South Sudan	Number: 1990	57,982 (54,684–61,401)	2,832 (1,904–3,835)	8,698 (6,041–11,406)	1,571 (845–2,410)	11,021 (7,400–14,410)	1,579 (1,068–2,332)	5,976 (2,324–10,304)	1,520 (828–2,510)	2,699 (1,474–4,100)	1,418 (678–2,367)	70 (19–155)
South Sudan	Number: 2015	54,779 (47,496–63,490)	3,016 (2,006–4,299)	8,139 (5,987–10,475)	2,011 (1,175–3,107)	8,320 (5,879–11,016)	2,313 (1,759–3,034)	4,243 (1,658–8,057)	1,862 (1,014–3,064)	2,798 (1,710–4,284)	1,438 (702–2,517)	1,341 (884–1,843)
South Sudan	Number: Cumulative change (%)	-5.4 (-18.9 to 9.8)	9.6 (-29.7 to 64.7)	-3.2 (-35.6 to 42.3)	36.8 (-26.0 to 130.3)	-21.4 (-51.5 to 17.6)	54.7 (-6.8 to 125.8)	-23.4 (-65.6 to 42.1)	32.3 (-37.5 to 138.8)	13.0 (-42.3 to 101.0)	14.6 (-54.5 to 128.3)	2,591.5 (864.4–6,055.0)
South Sudan	Rate: 1990	1,374.1 (1,287.3–1,466.7)	59.1 (39.8–79.9)	199.9 (138.4–265.4)	32.8 (17.8–50.2)	260.1 (174.2–342.9)	35.6 (24.2–52.3)	145.8 (58.4–247.5)	31.9 (17.5–52.6)	63.8 (35.4–96.7)	29.8 (14.3–49.7)	1.7 (0.5–3.7)
South Sudan	Rate: 2015	745.6 (646.4–872.4)	37.4 (24.9–53.3)	108.5 (80.1–139.9)	24.9 (14.6–38.5)	113.7 (80.4–150.0)	30.2 (23.2–39.5)	58.7 (23.2–109.6)	23.2 (12.6–38.0)	38.2 (23.6–58.3)	17.9 (8.7–31.1)	19.7 (12.9–27.1)
South Sudan	Rate: Cumulative change (%)	-45.7 (-53.9 to -36.1)	-35.0 (-58.4 to -2.0)	-43.8 (-62.6 to -17.6)	-18.8 (-55.9 to 36.4)	-54.5 (-72.0 to -31.7)	-10.2 (-45.9 to 31.8)	-56.8 (-80.4 to -20.2)	-21.7 (-62.7 to 40.6)	-34.8 (-66.3 to 16.9)	-32.2 (-72.9 to 34.9)	1,513.4 (497.2–3,482.9)
Tanzania	Number: 1990	189,448 (185,868–193,405)	7,433 (4,994–10,094)	32,788 (26,311–39,244)	8,122 (5,298–11,453)	24,724 (18,832–30,950)	7,289 (5,744–9,197)	36,464 (23,675–50,128)	6,318 (3,606–9,953)	4,463 (3,020–6,454)	5,288 (2,653–8,827)	2,748 (2,298–3,228)
Tanzania	Number: 2015	147,156 (134,654–162,158)	8,900 (5,950–12,408)	19,868 (15,871–24,779)	10,108 (6,590–14,249)	7,784 (5,713–10,330)	15,797 (10,078–20,826)	10,523 (5,039–17,139)	8,978 (5,030–14,353)	5,267 (3,488–7,887)	5,727 (2,751–9,932)	7,996 (6,457–9,626)
Tanzania	Number: Cumulative change (%)	-22.3 (-29.1 to -14.7)	24.7 (-25.3 to 97.5)	-38.4 (-54.8 to -17.0)	30.9 (-27.3 to 104.8)	-67.7 (-78.9 to -52.9)	121.9 (24.0–208.6)	-70.4 (-84.7 to -50.7)	52.4 (-26.9 to 167.2)	24.8 (-32.1 to 116.3)	22.8 (-55.1 to 141.2)	191.3 (155.3–230.6)
Tanzania	Rate: 1990	1,050.9 (1,030.4–1,073.0)	36.5 (24.6–49.5)	177.5 (141.9–212.6)	39.9 (26.0–56.2)	137.9 (105.3–172.3)	38.5 (30.4–48.5)	208.3 (136.3–285.2)	31.2 (17.8–49.0)	25.4 (17.3–36.1)	26.0 (13.2–43.4)	15.4 (12.8–18.1)
Tanzania	Rate: 2015	424.8 (387.1–466.9)	23.7 (15.8–33.0)	56.0 (45.1–69.5)	26.9 (17.5–37.9)	23.0 (17.1–30.2)	43.6 (27.8–57.5)	30.4 (15.0–48.9)	23.9 (13.4–38.2)	15.4 (10.4–22.8)	15.3 (7.3–26.5)	27.3 (22.3–32.7)
Tanzania	Rate: Cumulative change (%)	-59.6 (-63.2 to -55.5)	-32.5 (-59.5 to 6.7)	-67.9 (-76.4 to -56.7)	-29.1 (-60.6 to 10.6)	-82.9 (-88.6 to -75.5)	16.0 (-35.5 to 61.6)	-85.0 (-92.1 to -75.3)	-17.7 (-60.5 to 43.8)	-36.1 (-64.2 to 7.3)	-33.6 (-75.6 to 30.6)	77.4 (56.3–99.9)
Uganda	Number: 1990	154,236 (151,135–157,639)	8,172 (5,884–10,731)	14,971 (11,503–18,916)	7,132 (4,586–10,106)	13,495 (9,622–17,909)	4,250 (2,849–5,627)	47,457 (36,543–58,393)	4,249 (2,343–7,130)	6,271 (3,886–10,077)	3,464 (1,747–6,050)	5,772 (4,850–6,777)
Uganda	Number: 2015	135,123 (125,810–145,882)	9,513 (6,541–12,670)	14,178 (11,144–18,128)	10,792 (7,089–14,521)	9,074 (6,425–12,165)	10,259 (6,917–13,650)	12,380 (5,563–20,021)	7,433 (4,174–11,775)	7,444 (4,862–11,246)	4,679 (2,346–8,027)	6,474 (5,244–8,113)
Uganda	Number: Cumulative change (%)	-12.4 (-18.6 to -5.5)	20.1 (-24.7 to 75.2)	-3.4 (-30.4 to 33.0)	59.4 (-9.3 to 149.5)	-30.0 (-57.2 to 6.1)	150.0 (46.5–257.3)	-73.6 (-87.9 to -55.7)	91.3 (-10.0 to 229.8)	28.1 (-36.7 to 119.3)	55.5 (-44.6 to 211.8)	12.5 (-6.1 to 35.4)
Uganda	Rate: 1990	1,154.4 (1,127.6–1,182.6)	52.3 (37.6–68.6)	109.3 (84.8–137.5)	45.6 (29.4–64.5)	100.5 (72.5–132.5)	29.6 (19.9–39.3)	364.1 (281.4–447.1)	27.3 (15.0–45.9)	46.9 (29.5–74.4)	22.3 (11.2–38.8)	43.5 (36.4–51.2)
Uganda	Rate: 2015	490.4 (456.3–529.1)	31.4 (21.6–41.9)	50.5 (39.9–64.1)	35.7 (23.4–48.0)	32.8 (23.7–43.4)	35.5 (23.9–47.3)	44.8 (20.6–72.3)	24.6 (13.9–39.0)	27.0 (17.9–40.1)	15.5 (7.8–26.6)	28.1 (22.8–35.1)
Uganda	Rate: Cumulative change (%)	-57.5 (-60.6 to -54.1)	-38.0 (-61.0 to -9.5)	-52.8 (-66.0 to -36.1)	-17.7 (-53.0 to 29.1)	-66.2 (-79.0 to -49.6)	24.3 (-27.9 to 77.3)	-87.5 (-94.1 to -79.4)	-1.6 (-53.5 to 70.0)	-38.3 (-68.9 to 4.4)	-20.0 (-71.3 to 60.1)	-35.2 (-46.0 to -22.4)
Zambia	Number: 1990	68,529 (65,906–71,305)	2,840 (1,955–3,773)	10,650 (8,418–12,977)	2,543 (1,616–3,563)	10,051 (7,503–13,126)	2,269 (1,793–2,900)	13,383 (8,842–18,379)	1,881 (1,009–3,046)	2,884 (1,812–4,589)	1,364 (645–2,349)	3,242 (2,696–3,801)
Zambia	Number: 2015	54,933 (48,743–61,493)	2,596 (1,702–3,564)	5,712 (4,356–7,358)	3,158 (2,144–4,294)	4,058 (2,902–5,430)	3,586 (2,125–4,876)	5,189 (3,060–7,862)	2,518 (1,431–3,992)	2,059 (1,483–2,881)	1,420 (691–2,506)	11,402 (9,739–13,081)
Zambia	Number: Cumulative change (%)	-19.8 (-28.9 to -9.8)	-5.7 (-42.7 to 38.9)	-45.4 (-62.1 to -26.4)	31.2 (-24.1 to 113.0)	-58.4 (-74.2 to -39.2)	61.6 (-12.5 to 132.0)	-59.6 (-78.7 to -33.3)	45.0 (-29.9 to 149.4)	-22.7 (-58.6 to 30.0)	18.9 (-53.2 to 138.5)	253.3 (206.6–306.6)
Zambia	Rate: 1990	1,156.7 (1,110.9–1,203.8)	41.7 (28.9–55.4)	175.0 (137.9–214.1)	37.3 (23.7–52.2)	170.8 (128.0–221.3)	36.2 (28.6–46.3)	232.2 (154.7–316.9)	27.8 (14.9–45.0)	50.2 (31.9–79.0)	20.2 (9.5–34.7)	55.2 (45.8–64.7)
Zambia	Rate: 2015	521.6 (466.0–581.7)	22.1 (14.5–30.3)	51.9 (40.0–66.3)	26.9 (18.3–36.5)	38.0 (27.7–50.4)	31.9 (18.9–43.3)	48.9 (29.4–73.4)	21.5 (12.2–34.0)	20.0 (14.7–27.6)	12.1 (5.9–21.3)	116.2 (99.3–132.7)
Zambia	Rate: Cumulative change (%)	-54.9 (-59.8 to -49.6)	-45.4 (-66.8 to -19.5)	-69.9 (-78.9 to -59.2)	-24.0 (-55.9 to 22.9)	-77.1 (-85.5 to -66.6)	-9.8 (-51.6 to 30.2)	-78.1 (-88.2 to -64.6)	-16.4 (-59.4 to 43.4)	-57.1 (-76.5 to -30.1)	-31.5 (-73.0 to 35.5)	111.7 (83.6–143.7)
Central Sub-Saharan Africa	Number: 1990	456,634 (437,526–476,071)	20,547 (15,033–27,008)	57,016 (44,772–68,842)	15,424 (10,136–21,581)	39,323 (29,683–49,805)	14,638 (8,188–31,595)	95,256 (70,130–122,537)	17,562 (11,338–25,177)	14,185 (10,117–19,151)	12,130 (6,259–19,622)	2,833 (2,365–3,332)
Central Sub-Saharan Africa	Number: 2015	462,738 (407,916–523,592)	26,521 (19,021–34,515)	59,690 (47,575–73,289)	25,282 (16,308–36,295)	30,751 (23,253–40,647)	25,084 (17,893–38,698)	75,807 (51,004–103,284)	26,449 (16,884–38,542)	18,196 (13,007–26,216)	15,497 (8,060–25,347)	12,202 (10,761–13,712)
Central Sub-Saharan Africa	Number: Cumulative change (%)	1.4 (-11.6 to 15.2)	32.2 (-8.0 to 86.0)	6.4 (-22.4 to 40.9)	70.0 (1.2–157.7)	-20.0 (-45.4 to 12.2)	91.9 (17.2–162.7)	-19.2 (-44.4 to 13.2)	56.8 (-6.5 to 150.8)	33.2 (-19.3 to 110.8)	37.4 (-34.3 to 142.4)	332.9 (281.9–388.8)
Central Sub-Saharan Africa	Rate: 1990	1,160.0 (1,110.7–1,208.7)	44.8 (32.8–58.9)	139.7 (110.2–168.7)	33.7 (22.1–47.0)	98.5 (74.6–124.1)	34.4 (19.0–75.2)	248.6 (185.3–318.5)	38.4 (24.8–54.9)	36.8 (26.4–49.4)	26.6 (13.8–42.8)	7.4 (6.1–8.7)
Central Sub-Saharan Africa	Rate: 2015	591.5 (524.1–666.0)	30.7 (22.0–40.0)	74.4 (59.6–91.2)	29.3 (18.9–42.1)	38.8 (29.7–51.0)	30.4 (21.7–47.1)	98.2 (67.4–132.8)	30.7 (19.6–44.7)	23.7 (17.1–33.3)	18.0 (9.4–29.4)	18.2 (16.1–20.6)
Central Sub-Saharan Africa	Rate: Cumulative change (%)	-49.0 (-55.4 to -42.2)	-29.7 (-51.1 to -1.0)	-45.9 (-60.5 to -28.7)	-9.7 (-46.1 to 36.9)	-59.7 (-72.4 to -43.6)	-0.3 (-40.0 to 36.9)	-60.0 (-72.3 to -44.8)	-16.8 (-50.4 to 32.9)	-33.3 (-58.3 to 4.0)	-27.2 (-65.1 to 27.6)	148.9 (119.2–181.3)
Angola	Number: 1990	120,010 (110,056–130,907)	4,796 (3,092–6,797)	17,468 (10,472–24,482)	3,492 (1,929–5,362)	14,436 (8,410–20,587)	4,041 (1,922–9,098)	10,721 (4,382–18,462)	4,606 (2,632–6,948)	4,600 (2,370–7,517)	3,148 (1,519–5,500)	94 (33–195)
Angola	Number: 2015	100,158 (82,877–121,296)	5,507 (3,376–8,047)	12,825 (9,118–17,059)	4,947 (2,884–7,557)	6,863 (4,351–10,128)	6,565 (4,438–10,255)	12,180 (6,375–19,907)	6,777 (3,728–10,419)	4,528 (2,740–7,007)	3,480 (1,629–6,006)	2,166 (1,739–2,645)
Angola	Number: Cumulative change (%)	-16.3 (-32.7 to 2.2)	19.1 (-31.5 to 80.1)	-22.0 (-51.6 to 27.4)	51.5 (-22.4 to 158.2)	-48.8 (-72.5 to -11.3)	93.8 (-3.5 to 205.0)	30.5 (-39.4 to 157.3)	57.0 (-21.5 to 170.4)	10.7 (-49.6 to 125.2)	26.0 (-53.5 to 160.7)	2,967.0 (1,041.8–6,207.8)
Angola	Rate: 1990	1,349.0 (1,230.5–1,479.0)	45.1 (29.0–63.9)	188.9 (113.0–263.3)	32.9 (18.1–50.6)	158.6 (92.9–225.6)	41.7 (19.2–96.3)	124.2 (52.6–209.5)	43.4 (24.8–65.4)	52.6 (27.7–84.0)	29.8 (14.5–51.8)	1.1 (0.4–2.2)
Angola	Rate: 2015	544.9 (449.6–663.0)	26.9 (16.5–39.3)	68.2 (49.1–90.4)	24.2 (14.1–36.9)	36.8 (23.8–53.5)	33.7 (22.8–52.4)	66.9 (35.5–108.3)	33.1 (18.3–50.8)	25.0 (15.4–38.0)	17.0 (8.0–29.3)	13.3 (10.7–16.2)
Angola	Rate: Cumulative change (%)	-59.5 (-67.4 to -50.1)	-38.2 (-64.4 to -6.8)	-61.5 (-76.1 to -36.5)	-21.4 (-59.6 to 34.3)	-75.0 (-86.6 to -56.8)	-2.4 (-52.8 to 55.5)	-38.5 (-70.9 to 17.7)	-18.7 (-59.1 to 40.1)	-46.7 (-74.8 to 5.1)	-34.9 (-75.9 to 33.9)	1,524.0 (524.8–3,174.0)
Central African Republic	Number: 1990	23,773 (22,375–25,295)	1,402 (920–1,911)	3,524 (2,624–4,439)	940 (548–1,390)	2,523 (1,732–3,499)	795 (414–1,750)	3,538 (1,931–5,237)	995 (553–1,617)	893 (526–1,361)	690 (321–1,217)	264 (185–382)
Central African Republic	Number: 2015	24,825 (20,642–29,629)	1,526 (972–2,256)	3,476 (2,509–4,676)	1,143 (662–1,774)	1,957 (1,293–2,753)	994 (578–1,792)	4,087 (1,919–6,378)	1,230 (640–2,006)	916 (567–1,448)	811 (336–1,487)	1,013 (861–1,172)
Central African Republic	Number: Cumulative change (%)	4.6 (-14.1 to 26.1)	12.3 (-32.9 to 68.7)	1.0 (-31.9 to 43.5)	29.9 (-34.2 to 126.7)	-19.0 (-52.5 to 27.8)	36.0 (-13.8 to 87.5)	19.7 (-31.3 to 88.6)	34.0 (-39.5 to 156.2)	10.5 (-43.9 to 97.2)	33.8 (-52.8 to 174.1)	297.3 (176.6–419.0)
Central African Republic	Rate: 1990	1,235.1 (1,166.4–1,310.8)	64.4 (42.3–87.7)	178.1 (131.4–225.3)	43.2 (25.2–63.9)	129.9 (90.0–178.4)	39.0 (20.2–86.7)	190.7 (104.4–281.9)	45.9 (25.6–74.7)	47.1 (28.2–71.1)	31.9 (14.9–56.0)	14.0 (9.8–20.2)
Central African Republic	Rate: 2015	910.6 (759.0–1,092.5)	51.6 (32.8–76.3)	125.0 (90.0–167.4)	38.7 (22.4–59.8)	71.5 (47.7–100.6)	35.0 (20.3–63.3)	153.2 (72.9–238.6)	41.7 (21.9–68.0)	33.9 (21.2–53.2)	27.5 (11.4–50.5)	40.9 (34.8–47.3)
Central African Republic	Rate: Cumulative change (%)	-26.2 (-39.2 to -10.9)	-17.4 (-50.7 to 23.3)	-28.1 (-51.2 to 1.5)	-4.5 (-51.6 to 66.4)	-42.6 (-66.1 to -9.9)	-2.0 (-38.1 to 34.7)	-16.8 (-51.8 to 31.0)	-1.7 (-55.3 to 87.5)	-22.7 (-59.9 to 36.1)	-1.9 (-65.3 to 101.0)	203.7 (112.2–296.0)
Congo	Number: 1990	9,820 (9,309–10,311)	505 (303–729)	1,000 (768–1,278)	442 (256–673)	612 (421–838)	489 (332–801)	1,331 (793–1,899)	560 (311–886)	326 (200–491)	307 (143–550)	255 (206–310)
Congo	Number: 2015	11,420 (9,728–13,477)	698 (385–1,068)	1,088 (789–1,458)	706 (390–1,082)	456 (286–667)	898 (643–1,210)	1,542 (760–2,509)	907 (479–1,400)	408 (255–627)	459 (216–811)	981 (841–1,128)
Congo	Number: Cumulative change (%)	16.4 (-2.2 to 37.9)	45.0 (-24.7 to 142.0)	11.3 (-27.1 to 59.9)	71.2 (-17.4 to 189.4)	-22.8 (-56.0 to 23.9)	93.2 (21.2–176.1)	18.2 (-34.2 to 87.6)	76.4 (-20.3 to 217.9)	32.0 (-29.1 to 131.1)	74.5 (-42.0 to 263.3)	287.7 (227.0–351.9)
Congo	Rate: 1990	650.4 (616.6–683.6)	30.5 (18.3–44.0)	64.8 (49.8–82.3)	26.7 (15.5–40.7)	40.2 (27.8–54.6)	30.6 (20.8–50.7)	89.1 (53.7–127.9)	33.9 (18.9–53.5)	21.8 (13.7–32.5)	18.6 (8.7–33.2)	16.9 (13.6–20.4)
Congo	Rate: 2015	407.6 (348.0–478.8)	23.0 (12.7–35.2)	38.0 (27.9–50.6)	23.3 (12.9–35.7)	16.1 (10.3–23.4)	30.4 (21.9–40.9)	55.5 (28.3–89.4)	29.9 (15.8–46.1)	14.7 (9.5–22.2)	15.1 (7.2–26.7)	38.7 (33.3–44.5)
Congo	Rate: Cumulative change (%)	-37.3 (-47.1 to -25.7)	-20.9 (-58.9 to 32.0)	-39.9 (-60.2 to -14.1)	-6.7 (-54.9 to 57.7)	-58.4 (-75.6 to -34.4)	4.5 (-34.6 to 49.1)	-36.4 (-63.9 to -0.6)	-3.9 (-56.5 to 73.1)	-29.2 (-60.6 to 21.7)	-5.1 (-68.3 to 97.5)	131.7 (94.5–170.2)
Democratic Republic of the Congo	Number: 1990	296,524 (279,792–313,633)	13,450 (8,640–18,657)	34,193 (25,931–43,027)	10,237 (5,766–15,676)	21,262 (14,907–28,001)	8,977 (4,842–19,584)	78,548 (58,545–99,793)	10,994 (5,941–17,562)	8,130 (5,240–11,709)	7,749 (2,995–14,428)	2,198 (1,760–2,694)
Democratic Republic of the Congo	Number: 2015	320,520 (269,580–377,568)	18,401 (11,791–25,982)	41,781 (30,872–54,339)	18,158 (9,709–28,624)	21,314 (14,448–30,058)	16,118 (10,704–26,407)	56,938 (37,107–79,503)	16,992 (8,928–27,820)	12,137 (7,519–19,163)	10,472 (3,844–20,089)	7,854 (6,535–9,296)
Democratic Republic of the Congo	Number: Cumulative change (%)	8.2 (-10.4 to 28.8)	43.5 (-15.6 to 133.6)	24.9 (-16.6 to 75.4)	90.0 (-4.8 to 226.2)	3.8 (-38.2 to 59.0)	100.1 (19.7–186.9)	-26.3 (-52.0 to 5.6)	68.7 (-22.5 to 222.6)	57.9 (-18.2 to 176.2)	56.9 (-46.5 to 248.9)	259.4 (207.3–314.4)
Democratic Republic of the Congo	Rate: 1990	1,132.9 (1,070.1–1,197.1)	44.2 (28.6–61.3)	125.9 (95.4–158.7)	33.7 (19.0–51.6)	80.2 (56.8–104.8)	31.7 (16.9–69.9)	309.0 (231.4–391.2)	36.2 (19.6–57.7)	31.8 (20.8–45.4)	25.6 (10.0–47.5)	8.6 (6.9–10.6)
Democratic Republic of the Congo	Rate: 2015	605.4 (512.5–709.4)	31.5 (20.2–44.6)	77.0 (57.4–99.5)	31.1 (16.7–49.1)	39.7 (27.1–55.5)	28.9 (19.3–47.2)	109.1 (72.0–151.5)	29.2 (15.3–47.7)	23.4 (14.8–36.0)	18.0 (6.6–34.6)	17.5 (14.5–20.8)
Democratic Republic of the Congo	Rate: Cumulative change (%)	-46.5 (-55.4 to -36.5)	-25.1 (-56.0 to 21.6)	-37.5 (-58.1 to -12.6)	-1.0 (-50.4 to 69.7)	-48.8 (-69.0 to -22.0)	2.2 (-39.8 to 47.5)	-64.2 (-76.3 to -49.4)	-12.2 (-59.6 to 68.0)	-22.4 (-58.0 to 32.7)	-18.5 (-72.2 to 80.8)	103.8 (73.4–135.7)
Equatorial Guinea	Number: 1990	3,163 (2,905–3,429)	172 (112–241)	500 (372–628)	131 (76–198)	294 (191–411)	118 (63–249)	533 (344–739)	144 (81–226)	119 (69–189)	92 (43–165)	9 (2–25)
Equatorial Guinea	Number: 2015	2,954 (2,395–3,652)	168 (66–292)	263 (162–409)	134 (64–227)	74 (42–118)	203 (119–310)	754 (400–1,115)	237 (122–372)	103 (57–174)	120 (44–245)	136 (114–161)
Equatorial Guinea	Number: Cumulative change (%)	-6.4 (-25.9 to 17.4)	0.2 (-58.6 to 76.6)	-46.3 (-68.6 to -14.9)	12.0 (-56.3 to 121.8)	-73.5 (-86.6 to -53.7)	98.7 (-6.5 to 221.2)	49.3 (-25.4 to 149.4)	78.0 (-18.7 to 228.5)	-6.6 (-58.1 to 76.6)	58.3 (-58.0 to 283.4)	2,367.0 (434.3–4,989.8)
Equatorial Guinea	Rate: 1990	1,200.3 (1,089.8–1,314.5)	54.2 (35.3–75.9)	180.8 (134.7–227.1)	41.5 (24.1–62.3)	109.1 (72.1–150.1)	40.9 (21.6–86.4)	211.6 (137.0–293.3)	45.5 (25.9–71.1)	47.0 (28.3–72.2)	29.4 (13.9–52.2)	3.5 (1.0–9.6)
Equatorial Guinea	Rate: 2015	606.1 (488.8–749.1)	31.7 (12.6–55.1)	53.0 (33.4–81.8)	25.4 (12.1–42.8)	15.1 (8.7–24.0)	39.6 (23.4–60.2)	155.7 (82.6–230.5)	44.8 (23.2–70.3)	21.2 (12.3–35.5)	22.8 (8.3–46.4)	32.4 (27.0–38.1)
Equatorial Guinea	Rate: Cumulative change (%)	-49.4 (-59.8 to -36.3)	-40.1 (-75.2 to 5.7)	-70.0 (-82.1 to -52.8)	-33.1 (-73.8 to 32.6)	-85.5 (-92.4 to -75.0)	12.7 (-47.4 to 81.8)	-22.6 (-60.3 to 28.5)	6.3 (-51.2 to 96.1)	-51.3 (-77.2 to -11.0)	-6.0 (-75.0 to 128.7)	1,420.5 (229.4–2,966.4)
Gabon	Number: 1990	3,344 (3,220–3,479)	220 (112–344)	328 (239–430)	180 (96–280)	194 (126–280)	215 (145–309)	583 (354–807)	261 (140–403)	115 (69–180)	141 (63–263)	10 (7–14)
Gabon	Number: 2015	2,858 (2,585–3,155)	218 (97–360)	255 (189–336)	191 (104–297)	85 (54–129)	304 (219–401)	304 (109–569)	305 (170–469)	102 (60–164)	153 (59–284)	48 (30–67)
Gabon	Number: Cumulative change (%)	-14.5 (-23.4 to -4.9)	2.6 (-48.4 to 62.0)	-20.5 (-44.4 to 7.0)	16.2 (-45.6 to 113.8)	-53.9 (-73.4 to -26.1)	46.5 (-4.1 to 102.3)	-48.2 (-78.1 to -13.5)	26.1 (-38.8 to 118.2)	-5.9 (-51.0 to 66.9)	25.7 (-57.2 to 154.9)	367.1 (209.1–571.0)
Gabon	Rate: 1990	570.5 (549.4–593.1)	34.3 (17.6–53.7)	54.7 (40.6–70.9)	28.1 (15.1–43.9)	32.8 (21.7–47.0)	34.8 (23.4–49.8)	100.8 (62.3–138.3)	40.8 (22.0–63.1)	19.8 (12.3–30.6)	22.1 (10.0–41.1)	1.8 (1.3–2.4)
Gabon	Rate: 2015	319.1 (288.9–352.4)	23.4 (10.4–38.6)	28.4 (21.3–37.1)	20.5 (11.2–31.9)	9.6 (6.2–14.3)	33.1 (23.9–43.7)	34.2 (12.7–63.5)	32.7 (18.2–50.3)	11.6 (7.0–18.4)	16.4 (6.4–30.5)	5.9 (3.7–8.3)
Gabon	Rate: Cumulative change (%)	-44.0 (-49.8 to -37.8)	-29.5 (-64.6 to 11.2)	-47.1 (-62.6 to -28.9)	-20.2 (-62.8 to 46.9)	-69.5 (-82.0 to -51.5)	-1.3 (-35.5 to 36.1)	-66.2 (-85.6 to -44.4)	-13.5 (-58.0 to 49.5)	-38.6 (-67.1 to 5.6)	-13.9 (-70.6 to 74.9)	230.5 (119.1–373.3)

© 2017 Global Burden of Disease Child and Adolescent Health Collaboration. JAMA Pediatrics.

eTable 2. Number of Deaths, Death Rates (per 100,000 population), and Cumulative Percent Change with 95% Uncertainty Intervals (UI) for the Top 10 Global Causes of Death in 195 Countries and Territories, Aged Under 5 Years, Both Sexes, 1990 and 2015 *Note: best viewed by enlarging in browser.*

Location	Measure	All causes	Neonatal preterm birth complications	Neonatal encephalopathy due to birth asphyxia and trauma	Lower respiratory infections	Diarrheal diseases	Congenital anomalies	Malaria	Neonatal sepsis and other neonatal infections	Other neonatal disorders	Protein-energy malnutrition	Meningitis
Global	Number: 1990	12,120,128 (12,028,115–12,215,202)	1,795,211 (1,685,077–1,918,767)	915,323 (810,730–1,021,011)	2,125,305 (1,942,797–2,285,845)	1,387,614 (1,234,489–1,523,450)	646,946 (489,890–901,913)	701,371 (505,264–909,030)	329,296 (247,585–418,271)	351,304 (265,375–451,582)	402,153 (322,751–485,797)	318,443 (256,880–372,267)
Global	Number: 2015	5,820,849 (5,694,979–5,947,525)	805,778 (747,783–876,860)	740,424 (680,024–809,507)	703,917 (658,172–752,599)	498,888 (453,291–544,832)	496,584 (459,286–543,130)	474,146 (357,188–592,695)	351,667 (275,540–428,214)	220,247 (174,247–266,264)	174,414 (137,967–218,659)	173,058 (140,696–210,560)
Global	Number: Cumulative change (%)	-52.0 (-53.1 to -50.9)	-55.1 (-58.3 to -51.7)	-18.8 (-27.1 to -9.3)	-66.8 (-69.8 to -63.4)	-63.9 (-68.2 to -58.7)	-21.3 (-41.2 to -3.1)	-31.1 (-48.4 to -9.4)	8.0 (-11.9 to 32.5)	-36.2 (-50.6 to -19.3)	-56.2 (-66.5 to -43.1)	-45.1 (-55.2 to -26.6)
Global	Rate: 1990	1,863.8 (1,849.5–1,878.2)	271.1 (254.5–289.9)	138.2 (122.4–154.2)	327.8 (299.4–352.6)	214.8 (191.0–236.0)	98.8 (74.7–138.0)	109.5 (78.8–141.7)	49.8 (37.4–63.3)	53.2 (40.2–68.4)	62.6 (50.2–75.7)	49.3 (39.8–57.8)
Global	Rate: 2015	863.0 (844.3–881.8)	119.1 (110.6–129.6)	109.5 (100.5–119.7)	104.4 (97.6–111.7)	74.1 (67.3–80.9)	73.6 (68.0–80.5)	70.5 (53.1–88.2)	52.0 (40.8–63.3)	32.6 (25.8–39.4)	25.9 (20.5–32.5)	25.7 (20.9–31.3)
Global	Rate: Cumulative change (%)	-53.7 (-54.7 to -52.6)	-56.0 (-59.2 to -52.7)	-20.5 (-28.6 to -11.2)	-68.1 (-71.0 to -64.8)	-65.4 (-69.5 to -60.4)	-23.7 (-43.0 to -6.0)	-34.3 (-50.8 to -13.7)	5.5 (-14.0 to 29.6)	-37.7 (-51.7 to -21.2)	-58.2 (-68.0 to -45.6)	-47.4 (-57.1 to -29.7)
High SDI	Number: 1990	192,746 (190,282–195,535)	42,760 (41,201–45,248)	17,881 (16,834–18,986)	15,056 (14,362–15,878)	3,168 (2,942–3,406)	46,449 (38,390–48,500)	1 (0–3)	4,644 (3,765–7,181)	9,008 (8,499–9,583)	328 (291–369)	3,693 (3,510–3,903)
High SDI	Number: 2015	74,543 (73,208–75,905)	13,493 (12,118–14,234)	5,509 (4,985–5,922)	3,437 (3,197–3,675)	763 (709–811)	21,471 (20,050–24,796)	0 (0–0)	2,920 (2,334–4,355)	5,802 (5,173–6,245)	73 (63–83)	734 (687–787)
High SDI	Number: Cumulative change (%)	-61.3 (-62.2 to -60.5)	-68.4 (-72.9 to -66.3)	-69.2 (-72.6 to -66.5)	-77.1 (-79.3 to -75.1)	-75.9 (-78.4 to -73.3)	-53.4 (-58.1 to -35.2)	-93.6 (-97.7 to -88.9)	-36.6 (-45.6 to -29.6)	-35.5 (-44.5 to -29.6)	-77.7 (-81.2 to -73.6)	-80.1 (-81.8 to -78.3)
High SDI	Rate: 1990	261.8 (258.5–265.6)	58.9 (56.8–62.4)	24.6 (23.2–26.2)	20.3 (19.4–21.4)	4.3 (4.0–4.6)	63.3 (52.3–66.1)	0.0 (0.0–0.0)	6.4 (5.2–9.9)	12.4 (11.7–13.2)	0.4 (0.4–0.5)	4.9 (4.7–5.2)
High SDI	Rate: 2015	108.6 (106.7–110.6)	19.8 (17.8–20.9)	8.1 (7.3–8.7)	5.0 (4.6–5.3)	1.1 (1.0–1.2)	31.3 (29.2–36.2)	0.0 (0.0–0.0)	4.3 (3.4–6.4)	8.5 (7.6–9.2)	0.1 (0.1–0.1)	1.1 (1.0–1.1)
High SDI	Rate: Cumulative change (%)	-58.5 (-59.5 to -57.6)	-66.3 (-71.1 to -64.1)	-67.1 (-70.8 to -64.3)	-75.4 (-77.7 to -73.2)	-74.1 (-76.8 to -71.3)	-50.1 (-55.1 to -30.6)	-93.1 (-97.5 to -88.0)	-32.4 (-42.1 to -25.0)	-31.2 (-40.8 to -25.0)	-76.0 (-79.8 to -71.7)	-78.5 (-80.4 to -76.5)
High-middle SDI	Number: 1990	1,317,064 (1,286,934–1,349,293)	286,715 (272,767–303,522)	96,928 (89,645–106,292)	232,657 (218,584–246,848)	100,439 (93,110–108,241)	150,461 (115,473–178,581)	291 (235–358)	25,555 (21,607–32,750)	54,939 (46,951–63,782)	25,963 (21,192–30,935)	25,410 (18,057–31,320)
High-middle SDI	Number: 2015	374,323 (359,934–389,420)	75,776 (70,021–81,741)	49,568 (45,191–54,954)	36,619 (33,537–40,364)	9,640 (8,592–10,774)	73,065 (61,668–79,915)	102 (45–185)	18,756 (14,773–21,786)	21,014 (18,635–23,402)	3,469 (3,008–4,089)	4,808 (4,078–5,598)
High-middle SDI	Number: Cumulative change (%)	-71.6 (-72.9 to -70.2)	-73.6 (-75.7 to -71.2)	-48.8 (-53.6 to -43.6)	-84.2 (-85.9 to -82.3)	-90.4 (-91.7 to -89.0)	-50.6 (-64.1 to -37.2)	-64.4 (-84.4 to -33.9)	-26.1 (-36.0 to -15.6)	-61.4 (-68.0 to -53.8)	-86.5 (-89.4 to -81.8)	-80.6 (-85.1 to -74.7)
High-middle SDI	Rate: 1990	952.1 (930.4–975.4)	208.0 (197.9–220.2)	70.3 (65.0–77.1)	168.1 (158.0–178.4)	72.5 (67.3–78.2)	108.9 (83.6–129.2)	0.2 (0.2–0.3)	18.6 (15.7–23.8)	39.9 (34.1–46.3)	18.7 (15.3–22.3)	18.3 (13.0–22.6)
High-middle SDI	Rate: 2015	322.4 (309.9–335.4)	66.1 (61.1–71.3)	43.3 (39.4–48.0)	31.3 (28.7–34.5)	8.2 (7.3–9.2)	63.0 (53.1–68.8)	0.1 (0.0–0.2)	16.3 (12.9–19.0)	18.3 (16.2–20.4)	2.9 (2.5–3.5)	4.1 (3.5–4.8)
High-middle SDI	Rate: Cumulative change (%)	-66.1 (-67.7 to -64.6)	-68.2 (-70.8 to -65.4)	-38.4 (-44.2 to -32.2)	-81.4 (-83.4 to -79.1)	-88.7 (-90.2 to -87.0)	-41.3 (-57.2 to -25.3)	-58.5 (-81.8 to -22.9)	-11.3 (-23.1 to 1.3)	-53.7 (-61.6 to -44.6)	-84.1 (-87.6 to -78.6)	-77.1 (-82.4 to -70.2)
Middle SDI	Number: 1990	2,978,454 (2,911,354–3,053,298)	575,921 (543,773–609,110)	240,833 (219,106–265,021)	589,556 (536,442–642,905)	284,763 (252,976–312,589)	203,572 (153,133–266,458)	7,691 (5,372–10,546)	62,606 (45,982–76,729)	87,112 (70,977–107,110)	56,192 (44,095–67,452)	68,391 (49,814–85,410)
Middle SDI	Number: 2015	866,659 (834,747–900,826)	178,438 (164,777–194,477)	133,759 (123,079–145,797)	94,450 (86,837–102,739)	41,565 (36,545–46,895)	120,292 (104,866–132,571)	2,919 (1,910–4,210)	44,875 (32,922–54,463)	36,854 (31,258–43,092)	7,521 (6,461–8,841)	16,724 (13,251–20,287)
Middle SDI	Number: Cumulative change (%)	-70.9 (-72.2 to -69.6)	-69.0 (-71.5 to -66.3)	-44.3 (-49.9 to -37.5)	-83.9 (-85.8 to -81.8)	-85.3 (-87.5 to -83.0)	-38.9 (-58.7 to -19.1)	-60.5 (-77.5 to -38.8)	-27.8 (-39.2 to -14.3)	-57.1 (-66.9 to -46.8)	-86.4 (-89.4 to -82.9)	-75.1 (-80.7 to -67.4)
Middle SDI	Rate: 1990	1,457.0 (1,423.8–1,493.9)	279.4 (263.8–295.5)	116.8 (106.3–128.6)	288.7 (262.5–314.9)	139.7 (124.0–153.3)	99.3 (74.7–130.0)	3.8 (2.7–5.2)	30.4 (22.4–37.3)	42.3 (34.5–52.0)	27.6 (21.7–33.2)	33.6 (24.5–41.9)
Middle SDI	Rate: 2015	483.2 (465.4–502.2)	99.6 (92.0–108.5)	74.6 (68.7–81.4)	52.6 (48.4–57.2)	23.1 (20.3–26.1)	67.0 (58.4–73.9)	1.6 (1.1–2.3)	25.0 (18.4–30.4)	20.6 (17.4–24.0)	4.2 (3.6–4.9)	9.3 (7.4–11.3)
Middle SDI	Rate: Cumulative change (%)	-66.8 (-68.4 to -65.4)	-64.3 (-67.2 to -61.2)	-35.9 (-42.4 to -28.2)	-81.7 (-83.9 to -79.4)	-83.4 (-85.8 to -80.7)	-30.2 (-52.8 to -7.5)	-55.4 (-74.7 to -31.0)	-17.2 (-30.2 to -1.6)	-50.8 (-62.0 to -39.0)	-84.6 (-88.0 to -80.6)	-71.8 (-78.1 to -63.1)
Low-middle SDI	Number: 1990	5,405,434 (5,357,803–5,453,613)	765,273 (687,681–838,028)	459,777 (387,377–528,140)	971,800 (873,261–1,055,088)	696,133 (612,196–774,196)	172,601 (121,598–280,344)	322,489 (231,070–420,645)	149,124 (103,802–204,753)	137,559 (81,589–202,262)	177,101 (129,007–238,164)	133,153 (107,717–158,094)
Low-middle SDI	Number: 2015	2,816,798 (2,722,276–2,914,081)	410,824 (367,295–458,314)	432,718 (386,238–481,556)	337,407 (309,241–367,726)	281,112 (247,787–315,897)	175,681 (154,572–200,658)	223,302 (160,255–285,844)	173,049 (126,369–221,638)	99,004 (74,395–129,011)	72,498 (51,771–102,665)	78,296 (63,067–102,832)
Low-middle SDI	Number: Cumulative change (%)	-47.9 (-49.6 to -46.0)	-46.2 (-52.2 to -39.7)	-5.2 (-18.6 to 12.1)	-65.2 (-68.9 to -61.3)	-59.4 (-65.9 to -52.0)	7.0 (-28.9 to 31.8)	-29.3 (-49.6 to -4.0)	19.3 (-16.1 to 62.2)	-23.4 (-52.2 to 14.7)	-57.9 (-72.7 to -37.6)	-40.4 (-55.0 to -18.2)
Low-middle SDI	Rate: 1990	3,022.4 (2,994.4–3,051.0)	408.6 (367.0–447.7)	245.2 (206.7–281.5)	548.2 (492.0–596.0)	395.8 (347.2–441.1)	94.7 (66.3–154.5)	187.1 (134.0–243.8)	79.9 (55.7–109.7)	74.2 (44.2–109.0)	102.2 (74.5–138.2)	75.9 (61.1–90.4)
Low-middle SDI	Rate: 2015	1,314.6 (1,270.9–1,360.4)	189.7 (169.6–211.7)	199.8 (178.4–222.4)	158.1 (144.9–172.3)	132.1 (116.4–148.5)	81.8 (71.9–93.4)	105.6 (75.8–135.2)	80.0 (58.5–102.4)	45.9 (34.5–59.7)	34.2 (24.4–48.5)	36.8 (29.6–48.2)
Low-middle SDI	Rate: Cumulative change (%)	-56.5 (-58.0 to -54.9)	-53.5 (-58.7 to -47.8)	-17.9 (-29.5 to -3.0)	-71.1 (-74.3 to -67.8)	-66.5 (-71.8 to -60.4)	-9.0 (-40.0 to 12.3)	-42.4 (-59.0 to -21.7)	2.9 (-27.6 to 39.8)	-34.3 (-58.9 to -1.5)	-65.6 (-77.7 to -48.9)	-50.9 (-63.0 to -32.5)
Low SDI	Number: 1990	2,221,288 (2,197,906–2,244,088)	123,952 (104,152–145,779)	99,603 (77,284–120,973)	315,277 (269,108–363,771)	302,363 (247,201–356,711)	73,557 (50,163–125,135)	370,733 (263,373–487,112)	87,218 (64,660–117,569)	62,494 (40,922–88,562)	142,375 (106,060–181,386)	87,630 (63,661–111,763)
Low SDI	Number: 2015	1,686,241 (1,624,616–1,754,269)	126,934 (111,052–144,139)	118,676 (99,577–140,545)	231,654 (208,414–256,227)	165,627 (146,579–184,734)	105,804 (88,300–134,162)	247,773 (189,440–311,021)	111,929 (88,170–142,382)	57,449 (37,636–77,990)	90,791 (69,539–117,226)	72,425 (55,958–91,248)
Low SDI	Number: Cumulative change (%)	-24.1 (-27.2 to -21.0)	3.2 (-12.6 to 24.3)	20.4 (-0.2 to 46.5)	-26.0 (-38.1 to -12.7)	-44.6 (-54.6 to -31.6)	52.3 (6.3–95.9)	-31.7 (-49.0 to -8.7)	30.4 (0.4–67.7)	-5.0 (-36.4 to 36.3)	-34.9 (-54.1 to -11.0)	-15.5 (-36.9 to 15.6)
Low SDI	Rate: 1990	4,251.3 (4,204.9–4,297.1)	213.8 (179.7–251.4)	171.7 (133.4–208.5)	600.2 (509.6–696.3)	588.6 (479.2–696.5)	134.5 (90.5–233.4)	740.8 (527.9–970.4)	151.1 (112.0–202.9)	108.4 (71.2–153.5)	284.9 (211.5–365.7)	169.2 (121.9–215.7)
Low SDI	Rate: 2015	1,789.9 (1,723.5–1,863.7)	129.8 (113.6–147.4)	121.3 (101.8–143.7)	246.2 (221.4–272.1)	177.8 (157.4–198.5)	110.8 (92.3–140.9)	270.0 (206.8–339.0)	114.6 (90.4–145.8)	58.9 (38.6–79.8)	98.5 (75.6–127.2)	77.5 (59.9–97.8)
Low SDI	Rate: Cumulative change (%)	-57.9 (-59.6 to -56.2)	-38.8 (-48.1 to -26.3)	-28.6 (-40.8 to -13.2)	-58.6 (-65.5 to -51.0)	-69.4 (-75.0 to -62.1)	-12.3 (-39.8 to 13.8)	-62.8 (-72.1 to -50.3)	-22.9 (-40.5 to -1.0)	-43.9 (-62.4 to -19.5)	-64.7 (-75.2 to -51.7)	-53.1 (-65.3 to -35.9)
High-income	Number: 1990	137,192 (135,449–139,519)	33,329 (32,138–35,689)	10,301 (9,815–11,041)	7,208 (6,872–7,591)	1,615 (1,501–1,737)	33,096 (27,006–34,686)	2 (1–4)	3,725 (2,658–5,020)	6,281 (5,942–6,731)	825 (722–943)	2,384 (2,281–2,508)
High-income	Number: 2015	59,662 (58,824–60,541)	12,312 (11,195–12,959)	3,866 (3,530–4,163)	1,972 (1,833–2,120)	683 (637–731)	17,105 (16,046–19,350)	0 (0–0)	2,111 (1,705–3,239)	4,709 (4,238–5,062)	125 (106–146)	534 (503–570)
High-income	Number: Cumulative change (%)	-56.5 (-57.4 to -55.6)	-63.0 (-68.2 to -60.5)	-62.4 (-67.2 to -58.9)	-72.6 (-75.1 to -70.1)	-57.6 (-62.0 to -53.2)	-47.9 (-53.5 to -28.2)	-86.7 (-93.7 to -80.3)	-43.1 (-52.2 to -26.7)	-24.9 (-34.6 to -17.3)	-84.7 (-87.6 to -81.4)	-77.6 (-79.2 to -75.8)
High-income	Rate: 1990	229.8 (226.8–233.7)	56.2 (54.2–60.2)	17.4 (16.5–18.6)	12.0 (11.4–12.6)	2.7 (2.5–2.9)	55.5 (45.3–58.2)	0.0 (0.0–0.0)	6.3 (4.5–8.5)	10.6 (10.0–11.4)	1.4 (1.2–1.6)	4.0 (3.8–4.2)
High-income	Rate: 2015	103.6 (102.1–105.1)	21.6 (19.6–22.7)	6.8 (6.2–7.3)	3.4 (3.2–3.7)	1.2 (1.1–1.3)	29.7 (27.9–33.6)	0.0 (0.0–0.0)	3.7 (3.0–5.7)	8.2 (7.4–8.9)	0.2 (0.2–0.3)	0.9 (0.9–1.0)
High-income	Rate: Cumulative change (%)	-54.9 (-55.8 to -54.0)	-61.6 (-66.9 to -59.0)	-61.0 (-66.0 to -57.3)	-71.7 (-74.2 to -69.0)	-56.1 (-60.6 to -51.5)	-46.0 (-51.8 to -25.6)	-86.3 (-93.5 to -79.8)	-41.0 (-50.3 to -23.9)	-22.0 (-32.1 to -14.1)	-84.2 (-87.3 to -80.8)	-76.8 (-78.5 to -75.0)
High-income North America	Number: 1990	48,282 (48,084–48,472)	12,644 (12,248–13,190)	3,113 (2,956–3,310)	1,561 (1,458–1,673)	240 (221–259)	10,742 (9,398–11,175)	0 (0–0)	963 (682–1,341)	3,264 (3,083–3,485)	24 (22–27)	717 (670–763)
High-income North America	Number: 2015	26,104 (25,878–26,326)	5,914 (5,442–6,243)	1,683 (1,552–1,813)	586 (537–642)	307 (276–338)	6,437 (5,975–7,556)	0 (0–0)	730 (562–1,105)	2,382 (2,147–2,579)	13 (11–16)	183 (169–197)
High-income North America	Number: Cumulative change (%)	-45.9 (-46.4 to -45.4)	-53.2 (-58.8 to -50.1)	-45.8 (-52.4 to -40.9)	-62.4 (-66.5 to -58.0)	28.1 (11.6–45.1)	-39.8 (-45.9 to -19.0)	nan (nan–nan)	-24.1 (-33.8 to -10.3)	-26.9 (-35.6 to -18.8)	-43.7 (-53.3 to -33.6)	-74.3 (-76.8 to -71.6)
High-income North America	Rate: 1990	227.7 (226.8–228.6)	59.7 (57.8–62.3)	14.7 (14.0–15.6)	7.3 (6.9–7.9)	1.1 (1.0–1.2)	50.7 (44.3–52.7)	0.0 (0.0–0.0)	4.5 (3.2–6.3)	15.4 (14.6–16.5)	0.1 (0.1–0.1)	3.4 (3.2–3.6)
High-income North America	Rate: 2015	121.1 (120.0–122.1)	27.6 (25.4–29.2)	7.9 (7.3–8.5)	2.7 (2.5–3.0)	1.4 (1.3–1.6)	29.9 (27.7–35.1)	0.0 (0.0–0.0)	3.4 (2.6–5.2)	11.1 (10.0–12.1)	0.1 (0.1–0.1)	0.8 (0.8–0.9)
High-income North America	Rate: Cumulative change (%)	-46.8 (-47.3 to -46.4)	-53.7 (-59.2 to -50.6)	-46.4 (-52.9 to -41.5)	-63.2 (-67.3 to -59.0)	26.0 (9.8–42.7)	-40.7 (-46.7 to -20.2)	nan (nan–nan)	-25.0 (-34.5 to -11.3)	-27.7 (-36.3 to -19.7)	-45.0 (-54.3 to -35.1)	-74.9 (-77.3 to -72.3)
Canada	Number: 1990	3,250 (3,194–3,301)	726 (657–818)	285 (244–328)	94 (83–107)	6 (5–8)	981 (744–1,074)	0 (0–0)	47 (32–65)	144 (119–172)	1 (1–2)	45 (40–51)
Canada	Number: 2015	1,943 (1,842–2,057)	397 (349–448)	195 (163–231)	47 (39–55)	14 (12–17)	513 (460–574)	0 (0–0)	40 (25–55)	208 (173–248)	0 (0–1)	19 (16–22)
Canada	Number: Cumulative change (%)	-40.2 (-43.4 to -36.6)	-45.0 (-54.6 to -36.4)	-30.8 (-45.2 to -15.1)	-49.9 (-59.4 to -38.7)	112.3 (62.6–171.7)	-47.0 (-55.3 to -25.3)	nan (nan–nan)	-14.7 (-33.8 to 7.4)	46.3 (10.8–86.8)	-34.9 (-57.8 to -4.5)	-57.4 (-64.6 to -49.7)
Canada	Rate: 1990	172.9 (169.9–175.6)	38.8 (35.1–43.6)	15.2 (13.0–17.5)	5.0 (4.4–5.7)	0.4 (0.3–0.4)	52.2 (39.6–57.2)	0.0 (0.0–0.0)	2.5 (1.7–3.5)	7.7 (6.4–9.2)	0.1 (0.1–0.1)	2.4 (2.1–2.8)
Canada	Rate: 2015	103.0 (97.6–109.0)	21.2 (18.6–23.9)	10.4 (8.7–12.4)	2.5 (2.1–2.9)	0.8 (0.6–0.9)	27.2 (24.4–30.5)	0.0 (0.0–0.0)	2.2 (1.4–3.0)	11.1 (9.2–13.2)	0.1 (0.0–0.1)	1.0 (0.9–1.2)
Canada	Rate: Cumulative change (%)	-40.4 (-43.6 to -36.9)	-45.1 (-54.6 to -36.4)	-30.8 (-45.3 to -15.1)	-50.3 (-59.7 to -39.2)	111.1 (61.8–170.1)	-47.3 (-55.5 to -25.5)	nan (nan–nan)	-14.8 (-33.8 to 7.3)	46.2 (10.6–86.7)	-35.4 (-58.1 to -5.0)	-57.7 (-64.9 to -50.1)
Greenland	Number: 1990	89 (84–94)	27 (23–31)	9 (7–11)	4 (3–6)	0 (0–0)	12 (6–16)	0 (0–0)	4 (2–7)	7 (4–10)	0 (0–0)	2 (1–3)
Greenland	Number: 2015	24 (21–26)	7 (5–8)	2 (2–3)	0 (0–1)	0 (0–0)	4 (3–4)	0 (0–0)	1 (0–2)	1 (1–2)	0 (0–0)	0 (0–0)
Greenland	Number: Cumulative change (%)	-73.1 (-75.8 to -70.1)	-74.4 (-79.4 to -68.9)	-66.8 (-75.7 to -55.4)	-82.5 (-87.9 to -75.8)	-25.9 (-63.5 to 27.8)	-66.9 (-77.0 to -47.9)	nan (nan–nan)	-64.8 (-85.0 to -42.5)	-71.4 (-82.5 to -59.2)	-67.1 (-85.5 to -40.8)	-82.7 (-88.3 to -76.0)
Greenland	Rate: 1990	772.2 (728.5–815.8)	232.0 (199.9–268.6)	76.6 (60.6–94.3)	43.8 (31.4–57.7)	1.8 (1.1–2.8)	111.1 (58.3–145.3)	0.0 (0.0–0.0)	35.5 (18.8–59.1)	60.4 (42.1–93.0)	0.0 (0.0–0.1)	20.2 (13.5–28.0)
Greenland	Rate: 2015	310.3 (283.2–341.4)	91.1 (75.3–108.0)	38.1 (29.8–47.6)	10.9 (8.6–13.6)	1.8 (1.2–2.8)	51.8 (40.7–62.8)	0.0 (0.0–0.0)	17.6 (9.6–29.3)	25.2 (19.1–33.1)	0.0 (0.0–0.0)	4.8 (3.7–6.2)
Greenland	Rate: Cumulative change (%)	-59.8 (-63.8 to -55.3)	-60.5 (-68.3 to -52.0)	-49.4 (-63.0 to -32.3)	-74.4 (-82.2 to -64.6)	9.8 (-46.0 to 89.5)	-50.5 (-65.6 to -21.8)	nan (nan–nan)	-46.1 (-77.1 to -12.2)	-56.2 (-73.3 to -37.4)	-52.9 (-79.0 to -15.4)	-75.4 (-83.5 to -65.9)
United States	Number: 1990	44,918 (44,731–45,098)	11,884 (11,499–12,374)	2,817 (2,663–3,001)	1,460 (1,360–1,572)	233 (215–252)	9,742 (8,636–10,126)	0 (0–0)	911 (646–1,271)	3,111 (2,930–3,325)	23 (21–25)	669 (625–715)
United States	Number: 2015	24,128 (23,940–24,327)	5,508 (5,046–5,823)	1,484 (1,363–1,612)	538 (488–591)	292 (262–322)	5,917 (5,482–7,002)	0 (0–0)	688 (532–1,049)	2,170 (1,954–2,359)	12 (10–15)	163 (150–177)
United States	Number: Cumulative change (%)	-46.3 (-46.8 to -45.8)	-53.6 (-59.1 to -50.6)	-47.2 (-53.7 to -42.0)	-63.1 (-67.4 to -58.4)	25.8 (9.1–43.3)	-39.0 (-45.5 to -18.5)	nan (nan–nan)	-24.4 (-34.6 to -9.8)	-30.1 (-38.6 to -22.4)	-44.2 (-54.1 to -34.2)	-75.5 (-77.9 to -72.7)
United States	Rate: 1990	232.8 (231.8–233.7)	61.6 (59.6–64.2)	14.6 (13.8–15.6)	7.6 (7.0–8.1)	1.2 (1.1–1.3)	50.5 (44.7–52.5)	0.0 (0.0–0.0)	4.7 (3.4–6.6)	16.1 (15.2–17.3)	0.1 (0.1–0.1)	3.5 (3.2–3.7)
United States	Rate: 2015	122.7 (121.8–123.7)	28.2 (25.9–29.9)	7.6 (7.0–8.3)	2.7 (2.5–3.0)	1.5 (1.3–1.6)	30.1 (27.9–35.7)	0.0 (0.0–0.0)	3.5 (2.7–5.4)	11.1 (10.0–12.1)	0.1 (0.1–0.1)	0.8 (0.8–0.9)
United States	Rate: Cumulative change (%)	-47.3 (-47.7 to -46.8)	-54.2 (-59.6 to -51.2)	-47.9 (-54.3 to -42.7)	-63.9 (-68.1 to -59.4)	23.5 (7.1–40.7)	-40.0 (-46.5 to -19.9)	nan (nan–nan)	-25.3 (-35.4 to -10.9)	-31.0 (-39.3 to -23.4)	-45.5 (-55.2 to -35.7)	-76.1 (-78.5 to -73.4)
Australasia	Number: 1990	3,062 (3,006–3,115)	658 (605–719)	277 (247–313)	103 (93–113)	9 (8–11)	753 (637–815)	0 (0–0)	60 (45–93)	77 (66–89)	1 (0–1)	45 (40–50)
Australasia	Number: 2015	1,564 (1,503–1,628)	205 (172–237)	150 (123–176)	46 (40–53)	15 (12–17)	463 (406–546)	0 (0–0)	36 (22–70)	141 (116–166)	0 (0–1)	14 (13–16)
Australasia	Number: Cumulative change (%)	-48.9 (-51.1 to -46.6)	-68.7 (-75.4 to -63.1)	-45.2 (-56.8 to -34.0)	-54.8 (-61.5 to -46.8)	56.3 (28.3–90.9)	-38.0 (-48.3 to -13.7)	nan (nan–nan)	-41.1 (-60.2 to -12.9)	84.1 (39.6–133.1)	-21.6 (-46.8 to 10.5)	-67.7 (-71.8 to -63.0)
Australasia	Rate: 1990	202.4 (198.8–206.0)	43.6 (40.1–47.6)	18.3 (16.4–20.7)	6.8 (6.2–7.5)	0.6 (0.6–0.7)	49.8 (42.2–53.9)	0.0 (0.0–0.0)	4.0 (3.0–6.2)	5.1 (4.4–5.9)	0.1 (0.1–0.1)	3.0 (2.7–3.3)
Australasia	Rate: 2015	84.9 (81.6–88.4)	11.2 (9.4–12.9)	8.2 (6.8–9.6)	2.5 (2.2–2.9)	0.8 (0.7–1.0)	25.2 (22.1–29.7)	0.0 (0.0–0.0)	2.0 (1.2–3.9)	7.7 (6.3–9.1)	0.1 (0.0–0.1)	0.8 (0.7–0.9)
Australasia	Rate: Cumulative change (%)	-58.0 (-59.9 to -56.1)	-74.1 (-79.7 to -69.5)	-54.8 (-64.4 to -45.5)	-63.0 (-68.5 to -56.4)	27.7 (4.8–55.9)	-49.0 (-57.5 to -29.0)	nan (nan–nan)	-51.4 (-67.2 to -28.1)	52.0 (15.2–92.5)	-36.0 (-56.4 to -9.7)	-73.6 (-76.9 to -69.7)
Australia	Number: 1990	2,432 (2,379–2,482)	554 (502–613)	230 (201–265)	67 (59–77)	7 (6–8)	600 (490–659)	0 (0–0)	56 (41–88)	69 (57–81)	1 (0–1)	35 (30–39)
Australia	Number: 2015	1,246 (1,190–1,308)	152 (123–182)	135 (109–160)	34 (28–40)	11 (9–14)	374 (324–431)	0 (0–0)	30 (16–66)	124 (99–148)	0 (0–1)	9 (8–11)
Australia	Number: Cumulative change (%)	-48.7 (-51.4 to -45.9)	-72.4 (-78.7 to -66.3)	-40.8 (-54.7 to -27.3)	-49.4 (-59.7 to -36.5)	61.1 (24.8–105.6)	-37.0 (-48.7 to -11.2)	nan (nan–nan)	-46.9 (-68.7 to -13.2)	82.6 (34.7–136.1)	-19.5 (-46.2 to 14.2)	-72.0 (-76.6 to -66.8)
Australia	Rate: 1990	197.0 (192.7–201.1)	44.9 (40.7–49.8)	18.7 (16.3–21.5)	5.5 (4.8–6.2)	0.6 (0.5–0.7)	48.6 (39.7–53.4)	0.0 (0.0–0.0)	4.5 (3.4–7.2)	5.6 (4.7–6.6)	0.1 (0.1–0.1)	2.9 (2.5–3.2)
Australia	Rate: 2015	80.6 (77.0–84.6)	9.9 (8.0–11.8)	8.8 (7.1–10.4)	2.2 (1.8–2.6)	0.7 (0.6–0.9)	24.2 (21.0–27.9)	0.0 (0.0–0.0)	2.0 (1.1–4.3)	8.1 (6.5–9.6)	0.1 (0.0–0.1)	0.6 (0.5–0.7)
Australia	Rate: Cumulative change (%)	-59.1 (-61.2 to -56.8)	-77.9 (-83.0 to -73.1)	-52.6 (-63.7 to -41.7)	-59.7 (-67.9 to -49.4)	28.2 (-0.7 to 63.5)	-49.7 (-59.0 to -29.0)	nan (nan–nan)	-57.5 (-74.9 to -30.5)	46.2 (7.8–89.1)	-36.0 (-57.1 to -9.1)	-77.7 (-81.4 to -73.6)
New Zealand	Number: 1990	629 (613–645)	104 (94–115)	46 (40–53)	35 (31–40)	2 (2–3)	153 (140–165)	0 (0–0)	4 (2–5)	8 (6–10)	0 (0–0)	10 (8–11)
New Zealand	Number: 2015	317 (301–336)	53 (44–61)	15 (12–18)	12 (10–14)	3 (3–4)	89 (77–113)	0 (0–0)	5 (1–7)	16 (13–20)	0 (0–0)	4 (4–5)
New Zealand	Number: Cumulative change (%)	-49.5 (-52.4 to -46.3)	-48.6 (-58.1 to -40.0)	-66.5 (-74.3 to -57.9)	-64.9 (-72.1 to -56.9)	44.9 (11.6–85.1)	-41.5 (-50.8 to -21.7)	nan (nan–nan)	23.8 (-27.2 to 77.2)	101.1 (49.5–163.4)	-46.1 (-68.9 to -14.7)	-52.5 (-60.3 to -43.1)
New Zealand	Rate: 1990	226.8 (220.9–232.7)	37.5 (33.9–41.4)	16.8 (14.5–19.3)	12.8 (11.3–14.5)	0.9 (0.8–1.1)	55.2 (50.8–59.7)	0.0 (0.0–0.0)	1.6 (0.8–2.1)	3.0 (2.4–3.6)	0.0 (0.0–0.0)	3.7 (3.3–4.3)
New Zealand	Rate: 2015	107.6 (101.9–113.8)	18.4 (15.5–21.0)	5.3 (4.3–6.4)	4.2 (3.5–5.0)	1.3 (1.0–1.5)	30.3 (26.4–38.5)	0.0 (0.0–0.0)	1.9 (0.7–2.7)	5.7 (4.6–7.0)	0.0 (0.0–0.0)	1.6 (1.4–1.9)
New Zealand	Rate: Cumulative change (%)	-52.6 (-55.4 to -49.6)	-50.8 (-60.0 to -42.7)	-68.0 (-75.5 to -59.8)	-67.2 (-73.9 to -59.6)	34.9 (3.7–72.1)	-44.8 (-53.6 to -26.1)	nan (nan–nan)	18.4 (-30.3 to 69.7)	92.0 (42.6–151.4)	-50.0 (-71.0 to -21.3)	-56.0 (-63.2 to -47.1)
High-income Asia Pacific	Number: 1990	16,620 (14,919–18,844)	2,632 (2,234–3,277)	886 (775–1,048)	1,066 (931–1,237)	172 (145–206)	5,195 (3,533–6,054)	1 (0–3)	489 (347–783)	635 (551–759)	5 (4–6)	249 (213–289)
High-income Asia Pacific	Number: 2015	4,457 (4,230–4,688)	519 (403–609)	205 (174–232)	205 (184–225)	46 (41–51)	1,551 (1,432–1,813)	0 (0–0)	172 (116–298)	303 (246–347)	4 (3–4)	27 (24–29)
High-income Asia Pacific	Number: Cumulative change (%)	-73.1 (-76.5 to -69.8)	-79.9 (-86.5 to -74.7)	-76.6 (-82.7 to -71.6)	-80.6 (-84.2 to -77.2)	-72.7 (-78.3 to -66.7)	-69.3 (-75.3 to -48.5)	-95.7 (-98.7 to -91.7)	-64.4 (-75.3 to -46.8)	-51.8 (-64.6 to -41.6)	-25.1 (-43.5 to -4.3)	-88.9 (-90.8 to -87.0)
High-income Asia Pacific	Rate: 1990	166.8 (149.7–189.4)	27.0 (22.9–33.6)	9.1 (7.9–10.7)	10.6 (9.3–12.3)	1.7 (1.4–2.1)	52.5 (35.7–61.3)	0.0 (0.0–0.0)	5.0 (3.5–8.0)	6.5 (5.6–7.8)	0.1 (0.0–0.1)	2.5 (2.1–2.9)
High-income Asia Pacific	Rate: 2015	58.8 (55.8–61.8)	7.0 (5.4–8.2)	2.8 (2.3–3.1)	2.7 (2.4–2.9)	0.6 (0.5–0.7)	20.5 (18.9–24.0)	0.0 (0.0–0.0)	2.3 (1.6–4.0)	4.1 (3.3–4.6)	0.1 (0.0–0.1)	0.4 (0.3–0.4)
High-income Asia Pacific	Rate: Cumulative change (%)	-64.6 (-69.1 to -60.3)	-73.8 (-82.3 to -66.9)	-69.4 (-77.4 to -62.9)	-74.5 (-79.3 to -70.0)	-64.2 (-71.6 to -56.3)	-59.8 (-67.6 to -32.4)	-94.4 (-98.3 to -89.1)	-53.5 (-67.7 to -30.4)	-37.0 (-53.7 to -23.6)	-1.7 (-25.9 to 25.8)	-85.4 (-87.9 to -82.8)
Brunei	Number: 1990	80 (76–85)	11 (8–14)	3 (1–5)	7 (6–8)	0 (0–1)	22 (18–25)	0 (0–0)	2 (0–3)	3 (1–6)	0 (0–0)	1 (1–1)
Brunei	Number: 2015	59 (53–66)	8 (6–10)	2 (1–3)	5 (4–6)	0 (0–0)	16 (13–18)	0 (0–0)	2 (1–3)	3 (2–5)	0 (0–0)	0 (0–0)
Brunei	Number: Cumulative change (%)	-26.3 (-35.3 to -16.0)	-23.0 (-48.8 to 6.7)	-6.6 (-53.2 to 63.3)	-29.8 (-45.9 to -13.4)	-41.0 (-60.0 to -17.7)	-26.5 (-38.6 to -12.8)	nan (nan–nan)	31.4 (-38.5 to 145.5)	7.7 (-51.2 to 103.2)	-24.8 (-59.3 to 23.8)	-47.2 (-61.4 to -30.3)
Brunei	Rate: 1990	208.5 (197.1–221.3)	28.0 (21.0–35.9)	8.5 (4.7–13.1)	19.1 (15.9–22.9)	2.1 (1.6–2.7)	56.4 (47.5–64.5)	0.0 (0.0–0.0)	5.4 (2.4–9.6)	9.8 (5.0–16.4)	0.1 (0.0–0.1)	3.6 (2.9–4.3)

© 2017 Global Burden of Disease Child and Adolescent Health Collaboration. JAMA Pediatrics.

eTable 2. Number of Deaths, Death Rates (per 100,000 population), and Cumulative Percent Change with 95% Uncertainty Intervals (UI) for the Top 10 Global Causes of Death in 195 Countries and Territories, Aged Under 5 Years, Both Sexes, 1990 and 2015*Note: best viewed by enlarging in browser.*

Location	Measure	All causes	Neonatal preterm birth complications	Neonatal encephalopathy due to birth asphyxia and trauma	Lower respiratory infections	Diarrheal diseases	Congenital anomalies	Malaria	Neonatal sepsis and other neonatal infections	Other neonatal disorders	Protein-energy malnutrition	Meningitis
Brunei	Rate: 2015	181.5 (162.5–203.5)	25.6 (19.4–32.4)	8.8 (5.8–12.3)	15.5 (13.0–18.3)	1.5 (1.1–1.9)	49.3 (40.3–57.9)	0.0 (0.0–0.0)	7.7 (4.7–11.9)	11.3 (7.1–16.6)	0.0 (0.0–0.1)	2.2 (1.7–2.7)
Brunei	Rate: Cumulative change (%)	-12.8 (-23.6 to -0.5)	-5.9 (-37.5 to 30.4)	14.0 (-42.9 to 99.1)	-17.9 (-36.6 to 1.9)	-30.6 (-53.0 to -3.1)	-12.1 (-26.4 to 4.0)	nan (nan–nan)	60.4 (-24.9 to 199.5)	31.4 (-40.5 to 148.2)	-12.8 (-53.1 to 43.2)	-38.2 (-54.8 to -18.6)
Japan	Number: 1990	7,995 (7,912–8,075)	1,079 (1,023–1,159)	522 (489–567)	417 (391–448)	48 (45–52)	2,787 (2,487–2,889)	0 (0–2)	159 (104–197)	365 (339–393)	2 (2–2)	92 (86–99)
Japan	Number: 2015	2,841 (2,775–2,902)	227 (186–253)	136 (115–152)	149 (131–166)	36 (32–40)	1,147 (1,071–1,361)	0 (0–0)	62 (48–97)	183 (152–205)	3 (3–4)	19 (17–21)
Japan	Number: Cumulative change (%)	-64.5 (-65.3 to -63.6)	-78.9 (-83.6 to -76.1)	-73.9 (-79.2 to -70.4)	-64.0 (-69.9 to -59.4)	-23.7 (-36.8 to -13.2)	-58.7 (-61.9 to -45.3)	-100.0 (-100.0 to -100.0)	-60.0 (-69.0 to -42.3)	-49.6 (-60.8 to -42.2)	47.0 (16.3–77.1)	-78.7 (-81.8 to -76.3)
Japan	Rate: 1990	124.7 (123.4–126.0)	17.3 (16.4–18.6)	8.4 (7.9–9.1)	6.4 (6.0–6.9)	0.7 (0.7–0.8)	43.8 (39.1–45.4)	0.0 (0.0–0.0)	2.6 (1.7–3.2)	5.9 (5.4–6.3)	0.0 (0.0–0.0)	1.4 (1.3–1.5)
Japan	Rate: 2015	55.5 (54.2–56.7)	4.5 (3.7–5.1)	2.7 (2.3–3.0)	2.9 (2.5–3.2)	0.7 (0.6–0.8)	22.5 (21.0–26.7)	0.0 (0.0–0.0)	1.2 (1.0–1.9)	3.7 (3.0–4.1)	0.1 (0.1–0.1)	0.4 (0.3–0.4)
Japan	Rate: Cumulative change (%)	-55.5 (-56.6 to -54.4)	-73.8 (-79.7 to -70.4)	-67.7 (-74.3 to -63.3)	-54.7 (-62.2 to -48.8)	-3.7 (-20.2 to 9.7)	-48.4 (-52.4 to -31.7)	-100.0 (-100.0 to -100.0)	-50.4 (-61.6 to -28.5)	-37.5 (-51.4 to -28.4)	85.0 (46.2–123.0)	-73.1 (-77.1 to -70.0)
Singapore	Number: 1990	380 (365–395)	75 (67–90)	25 (21–31)	45 (40–52)	6 (5–7)	141 (101–154)	0 (0–0)	2 (1–3)	10 (8–13)	0 (0–0)	5 (4–5)
Singapore	Number: 2015	82 (75–90)	10 (8–12)	3 (2–4)	11 (8–12)	0 (0–0)	28 (24–38)	0 (0–0)	1 (0–2)	8 (6–10)	0 (0–0)	0 (0–0)
Singapore	Number: Cumulative change (%)	-78.3 (-80.4 to -76.0)	-86.1 (-90.4 to -83.0)	-86.0 (-90.5 to -81.7)	-75.5 (-82.7 to -70.1)	-87.4 (-91.5 to -83.7)	-79.3 (-83.7 to -62.0)	nan (nan–nan)	-35.0 (-66.0 to -7.1)	-18.4 (-41.7 to 9.6)	-73.7 (-82.7 to -61.2)	-90.7 (-93.1 to -88.3)
Singapore	Rate: 1990	158.0 (151.7–164.4)	30.8 (27.2–36.6)	10.4 (8.6–12.8)	19.2 (17.1–22.0)	2.6 (2.2–3.2)	58.4 (42.2–64.0)	0.0 (0.0–0.0)	1.0 (0.7–1.6)	4.3 (3.4–5.3)	0.0 (0.0–0.0)	2.2 (1.9–2.5)
Singapore	Rate: 2015	44.3 (40.5–48.6)	5.7 (4.6–6.8)	1.9 (1.5–2.4)	5.9 (4.7–6.9)	0.4 (0.3–0.5)	15.2 (12.9–20.7)	0.0 (0.0–0.0)	0.9 (0.5–1.2)	4.6 (3.7–5.5)	0.0 (0.0–0.0)	0.3 (0.2–0.3)
Singapore	Rate: Cumulative change (%)	-72.0 (-74.7 to -69.1)	-81.3 (-87.1 to -77.1)	-81.2 (-87.2 to -75.5)	-69.1 (-78.2 to -62.4)	-84.1 (-89.2 to -79.4)	-73.2 (-78.8 to -50.6)	nan (nan–nan)	-12.7 (-54.3 to 24.7)	9.5 (-21.7 to 47.2)	-67.7 (-78.9 to -52.5)	-88.2 (-91.4 to -85.2)
South Korea	Number: 1990	8,163 (6,457–10,388)	1,466 (1,075–2,069)	335 (237–477)	596 (466–764)	117 (89–149)	2,245 (924–3,058)	0 (0–0)	324 (184–594)	255 (177–364)	2 (1–4)	150 (116–187)
South Korea	Number: 2015	1,473 (1,277–1,706)	272 (190–346)	63 (47–80)	39 (32–48)	8 (6–10)	360 (297–430)	0 (0–0)	105 (56–200)	107 (77–140)	0 (0–0)	6 (5–7)
South Korea	Number: Cumulative change (%)	-81.6 (-86.2 to -76.5)	-80.6 (-88.8 to -71.9)	-80.2 (-88.2 to -70.7)	-93.2 (-95.1 to -91.2)	-92.6 (-94.8 to -89.7)	-81.9 (-89.1 to -56.8)	-90.5 (-92.8 to -87.7)	-66.9 (-80.5 to -47.5)	-56.1 (-73.9 to -34.3)	-92.7 (-95.6 to -88.7)	-95.6 (-96.7 to -94.1)
South Korea	Rate: 1990	249.1 (196.9–316.6)	45.2 (33.1–63.8)	10.3 (7.3–14.7)	18.1 (14.2–23.2)	3.6 (2.7–4.6)	68.8 (28.3–93.7)	0.0 (0.0–0.0)	10.0 (5.7–18.3)	7.9 (5.5–11.2)	0.1 (0.1–0.1)	4.6 (3.5–5.7)
South Korea	Rate: 2015	65.6 (56.9–76.0)	12.3 (8.6–15.6)	2.8 (2.1–3.6)	1.7 (1.4–2.1)	0.4 (0.3–0.5)	16.1 (13.3–19.2)	0.0 (0.0–0.0)	4.7 (2.5–9.0)	4.8 (3.5–6.3)	0.0 (0.0–0.0)	0.3 (0.2–0.3)
South Korea	Rate: Cumulative change (%)	-73.1 (-79.9 to -65.7)	-71.7 (-83.7 to -58.9)	-71.0 (-82.8 to -57.1)	-90.2 (-92.9 to -87.2)	-89.2 (-92.5 to -85.0)	-73.7 (-84.2 to -37.0)	-86.1 (-89.5 to -82.0)	-51.6 (-71.6 to -23.4)	-35.8 (-61.9 to -3.9)	-89.4 (-93.7 to -83.5)	-93.6 (-95.2 to -91.4)
Western Europe	Number: 1990	42,449 (42,184–42,699)	10,249 (9,821–11,191)	4,157 (3,943–4,452)	1,484 (1,400–1,628)	239 (223–259)	11,584 (9,029–12,179)	0 (0–0)	1,041 (781–1,522)	1,225 (1,139–1,329)	17 (15–20)	795 (748–855)
Western Europe	Number: 2015	15,817 (15,113–16,584)	3,090 (2,686–3,336)	1,247 (1,092–1,385)	417 (383–454)	153 (137–169)	5,063 (4,570–5,926)	0 (0–0)	514 (383–887)	1,205 (1,030–1,358)	9 (8–11)	168 (155–182)
Western Europe	Number: Cumulative change (%)	-62.7 (-64.4 to -60.9)	-69.8 (-76.0 to -66.8)	-69.9 (-75.1 to -66.1)	-71.8 (-75.4 to -68.4)	-35.8 (-45.2 to -27.3)	-55.7 (-61.8 to -33.9)	nan (nan–nan)	-50.9 (-60.6 to -31.7)	-1.4 (-19.7 to 12.7)	-43.1 (-55.9 to -31.4)	-78.8 (-81.0 to -76.5)
Western Europe	Rate: 1990	193.0 (191.8–194.2)	47.1 (45.1–51.4)	19.1 (18.1–20.4)	6.7 (6.3–7.3)	1.1 (1.0–1.2)	52.8 (41.1–55.5)	0.0 (0.0–0.0)	4.8 (3.6–7.0)	5.6 (5.2–6.1)	0.1 (0.1–0.1)	3.6 (3.4–3.8)
Western Europe	Rate: 2015	73.2 (70.0–76.8)	14.4 (12.5–15.6)	5.8 (5.1–6.5)	1.9 (1.8–2.1)	0.7 (0.6–0.8)	23.5 (21.2–27.5)	0.0 (0.0–0.0)	2.4 (1.8–4.1)	5.6 (4.8–6.3)	0.0 (0.0–0.1)	0.8 (0.7–0.8)
Western Europe	Rate: Cumulative change (%)	-62.1 (-63.8 to -60.2)	-69.3 (-75.5 to -66.2)	-69.4 (-74.7 to -65.5)	-71.3 (-75.0 to -67.8)	-34.6 (-44.2 to -25.9)	-55.0 (-61.1 to -32.7)	nan (nan–nan)	-50.0 (-60.0 to -30.5)	0.4 (-18.3 to 14.7)	-42.1 (-55.1 to -30.1)	-78.3 (-80.7 to -76.1)
Andorra	Number: 1990	3 (3–4)	0 (0–1)	0 (0–0)	0 (0–0)	0 (0–0)	1 (0–1)	0 (0–0)	0 (0–0)	0 (0–0)	0 (0–0)	0 (0–0)
Andorra	Number: 2015	1 (1–1)	0 (0–0)	0 (0–0)	0 (0–0)	0 (0–0)	0 (0–0)	0 (0–0)	0 (0–0)	0 (0–0)	0 (0–0)	0 (0–0)
Andorra	Number: Cumulative change (%)	-63.7 (-70.0 to -55.4)	-75.9 (-85.1 to -64.8)	-70.5 (-84.2 to -49.5)	-75.5 (-85.9 to -60.3)	-53.5 (-78.8 to -14.5)	-53.3 (-66.6 to -36.3)	nan (nan–nan)	-49.6 (-81.8 to 2.1)	-47.0 (-84.5 to 21.2)	-50.7 (-92.3 to 36.9)	-73.9 (-90.3 to -49.3)
Andorra	Rate: 1990	125.1 (105.3–147.3)	26.6 (19.4–35.2)	12.7 (7.9–18.5)	3.9 (2.7–5.4)	0.5 (0.3–0.8)	39.2 (31.1–48.2)	0.0 (0.0–0.0)	2.9 (1.4–5.4)	5.0 (2.2–8.8)	0.0 (0.0–0.1)	2.1 (1.2–3.5)
Andorra	Rate: 2015	39.1 (32.9–46.5)	5.6 (3.8–7.6)	3.2 (1.9–4.8)	0.8 (0.5–1.2)	0.2 (0.1–0.3)	15.8 (11.5–21.2)	0.0 (0.0–0.0)	1.2 (0.6–2.1)	2.0 (0.8–3.5)	0.0 (0.0–0.0)	0.4 (0.2–0.8)
Andorra	Rate: Cumulative change (%)	-68.6 (-74.1 to -61.5)	-78.4 (-86.6 to -68.5)	-73.6 (-85.9 to -54.8)	-79.3 (-88.1 to -66.2)	-60.2 (-82.1 to -26.9)	-59.2 (-70.9 to -44.4)	nan (nan–nan)	-54.9 (-83.7 to -8.3)	-52.7 (-86.3 to 8.6)	-59.4 (-93.6 to 11.3)	-78.3 (-91.9 to -57.9)
Austria	Number: 1990	854 (822–888)	184 (164–216)	123 (107–141)	30 (26–34)	1 (1–1)	242 (156–271)	0 (0–0)	7 (4–10)	20 (17–25)	0 (0–0)	11 (9–12)
Austria	Number: 2015	288 (260–320)	53 (44–62)	23 (19–28)	6 (5–7)	4 (3–5)	102 (90–116)	0 (0–0)	3 (2–6)	19 (15–23)	0 (0–0)	2 (2–3)
Austria	Number: Cumulative change (%)	-66.3 (-69.8 to -62.3)	-71.0 (-76.8 to -65.0)	-81.1 (-85.2 to -76.2)	-77.7 (-82.2 to -72.3)	184.2 (114.6–265.5)	-56.6 (-64.2 to -35.1)	nan (nan–nan)	-48.9 (-64.4 to -28.3)	-6.1 (-30.2 to 20.1)	-27.8 (-58.3 to 16.6)	-76.7 (-80.9 to -71.7)
Austria	Rate: 1990	198.0 (190.6–206.0)	42.8 (38.2–50.4)	28.7 (25.1–33.0)	7.0 (6.1–8.0)	0.4 (0.3–0.4)	56.2 (36.2–63.1)	0.0 (0.0–0.0)	1.8 (1.2–2.5)	4.9 (4.0–5.9)	0.1 (0.0–0.1)	2.6 (2.2–3.0)
Austria	Rate: 2015	72.1 (65.1–80.2)	13.3 (11.2–15.6)	5.8 (4.8–7.1)	1.7 (1.4–2.0)	1.1 (0.9–1.3)	25.8 (22.6–29.1)	0.0 (0.0–0.0)	1.0 (0.7–1.6)	4.9 (3.9–6.0)	0.0 (0.0–0.1)	0.6 (0.5–0.8)
Austria	Rate: Cumulative change (%)	-63.6 (-67.4 to -59.3)	-68.7 (-74.9 to -62.2)	-79.6 (-84.0 to -74.3)	-75.9 (-80.8 to -70.1)	206.6 (131.6–294.2)	-53.2 (-61.4 to -30.0)	nan (nan–nan)	-44.8 (-61.6 to -22.5)	1.4 (-24.6 to 29.7)	-22.1 (-55.0 to 25.7)	-74.8 (-79.4 to -69.5)
Belgium	Number: 1990	1,195 (1,173–1,218)	254 (229–284)	127 (108–150)	30 (25–34)	6 (5–7)	289 (173–322)	0 (0–0)	29 (18–59)	40 (33–49)	0 (0–0)	15 (13–18)
Belgium	Number: 2015	495 (455–540)	54 (43–65)	52 (42–64)	12 (10–14)	6 (5–7)	149 (129–170)	0 (0–0)	16 (8–35)	44 (35–53)	0 (0–0)	5 (4–7)
Belgium	Number: Cumulative change (%)	-58.6 (-62.0 to -54.9)	-78.7 (-83.2 to -73.8)	-58.2 (-67.7 to -47.5)	-58.4 (-67.1 to -49.0)	1.7 (-21.1 to 31.2)	-47.0 (-57.4 to -17.0)	nan (nan–nan)	-44.8 (-60.2 to -25.6)	10.9 (-15.7 to 41.7)	83.7 (10.3–184.6)	-62.8 (-69.6 to -54.8)
Belgium	Rate: 1990	205.5 (201.8–209.4)	44.1 (39.6–49.2)	22.1 (18.8–26.0)	5.1 (4.4–5.9)	1.0 (0.9–1.2)	49.8 (29.9–55.6)	0.0 (0.0–0.0)	5.0 (3.2–10.2)	7.0 (5.7–8.6)	0.0 (0.0–0.0)	2.7 (2.3–3.2)
Belgium	Rate: 2015	77.9 (71.6–85.1)	8.5 (6.9–10.3)	8.4 (6.7–10.2)	1.9 (1.6–2.3)	1.0 (0.8–1.2)	23.5 (20.4–26.9)	0.0 (0.0–0.0)	2.6 (1.4–5.6)	7.0 (5.6–8.5)	0.0 (0.0–0.1)	0.9 (0.8–1.1)
Belgium	Rate: Cumulative change (%)	-62.1 (-65.2 to -58.7)	-80.5 (-84.6 to -76.0)	-61.7 (-70.4 to -51.9)	-62.0 (-69.9 to -53.3)	-7.0 (-27.9 to 19.9)	-51.5 (-61.1 to -24.0)	nan (nan–nan)	-49.5 (-63.6 to -31.9)	1.6 (-22.8 to 29.7)	67.9 (0.8–160.3)	-66.0 (-72.2 to -58.7)
Cyprus	Number: 1990	151 (145–157)	33 (23–43)	11 (7–17)	12 (9–15)	3 (2–4)	26 (18–34)	0 (0–0)	7 (3–12)	19 (10–30)	0 (0–0)	2 (1–2)
Cyprus	Number: 2015	32 (29–36)	5 (3–6)	2 (1–3)	1 (1–1)	0 (0–1)	8 (7–9)	0 (0–0)	1 (1–3)	4 (2–6)	0 (0–0)	0 (0–0)
Cyprus	Number: Cumulative change (%)	-78.5 (-80.9 to -75.7)	-83.9 (-89.6 to -76.0)	-77.7 (-88.7 to -61.7)	-87.5 (-91.4 to -82.7)	-71.3 (-81.9 to -58.2)	-67.9 (-77.6 to -55.1)	nan (nan–nan)	-68.0 (-87.9 to -40.6)	-75.6 (-87.9 to -55.3)	-83.3 (-92.5 to -69.4)	-86.8 (-91.3 to -81.1)
Cyprus	Rate: 1990	271.3 (260.6–281.8)	60.7 (42.5–78.7)	21.5 (13.0–31.8)	21.8 (16.5–27.7)	5.7 (4.0–7.8)	47.8 (32.8–61.1)	0.0 (0.0–0.0)	12.7 (6.1–23.2)	35.8 (19.6–55.2)	0.1 (0.1–0.2)	4.0 (2.9–5.1)
Cyprus	Rate: 2015	97.8 (87.0–110.4)	16.0 (11.5–21.0)	7.5 (4.6–10.8)	4.4 (3.6–5.4)	2.6 (2.0–3.3)	24.9 (21.2–29.3)	0.0 (0.0–0.0)	6.1 (3.2–10.7)	13.3 (8.5–19.3)	0.0 (0.0–0.0)	0.8 (0.7–1.0)
Cyprus	Rate: Cumulative change (%)	-63.9 (-68.0 to -59.2)	-72.8 (-82.4 to -59.3)	-62.3 (-80.8 to -35.1)	-79.2 (-85.7 to -71.1)	-52.1 (-69.9 to -30.1)	-46.1 (-62.4 to -24.7)	nan (nan–nan)	-45.9 (-79.7 to 0.6)	-58.7 (-79.5 to -24.2)	-72.3 (-87.7 to -49.4)	-78.1 (-85.6 to -68.6)
Denmark	Number: 1990	562 (540–586)	140 (123–160)	56 (47–67)	13 (11–15)	2 (2–2)	205 (166–226)	0 (0–0)	2 (1–3)	23 (19–29)	0 (0–0)	17 (15–20)
Denmark	Number: 2015	209 (183–241)	57 (44–69)	17 (12–21)	5 (4–6)	3 (3–4)	68 (56–91)	0 (0–0)	1 (1–2)	8 (6–11)	0 (0–0)	2 (2–3)
Denmark	Number: Cumulative change (%)	-62.7 (-67.6 to -57.0)	-59.0 (-71.6 to -48.9)	-69.1 (-78.4 to -59.4)	-60.8 (-71.2 to -48.7)	55.6 (17.9–96.4)	-66.0 (-73.9 to -44.4)	nan (nan–nan)	-27.5 (-51.5 to -0.9)	-61.9 (-74.1 to -47.6)	-62.1 (-80.2 to -34.6)	-84.1 (-87.7 to -80.0)
Denmark	Rate: 1990	188.4 (180.9–196.4)	46.5 (41.0–53.2)	18.7 (15.7–22.2)	4.5 (3.8–5.3)	0.9 (0.7–1.0)	68.8 (55.7–75.6)	0.0 (0.0–0.0)	0.8 (0.6–1.2)	7.9 (6.4–9.8)	0.1 (0.1–0.1)	6.1 (5.2–7.1)
Denmark	Rate: 2015	72.5 (63.3–83.4)	19.9 (15.7–24.2)	6.0 (4.5–7.4)	1.8 (1.4–2.2)	1.3 (1.1–1.6)	23.8 (19.5–31.8)	0.0 (0.0–0.0)	0.6 (0.4–0.9)	3.1 (2.3–4.0)	0.0 (0.0–0.0)	1.0 (0.8–1.2)
Denmark	Rate: Cumulative change (%)	-61.5 (-66.6 to -55.5)	-56.8 (-70.1 to -46.2)	-67.5 (-77.2 to -57.3)	-60.2 (-70.7 to -48.0)	58.0 (19.8–99.9)	-64.8 (-72.9 to -42.3)	nan (nan–nan)	-23.6 (-49.0 to 4.5)	-59.9 (-72.7 to -44.8)	-61.8 (-79.9 to -34.4)	-84.1 (-87.7 to -80.0)
Finland	Number: 1990	443 (419–470)	99 (85–115)	27 (22–34)	15 (13–17)	1 (1–2)	163 (148–178)	0 (0–0)	4 (2–6)	36 (28–44)	0 (0–0)	6 (5–7)
Finland	Number: 2015	140 (115–171)	19 (13–25)	9 (6–12)	1 (1–2)	0 (0–0)	57 (45–75)	0 (0–0)	2 (1–3)	12 (8–16)	0 (0–0)	1 (0–1)
Finland	Number: Cumulative change (%)	-68.3 (-74.3 to -61.1)	-79.8 (-87.1 to -72.9)	-65.6 (-77.4 to -50.9)	-87.4 (-91.0 to -83.2)	-71.9 (-80.8 to -61.2)	-64.6 (-72.7 to -51.5)	nan (nan–nan)	-39.2 (-64.0 to -10.4)	-65.6 (-77.1 to -51.6)	-52.6 (-74.3 to -22.6)	-83.2 (-87.8 to -77.5)
Finland	Rate: 1990	142.8 (134.9–151.4)	32.3 (27.6–37.3)	9.0 (7.2–11.2)	4.9 (4.2–5.5)	0.6 (0.5–0.7)	52.6 (47.8–57.7)	0.0 (0.0–0.0)	1.4 (0.9–1.9)	11.7 (9.2–14.4)	0.0 (0.0–0.0)	2.0 (1.7–2.4)
Finland	Rate: 2015	49.0 (40.2–59.9)	7.0 (4.9–8.9)	3.3 (2.4–4.4)	0.7 (0.5–0.9)	0.2 (0.1–0.3)	20.1 (15.8–26.4)	0.0 (0.0–0.0)	0.9 (0.4–1.3)	4.3 (3.1–5.7)	0.0 (0.0–0.0)	0.4 (0.3–0.5)
Finland	Rate: Cumulative change (%)	-65.6 (-72.1 to -57.8)	-78.1 (-86.0 to -70.6)	-62.6 (-75.4 to -46.5)	-86.4 (-90.2 to -81.8)	-69.5 (-79.3 to -58.0)	-61.6 (-70.4 to -47.3)	nan (nan–nan)	-33.9 (-60.9 to -2.5)	-62.6 (-75.1 to -47.4)	-48.7 (-72.2 to -16.3)	-81.9 (-86.9 to -75.7)
France	Number: 1990	6,650 (6,527–6,772)	964 (864–1,078)	754 (662–848)	138 (119–156)	74 (64–85)	1,584 (1,343–1,713)	0 (0–0)	259 (195–399)	175 (148–205)	2 (2–3)	113 (101–126)
France	Number: 2015	2,575 (2,114–3,144)	217 (150–289)	260 (186–350)	45 (35–57)	36 (27–47)	749 (581–967)	0 (0–0)	112 (59–232)	284 (202–373)	2 (1–2)	23 (18–29)
France	Number: Cumulative change (%)	-61.3 (-68.3 to -52.7)	-77.3 (-84.7 to -69.6)	-65.2 (-76.2 to -52.3)	-66.9 (-75.3 to -56.3)	-50.2 (-63.8 to -32.9)	-52.2 (-64.4 to -29.8)	nan (nan–nan)	-57.8 (-74.8 to -29.4)	63.7 (12.5–121.8)	-27.3 (-55.5 to 9.6)	-79.1 (-83.9 to -73.6)
France	Rate: 1990	181.8 (178.4–185.2)	26.7 (23.9–29.9)	20.9 (18.3–23.5)	3.8 (3.3–4.2)	2.0 (1.8–2.3)	43.6 (36.9–47.1)	0.0 (0.0–0.0)	7.2 (5.4–11.1)	4.8 (4.1–5.7)	0.1 (0.1–0.1)	3.1 (2.7–3.4)
France	Rate: 2015	67.0 (55.0–81.9)	5.7 (3.9–7.6)	6.9 (4.9–9.2)	1.2 (0.9–1.5)	1.0 (0.7–1.2)	19.5 (15.2–25.2)	0.0 (0.0–0.0)	2.9 (1.6–6.1)	7.5 (5.3–9.8)	0.1 (0.0–0.1)	0.6 (0.5–0.8)
France	Rate: Cumulative change (%)	-63.1 (-69.8 to -54.9)	-78.5 (-85.5 to -71.1)	-67.0 (-77.4 to -54.7)	-68.5 (-76.5 to -58.3)	-52.6 (-65.6 to -36.3)	-54.7 (-66.2 to -33.4)	nan (nan–nan)	-59.9 (-76.2 to -33.0)	55.5 (6.8–110.7)	-30.6 (-57.6 to 4.9)	-80.1 (-84.7 to -74.8)
Germany	Number: 1990	7,681 (7,545–7,823)	2,134 (1,941–2,380)	453 (378–539)	163 (142–186)	20 (17–24)	2,088 (1,441–2,309)	0 (0–0)	199 (122–399)	206 (164–255)	0 (0–1)	122 (107–139)
Germany	Number: 2015	2,411 (2,108–2,773)	532 (422–651)	178 (140–224)	42 (34–51)	17 (13–21)	797 (678–941)	0 (0–0)	87 (46–194)	129 (100–162)	0 (0–0)	22 (18–26)
Germany	Number: Cumulative change (%)	-68.6 (-72.6 to -63.8)	-75.0 (-81.0 to -69.2)	-60.1 (-70.7 to -48.0)	-73.7 (-79.4 to -67.4)	-15.8 (-35.6 to 7.9)	-61.1 (-69.2 to -41.4)	nan (nan–nan)	-56.9 (-70.4 to -40.0)	-36.0 (-54.8 to -14.2)	-33.0 (-61.1 to 4.9)	-81.7 (-85.3 to -77.7)
Germany	Rate: 1990	189.1 (185.7–192.6)	53.2 (48.4–59.3)	11.3 (9.4–13.4)	4.0 (3.5–4.5)	0.5 (0.4–0.6)	51.5 (35.6–57.0)	0.0 (0.0–0.0)	5.0 (3.1–9.9)	5.1 (4.1–6.4)	0.0 (0.0–0.0)	3.0 (2.6–3.4)
Germany	Rate: 2015	72.2 (63.2–83.1)	16.0 (12.7–19.6)	5.4 (4.2–6.8)	1.3 (1.0–1.5)	0.5 (0.4–0.6)	23.9 (20.3–28.2)	0.0 (0.0–0.0)	2.6 (1.4–5.8)	3.9 (3.0–4.9)	0.0 (0.0–0.0)	0.7 (0.5–0.8)
Germany	Rate: Cumulative change (%)	-61.8 (-66.7 to -55.9)	-69.8 (-77.2 to -62.9)	-51.8 (-64.7 to -37.2)	-67.8 (-74.8 to -60.0)	3.2 (-21.0 to 32.3)	-52.7 (-62.7 to -28.9)	nan (nan–nan)	-48.1 (-64.3 to -27.6)	-22.8 (-45.5 to 3.5)	-17.9 (-52.4 to 28.8)	-77.4 (-81.8 to -72.5)
Greece	Number: 1990	1,108 (1,078–1,135)	348 (312–448)	84 (70–112)	42 (33–75)	1 (0–1)	411 (163–462)	0 (0–0)	21 (5–33)	9 (7–12)	0 (0–0)	7 (6–13)
Greece	Number: 2015	310 (288–334)	79 (69–92)	18 (14–23)	12 (9–18)	0 (0–0)	139 (95–159)	0 (0–0)	3 (2–6)	7 (5–9)	0 (0–0)	1 (1–2)
Greece	Number: Cumulative change (%)	-72.0 (-74.1 to -69.7)	-76.9 (-80.6 to -73.3)	-78.3 (-83.3 to -72.9)	-69.7 (-76.8 to -62.0)	-66.1 (-74.7 to -56.2)	-64.6 (-71.3 to -41.0)	nan (nan–nan)	-74.7 (-91.6 to -37.4)	-24.6 (-44.3 to -0.7)	-92.4 (-95.3 to -88.9)	-76.0 (-81.1 to -69.4)
Greece	Rate: 1990	214.8 (209.2–220.1)	68.8 (61.7–88.5)	16.6 (13.9–22.1)	8.1 (6.5–14.5)	0.2 (0.2–0.4)	79.9 (31.6–89.8)	0.0 (0.0–0.0)	4.3 (1.0–6.6)	1.9 (1.5–2.5)	0.1 (0.1–0.2)	1.5 (1.2–2.5)
Greece	Rate: 2015	67.4 (62.7–72.7)	17.9 (15.5–20.6)	4.0 (3.2–5.1)	2.7 (2.1–4.0)	0.1 (0.1–0.1)	30.3 (20.8–34.7)	0.0 (0.0–0.0)	0.8 (0.5–1.4)	1.6 (1.2–2.1)	0.0 (0.0–0.0)	0.4 (0.3–0.6)
Greece	Rate: Cumulative change (%)	-68.6 (-71.0 to -66.1)	-73.9 (-78.0 to -69.8)	-75.4 (-81.1 to -69.3)	-66.1 (-74.1 to -57.2)	-62.1 (-71.8 to -50.9)	-60.3 (-67.8 to -33.6)	nan (nan–nan)	-71.4 (-90.5 to -29.3)	-14.7 (-37.1 to 12.3)	-91.6 (-94.8 to -87.7)	-73.3 (-78.9 to -65.7)
Iceland	Number: 1990	28 (26–31)	6 (5–7)	2 (1–2)	2 (2–2)	0 (0–0)	7 (6–8)	0 (0–0)	0 (0–1)	0 (0–0)	0 (0–0)	0 (0–0)
Iceland	Number: 2015	8 (7–10)	1 (0–1)	0 (0–0)	0 (0–0)	0 (0–0)	3 (2–4)	0 (0–0)	0 (0–0)	0 (0–0)	0 (0–0)	0 (0–0)
Iceland	Number: Cumulative change (%)	-69.0 (-73.2 to -64.0)	-80.7 (-86.4 to -75.7)	-73.5 (-80.3 to -65.3)	-72.3 (-78.7 to -65.0)	-27.1 (-48.2 to -1.9)	-57.4 (-65.9 to -46.2)	nan (nan–nan)	-51.8 (-67.2 to -30.7)	-6.3 (-39.6 to 29.8)	-57.2 (-73.5 to -32.8)	-83.5 (-87.4 to -78.4)
Iceland	Rate: 1990	134.4 (124.1–145.8)	29.9 (26.0–34.0)	9.6 (7.9–11.5)	11.0 (9.6–12.5)	0.6 (0.5–0.7)	36.5 (32.7–41.0)	0.0 (0.0–0.0)	3.0 (2.0–5.3)	1.5 (1.2–1.9)	0.0 (0.0–0.0)	2.9 (2.5–3.4)
Iceland	Rate: 2015	41.3 (36.1–47.5)	5.8 (3.9–7.2)	2.5 (1.9–3.2)	3.0 (2.4–3.7)	0.4 (0.3–0.5)	15.4 (12.5–19.2)	0.0 (0.0–0.0)	1.5 (0.9–3.0)	1.4 (1.0–1.9)	0.0 (0.0–0.0)	0.5 (0.4–0.6)
Iceland	Rate: Cumulative change (%)	-69.2 (-73.4 to -64.1)	-80.6 (-86.3 to -75.5)	-73.3 (-80.1 to -65.0)	-72.6 (-78.9 to -65.3)	-27.7 (-48.7 to -2.8)	-57.6 (-66.0 to -46.4)	nan (nan–nan)	-51.5 (-67.0 to -30.2)	-5.6 (-39.2 to 30.8)	-57.8 (-73.9 to -33.8)	-83.8 (-87.6 to -78.9)
Ireland	Number: 1990	497 (477–518)	104 (92–120)	35 (29–42)	22 (19–25)	1 (1–1)	167 (134–184)	0 (0–0)	4 (2–8)	8 (6–10)	0 (0–0)	8 (7–9)
Ireland	Number: 2015	242 (221–266)	35 (29–42)	13 (10–16)	8 (7–10)	2 (2–3)	101 (88–115)	0 (0–0)	4 (3–7)	11 (9–14)	0 (0–0)	2 (2–3)
Ireland	Number: Cumulative change (%)	-51.2 (-56.1 to -46.0)	-65.9 (-73.6 to -58.2)	-61.0 (-70.9 to -48.8)	-61.2 (-68.8 to -51.1)	66.0 (23.9–115.7)	-39.0 (-49.3 to -20.1)	nan (nan–nan)	4.1 (-31.3 to 47.6)	37.8 (-0.1 to 83.0)	-58.3 (-74.6 to -33.8)	-68.4 (-75.0 to -61.5)
Ireland	Rate: 1990	194.8 (187.1–203.0)	42.1 (37.2–48.7)	14.2 (11.9–16.9)	8.5 (7.4–9.7)	0.6 (0.5–0.7)	66.1 (53.2–72.9)	0.0 (0.0–0.0)	1.8 (1.2–3.5)	3.4 (2.8–4.1)	0.0 (0.0–0.0)	3.2 (2.7–3.7)
Ireland	Rate: 2015	71.7 (65.5–78.9)	10.7 (8.8–12.7)	4.1 (3.3–5.0)	2.5 (2.1–3.0)	0.7 (0.6–0.9)	30.1 (26.3–34.4)	0.0 (0.0–0.0)	1.3 (0.9–2.1)	3.5 (2.7–4.4)	0.0 (0.0–0.0)	0.8 (0.6–0.9)
Ireland	Rate: Cumulative change (%)	-63.2 (-66.8 to -59.2)	-74.5 (-80.2 to -68.7)	-70.8 (-78.2 to -61.6)	-70.5 (-76.3 to -62.8)	26.1 (-6.0 to 63.9)	-54.1 (-61.9 to -39.9)	nan (nan–nan)	-22.2 (-48.6 to 10.4)	3.5 (-25.2 to 38.0)	-67.8 (-80.5 to -48.8)	-75.5 (-80.6 to -70.3)
Israel	Number: 1990	1,213 (1,175–1,252)	325 (292–369)	70 (57–85)	61 (53–72)	8 (7–10)	336 (219–372)	0 (0–0)	26 (16–53)	46 (38–57)	1 (0–1)	17 (14–20)
Israel	Number: 2015	638 (590–693)	88 (68–106)	32 (25–39)	18 (15–21)	8 (6–10)	243 (209–273)	0 (0–0)	20 (11–45)	56 (45–68)	1 (1–1)	6 (5–7)

© 2017 Global Burden of Disease Child and Adolescent Health Collaboration. JAMA Pediatrics.

eTable 2. Number of Deaths, Death Rates (per 100,000 population), and Cumulative Percent Change with 95% Uncertainty Intervals (UI) for the Top 10 Global Causes of Death in 195 Countries and Territories, Aged Under 5 Years, Both Sexes, 1990 and 2015*Note: best viewed by enlarging in browser.*

Location	Measure	All causes	Neonatal preterm birth complications	Neonatal encephalopathy due to birth asphyxia and trauma	Lower respiratory infections	Diarrheal diseases	Congenital anomalies	Malaria	Neonatal sepsis and other neonatal infections	Other neonatal disorders	Protein-energy malnutrition	Meningitis
Israel	Number: Cumulative change (%)	-47.4 (-51.8 to -42.8)	-72.6 (-79.5 to -66.4)	-53.4 (-65.8 to -39.8)	-70.0 (-75.9 to -63.3)	-1.2 (-24.5 to 26.6)	-26.1 (-41.3 to 10.8)	nan (nan–nan)	-22.0 (-44.8 to 7.0)	22.2 (-11.0 to 58.1)	35.8 (-10.1 to 91.3)	-63.2 (-69.8 to -54.6)
Israel	Rate: 1990	250.3 (242.4–258.2)	67.0 (60.3–76.1)	14.5 (11.9–17.6)	12.7 (11.1–15.0)	1.8 (1.5–2.2)	69.3 (45.3–76.8)	0.0 (0.0–0.0)	5.5 (3.3–10.9)	9.7 (7.8–11.8)	0.2 (0.2–0.3)	3.6 (3.1–4.2)
Israel	Rate: 2015	78.4 (72.6–85.1)	11.0 (8.4–13.2)	4.0 (3.1–4.9)	2.2 (1.9–2.7)	1.0 (0.9–1.2)	29.9 (25.6–33.6)	0.0 (0.0–0.0)	2.6 (1.5–5.6)	7.0 (5.6–8.5)	0.2 (0.1–0.2)	0.8 (0.7–0.9)
Israel	Rate: Cumulative change (%)	-68.7 (-71.3 to -65.9)	-83.6 (-87.7 to -79.9)	-72.1 (-79.5 to -63.9)	-82.2 (-85.7 to -78.2)	-41.4 (-55.1 to -24.9)	-56.0 (-65.0 to -33.9)	nan (nan–nan)	-53.3 (-67.0 to -35.9)	-26.8 (-46.7 to -5.2)	-19.6 (-46.8 to 13.2)	-78.2 (-82.1 to -73.1)
Italy	Number: 1990	5,339 (5,231–5,450)	1,737 (1,605–1,889)	682 (599–771)	222 (193–267)	15 (12–18)	1,478 (1,150–1,598)	0 (0–0)	91 (40–122)	212 (181–249)	0 (0–1)	51 (44–61)
Italy	Number: 2015	1,579 (1,331–1,880)	331 (259–413)	133 (102–169)	30 (24–37)	10 (7–13)	528 (423–662)	0 (0–0)	46 (25–65)	198 (154–255)	1 (1–2)	9 (7–11)
Italy	Number: Cumulative change (%)	-70.4 (-75.1 to -64.7)	-80.9 (-85.5 to -75.7)	-80.4 (-85.6 to -74.1)	-86.1 (-89.6 to -82.1)	-30.3 (-49.7 to -5.8)	-63.7 (-72.3 to -43.1)	nan (nan–nan)	-47.1 (-63.0 to -26.9)	-5.4 (-30.7 to 29.5)	125.1 (41.9–239.2)	-82.0 (-86.3 to -77.0)
Italy	Rate: 1990	195.5 (191.4–199.6)	64.2 (59.4–69.9)	25.2 (22.2–28.5)	8.1 (7.0–9.7)	0.6 (0.5–0.7)	54.1 (42.1–58.5)	0.0 (0.0–0.0)	3.4 (1.5–4.5)	7.8 (6.7–9.2)	0.0 (0.0–0.0)	1.9 (1.6–2.2)
Italy	Rate: 2015	64.0 (54.0–76.2)	13.6 (10.7–17.0)	5.5 (4.2–7.0)	1.2 (1.0–1.5)	0.4 (0.3–0.5)	21.4 (17.2–26.9)	0.0 (0.0–0.0)	1.9 (1.1–2.7)	8.2 (6.3–10.5)	0.1 (0.1–0.1)	0.4 (0.3–0.5)
Italy	Rate: Cumulative change (%)	-67.3 (-72.5 to -60.9)	-78.7 (-83.9 to -73.1)	-78.2 (-84.0 to -71.2)	-84.7 (-88.5 to -80.3)	-22.8 (-44.4 to 4.6)	-59.8 (-69.3 to -37.0)	nan (nan–nan)	-41.2 (-58.9 to -18.8)	5.2 (-23.0 to 44.0)	149.4 (57.2–276.0)	-80.2 (-84.9 to -74.7)
Luxembourg	Number: 1990	42 (39–45)	6 (5–7)	5 (4–6)	2 (1–2)	0 (0–0)	7 (6–8)	0 (0–0)	0 (0–1)	3 (2–3)	0 (0–0)	0 (0–0)
Luxembourg	Number: 2015	16 (14–18)	1 (1–1)	1 (0–1)	0 (0–0)	0 (0–0)	4 (3–5)	0 (0–0)	0 (0–0)	2 (1–2)	0 (0–0)	0 (0–0)
Luxembourg	Number: Cumulative change (%)	-61.7 (-66.8 to -56.2)	-76.4 (-82.8 to -70.0)	-79.5 (-84.5 to -73.6)	-63.6 (-71.9 to -53.5)	41.9 (5.2–85.7)	-43.9 (-56.6 to -17.7)	nan (nan–nan)	-44.8 (-60.6 to -24.4)	-34.3 (-51.2 to -15.3)	-47.6 (-66.8 to -22.6)	-78.6 (-83.2 to -73.2)
Luxembourg	Rate: 1990	187.0 (174.2–201.0)	26.8 (23.2–30.6)	23.3 (20.1–26.8)	9.1 (7.7–10.6)	1.9 (1.6–2.3)	34.4 (29.5–38.5)	0.0 (0.0–0.0)	3.3 (2.1–6.1)	14.5 (12.0–17.3)	0.1 (0.1–0.1)	2.4 (2.0–2.8)
Luxembourg	Rate: 2015	52.7 (46.7–59.8)	4.6 (3.4–5.8)	3.5 (2.7–4.4)	2.4 (1.9–2.9)	2.0 (1.6–2.4)	14.1 (11.5–18.5)	0.0 (0.0–0.0)	1.4 (0.8–2.8)	7.0 (5.4–8.6)	0.0 (0.0–0.1)	0.4 (0.3–0.4)
Luxembourg	Rate: Cumulative change (%)	-71.7 (-75.5 to -67.7)	-82.5 (-87.2 to -77.8)	-84.9 (-88.5 to -80.4)	-73.3 (-79.4 to -65.9)	4.3 (-22.7 to 36.2)	-58.6 (-68.0 to -39.2)	nan (nan–nan)	-59.1 (-70.9 to -44.0)	-51.4 (-63.9 to -37.3)	-61.6 (-75.7 to -43.4)	-84.3 (-87.7 to -80.4)
Malta	Number: 1990	55 (51–58)	19 (17–21)	4 (3–4)	3 (2–3)	0 (0–0)	18 (15–20)	0 (0–0)	0 (0–1)	0 (0–0)	0 (0–0)	0 (0–0)
Malta	Number: 2015	23 (20–26)	6 (5–7)	1 (1–1)	0 (0–1)	0 (0–0)	9 (7–10)	0 (0–0)	0 (0–0)	1 (0–1)	0 (0–0)	0 (0–0)
Malta	Number: Cumulative change (%)	-57.6 (-62.7 to -52.0)	-65.5 (-71.4 to -59.4)	-64.5 (-73.0 to -54.3)	-71.7 (-77.5 to -65.2)	25.2 (-5.0 to 59.8)	-47.5 (-55.3 to -38.3)	nan (nan–nan)	-47.8 (-59.9 to -33.6)	35.9 (-0.3 to 82.2)	-67.3 (-79.4 to -49.0)	-83.5 (-86.7 to -79.8)
Malta	Rate: 1990	198.4 (185.2–212.5)	69.9 (63.4–77.0)	14.6 (12.2–17.1)	11.7 (10.1–13.4)	1.2 (0.9–1.4)	65.2 (55.3–72.6)	0.0 (0.0–0.0)	2.3 (1.6–4.2)	2.9 (2.3–3.5)	0.1 (0.1–0.1)	2.2 (1.9–2.6)
Malta	Rate: 2015	128.4 (114.8–144.3)	36.6 (30.7–43.7)	7.8 (6.2–9.7)	5.1 (4.2–6.0)	2.2 (1.8–2.7)	52.2 (42.2–60.4)	0.0 (0.0–0.0)	1.8 (1.2–3.3)	5.9 (4.6–7.4)	0.0 (0.0–0.1)	0.6 (0.5–0.7)
Malta	Rate: Cumulative change (%)	-35.2 (-42.9 to -26.7)	-47.6 (-56.5 to -38.3)	-46.0 (-59.0 to -30.4)	-56.5 (-65.5 to -46.6)	92.0 (45.7–145.2)	-19.8 (-31.8 to -5.7)	nan (nan–nan)	-20.6 (-38.9 to 1.0)	106.8 (51.7–177.3)	-49.6 (-68.4 to -21.3)	-74.7 (-79.4 to -68.9)
Netherlands	Number: 1990	1,658 (1,610–1,707)	351 (313–397)	187 (162–215)	38 (33–44)	4 (3–5)	531 (449–577)	0 (0–0)	39 (17–53)	51 (42–60)	0 (0–0)	46 (40–52)
Netherlands	Number: 2015	693 (628–769)	108 (91–127)	64 (52–78)	17 (14–20)	6 (5–7)	227 (195–281)	0 (0–0)	28 (13–38)	70 (56–85)	0 (0–0)	11 (9–13)
Netherlands	Number: Cumulative change (%)	-58.1 (-62.4 to -53.5)	-69.1 (-75.4 to -62.8)	-65.4 (-73.3 to -56.1)	-55.2 (-64.6 to -44.5)	54.4 (17.1–96.5)	-56.8 (-64.8 to -36.7)	nan (nan–nan)	-28.0 (-46.1 to -9.1)	40.1 (3.5–81.0)	-47.8 (-67.4 to -24.2)	-74.7 (-79.4 to -69.7)
Netherlands	Rate: 1990	179.0 (173.9–184.3)	37.9 (33.8–42.9)	20.2 (17.5–23.3)	4.2 (3.6–4.8)	0.5 (0.4–0.5)	57.3 (48.4–62.3)	0.0 (0.0–0.0)	4.3 (1.9–5.8)	5.5 (4.6–6.6)	0.1 (0.1–0.1)	5.0 (4.4–5.7)
Netherlands	Rate: 2015	79.9 (72.4–88.7)	12.5 (10.6–14.8)	7.5 (6.1–9.1)	2.0 (1.6–2.3)	0.7 (0.6–0.9)	26.2 (22.5–32.5)	0.0 (0.0–0.0)	3.3 (1.5–4.5)	8.2 (6.6–9.9)	0.0 (0.0–0.1)	1.3 (1.1–1.5)
Netherlands	Rate: Cumulative change (%)	-55.3 (-59.8 to -50.4)	-66.7 (-73.6 to -60.0)	-62.7 (-71.3 to -52.7)	-52.6 (-62.7 to -41.3)	63.4 (23.9–108.2)	-53.8 (-62.4 to -32.3)	nan (nan–nan)	-22.5 (-42.0 to -2.2)	50.8 (11.4–94.9)	-44.5 (-65.3 to -19.3)	-73.3 (-78.2 to -68.1)
Norway	Number: 1990	515 (494–537)	66 (56–76)	95 (84–107)	19 (17–22)	1 (1–2)	141 (129–154)	0 (0–0)	4 (2–6)	30 (25–35)	0 (0–0)	12 (10–14)
Norway	Number: 2015	165 (149–183)	19 (14–23)	22 (16–27)	4 (3–5)	2 (2–3)	58 (49–81)	0 (0–0)	2 (1–3)	11 (8–14)	0 (0–0)	1 (1–2)
Norway	Number: Cumulative change (%)	-68.0 (-71.3 to -64.3)	-70.1 (-79.8 to -62.5)	-76.4 (-82.8 to -70.9)	-76.0 (-81.8 to -69.6)	50.4 (13.9–93.1)	-58.2 (-66.4 to -38.7)	nan (nan–nan)	-45.9 (-63.3 to -22.0)	-61.2 (-72.9 to -48.6)	-3.6 (-40.5 to 50.3)	-84.2 (-87.6 to -80.3)
Norway	Rate: 1990	183.6 (176.2–191.4)	23.5 (20.2–27.3)	34.0 (30.0–38.0)	7.0 (6.1–8.0)	0.7 (0.6–0.8)	50.4 (45.9–55.0)	0.0 (0.0–0.0)	1.6 (0.8–2.2)	10.7 (8.9–12.6)	0.0 (0.0–0.0)	4.5 (3.8–5.3)
Norway	Rate: 2015	55.3 (50.0–61.4)	6.6 (4.9–7.9)	7.6 (5.6–9.1)	1.6 (1.2–1.9)	1.0 (0.7–1.2)	19.7 (16.5–27.2)	0.0 (0.0–0.0)	0.8 (0.5–1.1)	3.9 (2.8–4.8)	0.0 (0.0–0.0)	0.7 (0.5–0.8)
Norway	Rate: Cumulative change (%)	-69.9 (-73.0 to -66.4)	-71.6 (-80.8 to -64.5)	-77.6 (-83.7 to -72.4)	-77.5 (-83.0 to -71.5)	39.8 (5.9–79.4)	-60.6 (-68.4 to -42.2)	nan (nan–nan)	-48.8 (-65.2 to -26.0)	-63.2 (-74.3 to -51.3)	-10.2 (-44.6 to 39.9)	-85.4 (-88.5 to -81.7)
Portugal	Number: 1990	1,631 (1,609–1,653)	367 (324–446)	137 (116–168)	136 (120–169)	28 (23–35)	435 (224–486)	0 (0–0)	42 (13–60)	26 (21–34)	5 (4–6)	41 (36–50)
Portugal	Number: 2015	250 (237–265)	25 (21–29)	14 (11–17)	11 (9–13)	4 (3–4)	88 (78–97)	0 (0–0)	9 (5–12)	23 (19–28)	0 (0–1)	3 (2–3)
Portugal	Number: Cumulative change (%)	-84.6 (-85.4 to -83.7)	-93.0 (-94.9 to -91.7)	-89.6 (-92.2 to -86.9)	-91.7 (-93.4 to -89.7)	-85.3 (-89.0 to -81.5)	-78.6 (-83.0 to -58.5)	nan (nan–nan)	-74.4 (-84.2 to -55.6)	-9.8 (-34.2 to 16.8)	-85.2 (-90.1 to -79.3)	-92.3 (-93.7 to -90.7)
Portugal	Rate: 1990	290.9 (286.9–294.9)	67.1 (59.3–81.6)	25.0 (21.2–30.8)	24.1 (21.3–30.0)	5.0 (4.1–6.3)	78.1 (40.4–87.5)	0.0 (0.0–0.0)	7.7 (2.5–11.1)	4.9 (4.0–6.3)	0.9 (0.7–1.2)	7.2 (6.3–8.8)
Portugal	Rate: 2015	60.4 (57.2–64.0)	6.4 (5.4–7.4)	3.5 (2.8–4.3)	2.7 (2.3–3.2)	1.0 (0.8–1.1)	21.4 (19.1–23.7)	0.0 (0.0–0.0)	2.4 (1.4–3.1)	5.9 (4.8–7.0)	0.2 (0.1–0.2)	0.7 (0.6–0.9)
Portugal	Rate: Cumulative change (%)	-79.2 (-80.4 to -78.0)	-90.5 (-93.0 to -88.7)	-85.8 (-89.4 to -82.1)	-88.8 (-91.1 to -86.2)	-80.2 (-85.2 to -75.1)	-71.2 (-77.1 to -43.9)	nan (nan–nan)	-65.2 (-78.5 to -39.5)	22.9 (-10.4 to 58.9)	-80.1 (-86.6 to -72.0)	-89.6 (-91.4 to -87.4)
Spain	Number: 1990	3,845 (3,760–3,931)	839 (755–921)	333 (285–383)	149 (130–178)	29 (24–35)	1,169 (764–1,288)	0 (0–0)	160 (113–281)	101 (83–121)	2 (1–3)	107 (92–126)
Spain	Number: 2015	1,255 (1,132–1,399)	154 (116–187)	94 (72–115)	28 (23–34)	9 (7–10)	414 (353–489)	0 (0–0)	94 (64–162)	137 (104–170)	1 (0–1)	17 (14–20)
Spain	Number: Cumulative change (%)	-67.3 (-70.7 to -63.5)	-81.6 (-86.5 to -77.3)	-71.5 (-79.1 to -64.2)	-80.9 (-85.0 to -76.0)	-68.5 (-76.4 to -59.5)	-63.5 (-71.3 to -38.0)	nan (nan–nan)	-39.9 (-54.6 to -24.6)	37.3 (-1.6 to 77.9)	-56.5 (-72.8 to -35.4)	-83.9 (-87.0 to -80.4)
Spain	Rate: 1990	192.5 (188.3–196.7)	42.9 (38.7–47.2)	17.1 (14.6–19.6)	7.4 (6.5–8.8)	1.4 (1.2–1.8)	58.8 (38.5–64.8)	0.0 (0.0–0.0)	8.2 (5.8–14.4)	5.2 (4.3–6.2)	0.1 (0.1–0.2)	5.2 (4.5–6.1)
Spain	Rate: 2015	61.1 (55.1–68.1)	7.7 (5.8–9.3)	4.7 (3.6–5.8)	1.4 (1.1–1.6)	0.4 (0.4–0.5)	20.2 (17.2–23.9)	0.0 (0.0–0.0)	4.7 (3.2–8.0)	6.8 (5.2–8.5)	0.0 (0.0–0.1)	0.8 (0.7–1.0)
Spain	Rate: Cumulative change (%)	-68.2 (-71.5 to -64.5)	-82.1 (-86.9 to -78.0)	-72.3 (-79.7 to -65.2)	-81.5 (-85.5 to -76.8)	-69.5 (-77.2 to -60.7)	-64.5 (-72.1 to -39.7)	nan (nan–nan)	-41.7 (-55.9 to -26.7)	33.5 (-4.4 to 73.0)	-57.7 (-73.7 to -36.8)	-84.3 (-87.3 to -80.9)
Sweden	Number: 1990	824 (791–857)	141 (123–159)	116 (102–131)	23 (19–27)	1 (1–1)	285 (259–329)	0 (0–0)	17 (6–23)	24 (20–29)	0 (0–0)	10 (8–11)
Sweden	Number: 2015	307 (279–339)	37 (24–45)	33 (23–41)	7 (4–9)	2 (1–3)	113 (94–177)	0 (0–0)	9 (4–13)	28 (19–35)	0 (0–0)	2 (1–2)
Sweden	Number: Cumulative change (%)	-62.7 (-66.4 to -58.7)	-73.6 (-82.4 to -67.1)	-70.8 (-79.5 to -63.6)	-68.4 (-75.9 to -60.4)	79.1 (36.7–122.0)	-60.3 (-67.4 to -46.1)	nan (nan–nan)	-42.7 (-57.9 to -21.5)	15.8 (-16.8 to 52.8)	-37.1 (-58.4 to -10.7)	-78.1 (-82.3 to -73.1)
Sweden	Rate: 1990	146.9 (141.1–152.8)	25.0 (21.9–28.3)	20.6 (18.2–23.3)	4.3 (3.5–4.9)	0.3 (0.2–0.3)	50.7 (46.0–58.6)	0.0 (0.0–0.0)	3.0 (1.2–4.2)	4.4 (3.7–5.2)	0.0 (0.0–0.0)	1.8 (1.6–2.1)
Sweden	Rate: 2015	53.2 (48.4–58.8)	6.4 (4.3–7.8)	5.9 (4.1–7.1)	1.3 (0.9–1.6)	0.4 (0.3–0.5)	19.7 (16.4–30.7)	0.0 (0.0–0.0)	1.7 (0.7–2.4)	5.0 (3.4–6.1)	0.0 (0.0–0.0)	0.4 (0.3–0.5)
Sweden	Rate: Cumulative change (%)	-63.8 (-67.3 to -59.9)	-74.1 (-82.8 to -67.7)	-71.4 (-79.9 to -64.3)	-69.6 (-76.8 to -61.9)	71.8 (31.5–113.0)	-61.4 (-68.2 to -47.5)	nan (nan–nan)	-43.8 (-58.7 to -23.0)	13.5 (-18.5 to 49.7)	-39.0 (-59.6 to -13.5)	-79.0 (-83.0 to -74.2)
Switzerland	Number: 1990	698 (673–723)	150 (129–186)	53 (43–65)	22 (19–26)	6 (5–7)	260 (179–290)	0 (0–0)	4 (3–7)	34 (27–44)	0 (0–0)	14 (12–17)
Switzerland	Number: 2015	345 (301–396)	58 (48–72)	37 (29–47)	9 (7–10)	4 (3–5)	125 (106–145)	0 (0–0)	6 (2–8)	24 (19–31)	0 (0–0)	1 (1–2)
Switzerland	Number: Cumulative change (%)	-50.6 (-57.0 to -43.3)	-60.4 (-69.7 to -50.6)	-28.9 (-47.5 to -5.2)	-59.5 (-68.6 to -48.7)	-28.5 (-45.6 to -8.7)	-51.0 (-60.8 to -27.0)	nan (nan–nan)	51.1 (-34.5 to 144.9)	-27.1 (-49.2 to -0.9)	-54.5 (-72.5 to -30.3)	-87.4 (-89.9 to -84.7)
Switzerland	Rate: 1990	178.8 (172.6–185.4)	38.5 (33.2–47.8)	13.8 (11.2–16.7)	5.8 (5.0–6.7)	1.7 (1.5–2.0)	66.6 (46.1–74.2)	0.0 (0.0–0.0)	1.1 (0.8–1.9)	9.0 (7.1–11.3)	0.1 (0.0–0.1)	3.8 (3.3–4.4)
Switzerland	Rate: 2015	82.9 (72.5–95.1)	14.1 (11.6–17.3)	9.1 (7.2–11.3)	2.2 (1.8–2.6)	1.1 (0.9–1.4)	30.0 (25.6–34.8)	0.0 (0.0–0.0)	1.4 (0.7–2.1)	6.0 (4.7–7.4)	0.0 (0.0–0.0)	0.4 (0.4–0.5)
Switzerland	Rate: Cumulative change (%)	-53.6 (-59.7 to -46.8)	-62.9 (-71.6 to -53.8)	-33.4 (-50.8 to -11.2)	-62.0 (-70.6 to -51.9)	-33.0 (-49.0 to -14.5)	-54.1 (-63.3 to -31.6)	nan (nan–nan)	41.5 (-38.6 to 129.3)	-31.8 (-52.5 to -7.2)	-57.4 (-74.3 to -34.6)	-88.2 (-90.5 to -85.6)
United Kingdom	Number: 1990	7,402 (7,319–7,486)	1,962 (1,891–2,040)	789 (745–836)	332 (311–353)	29 (26–31)	1,718 (1,654–1,793)	0 (0–0)	116 (61–140)	150 (139–161)	1 (0–1)	185 (172–199)
United Kingdom	Number: 2015	3,809 (3,713–3,905)	1,198 (1,053–1,273)	233 (207–257)	151 (135–165)	30 (26–33)	1,066 (981–1,374)	0 (0–0)	60 (45–102)	126 (110–140)	0 (0–0)	51 (46–56)
United Kingdom	Number: Cumulative change (%)	-48.5 (-50.0 to -47.1)	-38.9 (-47.4 to -34.1)	-70.4 (-73.8 to -67.0)	-54.5 (-59.4 to -49.1)	3.4 (-8.5 to 16.3)	-38.0 (-43.5 to -23.2)	nan (nan–nan)	-45.0 (-64.0 to -9.6)	-15.8 (-27.5 to -3.9)	-46.4 (-55.2 to -37.0)	-72.2 (-75.0 to -69.0)
United Kingdom	Rate: 1990	195.3 (193.1–197.5)	52.0 (50.2–54.1)	20.9 (19.8–22.2)	8.7 (8.2–9.3)	0.8 (0.7–0.8)	45.4 (43.7–47.3)	0.0 (0.0–0.0)	3.1 (1.6–3.7)	4.0 (3.7–4.3)	0.0 (0.0–0.0)	4.9 (4.5–5.2)
United Kingdom	Rate: 2015	96.1 (93.6–98.5)	30.4 (26.7–32.3)	5.9 (5.3–6.5)	3.8 (3.4–4.1)	0.8 (0.7–0.8)	26.9 (24.8–34.7)	0.0 (0.0–0.0)	1.5 (1.2–2.6)	3.2 (2.8–3.6)	0.0 (0.0–0.0)	1.3 (1.2–1.4)
United Kingdom	Rate: Cumulative change (%)	-50.8 (-52.2 to -49.4)	-41.6 (-49.7 to -37.0)	-71.8 (-75.0 to -68.4)	-56.5 (-61.2 to -51.3)	-1.1 (-12.5 to 11.3)	-40.7 (-46.0 to -26.5)	nan (nan–nan)	-47.5 (-65.6 to -13.6)	-19.6 (-30.8 to -8.2)	-48.8 (-57.2 to -39.8)	-73.4 (-76.1 to -70.4)
England	Number: 1990	6,210 (6,134–6,291)	1,667 (1,605–1,736)	668 (626–711)	272 (252–293)	24 (22–26)	1,411 (1,352–1,489)	0 (0–0)	95 (51–117)	120 (109–130)	0 (0–0)	165 (152–178)
England	Number: 2015	3,284 (3,201–3,369)	1,062 (927–1,133)	197 (174–218)	124 (110–137)	21 (19–24)	906 (826–1,174)	0 (0–0)	48 (35–87)	103 (89–117)	0 (0–0)	46 (42–51)
England	Number: Cumulative change (%)	-47.1 (-48.7 to -45.5)	-36.2 (-45.2 to -31.0)	-70.4 (-74.0 to -66.6)	-54.2 (-59.7 to -48.0)	-11.8 (-23.3 to 1.4)	-35.8 (-41.8 to -20.0)	nan (nan–nan)	-46.1 (-66.3 to -8.4)	-13.5 (-26.5 to -0.0)	-40.7 (-50.9 to -29.2)	-71.7 (-74.8 to -68.4)
England	Rate: 1990	196.2 (193.8–198.8)	52.9 (50.9–55.1)	21.2 (19.9–22.6)	8.6 (8.0–9.2)	0.8 (0.7–0.8)	44.6 (42.8–47.1)	0.0 (0.0–0.0)	3.0 (1.6–3.7)	3.8 (3.5–4.1)	0.0 (0.0–0.0)	5.2 (4.8–5.6)
England	Rate: 2015	96.4 (94.0–98.9)	31.3 (27.3–33.4)	5.8 (5.2–6.4)	3.6 (3.2–4.0)	0.6 (0.6–0.7)	26.6 (24.3–34.5)	0.0 (0.0–0.0)	1.4 (1.0–2.6)	3.0 (2.6–3.4)	0.0 (0.0–0.0)	1.4 (1.2–1.5)
England	Rate: Cumulative change (%)	-50.9 (-52.3 to -49.4)	-40.8 (-49.1 to -35.9)	-72.5 (-75.8 to -69.0)	-57.4 (-62.6 to -51.7)	-17.9 (-28.7 to -5.7)	-40.4 (-46.0 to -25.6)	nan (nan–nan)	-50.0 (-68.8 to -14.9)	-19.7 (-31.8 to -7.2)	-44.9 (-54.4 to -34.2)	-73.7 (-76.6 to -70.6)
Northern Ireland	Number: 1990	238 (221–255)	65 (58–74)	20 (17–24)	19 (16–21)	0 (0–1)	78 (69–86)	0 (0–0)	5 (2–7)	3 (2–3)	0 (0–0)	2 (2–3)
Northern Ireland	Number: 2015	132 (107–161)	37 (28–47)	6 (4–8)	9 (7–11)	2 (1–3)	45 (35–57)	0 (0–0)	3 (1–4)	3 (2–4)	0 (0–0)	0 (0–1)
Northern Ireland	Number: Cumulative change (%)	-44.5 (-55.7 to -31.4)	-43.4 (-57.4 to -27.2)	-67.5 (-77.1 to -55.6)	-52.5 (-63.0 to -39.2)	194.7 (111.8–295.3)	-41.5 (-54.6 to -24.7)	nan (nan–nan)	-35.1 (-59.8 to -0.7)	13.6 (-22.8 to 60.6)	-67.5 (-80.1 to -52.0)	-71.5 (-78.2 to -63.8)
Northern Ireland	Rate: 1990	188.9 (175.4–202.6)	53.0 (47.0–59.8)	16.7 (14.0–19.6)	14.9 (13.0–16.9)	0.7 (0.6–0.8)	62.2 (55.0–68.7)	0.0 (0.0–0.0)	4.1 (1.7–5.7)	2.6 (2.1–3.2)	0.0 (0.0–0.0)	2.2 (1.9–2.6)
Northern Ireland	Rate: 2015	109.0 (88.8–133.7)	30.8 (23.6–39.4)	5.6 (4.1–7.4)	7.4 (5.9–9.0)	2.1 (1.6–2.7)	37.7 (29.7–47.2)	0.0 (0.0–0.0)	2.6 (1.4–3.6)	3.0 (2.2–4.1)	0.0 (0.0–0.0)	0.7 (0.5–0.8)
Northern Ireland	Rate: Cumulative change (%)	-42.2 (-53.7 to -28.5)	-41.6 (-56.0 to -24.8)	-66.4 (-76.3 to -54.1)	-50.2 (-61.3 to -36.3)	209.3 (122.2–316.8)	-39.1 (-52.8 to -21.6)	nan (nan–nan)	-33.0 (-58.5 to 2.5)	17.5 (-20.2 to 66.3)	-65.8 (-79.1 to -49.5)	-69.9 (-76.9 to -61.6)
Scotland	Number: 1990	616 (591–639)	137 (123–152)	66 (58–75)	26 (22–29)	2 (2–3)	151 (138–163)	0 (0–0)	11 (4–15)	19 (16–23)	0 (0–0)	8 (7–10)
Scotland	Number: 2015	244 (210–284)	54 (43–66)	20 (15–25)	11 (9–13)	4 (3–5)	71 (59–91)	0 (0–0)	6 (3–9)	14 (10–18)	0 (0–0)	1 (1–2)
Scotland	Number: Cumulative change (%)	-60.3 (-66.0 to -53.8)	-60.6 (-69.2 to -51.3)	-69.7 (-77.1 to -60.9)	-57.2 (-66.4 to -46.9)	91.1 (41.3–150.6)	-52.2 (-61.4 to -38.5)	nan (nan–nan)	-39.0 (-60.2 to -7.6)	-26.6 (-48.3 to -2.1)	-69.5 (-80.2 to -55.3)	-77.7 (-82.4 to -72.5)
Scotland	Rate: 1990	196.1 (188.2–203.5)	44.2 (39.8–49.0)	21.5 (18.8–24.3)	8.2 (7.2–9.4)	0.8 (0.7–0.9)	48.1 (44.2–52.1)	0.0 (0.0–0.0)	3.6 (1.6–4.9)	6.3 (5.3–7.4)	0.0 (0.0–0.1)	2.7 (2.3–3.1)
Scotland	Rate: 2015	89.8 (77.3–104.5)	20.1 (16.1–24.7)	7.5 (5.7–9.5)	4.0 (3.3–4.9)	1.7 (1.4–2.1)	26.4 (21.8–33.7)	0.0 (0.0–0.0)	2.4 (1.4–3.4)	5.3 (4.0–6.9)	0.0 (0.0–0.0)	0.7 (0.6–0.8)
Scotland	Rate: Cumulative change (%)	-54.2 (-60.8 to -46.6)	-54.4 (-64.3 to -43.6)	-64.8 (-73.4 to -54.7)	-50.7 (-61.3 to -38.8)	119.7 (62.2–188.4)	-44.9 (-55.5 to -29.0)	nan (nan–nan)	-29.3 (-53.8 to 7.1)	-14.9 (-40.0 to 13.6)	-64.9 (-77.3 to -48.6)	-74.4 (-79.7 to -68.4)
Wales	Number: 1990	336 (321–351)	90 (83–99)	33 (29–38)	14 (12–16)	1 (1–1)	77 (70–86)	0 (0–0)	4 (2–5)	7 (6–8)	0 (0–0)	8 (6–9)
Wales	Number: 2015	147 (137–159)	44 (37–50)	8 (7–10)	6 (5–7)	1 (0–1)	42 (36–55)	0 (0–0)	2 (1–4)	4 (3–5)	0 (0–0)	2 (1–2)
Wales	Number: Cumulative change (%)	-56.1 (-59.6 to -52.2)	-50.8 (-58.2 to -43.5)	-74.0 (-79.1 to -67.3)	-57.5 (-65.8 to -48.0)	-3.6 (-29.1 to 26.9)	-45.3 (-53.5 to -33.8)	nan (nan–nan)	-48.1 (-68.0 to -17.6)	-34.5 (-51.0 to -15.9)	-51.9 (-70.6 to -28.6)	-75.0 (-79.4 to -69.8)
Wales	Rate: 1990	181.9 (173.7–190.0)	49.5 (45.2–54.0)	18.3 (15.9–21.0)	7.9 (6.8–8.9)	0.7 (0.6–0.8)	42.0 (38.2–46.7)	0.0 (0.0–0.0)	2.2 (1.5–3.0)	4.0 (3.3–4.8)	0.0 (0.0–0.0)	4.4 (3.7–5.3)
Wales	Rate: 2015	89.4 (83.2–96.2)	27.4 (22.8–31.2)	5.3 (4.4–6.4)	3.7 (3.0–4.4)	0.7 (0.6–0.9)	25.7 (22.3–33.8)	0.0 (0.0–0.0)	1.3 (0.8–2.5)	2.9 (2.3–3.5)	0.0 (0.0–0.0)	1.2 (1.0–1.4)
Wales	Rate: Cumulative change (%)	-50.8 (-54.8 to -46.5)	-44.5 (-52.9 to -36.3)	-70.7 (-76.5 to -63.1)	-52.7 (-62.0 to -42.2)	7.8 (-20.8 to 42.3)	-38.7 (-48.0 to -25.8)	nan (nan–nan)	-41.5 (-64.0 to -7.1)	-26.3 (-44.8 to -5.3)	-46.5 (-67.3 to -20.6)	-72.3 (-77.3 to -66.6)
Southern Latin America	Number: 1990	26,778 (26,637–26,915)	7,143 (6,659–7,655)	1,866 (1,619–2,156)	2,993 (2,785–3,214)	952 (859–1,056)	4,819 (4,351–5,126)	0 (0–0)	1,171 (711–1,487)	1,078 (893–1,272)	776 (674–894)	576 (531–623)
Southern Latin America	Number: 2015	11,718 (11,362–12,080)	2,582 (2,331–2,837)	579 (499–660)	715 (627–808)	161 (137–187)	3,589 (3,242–3,887)	0 (0–0)	658 (493–903)	677 (582–778)	96 (78–117)	140 (122–161)
Southern Latin America	Number: Cumulative change (%)	-56.2 (-57.6 to -54.9)	-63.8 (-67.1 to -60.3)	-68.8 (-74.4 to -62.6)	-76.0 (-79.5 to -72.2)	-83.0 (-86.0 to -79.7)	-25.4 (-32.5 to -18.1)	-73.3 (-76.4 to -69.8)	-42.4 (-57.3 to -23.8)	-36.5 (-49.5 to -21.6)	-87.4 (-90.3 to -84.2)	-75.6 (-79.0 to -71.3)
Southern Latin America	Rate: 1990	526.0 (523.2–528.7)	140.3 (130.8–150.4)	36.7 (31.8–42.4)	58.7 (54.7–63.1)	18.7 (16.9–20.7)	94.6 (85.4–100.7)	0.0 (0.0–0.0)	23.0 (14.0–29.2)	21.2 (17.5–25.0)	15.2 (13.2–17.6)	11.3 (10.4–12.2)
Southern Latin America	Rate: 2015	232.0 (224.9–239.1)	51.4 (46.5–56.5)	11.5 (9.9–13.1)	14.1 (12.3–15.9)	3.2 (2.7–3.7)	71.1 (64.2–77.0)	0.0 (0.0–0.0)	13.1 (9.8–18.0)	13.5 (11.6–15.5)	1.9 (1.5–2.3)	2.8 (2.4–3.2)
Southern Latin America	Rate: Cumulative change (%)	-55.9 (-57.2 to -54.5)	-63.3 (-66.7 to -59.8)	-68.3 (-74.0 to -62.1)	-76.0 (-79.4 to -72.1)	-82.9 (-85.9 to -79.7)	-24.8 (-31.9 to -17.4)	-73.4 (-76.5 to -70.0)	-41.6 (-56.7 to -22.8)	-35.6 (-48.8 to -20.6)	-87.5 (-90.4 to -84.2)	-75.6 (-79.0 to -71.3)

© 2017 Global Burden of Disease Child and Adolescent Health Collaboration. JAMA Pediatrics.

eTable 2. Number of Deaths, Death Rates (per 100,000 population), and Cumulative Percent Change with 95% Uncertainty Intervals (UI) for the Top 10 Global Causes of Death in 195 Countries and Territories, Aged Under 5 Years, Both Sexes, 1990 and 2015 *Note: best viewed by enlarging in browser.*

Location	Measure	All causes	Neonatal preterm birth complications	Neonatal encephalopathy due to birth asphyxia and trauma	Lower respiratory infections	Diarrheal diseases	Congenital anomalies	Malaria	Neonatal sepsis and other neonatal infections	Other neonatal disorders	Protein-energy malnutrition	Meningitis
Argentina	Number: 1990	20,101 (19,975–20,226)	5,536 (5,087–5,985)	1,389 (1,156–1,665)	1,924 (1,741–2,112)	774 (682–877)	3,232 (2,830–3,512)	0 (0–0)	952 (541–1,249)	921 (744–1,104)	710 (606–828)	422 (380–464)
Argentina	Number: 2015	9,468 (9,158–9,812)	2,174 (1,957–2,415)	478 (402–560)	584 (503–668)	140 (118–165)	2,716 (2,404–3,001)	0 (0–0)	556 (402–748)	548 (458–643)	84 (66–104)	116 (100–137)
Argentina	Number: Cumulative change (%)	-52.9 (-54.5 to -51.2)	-60.7 (-64.6 to -56.2)	-65.2 (-72.8 to -56.2)	-69.5 (-74.6 to -63.8)	-81.7 (-85.3 to -77.7)	-15.7 (-25.8 to -5.8)	-73.3 (-76.4 to -69.8)	-39.6 (-57.2 to -18.7)	-39.7 (-53.8 to -23.1)	-88.0 (-91.1 to -84.4)	-72.2 (-76.8 to -66.6)
Argentina	Rate: 1990	585.5 (581.9–589.2)	161.0 (148.0–174.1)	40.4 (33.6–48.4)	56.1 (50.8–61.6)	22.6 (19.9–25.6)	94.1 (82.4–102.2)	0.0 (0.0–0.0)	27.7 (15.8–36.4)	26.8 (21.7–32.1)	20.7 (17.7–24.1)	12.3 (11.1–13.5)
Argentina	Rate: 2015	258.1 (249.6–267.5)	59.6 (53.7–66.2)	13.1 (11.0–15.4)	15.9 (13.7–18.2)	3.8 (3.2–4.5)	74.1 (65.5–81.9)	0.0 (0.0–0.0)	15.2 (11.0–20.5)	15.0 (12.5–17.6)	2.3 (1.8–2.8)	3.2 (2.7–3.7)
Argentina	Rate: Cumulative change (%)	-55.9 (-57.4 to -54.3)	-62.9 (-66.6 to -58.8)	-67.2 (-74.4 to -58.8)	-71.6 (-76.4 to -66.2)	-83.0 (-86.4 to -79.3)	-21.1 (-30.5 to -11.7)	-75.3 (-78.2 to -72.2)	-43.2 (-59.7 to -23.5)	-43.2 (-56.5 to -27.6)	-88.9 (-91.8 to -85.5)	-74.2 (-78.4 to -69.0)
Chile	Number: 1990	5,371 (5,314–5,427)	1,318 (1,202–1,448)	335 (270–402)	926 (858–999)	119 (102–138)	1,302 (1,177–1,404)	0 (0–0)	157 (114–231)	100 (80–125)	21 (16–25)	131 (117–147)
Chile	Number: 2015	1,784 (1,697–1,878)	337 (294–379)	71 (58–86)	94 (80–110)	10 (8–12)	720 (651–783)	0 (0–0)	68 (44–120)	97 (78–115)	6 (4–7)	21 (18–24)
Chile	Number: Cumulative change (%)	-66.8 (-68.5 to -65.0)	-74.3 (-78.2 to -70.8)	-78.4 (-83.5 to -72.3)	-89.8 (-91.5 to -87.7)	-91.3 (-93.1 to -89.1)	-44.6 (-50.7 to -38.1)	nan (nan–nan)	-56.7 (-70.7 to -38.6)	-2.1 (-27.9 to 27.3)	-69.6 (-78.6 to -59.7)	-83.8 (-86.6 to -80.4)
Chile	Rate: 1990	388.3 (384.1–392.3)	95.7 (87.3–105.1)	24.3 (19.7–29.2)	66.7 (61.9–72.0)	8.6 (7.3–10.0)	94.2 (85.2–101.6)	0.0 (0.0–0.0)	11.5 (8.3–16.8)	7.3 (5.9–9.1)	1.5 (1.2–1.9)	9.5 (8.4–10.6)
Chile	Rate: 2015	155.7 (148.2–164.0)	29.7 (25.9–33.4)	6.3 (5.1–7.6)	8.2 (7.0–9.6)	0.9 (0.7–1.1)	63.0 (56.9–68.4)	0.0 (0.0–0.0)	6.0 (3.9–10.6)	8.5 (6.9–10.2)	0.5 (0.4–0.7)	1.8 (1.6–2.2)
Chile	Rate: Cumulative change (%)	-59.9 (-61.9 to -57.7)	-68.9 (-73.5 to -64.6)	-73.8 (-80.1 to -66.4)	-87.7 (-89.8 to -85.2)	-89.5 (-91.7 to -86.9)	-33.0 (-40.4 to -25.2)	nan (nan–nan)	-47.5 (-64.5 to -25.5)	18.7 (-12.7 to 54.4)	-63.5 (-74.3 to -51.7)	-80.5 (-83.9 to -76.5)
Uruguay	Number: 1990	1,305 (1,260–1,352)	288 (260–316)	141 (120–162)	142 (126–158)	59 (50–69)	284 (259–317)	0 (0–0)	61 (39–78)	55 (45–66)	44 (35–54)	21 (18–24)
Uruguay	Number: 2015	466 (373–582)	70 (53–90)	29 (21–38)	36 (27–46)	10 (7–13)	151 (116–197)	0 (0–0)	33 (22–45)	32 (23–42)	6 (4–8)	2 (1–2)
Uruguay	Number: Cumulative change (%)	-64.3 (-71.5 to -55.5)	-75.6 (-81.5 to -68.4)	-79.1 (-84.8 to -71.6)	-74.3 (-81.1 to -66.1)	-82.1 (-87.4 to -75.5)	-46.6 (-59.1 to -31.7)	nan (nan–nan)	-43.8 (-58.7 to -25.9)	-41.0 (-58.7 to -19.3)	-86.3 (-91.2 to -80.3)	-90.3 (-93.1 to -87.2)
Uruguay	Rate: 1990	475.2 (459.1–492.4)	105.1 (94.9–115.0)	51.4 (43.9–59.1)	51.8 (45.9–57.7)	21.5 (18.3–25.2)	103.4 (94.5–115.7)	0.0 (0.0–0.0)	22.3 (14.3–28.5)	20.3 (16.5–24.3)	16.2 (12.9–19.9)	7.9 (6.9–8.9)
Uruguay	Rate: 2015	196.3 (156.9–245.4)	29.9 (22.7–38.4)	12.5 (9.2–16.5)	15.2 (11.3–19.6)	4.4 (3.2–5.8)	63.9 (49.1–83.3)	0.0 (0.0–0.0)	14.4 (9.4–19.3)	13.8 (10.1–18.1)	2.5 (1.7–3.5)	0.9 (0.7–1.1)
Uruguay	Rate: Cumulative change (%)	-58.7 (-67.1 to -48.5)	-71.5 (-78.4 to -63.1)	-75.6 (-82.3 to -66.9)	-70.5 (-78.2 to -61.1)	-79.4 (-85.5 to -71.8)	-38.1 (-52.6 to -21.0)	nan (nan–nan)	-34.4 (-51.8 to -13.6)	-31.2 (-51.8 to -5.9)	-84.3 (-89.9 to -77.5)	-88.9 (-92.1 to -85.3)
Central Europe, Eastern Europe, and Central Asia	Number: 1990	222,204 (217,750–226,709)	31,374 (29,169–33,573)	24,528 (21,903–27,024)	65,159 (62,773–67,525)	15,901 (15,036–16,840)	30,298 (26,623–31,974)	5 (4–7)	3,904 (3,145–5,600)	9,279 (8,143–10,642)	540 (450–615)	4,638 (4,274–4,967)
Central Europe, Eastern Europe, and Central Asia	Number: 2015	77,900 (73,411–82,691)	11,384 (10,175–12,661)	10,602 (9,246–12,012)	15,935 (14,112–17,813)	1,967 (1,619–2,349)	14,852 (13,588–16,063)	1 (0–2)	2,725 (2,167–3,470)	4,538 (3,856–5,380)	155 (100–271)	991 (863–1,197)
Central Europe, Eastern Europe, and Central Asia	Number: Cumulative change (%)	-64.9 (-67.0 to -62.6)	-63.7 (-67.7 to -59.4)	-56.7 (-62.7 to -49.9)	-75.5 (-78.6 to -72.6)	-87.6 (-89.9 to -85.0)	-50.8 (-56.2 to -41.7)	-79.8 (-90.8 to -62.4)	-29.2 (-43.7 to -12.9)	-50.8 (-60.2 to -39.2)	-71.1 (-81.4 to -50.6)	-78.5 (-81.9 to -73.2)
Central Europe, Eastern Europe, and Central Asia	Rate: 1990	671.2 (657.7–685.0)	97.2 (90.4–104.0)	76.0 (67.8–83.7)	195.1 (188.0–202.2)	47.6 (45.0–50.4)	92.2 (80.9–97.3)	0.0 (0.0–0.0)	12.1 (9.7–17.3)	28.7 (25.2–32.9)	1.6 (1.3–1.8)	13.8 (12.7–14.8)
Central Europe, Eastern Europe, and Central Asia	Rate: 2015	285.5 (269.1–303.1)	42.1 (37.6–46.8)	39.2 (34.2–44.4)	58.1 (51.5–65.0)	7.2 (5.9–8.6)	54.5 (49.8–58.9)	0.0 (0.0–0.0)	10.0 (8.0–12.8)	16.8 (14.2–19.9)	0.6 (0.4–1.0)	3.6 (3.1–4.4)
Central Europe, Eastern Europe, and Central Asia	Rate: Cumulative change (%)	-57.5 (-60.0 to -54.7)	-56.7 (-61.5 to -51.6)	-48.3 (-55.5 to -40.2)	-70.2 (-73.9 to -66.6)	-84.9 (-87.7 to -81.8)	-40.7 (-47.2 to -29.8)	-74.9 (-88.7 to -53.4)	-15.5 (-32.8 to 3.8)	-41.3 (-52.5 to -27.5)	-64.7 (-77.3 to -39.6)	-73.7 (-77.8 to -67.2)
Eastern Europe	Number: 1990	64,839 (62,748–66,995)	9,661 (8,581–10,595)	8,884 (8,003–9,783)	8,712 (8,122–9,400)	1,842 (1,643–2,061)	15,286 (12,187–16,525)	0 (0–0)	1,805 (1,274–3,380)	3,293 (2,901–3,738)	216 (176–262)	1,988 (1,818–2,178)
Eastern Europe	Number: 2015	21,951 (20,465–23,737)	2,390 (1,909–2,803)	2,082 (1,729–2,442)	1,790 (1,592–2,009)	191 (162–222)	6,182 (5,442–7,487)	0 (0–0)	1,318 (983–1,889)	1,584 (1,283–1,862)	25 (18–34)	364 (320–413)
Eastern Europe	Number: Cumulative change (%)	-66.1 (-68.7 to -63.3)	-75.2 (-79.9 to -71.1)	-76.5 (-80.6 to -71.9)	-79.4 (-82.3 to -76.6)	-89.6 (-91.5 to -87.6)	-59.0 (-65.4 to -37.8)	nan (nan–nan)	-22.4 (-48.7 to 0.3)	-51.7 (-61.1 to -42.5)	-88.1 (-91.8 to -82.9)	-81.6 (-84.2 to -78.7)
Eastern Europe	Rate: 1990	434.9 (421.2–449.0)	67.2 (59.7–73.8)	61.8 (55.7–68.1)	57.6 (53.7–62.1)	12.2 (10.9–13.7)	103.2 (82.1–111.7)	0.0 (0.0–0.0)	12.6 (8.9–23.5)	22.9 (20.2–26.0)	1.4 (1.1–1.7)	12.9 (11.8–14.1)
Eastern Europe	Rate: 2015	178.0 (165.9–192.5)	19.5 (15.6–22.9)	17.0 (14.1–19.9)	14.5 (12.9–16.2)	1.5 (1.3–1.8)	50.1 (44.2–60.7)	0.0 (0.0–0.0)	10.7 (8.0–15.4)	12.9 (10.5–15.2)	0.2 (0.2–0.3)	2.9 (2.6–3.3)
Eastern Europe	Rate: Cumulative change (%)	-59.1 (-62.2 to -55.6)	-70.9 (-76.5 to -66.0)	-72.4 (-77.2 to -67.0)	-74.8 (-78.4 to -71.4)	-87.3 (-89.6 to -84.9)	-50.8 (-58.5 to -25.3)	nan (nan–nan)	-9.0 (-39.9 to 17.6)	-43.3 (-54.4 to -32.4)	-85.4 (-89.9 to -79.0)	-77.2 (-80.4 to -73.5)
Belarus	Number: 1990	2,977 (2,578–3,425)	359 (246–494)	358 (239–493)	417 (343–503)	51 (40–65)	767 (546–943)	0 (0–0)	86 (41–160)	198 (100–333)	16 (11–22)	81 (67–98)
Belarus	Number: 2015	635 (505–799)	48 (29–73)	68 (40–103)	25 (18–34)	1 (1–2)	236 (168–322)	0 (0–0)	34 (17–58)	40 (19–67)	0 (0–1)	11 (8–15)
Belarus	Number: Cumulative change (%)	-78.5 (-83.5 to -72.3)	-85.8 (-92.9 to -75.7)	-79.9 (-89.6 to -66.7)	-93.8 (-95.9 to -91.3)	-97.0 (-98.2 to -95.5)	-67.9 (-79.8 to -46.1)	nan (nan–nan)	-53.8 (-82.2 to -6.0)	-76.4 (-92.0 to -50.3)	-94.8 (-97.3 to -90.6)	-85.9 (-90.5 to -79.7)
Belarus	Rate: 1990	424.8 (367.1–489.3)	52.9 (36.1–72.6)	52.7 (35.2–72.6)	59.2 (48.6–71.6)	7.3 (5.6–9.2)	110.3 (78.6–135.5)	0.0 (0.0–0.0)	12.7 (6.1–23.6)	29.1 (14.7–49.0)	2.3 (1.6–3.1)	11.4 (9.4–13.8)
Belarus	Rate: 2015	116.6 (92.6–146.6)	8.9 (5.4–13.5)	12.7 (7.5–19.1)	4.7 (3.4–6.3)	0.3 (0.2–0.4)	43.5 (31.0–59.1)	0.0 (0.0–0.0)	6.4 (3.2–10.8)	7.4 (3.6–12.4)	0.2 (0.1–0.2)	2.1 (1.5–2.9)
Belarus	Rate: Cumulative change (%)	-72.4 (-78.8 to -64.4)	-82.2 (-91.1 to -69.4)	-74.8 (-87.0 to -58.1)	-92.0 (-94.7 to -88.8)	-96.2 (-97.7 to -94.2)	-59.0 (-74.2 to -31.3)	nan (nan–nan)	-42.0 (-77.7 to 18.0)	-70.3 (-89.9 to -37.4)	-93.2 (-96.4 to -87.7)	-81.5 (-87.5 to -73.3)
Estonia	Number: 1990	375 (355–395)	27 (21–34)	31 (25–38)	35 (31–40)	4 (3–5)	85 (76–94)	0 (0–0)	14 (7–19)	58 (48–68)	2 (2–3)	9 (8–10)
Estonia	Number: 2015	46 (37–58)	3 (2–5)	3 (2–5)	2 (2–3)	0 (0–0)	16 (11–24)	0 (0–0)	3 (2–4)	2 (1–3)	0 (0–0)	0 (0–0)
Estonia	Number: Cumulative change (%)	-87.5 (-90.1 to -84.5)	-86.7 (-92.7 to -79.6)	-87.6 (-92.3 to -82.6)	-91.7 (-94.1 to -88.8)	-95.9 (-97.3 to -94.3)	-81.1 (-86.5 to -72.5)	nan (nan–nan)	-77.4 (-86.6 to -64.4)	-95.9 (-97.4 to -94.0)	-95.3 (-97.1 to -92.8)	-93.8 (-95.6 to -91.8)
Estonia	Rate: 1990	358.1 (339.2–377.0)	27.4 (21.6–34.3)	31.5 (25.4–38.1)	33.7 (29.6–38.0)	4.3 (3.5–5.3)	81.6 (73.1–91.4)	0.0 (0.0–0.0)	14.3 (7.5–19.6)	57.7 (48.5–68.1)	2.5 (2.0–3.1)	8.6 (7.6–9.7)
Estonia	Rate: 2015	67.2 (53.3–83.2)	5.2 (3.1–7.3)	5.7 (3.7–7.8)	4.2 (3.0–5.5)	0.3 (0.2–0.4)	23.1 (16.8–35.4)	0.0 (0.0–0.0)	4.5 (2.9–6.5)	3.5 (2.3–4.9)	0.2 (0.1–0.3)	0.8 (0.6–1.1)
Estonia	Rate: Cumulative change (%)	-81.2 (-85.1 to -76.6)	-80.4 (-89.3 to -70.0)	-81.7 (-88.6 to -74.3)	-87.4 (-91.1 to -83.1)	-93.8 (-95.9 to -91.3)	-71.7 (-79.8 to -58.9)	nan (nan–nan)	-66.8 (-80.2 to -47.6)	-93.9 (-96.1 to -91.2)	-92.8 (-95.6 to -89.0)	-90.6 (-93.2 to -87.5)
Latvia	Number: 1990	663 (632–696)	41 (32–50)	86 (72–100)	48 (42–54)	6 (5–8)	185 (168–202)	0 (0–0)	10 (6–14)	78 (65–92)	3 (2–5)	21 (18–24)
Latvia	Number: 2015	104 (75–138)	4 (2–6)	16 (10–23)	5 (3–7)	0 (0–0)	35 (23–53)	0 (0–0)	3 (1–5)	6 (3–9)	0 (0–0)	1 (0–1)
Latvia	Number: Cumulative change (%)	-84.3 (-88.6 to -79.0)	-89.7 (-94.5 to -84.2)	-81.0 (-88.1 to -71.8)	-89.3 (-92.8 to -84.7)	-94.8 (-96.7 to -92.6)	-81.0 (-87.7 to -71.0)	nan (nan–nan)	-66.6 (-80.0 to -49.7)	-91.9 (-95.1 to -87.9)	-91.4 (-95.4 to -86.1)	-95.3 (-96.7 to -93.4)
Latvia	Rate: 1990	368.9 (351.1–387.5)	23.8 (18.9–29.3)	49.7 (41.8–57.6)	26.8 (23.4–30.3)	3.8 (3.1–4.6)	103.8 (94.4–113.7)	0.0 (0.0–0.0)	5.9 (3.9–8.5)	45.2 (37.9–53.1)	2.2 (1.5–2.9)	11.7 (10.3–13.2)
Latvia	Rate: 2015	105.9 (77.0–140.5)	4.3 (2.5–6.4)	16.9 (10.6–24.4)	5.2 (3.6–7.2)	0.4 (0.2–0.5)	35.8 (24.0–54.9)	0.0 (0.0–0.0)	3.5 (2.0–5.5)	6.5 (4.0–9.5)	0.3 (0.2–0.5)	1.0 (0.7–1.4)
Latvia	Rate: Cumulative change (%)	-71.3 (-79.2 to -61.6)	-81.5 (-90.1 to -71.6)	-65.7 (-78.7 to -49.2)	-80.3 (-86.8 to -71.9)	-90.5 (-93.9 to -86.3)	-65.4 (-77.7 to -47.0)	nan (nan–nan)	-39.9 (-64.0 to -9.4)	-85.4 (-91.3 to -78.3)	-84.3 (-91.6 to -74.4)	-91.3 (-94.0 to -87.8)
Lithuania	Number: 1990	775 (746–804)	98 (84–112)	84 (71–97)	50 (44–57)	8 (7–10)	271 (242–295)	0 (0–0)	9 (5–19)	36 (29–43)	3 (2–5)	19 (17–22)
Lithuania	Number: 2015	122 (106–141)	10 (7–13)	9 (6–11)	8 (5–10)	0 (0–0)	47 (37–67)	0 (0–0)	6 (3–8)	5 (3–6)	0 (0–0)	1 (1–2)
Lithuania	Number: Cumulative change (%)	-84.2 (-86.3 to -81.7)	-89.4 (-93.0 to -86.2)	-89.0 (-92.2 to -85.6)	-83.6 (-88.7 to -79.0)	-92.8 (-95.3 to -90.1)	-82.2 (-86.0 to -72.6)	nan (nan–nan)	-22.1 (-75.4 to 32.4)	-85.6 (-90.2 to -80.2)	-95.7 (-97.7 to -92.8)	-91.5 (-93.9 to -89.3)
Lithuania	Rate: 1990	287.7 (277.2–298.9)	37.2 (31.9–42.6)	31.8 (27.2–36.7)	18.7 (16.3–21.3)	3.2 (2.6–3.9)	101.2 (90.3–110.0)	0.0 (0.0–0.0)	3.6 (2.1–7.2)	13.6 (11.0–16.4)	1.4 (0.9–1.9)	7.3 (6.4–8.3)
Lithuania	Rate: 2015	82.8 (72.1–95.7)	7.1 (5.0–9.2)	6.3 (4.6–8.1)	5.6 (4.0–6.9)	0.4 (0.3–0.5)	32.5 (25.7–45.9)	0.0 (0.0–0.0)	4.2 (2.2–5.8)	3.5 (2.6–4.5)	0.1 (0.1–0.2)	1.1 (0.8–1.4)
Lithuania	Rate: Cumulative change (%)	-71.2 (-75.1 to -66.5)	-80.7 (-87.2 to -74.8)	-80.0 (-85.8 to -73.7)	-70.0 (-79.4 to -61.8)	-86.8 (-91.4 to -81.8)	-67.6 (-74.4 to -50.1)	nan (nan–nan)	41.7 (-55.3 to 141.0)	-73.8 (-82.2 to -63.9)	-92.1 (-95.7 to -86.9)	-84.5 (-88.8 to -80.5)
Moldova	Number: 1990	2,490 (2,163–2,857)	212 (159–279)	406 (319–520)	507 (422–601)	90 (70–111)	508 (265–616)	0 (0–0)	119 (37–193)	49 (34–70)	2 (1–4)	63 (51–76)
Moldova	Number: 2015	462 (324–633)	45 (30–65)	43 (28–67)	71 (48–100)	6 (4–8)	140 (93–199)	0 (0–0)	24 (11–40)	23 (15–35)	0 (0–0)	5 (3–7)
Moldova	Number: Cumulative change (%)	-81.3 (-87.1 to -73.8)	-78.1 (-86.8 to -66.9)	-89.0 (-93.1 to -83.2)	-85.8 (-90.8 to -79.4)	-93.1 (-95.8 to -89.6)	-71.4 (-81.8 to -55.3)	nan (nan–nan)	-76.6 (-87.4 to -59.4)	-50.0 (-71.2 to -20.0)	-97.2 (-98.8 to -95.0)	-90.9 (-94.1 to -87.0)
Moldova	Rate: 1990	622.5 (541.0–713.6)	54.6 (41.0–71.8)	104.6 (82.3–134.1)	125.8 (105.0–149.4)	22.5 (17.7–27.9)	128.2 (66.8–155.7)	0.0 (0.0–0.0)	30.7 (9.7–49.9)	12.8 (9.0–18.1)	0.7 (0.4–1.1)	15.5 (12.7–18.6)
Moldova	Rate: 2015	218.6 (153.5–299.7)	21.8 (14.4–31.5)	21.1 (13.8–32.4)	33.6 (22.8–47.3)	2.9 (1.9–4.1)	66.5 (44.0–94.6)	0.0 (0.0–0.0)	11.9 (5.6–19.5)	11.5 (7.5–17.2)	0.0 (0.0–0.1)	2.6 (1.8–3.7)
Moldova	Rate: Cumulative change (%)	-64.6 (-75.6 to -50.5)	-59.0 (-75.3 to -38.1)	-79.5 (-87.1 to -68.6)	-73.0 (-82.5 to -61.0)	-86.9 (-92.1 to -80.3)	-46.3 (-65.8 to -16.1)	nan (nan–nan)	-56.3 (-76.5 to -24.2)	-6.5 (-46.1 to 49.6)	-94.7 (-97.7 to -90.5)	-82.7 (-88.8 to -75.2)
Russia	Number: 1990	43,643 (43,353–43,950)	7,070 (6,141–7,920)	6,129 (5,364–6,925)	5,923 (5,478–6,439)	1,385 (1,204–1,580)	9,737 (7,884–10,570)	0 (0–0)	1,219 (722–2,679)	2,160 (1,827–2,526)	48 (35–62)	1,338 (1,196–1,496)
Russia	Number: 2015	15,918 (15,632–16,205)	1,721 (1,369–1,984)	1,409 (1,150–1,645)	1,483 (1,308–1,668)	154 (129–182)	4,272 (3,853–5,435)	0 (0–0)	995 (738–1,535)	1,196 (962–1,416)	9 (6–12)	254 (224–289)
Russia	Number: Cumulative change (%)	-63.5 (-64.2 to -62.8)	-75.6 (-79.8 to -71.5)	-76.9 (-81.2 to -72.9)	-74.9 (-78.6 to -71.1)	-88.8 (-91.1 to -86.3)	-55.6 (-61.7 to -31.2)	nan (nan–nan)	-6.8 (-47.8 to 29.7)	-44.2 (-56.2 to -32.3)	-79.6 (-87.1 to -69.9)	-80.9 (-83.9 to -77.7)
Russia	Rate: 1990	432.0 (430.2–434.0)	73.0 (63.3–81.7)	63.2 (55.3–71.5)	57.6 (53.3–62.7)	13.5 (11.7–15.4)	96.9 (78.7–105.3)	0.0 (0.0–0.0)	12.6 (7.4–27.6)	22.2 (18.8–26.0)	0.5 (0.3–0.6)	12.7 (11.4–14.2)
Russia	Rate: 2015	179.1 (175.9–182.4)	19.5 (15.5–22.5)	15.9 (13.0–18.6)	16.6 (14.7–18.7)	1.7 (1.4–2.0)	48.1 (43.4–61.2)	0.0 (0.0–0.0)	11.2 (8.3–17.4)	13.5 (10.9–16.0)	0.1 (0.1–0.1)	2.9 (2.5–3.2)
Russia	Rate: Cumulative change (%)	-58.5 (-59.3 to -57.8)	-73.2 (-77.8 to -68.7)	-74.7 (-79.4 to -70.3)	-71.1 (-75.3 to -66.7)	-87.1 (-89.7 to -84.2)	-49.8 (-56.7 to -22.2)	nan (nan–nan)	2.2 (-42.7 to 42.3)	-38.8 (-51.9 to -25.6)	-76.2 (-85.0 to -64.8)	-77.5 (-81.1 to -73.8)
Ukraine	Number: 1990	13,914 (11,946–16,023)	1,851 (1,493–2,225)	1,787 (1,435–2,189)	1,730 (1,434–2,076)	294 (236–368)	3,732 (2,748–4,449)	0 (0–0)	346 (239–494)	712 (559–880)	137 (99–179)	453 (377–543)
Ukraine	Number: 2015	4,660 (3,220–6,368)	557 (357–826)	531 (327–798)	193 (127–270)	27 (17–41)	1,433 (968–2,012)	0 (0–0)	251 (143–377)	310 (190–465)	14 (8–22)	89 (59–125)
Ukraine	Number: Cumulative change (%)	-66.2 (-77.5 to -52.7)	-69.5 (-81.1 to -54.2)	-69.9 (-82.2 to -54.0)	-88.7 (-92.8 to -83.6)	-90.3 (-94.2 to -84.9)	-60.6 (-75.7 to -38.9)	nan (nan–nan)	-26.1 (-54.0 to 11.8)	-55.7 (-72.9 to -33.4)	-89.4 (-94.2 to -82.1)	-80.2 (-87.6 to -70.8)
Ukraine	Rate: 1990	441.5 (378.2–509.5)	60.4 (48.7–72.6)	58.3 (46.8–71.4)	54.3 (45.1–65.3)	9.3 (7.5–11.6)	119.3 (87.9–142.3)	0.0 (0.0–0.0)	11.3 (7.8–16.1)	23.2 (18.3–28.7)	4.3 (3.1–5.6)	14.1 (11.7–16.9)
Ukraine	Rate: 2015	196.2 (135.6–268.1)	23.7 (15.2–35.1)	22.6 (13.9–34.0)	8.1 (5.3–11.3)	1.2 (0.7–1.7)	60.3 (40.8–84.7)	0.0 (0.0–0.0)	10.7 (6.1–16.0)	13.2 (8.1–19.8)	0.6 (0.4–0.9)	3.7 (2.5–5.2)
Ukraine	Rate: Cumulative change (%)	-55.2 (-70.3 to -37.1)	-60.2 (-75.4 to -40.3)	-60.7 (-76.8 to -39.9)	-84.9 (-90.5 to -78.1)	-87.2 (-92.3 to -80.0)	-48.1 (-67.9 to -19.4)	nan (nan–nan)	-3.6 (-40.0 to 45.9)	-42.2 (-64.7 to -13.0)	-85.8 (-92.3 to -75.9)	-73.4 (-83.3 to -60.8)
Central Europe	Number: 1990	38,155 (37,564–38,780)	8,394 (7,789–9,148)	2,840 (2,595–3,088)	7,494 (7,072–7,903)	840 (764–922)	7,370 (6,285–7,989)	0 (0–0)	574 (357–724)	2,772 (2,476–3,182)	42 (27–54)	788 (710–853)
Central Europe	Number: 2015	6,778 (6,183–7,493)	1,330 (1,131–1,528)	423 (373–475)	776 (668–904)	66 (57–78)	2,096 (1,855–2,423)	0 (0–0)	164 (128–232)	532 (462–605)	8 (6–10)	75 (66–86)
Central Europe	Number: Cumulative change (%)	-82.2 (-83.9 to -80.3)	-84.1 (-87.2 to -81.0)	-85.1 (-87.2 to -82.7)	-89.6 (-91.2 to -87.8)	-92.0 (-93.4 to -90.4)	-71.3 (-76.0 to -62.5)	nan (nan–nan)	-70.4 (-79.3 to -56.3)	-80.7 (-84.5 to -77.1)	-80.4 (-85.4 to -73.6)	-90.4 (-91.9 to -88.3)
Central Europe	Rate: 1990	445.5 (438.4–452.9)	100.2 (93.0–109.2)	33.9 (31.0–36.9)	86.6 (81.7–91.4)	9.7 (8.9–10.7)	86.3 (73.5–93.7)	0.0 (0.0–0.0)	6.8 (4.3–8.6)	33.1 (29.6–38.0)	0.5 (0.3–0.6)	9.1 (8.2–9.8)
Central Europe	Rate: 2015	120.1 (109.6–132.9)	23.9 (20.3–27.5)	7.6 (6.7–8.5)	13.7 (11.8–15.9)	1.2 (1.0–1.4)	37.2 (32.9–43.0)	0.0 (0.0–0.0)	3.0 (2.3–4.2)	9.6 (8.3–10.9)	0.1 (0.1–0.2)	1.3 (1.2–1.5)
Central Europe	Rate: Cumulative change (%)	-73.0 (-75.5 to -70.1)	-76.0 (-80.7 to -71.3)	-77.5 (-80.7 to -73.9)	-84.2 (-86.7 to -81.4)	-87.9 (-90.0 to -85.5)	-56.6 (-63.6 to -43.1)	nan (nan–nan)	-55.4 (-68.9 to -34.2)	-70.9 (-76.7 to -65.6)	-70.1 (-77.8 to -59.7)	-85.4 (-87.7 to -82.3)
Albania	Number: 1990	3,285 (2,988–3,603)	171 (137–202)	69 (54–84)	1,489 (1,287–1,682)	89 (68–110)	229 (162–384)	0 (0–0)	5 (3–9)	156 (132–183)	29 (15–40)	81 (62–100)
Albania	Number: 2015	470 (347–632)	23 (15–35)	11 (7–18)	84 (60–114)	2 (1–3)	117 (73–168)	0 (0–0)	2 (1–3)	56 (38–81)	2 (1–3)	9 (5–16)
Albania	Number: Cumulative change (%)	-85.6 (-89.7 to -80.7)	-86.1 (-91.3 to -78.4)	-82.8 (-89.9 to -72.3)	-94.3 (-96.0 to -92.1)	-97.3 (-98.5 to -95.8)	-45.8 (-75.4 to -19.5)	nan (nan–nan)	-57.2 (-74.1 to -32.7)	-63.9 (-76.5 to -46.5)	-91.2 (-95.0 to -85.7)	-88.5 (-93.8 to -78.5)
Albania	Rate: 1990	854.0 (776.8–936.5)	44.9 (35.9–52.9)	18.1 (14.3–22.2)	386.9 (334.3–436.9)	23.1 (17.9–28.8)	59.7 (42.3–100.0)	0.0 (0.0–0.0)	1.4 (0.9–2.4)	41.0 (34.7–48.0)	7.6 (3.9–10.4)	21.2 (16.1–26.1)
Albania	Rate: 2015	248.5 (183.5–333.7)	12.2 (8.0–18.7)	6.1 (3.7–9.6)	44.8 (31.7–60.5)	1.2 (0.8–1.8)	61.6 (38.7–88.2)	0.0 (0.0–0.0)	1.1 (0.6–2.0)	29.2 (20.1–42.6)	1.3 (0.8–1.9)	4.9 (2.8–8.9)
Albania	Rate: Cumulative change (%)	-70.8 (-79.0 to -60.9)	-72.3 (-82.7 to -56.9)	-65.6 (-79.7 to -44.7)	-88.3 (-91.9 to -83.8)	-94.5 (-96.9 to -91.5)	9.5 (-50.2 to 62.0)	nan (nan–nan)	-14.8 (-48.4 to 34.0)	-28.0 (-53.2 to 6.6)	-82.0 (-89.8 to -71.0)	-76.5 (-87.4 to -56.0)
Bosnia and Herzegovina	Number: 1990	1,204 (1,165–1,241)	360 (301–417)	95 (57–145)	89 (64–112)	42 (28–57)	195 (153–231)	0 (0–0)	29 (14–50)	55 (27–95)	0 (0–0)	23 (15–33)
Bosnia and Herzegovina	Number: 2015	182 (160–207)	39 (30–49)	16 (11–23)	7 (5–10)	2 (1–3)	57 (45–69)	0 (0–0)	8 (4–13)	9 (5–15)	0 (0–0)	2 (1–3)
Bosnia and Herzegovina	Number: Cumulative change (%)	-84.8 (-86.8 to -82.7)	-88.9 (-91.8 to -85.3)	-81.2 (-89.8 to -68.6)	-91.4 (-94.4 to -87.2)	-94.9 (-97.5 to -91.5)	-70.1 (-78.8 to -59.3)	nan (nan–nan)	-68.2 (-87.5 to -40.8)	-79.2 (-92.0 to -58.2)	-32.9 (-82.9 to 71.2)	-88.9 (-95.1 to -80.4)
Bosnia and Herzegovina	Rate: 1990	376.0 (363.8–387.9)	116.0 (97.2–134.3)	30.8 (18.4–46.8)	27.4 (19.8–34.4)	13.1 (8.9–17.8)	61.1 (48.1–72.4)	0.0 (0.0–0.0)	9.3 (4.6–16.3)	17.6 (8.8–30.7)	0.0 (0.0–0.0)	7.1 (4.6–10.0)
Bosnia and Herzegovina	Rate: 2015	110.3 (96.7–125.2)	24.4 (18.7–30.5)	10.2 (6.8–14.2)	4.5 (3.2–6.1)	1.2 (0.7–2.0)	34.5 (27.3–42.0)	0.0 (0.0–0.0)	5.0 (2.5–8.4)	6.1 (3.4–9.6)	0.0 (0.0–0.0)	1.4 (0.8–2.4)
Bosnia and Herzegovina	Rate: Cumulative change (%)	-70.7 (-74.4 to -66.6)	-78.8 (-84.4 to -72.1)	-64.2 (-80.6 to -40.2)	-83.2 (-89.0 to -75.0)	-90.2 (-95.1 to -83.5)	-42.4 (-59.0 to -21.5)	nan (nan–nan)	-39.5 (-76.1 to 12.7)	-60.4 (-84.7 to -20.4)	33.1 (-67.0 to 243.6)	-78.3 (-90.4 to -61.6)
Bulgaria	Number: 1990	1,954 (1,890–2,018)	255 (222–292)	96 (78–117)	451 (412–519)	28 (23–33)	500 (298–560)	0 (0–0)	13 (9–23)	231 (197–276)	0 (0–0)	35 (30–40)
Bulgaria	Number: 2015	594 (446–785)	98 (71–128)	45 (31–62)	86 (62–116)	7 (5–11)	190 (137–254)	0 (0–0)	10 (5–15)	30 (21–43)	0 (0–0)	8 (6–11)
Bulgaria	Number: Cumulative change (%)	-69.6 (-77.3 to -59.7)	-61.3 (-72.8 to -48.4)	-52.6 (-68.5 to -31.4)	-80.8 (-86.3 to -73.8)	-71.4 (-81.0 to -57.9)	-61.1 (-73.0 to -43.6)	nan (nan–nan)	-17.2 (-58.1 to 34.1)	-86.6 (-91.1 to -80.8)	158.6 (53.3–304.9)	-75.7 (-82.7 to -66.8)
Bulgaria	Rate: 1990	373.9 (361.6–386.6)	50.3 (43.8–57.8)	19.0 (15.6–23.2)	85.4 (78.1–98.3)	5.3 (4.4–6.4)	96.2 (57.4–107.8)	0.0 (0.0–0.0)	2.7 (1.9–4.6)	45.7 (38.9–54.5)	0.0 (0.0–0.0)	6.6 (5.8–7.6)

© 2017 Global Burden of Disease Child and Adolescent Health Collaboration. JAMA Pediatrics.

eTable 2. Number of Deaths, Death Rates (per 100,000 population), and Cumulative Percent Change with 95% Uncertainty Intervals (UI) for the Top 10 Global Causes of Death in 195 Countries and Territories, Aged Under 5 Years, Both Sexes, 1990 and 2015 *Note: best viewed by enlarging in browser.*

Location	Measure	All causes	Neonatal preterm birth complications	Neonatal encephalopathy due to birth asphyxia and trauma	Lower respiratory infections	Diarrheal diseases	Congenital anomalies	Malaria	Neonatal sepsis and other neonatal infections	Other neonatal disorders	Protein-energy malnutrition	Meningitis
Bulgaria	Rate: 2015	178.2 (133.9–235.7)	29.9 (21.7–39.2)	13.8 (9.7–19.1)	25.8 (18.6–35.0)	2.4 (1.6–3.3)	57.0 (41.3–76.4)	0.0 (0.0–0.0)	3.3 (1.8–4.7)	9.4 (6.5–13.1)	0.1 (0.1–0.2)	2.5 (1.8–3.4)
Bulgaria	Rate: Cumulative change (%)	-52.3 (-64.4 to -36.9)	-40.2 (-57.9 to -20.2)	-26.7 (-51.3 to 6.1)	-69.7 (-78.3 to -58.6)	-55.0 (-70.2 to -33.5)	-39.3 (-57.9 to -11.9)	nan (nan–nan)	28.3 (-35.0 to 107.5)	-79.3 (-86.3 to -70.4)	314.3 (145.6–550.8)	-61.5 (-72.6 to -47.3)
Croatia	Number: 1990	663 (639–687)	143 (128–159)	70 (61–81)	31 (27–35)	5 (4–6)	172 (153–186)	0 (0–0)	5 (2–13)	94 (82–108)	0 (0–0)	9 (8–10)
Croatia	Number: 2015	178 (159–199)	24 (19–29)	20 (16–24)	4 (3–5)	0 (0–1)	62 (53–73)	0 (0–0)	8 (4–11)	21 (17–26)	0 (0–0)	1 (1–1)
Croatia	Number: Cumulative change (%)	-73.1 (-76.0 to -69.8)	-83.2 (-86.6 to -79.1)	-71.3 (-78.0 to -63.4)	-85.1 (-88.6 to -81.4)	-82.2 (-87.2 to -76.5)	-63.8 (-69.6 to -53.8)	nan (nan–nan)	128.0 (-48.4 to 309.5)	-77.0 (-82.1 to -71.2)	44.4 (-11.8 to 116.8)	-83.4 (-87.0 to -79.3)
Croatia	Rate: 1990	247.6 (238.3–256.4)	54.8 (49.0–60.9)	27.1 (23.4–31.3)	11.4 (10.1–13.0)	2.0 (1.7–2.5)	64.1 (57.2–69.5)	0.0 (0.0–0.0)	2.1 (1.0–5.2)	36.3 (31.7–41.4)	0.0 (0.0–0.0)	3.4 (3.0–3.9)
Croatia	Rate: 2015	90.1 (80.5–100.6)	12.3 (9.9–14.9)	10.4 (8.3–12.7)	2.3 (1.9–2.8)	0.5 (0.4–0.6)	31.3 (26.8–37.1)	0.0 (0.0–0.0)	4.3 (2.1–6.1)	11.1 (9.0–13.6)	0.0 (0.0–0.0)	0.8 (0.6–0.9)
Croatia	Rate: Cumulative change (%)	-63.6 (-67.6 to -59.2)	-77.5 (-82.0 to -72.0)	-61.5 (-70.5 to -50.9)	-79.7 (-84.5 to -74.6)	-75.7 (-82.6 to -67.9)	-50.9 (-58.9 to -37.3)	nan (nan–nan)	205.9 (-30.7 to 448.9)	-69.2 (-76.0 to -61.4)	97.5 (19.9–196.7)	-77.3 (-82.2 to -71.7)
Czech Republic	Number: 1990	1,524 (1,470–1,576)	434 (398–486)	164 (144–187)	89 (77–104)	4 (3–5)	391 (249–439)	0 (0–0)	11 (7–23)	55 (47–65)	0 (0–0)	26 (22–30)
Czech Republic	Number: 2015	268 (234–307)	35 (26–43)	28 (22–35)	13 (10–16)	2 (1–2)	74 (59–107)	0 (0–0)	10 (5–14)	32 (26–40)	0 (0–0)	3 (2–3)
Czech Republic	Number: Cumulative change (%)	-82.4 (-84.7 to -79.7)	-91.8 (-94.3 to -89.8)	-82.3 (-87.7 to -77.3)	-85.3 (-89.7 to -80.9)	-52.6 (-68.0 to -34.2)	-80.1 (-85.8 to -56.3)	nan (nan–nan)	9.9 (-63.4 to 81.7)	-40.1 (-57.4 to -22.1)	81.5 (3.8–186.1)	-87.6 (-91.0 to -83.9)
Czech Republic	Rate: 1990	245.1 (236.3–253.7)	70.8 (64.9–79.1)	26.8 (23.6–30.5)	14.4 (12.5–16.7)	0.7 (0.6–0.8)	63.0 (40.1–70.8)	0.0 (0.0–0.0)	1.9 (1.2–3.8)	9.0 (7.7–10.7)	0.0 (0.0–0.0)	4.1 (3.6–4.9)
Czech Republic	Rate: 2015	50.9 (44.5–58.4)	6.8 (5.2–8.3)	5.5 (4.3–6.8)	2.5 (1.9–3.1)	0.4 (0.3–0.5)	14.1 (11.3–20.4)	0.0 (0.0–0.0)	2.1 (1.1–2.8)	6.3 (5.0–7.7)	0.0 (0.0–0.0)	0.6 (0.5–0.7)
Czech Republic	Rate: Cumulative change (%)	-79.2 (-81.9 to -76.1)	-90.3 (-93.3 to -88.0)	-79.1 (-85.5 to -73.2)	-82.6 (-87.9 to -77.4)	-43.9 (-62.2 to -22.1)	-76.4 (-83.2 to -48.4)	nan (nan–nan)	29.5 (-56.9 to 114.5)	-29.3 (-49.7 to -8.0)	115.4 (22.7–239.6)	-85.4 (-89.4 to -81.0)
Hungary	Number: 1990	2,138 (2,068–2,203)	598 (549–650)	456 (409–504)	158 (142–176)	7 (6–8)	511 (472–549)	0 (0–0)	18 (11–24)	44 (37–51)	0 (0–0)	34 (30–38)
Hungary	Number: 2015	456 (342–604)	110 (75–155)	23 (15–33)	19 (13–25)	3 (2–5)	145 (102–214)	0 (0–0)	9 (5–13)	69 (46–97)	0 (0–0)	3 (2–5)
Hungary	Number: Cumulative change (%)	-78.7 (-84.1 to -71.8)	-81.6 (-87.7 to -73.8)	-94.9 (-96.7 to -92.7)	-87.5 (-91.4 to -83.0)	-48.0 (-65.4 to -28.4)	-71.6 (-80.2 to -58.3)	nan (nan–nan)	-49.4 (-66.5 to -28.7)	58.4 (-0.2 to 129.4)	8.1 (-40.6 to 66.0)	-88.8 (-92.0 to -85.1)
Hungary	Rate: 1990	349.6 (338.1–360.5)	98.5 (90.4–107.1)	75.1 (67.4–83.0)	25.8 (23.1–28.7)	1.2 (1.1–1.4)	83.4 (77.1–89.7)	0.0 (0.0–0.0)	3.1 (1.9–4.0)	7.2 (6.1–8.5)	0.1 (0.0–0.1)	5.6 (4.9–6.3)
Hungary	Rate: 2015	101.2 (75.8–134.4)	24.6 (16.8–34.8)	5.2 (3.4–7.5)	4.4 (3.1–5.7)	0.9 (0.6–1.2)	32.1 (22.8–47.6)	0.0 (0.0–0.0)	2.1 (1.2–3.1)	15.4 (10.4–21.8)	0.1 (0.0–0.1)	0.8 (0.6–1.1)
Hungary	Rate: Cumulative change (%)	-71.0 (-78.4 to -61.7)	-75.0 (-83.3 to -64.3)	-93.1 (-95.6 to -90.0)	-83.1 (-88.3 to -76.9)	-29.5 (-53.2 to -2.8)	-61.4 (-73.1 to -43.3)	nan (nan–nan)	-31.2 (-54.6 to -3.2)	115.5 (35.7–211.9)	46.6 (-19.7 to 124.5)	-84.8 (-89.2 to -79.8)
Macedonia	Number: 1990	1,238 (1,193–1,289)	371 (328–419)	63 (50–77)	147 (122–171)	172 (146–200)	141 (83–181)	0 (0–0)	3 (2–6)	170 (136–221)	0 (0–1)	21 (16–27)
Macedonia	Number: 2015	223 (157–306)	75 (51–106)	13 (8–20)	15 (10–22)	4 (2–6)	52 (35–72)	0 (0–0)	3 (1–5)	21 (14–31)	0 (0–0)	3 (2–5)
Macedonia	Number: Cumulative change (%)	-81.9 (-87.4 to -75.4)	-79.6 (-86.3 to -70.8)	-78.6 (-86.8 to -65.7)	-89.3 (-93.1 to -84.4)	-97.3 (-98.4 to -96.0)	-61.2 (-76.6 to -35.4)	nan (nan–nan)	1.3 (-52.1 to 64.4)	-87.1 (-91.9 to -80.3)	-90.1 (-95.8 to -81.5)	-82.3 (-89.0 to -72.4)
Macedonia	Rate: 1990	720.6 (693.7–750.4)	218.2 (193.1–246.5)	37.1 (29.5–45.8)	85.0 (70.8–99.2)	100.1 (84.9–115.9)	82.5 (48.3–105.4)	0.0 (0.0–0.0)	2.1 (1.4–3.6)	100.4 (80.2–130.3)	0.5 (0.3–0.8)	12.7 (9.5–15.7)
Macedonia	Rate: 2015	196.5 (138.3–269.1)	66.6 (45.8–93.8)	11.7 (7.6–17.8)	13.7 (9.2–19.4)	4.0 (2.6–5.9)	45.8 (31.6–63.3)	0.0 (0.0–0.0)	2.9 (1.6–4.5)	19.0 (12.4–27.9)	0.1 (0.0–0.1)	3.3 (2.2–4.6)
Macedonia	Rate: Cumulative change (%)	-72.7 (-80.9 to -62.8)	-69.3 (-79.4 to -56.1)	-67.8 (-80.1 to -48.4)	-83.7 (-89.5 to -76.4)	-95.9 (-97.5 to -94.0)	-41.5 (-64.7 to -2.4)	nan (nan–nan)	52.0 (-28.2 to 146.9)	-80.7 (-87.8 to -70.4)	-85.0 (-93.6 to -72.1)	-73.1 (-83.4 to -58.1)
Montenegro	Number: 1990	195 (149–247)	47 (30–67)	37 (23–53)	20 (13–28)	2 (1–3)	20 (14–28)	0 (0–0)	6 (2–11)	16 (8–28)	0 (0–0)	2 (1–3)
Montenegro	Number: 2015	37 (28–48)	7 (4–9)	7 (4–10)	2 (1–3)	0 (0–0)	6 (4–9)	0 (0–0)	1 (0–2)	2 (1–4)	0 (0–0)	0 (0–0)
Montenegro	Number: Cumulative change (%)	-80.4 (-86.8 to -71.7)	-84.3 (-91.4 to -74.5)	-78.5 (-88.7 to -64.6)	-86.7 (-92.7 to -78.3)	-90.6 (-96.5 to -81.1)	-64.6 (-80.3 to -41.8)	nan (nan–nan)	-68.6 (-89.6 to -37.5)	-81.2 (-93.8 to -59.9)	-68.2 (-97.5 to 10.9)	-83.6 (-94.6 to -64.1)
Montenegro	Rate: 1990	394.8 (301.7–499.0)	96.5 (62.7–136.8)	76.3 (48.1–109.7)	41.1 (27.8–57.4)	4.1 (2.1–7.0)	41.5 (28.6–57.9)	0.0 (0.0–0.0)	12.4 (5.7–22.9)	33.7 (16.9–58.5)	0.5 (0.1–1.3)	4.2 (2.0–7.9)
Montenegro	Rate: 2015	106.2 (80.7–136.7)	20.1 (13.5–28.2)	21.8 (14.3–31.6)	7.3 (4.9–10.5)	0.5 (0.3–0.8)	19.5 (13.5–27.6)	0.0 (0.0–0.0)	4.7 (2.3–8.2)	7.7 (3.6–13.2)	0.1 (0.0–0.3)	0.8 (0.4–1.5)
Montenegro	Rate: Cumulative change (%)	-72.4 (-81.5 to -60.1)	-77.8 (-87.9 to -64.0)	-69.6 (-84.0 to -49.9)	-81.3 (-89.8 to -69.6)	-86.8 (-95.0 to -73.4)	-50.1 (-72.3 to -18.1)	nan (nan–nan)	-55.6 (-85.4 to -11.5)	-73.4 (-91.2 to -42.9)	-55.1 (-96.5 to 56.2)	-77.0 (-92.5 to -49.3)
Poland	Number: 1990	10,297 (10,139–10,456)	3,320 (2,985–3,814)	789 (641–964)	760 (678–863)	103 (88–122)	2,973 (2,155–3,244)	0 (0–0)	321 (162–445)	334 (262–426)	1 (1–2)	200 (177–228)
Poland	Number: 2015	1,754 (1,308–2,337)	456 (315–637)	96 (65–135)	67 (49–89)	6 (4–8)	701 (517–942)	0 (0–0)	74 (45–125)	64 (42–92)	0 (0–1)	12 (9–17)
Poland	Number: Cumulative change (%)	-83.0 (-87.3 to -77.3)	-86.2 (-90.9 to -80.2)	-87.5 (-92.3 to -81.4)	-91.0 (-93.7 to -87.9)	-93.4 (-95.6 to -90.8)	-75.9 (-83.3 to -62.0)	nan (nan–nan)	-74.9 (-87.5 to -51.4)	-80.3 (-87.6 to -69.7)	-50.7 (-74.8 to -12.7)	-93.5 (-95.4 to -91.2)
Poland	Rate: 1990	366.3 (360.7–371.9)	120.5 (108.3–138.4)	28.6 (23.3–34.9)	26.7 (23.8–30.3)	3.7 (3.1–4.3)	105.8 (76.5–115.6)	0.0 (0.0–0.0)	11.7 (5.9–16.2)	12.1 (9.5–15.5)	0.1 (0.0–0.1)	7.0 (6.2–8.0)
Poland	Rate: 2015	92.1 (68.6–122.7)	24.3 (16.8–33.9)	5.1 (3.5–7.2)	3.5 (2.6–4.6)	0.4 (0.3–0.5)	36.8 (27.1–49.6)	0.0 (0.0–0.0)	4.0 (2.4–6.7)	3.4 (2.3–4.9)	0.0 (0.0–0.1)	0.7 (0.5–0.9)
Poland	Rate: Cumulative change (%)	-74.8 (-81.3 to -66.4)	-79.7 (-86.6 to -70.9)	-81.7 (-88.8 to -72.7)	-86.8 (-90.6 to -82.2)	-90.2 (-93.5 to -86.5)	-64.4 (-75.4 to -43.8)	nan (nan–nan)	-63.1 (-81.6 to -28.8)	-71.1 (-81.9 to -55.5)	-27.0 (-62.9 to 29.4)	-90.4 (-93.2 to -87.0)
Romania	Number: 1990	10,834 (10,674–10,998)	1,208 (1,012–1,414)	605 (506–732)	3,733 (3,510–3,957)	344 (285–410)	1,297 (1,063–1,401)	0 (0–0)	45 (28–63)	1,299 (1,085–1,507)	3 (2–4)	254 (216–287)
Romania	Number: 2015	1,514 (1,199–1,913)	158 (113–208)	59 (43–81)	410 (317–534)	28 (20–39)	365 (274–473)	0 (0–0)	10 (6–17)	160 (118–206)	2 (1–3)	16 (11–20)
Romania	Number: Cumulative change (%)	-86.0 (-88.9 to -82.4)	-86.7 (-90.8 to -81.7)	-90.0 (-93.3 to -86.2)	-89.0 (-91.6 to -85.6)	-91.5 (-94.1 to -88.0)	-70.9 (-80.5 to -59.0)	nan (nan–nan)	-76.2 (-85.5 to -64.1)	-87.5 (-91.4 to -82.8)	-21.3 (-55.2 to 31.1)	-93.6 (-95.4 to -91.3)
Romania	Rate: 1990	682.5 (672.7–692.6)	78.7 (65.9–92.1)	39.4 (33.0–47.7)	233.4 (218.3–247.7)	21.6 (17.9–25.8)	82.4 (67.6–89.0)	0.0 (0.0–0.0)	3.0 (1.9–4.1)	84.7 (70.7–98.2)	0.2 (0.1–0.3)	15.9 (13.6–18.0)
Romania	Rate: 2015	170.3 (134.7–214.9)	18.3 (13.1–24.1)	6.9 (5.0–9.3)	46.0 (35.5–60.0)	3.3 (2.3–4.5)	41.4 (31.1–53.5)	0.0 (0.0–0.0)	1.2 (0.8–2.0)	18.5 (13.7–23.8)	0.3 (0.2–0.4)	1.8 (1.3–2.3)
Romania	Rate: Cumulative change (%)	-75.0 (-80.2 to -68.6)	-76.5 (-83.7 to -67.6)	-82.3 (-88.2 to -75.5)	-80.3 (-85.0 to -74.3)	-84.8 (-89.4 to -78.5)	-48.2 (-65.3 to -27.0)	nan (nan–nan)	-58.0 (-74.4 to -36.5)	-77.9 (-84.7 to -69.5)	42.4 (-19.8 to 138.4)	-88.6 (-91.8 to -84.5)
Serbia	Number: 1990	3,441 (3,005–3,932)	1,084 (792–1,435)	331 (214–466)	335 (210–479)	29 (17–43)	594 (327–880)	0 (0–0)	105 (50–151)	220 (116–479)	5 (3–8)	75 (44–112)
Serbia	Number: 2015	699 (641–761)	197 (162–229)	76 (62–92)	34 (27–41)	3 (2–5)	190 (158–241)	0 (0–0)	19 (13–34)	46 (36–56)	0 (0–1)	7 (5–8)
Serbia	Number: Cumulative change (%)	-79.5 (-82.7 to -76.1)	-81.1 (-87.4 to -73.4)	-75.5 (-84.5 to -63.6)	-89.1 (-93.2 to -82.7)	-85.4 (-91.6 to -75.5)	-64.5 (-80.4 to -28.8)	nan (nan–nan)	-78.5 (-89.5 to -51.5)	-74.6 (-91.5 to -58.7)	-80.7 (-90.1 to -66.0)	-89.5 (-94.1 to -82.9)
Serbia	Rate: 1990	496.8 (433.6–566.9)	158.4 (115.8–209.7)	48.4 (31.3–68.2)	48.0 (30.2–69.0)	4.3 (2.5–6.3)	85.7 (47.1–127.1)	0.0 (0.0–0.0)	15.4 (7.3–22.1)	32.2 (17.0–69.8)	0.8 (0.5–1.3)	10.7 (6.3–16.1)
Serbia	Rate: 2015	157.8 (144.8–171.9)	45.0 (37.1–52.2)	17.5 (14.1–21.0)	7.6 (6.1–9.4)	0.9 (0.7–1.2)	42.9 (35.6–54.4)	0.0 (0.0–0.0)	4.5 (3.0–7.9)	10.5 (8.3–12.9)	0.2 (0.1–0.3)	1.6 (1.3–1.9)
Serbia	Rate: Cumulative change (%)	-68.0 (-73.0 to -62.5)	-70.5 (-80.3 to -58.6)	-61.9 (-75.8 to -43.2)	-83.0 (-89.4 to -73.0)	-77.1 (-86.9 to -61.6)	-44.4 (-69.4 to 11.6)	nan (nan–nan)	-66.6 (-83.7 to -24.5)	-60.5 (-86.8 to -35.6)	-69.8 (-84.6 to -46.3)	-83.6 (-90.7 to -73.1)
Slovakia	Number: 1990	1,145 (1,095–1,192)	333 (301–365)	42 (33–52)	176 (154–200)	9 (7–12)	265 (219–301)	0 (0–0)	5 (3–8)	86 (70–105)	0 (0–1)	20 (17–25)
Slovakia	Number: 2015	341 (293–398)	94 (76–113)	19 (15–25)	27 (22–33)	2 (2–3)	110 (91–134)	0 (0–0)	3 (2–5)	12 (9–15)	0 (0–0)	5 (4–7)
Slovakia	Number: Cumulative change (%)	-70.2 (-74.7 to -64.9)	-71.6 (-77.3 to -65.1)	-52.5 (-66.0 to -35.8)	-84.1 (-87.7 to -80.0)	-69.6 (-80.1 to -57.1)	-57.9 (-67.2 to -45.4)	nan (nan–nan)	-33.5 (-52.5 to -6.9)	-85.6 (-90.2 to -80.2)	-65.8 (-79.1 to -48.5)	-71.5 (-79.8 to -61.5)
Slovakia	Rate: 1990	290.6 (278.0–302.6)	86.3 (78.0–94.4)	11.0 (8.7–13.5)	44.3 (38.7–50.4)	2.4 (1.8–3.0)	67.5 (55.8–76.6)	0.0 (0.0–0.0)	1.3 (0.9–2.3)	22.4 (18.3–27.2)	0.2 (0.1–0.3)	5.2 (4.3–6.3)
Slovakia	Rate: 2015	122.4 (105.3–142.9)	34.1 (27.8–41.1)	7.2 (5.6–9.2)	9.9 (8.0–12.0)	1.0 (0.7–1.3)	39.8 (32.9–48.1)	0.0 (0.0–0.0)	1.2 (0.8–1.9)	4.4 (3.3–5.7)	0.1 (0.1–0.1)	2.1 (1.6–2.7)
Slovakia	Rate: Cumulative change (%)	-57.8 (-64.2 to -50.4)	-60.4 (-68.3 to -51.3)	-33.5 (-52.5 to -10.2)	-77.4 (-82.6 to -71.6)	-56.9 (-71.8 to -39.1)	-40.5 (-53.6 to -22.9)	nan (nan–nan)	-7.2 (-33.6 to 30.2)	-79.9 (-86.3 to -72.3)	-51.0 (-70.0 to -26.1)	-59.3 (-71.2 to -44.8)
Slovenia	Number: 1990	231 (220–243)	64 (59–70)	16 (14–19)	11 (9–12)	1 (0–1)	76 (69–83)	0 (0–0)	2 (1–4)	6 (4–7)	0 (0–0)	2 (2–3)
Slovenia	Number: 2015	56 (50–64)	10 (7–13)	3 (2–4)	1 (1–2)	0 (0–0)	23 (18–31)	0 (0–0)	1 (1–2)	3 (2–4)	0 (0–0)	0 (0–0)
Slovenia	Number: Cumulative change (%)	-75.4 (-78.5 to -72.0)	-83.5 (-88.1 to -79.8)	-81.1 (-86.5 to -74.7)	-82.8 (-87.3 to -77.7)	-80.7 (-86.2 to -74.2)	-69.7 (-75.9 to -61.8)	nan (nan–nan)	-32.0 (-54.4 to -7.6)	-37.2 (-54.7 to -14.7)	-83.8 (-91.6 to -72.0)	-86.1 (-89.3 to -82.1)
Slovenia	Rate: 1990	205.8 (195.5–216.6)	59.5 (54.2–65.0)	15.1 (13.0–17.6)	9.7 (8.2–11.2)	0.9 (0.8–1.1)	67.8 (62.2–74.5)	0.0 (0.0–0.0)	2.6 (1.8–3.7)	5.5 (4.5–6.7)	0.0 (0.0–0.1)	2.4 (2.0–2.8)
Slovenia	Rate: 2015	54.0 (47.7–61.0)	10.2 (7.2–12.4)	3.0 (2.2–3.9)	1.8 (1.3–2.3)	0.2 (0.1–0.3)	21.9 (17.4–29.7)	0.0 (0.0–0.0)	1.8 (1.1–2.4)	3.6 (2.7–4.7)	0.0 (0.0–0.0)	0.4 (0.3–0.5)
Slovenia	Rate: Cumulative change (%)	-73.7 (-77.0 to -70.1)	-82.8 (-87.6 to -78.9)	-80.2 (-85.9 to -73.6)	-81.4 (-86.2 to -75.8)	-78.9 (-84.9 to -71.8)	-67.7 (-74.3 to -59.4)	nan (nan–nan)	-29.0 (-52.4 to -3.7)	-34.4 (-52.8 to -10.9)	-82.7 (-91.0 to -70.2)	-84.7 (-88.2 to -80.3)
Central Asia	Number: 1990	119,208 (115,213–123,156)	13,318 (11,503–15,341)	12,803 (10,557–15,006)	48,952 (46,702–51,088)	13,218 (12,399–14,105)	7,641 (6,875–8,313)	5 (4–7)	1,524 (928–2,157)	3,213 (2,323–4,297)	280 (204–338)	1,861 (1,573–2,128)
Central Asia	Number: 2015	49,170 (45,149–53,673)	7,663 (6,564–8,837)	8,096 (6,823–9,506)	13,368 (11,650–15,206)	1,709 (1,366–2,100)	6,573 (5,138–7,723)	1 (0–2)	1,242 (759–1,736)	2,421 (1,827–3,207)	121 (67–240)	552 (445–723)
Central Asia	Number: Cumulative change (%)	-58.7 (-62.3 to -54.8)	-42.1 (-51.7 to -31.6)	-36.2 (-48.1 to -21.2)	-72.7 (-76.5 to -68.8)	-87.0 (-89.8 to -83.9)	-13.7 (-34.0 to 0.9)	-79.8 (-90.8 to -62.4)	-16.5 (-43.0 to 14.1)	-22.0 (-49.7 to 12.1)	-56.1 (-75.8 to -19.3)	-69.9 (-77.5 to -57.3)
Central Asia	Rate: 1990	1,255.0 (1,213.0–1,296.7)	139.4 (120.5–160.6)	134.1 (110.6–157.1)	516.0 (492.3–538.7)	139.3 (130.7–148.7)	80.3 (72.2–87.4)	0.1 (0.0–0.1)	16.0 (9.7–22.6)	33.7 (24.3–45.0)	3.0 (2.2–3.6)	19.7 (16.6–22.5)
Central Asia	Rate: 2015	528.6 (485.4–577.0)	82.7 (70.8–95.3)	87.3 (73.6–102.5)	143.4 (124.9–163.1)	18.3 (14.6–22.5)	70.7 (55.3–83.0)	0.0 (0.0–0.0)	13.4 (8.2–18.7)	26.1 (19.7–34.6)	1.3 (0.7–2.6)	5.9 (4.8–7.8)
Central Asia	Rate: Cumulative change (%)	-57.9 (-61.5 to -53.8)	-40.4 (-50.3 to -29.5)	-34.3 (-46.5 to -18.9)	-72.2 (-76.1 to -68.2)	-86.8 (-89.6 to -83.7)	-11.7 (-32.5 to 3.3)	-79.5 (-90.7 to -62.0)	-14.2 (-41.4 to 17.2)	-19.8 (-48.3 to 15.3)	-55.5 (-75.5 to -18.2)	-69.5 (-77.1 to -56.6)
Armenia	Number: 1990	3,580 (3,320–3,855)	616 (470–762)	121 (70–190)	1,062 (949–1,177)	365 (313–424)	451 (384–553)	0 (0–0)	182 (85–315)	229 (115–389)	1 (0–2)	11 (8–14)
Armenia	Number: 2015	588 (473–718)	91 (61–130)	24 (13–39)	101 (80–127)	10 (7–13)	183 (134–235)	0 (0–0)	45 (23–71)	47 (25–78)	0 (0–0)	1 (1–2)
Armenia	Number: Cumulative change (%)	-83.5 (-86.9 to -79.6)	-84.8 (-90.6 to -77.1)	-77.7 (-90.2 to -59.9)	-90.4 (-92.7 to -87.4)	-97.2 (-98.1 to -96.2)	-58.8 (-72.2 to -45.6)	-100.0 (-100.0 to -100.0)	-72.5 (-87.2 to -52.5)	-76.4 (-90.5 to -51.0)	-96.4 (-98.7 to -88.3)	-85.7 (-89.9 to -79.5)
Armenia	Rate: 1990	984.1 (912.5–1,059.1)	172.1 (131.2–212.9)	33.9 (19.6–53.1)	290.0 (259.2–321.2)	99.9 (85.6–116.2)	124.8 (106.4–152.6)	0.1 (0.0–0.1)	50.8 (23.8–88.0)	63.9 (32.1–108.7)	0.4 (0.2–0.7)	3.0 (2.4–3.8)
Armenia	Rate: 2015	306.6 (246.4–375.0)	48.6 (32.4–69.1)	13.1 (7.2–20.9)	52.4 (41.3–66.0)	5.2 (3.8–6.9)	95.6 (70.2–122.7)	0.0 (0.0–0.0)	24.0 (12.6–37.9)	25.0 (13.5–41.4)	0.0 (0.0–0.1)	0.8 (0.6–1.1)
Armenia	Rate: Cumulative change (%)	-68.8 (-75.1 to -61.3)	-71.2 (-82.3 to -56.6)	-57.8 (-81.5 to -24.0)	-81.9 (-86.1 to -76.2)	-94.7 (-96.3 to -92.8)	-22.4 (-47.7 to 2.5)	-100.0 (-100.0 to -100.0)	-48.0 (-75.8 to -10.5)	-55.3 (-81.9 to -7.5)	-93.3 (-97.6 to -78.1)	-72.9 (-80.8 to -61.3)
Azerbaijan	Number: 1990	15,847 (14,481–17,279)	1,571 (1,101–2,023)	1,581 (1,118–2,095)	7,753 (6,973–8,606)	1,506 (1,281–1,761)	996 (579–1,251)	0 (0–0)	65 (24–128)	126 (49–245)	101 (61–136)	101 (80–136)
Azerbaijan	Number: 2015	5,786 (4,652–7,064)	921 (597–1,273)	1,209 (845–1,593)	1,642 (1,245–2,135)	176 (115–248)	738 (466–1,021)	0 (0–0)	52 (19–104)	82 (34–166)	14 (8–23)	44 (31–61)
Azerbaijan	Number: Cumulative change (%)	-63.4 (-71.0 to -54.8)	-39.9 (-62.8 to -10.6)	-21.0 (-49.8 to 14.0)	-78.8 (-84.4 to -72.2)	-88.2 (-92.4 to -82.7)	-23.1 (-52.2 to 19.0)	-81.8 (-87.2 to -75.4)	-1.1 (-74.2 to 139.7)	-16.8 (-78.1 to 102.9)	-84.7 (-92.6 to -73.6)	-55.6 (-71.2 to -35.7)
Azerbaijan	Rate: 1990	1,721.9 (1,573.0–1,877.0)	166.2 (116.4–213.9)	167.4 (118.5–222.0)	846.8 (761.0–939.1)	164.2 (140.1–191.8)	107.3 (62.5–135.0)	0.1 (0.1–0.1)	7.0 (2.6–13.7)	13.5 (5.3–26.1)	11.2 (6.8–15.0)	11.3 (8.8–15.0)
Azerbaijan	Rate: 2015	614.9 (494.8–750.7)	98.8 (64.1–136.5)	129.6 (90.6–170.8)	173.7 (131.7–225.6)	18.7 (12.2–26.3)	78.5 (49.6–108.6)	0.0 (0.0–0.0)	5.6 (2.0–11.2)	8.9 (3.7–17.8)	1.6 (0.9–2.5)	4.7 (3.3–6.5)
Azerbaijan	Rate: Cumulative change (%)	-64.2 (-71.6 to -55.8)	-39.1 (-62.3 to -9.4)	-20.1 (-49.2 to 15.2)	-79.4 (-84.9 to -73.2)	-88.5 (-92.6 to -83.3)	-24.0 (-52.7 to 17.5)	-83.0 (-88.0 to -77.0)	-0.8 (-74.0 to 139.1)	-16.4 (-78.1 to 104.5)	-85.4 (-93.0 to -74.9)	-57.6 (-72.5 to -38.7)
Georgia	Number: 1990	3,811 (3,526–4,132)	583 (442–728)	831 (651–987)	1,344 (1,206–1,501)	164 (136–197)	212 (177–273)	0 (0–0)	91 (62–134)	52 (35–75)	0 (0–1)	37 (28–46)
Georgia	Number: 2015	950 (784–1,136)	194 (146–251)	143 (108–185)	73 (57–90)	12 (9–16)	143 (104–181)	0 (0–0)	64 (44–88)	143 (104–188)	0 (0–0)	9 (7–12)
Georgia	Number: Cumulative change (%)	-75.0 (-79.7 to -69.8)	-66.0 (-76.4 to -53.5)	-82.5 (-87.3 to -76.4)	-94.5 (-95.9 to -93.0)	-92.3 (-94.6 to -89.3)	-30.8 (-58.1 to -6.7)	-100.0 (-100.0 to -100.0)	-28.0 (-52.0 to 0.7)	191.9 (63.0–336.8)	-39.0 (-71.6 to 12.9)	-74.4 (-81.5 to -64.0)
Georgia	Rate: 1990	859.5 (796.3–931.8)	133.7 (101.4–166.7)	190.4 (149.1–226.0)	300.9 (270.3–336.1)	37.0 (30.4–44.4)	48.0 (40.1–62.0)	0.0 (0.0–0.1)	20.9 (14.4–30.8)	11.9 (8.1–17.2)	0.2 (0.1–0.3)	8.4 (6.4–10.3)
Georgia	Rate: 2015	357.0 (294.1–426.9)	74.4 (56.0–96.1)	54.8 (41.5–70.6)	26.9 (21.1–33.4)	4.6 (3.5–6.0)	53.7 (39.3–68.1)	0.0 (0.0–0.0)	24.4 (16.9–33.8)	54.6 (40.0–72.0)	0.2 (0.1–0.2)	3.5 (2.7–4.6)
Georgia	Rate: Cumulative change (%)	-58.4 (-66.3 to -49.7)	-43.3 (-60.5 to -22.3)	-70.9 (-78.8 to -60.5)	-91.0 (-93.2 to -88.5)	-87.4 (-91.1 to -82.3)	14.3 (-30.5 to 53.9)	-100.0 (-100.0 to -100.0)	20.0 (-19.9 to 67.9)	387.0 (171.8–628.5)	0.1 (-53.4 to 85.7)	-57.8 (-69.5 to -40.8)
Kazakhstan	Number: 1990	15,271 (14,164–16,346)	3,445 (2,972–3,851)	661 (483–1,003)	4,576 (4,147–5,041)	1,398 (1,213–1,584)	1,878 (1,687–2,098)	0 (0–3)	271 (117–437)	405 (322–512)	32 (18–43)	259 (226–296)
Kazakhstan	Number: 2015	6,543 (5,248–8,011)	1,884 (1,360–2,460)	427 (287–617)	800 (647–1,000)	93 (70–120)	1,382 (1,017–1,813)	0 (0–0)	282 (129–478)	310 (217–421)	14 (8–20)	71 (55–91)
Kazakhstan	Number: Cumulative change (%)	-57.1 (-65.6 to -47.3)	-45.2 (-59.3 to -29.4)	-34.1 (-55.5 to -7.6)	-82.4 (-86.2 to -77.5)	-93.3 (-95.1 to -91.0)	-26.2 (-45.8 to -3.0)	-100.0 (-100.0 to -100.0)	11.1 (-44.3 to 84.8)	-22.6 (-47.1 to 4.7)	-55.2 (-70.9 to -35.7)	-72.5 (-79.2 to -64.1)
Kazakhstan	Rate: 1990	811.9 (752.5–868.0)	186.7 (161.1–208.7)	35.9 (26.2–54.4)	241.6 (218.6–266.2)	73.9 (64.0–83.9)	100.5 (90.3–112.2)	0.1 (0.0–0.2)	14.7 (6.4–23.7)	22.0 (17.4–27.8)	1.7 (1.0–2.3)	13.7 (11.9–15.5)
Kazakhstan	Rate: 2015	356.4 (285.7–436.4)	103.5 (74.7–135.0)	23.5 (15.8–33.9)	43.3 (35.1–54.1)	5.1 (3.8–6.5)	75.2 (55.3–98.7)	0.0 (0.0–0.0)	15.5 (7.1–26.2)	17.0 (11.9–23.1)	0.8 (0.4–1.1)	3.8 (3.0–4.9)
Kazakhstan	Rate: Cumulative change (%)	-56.0 (-64.8 to -46.0)	-44.5 (-58.7 to -28.5)	-33.3 (-55.0 to -6.4)	-82.0 (-85.9 to -76.9)	-93.1 (-94.9 to -90.8)	-25.0 (-44.9 to -1.3)	-100.0 (-100.0 to -100.0)	12.5 (-43.6 to 87.2)	-21.7 (-46.5 to 5.9)	-54.0 (-70.1 to -33.9)	-71.7 (-78.6 to -63.0)
Kyrgyzstan	Number: 1990	8,836 (8,491–9,177)	1,020 (797–1,269)	1,167 (884–1,412)	3,686 (3,455–3,911)	807 (706–908)	605 (523–750)	0 (0–0)	95 (57–131)	303 (209–416)	7 (5–10)	163 (113–198)
Kyrgyzstan	Number: 2015	4,449 (4,017–4,907)	982 (822–1,148)	813 (663–971)	966 (837–1,109)	175 (137–217)	654 (526–790)	0 (0–0)	96 (67–128)	204 (158–262)	2 (1–3)	41 (29–64)

© 2017 Global Burden of Disease Child and Adolescent Health Collaboration. JAMA Pediatrics.

eTable 2. Number of Deaths, Death Rates (per 100,000 population), and Cumulative Percent Change with 95% Uncertainty Intervals (UI) for the Top 10 Global Causes of Death in 195 Countries and Territories, Aged Under 5 Years, Both Sexes, 1990 and 2015 *Note: best viewed by enlarging in browser.*

Location	Measure	All causes	Neonatal preterm birth complications	Neonatal encephalopathy due to birth asphyxia and trauma	Lower respiratory infections	Diarrheal diseases	Congenital anomalies	Malaria	Neonatal sepsis and other neonatal infections	Other neonatal disorders	Protein-energy malnutrition	Meningitis
Kyrgyzstan	Number: Cumulative change (%)	-49.6 (-55.0 to -44.1)	-1.9 (-26.3 to 27.0)	-29.1 (-47.0 to -4.8)	-73.8 (-77.6 to -69.4)	-78.1 (-83.4 to -71.8)	9.4 (-21.7 to 34.5)	-100.0 (-100.0 to -100.0)	4.9 (-27.4 to 41.5)	-29.6 (-54.9 to 4.8)	-68.7 (-81.3 to -51.9)	-72.9 (-84.0 to -47.4)
Kyrgyzstan	Rate: 1990	1,371.4 (1,317.9–1,424.3)	156.9 (122.6–195.1)	179.6 (136.1–217.2)	573.9 (538.0–609.1)	125.8 (110.2–141.8)	93.6 (80.8–116.1)	0.1 (0.1–0.1)	14.7 (8.9–20.3)	46.8 (32.3–64.1)	1.2 (0.8–1.6)	25.5 (17.7–30.9)
Kyrgyzstan	Rate: 2015	600.4 (542.2–662.0)	132.5 (110.9–155.0)	109.7 (89.5–131.0)	130.3 (113.0–149.6)	23.7 (18.5–29.3)	88.3 (70.9–106.5)	0.0 (0.0–0.0)	13.0 (9.1–17.3)	27.5 (21.4–35.4)	0.3 (0.2–0.5)	5.6 (3.9–8.7)
Kyrgyzstan	Rate: Cumulative change (%)	-56.2 (-60.9 to -51.4)	-13.9 (-35.4 to 11.4)	-37.8 (-53.5 to -16.5)	-77.3 (-80.6 to -73.4)	-81.0 (-85.6 to -75.7)	-4.6 (-31.8 to 17.4)	-100.0 (-100.0 to -100.0)	-8.4 (-36.7 to 23.6)	-38.4 (-60.6 to -8.3)	-73.0 (-83.9 to -58.4)	-76.5 (-86.1 to -54.4)
Mongolia	Number: 1990	6,325 (5,974–6,710)	591 (420–786)	749 (559–955)	2,926 (2,601–3,261)	88 (57–133)	343 (223–587)	0 (0–0)	73 (33–128)	103 (46–185)	10 (1–33)	269 (77–472)
Mongolia	Number: 2015	1,938 (1,611–2,334)	259 (173–356)	432 (326–555)	459 (347–603)	2 (1–3)	317 (227–443)	0 (0–0)	48 (22–90)	54 (25–95)	0 (0–1)	15 (7–25)
Mongolia	Number: Cumulative change (%)	-69.3 (-74.8 to -62.9)	-54.7 (-71.4 to -32.4)	-41.0 (-59.5 to -16.6)	-84.2 (-88.3 to -78.9)	-97.4 (-98.8 to -95.7)	0.1 (-49.1 to 51.1)	nan (nan–nan)	-24.8 (-72.4 to 49.6)	-37.3 (-79.0 to 42.4)	-88.3 (-99.1 to -65.8)	-91.1 (-98.1 to -75.1)
Mongolia	Rate: 1990	1,898.6 (1,792.8–2,014.1)	178.1 (126.7–236.8)	225.7 (168.5–287.5)	878.2 (781.0–978.2)	26.6 (17.3–40.0)	103.2 (67.1–176.6)	0.0 (0.0–0.0)	22.1 (10.0–38.8)	31.0 (14.1–55.9)	3.3 (0.4–10.2)	80.7 (23.2–142.2)
Mongolia	Rate: 2015	581.6 (484.0–700.5)	78.0 (52.2–107.1)	129.7 (98.1–166.9)	137.7 (104.1–180.9)	0.6 (0.4–1.0)	95.1 (68.2–133.0)	0.0 (0.0–0.0)	14.4 (6.8–27.1)	16.2 (7.6–28.7)	0.2 (0.0–0.4)	4.7 (2.3–7.7)
Mongolia	Rate: Cumulative change (%)	-69.3 (-74.9 to -62.9)	-54.8 (-71.5 to -32.5)	-41.1 (-59.6 to -16.8)	-84.3 (-88.3 to -79.0)	-97.4 (-98.8 to -95.7)	-0.1 (-49.2 to 50.5)	nan (nan–nan)	-25.3 (-72.4 to 49.0)	-37.6 (-79.1 to 41.3)	-88.4 (-99.1 to -66.0)	-91.1 (-98.1 to -75.0)
Tajikistan	Number: 1990	19,038 (17,660–20,468)	2,010 (1,422–2,582)	1,301 (771–1,922)	7,173 (6,414–7,971)	4,090 (3,560–4,656)	674 (566–831)	0 (0–0)	230 (97–437)	519 (244–878)	50 (35–72)	352 (285–442)
Tajikistan	Number: 2015	8,338 (6,860–10,008)	1,381 (1,017–1,804)	906 (569–1,335)	2,022 (1,494–2,636)	878 (579–1,244)	859 (576–1,147)	0 (0–1)	194 (90–359)	467 (221–810)	82 (32–200)	168 (118–255)
Tajikistan	Number: Cumulative change (%)	-56.1 (-64.3 to -46.5)	-29.4 (-53.5 to 0.0)	-26.2 (-59.4 to 25.7)	-71.6 (-79.6 to -62.2)	-78.4 (-86.0 to -69.0)	29.9 (-23.5 to 82.0)	718.5 (226.3–1,507.9)	-2.4 (-64.4 to 91.3)	5.2 (-64.2 to 126.0)	70.9 (-39.9 to 302.4)	-51.4 (-67.4 to -24.1)
Tajikistan	Rate: 1990	1,944.4 (1,803.8–2,094.7)	198.6 (140.4–255.1)	128.6 (76.2–189.9)	737.8 (656.6–820.9)	419.9 (365.7–477.4)	67.8 (56.9–83.9)	0.0 (0.0–0.0)	22.9 (9.7–43.4)	51.5 (24.3–87.2)	5.2 (3.7–7.5)	36.5 (29.4–46.0)
Tajikistan	Rate: 2015	681.2 (560.3–817.6)	111.9 (82.5–146.2)	73.4 (46.1–108.2)	165.7 (122.5–215.6)	71.9 (47.4–101.9)	70.0 (47.0–93.6)	0.1 (0.0–0.2)	15.8 (7.3–29.2)	37.9 (17.9–65.7)	6.7 (2.7–16.4)	13.9 (9.7–21.1)
Tajikistan	Rate: Cumulative change (%)	-64.9 (-71.4 to -57.2)	-42.1 (-61.9 to -17.9)	-39.5 (-66.7 to 3.2)	-77.4 (-83.8 to -69.9)	-82.7 (-88.8 to -75.3)	5.4 (-38.1 to 47.5)	546.4 (159.7–1,165.4)	-20.4 (-70.9 to 55.5)	-14.0 (-70.7 to 84.6)	34.6 (-52.3 to 215.2)	-61.3 (-74.1 to -39.8)
Turkmenistan	Number: 1990	12,053 (10,653–13,575)	1,089 (716–1,478)	1,025 (629–1,409)	5,634 (4,918–6,428)	1,590 (1,332–1,876)	654 (543–783)	0 (0–1)	106 (43–199)	265 (109–522)	33 (24–43)	162 (132–194)
Turkmenistan	Number: 2015	4,426 (3,418–5,589)	645 (412–911)	532 (335–762)	1,411 (1,022–1,866)	162 (105–231)	706 (399–1,003)	0 (0–0)	116 (52–228)	255 (106–519)	4 (2–8)	43 (27–68)
Turkmenistan	Number: Cumulative change (%)	-63.1 (-72.5 to -51.8)	-38.7 (-62.9 to -6.9)	-45.5 (-69.4 to -12.1)	-74.8 (-82.5 to -65.5)	-89.7 (-93.6 to -85.0)	9.4 (-40.8 to 57.1)	-100.0 (-100.0 to -100.0)	25.7 (-52.4 to 174.5)	13.0 (-62.3 to 140.8)	-84.9 (-91.7 to -74.5)	-72.7 (-83.2 to -57.1)
Turkmenistan	Rate: 1990	2,011.2 (1,775.2–2,266.9)	175.1 (115.1–237.6)	164.8 (101.3–226.4)	947.1 (824.4–1,080.0)	266.7 (222.9–314.9)	107.4 (89.1–128.4)	0.2 (0.1–0.2)	17.3 (6.9–32.3)	42.8 (17.6–84.1)	5.7 (4.1–7.5)	27.4 (22.4–33.0)
Turkmenistan	Rate: 2015	823.7 (636.0–1,040.1)	119.9 (76.6–169.3)	99.0 (62.3–141.7)	262.9 (190.4–347.8)	30.3 (19.6–43.0)	131.5 (74.4–186.6)	0.0 (0.0–0.0)	21.6 (9.7–42.5)	47.6 (19.9–96.5)	0.9 (0.5–1.5)	8.2 (5.1–12.8)
Turkmenistan	Rate: Cumulative change (%)	-58.8 (-69.3 to -46.1)	-29.1 (-57.0 to 7.6)	-37.0 (-64.6 to 1.6)	-72.0 (-80.7 to -61.9)	-88.5 (-92.8 to -83.3)	24.2 (-33.1 to 78.7)	-100.0 (-100.0 to -100.0)	44.5 (-45.1 to 214.6)	30.1 (-56.5 to 177.1)	-83.5 (-90.9 to -72.0)	-70.0 (-81.5 to -52.9)
Uzbekistan	Number: 1990	34,442 (31,503–37,723)	2,389 (1,521–3,433)	5,362 (4,033–6,680)	14,794 (13,220–16,441)	3,206 (2,745–3,717)	1,824 (1,468–2,136)	1 (1–2)	407 (165–766)	1,208 (591–2,105)	41 (18–66)	502 (409–603)
Uzbekistan	Number: 2015	16,149 (12,967–19,751)	1,301 (809–1,962)	3,605 (2,593–4,694)	5,890 (4,579–7,358)	197 (135–274)	1,587 (1,027–2,206)	0 (0–0)	341 (141–675)	856 (411–1,506)	2 (0–4)	156 (105–228)
Uzbekistan	Number: Cumulative change (%)	-53.0 (-62.7 to -41.5)	-42.4 (-69.4 to -5.0)	-31.1 (-54.4 to -2.3)	-60.0 (-69.3 to -49.0)	-93.8 (-95.9 to -91.1)	-12.6 (-40.7 to 18.8)	-100.0 (-100.0 to -100.0)	-0.5 (-68.6 to 118.8)	-17.7 (-72.2 to 78.2)	-93.2 (-98.1 to -80.4)	-68.3 (-79.8 to -50.3)
Uzbekistan	Rate: 1990	1,036.8 (947.9–1,136.1)	71.4 (45.4–102.5)	160.2 (120.5–199.5)	446.0 (398.5–495.6)	96.7 (82.8–112.0)	54.7 (44.1–64.0)	0.1 (0.0–0.1)	12.2 (4.9–22.9)	36.1 (17.7–62.9)	1.2 (0.6–2.0)	15.2 (12.4–18.3)
Uzbekistan	Rate: 2015	499.9 (401.5–611.4)	40.4 (25.1–61.0)	112.0 (80.6–145.8)	182.0 (141.5–227.3)	6.1 (4.2–8.5)	49.2 (31.8–68.4)	0.0 (0.0–0.0)	10.6 (4.4–21.0)	26.6 (12.8–46.8)	0.1 (0.0–0.1)	4.8 (3.3–7.0)
Uzbekistan	Rate: Cumulative change (%)	-51.7 (-61.6 to -39.8)	-40.1 (-68.2 to -1.1)	-28.4 (-52.6 to 1.6)	-59.1 (-68.5 to -47.7)	-93.6 (-95.8 to -90.9)	-9.7 (-38.7 to 22.7)	-100.0 (-100.0 to -100.0)	3.3 (-67.4 to 127.2)	-14.5 (-71.1 to 85.1)	-93.1 (-98.1 to -80.0)	-67.7 (-79.4 to -49.3)
Latin America and Caribbean	Number: 1990	560,752 (554,387–567,076)	90,318 (86,083–93,981)	41,963 (39,678–44,452)	94,000 (90,544–97,812)	106,025 (101,825–110,478)	40,806 (37,559–48,258)	555 (417–726)	24,779 (21,590–31,430)	14,601 (13,358–15,941)	22,721 (20,435–25,106)	12,253 (10,490–13,700)
Latin America and Caribbean	Number: 2015	179,942 (173,237–186,947)	29,994 (27,919–32,149)	16,870 (15,560–18,294)	21,423 (19,767–23,276)	9,367 (8,325–10,712)	34,801 (30,440–37,488)	38 (18–67)	15,892 (13,827–19,108)	9,775 (8,538–10,927)	4,335 (3,781–5,217)	2,868 (2,417–3,527)
Latin America and Caribbean	Number: Cumulative change (%)	-67.9 (-69.2 to -66.6)	-66.8 (-68.8 to -64.4)	-59.8 (-63.0 to -56.3)	-77.2 (-79.4 to -74.9)	-91.2 (-92.2 to -89.9)	-13.8 (-33.2 to -2.7)	-93.0 (-96.5 to -87.8)	-35.6 (-41.6 to -29.5)	-32.9 (-41.5 to -24.0)	-80.8 (-83.9 to -76.8)	-76.4 (-81.3 to -68.9)
Latin America and Caribbean	Rate: 1990	1,079.4 (1,067.1–1,091.6)	173.4 (165.2–180.4)	80.5 (76.2–85.3)	181.1 (174.5–188.5)	204.4 (196.3–213.0)	78.5 (72.2–92.8)	1.1 (0.8–1.4)	47.6 (41.5–60.4)	28.0 (25.7–30.6)	43.8 (39.4–48.4)	23.6 (20.2–26.4)
Latin America and Caribbean	Rate: 2015	376.5 (362.4–391.1)	63.2 (58.9–67.8)	35.6 (32.8–38.6)	44.6 (41.2–48.5)	19.5 (17.3–22.3)	72.9 (63.7–78.5)	0.1 (0.0–0.1)	33.5 (29.1–40.3)	20.6 (18.0–23.0)	9.0 (7.8–10.8)	6.0 (5.0–7.3)
Latin America and Caribbean	Rate: Cumulative change (%)	-65.1 (-66.5 to -63.7)	-63.5 (-65.8 to -60.9)	-55.8 (-59.3 to -52.0)	-75.3 (-77.8 to -72.9)	-90.5 (-91.6 to -89.1)	-6.2 (-27.2 to 6.0)	-92.5 (-96.3 to -86.9)	-29.4 (-36.0 to -22.7)	-26.4 (-35.9 to -16.7)	-79.4 (-82.7 to -75.1)	-74.5 (-79.8 to -66.4)
Central Latin America	Number: 1990	210,045 (206,919–213,319)	31,927 (30,164–33,460)	18,532 (17,435–19,554)	32,020 (30,807–33,274)	37,615 (36,256–38,952)	20,836 (19,873–22,122)	129 (102–160)	8,584 (7,042–11,489)	6,459 (5,934–6,968)	7,455 (6,966–7,934)	3,214 (3,040–3,402)
Central Latin America	Number: 2015	78,223 (74,231–82,718)	13,254 (11,982–14,477)	5,894 (5,310–6,495)	9,664 (8,864–10,539)	3,825 (3,474–4,219)	18,440 (16,733–19,903)	6 (3–10)	6,190 (4,975–7,866)	3,064 (2,656–3,477)	2,244 (1,985–2,544)	756 (681–856)
Central Latin America	Number: Cumulative change (%)	-62.8 (-64.8 to -60.6)	-58.5 (-61.8 to -54.9)	-68.2 (-71.2 to -65.1)	-69.8 (-72.5 to -67.0)	-89.8 (-90.8 to -88.7)	-11.4 (-20.3 to -3.5)	-95.1 (-97.2 to -91.6)	-27.5 (-36.2 to -18.8)	-52.5 (-58.1 to -46.6)	-69.9 (-73.8 to -65.2)	-76.5 (-78.8 to -73.7)
Central Latin America	Rate: 1990	887.9 (874.6–901.8)	134.2 (126.8–140.7)	77.9 (73.3–82.2)	135.5 (130.4–140.8)	159.4 (153.6–165.0)	87.9 (83.9–93.3)	0.5 (0.4–0.7)	36.1 (29.6–48.3)	27.2 (25.0–29.3)	31.6 (29.6–33.7)	13.6 (12.9–14.4)
Central Latin America	Rate: 2015	343.2 (325.7–362.9)	58.7 (53.0–64.1)	26.1 (23.5–28.7)	42.2 (38.7–46.0)	16.6 (15.1–18.4)	81.0 (73.5–87.5)	0.0 (0.0–0.0)	27.4 (22.0–34.8)	13.5 (11.7–15.4)	9.8 (8.6–11.1)	3.3 (3.0–3.7)
Central Latin America	Rate: Cumulative change (%)	-61.3 (-63.5 to -59.1)	-56.3 (-59.8 to -52.5)	-66.5 (-69.7 to -63.3)	-68.8 (-71.6 to -66.0)	-89.6 (-90.6 to -88.4)	-7.7 (-17.0 to 0.6)	-95.0 (-97.1 to -91.4)	-23.8 (-33.0 to -14.7)	-50.1 (-56.0 to -43.9)	-69.1 (-73.1 to -64.4)	-75.8 (-78.2 to -72.9)
Colombia	Number: 1990	29,066 (27,474–30,661)	4,225 (3,426–4,817)	2,806 (2,285–3,258)	4,107 (3,673–4,554)	4,095 (3,668–4,564)	3,153 (2,434–3,505)	48 (32–70)	1,242 (700–2,618)	781 (625–932)	1,159 (979–1,367)	920 (815–1,028)
Colombia	Number: 2015	11,882 (9,281–15,166)	1,616 (1,073–2,219)	681 (450–947)	1,234 (940–1,598)	335 (253–436)	2,919 (1,955–3,883)	2 (1–5)	1,047 (663–1,760)	643 (427–896)	542 (401–730)	175 (134–229)
Colombia	Number: Cumulative change (%)	-59.1 (-68.3 to -47.8)	-61.7 (-73.2 to -47.1)	-75.6 (-83.6 to -66.1)	-69.8 (-77.5 to -60.6)	-91.8 (-93.8 to -89.0)	-7.5 (-32.3 to 22.6)	-93.7 (-97.7 to -86.7)	-5.7 (-46.5 to 39.1)	-17.3 (-43.1 to 15.3)	-52.7 (-66.8 to -33.8)	-80.9 (-85.8 to -74.8)
Colombia	Rate: 1990	663.1 (626.9–699.5)	96.7 (78.4–110.3)	64.2 (52.3–74.6)	93.6 (83.7–103.8)	93.3 (83.6–104.0)	72.0 (55.6–80.0)	1.1 (0.7–1.6)	28.4 (16.0–59.9)	17.9 (14.3–21.3)	26.4 (22.3–31.1)	21.0 (18.6–23.4)
Colombia	Rate: 2015	323.6 (252.5–413.3)	44.7 (29.7–61.4)	18.8 (12.4–26.2)	33.4 (25.4–43.3)	9.0 (6.8–11.8)	79.8 (53.5–106.2)	0.1 (0.0–0.2)	28.9 (18.3–48.6)	17.8 (11.8–24.8)	14.6 (10.8–19.7)	4.7 (3.6–6.2)
Colombia	Rate: Cumulative change (%)	-51.1 (-62.2 to -37.7)	-53.7 (-67.6 to -36.1)	-70.6 (-80.2 to -59.1)	-64.1 (-73.2 to -53.2)	-90.3 (-92.7 to -87.0)	10.8 (-19.0 to 46.8)	-92.6 (-97.4 to -84.3)	13.8 (-35.4 to 67.9)	-0.2 (-31.3 to 39.3)	-44.0 (-60.7 to -21.9)	-77.3 (-83.2 to -70.1)
Costa Rica	Number: 1990	1,666 (1,487–1,869)	429 (365–503)	136 (107–167)	159 (134–189)	99 (83–119)	390 (225–472)	0 (0–0)	46 (28–63)	82 (64–103)	18 (14–23)	26 (21–31)
Costa Rica	Number: 2015	683 (524–886)	121 (89–163)	43 (30–60)	36 (25–50)	16 (11–22)	264 (175–354)	0 (0–0)	17 (9–33)	46 (33–62)	2 (1–4)	7 (5–10)
Costa Rica	Number: Cumulative change (%)	-58.8 (-69.7 to -46.0)	-71.4 (-79.6 to -61.1)	-67.5 (-78.7 to -53.0)	-76.9 (-84.2 to -68.2)	-83.5 (-88.8 to -77.2)	-31.1 (-52.3 to -3.7)	-77.4 (-83.7 to -70.1)	-60.9 (-80.4 to -27.4)	-42.2 (-61.5 to -17.9)	-83.8 (-89.9 to -76.0)	-70.4 (-79.6 to -59.1)
Costa Rica	Rate: 1990	417.2 (372.5–467.8)	107.5 (91.7–126.1)	34.1 (26.9–41.9)	39.7 (33.5–47.4)	24.9 (20.8–29.8)	97.7 (56.5–118.2)	0.0 (0.0–0.0)	11.6 (7.1–15.9)	20.6 (16.3–25.9)	4.5 (3.5–5.8)	6.5 (5.5–7.9)
Costa Rica	Rate: 2015	199.7 (153.3–259.3)	36.0 (26.6–48.4)	12.8 (9.0–17.9)	10.6 (7.5–14.7)	4.7 (3.3–6.5)	77.4 (51.3–103.6)	0.0 (0.0–0.0)	5.2 (2.9–10.0)	13.7 (9.9–18.5)	0.8 (0.6–1.2)	2.2 (1.6–3.1)
Costa Rica	Rate: Cumulative change (%)	-51.9 (-64.6 to -36.9)	-66.3 (-75.9 to -54.1)	-61.8 (-74.9 to -44.7)	-73.1 (-81.6 to -63.1)	-80.9 (-87.1 to -73.6)	-19.4 (-44.2 to 12.6)	-74.1 (-81.4 to -65.8)	-53.9 (-76.9 to -14.5)	-31.9 (-54.7 to -3.3)	-81.3 (-88.3 to -72.3)	-65.7 (-76.4 to -52.7)
El Salvador	Number: 1990	8,617 (7,901–9,414)	1,475 (1,267–1,680)	509 (400–621)	927 (769–1,095)	1,768 (1,472–2,083)	799 (597–1,092)	0 (0–0)	431 (321–629)	270 (213–337)	151 (113–195)	124 (100–150)
El Salvador	Number: 2015	1,192 (891–1,583)	193 (127–278)	61 (39–91)	156 (110–208)	49 (33–68)	381 (246–527)	0 (0–0)	73 (40–157)	33 (19–49)	14 (8–21)	14 (9–21)
El Salvador	Number: Cumulative change (%)	-86.1 (-89.9 to -81.5)	-86.8 (-91.2 to -80.8)	-87.8 (-92.5 to -81.2)	-82.9 (-88.5 to -75.9)	-97.2 (-98.2 to -95.9)	-50.1 (-74.5 to -23.1)	-91.8 (-94.5 to -88.7)	-83.1 (-90.8 to -70.8)	-87.4 (-93.2 to -79.5)	-90.6 (-94.3 to -85.5)	-88.1 (-92.5 to -81.6)
El Salvador	Rate: 1990	1,103.5 (1,011.8–1,205.5)	188.6 (162.0–214.9)	65.1 (51.2–79.5)	118.9 (98.6–140.3)	226.8 (188.7–267.1)	102.4 (76.5–139.9)	0.1 (0.1–0.1)	55.2 (41.2–80.5)	34.7 (27.3–43.2)	19.4 (14.5–25.1)	15.9 (12.9–19.3)
El Salvador	Rate: 2015	230.5 (172.4–306.2)	38.0 (24.9–54.6)	12.0 (7.8–17.9)	30.0 (21.3–40.1)	9.5 (6.4–13.1)	73.9 (47.8–102.5)	0.0 (0.0–0.0)	14.4 (7.9–30.8)	6.5 (3.9–9.6)	2.7 (1.7–4.0)	2.8 (1.8–4.1)
El Salvador	Rate: Cumulative change (%)	-79.0 (-84.7 to -72.0)	-79.8 (-86.5 to -70.5)	-81.2 (-88.5 to -71.2)	-74.4 (-82.8 to -63.8)	-95.8 (-97.2 to -93.9)	-24.5 (-61.4 to 16.5)	-87.9 (-91.9 to -83.3)	-74.1 (-85.9 to -55.3)	-80.8 (-89.6 to -68.7)	-86.0 (-91.5 to -78.5)	-82.1 (-88.8 to -72.6)
Guatemala	Number: 1990	28,022 (27,102–28,965)	2,422 (1,929–2,838)	841 (644–1,110)	5,936 (5,332–6,555)	6,190 (5,531–6,836)	754 (604–1,185)	35 (23–52)	1,093 (766–1,703)	916 (678–1,150)	1,380 (1,156–1,612)	178 (156–202)
Guatemala	Number: 2015	11,283 (9,525–13,406)	1,281 (1,002–1,573)	728 (553–906)	2,993 (2,417–3,646)	1,300 (1,023–1,624)	1,380 (1,119–1,680)	0 (0–0)	630 (441–1,025)	373 (285–465)	517 (378–675)	121 (95–153)
Guatemala	Number: Cumulative change (%)	-59.7 (-66.2 to -51.9)	-46.7 (-57.8 to -34.9)	-11.1 (-38.7 to 23.4)	-49.4 (-60.2 to -36.2)	-78.9 (-83.8 to -72.9)	90.8 (14.3–151.6)	-99.0 (-99.4 to -98.3)	-41.7 (-58.1 to -22.6)	-58.4 (-69.7 to -45.0)	-62.2 (-73.3 to -49.6)	-31.3 (-48.6 to -9.8)
Guatemala	Rate: 1990	1,715.7 (1,657.8–1,773.7)	142.8 (113.7–167.3)	49.7 (38.0–65.5)	364.4 (327.6–403.0)	383.4 (341.6–423.7)	45.2 (36.2–71.2)	2.2 (1.5–3.3)	64.5 (45.2–100.5)	54.1 (40.1–67.9)	85.6 (71.5–100.2)	11.0 (9.6–12.4)
Guatemala	Rate: 2015	534.3 (451.1–634.7)	60.6 (47.4–74.4)	34.4 (26.2–42.9)	141.8 (114.5–172.5)	61.7 (48.6–77.1)	65.3 (53.0–79.4)	0.0 (0.0–0.0)	29.8 (20.9–48.5)	17.7 (13.5–22.0)	24.5 (17.9–32.0)	5.8 (4.5–7.3)
Guatemala	Rate: Cumulative change (%)	-68.8 (-73.9 to -62.8)	-57.3 (-66.1 to -47.8)	-28.8 (-50.9 to -1.1)	-60.9 (-69.3 to -50.9)	-83.8 (-87.6 to -79.3)	50.8 (-9.8 to 99.0)	-99.2 (-99.6 to -98.7)	-53.3 (-66.4 to -38.0)	-66.8 (-75.8 to -56.0)	-71.1 (-79.5 to -61.5)	-46.9 (-60.3 to -30.4)
Honduras	Number: 1990	10,126 (9,763–10,519)	1,221 (920–1,511)	538 (330–805)	898 (752–1,055)	2,003 (1,704–2,323)	1,117 (778–1,706)	3 (2–5)	505 (258–855)	201 (103–349)	167 (104–257)	92 (58–138)
Honduras	Number: 2015	3,755 (3,279–4,320)	577 (435–724)	268 (177–387)	338 (260–428)	248 (179–327)	834 (651–1,065)	0 (0–0)	317 (177–483)	71 (40–111)	58 (29–99)	36 (21–53)
Honduras	Number: Cumulative change (%)	-62.9 (-67.6 to -57.2)	-51.8 (-65.7 to -34.2)	-46.6 (-71.6 to -9.5)	-61.9 (-72.1 to -49.7)	-87.5 (-91.2 to -82.9)	-21.4 (-53.4 to 10.0)	-87.3 (-95.5 to -73.1)	-31.2 (-67.5 to 17.2)	-59.6 (-84.2 to -20.3)	-62.9 (-83.2 to -30.1)	-58.1 (-77.1 to -26.3)
Honduras	Rate: 1990	1,142.6 (1,101.4–1,186.3)	135.0 (101.7–167.1)	59.5 (36.5–89.1)	101.4 (85.0–119.1)	227.8 (193.8–264.3)	125.2 (87.2–191.2)	0.4 (0.3–0.7)	56.0 (28.6–94.6)	22.4 (11.4–38.7)	19.3 (12.1–29.6)	10.5 (6.6–15.9)
Honduras	Rate: 2015	456.9 (398.6–525.8)	70.9 (53.4–88.9)	32.9 (21.8–47.5)	41.1 (31.6–52.1)	30.0 (21.7–39.6)	101.6 (79.4–129.7)	0.1 (0.0–0.1)	38.9 (21.8–59.3)	8.7 (5.0–13.7)	7.0 (3.5–12.0)	4.4 (2.6–6.5)
Honduras	Rate: Cumulative change (%)	-60.0 (-65.1 to -53.9)	-46.5 (-61.9 to -27.0)	-40.8 (-68.5 to 0.4)	-59.0 (-69.9 to -45.9)	-86.7 (-90.7 to -81.8)	-14.4 (-49.3 to 19.7)	-86.7 (-95.2 to -71.7)	-23.8 (-63.9 to 29.8)	-55.3 (-82.5 to -12.0)	-61.3 (-82.5 to -27.2)	-55.4 (-75.7 to -21.6)
Mexico	Number: 1990	105,285 (102,947–107,672)	16,938 (16,054–17,900)	11,476 (10,820–12,177)	16,693 (15,789–17,569)	18,101 (17,242–18,949)	11,749 (11,253–12,220)	5 (4–5)	3,826 (2,682–5,043)	3,597 (3,321–3,883)	3,765 (3,468–4,063)	1,230 (1,153–1,308)
Mexico	Number: 2015	36,119 (33,845–38,642)	6,719 (5,981–7,490)	3,010 (2,656–3,400)	3,343 (3,012–3,721)	1,094 (985–1,221)	9,795 (8,865–10,681)	1 (0–1)	2,998 (2,157–3,678)	1,540 (1,324–1,761)	769 (671–879)	207 (187–227)
Mexico	Number: Cumulative change (%)	-65.7 (-68.0 to -63.0)	-60.3 (-64.6 to -56.0)	-73.8 (-76.8 to -70.5)	-80.0 (-82.1 to -77.4)	-94.0 (-94.6 to -93.2)	-16.5 (-24.3 to -8.6)	-80.5 (-82.8 to -78.0)	-21.2 (-30.2 to -10.5)	-57.1 (-62.8 to -50.8)	-79.5 (-82.5 to -76.2)	-83.1 (-84.9 to -81.1)
Mexico	Rate: 1990	895.3 (875.3–915.6)	143.2 (135.7–151.3)	97.0 (91.5–102.9)	142.1 (134.4–149.6)	154.4 (147.1–161.7)	99.7 (95.5–103.7)	0.0 (0.0–0.1)	32.4 (22.7–42.7)	30.4 (28.1–32.9)	32.2 (29.6–34.7)	10.5 (9.8–11.2)
Mexico	Rate: 2015	315.8 (295.9–337.9)	59.2 (52.7–66.0)	26.5 (23.4–29.9)	29.1 (26.2–32.4)	9.5 (8.5–10.6)	85.7 (77.5–93.4)	0.0 (0.0–0.0)	26.4 (19.0–32.4)	13.6 (11.7–15.5)	6.7 (5.8–7.6)	1.8 (1.6–2.0)
Mexico	Rate: Cumulative change (%)	-64.7 (-67.1 to -62.0)	-58.7 (-63.1 to -54.2)	-72.7 (-75.8 to -69.3)	-79.5 (-81.7 to -76.9)	-93.9 (-94.5 to -93.1)	-14.0 (-22.0 to -5.7)	-80.5 (-82.7 to -77.9)	-18.0 (-27.4 to -6.9)	-55.4 (-61.3 to -48.9)	-79.3 (-82.2 to -75.9)	-82.8 (-84.7 to -80.8)
Nicaragua	Number: 1990	9,350 (9,041–9,683)	1,303 (1,128–1,495)	717 (567–899)	1,325 (1,131–1,534)	2,748 (2,454–3,049)	723 (385–1,042)	28 (17–41)	421 (270–549)	138 (102–188)	270 (203–335)	193 (154–244)
Nicaragua	Number: 2015	1,872 (1,584–2,221)	295 (228–371)	177 (128–238)	330 (263–408)	98 (68–133)	396 (263–530)	0 (0–0)	157 (113–206)	32 (23–41)	57 (40–76)	32 (23–47)
Nicaragua	Number: Cumulative change (%)	-80.0 (-83.2 to -76.2)	-77.2 (-82.9 to -70.6)	-75.0 (-82.7 to -65.8)	-74.8 (-81.2 to -66.8)	-96.4 (-97.5 to -95.1)	-41.5 (-68.7 to -13.6)	-99.4 (-99.7 to -99.0)	-61.8 (-73.1 to -47.2)	-76.2 (-84.7 to -65.5)	-78.4 (-85.1 to -69.9)	-83.1 (-88.1 to -76.0)
Nicaragua	Rate: 1990	1,316.4 (1,273.1–1,363.4)	182.4 (158.0–209.3)	100.5 (79.4–125.9)	187.1 (159.7–216.6)	387.9 (346.4–430.3)	101.7 (54.2–146.5)	4.0 (2.5–5.9)	59.1 (37.9–77.0)	19.5 (14.4–26.4)	38.2 (28.7–47.4)	27.4 (21.8–34.5)
Nicaragua	Rate: 2015	315.2 (266.4–374.0)	50.3 (39.0–63.4)	30.2 (22.0–40.6)	55.3 (44.2–68.6)	16.4 (11.4–22.3)	66.9 (44.3–89.6)	0.0 (0.0–0.0)	26.8 (19.4–35.2)	5.4 (4.0–7.1)	9.6 (6.7–12.7)	5.4 (3.9–7.9)
Nicaragua	Rate: Cumulative change (%)	-76.0 (-79.9 to -71.5)	-72.2 (-79.2 to -64.2)	-69.6 (-78.9 to -58.4)	-70.1 (-77.7 to -60.6)	-95.8 (-97.1 to -94.2)	-29.8 (-62.5 to 3.7)	-99.4 (-99.7 to -98.9)	-53.5 (-67.3 to -35.8)	-71.2 (-81.5 to -58.1)	-74.6 (-82.5 to -64.6)	-80.1 (-85.9 to -71.6)
Panama	Number: 1990	1,762 (1,563–1,967)	303 (254–355)	132 (98–177)	157 (126–196)	155 (121–196)	315 (158–430)	0 (0–0)	78 (56–112)	60 (44–81)	73 (54–94)	47 (38–58)
Panama	Number: 2015	1,316 (1,035–1,671)	177 (118–249)	109 (70–159)	165 (125–217)	86 (63–114)	299 (160–440)	0 (0–0)	87 (57–122)	54 (35–79)	49 (35–66)	24 (17–35)
Panama	Number: Cumulative change (%)	-24.9 (-42.7 to -2.8)	-41.4 (-61.2 to -18.5)	-16.2 (-46.0 to 22.0)	6.2 (-21.9 to 40.2)	-43.4 (-60.5 to -22.5)	-2.6 (-31.1 to 34.1)	22.8 (-9.3 to 64.0)	13.3 (-24.4 to 63.5)	-8.5 (-42.1 to 36.6)	-31.9 (-53.3 to -3.4)	-46.7 (-65.0 to -19.9)
Panama	Rate: 1990	563.9 (500.3–629.3)	97.2 (81.5–113.9)	42.5 (31.4–56.8)	50.4 (40.4–62.9)	49.9 (39.0–62.7)	100.8 (50.8–137.8)	0.0 (0.0–0.0)	25.2 (18.2–35.9)	19.4 (14.3–26.2)	23.6 (17.5–30.3)	15.1 (12.3–18.6)
Panama	Rate: 2015	360.2 (283.2–457.8)	48.8 (32.7–68.6)	30.1 (19.5–44.0)	45.0 (34.3–59.2)	23.7 (17.3–31.3)	82.1 (44.1–120.7)	0.0 (0.0–0.0)	23.9 (15.7–33.8)	14.9 (9.7–21.8)	13.3 (9.5–18.1)	6.8 (4.8–9.7)
Panama	Rate: Cumulative change (%)	-35.8 (-51.0 to -16.9)	-49.6 (-66.6 to -29.9)	-28.0 (-53.6 to 4.9)	-9.4 (-33.4 to 19.7)	-51.8 (-66.4 to -34.0)	-16.6 (-41.0 to 15.0)	4.2 (-23.1 to 39.1)	-2.6 (-35.0 to 40.6)	-21.3 (-50.3 to 17.4)	-42.1 (-60.3 to -17.8)	-54.6 (-70.2 to -31.7)
Venezuela	Number: 1990	16,146 (16,032–16,256)	3,607 (3,230–3,983)	1,373 (1,128–1,648)	1,815 (1,666–1,980)	2,451 (2,263–2,642)	1,833 (1,603–2,219)	6 (4–9)	937 (536–1,223)	408 (320–507)	468 (396–549)	401 (359–447)
Venezuela	Number: 2015	10,118 (9,563–10,744)	2,271 (1,951–2,647)	814 (678–972)	1,066 (938–1,214)	596 (512–692)	2,168 (1,932–2,415)	1 (0–2)	860 (537–1,091)	269 (217–328)	233 (183–294)	136 (109–160)
Venezuela	Number: Cumulative change (%)	-37.3 (-40.8 to -33.5)	-36.9 (-45.8 to -27.6)	-40.0 (-53.2 to -24.7)	-41.1 (-49.9 to -31.6)	-75.6 (-79.5 to -71.2)	19.2 (-1.2 to 39.5)	-80.3 (-91.7 to -59.7)	-6.6 (-27.2 to 14.0)	-33.1 (-50.9 to -13.4)	-49.8 (-62.2 to -35.3)	-65.9 (-73.4 to -58.5)
Venezuela	Rate: 1990	581.8 (577.7–585.8)	129.9 (116.3–143.4)	49.4 (40.6–59.4)	65.4 (60.1–71.4)	88.3 (81.6–95.2)	66.0 (57.7–79.9)	0.2 (0.2–0.4)	33.8 (19.3–44.0)	14.7 (11.5–18.3)	16.9 (14.3–19.8)	14.4 (13.0–16.1)
Venezuela	Rate: 2015	346.8 (327.6–368.3)	78.4 (67.4–91.4)	28.1 (23.4–33.6)	36.4 (32.0–41.4)	20.3 (17.5–23.6)	74.3 (66.2–82.8)	0.0 (0.0–0.1)	29.7 (18.5–37.6)	9.3 (7.5–11.3)	7.9 (6.2–10.0)	4.6 (3.7–5.5)
Venezuela	Rate: Cumulative change (%)	-40.4 (-43.7 to -36.7)	-39.5 (-48.0 to -30.6)	-42.5 (-55.1 to -27.8)	-44.2 (-52.5 to -35.1)	-77.0 (-80.6 to -72.8)	13.5 (-6.0 to 32.8)	-81.5 (-92.2 to -62.3)	-10.6 (-30.2 to 9.2)	-35.9 (-53.0 to -17.0)	-52.7 (-64.3 to -39.1)	-67.8 (-74.8 to -60.8)

© 2017 Global Burden of Disease Child and Adolescent Health Collaboration. JAMA Pediatrics.

eTable 2. Number of Deaths, Death Rates (per 100,000 population), and Cumulative Percent Change with 95% Uncertainty Intervals (UI) for the Top 10 Global Causes of Death in 195 Countries and Territories, Aged Under 5 Years, Both Sexes, 1990 and 2015 *Note: best viewed by enlarging in browser.*

Location	Measure	All causes	Neonatal preterm birth complications	Neonatal encephalopathy due to birth asphyxia and trauma	Lower respiratory infections	Diarrheal diseases	Congenital anomalies	Malaria	Neonatal sepsis and other neonatal infections	Other neonatal disorders	Protein-energy malnutrition	Meningitis
Andean Latin America	Number: 1990	92,434 (90,703–94,086)	8,778 (7,436–10,263)	4,969 (3,995–6,032)	23,232 (21,352–25,348)	13,155 (11,768–14,602)	3,438 (2,821–5,435)	25 (17–34)	4,127 (2,962–6,560)	1,557 (1,152–2,064)	3,761 (2,962–4,814)	1,141 (832–1,510)
Andean Latin America	Number: 2015	23,000 (21,093–24,946)	3,123 (2,542–3,663)	2,508 (1,960–3,073)	3,567 (3,102–4,092)	927 (732–1,146)	3,421 (2,698–4,062)	0 (0–1)	2,472 (1,874–3,481)	642 (448–886)	433 (309–641)	293 (230–388)
Andean Latin America	Number: Cumulative change (%)	-75.1 (-77.2 to -73.0)	-64.2 (-70.9 to -55.8)	-49.1 (-59.8 to -36.5)	-84.6 (-87.3 to -81.7)	-92.9 (-94.6 to -90.9)	4.2 (-41.3 to 29.4)	-96.3 (-97.6 to -94.4)	-38.3 (-53.7 to -16.6)	-57.5 (-73.0 to -36.8)	-88.3 (-92.6 to -82.3)	-73.3 (-82.3 to -58.4)
Andean Latin America	Rate: 1990	1,626.5 (1,595.5–1,656.7)	151.2 (128.1–176.8)	85.6 (68.8–103.9)	408.7 (376.0–446.2)	233.7 (209.0–259.3)	60.0 (49.2–95.0)	0.4 (0.3–0.6)	71.2 (51.1–113.2)	26.9 (19.9–35.6)	67.0 (52.8–85.8)	20.2 (14.7–26.8)
Andean Latin America	Rate: 2015	394.5 (361.7–427.9)	53.9 (43.9–63.2)	43.3 (33.8–53.0)	61.0 (53.1–70.0)	15.8 (12.5–19.6)	58.7 (46.4–69.7)	0.0 (0.0–0.0)	42.6 (32.3–60.0)	11.1 (7.7–15.3)	7.4 (5.3–10.9)	5.0 (3.9–6.6)
Andean Latin America	Rate: Cumulative change (%)	-75.7 (-77.8 to -73.6)	-64.1 (-70.9 to -55.7)	-49.0 (-59.8 to -36.4)	-85.0 (-87.7 to -82.3)	-93.2 (-94.8 to -91.2)	2.6 (-42.4 to 27.6)	-96.4 (-97.8 to -94.7)	-38.3 (-53.8 to -16.6)	-57.6 (-73.1 to -36.8)	-88.8 (-92.9 to -83.1)	-74.2 (-83.0 to -59.9)
Bolivia	Number: 1990	26,796 (26,137–27,524)	2,400 (1,689–3,128)	1,980 (1,279–2,763)	5,609 (4,650–6,656)	5,651 (4,468–6,862)	798 (438–1,595)	0 (0–0)	1,770 (968–2,982)	516 (229–929)	990 (419–1,912)	576 (316–981)
Bolivia	Number: 2015	7,423 (6,302–8,766)	971 (631–1,386)	1,092 (676–1,557)	930 (667–1,250)	469 (300–679)	666 (407–1,168)	0 (0–0)	987 (537–1,617)	282 (119–498)	125 (42–279)	116 (64–188)
Bolivia	Number: Cumulative change (%)	-72.3 (-76.6 to -67.3)	-58.6 (-74.4 to -38.9)	-42.6 (-66.2 to -9.3)	-83.2 (-88.6 to -76.2)	-91.5 (-94.9 to -86.9)	-8.0 (-58.4 to 37.9)	-45.4 (-75.9 to 0.4)	-40.3 (-69.1 to 3.7)	-35.8 (-79.5 to 38.4)	-84.1 (-96.7 to -61.0)	-76.8 (-90.8 to -54.9)
Bolivia	Rate: 1990	2,397.0 (2,335.1–2,461.8)	207.4 (146.0–269.9)	171.2 (110.7–239.3)	503.5 (418.2–598.0)	513.8 (406.8–623.6)	70.3 (38.7–140.5)	0.0 (0.0–0.0)	153.5 (84.1–258.2)	44.8 (19.8–80.2)	90.5 (38.4–175.6)	52.3 (28.6–89.6)
Bolivia	Rate: 2015	607.6 (515.6–717.5)	79.7 (51.8–113.6)	89.6 (55.4–127.7)	76.1 (54.6–102.3)	38.4 (24.5–55.5)	54.5 (33.3–95.7)	0.0 (0.0–0.0)	80.9 (44.1–132.6)	23.1 (9.8–40.8)	10.3 (3.5–22.8)	9.5 (5.3–15.4)
Bolivia	Rate: Cumulative change (%)	-74.6 (-78.6 to -70.0)	-60.7 (-75.7 to -42.0)	-45.5 (-67.9 to -14.1)	-84.7 (-89.7 to -78.2)	-92.4 (-95.5 to -88.2)	-14.5 (-61.5 to 28.3)	-51.2 (-78.6 to -10.4)	-43.6 (-70.7 to -2.2)	-39.3 (-80.7 to 31.1)	-85.8 (-97.0 to -65.0)	-79.1 (-91.8 to -59.4)
Ecuador	Number: 1990	14,723 (14,137–15,302)	1,659 (1,461–1,879)	645 (524–781)	3,010 (2,720–3,290)	2,612 (2,336–2,903)	908 (798–1,040)	18 (11–26)	431 (203–575)	409 (328–500)	786 (628–933)	197 (167–229)
Ecuador	Number: 2015	5,862 (5,156–6,691)	989 (802–1,200)	376 (289–476)	1,099 (926–1,318)	227 (178–289)	1,132 (738–1,416)	0 (0–0)	303 (197–396)	239 (183–303)	134 (96–191)	70 (51–98)
Ecuador	Number: Cumulative change (%)	-60.2 (-65.5 to -54.3)	-40.1 (-51.2 to -27.1)	-41.0 (-56.3 to -22.6)	-63.4 (-69.9 to -55.6)	-91.2 (-93.3 to -88.8)	25.8 (-25.4 to 61.2)	-97.9 (-98.9 to -96.1)	-26.2 (-49.7 to 8.3)	-40.8 (-57.1 to -20.4)	-82.6 (-88.4 to -73.0)	-64.1 (-74.2 to -49.4)
Ecuador	Rate: 1990	1,004.7 (964.7–1,044.3)	111.9 (98.5–126.7)	43.5 (35.4–52.7)	205.5 (185.9–224.6)	179.0 (160.0–199.1)	61.7 (54.2–70.7)	1.2 (0.8–1.8)	29.1 (13.7–38.8)	27.6 (22.1–33.7)	54.0 (43.2–64.0)	13.5 (11.4–15.7)
Ecuador	Rate: 2015	363.9 (319.9–415.6)	61.8 (50.0–74.9)	23.5 (18.0–29.7)	68.1 (57.4–81.7)	14.1 (11.1–17.9)	70.4 (45.9–87.9)	0.0 (0.0–0.0)	18.9 (12.3–24.8)	14.9 (11.4–18.9)	8.3 (6.0–11.9)	4.3 (3.2–6.1)
Ecuador	Rate: Cumulative change (%)	-63.7 (-68.6 to -58.5)	-44.6 (-54.8 to -32.5)	-45.4 (-59.6 to -28.4)	-66.8 (-72.7 to -59.8)	-92.1 (-94.0 to -89.9)	15.1 (-31.7 to 47.4)	-98.1 (-99.0 to -96.5)	-31.7 (-53.5 to 0.1)	-45.3 (-60.4 to -26.4)	-84.3 (-89.6 to -75.7)	-67.5 (-76.6 to -54.3)
Peru	Number: 1990	50,914 (49,460–52,410)	4,718 (3,724–5,929)	2,343 (1,833–2,961)	14,611 (13,207–16,176)	4,891 (4,224–5,562)	1,731 (1,365–2,735)	6 (4–10)	1,925 (1,290–3,416)	631 (444–921)	1,984 (1,620–2,432)	367 (295–486)
Peru	Number: 2015	9,715 (8,518–11,129)	1,161 (881–1,470)	1,038 (780–1,337)	1,537 (1,311–1,793)	230 (182–289)	1,622 (1,211–1,996)	0 (0–0)	1,181 (890–1,639)	120 (87–157)	172 (123–264)	106 (70–138)
Peru	Number: Cumulative change (%)	-80.9 (-83.2 to -78.1)	-74.9 (-81.8 to -66.3)	-55.1 (-67.1 to -40.4)	-89.4 (-91.3 to -87.3)	-95.3 (-96.4 to -93.9)	-1.2 (-51.8 to 29.3)	-93.7 (-96.3 to -90.3)	-35.2 (-56.4 to -8.4)	-79.9 (-88.2 to -70.1)	-91.2 (-94.1 to -87.3)	-69.8 (-84.7 to -57.2)
Peru	Rate: 1990	1,642.5 (1,596.1–1,691.0)	149.0 (117.6–187.4)	74.0 (57.9–93.5)	470.8 (425.4–520.8)	159.3 (137.5–180.9)	55.4 (43.7–87.8)	0.2 (0.1–0.3)	60.9 (40.8–108.1)	20.0 (14.0–29.1)	64.9 (53.0–79.5)	11.9 (9.6–15.8)
Peru	Rate: 2015	323.9 (284.0–371.1)	39.1 (29.6–49.5)	34.9 (26.3–45.0)	51.0 (43.6–59.6)	7.6 (6.1–9.6)	54.2 (40.4–66.7)	0.0 (0.0–0.0)	39.7 (29.9–55.1)	4.1 (2.9–5.3)	5.7 (4.1–8.7)	3.5 (2.3–4.6)
Peru	Rate: Cumulative change (%)	-80.3 (-82.7 to -77.4)	-73.3 (-80.6 to -64.1)	-52.2 (-64.9 to -36.5)	-89.1 (-91.1 to -86.9)	-95.2 (-96.4 to -93.7)	3.2 (-49.9 to 35.3)	-93.6 (-96.3 to -90.2)	-31.2 (-53.7 to -2.8)	-78.7 (-87.4 to -68.2)	-91.1 (-94.0 to -87.1)	-69.2 (-84.4 to -56.3)
Caribbean	Number: 1990	62,198 (59,868–64,659)	7,303 (6,464–8,178)	4,272 (3,400–5,195)	8,230 (6,977–9,588)	14,298 (12,317–16,506)	4,040 (3,199–5,185)	135 (53–263)	2,378 (1,713–3,360)	2,538 (1,686–3,613)	2,844 (1,885–4,345)	2,409 (1,713–3,464)
Caribbean	Number: 2015	24,979 (20,940–29,797)	3,476 (2,893–4,172)	2,384 (1,758–3,120)	3,292 (2,449–4,317)	2,763 (1,877–3,965)	3,177 (2,505–3,926)	25 (5–55)	1,959 (1,399–2,631)	1,544 (1,002–2,281)	633 (299–1,235)	814 (487–1,329)
Caribbean	Number: Cumulative change (%)	-59.8 (-66.6 to -51.9)	-52.2 (-61.3 to -42.3)	-43.4 (-60.2 to -23.4)	-59.6 (-71.7 to -45.1)	-80.5 (-86.9 to -71.4)	-19.7 (-47.4 to 4.3)	-78.2 (-94.7 to -46.8)	-15.6 (-40.5 to 19.8)	-36.6 (-62.9 to 2.0)	-76.3 (-90.8 to -50.5)	-64.7 (-82.0 to -40.3)
Caribbean	Rate: 1990	1,455.7 (1,400.7–1,513.3)	169.5 (150.0–189.8)	99.2 (79.0–120.6)	193.0 (163.5–225.0)	335.6 (289.0–387.7)	94.4 (74.7–121.2)	3.2 (1.2–6.2)	55.3 (39.9–78.2)	59.0 (39.2–84.0)	66.9 (44.3–102.3)	56.6 (40.2–81.3)
Caribbean	Rate: 2015	659.5 (553.3–786.4)	92.3 (76.8–110.8)	63.3 (46.7–82.9)	86.8 (64.5–113.8)	72.8 (49.4–104.4)	83.9 (66.2–103.7)	0.7 (0.2–1.5)	52.0 (37.2–69.9)	41.0 (26.6–60.6)	16.6 (7.9–32.4)	21.4 (12.8–35.0)
Caribbean	Rate: Cumulative change (%)	-54.7 (-62.3 to -45.8)	-45.4 (-55.8 to -34.0)	-35.3 (-54.5 to -12.4)	-54.6 (-68.2 to -38.4)	-78.1 (-85.3 to -67.9)	-9.1 (-40.6 to 17.9)	-75.7 (-94.1 to -40.8)	-3.6 (-32.1 to 36.8)	-27.7 (-57.6 to 16.6)	-73.5 (-89.7 to -44.6)	-60.5 (-79.9 to -33.3)
Antigua and Barbuda	Number: 1990	22 (17–27)	6 (5–8)	1 (1–2)	2 (1–3)	1 (1–1)	2 (2–3)	0 (0–0)	0 (0–2)	0 (0–1)	0 (0–1)	0 (0–0)
Antigua and Barbuda	Number: 2015	19 (13–25)	4 (3–7)	1 (0–2)	2 (1–2)	0 (0–0)	3 (2–4)	0 (0–0)	1 (0–2)	1 (0–1)	0 (0–0)	0 (0–0)
Antigua and Barbuda	Number: Cumulative change (%)	-13.8 (-41.0 to 20.7)	-27.9 (-53.6 to 11.5)	-3.2 (-41.5 to 50.8)	-15.7 (-42.8 to 18.5)	-56.2 (-70.6 to -38.9)	19.7 (-18.4 to 68.3)	nan (nan–nan)	21.8 (-24.6 to 90.2)	45.4 (-13.0 to 133.0)	-59.9 (-75.1 to -40.5)	-53.0 (-67.9 to -34.1)
Antigua and Barbuda	Rate: 1990	387.7 (307.2–481.1)	118.5 (87.9–151.2)	26.9 (19.0–35.7)	43.2 (34.0–54.6)	24.8 (19.5–31.4)	51.2 (39.3–64.5)	0.0 (0.0–0.0)	16.6 (8.9–35.8)	15.3 (10.9–20.6)	13.6 (10.4–17.7)	7.1 (5.6–9.0)
Antigua and Barbuda	Rate: 2015	264.1 (192.3–358.8)	67.1 (44.4–98.7)	20.2 (13.0–30.1)	28.8 (20.8–37.8)	8.6 (6.1–11.2)	48.3 (34.4–65.4)	0.0 (0.0–0.0)	15.5 (8.0–31.6)	17.3 (11.3–25.4)	4.3 (2.9–5.9)	2.6 (1.9–3.5)
Antigua and Barbuda	Rate: Cumulative change (%)	-30.8 (-52.7 to -3.1)	-42.3 (-62.9 to -10.8)	-22.5 (-53.2 to 20.7)	-32.2 (-54.1 to -4.6)	-64.8 (-76.3 to -50.8)	-4.1 (-34.6 to 35.0)	nan (nan–nan)	-2.6 (-39.7 to 52.1)	16.4 (-30.4 to 86.6)	-67.7 (-79.9 to -52.1)	-62.2 (-74.2 to -47.0)
The Bahamas	Number: 1990	199 (144–261)	53 (38–71)	26 (17–38)	20 (13–29)	4 (3–7)	19 (12–27)	0 (0–0)	15 (9–22)	19 (11–29)	1 (1–2)	2 (1–3)
The Bahamas	Number: 2015	94 (57–147)	21 (11–33)	13 (7–21)	8 (5–13)	0 (0–1)	14 (8–22)	0 (0–0)	9 (5–16)	10 (5–17)	0 (0–0)	0 (0–1)
The Bahamas	Number: Cumulative change (%)	-51.2 (-73.4 to -18.3)	-59.5 (-78.8 to -30.6)	-48.6 (-75.7 to -7.3)	-56.0 (-76.5 to -26.2)	-78.3 (-88.9 to -62.5)	-20.1 (-59.3 to 37.0)	nan (nan–nan)	-34.3 (-66.4 to 13.5)	-40.6 (-73.0 to 7.4)	-78.3 (-90.3 to -58.6)	-67.6 (-84.2 to -38.3)
The Bahamas	Rate: 1990	677.5 (491.5–888.1)	182.3 (129.3–243.0)	90.9 (60.0–128.9)	69.2 (45.5–99.9)	16.8 (10.5–25.6)	64.8 (41.5–94.2)	0.0 (0.0–0.0)	51.3 (31.7–77.1)	66.2 (40.6–100.8)	6.6 (3.8–10.1)	8.7 (5.3–13.0)
The Bahamas	Rate: 2015	335.1 (205.1–523.8)	75.1 (42.3–120.8)	46.5 (25.9–76.8)	30.1 (18.9–46.6)	3.5 (2.2–5.5)	50.8 (31.5–79.2)	0.0 (0.0–0.0)	33.8 (18.2–58.5)	38.6 (21.1–63.4)	1.4 (0.8–2.3)	2.7 (1.7–4.2)
The Bahamas	Rate: Cumulative change (%)	-48.9 (-72.1 to -14.5)	-57.5 (-77.7 to -27.1)	-46.0 (-74.5 to -2.7)	-54.2 (-75.5 to -23.2)	-77.4 (-88.5 to -61.0)	-16.5 (-57.5 to 43.0)	nan (nan–nan)	-31.1 (-64.8 to 19.0)	-37.7 (-71.7 to 12.7)	-77.5 (-89.9 to -57.1)	-66.4 (-83.6 to -35.9)
Barbados	Number: 1990	110 (77–151)	25 (17–34)	18 (12–25)	11 (7–16)	3 (1–4)	18 (12–25)	0 (0–0)	8 (4–16)	5 (3–7)	2 (1–3)	1 (1–2)
Barbados	Number: 2015	55 (34–86)	10 (5–17)	7 (4–12)	4 (3–7)	0 (0–0)	13 (7–20)	0 (0–0)	6 (3–12)	3 (1–6)	0 (0–0)	0 (0–0)
Barbados	Number: Cumulative change (%)	-47.7 (-71.4 to -12.7)	-57.6 (-78.4 to -28.2)	-55.4 (-77.8 to -21.8)	-54.8 (-76.2 to -22.6)	-78.0 (-88.8 to -61.6)	-24.7 (-60.3 to 28.6)	nan (nan–nan)	-23.1 (-59.0 to 33.3)	-25.8 (-64.2 to 38.6)	-80.5 (-90.6 to -65.8)	-74.9 (-86.4 to -57.2)
Barbados	Rate: 1990	546.7 (383.9–748.2)	128.5 (89.2–173.6)	91.4 (61.8–126.1)	55.0 (36.5–80.3)	14.8 (9.5–22.7)	90.5 (61.6–127.4)	0.0 (0.0–0.0)	43.3 (24.5–79.6)	26.0 (17.0–36.8)	11.2 (7.0–17.3)	8.5 (5.7–12.7)
Barbados	Rate: 2015	334.5 (205.3–521.6)	63.9 (34.9–104.9)	47.6 (26.3–77.3)	28.6 (18.1–43.8)	3.7 (2.3–5.7)	79.2 (47.8–123.4)	0.0 (0.0–0.0)	38.3 (19.6–72.9)	22.2 (11.9–36.6)	2.5 (1.4–3.9)	2.5 (1.6–3.7)
Barbados	Rate: Cumulative change (%)	-36.2 (-65.1 to 6.6)	-48.3 (-73.7 to -12.4)	-45.6 (-72.9 to -4.7)	-44.9 (-71.0 to -5.7)	-73.1 (-86.4 to -53.2)	-8.2 (-51.5 to 56.9)	nan (nan–nan)	-6.3 (-50.1 to 62.4)	-9.5 (-56.4 to 69.1)	-76.3 (-88.6 to -58.3)	-69.4 (-83.4 to -47.8)
Belize	Number: 1990	258 (224–297)	57 (46–66)	22 (17–28)	38 (31–46)	21 (16–26)	21 (15–29)	0 (0–0)	14 (9–23)	6 (5–8)	9 (6–12)	4 (3–5)
Belize	Number: 2015	135 (91–194)	24 (15–38)	15 (8–23)	15 (10–22)	4 (3–7)	19 (12–29)	0 (0–0)	12 (7–20)	6 (3–10)	2 (1–3)	1 (0–2)
Belize	Number: Cumulative change (%)	-47.3 (-66.0 to -23.0)	-56.3 (-73.7 to -32.3)	-31.8 (-62.6 to 7.0)	-59.3 (-73.6 to -38.5)	-76.5 (-85.6 to -63.1)	-1.2 (-49.1 to 56.1)	-90.3 (-95.9 to -81.1)	-5.5 (-43.3 to 42.5)	-1.9 (-46.4 to 59.7)	-76.7 (-87.1 to -62.3)	-68.2 (-82.1 to -46.4)
Belize	Rate: 1990	810.3 (703.6–929.9)	177.7 (145.0–208.3)	71.0 (55.1–89.0)	122.2 (99.8–146.3)	67.4 (52.3–84.7)	65.8 (49.8–91.8)	0.3 (0.2–0.5)	43.6 (30.6–72.5)	21.3 (15.7–27.7)	29.5 (20.9–38.6)	14.8 (11.2–18.8)
Belize	Rate: 2015	343.1 (233.1–493.8)	62.6 (38.2–96.5)	38.6 (22.4–59.2)	39.6 (27.2–57.0)	12.5 (8.5–18.3)	50.6 (32.9–75.7)	0.0 (0.0–0.0)	32.4 (19.2–51.9)	16.5 (9.4–26.0)	5.4 (3.3–8.4)	3.7 (2.4–5.6)
Belize	Rate: Cumulative change (%)	-57.3 (-72.5 to -37.6)	-64.5 (-78.6 to -45.0)	-44.7 (-69.6 to -13.1)	-67.0 (-78.6 to -50.2)	-81.0 (-88.3 to -70.2)	-20.0 (-58.8 to 26.5)	-92.1 (-96.7 to -84.7)	-23.4 (-54.0 to 15.5)	-20.4 (-56.5 to 29.5)	-81.2 (-89.6 to -69.4)	-74.3 (-85.5 to -56.6)
Bermuda	Number: 1990	9 (7–11)	2 (1–2)	1 (1–1)	0 (0–0)	0 (0–0)	1 (0–2)	0 (0–0)	0 (0–0)	1 (0–1)	0 (0–0)	0 (0–0)
Bermuda	Number: 2015	3 (2–4)	0 (0–0)	0 (0–0)	0 (0–0)	0 (0–0)	0 (0–1)	0 (0–0)	0 (0–0)	0 (0–0)	0 (0–0)	0 (0–0)
Bermuda	Number: Cumulative change (%)	-64.1 (-76.9 to -48.1)	-85.0 (-91.5 to -76.4)	-63.9 (-79.4 to -41.5)	-73.4 (-82.7 to -61.1)	-86.8 (-91.9 to -80.2)	-24.4 (-54.9 to 12.5)	nan (nan–nan)	-59.0 (-74.8 to -38.2)	-50.6 (-70.8 to -23.5)	-88.2 (-93.2 to -81.0)	-84.6 (-90.0 to -77.7)
Bermuda	Rate: 1990	229.4 (185.2–280.6)	51.7 (39.4–66.8)	33.8 (24.9–44.1)	19.2 (14.8–23.8)	4.9 (3.5–6.3)	32.6 (23.8–54.7)	0.0 (0.0–0.0)	6.4 (4.1–11.6)	30.6 (22.2–40.4)	1.3 (0.9–1.8)	3.5 (2.7–4.3)
Bermuda	Rate: 2015	88.5 (62.1–124.2)	8.2 (5.0–12.2)	12.8 (7.9–19.5)	5.5 (3.7–7.7)	0.7 (0.5–1.0)	25.9 (17.3–37.6)	0.0 (0.0–0.0)	2.8 (1.5–4.7)	15.9 (10.0–23.4)	0.2 (0.1–0.2)	0.6 (0.4–0.8)
Bermuda	Rate: Cumulative change (%)	-60.9 (-74.9 to -43.5)	-83.8 (-90.9 to -74.6)	-61.1 (-77.9 to -37.0)	-70.8 (-80.9 to -57.3)	-85.4 (-91.0 to -78.2)	-17.4 (-50.7 to 22.9)	nan (nan–nan)	-55.8 (-72.8 to -33.5)	-46.8 (-68.6 to -17.5)	-86.9 (-92.4 to -78.9)	-83.0 (-89.0 to -75.4)
Cuba	Number: 1990	2,429 (2,400–2,457)	371 (306–440)	329 (275–389)	276 (250–307)	119 (103–136)	504 (447–574)	0 (0–0)	83 (22–135)	73 (57–92)	8 (6–10)	159 (142–175)
Cuba	Number: 2015	704 (674–738)	77 (64–92)	66 (55–79)	66 (57–76)	13 (11–16)	200 (175–252)	0 (0–0)	31 (10–46)	84 (71–101)	1 (1–2)	13 (11–15)
Cuba	Number: Cumulative change (%)	-71.0 (-72.2 to -69.6)	-79.0 (-83.1 to -74.3)	-79.5 (-84.0 to -74.3)	-75.8 (-79.8 to -71.4)	-88.5 (-91.0 to -85.6)	-60.1 (-66.6 to -51.3)	nan (nan–nan)	-60.2 (-71.7 to -47.7)	17.3 (-12.6 to 52.0)	-76.9 (-83.6 to -68.3)	-91.4 (-92.7 to -89.8)
Cuba	Rate: 1990	280.7 (277.3–283.9)	43.4 (35.8–51.5)	38.5 (32.2–45.5)	31.8 (28.8–35.4)	13.7 (11.8–15.7)	58.4 (51.7–66.3)	0.0 (0.0–0.0)	9.8 (2.6–15.8)	8.6 (6.7–10.8)	0.9 (0.7–1.2)	18.2 (16.3–20.1)
Cuba	Rate: 2015	124.3 (118.9–130.3)	13.9 (11.6–16.5)	12.0 (9.9–14.3)	11.7 (10.1–13.5)	2.4 (2.0–2.9)	35.4 (31.0–44.6)	0.0 (0.0–0.0)	5.6 (1.8–8.3)	15.2 (12.8–18.1)	0.3 (0.2–0.4)	2.4 (2.0–2.7)
Cuba	Rate: Cumulative change (%)	-55.7 (-57.6 to -53.6)	-67.8 (-74.1 to -60.5)	-68.5 (-75.4 to -60.5)	-63.1 (-69.2 to -56.5)	-82.4 (-86.2 to -78.0)	-39.1 (-49.0 to -25.7)	nan (nan–nan)	-38.9 (-56.6 to -19.6)	80.2 (34.3–133.4)	-65.1 (-75.2 to -51.9)	-86.9 (-89.0 to -84.5)
Dominica	Number: 1990	41 (32–52)	8 (5–11)	5 (3–6)	3 (2–5)	3 (2–4)	5 (3–6)	0 (0–0)	3 (2–7)	1 (0–1)	1 (0–1)	0 (0–0)
Dominica	Number: 2015	30 (20–42)	5 (3–7)	4 (2–5)	3 (1–4)	1 (0–1)	4 (2–6)	0 (0–0)	3 (2–6)	1 (0–1)	0 (0–0)	0 (0–0)
Dominica	Number: Cumulative change (%)	-26.5 (-52.8 to 11.0)	-30.9 (-61.0 to 12.8)	-15.8 (-50.3 to 31.6)	-22.2 (-55.1 to 25.0)	-63.7 (-79.5 to -40.2)	-12.0 (-49.0 to 37.6)	nan (nan–nan)	7.8 (-39.0 to 71.6)	2.3 (-45.3 to 74.4)	-65.3 (-82.2 to -38.0)	-46.1 (-70.3 to -8.4)
Dominica	Rate: 1990	518.5 (403.9–648.3)	106.6 (69.9–146.0)	63.5 (44.7–85.2)	49.0 (36.6–63.6)	42.0 (31.1–55.4)	63.9 (47.0–84.6)	0.0 (0.0–0.0)	46.1 (26.0–91.5)	17.3 (10.9–24.9)	15.5 (10.5–21.7)	7.5 (5.5–10.2)
Dominica	Rate: 2015	546.4 (378.2–773.8)	102.3 (66.2–146.1)	75.5 (48.1–104.9)	54.3 (35.3–80.6)	21.7 (13.5–33.5)	80.5 (46.4–122.1)	0.0 (0.0–0.0)	67.1 (40.3–111.1)	24.5 (15.1–36.5)	7.5 (4.5–12.3)	5.8 (3.6–9.0)
Dominica	Rate: Cumulative change (%)	7.6 (-30.8 to 62.6)	1.0 (-42.9 to 64.8)	23.1 (-27.4 to 92.5)	14.0 (-34.2 to 83.2)	-46.8 (-70.0 to -12.3)	28.7 (-25.4 to 101.2)	nan (nan–nan)	57.6 (-10.9 to 150.7)	49.7 (-20.1 to 155.1)	-49.1 (-74.0 to -8.9)	-20.9 (-56.4 to 34.5)
Dominican Republic	Number: 1990	12,710 (12,060–13,392)	2,374 (2,043–2,709)	1,026 (821–1,256)	1,108 (961–1,264)	2,430 (2,143–2,713)	916 (522–1,231)	0 (0–1)	887 (677–1,286)	491 (372–627)	767 (526–938)	445 (341–526)
Dominican Republic	Number: 2015	5,406 (4,578–6,403)	1,202 (953–1,483)	446 (326–602)	536 (421–680)	212 (155–285)	839 (539–1,106)	0 (0–0)	841 (625–1,117)	220 (159–303)	73 (49–113)	129 (97–174)
Dominican Republic	Number: Cumulative change (%)	-57.4 (-64.5 to -49.6)	-49.1 (-60.3 to -37.1)	-56.0 (-69.1 to -39.8)	-51.3 (-62.9 to -37.0)	-91.2 (-93.7 to -88.0)	-5.1 (-45.3 to 25.7)	-78.6 (-88.0 to -65.2)	-3.1 (-29.4 to 31.3)	-54.3 (-69.4 to -35.1)	-90.1 (-94.1 to -81.5)	-70.1 (-79.8 to -54.7)
Dominican Republic	Rate: 1990	1,250.7 (1,186.6–1,317.9)	231.2 (199.1–263.8)	99.9 (80.0–122.3)	109.5 (94.9–125.1)	240.4 (211.9–268.3)	90.1 (51.4–121.2)	0.1 (0.1–0.1)	86.7 (66.1–125.6)	47.9 (36.3–61.1)	76.3 (52.3–93.2)	44.0 (33.8–52.2)
Dominican Republic	Rate: 2015	517.0 (438.0–612.5)	115.6 (91.7–142.6)	42.9 (31.3–57.9)	51.1 (40.1–64.9)	20.3 (14.7–27.1)	80.2 (51.5–105.8)	0.0 (0.0–0.0)	80.9 (60.1–107.4)	21.2 (15.3–29.1)	7.0 (4.7–10.8)	12.3 (9.3–16.6)
Dominican Republic	Rate: Cumulative change (%)	-58.6 (-65.5 to -51.0)	-49.8 (-60.8 to -37.9)	-56.6 (-69.5 to -40.6)	-53.0 (-64.3 to -39.3)	-91.5 (-93.9 to -88.5)	-7.6 (-46.8 to 22.3)	-79.7 (-88.6 to -67.1)	-4.5 (-30.4 to 29.4)	-54.9 (-69.8 to -35.9)	-90.5 (-94.4 to -82.4)	-71.3 (-80.6 to -56.4)
Grenada	Number: 1990	78 (51–112)	16 (10–23)	9 (6–14)	12 (7–18)	3 (2–6)	9 (5–14)	0 (0–0)	4 (2–8)	2 (1–4)	2 (1–3)	1 (0–2)
Grenada	Number: 2015	32 (19–51)	5 (2–9)	4 (2–7)	4 (2–7)	0 (0–1)	5 (3–8)	0 (0–0)	2 (1–5)	1 (0–2)	0 (0–0)	0 (0–0)
Grenada	Number: Cumulative change (%)	-56.4 (-78.2 to -24.6)	-62.0 (-82.5 to -30.2)	-51.9 (-78.7 to -11.9)	-59.5 (-80.3 to -31.6)	-81.2 (-90.9 to -65.5)	-34.4 (-69.2 to 16.0)	nan (nan–nan)	-32.2 (-69.2 to 24.8)	-40.5 (-75.3 to 16.3)	-81.1 (-91.9 to -64.0)	-72.4 (-87.3 to -50.0)
Grenada	Rate: 1990	566.1 (372.7–813.0)	119.6 (74.4–170.5)	72.7 (44.9–105.1)	86.2 (54.5–129.8)	27.8 (17.1–43.5)	65.9 (40.0–102.1)	0.0 (0.0–0.0)	35.0 (19.4–60.9)	22.0 (12.3–33.6)	14.6 (8.3–23.0)	9.3 (5.6–14.3)
Grenada	Rate: 2015	327.4 (195.7–521.6)	58.8 (30.4–100.6)	45.1 (23.6–75.5)	46.1 (28.4–71.7)	6.8 (4.0–11.0)	55.8 (32.9–88.4)	0.0 (0.0–0.0)	30.1 (14.9–52.0)	16.6 (8.3–29.0)	3.6 (2.1–5.8)	3.3 (2.0–5.2)
Grenada	Rate: Cumulative change (%)	-38.9 (-69.5 to 5.6)	-47.8 (-76.0 to -4.1)	-33.9 (-70.7 to 21.1)	-42.7 (-72.1 to -3.3)	-73.3 (-87.1 to -51.2)	-8.5 (-57.0 to 62.0)	nan (nan–nan)	-6.9 (-57.8 to 71.3)	-18.1 (-66.1 to 60.0)	-72.9 (-88.3 to -48.4)	-60.7 (-81.8 to -28.7)
Guyana	Number: 1990	1,255 (1,183–1,328)	279 (212–322)	103 (81–124)	126 (109–144)	215 (187–244)	84 (70–107)	6 (3–9)	71 (45–148)	44 (32–57)	88 (66–106)	12 (9–14)
Guyana	Number: 2015	411 (336–503)	82 (62–101)	51 (37–65)	33 (25–44)	28 (20–39)	49 (37–62)	0 (0–1)	45 (31–70)	29 (20–40)	10 (7–15)	2 (1–3)
Guyana	Number: Cumulative change (%)	-67.2 (-73.8 to -59.8)	-70.3 (-77.4 to -62.0)	-49.8 (-64.2 to -32.5)	-73.2 (-80.6 to -64.6)	-86.7 (-90.8 to -81.4)	-40.4 (-60.9 to -19.1)	-88.3 (-96.1 to -74.8)	-30.4 (-57.4 to 3.6)	-32.5 (-56.3 to -2.8)	-87.4 (-92.2 to -80.8)	-76.8 (-84.6 to -65.9)
Guyana	Rate: 1990	1,097.7 (1,035.0–1,161.7)	244.1 (185.5–282.3)	90.1 (71.6–108.8)	110.7 (95.6–126.6)	188.5 (164.2–213.9)	74.3 (61.8–94.3)	5.5 (3.4–8.0)	62.8 (39.7–129.8)	39.1 (28.5–50.4)	77.5 (58.1–93.4)	10.5 (8.7–12.4)
Guyana	Rate: 2015	578.0 (472.6–707.8)	114.9 (87.8–141.8)	71.5 (52.7–92.1)	47.4 (35.7–62.8)	40.1 (28.5–55.6)	69.8 (52.8–88.5)	1.0 (0.4–2.0)	64.1 (44.5–99.1)	41.2 (29.1–56.6)	15.5 (10.2–22.3)	3.9 (2.8–5.3)
Guyana	Rate: Cumulative change (%)	-47.3 (-57.9 to -35.5)	-52.5 (-63.8 to -39.3)	-19.7 (-42.7 to 8.1)	-56.9 (-68.8 to -43.0)	-78.6 (-85.2 to -70.0)	-4.2 (-37.2 to 29.9)	-80.9 (-93.6 to -58.9)	11.1 (-32.1 to 65.3)	8.1 (-30.1 to 55.6)	-79.6 (-87.3 to -69.0)	-62.5 (-75.1 to -45.1)
Haiti	Number: 1990	39,168 (36,976–41,508)	2,645 (1,971–3,415)	2,228 (1,433–3,071)	6,024 (4,827–7,333)	10,805 (8,902–12,872)	1,783 (1,344–2,480)	123 (42–246)	1,042 (531–1,743)	1,643 (835–2,673)	1,747 (868–3,180)	1,646 (980–2,661)
Haiti	Number: 2015	15,368 (11,664–20,111)	1,418 (919–2,045)	1,504 (940–2,199)	2,384 (1,584–3,372)	2,370 (1,522–3,518)	1,545 (1,043–2,134)	23 (4–52)	788 (389–1,299)	1,002 (466–1,703)	499 (182–1,068)	620 (312–1,121)
Haiti	Number: Cumulative change (%)	-60.7 (-70.4 to -48.6)	-45.1 (-66.2 to -17.4)	-29.3 (-60.6 to 11.2)	-59.8 (-74.9 to -40.7)	-77.8 (-86.1 to -66.3)	-9.2 (-53.2 to 38.7)	-76.8 (-95.4 to -40.8)	-17.0 (-64.0 to 53.9)	-32.7 (-72.2 to 32.6)	-66.6 (-91.2 to -12.3)	-59.2 (-83.1 to -20.4)
Haiti	Rate: 1990	3,262.1 (3,076.1–3,456.3)	210.5 (157.1–271.4)	177.4 (114.6–244.2)	504.4 (404.7–615.1)	907.0 (746.8–1,083.8)	146.6 (110.0–204.2)	10.6 (3.6–21.0)	83.3 (42.4–139.5)	131.4 (66.6–213.6)	148.3 (73.5–269.1)	139.0 (82.3–225.5)

© 2017 Global Burden of Disease Child and Adolescent Health Collaboration. JAMA Pediatrics.

eTable 2. Number of Deaths, Death Rates (per 100,000 population), and Cumulative Percent Change with 95% Uncertainty Intervals (UI) for the Top 10 Global Causes of Death in 195 Countries and Territories, Aged Under 5 Years, Both Sexes, 1990 and 2015 *Note: best viewed by enlarging in browser.*

Location	Measure	All causes	Neonatal preterm birth complications	Neonatal encephalopathy due to birth asphyxia and trauma	Lower respiratory infections	Diarrheal diseases	Congenital anomalies	Malaria	Neonatal sepsis and other neonatal infections	Other neonatal disorders	Protein-energy malnutrition	Meningitis
Haiti	Rate: 2015	1,224.4 (928.6–1,602.4)	112.3 (72.9–161.9)	119.1 (74.6–174.1)	190.3 (126.4–269.3)	189.4 (121.6–281.1)	123.0 (83.1–170.2)	1.9 (0.4–4.2)	62.5 (30.8–103.2)	79.4 (36.9–135.0)	39.9 (14.6–85.5)	49.6 (24.9–89.6)
Haiti	Rate: Cumulative change (%)	-62.4 (-71.6 to -50.8)	-45.4 (-66.3 to -17.9)	-29.7 (-60.8 to 10.5)	-61.6 (-76.2 to -43.4)	-78.9 (-86.8 to -67.6)	-11.9 (-55.0 to 34.8)	-78.3 (-95.7 to -44.4)	-17.7 (-64.4 to 52.9)	-33.4 (-72.4 to 30.5)	-68.5 (-91.7 to -17.2)	-61.3 (-84.0 to -24.3)
Jamaica	Number: 1990	1,696 (1,352–2,098)	482 (378–599)	127 (91–172)	146 (110–193)	178 (132–239)	201 (138–267)	0 (0–0)	81 (47–163)	54 (39–73)	98 (67–136)	31 (23–40)
Jamaica	Number: 2015	875 (599–1,254)	258 (159–380)	91 (55–138)	56 (39–79)	16 (10–24)	161 (96–246)	0 (0–0)	77 (44–133)	32 (20–49)	17 (10–28)	11 (6–19)
Jamaica	Number: Cumulative change (%)	-47.4 (-67.1 to -22.8)	-45.6 (-68.1 to -17.4)	-25.3 (-60.3 to 20.7)	-60.7 (-75.3 to -39.2)	-90.5 (-94.2 to -85.1)	-17.5 (-51.9 to 27.9)	nan (nan–nan)	3.2 (-41.9 to 69.9)	-37.3 (-65.9 to 1.6)	-81.2 (-90.0 to -65.0)	-63.4 (-80.4 to -32.8)
Jamaica	Rate: 1990	568.1 (453.5–702.2)	163.1 (127.9–202.8)	43.0 (31.0–58.2)	49.0 (36.9–64.4)	59.4 (43.9–79.2)	67.3 (46.5–89.5)	0.0 (0.0–0.0)	27.5 (16.1–55.4)	18.3 (13.2–24.9)	32.7 (22.2–45.2)	10.4 (7.8–13.6)
Jamaica	Rate: 2015	373.6 (255.4–535.1)	111.2 (68.7–163.6)	39.4 (23.8–59.5)	23.8 (16.6–33.5)	6.9 (4.6–10.2)	68.7 (40.9–104.9)	0.0 (0.0–0.0)	33.5 (19.0–57.6)	14.1 (8.7–21.1)	7.4 (4.6–12.0)	4.7 (2.8–8.0)
Jamaica	Rate: Cumulative change (%)	-33.1 (-58.1 to -1.7)	-30.8 (-59.5 to 5.0)	-5.0 (-49.5 to 53.4)	-50.1 (-68.7 to -22.7)	-88.0 (-92.7 to -81.0)	4.9 (-38.9 to 62.5)	nan (nan–nan)	31.2 (-26.2 to 116.0)	-20.3 (-56.7 to 29.1)	-76.1 (-87.3 to -55.3)	-53.5 (-75.0 to -14.4)
Puerto Rico	Number: 1990	980 (958–1,001)	377 (349–410)	60 (50–72)	74 (66–82)	5 (4–6)	170 (145–191)	0 (0–0)	27 (9–41)	65 (53–80)	1 (1–2)	14 (13–16)
Puerto Rico	Number: 2015	305 (276–338)	78 (66–93)	17 (13–22)	16 (13–19)	4 (3–4)	73 (62–84)	0 (0–0)	21 (5–34)	36 (28–45)	0 (0–0)	2 (1–2)
Puerto Rico	Number: Cumulative change (%)	-68.9 (-71.8 to -65.6)	-79.1 (-82.3 to -75.5)	-70.8 (-78.1 to -62.4)	-77.8 (-82.1 to -72.1)	-20.1 (-39.2 to 4.4)	-56.8 (-65.3 to -45.1)	nan (nan–nan)	-25.0 (-47.1 to 5.4)	-43.6 (-58.7 to -26.9)	-71.1 (-81.1 to -58.4)	-84.4 (-87.3 to -81.1)
Puerto Rico	Rate: 1990	317.1 (310.1–324.0)	122.9 (113.7–133.6)	19.7 (16.3–23.5)	24.0 (21.4–26.5)	1.6 (1.4–1.9)	55.2 (47.0–61.8)	0.0 (0.0–0.0)	9.0 (3.0–13.4)	21.4 (17.3–26.2)	0.5 (0.4–0.7)	4.7 (4.2–5.2)
Puerto Rico	Rate: 2015	144.0 (130.4–159.9)	37.6 (31.9–44.5)	8.3 (6.5–10.6)	7.7 (6.4–9.3)	1.9 (1.5–2.3)	34.4 (29.5–39.9)	0.0 (0.0–0.0)	10.3 (2.6–16.4)	17.4 (13.8–21.6)	0.2 (0.2–0.3)	1.1 (0.9–1.2)
Puerto Rico	Rate: Cumulative change (%)	-54.6 (-58.9 to -49.8)	-69.4 (-74.1 to -64.1)	-57.3 (-67.9 to -44.8)	-67.7 (-74.1 to -59.5)	16.2 (-11.6 to 51.8)	-37.0 (-49.5 to -19.9)	nan (nan–nan)	9.9 (-22.5 to 54.5)	-17.3 (-39.4 to 7.1)	-58.2 (-72.7 to -39.7)	-77.4 (-81.6 to -72.6)
Saint Lucia	Number: 1990	122 (83–171)	31 (20–42)	11 (7–15)	15 (10–23)	9 (5–14)	11 (7–16)	0 (0–0)	7 (3–14)	4 (3–6)	4 (2–6)	2 (1–3)
Saint Lucia	Number: 2015	49 (28–80)	10 (5–16)	5 (2–9)	5 (3–8)	1 (0–1)	8 (4–14)	0 (0–0)	4 (1–9)	2 (1–4)	0 (0–1)	0 (0–0)
Saint Lucia	Number: Cumulative change (%)	-58.2 (-78.0 to -26.8)	-66.2 (-83.4 to -40.8)	-49.9 (-76.0 to -10.5)	-65.5 (-83.0 to -38.3)	-87.6 (-93.8 to -77.4)	-16.1 (-58.8 to 56.9)	nan (nan–nan)	-38.3 (-69.8 to 8.8)	-37.2 (-71.4 to 17.2)	-82.0 (-91.4 to -66.2)	-79.8 (-89.4 to -64.3)
Saint Lucia	Rate: 1990	648.2 (443.9–903.4)	167.2 (112.1–225.8)	60.4 (40.0–85.0)	82.0 (53.6–122.3)	49.2 (31.1–76.1)	59.4 (38.5–85.6)	0.0 (0.0–0.0)	39.5 (19.6–79.4)	24.9 (16.3–35.0)	22.3 (13.6–34.7)	13.6 (8.7–20.5)
Saint Lucia	Rate: 2015	363.1 (209.1–593.9)	76.6 (39.8–126.1)	40.8 (20.5–67.8)	37.1 (22.2–59.9)	7.9 (4.8–13.1)	65.6 (36.6–108.6)	0.0 (0.0–0.0)	32.3 (13.7–69.2)	20.9 (10.6–35.3)	5.2 (3.1–8.8)	3.6 (2.2–5.7)
Saint Lucia	Rate: Cumulative change (%)	-41.5 (-69.2 to 2.4)	-52.6 (-76.8 to -17.1)	-29.7 (-66.3 to 25.4)	-51.9 (-76.4 to -13.7)	-82.7 (-91.4 to -68.5)	17.2 (-42.4 to 119.7)	nan (nan–nan)	-13.7 (-57.7 to 52.2)	-12.0 (-60.0 to 64.2)	-75.0 (-88.1 to -52.9)	-71.8 (-85.3 to -50.3)
Saint Vincent and the Grenadines	Number: 1990	106 (79–136)	32 (23–41)	13 (9–17)	11 (7–14)	9 (6–13)	9 (7–13)	0 (0–0)	3 (0–13)	0 (0–0)	6 (4–9)	2 (1–2)
Saint Vincent and the Grenadines	Number: 2015	38 (25–55)	9 (6–14)	3 (1–4)	3 (2–5)	1 (0–2)	5 (3–8)	0 (0–0)	4 (2–7)	2 (1–3)	0 (0–1)	0 (0–0)
Saint Vincent and the Grenadines	Number: Cumulative change (%)	-63.1 (-77.3 to -43.6)	-69.1 (-81.3 to -53.4)	-74.7 (-85.5 to -60.7)	-68.7 (-82.7 to -47.5)	-84.8 (-91.5 to -74.3)	-41.1 (-65.8 to -5.7)	nan (nan–nan)	1,037.1 (-57.6 to 2,766.4)	746.9 (368.8–1,262.3)	-85.1 (-92.1 to -74.4)	-82.3 (-90.0 to -71.2)
Saint Vincent and the Grenadines	Rate: 1990	825.0 (617.7–1,055.1)	254.5 (184.1–326.6)	102.1 (72.7–133.2)	85.7 (59.0–115.5)	73.8 (50.6–103.1)	76.2 (54.9–101.4)	0.0 (0.0–0.0)	30.2 (1.3–104.6)	2.3 (1.6–3.1)	49.0 (31.9–70.4)	15.7 (11.0–21.6)
Saint Vincent and the Grenadines	Rate: 2015	452.4 (306.6–652.1)	117.6 (74.8–171.6)	38.4 (23.5–55.9)	39.0 (25.7–58.9)	16.2 (10.6–25.5)	66.1 (41.9–98.1)	0.0 (0.0–0.0)	56.5 (33.6–95.7)	28.5 (17.2–42.5)	10.4 (6.6–16.2)	4.0 (2.7–6.0)
Saint Vincent and the Grenadines	Rate: Cumulative change (%)	-43.7 (-65.4 to -13.8)	-52.7 (-71.3 to -28.5)	-61.3 (-77.8 to -39.8)	-52.5 (-73.8 to -20.1)	-77.0 (-87.1 to -61.0)	-10.3 (-48.0 to 43.8)	nan (nan–nan)	1,639.5 (-35.0 to 4,283.8)	1,196.0 (617.5–1,984.1)	-77.5 (-88.1 to -61.3)	-73.1 (-84.8 to -56.2)
Suriname	Number: 1990	573 (523–628)	137 (112–158)	48 (37–61)	60 (51–70)	73 (61–85)	54 (39–67)	1 (1–2)	34 (23–65)	13 (9–17)	17 (12–21)	10 (8–13)
Suriname	Number: 2015	303 (252–366)	74 (59–91)	34 (26–44)	26 (19–33)	12 (8–16)	45 (32–60)	0 (0–0)	31 (22–50)	9 (6–11)	3 (2–4)	3 (2–4)
Suriname	Number: Cumulative change (%)	-46.9 (-56.5 to -36.1)	-45.5 (-57.4 to -32.5)	-27.5 (-48.1 to -1.4)	-55.9 (-67.4 to -41.2)	-83.4 (-88.4 to -77.1)	-14.2 (-40.8 to 14.5)	-91.0 (-96.4 to -81.8)	-5.1 (-33.1 to 32.0)	-29.2 (-52.0 to -2.0)	-79.9 (-87.8 to -68.7)	-68.1 (-79.5 to -49.5)
Suriname	Rate: 1990	1,070.1 (977.7–1,174.5)	254.3 (209.0–293.3)	90.3 (68.7–113.2)	112.7 (95.9–132.0)	137.1 (115.8–160.5)	100.8 (73.3–126.8)	3.2 (1.9–4.9)	64.6 (43.2–120.9)	24.5 (18.5–31.6)	32.0 (23.7–41.0)	20.4 (16.4–24.7)
Suriname	Rate: 2015	645.3 (536.0–778.7)	158.4 (126.8–195.0)	74.0 (56.2–94.2)	55.7 (42.2–72.0)	25.5 (18.3–35.2)	97.1 (68.1–129.0)	0.3 (0.1–0.5)	67.3 (47.1–108.8)	19.5 (14.3–25.3)	7.0 (4.8–10.3)	7.2 (5.1–10.6)
Suriname	Rate: Cumulative change (%)	-39.6 (-50.5 to -27.3)	-37.3 (-50.9 to -22.3)	-16.7 (-40.3 to 13.4)	-50.2 (-63.2 to -33.6)	-81.3 (-87.0 to -74.2)	-2.4 (-32.7 to 30.3)	-90.0 (-96.0 to -79.7)	9.0 (-23.1 to 51.4)	-18.7 (-44.9 to 12.5)	-77.5 (-86.3 to -64.9)	-64.0 (-77.0 to -43.1)
Trinidad and Tobago	Number: 1990	728 (637–826)	193 (164–226)	119 (93–147)	73 (61–87)	30 (24–36)	113 (94–133)	0 (0–0)	24 (7–38)	37 (27–47)	9 (7–11)	8 (7–10)
Trinidad and Tobago	Number: 2015	367 (245–536)	83 (51–124)	42 (25–64)	23 (15–35)	8 (5–13)	86 (54–127)	0 (0–0)	14 (5–24)	49 (30–72)	1 (1–2)	1 (1–2)
Trinidad and Tobago	Number: Cumulative change (%)	-49.2 (-67.9 to -24.3)	-56.5 (-74.7 to -32.5)	-63.7 (-79.7 to -42.6)	-67.8 (-79.8 to -49.5)	-71.1 (-82.5 to -54.0)	-22.5 (-52.8 to 19.0)	nan (nan–nan)	-36.2 (-64.8 to 1.8)	36.6 (-22.1 to 112.3)	-80.5 (-88.5 to -68.5)	-80.3 (-87.6 to -69.4)
Trinidad and Tobago	Rate: 1990	583.5 (510.4–662.2)	159.0 (134.7–185.6)	97.8 (76.8–120.8)	58.1 (48.2–69.1)	23.7 (19.4–28.7)	90.3 (75.2–106.5)	0.0 (0.0–0.0)	20.4 (6.6–31.7)	30.3 (22.8–39.2)	7.1 (5.4–9.0)	6.8 (5.5–8.1)
Trinidad and Tobago	Rate: 2015	397.2 (264.7–580.7)	91.1 (56.3–136.4)	46.4 (27.9–69.9)	25.1 (16.7–38.0)	9.2 (6.0–14.2)	93.5 (59.0–138.1)	0.0 (0.0–0.0)	16.2 (6.0–27.1)	53.8 (33.1–79.5)	1.9 (1.2–2.9)	1.8 (1.2–2.7)
Trinidad and Tobago	Rate: Cumulative change (%)	-31.4 (-56.8 to 2.2)	-42.1 (-66.4 to -10.2)	-51.7 (-73.0 to -23.6)	-56.2 (-72.6 to -31.3)	-60.6 (-76.2 to -37.4)	4.7 (-36.4 to 60.6)	nan (nan–nan)	-15.2 (-53.2 to 35.3)	81.8 (3.7–182.3)	-73.2 (-84.2 to -56.3)	-72.8 (-82.9 to -57.8)
Virgin Islands, U.S.	Number: 1990	38 (35–41)	10 (8–12)	3 (2–4)	2 (1–2)	0 (0–0)	5 (4–7)	0 (0–0)	1 (0–2)	3 (2–4)	0 (0–0)	0 (0–0)
Virgin Islands, U.S.	Number: 2015	12 (10–14)	2 (2–3)	1 (0–1)	0 (0–0)	0 (0–0)	2 (2–3)	0 (0–0)	0 (0–1)	0 (0–1)	0 (0–0)	0 (0–0)
Virgin Islands, U.S.	Number: Cumulative change (%)	-67.2 (-72.6 to -60.6)	-76.0 (-81.8 to -69.5)	-64.2 (-75.5 to -50.0)	-73.4 (-80.0 to -64.9)	-86.5 (-90.8 to -81.1)	-49.8 (-63.3 to -34.8)	nan (nan–nan)	-21.6 (-57.4 to 32.6)	-68.9 (-78.8 to -56.8)	-82.5 (-89.6 to -73.7)	-78.1 (-85.5 to -67.3)
Virgin Islands, U.S.	Rate: 1990	326.7 (303.0–353.0)	92.1 (77.2–107.6)	31.2 (24.3–38.9)	20.1 (16.8–23.6)	5.9 (4.7–7.3)	49.9 (41.3–62.4)	0.0 (0.0–0.0)	9.9 (5.6–19.8)	27.3 (21.2–34.6)	4.8 (3.4–6.3)	1.4 (1.1–1.8)
Virgin Islands, U.S.	Rate: 2015	178.3 (151.8–210.0)	36.8 (29.2–45.3)	18.4 (14.2–23.5)	8.8 (6.9–11.0)	1.3 (1.0–1.7)	41.4 (32.1–52.9)	0.0 (0.0–0.0)	11.5 (7.6–16.8)	14.0 (10.2–18.2)	1.3 (0.9–1.9)	0.5 (0.4–0.6)
Virgin Islands, U.S.	Rate: Cumulative change (%)	-45.3 (-54.3 to -34.1)	-59.7 (-69.5 to -48.7)	-39.8 (-58.8 to -15.9)	-55.9 (-66.8 to -41.4)	-77.5 (-84.7 to -68.5)	-16.2 (-38.9 to 8.4)	nan (nan–nan)	31.6 (-28.4 to 122.6)	-47.7 (-64.5 to -27.5)	-71.3 (-82.8 to -56.6)	-63.8 (-76.1 to -46.0)
Tropical Latin America	Number: 1990	196,074 (191,857–200,553)	42,309 (39,986–44,222)	14,189 (13,336–15,270)	30,517 (29,099–31,914)	40,956 (38,873–43,033)	12,490 (10,598–16,707)	264 (184–358)	9,689 (6,997–11,769)	4,045 (3,684–4,535)	8,659 (7,496–9,488)	5,488 (4,374–6,115)
Tropical Latin America	Number: 2015	53,739 (50,597–56,941)	10,140 (9,048–11,500)	6,083 (5,446–6,858)	4,899 (4,397–5,446)	1,851 (1,632–2,073)	9,761 (7,519–11,258)	5 (2–10)	5,271 (4,024–6,561)	4,525 (3,779–5,185)	1,024 (852–1,336)	1,003 (839–1,273)
Tropical Latin America	Number: Cumulative change (%)	-72.6 (-74.3 to -70.8)	-76.0 (-78.6 to -72.7)	-57.1 (-62.0 to -51.8)	-83.9 (-85.8 to -81.9)	-95.5 (-96.1 to -94.8)	-19.3 (-50.3 to 2.0)	-97.8 (-99.0 to -95.5)	-45.4 (-52.3 to -38.7)	12.5 (-12.2 to 33.2)	-88.1 (-90.4 to -83.3)	-81.3 (-85.6 to -73.7)
Tropical Latin America	Rate: 1990	1,073.9 (1,050.6–1,098.5)	232.5 (219.7–243.0)	78.0 (73.3–83.9)	167.0 (159.2–174.6)	224.6 (213.1–236.1)	68.5 (58.1–91.7)	1.4 (1.0–2.0)	53.3 (38.5–64.8)	22.2 (20.2–24.9)	47.4 (41.0–51.9)	30.0 (23.9–33.4)
Tropical Latin America	Rate: 2015	349.5 (329.0–370.4)	66.4 (59.2–75.3)	39.8 (35.6–44.9)	31.7 (28.4–35.2)	12.0 (10.5–13.4)	63.5 (49.0–73.3)	0.0 (0.0–0.1)	34.5 (26.3–42.9)	29.6 (24.7–33.9)	6.6 (5.5–8.6)	6.5 (5.4–8.2)
Tropical Latin America	Rate: Cumulative change (%)	-67.5 (-69.5 to -65.3)	-71.4 (-74.5 to -67.5)	-48.9 (-54.7 to -42.7)	-81.0 (-83.2 to -78.6)	-94.7 (-95.4 to -93.8)	-4.3 (-41.1 to 21.0)	-97.4 (-98.9 to -94.7)	-35.1 (-43.3 to -27.1)	33.8 (4.4–58.5)	-86.0 (-88.7 to -80.4)	-77.9 (-83.0 to -68.9)
Brazil	Number: 1990	191,504 (187,283–196,013)	41,385 (39,036–43,235)	13,783 (12,919–14,881)	29,778 (28,345–31,152)	40,369 (38,259–42,440)	12,061 (10,184–16,218)	264 (184–358)	9,420 (6,857–11,602)	3,916 (3,568–4,397)	8,564 (7,401–9,385)	5,347 (4,222–5,990)
Brazil	Number: 2015	51,226 (48,135–54,489)	9,587 (8,502–10,920)	5,833 (5,199–6,618)	4,677 (4,194–5,211)	1,760 (1,554–1,975)	9,241 (7,179–10,686)	5 (2–10)	5,111 (3,954–6,454)	4,405 (3,643–5,072)	937 (780–1,248)	945 (796–1,180)
Brazil	Number: Cumulative change (%)	-73.2 (-74.9 to -71.4)	-76.8 (-79.4 to -73.6)	-57.6 (-62.6 to -52.1)	-84.3 (-86.1 to -82.2)	-95.6 (-96.2 to -95.0)	-20.8 (-51.3 to 0.3)	-97.8 (-99.1 to -95.6)	-45.5 (-52.5 to -38.6)	13.1 (-12.3 to 34.9)	-89.0 (-91.1 to -84.0)	-81.9 (-86.0 to -74.8)
Brazil	Rate: 1990	1,089.4 (1,065.3–1,115.0)	236.3 (222.9–246.8)	78.7 (73.7–84.9)	169.2 (161.0–177.0)	230.0 (217.9–241.8)	68.7 (58.0–92.5)	1.5 (1.0–2.0)	53.8 (39.2–66.3)	22.4 (20.4–25.1)	48.7 (42.1–53.4)	30.3 (23.9–34.0)
Brazil	Rate: 2015	348.6 (327.5–370.8)	65.7 (58.3–74.8)	39.9 (35.6–45.3)	31.6 (28.4–35.3)	11.9 (10.5–13.4)	62.9 (48.9–72.8)	0.0 (0.0–0.1)	35.0 (27.1–44.2)	30.1 (24.9–34.7)	6.3 (5.3–8.4)	6.4 (5.4–8.0)
Brazil	Rate: Cumulative change (%)	-68.0 (-70.0 to -65.8)	-72.2 (-75.3 to -68.3)	-49.2 (-55.2 to -42.5)	-81.3 (-83.5 to -78.8)	-94.8 (-95.5 to -94.0)	-5.4 (-41.8 to 19.9)	-97.4 (-98.9 to -94.7)	-34.8 (-43.1 to -26.5)	35.6 (5.1–61.7)	-86.9 (-89.4 to -80.9)	-78.4 (-83.4 to -70.0)
Paraguay	Number: 1990	4,569 (4,265–4,865)	924 (786–1,091)	405 (318–494)	738 (621–856)	587 (481–703)	429 (364–505)	0 (0–0)	268 (75–381)	129 (99–163)	95 (61–143)	140 (102–179)
Paraguay	Number: 2015	2,513 (2,076–3,050)	552 (412–717)	250 (182–335)	222 (167–286)	90 (62–127)	519 (328–688)	0 (0–0)	159 (68–227)	119 (86–159)	86 (45–146)	58 (31–95)
Paraguay	Number: Cumulative change (%)	-44.9 (-55.6 to -32.4)	-39.9 (-55.1 to -21.2)	-37.6 (-57.1 to -12.8)	-69.6 (-77.7 to -59.8)	-84.3 (-90.1 to -76.5)	22.8 (-29.7 to 70.6)	-65.9 (-73.6 to -57.2)	-35.2 (-58.0 to -2.1)	-5.3 (-37.1 to 34.5)	-3.8 (-53.5 to 74.2)	-58.3 (-78.8 to -33.5)
Paraguay	Rate: 1990	674.7 (630.3–718.3)	135.7 (115.4–160.2)	59.6 (46.7–72.6)	109.3 (92.0–126.8)	87.0 (71.3–104.2)	63.2 (53.6–74.4)	0.0 (0.0–0.0)	39.4 (11.0–56.0)	19.0 (14.6–24.1)	14.2 (9.1–21.3)	20.9 (15.2–26.7)
Paraguay	Rate: 2015	368.7 (304.4–447.6)	81.3 (60.7–105.6)	36.8 (26.8–49.4)	32.5 (24.5–41.8)	13.3 (9.2–18.7)	76.2 (48.2–101.1)	0.0 (0.0–0.0)	23.5 (10.1–33.4)	17.7 (12.7–23.4)	12.6 (6.6–21.4)	8.5 (4.6–13.9)
Paraguay	Rate: Cumulative change (%)	-45.3 (-55.9 to -32.8)	-39.8 (-54.9 to -21.0)	-37.4 (-57.0 to -12.5)	-70.0 (-77.9 to -60.3)	-84.5 (-90.2 to -76.9)	22.3 (-29.9 to 70.0)	-66.7 (-74.3 to -58.2)	-35.1 (-57.9 to -1.9)	-5.1 (-37.0 to 34.8)	-5.4 (-54.5 to 70.6)	-58.9 (-79.1 to -34.5)
Southeast Asia, East Asia, and Oceania	Number: 1990	2,390,435 (2,318,947–2,466,903)	457,908 (431,414–484,352)	135,467 (118,453–153,693)	599,344 (544,325–652,560)	147,364 (120,843–171,649)	198,517 (147,112–264,099)	9,643 (5,657–14,930)	33,195 (25,846–43,003)	68,679 (55,107–84,266)	38,967 (25,367–49,722)	44,762 (29,778–60,503)
Southeast Asia, East Asia, and Oceania	Number: 2015	486,214 (462,350–510,457)	87,932 (80,019–96,902)	60,014 (53,801–67,352)	68,893 (62,197–75,991)	16,805 (13,565–20,223)	84,555 (75,961–94,340)	1,176 (652–1,886)	21,461 (16,733–27,894)	18,170 (14,811–22,519)	2,563 (2,097–3,270)	8,909 (6,925–11,438)
Southeast Asia, East Asia, and Oceania	Number: Cumulative change (%)	-79.7 (-80.8 to -78.5)	-80.8 (-82.8 to -78.6)	-55.4 (-62.3 to -47.6)	-88.5 (-90.0 to -86.8)	-88.5 (-91.2 to -85.2)	-55.9 (-69.5 to -39.9)	-87.0 (-93.7 to -77.0)	-34.4 (-49.6 to -16.2)	-73.1 (-80.0 to -65.1)	-93.2 (-95.3 to -89.8)	-79.3 (-86.0 to -70.6)
Southeast Asia, East Asia, and Oceania	Rate: 1990	1,257.4 (1,219.8–1,297.6)	240.8 (226.8–254.7)	71.2 (62.3–80.8)	315.1 (286.2–343.2)	77.5 (63.5–90.3)	104.4 (77.4–138.9)	5.1 (3.0–7.9)	17.5 (13.6–22.6)	36.1 (29.0–44.3)	20.5 (13.3–26.1)	23.5 (15.7–31.8)
Southeast Asia, East Asia, and Oceania	Rate: 2015	336.2 (319.8–353.0)	61.3 (55.8–67.6)	41.9 (37.5–47.0)	47.5 (42.9–52.3)	11.5 (9.3–13.9)	58.5 (52.6–65.3)	0.8 (0.4–1.3)	15.0 (11.7–19.4)	12.7 (10.3–15.7)	1.8 (1.4–2.2)	6.1 (4.8–7.9)
Southeast Asia, East Asia, and Oceania	Rate: Cumulative change (%)	-73.3 (-74.8 to -71.7)	-74.5 (-77.1 to -71.6)	-40.9 (-50.0 to -30.5)	-84.9 (-86.9 to -82.7)	-84.9 (-88.5 to -80.7)	-42.0 (-59.9 to -20.9)	-83.0 (-91.8 to -70.2)	-13.2 (-33.3 to 10.9)	-64.4 (-73.5 to -53.7)	-91.1 (-93.8 to -86.7)	-73.0 (-81.7 to -61.6)
East Asia	Number: 1990	1,506,630 (1,435,641–1,581,704)	333,663 (309,790–356,855)	72,246 (59,924–88,163)	395,506 (360,592–430,622)	58,941 (50,503–67,625)	153,386 (115,751–200,524)	3 (1–5)	7,371 (4,462–10,090)	38,942 (30,212–47,860)	27,269 (17,186–33,150)	21,506 (11,655–29,255)
East Asia	Number: 2015	214,532 (200,110–230,220)	39,620 (34,934–44,569)	27,558 (23,991–31,311)	25,389 (22,820–29,001)	1,990 (1,680–2,356)	48,860 (41,880–59,374)	0 (0–1)	2,766 (1,645–3,572)	7,121 (5,878–8,534)	817 (666–994)	2,109 (1,125–2,753)
East Asia	Number: Cumulative change (%)	-85.7 (-86.9 to -84.5)	-88.1 (-89.5 to -86.5)	-61.4 (-69.4 to -53.4)	-93.6 (-94.5 to -92.4)	-96.6 (-97.3 to -95.7)	-67.0 (-77.2 to -51.3)	-72.2 (-87.1 to -51.5)	-61.8 (-71.0 to -50.6)	-81.4 (-86.1 to -75.3)	-96.9 (-97.8 to -95.3)	-90.0 (-92.4 to -86.7)
East Asia	Rate: 1990	1,158.6 (1,104.0–1,216.2)	257.5 (239.1–275.4)	55.8 (46.3–68.0)	303.6 (276.8–330.5)	45.2 (38.7–51.9)	117.9 (89.0–154.1)	0.0 (0.0–0.0)	5.7 (3.4–7.8)	30.0 (23.3–36.9)	20.9 (13.2–25.4)	16.5 (8.9–22.4)
East Asia	Rate: 2015	254.0 (236.9–272.5)	47.4 (41.8–53.3)	33.0 (28.7–37.5)	30.0 (26.9–34.2)	2.3 (2.0–2.8)	57.8 (49.6–70.3)	0.0 (0.0–0.0)	3.3 (2.0–4.3)	8.5 (7.0–10.2)	1.0 (0.8–1.2)	2.5 (1.3–3.2)
East Asia	Rate: Cumulative change (%)	-78.1 (-79.8 to -76.1)	-81.6 (-83.7 to -79.0)	-40.1 (-52.5 to -27.7)	-90.1 (-91.5 to -88.4)	-94.8 (-95.9 to -93.5)	-49.2 (-64.9 to -25.0)	-57.7 (-80.4 to -26.4)	-40.9 (-55.1 to -23.6)	-71.2 (-78.5 to -61.7)	-95.3 (-96.6 to -92.8)	-84.7 (-88.4 to -79.7)
China	Number: 1990	1,480,311 (1,408,815–1,555,513)	328,530 (304,277–351,973)	70,391 (58,142–86,168)	389,570 (355,172–425,997)	58,377 (50,112–66,999)	149,690 (113,615–193,675)	0 (0–1)	7,166 (4,336–9,812)	38,126 (29,730–46,961)	26,897 (16,918–32,819)	21,191 (11,400–28,896)
China	Number: 2015	206,243 (192,112–220,798)	38,083 (33,565–42,783)	26,479 (22,922–30,161)	24,246 (21,761–27,730)	1,919 (1,609–2,276)	47,050 (40,402–57,497)	0 (0–0)	2,637 (1,576–3,422)	6,726 (5,545–8,083)	770 (625–941)	2,042 (1,076–2,680)
China	Number: Cumulative change (%)	-86.1 (-87.2 to -84.8)	-88.4 (-89.7 to -86.8)	-61.9 (-69.6 to -53.7)	-93.8 (-94.6 to -92.7)	-96.7 (-97.4 to -95.8)	-67.4 (-77.8 to -52.0)	-39.7 (-64.5 to -7.8)	-62.5 (-71.5 to -51.1)	-82.0 (-86.6 to -76.2)	-97.0 (-97.9 to -95.5)	-90.2 (-92.6 to -86.7)
China	Rate: 1990	1,170.7 (1,114.2–1,230.1)	260.9 (241.6–279.5)	55.9 (46.2–68.4)	307.5 (280.3–336.2)	46.0 (39.5–52.8)	118.4 (89.8–153.1)	0.0 (0.0–0.0)	5.7 (3.4–7.8)	30.3 (23.6–37.3)	21.2 (13.3–25.9)	16.7 (9.0–22.8)
China	Rate: 2015	252.5 (235.1–270.3)	47.1 (41.5–53.0)	32.8 (28.4–37.3)	29.6 (26.6–33.9)	2.3 (2.0–2.8)	57.6 (49.4–70.4)	0.0 (0.0–0.0)	3.3 (1.9–4.2)	8.3 (6.8–10.0)	0.9 (0.8–1.1)	2.5 (1.3–3.2)
China	Rate: Cumulative change (%)	-78.4 (-80.1 to -76.5)	-81.9 (-84.0 to -79.4)	-40.6 (-52.6 to -27.8)	-90.3 (-91.7 to -88.7)	-94.9 (-96.0 to -93.6)	-49.6 (-65.6 to -25.7)	-7.9 (-45.8 to 40.9)	-41.7 (-55.6 to -24.0)	-72.0 (-79.1 to -63.0)	-95.4 (-96.8 to -93.0)	-84.9 (-88.7 to -79.6)
North Korea	Number: 1990	23,446 (11,684–40,817)	4,695 (2,529–7,649)	1,758 (872–2,996)	5,867 (2,461–11,220)	545 (174–1,130)	2,881 (1,163–5,369)	2 (0–4)	146 (52–320)	614 (225–1,245)	366 (94–899)	278 (91–624)
North Korea	Number: 2015	6,905 (3,488–11,903)	1,368 (588–2,457)	1,017 (420–1,902)	1,111 (572–1,988)	63 (26–134)	1,361 (675–2,413)	0 (0–0)	76 (25–161)	237 (73–519)	45 (15–105)	54 (21–114)
North Korea	Number: Cumulative change (%)	-66.1 (-87.4 to -27.9)	-67.9 (-88.1 to -32.8)	-33.6 (-78.6 to 40.9)	-76.7 (-92.4 to -45.2)	-84.2 (-96.3 to -60.4)	-42.7 (-81.3 to 25.4)	-87.0 (-96.0 to -69.9)	-33.8 (-83.9 to 60.0)	-48.2 (-90.1 to 37.3)	-80.5 (-96.8 to -43.7)	-72.2 (-94.2 to -22.3)
North Korea	Rate: 1990	1,179.9 (586.9–2,056.2)	232.8 (125.4–379.4)	87.1 (43.2–148.5)	295.7 (124.1–565.5)	27.5 (8.7–57.1)	144.7 (58.5–270.2)	0.1 (0.0–0.2)	7.3 (2.6–15.9)	30.5 (11.2–62.0)	18.6 (4.8–45.6)	14.2 (4.7–31.7)
North Korea	Rate: 2015	395.7 (199.8–682.0)	78.6 (33.8–141.2)	58.4 (24.2–109.3)	63.6 (32.8–113.8)	3.6 (1.5–7.7)	77.9 (38.7–138.3)	0.0 (0.0–0.0)	4.4 (1.5–9.3)	13.6 (4.2–29.8)	2.6 (0.9–6.0)	3.1 (1.2–6.5)
North Korea	Rate: Cumulative change (%)	-61.3 (-85.7 to -17.8)	-62.8 (-86.3 to -22.0)	-23.0 (-75.2 to 63.4)	-73.6 (-91.4 to -37.8)	-82.1 (-95.9 to -55.2)	-34.6 (-78.8 to 42.7)	-85.5 (-95.5 to -66.6)	-23.4 (-81.4 to 85.2)	-40.1 (-88.5 to 59.2)	-78.0 (-96.4 to -36.7)	-68.8 (-93.4 to -12.9)
Taiwan	Number: 1990	2,872 (2,787–2,956)	437 (312–545)	96 (54–149)	68 (51–86)	19 (11–30)	814 (657–974)	0 (0–0)	59 (24–116)	200 (110–319)	5 (1–13)	37 (19–65)
Taiwan	Number: 2015	1,382 (957–1,962)	169 (95–264)	61 (34–102)	30 (19–45)	6 (3–11)	448 (277–698)	0 (0–0)	52 (22–93)	158 (82–253)	1 (0–2)	12 (6–21)
Taiwan	Number: Cumulative change (%)	-51.9 (-66.4 to -31.6)	-60.4 (-78.6 to -35.4)	-29.8 (-68.5 to 30.9)	-54.0 (-72.1 to -28.2)	-61.9 (-83.3 to -26.8)	-44.8 (-65.5 to -16.7)	nan (nan–nan)	3.3 (-61.5 to 101.7)	-13.0 (-61.0 to 65.6)	-68.2 (-93.5 to -17.2)	-62.3 (-85.0 to -25.0)
Taiwan	Rate: 1990	178.3 (173.1–183.6)	26.9 (19.3–33.5)	5.9 (3.3–9.2)	4.3 (3.2–5.4)	1.2 (0.7–1.9)	50.6 (40.8–60.5)	0.0 (0.0–0.0)	3.6 (1.5–7.2)	12.3 (6.8–19.6)	0.4 (0.1–0.9)	2.3 (1.2–4.1)
Taiwan	Rate: 2015	134.1 (92.9–190.3)	16.4 (9.2–25.6)	6.0 (3.3–9.9)	3.0 (1.9–4.5)	0.7 (0.4–1.1)	43.4 (26.9–67.6)	0.0 (0.0–0.0)	5.1 (2.2–9.0)	15.3 (7.9–24.5)	0.1 (0.0–0.3)	1.2 (0.6–2.1)
Taiwan	Rate: Cumulative change (%)	-24.8 (-47.4 to 6.8)	-37.9 (-66.4 to 1.4)	10.2 (-50.5 to 105.5)	-28.3 (-56.5 to 11.8)	-41.1 (-74.3 to 12.8)	-14.0 (-46.4 to 30.2)	nan (nan–nan)	62.0 (-39.6 to 215.9)	36.6 (-38.7 to 159.8)	-50.5 (-89.9 to 29.8)	-41.4 (-76.7 to 16.5)
Southeast Asia	Number: 1990	867,528 (847,752–888,123)	122,834 (107,894–140,671)	62,520 (50,203–75,168)	199,500 (161,998–232,991)	87,164 (60,597–109,777)	44,532 (30,970–68,280)	9,231 (5,260–14,550)	25,592 (19,423–34,184)	29,145 (20,472–40,974)	11,002 (6,205–20,443)	22,812 (14,251–34,022)
Southeast Asia	Number: 2015	260,360 (242,733–279,996)	47,066 (40,823–54,048)	31,717 (26,406–37,667)	41,014 (35,487–46,661)	14,370 (11,283–17,661)	34,922 (30,594–39,164)	1,021 (511–1,672)	18,379 (14,296–24,176)	10,376 (7,292–14,396)	1,447 (1,069–1,975)	6,526 (4,974–8,659)

© 2017 Global Burden of Disease Child and Adolescent Health Collaboration. JAMA Pediatrics.

eTable 2. Number of Deaths, Death Rates (per 100,000 population), and Cumulative Percent Change with 95% Uncertainty Intervals (UI) for the Top 10 Global Causes of Death in 195 Countries and Territories, Aged Under 5 Years, Both Sexes, 1990 and 2015 *Note: best viewed by enlarging in browser.*

Location	Measure	All causes	Neonatal preterm birth complications	Neonatal encephalopathy due to birth asphyxia and trauma	Lower respiratory infections	Diarrheal diseases	Congenital anomalies	Malaria	Neonatal sepsis and other neonatal infections	Other neonatal disorders	Protein-energy malnutrition	Meningitis
Southeast Asia	Number: Cumulative change (%)	-70.0 (-72.1 to -67.7)	-61.5 (-67.7 to -54.9)	-48.6 (-60.9 to -33.9)	-79.2 (-83.2 to -73.6)	-83.0 (-88.2 to -75.6)	-16.9 (-50.3 to 8.1)	-88.0 (-94.7 to -78.4)	-26.7 (-46.9 to -1.5)	-62.8 (-78.0 to -44.4)	-85.1 (-93.4 to -74.7)	-69.3 (-81.8 to -49.6)
Southeast Asia	Rate: 1990	1,471.0 (1,437.2–1,505.6)	206.3 (181.1–236.3)	105.0 (84.3–126.2)	339.1 (275.1–396.2)	148.4 (103.1–186.9)	75.2 (52.3–115.5)	15.8 (9.0–24.8)	43.0 (32.6–57.5)	49.0 (34.4–68.9)	18.8 (10.6–34.8)	38.8 (24.2–57.9)
Southeast Asia	Rate: 2015	443.0 (413.1–476.3)	80.6 (69.9–92.6)	54.3 (45.2–64.5)	69.5 (60.2–79.1)	24.3 (19.1–29.9)	59.5 (52.1–66.7)	1.7 (0.9–2.8)	31.5 (24.5–41.4)	17.8 (12.5–24.6)	2.4 (1.8–3.3)	11.0 (8.4–14.7)
Southeast Asia	Rate: Cumulative change (%)	-69.9 (-72.0 to -67.5)	-60.7 (-67.1 to -54.0)	-47.5 (-60.1 to -32.5)	-79.2 (-83.3 to -73.7)	-83.1 (-88.2 to -75.7)	-16.2 (-49.9 to 9.0)	-88.2 (-94.8 to -78.6)	-25.4 (-45.9 to 0.2)	-62.1 (-77.6 to -43.4)	-85.2 (-93.5 to -75.0)	-69.4 (-81.9 to -49.9)
Cambodia	Number: 1990	45,394 (43,967–46,858)	6,492 (5,081–7,887)	1,398 (840–2,097)	12,690 (10,160–15,176)	5,052 (3,471–6,788)	1,882 (929–4,195)	998 (457–1,760)	784 (335–1,474)	1,563 (612–2,899)	665 (226–1,488)	1,384 (694–2,420)
Cambodia	Number: 2015	11,603 (10,267–13,035)	2,610 (1,963–3,274)	1,036 (662–1,481)	2,135 (1,660–2,648)	459 (276–720)	1,366 (952–1,886)	118 (37–270)	592 (299–1,009)	638 (303–1,111)	62 (21–151)	328 (176–548)
Cambodia	Number: Cumulative change (%)	-74.4 (-77.5 to -71.2)	-59.1 (-71.2 to -45.5)	-19.7 (-59.7 to 39.6)	-83.0 (-87.2 to -77.7)	-90.5 (-95.1 to -83.6)	-11.2 (-59.7 to 35.9)	-86.8 (-95.9 to -69.9)	-8.0 (-67.1 to 102.9)	-50.2 (-83.5 to 13.0)	-87.2 (-97.5 to -63.9)	-72.8 (-89.6 to -47.1)
Cambodia	Rate: 1990	2,550.0 (2,469.4–2,633.7)	354.9 (277.8–431.0)	76.5 (46.1–114.7)	716.5 (572.7–856.7)	286.1 (196.7–384.2)	104.7 (51.5–234.3)	57.1 (26.1–100.8)	43.0 (18.4–81.0)	85.8 (33.6–159.1)	37.9 (13.0–84.6)	78.3 (39.2–137.0)
Cambodia	Rate: 2015	650.4 (575.5–730.6)	146.4 (110.0–183.6)	58.1 (37.1–83.0)	119.7 (93.1–148.5)	25.8 (15.5–40.4)	76.6 (53.4–105.7)	6.6 (2.1–15.2)	33.2 (16.8–56.6)	35.8 (17.0–62.3)	3.5 (1.2–8.5)	18.4 (9.9–30.8)
Cambodia	Rate: Cumulative change (%)	-74.5 (-77.6 to -71.2)	-58.1 (-70.5 to -44.0)	-17.7 (-58.7 to 42.7)	-83.1 (-87.3 to -77.8)	-90.6 (-95.1 to -83.8)	-10.3 (-59.4 to 37.7)	-87.0 (-95.9 to -70.5)	-6.0 (-66.3 to 107.7)	-49.2 (-83.1 to 15.0)	-87.4 (-97.5 to -64.7)	-73.1 (-89.6 to -47.8)
Indonesia	Number: 1990	397,066 (389,048–405,185)	50,390 (39,029–62,691)	36,577 (25,406–48,589)	78,878 (46,857–102,988)	49,572 (25,786–69,501)	13,361 (7,792–25,295)	2,677 (1,036–4,889)	8,387 (4,262–14,532)	12,074 (5,585–21,260)	5,782 (1,513–15,288)	12,763 (5,194–22,525)
Indonesia	Number: 2015	119,592 (105,683–135,944)	23,223 (18,149–29,136)	18,168 (13,116–23,619)	15,250 (11,018–19,133)	8,559 (5,635–11,633)	12,933 (10,232–15,817)	329 (157–553)	5,805 (2,883–10,298)	5,118 (2,524–8,974)	510 (225–943)	3,397 (1,977–5,389)
Indonesia	Number: Cumulative change (%)	-69.9 (-73.4 to -65.7)	-53.0 (-67.3 to -36.5)	-48.5 (-67.0 to -22.3)	-79.3 (-87.2 to -65.8)	-80.8 (-89.9 to -64.8)	11.2 (-51.0 to 74.4)	-84.6 (-95.2 to -66.6)	-22.4 (-68.9 to 51.3)	-50.7 (-82.5 to -3.0)	-86.0 (-97.5 to -63.3)	-67.3 (-87.6 to -29.1)
Indonesia	Rate: 1990	1,788.5 (1,752.7–1,825.1)	224.8 (174.0–279.4)	163.0 (113.2–216.4)	356.3 (211.4–465.6)	224.2 (116.4–314.3)	60.0 (35.0–113.8)	12.1 (4.7–22.2)	37.4 (19.1–64.9)	53.9 (25.0–94.8)	26.2 (6.8–69.4)	57.7 (23.4–101.9)
Indonesia	Rate: 2015	488.4 (431.5–555.1)	95.4 (74.6–119.7)	74.7 (53.9–97.1)	62.1 (44.9–77.8)	34.8 (22.9–47.2)	52.9 (41.9–64.7)	1.3 (0.6–2.2)	23.9 (11.8–42.3)	21.0 (10.4–36.9)	2.1 (0.9–3.8)	13.8 (8.0–21.9)
Indonesia	Rate: Cumulative change (%)	-72.7 (-75.9 to -68.9)	-56.7 (-69.9 to -41.5)	-52.5 (-69.6 to -28.3)	-81.3 (-88.5 to -69.2)	-82.7 (-91.0 to -68.4)	1.3 (-55.4 to 59.3)	-86.2 (-95.7 to -70.0)	-28.6 (-71.4 to 39.2)	-54.7 (-83.9 to -11.0)	-87.5 (-97.7 to -67.2)	-70.6 (-88.8 to -36.3)
Laos	Number: 1990	33,026 (30,986–35,273)	5,352 (3,902–6,587)	972 (533–1,574)	8,329 (6,237–10,771)	5,088 (3,279–7,040)	1,154 (486–3,113)	56 (16–111)	559 (237–1,101)	1,201 (488–2,246)	412 (129–898)	934 (386–1,699)
Laos	Number: 2015	10,744 (8,476–13,320)	2,301 (1,669–3,026)	771 (452–1,203)	2,384 (1,574–3,374)	811 (483–1,265)	980 (571–1,710)	7 (3–15)	462 (215–866)	539 (254–993)	64 (22–156)	346 (159–629)
Laos	Number: Cumulative change (%)	-67.4 (-74.5 to -58.9)	-56.4 (-68.5 to -40.4)	-11.5 (-62.5 to 68.1)	-70.8 (-81.2 to -57.2)	-83.3 (-91.2 to -72.1)	7.2 (-53.3 to 66.8)	-81.4 (-94.6 to -55.1)	1.8 (-67.1 to 147.9)	-44.9 (-82.4 to 26.2)	-78.1 (-95.7 to -40.0)	-56.9 (-83.7 to -12.5)
Laos	Rate: 1990	4,169.7 (3,917.3–4,447.0)	623.1 (454.5–769.2)	113.2 (62.2–183.0)	1,063.3 (793.9–1,377.0)	655.2 (422.1–906.8)	141.2 (58.9–383.4)	7.5 (2.2–15.1)	65.4 (27.8–128.9)	140.9 (57.5–262.0)	54.0 (16.8–117.5)	119.7 (49.4–216.7)
Laos	Rate: 2015	1,266.6 (998.8–1,571.0)	268.8 (195.0–353.5)	90.1 (52.9–140.5)	282.4 (186.3–399.7)	96.3 (57.4–150.1)	115.5 (67.2–201.5)	0.9 (0.4–1.9)	54.1 (25.3–101.2)	63.1 (29.8–116.0)	7.7 (2.7–18.7)	41.1 (18.9–74.6)
Laos	Rate: Cumulative change (%)	-69.6 (-76.2 to -61.6)	-56.2 (-68.4 to -40.6)	-11.2 (-62.2 to 69.0)	-72.9 (-82.5 to -60.0)	-84.6 (-92.0 to -74.2)	4.3 (-55.5 to 63.2)	-83.3 (-95.2 to -59.9)	1.7 (-67.1 to 147.3)	-45.1 (-82.3 to 25.2)	-80.2 (-96.0 to -45.3)	-60.1 (-84.9 to -19.3)
Malaysia	Number: 1990	8,506 (8,369–8,654)	1,480 (1,226–1,724)	575 (441–731)	781 (470–1,061)	204 (107–321)	1,192 (885–1,565)	3 (1–7)	461 (248–798)	827 (633–1,056)	19 (5–48)	251 (120–443)
Malaysia	Number: 2015	3,274 (2,846–3,784)	374 (276–493)	210 (153–282)	241 (180–318)	50 (31–75)	923 (683–1,146)	0 (0–1)	249 (145–384)	206 (149–275)	4 (1–9)	94 (53–163)
Malaysia	Number: Cumulative change (%)	-61.5 (-66.6 to -55.5)	-74.5 (-81.7 to -65.9)	-62.5 (-75.9 to -46.4)	-67.2 (-79.8 to -46.6)	-72.6 (-87.2 to -47.2)	-20.1 (-50.3 to 12.6)	-80.5 (-95.1 to -53.0)	-40.2 (-73.9 to 6.2)	-74.5 (-83.1 to -62.1)	-66.7 (-94.2 to -4.5)	-57.4 (-83.4 to -16.3)
Malaysia	Rate: 1990	346.0 (340.4–351.9)	59.6 (49.4–69.5)	23.2 (17.8–29.5)	31.9 (19.0–43.1)	8.3 (4.4–13.1)	48.2 (35.7–63.3)	0.2 (0.1–0.3)	18.6 (10.0–32.1)	33.3 (25.5–42.6)	0.8 (0.2–2.0)	10.3 (4.9–18.1)
Malaysia	Rate: 2015	132.9 (115.5–153.5)	15.2 (11.2–20.0)	8.5 (6.2–11.5)	9.8 (7.3–12.9)	2.0 (1.3–3.1)	37.4 (27.7–46.4)	0.0 (0.0–0.0)	10.1 (5.9–15.6)	8.4 (6.0–11.2)	0.2 (0.1–0.4)	3.8 (2.2–6.6)
Malaysia	Rate: Cumulative change (%)	-61.6 (-66.6 to -55.6)	-74.3 (-81.6 to -65.7)	-62.3 (-75.8 to -46.1)	-67.4 (-79.9 to -46.8)	-72.8 (-87.2 to -47.5)	-20.0 (-50.1 to 12.8)	-80.7 (-95.1 to -53.5)	-39.9 (-73.8 to 6.8)	-74.3 (-83.0 to -61.9)	-67.0 (-94.3 to -4.6)	-57.7 (-83.4 to -17.8)
Maldives	Number: 1990	615 (583–647)	45 (32–62)	43 (32–56)	72 (36–107)	42 (19–68)	75 (44–122)	0 (0–0)	10 (6–16)	47 (34–62)	3 (1–8)	9 (3–16)
Maldives	Number: 2015	93 (80–109)	9 (7–13)	10 (7–13)	5 (4–7)	1 (1–2)	22 (16–28)	0 (0–0)	3 (1–5)	7 (4–9)	0 (0–0)	1 (0–1)
Maldives	Number: Cumulative change (%)	-84.8 (-87.0 to -82.2)	-77.1 (-86.6 to -66.4)	-75.8 (-83.6 to -65.5)	-91.4 (-95.6 to -84.7)	-94.5 (-97.6 to -88.7)	-67.2 (-84.1 to -43.7)	nan (nan–nan)	-68.4 (-81.3 to -52.6)	-84.5 (-90.2 to -77.2)	-91.0 (-98.1 to -76.7)	-86.5 (-95.0 to -69.4)
Maldives	Rate: 1990	1,414.7 (1,340.9–1,487.9)	102.7 (74.0–143.0)	99.6 (74.1–129.2)	166.9 (84.6–246.6)	97.8 (44.4–157.7)	173.4 (101.9–281.2)	0.0 (0.0–0.0)	23.0 (14.6–37.9)	109.4 (78.9–143.8)	8.6 (2.4–20.0)	21.6 (8.5–37.7)
Maldives	Rate: 2015	254.5 (218.4–297.9)	27.1 (19.2–36.4)	28.1 (21.2–37.5)	15.2 (11.0–19.3)	5.4 (3.5–7.9)	60.4 (46.2–76.5)	0.0 (0.0–0.0)	8.4 (5.3–14.9)	19.7 (13.2–27.1)	0.6 (0.3–1.1)	2.8 (1.7–4.3)
Maldives	Rate: Cumulative change (%)	-82.0 (-84.6 to -78.9)	-72.6 (-83.9 to -59.7)	-71.0 (-80.3 to -58.7)	-89.8 (-94.8 to -81.9)	-93.5 (-97.2 to -86.7)	-61.0 (-81.1 to -33.0)	nan (nan–nan)	-62.2 (-77.6 to -43.4)	-81.5 (-88.2 to -72.7)	-89.4 (-97.8 to -72.6)	-84.1 (-94.1 to -63.9)
Mauritius	Number: 1990	494 (481–507)	172 (158–185)	57 (48–67)	34 (18–46)	27 (12–37)	57 (39–70)	0 (0–0)	21 (12–28)	8 (6–9)	1 (0–1)	6 (3–8)
Mauritius	Number: 2015	188 (174–203)	48 (41–57)	14 (12–18)	11 (8–13)	4 (3–5)	45 (29–53)	0 (0–0)	10 (6–13)	12 (9–14)	0 (0–0)	1 (1–2)
Mauritius	Number: Cumulative change (%)	-61.9 (-64.9 to -58.7)	-72.1 (-76.0 to -66.9)	-73.6 (-79.4 to -66.8)	-64.4 (-77.3 to -34.5)	-82.4 (-89.6 to -66.5)	-19.4 (-39.0 to 11.8)	nan (nan–nan)	-51.1 (-64.8 to -36.8)	49.5 (12.5–96.2)	-70.4 (-86.3 to -34.5)	-69.8 (-80.9 to -47.3)
Mauritius	Rate: 1990	462.6 (450.6–475.0)	160.7 (148.0–173.1)	53.2 (45.1–62.5)	32.8 (16.9–43.5)	25.9 (12.1–34.9)	54.0 (36.6–66.3)	0.0 (0.0–0.0)	20.2 (11.5–26.5)	7.7 (6.2–9.3)	1.1 (0.4–1.6)	5.8 (3.2–7.6)
Mauritius	Rate: 2015	275.2 (255.3–297.9)	71.3 (61.1–85.7)	22.2 (18.3–26.8)	16.6 (12.7–20.2)	6.3 (4.4–8.0)	66.7 (43.5–78.5)	0.0 (0.0–0.0)	15.3 (10.3–20.4)	18.0 (14.8–21.6)	0.4 (0.3–0.6)	2.5 (1.9–3.2)
Mauritius	Rate: Cumulative change (%)	-40.5 (-45.3 to -35.5)	-55.6 (-61.8 to -47.3)	-58.0 (-67.3 to -47.2)	-45.0 (-64.8 to 1.4)	-72.9 (-84.0 to -48.2)	26.2 (-4.4 to 75.2)	nan (nan–nan)	-22.3 (-44.1 to 0.4)	137.5 (78.6–211.7)	-55.0 (-79.2 to -1.0)	-53.3 (-70.6 to -18.3)
Myanmar	Number: 1990	126,216 (109,256–144,350)	14,363 (10,837–18,478)	11,193 (7,699–15,030)	42,335 (33,944–51,405)	14,726 (10,292–20,103)	4,978 (3,330–8,099)	4,566 (2,297–7,656)	1,652 (763–3,003)	2,751 (1,296–4,932)	337 (113–805)	3,028 (1,534–5,393)
Myanmar	Number: 2015	30,715 (24,583–38,400)	4,394 (3,003–6,068)	5,519 (3,884–7,393)	7,711 (5,720–10,508)	1,292 (772–2,006)	3,513 (2,541–4,686)	487 (201–916)	1,223 (543–2,374)	1,194 (590–2,103)	50 (16–122)	685 (374–1,222)
Myanmar	Number: Cumulative change (%)	-75.5 (-81.3 to -68.2)	-68.6 (-80.8 to -53.1)	-48.5 (-68.4 to -19.7)	-81.5 (-87.3 to -73.3)	-90.9 (-95.2 to -84.8)	-23.1 (-64.4 to 18.0)	-87.9 (-96.0 to -73.5)	-13.7 (-68.8 to 95.1)	-49.2 (-81.9 to 4.6)	-79.0 (-96.1 to -39.5)	-73.8 (-90.1 to -43.5)
Myanmar	Rate: 1990	2,365.2 (2,046.9–2,705.2)	266.4 (201.0–342.7)	207.5 (142.9–278.6)	796.0 (638.0–966.3)	277.1 (193.4–378.2)	93.0 (62.2–151.7)	85.9 (43.3–144.0)	30.7 (14.2–55.8)	51.2 (24.1–91.4)	6.3 (2.1–15.1)	57.0 (28.9–101.6)
Myanmar	Rate: 2015	670.4 (536.8–837.6)	96.7 (66.1–133.6)	121.5 (85.5–162.7)	167.9 (124.4–228.5)	28.0 (16.7–43.2)	76.9 (55.5–102.5)	10.5 (4.3–19.7)	26.9 (12.0–52.3)	26.3 (13.0–46.3)	1.1 (0.3–2.6)	14.9 (8.1–26.4)
Myanmar	Rate: Cumulative change (%)	-71.4 (-78.1 to -63.0)	-62.8 (-77.3 to -44.4)	-38.9 (-62.5 to -4.8)	-78.5 (-85.3 to -69.1)	-89.5 (-94.5 to -82.5)	-9.9 (-58.4 to 38.3)	-86.2 (-95.4 to -69.5)	2.1 (-63.0 to 130.8)	-40.0 (-78.5 to 23.4)	-76.2 (-95.5 to -31.8)	-69.8 (-88.6 to -35.2)
Philippines	Number: 1990	112,396 (109,124–115,803)	13,064 (10,429–15,380)	6,591 (5,225–8,019)	32,141 (23,345–37,457)	7,808 (5,641–9,346)	6,400 (5,366–7,470)	132 (72–199)	9,609 (7,387–13,515)	3,371 (2,499–4,252)	3,585 (2,415–4,528)	2,268 (1,622–2,728)
Philippines	Number: 2015	54,212 (48,946–60,346)	8,576 (6,717–10,243)	4,360 (3,153–5,432)	9,241 (7,840–10,778)	2,864 (2,319–3,435)	7,865 (6,616–9,325)	3 (1–6)	8,083 (6,290–11,057)	1,219 (898–1,554)	738 (554–965)	1,262 (1,024–1,524)
Philippines	Number: Cumulative change (%)	-51.7 (-57.0 to -46.1)	-34.1 (-46.1 to -21.5)	-33.2 (-50.6 to -12.5)	-70.6 (-77.0 to -60.1)	-62.4 (-72.6 to -48.1)	24.1 (0.7–54.8)	-97.1 (-98.9 to -93.8)	-15.1 (-31.4 to 3.8)	-63.2 (-73.6 to -51.0)	-78.7 (-85.3 to -67.8)	-43.0 (-58.4 to -19.9)
Philippines	Rate: 1990	1,167.0 (1,133.2–1,202.6)	132.9 (106.1–156.5)	67.1 (53.2–81.6)	335.8 (243.0–392.0)	81.6 (58.7–97.8)	65.9 (55.2–76.9)	1.4 (0.8–2.1)	97.9 (75.2–137.6)	34.3 (25.5–43.3)	37.5 (25.3–47.5)	23.7 (16.9–28.6)
Philippines	Rate: 2015	477.5 (431.1–531.5)	75.7 (59.3–90.4)	38.5 (27.8–47.9)	81.3 (69.0–94.8)	25.2 (20.4–30.2)	69.3 (58.3–82.1)	0.0 (0.0–0.1)	71.3 (55.4–97.5)	10.7 (7.9–13.7)	6.5 (4.9–8.5)	11.1 (9.0–13.4)
Philippines	Rate: Cumulative change (%)	-59.1 (-63.5 to -54.3)	-42.8 (-53.2 to -32.0)	-42.1 (-57.2 to -24.2)	-75.3 (-80.6 to -66.2)	-68.3 (-77.0 to -56.2)	6.3 (-13.7 to 32.6)	-97.6 (-99.1 to -94.8)	-26.5 (-40.6 to -10.1)	-68.2 (-77.1 to -57.6)	-82.1 (-87.7 to -72.8)	-52.0 (-65.0 to -32.4)
Sri Lanka	Number: 1990	14,758 (14,373–15,185)	3,222 (2,838–3,673)	1,439 (1,230–1,699)	1,729 (1,472–1,973)	1,157 (970–1,341)	674 (557–987)	0 (0–0)	329 (193–423)	1,767 (1,529–2,001)	117 (85–153)	271 (222–313)
Sri Lanka	Number: 2015	2,641 (2,106–3,307)	597 (460–757)	245 (181–318)	156 (117–206)	18 (13–24)	606 (455–785)	0 (0–0)	207 (134–279)	134 (99–171)	3 (2–5)	28 (21–36)
Sri Lanka	Number: Cumulative change (%)	-82.1 (-85.8 to -77.5)	-81.4 (-85.9 to -76.0)	-82.8 (-87.8 to -76.9)	-90.9 (-93.3 to -87.7)	-98.4 (-98.8 to -97.8)	-8.0 (-37.4 to 24.7)	nan (nan–nan)	-35.9 (-53.2 to -17.0)	-92.4 (-94.4 to -90.0)	-96.6 (-98.5 to -94.2)	-89.5 (-92.4 to -85.5)
Sri Lanka	Rate: 1990	848.7 (826.7–872.8)	187.7 (165.3–214.0)	83.9 (71.7–99.0)	98.9 (84.3–112.9)	65.9 (55.3–76.3)	38.9 (32.1–57.0)	0.0 (0.0–0.0)	19.2 (11.3–24.7)	102.9 (89.1–116.6)	6.7 (4.9–8.7)	15.6 (12.8–18.0)
Sri Lanka	Rate: 2015	164.6 (131.5–206.1)	38.1 (29.4–48.3)	15.6 (11.6–20.3)	9.6 (7.2–12.6)	1.1 (0.8–1.5)	37.6 (28.1–48.9)	0.0 (0.0–0.0)	13.2 (8.5–17.8)	8.5 (6.4–10.9)	0.2 (0.1–0.3)	1.7 (1.3–2.3)
Sri Lanka	Rate: Cumulative change (%)	-80.6 (-84.6 to -75.6)	-79.6 (-84.6 to -73.7)	-81.2 (-86.6 to -74.7)	-90.2 (-92.9 to -86.9)	-98.3 (-98.7 to -97.7)	-1.0 (-32.2 to 34.1)	nan (nan–nan)	-30.0 (-48.9 to -9.3)	-91.7 (-93.9 to -89.1)	-96.4 (-98.4 to -93.9)	-88.8 (-91.9 to -84.5)
Seychelles	Number: 1990	29 (27–32)	5 (4–7)	2 (1–3)	5 (4–7)	0 (0–0)	4 (3–7)	0 (0–0)	1 (0–2)	1 (0–2)	0 (0–0)	0 (0–1)
Seychelles	Number: 2015	17 (14–21)	2 (1–3)	1 (0–2)	3 (2–3)	0 (0–0)	4 (3–6)	0 (0–0)	1 (0–1)	0 (0–1)	0 (0–0)	0 (0–0)
Seychelles	Number: Cumulative change (%)	-40.6 (-51.5 to -27.9)	-61.2 (-75.1 to -43.9)	-31.2 (-65.1 to 15.3)	-46.7 (-59.2 to -32.2)	-76.9 (-84.6 to -67.2)	-5.0 (-31.9 to 26.6)	nan (nan–nan)	-7.3 (-59.7 to 84.1)	-28.5 (-72.9 to 41.2)	-80.3 (-90.7 to -59.3)	-38.3 (-55.2 to -16.1)
Seychelles	Rate: 1990	361.6 (332.2–392.7)	73.0 (53.7–93.8)	27.0 (16.3–39.9)	72.1 (59.0–84.8)	5.3 (4.1–6.9)	56.8 (40.1–89.9)	0.0 (0.0–0.0)	17.6 (8.9–31.0)	16.8 (8.4–29.2)	1.6 (0.8–2.3)	11.5 (9.1–13.8)
Seychelles	Rate: 2015	217.5 (181.6–261.4)	28.3 (19.8–39.2)	17.8 (10.7–26.8)	38.5 (29.6–47.2)	1.2 (0.9–1.6)	53.0 (39.0–77.6)	0.0 (0.0–0.0)	14.7 (7.4–24.1)	10.7 (5.3–19.1)	0.3 (0.2–0.4)	7.0 (5.4–9.0)
Seychelles	Rate: Cumulative change (%)	-39.7 (-50.8 to -26.8)	-60.4 (-74.5 to -42.6)	-29.7 (-64.3 to 18.2)	-46.1 (-58.8 to -31.5)	-76.7 (-84.5 to -66.8)	-3.5 (-30.9 to 28.7)	nan (nan–nan)	-5.3 (-58.9 to 88.4)	-27.0 (-72.3 to 44.2)	-80.1 (-90.7 to -59.0)	-37.6 (-54.7 to -15.6)
Thailand	Number: 1990	30,089 (27,212–33,294)	9,418 (7,880–11,258)	1,306 (889–1,829)	3,747 (2,877–4,604)	1,047 (715–1,371)	4,252 (2,121–5,706)	71 (38–114)	847 (602–1,229)	1,355 (1,015–1,798)	4 (1–8)	302 (197–417)
Thailand	Number: 2015	4,077 (3,402–4,896)	752 (550–997)	368 (218–560)	292 (213–385)	48 (31–72)	1,033 (681–1,351)	0 (0–1)	385 (180–562)	153 (103–220)	0 (0–1)	46 (27–73)
Thailand	Number: Cumulative change (%)	-86.4 (-89.0 to -83.3)	-91.9 (-94.2 to -89.2)	-70.8 (-83.9 to -51.8)	-92.1 (-94.4 to -88.9)	-95.2 (-97.2 to -92.4)	-73.1 (-84.7 to -46.8)	-98.5 (-99.5 to -96.5)	-52.9 (-75.9 to -26.5)	-88.3 (-93.1 to -81.9)	-78.9 (-95.6 to -44.0)	-83.9 (-91.5 to -71.3)
Thailand	Rate: 1990	573.0 (517.9–633.6)	181.0 (151.4–216.3)	25.1 (17.1–35.2)	71.3 (54.9–87.6)	19.8 (13.6–26.0)	81.4 (40.6–109.2)	1.3 (0.7–2.1)	16.3 (11.6–23.6)	26.0 (19.5–34.6)	0.1 (0.0–0.2)	5.7 (3.8–7.9)
Thailand	Rate: 2015	115.2 (96.2–138.5)	21.7 (15.9–28.8)	10.6 (6.3–16.2)	8.1 (5.9–10.7)	1.4 (0.9–2.0)	29.4 (19.4–38.5)	0.0 (0.0–0.1)	11.1 (5.2–16.2)	4.4 (3.0–6.3)	0.0 (0.0–0.1)	1.3 (0.8–2.0)
Thailand	Rate: Cumulative change (%)	-79.8 (-83.7 to -75.2)	-87.9 (-91.3 to -83.8)	-56.2 (-75.9 to -27.8)	-88.4 (-91.9 to -83.7)	-92.9 (-95.9 to -88.8)	-60.0 (-77.2 to -21.1)	-97.8 (-99.2 to -95.0)	-29.5 (-63.9 to 10.0)	-82.5 (-89.6 to -72.9)	-69.1 (-93.6 to -19.3)	-76.5 (-87.6 to -58.2)
Timor-Leste	Number: 1990	4,773 (4,427–5,123)	601 (472–741)	132 (78–210)	1,114 (532–1,571)	699 (305–1,064)	152 (66–376)	13 (2–32)	76 (31–140)	160 (60–303)	55 (11–148)	120 (35–234)
Timor-Leste	Number: 2015	1,690 (1,114–2,415)	315 (205–436)	132 (69–218)	304 (155–506)	112 (46–213)	171 (91–293)	1 (0–2)	75 (33–135)	90 (42–163)	9 (2–24)	50 (20–97)
Timor-Leste	Number: Cumulative change (%)	-64.5 (-77.0 to -48.8)	-46.6 (-66.1 to -21.5)	7.3 (-51.8 to 90.1)	-69.4 (-86.3 to -39.2)	-81.0 (-93.6 to -56.7)	42.9 (-48.4 to 152.2)	-83.8 (-97.9 to -47.9)	21.1 (-63.9 to 184.4)	-30.6 (-76.8 to 59.7)	-69.2 (-96.5 to -10.8)	-46.1 (-86.9 to 36.8)
Timor-Leste	Rate: 1990	3,353.8 (3,096.5–3,599.6)	389.1 (304.9–479.5)	85.8 (50.6–136.0)	785.8 (367.6–1,112.6)	496.4 (211.7–753.6)	103.2 (43.6–259.0)	10.2 (1.6–24.1)	49.4 (20.6–91.1)	104.5 (39.3–196.2)	40.4 (8.7–107.5)	85.5 (24.7–165.9)
Timor-Leste	Rate: 2015	802.0 (528.7–1,146.8)	149.4 (97.5–206.6)	62.5 (32.8–103.4)	144.6 (73.9–240.3)	53.2 (22.1–101.1)	81.2 (43.2–139.4)	0.6 (0.1–1.4)	35.7 (15.9–64.3)	42.7 (20.1–77.3)	4.5 (1.1–11.7)	23.8 (9.5–46.5)
Timor-Leste	Rate: Cumulative change (%)	-76.0 (-84.5 to -65.4)	-60.9 (-75.2 to -42.4)	-21.5 (-64.7 to 38.8)	-79.2 (-90.9 to -57.8)	-87.2 (-95.8 to -71.0)	1.3 (-64.5 to 80.1)	-89.4 (-98.6 to -65.6)	-11.6 (-73.7 to 106.6)	-49.5 (-83.0 to 17.2)	-79.7 (-97.7 to -39.7)	-63.6 (-91.1 to -8.2)
Vietnam	Number: 1990	93,125 (89,731–96,499)	18,075 (13,842–23,080)	2,153 (1,229–3,480)	17,399 (9,302–24,515)	1,633 (609–2,876)	10,291 (4,841–15,762)	700 (211–1,462)	2,819 (1,268–5,186)	3,978 (1,700–7,399)	4 (0–12)	1,443 (542–2,525)
Vietnam	Number: 2015	21,226 (18,629–24,293)	3,808 (2,668–5,178)	843 (469–1,374)	3,231 (2,568–4,003)	132 (78–207)	5,418 (3,605–7,129)	70 (29–127)	1,260 (656–2,128)	1,050 (528–1,822)	0 (0–1)	277 (131–440)
Vietnam	Number: Cumulative change (%)	-77.2 (-80.0 to -73.8)	-78.7 (-85.0 to -70.8)	-57.0 (-81.2 to -20.6)	-79.6 (-87.9 to -64.2)	-89.8 (-96.2 to -76.4)	-42.1 (-70.6 to -7.8)	-85.5 (-96.9 to -62.2)	-47.8 (-81.7 to 5.2)	-68.7 (-89.4 to -32.0)	-67.9 (-96.2 to -15.0)	-75.4 (-92.6 to -42.0)
Vietnam	Rate: 1990	995.3 (958.7–1,031.7)	191.9 (147.0–245.1)	22.9 (13.1–36.9)	185.8 (99.1–262.3)	17.5 (6.5–30.8)	109.6 (51.5–167.9)	7.5 (2.3–15.7)	30.0 (13.5–55.1)	42.3 (18.1–78.6)	0.1 (0.0–0.1)	15.5 (5.8–27.1)
Vietnam	Rate: 2015	275.7 (241.9–315.6)	49.8 (34.9–67.7)	11.0 (6.1–18.0)	41.8 (33.2–51.9)	1.7 (1.0–2.7)	70.5 (46.9–92.8)	0.9 (0.4–1.6)	16.5 (8.6–27.8)	13.7 (6.9–23.8)	0.0 (0.0–0.0)	3.6 (1.7–5.7)
Vietnam	Rate: Cumulative change (%)	-72.3 (-75.7 to -68.1)	-73.8 (-81.5 to -64.0)	-47.1 (-76.8 to -2.2)	-75.3 (-85.3 to -56.6)	-87.7 (-95.4 to -71.5)	-29.3 (-64.1 to 12.5)	-82.5 (-96.2 to -54.4)	-35.9 (-77.5 to 29.2)	-61.5 (-87.0 to -16.4)	-61.4 (-95.4 to 2.5)	-70.3 (-91.1 to -29.6)
Oceania	Number: 1990	16,276 (14,531–18,125)	1,410 (1,113–1,726)	700 (514–918)	4,337 (3,231–5,412)	1,257 (800–1,753)	598 (331–1,218)	409 (170–708)	231 (133–376)	592 (321–910)	695 (334–1,290)	443 (263–709)
Oceania	Number: 2015	11,321 (7,886–15,991)	1,244 (856–1,688)	738 (468–1,084)	2,489 (1,576–3,689)	444 (249–726)	772 (443–1,357)	154 (60–324)	314 (155–558)	671 (340–1,092)	299 (124–630)	273 (138–487)
Oceania	Number: Cumulative change (%)	-30.1 (-52.9 to 0.0)	-10.7 (-40.6 to 23.7)	8.9 (-37.8 to 72.9)	-41.3 (-64.6 to -6.9)	-62.7 (-81.3 to -32.6)	41.0 (-19.1 to 112.1)	-54.0 (-88.2 to 15.2)	45.0 (-30.1 to 168.5)	21.9 (-43.7 to 130.5)	-49.5 (-85.6 to 16.1)	-33.1 (-73.3 to 28.3)
Oceania	Rate: 1990	1,570.2 (1,400.1–1,745.5)	132.2 (104.3–161.7)	65.8 (48.3–86.2)	416.9 (309.3–520.7)	122.8 (77.9–172.1)	57.0 (31.4–116.4)	40.1 (16.7–69.8)	21.7 (12.5–35.4)	55.7 (30.4–85.4)	67.9 (32.6–126.0)	43.0 (25.5–69.0)
Oceania	Rate: 2015	816.7 (568.4–1,154.9)	89.2 (61.4–121.1)	53.0 (33.6–77.8)	179.4 (113.9–266.1)	32.2 (18.1–52.8)	55.6 (31.9–97.8)	11.2 (4.4–23.5)	22.6 (11.2–40.0)	48.2 (24.4–78.4)	21.7 (9.0–45.7)	19.7 (10.0–35.4)
Oceania	Rate: Cumulative change (%)	-47.7 (-64.8 to -25.2)	-31.7 (-54.5 to -5.4)	-16.8 (-52.4 to 32.0)	-56.0 (-73.6 to -30.2)	-72.3 (-86.1 to -50.0)	6.6 (-39.2 to 60.9)	-65.9 (-91.3 to -15.2)	10.8 (-46.6 to 104.6)	-7.0 (-57.1 to 75.7)	-62.5 (-89.4 to -14.3)	-50.1 (-80.1 to -3.8)
American Samoa	Number: 1990	32 (29–35)	6 (5–7)	3 (2–4)	3 (2–4)	0 (0–0)	3 (2–4)	0 (0–0)	0 (0–1)	1 (1–2)	0 (0–0)	0 (0–0)
American Samoa	Number: 2015	17 (14–20)	2 (1–3)	1 (1–2)	1 (1–1)	0 (0–0)	3 (2–4)	0 (0–0)	0 (0–0)	1 (0–1)	0 (0–0)	0 (0–0)
American Samoa	Number: Cumulative change (%)	-47.0 (-57.3 to -35.1)	-59.1 (-70.4 to -44.7)	-52.6 (-67.5 to -33.2)	-58.3 (-71.1 to -40.7)	-70.5 (-81.6 to -52.2)	-1.8 (-34.9 to 36.1)	nan (nan–nan)	-40.7 (-70.9 to 1.3)	-32.9 (-60.4 to 4.5)	-65.9 (-81.7 to -42.1)	-67.8 (-83.1 to -37.4)
American Samoa	Rate: 1990	377.1 (342.4–410.5)	68.7 (56.7–81.4)	41.1 (32.0–51.6)	43.9 (31.7–53.9)	6.2 (3.9–8.3)	37.2 (28.9–46.3)	0.0 (0.0–0.0)	8.1 (4.6–12.2)	21.8 (15.6–28.6)	7.6 (4.5–10.8)	7.6 (4.5–10.4)
American Samoa	Rate: 2015	145.1 (120.8–174.7)	20.7 (15.8–26.6)	14.2 (10.5–18.6)	12.9 (10.0–16.3)	1.2 (0.9–1.7)	26.1 (20.0–33.8)	0.0 (0.0–0.0)	3.3 (2.0–5.5)	10.4 (6.8–14.2)	1.7 (1.1–2.5)	1.6 (1.1–2.2)
American Samoa	Rate: Cumulative change (%)	-61.4 (-68.8 to -52.7)	-69.5 (-78.0 to -58.8)	-64.7 (-75.8 to -50.5)	-69.8 (-79.1 to -56.7)	-78.8 (-86.8 to -65.3)	-28.0 (-52.2 to -0.1)	nan (nan–nan)	-56.0 (-78.4 to -24.9)	-50.3 (-70.6 to -22.9)	-75.7 (-87.0 to -59.1)	-76.8 (-87.8 to -54.8)

© 2017 Global Burden of Disease Child and Adolescent Health Collaboration. JAMA Pediatrics.

eTable 2. Number of Deaths, Death Rates (per 100,000 population), and Cumulative Percent Change with 95% Uncertainty Intervals (UI) for the Top 10 Global Causes of Death in 195 Countries and Territories, Aged Under 5 Years, Both Sexes, 1990 and 2015 *Note: best viewed by enlarging in browser.*

Location	Measure	All causes	Neonatal preterm birth complications	Neonatal encephalopathy due to birth asphyxia and trauma	Lower respiratory infections	Diarrheal diseases	Congenital anomalies	Malaria	Neonatal sepsis and other neonatal infections	Other neonatal disorders	Protein-energy malnutrition	Meningitis
Federated States of Micronesia	Number: 1990	133 (108–162)	24 (18–30)	10 (6–15)	25 (18–34)	4 (2–7)	8 (5–14)	0 (0–0)	3 (1–6)	8 (3–13)	5 (2–12)	4 (2–7)
Federated States of Micronesia	Number: 2015	28 (20–39)	4 (2–6)	3 (1–5)	3 (2–5)	0 (0–0)	4 (2–5)	0 (0–0)	1 (0–2)	2 (0–3)	0 (0–1)	0 (0–1)
Federated States of Micronesia	Number: Cumulative change (%)	-78.5 (-85.8 to -69.2)	-81.4 (-89.5 to -70.5)	-69.8 (-84.9 to -47.1)	-85.8 (-91.8 to -77.4)	-91.8 (-96.3 to -84.1)	-49.5 (-77.0 to -16.0)	nan (nan–nan)	-58.4 (-85.3 to -12.3)	-71.0 (-90.7 to -34.8)	-84.6 (-96.5 to -61.5)	-84.7 (-95.1 to -67.9)
Federated States of Micronesia	Rate: 1990	847.2 (685.8–1,027.9)	152.2 (115.3–192.8)	68.0 (43.3–100.8)	163.9 (116.1–220.1)	30.6 (17.1–48.1)	56.2 (34.4–92.8)	0.0 (0.0–0.0)	21.7 (10.0–39.6)	50.9 (24.9–84.8)	38.0 (15.2–80.6)	26.7 (13.9–47.6)
Federated States of Micronesia	Rate: 2015	235.1 (167.5–325.3)	36.9 (22.0–56.5)	25.9 (14.2–43.3)	29.2 (18.3–42.8)	3.0 (1.6–5.3)	33.9 (21.8–48.2)	0.0 (0.0–0.0)	10.4 (4.9–19.7)	17.3 (6.4–33.1)	6.1 (2.0–14.0)	4.6 (2.0–8.8)
Federated States of Micronesia	Rate: Cumulative change (%)	-71.8 (-81.4 to -59.6)	-75.3 (-86.1 to -60.9)	-59.9 (-79.9 to -30.0)	-81.5 (-89.3 to -70.4)	-89.3 (-95.1 to -79.4)	-33.7 (-69.8 to 10.5)	nan (nan–nan)	-45.0 (-80.5 to 16.0)	-61.6 (-87.7 to -13.6)	-80.0 (-95.5 to -49.8)	-80.0 (-93.6 to -58.0)
Fiji	Number: 1990	573 (429–748)	93 (66–126)	71 (51–94)	117 (76–167)	22 (13–33)	50 (34–71)	0 (0–0)	23 (15–35)	25 (16–36)	7 (4–11)	14 (9–21)
Fiji	Number: 2015	537 (348–802)	94 (65–129)	55 (35–78)	94 (54–152)	26 (14–44)	77 (41–125)	0 (0–0)	22 (13–36)	20 (12–29)	5 (3–9)	13 (7–21)
Fiji	Number: Cumulative change (%)	-3.4 (-44.9 to 59.5)	4.8 (-39.1 to 65.3)	-19.5 (-53.6 to 27.3)	-14.9 (-59.3 to 53.4)	27.3 (-41.3 to 140.5)	63.8 (-28.3 to 210.2)	nan (nan–nan)	-1.2 (-46.6 to 60.1)	-14.5 (-57.4 to 53.1)	-15.5 (-63.5 to 62.8)	-2.1 (-54.8 to 79.8)
Fiji	Rate: 1990	553.4 (414.1–721.5)	91.0 (64.7–123.0)	69.1 (50.3–92.0)	113.3 (74.1–160.7)	21.4 (13.2–31.9)	48.7 (33.4–69.2)	0.0 (0.0–0.0)	23.1 (15.0–34.2)	24.5 (15.7–35.4)	7.2 (4.0–11.1)	14.0 (8.9–20.9)
Fiji	Rate: 2015	628.1 (406.9–937.6)	111.4 (77.2–152.3)	65.3 (42.4–92.5)	110.2 (63.8–177.5)	30.5 (17.3–51.4)	90.4 (48.7–147.1)	0.0 (0.0–0.0)	26.7 (15.6–42.8)	23.8 (14.8–35.3)	6.7 (3.5–11.1)	15.5 (8.7–25.1)
Fiji	Rate: Cumulative change (%)	17.1 (-33.2 to 93.2)	27.4 (-26.0 to 100.8)	-2.2 (-43.7 to 54.6)	3.2 (-50.8 to 85.8)	53.9 (-28.9 to 191.5)	98.4 (-13.1 to 275.7)	nan (nan–nan)	20.1 (-35.1 to 94.5)	3.8 (-48.2 to 85.8)	2.1 (-56.0 to 96.5)	18.5 (-45.4 to 117.2)
Guam	Number: 1990	44 (41–48)	7 (6–8)	4 (3–5)	5 (4–6)	0 (0–0)	7 (6–9)	0 (0–0)	1 (0–2)	2 (1–2)	0 (0–1)	0 (0–1)
Guam	Number: 2015	50 (41–62)	8 (5–11)	3 (2–5)	5 (4–6)	0 (0–0)	10 (7–13)	0 (0–0)	2 (1–3)	5 (3–7)	0 (0–0)	0 (0–0)
Guam	Number: Cumulative change (%)	13.3 (-9.4 to 40.5)	17.6 (-24.1 to 68.1)	-7.4 (-40.1 to 31.9)	1.3 (-28.1 to 37.7)	-44.6 (-63.3 to -20.7)	31.5 (-10.9 to 80.4)	nan (nan–nan)	64.2 (-4.6 to 148.8)	131.5 (43.4–258.6)	-32.2 (-57.7 to 2.3)	-38.5 (-68.1 to 33.2)
Guam	Rate: 1990	276.3 (253.2–301.4)	46.2 (38.3–54.2)	26.0 (20.5–32.0)	33.5 (27.7–40.1)	2.1 (1.6–2.6)	47.7 (39.5–56.9)	0.0 (0.0–0.0)	9.1 (6.1–15.5)	13.9 (10.5–18.2)	6.0 (4.1–8.0)	5.5 (3.0–7.5)
Guam	Rate: 2015	359.1 (290.9–443.8)	61.6 (42.6–83.9)	27.1 (19.2–36.1)	38.5 (29.4–49.2)	1.3 (0.9–1.7)	71.2 (50.1–93.2)	0.0 (0.0–0.0)	16.2 (10.4–23.2)	36.1 (25.0–51.1)	4.5 (3.1–6.3)	3.4 (2.4–5.0)
Guam	Rate: Cumulative change (%)	30.3 (4.2–61.7)	35.2 (-12.7 to 93.3)	6.5 (-31.1 to 51.8)	16.5 (-17.3 to 58.4)	-36.4 (-57.8 to -8.9)	51.3 (2.5–107.6)	nan (nan–nan)	89.0 (9.7–186.3)	166.4 (65.1–312.7)	-22.1 (-51.5 to 17.5)	-29.3 (-63.3 to 53.2)
Kiribati	Number: 1990	218 (181–258)	29 (22–37)	18 (12–25)	28 (19–37)	38 (26–51)	2 (1–3)	0 (0–0)	5 (2–8)	10 (4–17)	15 (8–26)	14 (8–23)
Kiribati	Number: 2015	124 (74–197)	19 (11–28)	15 (8–24)	16 (8–28)	7 (3–13)	3 (1–5)	0 (0–0)	7 (3–13)	10 (4–19)	9 (3–18)	5 (2–11)
Kiribati	Number: Cumulative change (%)	-42.6 (-66.9 to -8.2)	-34.7 (-62.8 to 3.1)	-12.6 (-58.8 to 53.9)	-41.0 (-72.5 to 6.7)	-80.0 (-91.1 to -60.2)	20.4 (-44.2 to 114.1)	nan (nan–nan)	68.8 (-36.2 to 274.9)	20.0 (-62.7 to 162.1)	-31.8 (-78.5 to 61.4)	-56.5 (-86.1 to -3.9)
Kiribati	Rate: 1990	1,796.9 (1,493.0–2,126.7)	236.6 (182.3–294.3)	148.2 (101.2–206.4)	234.2 (163.7–307.2)	316.6 (216.7–426.4)	21.6 (11.5–32.2)	0.0 (0.0–0.0)	40.8 (21.0–71.2)	80.8 (38.9–140.3)	132.4 (68.8–219.8)	123.8 (71.8–196.2)
Kiribati	Rate: 2015	815.5 (487.7–1,294.8)	123.7 (73.4–186.5)	100.4 (54.4–161.2)	106.7 (53.6–189.7)	48.2 (22.6–88.9)	19.9 (9.7–33.6)	0.0 (0.0–0.0)	49.4 (22.0–89.8)	67.7 (28.5–126.3)	63.5 (23.0–124.1)	38.5 (15.2–75.9)
Kiribati	Rate: Cumulative change (%)	-54.2 (-73.6 to -26.7)	-46.8 (-69.6 to -16.1)	-29.0 (-66.6 to 24.7)	-52.9 (-78.1 to -14.5)	-84.1 (-92.9 to -68.1)	-3.0 (-55.0 to 73.1)	nan (nan–nan)	37.2 (-48.0 to 204.8)	-2.6 (-69.7 to 113.8)	-46.0 (-82.9 to 28.6)	-65.4 (-88.9 to -23.5)
Marshall Islands	Number: 1990	80 (60–100)	14 (10–18)	5 (3–8)	15 (10–21)	4 (2–6)	4 (2–8)	0 (0–0)	1 (0–3)	4 (2–7)	3 (1–7)	2 (1–4)
Marshall Islands	Number: 2015	35 (21–53)	6 (3–10)	3 (1–5)	5 (2–8)	0 (0–1)	3 (2–5)	0 (0–0)	1 (0–2)	2 (0–4)	0 (0–2)	0 (0–1)
Marshall Islands	Number: Cumulative change (%)	-55.3 (-73.9 to -30.0)	-54.4 (-77.2 to -23.7)	-34.0 (-71.2 to 25.5)	-66.7 (-82.8 to -42.1)	-82.1 (-92.4 to -66.2)	-12.0 (-59.9 to 56.2)	nan (nan–nan)	-10.6 (-71.7 to 103.8)	-38.0 (-81.4 to 34.1)	-69.5 (-93.1 to -23.2)	-68.4 (-90.4 to -33.6)
Marshall Islands	Rate: 1990	902.4 (685.4–1,131.3)	157.7 (115.8–202.7)	62.4 (35.9–93.9)	178.2 (121.6–244.0)	45.8 (25.0–75.2)	52.4 (29.5–99.4)	0.0 (0.0–0.0)	19.3 (8.9–34.9)	51.1 (24.5–86.1)	42.8 (16.0–83.9)	28.5 (14.6–50.3)
Marshall Islands	Rate: 2015	385.7 (233.5–586.0)	70.8 (35.9–113.1)	38.6 (18.3–67.0)	55.5 (32.4–89.8)	7.1 (3.7–12.6)	40.8 (22.3–66.0)	0.0 (0.0–0.0)	14.8 (5.9–28.4)	27.8 (10.5–53.4)	9.9 (3.2–21.8)	7.6 (3.3–14.0)
Marshall Islands	Rate: Cumulative change (%)	-56.3 (-74.6 to -31.4)	-54.2 (-77.0 to -23.4)	-33.9 (-71.2 to 25.6)	-67.5 (-83.2 to -43.7)	-82.9 (-92.8 to -67.1)	-13.5 (-60.6 to 53.3)	nan (nan–nan)	-10.7 (-71.6 to 102.8)	-38.0 (-81.4 to 34.4)	-71.1 (-93.4 to -27.5)	-69.4 (-90.8 to -35.7)
Northern Mariana Islands	Number: 1990	15 (8–24)	2 (1–4)	1 (0–1)	1 (1–3)	0 (0–0)	1 (0–2)	0 (0–0)	0 (0–0)	1 (0–2)	0 (0–0)	0 (0–0)
Northern Mariana Islands	Number: 2015	9 (6–15)	1 (0–2)	0 (0–1)	0 (0–1)	0 (0–0)	2 (1–3)	0 (0–0)	0 (0–0)	0 (0–1)	0 (0–0)	0 (0–0)
Northern Mariana Islands	Number: Cumulative change (%)	-28.9 (-67.7 to 26.9)	-44.8 (-79.1 to 12.7)	-31.0 (-74.1 to 47.0)	-47.8 (-78.5 to 1.0)	-42.7 (-78.0 to 17.9)	53.1 (-40.2 to 196.3)	nan (nan–nan)	9.9 (-74.0 to 150.2)	-26.5 (-79.3 to 66.3)	-43.9 (-84.6 to 25.0)	-50.5 (-84.1 to 10.4)
Northern Mariana Islands	Rate: 1990	363.9 (209.7–567.6)	67.7 (33.5–111.6)	26.4 (12.5–44.3)	47.1 (25.3–77.5)	2.7 (1.3–4.5)	36.8 (19.5–60.5)	0.0 (0.0–0.0)	8.1 (3.0–16.5)	24.6 (9.8–47.6)	9.1 (3.9–17.8)	2.9 (1.4–5.4)
Northern Mariana Islands	Rate: 2015	122.1 (74.9–185.3)	17.5 (9.1–28.5)	8.4 (4.4–14.3)	11.3 (6.7–18.1)	0.7 (0.4–1.1)	25.9 (15.2–40.4)	0.0 (0.0–0.0)	3.7 (1.3–7.8)	7.6 (3.7–13.6)	2.1 (0.9–4.0)	0.6 (0.3–1.0)
Northern Mariana Islands	Rate: Cumulative change (%)	-63.1 (-83.1 to -34.3)	-70.5 (-88.9 to -39.8)	-63.2 (-86.2 to -21.6)	-72.9 (-88.9 to -48.0)	-70.8 (-88.8 to -39.4)	-19.7 (-68.5 to 55.7)	nan (nan–nan)	-41.5 (-86.1 to 33.4)	-60.9 (-89.0 to -11.5)	-71.5 (-92.3 to -36.1)	-74.5 (-91.9 to -42.3)
Papua New Guinea	Number: 1990	13,126 (11,442–14,832)	981 (713–1,272)	477 (309–678)	3,686 (2,696–4,694)	1,047 (626–1,513)	426 (198–964)	361 (144–633)	157 (74–282)	450 (215–738)	577 (246–1,128)	348 (182–590)
Papua New Guinea	Number: 2015	9,475 (6,181–14,041)	948 (578–1,368)	569 (321–889)	2,171 (1,304–3,312)	373 (192–648)	578 (284–1,136)	141 (51–303)	244 (103–466)	561 (248–963)	252 (87–567)	226 (99–436)
Papua New Guinea	Number: Cumulative change (%)	-27.3 (-54.5 to 8.3)	-1.2 (-41.6 to 45.5)	26.6 (-39.0 to 124.7)	-39.5 (-66.0 to -0.6)	-61.8 (-83.2 to -26.5)	53.3 (-23.2 to 150.1)	-51.1 (-88.4 to 26.0)	74.9 (-35.0 to 263.6)	39.7 (-46.0 to 180.6)	-46.5 (-87.8 to 33.9)	-26.8 (-76.2 to 55.5)
Papua New Guinea	Rate: 1990	1,958.7 (1,709.9–2,217.8)	140.3 (102.1–181.9)	68.3 (44.3–97.1)	546.5 (397.4–697.7)	158.9 (95.1–230.0)	62.5 (28.8–142.1)	54.9 (21.8–97.2)	22.7 (10.6–40.4)	64.7 (30.9–105.5)	87.7 (37.7–171.9)	52.4 (27.3–89.8)
Papua New Guinea	Rate: 2015	925.8 (604.1–1,372.8)	91.9 (56.1–132.6)	55.1 (31.1–86.2)	212.0 (127.6–323.5)	36.7 (19.0–63.7)	56.3 (27.7–110.7)	14.0 (5.1–29.9)	23.7 (10.0–45.2)	54.5 (24.1–93.5)	24.8 (8.6–55.9)	22.2 (9.7–42.8)
Papua New Guinea	Rate: Cumulative change (%)	-52.4 (-70.3 to -28.9)	-33.1 (-60.4 to -1.5)	-14.3 (-58.7 to 51.8)	-60.1 (-77.6 to -33.8)	-75.2 (-89.1 to -52.2)	2.2 (-49.1 to 66.9)	-68.3 (-92.5 to -18.5)	18.2 (-56.0 to 146.7)	-5.7 (-63.4 to 89.6)	-65.4 (-92.1 to -12.9)	-52.2 (-84.5 to 1.5)
Samoa	Number: 1990	111 (68–175)	19 (9–31)	10 (4–19)	16 (8–27)	2 (0–4)	10 (5–16)	0 (0–0)	3 (1–7)	7 (2–14)	3 (1–8)	2 (1–5)
Samoa	Number: 2015	42 (23–71)	5 (2–9)	4 (1–8)	4 (2–7)	0 (0–0)	7 (3–12)	0 (0–0)	1 (0–3)	2 (0–5)	0 (0–2)	0 (0–1)
Samoa	Number: Cumulative change (%)	-59.6 (-80.2 to -28.6)	-71.1 (-87.2 to -42.2)	-54.4 (-82.7 to -5.2)	-70.8 (-87.6 to -44.3)	-85.3 (-95.2 to -68.8)	-24.1 (-68.1 to 45.7)	nan (nan–nan)	-39.1 (-82.2 to 37.7)	-54.5 (-86.5 to 5.1)	-68.5 (-94.6 to -7.8)	-70.7 (-92.1 to -35.4)
Samoa	Rate: 1990	420.3 (257.2–661.0)	72.8 (37.2–121.6)	40.2 (18.1–72.5)	60.9 (31.8–101.8)	8.0 (3.0–16.0)	38.6 (20.3–62.5)	0.0 (0.0–0.0)	14.1 (5.6–28.3)	27.2 (9.6–55.9)	13.4 (4.2–30.7)	10.6 (4.3–21.4)
Samoa	Rate: 2015	176.6 (98.0–297.7)	21.8 (9.7–41.0)	18.2 (7.4–34.5)	17.9 (8.6–32.0)	1.1 (0.4–2.6)	29.5 (14.0–53.3)	0.0 (0.0–0.0)	8.0 (3.1–16.3)	11.8 (3.1–25.2)	3.4 (0.9–8.7)	2.8 (1.0–6.0)
Samoa	Rate: Cumulative change (%)	-55.3 (-78.1 to -20.9)	-67.8 (-85.7 to -35.4)	-49.1 (-80.7 to 6.1)	-67.8 (-86.3 to -38.3)	-83.9 (-94.7 to -65.8)	-15.8 (-64.6 to 61.8)	nan (nan–nan)	-32.1 (-80.3 to 53.4)	-49.2 (-85.1 to 17.3)	-65.6 (-94.0 to 0.8)	-67.7 (-91.3 to -29.1)
Solomon Islands	Number: 1990	553 (444–673)	85 (56–119)	33 (19–51)	97 (62–134)	39 (20–62)	24 (13–46)	15 (4–31)	8 (3–16)	24 (10–43)	21 (7–44)	14 (6–25)
Solomon Islands	Number: 2015	338 (244–462)	61 (34–94)	33 (17–52)	51 (34–75)	12 (6–20)	32 (20–48)	4 (1–10)	10 (4–19)	21 (7–39)	10 (3–22)	7 (3–12)
Solomon Islands	Number: Cumulative change (%)	-37.8 (-58.5 to -11.4)	-25.9 (-57.6 to 11.2)	4.8 (-51.0 to 82.9)	-44.3 (-68.0 to -7.7)	-65.9 (-83.3 to -34.7)	48.9 (-23.4 to 142.3)	-63.9 (-90.4 to -11.9)	38.6 (-51.9 to 199.6)	0.9 (-64.6 to 116.3)	-37.0 (-85.6 to 56.9)	-40.9 (-82.3 to 30.6)
Solomon Islands	Rate: 1990	937.4 (753.1–1,140.7)	142.3 (94.2–198.7)	56.4 (32.9–86.1)	165.1 (106.3–228.2)	67.3 (34.8–106.3)	41.4 (23.0–78.5)	26.4 (8.5–55.1)	14.5 (6.6–26.9)	40.1 (16.8–73.0)	36.5 (13.2–76.2)	24.0 (11.0–42.8)
Solomon Islands	Rate: 2015	406.6 (294.0–555.4)	74.9 (42.2–114.6)	40.1 (21.1–63.2)	61.7 (41.2–90.8)	14.6 (8.2–24.1)	39.7 (25.1–58.9)	5.4 (1.7–12.3)	12.6 (5.5–23.3)	25.4 (9.5–48.1)	12.1 (4.3–26.2)	8.4 (3.9–15.5)
Solomon Islands	Rate: Cumulative change (%)	-55.9 (-70.6 to -37.1)	-46.1 (-69.2 to -19.0)	-23.9 (-64.4 to 33.1)	-60.5 (-77.3 to -34.6)	-76.1 (-88.5 to -54.3)	6.5 (-45.3 to 73.2)	-74.9 (-93.3 to -39.0)	0.4 (-65.0 to 116.0)	-26.9 (-74.4 to 57.0)	-56.1 (-90.0 to 8.9)	-58.4 (-87.5 to -7.6)
Tonga	Number: 1990	78 (55–108)	15 (9–22)	2 (1–4)	9 (5–14)	1 (0–2)	6 (3–9)	0 (0–0)	4 (1–8)	6 (3–11)	2 (0–5)	5 (2–9)
Tonga	Number: 2015	46 (29–70)	8 (4–14)	1 (0–3)	4 (2–7)	0 (0–0)	5 (3–8)	0 (0–0)	3 (1–6)	4 (2–8)	1 (0–3)	2 (1–5)
Tonga	Number: Cumulative change (%)	-38.6 (-63.1 to -2.2)	-40.8 (-71.2 to 3.6)	-25.6 (-72.0 to 54.8)	-45.4 (-71.1 to -6.2)	-59.3 (-82.2 to -22.8)	-11.6 (-52.5 to 52.1)	nan (nan–nan)	-11.3 (-70.1 to 87.5)	-19.8 (-71.8 to 67.9)	-32.9 (-84.3 to 79.3)	-36.5 (-74.4 to 19.6)
Tonga	Rate: 1990	543.3 (380.8–749.8)	109.5 (68.6–156.0)	18.6 (9.3–31.4)	64.4 (39.4–100.5)	9.3 (4.9–15.6)	44.3 (27.7–65.9)	0.0 (0.0–0.0)	31.2 (13.9–57.4)	47.3 (22.3–82.3)	18.2 (6.4–39.9)	36.2 (17.4–63.7)
Tonga	Rate: 2015	364.6 (233.4–549.1)	70.2 (36.7–114.6)	14.0 (6.5–24.6)	37.0 (22.7–57.3)	3.8 (2.1–6.2)	41.7 (24.8–66.4)	0.0 (0.0–0.0)	27.3 (11.4–51.3)	38.1 (16.4–70.4)	10.5 (3.9–23.0)	23.0 (11.6–40.8)
Tonga	Rate: Cumulative change (%)	-30.6 (-58.4 to 10.6)	-32.7 (-67.3 to 17.8)	-15.6 (-68.3 to 75.6)	-38.6 (-67.5 to 5.8)	-54.5 (-80.2 to -13.1)	-0.2 (-46.3 to 71.8)	nan (nan–nan)	0.7 (-66.2 to 112.6)	-9.0 (-67.9 to 90.0)	-24.9 (-82.5 to 101.8)	-28.7 (-71.5 to 34.4)
Vanuatu	Number: 1990	205 (167–245)	35 (25–47)	14 (8–21)	36 (21–51)	12 (5–19)	10 (5–19)	5 (1–11)	4 (2–7)	10 (5–19)	7 (2–15)	5 (2–9)
Vanuatu	Number: 2015	185 (135–252)	34 (23–47)	17 (9–27)	34 (22–50)	6 (3–10)	16 (9–25)	2 (0–4)	6 (2–12)	12 (6–21)	5 (2–12)	4 (1–7)
Vanuatu	Number: Cumulative change (%)	-8.5 (-36.4 to 26.9)	-1.5 (-40.4 to 44.4)	31.6 (-36.1 to 128.8)	4.5 (-43.2 to 82.0)	-39.2 (-73.6 to 18.6)	67.7 (-9.1 to 174.8)	-41.7 (-88.4 to 53.7)	72.9 (-39.5 to 287.4)	32.0 (-49.6 to 172.8)	8.0 (-77.5 to 188.6)	0.3 (-69.6 to 130.9)
Vanuatu	Rate: 1990	813.6 (661.4–972.3)	140.4 (99.0–185.3)	55.7 (33.0–83.7)	143.0 (85.0–202.2)	48.0 (23.6–76.4)	41.3 (22.9–75.1)	20.2 (5.4–45.0)	17.3 (8.1–31.0)	42.8 (20.3–75.4)	30.2 (10.6–62.9)	20.2 (8.7–38.5)
Vanuatu	Rate: 2015	558.5 (406.2–758.7)	103.4 (69.5–143.6)	52.6 (29.5–82.3)	104.8 (68.5–151.8)	19.3 (10.8–31.3)	48.7 (29.0–77.6)	6.4 (1.8–15.0)	19.7 (8.5–37.3)	37.6 (18.3–63.9)	17.4 (6.2–36.7)	12.5 (5.5–22.9)
Vanuatu	Rate: Cumulative change (%)	-30.5 (-51.6 to -3.6)	-24.4 (-54.3 to 10.8)	0.9 (-50.9 to 75.5)	-20.5 (-56.9 to 38.5)	-53.9 (-80.1 to -9.5)	28.0 (-30.6 to 109.7)	-55.8 (-91.2 to 15.8)	32.5 (-54.2 to 197.0)	1.1 (-61.3 to 109.2)	-18.3 (-82.9 to 117.6)	-23.8 (-76.9 to 76.5)
North Africa and Middle East	Number: 1990	892,340 (873,289–913,238)	153,951 (140,401–171,700)	28,635 (23,501–34,111)	159,782 (136,529–183,336)	119,436 (101,982–137,136)	99,843 (64,918–166,907)	4,056 (1,709–7,005)	17,328 (11,731–22,308)	37,480 (28,604–47,079)	10,375 (6,406–15,815)	21,073 (13,230–28,900)
North Africa and Middle East	Number: 2015	404,011 (378,251–431,885)	83,998 (75,004–93,033)	19,930 (16,625–23,789)	49,979 (42,624–57,949)	23,676 (18,861–29,430)	76,064 (64,291–93,406)	3,421 (1,359–6,541)	17,737 (10,289–22,632)	19,646 (15,164–24,838)	2,472 (1,684–3,576)	9,519 (7,032–13,335)
North Africa and Middle East	Number: Cumulative change (%)	-54.7 (-57.7 to -51.4)	-45.3 (-51.5 to -39.0)	-29.7 (-43.8 to -12.2)	-68.6 (-73.4 to -62.8)	-80.1 (-84.4 to -75.3)	-20.0 (-42.9 to 10.7)	-9.7 (-57.9 to 70.5)	3.4 (-21.7 to 36.9)	-46.7 (-61.8 to -28.7)	-74.8 (-85.5 to -60.7)	-52.9 (-69.3 to -30.1)
North Africa and Middle East	Rate: 1990	1,672.9 (1,637.0–1,712.5)	285.0 (260.0–318.0)	53.0 (43.5–63.1)	300.6 (256.8–345.0)	224.8 (191.9–258.2)	186.5 (121.2–312.1)	7.7 (3.2–13.3)	32.2 (21.8–41.4)	69.5 (53.1–87.4)	19.6 (12.1–29.8)	39.7 (24.9–54.5)
North Africa and Middle East	Rate: 2015	625.8 (586.0–669.1)	130.0 (116.1–144.0)	30.8 (25.7–36.8)	77.4 (66.0–89.8)	36.6 (29.2–45.5)	117.7 (99.5–144.6)	5.3 (2.1–10.1)	27.5 (15.9–35.0)	30.4 (23.5–38.4)	3.8 (2.6–5.6)	14.8 (10.9–20.6)
North Africa and Middle East	Rate: Cumulative change (%)	-62.6 (-65.0 to -59.8)	-54.3 (-59.5 to -49.0)	-41.2 (-53.0 to -26.6)	-74.1 (-78.1 to -69.4)	-83.6 (-87.1 to -79.7)	-33.7 (-52.7 to -8.2)	-25.9 (-65.5 to 40.0)	-13.8 (-34.8 to 14.1)	-55.5 (-68.1 to -40.5)	-79.3 (-88.1 to -67.8)	-61.3 (-74.8 to -42.6)
North Africa and Middle East	Number: 1990	892,340 (873,289–913,238)	153,951 (140,401–171,700)	28,635 (23,501–34,111)	159,782 (136,529–183,336)	119,436 (101,982–137,136)	99,843 (64,918–166,907)	4,056 (1,709–7,005)	17,328 (11,731–22,308)	37,480 (28,604–47,079)	10,375 (6,406–15,815)	21,073 (13,230–28,900)
North Africa and Middle East	Number: 2015	404,011 (378,251–431,885)	83,998 (75,004–93,033)	19,930 (16,625–23,789)	49,979 (42,624–57,949)	23,676 (18,861–29,430)	76,064 (64,291–93,406)	3,421 (1,359–6,541)	17,737 (10,289–22,632)	19,646 (15,164–24,838)	2,472 (1,684–3,576)	9,519 (7,032–13,335)
North Africa and Middle East	Number: Cumulative change (%)	-54.7 (-57.7 to -51.4)	-45.3 (-51.5 to -39.0)	-29.7 (-43.8 to -12.2)	-68.6 (-73.4 to -62.8)	-80.1 (-84.4 to -75.3)	-20.0 (-42.9 to 10.7)	-9.7 (-57.9 to 70.5)	3.4 (-21.7 to 36.9)	-46.7 (-61.8 to -28.7)	-74.8 (-85.5 to -60.7)	-52.9 (-69.3 to -30.1)
North Africa and Middle East	Rate: 1990	1,672.9 (1,637.0–1,712.5)	285.0 (260.0–318.0)	53.0 (43.5–63.1)	300.6 (256.8–345.0)	224.8 (191.9–258.2)	186.5 (121.2–312.1)	7.7 (3.2–13.3)	32.2 (21.8–41.4)	69.5 (53.1–87.4)	19.6 (12.1–29.8)	39.7 (24.9–54.5)
North Africa and Middle East	Rate: 2015	625.8 (586.0–669.1)	130.0 (116.1–144.0)	30.8 (25.7–36.8)	77.4 (66.0–89.8)	36.6 (29.2–45.5)	117.7 (99.5–144.6)	5.3 (2.1–10.1)	27.5 (15.9–35.0)	30.4 (23.5–38.4)	3.8 (2.6–5.6)	14.8 (10.9–20.6)
North Africa and Middle East	Rate: Cumulative change (%)	-62.6 (-65.0 to -59.8)	-54.3 (-59.5 to -49.0)	-41.2 (-53.0 to -26.6)	-74.1 (-78.1 to -69.4)	-83.6 (-87.1 to -79.7)	-33.7 (-52.7 to -8.2)	-25.9 (-65.5 to 40.0)	-13.8 (-34.8 to 14.1)	-55.5 (-68.1 to -40.5)	-79.3 (-88.1 to -67.8)	-61.3 (-74.8 to -42.6)
Afghanistan	Number: 1990	98,130 (92,145–104,039)	7,548 (4,789–11,141)	3,342 (1,812–5,036)	21,295 (10,468–29,355)	9,992 (4,425–15,229)	6,642 (2,606–15,336)	1,844 (248–4,340)	925 (376–1,764)	4,132 (1,557–7,378)	752 (210–1,658)	4,902 (2,102–8,402)
Afghanistan	Number: 2015	89,268 (77,273–101,946)	10,547 (7,409–14,335)	5,200 (3,262–7,661)	19,116 (13,859–24,649)	6,137 (3,870–8,963)	9,988 (5,348–19,640)	1,825 (257–4,471)	2,308 (1,104–4,105)	4,683 (1,919–8,012)	602 (233–1,316)	5,261 (3,000–8,672)
Afghanistan	Number: Cumulative change (%)	-8.9 (-22.2 to 5.5)	46.4 (-6.0 to 122.5)	68.1 (-11.7 to 185.0)	-1.4 (-39.4 to 75.4)	-29.1 (-66.2 to 36.0)	73.8 (5.3–164.4)	35.9 (-70.3 to 241.6)	200.7 (13.3–560.8)	26.9 (-44.0 to 136.7)	18.0 (-75.2 to 221.0)	28.6 (-49.4 to 173.3)
Afghanistan	Rate: 1990	3,577.1 (3,364.3–3,790.8)	255.2 (161.5–377.5)	113.3 (61.7–169.7)	781.6 (371.7–1,079.1)	367.9 (159.4–561.7)	235.2 (90.6–545.3)	68.7 (9.3–163.3)	31.4 (12.8–59.8)	140.5 (53.1–250.1)	28.0 (7.8–61.8)	181.6 (76.8–312.1)
Afghanistan	Rate: 2015	1,755.0 (1,518.2–2,005.5)	204.5 (144.0–278.1)	100.9 (63.3–148.8)	377.2 (273.3–486.4)	121.3 (76.4–177.1)	195.9 (104.7–385.6)	36.1 (5.1–88.4)	44.9 (21.5–80.0)	91.0 (37.3–155.7)	11.9 (4.6–26.2)	104.0 (59.2–171.5)
Afghanistan	Rate: Cumulative change (%)	-50.9 (-58.1 to -43.1)	-16.0 (-45.9 to 27.6)	-3.8 (-49.1 to 63.1)	-46.7 (-67.7 to -3.7)	-61.8 (-81.8 to -25.7)	-2.8 (-42.6 to 50.1)	-27.3 (-84.7 to 83.6)	72.1 (-34.9 to 279.0)	-27.6 (-67.8 to 34.5)	-37.2 (-86.8 to 73.1)	-31.0 (-73.1 to 47.3)
Algeria	Number: 1990	45,620 (42,273–49,196)	11,081 (9,017–13,155)	2,745 (1,651–4,058)	3,535 (2,517–4,839)	3,828 (2,506–5,327)	5,531 (3,938–8,060)	0 (0–0)	2,790 (1,497–4,520)	2,043 (971–3,560)	1,728 (708–3,506)	1,197 (640–2,140)
Algeria	Number: 2015	21,305 (17,439–26,064)	5,561 (4,106–7,233)	1,826 (1,103–2,712)	949 (659–1,306)	601 (370–909)	4,140 (3,174–5,444)	0 (0–0)	2,103 (1,156–3,324)	1,202 (580–2,074)	427 (171–901)	395 (229–669)
Algeria	Number: Cumulative change (%)	-53.2 (-62.0 to -42.5)	-49.2 (-63.5 to -31.2)	-28.8 (-62.5 to 22.0)	-72.0 (-83.5 to -56.3)	-83.4 (-91.4 to -71.6)	-21.8 (-50.5 to 11.3)	nan (nan–nan)	-17.8 (-59.7 to 56.9)	-31.0 (-77.0 to 47.9)	-69.3 (-91.8 to -26.5)	-62.5 (-85.5 to -23.9)
Algeria	Rate: 1990	1,137.9 (1,054.2–1,227.6)	277.3 (225.6–329.2)	68.7 (41.3–101.5)	88.0 (62.6–120.4)	95.5 (62.5–132.9)	138.2 (98.4–201.3)	0.0 (0.0–0.0)	69.9 (37.4–113.2)	51.1 (24.3–89.1)	43.1 (17.7–87.3)	29.8 (15.9–53.3)
Algeria	Rate: 2015	469.6 (384.4–574.5)	123.0 (90.9–159.9)	40.4 (24.4–60.0)	20.8 (14.5–28.7)	13.2 (8.1–20.0)	91.1 (69.9–119.8)	0.0 (0.0–0.0)	46.5 (25.5–73.5)	26.5 (12.8–45.9)	9.4 (3.7–19.8)	8.7 (5.0–14.7)
Algeria	Rate: Cumulative change (%)	-58.7 (-66.5 to -49.2)	-55.1 (-67.7 to -39.3)	-37.1 (-66.9 to 8.0)	-75.3 (-85.4 to -61.5)	-85.4 (-92.4 to -75.1)	-31.1 (-56.4 to -2.0)	nan (nan–nan)	-27.5 (-64.4 to 38.5)	-39.1 (-79.7 to 30.6)	-73.0 (-92.7 to -35.3)	-66.9 (-87.2 to -32.9)
Bahrain	Number: 1990	305 (288–321)	87 (76–99)	17 (12–22)	22 (17–28)	15 (11–20)	80 (68–93)	0 (0–0)	3 (2–8)	14 (10–19)	1 (0–2)	3 (2–4)
Bahrain	Number: 2015	131 (113–150)	25 (20–30)	6 (4–7)	8 (6–10)	2 (2–3)	53 (42–67)	0 (0–0)	5 (3–7)	6 (4–7)	0 (0–0)	0 (0–1)
Bahrain	Number: Cumulative change (%)	-57.0 (-63.0 to -50.4)	-70.9 (-77.6 to -63.4)	-63.2 (-75.6 to -47.4)	-62.1 (-72.2 to -48.3)	-80.6 (-87.0 to -72.2)	-32.7 (-50.1 to -10.6)	nan (nan–nan)	60.2 (-34.1 to 160.5)	-55.1 (-74.7 to -32.3)	-63.1 (-80.3 to -38.8)	-76.3 (-85.2 to -61.2)
Bahrain	Rate: 1990	438.0 (413.4–461.9)	125.3 (110.4–142.5)	24.7 (18.4–31.7)	32.6 (24.8–40.5)	22.4 (16.8–28.7)	115.4 (98.1–134.1)	0.0 (0.0–0.0)	5.6 (3.2–12.4)	20.6 (14.9–28.4)	2.2 (1.4–3.2)	4.7 (3.5–6.0)

© 2017 Global Burden of Disease Child and Adolescent Health Collaboration. JAMA Pediatrics.

eTable 2. Number of Deaths, Death Rates (per 100,000 population), and Cumulative Percent Change with 95% Uncertainty Intervals (UI) for the Top 10 Global Causes of Death in 195 Countries and Territories, Aged Under 5 Years, Both Sexes, 1990 and 2015 *Note: best viewed by enlarging in browser.*

Location	Measure	All causes	Neonatal preterm birth complications	Neonatal encephalopathy due to birth asphyxia and trauma	Lower respiratory infections	Diarrheal diseases	Congenital anomalies	Malaria	Neonatal sepsis and other neonatal infections	Other neonatal disorders	Protein-energy malnutrition	Meningitis
Bahrain	Rate: 2015	135.5 (117.3–155.4)	26.3 (21.1–32.1)	6.4 (4.8–8.2)	8.7 (6.4–11.0)	3.0 (2.1–4.0)	55.4 (43.8–69.4)	0.0 (0.0–0.0)	5.5 (3.6–7.4)	6.4 (4.7–8.3)	0.5 (0.3–0.8)	0.8 (0.6–1.1)
Bahrain	Rate: Cumulative change (%)	-69.0 (-73.3 to -64.3)	-78.9 (-83.8 to -73.5)	-73.4 (-82.3 to -61.8)	-72.8 (-80.1 to -62.9)	-86.1 (-90.6 to -80.1)	-51.5 (-64.1 to -35.6)	nan (nan–nan)	16.0 (-52.3 to 88.7)	-67.5 (-81.7 to -50.9)	-73.6 (-85.9 to -56.3)	-83.0 (-89.4 to -72.3)
Egypt	Number: 1990	154,657 (147,267–161,976)	17,401 (14,761–20,041)	2,143 (1,459–2,916)	45,903 (38,470–52,245)	36,696 (30,270–42,515)	14,199 (11,054–23,779)	0 (0–1)	1,224 (728–1,835)	4,769 (3,345–6,355)	329 (183–555)	1,135 (908–1,381)
Egypt	Number: 2015	53,291 (42,061–67,411)	8,517 (6,286–10,886)	803 (498–1,225)	9,759 (7,375–12,764)	4,023 (2,883–5,672)	15,317 (11,376–19,747)	1 (0–1)	1,475 (760–2,192)	2,584 (1,755–3,607)	246 (163–357)	285 (205–397)
Egypt	Number: Cumulative change (%)	-65.5 (-72.9 to -56.2)	-50.7 (-64.8 to -34.0)	-61.2 (-77.9 to -38.8)	-78.5 (-84.6 to -70.9)	-88.9 (-92.5 to -83.6)	14.4 (-44.6 to 59.8)	21.1 (-14.9 to 69.4)	26.0 (-28.2 to 102.4)	-43.9 (-65.3 to -12.3)	-16.3 (-61.1 to 48.8)	-74.3 (-82.5 to -61.1)
Egypt	Rate: 1990	1,744.5 (1,661.2–1,827.8)	194.0 (164.6–223.4)	23.9 (16.3–32.5)	518.7 (434.2–590.7)	415.3 (342.3–481.3)	159.5 (124.0–267.5)	0.0 (0.0–0.0)	13.7 (8.1–20.5)	53.2 (37.3–70.9)	3.7 (2.1–6.3)	12.9 (10.3–15.7)
Egypt	Rate: 2015	444.7 (351.0–562.2)	70.8 (52.3–90.6)	6.7 (4.1–10.2)	81.4 (61.5–106.4)	33.5 (24.1–47.3)	127.6 (94.7–164.4)	0.0 (0.0–0.0)	12.3 (6.3–18.2)	21.5 (14.6–30.0)	2.1 (1.4–3.0)	2.4 (1.7–3.3)
Egypt	Rate: Cumulative change (%)	-74.5 (-79.9 to -67.6)	-63.2 (-73.7 to -50.8)	-71.1 (-83.5 to -54.3)	-84.2 (-88.7 to -78.6)	-91.8 (-94.4 to -87.9)	-15.1 (-59.1 to 18.6)	-9.6 (-36.4 to 26.4)	-6.2 (-46.5 to 50.6)	-58.2 (-74.2 to -34.7)	-38.5 (-71.5 to 9.1)	-81.0 (-87.1 to -71.3)
Iran	Number: 1990	140,653 (126,672–156,089)	26,289 (20,449–32,907)	2,090 (1,106–3,430)	16,406 (11,199–21,639)	7,359 (4,636–10,997)	25,483 (9,895–37,316)	9 (4–18)	251 (69–709)	9,315 (5,146–14,399)	4,134 (1,746–8,449)	1,770 (931–2,807)
Iran	Number: 2015	19,914 (15,278–25,325)	4,958 (3,296–6,930)	915 (509–1,477)	1,161 (803–1,651)	238 (140–370)	5,908 (3,984–7,965)	0 (0–1)	415 (177–752)	2,026 (1,166–3,211)	104 (36–229)	162 (91–251)
Iran	Number: Cumulative change (%)	-85.8 (-89.4 to -81.8)	-80.8 (-87.8 to -72.1)	-51.2 (-79.1 to -7.7)	-92.6 (-95.6 to -88.4)	-96.5 (-98.3 to -94.0)	-73.7 (-86.2 to -46.6)	-88.4 (-95.9 to -74.1)	191.6 (-57.6 to 651.8)	-75.9 (-89.4 to -54.4)	-96.8 (-99.3 to -91.9)	-89.5 (-95.7 to -78.3)
Iran	Rate: 1990	1,579.0 (1,422.3–1,752.6)	298.8 (232.5–374.1)	23.8 (12.6–39.0)	184.3 (125.9–243.1)	82.6 (51.8–123.2)	288.1 (111.9–421.3)	0.1 (0.0–0.2)	2.9 (0.8–8.1)	105.9 (58.6–163.7)	46.4 (19.5–95.4)	19.8 (10.4–31.3)
Iran	Rate: 2015	301.4 (230.9–383.2)	75.9 (50.5–106.2)	14.0 (7.8–22.6)	17.4 (12.0–24.7)	3.6 (2.1–5.6)	89.4 (60.4–120.6)	0.0 (0.0–0.0)	6.3 (2.7–11.5)	31.0 (17.8–49.2)	1.6 (0.6–3.5)	2.4 (1.4–3.8)
Iran	Rate: Cumulative change (%)	-80.8 (-85.7 to -75.4)	-74.2 (-83.6 to -62.5)	-34.3 (-71.9 to 24.5)	-90.2 (-94.1 to -84.5)	-95.3 (-97.8 to -92.0)	-64.7 (-81.6 to -28.6)	-84.5 (-94.6 to -65.7)	290.4 (-43.2 to 903.6)	-67.6 (-85.8 to -38.7)	-95.8 (-99.0 to -89.2)	-86.0 (-94.2 to -71.0)
Iraq	Number: 1990	35,176 (33,731–36,559)	7,790 (6,165–9,379)	1,402 (805–2,217)	4,642 (3,403–5,963)	3,430 (2,449–4,621)	5,131 (3,037–8,924)	1 (1–1)	3,398 (1,473–5,473)	1,373 (641–2,437)	285 (106–637)	1,017 (543–1,847)
Iraq	Number: 2015	33,478 (29,472–37,772)	7,310 (5,285–9,276)	1,597 (939–2,476)	2,696 (2,020–3,455)	1,239 (790–1,782)	6,953 (4,678–11,271)	0 (0–0)	4,669 (2,125–7,215)	1,340 (617–2,397)	144 (54–327)	651 (323–1,169)
Iraq	Number: Cumulative change (%)	-4.8 (-16.2 to 8.6)	-4.7 (-33.3 to 28.0)	24.7 (-46.4 to 122.7)	-40.4 (-59.4 to -15.9)	-62.5 (-77.8 to -42.2)	43.5 (-11.1 to 105.4)	-100.0 (-100.0 to -100.0)	45.9 (-21.0 to 140.3)	15.1 (-60.6 to 158.5)	-33.4 (-84.4 to 69.5)	-29.2 (-68.5 to 38.8)
Iraq	Rate: 1990	1,128.1 (1,081.8–1,173.7)	245.6 (194.4–296.0)	44.3 (25.4–70.4)	150.0 (109.8–192.8)	110.6 (79.0–148.9)	164.1 (97.1–285.8)	0.0 (0.0–0.1)	107.2 (46.5–172.4)	43.6 (20.3–77.1)	9.3 (3.5–20.6)	33.1 (17.7–60.0)
Iraq	Rate: 2015	565.4 (497.8–637.6)	122.2 (88.3–155.1)	26.7 (15.7–41.4)	45.7 (34.3–58.7)	21.0 (13.4–30.0)	117.2 (79.0–190.0)	0.0 (0.0–0.0)	78.0 (35.5–120.6)	22.4 (10.4–40.2)	2.5 (0.9–5.6)	11.1 (5.5–20.0)
Iraq	Rate: Cumulative change (%)	-49.9 (-55.9 to -42.8)	-49.5 (-64.6 to -32.1)	-33.9 (-71.6 to 17.8)	-68.7 (-78.7 to -56.1)	-80.3 (-88.4 to -69.5)	-24.4 (-53.2 to 8.4)	-100.0 (-100.0 to -100.0)	-22.7 (-58.2 to 27.3)	-39.2 (-79.2 to 36.6)	-65.1 (-91.7 to -11.7)	-62.9 (-83.5 to -26.8)
Jordan	Number: 1990	3,751 (3,633–3,876)	944 (768–1,120)	196 (121–302)	470 (366–585)	104 (66–153)	839 (676–1,059)	0 (0–0)	196 (107–324)	174 (93–280)	9 (4–17)	51 (28–94)
Jordan	Number: 2015	2,920 (2,605–3,288)	630 (492–785)	189 (126–262)	269 (216–328)	24 (16–33)	912 (771–1,066)	0 (0–0)	228 (135–359)	86 (46–141)	3 (1–6)	28 (17–44)
Jordan	Number: Cumulative change (%)	-22.1 (-30.2 to -12.4)	-32.5 (-48.9 to -12.3)	3.7 (-45.5 to 66.3)	-41.2 (-58.9 to -19.7)	-75.0 (-86.7 to -59.4)	11.0 (-20.9 to 42.5)	nan (nan–nan)	28.7 (-40.6 to 121.8)	-44.0 (-77.1 to 16.7)	-52.9 (-85.5 to 4.5)	-38.1 (-75.3 to 19.3)
Jordan	Rate: 1990	671.5 (650.8–694.4)	165.3 (134.7–196.1)	34.5 (21.4–53.2)	85.1 (66.3–105.9)	19.0 (12.0–27.8)	149.7 (120.4–189.3)	0.0 (0.0–0.0)	34.4 (18.9–56.9)	30.8 (16.5–49.4)	1.7 (0.7–3.2)	9.5 (5.1–17.1)
Jordan	Rate: 2015	303.2 (270.6–341.4)	65.5 (51.1–81.5)	19.6 (13.1–27.3)	28.0 (22.5–34.1)	2.5 (1.7–3.5)	94.6 (79.9–110.6)	0.0 (0.0–0.0)	23.7 (14.0–37.3)	9.0 (4.9–14.7)	0.4 (0.2–0.6)	2.9 (1.8–4.6)
Jordan	Rate: Cumulative change (%)	-54.8 (-59.5 to -49.2)	-60.0 (-69.7 to -48.0)	-38.8 (-67.8 to -1.5)	-66.3 (-76.2 to -53.8)	-85.7 (-92.4 to -76.6)	-35.5 (-53.9 to -17.0)	nan (nan–nan)	-24.0 (-65.0 to 30.8)	-67.0 (-86.5 to -31.2)	-73.2 (-91.6 to -40.8)	-64.7 (-85.9 to -32.3)
Kuwait	Number: 1990	691 (629–757)	89 (72–108)	64 (51–79)	62 (50–73)	12 (9–14)	226 (190–259)	0 (0–0)	15 (7–23)	20 (14–26)	0 (0–0)	5 (4–7)
Kuwait	Number: 2015	607 (495–733)	151 (118–186)	16 (12–21)	36 (28–46)	4 (3–5)	273 (216–340)	0 (0–0)	8 (5–14)	9 (6–11)	0 (0–0)	4 (3–5)
Kuwait	Number: Cumulative change (%)	-11.9 (-29.8 to 7.9)	71.9 (26.5–122.0)	-73.4 (-81.5 to -63.2)	-40.9 (-57.2 to -19.4)	-65.8 (-76.2 to -52.2)	22.0 (-7.1 to 57.1)	nan (nan–nan)	-37.4 (-70.8 to 20.1)	-52.6 (-69.4 to -30.6)	-85.9 (-91.9 to -77.6)	-28.2 (-48.9 to -1.2)
Kuwait	Rate: 1990	328.5 (299.6–359.6)	44.6 (36.2–54.5)	32.1 (25.7–39.8)	29.1 (23.8–34.7)	5.8 (4.5–7.1)	109.4 (92.4–125.6)	0.0 (0.0–0.0)	7.7 (3.6–11.5)	10.0 (7.3–13.1)	0.3 (0.2–0.4)	2.8 (2.2–3.6)
Kuwait	Rate: 2015	167.9 (137.1–202.5)	41.6 (32.8–51.5)	4.7 (3.5–6.0)	10.0 (7.9–12.7)	1.1 (0.9–1.5)	75.6 (59.9–93.9)	0.0 (0.0–0.0)	2.4 (1.5–4.0)	2.5 (1.9–3.3)	0.0 (0.0–0.0)	1.2 (0.9–1.5)
Kuwait	Rate: Cumulative change (%)	-48.7 (-59.1 to -37.1)	-5.4 (-30.4 to 22.2)	-85.3 (-89.8 to -79.7)	-65.1 (-74.8 to -52.3)	-80.0 (-86.1 to -72.1)	-30.3 (-46.9 to -10.4)	nan (nan–nan)	-65.5 (-83.9 to -33.7)	-73.8 (-83.1 to -61.7)	-91.6 (-95.2 to -86.6)	-57.2 (-69.6 to -41.0)
Lebanon	Number: 1990	2,415 (2,137–2,725)	635 (532–752)	125 (73–190)	241 (173–314)	94 (52–148)	562 (433–699)	0 (0–0)	40 (17–77)	90 (44–154)	7 (2–18)	33 (17–55)
Lebanon	Number: 2015	672 (517–873)	149 (100–207)	42 (22–68)	25 (15–38)	6 (3–11)	276 (189–377)	0 (0–0)	32 (16–56)	31 (13–57)	0 (0–2)	5 (2–10)
Lebanon	Number: Cumulative change (%)	-72.0 (-79.0 to -62.7)	-76.1 (-84.9 to -65.3)	-63.9 (-83.0 to -33.8)	-89.1 (-94.0 to -82.1)	-92.4 (-96.8 to -85.4)	-49.5 (-70.4 to -23.4)	nan (nan–nan)	0.0 (-60.7 to 123.9)	-59.9 (-86.2 to -10.7)	-82.7 (-96.7 to -55.8)	-82.4 (-93.9 to -62.3)
Lebanon	Rate: 1990	729.7 (644.9–822.6)	193.8 (162.6–229.6)	38.2 (22.4–58.3)	72.5 (52.1–94.6)	28.4 (15.9–44.9)	170.6 (131.4–211.7)	0.0 (0.0–0.0)	12.3 (5.2–23.8)	27.5 (13.5–47.0)	2.4 (0.8–5.6)	9.9 (5.3–16.8)
Lebanon	Rate: 2015	166.6 (128.3–215.9)	36.2 (24.2–50.1)	10.2 (5.6–16.5)	6.5 (4.0–9.7)	1.6 (0.9–2.9)	68.3 (46.5–93.3)	0.0 (0.0–0.0)	8.0 (3.9–13.7)	7.6 (3.3–14.0)	0.3 (0.1–0.6)	1.3 (0.6–2.5)
Lebanon	Rate: Cumulative change (%)	-77.1 (-82.8 to -69.6)	-81.1 (-88.1 to -72.5)	-71.3 (-86.5 to -47.5)	-90.7 (-94.9 to -84.7)	-93.6 (-97.3 to -87.7)	-58.9 (-75.9 to -37.5)	nan (nan–nan)	-20.9 (-69.0 to 76.7)	-68.1 (-89.0 to -29.1)	-85.3 (-97.2 to -61.4)	-85.0 (-94.9 to -67.8)
Libya	Number: 1990	5,300 (4,893–5,764)	1,234 (1,008–1,481)	211 (117–340)	536 (389–703)	266 (160–399)	1,081 (818–1,462)	0 (0–0)	72 (32–137)	190 (93–335)	19 (7–44)	76 (38–130)
Libya	Number: 2015	2,294 (1,830–2,857)	517 (354–712)	100 (57–156)	100 (69–138)	32 (19–50)	638 (486–815)	0 (0–0)	71 (33–129)	83 (38–147)	4 (1–9)	18 (9–30)
Libya	Number: Cumulative change (%)	-56.6 (-65.9 to -45.1)	-57.7 (-71.6 to -40.5)	-48.6 (-75.6 to -7.7)	-80.5 (-88.3 to -69.4)	-86.9 (-93.6 to -77.1)	-38.9 (-61.0 to -12.2)	nan (nan–nan)	19.1 (-56.7 to 169.8)	-49.7 (-82.7 to 3.7)	-69.0 (-93.8 to -21.3)	-73.0 (-90.3 to -48.1)
Libya	Rate: 1990	862.7 (795.0–939.3)	203.5 (166.2–244.2)	34.8 (19.4–56.1)	86.7 (62.8–113.9)	43.3 (25.9–64.8)	176.6 (133.7–238.6)	0.0 (0.0–0.0)	11.9 (5.4–22.7)	31.4 (15.4–55.2)	3.2 (1.2–7.1)	12.3 (6.2–21.1)
Libya	Rate: 2015	357.6 (284.4–446.5)	82.5 (56.6–113.5)	15.9 (9.1–24.9)	15.4 (10.7–21.1)	5.0 (2.9–7.8)	99.9 (75.9–127.9)	0.0 (0.0–0.0)	11.4 (5.3–20.7)	13.3 (6.2–23.4)	0.7 (0.3–1.5)	2.8 (1.4–4.6)
Libya	Rate: Cumulative change (%)	-58.4 (-67.4 to -47.3)	-59.1 (-72.5 to -42.4)	-50.3 (-76.5 to -10.9)	-81.5 (-88.9 to -71.1)	-87.6 (-94.0 to -78.2)	-41.4 (-62.5 to -15.6)	nan (nan–nan)	15.1 (-58.2 to 161.8)	-51.4 (-83.3 to 0.2)	-70.6 (-94.1 to -25.0)	-74.4 (-90.8 to -50.7)
Morocco	Number: 1990	51,708 (49,879–53,616)	9,450 (7,498–11,558)	3,292 (2,162–4,696)	6,653 (5,302–8,167)	8,372 (6,521–10,187)	3,922 (2,754–7,588)	0 (0–0)	3,089 (1,730–4,832)	1,652 (817–2,937)	304 (97–736)	1,932 (799–3,252)
Morocco	Number: 2015	15,696 (13,042–18,935)	4,263 (3,059–5,498)	1,717 (1,150–2,416)	991 (720–1,330)	294 (191–433)	2,594 (1,621–4,480)	0 (0–0)	1,596 (978–2,385)	364 (182–628)	101 (44–207)	270 (165–419)
Morocco	Number: Cumulative change (%)	-69.6 (-74.9 to -63.1)	-54.0 (-68.8 to -32.7)	-44.9 (-69.1 to -9.5)	-84.8 (-89.8 to -78.4)	-96.4 (-97.8 to -94.6)	-31.9 (-56.2 to -0.1)	nan (nan–nan)	-43.9 (-72.3 to -5.9)	-74.2 (-90.8 to -44.9)	-50.6 (-90.2 to 30.6)	-83.5 (-92.7 to -70.1)
Morocco	Rate: 1990	1,475.7 (1,423.4–1,529.8)	269.1 (213.6–329.3)	93.7 (61.5–133.6)	190.2 (151.5–233.5)	239.5 (186.5–291.6)	112.0 (78.7–216.8)	0.0 (0.0–0.0)	88.1 (49.4–137.7)	47.1 (23.3–84.0)	8.7 (2.8–21.1)	55.2 (22.8–92.9)
Morocco	Rate: 2015	462.9 (384.5–558.5)	126.2 (90.6–162.7)	50.8 (34.0–71.6)	29.1 (21.2–39.1)	8.6 (5.6–12.7)	76.5 (47.8–132.1)	0.0 (0.0–0.0)	47.2 (28.9–70.6)	10.8 (5.4–18.6)	3.0 (1.3–6.1)	7.9 (4.8–12.3)
Morocco	Rate: Cumulative change (%)	-68.6 (-74.1 to -61.9)	-52.2 (-67.5 to -30.1)	-42.8 (-67.9 to -5.9)	-84.4 (-89.5 to -77.8)	-96.3 (-97.8 to -94.4)	-29.7 (-54.8 to 3.2)	nan (nan–nan)	-41.8 (-71.3 to -2.1)	-73.2 (-90.5 to -42.8)	-49.3 (-89.9 to 33.7)	-83.1 (-92.6 to -69.2)
Palestine	Number: 1990	3,729 (3,417–4,066)	1,366 (1,141–1,599)	236 (140–357)	315 (220–429)	67 (42–103)	650 (442–1,028)	0 (0–0)	251 (45–479)	77 (27–179)	6 (2–15)	48 (25–87)
Palestine	Number: 2015	2,584 (2,078–3,167)	893 (668–1,134)	202 (136–289)	100 (71–134)	16 (11–23)	600 (418–933)	0 (0–0)	223 (44–373)	100 (56–163)	1 (0–3)	16 (10–24)
Palestine	Number: Cumulative change (%)	-30.5 (-45.6 to -13.8)	-33.9 (-53.0 to -12.8)	-7.1 (-53.2 to 61.4)	-66.8 (-79.7 to -49.5)	-73.9 (-85.6 to -58.7)	-4.8 (-38.9 to 36.5)	nan (nan–nan)	-1.3 (-49.2 to 67.8)	77.1 (-53.8 to 284.0)	-70.2 (-93.0 to -29.5)	-60.7 (-83.9 to -27.6)
Palestine	Rate: 1990	819.1 (752.3–893.9)	293.0 (244.5–342.9)	51.3 (30.4–77.6)	71.2 (49.8–96.8)	14.9 (9.6–23.1)	142.7 (97.0–225.6)	0.0 (0.0–0.0)	54.2 (9.7–103.2)	16.9 (6.1–39.1)	1.6 (0.6–3.6)	11.0 (5.8–19.5)
Palestine	Rate: 2015	356.0 (286.2–436.1)	122.3 (91.5–155.4)	27.7 (18.7–39.7)	14.0 (10.0–18.8)	2.3 (1.6–3.2)	82.7 (57.7–128.6)	0.0 (0.0–0.0)	30.7 (6.0–51.2)	13.8 (7.7–22.4)	0.2 (0.1–0.4)	2.3 (1.4–3.4)
Palestine	Rate: Cumulative change (%)	-56.4 (-65.9 to -46.0)	-57.8 (-70.1 to -44.2)	-41.2 (-70.3 to 2.0)	-79.5 (-87.5 to -68.8)	-83.8 (-91.0 to -74.5)	-40.2 (-61.5 to -14.2)	nan (nan–nan)	-37.2 (-67.7 to 6.3)	12.1 (-70.9 to 144.3)	-81.7 (-95.8 to -56.8)	-75.7 (-90.0 to -55.7)
Oman	Number: 1990	2,954 (2,551–3,408)	563 (394–752)	92 (47–156)	310 (193–459)	56 (31–91)	389 (254–659)	0 (0–0)	8 (2–19)	458 (271–686)	39 (16–79)	31 (14–60)
Oman	Number: 2015	749 (663–838)	135 (100–171)	37 (24–54)	35 (27–46)	3 (2–5)	214 (172–267)	0 (0–0)	9 (2–19)	99 (64–137)	3 (1–6)	5 (3–8)
Oman	Number: Cumulative change (%)	-74.4 (-79.0 to -69.6)	-75.0 (-84.3 to -63.4)	-53.7 (-79.2 to -16.5)	-87.5 (-92.9 to -79.7)	-92.7 (-96.8 to -86.3)	-39.8 (-71.6 to -11.0)	nan (nan–nan)	34.8 (-64.0 to 248.1)	-76.3 (-87.7 to -57.9)	-90.1 (-97.4 to -76.8)	-79.2 (-92.8 to -55.9)
Oman	Rate: 1990	877.3 (757.8–1,012.0)	167.3 (117.2–223.4)	27.6 (14.1–46.6)	92.3 (57.5–136.5)	16.8 (9.2–27.3)	115.7 (75.5–195.8)	0.0 (0.0–0.0)	2.6 (0.8–5.9)	136.2 (80.6–203.9)	11.8 (4.9–23.6)	9.2 (4.3–17.9)
Oman	Rate: 2015	192.4 (170.5–215.2)	34.3 (25.4–43.4)	9.6 (6.1–13.7)	9.3 (7.0–12.2)	0.9 (0.6–1.4)	54.7 (43.8–68.2)	0.0 (0.0–0.0)	2.3 (0.6–4.9)	25.3 (16.5–34.8)	0.8 (0.4–1.6)	1.4 (0.8–2.3)
Oman	Rate: Cumulative change (%)	-77.9 (-81.9 to -73.7)	-78.6 (-86.6 to -68.8)	-60.5 (-82.2 to -28.5)	-89.1 (-93.8 to -82.3)	-93.7 (-97.2 to -88.1)	-48.3 (-75.7 to -23.7)	nan (nan–nan)	14.9 (-69.3 to 196.7)	-79.8 (-89.5 to -64.1)	-91.4 (-97.8 to -79.9)	-81.9 (-93.8 to -61.6)
Qatar	Number: 1990	230 (181–290)	83 (62–108)	7 (4–12)	6 (4–10)	1 (0–2)	64 (45–87)	0 (0–0)	1 (0–3)	7 (3–13)	0 (0–0)	1 (0–3)
Qatar	Number: 2015	221 (160–293)	62 (43–88)	10 (5–15)	4 (2–6)	0 (0–1)	86 (60–118)	0 (0–0)	1 (0–2)	11 (6–18)	0 (0–0)	1 (1–3)
Qatar	Number: Cumulative change (%)	-2.1 (-35.9 to 38.8)	-23.1 (-51.5 to 13.2)	41.0 (-36.1 to 163.5)	-28.3 (-62.0 to 18.9)	-24.5 (-68.1 to 46.0)	39.0 (-14.3 to 112.4)	nan (nan–nan)	10.9 (-69.4 to 139.6)	76.7 (-34.1 to 259.2)	-47.0 (-87.2 to 23.7)	9.7 (-57.0 to 127.6)
Qatar	Rate: 1990	451.7 (355.0–568.6)	165.1 (123.2–213.5)	15.5 (8.6–24.5)	13.3 (8.5–19.5)	2.7 (1.4–4.5)	126.6 (89.0–170.7)	0.0 (0.0–0.0)	3.3 (0.9–7.1)	15.4 (7.3–27.1)	0.1 (0.1–0.3)	3.8 (1.8–6.4)
Qatar	Rate: 2015	177.5 (129.1–235.6)	49.7 (34.4–69.6)	8.0 (4.6–12.5)	3.7 (2.4–5.6)	0.7 (0.4–1.2)	69.2 (48.4–94.8)	0.0 (0.0–0.0)	1.1 (0.5–1.9)	9.4 (5.3–14.9)	0.0 (0.0–0.1)	1.5 (0.8–2.5)
Qatar	Rate: Cumulative change (%)	-59.9 (-73.7 to -43.3)	-69.2 (-80.6 to -54.6)	-43.0 (-74.2 to 6.7)	-70.2 (-84.3 to -50.5)	-68.6 (-86.7 to -39.5)	-43.4 (-65.1 to -13.3)	nan (nan–nan)	-55.6 (-87.8 to -3.9)	-28.8 (-73.6 to 44.6)	-77.7 (-94.6 to -48.6)	-54.4 (-82.1 to -4.7)
Saudi Arabia	Number: 1990	26,687 (24,635–28,941)	8,612 (7,353–9,687)	1,304 (968–1,679)	1,042 (679–1,511)	1,265 (792–1,860)	5,585 (4,328–8,141)	0 (0–1)	2,186 (1,610–2,919)	1,114 (716–1,675)	107 (46–188)	194 (84–330)
Saudi Arabia	Number: 2015	7,138 (6,428–7,911)	1,777 (1,299–2,121)	390 (295–479)	131 (110–157)	103 (82–127)	2,596 (2,186–3,246)	0 (0–0)	842 (659–1,137)	262 (167–357)	9 (6–12)	25 (14–34)
Saudi Arabia	Number: Cumulative change (%)	-73.2 (-76.2 to -70.1)	-79.3 (-84.9 to -74.5)	-69.4 (-79.0 to -59.3)	-86.5 (-91.6 to -79.4)	-91.2 (-94.7 to -86.2)	-51.7 (-68.8 to -33.7)	-77.9 (-94.3 to -49.6)	-60.7 (-71.3 to -48.1)	-74.5 (-88.5 to -59.2)	-90.1 (-95.4 to -83.0)	-85.4 (-92.0 to -75.6)
Saudi Arabia	Rate: 1990	970.2 (895.3–1,052.7)	310.1 (264.7–348.9)	47.1 (34.9–60.7)	38.4 (25.1–55.7)	46.2 (29.0–67.8)	202.9 (157.1–296.1)	0.0 (0.0–0.1)	78.8 (58.1–105.2)	40.2 (25.8–60.7)	3.9 (1.7–6.9)	7.1 (3.1–12.1)
Saudi Arabia	Rate: 2015	236.4 (212.9–262.0)	59.2 (43.3–70.7)	13.0 (9.8–16.0)	4.3 (3.6–5.2)	3.4 (2.7–4.2)	85.9 (72.3–107.4)	0.0 (0.0–0.0)	28.0 (21.9–37.9)	8.7 (5.6–11.9)	0.3 (0.2–0.4)	0.8 (0.5–1.1)
Saudi Arabia	Rate: Cumulative change (%)	-75.6 (-78.3 to -72.7)	-80.8 (-86.0 to -76.4)	-71.8 (-80.7 to -62.5)	-87.9 (-92.5 to -81.6)	-92.1 (-95.2 to -87.6)	-56.1 (-71.7 to -39.6)	-80.2 (-94.9 to -54.8)	-63.7 (-73.6 to -52.1)	-76.5 (-89.4 to -62.4)	-91.2 (-95.9 to -84.9)	-87.0 (-92.9 to -78.3)
Sudan	Number: 1990	110,413 (104,203–117,125)	18,827 (15,025–22,783)	2,610 (1,444–4,174)	19,520 (14,764–25,047)	18,144 (12,965–24,106)	9,218 (4,527–20,643)	2,136 (868–3,877)	762 (308–1,507)	2,687 (1,153–4,861)	905 (332–1,999)	2,409 (1,226–3,924)
Sudan	Number: 2015	73,176 (59,029–90,691)	20,156 (15,890–24,814)	2,803 (1,584–4,421)	8,684 (5,922–12,425)	7,620 (4,609–11,744)	10,870 (6,763–18,266)	1,568 (591–3,005)	1,500 (718–2,840)	2,576 (1,192–4,804)	500 (173–1,076)	1,511 (708–2,501)
Sudan	Number: Cumulative change (%)	-33.6 (-46.7 to -17.8)	8.1 (-14.7 to 34.2)	16.1 (-42.7 to 106.0)	-54.6 (-70.8 to -32.2)	-57.1 (-74.1 to -32.5)	35.8 (-21.2 to 101.6)	-20.6 (-64.5 to 47.3)	144.1 (-10.1 to 470.6)	13.4 (-61.8 to 134.7)	-29.1 (-84.7 to 84.3)	-29.8 (-72.5 to 38.8)
Sudan	Rate: 1990	2,962.5 (2,789.7–3,144.7)	466.6 (372.1–565.0)	64.9 (35.8–103.9)	534.3 (400.3–689.4)	490.1 (348.4–651.6)	238.6 (115.6–538.2)	58.9 (24.0–107.3)	19.0 (7.7–37.5)	67.1 (29.0–120.6)	25.0 (9.1–55.4)	66.2 (33.5–108.4)
Sudan	Rate: 2015	1,174.5 (946.7–1,457.6)	319.1 (251.5–392.9)	44.5 (25.1–70.0)	140.7 (95.6–201.2)	123.0 (74.3–190.2)	173.9 (108.1–292.5)	25.5 (9.6–48.8)	23.8 (11.4–45.0)	40.9 (19.0–76.2)	8.1 (2.8–17.4)	24.5 (11.5–40.7)
Sudan	Rate: Cumulative change (%)	-60.3 (-68.2 to -50.7)	-30.9 (-45.5 to -14.2)	-25.9 (-63.3 to 31.6)	-73.1 (-82.7 to -59.6)	-74.3 (-84.6 to -59.5)	-15.4 (-51.7 to 26.0)	-53.2 (-79.0 to -13.8)	55.1 (-42.7 to 261.2)	-28.0 (-75.6 to 48.9)	-58.2 (-91.0 to 9.5)	-58.4 (-83.8 to -17.1)
Syria	Number: 1990	18,855 (17,515–20,298)	2,887 (2,233–3,543)	1,645 (1,053–2,327)	2,730 (1,689–3,580)	1,606 (982–2,239)	2,526 (1,716–3,326)	0 (0–0)	372 (167–694)	1,011 (480–1,679)	151 (59–295)	589 (318–955)
Syria	Number: 2015	10,116 (7,858–12,429)	714 (494–962)	635 (368–972)	750 (572–935)	49 (31–73)	1,700 (1,295–2,034)	0 (0–0)	297 (161–489)	355 (157–603)	28 (12–63)	143 (85–225)
Syria	Number: Cumulative change (%)	-46.2 (-58.8 to -33.1)	-74.8 (-83.2 to -64.5)	-59.8 (-78.3 to -34.6)	-70.8 (-81.1 to -52.9)	-96.7 (-98.3 to -94.3)	-29.9 (-53.1 to -2.4)	nan (nan–nan)	-4.9 (-58.1 to 96.3)	-61.2 (-84.5 to -26.3)	-75.8 (-93.6 to -44.3)	-72.7 (-88.1 to -48.2)
Syria	Rate: 1990	883.3 (820.3–950.9)	134.8 (104.3–165.5)	76.8 (49.2–108.7)	128.1 (79.2–168.0)	75.3 (46.0–104.9)	118.3 (80.3–155.7)	0.0 (0.0–0.0)	17.4 (7.8–32.5)	47.3 (22.4–78.5)	7.1 (2.8–13.9)	27.6 (14.9–44.8)
Syria	Rate: 2015	454.2 (355.0–556.4)	33.7 (23.3–45.4)	30.0 (17.4–45.9)	33.2 (25.4–41.2)	2.2 (1.4–3.3)	78.6 (59.7–94.2)	0.0 (0.0–0.0)	14.0 (7.6–23.0)	16.7 (7.4–28.4)	1.3 (0.5–2.8)	6.4 (3.8–10.0)
Syria	Rate: Cumulative change (%)	-48.5 (-60.1 to -36.3)	-74.6 (-83.0 to -64.1)	-59.4 (-78.1 to -33.9)	-72.5 (-82.1 to -55.7)	-96.8 (-98.4 to -94.5)	-30.8 (-53.6 to -4.0)	nan (nan–nan)	-4.5 (-57.9 to 97.1)	-61.1 (-84.4 to -25.8)	-77.2 (-93.9 to -48.2)	-74.0 (-88.7 to -50.4)
Tunisia	Number: 1990	10,694 (10,182–11,251)	2,692 (2,174–3,214)	711 (423–1,085)	954 (676–1,264)	794 (505–1,146)	1,371 (892–2,302)	0 (0–0)	570 (303–942)	591 (285–983)	66 (22–157)	278 (133–488)
Tunisia	Number: 2015	2,775 (2,385–3,243)	752 (571–961)	253 (171–356)	110 (83–142)	37 (24–52)	688 (532–914)	0 (0–0)	249 (154–366)	116 (63–192)	5 (2–11)	39 (24–62)
Tunisia	Number: Cumulative change (%)	-74.0 (-77.9 to -69.3)	-71.7 (-79.7 to -61.0)	-61.5 (-79.2 to -36.6)	-87.9 (-92.1 to -82.2)	-95.0 (-97.4 to -91.7)	-46.1 (-70.4 to -22.5)	nan (nan–nan)	-52.0 (-77.4 to -19.0)	-77.2 (-91.2 to -53.2)	-87.5 (-97.7 to -67.6)	-83.7 (-93.3 to -68.1)
Tunisia	Rate: 1990	1,027.3 (978.0–1,080.5)	261.6 (211.3–312.3)	69.1 (41.1–105.3)	90.9 (64.7–120.3)	76.0 (48.4–110.0)	132.0 (86.0–222.2)	0.0 (0.0–0.0)	55.5 (29.5–91.6)	57.4 (27.8–95.4)	6.3 (2.1–15.0)	26.6 (12.7–46.4)
Tunisia	Rate: 2015	283.6 (243.7–331.5)	77.0 (58.5–98.5)	26.0 (17.6–36.5)	11.3 (8.5–14.5)	3.8 (2.5–5.4)	70.3 (54.4–93.3)	0.0 (0.0–0.0)	25.5 (15.7–37.5)	11.9 (6.5–19.7)	0.6 (0.2–1.2)	4.0 (2.5–6.3)
Tunisia	Rate: Cumulative change (%)	-72.4 (-76.5 to -67.4)	-70.2 (-78.6 to -59.0)	-59.4 (-78.0 to -33.0)	-87.1 (-91.6 to -80.9)	-94.7 (-97.2 to -91.2)	-42.8 (-68.6 to -18.1)	nan (nan–nan)	-49.5 (-76.3 to -14.7)	-76.0 (-90.7 to -50.9)	-86.7 (-97.6 to -65.2)	-82.5 (-92.8 to -66.0)
Turkey	Number: 1990	99,770 (93,527–106,117)	21,086 (16,854–25,482)	4,311 (2,635–6,309)	20,457 (15,925–25,219)	12,897 (9,083–17,241)	8,998 (3,472–14,432)	6 (4–8)	613 (242–1,146)	5,509 (2,644–9,388)	914 (340–2,046)	4,071 (1,301–7,776)
Turkey	Number: 2015	21,375 (16,592–27,447)	5,443 (3,595–7,806)	1,597 (959–2,444)	984 (670–1,415)	244 (148–387)	5,904 (3,080–8,645)	0 (0–0)	922 (188–1,693)	2,121 (1,211–3,255)	65 (29–124)	195 (101–310)

© 2017 Global Burden of Disease Child and Adolescent Health Collaboration. JAMA Pediatrics.

eTable 2. Number of Deaths, Death Rates (per 100,000 population), and Cumulative Percent Change with 95% Uncertainty Intervals (UI) for the Top 10 Global Causes of Death in 195 Countries and Territories, Aged Under 5 Years, Both Sexes, 1990 and 2015 *Note: best viewed by enlarging in browser.*

Location	Measure	All causes	Neonatal preterm birth complications	Neonatal encephalopathy due to birth asphyxia and trauma	Lower respiratory infections	Diarrheal diseases	Congenital anomalies	Malaria	Neonatal sepsis and other neonatal infections	Other neonatal disorders	Protein-energy malnutrition	Meningitis
Turkey	Number: Cumulative change (%)	-78.5 (-83.5 to -72.3)	-74.0 (-83.2 to -63.4)	-60.2 (-80.9 to -31.4)	-95.1 (-97.0 to -92.5)	-98.0 (-99.0 to -96.6)	-28.9 (-67.3 to 8.6)	-91.4 (-94.6 to -87.7)	61.9 (-38.8 to 236.6)	-56.1 (-81.3 to -16.3)	-90.4 (-97.9 to -78.1)	-92.6 (-98.2 to -80.8)
Turkey	Rate: 1990	1,485.6 (1,392.3–1,580.3)	313.4 (250.5–378.8)	64.0 (39.2–93.6)	305.1 (237.5–375.9)	192.6 (135.6–258.0)	134.3 (51.8–215.5)	0.1 (0.1–0.1)	9.1 (3.6–17.0)	82.0 (39.4–139.7)	13.7 (5.1–30.6)	60.7 (19.4–116.3)
Turkey	Rate: 2015	340.5 (264.1–437.5)	87.3 (57.7–125.0)	25.6 (15.4–39.1)	15.6 (10.6–22.4)	3.9 (2.3–6.1)	94.0 (49.0–137.7)	0.0 (0.0–0.0)	14.8 (3.0–27.1)	34.0 (19.4–52.1)	1.0 (0.5–2.0)	3.1 (1.6–4.9)
Turkey	Rate: Cumulative change (%)	-77.0 (-82.4 to -70.3)	-71.9 (-81.8 to -60.6)	-57.2 (-79.4 to -26.0)	-94.8 (-96.8 to -92.0)	-97.9 (-98.9 to -96.4)	-24.1 (-65.1 to 16.0)	-90.9 (-94.2 to -87.0)	74.0 (-34.2 to 261.6)	-52.8 (-79.8 to -9.7)	-89.8 (-97.8 to -76.9)	-92.2 (-98.1 to -79.7)
United Arab Emirates	Number: 1990	1,121 (719–1,643)	314 (179–478)	64 (30–112)	37 (16–66)	19 (8–34)	250 (120–425)	0 (0–0)	31 (12–63)	68 (26–130)	7 (1–17)	18 (6–37)
United Arab Emirates	Number: 2015	537 (337–825)	100 (54–166)	32 (14–57)	10 (5–16)	2 (1–4)	202 (118–321)	0 (0–0)	34 (15–63)	29 (12–54)	1 (0–4)	6 (3–11)
United Arab Emirates	Number: Cumulative change (%)	-48.9 (-73.6 to -9.7)	-65.3 (-84.1 to -33.7)	-40.2 (-82.3 to 36.8)	-66.3 (-88.5 to -27.7)	-84.2 (-94.9 to -65.9)	-7.3 (-61.1 to 84.3)	nan (nan–nan)	38.2 (-59.1 to 208.5)	-45.4 (-85.9 to 34.8)	-57.8 (-93.6 to 10.6)	-50.1 (-86.1 to 21.4)
United Arab Emirates	Rate: 1990	502.9 (323.1–737.3)	139.8 (79.9–212.9)	28.9 (13.5–50.0)	16.8 (7.3–30.0)	8.6 (3.6–15.7)	112.0 (53.6–189.6)	0.0 (0.0–0.0)	14.0 (5.5–28.4)	30.3 (11.8–58.2)	3.2 (0.8–7.6)	8.2 (2.9–16.8)
United Arab Emirates	Rate: 2015	112.2 (70.4–172.5)	21.1 (11.4–34.8)	6.8 (3.0–12.1)	2.1 (1.2–3.5)	0.5 (0.3–0.9)	42.2 (24.7–67.1)	0.0 (0.0–0.0)	7.2 (3.2–13.3)	6.1 (2.6–11.3)	0.4 (0.1–0.8)	1.4 (0.7–2.5)
United Arab Emirates	Rate: Cumulative change (%)	-76.2 (-87.7 to -58.0)	-83.7 (-92.5 to -68.8)	-71.9 (-91.7 to -35.7)	-84.4 (-94.7 to -66.5)	-92.7 (-97.6 to -84.1)	-56.7 (-81.8 to -13.6)	nan (nan–nan)	-35.0 (-80.8 to 45.0)	-74.3 (-93.4 to -36.8)	-80.6 (-97.0 to -49.2)	-77.0 (-93.6 to -43.8)
Yemen	Number: 1990	78,956 (76,596–81,508)	14,873 (11,479–17,805)	1,999 (1,039–3,338)	14,543 (8,648–19,786)	14,340 (8,356–19,903)	7,025 (3,230–17,261)	53 (10–134)	510 (208–1,053)	2,155 (823–4,087)	595 (196–1,284)	1,292 (501–2,360)
Yemen	Number: 2015	45,461 (41,149–50,496)	11,266 (8,669–13,782)	1,538 (861–2,426)	4,012 (2,917–5,288)	2,974 (1,715–4,580)	6,085 (3,993–9,485)	22 (3–55)	727 (324–1,334)	1,538 (623–2,868)	216 (86–449)	482 (211–791)
Yemen	Number: Cumulative change (%)	-42.4 (-47.9 to -36.2)	-23.8 (-38.2 to -7.1)	-15.9 (-59.6 to 50.1)	-70.4 (-82.1 to -52.7)	-77.5 (-89.1 to -60.4)	4.9 (-45.7 to 63.8)	-42.2 (-89.9 to 63.6)	76.2 (-37.1 to 307.0)	-14.2 (-72.3 to 85.2)	-48.3 (-89.2 to 34.5)	-54.6 (-85.4 to -0.0)
Yemen	Rate: 1990	2,813.2 (2,726.2–2,905.3)	494.5 (381.7–592.8)	66.6 (34.9–110.9)	527.6 (306.0–720.7)	513.9 (295.7–714.7)	244.0 (110.9–606.9)	1.9 (0.4–4.9)	17.1 (7.0–35.0)	72.2 (27.7–136.9)	21.6 (7.2–46.9)	47.1 (17.8–85.4)
Yemen	Rate: 2015	1,118.4 (1,012.2–1,241.5)	274.3 (211.0–335.6)	37.5 (21.0–59.1)	98.9 (72.1–130.3)	73.2 (42.3–112.6)	149.1 (97.8–232.3)	0.5 (0.1–1.4)	17.7 (7.9–32.6)	37.5 (15.2–69.9)	5.3 (2.1–11.0)	11.9 (5.2–19.5)
Yemen	Rate: Cumulative change (%)	-60.2 (-64.2 to -55.9)	-44.2 (-54.8 to -32.0)	-38.5 (-70.5 to 9.2)	-79.8 (-87.8 to -67.3)	-84.5 (-92.5 to -73.0)	-25.5 (-62.2 to 18.1)	-60.7 (-93.1 to 13.6)	28.4 (-54.1 to 195.7)	-37.6 (-79.9 to 34.9)	-65.1 (-92.6 to -8.4)	-69.1 (-90.1 to -32.1)
South Asia	Number: 1990	4,242,360 (4,202,759–4,282,274)	831,361 (757,132–909,394)	497,476 (427,049–565,767)	699,109 (631,409–755,908)	502,326 (446,268–548,765)	124,731 (87,201–157,075)	39,527 (31,706–48,524)	79,611 (48,880–116,828)	111,976 (53,420–170,094)	85,166 (63,648–108,086)	99,829 (82,240–117,797)
South Asia	Number: 2015	1,749,398 (1,691,392–1,808,100)	379,162 (339,652–428,597)	413,928 (376,848–455,579)	205,488 (186,608–227,348)	143,342 (124,888–164,137)	98,700 (74,392–115,870)	8,119 (4,502–13,343)	61,781 (37,086–84,394)	71,394 (50,245–93,100)	18,449 (14,119–24,851)	40,942 (32,858–51,854)
South Asia	Number: Cumulative change (%)	-58.8 (-60.2 to -57.4)	-54.3 (-59.5 to -48.8)	-16.3 (-27.3 to -3.2)	-70.5 (-74.0 to -66.9)	-71.4 (-75.8 to -66.3)	-18.7 (-52.0 to 0.4)	-79.1 (-89.0 to -64.7)	-19.3 (-50.6 to 19.5)	-30.3 (-59.3 to 14.0)	-77.7 (-84.6 to -66.7)	-58.5 (-67.9 to -44.5)
South Asia	Rate: 1990	2,556.3 (2,532.2–2,581.3)	485.5 (442.0–531.3)	290.3 (249.2–330.1)	426.7 (385.1–461.8)	308.2 (273.2–336.4)	74.2 (51.8–93.9)	24.7 (19.7–30.4)	46.7 (28.7–68.6)	66.2 (31.7–100.6)	52.8 (39.4–66.9)	61.5 (50.5–72.6)
South Asia	Rate: 2015	1,047.5 (1,012.8–1,082.7)	226.7 (203.1–256.3)	247.5 (225.3–272.4)	123.3 (112.0–136.4)	86.0 (74.9–98.5)	59.1 (44.6–69.4)	4.9 (2.7–8.0)	37.0 (22.2–50.6)	42.8 (30.1–55.8)	11.1 (8.5–14.9)	24.6 (19.7–31.1)
South Asia	Rate: Cumulative change (%)	-59.0 (-60.5 to -57.6)	-53.2 (-58.5 to -47.5)	-14.2 (-25.5 to -0.9)	-71.0 (-74.5 to -67.5)	-72.0 (-76.3 to -66.9)	-18.0 (-51.7 to 1.4)	-80.0 (-89.5 to -66.1)	-17.5 (-49.5 to 22.0)	-29.4 (-58.6 to 15.3)	-78.4 (-85.1 to -67.7)	-59.6 (-68.8 to -45.9)
South Asia	Number: 1990	4,242,360 (4,202,759–4,282,274)	831,361 (757,132–909,394)	497,476 (427,049–565,767)	699,109 (631,409–755,908)	502,326 (446,268–548,765)	124,731 (87,201–157,075)	39,527 (31,706–48,524)	79,611 (48,880–116,828)	111,976 (53,420–170,094)	85,166 (63,648–108,086)	99,829 (82,240–117,797)
South Asia	Number: 2015	1,749,398 (1,691,392–1,808,100)	379,162 (339,652–428,597)	413,928 (376,848–455,579)	205,488 (186,608–227,348)	143,342 (124,888–164,137)	98,700 (74,392–115,870)	8,119 (4,502–13,343)	61,781 (37,086–84,394)	71,394 (50,245–93,100)	18,449 (14,119–24,851)	40,942 (32,858–51,854)
South Asia	Number: Cumulative change (%)	-58.8 (-60.2 to -57.4)	-54.3 (-59.5 to -48.8)	-16.3 (-27.3 to -3.2)	-70.5 (-74.0 to -66.9)	-71.4 (-75.8 to -66.3)	-18.7 (-52.0 to 0.4)	-79.1 (-89.0 to -64.7)	-19.3 (-50.6 to 19.5)	-30.3 (-59.3 to 14.0)	-77.7 (-84.6 to -66.7)	-58.5 (-67.9 to -44.5)
South Asia	Rate: 1990	2,556.3 (2,532.2–2,581.3)	485.5 (442.0–531.3)	290.3 (249.2–330.1)	426.7 (385.1–461.8)	308.2 (273.2–336.4)	74.2 (51.8–93.9)	24.7 (19.7–30.4)	46.7 (28.7–68.6)	66.2 (31.7–100.6)	52.8 (39.4–66.9)	61.5 (50.5–72.6)
South Asia	Rate: 2015	1,047.5 (1,012.8–1,082.7)	226.7 (203.1–256.3)	247.5 (225.3–272.4)	123.3 (112.0–136.4)	86.0 (74.9–98.5)	59.1 (44.6–69.4)	4.9 (2.7–8.0)	37.0 (22.2–50.6)	42.8 (30.1–55.8)	11.1 (8.5–14.9)	24.6 (19.7–31.1)
South Asia	Rate: Cumulative change (%)	-59.0 (-60.5 to -57.6)	-53.2 (-58.5 to -47.5)	-14.2 (-25.5 to -0.9)	-71.0 (-74.5 to -67.5)	-72.0 (-76.3 to -66.9)	-18.0 (-51.7 to 1.4)	-80.0 (-89.5 to -66.1)	-17.5 (-49.5 to 22.0)	-29.4 (-58.6 to 15.3)	-78.4 (-85.1 to -67.7)	-59.6 (-68.8 to -45.9)
Bangladesh	Number: 1990	512,489 (503,482–522,381)	82,399 (64,204–97,741)	30,550 (21,688–39,997)	108,953 (95,910–122,530)	78,343 (65,767–91,679)	12,532 (6,870–28,802)	33 (3–83)	9,467 (3,857–17,715)	11,777 (3,387–25,599)	24,421 (12,013–40,935)	894 (419–1,970)
Bangladesh	Number: 2015	122,646 (114,779–131,560)	14,544 (10,102–20,303)	30,689 (24,480–37,189)	21,274 (17,750–25,078)	3,826 (2,661–5,311)	8,841 (5,301–15,442)	4 (1–9)	5,542 (2,713–9,372)	4,739 (1,603–9,215)	2,778 (1,590–4,349)	937 (273–1,749)
Bangladesh	Number: Cumulative change (%)	-76.1 (-77.6 to -74.3)	-82.2 (-88.1 to -75.6)	4.1 (-28.5 to 49.2)	-80.4 (-84.0 to -76.5)	-95.1 (-96.7 to -92.8)	-21.0 (-66.3 to 14.0)	-78.9 (-96.1 to -42.3)	-28.7 (-73.8 to 62.8)	-49.9 (-83.8 to 7.7)	-87.1 (-94.5 to -75.4)	48.9 (-85.3 to 222.0)
Bangladesh	Rate: 1990	3,006.2 (2,951.5–3,063.4)	465.2 (362.7–551.7)	172.0 (122.2–225.2)	642.4 (566.6–723.1)	469.5 (393.4–548.8)	72.3 (39.6–167.3)	0.2 (0.0–0.5)	53.8 (22.0–100.5)	66.8 (19.4–144.9)	147.1 (72.4–247.3)	5.4 (2.5–11.8)
Bangladesh	Rate: 2015	812.2 (760.1–871.4)	96.7 (67.2–134.9)	203.9 (162.6–247.0)	141.0 (117.5–166.3)	25.3 (17.6–35.1)	58.7 (35.2–102.4)	0.0 (0.0–0.1)	36.9 (18.1–62.4)	31.5 (10.7–61.2)	18.3 (10.4–28.7)	6.2 (1.8–11.5)
Bangladesh	Rate: Cumulative change (%)	-73.0 (-74.7 to -71.0)	-79.1 (-85.9 to -71.3)	22.8 (-15.5 to 75.9)	-78.0 (-82.1 to -73.6)	-94.6 (-96.3 to -92.1)	-9.0 (-61.3 to 31.2)	-77.1 (-95.7 to -37.1)	-16.5 (-69.3 to 89.5)	-41.4 (-80.9 to 25.4)	-85.9 (-93.9 to -73.0)	64.4 (-83.9 to 253.8)
Bhutan	Number: 1990	2,406 (2,255–2,567)	544 (420–672)	278 (172–404)	272 (171–375)	237 (99–418)	69 (46–103)	20 (7–42)	38 (17–70)	51 (21–89)	44 (14–92)	79 (34–145)
Bhutan	Number: 2015	596 (527–677)	164 (119–214)	129 (82–180)	43 (29–60)	26 (11–47)	35 (28–44)	3 (1–8)	18 (8–34)	22 (9–38)	6 (2–13)	17 (8–30)
Bhutan	Number: Cumulative change (%)	-75.2 (-78.4 to -71.7)	-69.4 (-78.2 to -59.2)	-51.3 (-70.9 to -25.1)	-83.1 (-89.7 to -73.8)	-88.0 (-93.7 to -79.7)	-45.7 (-68.5 to -19.4)	-75.3 (-94.3 to -37.8)	-44.0 (-81.4 to 33.3)	-48.9 (-83.9 to 14.3)	-80.6 (-95.8 to -47.0)	-75.2 (-89.7 to -49.4)
Bhutan	Rate: 1990	2,657.2 (2,490.8–2,835.4)	589.8 (454.5–728.7)	301.2 (187.1–437.1)	304.1 (191.3–420.4)	265.0 (110.8–467.2)	76.3 (51.4–113.8)	22.9 (8.3–47.4)	41.7 (18.9–77.6)	57.0 (23.9–98.5)	49.6 (16.2–103.3)	88.5 (38.1–163.6)
Bhutan	Rate: 2015	919.8 (813.2–1,044.5)	254.9 (185.4–332.4)	200.2 (127.7–280.0)	67.1 (45.7–92.5)	41.2 (17.5–73.2)	54.9 (43.4–69.4)	5.9 (1.9–12.9)	28.3 (12.8–52.9)	34.4 (14.7–59.5)	9.6 (3.2–20.2)	26.1 (12.8–46.0)
Bhutan	Rate: Cumulative change (%)	-65.3 (-69.8 to -60.4)	-56.3 (-68.9 to -41.7)	-30.3 (-58.4 to 7.1)	-76.7 (-85.8 to -63.8)	-83.4 (-91.4 to -71.9)	-23.6 (-55.7 to 13.5)	-66.6 (-92.2 to -15.4)	-20.2 (-73.4 to 90.0)	-27.6 (-77.3 to 61.8)	-73.6 (-94.3 to -27.6)	-66.1 (-85.8 to -31.0)
India	Number: 1990	3,156,028 (3,120,104–3,193,448)	670,447 (611,230–732,588)	363,134 (309,873–413,775)	493,335 (435,333–539,066)	337,590 (291,868–373,913)	99,459 (60,220–127,840)	37,498 (29,830–46,221)	57,409 (35,454–85,192)	83,001 (39,542–128,360)	53,472 (41,512–65,216)	76,867 (62,303–91,455)
India	Number: 2015	1,263,097 (1,208,482–1,318,032)	307,619 (275,366–345,257)	272,346 (245,766–303,641)	140,649 (125,325–158,128)	104,643 (91,780–119,218)	74,853 (50,451–91,790)	7,284 (3,886–12,125)	42,759 (25,309–59,305)	48,785 (34,339–65,375)	12,624 (9,189–17,452)	24,515 (20,045–30,302)
India	Number: Cumulative change (%)	-60.0 (-61.7 to -58.2)	-54.0 (-59.5 to -48.4)	-24.5 (-35.1 to -11.6)	-71.4 (-75.3 to -67.3)	-68.8 (-73.8 to -62.8)	-22.7 (-52.0 to -2.0)	-80.2 (-89.9 to -66.3)	-22.3 (-54.6 to 16.3)	-35.3 (-64.6 to 7.1)	-75.9 (-83.6 to -65.3)	-67.7 (-74.5 to -56.6)
India	Rate: 1990	2,508.6 (2,479.5–2,540.1)	517.5 (471.7–565.6)	280.2 (239.3–319.2)	397.6 (350.1–435.0)	272.7 (235.6–302.5)	78.1 (47.2–100.6)	30.8 (24.5–38.1)	44.5 (27.5–66.0)	64.9 (31.1–100.2)	43.6 (33.8–53.2)	62.4 (50.5–74.2)
India	Rate: 2015	1,023.2 (978.9–1,067.8)	249.3 (223.2–279.8)	220.7 (199.2–246.0)	114.0 (101.6–128.1)	84.8 (74.4–96.7)	60.7 (40.9–74.4)	5.9 (3.1–9.8)	34.7 (20.6–48.1)	39.6 (27.9–53.1)	10.2 (7.4–14.1)	19.8 (16.2–24.5)
India	Rate: Cumulative change (%)	-59.2 (-61.0 to -57.3)	-51.7 (-57.5 to -45.8)	-20.7 (-31.8 to -7.2)	-71.2 (-75.1 to -67.0)	-68.7 (-73.7 to -62.7)	-20.1 (-50.6 to 1.4)	-80.6 (-90.1 to -66.9)	-18.6 (-52.2 to 21.9)	-32.9 (-63.1 to 10.8)	-76.2 (-83.7 to -65.7)	-67.8 (-74.6 to -56.5)
Nepal	Number: 1990	97,512 (95,211–99,761)	5,701 (3,512–8,274)	12,736 (8,762–16,355)	26,182 (22,619–30,048)	13,558 (10,371–16,709)	1,365 (983–1,943)	207 (83–453)	1,518 (633–2,763)	2,092 (603–4,375)	2,050 (866–4,151)	2,333 (1,170–4,136)
Nepal	Number: 2015	21,379 (19,770–23,165)	1,810 (1,037–2,833)	5,605 (4,275–6,908)	4,362 (3,600–5,133)	1,256 (872–1,684)	981 (621–1,291)	45 (17–106)	900 (416–1,653)	864 (304–1,669)	244 (100–516)	477 (227–869)
Nepal	Number: Cumulative change (%)	-78.1 (-79.9 to -76.1)	-67.1 (-81.8 to -48.0)	-54.7 (-68.2 to -36.2)	-83.2 (-86.8 to -79.3)	-90.5 (-93.9 to -86.1)	-23.3 (-67.0 to 14.5)	-72.9 (-92.5 to -35.9)	-30.8 (-74.8 to 50.2)	-46.3 (-85.0 to 24.7)	-85.1 (-96.2 to -63.5)	-77.2 (-89.8 to -54.3)
Nepal	Rate: 1990	3,006.0 (2,934.1–3,077.9)	166.2 (102.4–241.2)	370.4 (255.3–475.5)	816.4 (700.7–939.3)	429.7 (328.5–528.4)	40.7 (29.3–58.5)	6.8 (2.7–15.0)	44.5 (18.6–80.8)	61.4 (17.9–127.9)	65.8 (27.7–133.2)	74.3 (37.1–132.6)
Nepal	Rate: 2015	767.3 (709.4–831.2)	65.3 (37.4–102.2)	202.3 (154.3–249.3)	156.6 (129.2–184.1)	44.9 (31.2–60.4)	35.4 (22.4–46.5)	1.6 (0.6–3.8)	32.5 (15.0–59.7)	31.2 (11.0–60.3)	8.7 (3.6–18.3)	17.1 (8.1–31.1)
Nepal	Rate: Cumulative change (%)	-74.5 (-76.6 to -72.2)	-59.2 (-77.4 to -35.6)	-43.8 (-60.6 to -20.9)	-80.7 (-84.8 to -76.1)	-89.3 (-93.2 to -84.2)	-7.2 (-60.8 to 39.3)	-70.6 (-92.0 to -28.3)	-14.7 (-68.9 to 84.0)	-34.1 (-81.6 to 51.6)	-83.5 (-95.7 to -60.3)	-74.3 (-88.6 to -48.6)
Pakistan	Number: 1990	473,924 (462,251–485,545)	72,269 (55,168–90,074)	90,776 (69,788–112,167)	70,364 (57,490–83,616)	72,596 (58,023–85,784)	11,306 (8,963–14,420)	1,768 (835–3,213)	11,177 (5,244–19,837)	15,052 (6,348–26,915)	5,177 (1,993–10,611)	19,655 (12,428–30,131)
Pakistan	Number: 2015	341,678 (315,755–371,034)	55,023 (38,213–73,846)	105,157 (83,448–128,075)	39,158 (31,119–48,157)	33,589 (24,802–44,073)	13,988 (10,626–17,440)	781 (349–1,459)	12,561 (5,883–22,117)	16,982 (8,294–28,089)	2,795 (1,348–5,548)	14,994 (9,655–23,475)
Pakistan	Number: Cumulative change (%)	-27.9 (-33.8 to -21.7)	-22.2 (-49.3 to 10.9)	18.1 (-13.1 to 55.7)	-43.6 (-58.2 to -26.6)	-53.0 (-67.9 to -35.4)	26.8 (-17.4 to 72.3)	-48.3 (-82.6 to 10.9)	29.1 (-53.3 to 177.0)	32.8 (-50.9 to 173.5)	-30.2 (-81.0 to 78.4)	-18.9 (-58.6 to 38.3)
Pakistan	Rate: 1990	2,393.2 (2,335.6–2,451.6)	353.0 (269.6–440.0)	443.4 (340.7–548.0)	360.6 (293.9–428.7)	375.4 (299.0–444.5)	56.4 (44.7–72.1)	9.3 (4.3–17.2)	55.0 (25.9–97.5)	74.5 (31.5–133.4)	27.2 (10.4–56.7)	102.1 (63.9–157.0)
Pakistan	Rate: 2015	1,333.3 (1,232.2–1,448.7)	212.2 (147.5–284.7)	405.4 (321.7–494.0)	154.4 (122.6–190.0)	133.0 (98.5–174.2)	54.5 (41.4–68.0)	3.1 (1.4–5.9)	48.6 (22.8–85.6)	66.0 (32.3–108.9)	11.2 (5.4–22.3)	59.4 (38.2–93.0)
Pakistan	Rate: Cumulative change (%)	-44.3 (-48.8 to -39.5)	-38.6 (-59.9 to -12.6)	-6.8 (-31.4 to 22.9)	-56.6 (-67.8 to -43.4)	-64.0 (-75.5 to -50.5)	-0.9 (-35.6 to 34.6)	-60.7 (-86.9 to -15.0)	1.6 (-63.3 to 117.7)	4.1 (-61.3 to 114.5)	-46.9 (-85.3 to 35.3)	-38.1 (-68.4 to 5.7)
Sub-Saharan Africa	Number: 1990	3,674,843 (3,641,175–3,710,357)	196,966 (170,440–226,421)	176,950 (145,437–209,038)	500,699 (427,900–573,245)	494,945 (409,640–579,416)	119,651 (84,222–194,696)	647,580 (454,586–854,764)	166,751 (126,304–217,525)	103,005 (74,810–138,772)	243,557 (181,664–313,379)	133,501 (100,304–168,530)
Sub-Saharan Africa	Number: 2015	2,863,719 (2,770,390–2,964,026)	200,994 (175,652–229,643)	215,210 (179,820–256,225)	340,225 (307,807–377,040)	303,045 (265,936–342,155)	170,503 (145,334–208,453)	461,388 (344,786–576,373)	229,956 (180,234–292,799)	92,012 (68,864–120,339)	146,313 (112,542–187,947)	109,292 (83,617–141,840)
Sub-Saharan Africa	Number: Cumulative change (%)	-22.1 (-24.8 to -19.0)	2.5 (-11.7 to 19.0)	22.5 (2.0–47.8)	-31.6 (-41.7 to -20.2)	-38.2 (-49.4 to -24.6)	49.8 (5.1–89.3)	-27.0 (-46.5 to -2.3)	39.8 (9.6–79.0)	-8.6 (-35.1 to 26.5)	-38.6 (-57.0 to -16.7)	-16.8 (-36.9 to 12.6)
Sub-Saharan Africa	Rate: 1990	3,912.2 (3,875.7–3,949.3)	191.2 (165.4–219.7)	171.6 (140.9–202.8)	529.9 (450.8–608.9)	534.6 (440.8–627.4)	122.3 (85.0–201.3)	717.6 (503.9–947.6)	162.4 (123.1–211.5)	100.5 (73.1–135.4)	269.7 (200.9–348.3)	143.1 (107.1–181.2)
Sub-Saharan Africa	Rate: 2015	1,748.9 (1,691.2–1,810.9)	118.4 (103.5–135.3)	126.8 (106.0–150.9)	207.9 (188.1–230.9)	187.1 (164.0–211.2)	102.8 (87.4–126.0)	289.5 (216.7–361.4)	135.7 (106.4–172.7)	54.3 (40.7–71.0)	91.3 (70.2–117.3)	67.2 (51.4–87.4)
Sub-Saharan Africa	Rate: Cumulative change (%)	-55.3 (-56.9 to -53.6)	-37.8 (-46.4 to -27.8)	-25.6 (-38.1 to -10.3)	-60.5 (-66.4 to -53.7)	-64.6 (-71.2 to -56.7)	-11.3 (-38.6 to 12.8)	-58.7 (-69.6 to -44.8)	-15.3 (-33.6 to 8.3)	-44.7 (-60.7 to -23.4)	-65.4 (-75.8 to -52.9)	-52.2 (-63.9 to -34.8)
Southern Sub-Saharan Africa	Number: 1990	121,198 (117,515–125,301)	11,693 (10,224–13,256)	7,794 (6,684–8,986)	19,235 (17,868–20,588)	28,321 (26,495–30,294)	4,251 (3,747–5,058)	605 (296–967)	3,686 (2,662–5,010)	12,584 (11,251–13,764)	5,849 (4,930–6,931)	1,944 (1,663–2,252)
Southern Sub-Saharan Africa	Number: 2015	83,413 (78,384–88,687)	10,049 (8,763–11,333)	9,157 (7,684–10,766)	7,603 (6,604–8,630)	9,964 (8,477–11,539)	3,905 (3,339–4,443)	634 (214–1,313)	4,274 (3,019–5,932)	6,571 (5,670–7,470)	2,792 (1,939–3,934)	1,172 (852–1,667)
Southern Sub-Saharan Africa	Number: Cumulative change (%)	-31.2 (-35.8 to -26.2)	-13.6 (-27.0 to 1.8)	18.3 (-5.6 to 43.7)	-60.4 (-66.4 to -53.8)	-64.8 (-70.5 to -58.7)	-7.2 (-29.3 to 9.6)	3.7 (-48.6 to 92.0)	18.0 (-16.1 to 61.5)	-47.6 (-55.0 to -39.1)	-52.0 (-66.8 to -33.8)	-39.2 (-56.8 to -11.3)
Southern Sub-Saharan Africa	Rate: 1990	1,540.4 (1,493.1–1,592.6)	146.5 (128.1–166.1)	97.7 (83.8–112.6)	244.9 (227.3–262.1)	361.8 (338.5–387.1)	53.8 (47.4–64.1)	7.8 (3.8–12.4)	46.3 (33.4–62.8)	157.8 (141.1–172.6)	75.3 (63.4–89.2)	24.8 (21.3–28.8)
Southern Sub-Saharan Africa	Rate: 2015	930.7 (874.4–989.7)	111.4 (97.2–125.7)	101.5 (85.2–119.4)	84.9 (73.7–96.4)	111.6 (95.0–129.3)	43.5 (37.2–49.5)	7.1 (2.4–14.8)	47.4 (33.5–65.8)	72.9 (62.9–82.8)	31.4 (21.8–44.3)	13.1 (9.6–18.7)
Southern Sub-Saharan Africa	Rate: Cumulative change (%)	-39.6 (-43.6 to -35.2)	-23.5 (-35.4 to -9.9)	4.7 (-16.4 to 27.2)	-65.2 (-70.5 to -59.5)	-69.1 (-74.1 to -63.7)	-18.4 (-37.8 to -3.5)	-9.1 (-54.9 to 68.5)	4.3 (-25.8 to 42.6)	-53.7 (-60.2 to -46.1)	-58.0 (-70.9 to -42.1)	-46.7 (-62.1 to -22.4)
Botswana	Number: 1990	2,563 (2,382–2,755)	275 (175–389)	166 (94–257)	371 (290–455)	621 (485–742)	89 (69–114)	11 (3–22)	72 (29–134)	279 (160–405)	120 (56–215)	32 (16–53)
Botswana	Number: 2015	1,553 (1,268–1,905)	209 (132–301)	152 (87–236)	114 (68–171)	144 (82–226)	82 (56–118)	4 (0–11)	77 (36–140)	203 (111–314)	37 (13–74)	13 (6–22)
Botswana	Number: Cumulative change (%)	-39.3 (-51.3 to -24.6)	-20.9 (-53.1 to 21.4)	-0.7 (-50.8 to 75.9)	-68.6 (-81.3 to -50.2)	-76.5 (-86.7 to -62.2)	-5.7 (-39.6 to 35.2)	-66.8 (-89.5 to -28.1)	27.0 (-55.7 to 184.0)	-22.3 (-62.3 to 34.5)	-64.7 (-89.8 to -19.1)	-55.7 (-82.9 to -7.8)
Botswana	Rate: 1990	1,123.1 (1,043.7–1,206.9)	120.3 (76.7–170.4)	72.6 (41.5–112.3)	162.7 (127.5–199.4)	272.8 (212.9–325.8)	39.0 (30.3–50.1)	4.9 (1.7–9.8)	31.5 (12.8–58.8)	122.3 (70.2–177.1)	52.8 (25.0–94.8)	14.4 (7.2–23.4)
Botswana	Rate: 2015	582.7 (475.8–714.3)	78.3 (49.7–112.9)	57.1 (32.6–88.3)	43.0 (25.8–64.4)	54.4 (31.2–85.2)	31.0 (21.1–44.5)	1.5 (0.2–4.4)	29.0 (13.5–52.5)	76.3 (41.8–117.5)	14.2 (5.1–28.2)	4.9 (2.3–8.6)
Botswana	Rate: Cumulative change (%)	-48.0 (-58.2 to -35.4)	-32.3 (-59.8 to 3.9)	-15.0 (-57.8 to 50.6)	-73.1 (-83.9 to -57.4)	-79.8 (-88.5 to -67.5)	-19.3 (-48.3 to 15.8)	-71.3 (-90.9 to -37.8)	8.6 (-62.1 to 142.6)	-33.5 (-67.7 to 15.0)	-69.6 (-91.2 to -30.3)	-61.9 (-85.3 to -20.7)
Lesotho	Number: 1990	5,170 (4,950–5,390)	629 (413–862)	328 (181–515)	930 (757–1,100)	1,334 (1,074–1,547)	118 (79–185)	0 (0–0)	117 (48–230)	626 (342–938)	251 (118–437)	66 (33–108)
Lesotho	Number: 2015	5,040 (4,360–5,848)	660 (419–905)	405 (225–622)	680 (501–893)	803 (583–1,049)	141 (103–196)	0 (0–0)	160 (70–296)	571 (317–884)	164 (77–306)	56 (29–98)
Lesotho	Number: Cumulative change (%)	-2.4 (-16.9 to 13.2)	9.6 (-37.8 to 69.6)	34.7 (-36.7 to 143.4)	-25.9 (-48.3 to 2.1)	-39.0 (-57.3 to -16.3)	25.4 (-17.2 to 80.5)	nan (nan–nan)	60.8 (-44.9 to 258.4)	-0.9 (-54.5 to 79.6)	-24.7 (-73.3 to 60.6)	-5.6 (-59.6 to 91.8)
Lesotho	Rate: 1990	1,955.4 (1,872.4–2,039.2)	233.8 (153.6–320.1)	122.1 (67.4–191.4)	353.3 (287.2–418.0)	509.6 (409.3–591.0)	44.7 (29.8–69.9)	0.0 (0.0–0.0)	43.9 (18.2–85.5)	233.1 (127.2–348.9)	96.6 (45.5–168.0)	25.5 (12.7–41.2)
Lesotho	Rate: 2015	1,775.8 (1,533.5–2,060.4)	228.2 (144.9–312.5)	140.0 (78.0–214.8)	240.5 (177.4–316.3)	287.1 (208.5–373.9)	49.6 (36.5–68.7)	0.0 (0.0–0.0)	55.6 (24.4–102.6)	197.3 (109.9–305.4)	59.6 (28.1–110.4)	20.2 (10.5–35.0)
Lesotho	Rate: Cumulative change (%)	-9.1 (-22.7 to 5.5)	1.9 (-42.1 to 57.8)	25.2 (-41.1 to 125.5)	-31.0 (-51.9 to -5.1)	-43.0 (-60.1 to -21.6)	16.6 (-23.1 to 67.9)	nan (nan–nan)	49.2 (-48.7 to 231.5)	-7.9 (-57.6 to 66.5)	-29.2 (-75.0 to 52.0)	-11.7 (-62.5 to 78.7)
Namibia	Number: 1990	3,749 (3,542–3,962)	407 (268–567)	231 (126–365)	529 (411–647)	893 (692–1,069)	102 (75–141)	156 (51–301)	101 (44–191)	431 (243–629)	182 (87–330)	47 (25–75)
Namibia	Number: 2015	2,895 (2,319–3,548)	410 (268–577)	285 (162–439)	301 (201–418)	405 (257–583)	125 (91–168)	61 (12–143)	131 (58–251)	388 (206–628)	97 (39–185)	32 (16–55)
Namibia	Number: Cumulative change (%)	-22.7 (-38.4 to -4.7)	4.6 (-38.0 to 63.3)	34.6 (-35.9 to 141.0)	-41.9 (-63.9 to -13.6)	-53.7 (-71.9 to -29.4)	26.3 (-17.7 to 80.3)	-55.0 (-89.4 to 8.8)	50.4 (-47.1 to 234.5)	-3.6 (-56.1 to 68.1)	-37.9 (-80.3 to 36.5)	-24.3 (-70.4 to 59.4)
Namibia	Rate: 1990	1,518.1 (1,434.4–1,603.8)	159.3 (104.7–221.8)	90.6 (49.5–142.6)	214.9 (165.6–262.2)	367.2 (283.6–440.9)	40.8 (30.1–56.4)	65.2 (21.6–125.4)	39.8 (17.6–74.8)	168.8 (95.4–246.0)	76.6 (36.6–138.9)	19.7 (10.4–31.3)
Namibia	Rate: 2015	845.5 (677.4–1,036.2)	118.2 (77.3–166.3)	82.4 (46.8–126.7)	88.3 (59.0–122.5)	119.7 (76.6–172.3)	36.6 (26.5–49.1)	18.3 (3.6–42.8)	38.0 (16.8–72.5)	112.0 (59.6–181.0)	29.2 (11.8–55.7)	9.5 (4.9–16.6)
Namibia	Rate: Cumulative change (%)	-44.2 (-55.7 to -31.2)	-22.8 (-54.2 to 20.5)	-0.7 (-52.6 to 77.7)	-58.0 (-74.0 to -37.3)	-66.8 (-79.7 to -49.3)	-7.8 (-40.1 to 31.5)	-67.8 (-92.4 to -22.1)	10.7 (-61.0 to 146.2)	-28.9 (-67.6 to 23.8)	-55.7 (-86.0 to -1.1)	-45.6 (-78.8 to 15.5)
South Africa	Number: 1990	81,793 (78,184–85,732)	7,490 (6,707–8,310)	4,343 (3,771–4,922)	13,898 (12,805–15,017)	20,225 (18,828–21,861)	2,823 (2,481–3,352)	57 (25–112)	1,893 (1,376–2,540)	10,301 (9,272–11,236)	3,688 (3,167–4,288)	1,175 (975–1,336)
South Africa	Number: 2015	42,539 (39,279–46,004)	5,483 (4,912–6,052)	3,899 (3,479–4,384)	3,306 (2,726–3,967)	3,026 (2,416–3,759)	2,109 (1,601–2,524)	5 (2–11)	1,720 (1,274–2,197)	4,578 (3,955–5,114)	824 (654–1,021)	316 (249–410)
South Africa	Number: Cumulative change (%)	-48.0 (-52.6 to -42.8)	-26.5 (-36.1 to -16.0)	-9.6 (-24.7 to 9.5)	-76.1 (-80.9 to -70.8)	-85.0 (-88.2 to -81.0)	-24.3 (-47.9 to -6.0)	-87.6 (-96.6 to -70.3)	-8.5 (-22.8 to 5.7)	-55.4 (-61.8 to -48.6)	-77.5 (-82.8 to -71.7)	-72.6 (-79.6 to -59.0)
South Africa	Rate: 1990	1,598.3 (1,527.5–1,676.1)	144.5 (129.4–160.3)	83.8 (72.7–94.9)	272.1 (250.7–294.0)	397.1 (369.7–429.5)	55.0 (48.3–65.3)	1.1 (0.5–2.2)	36.6 (26.6–49.1)	198.9 (179.0–216.9)	72.8 (62.5–84.7)	23.1 (19.1–26.3)
South Africa	Rate: 2015	796.3 (735.2–861.1)	102.6 (91.9–113.2)	72.9 (65.1–82.0)	61.9 (51.1–74.3)	56.7 (45.3–70.4)	39.5 (30.0–47.2)	0.1 (0.0–0.2)	32.2 (23.8–41.1)	85.6 (74.0–95.7)	15.5 (12.3–19.2)	5.9 (4.7–7.7)
South Africa	Rate: Cumulative change (%)	-50.1 (-54.6 to -45.2)	-28.8 (-38.0 to -18.6)	-12.3 (-27.0 to 6.2)	-77.2 (-81.7 to -72.1)	-85.7 (-88.8 to -81.9)	-27.2 (-49.9 to -9.7)	-88.3 (-96.8 to -71.9)	-11.4 (-25.3 to 2.3)	-56.8 (-63.0 to -50.2)	-78.6 (-83.6 to -73.1)	-73.9 (-80.5 to -60.9)

© 2017 Global Burden of Disease Child and Adolescent Health Collaboration. JAMA Pediatrics.

eTable 2. Number of Deaths, Death Rates (per 100,000 population), and Cumulative Percent Change with 95% Uncertainty Intervals (UI) for the Top 10 Global Causes of Death in 195 Countries and Territories, Aged Under 5 Years, Both Sexes, 1990 and 2015 *Note: best viewed by enlarging in browser.*

Location	Measure	All causes	Neonatal preterm birth complications	Neonatal encephalopathy due to birth asphyxia and trauma	Lower respiratory infections	Diarrheal diseases	Congenital anomalies	Malaria	Neonatal sepsis and other neonatal infections	Other neonatal disorders	Protein-energy malnutrition	Meningitis
Swaziland	Number: 1990	2,568 (2,438–2,714)	214 (139–293)	129 (76–195)	474 (381–569)	714 (574–843)	74 (56–101)	0 (0–0)	62 (28–116)	231 (138–330)	128 (58–226)	42 (20–69)
Swaziland	Number: 2015	2,064 (1,738–2,421)	202 (140–270)	140 (82–211)	303 (216–409)	362 (242–485)	70 (51–91)	0 (0–0)	76 (35–137)	204 (118–304)	76 (32–144)	30 (15–50)
Swaziland	Number: Cumulative change (%)	-19.6 (-32.6 to -4.3)	-1.5 (-38.9 to 52.7)	17.0 (-41.8 to 108.6)	-35.1 (-57.1 to -8.4)	-48.6 (-67.2 to -27.5)	-3.9 (-30.8 to 27.8)	-54.5 (-92.1 to 27.2)	41.4 (-45.5 to 193.5)	-6.6 (-52.1 to 52.1)	-31.3 (-77.7 to 55.9)	-18.6 (-66.7 to 68.1)
Swaziland	Rate: 1990	1,486.1 (1,409.9–1,571.3)	121.7 (79.3–166.3)	73.6 (43.4–110.5)	274.2 (220.6–329.9)	415.5 (334.2–491.0)	42.6 (32.5–58.2)	0.1 (0.1–0.2)	35.5 (16.3–66.0)	131.5 (78.5–187.4)	75.8 (34.6–132.2)	24.5 (12.1–40.6)
Swaziland	Rate: 2015	1,153.0 (970.7–1,354.1)	111.8 (77.7–149.2)	77.8 (45.5–116.5)	169.7 (121.2–229.3)	203.3 (135.9–273.1)	39.0 (28.8–50.9)	0.1 (0.0–0.2)	42.5 (19.4–75.7)	112.7 (65.2–167.9)	43.2 (18.3–81.4)	17.1 (8.5–28.2)
Swaziland	Rate: Cumulative change (%)	-22.3 (-35.0 to -7.5)	-4.0 (-40.5 to 48.8)	13.9 (-43.3 to 102.9)	-37.2 (-58.6 to -11.5)	-50.4 (-68.3 to -29.8)	-6.7 (-33.0 to 24.3)	-56.2 (-92.4 to 22.8)	37.7 (-46.8 to 185.9)	-9.0 (-53.3 to 48.1)	-34.0 (-78.7 to 48.3)	-21.5 (-68.0 to 59.6)
Zimbabwe	Number: 1990	25,352 (24,558–26,144)	2,675 (1,845–3,642)	2,593 (1,775–3,504)	3,031 (2,499–3,679)	4,533 (3,693–5,388)	1,043 (844–1,293)	380 (161–655)	1,439 (811–2,276)	712 (372–1,207)	1,478 (885–2,188)	579 (392–835)
Zimbabwe	Number: 2015	29,319 (26,293–32,852)	3,083 (2,142–4,107)	4,272 (3,027–5,643)	2,896 (2,238–3,670)	5,222 (4,020–6,655)	1,375 (1,090–1,697)	563 (180–1,188)	2,107 (1,226–3,389)	625 (260–1,168)	1,591 (798–2,647)	723 (424–1,201)
Zimbabwe	Number: Cumulative change (%)	15.7 (3.2–29.8)	20.5 (-27.7 to 84.5)	70.6 (7.3–147.4)	-3.2 (-30.9 to 28.5)	16.7 (-15.1 to 55.8)	33.3 (3.3–68.5)	46.9 (-30.2 to 173.7)	58.9 (-25.3 to 186.8)	-0.7 (-67.1 to 111.0)	14.7 (-48.2 to 112.2)	31.2 (-34.0 to 142.8)
Zimbabwe	Rate: 1990	1,379.5 (1,335.1–1,422.4)	143.5 (99.0–195.3)	139.1 (95.2–187.9)	165.1 (136.2–200.4)	248.1 (202.1–294.8)	56.4 (45.7–70.0)	20.9 (8.9–36.0)	77.3 (43.6–122.2)	38.2 (20.0–64.9)	81.6 (48.7–120.4)	31.6 (21.4–45.8)
Zimbabwe	Rate: 2015	1,151.6 (1,032.0–1,290.6)	119.2 (82.9–158.8)	165.2 (117.1–218.1)	114.0 (88.0–144.7)	206.6 (159.3–263.5)	53.7 (42.6–66.1)	22.6 (7.2–47.5)	81.6 (47.5–131.2)	24.2 (10.1–45.2)	63.6 (32.0–105.5)	28.6 (16.7–47.5)
Zimbabwe	Rate: Cumulative change (%)	-16.5 (-25.5 to -6.3)	-13.1 (-47.9 to 33.0)	23.0 (-22.7 to 78.3)	-30.0 (-50.1 to -7.1)	-15.6 (-38.6 to 12.7)	-3.8 (-25.4 to 21.7)	6.9 (-49.3 to 98.7)	14.5 (-46.0 to 106.6)	-28.4 (-76.2 to 52.1)	-16.8 (-62.9 to 53.1)	-5.0 (-52.3 to 75.6)
Western Sub-Saharan Africa	Number: 1990	1,693,752 (1,670,829–1,716,156)	84,305 (66,917–103,056)	81,707 (62,235–103,618)	215,092 (176,677–253,879)	232,844 (186,393–280,760)	50,999 (30,014–98,187)	336,350 (227,299–456,062)	96,745 (68,550–130,656)	32,635 (20,348–48,474)	119,692 (77,986–177,324)	67,494 (48,759–89,363)
Western Sub-Saharan Africa	Number: 2015	1,473,420 (1,401,460–1,550,687)	93,613 (74,484–114,561)	105,859 (80,488–135,229)	151,799 (131,996–174,235)	184,304 (154,940–218,764)	67,192 (46,494–108,462)	326,147 (252,465–402,705)	141,738 (104,869–184,083)	29,955 (19,426–45,545)	70,189 (47,606–101,018)	58,359 (41,647–80,898)
Western Sub-Saharan Africa	Number: Cumulative change (%)	-13.0 (-17.3 to -8.6)	12.2 (-13.5 to 39.3)	31.6 (-3.3 to 72.7)	-28.8 (-41.3 to -13.7)	-19.8 (-37.2 to 2.1)	39.0 (3.3–84.9)	0.0 (-27.6 to 36.9)	49.8 (7.6–106.9)	-3.4 (-47.0 to 60.2)	-38.3 (-64.6 to -3.8)	-11.7 (-38.9 to 26.6)
Western Sub-Saharan Africa	Rate: 1990	4,478.4 (4,416.2–4,541.6)	199.8 (158.6–244.4)	193.6 (147.4–245.1)	564.5 (461.4–667.9)	626.2 (497.9–758.6)	128.4 (74.2–251.3)	931.7 (633.6–1,259.7)	229.9 (162.8–309.9)	78.0 (48.8–115.6)	330.9 (214.2–492.1)	179.3 (128.6–238.4)
Western Sub-Saharan Africa	Rate: 2015	2,133.3 (2,028.8–2,245.2)	129.4 (103.0–158.4)	146.3 (111.3–186.9)	219.6 (190.4–252.2)	269.8 (226.3–320.6)	95.5 (65.7–154.9)	486.8 (376.6–601.1)	196.2 (145.3–254.7)	41.6 (27.0–63.2)	104.2 (70.8–149.9)	85.1 (60.7–118.5)
Western Sub-Saharan Africa	Rate: Cumulative change (%)	-52.4 (-54.7 to -49.9)	-34.6 (-49.5 to -18.8)	-23.2 (-43.5 to 0.8)	-60.7 (-67.8 to -52.2)	-56.3 (-65.9 to -44.1)	-21.1 (-42.2 to 5.5)	-46.1 (-60.9 to -26.5)	-12.8 (-37.3 to 20.0)	-43.9 (-69.1 to -7.2)	-66.8 (-81.1 to -48.6)	-51.5 (-66.8 to -29.9)
Benin	Number: 1990	40,605 (39,610–41,632)	2,499 (1,691–3,440)	2,055 (1,243–3,003)	7,017 (4,652–9,091)	5,455 (3,275–7,457)	1,379 (738–2,662)	4,770 (2,010–7,905)	2,301 (1,288–3,414)	723 (356–1,312)	3,791 (1,642–6,567)	1,840 (965–2,965)
Benin	Number: 2015	29,719 (27,060–32,775)	2,560 (1,651–3,556)	2,359 (1,458–3,421)	4,026 (3,074–5,115)	2,279 (1,467–3,173)	1,597 (1,088–2,437)	6,218 (4,027–8,539)	2,784 (1,600–4,131)	580 (256–1,153)	1,653 (713–3,046)	1,309 (729–2,186)
Benin	Number: Cumulative change (%)	-26.8 (-33.2 to -19.3)	5.9 (-35.5 to 61.8)	21.6 (-35.0 to 99.4)	-39.7 (-60.0 to -11.0)	-54.8 (-75.7 to -26.4)	27.1 (-21.9 to 90.9)	55.6 (-27.2 to 197.1)	30.6 (-35.0 to 133.1)	-7.3 (-69.1 to 110.2)	-44.7 (-82.6 to 8.1)	-19.9 (-65.0 to 57.2)
Benin	Rate: 1990	4,092.2 (3,988.3–4,204.8)	225.9 (153.0–310.5)	185.8 (112.4–270.9)	706.0 (461.0–917.0)	560.3 (333.8–769.9)	133.0 (69.5–259.8)	508.4 (214.0–840.8)	208.6 (116.7–308.6)	66.0 (32.8–118.4)	400.9 (173.7–704.3)	186.7 (96.7–304.3)
Benin	Rate: 2015	1,654.0 (1,505.5–1,825.9)	138.0 (89.2–191.7)	127.2 (78.6–184.4)	224.2 (170.8–285.3)	127.9 (82.5–178.6)	87.9 (59.7–134.5)	354.9 (230.0–486.6)	150.3 (86.5–223.0)	31.4 (13.9–62.2)	93.7 (40.5–172.3)	73.2 (40.7–122.7)
Benin	Rate: Cumulative change (%)	-59.6 (-63.1 to -55.4)	-36.9 (-61.5 to -3.2)	-27.5 (-61.1 to 18.4)	-66.5 (-78.0 to -50.2)	-75.2 (-86.8 to -59.7)	-27.0 (-56.1 to 10.8)	-16.4 (-61.1 to 59.5)	-22.3 (-61.3 to 38.0)	-45.2 (-81.6 to 24.2)	-70.3 (-90.8 to -41.5)	-55.6 (-80.9 to -12.6)
Burkina Faso	Number: 1990	84,298 (82,511–86,253)	2,021 (1,317–2,934)	3,387 (2,049–4,947)	13,476 (10,520–16,802)	14,054 (10,067–18,362)	2,640 (1,520–5,164)	22,147 (14,239–30,054)	3,485 (2,030–5,222)	1,231 (568–2,227)	3,454 (1,393–6,894)	3,656 (2,080–5,711)
Burkina Faso	Number: 2015	77,490 (71,471–84,385)	3,138 (2,039–4,499)	4,552 (2,804–6,552)	10,071 (7,653–12,730)	7,863 (5,522–10,703)	3,668 (2,290–6,178)	24,856 (18,414–31,532)	5,161 (3,017–7,637)	1,114 (528–2,062)	4,017 (1,778–7,414)	3,804 (2,279–5,876)
Burkina Faso	Number: Cumulative change (%)	-8.1 (-15.2 to 0.3)	63.5 (-3.0 to 163.2)	41.6 (-20.2 to 127.4)	-24.0 (-45.5 to 3.2)	-42.4 (-63.2 to -15.6)	45.7 (2.3–98.0)	16.2 (-20.0 to 63.4)	57.7 (-15.2 to 167.2)	4.8 (-64.1 to 120.1)	45.4 (-57.7 to 237.2)	12.5 (-44.8 to 96.6)
Burkina Faso	Rate: 1990	4,798.8 (4,692.0–4,919.0)	101.7 (66.8–147.7)	170.3 (103.6–248.3)	755.4 (584.5–946.4)	810.1 (575.0–1,069.1)	141.6 (80.7–283.0)	1,321.1 (847.8–1,789.8)	175.7 (102.6–262.9)	62.4 (28.9–112.1)	206.5 (83.6–409.7)	207.6 (115.8–330.3)
Burkina Faso	Rate: 2015	2,388.6 (2,203.0–2,600.8)	91.6 (59.5–131.3)	132.8 (82.1–191.0)	308.4 (234.7–391.5)	244.1 (171.7–331.8)	110.2 (68.3–187.5)	787.0 (583.4–998.9)	150.8 (88.3–223.1)	32.7 (15.5–60.2)	126.1 (55.1–231.0)	117.5 (69.8–182.2)
Burkina Faso	Rate: Cumulative change (%)	-50.2 (-54.2 to -45.5)	-5.3 (-43.7 to 52.6)	-17.8 (-53.6 to 31.7)	-58.4 (-70.6 to -43.0)	-68.9 (-80.3 to -54.6)	-17.9 (-43.1 to 12.8)	-38.4 (-57.6 to -13.4)	-8.7 (-50.7 to 53.8)	-39.5 (-79.2 to 26.9)	-23.4 (-77.9 to 76.9)	-38.5 (-70.4 to 9.2)
Cameroon	Number: 1990	72,001 (70,143–74,029)	3,867 (2,538–5,490)	3,736 (2,263–5,559)	9,659 (6,745–12,588)	11,106 (7,007–15,224)	2,892 (1,845–4,701)	9,819 (5,249–14,628)	5,056 (2,949–7,341)	1,473 (705–2,580)	4,514 (1,955–8,296)	2,344 (1,313–3,921)
Cameroon	Number: 2015	75,042 (66,569–84,877)	5,397 (3,376–7,671)	5,413 (3,290–7,899)	9,540 (7,078–12,537)	6,258 (4,045–9,062)	4,384 (2,946–6,836)	15,045 (8,836–21,378)	7,948 (4,708–11,458)	1,576 (679–2,925)	3,277 (1,365–6,490)	2,527 (1,444–4,243)
Cameroon	Number: Cumulative change (%)	4.3 (-7.9 to 17.9)	45.4 (-16.5 to 126.4)	54.7 (-19.2 to 162.6)	2.3 (-32.3 to 49.8)	-40.5 (-65.9 to -0.5)	56.5 (9.1–119.9)	65.5 (-1.2 to 176.5)	65.4 (-7.5 to 165.6)	21.9 (-60.3 to 153.1)	-10.9 (-74.6 to 108.6)	19.3 (-45.9 to 123.3)
Cameroon	Rate: 1990	3,011.6 (2,930.6–3,100.5)	150.5 (98.8–213.5)	145.4 (88.4–216.2)	402.9 (279.3–525.7)	470.9 (297.7–649.9)	117.3 (74.3–192.9)	428.4 (228.6–638.4)	197.1 (115.3–286.6)	57.7 (27.7–101.0)	195.6 (84.5–365.3)	98.6 (54.0–164.7)
Cameroon	Rate: 2015	1,917.0 (1,699.3–2,170.0)	133.5 (83.5–189.7)	133.8 (81.4–195.2)	243.8 (180.1–320.6)	161.2 (103.5–232.9)	110.7 (74.3–173.9)	394.3 (231.8–561.5)	196.8 (116.8–283.3)	39.1 (16.9–72.5)	85.4 (35.2–169.3)	64.8 (36.9–109.4)
Cameroon	Rate: Cumulative change (%)	-36.3 (-44.0 to -27.9)	-7.7 (-46.9 to 43.9)	-1.7 (-48.6 to 66.8)	-37.2 (-58.7 to -7.6)	-63.7 (-79.4 to -39.8)	-2.4 (-32.5 to 37.3)	-0.5 (-40.5 to 66.7)	5.0 (-41.2 to 67.9)	-22.8 (-74.7 to 61.1)	-46.3 (-84.7 to 26.1)	-27.0 (-67.5 to 38.3)
Cape Verde	Number: 1990	786 (757–816)	96 (69–120)	56 (34–83)	84 (63–108)	163 (116–205)	71 (54–92)	0 (0–0)	55 (28–83)	7 (3–15)	48 (22–87)	22 (11–41)
Cape Verde	Number: 2015	309 (282–338)	51 (38–66)	43 (29–58)	30 (23–38)	9 (6–14)	43 (34–55)	0 (0–0)	45 (27–64)	7 (3–13)	5 (2–9)	4 (2–7)
Cape Verde	Number: Cumulative change (%)	-60.7 (-64.4 to -56.6)	-45.0 (-62.8 to -21.4)	-18.2 (-53.0 to 40.7)	-63.4 (-74.5 to -48.6)	-93.9 (-96.2 to -90.5)	-38.2 (-56.6 to -12.6)	nan (nan–nan)	-10.1 (-55.4 to 59.8)	17.7 (-62.8 to 165.7)	-87.2 (-96.1 to -72.5)	-74.2 (-90.5 to -52.1)
Cape Verde	Rate: 1990	1,195.5 (1,151.2–1,240.7)	145.3 (104.9–182.2)	85.0 (52.3–125.4)	128.1 (96.7–164.2)	249.7 (176.6–313.3)	108.4 (82.8–140.7)	0.0 (0.0–0.0)	83.3 (43.2–125.9)	11.9 (4.9–22.8)	73.8 (35.0–132.7)	34.8 (17.4–63.7)
Cape Verde	Rate: 2015	582.5 (531.3–636.5)	97.8 (72.0–125.9)	81.4 (56.2–109.6)	56.4 (43.7–71.8)	18.3 (12.1–26.4)	81.2 (64.5–104.2)	0.0 (0.0–0.0)	85.3 (51.1–121.1)	14.1 (7.2–25.0)	9.7 (4.4–18.5)	9.4 (5.4–14.3)
Cape Verde	Rate: Cumulative change (%)	-51.2 (-55.9 to -46.2)	-31.0 (-53.3 to -1.4)	2.5 (-41.0 to 76.2)	-54.8 (-68.6 to -36.5)	-92.5 (-95.3 to -88.3)	-23.2 (-46.1 to 8.6)	nan (nan–nan)	12.7 (-44.0 to 100.3)	47.3 (-53.4 to 232.8)	-84.3 (-95.2 to -66.4)	-68.2 (-88.4 to -40.8)
Chad	Number: 1990	58,750 (57,250–60,363)	2,736 (1,803–3,725)	2,052 (1,081–3,217)	9,256 (6,981–11,529)	11,655 (8,361–15,118)	1,424 (790–2,853)	4,854 (1,811–9,770)	2,738 (1,441–4,142)	1,255 (416–2,454)	4,109 (1,817–7,368)	2,241 (1,274–3,639)
Chad	Number: 2015	80,120 (72,569–88,895)	5,116 (3,465–7,174)	4,073 (2,421–6,105)	13,589 (10,235–17,533)	15,781 (11,143–20,808)	3,137 (1,764–5,878)	7,859 (2,402–16,142)	5,705 (3,442–8,266)	1,466 (667–2,714)	4,954 (2,129–9,137)	3,991 (2,261–6,469)
Chad	Number: Cumulative change (%)	36.4 (23.3–51.1)	94.0 (22.2–195.4)	115.3 (13.7–278.9)	49.6 (6.0–102.3)	39.0 (-9.9 to 100.8)	126.3 (59.1–213.2)	68.3 (-19.4 to 190.3)	127.2 (20.7–306.2)	44.2 (-50.9 to 198.3)	42.7 (-52.1 to 221.7)	94.1 (-10.5 to 258.5)
Chad	Rate: 1990	4,642.1 (4,506.1–4,784.0)	189.8 (125.7–257.9)	142.4 (75.1–223.2)	726.3 (542.3–917.7)	937.6 (668.3–1,222.8)	106.4 (57.4–216.9)	409.0 (153.8–818.5)	190.6 (100.2–287.1)	87.8 (29.4–171.6)	342.5 (149.3–625.9)	177.3 (99.4–290.4)
Chad	Rate: 2015	2,866.6 (2,598.7–3,179.6)	170.6 (115.5–239.0)	135.8 (80.9–203.1)	487.4 (364.0–629.9)	573.9 (402.8–757.6)	109.6 (61.2–207.3)	293.7 (90.5–603.0)	190.6 (115.3–275.7)	49.2 (22.5–90.7)	183.6 (79.0–341.8)	144.1 (80.5–237.1)
Chad	Rate: Cumulative change (%)	-38.2 (-44.4 to -31.6)	-6.8 (-41.2 to 41.4)	3.5 (-45.1 to 81.8)	-31.5 (-51.8 to -6.9)	-37.1 (-59.6 to -8.2)	6.3 (-25.3 to 47.8)	-25.3 (-64.3 to 28.8)	9.0 (-42.1 to 93.9)	-31.0 (-76.4 to 41.9)	-36.2 (-79.2 to 46.3)	-11.0 (-60.0 to 65.7)
Cote d'Ivoire	Number: 1990	75,978 (73,898–78,152)	5,507 (3,681–7,580)	4,478 (2,565–6,759)	9,708 (6,875–12,374)	9,132 (6,312–11,951)	2,831 (1,657–5,017)	16,762 (11,251–21,905)	5,868 (3,328–8,615)	1,858 (890–3,301)	3,841 (1,590–7,433)	1,947 (1,105–3,183)
Cote d'Ivoire	Number: 2015	77,062 (70,333–84,798)	7,038 (4,685–9,624)	6,127 (3,585–8,859)	9,363 (7,135–12,057)	5,985 (3,873–8,435)	3,866 (2,541–6,581)	19,988 (15,004–25,123)	8,380 (5,087–12,234)	1,710 (781–3,170)	2,630 (1,083–5,437)	1,933 (1,124–3,314)
Cote d'Ivoire	Number: Cumulative change (%)	1.5 (-7.6 to 11.4)	32.7 (-17.4 to 100.2)	47.2 (-26.8 to 149.5)	-0.7 (-30.6 to 45.4)	-31.9 (-58.9 to 2.4)	41.9 (0.1–91.4)	24.1 (-12.1 to 71.8)	52.8 (-18.8 to 165.4)	4.9 (-61.9 to 125.0)	-13.6 (-77.6 to 105.4)	8.3 (-49.4 to 106.4)
Cote d'Ivoire	Rate: 1990	3,308.3 (3,212.2–3,402.6)	221.8 (148.3–304.9)	180.4 (103.6–271.9)	419.3 (294.9–536.1)	401.6 (275.7–528.2)	119.2 (69.3–214.0)	770.6 (514.7–1,008.9)	237.1 (135.1–347.1)	75.4 (36.3–133.5)	173.8 (71.6–334.5)	84.8 (47.9–137.4)
Cote d'Ivoire	Rate: 2015	2,008.1 (1,833.3–2,217.5)	176.2 (117.3–240.8)	153.3 (89.8–221.6)	243.3 (185.1–314.0)	157.1 (101.8–221.0)	99.1 (65.0–169.3)	540.3 (404.3–678.1)	210.1 (127.7–306.5)	43.0 (19.8–79.4)	70.2 (29.1–145.1)	50.5 (29.6–86.4)
Cote d'Ivoire	Rate: Cumulative change (%)	-39.3 (-44.8 to -33.2)	-17.5 (-48.6 to 24.5)	-8.6 (-54.5 to 55.1)	-40.2 (-58.5 to -12.6)	-59.3 (-75.5 to -38.6)	-13.4 (-39.4 to 17.2)	-27.0 (-48.4 to 1.6)	-5.3 (-49.4 to 64.1)	-35.1 (-76.2 to 39.2)	-49.0 (-86.6 to 23.6)	-34.9 (-69.7 to 24.9)
The Gambia	Number: 1990	4,674 (4,474–4,881)	369 (249–506)	249 (144–376)	844 (689–998)	473 (353–603)	192 (131–288)	451 (255–698)	422 (240–619)	128 (60–234)	305 (198–445)	176 (120–246)
The Gambia	Number: 2015	3,696 (3,381–4,025)	397 (252–563)	341 (198–515)	457 (354–577)	398 (283–532)	282 (214–361)	54 (15–115)	550 (331–775)	117 (40–233)	207 (93–384)	155 (88–247)
The Gambia	Number: Cumulative change (%)	-20.9 (-28.5 to -12.9)	11.6 (-34.4 to 76.4)	48.0 (-27.0 to 159.2)	-45.2 (-60.0 to -26.9)	-13.7 (-44.1 to 24.3)	53.4 (1.0–122.0)	-87.6 (-95.7 to -74.4)	38.8 (-24.9 to 132.7)	5.3 (-67.6 to 143.9)	-28.6 (-71.1 to 40.8)	-8.5 (-50.4 to 60.2)
The Gambia	Rate: 1990	2,460.3 (2,351.4–2,573.4)	180.3 (121.8–246.7)	121.7 (71.2–183.6)	445.6 (361.2–528.7)	256.0 (190.9–327.7)	98.4 (66.7–149.7)	250.4 (141.9–387.9)	206.3 (117.7–302.0)	62.9 (29.6–114.5)	169.5 (108.9–248.4)	94.1 (63.5–132.0)
The Gambia	Rate: 2015	949.7 (867.6–1,035.9)	99.7 (63.4–141.4)	85.8 (49.9–129.2)	118.2 (91.2–149.6)	104.3 (74.0–140.1)	72.2 (54.9–92.4)	14.6 (4.2–30.6)	138.1 (83.5–194.5)	29.4 (10.3–58.6)	55.0 (24.4–101.7)	40.5 (23.0–64.3)
The Gambia	Rate: Cumulative change (%)	-61.4 (-65.1 to -57.5)	-42.6 (-66.2 to -9.2)	-23.9 (-62.3 to 33.1)	-73.1 (-80.5 to -64.2)	-58.2 (-73.1 to -39.7)	-23.1 (-50.1 to 11.8)	-94.0 (-97.9 to -87.7)	-28.7 (-61.4 to 18.8)	-46.1 (-83.5 to 24.3)	-66.0 (-86.3 to -33.1)	-55.3 (-75.8 to -21.5)
Ghana	Number: 1990	68,705 (67,110–70,469)	6,126 (4,360–8,106)	4,406 (2,713–6,519)	7,110 (5,601–8,795)	9,761 (7,436–12,248)	2,269 (1,704–3,061)	9,348 (6,533–12,249)	5,885 (3,287–9,004)	1,581 (782–2,775)	4,064 (1,907–7,632)	1,544 (971–2,340)
Ghana	Number: 2015	48,539 (44,384–53,405)	4,470 (2,802–6,337)	3,788 (2,197–5,657)	4,682 (3,485–6,054)	1,698 (1,049–2,449)	3,422 (2,399–4,520)	8,149 (4,574–12,446)	8,649 (5,617–11,678)	2,239 (1,060–4,094)	3,087 (1,490–5,559)	1,366 (820–2,323)
Ghana	Number: Cumulative change (%)	-29.3 (-35.6 to -22.2)	-24.6 (-57.1 to 15.6)	-8.0 (-54.9 to 61.6)	-32.9 (-54.4 to -4.1)	-82.2 (-89.8 to -72.1)	55.1 (-4.7 to 115.4)	-11.6 (-48.3 to 33.8)	59.2 (-7.8 to 176.2)	64.1 (-41.5 to 249.3)	-9.1 (-70.4 to 105.5)	-5.5 (-53.5 to 71.8)
Ghana	Rate: 1990	2,677.4 (2,614.1–2,746.0)	225.3 (160.4–298.4)	162.1 (100.0–239.9)	278.0 (217.7–346.3)	385.5 (292.4–485.3)	86.2 (64.5–116.6)	378.6 (264.4–496.5)	216.9 (121.4–331.6)	58.5 (28.9–102.3)	163.3 (76.4–308.7)	60.8 (38.1–93.2)
Ghana	Rate: 2015	1,166.3 (1,065.3–1,283.4)	105.6 (66.2–149.7)	89.5 (52.0–133.5)	112.8 (83.8–146.0)	41.1 (25.4–59.3)	81.7 (57.2–108.3)	199.9 (112.1–305.4)	204.4 (132.9–275.9)	53.0 (25.1–96.8)	75.4 (36.4–135.2)	33.1 (19.8–56.0)
Ghana	Rate: Cumulative change (%)	-56.4 (-60.4 to -52.1)	-51.6 (-72.4 to -25.8)	-41.0 (-71.0 to 3.6)	-58.6 (-72.0 to -40.7)	-89.1 (-93.8 to -82.8)	-2.4 (-40.4 to 36.0)	-46.4 (-68.7 to -19.1)	2.0 (-40.8 to 77.3)	4.8 (-62.5 to 122.7)	-44.6 (-82.1 to 25.1)	-41.9 (-71.7 to 4.8)
Guinea	Number: 1990	63,041 (61,361–64,929)	3,376 (2,229–4,628)	2,843 (1,677–4,258)	11,983 (8,920–15,030)	7,001 (4,664–9,689)	1,772 (872–3,805)	9,984 (5,162–15,304)	3,147 (1,777–4,835)	1,087 (508–1,982)	4,368 (1,913–7,981)	2,723 (1,546–4,308)
Guinea	Number: 2015	49,119 (44,534–53,904)	3,596 (2,447–5,008)	3,459 (2,130–4,987)	7,135 (5,536–8,995)	2,984 (2,010–4,180)	1,952 (1,130–3,427)	13,053 (9,797–16,485)	3,664 (2,056–5,380)	780 (346–1,450)	2,042 (808–3,944)	1,804 (1,084–2,765)
Guinea	Number: Cumulative change (%)	-22.1 (-29.7 to -14.4)	11.0 (-31.5 to 71.8)	31.0 (-32.7 to 125.7)	-38.7 (-58.0 to -12.3)	-55.2 (-74.4 to -26.3)	19.9 (-23.4 to 78.6)	43.4 (-15.2 to 144.1)	24.7 (-35.5 to 112.4)	-16.2 (-73.1 to 75.4)	-44.2 (-83.4 to 28.4)	-27.0 (-66.7 to 41.5)
Guinea	Rate: 1990	5,403.4 (5,250.3–5,572.1)	248.5 (164.3–340.3)	209.0 (123.8–312.9)	1,025.8 (749.7–1,303.9)	611.8 (400.1–855.4)	142.8 (68.5–314.6)	923.6 (477.9–1,423.0)	232.5 (131.4–356.2)	81.0 (38.1–146.4)	400.8 (173.9–735.4)	235.9 (129.0–382.7)
Guinea	Rate: 2015	2,351.6 (2,130.0–2,585.6)	164.2 (111.8–228.5)	157.9 (97.4–227.3)	341.0 (264.1–430.2)	143.8 (97.0–202.0)	91.5 (52.6–162.1)	644.6 (482.4–814.8)	167.6 (94.2–245.6)	35.8 (16.0–66.2)	100.3 (40.0–196.8)	86.8 (52.0–134.0)
Guinea	Rate: Cumulative change (%)	-56.5 (-60.7 to -52.0)	-31.2 (-57.5 to 6.6)	-18.7 (-58.2 to 40.4)	-65.7 (-76.8 to -50.7)	-75.2 (-86.0 to -58.9)	-29.4 (-56.3 to 6.1)	-23.4 (-54.7 to 29.8)	-22.9 (-60.0 to 31.0)	-48.5 (-83.5 to 7.4)	-70.0 (-91.2 to -31.2)	-59.2 (-81.5 to -22.0)
Guinea-Bissau	Number: 1990	9,824 (9,336–10,309)	534 (362–727)	419 (254–617)	1,371 (1,037–1,733)	1,926 (1,440–2,475)	274 (141–558)	1,292 (597–2,159)	510 (297–763)	172 (80–321)	642 (288–1,175)	432 (250–662)
Guinea-Bissau	Number: 2015	8,246 (7,359–9,277)	608 (414–815)	498 (312–719)	1,150 (864–1,509)	1,204 (821–1,652)	392 (216–749)	479 (174–977)	646 (361–955)	144 (68–262)	544 (224–1,016)	497 (271–832)
Guinea-Bissau	Number: Cumulative change (%)	-16.0 (-25.5 to -4.5)	18.0 (-24.1 to 75.4)	26.3 (-31.5 to 107.0)	-14.3 (-42.4 to 20.7)	-36.0 (-58.8 to -7.5)	49.8 (1.9–111.7)	-58.6 (-86.9 to -6.1)	35.0 (-29.9 to 132.5)	-1.3 (-67.6 to 106.9)	1.5 (-70.1 to 131.0)	24.7 (-44.6 to 132.8)
Guinea-Bissau	Rate: 1990	4,838.4 (4,593.0–5,072.1)	235.3 (159.7–320.3)	184.6 (112.2–271.8)	674.9 (505.8–857.7)	971.8 (718.1–1,256.2)	129.3 (65.5–267.2)	669.2 (310.3–1,117.4)	225.5 (131.6–336.9)	76.7 (35.9–142.4)	331.0 (148.3–611.2)	214.7 (122.7–329.8)
Guinea-Bissau	Rate: 2015	2,707.2 (2,413.7–3,046.7)	189.2 (128.8–253.3)	154.9 (97.1–223.4)	380.4 (283.0–504.4)	401.8 (273.0–554.0)	126.8 (69.4–243.5)	162.9 (59.3–332.9)	201.4 (112.9–297.4)	45.3 (21.4–82.0)	184.3 (74.9–350.4)	165.5 (90.0–278.2)
Guinea-Bissau	Rate: Cumulative change (%)	-44.0 (-50.4 to -36.3)	-16.8 (-46.3 to 23.7)	-10.9 (-51.5 to 45.7)	-42.3 (-61.6 to -18.4)	-57.6 (-73.2 to -38.6)	3.4 (-30.0 to 46.9)	-72.8 (-91.4 to -38.4)	-4.9 (-50.4 to 62.3)	-30.7 (-77.0 to 44.9)	-33.1 (-80.3 to 53.3)	-16.3 (-63.4 to 55.6)
Liberia	Number: 1990	21,919 (21,142–22,726)	1,178 (818–1,566)	959 (595–1,392)	3,554 (2,629–4,461)	3,973 (2,779–5,165)	660 (341–1,417)	3,068 (1,506–4,809)	1,160 (668–1,764)	384 (189–671)	1,474 (642–2,673)	953 (581–1,514)
Liberia	Number: 2015	11,014 (10,156–11,995)	952 (616–1,346)	929 (591–1,359)	1,499 (1,127–1,925)	1,225 (808–1,683)	631 (416–962)	1,553 (794–2,353)	1,085 (659–1,581)	218 (91–412)	473 (190–971)	475 (250–877)
Liberia	Number: Cumulative change (%)	-49.7 (-54.0 to -44.7)	-16.3 (-50.0 to 31.1)	2.6 (-42.9 to 71.1)	-56.6 (-71.2 to -37.4)	-68.1 (-80.8 to -51.6)	8.9 (-42.7 to 72.6)	-43.6 (-75.3 to 4.6)	0.1 (-47.6 to 68.4)	-34.0 (-79.5 to 46.7)	-61.1 (-88.9 to -9.2)	-46.6 (-76.7 to 5.2)
Liberia	Rate: 1990	5,198.1 (5,007.3–5,395.1)	261.6 (181.9–346.9)	212.9 (132.3–308.5)	842.7 (619.8–1,062.2)	949.8 (663.1–1,239.8)	153.1 (78.3–331.0)	751.1 (369.2–1,177.2)	258.4 (150.0–392.0)	86.2 (42.5–150.7)	358.2 (155.2–652.2)	226.9 (138.2–362.5)
Liberia	Rate: 2015	1,509.5 (1,390.6–1,646.1)	127.2 (82.4–179.8)	124.2 (79.1–181.5)	205.5 (154.0–264.3)	168.8 (111.5–232.6)	85.8 (56.6–131.0)	218.5 (112.0–330.5)	145.2 (88.3–211.3)	29.3 (12.3–55.2)	65.9 (26.6–134.8)	65.4 (34.6–120.2)
Liberia	Rate: Cumulative change (%)	-70.9 (-73.5 to -68.0)	-49.7 (-69.9 to -21.3)	-38.3 (-65.6 to 2.8)	-74.9 (-83.4 to -63.7)	-81.6 (-88.9 to -72.0)	-35.9 (-66.8 to 2.1)	-67.6 (-85.7 to -39.9)	-39.9 (-68.4 to 0.9)	-60.6 (-87.7 to -12.2)	-77.7 (-93.7 to -48.2)	-69.1 (-86.6 to -39.0)
Mali	Number: 1990	93,071 (90,938–95,292)	6,068 (4,131–8,147)	4,402 (2,560–6,614)	8,036 (6,004–10,422)	12,720 (9,268–16,962)	2,619 (1,286–5,659)	22,654 (14,089–31,978)	5,364 (2,997–8,291)	1,983 (863–3,742)	9,430 (4,925–16,033)	3,793 (2,237–5,822)
Mali	Number: 2015	96,662 (87,328–107,468)	7,886 (5,277–11,115)	6,214 (3,581–9,282)	6,155 (4,563–8,080)	7,311 (4,988–10,062)	3,597 (2,017–7,007)	31,342 (24,288–38,571)	7,577 (4,279–11,331)	1,656 (770–3,034)	7,453 (3,383–13,276)	3,509 (1,981–5,615)
Mali	Number: Cumulative change (%)	3.9 (-6.4 to 15.1)	34.0 (-12.5 to 98.6)	50.2 (-21.7 to 154.7)	-21.7 (-46.6 to 9.2)	-40.7 (-63.1 to -12.3)	45.5 (1.7–102.3)	44.8 (0.6–107.7)	51.7 (-23.1 to 169.3)	-2.4 (-67.1 to 100.5)	-11.4 (-67.2 to 79.6)	-1.9 (-52.5 to 71.0)
Mali	Rate: 1990	5,433.4 (5,295.9–5,585.8)	309.7 (211.0–415.8)	224.6 (132.2–336.5)	465.5 (342.2–608.1)	748.6 (542.9–1,006.4)	145.0 (69.4–323.2)	1,403.5 (871.7–1,979.3)	274.8 (154.4–424.3)	102.2 (45.0–192.7)	583.9 (298.4–1,000.1)	219.1 (128.7–335.5)
Mali	Rate: 2015	2,860.3 (2,581.4–3,183.4)	219.2 (146.9–308.9)	172.7 (99.5–257.6)	181.3 (134.0–239.4)	217.3 (147.5–299.1)	103.7 (57.7–203.5)	959.4 (740.9–1,181.2)	211.0 (119.4–315.4)	46.3 (21.8–84.6)	227.7 (102.0–404.9)	103.5 (58.3–165.4)
Mali	Rate: Cumulative change (%)	-47.3 (-52.6 to -41.6)	-27.1 (-52.2 to 8.2)	-18.2 (-57.3 to 38.4)	-60.1 (-73.2 to -44.1)	-70.0 (-81.5 to -55.2)	-23.6 (-47.3 to 7.2)	-28.5 (-50.2 to 2.8)	-17.6 (-58.2 to 45.8)	-47.2 (-82.0 to 8.4)	-56.1 (-84.0 to -10.5)	-49.8 (-75.6 to -11.5)
Mauritania	Number: 1990	8,766 (8,470–9,062)	698 (451–970)	563 (328–852)	1,111 (631–1,516)	1,253 (565–1,820)	372 (220–587)	158 (38–366)	803 (453–1,173)	257 (114–475)	557 (171–1,077)	226 (103–391)
Mauritania	Number: 2015	7,311 (6,674–8,044)	837 (559–1,147)	764 (471–1,103)	890 (689–1,092)	708 (484–963)	595 (437–825)	231 (75–470)	1,031 (627–1,521)	204 (95–402)	332 (151–635)	226 (130–372)
Mauritania	Number: Cumulative change (%)	-16.6 (-24.1 to -8.0)	25.7 (-26.0 to 101.5)	45.4 (-22.9 to 147.8)	-14.4 (-45.4 to 41.4)	-35.6 (-67.1 to 27.4)	69.3 (12.4–149.5)	87.8 (-39.8 to 332.3)	37.3 (-25.1 to 129.7)	-6.4 (-68.4 to 108.6)	-20.5 (-77.6 to 92.7)	15.1 (-53.3 to 128.6)
Mauritania	Rate: 1990	2,356.8 (2,277.2–2,434.6)	177.7 (115.1–247.1)	143.5 (83.6–216.8)	299.5 (166.7–411.2)	343.5 (152.2–504.0)	98.1 (57.3–156.1)	44.7 (10.7–103.1)	205.1 (116.1–298.9)	65.8 (29.3–121.5)	155.8 (47.9–304.9)	61.5 (27.3–107.9)

© 2017 Global Burden of Disease Child and Adolescent Health Collaboration. JAMA Pediatrics.

eTable 2. Number of Deaths, Death Rates (per 100,000 population), and Cumulative Percent Change with 95% Uncertainty Intervals (UI) for the Top 10 Global Causes of Death in 195 Countries and Territories, Aged Under 5 Years, Both Sexes, 1990 and 2015 *Note: best viewed by enlarging in browser.*

Location	Measure	All causes	Neonatal preterm birth complications	Neonatal encephalopathy due to birth asphyxia and trauma	Lower respiratory infections	Diarrheal diseases	Congenital anomalies	Malaria	Neonatal sepsis and other neonatal infections	Other neonatal disorders	Protein-energy malnutrition	Meningitis
Mauritania	Rate: 2015	1,155.7 (1,054.0–1,274.1)	130.3 (87.1–178.5)	118.9 (73.5–171.6)	141.4 (109.4–174.3)	113.4 (77.2–154.1)	93.9 (69.0–130.3)	37.5 (12.2–75.9)	160.6 (97.9–236.7)	31.9 (15.0–62.6)	53.5 (24.3–103.0)	36.1 (20.8–59.2)
Mauritania	Rate: Cumulative change (%)	-50.9 (-55.5 to -45.8)	-23.2 (-54.7 to 23.0)	-11.2 (-52.8 to 50.6)	-49.4 (-68.0 to -15.1)	-62.2 (-81.0 to -24.6)	1.7 (-33.2 to 50.5)	8.3 (-65.4 to 149.9)	-16.3 (-54.1 to 39.9)	-43.0 (-80.6 to 26.7)	-54.1 (-87.2 to 11.9)	-32.1 (-72.8 to 34.4)
Niger	Number: 1990	123,917 (121,300–126,714)	3,983 (2,686–5,567)	2,960 (1,584–4,507)	20,201 (14,884–26,042)	28,974 (21,339–37,071)	2,332 (1,068–5,458)	15,706 (5,520–29,409)	3,843 (2,134–5,696)	1,685 (677–3,190)	10,815 (5,049–19,382)	9,863 (5,151–16,854)
Niger	Number: 2015	105,553 (96,568–116,112)	5,310 (3,345–7,771)	4,653 (2,924–6,526)	14,121 (10,670–18,283)	19,868 (14,420–26,546)	3,528 (2,014–6,592)	23,273 (13,575–33,195)	5,130 (3,027–7,273)	1,329 (468–2,432)	6,143 (2,700–10,944)	8,655 (4,920–13,829)
Niger	Number: Cumulative change (%)	-14.8 (-21.8 to -6.6)	38.0 (-15.3 to 121.6)	68.2 (-4.3 to 169.6)	-28.3 (-51.5 to 1.1)	-29.6 (-54.1 to 0.9)	73.1 (3.1–157.6)	75.2 (-14.6 to 234.4)	42.7 (-19.9 to 136.9)	-7.2 (-68.8 to 107.0)	-34.1 (-76.9 to 41.8)	-2.2 (-54.4 to 72.0)
Niger	Rate: 1990	7,419.5 (7,232.9–7,619.5)	192.2 (130.2–268.4)	142.7 (76.4–216.8)	1,207.8 (876.8–1,579.7)	1,776.0 (1,288.4–2,297.6)	127.4 (54.8–307.8)	988.1 (350.3–1,831.7)	185.8 (104.6–274.3)	82.2 (33.1–153.9)	689.3 (320.2–1,254.5)	598.1 (303.8–1,039.1)
Niger	Rate: 2015	2,465.0 (2,249.4–2,710.1)	113.0 (71.1–165.5)	99.0 (62.2–138.8)	329.9 (248.7–430.9)	470.8 (341.2–627.9)	78.7 (44.5–148.3)	562.6 (329.5–802.1)	109.2 (64.7–154.5)	28.4 (10.0–51.7)	148.5 (64.7–268.0)	204.1 (115.0–329.2)
Niger	Rate: Cumulative change (%)	-66.8 (-69.6 to -63.5)	-39.2 (-62.7 to -2.1)	-25.8 (-57.5 to 19.1)	-71.9 (-81.4 to -60.3)	-72.7 (-82.4 to -60.4)	-27.4 (-58.9 to 10.3)	-32.7 (-67.2 to 27.6)	-37.2 (-64.9 to 3.9)	-59.4 (-86.1 to -8.9)	-74.8 (-91.4 to -44.7)	-61.6 (-82.3 to -30.0)
Nigeria	Number: 1990	855,260 (832,876–876,402)	38,660 (25,644–54,336)	43,596 (27,343–61,598)	95,839 (71,771–123,953)	96,765 (66,998–131,171)	25,477 (14,034–51,282)	194,446 (126,448–267,085)	49,473 (27,891–73,664)	16,673 (7,995–29,117)	60,208 (26,644–110,307)	31,458 (19,920–48,506)
Nigeria	Number: 2015	726,575 (657,535–799,166)	39,839 (25,561–57,272)	56,403 (34,647–82,330)	59,644 (45,647–76,949)	102,676 (77,537–131,786)	31,597 (19,340–53,632)	161,211 (114,115–208,517)	76,019 (44,170–107,827)	15,309 (6,655–28,550)	29,733 (12,833–56,970)	25,036 (14,764–43,298)
Nigeria	Number: Cumulative change (%)	-15.0 (-23.2 to -6.1)	7.0 (-39.1 to 61.8)	35.6 (-24.5 to 117.7)	-36.3 (-55.5 to -12.3)	10.6 (-28.5 to 61.8)	32.3 (-10.6 to 81.1)	-14.0 (-41.8 to 25.1)	64.7 (-12.9 to 178.3)	5.6 (-65.6 to 118.9)	-41.4 (-81.1 to 23.1)	-15.4 (-58.8 to 55.1)
Nigeria	Rate: 1990	4,797.6 (4,673.8–4,929.7)	193.6 (128.4–272.1)	218.2 (137.3–308.1)	531.4 (396.1–693.8)	552.7 (381.7–755.4)	135.6 (73.1–279.0)	1,139.3 (742.8–1,566.4)	248.5 (140.3–369.2)	84.2 (40.4–146.2)	352.8 (154.1–654.9)	176.7 (109.9–275.7)
Nigeria	Rate: 2015	2,236.0 (2,019.4–2,462.2)	117.0 (75.1–168.2)	165.6 (101.8–241.7)	183.3 (140.2–237.0)	319.4 (240.2–410.6)	95.5 (58.1–162.4)	511.6 (362.5–663.9)	223.7 (130.5–316.8)	45.1 (19.6–84.1)	93.9 (40.2–179.5)	77.5 (45.6–133.9)
Nigeria	Rate: Cumulative change (%)	-53.4 (-58.0 to -48.5)	-37.2 (-64.3 to -5.3)	-20.5 (-55.7 to 27.5)	-64.6 (-75.5 to -51.0)	-39.6 (-61.6 to -11.6)	-24.4 (-49.8 to 4.0)	-53.4 (-68.4 to -32.3)	-3.6 (-48.9 to 62.7)	-38.6 (-79.8 to 26.3)	-68.3 (-90.1 to -32.6)	-53.2 (-77.4 to -13.8)
Sao Tome and Principe	Number: 1990	470 (452–488)	27 (17–38)	26 (16–38)	98 (70–121)	77 (53–100)	25 (16–37)	0 (0–2)	33 (19–48)	9 (4–16)	35 (16–61)	7 (4–12)
Sao Tome and Principe	Number: 2015	242 (215–274)	21 (13–30)	25 (15–36)	36 (26–47)	19 (13–27)	26 (20–32)	0 (0–0)	31 (18–44)	5 (2–11)	13 (5–25)	3 (1–6)
Sao Tome and Principe	Number: Cumulative change (%)	-48.3 (-54.4 to -41.3)	-18.8 (-51.8 to 31.1)	0.9 (-45.7 to 65.7)	-62.2 (-75.0 to -45.9)	-73.6 (-84.7 to -57.9)	9.3 (-29.6 to 59.2)	-68.2 (-96.4 to -11.2)	1.5 (-45.6 to 68.5)	-28.7 (-76.1 to 58.6)	-55.2 (-84.9 to -2.4)	-48.8 (-79.6 to 3.8)
Sao Tome and Principe	Rate: 1990	2,293.5 (2,207.0–2,385.1)	126.7 (81.9–178.1)	124.1 (77.3–177.3)	479.9 (345.4–596.9)	380.2 (258.9–495.6)	120.2 (79.8–180.6)	4.4 (0.1–12.9)	154.3 (90.0–224.4)	43.5 (20.5–78.7)	177.0 (84.1–307.9)	38.8 (20.3–62.1)
Sao Tome and Principe	Rate: 2015	795.0 (706.5–898.2)	69.1 (44.1–98.5)	82.8 (51.0–120.1)	118.3 (87.7–154.5)	64.1 (42.9–90.4)	85.1 (65.9–107.5)	0.7 (0.0–2.6)	102.8 (61.0–145.4)	18.7 (7.5–36.5)	45.0 (19.2–84.1)	11.9 (6.1–21.8)
Sao Tome and Principe	Rate: Cumulative change (%)	-65.3 (-69.4 to -60.7)	-43.1 (-66.2 to -8.1)	-29.3 (-61.9 to 16.1)	-74.6 (-83.4 to -63.6)	-82.4 (-89.8 to -71.9)	-25.4 (-52.4 to 8.9)	-79.1 (-97.6 to -41.5)	-29.1 (-61.9 to 17.0)	-50.3 (-83.3 to 10.3)	-70.4 (-90.0 to -35.5)	-65.7 (-86.6 to -30.1)
Senegal	Number: 1990	46,018 (45,108–46,985)	2,730 (1,805–3,764)	2,217 (1,273–3,327)	5,575 (4,446–6,810)	9,320 (7,268–11,394)	1,620 (968–2,857)	7,677 (4,820–11,044)	3,014 (1,782–4,491)	945 (443–1,659)	3,488 (1,638–6,141)	1,770 (1,162–2,600)
Senegal	Number: 2015	29,093 (27,242–31,239)	2,752 (1,741–3,908)	2,622 (1,666–3,770)	3,562 (2,806–4,390)	4,281 (3,149–5,574)	2,238 (1,608–3,104)	1,303 (526–2,283)	3,294 (2,114–4,633)	666 (270–1,274)	1,596 (711–3,005)	1,414 (812–2,384)
Senegal	Number: Cumulative change (%)	-36.8 (-40.9 to -32.1)	4.0 (-35.7 to 60.8)	25.8 (-28.0 to 99.9)	-34.9 (-53.5 to -12.0)	-53.3 (-68.4 to -35.4)	49.1 (-7.2 to 121.2)	-82.5 (-92.2 to -68.6)	15.8 (-34.3 to 86.2)	-18.8 (-71.9 to 72.5)	-46.9 (-82.9 to 17.2)	-16.2 (-57.9 to 49.9)
Senegal	Rate: 1990	3,194.8 (3,124.6–3,265.8)	175.0 (115.9–241.7)	142.1 (81.8–213.3)	386.4 (305.8–474.4)	662.0 (516.0–811.0)	108.8 (64.2–194.7)	554.0 (347.8–797.4)	193.6 (114.7–288.3)	60.9 (28.5–106.6)	251.6 (118.4–447.7)	123.7 (81.0–180.6)
Senegal	Rate: 2015	1,095.4 (1,025.2–1,176.1)	101.0 (63.9–143.6)	96.3 (61.2–138.3)	134.6 (105.8–166.1)	163.8 (120.4–214.2)	83.6 (60.0–115.8)	50.4 (20.4–88.4)	121.0 (77.7–170.2)	24.5 (10.0–46.8)	61.5 (27.3–115.7)	53.8 (30.8–91.2)
Senegal	Rate: Cumulative change (%)	-65.7 (-68.0 to -63.1)	-40.4 (-63.2 to -7.8)	-28.0 (-58.7 to 14.5)	-64.5 (-74.9 to -51.6)	-74.8 (-83.1 to -65.0)	-16.7 (-48.9 to 24.4)	-90.6 (-95.8 to -83.1)	-33.8 (-62.4 to 6.2)	-53.7 (-84.0 to -2.0)	-71.5 (-91.0 to -36.3)	-54.3 (-77.3 to -17.1)
Sierra Leone	Number: 1990	42,871 (41,766–43,958)	2,356 (1,605–3,151)	2,037 (1,327–2,922)	7,092 (5,306–8,974)	5,773 (3,989–7,864)	1,310 (618–3,018)	8,524 (4,075–13,611)	2,025 (1,115–3,118)	711 (335–1,238)	3,108 (1,390–5,651)	1,913 (1,168–3,051)
Sierra Leone	Number: 2015	27,874 (26,050–29,773)	1,901 (1,276–2,578)	1,900 (1,189–2,698)	3,612 (2,620–4,805)	2,189 (1,393–3,195)	1,213 (631–2,359)	6,719 (4,331–8,980)	2,001 (1,119–3,063)	422 (194–785)	1,314 (521–2,575)	1,196 (692–2,067)
Sierra Leone	Number: Cumulative change (%)	-35.0 (-39.6 to -30.1)	-16.2 (-49.1 to 28.9)	-2.1 (-46.5 to 53.9)	-48.0 (-64.5 to -25.9)	-60.7 (-77.9 to -37.7)	1.8 (-36.0 to 54.0)	-12.9 (-50.3 to 41.7)	6.5 (-48.5 to 90.8)	-31.2 (-76.0 to 46.3)	-50.3 (-84.9 to 16.6)	-32.5 (-68.9 to 24.2)
Sierra Leone	Rate: 1990	5,790.8 (5,632.1–5,955.4)	282.4 (193.0–377.5)	243.9 (159.0–349.2)	951.9 (707.7–1,205.8)	790.0 (545.6–1,075.1)	169.3 (78.4–394.0)	1,223.3 (588.1–1,950.3)	243.5 (134.7–373.9)	86.4 (40.8–149.7)	439.1 (195.0–827.9)	260.1 (158.6–418.6)
Sierra Leone	Rate: 2015	2,663.7 (2,484.1–2,846.7)	174.3 (117.0–236.1)	174.1 (109.2–246.9)	344.8 (249.3–458.2)	210.5 (134.0–307.6)	114.3 (59.1–223.2)	658.8 (426.3–881.7)	183.7 (102.7–280.9)	39.0 (17.9–72.0)	127.8 (50.9–252.5)	114.8 (66.9–197.7)
Sierra Leone	Rate: Cumulative change (%)	-54.0 (-57.2 to -50.6)	-36.0 (-60.9 to -1.4)	-25.1 (-59.0 to 17.6)	-63.0 (-74.7 to -47.2)	-72.3 (-84.4 to -56.2)	-25.2 (-53.8 to 14.1)	-40.6 (-66.0 to -3.7)	-18.8 (-60.7 to 44.9)	-47.8 (-81.7 to 10.6)	-65.7 (-89.7 to -21.8)	-52.3 (-78.1 to -11.7)
Togo	Number: 1990	22,767 (22,078–23,461)	1,466 (985–1,983)	1,255 (764–1,870)	3,065 (2,443–3,787)	3,250 (2,361–4,222)	831 (502–1,460)	4,674 (3,303–6,059)	1,554 (873–2,300)	464 (215–853)	1,433 (650–2,680)	577 (345–981)
Togo	Number: 2015	19,741 (18,286–21,392)	1,736 (1,182–2,395)	1,688 (1,047–2,441)	2,229 (1,735–2,822)	1,559 (1,056–2,153)	1,016 (685–1,573)	4,803 (3,528–6,160)	2,028 (1,124–3,059)	403 (170–830)	706 (308–1,435)	446 (261–725)
Togo	Number: Cumulative change (%)	-13.3 (-20.1 to -5.8)	22.8 (-26.6 to 83.0)	43.8 (-24.5 to 139.6)	-26.0 (-47.4 to 1.7)	-50.7 (-68.9 to -27.4)	28.1 (-10.8 to 74.1)	5.4 (-26.0 to 45.9)	39.7 (-32.4 to 131.5)	1.9 (-67.4 to 131.7)	-41.6 (-83.4 to 32.6)	-15.0 (-63.2 to 58.3)
Togo	Rate: 1990	3,195.4 (3,098.6–3,290.2)	191.9 (129.0–259.3)	164.3 (100.2–244.9)	429.9 (341.5–534.4)	463.7 (337.6–605.0)	113.2 (68.3–200.4)	682.2 (480.1–883.9)	203.8 (114.7–300.9)	61.2 (28.4–112.2)	208.4 (94.2–395.8)	81.6 (48.3–138.2)
Togo	Rate: 2015	1,657.2 (1,534.8–1,799.1)	141.7 (96.5–195.4)	137.7 (85.5–199.3)	187.3 (145.4–237.1)	132.1 (89.6–182.9)	84.3 (56.8–130.9)	412.8 (303.4–529.5)	165.7 (92.0–249.9)	33.0 (13.9–67.7)	60.5 (26.5–124.7)	37.7 (22.0–61.3)
Togo	Rate: Cumulative change (%)	-48.1 (-52.2 to -43.6)	-23.5 (-54.2 to 14.0)	-10.4 (-52.9 to 49.1)	-55.6 (-68.7 to -38.8)	-70.7 (-81.6 to -57.0)	-21.8 (-45.9 to 6.5)	-37.9 (-56.4 to -13.9)	-13.0 (-57.9 to 44.1)	-36.8 (-79.7 to 43.9)	-65.5 (-90.2 to -21.7)	-49.1 (-77.8 to -3.2)
Eastern Sub-Saharan Africa	Number: 1990	1,445,114 (1,429,910–1,461,075)	80,419 (66,899–93,795)	72,023 (56,989–87,551)	211,855 (177,731–244,128)	197,141 (155,248–237,383)	50,313 (40,377–64,451)	223,357 (156,514–297,949)	48,757 (35,291–67,232)	45,654 (31,074–63,743)	91,973 (67,245–122,148)	51,660 (36,730–69,133)
Eastern Sub-Saharan Africa	Number: 2015	908,802 (878,910–940,565)	70,810 (60,850–80,898)	74,910 (63,630–87,232)	126,243 (111,400–141,668)	81,338 (70,185–93,539)	75,758 (52,526–91,602)	66,276 (37,221–97,033)	57,493 (43,328–75,528)	39,987 (28,955–52,229)	47,872 (36,566–61,129)	35,033 (27,180–44,693)
Eastern Sub-Saharan Africa	Number: Cumulative change (%)	-37.1 (-39.4 to -34.8)	-11.3 (-25.9 to 5.6)	5.3 (-15.1 to 31.0)	-39.9 (-51.0 to -27.1)	-58.1 (-67.8 to -46.1)	55.1 (-12.9 to 103.4)	-69.6 (-82.2 to -53.0)	20.3 (-11.8 to 57.2)	-9.3 (-39.5 to 29.7)	-46.4 (-64.1 to -25.0)	-29.8 (-51.3 to 0.6)
Eastern Sub-Saharan Africa	Rate: 1990	3,875.3 (3,832.2–3,920.3)	197.2 (164.4–230.0)	176.5 (139.8–214.4)	565.6 (472.9–653.7)	537.5 (420.4–651.0)	130.0 (103.4–168.1)	620.9 (435.1–824.2)	120.3 (87.2–165.6)	112.4 (76.6–157.0)	257.0 (186.4–343.4)	139.7 (98.9–188.5)
Eastern Sub-Saharan Africa	Rate: 2015	1,421.1 (1,374.8–1,471.1)	107.8 (92.7–123.2)	114.1 (96.9–132.8)	198.0 (174.5–222.4)	128.7 (111.2–148.1)	117.7 (81.8–142.4)	105.7 (59.4–154.9)	87.7 (66.2–115.2)	61.0 (44.1–79.6)	76.4 (58.3–97.4)	55.2 (42.7–70.4)
Eastern Sub-Saharan Africa	Rate: Cumulative change (%)	-63.3 (-64.6 to -61.9)	-44.9 (-53.9 to -34.4)	-34.6 (-47.2 to -18.6)	-64.7 (-71.3 to -57.0)	-75.7 (-81.4 to -68.6)	-6.5 (-48.3 to 23.3)	-82.6 (-89.8 to -73.1)	-25.6 (-45.4 to -2.7)	-43.9 (-62.5 to -19.8)	-69.4 (-79.5 to -56.9)	-59.0 (-71.7 to -40.7)
Burundi	Number: 1990	47,231 (44,416–49,901)	2,830 (2,015–3,673)	1,866 (1,127–2,754)	6,022 (4,557–7,782)	5,559 (3,895–7,473)	1,622 (1,113–2,302)	12,401 (7,559–17,100)	1,545 (854–2,541)	1,355 (692–2,258)	3,634 (1,721–6,414)	1,207 (673–1,926)
Burundi	Number: 2015	36,468 (28,628–46,278)	3,193 (2,008–4,522)	2,652 (1,722–3,814)	5,261 (3,654–7,427)	4,093 (2,594–6,151)	2,888 (1,927–4,140)	3,296 (1,392–5,611)	2,042 (1,117–3,364)	1,379 (660–2,502)	2,852 (1,256–5,234)	1,358 (773–2,277)
Burundi	Number: Cumulative change (%)	-22.7 (-39.5 to -1.0)	15.4 (-27.6 to 72.0)	51.3 (-18.3 to 150.8)	-10.7 (-43.4 to 32.1)	-23.4 (-57.1 to 25.3)	86.4 (1.0–181.0)	-72.4 (-88.3 to -50.0)	43.5 (-35.7 to 159.0)	13.5 (-55.3 to 132.8)	-9.3 (-69.3 to 96.4)	26.5 (-44.2 to 162.6)
Burundi	Rate: 1990	3,905.7 (3,664.4–4,133.8)	214.3 (153.0–277.6)	141.3 (85.5–208.4)	494.7 (372.6–643.7)	468.0 (324.6–636.4)	128.8 (87.6–185.1)	1,054.5 (643.2–1,457.4)	117.6 (65.3–193.4)	103.1 (52.9–171.2)	312.2 (146.5–552.0)	100.3 (55.3–162.4)
Burundi	Rate: 2015	1,635.9 (1,279.9–2,084.2)	136.9 (86.2–193.9)	113.7 (74.0–163.3)	236.9 (163.8–334.7)	187.4 (117.8–282.0)	127.7 (85.1–182.9)	152.1 (64.4–259.0)	87.8 (48.1–144.7)	59.3 (28.4–107.7)	132.2 (58.0–243.4)	61.7 (34.8–103.0)
Burundi	Rate: Cumulative change (%)	-58.1 (-67.3 to -46.1)	-34.7 (-59.0 to -2.8)	-14.4 (-53.7 to 41.8)	-51.0 (-69.1 to -27.3)	-58.3 (-76.7 to -31.7)	4.1 (-44.1 to 58.9)	-85.0 (-93.7 to -72.9)	-19.0 (-63.6 to 46.1)	-35.9 (-74.7 to 31.6)	-50.9 (-83.6 to 7.8)	-30.5 (-70.0 to 45.6)
Comoros	Number: 1990	2,101 (1,982–2,227)	191 (137–253)	122 (76–181)	388 (269–495)	258 (155–373)	115 (85–145)	8 (2–17)	112 (63–178)	92 (44–160)	160 (71–284)	62 (33–96)
Comoros	Number: 2015	1,152 (850–1,544)	140 (92–199)	124 (74–183)	163 (105–241)	83 (45–135)	125 (72–192)	1 (0–5)	100 (52–164)	65 (28–123)	42 (15–88)	31 (15–55)
Comoros	Number: Cumulative change (%)	-45.1 (-60.3 to -26.3)	-24.6 (-53.5 to 15.2)	7.2 (-44.4 to 79.2)	-56.3 (-74.7 to -28.9)	-65.4 (-83.2 to -36.1)	11.9 (-41.5 to 84.6)	-73.7 (-96.8 to -20.4)	-3.2 (-54.5 to 75.6)	-20.3 (-71.9 to 75.2)	-68.2 (-91.5 to -25.5)	-44.3 (-77.2 to 19.0)
Comoros	Rate: 1990	2,630.4 (2,479.8–2,786.1)	227.9 (164.1–301.7)	146.5 (91.2–216.1)	487.2 (335.9–622.9)	329.1 (195.8–476.6)	143.0 (104.9–181.3)	11.1 (2.6–22.6)	135.2 (76.5–214.3)	111.1 (54.5–190.7)	207.9 (92.4–370.0)	79.1 (42.0–123.6)
Comoros	Rate: 2015	919.2 (678.5–1,232.7)	110.7 (73.4–157.6)	98.1 (59.1–145.1)	130.8 (84.1–192.5)	67.4 (36.8–108.7)	100.0 (58.1–153.6)	1.5 (0.1–4.2)	79.9 (41.3–130.0)	51.4 (22.7–97.7)	34.4 (12.8–70.7)	25.0 (12.6–44.1)
Comoros	Rate: Cumulative change (%)	-65.0 (-74.7 to -53.0)	-50.0 (-69.2 to -23.8)	-29.0 (-63.0 to 18.9)	-72.1 (-83.9 to -54.3)	-78.1 (-89.4 to -59.6)	-27.9 (-62.5 to 19.4)	-83.6 (-98.0 to -49.7)	-36.2 (-70.0 to 14.9)	-47.4 (-81.5 to 14.8)	-80.1 (-94.7 to -53.1)	-64.6 (-85.5 to -24.7)
Djibouti	Number: 1990	2,455 (2,313–2,599)	201 (133–272)	126 (72–194)	433 (309–549)	356 (236–496)	156 (119–196)	0 (0–1)	117 (64–191)	93 (42–164)	215 (99–372)	84 (44–133)
Djibouti	Number: 2015	1,439 (1,251–1,662)	135 (90–184)	103 (59–157)	225 (150–299)	128 (74–186)	139 (97–186)	1 (0–3)	93 (52–154)	63 (29–112)	91 (39–169)	48 (25–81)
Djibouti	Number: Cumulative change (%)	-41.3 (-49.4 to -31.3)	-30.4 (-55.8 to 2.0)	-12.8 (-55.2 to 48.5)	-46.6 (-67.5 to -22.8)	-62.1 (-80.6 to -36.0)	-8.5 (-40.7 to 30.1)	145.9 (-76.9 to 666.9)	-13.3 (-60.5 to 56.6)	-23.6 (-71.4 to 55.3)	-50.4 (-84.6 to 8.6)	-36.2 (-72.7 to 25.2)
Djibouti	Rate: 1990	2,459.8 (2,320.9–2,605.3)	185.8 (123.5–251.4)	116.8 (66.9–179.4)	435.7 (306.7–555.1)	366.0 (239.0–509.5)	151.4 (116.6–190.5)	0.7 (0.2–1.4)	109.3 (59.9–177.1)	86.9 (39.4–151.5)	227.0 (103.9–396.7)	86.5 (44.4–138.3)
Djibouti	Rate: 2015	1,374.4 (1,195.5–1,587.6)	128.9 (85.4–175.4)	97.8 (56.3–149.2)	215.2 (144.2–286.3)	122.7 (71.2–178.4)	133.6 (92.7–178.4)	1.2 (0.1–3.4)	89.3 (49.6–146.7)	60.1 (28.0–106.3)	87.7 (37.7–162.4)	46.9 (24.7–77.5)
Djibouti	Rate: Cumulative change (%)	-44.1 (-51.8 to -34.6)	-28.4 (-54.5 to 5.0)	-10.3 (-53.8 to 52.9)	-49.1 (-69.3 to -26.1)	-64.6 (-82.3 to -39.7)	-9.8 (-41.4 to 28.9)	124.1 (-79.2 to 603.4)	-11.1 (-59.5 to 60.8)	-21.8 (-70.6 to 59.7)	-54.8 (-86.1 to -0.8)	-39.7 (-74.6 to 18.9)
Eritrea	Number: 1990	19,329 (18,521–20,143)	960 (640–1,356)	619 (351–943)	3,240 (1,834–4,310)	3,559 (1,854–4,983)	608 (394–835)	493 (164–918)	546 (282–891)	540 (244–965)	1,831 (663–3,144)	533 (243–875)
Eritrea	Number: 2015	11,730 (9,864–13,973)	949 (624–1,308)	782 (477–1,156)	1,996 (1,421–2,646)	1,788 (1,175–2,540)	927 (650–1,221)	173 (40–474)	649 (341–1,038)	454 (209–800)	929 (423–1,616)	441 (242–728)
Eritrea	Number: Cumulative change (%)	-39.3 (-49.2 to -27.4)	2.5 (-35.3 to 55.0)	36.2 (-31.0 to 132.3)	-33.6 (-60.3 to 12.0)	-43.3 (-70.6 to 2.0)	60.6 (-7.8 to 151.9)	-60.4 (-89.8 to 7.0)	30.1 (-40.6 to 133.3)	-4.7 (-63.1 to 82.2)	-34.1 (-79.2 to 50.7)	-2.5 (-60.0 to 108.9)
Eritrea	Rate: 1990	3,242.3 (3,105.9–3,386.6)	152.8 (101.8–216.0)	98.6 (55.8–150.1)	543.2 (303.6–723.5)	603.5 (310.3–847.9)	99.5 (64.0–137.2)	84.2 (28.0–157.0)	87.2 (45.2–142.3)	86.1 (39.0–153.5)	314.7 (113.3–544.1)	89.9 (40.2–148.1)
Eritrea	Rate: 2015	1,406.6 (1,181.8–1,677.4)	112.9 (74.2–155.6)	93.1 (56.8–137.5)	239.8 (170.5–317.8)	215.2 (141.3–305.7)	111.1 (77.9–146.3)	20.9 (4.8–57.2)	77.4 (40.7–123.7)	54.1 (24.9–95.2)	112.0 (51.0–195.0)	53.1 (29.2–87.6)
Eritrea	Rate: Cumulative change (%)	-56.6 (-63.7 to -48.0)	-23.4 (-51.6 to 15.6)	1.8 (-48.4 to 73.6)	-52.2 (-71.7 to -19.1)	-59.6 (-79.2 to -27.1)	18.0 (-33.0 to 85.2)	-72.0 (-92.9 to -24.4)	-2.9 (-55.6 to 72.1)	-28.9 (-72.5 to 35.9)	-53.7 (-85.5 to 6.6)	-30.0 (-71.5 to 49.7)
Ethiopia	Number: 1990	449,901 (437,573–462,837)	25,712 (16,032–35,375)	26,536 (16,282–38,292)	63,697 (38,724–84,385)	61,100 (29,555–87,958)	15,512 (9,435–23,206)	18,891 (5,451–37,649)	16,282 (8,188–28,693)	17,883 (7,563–32,810)	28,914 (9,658–54,163)	20,066 (9,615–33,499)
Ethiopia	Number: 2015	189,635 (172,292–209,497)	14,640 (9,114–20,518)	18,540 (11,553–26,368)	25,970 (19,065–33,397)	14,662 (9,205–21,084)	17,225 (10,520–22,753)	620 (209–1,184)	14,591 (7,732–23,794)	11,647 (5,082–20,367)	8,360 (3,800–14,605)	7,861 (4,680–12,094)
Ethiopia	Number: Cumulative change (%)	-57.8 (-61.8 to -53.3)	-40.9 (-63.9 to -9.7)	-26.7 (-57.0 to 16.4)	-56.8 (-73.2 to -31.5)	-73.0 (-86.5 to -48.5)	20.7 (-47.4 to 97.5)	-95.8 (-98.8 to -90.1)	-0.8 (-54.3 to 77.3)	-25.0 (-73.4 to 49.5)	-62.6 (-89.4 to -9.5)	-55.0 (-80.0 to -12.0)
Ethiopia	Rate: 1990	4,655.6 (4,524.4–4,803.2)	237.7 (148.5–326.4)	245.0 (150.6–352.9)	655.8 (387.3–873.9)	647.1 (300.5–934.4)	153.8 (91.6–232.9)	205.3 (58.9–407.9)	151.7 (76.5–265.2)	166.1 (70.7–302.9)	316.1 (104.8–600.8)	210.8 (99.1–358.8)
Ethiopia	Rate: 2015	1,266.8 (1,151.1–1,402.0)	96.2 (60.0–134.7)	121.8 (75.9–173.2)	174.0 (127.2–224.1)	98.9 (62.1–142.1)	114.8 (70.1–151.7)	4.2 (1.4–8.0)	96.1 (51.0–156.5)	76.6 (33.4–133.8)	56.7 (25.6–99.1)	52.9 (31.3–81.3)
Ethiopia	Rate: Cumulative change (%)	-72.8 (-75.4 to -69.8)	-58.0 (-74.3 to -35.9)	-47.9 (-69.4 to -17.3)	-71.7 (-82.9 to -54.2)	-82.7 (-91.5 to -66.8)	-18.3 (-65.0 to 35.4)	-97.4 (-99.2 to -93.8)	-30.0 (-67.5 to 24.7)	-47.0 (-81.1 to 5.6)	-76.7 (-93.5 to -43.7)	-70.9 (-87.4 to -41.2)
Kenya	Number: 1990	90,365 (88,547–92,303)	4,530 (3,875–5,241)	7,009 (5,645–8,126)	17,299 (15,559–19,201)	12,143 (10,644–13,850)	4,515 (3,929–5,147)	8,739 (5,193–12,056)	2,725 (1,955–3,685)	2,177 (1,615–2,870)	4,480 (3,559–5,504)	3,122 (2,170–4,206)
Kenya	Number: 2015	79,235 (75,088–83,721)	7,646 (5,875–9,500)	8,678 (7,377–9,997)	11,999 (10,208–13,936)	8,915 (7,256–10,762)	6,368 (3,913–8,037)	3,205 (1,133–5,952)	3,970 (2,807–5,210)	2,416 (1,727–3,265)	3,094 (2,346–4,141)	2,524 (1,670–4,220)
Kenya	Number: Cumulative change (%)	-12.3 (-17.4 to -7.0)	70.2 (26.1–123.8)	24.8 (2.7–50.0)	-30.5 (-40.9 to -19.2)	-26.4 (-39.6 to -11.1)	40.6 (1.8–65.9)	-63.6 (-82.7 to -36.7)	47.9 (11.2–95.3)	13.4 (-24.2 to 57.4)	-30.0 (-48.6 to -2.7)	-19.2 (-40.8 to 13.6)
Kenya	Rate: 1990	1,971.3 (1,931.0–2,013.2)	95.8 (82.0–110.8)	148.3 (119.4–171.9)	377.5 (339.3–418.8)	266.9 (233.8–304.0)	97.5 (84.9–111.1)	194.6 (115.7–268.1)	57.8 (41.5–78.1)	46.1 (34.2–60.8)	99.5 (79.2–122.5)	68.4 (47.6–92.3)
Kenya	Rate: 2015	1,063.0 (1,006.8–1,123.4)	101.3 (77.8–125.9)	115.0 (97.8–132.5)	161.1 (137.1–187.1)	120.3 (98.0–145.3)	85.2 (52.4–107.6)	43.6 (15.4–80.9)	52.7 (37.2–69.1)	32.0 (22.9–43.3)	42.0 (31.9–56.2)	34.0 (22.5–56.9)
Kenya	Rate: Cumulative change (%)	-46.1 (-49.2 to -42.8)	6.7 (-21.0 to 40.3)	-21.8 (-35.6 to -6.0)	-57.2 (-63.7 to -50.2)	-54.8 (-62.9 to -45.3)	-12.8 (-37.1 to 3.0)	-77.8 (-89.5 to -61.3)	-7.4 (-30.4 to 22.2)	-29.0 (-52.6 to -1.5)	-57.2 (-68.6 to -40.6)	-50.4 (-63.7 to -30.1)
Madagascar	Number: 1990	72,088 (68,486–75,561)	5,998 (4,653–7,370)	2,394 (1,734–3,152)	10,814 (7,544–13,077)	13,590 (8,676–16,837)	2,644 (1,988–3,293)	4,395 (1,865–7,470)	3,024 (1,712–4,690)	2,561 (1,214–4,099)	6,410 (3,615–8,829)	1,797 (968–2,932)
Madagascar	Number: 2015	52,348 (43,929–62,462)	5,333 (3,806–7,066)	2,770 (1,657–4,177)	7,804 (5,600–10,432)	6,672 (4,509–9,234)	3,647 (2,464–4,926)	2,236 (983–3,928)	3,569 (2,032–5,557)	2,389 (1,203–4,035)	4,102 (2,026–6,955)	1,392 (790–2,356)
Madagascar	Number: Cumulative change (%)	-27.3 (-40.0 to -12.4)	-9.9 (-37.2 to 21.5)	19.3 (-34.3 to 90.7)	-25.5 (-51.1 to 10.8)	-47.9 (-68.1 to -14.2)	41.7 (-16.6 to 109.7)	-42.9 (-75.5 to 10.8)	27.0 (-38.8 to 123.5)	7.2 (-59.8 to 121.6)	-23.4 (-70.2 to 33.7)	-13.7 (-62.0 to 79.5)
Madagascar	Rate: 1990	3,175.8 (3,014.8–3,334.0)	241.5 (187.9–297.4)	96.3 (69.7–127.0)	469.9 (324.6–572.4)	615.2 (388.7–767.5)	111.3 (82.8–139.1)	201.4 (84.1–345.5)	122.8 (69.2–189.5)	104.3 (49.7–168.5)	299.4 (167.9–414.3)	79.1 (42.3–132.2)
Madagascar	Rate: 2015	1,352.1 (1,132.4–1,619.2)	133.9 (95.6–177.3)	69.6 (41.7–104.7)	201.3 (143.6–269.7)	174.6 (117.6–243.1)	93.3 (63.1–126.0)	59.1 (26.0–104.2)	89.9 (51.1–139.8)	60.2 (30.2–101.6)	108.6 (53.8–186.2)	36.1 (20.3–61.5)
Madagascar	Rate: Cumulative change (%)	-57.4 (-64.9 to -48.6)	-43.8 (-60.8 to -24.2)	-25.5 (-59.0 to 19.4)	-55.6 (-70.9 to -33.2)	-69.7 (-81.6 to -49.8)	-13.6 (-49.8 to 28.1)	-67.0 (-86.0 to -36.3)	-21.3 (-62.4 to 38.9)	-33.6 (-75.1 to 37.6)	-56.4 (-83.2 to -24.2)	-48.8 (-78.2 to 5.7)
Malawi	Number: 1990	97,375 (93,500–101,229)	5,886 (4,284–7,659)	4,038 (2,602–5,660)	10,121 (7,448–13,227)	16,991 (12,120–22,330)	2,835 (1,955–4,332)	30,713 (19,653–41,741)	2,488 (1,292–4,104)	2,079 (1,012–3,619)	5,087 (2,734–8,487)	3,668 (2,041–6,153)
Malawi	Number: 2015	54,386 (46,873–62,558)	4,129 (2,829–5,624)	4,082 (2,540–5,733)	8,105 (6,060–10,492)	5,400 (3,660–7,542)	3,703 (2,715–4,728)	8,488 (4,936–12,684)	2,827 (1,550–4,701)	1,807 (809–3,401)	3,064 (1,386–5,665)	2,292 (1,374–3,733)
Malawi	Number: Cumulative change (%)	-44.1 (-52.0 to -35.4)	-28.2 (-53.2 to 3.3)	6.9 (-41.9 to 76.8)	-17.6 (-46.0 to 17.7)	-67.2 (-79.9 to -51.2)	39.4 (-27.3 to 105.5)	-71.2 (-84.4 to -53.5)	25.8 (-44.9 to 142.6)	0.6 (-68.1 to 104.7)	-32.6 (-75.9 to 41.2)	-30.2 (-71.1 to 30.9)
Malawi	Rate: 1990	5,222.4 (5,007.7–5,441.5)	278.6 (204.3–363.1)	190.9 (123.5–267.0)	540.1 (394.2–710.7)	927.4 (657.0–1,227.8)	142.7 (97.2–222.1)	1,708.5 (1,096.1–2,329.8)	118.4 (61.8–195.1)	98.9 (48.4–171.4)	286.2 (151.1–479.0)	197.6 (108.7–338.1)
Malawi	Rate: 2015	1,775.7 (1,525.2–2,043.3)	129.7 (88.9–176.5)	128.2 (80.0–179.9)	265.0 (197.7–344.0)	178.7 (121.1–247.9)	119.3 (87.5–152.4)	283.4 (165.1–422.4)	89.0 (48.8–147.9)	56.9 (25.5–106.9)	102.8 (46.4–192.6)	75.5 (45.1–123.4)
Malawi	Rate: Cumulative change (%)	-66.0 (-70.8 to -60.6)	-52.4 (-69.0 to -31.5)	-29.0 (-61.3 to 17.5)	-49.5 (-67.1 to -27.2)	-80.1 (-87.8 to -70.1)	-10.2 (-53.7 to 33.0)	-82.7 (-90.6 to -71.9)	-16.9 (-63.4 to 59.5)	-33.5 (-78.8 to 34.3)	-59.7 (-85.8 to -13.3)	-57.0 (-82.3 to -18.7)
Mozambique	Number: 1990	132,115 (129,221–135,329)	6,583 (4,221–9,356)	6,134 (3,786–8,980)	14,818 (11,180–19,476)	13,791 (9,322–19,231)	3,792 (2,677–5,301)	44,399 (30,795–56,391)	4,406 (2,277–7,211)	4,159 (2,042–6,893)	5,071 (2,248–9,531)	3,340 (1,826–5,486)
Mozambique	Number: 2015	83,363 (76,663–91,079)	5,131 (3,257–7,120)	6,906 (4,502–9,730)	6,557 (4,887–8,541)	4,382 (2,782–6,400)	6,054 (4,233–7,904)	16,345 (10,369–22,017)	5,228 (2,823–8,329)	3,861 (1,808–6,763)	1,559 (650–3,129)	2,009 (1,118–3,279)

© 2017 Global Burden of Disease Child and Adolescent Health Collaboration. JAMA Pediatrics.

eTable 2. Number of Deaths, Death Rates (per 100,000 population), and Cumulative Percent Change with 95% Uncertainty Intervals (UI) for the Top 10 Global Causes of Death in 195 Countries and Territories, Aged Under 5 Years, Both Sexes, 1990 and 2015*Note: best viewed by enlarging in browser.*

Location	Measure	All causes	Neonatal preterm birth complications	Neonatal encephalopathy due to birth asphyxia and trauma	Lower respiratory infections	Diarrheal diseases	Congenital anomalies	Malaria	Neonatal sepsis and other neonatal infections	Other neonatal disorders	Protein-energy malnutrition	Meningitis
Mozambique	Number: Cumulative change (%)	-36.9 (-42.2 to -30.9)	-18.5 (-52.6 to 28.2)	20.9 (-37.0 to 99.3)	-54.3 (-71.2 to -32.3)	-66.5 (-82.1 to -44.4)	68.5 (-11.9 to 152.6)	-61.8 (-78.0 to -40.3)	32.0 (-40.5 to 163.2)	6.3 (-62.2 to 121.8)	-63.6 (-89.7 to -19.5)	-32.2 (-72.5 to 38.7)
Mozambique	Rate: 1990	4,943.8 (4,823.2–5,068.7)	221.3 (141.9–315.0)	206.0 (127.0–301.6)	547.4 (411.9–719.6)	524.3 (354.3–732.5)	136.0 (95.5–193.3)	1,726.2 (1,199.7–2,191.7)	149.3 (77.2–244.0)	140.5 (69.4–232.8)	198.7 (87.8–370.0)	125.3 (68.2–208.3)
Mozambique	Rate: 2015	1,656.2 (1,521.8–1,810.8)	98.6 (62.6–136.8)	132.6 (86.6–186.9)	130.1 (96.6–169.9)	88.0 (55.8–127.5)	119.2 (83.3–155.4)	332.2 (209.8–446.7)	100.7 (54.4–160.3)	74.3 (34.8–130.0)	31.9 (13.2–64.5)	40.2 (22.2–64.9)
Mozambique	Rate: Cumulative change (%)	-66.5 (-69.3 to -63.3)	-53.5 (-73.0 to -26.6)	-30.9 (-64.0 to 13.9)	-75.4 (-84.5 to -63.5)	-82.3 (-90.6 to -70.3)	-7.2 (-52.0 to 39.6)	-80.0 (-88.5 to -68.9)	-25.1 (-66.1 to 49.0)	-39.6 (-78.5 to 26.8)	-81.0 (-94.6 to -57.4)	-63.8 (-85.5 to -25.2)
Rwanda	Number: 1990	50,553 (48,276–52,838)	3,369 (2,366–4,455)	2,185 (1,361–3,160)	8,963 (6,593–11,508)	4,772 (2,724–7,473)	2,155 (1,394–2,992)	6,590 (1,945–13,026)	1,917 (1,114–3,105)	1,600 (832–2,688)	4,732 (2,400–8,206)	1,630 (898–2,622)
Rwanda	Number: 2015	23,768 (20,026–28,285)	2,234 (1,494–3,040)	1,996 (1,209–2,876)	4,079 (3,016–5,379)	1,590 (924–2,401)	2,363 (1,551–3,300)	800 (249–1,572)	1,741 (980–2,821)	1,029 (507–1,755)	1,589 (697–3,059)	960 (534–1,564)
Rwanda	Number: Cumulative change (%)	-52.9 (-60.6 to -43.8)	-31.7 (-56.3 to 3.3)	-2.8 (-48.4 to 64.9)	-53.1 (-69.3 to -30.4)	-64.1 (-81.7 to -38.0)	14.2 (-34.2 to 73.3)	-84.7 (-95.7 to -62.7)	-2.2 (-53.9 to 80.6)	-27.6 (-73.1 to 48.3)	-62.1 (-87.1 to -21.0)	-34.4 (-72.4 to 25.0)
Rwanda	Rate: 1990	3,416.3 (3,265.6–3,573.6)	220.4 (154.8–291.3)	142.9 (89.0–207.1)	606.4 (445.9–780.9)	325.3 (186.2–510.4)	144.1 (93.3–200.5)	450.9 (132.8–891.0)	125.9 (73.2–203.6)	105.0 (54.8–176.6)	324.6 (163.7–563.4)	110.7 (60.6–178.5)
Rwanda	Rate: 2015	1,378.2 (1,161.5–1,640.1)	128.6 (86.1–174.9)	114.9 (69.6–165.6)	237.0 (175.2–312.7)	92.6 (53.8–139.8)	137.2 (90.1–191.6)	46.6 (14.5–91.6)	100.4 (56.6–162.7)	59.3 (29.3–101.2)	92.6 (40.6–178.3)	55.8 (31.1–91.0)
Rwanda	Rate: Cumulative change (%)	-59.6 (-66.2 to -51.7)	-39.9 (-61.6 to -9.1)	-14.5 (-54.6 to 44.8)	-59.7 (-73.8 to -40.1)	-69.4 (-84.5 to -47.1)	-0.7 (-43.1 to 50.8)	-87.0 (-96.3 to -68.3)	-14.2 (-59.5 to 59.0)	-36.4 (-76.4 to 30.1)	-67.8 (-89.1 to -32.8)	-43.7 (-76.3 to 7.0)
Somalia	Number: 1990	56,010 (53,434–58,621)	2,835 (1,850–3,898)	1,583 (809–2,462)	12,084 (9,452–14,828)	10,316 (7,091–13,722)	1,759 (1,207–2,675)	2,866 (1,134–5,402)	1,596 (797–2,691)	1,550 (673–2,836)	5,439 (2,681–9,021)	1,630 (906–2,534)
Somalia	Number: 2015	51,688 (46,028–58,277)	3,212 (2,086–4,568)	2,164 (1,252–3,258)	11,116 (8,663–13,957)	9,140 (6,412–12,170)	2,321 (1,755–2,972)	1,956 (695–3,534)	1,854 (954–2,993)	1,586 (618–2,990)	4,264 (2,171–7,240)	1,670 (962–2,677)
Somalia	Number: Cumulative change (%)	-7.6 (-18.3 to 5.4)	16.7 (-25.6 to 70.7)	47.0 (-22.2 to 144.9)	-6.5 (-31.8 to 25.2)	-8.7 (-41.1 to 29.6)	38.1 (-14.7 to 92.1)	-25.4 (-68.6 to 39.0)	25.0 (-39.5 to 113.5)	13.4 (-54.9 to 124.4)	-11.1 (-63.4 to 86.0)	12.9 (-48.8 to 124.2)
Somalia	Rate: 1990	4,239.8 (4,037.2–4,447.2)	198.1 (129.6–272.8)	110.5 (56.6–171.8)	914.8 (713.5–1,125.0)	793.8 (545.9–1,056.3)	129.4 (88.3–199.6)	224.0 (88.4–423.4)	112.1 (56.2–188.2)	108.9 (47.1–198.4)	426.3 (208.8–713.3)	124.5 (68.2–194.9)
Somalia	Rate: 2015	2,434.0 (2,162.7–2,748.9)	143.1 (93.0–203.4)	96.4 (55.7–144.7)	525.2 (408.2–660.8)	437.3 (305.3–583.7)	107.4 (81.1–138.0)	94.6 (33.6–171.3)	83.0 (42.7–134.1)	70.9 (27.7–133.6)	207.0 (104.3–351.9)	79.5 (45.9–129.1)
Somalia	Rate: Cumulative change (%)	-42.5 (-49.2 to -34.3)	-25.6 (-52.5 to 8.9)	-6.3 (-50.3 to 55.9)	-41.6 (-57.9 to -21.5)	-43.2 (-63.5 to -18.7)	-12.9 (-46.8 to 21.0)	-53.8 (-80.6 to -13.8)	-20.5 (-61.4 to 36.6)	-27.9 (-71.2 to 42.1)	-44.9 (-77.4 to 16.1)	-29.3 (-68.6 to 41.1)
South Sudan	Number: 1990	53,055 (50,200–55,907)	2,832 (1,904–3,835)	1,571 (845–2,410)	8,383 (5,728–11,022)	10,393 (6,979–13,741)	1,506 (1,004–2,285)	5,380 (1,940–9,476)	1,520 (828–2,510)	1,418 (678–2,367)	4,574 (2,079–7,683)	2,503 (1,301–3,848)
South Sudan	Number: 2015	46,411 (39,803–53,767)	3,016 (2,006–4,299)	2,011 (1,175–3,107)	7,605 (5,482–9,975)	7,313 (5,004–9,906)	2,155 (1,610–2,850)	3,739 (1,329–7,232)	1,862 (1,014–3,064)	1,438 (702–2,517)	3,179 (1,558–5,653)	2,403 (1,308–3,819)
South Sudan	Number: Cumulative change (%)	-12.4 (-25.9 to 2.4)	9.6 (-29.7 to 64.7)	36.8 (-26.0 to 130.3)	-6.0 (-38.8 to 39.3)	-26.6 (-55.9 to 12.7)	51.4 (-10.2 to 120.5)	-24.5 (-66.9 to 43.8)	32.3 (-37.5 to 138.8)	14.6 (-54.5 to 128.3)	-19.9 (-71.0 to 68.2)	5.7 (-50.0 to 95.7)
South Sudan	Rate: 1990	4,565.5 (4,320.9–4,812.2)	224.4 (150.9–303.4)	124.4 (67.4–190.6)	717.8 (488.8–956.0)	905.1 (600.2–1,202.5)	125.4 (82.8–191.4)	477.0 (172.1–844.3)	121.1 (66.3–199.7)	112.9 (54.1–188.5)	406.5 (183.8–685.1)	216.1 (112.2–336.0)
South Sudan	Rate: 2015	2,316.7 (1,984.0–2,684.4)	141.8 (94.5–202.2)	94.5 (55.4–146.0)	379.8 (272.9–499.6)	370.4 (253.7–502.5)	105.2 (78.3–139.1)	191.9 (68.2–371.7)	87.9 (47.9–144.0)	67.8 (33.2–118.2)	163.7 (79.5–291.4)	121.0 (65.7–193.6)
South Sudan	Rate: Cumulative change (%)	-49.2 (-57.1 to -40.5)	-35.0 (-58.4 to -2.0)	-18.8 (-55.9 to 36.4)	-45.1 (-64.3 to -17.3)	-57.2 (-74.6 to -33.7)	-11.0 (-47.5 to 30.6)	-56.2 (-80.8 to -16.7)	-21.7 (-62.7 to 40.6)	-32.2 (-72.9 to 34.9)	-53.5 (-83.3 to -1.9)	-38.2 (-71.0 to 16.2)
Tanzania	Number: 1990	170,296 (166,770–174,112)	7,433 (4,994–10,094)	8,122 (5,298–11,453)	31,172 (24,821–37,440)	22,417 (16,577–28,525)	6,876 (5,340–8,824)	32,878 (20,554–46,092)	6,318 (3,606–9,953)	5,288 (2,653–8,827)	10,126 (5,679–15,953)	3,741 (2,346–5,752)
Tanzania	Number: 2015	121,970 (109,710–136,259)	8,900 (5,950–12,408)	10,108 (6,590–14,249)	17,712 (13,739–22,668)	5,852 (3,909–8,264)	14,868 (9,552–19,669)	9,560 (4,130–16,264)	8,978 (5,030–14,353)	5,727 (2,751–9,932)	6,786 (3,239–12,381)	4,137 (2,358–6,684)
Tanzania	Number: Cumulative change (%)	-28.4 (-35.7 to -20.3)	24.7 (-25.3 to 97.5)	30.9 (-27.3 to 104.8)	-42.2 (-58.8 to -21.3)	-73.1 (-84.5 to -57.1)	122.1 (23.4–213.6)	-70.0 (-86.0 to -47.8)	52.4 (-26.9 to 167.2)	22.8 (-55.1 to 141.2)	-26.6 (-71.6 to 50.1)	19.3 (-44.4 to 124.4)
Tanzania	Rate: 1990	3,447.3 (3,372.6–3,530.3)	138.6 (93.4–188.0)	151.3 (98.6–213.4)	629.2 (497.3–759.1)	458.9 (339.7–585.5)	134.6 (103.8–173.3)	692.4 (433.5–968.0)	118.2 (67.5–186.0)	98.8 (50.2–164.5)	210.9 (117.9–334.0)	76.1 (47.0–116.7)
Tanzania	Rate: 2015	1,263.0 (1,135.7–1,410.7)	89.9 (60.1–125.2)	102.1 (66.5–143.9)	184.1 (142.7–235.1)	61.2 (40.9–86.3)	153.1 (98.5–202.8)	101.4 (43.7–172.7)	90.8 (50.9–145.1)	57.9 (27.9–100.6)	71.5 (34.0–130.0)	43.1 (24.6–70.1)
Tanzania	Rate: Cumulative change (%)	-63.4 (-67.2 to -59.2)	-32.5 (-59.5 to 6.7)	-29.1 (-60.6 to 10.6)	-70.2 (-78.8 to -59.3)	-86.2 (-92.0 to -78.0)	17.0 (-36.0 to 66.2)	-84.9 (-92.9 to -73.8)	-17.7 (-60.5 to 43.8)	-33.6 (-75.6 to 30.6)	-62.9 (-85.7 to -23.9)	-38.7 (-72.1 to 15.6)
Uganda	Number: 1990	139,596 (136,612–142,869)	8,172 (5,884–10,731)	7,132 (4,586–10,106)	14,131 (10,699–17,986)	12,520 (8,673–16,867)	4,053 (2,686–5,409)	43,414 (32,408–54,090)	4,249 (2,343–7,130)	3,464 (1,747–6,050)	7,044 (3,314–12,669)	5,802 (3,379–9,623)
Uganda	Number: 2015	113,046 (104,198–123,259)	9,513 (6,541–12,670)	10,792 (7,089–14,521)	12,506 (9,392–16,378)	7,894 (5,309–10,946)	9,616 (6,498–12,799)	11,301 (4,762–18,865)	7,433 (4,174–11,775)	4,679 (2,346–8,027)	5,927 (2,756–11,107)	6,429 (3,855–10,355)
Uganda	Number: Cumulative change (%)	-19.0 (-25.1 to -11.9)	20.1 (-24.7 to 75.2)	59.4 (-9.3 to 149.5)	-9.5 (-37.2 to 27.0)	-34.0 (-62.2 to 3.9)	146.4 (43.0–255.8)	-73.6 (-88.8 to -53.8)	91.3 (-10.0 to 229.8)	55.5 (-44.6 to 211.8)	-2.8 (-65.1 to 109.0)	21.0 (-44.3 to 119.8)
Uganda	Rate: 1990	3,780.8 (3,694.0–3,869.7)	198.4 (142.6–260.3)	173.1 (111.4–244.9)	381.5 (289.0–490.4)	342.0 (238.0–462.5)	104.4 (69.4–140.7)	1,223.5 (916.5–1,526.8)	103.7 (57.1–174.2)	84.5 (42.6–147.2)	198.1 (92.8–361.5)	158.8 (91.0–264.2)
Uganda	Rate: 2015	1,467.0 (1,350.3–1,600.6)	119.3 (82.0–158.8)	135.3 (89.0–182.0)	163.3 (123.1–214.4)	103.8 (69.7–143.6)	123.7 (83.6–165.0)	150.5 (63.4–250.2)	93.5 (52.6–147.9)	58.8 (29.6–100.9)	78.6 (36.3–147.0)	84.4 (50.5–134.9)
Uganda	Rate: Cumulative change (%)	-61.2 (-64.3 to -57.6)	-38.0 (-61.0 to -9.5)	-17.7 (-53.0 to 29.1)	-56.2 (-69.7 to -38.1)	-68.2 (-82.1 to -50.1)	23.4 (-29.5 to 79.1)	-87.5 (-94.7 to -78.4)	-1.6 (-53.5 to 70.0)	-20.0 (-71.3 to 60.1)	-54.0 (-83.8 to -2.6)	-41.8 (-73.6 to 6.3)
Zambia	Number: 1990	61,910 (59,297–64,729)	2,840 (1,955–3,773)	2,543 (1,616–3,563)	10,177 (7,952–12,528)	9,270 (6,754–12,313)	2,134 (1,664–2,748)	12,067 (7,678–16,976)	1,881 (1,009–3,046)	1,364 (645–2,349)	4,202 (2,021–7,455)	2,440 (1,384–4,085)
Zambia	Number: 2015	41,673 (35,835–47,967)	2,596 (1,702–3,564)	3,158 (2,144–4,294)	5,072 (3,737–6,710)	3,374 (2,286–4,711)	3,312 (1,986–4,555)	4,511 (2,488–7,125)	2,518 (1,431–3,992)	1,420 (691–2,506)	2,001 (1,021–3,485)	1,453 (913–2,243)
Zambia	Number: Cumulative change (%)	-32.6 (-42.4 to -22.3)	-5.7 (-42.7 to 38.9)	31.2 (-24.1 to 113.0)	-49.2 (-66.0 to -29.7)	-62.3 (-78.6 to -42.5)	59.2 (-15.6 to 132.2)	-60.7 (-81.2 to -31.0)	45.0 (-29.9 to 149.4)	18.9 (-53.2 to 138.5)	-44.6 (-79.8 to 18.8)	-33.3 (-70.4 to 26.7)
Zambia	Rate: 1990	3,820.4 (3,655.5–4,001.4)	158.4 (109.5–210.1)	141.7 (90.1–198.2)	624.5 (485.1–771.5)	581.8 (420.3–771.4)	126.0 (97.4–163.4)	772.0 (492.2–1,079.4)	105.5 (56.5–170.7)	76.5 (36.2–131.6)	269.9 (127.5–474.9)	152.1 (84.0–258.7)
Zambia	Rate: 2015	1,390.4 (1,193.5–1,606.1)	83.8 (55.0–115.0)	102.0 (69.3–138.6)	169.2 (124.4–223.8)	113.8 (77.3–158.6)	109.4 (65.7–150.5)	154.0 (84.8–243.4)	81.5 (46.3–129.0)	45.9 (22.3–81.0)	68.4 (34.8–119.7)	48.9 (30.5–75.4)
Zambia	Rate: Cumulative change (%)	-63.6 (-68.9 to -57.9)	-45.4 (-66.8 to -19.5)	-24.0 (-55.9 to 22.9)	-72.4 (-81.6 to -61.6)	-79.7 (-88.6 to -69.3)	-10.7 (-53.4 to 31.5)	-79.0 (-90.0 to -63.3)	-16.4 (-59.4 to 43.4)	-31.5 (-73.0 to 35.5)	-70.4 (-89.3 to -36.2)	-63.8 (-84.2 to -31.2)
Central Sub-Saharan Africa	Number: 1990	414,777 (395,886–434,189)	20,547 (15,033–27,008)	15,424 (10,136–21,581)	54,515 (42,600–66,537)	36,637 (27,483–46,903)	14,087 (7,801–30,519)	87,267 (62,508–114,072)	17,562 (11,338–25,177)	12,130 (6,259–19,622)	26,042 (15,878–38,115)	12,403 (8,397–17,224)
Central Sub-Saharan Africa	Number: 2015	398,083 (345,269–457,698)	26,521 (19,021–34,515)	25,282 (16,308–36,295)	54,578 (42,519–68,157)	27,438 (19,927–37,260)	23,647 (16,707–36,617)	68,329 (43,461–95,882)	26,449 (16,884–38,542)	15,497 (8,060–25,347)	25,458 (14,228–43,805)	14,727 (9,598–22,812)
Central Sub-Saharan Africa	Number: Cumulative change (%)	-3.9 (-17.5 to 10.9)	32.2 (-8.0 to 86.0)	70.0 (1.2–157.7)	1.9 (-27.0 to 37.0)	-23.2 (-50.9 to 10.8)	88.5 (14.5–159.1)	-20.4 (-47.7 to 14.7)	56.8 (-6.5 to 150.8)	37.4 (-34.3 to 142.4)	3.6 (-48.4 to 88.0)	24.3 (-33.2 to 110.6)
Central Sub-Saharan Africa	Rate: 1990	3,809.2 (3,631.3–3,992.1)	170.0 (124.4–223.4)	127.7 (84.0–178.4)	495.4 (384.9–605.1)	336.2 (249.4–430.1)	122.9 (66.6–270.7)	833.4 (594.2–1,089.1)	145.7 (94.2–208.4)	100.9 (52.5–162.6)	245.3 (149.4–359.2)	114.6 (77.0–159.9)
Central Sub-Saharan Africa	Rate: 2015	1,830.7 (1,582.4–2,108.3)	116.6 (83.7–151.8)	111.3 (71.8–159.6)	250.8 (194.6–313.3)	126.6 (91.6–171.9)	106.6 (74.8–166.3)	323.9 (206.6–454.8)	116.5 (74.5–169.6)	68.3 (35.6–111.5)	119.2 (66.1–204.2)	68.3 (44.2–106.0)
Central Sub-Saharan Africa	Rate: Cumulative change (%)	-51.9 (-58.7 to -44.4)	-29.7 (-51.1 to -1.0)	-9.7 (-46.1 to 36.9)	-48.4 (-63.2 to -30.4)	-61.3 (-75.1 to -43.9)	-1.7 (-41.2 to 36.2)	-60.5 (-74.0 to -43.2)	-16.8 (-50.4 to 32.9)	-27.2 (-65.1 to 27.6)	-48.5 (-74.7 to -6.7)	-37.4 (-66.6 to 6.8)
Angola	Number: 1990	108,922 (99,967–118,896)	4,796 (3,092–6,797)	3,492 (1,929–5,362)	16,751 (9,924–23,678)	13,609 (7,798–19,673)	3,890 (1,832–8,771)	9,717 (3,871–17,309)	4,606 (2,632–6,948)	3,148 (1,519–5,500)	8,166 (3,216–15,250)	4,084 (1,960–6,937)
Angola	Number: 2015	86,594 (68,997–105,800)	5,507 (3,376–8,047)	4,947 (2,884–7,557)	11,621 (7,863–16,016)	6,116 (3,664–9,349)	6,208 (4,142–9,792)	11,106 (5,524–18,754)	6,777 (3,728–10,419)	3,480 (1,629–6,006)	5,161 (2,246–9,592)	3,752 (1,996–6,360)
Angola	Number: Cumulative change (%)	-20.3 (-37.5 to -0.6)	19.1 (-31.5 to 80.1)	51.5 (-22.4 to 158.2)	-26.2 (-56.1 to 20.7)	-51.4 (-75.4 to -13.9)	91.0 (-5.4 to 205.0)	33.5 (-42.4 to 174.9)	57.0 (-21.5 to 170.4)	26.0 (-53.5 to 160.7)	-21.5 (-75.9 to 85.4)	5.5 (-58.4 to 119.5)
Angola	Rate: 1990	4,388.3 (4,022.8–4,794.7)	171.2 (110.0–242.3)	124.8 (68.8–191.8)	670.7 (390.9–957.5)	548.9 (315.5–798.0)	148.4 (67.3–344.6)	407.8 (161.3–724.8)	164.8 (94.3–248.3)	113.1 (55.2–196.5)	339.7 (131.7–641.6)	166.2 (79.7–283.1)
Angola	Rate: 2015	1,679.2 (1,333.2–2,057.4)	102.0 (62.4–149.1)	91.7 (53.4–139.9)	225.5 (152.9–311.9)	119.1 (71.9–182.8)	118.1 (78.5–186.5)	221.8 (109.2–374.5)	125.7 (69.3–192.9)	64.7 (30.4–111.1)	101.9 (44.1–191.8)	73.5 (38.7–124.3)
Angola	Rate: Cumulative change (%)	-61.6 (-70.0 to -52.0)	-38.2 (-64.4 to -6.8)	-21.4 (-59.6 to 34.3)	-64.1 (-78.9 to -40.6)	-76.4 (-88.2 to -57.3)	-3.3 (-54.0 to 55.8)	-36.3 (-72.5 to 31.2)	-18.7 (-59.1 to 40.1)	-34.9 (-75.9 to 33.9)	-62.6 (-88.6 to -11.9)	-48.8 (-79.8 to 9.9)
Central African Republic	Number: 1990	21,211 (19,843–22,710)	1,402 (920–1,911)	940 (548–1,390)	3,362 (2,473–4,272)	2,323 (1,554–3,283)	761 (391–1,697)	3,111 (1,587–4,705)	995 (553–1,617)	690 (321–1,217)	1,374 (599–2,393)	773 (431–1,241)
Central African Republic	Number: 2015	20,576 (16,592–25,488)	1,526 (972–2,256)	1,143 (662–1,774)	3,222 (2,246–4,423)	1,697 (1,046–2,496)	934 (527–1,720)	3,448 (1,503–5,593)	1,230 (640–2,006)	811 (336–1,487)	1,266 (545–2,367)	737 (384–1,281)
Central African Republic	Number: Cumulative change (%)	-2.8 (-23.5 to 21.0)	12.3 (-32.9 to 68.7)	29.9 (-34.2 to 126.7)	-1.8 (-35.5 to 41.3)	-23.2 (-57.5 to 27.3)	33.8 (-15.7 to 87.6)	15.4 (-37.0 to 89.2)	34.0 (-39.5 to 156.2)	33.8 (-52.8 to 174.1)	8.6 (-65.6 to 148.9)	4.6 (-54.4 to 105.4)
Central African Republic	Rate: 1990	4,010.2 (3,750.0–4,295.5)	244.4 (160.3–332.7)	164.1 (95.7–242.3)	633.7 (462.1–810.4)	440.9 (295.4–621.1)	139.1 (70.7–315.2)	614.7 (312.0–926.2)	174.2 (97.3–283.4)	121.0 (56.4–212.5)	266.5 (117.4–460.3)	147.7 (81.5–238.3)
Central African Republic	Rate: 2015	2,769.2 (2,231.5–3,420.0)	195.7 (124.4–289.4)	146.7 (85.1–227.1)	433.8 (301.0–596.6)	229.7 (141.4–338.5)	123.2 (69.3–228.4)	478.7 (208.6–778.9)	158.3 (83.0–257.9)	104.4 (43.4–191.7)	173.7 (75.7–322.1)	100.1 (51.9–173.6)
Central African Republic	Rate: Cumulative change (%)	-30.8 (-45.7 to -13.7)	-17.4 (-50.7 to 23.3)	-4.5 (-51.6 to 66.4)	-29.8 (-54.3 to 1.5)	-45.3 (-69.6 to -8.6)	-3.0 (-39.9 to 36.0)	-18.9 (-56.0 to 33.5)	-1.7 (-55.3 to 87.5)	-1.9 (-65.3 to 101.0)	-23.2 (-75.7 to 74.1)	-25.5 (-67.7 to 46.6)
Congo	Number: 1990	8,321 (7,830–8,784)	505 (303–729)	442 (256–673)	913 (676–1,193)	540 (354–751)	465 (314–758)	1,183 (654–1,733)	560 (311–886)	307 (143–550)	462 (200–906)	267 (146–438)
Congo	Number: 2015	9,241 (7,534–11,147)	698 (385–1,068)	706 (390–1,082)	932 (636–1,301)	373 (209–586)	844 (592–1,157)	1,322 (567–2,302)	907 (479–1,400)	459 (216–811)	413 (159–812)	311 (157–540)
Congo	Number: Cumulative change (%)	11.2 (-10.5 to 35.5)	45.0 (-24.7 to 142.0)	71.2 (-17.4 to 189.4)	4.9 (-35.1 to 57.8)	-27.6 (-63.4 to 24.4)	91.0 (17.7–177.6)	13.9 (-40.6 to 87.6)	76.4 (-20.3 to 217.9)	74.5 (-42.0 to 263.3)	6.6 (-68.1 to 146.8)	26.0 (-44.9 to 154.6)
Congo	Rate: 1990	1,998.4 (1,880.8–2,109.4)	115.8 (69.5–167.0)	101.4 (58.9–154.3)	219.0 (162.0–287.5)	130.0 (85.3–181.2)	109.1 (73.4–179.4)	290.9 (161.2–427.7)	128.5 (71.6–203.2)	70.6 (32.9–126.0)	112.8 (48.4–222.3)	64.7 (35.2–105.7)
Congo	Rate: 2015	1,176.6 (959.4–1,419.5)	87.3 (48.1–133.4)	88.3 (48.8–135.4)	118.9 (81.0–165.8)	47.8 (26.8–74.8)	106.5 (74.7–146.8)	171.2 (73.5–299.5)	113.4 (60.0–175.0)	57.5 (27.2–101.4)	53.3 (20.6–104.0)	40.0 (20.2–69.0)
Congo	Rate: Cumulative change (%)	-41.0 (-52.7 to -28.0)	-20.9 (-58.9 to 32.0)	-6.7 (-54.9 to 57.7)	-44.2 (-65.6 to -15.9)	-61.6 (-80.5 to -34.4)	3.0 (-36.8 to 50.0)	-40.0 (-68.6 to -1.2)	-3.9 (-56.5 to 73.1)	-5.1 (-68.3 to 97.5)	-43.7 (-83.4 to 31.3)	-33.2 (-70.6 to 35.2)
Democratic Republic of the Congo	Number: 1990	270,533 (254,527–287,637)	13,450 (8,640–18,657)	10,237 (5,766–15,676)	32,706 (24,567–41,424)	19,715 (13,489–26,420)	8,648 (4,621–19,050)	72,240 (52,071–93,017)	10,994 (5,941–17,562)	7,749 (2,995–14,428)	15,711 (8,140–25,784)	7,073 (4,265–10,664)
Democratic Republic of the Congo	Number: 2015	276,807 (226,504–333,422)	18,401 (11,791–25,982)	18,158 (9,709–28,624)	38,357 (27,490–50,710)	19,117 (12,222–27,809)	15,180 (9,813–25,337)	51,500 (32,049–73,479)	16,992 (8,928–27,820)	10,472 (3,844–20,089)	18,437 (8,461–34,576)	9,759 (5,354–16,600)
Democratic Republic of the Congo	Number: Cumulative change (%)	2.5 (-17.5 to 24.4)	43.5 (-15.6 to 133.6)	90.0 (-4.8 to 226.2)	20.1 (-21.6 to 72.3)	0.8 (-42.6 to 60.0)	96.1 (15.6–184.4)	-27.4 (-54.1 to 7.7)	68.7 (-22.5 to 222.6)	56.9 (-46.5 to 248.9)	29.9 (-50.7 to 168.5)	48.0 (-34.5 to 183.1)
Democratic Republic of the Congo	Rate: 1990	3,746.0 (3,511.5–3,975.3)	167.7 (108.4–232.5)	127.8 (72.1–195.7)	446.3 (334.5–571.1)	271.7 (186.9–364.0)	113.5 (59.5–252.6)	1,041.4 (750.2–1,342.9)	137.4 (74.3–218.8)	97.1 (38.0–180.3)	222.4 (114.7–371.4)	98.2 (58.3–149.9)
Democratic Republic of the Congo	Rate: 2015	1,885.9 (1,537.7–2,277.7)	119.7 (76.7–169.1)	118.2 (63.3–186.2)	260.9 (186.5–344.8)	130.7 (83.9–190.2)	101.3 (65.4–170.2)	362.1 (225.3–516.9)	110.7 (58.2–181.0)	68.3 (25.1–131.2)	128.0 (58.7–240.1)	67.0 (36.7–115.0)
Democratic Republic of the Congo	Rate: Cumulative change (%)	-49.6 (-59.6 to -38.6)	-25.1 (-56.0 to 21.6)	-1.0 (-50.4 to 69.7)	-40.1 (-61.1 to -14.0)	-50.0 (-71.7 to -20.2)	0.5 (-41.9 to 47.4)	-64.6 (-77.6 to -47.5)	-12.2 (-59.6 to 68.0)	-18.5 (-72.2 to 80.8)	-36.3 (-75.6 to 30.7)	-26.6 (-67.8 to 40.5)
Equatorial Guinea	Number: 1990	2,900 (2,657–3,145)	172 (112–241)	131 (76–198)	483 (350–611)	277 (177–393)	114 (61–241)	488 (308–685)	144 (81–226)	92 (43–165)	200 (93–356)	107 (57–175)
Equatorial Guinea	Number: 2015	2,515 (1,975–3,192)	168 (66–292)	134 (64–227)	233 (135–382)	64 (33–108)	193 (112–301)	691 (360–1,045)	237 (122–372)	120 (44–245)	94 (31–196)	85 (40–155)
Equatorial Guinea	Number: Cumulative change (%)	-13.0 (-32.6 to 10.4)	0.2 (-58.6 to 76.6)	12.0 (-56.3 to 121.8)	-50.6 (-72.9 to -19.3)	-75.6 (-88.8 to -54.5)	96.3 (-8.2 to 219.7)	50.7 (-29.5 to 158.8)	78.0 (-18.7 to 228.5)	58.3 (-58.0 to 283.4)	-44.8 (-85.2 to 25.2)	-11.4 (-67.9 to 78.6)
Equatorial Guinea	Rate: 1990	3,869.8 (3,538.7–4,202.7)	205.7 (133.8–287.9)	157.6 (91.4–236.4)	640.2 (463.8–815.3)	370.8 (238.2–523.8)	145.4 (76.0–310.8)	688.3 (433.1–967.6)	172.6 (98.3–270.0)	111.6 (52.6–198.0)	277.5 (127.4–498.9)	145.0 (77.6–238.5)
Equatorial Guinea	Rate: 2015	1,874.5 (1,469.6–2,384.0)	120.3 (47.7–209.2)	96.3 (45.8–162.3)	173.3 (100.8–283.7)	47.9 (25.0–81.3)	141.3 (81.4–219.9)	530.1 (276.8–800.1)	170.1 (87.9–266.9)	86.4 (31.6–176.2)	71.5 (23.9–148.4)	64.3 (30.8–116.5)
Equatorial Guinea	Rate: Cumulative change (%)	-51.4 (-62.3 to -38.1)	-40.1 (-75.2 to 5.7)	-33.1 (-73.8 to 32.6)	-72.3 (-84.7 to -54.6)	-86.4 (-93.7 to -74.6)	14.0 (-47.7 to 86.2)	-18.0 (-61.6 to 41.0)	6.3 (-51.2 to 96.1)	-6.0 (-75.0 to 128.7)	-69.7 (-92.0 to -30.3)	-50.7 (-82.2 to -0.4)
Gabon	Number: 1990	2,888 (2,766–3,013)	220 (112–344)	180 (96–280)	297 (206–406)	172 (106–254)	206 (138–296)	526 (301–741)	261 (140–403)	141 (63–263)	127 (53–253)	96 (51–163)
Gabon	Number: 2015	2,347 (2,086–2,625)	218 (97–360)	191 (104–297)	211 (146–292)	68 (38–111)	287 (204–383)	259 (78–507)	305 (170–469)	153 (59–284)	84 (31–177)	78 (38–139)
Gabon	Number: Cumulative change (%)	-18.7 (-28.2 to -8.1)	2.6 (-48.4 to 62.0)	16.2 (-45.6 to 113.8)	-27.4 (-52.0 to 2.0)	-58.0 (-78.6 to -27.5)	44.3 (-6.6 to 101.5)	-51.4 (-81.9 to -15.7)	26.1 (-38.8 to 118.2)	25.7 (-57.2 to 154.9)	-19.6 (-77.4 to 93.3)	-12.5 (-62.2 to 75.7)
Gabon	Rate: 1990	1,774.8 (1,700.7–1,853.2)	130.3 (66.7–204.0)	106.7 (57.4–166.5)	182.6 (127.0–249.1)	106.1 (66.1–156.2)	124.7 (83.6–180.2)	333.6 (189.5–469.7)	154.8 (83.6–239.4)	84.0 (37.8–156.0)	79.5 (33.3–158.8)	59.7 (31.5–100.7)
Gabon	Rate: 2015	962.3 (855.2–1,076.2)	88.8 (39.5–146.3)	77.9 (42.6–121.0)	86.5 (60.0–119.8)	28.2 (15.6–45.6)	117.3 (83.7–156.6)	107.6 (32.8–210.3)	124.0 (69.2–190.7)	62.4 (24.2–115.8)	34.9 (12.9–72.9)	32.4 (16.0–57.7)
Gabon	Rate: Cumulative change (%)	-45.7 (-52.1 to -38.7)	-29.5 (-64.6 to 11.2)	-20.2 (-62.8 to 46.9)	-51.4 (-67.9 to -31.7)	-72.0 (-85.6 to -52.1)	-2.2 (-36.9 to 36.9)	-68.1 (-88.1 to -44.7)	-13.5 (-58.0 to 49.5)	-13.9 (-70.6 to 74.9)	-46.9 (-85.1 to 27.7)	-41.8 (-74.7 to 17.3)

© 2017 Global Burden of Disease Child and Adolescent Health Collaboration. JAMA Pediatrics.

eTable 3. Prevalent Cases, Rates (per 100,000 population), Years Lived with Disability (YLDs), and Cumulative Percent Change with 95% Uncertainty Interval (UI) for the Top 10 Global Causes of YLDs in Children and Adolescents in 195 Countries and Territories, Aged 0 to 19 Years, Both Sexes, 1990 and 2015. *Note: best viewed by enlarging in browser.*

Location	Measure	All causes	Iron-deficiency anemia	Skin and subcutaneous diseases	Asthma	Hemoglobinopathies and hemolytic anemias	Diarrheal diseases	Congenital anomalies	Protein-energy malnutrition	Epilepsy	Malaria	Neonatal preterm birth complications
Global	Prevalence Number: 1990	2,096,114,286 (2,094,418,127–2,097,905,277)	677,577,942 (673,476,771–681,770,346)	744,257,866 (740,651,409–747,923,727)	166,973,265 (143,108,405–193,573,462)	527,619,023 (522,781,137–533,758,859)	26,003,495 (24,326,649–27,845,783)	26,586,429 (25,735,926–27,367,490)	25,491,729 (24,577,932–26,450,748)	7,889,236 (7,093,040–8,763,880)	134,825,184 (117,856,399–152,253,386)	18,846,345 (16,979,581–20,792,274)
Global	Prevalence Number: 2015	2,289,784,742 (2,287,577,684–2,292,035,343)	713,016,539 (708,692,642–717,675,401)	841,794,320 (836,790,432–846,561,632)	158,151,385 (134,040,942–184,220,386)	590,315,873 (584,932,026–597,506,642)	23,261,098 (22,027,949–24,589,762)	33,930,983 (32,884,189–34,826,762)	22,448,815 (21,516,068–23,405,131)	8,507,896 (7,554,931–9,636,850)	185,157,379 (158,229,972–215,142,525)	19,663,514 (17,651,060–21,816,757)
Global	Prevalence Number: Cumulative change (%)	9.2 (9.1–9.3)	5.2 (4.3–6.2)	13.1 (12.5–13.7)	-5.3 (-8.7 to -1.7)	11.9 (10.7–13.1)	-10.5 (-12.6 to -8.6)	27.7 (23.8–31.5)	-11.9 (-16.7 to -6.9)	7.8 (2.8–12.9)	37.2 (29.2–46.4)	4.3 (1.2–7.7)
Global	Prevalence Rate: 1990	92,938.2 (92,864.7–93,018.5)	29,794.5 (29,621.5–29,969.0)	33,344.6 (33,186.0–33,514.6)	7,475.1 (6,405.8–8,638.5)	23,335.6 (23,122.2–23,606.5)	1,101.4 (1,031.6–1,177.9)	1,174.6 (1,136.9–1,209.2)	1,051.4 (1,013.7–1,090.9)	349.2 (313.5–388.2)	5,929.8 (5,184.6–6,697.5)	808.0 (727.6–891.6)
Global	Prevalence Rate: 2015	91,528.3 (91,440.9–91,617.8)	28,435.1 (28,264.6–28,618.9)	33,722.4 (33,522.2–33,913.6)	6,340.4 (5,375.4–7,390.3)	23,585.0 (23,369.5–23,872.7)	920.3 (871.6–972.4)	1,355.3 (1,313.4–1,391.2)	880.8 (844.2–918.3)	340.1 (301.8–385.4)	7,397.1 (6,322.4–8,594.0)	779.7 (699.8–865.0)
Global	Prevalence Rate: Cumulative change (%)	-1.5 (-1.6 to -1.4)	-4.6 (-5.4 to -3.7)	1.1 (0.6–1.7)	-15.2 (-18.3 to -12.0)	1.1 (0.0–2.2)	-16.4 (-18.2 to -14.7)	15.4 (12.0–18.9)	-16.2 (-20.8 to -11.4)	-2.6 (-7.2 to 1.9)	24.7 (17.4–32.7)	-3.5 (-6.5 to -0.3)
Global	YLDs Number: 1990	147,435,249 (111,701,992–187,797,915)	28,393,181 (20,627,358–37,642,670)	16,285,107 (11,107,036–22,842,153)	7,556,805 (5,067,820–10,374,727)	4,472,672 (3,244,976–5,921,027)	4,215,189 (3,060,249–5,563,405)	2,055,632 (1,399,553–2,875,110)	3,143,260 (2,188,166–4,219,816)	2,983,904 (2,175,079–3,809,871)	2,106,319 (1,521,751–2,807,897)	2,222,961 (1,752,098–2,751,039)
Global	YLDs Number: 2015	153,738,779 (116,785,455–196,217,161)	28,929,775 (20,830,847–38,454,844)	18,299,658 (12,574,942–25,679,362)	7,170,928 (4,774,552–9,817,467)	4,676,015 (3,382,839–6,203,593)	3,780,968 (2,734,670–4,977,004)	3,169,555 (2,137,768–4,398,279)	2,779,412 (1,925,398–3,696,012)	2,512,221 (1,802,858–3,250,325)	2,471,320 (1,790,256–3,265,298)	2,222,098 (1,740,048–2,778,174)
Global	YLDs Number: Cumulative change (%)	4.3 (3.1–5.6)	1.8 (0.2–3.5)	12.4 (11.5–13.3)	-5.1 (-8.6 to -1.6)	4.5 (3.0–6.0)	-10.3 (-12.3 to -8.2)	54.5 (38.2–72.2)	-11.5 (-16.5 to -6.4)	-15.8 (-20.9 to -10.2)	17.4 (13.8–20.7)	-0.1 (-3.2 to 3.2)
Global	YLDs Rate: 1990	6,562.3 (4,973.8–8,358.3)	1,247.5 (905.7–1,653.5)	725.8 (495.0–1,020.8)	338.3 (226.8–465.0)	198.2 (143.8–262.3)	178.5 (129.5–236.2)	90.8 (61.8–127.1)	129.6 (90.2–174.0)	132.0 (96.2–168.5)	92.8 (67.1–123.8)	97.9 (77.2–121.2)
Global	YLDs Rate: 2015	6,151.2 (4,672.3–7,850.8)	1,154.3 (831.2–1,534.6)	732.3 (503.0–1,028.0)	287.5 (191.4–393.4)	187.0 (135.3–248.1)	149.6 (108.2–196.8)	126.6 (85.4–175.7)	109.1 (75.5–145.0)	100.4 (72.1–129.9)	98.8 (71.5–130.5)	88.7 (69.5–110.9)
Global	YLDs Rate: Cumulative change (%)	-6.3 (-7.3 to -5.1)	-7.5 (-8.9 to -6.0)	0.9 (0.1–1.8)	-15.0 (-18.2 to -11.8)	-5.6 (-7.0 to -4.3)	-16.2 (-18.0 to -14.3)	39.7 (25.0–55.8)	-15.8 (-20.5 to -10.9)	-23.9 (-28.5 to -18.9)	6.4 (3.3–9.4)	-9.4 (-12.2 to -6.4)
High SDI	Prevalence Number: 1990	261,772,160 (261,062,236–262,524,122)	69,676,676 (68,443,010–71,092,670)	94,163,160 (93,444,121–94,902,829)	25,726,364 (22,372,202–29,193,738)	40,536,781 (40,037,589–41,078,502)	354,139 (332,352–377,695)	5,211,031 (5,015,820–5,436,305)	388,220 (277,239–538,639)	920,687 (817,027–1,049,850)	951 (521–1,531)	3,134,609 (2,912,640–3,361,128)
High SDI	Prevalence Number: 2015	226,113,174 (225,453,808–226,774,477)	61,970,977 (60,999,948–63,102,556)	81,551,525 (80,972,053–82,096,373)	16,969,001 (15,175,805–18,916,879)	33,076,375 (32,660,805–33,536,572)	208,573 (196,177–220,911)	4,816,429 (4,657,472–4,973,598)	233,687 (159,978–342,135)	797,827 (717,620–887,768)	173 (117–270)	2,609,151 (2,411,974–2,803,612)
High SDI	Prevalence Number: Cumulative change (%)	-13.6 (-13.9 to -13.4)	-11.0 (-13.3 to -8.5)	-13.4 (-14.1 to -12.7)	-33.9 (-37.0 to -30.6)	-18.4 (-19.6 to -17.3)	-41.1 (-43.4 to -39.2)	-7.5 (-11.1 to -4.3)	-37.2 (-64.1 to 1.0)	-13.2 (-17.9 to -8.2)	-80.1 (-89.6 to -65.6)	-16.8 (-19.6 to -13.9)
High SDI	Prevalence Rate: 1990	83,703.4 (83,465.8–83,947.1)	22,655.0 (22,230.0–23,144.2)	29,597.0 (29,372.7–29,825.9)	8,176.4 (7,108.7–9,283.0)	13,035.6 (12,875.8–13,210.8)	120.1 (112.4–128.1)	1,675.5 (1,613.2–1,748.0)	134.2 (95.8–186.2)	294.9 (261.7–336.5)	0.3 (0.2–0.5)	1,042.2 (969.1–1,115.1)
High SDI	Prevalence Rate: 2015	82,348.1 (82,100.0–82,595.8)	22,899.3 (22,535.2–23,325.4)	29,298.3 (29,091.0–29,496.4)	6,194.1 (5,534.3–6,902.0)	12,085.8 (11,933.9–12,253.6)	78.2 (73.5–82.9)	1,759.4 (1,701.3–1,816.8)	88.2 (60.4–129.2)	290.8 (261.9–323.7)	0.1 (0.0–0.1)	967.9 (895.4–1,039.5)
High SDI	Prevalence Rate: Cumulative change (%)	-1.6 (-1.9 to -1.3)	1.1 (-1.6 to 4.1)	-1.0 (-1.8 to -0.2)	-24.1 (-27.7 to -20.2)	-7.3 (-8.6 to -6.0)	-34.8 (-37.4 to -32.7)	5.0 (0.9–8.7)	-31.4 (-60.8 to 10.4)	-1.2 (-6.6 to 4.4)	-77.3 (-88.2 to -61.0)	-7.1 (-10.2 to -4.0)
High SDI	YLDs Number: 1990	16,531,570 (12,555,590–20,967,878)	2,528,546 (1,801,838–3,409,214)	2,171,286 (1,506,023–2,995,531)	1,171,081 (795,708–1,590,975)	444,284 (321,648–591,864)	58,260 (41,972–76,633)	426,402 (286,811–587,204)	48,643 (29,319–75,239)	278,375 (199,690–357,654)	21 (11–37)	342,115 (270,406–428,958)
High SDI	YLDs Number: 2015	13,873,053 (10,607,200–17,591,021)	2,268,673 (1,630,930–3,046,573)	1,903,844 (1,322,594–2,612,866)	773,452 (522,441–1,042,406)	333,309 (240,121–443,955)	34,413 (24,798–45,661)	525,720 (332,614–753,178)	29,393 (16,948–46,348)	142,020 (98,958–190,788)	5 (2–9)	234,807 (183,383–297,923)
High SDI	YLDs Number: Cumulative change (%)	-16.1 (-17.1 to -14.9)	-10.3 (-12.8 to -7.0)	-12.3 (-12.9 to -11.7)	-33.9 (-36.9 to -30.5)	-25.0 (-26.7 to -23.1)	-40.9 (-43.8 to -38.1)	22.8 (9.3–42.3)	-37.0 (-63.8 to 1.7)	-49.0 (-57.3 to -40.5)	-72.9 (-85.8 to -55.3)	-31.4 (-34.2 to -28.7)
High SDI	YLDs Rate: 1990	5,217.2 (3,960.1–6,618.3)	824.9 (587.3–1,112.0)	690.0 (479.5–949.4)	372.3 (252.7–505.9)	143.5 (103.9–191.1)	19.8 (14.2–26.0)	137.2 (92.2–188.9)	16.8 (10.1–26.0)	89.2 (64.1–114.7)	0.0 (0.0–0.0)	110.0 (87.0–137.9)
High SDI	YLDs Rate: 2015	5,004.2 (3,824.3–6,348.0)	844.3 (607.2–1,133.2)	690.2 (480.5–946.1)	282.4 (191.0–379.7)	123.7 (89.1–164.7)	12.9 (9.3–17.1)	192.1 (121.5–275.2)	11.1 (6.4–17.5)	51.8 (36.1–69.5)	0.0 (0.0–0.0)	85.8 (67.0–108.9)
High SDI	YLDs Rate: Cumulative change (%)	-4.1 (-5.3 to -2.7)	2.4 (-0.7 to 6.2)	0.0 (-0.6 to 0.7)	-24.0 (-27.6 to -20.2)	-13.9 (-15.9 to -11.7)	-34.7 (-37.9 to -31.5)	39.4 (24.1–61.6)	-31.2 (-60.5 to 11.1)	-42.0 (-51.4 to -32.3)	-69.0 (-83.7 to -48.6)	-22.1 (-25.2 to -19.1)
High-middle SDI	Prevalence Number: 1990	475,569,453 (474,830,926–476,298,698)	132,488,907 (130,993,489–133,917,994)	173,100,610 (171,853,077–174,292,029)	38,845,320 (33,300,839–44,787,532)	87,407,904 (86,619,278–88,173,103)	3,898,746 (3,634,440–4,196,499)	6,901,069 (6,586,300–7,226,194)	3,300,871 (2,982,351–3,690,110)	1,961,607 (1,766,516–2,159,997)	4,262,467 (3,220,051–5,417,825)	4,903,152 (4,449,892–5,381,991)
High-middle SDI	Prevalence Number: 2015	426,126,367 (425,413,822–426,864,299)	116,899,236 (115,702,302–118,157,014)	161,438,549 (160,380,027–162,468,725)	31,001,285 (26,289,681–36,118,641)	78,113,169 (77,421,080–78,792,488)	2,383,153 (2,232,325–2,538,805)	7,606,302 (7,277,354–7,938,451)	1,996,215 (1,835,269–2,209,471)	1,685,760 (1,493,509–1,912,783)	1,903,940 (1,294,711–2,749,965)	4,007,704 (3,624,318–4,405,864)
High-middle SDI	Prevalence Number: Cumulative change (%)	-10.4 (-10.6 to -10.2)	-11.8 (-13.0 to -10.4)	-6.7 (-7.3 to -6.1)	-20.2 (-23.9 to -16.7)	-10.6 (-11.5 to -9.8)	-38.8 (-41.7 to -36.5)	10.3 (5.1–15.6)	-39.3 (-47.2 to -30.8)	-14.1 (-19.4 to -8.5)	-55.7 (-62.2 to -44.2)	-18.3 (-21.4 to -14.9)
High-middle SDI	Prevalence Rate: 1990	92,278.1 (92,135.7–92,420.9)	25,732.7 (25,450.6–26,003.0)	33,554.7 (33,317.9–33,790.5)	7,599.3 (6,509.7–8,754.9)	16,955.0 (16,801.9–17,103.2)	744.2 (694.1–800.3)	1,337.9 (1,276.9–1,401.0)	623.1 (563.0–696.6)	380.5 (342.7–419.1)	832.8 (629.2–1,057.2)	946.6 (859.1–1,039.2)
High-middle SDI	Prevalence Rate: 2015	89,978.8 (89,825.5–90,137.2)	24,949.6 (24,692.9–25,223.6)	33,673.5 (33,451.2–33,892.2)	6,566.9 (5,563.3–7,652.5)	16,523.8 (16,377.4–16,667.8)	518.6 (485.3–552.7)	1,609.4 (1,539.8–1,679.5)	439.1 (403.7–486.0)	356.6 (315.5–404.9)	407.3 (275.7–591.1)	865.5 (783.1–950.6)
High-middle SDI	Prevalence Rate: Cumulative change (%)	-2.5 (-2.7 to -2.3)	-3.0 (-4.3 to -1.6)	0.4 (-0.3 to 1.0)	-13.6 (-17.6 to -9.8)	-2.5 (-3.5 to -1.6)	-30.3 (-33.5 to -27.6)	20.4 (14.7–26.1)	-29.3 (-38.5 to -19.4)	-6.3 (-12.0 to -0.1)	-51.5 (-59.0 to -38.3)	-8.6 (-12.1 to -4.9)
High-middle SDI	YLDs Number: 1990	31,598,302 (24,027,945–40,093,192)	5,242,882 (3,759,142–7,035,477)	3,857,551 (2,609,423–5,442,408)	1,824,744 (1,220,536–2,518,933)	814,861 (587,189–1,087,210)	661,550 (478,381–871,279)	518,610 (355,979–723,387)	426,932 (293,990–588,307)	778,105 (558,532–994,211)	116,645 (81,665–156,171)	675,300 (538,667–833,964)
High-middle SDI	YLDs Number: 2015	26,454,141 (20,114,959–33,580,320)	4,564,413 (3,266,865–6,105,815)	3,547,547 (2,396,323–5,010,334)	1,445,951 (953,681–1,974,481)	717,114 (515,616–954,893)	402,587 (287,251–534,902)	684,409 (451,277–954,707)	257,535 (175,050–347,089)	444,219 (316,209–581,731)	53,564 (36,852–74,794)	489,408 (384,582–615,593)
High-middle SDI	YLDs Number: Cumulative change (%)	-16.3 (-17.1 to -15.4)	-12.9 (-14.6 to -11.3)	-8.0 (-8.8 to -7.3)	-20.8 (-24.4 to -17.2)	-12.0 (-13.6 to -10.3)	-39.1 (-41.9 to -36.7)	32.1 (16.0–49.7)	-39.4 (-47.5 to -31.1)	-42.9 (-47.9 to -37.8)	-54.2 (-58.7 to -49.0)	-27.6 (-30.4 to -24.2)
High-middle SDI	YLDs Rate: 1990	6,137.6 (4,667.4–7,789.2)	1,024.1 (734.0–1,374.5)	747.7 (505.7–1,055.4)	356.9 (238.4–492.8)	159.7 (115.1–212.9)	126.3 (91.2–166.4)	100.5 (69.0–140.2)	80.6 (55.5–111.1)	150.9 (108.3–192.9)	22.9 (16.0–30.6)	131.0 (104.5–161.7)
High-middle SDI	YLDs Rate: 2015	5,549.0 (4,216.1–7,043.1)	981.0 (702.5–1,311.4)	744.6 (504.8–1,048.8)	306.4 (202.1–417.7)	153.6 (110.4–204.5)	87.6 (62.6–116.5)	144.9 (95.6–202.2)	56.7 (38.5–76.4)	94.0 (66.9–123.1)	11.5 (7.9–16.1)	103.7 (81.6–130.5)
High-middle SDI	YLDs Rate: Cumulative change (%)	-9.6 (-10.4 to -8.7)	-4.2 (-6.0 to -2.4)	-0.4 (-1.2 to 0.4)	-14.2 (-18.1 to -10.3)	-3.8 (-5.6 to -2.0)	-30.6 (-33.8 to -27.8)	44.3 (26.7–63.6)	-29.4 (-38.8 to -19.7)	-37.7 (-43.2 to -32.0)	-49.8 (-54.8 to -44.0)	-20.9 (-24.0 to -17.2)
Middle SDI	Prevalence Number: 1990	675,706,756 (674,830,509–676,599,285)	209,293,175 (207,065,998–211,570,855)	247,014,289 (244,816,225–249,206,679)	48,873,807 (41,465,772–56,720,447)	166,521,054 (165,085,936–167,917,638)	7,915,772 (7,365,144–8,535,710)	7,960,921 (7,518,667–8,439,529)	7,403,041 (6,897,620–7,982,894)	2,300,909 (2,064,202–2,552,511)	16,451,831 (13,213,159–19,860,141)	6,314,971 (5,668,524–7,041,727)
Middle SDI	Prevalence Number: 2015	620,437,513 (619,244,561–621,763,365)	183,177,818 (180,870,438–185,300,627)	235,523,024 (233,158,854–237,845,693)	40,051,924 (33,810,050–46,658,480)	147,366,794 (146,063,580–148,582,589)	5,365,803 (5,054,016–5,678,547)	9,523,656 (9,086,405–9,956,874)	5,312,581 (4,883,572–5,786,041)	2,279,318 (2,014,380–2,596,347)	8,147,684 (6,135,250–10,600,510)	6,206,477 (5,593,624–6,870,250)
Middle SDI	Prevalence Number: Cumulative change (%)	-8.2 (-8.4 to -8.0)	-12.5 (-14.1 to -10.9)	-4.7 (-5.6 to -3.7)	-18.0 (-20.9 to -15.1)	-11.5 (-12.3 to -10.7)	-32.2 (-34.7 to -29.8)	19.8 (12.9–26.6)	-28.1 (-35.9 to -19.8)	-1.0 (-7.5 to 5.8)	-50.7 (-56.5 to -42.4)	-1.7 (-6.5 to 3.5)
Middle SDI	Prevalence Rate: 1990	94,221.0 (94,100.3–94,341.8)	29,062.4 (28,769.6–29,364.2)	34,548.6 (34,239.0–34,858.6)	6,925.9 (5,885.1–8,028.4)	23,198.3 (22,999.0–23,392.6)	1,062.3 (990.0–1,143.6)	1,106.8 (1,045.2–1,173.2)	967.3 (901.2–1,043.0)	320.4 (287.1–355.8)	2,323.8 (1,860.5–2,807.1)	855.2 (766.8–955.2)
Middle SDI	Prevalence Rate: 2015	91,244.6 (91,068.3–91,439.6)	26,994.5 (26,658.4–27,303.5)	34,525.8 (34,179.3–34,868.1)	5,918.1 (4,995.2–6,897.7)	21,679.5 (21,487.8–21,858.1)	789.8 (743.8–835.8)	1,401.3 (1,336.9–1,465.0)	780.4 (717.4–849.9)	335.4 (296.5–382.2)	1,203.7 (905.0–1,566.8)	912.3 (822.2–1,009.8)
Middle SDI	Prevalence Rate: Cumulative change (%)	-3.2 (-3.4 to -3.0)	-7.1 (-8.8 to -5.5)	-0.1 (-1.1 to 0.9)	-14.5 (-17.5 to -11.5)	-6.5 (-7.4 to -5.7)	-25.6 (-28.2 to -23.1)	26.8 (19.4–34.0)	-19.2 (-28.0 to -9.9)	4.7 (-2.2 to 11.8)	-48.4 (-54.6 to -39.3)	6.7 (1.4–12.3)
Middle SDI	YLDs Number: 1990	44,377,200 (33,657,578–56,527,174)	8,315,737 (6,013,354–11,071,102)	5,186,179 (3,497,389–7,320,282)	2,156,165 (1,437,676–2,973,512)	1,489,333 (1,073,186–1,991,991)	1,253,952 (911,465–1,653,276)	578,362 (382,224–826,617)	891,684 (619,063–1,199,003)	875,469 (643,680–1,121,341)	386,384 (276,625–519,254)	783,265 (615,275–972,596)
Middle SDI	YLDs Number: 2015	38,514,608 (29,300,345–48,823,187)	7,075,700 (5,079,928–9,475,448)	4,977,816 (3,348,434–7,027,728)	1,785,676 (1,185,044–2,442,201)	1,328,362 (957,100–1,775,115)	856,900 (615,565–1,127,523)	847,432 (559,857–1,199,258)	645,602 (442,840–873,323)	618,917 (442,332–812,783)	193,942 (137,922–264,070)	765,470 (604,526–952,548)
Middle SDI	YLDs Number: Cumulative change (%)	-13.2 (-14.7 to -11.7)	-14.9 (-17.2 to -12.9)	-4.0 (-5.0 to -3.1)	-17.2 (-20.0 to -14.1)	-10.8 (-12.5 to -9.1)	-31.6 (-34.2 to -29.2)	47.3 (24.5–72.6)	-27.4 (-35.2 to -19.0)	-29.3 (-35.2 to -22.9)	-49.8 (-52.9 to -46.8)	-2.2 (-6.9 to 2.8)
Middle SDI	YLDs Rate: 1990	6,198.1 (4,702.8–7,902.0)	1,161.4 (839.9–1,546.0)	724.5 (487.7–1,022.9)	305.5 (203.6–420.9)	210.0 (151.4–280.4)	168.3 (122.3–221.6)	80.4 (53.1–114.9)	116.5 (80.9–156.7)	121.9 (89.5–156.0)	54.8 (39.2–73.6)	108.8 (85.6–135.1)
Middle SDI	YLDs Rate: 2015	5,655.6 (4,300.2–7,171.1)	1,047.0 (751.6–1,401.8)	731.0 (492.0–1,032.2)	263.9 (175.2–361.4)	197.0 (142.0–263.1)	126.1 (90.6–165.9)	124.7 (82.4–176.4)	94.8 (65.1–128.3)	91.1 (65.1–119.7)	28.8 (20.4–39.2)	112.7 (89.0–140.2)
Middle SDI	YLDs Rate: Cumulative change (%)	-8.7 (-10.3 to -7.2)	-9.9 (-12.2 to -7.8)	0.9 (-0.1 to 1.9)	-13.6 (-16.6 to -10.5)	-6.2 (-8.0 to -4.4)	-25.0 (-27.7 to -22.3)	55.9 (31.7–82.8)	-18.4 (-27.2 to -9.0)	-25.3 (-31.6 to -18.5)	-47.6 (-50.8 to -44.3)	3.7 (-1.3 to 9.1)
Low-middle SDI	Prevalence Number: 1990	539,990,173 (539,232,963–540,709,098)	216,022,195 (213,279,912–218,665,275)	177,695,844 (175,687,714–179,563,173)	41,737,891 (35,369,231–48,695,324)	186,185,938 (182,271,526–191,173,555)	10,041,879 (9,421,104–10,719,735)	5,109,896 (4,711,547–5,565,596)	11,837,870 (11,335,395–12,344,686)	2,368,950 (2,106,071–2,663,975)	77,432,363 (67,455,608–87,910,562)	3,641,404 (3,122,867–4,212,826)

© 2017 Global Burden of Disease Child and Adolescent Health Collaboration. JAMA Pediatrics.

eTable 3. Prevalent Cases, Rates (per 100,000 population), Years Lived with Disability (YLDs), and Cumulative Percent Change with 95% Uncertainty Interval (UI) for the Top 10 Global Causes of YLDs in Children and Adolescents in 195 Countries and Territories, Aged 0 to 19 Years, Both Sexes, 1990 and 2015. *Note: best viewed by enlarging in browser.*

Location	Measure	All causes	Iron-deficiency anemia	Skin and subcutaneous diseases	Asthma	Hemoglobinopathies and hemolytic anemias	Diarrheal diseases	Congenital anomalies	Protein-energy malnutrition	Epilepsy	Malaria	Neonatal preterm birth complications
Low-middle SDI	Prevalence Number: 2015	728,493,883 (727,319,700–729,708,303)	264,078,445 (260,554,223–267,447,598)	254,287,797 (251,482,578–257,142,174)	47,579,774 (39,760,786–56,341,242)	242,252,419 (237,934,530–248,283,768)	10,271,205 (9,765,080–10,857,560)	8,677,889 (8,083,042–9,298,604)	10,895,631 (10,243,778–11,627,217)	3,043,617 (2,668,827–3,479,354)	96,717,358 (81,040,969–114,410,967)	5,147,836 (4,483,808–5,880,467)
Low-middle SDI	Prevalence Number: Cumulative change (%)	34.9 (34.7–35.2)	22.3 (20.0–24.6)	43.1 (41.4–44.8)	14.0 (9.5–18.3)	30.1 (26.6–33.6)	2.3 (-0.4 to 4.9)	70.2 (55.2–84.2)	-7.9 (-14.5 to -0.3)	28.6 (17.8–39.8)	24.8 (14.4–36.1)	41.6 (32.9–50.1)
Low-middle SDI	Prevalence Rate: 1990	95,608.3 (95,477.9–95,727.8)	37,719.5 (37,264.4–38,142.4)	32,635.8 (32,273.5–32,977.2)	7,419.5 (6,311.5–8,622.1)	32,874.4 (32,180.4–33,759.2)	1,631.7 (1,535.7–1,736.9)	894.4 (824.2–975.1)	1,816.2 (1,739.1–1,893.9)	421.3 (375.2–472.8)	13,306.6 (11,630.6–15,084.7)	593.7 (508.3–686.3)
Low-middle SDI	Prevalence Rate: 2015	94,108.9 (93,955.5–94,263.5)	33,931.0 (33,489.2–34,350.7)	33,194.6 (32,831.9–33,559.1)	6,135.7 (5,126.4–7,251.0)	31,256.8 (30,697.0–32,037.3)	1,299.7 (1,235.5–1,373.4)	1,118.4 (1,041.9–1,198.5)	1,359.1 (1,277.8–1,450.3)	394.1 (345.5–450.5)	12,426.5 (10,432.8–14,682.7)	653.7 (569.9–747.1)
Low-middle SDI	Prevalence Rate: Cumulative change (%)	-1.6 (-1.7 to -1.4)	-10.0 (-11.6 to -8.4)	1.7 (0.5–2.9)	-17.3 (-20.5 to -14.4)	-4.9 (-7.5 to -2.4)	-20.3 (-22.3 to -18.5)	25.3 (14.2–35.7)	-25.1 (-30.5 to -18.9)	-6.4 (-14.2 to 1.8)	-6.7 (-14.2 to 1.6)	10.3 (3.3–17.0)
Low-middle SDI	YLDs Number: 1990	43,048,564 (32,542,108–55,140,152)	9,999,096 (7,284,182–13,178,690)	3,926,601 (2,698,805–5,452,660)	1,881,407 (1,255,377–2,598,623)	1,462,601 (1,066,039–1,920,347)	1,621,904 (1,177,629–2,137,616)	428,623 (277,683–642,894)	1,456,033 (1,002,805–1,947,949)	919,242 (653,587–1,186,097)	1,051,240 (754,142–1,403,397)	381,471 (292,347–486,070)
Low-middle SDI	YLDs Number: 2015	53,056,669 (40,095,727–67,467,519)	11,300,366 (8,123,723–14,975,090)	5,505,932 (3,765,166–7,691,347)	2,153,411 (1,422,326–2,965,053)	1,798,392 (1,303,588–2,388,630)	1,668,008 (1,206,740–2,189,947)	840,951 (554,138–1,209,660)	1,348,008 (926,071–1,796,843)	1,042,262 (759,118–1,345,656)	1,097,615 (792,347–1,449,358)	634,062 (492,274–796,334)
Low-middle SDI	YLDs Number: Cumulative change (%)	23.3 (20.8–25.5)	12.9 (9.2–16.8)	40.2 (38.6–41.7)	14.4 (9.9–18.9)	22.9 (18.8–26.8)	2.9 (-0.0 to 5.5)	98.3 (67.0–137.4)	-7.3 (-14.1 to -0.0)	13.6 (3.1–24.6)	4.5 (-1.1 to 9.6)	66.5 (55.7–77.7)
Low-middle SDI	YLDs Rate: 1990	7,686.1 (5,816.2–9,787.6)	1,721.2 (1,254.1–2,266.4)	708.0 (483.6–988.1)	334.3 (223.2–458.3)	252.9 (184.4–331.9)	263.5 (191.0–348.6)	75.0 (48.5–112.6)	223.4 (153.9–298.9)	163.4 (116.4–211.0)	180.1 (129.4–239.9)	65.2 (49.8–83.1)
Low-middle SDI	YLDs Rate: 2015	6,877.7 (5,200.0–8,739.6)	1,444.6 (1,037.8–1,914.1)	714.9 (487.0–998.0)	277.7 (183.7–381.7)	230.1 (166.7–305.5)	211.1 (152.6–277.2)	108.3 (71.4–155.8)	168.2 (115.5–224.1)	134.9 (98.1–174.3)	140.4 (101.4–185.4)	81.5 (63.3–102.3)
Low-middle SDI	YLDs Rate: Cumulative change (%)	-10.5 (-12.1 to -9.1)	-16.1 (-18.8 to -13.4)	1.0 (0.2–1.7)	-17.0 (-20.2 to -13.9)	-9.1 (-12.0 to -6.2)	-19.9 (-22.0 to -17.9)	46.1 (22.7–75.6)	-24.7 (-30.1 to -18.7)	-17.3 (-25.0 to -9.3)	-22.0 (-26.0 to -18.3)	25.3 (16.9–34.0)
Low SDI	Prevalence Number: 1990	142,295,759 (142,119,117–142,472,861)	50,359,075 (49,221,220–51,372,438)	52,042,094 (51,123,716–52,988,909)	11,525,591 (9,678,802–13,583,476)	47,897,273 (47,251,494–48,597,377)	3,843,047 (3,576,647–4,111,434)	1,345,716 (1,210,397–1,492,177)	2,622,342 (2,330,507–2,948,974)	320,898 (271,412–379,064)	36,934,833 (33,735,442–40,416,014)	816,113 (651,434–994,839)
Low SDI	Prevalence Number: 2015	287,519,634 (287,057,868–287,985,488)	86,753,142 (85,151,263–88,452,866)	108,638,223 (106,623,542–110,523,861)	22,328,931 (18,648,452–26,261,589)	89,910,958 (88,580,573–91,308,320)	5,059,868 (4,759,086–5,413,296)	3,262,823 (2,972,301–3,574,544)	4,053,749 (3,669,047–4,471,786)	692,913 (579,976–818,862)	78,540,101 (68,733,293–89,055,019)	1,678,626 (1,355,713–2,021,259)
Low SDI	Prevalence Number: Cumulative change (%)	102.1 (101.7–102.4)	72.3 (67.4–77.6)	108.8 (104.5–112.9)	93.8 (84.5–104.4)	87.7 (84.7–90.6)	31.7 (26.8–37.0)	143.1 (120.3–168.5)	55.4 (32.7–81.0)	116.7 (86.8–148.6)	112.4 (99.6–124.5)	106.3 (89.3–123.5)
Low SDI	Prevalence Rate: 1990	97,188.6 (97,081.8–97,297.3)	32,554.4 (31,922.6–33,115.5)	37,142.0 (36,459.2–37,782.2)	8,167.1 (6,886.4–9,608.5)	32,581.4 (32,140.1–33,057.7)	2,270.8 (2,126.4–2,418.8)	897.3 (806.4–997.0)	1,403.8 (1,247.1–1,577.8)	215.6 (182.6–253.6)	25,115.3 (22,974.3–27,489.7)	462.7 (368.9–567.5)
Low SDI	Prevalence Rate: 2015	95,950.0 (95,804.8–96,099.9)	27,922.7 (27,451.3–28,426.5)	37,242.9 (36,583.4–37,860.4)	7,575.1 (6,352.7–8,903.2)	29,897.1 (29,455.1–30,362.1)	1,573.8 (1,482.3–1,677.8)	1,077.5 (981.4–1,180.9)	1,173.8 (1,062.8–1,294.9)	230.0 (192.8–271.5)	26,023.8 (22,769.5–29,536.8)	511.0 (413.2–612.4)
Low SDI	Prevalence Rate: Cumulative change (%)	-1.3 (-1.4 to -1.1)	-14.2 (-16.4 to -11.9)	0.3 (-1.6 to 2.1)	-7.3 (-11.7 to -2.2)	-8.2 (-9.7 to -6.8)	-30.7 (-33.1 to -28.1)	20.4 (8.6–33.2)	-15.9 (-28.2 to -2.2)	7.0 (-7.9 to 22.6)	3.5 (-2.6 to 9.4)	10.8 (0.2–21.1)
Low SDI	YLDs Number: 1990	11,795,004 (8,864,597–15,159,223)	2,292,451 (1,655,504–3,038,297)	1,132,595 (782,183–1,569,009)	516,726 (340,870–713,792)	260,013 (188,840–345,573)	617,159 (445,848–818,010)	102,468 (65,905–153,075)	318,918 (219,902–435,335)	131,067 (93,603–172,574)	550,540 (401,173–719,511)	38,729 (28,990–49,905)
Low SDI	YLDs Number: 2015	21,751,788 (16,308,572–28,049,376)	3,706,095 (2,690,650–4,943,923)	2,352,608 (1,618,408–3,270,352)	1,005,581 (668,260–1,378,377)	497,047 (361,135–663,347)	816,972 (592,221–1,075,469)	269,420 (179,406–385,822)	497,910 (340,072–667,395)	263,420 (185,702–348,914)	1,125,089 (816,393–1,479,059)	96,395 (71,338–124,338)
Low SDI	YLDs Number: Cumulative change (%)	84.3 (78.0–93.2)	61.7 (53.4–69.9)	107.6 (106.0–109.3)	94.6 (84.9–105.5)	91.1 (84.3–98.3)	32.4 (27.4–37.9)	165.8 (138.1–199.0)	56.9 (34.2–82.4)	101.5 (73.5–132.4)	104.4 (97.3–111.6)	149.2 (131.0–166.6)
Low SDI	YLDs Rate: 1990	8,150.8 (6,143.4–10,498.0)	1,446.8 (1,043.4–1,924.2)	791.0 (543.3–1,098.3)	366.0 (242.6–501.5)	170.4 (123.9–225.8)	364.5 (264.2–483.5)	68.0 (43.2–102.1)	170.7 (117.7–232.9)	88.0 (62.4–115.6)	368.3 (267.9–481.7)	23.4 (17.4–30.0)
Low SDI	YLDs Rate: 2015	7,340.5 (5,507.8–9,481.7)	1,178.5 (856.2–1,570.1)	795.0 (546.3–1,106.4)	341.1 (227.0–465.5)	161.5 (117.2–215.3)	254.0 (184.2–332.8)	88.7 (59.0–127.4)	144.2 (98.4–193.3)	87.4 (62.2–115.6)	368.0 (267.5–483.0)	30.7 (22.7–39.5)
Low SDI	YLDs Rate: Cumulative change (%)	-10.0 (-13.0 to -5.8)	-18.5 (-22.3 to -14.9)	0.5 (-0.1 to 1.1)	-6.8 (-11.4 to -1.7)	-5.3 (-8.3 to -2.0)	-30.3 (-32.7 to -27.6)	32.0 (17.7–49.3)	-15.2 (-27.4 to -1.4)	-0.4 (-14.2 to 14.7)	-0.1 (-3.2 to 3.3)	31.4 (21.4–41.0)
High-income	Prevalence Number: 1990	206,681,065 (206,141,554–207,251,624)	54,011,157 (53,348,211–54,681,222)	71,558,839 (71,022,978–72,040,960)	23,066,908 (20,151,991–26,003,403)	34,295,918 (33,881,974–34,682,213)	84,005 (79,952–88,771)	3,937,417 (3,830,616–4,038,711)	2,442 (1,647–3,454)	789,542 (700,190–892,305)	611 (316–962)	2,583,897 (2,418,867–2,766,026)
High-income	Prevalence Number: 2015	197,089,075 (196,568,830–197,664,133)	54,753,398 (54,122,516–55,412,506)	68,540,332 (68,094,405–69,019,755)	16,729,594 (14,926,814–18,696,753)	29,386,376 (28,975,077–29,794,694)	66,181 (62,654–69,945)	4,082,173 (3,963,288–4,195,198)	452 (309–651)	763,614 (687,054–849,555)	170 (114–266)	2,326,020 (2,159,784–2,501,907)
High-income	Prevalence Number: Cumulative change (%)	-4.6 (-4.9 to -4.3)	1.4 (-0.4 to 3.1)	-4.2 (-4.9 to -3.5)	-27.4 (-30.5 to -24.1)	-14.3 (-15.6 to -13.1)	-21.2 (-23.3 to -19.4)	3.7 (0.8–6.6)	-81.4 (-84.6 to -77.8)	-3.2 (-8.1 to 1.9)	-69.7 (-84.0 to -46.4)	-10.0 (-12.8 to -7.1)
High-income	Prevalence Rate: 1990	81,638.3 (81,415.2–81,873.7)	21,892.6 (21,605.7–22,176.7)	27,579.1 (27,381.5–27,762.5)	9,076.8 (7,903.0–10,292.8)	13,656.3 (13,492.7–13,809.7)	35.5 (33.8–37.6)	1,566.8 (1,524.4–1,606.9)	1.1 (0.7–1.5)	313.3 (277.9–354.4)	0.2 (0.1–0.4)	1,063.4 (997.0–1,136.3)
High-income	Prevalence Rate: 2015	81,132.8 (80,908.5–81,384.5)	23,015.3 (22,735.2–23,309.5)	27,695.4 (27,519.7–27,883.4)	6,879.9 (6,138.8–7,692.3)	12,170.4 (11,999.9–12,338.7)	28.8 (27.3–30.5)	1,690.3 (1,641.3–1,737.1)	0.2 (0.1–0.3)	315.0 (283.0–350.2)	0.1 (0.0–0.1)	995.0 (925.9–1,068.0)
High-income	Prevalence Rate: Cumulative change (%)	-0.6 (-0.9 to -0.3)	5.1 (3.2–7.0)	0.4 (-0.3 to 1.2)	-24.1 (-27.5 to -20.6)	-10.9 (-12.2 to -9.7)	-18.8 (-21.0 to -16.9)	7.9 (4.9–10.9)	-81.1 (-84.4 to -77.5)	0.7 (-4.6 to 5.9)	-68.3 (-83.1 to -44.0)	-6.4 (-9.3 to -3.5)
High-income	YLDs Number: 1990	13,158,504 (10,005,772–16,705,877)	1,944,199 (1,382,644–2,627,610)	1,709,999 (1,201,243–2,338,486)	1,050,907 (711,282–1,418,043)	368,392 (267,351–489,307)	13,883 (10,035–18,315)	346,707 (235,129–476,957)	297 (171–465)	219,245 (157,506–280,534)	14 (7–25)	266,357 (208,932–333,370)
High-income	YLDs Number: 2015	12,337,199 (9,403,116–15,654,231)	2,017,226 (1,438,503–2,722,378)	1,643,318 (1,152,875–2,249,303)	762,830 (517,840–1,025,262)	293,317 (211,563–391,475)	10,946 (7,811–14,579)	469,202 (300,677–670,922)	55 (32–87)	128,266 (88,617–176,429)	5 (2–9)	206,493 (161,116–261,802)
High-income	YLDs Number: Cumulative change (%)	-6.2 (-7.3 to -5.1)	3.8 (1.5–6.1)	-3.9 (-4.5 to -3.4)	-27.3 (-30.4 to -24.0)	-20.4 (-22.0 to -18.7)	-21.1 (-24.5 to -17.8)	34.9 (20.3–58.1)	-81.0 (-85.1 to -75.9)	-41.6 (-50.5 to -31.8)	-60.9 (-78.8 to -32.3)	-22.5 (-25.3 to -19.9)
High-income	YLDs Rate: 1990	5,102.6 (3,879.6–6,489.0)	792.8 (563.9–1,072.2)	670.9 (472.5–917.1)	413.7 (280.7–559.3)	147.7 (107.3–196.1)	5.9 (4.2–7.7)	138.1 (93.6–190.0)	0.1 (0.1–0.2)	87.1 (62.7–111.0)	0.0 (0.0–0.0)	105.9 (83.2–132.6)
High-income	YLDs Rate: 2015	5,004.0 (3,814.9–6,350.3)	850.4 (606.3–1,147.1)	672.8 (473.4–921.1)	313.8 (212.7–422.3)	122.3 (88.2–163.1)	4.8 (3.4–6.3)	194.4 (124.6–278.0)	0.0 (0.0–0.0)	52.9 (36.5–72.3)	0.0 (0.0–0.0)	85.3 (66.6–108.1)
High-income	YLDs Rate: Cumulative change (%)	-1.9 (-3.0 to -0.8)	7.3 (4.7–9.8)	0.3 (-0.4 to 0.8)	-24.0 (-27.4 to -20.5)	-17.3 (-18.9 to -15.4)	-18.8 (-22.2 to -15.3)	40.3 (25.2–64.4)	-80.8 (-85.0 to -75.6)	-39.3 (-48.5 to -29.3)	-59.3 (-77.7 to -30.4)	-19.5 (-22.3 to -16.7)
High-income North America	Prevalence Number: 1990	64,422,677 (64,204,898–64,639,963)	16,885,441 (16,557,660–17,187,656)	20,501,562 (20,360,543–20,639,427)	6,179,427 (5,257,482–7,134,646)	16,577,513 (16,399,180–16,755,528)	3,976 (3,754–4,207)	1,406,975 (1,352,186–1,456,243)	84 (57–119)	258,541 (226,512–296,164)	0 (0–0)	1,142,229 (1,073,019–1,215,809)
High-income North America	Prevalence Number: 2015	73,330,440 (73,100,348–73,573,728)	21,068,515 (20,772,423–21,426,954)	24,019,562 (23,888,645–24,150,930)	5,707,411 (5,504,016–5,906,373)	14,931,029 (14,761,905–15,095,967)	4,563 (4,269–4,879)	1,711,199 (1,644,222–1,777,597)	57 (39–82)	307,601 (288,185–325,119)	0 (0–0)	1,139,482 (1,055,149–1,229,010)
High-income North America	Prevalence Number: Cumulative change (%)	13.8 (13.4–14.3)	24.8 (21.9–27.9)	17.2 (16.3–18.0)	-6.9 (-18.7 to 6.3)	-9.9 (-11.0 to -8.9)	14.8 (11.5–18.2)	21.7 (16.9–26.7)	-31.5 (-40.6 to -22.2)	19.6 (6.5–32.9)	11.9 (10.3–13.2)	-0.2 (-4.9 to 4.8)
High-income North America	Prevalence Rate: 1990	80,591.3 (80,315.8–80,865.5)	21,214.7 (20,804.9–21,590.3)	25,541.8 (25,360.9–25,714.5)	7,770.4 (6,606.6–8,989.3)	20,748.7 (20,525.9–20,971.1)	5.0 (4.7–5.3)	1,760.8 (1,692.2–1,822.7)	0.1 (0.1–0.1)	323.5 (283.3–370.7)	0.0 (0.0–0.0)	1,424.1 (1,337.5–1,516.1)
High-income North America	Prevalence Rate: 2015	79,912.1 (79,644.2–80,189.7)	23,474.8 (23,133.9–23,889.7)	25,692.2 (25,553.2–25,830.3)	6,277.8 (6,052.7–6,497.4)	16,372.9 (16,187.8–16,553.6)	5.3 (4.9–5.6)	1,876.6 (1,803.4–1,949.1)	0.1 (0.0–0.1)	335.3 (314.0–354.4)	0.0 (0.0–0.0)	1,294.7 (1,199.1–1,394.7)
High-income North America	Prevalence Rate: Cumulative change (%)	-0.8 (-1.2 to -0.4)	10.7 (8.0–13.5)	0.6 (-0.2 to 1.3)	-18.6 (-29.0 to -6.9)	-21.1 (-22.0 to -20.2)	6.1 (3.0–9.5)	6.6 (2.5–11.0)	-35.9 (-44.6 to -27.0)	4.2 (-7.1 to 15.9)	0.3 (0.1–0.5)	-9.1 (-13.3 to -4.6)
High-income North America	YLDs Number: 1990	4,095,640 (3,118,651–5,172,386)	586,561 (420,643–792,597)	553,779 (393,592–750,663)	281,767 (187,254–384,617)	166,690 (121,472–220,514)	671 (484–899)	138,065 (93,281–191,056)	10 (6–16)	72,353 (51,592–92,459)	0 (0–0)	114,214 (90,036–142,780)
High-income North America	YLDs Number: 2015	4,837,450 (3,713,052–6,084,805)	768,122 (550,442–1,032,027)	638,731 (454,525–865,940)	260,458 (178,931–347,761)	129,016 (92,889–172,868)	768 (549–1,025)	215,027 (137,023–310,468)	7 (4–11)	50,603 (34,468–70,636)	0 (0–0)	100,598 (78,205–128,366)
High-income North America	YLDs Number: Cumulative change (%)	18.1 (16.2–20.4)	31.0 (27.0–34.9)	15.3 (14.4–16.3)	-6.9 (-18.7 to 6.4)	-22.6 (-24.4 to -20.7)	14.5 (7.1–22.4)	55.6 (37.7–90.0)	-31.4 (-40.6 to -22.1)	-30.0 (-43.7 to -15.7)	13.3 (9.5–16.6)	-12.0 (-15.8 to -8.0)
High-income North America	YLDs Rate: 1990	5,109.9 (3,890.4–6,459.2)	740.6 (531.1–1,000.9)	692.8 (492.5–938.2)	354.3 (235.5–484.3)	210.8 (153.6–278.9)	0.8 (0.6–1.1)	172.8 (116.7–239.1)	0.0 (0.0–0.0)	90.5 (64.6–115.7)	0.0 (0.0–0.0)	143.0 (112.8–178.8)
High-income North America	YLDs Rate: 2015	5,181.8 (3,977.3–6,514.9)	857.7 (614.6–1,151.5)	694.2 (494.9–937.9)	286.6 (196.8–382.5)	142.2 (102.5–190.7)	0.9 (0.6–1.2)	235.9 (150.4–340.6)	0.0 (0.0–0.0)	55.2 (37.7–77.1)	0.0 (0.0–0.0)	110.1 (85.6–140.2)
High-income North America	YLDs Rate: Cumulative change (%)	1.4 (-0.3 to 3.3)	15.9 (12.4–19.3)	0.2 (-0.5 to 1.0)	-18.5 (-29.0 to -6.7)	-32.6 (-34.1 to -30.8)	5.9 (-1.3 to 13.4)	36.4 (20.7–66.6)	-35.9 (-44.6 to -27.0)	-39.0 (-50.9 to -26.5)	0.6 (-2.3 to 3.6)	-23.1 (-26.4 to -19.7)
Canada	Prevalence Number: 1990	6,221,067 (6,130,909–6,311,109)	1,943,496 (1,790,611–2,079,626)	2,090,265 (2,035,140–2,146,773)	640,722 (533,656–755,408)	1,205,508 (1,147,157–1,271,719)	580 (551–611)	135,497 (119,695–154,757)	6 (4–9)	9,651 (7,565–11,913)	0 (0–0)	86,289 (76,738–99,973)
Canada	Prevalence Number: 2015	6,281,455 (6,190,711–6,360,208)	1,948,339 (1,815,962–2,078,434)	2,218,477 (2,157,285–2,280,804)	559,526 (460,192–660,211)	896,437 (855,135–941,528)	661 (601–727)	133,341 (117,624–155,245)	3 (2–5)	10,419 (7,953–13,095)	0 (0–0)	84,901 (75,097–98,754)
Canada	Prevalence Number: Cumulative change (%)	1.0 (-0.9 to 2.8)	0.5 (-8.7 to 11.9)	6.2 (2.2–9.8)	-12.5 (-23.3 to -1.6)	-25.6 (-30.4 to -20.6)	13.9 (6.3–22.1)	-1.1 (-17.3 to 18.1)	-39.0 (-55.9 to -18.6)	9.3 (-19.6 to 39.0)	-1.2 (-2.3 to -0.1)	-1.0 (-17.8 to 18.7)

© 2017 Global Burden of Disease Child and Adolescent Health Collaboration. JAMA Pediatrics.

eTable 3. Prevalent Cases, Rates (per 100,000 population), Years Lived with Disability (YLDs), and Cumulative Percent Change with 95% Uncertainty Interval (UI) for the Top 10 Global Causes of YLDs in Children and Adolescents in 195 Countries and Territories, Aged 0 to 19 Years, Both Sexes, 1990 and 2015. *Note: best viewed by enlarging in browser.*

Location	Measure	All causes	Iron-deficiency anemia	Skin and subcutaneous diseases	Asthma	Hemoglobinopathies and hemolytic anemias	Diarrheal diseases	Congenital anomalies	Protein-energy malnutrition	Epilepsy	Malaria	Neonatal preterm birth complications
Canada	Prevalence Rate: 1990	81,322.6 (80,110.1–82,529.6)	25,574.7 (23,459.6–27,449.5)	27,078.5 (26,366.2–27,811.5)	8,368.6 (6,967.5–9,873.4)	15,814.3 (15,050.4–16,678.3)	7.9 (7.5–8.3)	1,779.4 (1,571.7–2,031.8)	0.1 (0.1–0.1)	126.0 (98.6–155.5)	0.0 (0.0–0.0)	1,158.1 (1,032.2–1,335.6)
Canada	Prevalence Rate: 2015	80,193.7 (78,944.0–81,258.2)	25,576.7 (23,862.8–27,358.1)	27,511.1 (26,760.5–28,238.9)	7,343.7 (6,015.5–8,727.5)	11,504.6 (10,978.2–12,089.8)	8.9 (8.1–9.8)	1,716.8 (1,511.1–2,002.0)	0.1 (0.0–0.1)	132.5 (101.2–166.0)	0.0 (0.0–0.0)	1,123.9 (996.3–1,302.2)
Canada	Prevalence Rate: Cumulative change (%)	-1.4 (-3.3 to 0.5)	0.2 (-9.1 to 12.0)	1.6 (-1.9 to 5.1)	-12.1 (-23.0 to -1.0)	-27.2 (-32.0 to -22.3)	13.1 (5.5–21.6)	-3.0 (-19.0 to 15.6)	-39.6 (-56.4 to -18.6)	6.4 (-22.0 to 35.9)	0.0 (-0.0 to 0.0)	-2.4 (-18.6 to 16.3)
Canada	YLDs Number: 1990	398,392 (302,322–506,569)	71,010 (49,808–96,583)	65,983 (47,069–88,685)	29,176 (19,274–39,969)	6,614 (4,719–8,970)	97 (67–135)	13,987 (9,510–19,082)	0 (0–1)	2,651 (1,727–3,708)	0 (0–0)	7,741 (5,945–9,879)
Canada	YLDs Number: 2015	407,835 (313,007–515,415)	69,976 (49,523–95,501)	67,878 (48,550–90,753)	25,479 (16,770–35,017)	6,731 (4,760–9,012)	110 (74–153)	15,166 (10,009–21,530)	0 (0–0)	1,697 (1,028–2,565)	0 (0–0)	7,226 (5,546–9,231)
Canada	YLDs Number: Cumulative change (%)	2.4 (-0.3 to 5.4)	-1.2 (-11.7 to 10.7)	2.9 (0.7–4.9)	-12.5 (-23.6 to -1.6)	1.9 (-7.9 to 12.2)	14.1 (-13.4 to 45.5)	8.6 (-9.9 to 33.5)	-39.0 (-55.9 to -18.8)	-34.9 (-57.3 to -8.1)	-3.7 (-16.1 to 10.9)	-6.5 (-14.5 to 2.1)
Canada	YLDs Rate: 1990	5,152.5 (3,906.4–6,541.3)	936.4 (656.7–1,273.8)	866.4 (617.2–1,165.4)	381.1 (251.0–523.9)	86.5 (61.7–117.3)	1.3 (0.9–1.8)	183.8 (124.9–250.4)	0.0 (0.0–0.0)	34.6 (22.6–48.5)	0.0 (0.0–0.0)	101.6 (78.1–129.6)
Canada	YLDs Rate: 2015	5,089.5 (3,902.9–6,454.1)	946.8 (672.9–1,298.9)	873.6 (626.0–1,163.7)	334.5 (220.2–462.2)	88.6 (63.1–118.5)	1.5 (1.0–2.1)	195.2 (129.1–276.3)	0.0 (0.0–0.0)	21.6 (13.1–32.6)	0.0 (0.0–0.0)	92.9 (71.5–118.8)
Canada	YLDs Rate: Cumulative change (%)	-1.2 (-4.0 to 1.8)	1.4 (-9.2 to 13.1)	0.8 (-1.1 to 2.8)	-12.1 (-23.2 to -0.6)	2.6 (-7.1 to 12.5)	13.4 (-14.6 to 45.5)	6.4 (-11.7 to 30.6)	-39.6 (-56.4 to -18.6)	-36.6 (-58.5 to -10.6)	-0.1 (-12.8 to 15.4)	-8.4 (-16.2 to 0.2)
Greenland	Prevalence Number: 1990	16,704 (16,355–17,016)	5,291 (4,765–5,843)	4,998 (4,820–5,193)	1,232 (982–1,476)	3,501 (3,333–3,661)	2 (2–2)	346 (300–405)	0 (0–0)	56 (44–70)	0 (0–0)	450 (404–503)
Greenland	Prevalence Number: 2015	14,122 (13,852–14,349)	4,325 (3,972–4,601)	4,579 (4,418–4,754)	1,011 (821–1,225)	2,250 (2,150–2,356)	1 (1–1)	300 (263–348)	0 (0–0)	39 (30–49)	0 (0–0)	319 (285–364)
Greenland	Prevalence Number: Cumulative change (%)	-15.4 (-17.6 to -13.3)	-17.9 (-28.2 to -4.3)	-8.3 (-12.5 to -3.7)	-17.8 (-22.8 to -12.4)	-35.7 (-39.9 to -31.6)	-24.9 (-28.0 to -21.5)	-12.7 (-27.9 to 5.5)	-57.6 (-80.2 to -31.8)	-28.9 (-48.7 to -6.4)	-19.7 (-21.9 to -17.4)	-29.0 (-37.8 to -19.0)
Greenland	Prevalence Rate: 1990	80,245.5 (79,033.3–81,415.8)	23,485.5 (21,741.6–25,128.2)	26,026.9 (25,149.4–26,909.2)	6,481.8 (5,240.1–7,686.3)	15,969.1 (15,194.2–16,698.8)	6.6 (6.3–7.0)	1,566.7 (1,354.2–1,839.3)	0.0 (0.0–0.1)	271.5 (212.1–338.3)	0.0 (0.0–0.0)	1,552.9 (1,363.2–1,797.9)
Greenland	Prevalence Rate: 2015	79,227.7 (77,991.0–80,367.1)	23,683.6 (22,269.6–25,004.4)	26,613.0 (25,770.7–27,578.1)	6,046.1 (4,896.8–7,244.2)	12,154.4 (11,622.1–12,722.5)	6.6 (6.2–7.1)	1,615.2 (1,412.7–1,869.2)	0.0 (0.0–0.0)	223.6 (169.9–280.2)	0.0 (0.0–0.0)	1,427.1 (1,252.4–1,671.6)
Greenland	Prevalence Rate: Cumulative change (%)	-1.3 (-3.2 to 0.8)	1.1 (-8.9 to 13.3)	2.3 (-2.0 to 7.2)	-6.7 (-12.5 to -0.5)	-23.8 (-28.7 to -19.0)	0.3 (-3.5 to 4.2)	3.9 (-14.7 to 25.9)	-36.7 (-67.8 to -8.4)	-16.4 (-39.4 to 9.6)	0.0 (-0.0 to 0.0)	-7.5 (-22.5 to 8.6)
Greenland	YLDs Number: 1990	964 (735–1,223)	177 (123–245)	172 (121–230)	56 (36–78)	20 (14–27)	0 (0–0)	36 (24–52)	0 (0–0)	16 (10–23)	0 (0–0)	41 (31–51)
Greenland	YLDs Number: 2015	843 (649–1,068)	149 (104–202)	149 (106–201)	46 (29–63)	14 (10–19)	0 (0–0)	37 (23–55)	0 (0–0)	6 (4–10)	0 (0–0)	26 (20–33)
Greenland	YLDs Number: Cumulative change (%)	-12.5 (-15.4 to -9.2)	-15.7 (-26.6 to -2.0)	-13.2 (-15.3 to -11.1)	-17.9 (-23.6 to -11.8)	-28.6 (-36.2 to -19.1)	-23.5 (-41.2 to -1.9)	4.3 (-18.6 to 29.9)	-57.5 (-80.1 to -31.9)	-58.4 (-73.0 to -41.6)	-17.2 (-30.6 to -4.5)	-35.3 (-40.4 to -30.0)
Greenland	YLDs Rate: 1990	5,211.4 (3,984.1–6,537.0)	856.2 (606.1–1,158.7)	816.8 (576.7–1,097.4)	295.6 (192.6–406.3)	109.2 (78.0–147.3)	1.1 (0.8–1.5)	164.1 (107.5–234.2)	0.0 (0.0–0.0)	78.3 (50.6–110.5)	0.0 (0.0–0.0)	188.0 (146.5–235.8)
Greenland	YLDs Rate: 2015	5,085.3 (3,888.7–6,434.3)	874.8 (620.6–1,177.9)	826.0 (583.1–1,111.2)	275.6 (178.8–378.6)	87.3 (62.0–117.1)	1.1 (0.8–1.6)	202.8 (125.1–295.5)	0.0 (0.0–0.0)	37.7 (22.8–56.3)	0.0 (0.0–0.0)	144.8 (112.3–183.8)
Greenland	YLDs Rate: Cumulative change (%)	-2.4 (-4.7 to 0.0)	2.4 (-6.4 to 13.0)	1.1 (-0.7 to 3.0)	-6.7 (-12.8 to 0.4)	-19.9 (-26.8 to -12.6)	1.6 (-18.7 to 26.5)	24.4 (-3.2 to 54.7)	-36.5 (-67.7 to -8.4)	-50.9 (-68.1 to -31.2)	1.7 (-8.5 to 12.1)	-22.9 (-29.0 to -16.3)
United States	Prevalence Number: 1990	58,155,101 (57,961,959–58,346,304)	14,928,726 (14,621,435–15,204,336)	18,396,945 (18,279,530–18,515,798)	5,534,624 (4,711,345–6,401,928)	15,360,783 (15,199,505–15,522,349)	3,391 (3,197–3,596)	1,270,478 (1,222,226–1,317,529)	77 (53–110)	248,713 (217,319–285,381)	0 (0–0)	1,054,950 (991,840–1,122,644)
United States	Prevalence Number: 2015	67,009,876 (66,796,709–67,240,621)	19,108,714 (18,825,056–19,433,548)	21,788,267 (21,679,059–21,899,786)	5,144,927 (5,011,101–5,272,439)	14,027,257 (13,877,404–14,170,662)	3,899 (3,658–4,158)	1,576,975 (1,517,858–1,639,785)	53 (36–76)	297,038 (278,938–313,797)	0 (0–0)	1,053,883 (974,455–1,141,647)
United States	Prevalence Number: Cumulative change (%)	15.2 (14.8–15.7)	28.0 (24.9–31.3)	18.4 (17.6–19.3)	-6.2 (-19.1 to 8.7)	-8.7 (-9.7 to -7.7)	15.0 (12.1–18.1)	24.2 (19.2–29.4)	-30.8 (-40.5 to -20.8)	20.0 (6.7–33.9)	12.9 (11.2–14.3)	-0.1 (-5.2 to 5.1)
United States	Prevalence Rate: 1990	80,507.8 (80,238.9–80,777.4)	20,727.9 (20,309.9–21,105.5)	25,386.5 (25,226.0–25,551.6)	7,708.9 (6,556.7–8,916.8)	21,268.8 (21,045.7–21,492.9)	4.7 (4.4–4.9)	1,758.9 (1,692.1–1,824.1)	0.1 (0.1–0.2)	344.3 (300.8–395.7)	0.0 (0.0–0.0)	1,451.7 (1,364.4–1,545.4)
United States	Prevalence Rate: 2015	79,894.9 (79,634.7–80,182.2)	23,279.2 (22,912.7–23,693.8)	25,528.2 (25,400.5–25,654.7)	6,185.8 (6,021.6–6,337.9)	16,824.6 (16,644.6–16,996.9)	4.9 (4.6–5.3)	1,891.6 (1,820.9–1,966.5)	0.1 (0.0–0.1)	354.1 (332.5–374.5)	0.0 (0.0–0.0)	1,310.6 (1,213.1–1,417.2)
United States	Prevalence Rate: Cumulative change (%)	-0.8 (-1.2 to -0.4)	12.3 (9.5–15.1)	0.6 (-0.1 to 1.3)	-19.1 (-30.3 to -6.1)	-20.9 (-21.8 to -20.0)	5.6 (2.8–8.5)	7.6 (3.3–12.1)	-35.6 (-44.7 to -25.9)	3.4 (-8.2 to 15.3)	0.0 (-0.0 to 0.0)	-9.7 (-14.3 to -5.0)
United States	YLDs Number: 1990	3,694,413 (2,819,093–4,659,089)	515,097 (368,525–695,959)	487,367 (346,234–660,780)	252,405 (168,148–344,289)	159,979 (116,662–211,600)	572 (410–765)	123,976 (83,344–172,330)	9 (5–14)	69,651 (49,746–89,240)	0 (0–0)	106,378 (84,261–132,909)
United States	YLDs Number: 2015	4,427,113 (3,394,367–5,565,828)	697,734 (499,334–937,229)	570,484 (405,316–775,171)	234,844 (161,879–312,451)	122,226 (88,068–164,072)	657 (470–873)	199,750 (127,105–289,134)	6 (3–10)	48,881 (33,145–68,217)	0 (0–0)	93,311 (72,618–118,796)
United States	YLDs Number: Cumulative change (%)	19.9 (17.7–22.4)	35.5 (31.4–39.9)	17.0 (16.1–18.0)	-6.2 (-19.1 to 8.8)	-23.6 (-25.4 to -21.7)	14.8 (7.4–22.0)	60.9 (42.4–96.1)	-30.7 (-40.3 to -20.6)	-29.8 (-43.6 to -14.9)	14.6 (10.7–18.2)	-12.4 (-16.3 to -8.3)
United States	YLDs Rate: 1990	5,105.2 (3,894.0–6,443.8)	718.8 (514.1–971.3)	674.8 (479.7–913.7)	351.6 (234.0–479.6)	224.1 (163.4–296.4)	0.8 (0.6–1.1)	171.6 (115.4–238.6)	0.0 (0.0–0.0)	96.4 (68.8–123.6)	0.0 (0.0–0.0)	147.4 (116.7–184.2)
United States	YLDs Rate: 2015	5,193.3 (3,981.4–6,543.8)	850.0 (608.5–1,142.3)	677.8 (482.7–919.1)	282.4 (194.7–375.8)	146.9 (105.9–197.1)	0.8 (0.6–1.1)	239.7 (152.5–346.9)	0.0 (0.0–0.0)	58.3 (39.6–81.4)	0.0 (0.0–0.0)	111.7 (86.9–142.1)
United States	YLDs Rate: Cumulative change (%)	1.7 (-0.1 to 3.8)	18.3 (14.7–22.1)	0.5 (-0.3 to 1.3)	-19.0 (-30.2 to -5.9)	-34.5 (-36.1 to -32.8)	5.4 (-1.5 to 12.1)	39.5 (23.4–70.1)	-35.5 (-44.6 to -25.9)	-39.5 (-51.4 to -26.7)	0.3 (-2.8 to 3.3)	-24.3 (-27.7 to -20.8)
Australasia	Prevalence Number: 1990	4,883,295 (4,806,867–4,954,551)	1,336,901 (1,264,952–1,449,115)	1,817,117 (1,769,222–1,866,851)	1,093,591 (966,226–1,222,623)	276,912 (253,844–299,064)	1,257 (1,177–1,344)	99,368 (88,995–113,969)	4 (3–6)	16,430 (13,243–19,734)	0 (0–0)	59,339 (53,422–66,756)
Australasia	Prevalence Number: 2015	5,588,277 (5,521,490–5,661,287)	1,640,974 (1,540,908–1,756,972)	2,146,577 (2,090,759–2,204,814)	832,472 (696,175–977,206)	283,121 (261,259–305,440)	1,639 (1,497–1,782)	115,479 (102,836–131,013)	3 (2–5)	15,614 (12,533–19,426)	0 (0–0)	64,327 (57,852–72,545)
Australasia	Prevalence Number: Cumulative change (%)	14.4 (12.4–16.5)	23.0 (9.0–36.1)	18.2 (13.9–22.3)	-24.0 (-30.9 to -16.9)	2.5 (-8.5 to 13.7)	30.4 (24.4–37.9)	16.7 (-0.1 to 35.3)	-25.9 (-44.7 to -2.1)	-4.2 (-23.3 to 19.2)	16.6 (15.7–18.0)	8.7 (-4.2 to 22.1)
Australasia	Prevalence Rate: 1990	78,147.9 (76,836.9–79,351.7)	21,921.9 (20,684.7–23,891.5)	28,617.2 (27,855.4–29,403.8)	17,432.9 (15,356.4–19,528.9)	4,477.3 (4,108.1–4,834.2)	21.3 (20.0–22.9)	1,605.0 (1,437.0–1,842.0)	0.1 (0.1–0.1)	263.0 (212.7–316.2)	0.0 (0.0–0.0)	978.2 (881.8–1,098.2)
Australasia	Prevalence Rate: 2015	77,136.4 (76,167.7–78,188.1)	23,318.7 (21,869.7–24,948.6)	29,084.6 (28,326.8–29,868.4)	11,552.4 (9,625.7–13,602.1)	3,944.8 (3,640.6–4,254.9)	23.2 (21.2–25.3)	1,603.0 (1,427.9–1,819.5)	0.0 (0.0–0.1)	215.5 (172.7–269.2)	0.0 (0.0–0.0)	899.9 (809.9–1,013.9)
Australasia	Prevalence Rate: Cumulative change (%)	-1.3 (-3.1 to 0.6)	6.7 (-5.7 to 18.2)	1.7 (-2.1 to 5.1)	-33.8 (-39.9 to -27.5)	-11.7 (-21.2 to -2.0)	8.9 (3.9–15.1)	0.3 (-14.1 to 16.2)	-38.3 (-53.9 to -18.2)	-17.4 (-34.0 to 2.8)	-0.0 (-0.0 to 0.0)	-7.8 (-18.5 to 3.3)
Australasia	YLDs Number: 1990	353,499 (268,872–446,799)	48,766 (34,273–67,188)	42,459 (29,641–58,199)	49,764 (33,423–66,622)	4,193 (2,988–5,714)	209 (145–283)	11,028 (7,327–15,410)	0 (0–0)	4,475 (3,040–6,049)	0 (0–0)	6,100 (4,717–7,826)
Australasia	YLDs Number: 2015	390,234 (297,725–493,783)	59,268 (42,071–80,504)	49,823 (34,688–68,504)	37,895 (25,333–52,132)	4,278 (3,034–5,724)	271 (187–372)	15,090 (9,335–22,524)	0 (0–0)	2,535 (1,576–3,666)	0 (0–0)	6,068 (4,701–7,754)
Australasia	YLDs Number: Cumulative change (%)	10.4 (7.3–13.5)	21.9 (6.6–36.8)	17.3 (15.0–19.8)	-23.9 (-30.9 to -16.7)	2.2 (-7.7 to 12.1)	30.5 (6.6–56.5)	37.1 (11.1–75.6)	-25.9 (-44.6 to -2.2)	-42.9 (-59.7 to -22.8)	17.3 (1.8–31.7)	-0.4 (-8.0 to 8.5)
Australasia	YLDs Rate: 1990	5,551.4 (4,213.7–7,033.5)	807.1 (567.2–1,112.4)	678.7 (472.8–930.5)	793.6 (533.2–1,063.7)	68.7 (48.9–93.5)	3.6 (2.5–4.8)	178.2 (118.5–249.0)	0.0 (0.0–0.0)	71.7 (48.8–96.6)	0.0 (0.0–0.0)	98.5 (76.1–126.4)
Australasia	YLDs Rate: 2015	5,302.5 (4,045.2–6,721.0)	858.4 (609.2–1,164.2)	685.5 (478.4–941.0)	526.0 (352.3–725.4)	61.7 (43.7–82.4)	3.9 (2.7–5.3)	209.5 (130.0–312.9)	0.0 (0.0–0.0)	35.0 (21.7–50.8)	0.0 (0.0–0.0)	84.2 (65.2–107.6)
Australasia	YLDs Rate: Cumulative change (%)	-4.4 (-7.2 to -1.7)	6.7 (-7.0 to 20.0)	1.0 (-1.1 to 3.1)	-33.7 (-40.0 to -27.3)	-10.1 (-18.9 to -1.2)	8.9 (-11.4 to 31.2)	17.8 (-4.5 to 50.9)	-38.3 (-54.0 to -18.1)	-50.8 (-65.2 to -33.6)	2.2 (-11.4 to 14.7)	-14.4 (-20.8 to -6.8)
Australia	Prevalence Number: 1990	4,054,014 (3,982,752–4,121,816)	1,136,177 (1,066,365–1,249,572)	1,504,928 (1,458,386–1,551,092)	906,309 (800,603–1,013,823)	235,027 (212,395–256,486)	1,040 (971–1,118)	82,636 (72,723–96,906)	4 (2–6)	12,996 (9,998–15,979)	0 (0–0)	47,999 (42,835–54,588)
Australia	Prevalence Number: 2015	4,643,102 (4,578,980–4,711,562)	1,396,474 (1,297,014–1,515,742)	1,783,886 (1,731,006–1,839,237)	678,379 (563,419–802,116)	227,612 (206,293–248,327)	1,387 (1,259–1,513)	96,509 (84,228–111,477)	3 (2–4)	12,678 (9,858–16,039)	0 (0–0)	52,737 (46,845–60,610)
Australia	Prevalence Number: Cumulative change (%)	14.5 (12.1–17.0)	23.3 (7.5–38.6)	18.6 (13.6–23.4)	-25.2 (-32.7 to -17.1)	-2.9 (-15.0 to 9.8)	33.3 (26.0–41.8)	17.5 (-2.3 to 41.2)	-23.5 (-44.1 to 2.7)	-1.2 (-25.7 to 30.2)	17.4 (16.2–19.0)	10.3 (-4.2 to 27.4)
Australia	Prevalence Rate: 1990	78,386.0 (76,924.4–79,784.7)	22,506.4 (21,030.8–24,934.6)	28,641.4 (27,762.4–29,518.6)	17,439.1 (15,358.2–19,569.5)	4,593.8 (4,155.3–5,013.0)	21.5 (20.0–23.1)	1,613.6 (1,420.3–1,893.2)	0.1 (0.1–0.1)	251.4 (193.7–308.9)	0.0 (0.0–0.0)	960.0 (859.1–1,088.9)
Australia	Prevalence Rate: 2015	77,159.7 (76,050.6–78,345.6)	23,829.9 (22,111.6–25,806.8)	29,132.9 (28,274.2–30,006.6)	11,350.7 (9,392.6–13,442.5)	3,814.6 (3,456.7–4,155.2)	23.6 (21.4–25.7)	1,612.0 (1,408.4–1,864.1)	0.1 (0.0–0.1)	210.8 (164.0–267.1)	0.0 (0.0–0.0)	884.7 (786.2–1,016.0)

© 2017 Global Burden of Disease Child and Adolescent Health Collaboration. JAMA Pediatrics.

eTable 3. Prevalent Cases, Rates (per 100,000 population), Years Lived with Disability (YLDs), and Cumulative Percent Change with 95% Uncertainty Interval (UI) for the Top 10 Global Causes of YLDs in Children and Adolescents in 195 Countries and Territories, Aged 0 to 19 Years, Both Sexes, 1990 and 2015. *Note: best viewed by enlarging in browser.*

Location	Measure	All causes	Iron-deficiency anemia	Skin and subcutaneous diseases	Asthma	Hemoglobinopathies and hemolytic anemias	Diarrheal diseases	Congenital anomalies	Protein-energy malnutrition	Epilepsy	Malaria	Neonatal preterm birth complications
Australia	Prevalence Rate: Cumulative change (%)	-1.6 (-3.8 to 0.7)	6.3 (-8.1 to 19.8)	1.7 (-2.7 to 5.9)	-35.0 (-41.6 to -27.8)	-16.7 (-27.1 to -5.7)	9.7 (3.8–16.7)	0.5 (-16.5 to 20.6)	-37.7 (-54.5 to -16.0)	-15.1 (-36.0 to 11.8)	-0.0 (-0.0 to 0.0)	-7.5 (-19.5 to 6.7)
Australia	YLDs Number: 1990	293,416 (223,370–371,295)	41,703 (29,332–57,609)	35,282 (24,623–48,402)	41,243 (27,723–55,653)	2,707 (1,894–3,730)	173 (119–236)	9,118 (6,021–12,778)	0 (0–0)	3,542 (2,337–4,878)	0 (0–0)	5,009 (3,856–6,444)
Australia	YLDs Number: 2015	324,231 (247,255–410,223)	50,545 (35,603–68,630)	41,550 (28,896–57,139)	30,888 (20,681–42,909)	2,927 (2,061–3,933)	229 (155–316)	12,516 (7,802–18,715)	0 (0–0)	2,058 (1,248–3,005)	0 (0–0)	5,002 (3,870–6,394)
Australia	YLDs Number: Cumulative change (%)	10.5 (7.1–14.0)	21.6 (4.1–39.3)	17.8 (15.0–20.6)	-25.1 (-32.9 to -16.9)	8.5 (-6.2 to 22.4)	33.5 (5.2–64.7)	37.7 (9.2–75.9)	-23.5 (-44.1 to 2.7)	-41.0 (-60.6 to -15.8)	17.9 (-0.7 to 36.1)	0.0 (-9.0 to 10.0)
Australia	YLDs Rate: 1990	5,562.7 (4,226.3–7,048.1)	832.4 (585.2–1,156.0)	681.6 (474.7–931.5)	793.9 (535.7–1,070.8)	53.7 (37.6–73.9)	3.6 (2.5–4.9)	178.2 (117.8–249.3)	0.0 (0.0–0.0)	68.6 (45.3–94.2)	0.0 (0.0–0.0)	97.8 (75.3–126.0)
Australia	YLDs Rate: 2015	5,310.4 (4,047.8–6,738.3)	878.5 (619.4–1,189.8)	688.6 (482.1–946.9)	517.0 (343.9–718.6)	50.7 (35.8–68.1)	3.9 (2.6–5.4)	209.1 (130.8–312.5)	0.0 (0.0–0.0)	34.2 (20.9–50.1)	0.0 (0.0–0.0)	83.5 (64.6–107.0)
Australia	YLDs Rate: Cumulative change (%)	-4.5 (-7.6 to -1.4)	6.0 (-9.8 to 21.6)	1.0 (-1.4 to 3.5)	-34.9 (-41.7 to -27.7)	-5.3 (-18.3 to 7.1)	9.9 (-13.3 to 35.9)	17.7 (-6.8 to 50.9)	-37.6 (-54.5 to -16.1)	-49.3 (-66.0 to -27.4)	2.3 (-14.2 to 17.6)	-14.4 (-21.9 to -5.9)
New Zealand	Prevalence Number: 1990	829,281 (814,913–842,067)	200,724 (183,252–217,308)	312,189 (302,913–321,619)	187,282 (161,793–213,938)	41,884 (36,880–46,192)	216 (200–233)	16,731 (14,892–19,288)	0 (0–0)	3,433 (2,662–4,153)	0 (0–0)	11,340 (9,977–12,993)
New Zealand	Prevalence Number: 2015	945,174 (929,053–959,280)	244,500 (229,206–257,303)	362,690 (351,850–374,048)	154,093 (129,140–180,024)	55,509 (51,377–59,376)	252 (232–275)	18,969 (16,735–22,044)	0 (0–0)	2,935 (2,252–3,672)	0 (0–0)	11,589 (10,155–13,332)
New Zealand	Prevalence Number: Cumulative change (%)	14.0 (11.5–16.5)	22.1 (11.0–33.7)	16.2 (11.4–21.0)	-17.7 (-25.8 to -9.4)	33.1 (17.5–53.0)	16.6 (12.3–22.0)	14.0 (-5.3 to 35.9)	-49.0 (-62.9 to -31.1)	-13.5 (-35.0 to 13.1)	12.9 (12.7–13.2)	2.6 (-11.9 to 18.1)
New Zealand	Prevalence Rate: 1990	77,029.8 (75,646.5–78,265.6)	19,146.8 (17,521.6–20,753.6)	28,498.1 (27,658.7–29,379.0)	17,395.0 (15,028.1–19,921.4)	3,920.9 (3,451.9–4,317.8)	20.7 (19.2–22.4)	1,563.4 (1,391.5–1,802.6)	0.0 (0.0–0.1)	318.3 (246.8–385.0)	0.0 (0.0–0.0)	1,063.0 (935.7–1,217.4)
New Zealand	Prevalence Rate: 2015	77,023.5 (75,627.2–78,253.4)	20,789.1 (19,470.0–21,886.7)	28,846.3 (27,980.0–29,745.5)	12,527.4 (10,430.3–14,725.2)	4,589.0 (4,250.4–4,905.3)	21.7 (20.0–23.7)	1,558.7 (1,374.6–1,815.3)	0.0 (0.0–0.0)	238.2 (182.9–299.1)	0.0 (0.0–0.0)	976.7 (859.8–1,119.6)
New Zealand	Prevalence Rate: Cumulative change (%)	0.0 (-2.3 to 2.3)	8.8 (-1.1 to 18.8)	1.3 (-2.9 to 5.4)	-28.0 (-35.3 to -20.5)	17.6 (3.8–35.1)	4.8 (0.9–9.6)	0.3 (-16.8 to 19.7)	-53.9 (-66.6 to -36.9)	-24.3 (-43.1 to -1.0)	-0.0 (-0.0 to 0.0)	-7.8 (-20.5 to 5.7)
New Zealand	YLDs Number: 1990	60,083 (45,861–75,789)	7,063 (4,898–9,575)	7,176 (5,007–9,804)	8,520 (5,790–11,564)	1,486 (1,068–1,984)	36 (25–49)	1,909 (1,259–2,673)	0 (0–0)	932 (620–1,289)	0 (0–0)	1,090 (838–1,400)
New Zealand	YLDs Number: 2015	66,003 (50,075–83,055)	8,722 (6,160–11,804)	8,273 (5,765–11,422)	7,007 (4,705–9,761)	1,351 (964–1,807)	42 (29–57)	2,573 (1,582–3,902)	0 (0–0)	476 (297–701)	0 (0–0)	1,066 (811–1,364)
New Zealand	YLDs Number: Cumulative change (%)	9.9 (7.0–12.8)	23.8 (10.9–37.1)	15.2 (12.8–17.5)	-17.7 (-25.9 to -9.4)	-9.0 (-17.3 to 0.8)	18.2 (-6.1 to 44.0)	34.8 (7.5–79.0)	-49.0 (-62.9 to -31.1)	-48.3 (-65.2 to -27.9)	15.4 (2.7–28.4)	-2.0 (-10.8 to 7.3)
New Zealand	YLDs Rate: 1990	5,496.4 (4,194.8–6,957.3)	685.5 (477.3–926.9)	664.8 (464.7–904.6)	791.7 (536.8–1,076.2)	141.8 (101.8–189.4)	3.5 (2.4–4.7)	178.5 (117.5–249.7)	0.0 (0.0–0.0)	86.5 (57.5–119.9)	0.0 (0.0–0.0)	102.0 (78.5–131.0)
New Zealand	YLDs Rate: 2015	5,264.9 (3,987.6–6,646.7)	758.0 (535.6–1,026.8)	670.4 (468.7–923.7)	569.9 (379.2–791.1)	116.6 (83.2–156.1)	3.6 (2.5–4.9)	211.6 (130.0–321.5)	0.0 (0.0–0.0)	38.7 (24.1–57.0)	0.0 (0.0–0.0)	87.7 (66.6–112.4)
New Zealand	YLDs Rate: Cumulative change (%)	-4.2 (-6.8 to -1.5)	10.8 (-0.8 to 22.2)	0.8 (-1.3 to 2.8)	-28.0 (-35.4 to -20.3)	-17.7 (-25.1 to -9.0)	6.2 (-15.9 to 29.5)	18.6 (-5.4 to 57.1)	-53.9 (-66.5 to -37.1)	-54.7 (-69.6 to -37.0)	2.6 (-8.6 to 14.1)	-13.9 (-21.4 to -5.8)
High-income Asia Pacific	Prevalence Number: 1990	39,864,399 (39,647,230–40,058,327)	9,707,566 (9,570,732–9,854,909)	15,776,646 (15,580,058–15,970,957)	4,515,866 (3,912,578–5,148,440)	2,091,815 (2,030,710–2,154,150)	12,019 (11,323–12,847)	886,715 (835,573–946,162)	28 (18–41)	145,869 (123,520–170,587)	547 (259–896)	349,192 (319,217–381,648)
High-income Asia Pacific	Prevalence Number: 2015	26,481,561 (26,342,078–26,625,942)	6,465,007 (6,353,401–6,574,989)	10,625,742 (10,488,674–10,753,462)	1,693,356 (1,421,478–1,988,558)	1,375,105 (1,337,615–1,417,456)	6,409 (6,062–6,795)	617,514 (581,024–655,940)	22 (14–32)	86,975 (73,199–103,710)	170 (114–266)	221,380 (201,051–244,471)
High-income Asia Pacific	Prevalence Number: Cumulative change (%)	-33.6 (-33.9 to -33.2)	-33.4 (-34.5 to -32.2)	-32.6 (-33.6 to -31.7)	-62.5 (-65.6 to -59.1)	-34.2 (-36.5 to -32.0)	-46.7 (-48.1 to -45.4)	-30.3 (-34.6 to -25.8)	-22.6 (-36.8 to -8.5)	-40.3 (-47.7 to -32.8)	-65.2 (-82.5 to -35.6)	-36.5 (-41.6 to -31.5)
High-income Asia Pacific	Prevalence Rate: 1990	79,623.3 (79,128.4–80,050.0)	19,198.6 (18,938.3–19,472.1)	30,222.3 (29,862.3–30,594.3)	9,083.5 (7,838.0–10,396.9)	4,208.8 (4,084.0–4,336.0)	27.9 (26.2–30.0)	1,801.6 (1,695.5–1,925.8)	0.1 (0.0–0.1)	295.6 (250.5–345.2)	1.2 (0.6–1.9)	780.4 (716.5–847.7)
High-income Asia Pacific	Prevalence Rate: 2015	79,059.0 (78,621.3–79,501.0)	19,258.7 (18,958.1–19,552.2)	30,803.2 (30,419.4–31,161.3)	5,093.5 (4,279.3–5,990.1)	4,121.0 (4,008.6–4,247.1)	20.7 (19.6–22.0)	1,860.8 (1,751.5–1,976.2)	0.1 (0.0–0.1)	262.3 (221.6–312.9)	0.5 (0.4–0.9)	702.2 (640.1–772.4)
High-income Asia Pacific	Prevalence Rate: Cumulative change (%)	-0.7 (-1.3 to -0.1)	0.3 (-1.3 to 1.9)	1.9 (0.5–3.3)	-43.9 (-48.6 to -38.9)	-2.1 (-5.5 to 1.3)	-25.7 (-28.0 to -23.8)	3.4 (-3.1 to 10.0)	7.4 (-13.8 to 27.5)	-11.1 (-22.4 to -0.4)	-49.0 (-74.2 to -7.0)	-9.9 (-16.5 to -3.2)
High-income Asia Pacific	YLDs Number: 1990	2,474,189 (1,875,598–3,149,074)	371,744 (265,051–501,027)	388,308 (270,297–530,701)	205,957 (138,590–281,329)	34,419 (24,796–45,860)	1,996 (1,435–2,669)	68,906 (46,121–95,961)	3 (2–5)	39,820 (28,584–52,303)	13 (6–23)	32,950 (25,553–41,631)
High-income Asia Pacific	YLDs Number: 2015	1,520,962 (1,156,126–1,932,005)	251,785 (178,422–336,387)	262,974 (183,632–361,245)	77,277 (50,996–105,928)	20,599 (14,751–27,381)	1,068 (768–1,427)	64,847 (41,394–93,008)	2 (1–4)	14,260 (9,478–20,234)	5 (2–9)	16,610 (12,647–21,280)
High-income Asia Pacific	YLDs Number: Cumulative change (%)	-38.5 (-39.4 to -37.6)	-32.3 (-33.6 to -31.2)	-32.3 (-33.4 to -31.2)	-62.5 (-65.6 to -59.2)	-40.2 (-41.7 to -38.7)	-46.4 (-51.1 to -41.3)	-6.0 (-14.3 to 10.4)	-22.6 (-36.8 to -8.7)	-64.2 (-71.3 to -56.4)	-56.2 (-76.6 to -22.2)	-49.6 (-53.6 to -45.2)
High-income Asia Pacific	YLDs Rate: 1990	4,760.2 (3,601.5–6,067.8)	749.6 (534.1–1,007.3)	772.8 (540.0–1,061.9)	414.5 (277.9–566.1)	70.6 (50.9–94.3)	4.6 (3.3–6.2)	140.1 (93.7–194.9)	0.0 (0.0–0.0)	80.8 (58.2–106.8)	0.0 (0.0–0.1)	66.6 (51.7–84.2)
High-income Asia Pacific	YLDs Rate: 2015	4,430.2 (3,370.3–5,637.0)	760.2 (540.4–1,017.6)	782.2 (548.3–1,071.7)	232.5 (153.5–319.6)	62.8 (45.0–83.6)	3.5 (2.5–4.6)	195.6 (124.8–280.8)	0.0 (0.0–0.0)	43.0 (28.7–61.1)	0.0 (0.0–0.0)	49.9 (38.0–64.0)
High-income Asia Pacific	YLDs Rate: Cumulative change (%)	-6.9 (-8.2 to -5.5)	1.4 (-0.4 to 3.0)	1.2 (-0.4 to 2.8)	-43.9 (-48.6 to -39.0)	-11.0 (-13.3 to -8.8)	-25.4 (-31.9 to -18.5)	39.4 (27.1–64.3)	7.3 (-13.7 to 27.7)	-46.8 (-57.5 to -35.2)	-35.6 (-65.4 to 13.6)	-25.1 (-30.9 to -18.9)
Brunei	Prevalence Number: 1990	90,643 (89,312–91,852)	23,470 (22,780–24,290)	32,831 (31,771–33,799)	10,628 (8,678–12,891)	6,422 (5,990–6,866)	35 (32–38)	2,243 (1,995–2,541)	0 (0–0)	665 (506–839)	150 (88–291)	876 (774–1,001)
Brunei	Prevalence Number: 2015	106,158 (104,709–107,505)	27,104 (26,109–28,397)	41,879 (40,493–43,134)	10,916 (8,891–13,261)	5,951 (5,524–6,417)	27 (25–30)	2,587 (2,282–2,939)	0 (0–0)	716 (539–923)	143 (93–229)	928 (809–1,085)
Brunei	Prevalence Number: Cumulative change (%)	17.1 (15.1–19.2)	15.5 (10.6–21.9)	27.6 (22.3–33.1)	2.8 (-3.9 to 10.3)	-7.2 (-15.6 to 2.5)	-20.8 (-24.6 to -17.5)	15.9 (-2.8 to 35.8)	-13.2 (-44.1 to 22.2)	9.6 (-22.6 to 48.3)	-0.5 (-25.1 to 19.8)	6.3 (-8.9 to 25.0)
Brunei	Prevalence Rate: 1990	80,091.7 (79,062.0–81,106.3)	21,278.1 (20,655.3–21,962.8)	30,005.5 (29,041.5–30,889.4)	9,495.8 (7,777.6–11,483.8)	5,622.3 (5,245.4–6,009.0)	26.4 (24.4–28.9)	1,943.1 (1,727.2–2,199.5)	0.1 (0.0–0.1)	580.6 (443.9–733.3)	122.6 (73.1–230.2)	684.9 (600.6–793.9)
Brunei	Prevalence Rate: 2015	79,457.4 (78,354.9–80,479.2)	20,570.2 (19,872.6–21,477.4)	30,496.2 (29,475.6–31,433.6)	8,303.2 (6,751.2–10,090.1)	4,461.9 (4,143.9–4,805.7)	21.7 (20.1–23.6)	1,942.3 (1,714.3–2,205.3)	0.1 (0.0–0.1)	536.5 (405.0–694.4)	112.5 (72.5–180.2)	714.3 (624.7–832.8)
Brunei	Prevalence Rate: Cumulative change (%)	-0.8 (-2.5 to 1.0)	-3.3 (-6.8 to 1.5)	1.7 (-2.4 to 6.0)	-12.5 (-18.1 to -6.2)	-20.5 (-27.7 to -12.3)	-17.7 (-21.4 to -14.3)	0.4 (-15.9 to 17.7)	-17.5 (-45.8 to 15.2)	-5.9 (-33.6 to 27.0)	-5.5 (-26.0 to 12.8)	4.9 (-11.2 to 23.7)
Brunei	YLDs Number: 1990	5,406 (4,118–6,819)	924 (658–1,236)	835 (586–1,148)	484 (318–674)	58 (41–79)	5 (4–8)	264 (148–452)	0 (0–0)	215 (133–311)	4 (2–9)	65 (48–85)
Brunei	YLDs Number: 2015	6,492 (4,909–8,199)	1,058 (753–1,435)	1,007 (702–1,393)	497 (325–696)	62 (44–84)	4 (3–6)	361 (210–607)	0 (0–0)	123 (74–184)	4 (2–8)	72 (54–94)
Brunei	YLDs Number: Cumulative change (%)	20.1 (15.5–25.3)	14.6 (9.7–21.6)	20.5 (17.1–24.0)	2.8 (-4.2 to 10.9)	6.2 (-0.9 to 15.2)	-19.8 (-36.3 to -1.8)	46.3 (-23.8 to 142.6)	-13.0 (-43.8 to 22.6)	-40.7 (-63.1 to -13.0)	1.8 (-26.4 to 33.0)	12.0 (0.8–24.1)
Brunei	YLDs Rate: 1990	4,960.2 (3,779.7–6,245.3)	836.8 (598.2–1,117.6)	740.2 (517.9–1,018.7)	432.8 (285.5–603.2)	52.9 (37.8–71.1)	4.4 (3.0–6.0)	228.6 (128.8–390.5)	0.0 (0.0–0.0)	187.3 (116.4–273.0)	4.1 (2.0–7.9)	56.6 (42.0–74.4)
Brunei	YLDs Rate: 2015	4,791.0 (3,619.7–6,052.3)	817.2 (581.5–1,103.5)	748.2 (523.4–1,030.9)	378.6 (248.3–529.5)	47.7 (34.0–64.5)	3.6 (2.5–5.0)	271.2 (158.5–457.4)	0.0 (0.0–0.0)	92.7 (55.1–138.3)	3.7 (1.9–6.5)	54.8 (40.8–71.0)
Brunei	YLDs Rate: Cumulative change (%)	-3.4 (-6.9 to 0.6)	-2.3 (-6.1 to 3.1)	1.1 (-1.0 to 3.2)	-12.4 (-18.2 to -6.1)	-9.9 (-15.6 to -2.9)	-16.6 (-33.3 to 2.0)	27.1 (-33.9 to 110.8)	-17.5 (-45.8 to 15.4)	-49.0 (-68.4 to -25.3)	-5.1 (-30.3 to 22.6)	-2.9 (-13.0 to 7.8)
Japan	Prevalence Number: 1990	26,501,571 (26,419,335–26,582,985)	6,140,967 (6,083,183–6,198,590)	10,778,043 (10,698,947–10,857,824)	3,250,845 (2,760,557–3,774,179)	1,363,176 (1,338,004–1,387,328)	7,193 (6,796–7,647)	596,751 (568,291–623,590)	15 (10–22)	77,229 (66,022–89,886)	0 (0–0)	209,312 (192,312–226,539)
Japan	Prevalence Number: 2015	17,437,131 (17,370,407–17,501,863)	4,180,655 (4,146,212–4,218,493)	7,047,782 (6,987,414–7,102,453)	1,109,204 (926,000–1,317,131)	903,346 (887,431–920,199)	4,504 (4,230–4,809)	425,923 (405,873–447,231)	20 (13–30)	51,405 (43,730–59,955)	0 (0–0)	140,655 (129,423–152,677)
Japan	Prevalence Number: Cumulative change (%)	-34.2 (-34.4 to -34.0)	-31.9 (-32.7 to -31.1)	-34.6 (-35.2 to -34.0)	-65.9 (-69.0 to -62.2)	-33.7 (-35.0 to -32.4)	-37.4 (-38.1 to -36.7)	-28.6 (-31.8 to -25.0)	33.4 (7.4–53.5)	-33.4 (-37.3 to -29.2)	-23.8 (-36.0 to -3.0)	-32.8 (-35.9 to -29.5)
Japan	Prevalence Rate: 1990	79,725.9 (79,456.0–79,994.9)	18,508.6 (18,335.5–18,680.9)	30,958.0 (30,736.1–31,180.3)	9,827.7 (8,328.0–11,431.0)	4,146.2 (4,070.3–4,219.5)	25.4 (23.9–27.1)	1,825.2 (1,738.9–1,907.3)	0.1 (0.0–0.1)	233.8 (200.9–272.1)	0.0 (0.0–0.0)	710.4 (654.8–764.5)
Japan	Prevalence Rate: 2015	79,171.3 (78,859.9–79,481.1)	18,870.7 (18,709.7–19,042.8)	31,416.3 (31,154.8–31,659.2)	5,026.3 (4,194.0–5,968.9)	4,107.2 (4,035.2–4,183.6)	21.8 (20.4–23.3)	1,945.3 (1,853.3–2,041.8)	0.1 (0.1–0.1)	233.9 (199.1–273.1)	0.0 (0.0–0.0)	671.2 (618.5–727.4)
Japan	Prevalence Rate: Cumulative change (%)	-0.7 (-1.1 to -0.3)	2.0 (0.8–3.2)	1.5 (0.6–2.4)	-48.8 (-53.4 to -43.4)	-0.9 (-2.8 to 1.0)	-14.3 (-15.2 to -13.4)	6.6 (1.9–12.0)	87.3 (51.5–116.5)	0.0 (-5.8 to 6.1)	7.6 (-10.1 to 36.7)	-5.5 (-9.5 to -1.3)
Japan	YLDs Number: 1990	1,623,385 (1,229,309–2,060,322)	232,878 (166,720–314,626)	273,786 (191,087–377,460)	148,344 (97,856–203,592)	25,880 (18,679–34,481)	1,195 (864–1,591)	51,210 (33,722–72,083)	1 (1–3)	21,218 (15,017–27,559)	0 (0–0)	19,146 (14,641–24,399)

© 2017 Global Burden of Disease Child and Adolescent Health Collaboration. JAMA Pediatrics.

eTable 3. Prevalent Cases, Rates (per 100,000 population), Years Lived with Disability (YLDs), and Cumulative Percent Change with 95% Uncertainty Interval (UI) for the Top 10 Global Causes of YLDs in Children and Adolescents in 195 Countries and Territories, Aged 0 to 19 Years, Both Sexes, 1990 and 2015. *Note: best viewed by enlarging in browser.*

Location	Measure	All causes	Iron-deficiency anemia	Skin and subcutaneous diseases	Asthma	Hemoglobinopathies and hemolytic anemias	Diarrheal diseases	Congenital anomalies	Protein-energy malnutrition	Epilepsy	Malaria	Neonatal preterm birth complications
Japan	YLDs Number: 2015	988,731 (752,853–1,255,832)	163,418 (117,025–219,375)	182,740 (127,136–249,788)	50,629 (33,514–69,783)	15,357 (11,035–20,422)	750 (540–994)	46,428 (29,514–67,055)	2 (1–4)	8,418 (5,586–11,756)	0 (0–0)	10,313 (7,849–13,362)
Japan	YLDs Number: Cumulative change (%)	-39.1 (-40.1 to -38.1)	-29.8 (-30.7 to -28.9)	-33.2 (-34.7 to -31.8)	-65.8 (-69.0 to -62.2)	-40.7 (-42.0 to -39.2)	-37.2 (-41.6 to -32.6)	-9.4 (-17.2 to 9.6)	33.1 (7.2–52.8)	-60.5 (-67.1 to -53.3)	-22.9 (-36.1 to -3.3)	-46.1 (-50.5 to -41.7)
Japan	YLDs Rate: 1990	4,688.5 (3,545.9–5,960.6)	712.7 (510.6–962.3)	821.5 (574.9–1,123.9)	448.7 (295.0–622.0)	81.2 (58.7–108.2)	4.2 (3.1–5.6)	156.8 (103.4–220.1)	0.0 (0.0–0.0)	64.3 (45.6–83.3)	0.0 (0.0–0.0)	58.2 (44.7–74.4)
Japan	YLDs Rate: 2015	4,409.6 (3,359.5–5,602.2)	738.4 (528.4–991.8)	827.9 (577.6–1,127.8)	229.5 (151.8–316.3)	70.2 (50.5–93.3)	3.6 (2.6–4.8)	212.2 (134.9–306.3)	0.0 (0.0–0.0)	38.3 (25.5–53.6)	0.0 (0.0–0.0)	47.0 (35.8–60.8)
Japan	YLDs Rate: Cumulative change (%)	-5.9 (-7.5 to -4.4)	3.6 (2.4–4.9)	0.8 (-1.4 to 2.8)	-48.8 (-53.5 to -43.3)	-13.6 (-15.6 to -11.5)	-14.1 (-20.2 to -7.8)	35.3 (23.6–63.7)	87.1 (51.3–115.9)	-40.7 (-50.7 to -30.0)	9.3 (-9.1 to 37.2)	-19.2 (-25.3 to -12.8)
Singapore	Prevalence Number: 1990	675,829 (666,826–684,644)	181,300 (172,783–191,199)	250,227 (241,926–258,062)	75,282 (64,122–85,260)	45,335 (42,446–48,164)	307 (276–347)	15,495 (13,772–17,886)	0 (0–0)	2,330 (1,822–2,875)	13 (8–21)	6,171 (5,478–7,078)
Singapore	Prevalence Number: 2015	697,624 (688,742–706,656)	175,144 (166,837–186,273)	280,540 (272,207–288,948)	46,187 (38,336–54,452)	44,223 (41,480–47,248)	149 (140–158)	14,793 (13,024–17,291)	0 (0–0)	2,149 (1,592–2,709)	13 (8–22)	4,899 (4,218–5,843)
Singapore	Prevalence Number: Cumulative change (%)	3.2 (1.5–5.0)	-3.3 (-11.5 to 4.0)	12.2 (7.4–17.0)	-38.6 (-47.3 to -30.6)	-2.3 (-10.8 to 6.8)	-51.2 (-56.7 to -46.4)	-4.0 (-19.7 to 13.8)	-51.0 (-60.7 to -39.0)	-6.6 (-32.5 to 23.0)	0.5 (-19.9 to 19.5)	-20.3 (-33.4 to -4.4)
Singapore	Prevalence Rate: 1990	79,176.3 (78,126.8–80,218.2)	21,544.0 (20,596.3–22,713.7)	28,879.5 (27,938.8–29,761.2)	8,963.4 (7,589.9–10,163.8)	5,314.4 (4,976.0–5,646.6)	35.6 (32.1–40.4)	1,812.3 (1,610.1–2,089.9)	0.0 (0.0–0.0)	273.3 (213.6–337.3)	1.6 (1.1–2.6)	703.3 (622.0–810.2)
Singapore	Prevalence Rate: 2015	77,645.7 (76,549.0–78,752.4)	19,490.5 (18,563.2–20,734.1)	29,705.2 (28,861.6–30,563.6)	5,161.5 (4,261.2–6,127.9)	4,954.1 (4,640.2–5,285.9)	18.7 (17.5–20.0)	1,669.6 (1,468.3–1,948.7)	0.0 (0.0–0.0)	244.0 (181.0–308.2)	1.7 (1.1–2.8)	594.5 (516.5–701.7)
Singapore	Prevalence Rate: Cumulative change (%)	-1.9 (-3.6 to -0.2)	-9.4 (-16.9 to -2.8)	2.9 (-1.4 to 7.3)	-42.3 (-50.7 to -34.8)	-6.6 (-14.7 to 2.0)	-47.3 (-53.2 to -41.9)	-7.4 (-22.6 to 9.8)	-50.7 (-60.6 to -37.9)	-9.6 (-34.9 to 18.5)	3.0 (-17.8 to 23.3)	-15.1 (-28.7 to 1.1)
Singapore	YLDs Number: 1990	39,950 (30,277–50,830)	6,989 (4,987–9,358)	5,747 (3,984–7,954)	3,435 (2,312–4,729)	481 (342–648)	50 (35–69)	1,138 (770–1,560)	0 (0–0)	642 (427–895)	0 (0–0)	556 (423–714)
Singapore	YLDs Number: 2015	39,610 (30,263–50,077)	6,825 (4,875–9,167)	6,249 (4,312–8,629)	2,110 (1,393–2,912)	445 (314–599)	24 (16–33)	1,632 (1,014–2,394)	0 (0–0)	369 (224–558)	0 (0–0)	423 (317–542)
Singapore	YLDs Number: Cumulative change (%)	-0.8 (-3.4 to 1.9)	-2.2 (-11.7 to 5.6)	8.7 (5.9–11.1)	-38.5 (-47.2 to -30.5)	-7.2 (-20.5 to 3.6)	-50.8 (-62.0 to -37.7)	42.8 (17.6–70.6)	-50.6 (-60.3 to -38.6)	-41.5 (-62.1 to -18.3)	2.8 (-22.4 to 31.7)	-23.7 (-33.1 to -14.2)
Singapore	YLDs Rate: 1990	4,662.0 (3,531.8–5,938.8)	845.4 (605.2–1,129.3)	670.1 (466.3–924.5)	409.1 (275.4–563.6)	58.1 (41.3–78.3)	5.9 (4.1–8.1)	133.2 (90.2–182.7)	0.0 (0.0–0.0)	75.3 (50.2–104.9)	0.1 (0.0–0.1)	65.1 (49.6–83.6)
Singapore	YLDs Rate: 2015	4,241.1 (3,234.2–5,361.2)	776.6 (554.5–1,043.1)	683.3 (474.8–945.1)	235.9 (154.9–326.8)	50.3 (35.5–67.7)	3.1 (2.1–4.2)	184.5 (114.8–271.6)	0.0 (0.0–0.0)	42.1 (25.5–63.4)	0.1 (0.0–0.1)	47.6 (35.7–61.0)
Singapore	YLDs Rate: Cumulative change (%)	-9.0 (-11.5 to -6.4)	-8.0 (-16.8 to -0.2)	2.0 (-0.1 to 3.9)	-42.2 (-50.4 to -34.6)	-13.2 (-25.9 to -2.9)	-46.8 (-58.9 to -31.8)	38.0 (13.4–64.5)	-50.4 (-60.3 to -37.7)	-43.3 (-63.2 to -20.6)	2.9 (-22.3 to 33.8)	-26.8 (-35.8 to -17.8)
South Korea	Prevalence Number: 1990	12,596,354 (12,432,401–12,748,474)	3,361,828 (3,245,738–3,490,633)	4,715,544 (4,561,161–4,864,802)	1,179,110 (1,011,147–1,318,458)	676,881 (629,700–725,733)	4,483 (4,183–4,867)	272,224 (239,479–310,658)	12 (7–20)	65,643 (51,322–81,379)	383 (104–703)	132,831 (115,780–154,191)
South Korea	Prevalence Number: 2015	8,240,647 (8,130,190–8,351,434)	2,082,102 (1,969,396–2,186,421)	3,255,540 (3,152,661–3,354,656)	527,047 (432,656–624,740)	421,584 (391,725–453,438)	1,727 (1,628–1,838)	174,210 (152,125–200,222)	1 (0–1)	32,703 (24,563–41,819)	12 (8–17)	74,897 (64,253–90,022)
South Korea	Prevalence Number: Cumulative change (%)	-34.6 (-35.6 to -33.4)	-38.1 (-41.0 to -35.0)	-30.9 (-33.9 to -28.0)	-55.3 (-61.5 to -50.6)	-37.6 (-43.5 to -31.2)	-61.4 (-64.5 to -58.8)	-35.6 (-46.3 to -23.5)	-90.2 (-93.2 to -86.4)	-49.6 (-62.6 to -34.8)	7,768.9 (-98.1 to -89.5)	-43.2 (-53.9 to -30.3)
South Korea	Prevalence Rate: 1990	79,475.4 (78,324.1–80,501.0)	20,583.5 (19,890.4–21,365.0)	28,783.4 (27,837.4–29,654.1)	7,521.1 (6,430.3–8,411.4)	4,276.7 (3,974.1–4,591.0)	32.4 (30.1–35.4)	1,750.4 (1,544.0–1,994.5)	0.1 (0.1–0.1)	422.8 (330.3–522.6)	2.5 (0.7–4.6)	928.2 (815.6–1,066.1)
South Korea	Prevalence Rate: 2015	78,835.0 (77,669.0–79,992.0)	19,874.1 (19,116.3–20,612.1)	29,526.0 (28,632.8–30,408.4)	5,215.9 (4,254.2–6,224.8)	4,058.8 (3,774.5–4,365.8)	18.4 (17.3–19.7)	1,694.1 (1,481.7–1,943.8)	0.0 (0.0–0.0)	323.5 (243.2–415.6)	0.1 (0.1–0.2)	779.6 (674.8–926.2)
South Korea	Prevalence Rate: Cumulative change (%)	-0.8 (-2.5 to 1.1)	-3.4 (-7.4 to 0.1)	2.6 (-1.9 to 6.7)	-30.6 (-40.4 to -22.9)	-4.9 (-14.0 to 4.9)	-43.2 (-48.1 to -39.1)	-2.7 (-19.0 to 15.5)	-86.0 (-90.5 to -80.5)	-22.6 (-42.8 to 0.5)	11,110.0 (-97.2 to -84.7)	-15.5 (-30.1 to 2.4)
South Korea	YLDs Number: 1990	805,446 (613,731–1,025,454)	130,952 (92,641–175,160)	107,939 (75,240–149,200)	53,693 (35,881–74,128)	7,997 (5,720–10,681)	744 (508–1,021)	16,292 (11,278–22,376)	1 (0–2)	17,744 (12,018–25,005)	8 (2–16)	13,182 (10,198–16,523)
South Korea	YLDs Number: 2015	486,127 (368,600–615,685)	80,482 (57,018–108,284)	72,977 (50,196–100,602)	24,040 (15,872–33,404)	4,733 (3,342–6,360)	288 (198–402)	16,425 (10,783–23,350)	0 (0–0)	5,348 (3,349–8,028)	0 (0–0)	5,799 (4,393–7,542)
South Korea	YLDs Number: Cumulative change (%)	-39.6 (-40.9 to -38.4)	-38.5 (-42.0 to -35.9)	-32.4 (-33.9 to -30.8)	-55.2 (-61.3 to -50.2)	-40.8 (-45.3 to -35.9)	-60.9 (-69.5 to -51.1)	0.6 (-14.3 to 17.7)	-90.1 (-93.2 to -86.3)	-69.5 (-78.9 to -58.6)	4,922.5 (-98.1 to -89.5)	-56.0 (-61.0 to -50.1)
South Korea	YLDs Rate: 1990	4,918.9 (3,749.2–6,272.1)	821.1 (579.9–1,096.9)	678.4 (473.3–933.5)	342.7 (228.4–472.7)	49.9 (35.8–66.7)	5.4 (3.7–7.5)	104.9 (72.4–144.3)	0.0 (0.0–0.0)	114.5 (78.0–161.0)	0.1 (0.0–0.1)	84.4 (65.2–105.9)
South Korea	YLDs Rate: 2015	4,476.3 (3,391.6–5,689.5)	803.5 (568.3–1,075.3)	689.9 (482.0–945.1)	238.0 (156.8–333.4)	47.0 (33.5–63.3)	3.1 (2.1–4.3)	159.9 (105.2–227.5)	0.0 (0.0–0.0)	53.0 (33.2–80.2)	0.0 (0.0–0.0)	56.3 (42.9–73.2)
South Korea	YLDs Rate: Cumulative change (%)	-9.0 (-10.9 to -7.2)	-2.1 (-7.2 to 1.8)	1.7 (-0.4 to 3.9)	-30.5 (-40.4 to -22.3)	-5.6 (-12.7 to 1.8)	-42.5 (-54.7 to -27.9)	52.2 (29.6–78.3)	-86.0 (-90.5 to -80.4)	-53.2 (-67.6 to -36.2)	7,228.4 (-97.2 to -84.7)	-33.3 (-40.8 to -24.5)
Western Europe	Prevalence Number: 1990	81,080,865 (80,749,861–81,414,644)	21,203,531 (20,749,526–21,736,161)	28,224,583 (27,837,172–28,626,856)	9,323,036 (8,117,963–10,520,454)	14,020,716 (13,684,685–14,345,171)	43,095 (41,443–44,807)	1,265,055 (1,208,118–1,324,118)	71 (47–101)	289,210 (255,441–331,452)	0 (0–0)	865,798 (796,574–939,633)
Western Europe	Prevalence Number: 2015	74,207,193 (73,820,404–74,591,011)	20,289,274 (19,816,924–20,841,135)	25,919,813 (25,563,334–26,259,212)	6,752,467 (5,746,895–7,798,345)	11,491,219 (11,152,642–11,819,942)	38,753 (37,069–40,537)	1,320,347 (1,252,293–1,393,928)	43 (29–62)	271,816 (232,572–317,542)	0 (0–0)	737,461 (680,120–794,737)
Western Europe	Prevalence Number: Cumulative change (%)	-8.5 (-9.0 to -8.0)	-4.3 (-7.3 to -1.1)	-8.2 (-9.7 to -6.7)	-27.6 (-30.9 to -24.1)	-18.0 (-20.6 to -15.4)	-10.1 (-11.1 to -9.0)	4.4 (-1.4 to 10.5)	-38.1 (-48.0 to -28.4)	-6.0 (-14.6 to 3.0)	-4.6 (-5.7 to -3.2)	-14.8 (-19.7 to -9.8)
Western Europe	Prevalence Rate: 1990	82,588.1 (82,228.4–82,936.4)	22,600.7 (22,078.5–23,179.4)	27,739.4 (27,363.6–28,124.1)	9,339.7 (8,122.1–10,577.9)	14,430.4 (14,085.9–14,766.7)	48.4 (46.5–50.4)	1,303.8 (1,245.2–1,364.8)	0.1 (0.1–0.1)	296.2 (262.1–338.5)	0.0 (0.0–0.0)	935.1 (861.7–1,011.8)
Western Europe	Prevalence Rate: 2015	82,398.2 (81,953.0–82,830.1)	22,999.8 (22,448.0–23,657.1)	28,333.1 (27,947.2–28,706.4)	7,433.7 (6,316.4–8,588.9)	12,828.0 (12,450.3–13,194.7)	45.1 (43.1–47.2)	1,473.5 (1,397.7–1,555.7)	0.1 (0.0–0.1)	302.5 (258.4–354.1)	0.0 (0.0–0.0)	847.7 (783.0–913.0)
Western Europe	Prevalence Rate: Cumulative change (%)	-0.2 (-0.8 to 0.4)	1.8 (-1.8 to 5.3)	2.1 (0.5–3.7)	-20.5 (-23.9 to -16.7)	-11.1 (-13.9 to -8.3)	-6.7 (-7.8 to -5.6)	13.1 (6.7–19.6)	-35.7 (-46.2 to -25.2)	2.2 (-7.3 to 12.0)	0.3 (0.2–0.5)	-9.3 (-14.3 to -4.2)
Western Europe	YLDs Number: 1990	5,211,605 (3,963,956–6,619,140)	755,107 (535,855–1,031,905)	597,143 (411,601–830,692)	424,387 (288,822–571,835)	150,721 (109,364–201,397)	7,117 (5,142–9,334)	109,886 (75,795–150,743)	8 (5–14)	79,308 (56,668–102,552)	0 (0–0)	92,495 (72,257–115,744)
Western Europe	YLDs Number: 2015	4,510,597 (3,435,779–5,722,260)	737,447 (523,582–1,000,456)	551,452 (379,222–769,127)	307,706 (205,479–420,908)	126,465 (91,310–169,088)	6,406 (4,604–8,441)	141,916 (88,338–206,055)	5 (3–8)	44,328 (29,494–62,443)	0 (0–0)	65,889 (51,091–83,276)
Western Europe	YLDs Number: Cumulative change (%)	-13.4 (-14.5 to -12.3)	-2.3 (-6.4 to 1.8)	-7.6 (-8.3 to -6.9)	-27.5 (-30.7 to -24.0)	-16.1 (-18.9 to -12.6)	-10.0 (-14.0 to -5.7)	28.4 (10.5–53.7)	-37.7 (-48.7 to -26.0)	-44.3 (-55.3 to -32.4)	-3.3 (-6.2 to -0.3)	-28.8 (-32.5 to -24.8)
Western Europe	YLDs Rate: 1990	5,175.9 (3,932.8–6,583.7)	810.2 (574.9–1,108.5)	597.7 (411.8–828.2)	425.3 (288.3–576.0)	156.9 (113.7–209.5)	8.0 (5.8–10.5)	113.3 (78.1–155.3)	0.0 (0.0–0.0)	81.3 (58.0–105.0)	0.0 (0.0–0.0)	94.9 (74.0–118.7)
Western Europe	YLDs Rate: 2015	4,952.6 (3,771.4–6,286.7)	836.5 (593.7–1,135.4)	607.3 (417.1–843.2)	338.8 (226.5–463.8)	141.5 (102.2–189.3)	7.5 (5.4–9.8)	158.4 (98.6–230.1)	0.0 (0.0–0.0)	49.3 (32.9–69.5)	0.0 (0.0–0.0)	73.4 (56.9–92.7)
Western Europe	YLDs Rate: Cumulative change (%)	-4.3 (-5.5 to -3.0)	3.3 (-1.2 to 7.8)	1.6 (0.8–2.4)	-20.4 (-23.9 to -16.6)	-9.8 (-12.8 to -6.2)	-6.6 (-10.8 to -2.2)	39.0 (19.7–66.2)	-35.1 (-47.3 to -22.5)	-39.5 (-51.4 to -26.7)	1.3 (-1.7 to 4.6)	-22.7 (-26.6 to -18.5)
Andorra	Prevalence Number: 1990	10,816 (10,636–10,975)	2,885 (2,660–3,142)	3,851 (3,677–4,031)	1,111 (916–1,319)	2,054 (1,825–2,370)	3 (3–3)	207 (184–239)	0 (0–0)	37 (27–48)	0 (0–0)	84 (72–98)
Andorra	Prevalence Number: 2015	13,461 (13,218–13,660)	3,707 (3,442–3,980)	4,797 (4,588–4,989)	1,149 (929–1,395)	2,374 (2,057–2,867)	3 (3–4)	250 (220–286)	0 (0–0)	42 (30–57)	0 (0–0)	96 (82–118)
Andorra	Prevalence Number: Cumulative change (%)	24.5 (21.7–27.0)	28.7 (13.6–41.8)	24.6 (17.7–31.9)	3.5 (-7.2 to 16.0)	16.2 (-4.4 to 43.1)	8.7 (2.4–13.8)	21.2 (1.3–43.8)	-43.4 (-80.6 to 14.7)	15.9 (-16.3 to 51.1)	29.9 (28.3–31.8)	15.6 (-4.6 to 41.5)
Andorra	Prevalence Rate: 1990	83,497.7 (82,011.6–84,745.4)	23,246.9 (21,291.2–25,430.7)	28,682.3 (27,400.9–29,993.7)	8,452.5 (6,943.3–10,039.6)	15,998.2 (14,216.1–18,462.4)	29.4 (26.9–32.0)	1,615.5 (1,435.1–1,864.2)	0.0 (0.0–0.1)	290.1 (213.7–376.1)	0.0 (0.0–0.0)	677.6 (586.7–789.4)
Andorra	Prevalence Rate: 2015	83,086.0 (81,593.9–84,332.0)	23,467.5 (21,763.0–25,237.3)	29,127.2 (27,879.0–30,287.3)	6,987.0 (5,644.8–8,469.9)	14,742.6 (12,778.1–17,815.8)	25.1 (23.0–27.4)	1,553.3 (1,366.6–1,781.6)	0.0 (0.0–0.0)	265.1 (188.5–353.2)	0.0 (0.0–0.0)	641.3 (547.2–778.5)
Andorra	Prevalence Rate: Cumulative change (%)	-0.5 (-2.7 to 1.6)	1.2 (-11.1 to 11.5)	1.6 (-4.1 to 7.4)	-17.2 (-25.9 to -7.3)	-7.4 (-23.7 to 14.2)	-14.6 (-19.7 to -10.5)	-3.3 (-19.2 to 14.7)	-54.5 (-85.4 to -2.8)	-7.4 (-33.2 to 20.8)	0.0 (-0.0 to 0.0)	-4.9 (-20.8 to 15.4)
Andorra	YLDs Number: 1990	681 (516–865)	101 (71–140)	84 (57–116)	50 (33–70)	26 (16–37)	0 (0–0)	21 (12–36)	0 (0–0)	11 (7–16)	0 (0–0)	8 (6–10)
Andorra	YLDs Number: 2015	809 (611–1,030)	133 (92–181)	105 (72–147)	52 (33–73)	29 (18–41)	0 (0–0)	34 (16–65)	0 (0–0)	7 (4–11)	0 (0–0)	8 (6–10)
Andorra	YLDs Number: Cumulative change (%)	18.7 (14.3–23.8)	31.4 (11.1–43.3)	25.7 (22.8–28.3)	3.6 (-7.7 to 16.1)	12.3 (3.9–21.0)	9.5 (-10.5 to 32.3)	63.5 (-17.6 to 190.3)	-43.3 (-80.3 to 14.0)	-34.7 (-58.3 to -5.7)	31.3 (20.3–43.0)	-1.3 (-12.6 to 11.8)

© 2017 Global Burden of Disease Child and Adolescent Health Collaboration. JAMA Pediatrics.

eTable 3. Prevalent Cases, Rates (per 100,000 population), Years Lived with Disability (YLDs), and Cumulative Percent Change with 95% Uncertainty Interval (UI) for the Top 10 Global Causes of YLDs in Children and Adolescents in 195 Countries and Territories, Aged 0 to 19 Years, Both Sexes, 1990 and 2015. *Note: best viewed by enlarging in browser.*

Location	Measure	All causes	Iron-deficiency anemia	Skin and subcutaneous diseases	Asthma	Hemoglobinopathies and hemolytic anemias	Diarrheal diseases	Congenital anomalies	Protein-energy malnutrition	Epilepsy	Malaria	Neonatal preterm birth complications
Andorra	YLDs Rate: 1990	5,130.4 (3,876.5–6,530.0)	823.7 (578.2–1,144.4)	638.7 (438.1–886.6)	385.2 (253.0–535.2)	204.6 (127.0–297.3)	4.9 (3.4–6.6)	169.6 (97.7–284.1)	0.0 (0.0–0.0)	87.1 (55.1–124.4)	0.0 (0.0–0.0)	64.9 (48.3–84.4)
Andorra	YLDs Rate: 2015	4,934.5 (3,724.0–6,289.4)	839.1 (581.4–1,146.2)	645.3 (444.6–899.5)	318.6 (205.9–444.4)	180.1 (116.1–259.0)	4.2 (2.8–5.7)	211.3 (104.6–407.6)	0.0 (0.0–0.0)	44.6 (25.8–69.3)	0.0 (0.0–0.0)	50.9 (37.4–66.1)
Andorra	YLDs Rate: Cumulative change (%)	-3.8 (-7.5 to 0.4)	2.1 (-13.6 to 12.1)	1.0 (-1.2 to 3.2)	-17.2 (-26.1 to -7.0)	-11.6 (-18.4 to -4.7)	-14.0 (-29.8 to 4.4)	30.3 (-34.3 to 131.4)	-54.4 (-85.2 to -3.0)	-47.9 (-66.5 to -24.9)	0.2 (-8.4 to 9.7)	-21.3 (-30.1 to -11.3)
Austria	Prevalence Number: 1990	1,550,836 (1,525,475–1,575,368)	420,394 (395,115–443,735)	541,490 (518,370–564,575)	155,257 (136,026–174,409)	262,907 (240,913–285,561)	436 (417–455)	29,881 (26,669–34,047)	0 (0–1)	5,336 (4,005–6,831)	0 (0–0)	18,952 (17,009–21,664)
Austria	Prevalence Number: 2015	1,412,410 (1,391,530–1,432,231)	379,078 (351,783–409,062)	513,724 (490,590–537,620)	121,029 (98,869–145,343)	213,443 (190,835–238,827)	550 (514–592)	30,201 (26,244–35,088)	0 (0–0)	4,766 (3,423–6,317)	0 (0–0)	15,549 (13,568–18,097)
Austria	Prevalence Number: Cumulative change (%)	-8.9 (-10.7 to -7.1)	-9.7 (-18.0 to -1.5)	-5.1 (-10.6 to 0.3)	-22.1 (-31.4 to -11.6)	-18.6 (-28.7 to -7.4)	26.2 (19.9–33.4)	1.5 (-14.8 to 20.1)	-31.0 (-56.2 to 2.1)	-9.6 (-33.1 to 16.8)	-9.7 (-10.3 to -9.1)	-17.5 (-31.9 to -2.5)
Austria	Prevalence Rate: 1990	83,358.9 (81,943.8–84,722.2)	23,554.5 (22,068.9–24,995.6)	28,266.1 (27,077.1–29,490.2)	8,184.0 (7,173.0–9,244.6)	14,256.3 (13,059.5–15,481.4)	25.5 (24.3–26.6)	1,619.4 (1,444.9–1,844.3)	0.1 (0.0–0.1)	287.6 (216.2–368.0)	0.0 (0.0–0.0)	1,070.1 (966.0–1,216.6)
Austria	Prevalence Rate: 2015	82,556.8 (81,235.0–83,757.4)	23,179.6 (21,465.6–25,198.4)	28,838.5 (27,597.8–30,155.6)	7,020.6 (5,721.2–8,473.9)	12,615.1 (11,287.8–14,115.1)	35.0 (32.6–37.7)	1,782.6 (1,549.4–2,073.0)	0.0 (0.0–0.1)	279.8 (200.9–372.9)	0.0 (0.0–0.0)	951.6 (832.9–1,102.2)
Austria	Prevalence Rate: Cumulative change (%)	-1.0 (-3.0 to 1.1)	-1.5 (-10.8 to 8.0)	2.1 (-3.8 to 7.6)	-14.3 (-24.8 to -2.4)	-11.3 (-22.2 to 0.8)	37.4 (30.3–45.4)	10.5 (-7.2 to 30.8)	-25.3 (-53.1 to 12.0)	-1.6 (-27.3 to 27.0)	-0.0 (-0.0 to 0.0)	-10.7 (-25.6 to 5.1)
Austria	YLDs Number: 1990	99,329 (75,799–126,141)	15,037 (10,480–20,220)	11,851 (8,199–16,436)	7,071 (4,745–9,593)	2,585 (1,838–3,490)	72 (50–99)	2,861 (1,789–4,127)	0 (0–0)	1,466 (942–2,080)	0 (0–0)	1,945 (1,491–2,431)
Austria	YLDs Number: 2015	88,023 (67,251–111,211)	13,505 (9,536–18,401)	11,094 (7,647–15,370)	5,515 (3,585–7,643)	1,911 (1,365–2,574)	91 (63–125)	3,715 (2,153–5,630)	0 (0–0)	777 (462–1,184)	0 (0–0)	1,401 (1,060–1,784)
Austria	YLDs Number: Cumulative change (%)	-11.4 (-13.7 to -8.9)	-10.1 (-18.5 to -0.4)	-6.4 (-8.6 to -4.3)	-22.0 (-31.6 to -11.7)	-26.0 (-31.1 to -19.7)	27.5 (3.3–55.4)	30.4 (5.3–68.8)	-31.0 (-56.2 to 2.1)	-46.4 (-63.1 to -26.5)	-9.8 (-18.0 to 0.3)	-27.9 (-34.9 to -21.0)
Austria	YLDs Rate: 1990	5,225.6 (3,987.2–6,650.2)	848.1 (590.8–1,143.2)	627.9 (434.4–867.1)	372.9 (250.4–504.6)	142.2 (100.9–192.2)	4.2 (2.9–5.8)	155.2 (97.1–224.1)	0.0 (0.0–0.0)	79.1 (51.1–112.0)	0.0 (0.0–0.0)	105.0 (80.4–131.1)
Austria	YLDs Rate: 2015	5,007.2 (3,826.7–6,341.1)	838.6 (589.5–1,146.3)	635.9 (440.3–878.2)	320.1 (207.8–442.0)	117.1 (84.0–157.2)	5.8 (4.0–7.9)	219.5 (127.1–332.9)	0.0 (0.0–0.0)	45.7 (27.3–69.8)	0.0 (0.0–0.0)	82.4 (62.3–104.9)
Austria	YLDs Rate: Cumulative change (%)	-4.2 (-6.9 to -1.3)	-0.9 (-11.3 to 10.3)	1.3 (-1.0 to 3.5)	-14.2 (-24.9 to -2.4)	-17.5 (-23.3 to -10.0)	38.9 (12.3–70.7)	42.0 (14.7–83.7)	-25.3 (-53.0 to 12.0)	-41.6 (-59.8 to -20.1)	0.8 (-8.9 to 12.5)	-21.5 (-28.9 to -14.0)
Belgium	Prevalence Number: 1990	1,913,977 (1,884,244–1,943,683)	440,091 (397,241–483,622)	712,387 (685,960–740,017)	200,073 (173,635–228,077)	311,731 (284,746–343,666)	725 (663–787)	42,659 (38,112–48,544)	0 (0–1)	7,694 (5,976–9,566)	0 (0–0)	18,104 (16,023–21,146)
Belgium	Prevalence Number: 2015	1,981,245 (1,943,022–2,020,198)	469,473 (419,192–514,114)	745,344 (715,197–774,835)	136,237 (113,245–159,748)	284,010 (248,184–323,898)	787 (720–857)	41,851 (37,359–46,771)	1 (0–1)	8,223 (6,340–10,055)	0 (0–0)	20,543 (17,855–24,228)
Belgium	Prevalence Number: Cumulative change (%)	3.5 (1.1–6.1)	7.0 (-6.9 to 19.1)	4.7 (-0.3 to 10.0)	-31.9 (-38.3 to -25.2)	-8.7 (-22.0 to 6.4)	8.6 (4.8–12.8)	-1.4 (-16.4 to 14.5)	78.4 (26.4–143.2)	8.3 (-19.2 to 38.5)	3.8 (2.9–4.8)	14.2 (-6.0 to 36.6)
Belgium	Prevalence Rate: 1990	77,254.8 (75,985.7–78,525.4)	18,521.0 (16,689.1–20,478.0)	28,042.7 (26,984.9–29,117.4)	7,950.9 (6,893.3–9,065.2)	12,707.5 (11,604.2–14,005.9)	31.5 (28.7–34.2)	1,737.7 (1,551.5–1,977.6)	0.0 (0.0–0.0)	314.6 (244.8–392.6)	0.0 (0.0–0.0)	764.8 (680.8–888.7)
Belgium	Prevalence Rate: 2015	78,013.3 (76,457.1–79,596.1)	19,090.4 (17,001.7–20,893.6)	28,677.1 (27,536.7–29,820.0)	5,339.5 (4,427.6–6,284.6)	11,250.0 (9,833.0–12,824.7)	32.0 (29.3–34.9)	1,655.7 (1,478.4–1,850.7)	0.0 (0.0–0.1)	325.4 (250.6–397.8)	0.0 (0.0–0.0)	825.3 (719.1–970.7)
Belgium	Prevalence Rate: Cumulative change (%)	1.0 (-1.4 to 3.6)	3.4 (-10.5 to 15.7)	2.3 (-2.5 to 7.4)	-32.9 (-39.2 to -26.3)	-11.2 (-24.2 to 3.3)	1.7 (-1.9 to 5.7)	-4.3 (-18.8 to 11.2)	67.1 (17.4–130.0)	4.8 (-22.0 to 34.0)	-0.0 (-0.0 to 0.0)	8.5 (-10.2 to 29.6)
Belgium	YLDs Number: 1990	123,783 (93,875–156,779)	15,262 (10,382–21,312)	15,342 (10,572–21,204)	9,126 (6,169–12,465)	2,419 (1,702–3,273)	120 (83–163)	3,270 (2,209–4,516)	0 (0–0)	2,117 (1,393–2,945)	0 (0–0)	1,862 (1,438–2,341)
Belgium	YLDs Number: 2015	121,633 (92,675–154,420)	16,501 (11,464–22,994)	15,952 (10,986–22,167)	6,215 (4,111–8,576)	2,425 (1,727–3,281)	130 (89–178)	4,831 (2,909–7,097)	0 (0–0)	1,338 (840–1,913)	0 (0–0)	1,791 (1,372–2,296)
Belgium	YLDs Number: Cumulative change (%)	-1.7 (-4.7 to 1.1)	8.7 (-12.0 to 24.4)	4.0 (1.5–6.2)	-31.9 (-38.4 to -25.1)	0.4 (-7.6 to 7.3)	8.9 (-12.0 to 31.5)	46.8 (20.1–75.4)	78.4 (26.6–143.2)	-35.9 (-55.9 to -12.3)	3.9 (-9.6 to 16.0)	-3.7 (-14.3 to 8.4)
Belgium	YLDs Rate: 1990	4,901.4 (3,714.1–6,221.2)	642.9 (436.6–897.9)	612.1 (424.1–846.2)	362.8 (245.4–493.2)	99.9 (70.2–135.2)	5.2 (3.6–7.1)	133.3 (89.9–184.1)	0.0 (0.0–0.0)	86.7 (56.9–121.1)	0.0 (0.0–0.0)	75.6 (58.6–95.1)
Belgium	YLDs Rate: 2015	4,719.0 (3,600.1–6,008.5)	677.2 (470.5–942.8)	620.9 (427.8–862.3)	243.7 (161.5–337.7)	99.2 (70.7–133.8)	5.3 (3.6–7.3)	191.2 (115.1–281.2)	0.0 (0.0–0.0)	53.0 (33.2–75.8)	0.0 (0.0–0.0)	70.8 (54.4–90.7)
Belgium	YLDs Rate: Cumulative change (%)	-3.7 (-6.8 to -0.8)	5.9 (-14.4 to 21.7)	1.4 (-0.9 to 3.6)	-32.9 (-39.3 to -26.0)	-0.6 (-8.8 to 6.5)	1.9 (-17.6 to 22.8)	42.6 (16.7–70.1)	67.1 (17.7–129.8)	-38.0 (-57.1 to -15.0)	1.2 (-12.0 to 12.8)	-6.2 (-16.5 to 5.4)
Cyprus	Prevalence Number: 1990	199,250 (196,033–202,431)	47,355 (42,774–53,480)	67,384 (64,560–70,539)	22,001 (18,377–25,991)	55,803 (53,270–58,332)	82 (75–91)	3,556 (3,114–4,095)	0 (0–0)	633 (483–808)	0 (0–0)	2,663 (2,330–3,066)
Cyprus	Prevalence Number: 2015	162,887 (160,562–165,121)	37,380 (35,382–39,607)	59,439 (56,782–62,265)	14,321 (11,859–16,889)	43,672 (41,674–45,758)	47 (43–51)	2,844 (2,494–3,321)	0 (0–0)	469 (347–608)	0 (0–0)	1,992 (1,743–2,285)
Cyprus	Prevalence Number: Cumulative change (%)	-18.2 (-19.8 to -16.6)	-20.8 (-28.2 to -13.4)	-11.7 (-17.0 to -6.3)	-34.8 (-39.6 to -29.6)	-21.7 (-26.4 to -16.9)	-42.9 (-46.6 to -40.2)	-19.5 (-33.2 to -3.6)	-77.4 (-87.0 to -64.5)	-25.0 (-45.2 to -2.1)	-24.5 (-27.2 to -22.0)	-24.9 (-35.7 to -14.1)
Cyprus	Prevalence Rate: 1990	84,368.0 (82,967.5–85,738.3)	20,521.4 (18,512.2–23,244.5)	27,917.4 (26,760.0–29,208.5)	9,233.4 (7,697.3–10,940.5)	23,731.4 (22,659.8–24,804.6)	36.9 (33.6–41.1)	1,512.9 (1,326.1–1,742.0)	0.1 (0.1–0.2)	269.8 (206.3–343.0)	0.0 (0.0–0.0)	1,165.3 (1,021.9–1,338.9)
Cyprus	Prevalence Rate: 2015	84,169.5 (82,886.2–85,434.1)	21,245.2 (20,011.8–22,599.5)	28,555.6 (27,342.9–29,843.9)	7,060.6 (5,848.7–8,404.3)	23,001.8 (21,944.9–24,084.4)	30.9 (28.5–33.8)	1,498.3 (1,314.2–1,746.7)	0.0 (0.0–0.0)	245.9 (183.7–317.8)	0.0 (0.0–0.0)	1,160.6 (1,027.6–1,319.5)
Cyprus	Prevalence Rate: Cumulative change (%)	-0.2 (-2.3 to 1.8)	3.9 (-5.6 to 13.5)	2.4 (-3.5 to 8.4)	-23.5 (-29.1 to -17.2)	-3.0 (-8.8 to 2.9)	-16.1 (-21.2 to -11.9)	-0.4 (-17.2 to 19.4)	-68.5 (-82.6 to -49.9)	-7.8 (-32.3 to 19.3)	0.0 (-0.0 to 0.0)	0.0 (-13.8 to 13.5)
Cyprus	YLDs Number: 1990	12,426 (9,434–15,758)	1,600 (1,083–2,224)	1,469 (1,014–2,040)	1,002 (658–1,379)	731 (523–967)	13 (9–18)	352 (193–604)	0 (0–0)	172 (114–244)	0 (0–0)	306 (235–392)
Cyprus	YLDs Number: 2015	10,140 (7,691–12,819)	1,291 (917–1,751)	1,256 (863–1,749)	651 (433–897)	520 (374–698)	7 (5–10)	330 (177–564)	0 (0–0)	76 (44–114)	0 (0–0)	226 (172–292)
Cyprus	YLDs Number: Cumulative change (%)	-18.4 (-21.2 to -15.3)	-18.8 (-28.9 to -7.9)	-14.5 (-16.9 to -12.3)	-34.9 (-39.9 to -29.5)	-28.8 (-33.1 to -24.0)	-42.5 (-52.4 to -31.9)	0.3 (-48.5 to 71.9)	-77.3 (-86.9 to -64.4)	-55.2 (-69.5 to -38.1)	-22.6 (-29.7 to -15.4)	-26.0 (-32.9 to -18.1)
Cyprus	YLDs Rate: 1990	5,188.0 (3,939.5–6,584.4)	696.7 (472.0–970.3)	615.2 (425.1–852.5)	420.6 (275.7–580.6)	312.6 (223.8–412.9)	6.1 (4.3–8.4)	150.1 (82.4–257.7)	0.0 (0.0–0.0)	73.5 (48.5–104.3)	0.0 (0.0–0.0)	130.4 (100.1–166.6)
Cyprus	YLDs Rate: 2015	4,976.3 (3,767.1–6,295.3)	741.1 (522.1–1,004.3)	625.3 (431.6–866.5)	321.7 (213.3–443.2)	281.4 (202.1–377.5)	5.1 (3.6–7.0)	174.5 (93.6–297.9)	0.0 (0.0–0.0)	40.0 (23.6–60.7)	0.0 (0.0–0.0)	118.6 (89.8–153.5)
Cyprus	YLDs Rate: Cumulative change (%)	-4.1 (-7.5 to -0.4)	7.1 (-6.1 to 21.4)	1.6 (-0.7 to 3.8)	-23.4 (-29.2 to -17.1)	-9.9 (-15.7 to -3.9)	-15.5 (-30.0 to 0.2)	24.3 (-36.5 to 111.9)	-68.4 (-82.6 to -49.9)	-44.9 (-62.3 to -23.4)	1.4 (-6.9 to 10.5)	-9.0 (-17.3 to 0.6)
Denmark	Prevalence Number: 1990	1,034,385 (1,013,061–1,052,439)	239,128 (214,367–261,272)	390,136 (375,046–407,607)	89,688 (75,264–106,693)	192,619 (171,436–227,582)	336 (307–365)	24,047 (21,628–27,215)	1 (0–2)	2,686 (2,071–3,288)	0 (0–0)	10,828 (9,589–12,462)
Denmark	Prevalence Number: 2015	1,117,538 (1,097,766–1,136,210)	291,278 (269,771–313,068)	424,984 (407,201–443,035)	81,124 (67,289–96,818)	189,326 (164,913–230,736)	380 (350–415)	22,517 (19,779–25,849)	0 (0–0)	3,389 (2,628–4,219)	0 (0–0)	10,203 (8,868–12,085)
Denmark	Prevalence Number: Cumulative change (%)	8.1 (5.6–10.8)	22.2 (6.5–37.7)	9.0 (3.0–15.0)	-9.3 (-20.3 to 4.5)	-1.1 (-19.7 to 22.7)	13.2 (7.6–19.5)	-5.9 (-20.6 to 12.2)	-62.1 (-72.9 to -48.4)	27.7 (-2.8 to 67.3)	10.6 (9.2–12.3)	-5.2 (-20.0 to 13.0)
Denmark	Prevalence Rate: 1990	82,548.0 (80,733.9–84,063.0)	20,266.4 (18,017.9–22,328.2)	29,685.8 (28,604.0–30,908.4)	7,107.5 (5,940.7–8,473.3)	15,544.3 (13,844.6–18,377.4)	29.7 (27.0–32.4)	1,941.4 (1,746.8–2,194.0)	0.1 (0.1–0.2)	213.8 (165.9–262.4)	0.0 (0.0–0.0)	897.5 (798.8–1,029.1)
Denmark	Prevalence Rate: 2015	82,637.2 (81,097.5–84,105.1)	22,656.1 (20,788.2–24,693.6)	30,295.2 (29,012.7–31,528.3)	5,883.6 (4,854.5–7,044.3)	14,179.1 (12,354.5–17,279.8)	32.0 (29.4–35.2)	1,685.1 (1,477.5–1,936.2)	0.0 (0.0–0.1)	250.1 (195.0–310.7)	0.0 (0.0–0.0)	815.0 (714.4–955.8)
Denmark	Prevalence Rate: Cumulative change (%)	0.1 (-2.3 to 2.9)	12.2 (-3.4 to 28.1)	2.1 (-3.3 to 7.5)	-17.0 (-27.0 to -4.5)	-8.2 (-25.5 to 14.1)	8.1 (2.4–14.4)	-12.8 (-26.3 to 4.0)	-63.5 (-74.4 to -49.6)	18.4 (-9.6 to 54.9)	0.0 (-0.0 to 0.0)	-8.7 (-22.6 to 7.7)
Denmark	YLDs Number: 1990	66,095 (50,376–83,839)	7,647 (5,260–10,727)	9,365 (6,527–12,937)	4,087 (2,718–5,642)	3,026 (2,009–4,247)	55 (38–76)	2,236 (1,414–3,180)	0 (0–0)	740 (484–1,036)	0 (0–0)	1,042 (799–1,320)
Denmark	YLDs Number: 2015	69,436 (53,020–87,645)	10,454 (7,285–14,312)	10,293 (7,172–14,128)	3,692 (2,433–5,141)	2,320 (1,492–3,246)	63 (43–86)	2,596 (1,493–4,463)	0 (0–0)	550 (348–811)	0 (0–0)	867 (655–1,110)
Denmark	YLDs Number: Cumulative change (%)	5.1 (1.8–8.5)	37.6 (13.8–56.9)	9.9 (6.5–13.4)	-9.3 (-20.6 to 4.6)	-23.3 (-30.9 to -14.5)	13.7 (-8.2 to 37.4)	16.8 (-12.3 to 88.6)	-62.0 (-72.9 to -48.4)	-24.5 (-48.1 to 5.6)	11.9 (-3.9 to 31.0)	-16.7 (-25.7 to -7.7)
Denmark	YLDs Rate: 1990	5,080.4 (3,875.3–6,458.8)	660.1 (451.4–920.6)	737.5 (515.8–1,008.6)	324.2 (215.5–448.1)	248.3 (166.3–349.3)	4.9 (3.4–6.8)	180.7 (114.4–257.0)	0.0 (0.0–0.0)	59.0 (39.0–81.8)	0.0 (0.0–0.0)	83.9 (64.2–106.7)
Denmark	YLDs Rate: 2015	4,978.9 (3,797.3–6,290.0)	816.4 (567.0–1,129.9)	752.4 (527.8–1,033.1)	267.9 (176.7–372.3)	176.2 (113.6–246.6)	5.3 (3.7–7.3)	194.5 (112.0–334.3)	0.0 (0.0–0.0)	40.7 (25.7–59.7)	0.0 (0.0–0.0)	64.5 (48.6–82.7)

© 2017 Global Burden of Disease Child and Adolescent Health Collaboration. JAMA Pediatrics.

eTable 3. Prevalent Cases, Rates (per 100,000 population), Years Lived with Disability (YLDs), and Cumulative Percent Change with 95% Uncertainty Interval (UI) for the Top 10 Global Causes of YLDs in Children and Adolescents in 195 Countries and Territories, Aged 0 to 19 Years, Both Sexes, 1990 and 2015. *Note: best viewed by enlarging in browser.*

Location	Measure	All causes	Iron-deficiency anemia	Skin and subcutaneous diseases	Asthma	Hemoglobinopathies and hemolytic anemias	Diarrheal diseases	Congenital anomalies	Protein-energy malnutrition	Epilepsy	Malaria	Neonatal preterm birth complications
Denmark	YLDs Rate: Cumulative change (%)	-2.0 (-5.6 to 1.4)	24.6 (0.4–44.3)	2.0 (-1.2 to 5.3)	-17.0 (-27.4 to -4.4)	-29.1 (-36.4 to -20.5)	8.5 (-12.8 to 32.1)	8.3 (-18.8 to 75.2)	-63.4 (-74.4 to -49.6)	-30.0 (-52.2 to -2.7)	-0.4 (-15.5 to 17.3)	-23.0 (-31.1 to -14.8)
Finland	Prevalence Number: 1990	1,055,580 (1,041,238–1,072,029)	328,360 (301,406–360,557)	373,139 (357,862–388,997)	136,393 (109,974–163,106)	190,283 (169,363–225,884)	419 (388–453)	21,096 (18,560–24,254)	0 (0–0)	3,049 (2,379–3,747)	0 (0–0)	12,018 (10,665–13,865)
Finland	Prevalence Number: 2015	1,012,657 (998,190–1,026,870)	285,131 (266,244–305,890)	372,553 (356,919–388,500)	117,838 (96,229–140,767)	166,942 (143,438–207,429)	315 (297–333)	19,400 (17,148–22,580)	0 (0–0)	3,234 (2,477–4,043)	0 (0–0)	9,618 (8,349–11,311)
Finland	Prevalence Number: Cumulative change (%)	-4.1 (-6.0 to -2.2)	-13.0 (-21.5 to -5.5)	-0.1 (-5.4 to 5.1)	-13.1 (-28.7 to 1.2)	-11.7 (-28.6 to 10.4)	-24.8 (-28.1 to -21.6)	-7.5 (-22.6 to 10.4)	-44.6 (-59.2 to -26.2)	7.5 (-19.4 to 39.6)	-8.3 (-9.1 to -7.3)	-19.6 (-33.2 to -3.5)
Finland	Prevalence Rate: 1990	82,901.7 (81,777.4–84,233.6)	26,005.3 (23,769.7–28,692.1)	29,165.3 (27,961.3–30,402.2)	10,636.7 (8,612.3–12,716.5)	14,996.9 (13,345.8–17,804.5)	34.3 (31.7–37.1)	1,663.6 (1,463.3–1,912.3)	0.0 (0.0–0.0)	240.1 (187.4–294.8)	0.0 (0.0–0.0)	969.0 (861.4–1,113.9)
Finland	Prevalence Rate: 2015	83,539.8 (82,262.4–84,783.1)	24,465.1 (22,691.1–26,319.2)	29,696.3 (28,466.5–30,980.7)	9,752.8 (7,934.8–11,707.8)	13,906.8 (11,970.4–17,260.0)	27.7 (26.1–29.3)	1,612.9 (1,425.4–1,874.2)	0.0 (0.0–0.0)	265.5 (203.6–332.8)	0.0 (0.0–0.0)	827.8 (722.0–969.1)
Finland	Prevalence Rate: Cumulative change (%)	0.8 (-1.3 to 2.8)	-5.7 (-15.1 to 2.5)	1.9 (-3.6 to 7.2)	-7.8 (-24.4 to 7.5)	-6.7 (-24.6 to 16.5)	-19.1 (-22.8 to -15.6)	-2.5 (-18.5 to 16.3)	-42.5 (-57.8 to -23.4)	12.0 (-16.0 to 45.1)	0.0 (-0.0 to 0.0)	-14.1 (-28.3 to 2.5)
Finland	YLDs Number: 1990	68,100 (51,430–86,668)	12,031 (8,391–16,347)	8,978 (6,236–12,310)	6,204 (4,074–8,659)	2,227 (1,500–3,088)	69 (48–95)	1,416 (904–1,982)	0 (0–0)	836 (553–1,181)	0 (0–0)	1,226 (930–1,563)
Finland	YLDs Number: 2015	63,836 (48,508–80,771)	10,184 (7,139–13,807)	8,750 (6,026–12,096)	5,368 (3,495–7,411)	1,939 (1,274–2,678)	52 (36–71)	2,126 (1,264–3,182)	0 (0–0)	526 (329–770)	0 (0–0)	824 (624–1,040)
Finland	YLDs Number: Cumulative change (%)	-6.2 (-9.4 to -3.3)	-15.1 (-25.2 to -6.9)	-2.6 (-5.0 to -0.3)	-13.0 (-28.5 to 1.1)	-13.1 (-20.4 to -6.6)	-24.4 (-38.6 to -9.0)	49.5 (21.7–79.5)	-44.5 (-59.1 to -26.3)	-36.1 (-56.7 to -11.8)	-10.9 (-19.0 to -2.6)	-32.6 (-40.0 to -24.7)
Finland	YLDs Rate: 1990	5,317.4 (4,015.5–6,769.2)	946.3 (657.8–1,290.7)	704.4 (489.3–965.5)	483.9 (318.1–673.7)	173.6 (117.4–240.5)	5.7 (4.0–7.8)	111.7 (71.3–156.3)	0.0 (0.0–0.0)	65.9 (43.6–92.9)	0.0 (0.0–0.0)	96.5 (73.0–123.1)
Finland	YLDs Rate: 2015	5,146.5 (3,916.9–6,535.1)	889.1 (621.3–1,208.9)	712.9 (490.4–980.0)	444.5 (288.1–613.1)	168.1 (110.1–232.7)	4.6 (3.2–6.3)	177.0 (105.4–264.6)	0.0 (0.0–0.0)	43.2 (27.2–63.5)	0.0 (0.0–0.0)	68.3 (51.7–86.4)
Finland	YLDs Rate: Cumulative change (%)	-3.2 (-6.6 to -0.1)	-5.8 (-17.2 to 4.0)	1.2 (-1.0 to 3.3)	-7.6 (-24.2 to 7.5)	-3.3 (-11.9 to 4.3)	-18.8 (-34.1 to -2.3)	57.8 (28.2–89.2)	-42.4 (-57.8 to -23.4)	-33.4 (-54.9 to -7.8)	-0.8 (-9.9 to 8.6)	-28.9 (-36.8 to -20.6)
France	Prevalence Number: 1990	12,989,335 (12,846,929–13,139,527)	3,413,301 (3,214,094–3,667,212)	4,445,381 (4,267,898–4,616,223)	1,457,786 (1,285,065–1,631,130)	1,721,112 (1,624,394–1,833,876)	7,038 (6,595–7,518)	167,021 (150,958–186,257)	11 (7–16)	53,326 (41,998–65,002)	0 (0–0)	141,816 (124,664–164,111)
France	Prevalence Number: 2015	12,716,535 (12,506,233–12,904,459)	3,484,415 (3,321,827–3,812,347)	4,371,304 (4,204,889–4,529,747)	1,005,000 (828,164–1,188,750)	1,454,425 (1,371,015–1,544,430)	6,497 (6,082–6,943)	187,318 (166,222–212,551)	8 (5–12)	57,275 (44,475–71,477)	0 (0–0)	131,570 (113,520–154,435)
France	Prevalence Number: Cumulative change (%)	-2.1 (-4.0 to -0.4)	2.3 (-8.6 to 14.8)	-1.6 (-6.5 to 3.5)	-31.1 (-39.6 to -22.5)	-15.4 (-21.9 to -8.6)	-7.7 (-10.4 to -4.9)	12.6 (-3.6 to 31.1)	-27.1 (-48.2 to 0.8)	8.7 (-18.1 to 41.5)	1.4 (0.6–2.1)	-6.7 (-22.6 to 10.5)
France	Prevalence Rate: 1990	82,231.6 (81,274.6–83,268.7)	22,291.2 (20,986.3–24,022.2)	27,437.9 (26,378.8–28,481.6)	9,054.9 (7,943.1–10,175.5)	11,002.4 (10,388.9–11,725.6)	47.9 (44.8–51.2)	1,067.7 (964.9–1,192.1)	0.1 (0.1–0.1)	339.7 (267.4–413.8)	0.0 (0.0–0.0)	942.3 (831.6–1,085.8)
France	Prevalence Rate: 2015	81,753.8 (80,389.7–82,992.7)	22,542.0 (21,465.1–24,711.2)	27,956.8 (26,890.6–28,973.2)	6,423.7 (5,294.6–7,591.9)	9,378.6 (8,841.0–9,960.0)	43.1 (40.3–46.1)	1,208.6 (1,072.4–1,371.1)	0.1 (0.0–0.1)	369.1 (287.0–460.3)	0.0 (0.0–0.0)	866.6 (749.5–1,014.1)
France	Prevalence Rate: Cumulative change (%)	-0.6 (-2.5 to 1.2)	1.3 (-9.8 to 13.9)	1.9 (-3.1 to 7.3)	-29.1 (-37.7 to -20.4)	-14.7 (-21.1 to -7.8)	-10.0 (-12.7 to -7.3)	13.6 (-2.7 to 32.3)	-28.7 (-49.6 to -1.0)	9.9 (-17.3 to 43.4)	-0.0 (-0.0 to 0.0)	-7.6 (-22.7 to 9.1)
France	YLDs Number: 1990	813,151 (617,568–1,038,956)	121,365 (85,508–165,007)	91,677 (62,760–128,185)	66,322 (44,709–89,422)	21,106 (15,110–28,043)	1,162 (814–1,562)	13,238 (9,117–18,203)	1 (0–2)	14,677 (9,799–20,210)	0 (0–0)	13,901 (10,751–17,385)
France	YLDs Number: 2015	749,370 (567,721–949,899)	127,317 (89,404–172,721)	90,546 (61,988–126,603)	45,828 (30,045–63,703)	16,523 (11,860–22,119)	1,076 (762–1,435)	18,742 (11,718–27,649)	1 (0–1)	9,327 (5,940–13,667)	0 (0–0)	10,881 (8,223–13,864)
France	YLDs Number: Cumulative change (%)	-7.8 (-10.4 to -4.9)	5.2 (-9.3 to 23.3)	-1.2 (-3.3 to 0.9)	-31.0 (-39.5 to -22.5)	-21.7 (-26.7 to -16.3)	-6.9 (-20.4 to 7.5)	41.1 (14.8–76.1)	-27.1 (-48.1 to 0.4)	-35.6 (-55.3 to -10.6)	1.9 (-6.5 to 11.6)	-21.7 (-29.9 to -13.4)
France	YLDs Rate: 1990	5,053.6 (3,840.3–6,465.8)	799.3 (562.4–1,084.9)	572.5 (394.0–797.5)	412.1 (277.7–556.6)	135.7 (97.4–180.5)	7.9 (5.5–10.6)	84.7 (58.3–116.5)	0.0 (0.0–0.0)	93.6 (62.8–129.5)	0.0 (0.0–0.0)	88.6 (68.7–110.7)
France	YLDs Rate: 2015	4,793.9 (3,631.5–6,080.6)	819.4 (574.1–1,110.3)	580.5 (397.6–811.5)	293.0 (192.1–406.8)	105.4 (75.6–141.2)	7.1 (5.0–9.5)	121.0 (75.7–178.5)	0.0 (0.0–0.0)	60.1 (38.3–88.0)	0.0 (0.0–0.0)	70.1 (53.0–89.3)
France	YLDs Rate: Cumulative change (%)	-5.1 (-7.9 to -2.0)	2.8 (-11.9 to 21.1)	1.4 (-0.8 to 3.6)	-29.0 (-37.5 to -20.2)	-22.3 (-27.5 to -17.0)	-9.3 (-22.5 to 5.2)	42.3 (15.8–77.3)	-28.6 (-49.5 to -1.1)	-34.9 (-55.2 to -9.8)	-0.1 (-8.5 to 9.7)	-20.9 (-29.1 to -12.7)
Germany	Prevalence Number: 1990	14,002,199 (13,860,752–14,150,973)	4,359,551 (4,098,392–4,727,710)	4,934,964 (4,789,209–5,084,700)	1,494,500 (1,277,817–1,733,627)	1,934,833 (1,745,324–2,137,821)	5,236 (4,934–5,530)	248,957 (221,643–281,693)	3 (2–5)	53,287 (41,533–66,123)	0 (0–0)	151,237 (132,791–176,307)
Germany	Prevalence Number: 2015	11,655,679 (11,478,344–11,842,336)	3,589,915 (3,351,647–3,951,732)	4,266,800 (4,126,760–4,404,900)	803,674 (662,075–953,744)	1,402,504 (1,202,118–1,627,464)	4,859 (4,568–5,163)	278,172 (242,925–328,276)	2 (1–4)	45,312 (35,092–57,050)	0 (0–0)	113,183 (98,471–131,985)
Germany	Prevalence Number: Cumulative change (%)	-16.8 (-18.2 to -15.2)	-17.6 (-24.7 to -10.2)	-13.5 (-17.1 to -10.0)	-46.2 (-51.4 to -40.7)	-27.3 (-38.9 to -15.1)	-7.2 (-10.6 to -3.5)	12.3 (-6.0 to 33.4)	-29.6 (-50.0 to -2.6)	-13.8 (-34.8 to 11.5)	-18.7 (-20.2 to -17.2)	-24.7 (-36.9 to -10.2)
Germany	Prevalence Rate: 1990	81,682.4 (80,828.3–82,596.1)	26,025.8 (24,443.5–28,180.4)	28,268.2 (27,450.4–29,116.8)	8,640.0 (7,383.8–10,035.6)	11,366.4 (10,249.7–12,556.0)	32.1 (30.2–33.9)	1,461.8 (1,301.1–1,653.4)	0.0 (0.0–0.0)	312.2 (243.0–387.5)	0.0 (0.0–0.0)	916.3 (807.7–1,062.5)
Germany	Prevalence Rate: 2015	81,121.9 (79,788.5–82,525.5)	25,704.2 (23,897.4–28,488.0)	28,821.2 (27,908.1–29,712.1)	5,510.1 (4,533.7–6,555.8)	9,837.5 (8,436.9–11,406.2)	36.5 (34.3–38.8)	1,953.0 (1,706.8–2,307.7)	0.0 (0.0–0.0)	316.9 (245.5–397.8)	0.0 (0.0–0.0)	826.1 (722.0–957.3)
Germany	Prevalence Rate: Cumulative change (%)	-0.7 (-2.6 to 1.3)	-1.1 (-10.4 to 8.2)	2.0 (-2.2 to 6.2)	-36.2 (-42.4 to -29.6)	-13.2 (-27.1 to 1.4)	13.9 (9.7–18.5)	34.2 (12.5–59.7)	-15.1 (-40.8 to 19.0)	2.9 (-21.9 to 33.5)	0.0 (-0.0 to 0.0)	-9.4 (-23.6 to 7.5)
Germany	YLDs Number: 1990	915,560 (693,447–1,163,928)	160,052 (113,308–219,686)	103,794 (71,776–144,148)	68,007 (45,331–91,151)	8,897 (6,417–12,124)	863 (595–1,178)	16,197 (10,988–22,217)	0 (0–0)	14,672 (9,792–20,391)	0 (0–0)	15,580 (12,041–19,749)
Germany	YLDs Number: 2015	738,833 (561,225–933,501)	133,817 (94,331–185,278)	88,751 (60,635–123,610)	36,609 (24,014–50,313)	6,926 (4,878–9,504)	804 (552–1,085)	28,022 (16,394–42,608)	0 (0–0)	7,390 (4,581–10,773)	0 (0–0)	10,112 (7,682–12,845)
Germany	YLDs Number: Cumulative change (%)	-19.3 (-21.6 to -16.8)	-16.3 (-25.5 to -7.2)	-14.5 (-16.5 to -12.6)	-46.2 (-51.7 to -40.1)	-22.0 (-30.6 to -13.4)	-5.9 (-24.8 to 16.7)	71.6 (31.8–114.0)	-29.5 (-49.9 to -2.7)	-48.8 (-65.5 to -27.8)	-15.7 (-23.0 to -8.6)	-35.1 (-41.5 to -28.0)
Germany	YLDs Rate: 1990	5,269.7 (3,989.1–6,710.3)	960.3 (678.3–1,319.1)	600.5 (417.3–830.0)	393.3 (262.9–527.4)	53.1 (38.3–72.1)	5.3 (3.6–7.2)	95.2 (64.6–130.6)	0.0 (0.0–0.0)	86.0 (57.3–119.6)	0.0 (0.0–0.0)	91.2 (70.4–115.8)
Germany	YLDs Rate: 2015	5,029.1 (3,809.3–6,372.6)	955.7 (672.0–1,333.3)	609.8 (417.6–842.3)	251.1 (164.7–345.9)	48.3 (34.1–66.5)	6.0 (4.2–8.1)	196.9 (115.3–299.3)	0.0 (0.0–0.0)	51.7 (32.2–75.1)	0.0 (0.0–0.0)	70.6 (53.7–89.8)
Germany	YLDs Rate: Cumulative change (%)	-4.5 (-7.6 to -1.4)	-0.3 (-12.4 to 11.4)	1.5 (-0.8 to 3.7)	-36.1 (-42.7 to -29.1)	-9.0 (-19.3 to 1.8)	15.5 (-7.8 to 43.6)	105.3 (57.5–156.2)	-15.1 (-40.6 to 18.6)	-38.9 (-59.0 to -13.7)	3.3 (-5.5 to 13.2)	-22.6 (-30.3 to -14.4)
Greece	Prevalence Number: 1990	2,291,126 (2,274,017–2,309,510)	565,978 (531,002–610,340)	771,690 (741,583–802,989)	151,053 (126,844–178,364)	455,245 (434,684–475,731)	434 (414–454)	41,706 (36,275–49,188)	2 (1–3)	6,512 (4,844–8,537)	0 (0–0)	25,363 (22,116–29,793)
Greece	Prevalence Number: 2015	1,727,973 (1,699,376–1,754,675)	465,008 (434,468–501,261)	577,637 (555,173–599,821)	127,972 (106,639–151,091)	279,572 (267,424–293,096)	309 (295–322)	31,468 (27,470–36,780)	0 (0–0)	4,850 (3,517–6,430)	0 (0–0)	18,118 (15,649–21,571)
Greece	Prevalence Number: Cumulative change (%)	-24.6 (-25.9 to -23.4)	-17.7 (-26.6 to -8.8)	-25.1 (-28.9 to -21.2)	-15.0 (-23.6 to 1.3)	-38.5 (-42.4 to -34.5)	-28.7 (-30.8 to -26.6)	-24.0 (-37.9 to -8.0)	-89.4 (-91.9 to -86.8)	-24.6 (-44.5 to -1.4)	-18.2 (-20.5 to -15.4)	-28.2 (-40.8 to -12.5)
Greece	Prevalence Rate: 1990	83,312.8 (82,598.6–84,038.7)	21,990.4 (20,565.4–23,866.3)	26,777.1 (25,740.5–27,845.1)	5,373.8 (4,533.2–6,348.9)	16,888.2 (16,134.8–17,652.2)	19.0 (18.1–20.0)	1,549.3 (1,347.9–1,823.4)	0.1 (0.1–0.2)	239.3 (179.5–311.5)	0.0 (0.0–0.0)	1,033.3 (905.6–1,201.0)
Greece	Prevalence Rate: 2015	82,613.2 (81,215.3–83,940.3)	22,605.4 (21,009.0–24,552.5)	27,302.4 (26,246.6–28,351.9)	6,044.1 (5,030.4–7,133.9)	13,455.0 (12,870.9–14,104.6)	15.9 (15.2–16.6)	1,515.4 (1,321.5–1,770.4)	0.0 (0.0–0.0)	232.6 (168.6–307.8)	0.0 (0.0–0.0)	924.0 (801.4–1,093.8)
Greece	Prevalence Rate: Cumulative change (%)	-0.8 (-2.7 to 0.9)	3.0 (-8.7 to 16.0)	2.0 (-3.0 to 7.4)	12.8 (1.6–33.1)	-20.3 (-25.2 to -15.1)	-16.6 (-19.2 to -14.0)	-1.4 (-19.3 to 19.4)	-87.7 (-91.0 to -84.4)	-1.6 (-27.8 to 29.0)	0.0 (-0.0 to 0.0)	-10.2 (-24.7 to 7.7)
Greece	YLDs Number: 1990	138,752 (105,298–176,309)	19,533 (13,473–26,986)	14,793 (10,000–20,785)	6,885 (4,523–9,480)	5,614 (4,083–7,422)	71 (49–98)	2,867 (1,904–3,988)	0 (0–0)	1,769 (1,111–2,577)	0 (0–0)	3,024 (2,327–3,803)
Greece	YLDs Number: 2015	99,740 (75,842–125,994)	16,834 (11,712–22,784)	11,182 (7,612–15,802)	5,834 (3,933–8,078)	3,665 (2,619–4,919)	51 (35–70)	2,572 (1,635–3,697)	0 (0–0)	789 (466–1,193)	0 (0–0)	1,691 (1,289–2,159)
Greece	YLDs Number: Cumulative change (%)	-28.1 (-30.1 to -25.9)	-13.5 (-24.2 to -0.9)	-24.4 (-26.1 to -22.5)	-14.9 (-24.1 to 1.2)	-34.7 (-40.6 to -29.0)	-27.6 (-43.0 to -10.4)	-10.4 (-21.2 to 4.6)	-89.4 (-91.8 to -86.8)	-54.7 (-69.3 to -36.8)	-19.2 (-28.5 to -9.1)	-44.0 (-50.1 to -37.8)
Greece	YLDs Rate: 1990	4,868.2 (3,688.4–6,183.7)	754.9 (522.1–1,049.9)	522.5 (354.6–734.2)	245.1 (161.2–337.0)	203.6 (148.0–268.5)	3.2 (2.2–4.3)	106.6 (70.9–148.4)	0.0 (0.0–0.0)	65.1 (41.0–94.3)	0.0 (0.0–0.0)	111.4 (85.9–140.1)
Greece	YLDs Rate: 2015	4,719.0 (3,582.5–5,961.8)	813.9 (561.7–1,108.9)	530.2 (361.2–747.7)	275.6 (185.7–381.0)	174.2 (124.2–233.0)	2.6 (1.8–3.6)	123.9 (78.7–178.2)	0.0 (0.0–0.0)	37.9 (22.3–57.2)	0.0 (0.0–0.0)	81.1 (61.8–103.6)
Greece	YLDs Rate: Cumulative change (%)	-3.1 (-6.0 to 0.2)	8.3 (-6.3 to 25.4)	1.5 (-0.8 to 3.8)	12.9 (1.2–32.7)	-14.5 (-22.4 to -6.4)	-15.2 (-32.9 to 5.2)	16.1 (2.0–35.6)	-87.6 (-90.9 to -84.3)	-41.0 (-59.8 to -17.9)	-0.6 (-13.0 to 12.5)	-27.1 (-35.0 to -19.1)
Iceland	Prevalence Number: 1990	71,186 (69,990–72,177)	17,051 (15,955–18,101)	23,346 (22,312–24,383)	6,764 (5,632–7,987)	12,653 (11,341–14,793)	19 (18–21)	1,315 (1,158–1,515)	0 (0–0)	203 (159–246)	0 (0–0)	721 (623–837)

© 2017 Global Burden of Disease Child and Adolescent Health Collaboration. JAMA Pediatrics.

eTable 3. Prevalent Cases, Rates (per 100,000 population), Years Lived with Disability (YLDs), and Cumulative Percent Change with 95% Uncertainty Interval (UI) for the Top 10 Global Causes of YLDs in Children and Adolescents in 195 Countries and Territories, Aged 0 to 19 Years, Both Sexes, 1990 and 2015. *Note: best viewed by enlarging in browser.*

Location	Measure	All causes	Iron-deficiency anemia	Skin and subcutaneous diseases	Asthma	Hemoglobinopathies and hemolytic anemias	Diarrheal diseases	Congenital anomalies	Protein-energy malnutrition	Epilepsy	Malaria	Neonatal preterm birth complications
Iceland	Prevalence Number: 2015	74,450 (73,294–75,614)	20,177 (18,271–22,005)	25,138 (24,045–26,237)	5,903 (4,853–7,069)	12,141 (10,633–14,596)	18 (16–20)	1,312 (1,152–1,513)	0 (0–0)	219 (169–276)	0 (0–0)	665 (568–801)
Iceland	Prevalence Number: Cumulative change (%)	4.6 (2.4–6.8)	18.5 (4.1–31.9)	7.7 (1.5–14.7)	-12.6 (-22.8 to -1.3)	-3.5 (-21.0 to 16.7)	-7.3 (-10.5 to -3.5)	0.4 (-16.3 to 19.8)	-49.7 (-62.5 to -32.7)	9.3 (-18.5 to 41.3)	2.9 (2.5–3.4)	-7.2 (-22.2 to 11.4)
Iceland	Prevalence Rate: 1990	83,768.5 (82,326.4–84,952.7)	20,379.5 (19,043.4–21,648.4)	27,132.4 (25,944.5–28,356.3)	7,910.7 (6,581.1–9,359.8)	14,941.1 (13,386.7–17,469.7)	24.2 (22.0–26.4)	1,553.7 (1,367.6–1,790.3)	0.0 (0.0–0.0)	239.2 (187.9–290.0)	0.0 (0.0–0.0)	862.7 (746.7–999.7)
Iceland	Prevalence Rate: 2015	84,102.9 (82,751.5–85,446.4)	23,462.6 (21,275.3–25,519.3)	27,678.9 (26,484.3–28,854.4)	6,641.0 (5,438.3–7,972.6)	13,797.6 (12,080.7–16,583.9)	21.7 (20.0–23.6)	1,489.0 (1,307.1–1,717.7)	0.0 (0.0–0.0)	247.9 (191.2–313.2)	0.0 (0.0–0.0)	776.5 (665.6–931.0)
Iceland	Prevalence Rate: Cumulative change (%)	0.4 (-1.8 to 2.6)	15.3 (1.5–28.3)	2.1 (-3.8 to 8.5)	-15.9 (-25.8 to -4.9)	-7.2 (-23.9 to 12.3)	-10.2 (-13.3 to -6.4)	-3.5 (-19.6 to 15.2)	-51.5 (-64.0 to -35.0)	4.9 (-21.6 to 35.6)	0.0 (-0.0 to 0.0)	-9.5 (-23.9 to 8.0)
Iceland	YLDs Number: 1990	4,201 (3,196–5,313)	569 (389–774)	449 (303–627)	308 (205–425)	200 (133–284)	3 (2–4)	112 (73–159)	0 (0–0)	56 (38–77)	0 (0–0)	72 (55–93)
Iceland	YLDs Number: 2015	4,298 (3,279–5,453)	708 (485–976)	479 (321–671)	269 (176–371)	164 (109–232)	3 (2–4)	156 (90–248)	0 (0–0)	35 (21–53)	0 (0–0)	57 (42–73)
Iceland	YLDs Number: Cumulative change (%)	2.4 (-1.2 to 5.7)	24.8 (5.8–41.3)	6.6 (3.9–9.2)	-12.6 (-22.9 to -1.2)	-17.7 (-24.1 to -10.7)	-5.8 (-24.7 to 15.4)	38.2 (7.2–77.0)	-49.6 (-62.3 to -32.8)	-35.7 (-57.0 to -9.2)	4.1 (-5.2 to 14.2)	-21.5 (-29.7 to -12.6)
Iceland	YLDs Rate: 1990	4,899.9 (3,729.9–6,203.7)	681.6 (466.5–929.1)	525.3 (354.9–731.9)	360.7 (239.7–497.7)	236.7 (157.5–335.8)	4.0 (2.7–5.5)	132.6 (86.9–188.8)	0.0 (0.0–0.0)	66.2 (45.3–91.4)	0.0 (0.0–0.0)	85.8 (65.1–110.3)
Iceland	YLDs Rate: 2015	4,786.1 (3,654.5–6,083.3)	832.4 (570.8–1,147.1)	533.0 (357.7–744.8)	302.8 (198.6–418.2)	191.3 (126.6–270.4)	3.6 (2.4–5.0)	177.4 (102.8–283.0)	0.0 (0.0–0.0)	40.4 (24.8–61.1)	0.0 (0.0–0.0)	64.6 (48.4–83.6)
Iceland	YLDs Rate: Cumulative change (%)	-2.3 (-5.8 to 1.0)	22.5 (3.2–38.7)	1.5 (-1.1 to 3.8)	-15.9 (-26.1 to -4.9)	-19.1 (-25.5 to -12.2)	-8.7 (-27.0 to 12.0)	32.8 (3.3–70.0)	-51.4 (-63.9 to -34.7)	-38.3 (-58.6 to -12.7)	2.1 (-6.9 to 12.2)	-24.6 (-32.5 to -16.0)
Ireland	Prevalence Number: 1990	1,080,622 (1,066,220–1,094,123)	301,430 (270,826–330,008)	383,264 (368,333–398,648)	178,090 (150,681–206,531)	201,502 (178,993–236,717)	282 (264–304)	21,598 (19,644–24,025)	0 (0–0)	4,194 (3,148–5,393)	0 (0–0)	11,152 (9,759–13,174)
Ireland	Prevalence Number: 2015	1,057,355 (1,039,003–1,076,550)	330,223 (302,966–361,908)	365,520 (350,601–380,905)	116,054 (95,704–138,260)	183,233 (159,554–220,990)	367 (333–401)	21,272 (19,056–24,253)	0 (0–0)	3,805 (2,799–4,964)	0 (0–0)	9,588 (8,382–11,158)
Ireland	Prevalence Number: Cumulative change (%)	-2.1 (-4.1 to -0.2)	9.9 (-4.8 to 27.6)	-4.6 (-9.4 to 0.6)	-34.8 (-41.0 to -28.2)	-8.6 (-24.8 to 11.5)	30.2 (23.2–38.6)	-1.2 (-14.9 to 14.1)	-59.6 (-69.4 to -46.7)	-8.2 (-32.1 to 21.0)	7.3 (3.5–11.7)	-13.4 (-28.2 to 4.3)
Ireland	Prevalence Rate: 1990	83,022.8 (81,852.5–84,134.7)	23,868.7 (21,077.5–26,337.3)	28,727.8 (27,604.4–29,856.7)	13,278.5 (11,225.2–15,439.1)	15,660.5 (13,903.4–18,409.4)	25.0 (23.3–27.1)	1,683.0 (1,530.9–1,872.5)	0.0 (0.0–0.1)	320.1 (240.5–409.4)	0.0 (0.0–0.0)	952.3 (841.3–1,107.6)
Ireland	Prevalence Rate: 2015	83,238.8 (81,875.9–84,694.1)	25,427.7 (23,329.0–27,800.1)	29,520.1 (28,339.2–30,793.3)	9,199.8 (7,598.8–10,971.6)	14,351.2 (12,496.5–17,329.6)	27.8 (25.3–30.3)	1,665.6 (1,492.2–1,899.5)	0.0 (0.0–0.0)	300.5 (220.9–392.4)	0.0 (0.0–0.0)	748.3 (653.9–871.3)
Ireland	Prevalence Rate: Cumulative change (%)	0.3 (-1.7 to 2.3)	7.0 (-8.1 to 24.7)	2.8 (-2.4 to 8.4)	-30.7 (-36.9 to -23.7)	-7.8 (-24.3 to 12.5)	11.2 (5.3–18.5)	-0.7 (-14.4 to 14.8)	-62.2 (-71.7 to -49.7)	-5.0 (-30.1 to 25.3)	-0.0 (-0.0 to 0.0)	-21.0 (-33.8 to -5.3)
Ireland	YLDs Number: 1990	73,527 (55,724–93,608)	10,778 (7,629–14,685)	8,928 (6,192–12,304)	8,092 (5,382–11,033)	2,663 (1,727–3,696)	46 (31–64)	2,295 (1,648–3,050)	0 (0–0)	1,127 (716–1,594)	0 (0–0)	1,008 (758–1,288)
Ireland	YLDs Number: 2015	65,515 (49,701–83,246)	12,076 (8,545–16,583)	8,710 (6,059–11,983)	5,283 (3,503–7,377)	2,253 (1,537–3,094)	60 (42–83)	2,521 (1,679–3,577)	0 (0–0)	623 (366–968)	0 (0–0)	648 (475–839)
Ireland	YLDs Number: Cumulative change (%)	-10.9 (-13.9 to -7.1)	12.7 (-6.8 to 36.1)	-2.4 (-4.7 to 0.3)	-34.7 (-40.9 to -27.8)	-15.0 (-22.6 to -0.9)	30.7 (6.0–62.1)	9.9 (-9.3 to 37.1)	-59.6 (-69.3 to -46.8)	-44.0 (-62.8 to -20.4)	7.7 (-7.5 to 26.9)	-35.7 (-43.3 to -27.6)
Ireland	YLDs Rate: 1990	5,515.7 (4,175.8–7,022.3)	847.0 (593.3–1,162.1)	679.9 (473.2–935.1)	603.6 (401.4–825.5)	201.9 (130.4–280.2)	4.2 (2.8–5.7)	179.0 (128.3–238.8)	0.0 (0.0–0.0)	86.2 (54.7–121.7)	0.0 (0.0–0.0)	78.5 (59.1–100.4)
Ireland	YLDs Rate: 2015	5,248.3 (3,981.6–6,653.4)	918.5 (650.1–1,258.3)	691.3 (479.3–953.7)	418.7 (278.4–582.6)	173.4 (118.7–238.1)	4.6 (3.2–6.3)	197.3 (131.4–280.2)	0.0 (0.0–0.0)	49.2 (29.0–76.3)	0.0 (0.0–0.0)	50.8 (37.3–66.0)
Ireland	YLDs Rate: Cumulative change (%)	-4.8 (-8.2 to -0.5)	9.2 (-10.7 to 34.5)	1.7 (-0.7 to 4.1)	-30.6 (-37.2 to -23.4)	-13.7 (-21.5 to 1.3)	11.8 (-9.1 to 38.5)	10.3 (-8.8 to 37.4)	-62.1 (-71.6 to -49.6)	-42.2 (-61.9 to -17.7)	1.9 (-14.1 to 21.5)	-35.3 (-42.9 to -27.3)
Israel	Prevalence Number: 1990	1,544,946 (1,523,511–1,564,170)	464,206 (420,446–497,463)	512,082 (488,588–537,167)	153,905 (126,961–182,689)	284,537 (272,734–297,838)	584 (539–638)	27,512 (24,141–32,066)	3 (2–5)	4,378 (3,490–5,387)	0 (0–0)	18,455 (16,134–21,656)
Israel	Prevalence Number: 2015	2,388,980 (2,357,097–2,419,931)	755,324 (699,242–801,829)	790,632 (755,402–823,331)	161,075 (132,364–192,773)	404,119 (384,616–422,540)	853 (783–935)	41,368 (36,262–48,038)	4 (2–7)	6,660 (5,163–8,292)	0 (0–0)	25,549 (22,293–30,110)
Israel	Prevalence Number: Cumulative change (%)	54.6 (51.9–57.4)	63.1 (45.0–78.7)	54.5 (45.8–63.4)	4.8 (-6.3 to 16.1)	42.1 (33.6–50.8)	46.1 (40.1–51.8)	51.4 (23.9–85.6)	38.0 (-1.7 to 87.3)	54.1 (17.5–98.7)	60.7 (58.1–63.7)	39.2 (17.9–63.6)
Israel	Prevalence Rate: 1990	84,245.9 (83,071.0–85,294.9)	25,312.9 (22,919.1–27,150.0)	27,896.5 (26,620.7–29,257.1)	8,389.3 (6,918.4–9,959.0)	15,517.1 (14,874.4–16,243.0)	32.0 (29.5–34.9)	1,500.6 (1,316.7–1,749.0)	0.2 (0.1–0.3)	238.9 (190.6–293.9)	0.0 (0.0–0.0)	1,004.0 (877.3–1,178.5)
Israel	Prevalence Rate: 2015	84,167.7 (83,080.7–85,231.1)	25,974.3 (24,121.3–27,483.6)	28,441.2 (27,165.8–29,639.2)	5,739.1 (4,721.6–6,862.2)	14,153.4 (13,467.9–14,797.0)	28.3 (26.0–30.9)	1,449.2 (1,269.7–1,682.8)	0.2 (0.1–0.2)	234.4 (181.4–292.3)	0.0 (0.0–0.0)	866.8 (754.4–1,024.1)
Israel	Prevalence Rate: Cumulative change (%)	-0.1 (-1.8 to 1.7)	2.8 (-8.4 to 12.9)	2.0 (-3.8 to 8.0)	-31.5 (-38.9 to -23.8)	-8.7 (-14.2 to -3.2)	-11.5 (-15.1 to -8.0)	-2.8 (-20.4 to 19.1)	-16.7 (-40.3 to 12.7)	-0.6 (-24.2 to 28.3)	-0.0 (-0.0 to 0.0)	-13.2 (-26.6 to 2.5)
Israel	YLDs Number: 1990	94,803 (71,950–120,500)	17,214 (11,883–23,693)	11,102 (7,620–15,285)	7,015 (4,589–9,555)	2,331 (1,661–3,157)	96 (68–132)	1,864 (1,262–2,546)	0 (0–0)	1,188 (777–1,653)	0 (0–0)	1,865 (1,416–2,374)
Israel	YLDs Number: 2015	137,128 (105,147–173,836)	27,917 (19,553–38,385)	17,221 (11,858–23,752)	7,344 (4,853–10,156)	2,716 (1,944–3,680)	141 (96–197)	4,539 (2,776–6,642)	0 (0–0)	1,087 (694–1,616)	0 (0–0)	2,085 (1,571–2,665)
Israel	YLDs Number: Cumulative change (%)	44.7 (39.6–49.0)	62.6 (38.7–78.1)	55.2 (51.6–58.5)	4.9 (-6.4 to 16.5)	16.6 (6.7–25.1)	46.9 (19.4–77.4)	142.9 (85.6–195.0)	37.8 (-1.8 to 87.0)	-7.2 (-36.2 to 27.6)	60.4 (48.7–73.6)	12.0 (0.9–23.5)
Israel	YLDs Rate: 1990	5,165.0 (3,919.7–6,567.5)	937.0 (646.4–1,288.6)	605.4 (415.5–833.3)	382.4 (250.3–521.1)	126.6 (90.2–171.4)	5.3 (3.7–7.2)	101.7 (68.9–138.9)	0.0 (0.0–0.0)	64.9 (42.4–90.3)	0.0 (0.0–0.0)	101.7 (77.2–129.6)
Israel	YLDs Rate: 2015	4,896.7 (3,755.6–6,195.2)	961.5 (673.9–1,319.1)	613.0 (422.5–847.2)	261.6 (173.1–362.0)	95.1 (68.1–128.7)	4.7 (3.2–6.5)	159.0 (97.4–232.6)	0.0 (0.0–0.0)	38.3 (24.4–57.0)	0.0 (0.0–0.0)	73.3 (55.2–93.7)
Israel	YLDs Rate: Cumulative change (%)	-5.2 (-8.3 to -2.3)	2.9 (-11.9 to 12.8)	1.2 (-1.0 to 3.4)	-31.4 (-38.9 to -23.8)	-24.8 (-31.1 to -19.3)	-11.0 (-27.8 to 7.3)	55.9 (19.0–89.3)	-16.7 (-40.4 to 12.6)	-40.2 (-58.8 to -17.9)	0.3 (-7.0 to 8.3)	-27.8 (-34.9 to -20.4)
Italy	Prevalence Number: 1990	11,658,354 (11,511,460–11,796,391)	2,247,762 (2,078,909–2,472,850)	4,034,725 (3,871,212–4,224,127)	793,377 (649,241–954,242)	3,619,477 (3,449,402–3,757,856)	2,830 (2,633–3,035)	168,346 (151,327–189,412)	6 (4–9)	34,873 (27,566–42,376)	0 (0–0)	119,659 (105,635–138,574)
Italy	Prevalence Number: 2015	9,477,514 (9,333,361–9,607,532)	1,884,827 (1,660,097–2,116,756)	3,187,604 (3,056,520–3,330,904)	500,052 (402,769–605,710)	2,782,211 (2,650,509–2,907,886)	2,516 (2,314–2,727)	159,796 (140,634–185,439)	11 (7–17)	26,997 (21,038–33,291)	0 (0–0)	84,608 (74,774–97,579)
Italy	Prevalence Number: Cumulative change (%)	-18.7 (-20.3 to -17.2)	-15.8 (-31.3 to -1.6)	-20.9 (-25.5 to -16.6)	-36.6 (-48.4 to -26.3)	-23.1 (-28.0 to -18.1)	-11.1 (-14.5 to -7.3)	-4.7 (-20.1 to 13.2)	83.6 (44.0–132.1)	-21.6 (-41.5 to 1.2)	-10.5 (-13.3 to -7.4)	-28.9 (-39.9 to -16.7)
Italy	Prevalence Rate: 1990	82,862.9 (81,634.5–83,979.2)	18,294.2 (16,719.2–20,381.2)	26,413.4 (25,433.9–27,496.0)	5,482.9 (4,487.4–6,526.6)	26,210.2 (24,977.7–27,192.3)	24.4 (22.6–26.2)	1,218.9 (1,096.6–1,370.9)	0.1 (0.0–0.1)	248.5 (197.5–301.6)	0.0 (0.0–0.0)	939.5 (837.0–1,072.8)
Italy	Prevalence Rate: 2015	82,168.6 (80,825.8–83,379.4)	17,328.6 (15,079.7–19,602.8)	26,905.9 (25,842.1–28,082.0)	4,246.9 (3,418.0–5,163.5)	24,355.2 (23,224.9–25,452.8)	24.2 (22.2–26.4)	1,398.5 (1,230.2–1,625.6)	0.1 (0.1–0.2)	235.0 (183.4–289.1)	0.0 (0.0–0.0)	791.5 (705.2–906.7)
Italy	Prevalence Rate: Cumulative change (%)	-0.8 (-2.9 to 1.1)	-4.8 (-23.6 to 12.6)	1.9 (-3.5 to 7.3)	-22.2 (-36.3 to -9.5)	-7.0 (-12.9 to -0.9)	-0.8 (-4.7 to 3.8)	15.2 (-3.3 to 36.8)	117.7 (68.5–178.1)	-4.3 (-28.2 to 23.6)	-0.0 (-0.0 to 0.0)	-15.4 (-27.5 to -1.8)
Italy	YLDs Number: 1990	743,625 (569,382–938,540)	70,228 (46,967–99,133)	82,976 (56,586–116,348)	36,131 (23,668–50,797)	50,883 (36,811–68,366)	471 (329–644)	16,051 (10,864–22,030)	0 (0–1)	9,552 (6,416–13,247)	0 (0–0)	14,376 (11,127–18,327)
Italy	YLDs Number: 2015	555,311 (421,226–705,822)	59,263 (40,039–82,527)	66,423 (45,462–92,952)	22,804 (14,718–32,290)	44,052 (31,748–58,778)	418 (289–585)	17,316 (10,432–26,028)	1 (0–2)	4,391 (2,708–6,337)	0 (0–0)	7,883 (6,050–10,072)
Italy	YLDs Number: Cumulative change (%)	-25.3 (-28.4 to -22.4)	-14.8 (-38.4 to 2.6)	-19.9 (-21.9 to -17.8)	-36.5 (-48.1 to -25.5)	-13.3 (-20.9 to -3.3)	-10.6 (-28.6 to 10.5)	7.6 (-13.7 to 41.2)	83.5 (43.9–131.5)	-53.4 (-67.8 to -35.7)	-7.8 (-21.7 to 4.7)	-45.0 (-51.3 to -37.9)
Italy	YLDs Rate: 1990	5,006.8 (3,820.3–6,331.1)	579.5 (380.5–822.0)	561.3 (386.7–778.6)	250.0 (163.8–350.2)	377.8 (272.6–507.2)	4.1 (2.8–5.6)	116.4 (78.6–159.9)	0.0 (0.0–0.0)	68.2 (46.0–94.4)	0.0 (0.0–0.0)	103.3 (79.8–131.9)
Italy	YLDs Rate: 2015	4,715.3 (3,569.6–5,982.3)	544.7 (368.6–760.2)	565.9 (388.2–790.5)	193.7 (125.0–275.2)	381.5 (274.9–509.4)	4.0 (2.8–5.6)	151.6 (91.3–227.7)	0.0 (0.0–0.0)	38.3 (23.8–55.0)	0.0 (0.0–0.0)	68.5 (52.8–87.7)
Italy	YLDs Rate: Cumulative change (%)	-5.8 (-10.6 to -1.9)	-4.8 (-33.8 to 16.1)	0.8 (-1.6 to 3.0)	-22.1 (-36.4 to -8.6)	1.2 (-8.1 to 12.0)	-0.1 (-21.1 to 24.2)	30.0 (4.5–70.9)	117.7 (69.1–177.9)	-43.1 (-60.7 to -21.8)	0.9 (-16.0 to 16.0)	-33.4 (-40.8 to -25.0)
Luxembourg	Prevalence Number: 1990	73,227 (72,058–74,500)	19,160 (17,540–21,906)	25,150 (24,075–26,295)	10,150 (8,509–11,995)	13,691 (12,231–16,214)	28 (26–30)	1,322 (1,160–1,528)	0 (0–0)	305 (232–389)	0 (0–0)	698 (613–812)
Luxembourg	Prevalence Number: 2015	106,532 (104,741–108,270)	28,639 (26,728–31,652)	38,169 (36,505–39,738)	11,228 (9,252–13,617)	18,230 (15,870–22,209)	36 (33–39)	1,879 (1,650–2,174)	0 (0–0)	400 (289–531)	0 (0–0)	835 (717–985)
Luxembourg	Prevalence Number: Cumulative change (%)	45.5 (42.3–48.6)	49.8 (33.1–60.4)	51.8 (43.6–60.3)	10.7 (-0.8 to 23.6)	33.9 (8.9–65.2)	29.1 (24.6–34.2)	43.0 (18.5–71.3)	-43.8 (-59.6 to -25.3)	32.9 (-5.2 to 77.2)	42.5 (40.6–44.2)	20.3 (-0.7 to 44.0)

© 2017 Global Burden of Disease Child and Adolescent Health Collaboration. JAMA Pediatrics.

eTable 3. Prevalent Cases, Rates (per 100,000 population), Years Lived with Disability (YLDs), and Cumulative Percent Change with 95% Uncertainty Interval (UI) for the Top 10 Global Causes of YLDs in Children and Adolescents in 195 Countries and Territories, Aged 0 to 19 Years, Both Sexes, 1990 and 2015. *Note: best viewed by enlarging in browser.*

Location	Measure	All causes	Iron-deficiency anemia	Skin and subcutaneous diseases	Asthma	Hemoglobinopathies and hemolytic anemias	Diarrheal diseases	Congenital anomalies	Protein-energy malnutrition	Epilepsy	Malaria	Neonatal preterm birth complications
Luxembourg	Prevalence Rate: 1990	83,571.4 (82,206.8–85,041.9)	22,140.8 (20,220.7–25,392.0)	28,375.9 (27,184.0–29,634.8)	11,532.7 (9,643.6–13,643.4)	15,672.9 (14,002.5–18,564.9)	33.0 (30.5–35.8)	1,513.8 (1,328.3–1,748.0)	0.1 (0.1–0.2)	348.7 (265.0–445.1)	0.0 (0.0–0.0)	799.3 (702.7–929.6)
Luxembourg	Prevalence Rate: 2015	83,219.0 (81,764.1–84,650.4)	22,988.1 (21,423.3–25,475.6)	29,091.6 (27,835.8–30,283.3)	8,657.4 (7,109.9–10,535.7)	14,340.7 (12,477.3–17,476.9)	30.6 (28.4–33.2)	1,478.6 (1,297.5–1,710.9)	0.0 (0.0–0.1)	313.3 (226.3–413.6)	0.0 (0.0–0.0)	673.2 (580.7–791.6)
Luxembourg	Prevalence Rate: Cumulative change (%)	-0.4 (-2.6 to 1.8)	4.1 (-7.6 to 11.9)	2.6 (-2.9 to 8.2)	-24.9 (-32.7 to -16.1)	-8.0 (-25.2 to 13.6)	-7.3 (-10.6 to -3.6)	-1.7 (-18.6 to 17.8)	-60.1 (-71.4 to -46.7)	-9.0 (-34.9 to 21.3)	-0.0 (-0.0 to 0.0)	-15.3 (-30.0 to 1.1)
Luxembourg	YLDs Number: 1990	4,782 (3,603–6,033)	674 (456–926)	558 (384–773)	461 (306–635)	173 (112–254)	4 (3–6)	127 (83–180)	0 (0–0)	86 (55–122)	0 (0–0)	67 (50–86)
Luxembourg	YLDs Number: 2015	6,643 (5,050–8,385)	1,028 (711–1,409)	833 (573–1,156)	511 (336–713)	226 (149–329)	6 (4–8)	251 (148–410)	0 (0–0)	72 (42–110)	0 (0–0)	68 (50–88)
Luxembourg	YLDs Number: Cumulative change (%)	39.0 (35.2–42.7)	53.3 (32.8–63.4)	49.3 (45.8–52.7)	10.8 (-0.9 to 23.8)	30.8 (22.1–39.2)	30.0 (8.3–54.4)	96.4 (55.1–172.3)	-43.7 (-59.6 to -25.3)	-14.9 (-45.4 to 24.7)	46.4 (33.1–61.6)	2.8 (-9.6 to 16.5)
Luxembourg	YLDs Rate: 1990	5,410.7 (4,080.9–6,829.7)	782.5 (530.4–1,076.5)	634.5 (438.2–879.8)	525.0 (348.6–722.6)	199.7 (129.6–293.1)	5.5 (3.7–7.5)	145.8 (95.5–206.8)	0.0 (0.0–0.0)	98.9 (64.0–140.5)	0.0 (0.0–0.0)	76.7 (57.8–98.6)
Luxembourg	YLDs Rate: 2015	5,093.1 (3,871.5–6,444.0)	826.0 (570.4–1,132.9)	644.5 (443.8–889.3)	394.5 (258.7–551.0)	178.4 (118.3–259.8)	5.1 (3.5–6.8)	197.7 (116.7–323.5)	0.0 (0.0–0.0)	56.6 (33.3–86.0)	0.0 (0.0–0.0)	53.9 (39.6–69.4)
Luxembourg	YLDs Rate: Cumulative change (%)	-5.8 (-8.5 to -3.2)	6.0 (-8.1 to 13.5)	1.6 (-0.8 to 3.7)	-24.8 (-32.8 to -15.8)	-10.4 (-16.4 to -4.4)	-6.6 (-22.3 to 11.7)	35.0 (6.5–87.1)	-60.0 (-71.3 to -46.7)	-41.7 (-62.6 to -14.9)	2.3 (-7.2 to 12.5)	-29.6 (-38.1 to -20.2)
Malta	Prevalence Number: 1990	92,760 (91,240–94,163)	25,817 (23,533–28,008)	30,605 (29,387–31,966)	11,156 (9,771–12,543)	18,438 (16,378–21,306)	27 (25–29)	2,204 (1,973–2,502)	0 (0–0)	286 (215–368)	0 (0–0)	1,185 (1,054–1,364)
Malta	Prevalence Number: 2015	73,140 (71,883–74,295)	19,614 (17,826–21,869)	26,262 (25,089–27,478)	7,745 (6,327–9,207)	13,311 (11,570–15,852)	19 (18–22)	1,823 (1,615–2,092)	0 (0–0)	254 (188–333)	0 (0–0)	808 (720–935)
Malta	Prevalence Number: Cumulative change (%)	-21.1 (-22.9 to -19.4)	-23.9 (-31.0 to -15.6)	-14.1 (-18.7 to -9.1)	-30.6 (-39.8 to -21.6)	-27.4 (-40.9 to -11.8)	-26.9 (-30.7 to -22.3)	-16.9 (-29.8 to -2.1)	-64.7 (-73.2 to -52.8)	-10.0 (-32.2 to 16.6)	-27.9 (-30.5 to -25.7)	-31.5 (-41.9 to -20.7)
Malta	Prevalence Rate: 1990	83,532.4 (82,159.5–84,798.5)	23,289.1 (21,200.5–25,273.3)	27,447.4 (26,357.1–28,664.8)	9,991.6 (8,747.0–11,222.3)	16,614.6 (14,759.8–19,201.2)	25.2 (23.3–27.3)	1,988.0 (1,781.0–2,256.0)	0.1 (0.1–0.2)	258.3 (193.6–331.9)	0.0 (0.0–0.0)	1,084.1 (966.7–1,245.6)
Malta	Prevalence Rate: 2015	83,553.3 (81,974.4–84,958.8)	24,057.3 (21,919.5–26,991.1)	28,225.9 (27,051.8–29,487.2)	8,651.4 (7,041.7–10,379.6)	15,399.9 (13,400.4–18,372.9)	26.9 (24.7–30.1)	2,113.1 (1,874.3–2,428.7)	0.1 (0.0–0.1)	291.7 (216.2–381.4)	0.0 (0.0–0.0)	999.2 (896.5–1,145.5)
Malta	Prevalence Rate: Cumulative change (%)	0.0 (-2.3 to 2.3)	3.4 (-5.9 to 15.7)	2.9 (-2.5 to 8.9)	-13.5 (-25.4 to -1.4)	-6.8 (-24.2 to 13.3)	7.1 (1.6–14.0)	6.8 (-9.9 to 26.1)	-51.0 (-63.4 to -33.9)	14.3 (-14.1 to 47.7)	0.0 (-0.0 to 0.0)	-7.4 (-20.9 to 6.2)
Malta	YLDs Number: 1990	5,849 (4,426–7,457)	919 (648–1,253)	657 (449–909)	507 (342–685)	218 (134–320)	4 (3–6)	211 (144–288)	0 (0–0)	77 (51–110)	0 (0–0)	119 (90–154)
Malta	YLDs Number: 2015	4,697 (3,588–5,961)	695 (485–958)	546 (373–755)	352 (233–489)	149 (98–211)	3 (2–4)	222 (149–313)	0 (0–0)	41 (24–62)	0 (0–0)	78 (57–101)
Malta	YLDs Number: Cumulative change (%)	-19.7 (-21.7 to -17.0)	-24.2 (-31.1 to -12.5)	-17.0 (-19.3 to -14.6)	-30.6 (-39.9 to -21.6)	-30.9 (-37.6 to -22.0)	-25.9 (-40.8 to -7.7)	5.4 (-9.5 to 21.8)	-64.6 (-73.2 to -52.8)	-45.9 (-63.4 to -24.9)	-26.5 (-33.5 to -18.1)	-34.6 (-40.9 to -28.4)
Malta	YLDs Rate: 1990	5,249.2 (3,973.3–6,694.2)	825.4 (581.0–1,126.7)	590.6 (404.2–816.4)	454.9 (307.0–613.1)	194.9 (119.8–285.8)	4.2 (2.9–5.6)	190.6 (129.7–260.5)	0.0 (0.0–0.0)	69.9 (46.3–99.5)	0.0 (0.0–0.0)	107.8 (81.3–139.3)
Malta	YLDs Rate: 2015	5,157.8 (3,920.2–6,559.5)	861.2 (597.8–1,197.5)	603.1 (415.6–830.3)	394.0 (259.9–548.9)	178.1 (116.8–251.3)	4.5 (3.1–6.1)	258.3 (172.8–364.2)	0.0 (0.0–0.0)	47.6 (28.5–71.6)	0.0 (0.0–0.0)	90.7 (67.3–117.6)
Malta	YLDs Rate: Cumulative change (%)	-1.7 (-4.4 to 2.1)	4.6 (-5.3 to 22.9)	2.1 (-0.3 to 4.4)	-13.4 (-25.4 to -1.6)	-7.8 (-16.5 to 5.0)	8.6 (-13.7 to 35.6)	35.7 (16.4–56.4)	-51.0 (-63.3 to -33.9)	-31.2 (-53.8 to -4.5)	2.6 (-7.1 to 14.6)	-15.9 (-24.0 to -8.1)
Netherlands	Prevalence Number: 1990	3,080,475 (3,031,452–3,130,957)	737,736 (673,331–800,392)	1,123,399 (1,077,416–1,169,558)	329,992 (272,683–393,460)	463,988 (420,628–510,809)	1,872 (1,780–1,968)	68,131 (62,384–75,010)	2 (1–4)	10,445 (8,279–12,658)	0 (0–0)	33,071 (29,032–38,437)
Netherlands	Prevalence Number: 2015	3,195,738 (3,135,958–3,251,108)	728,809 (624,839–846,102)	1,182,390 (1,136,455–1,230,744)	320,716 (263,482–385,727)	425,067 (368,241–487,437)	2,027 (1,922–2,134)	65,324 (58,437–72,961)	1 (1–2)	12,287 (9,485–15,287)	0 (0–0)	31,456 (27,789–36,311)
Netherlands	Prevalence Number: Cumulative change (%)	3.8 (1.4–6.2)	-1.2 (-12.4 to 13.4)	5.3 (-0.5 to 11.1)	-2.7 (-10.6 to 6.6)	-8.1 (-22.5 to 8.4)	8.3 (4.7–12.0)	-3.9 (-16.3 to 10.0)	-43.1 (-59.8 to -24.8)	19.0 (-10.6 to 54.3)	5.0 (3.3–6.3)	-4.3 (-19.9 to 13.0)
Netherlands	Prevalence Rate: 1990	80,652.9 (79,302.7–82,041.0)	20,231.1 (18,478.7–22,149.6)	28,415.8 (27,275.2–29,581.1)	8,606.9 (7,065.0–10,311.2)	12,272.0 (11,123.0–13,507.3)	52.2 (49.6–55.0)	1,798.8 (1,645.9–1,981.3)	0.1 (0.1–0.1)	273.6 (216.4–331.8)	0.0 (0.0–0.0)	893.0 (785.3–1,034.0)
Netherlands	Prevalence Rate: 2015	80,460.2 (78,817.0–81,969.3)	19,392.1 (16,395.2–22,849.2)	29,014.2 (27,914.2–30,195.2)	7,936.0 (6,516.4–9,573.9)	10,831.4 (9,383.0–12,423.2)	56.7 (53.6–59.8)	1,661.9 (1,487.0–1,855.6)	0.0 (0.0–0.1)	311.5 (241.1–387.0)	0.0 (0.0–0.0)	845.3 (751.7–967.0)
Netherlands	Prevalence Rate: Cumulative change (%)	-0.2 (-2.6 to 2.3)	-4.1 (-16.7 to 11.7)	2.2 (-3.3 to 7.5)	-7.7 (-15.3 to 1.2)	-11.4 (-25.3 to 4.4)	8.5 (4.8–12.3)	-7.4 (-19.5 to 6.0)	-42.0 (-59.8 to -22.8)	15.2 (-13.3 to 49.2)	0.0 (-0.0 to 0.0)	-4.8 (-19.7 to 11.7)
Netherlands	YLDs Number: 1990	195,275 (148,721–247,096)	25,910 (18,196–35,493)	23,762 (16,307–32,920)	15,051 (9,959–20,745)	3,161 (2,239–4,279)	309 (216–416)	6,550 (4,397–9,171)	0 (0–0)	2,884 (1,867–3,930)	0 (0–0)	3,007 (2,247–3,887)
Netherlands	YLDs Number: 2015	196,289 (149,519–248,072)	25,774 (17,706–36,258)	25,080 (17,089–34,998)	14,634 (9,533–20,333)	3,637 (2,597–4,889)	335 (232–450)	7,162 (4,458–10,377)	0 (0–0)	2,006 (1,227–2,916)	0 (0–0)	2,646 (1,982–3,396)
Netherlands	YLDs Number: Cumulative change (%)	0.5 (-2.4 to 3.8)	-0.4 (-14.1 to 20.5)	5.6 (3.0–8.0)	-2.7 (-10.5 to 7.0)	15.3 (5.2–27.7)	8.5 (-7.5 to 25.7)	9.6 (-10.6 to 41.1)	-43.0 (-59.8 to -24.9)	-29.5 (-51.9 to -1.8)	9.0 (-3.2 to 19.9)	-11.9 (-21.1 to -2.6)
Netherlands	YLDs Rate: 1990	4,987.5 (3,791.8–6,332.7)	718.9 (503.2–987.9)	612.1 (421.0–849.7)	392.7 (259.9–543.4)	86.8 (61.4–117.0)	8.6 (6.0–11.7)	173.1 (116.3–242.4)	0.0 (0.0–0.0)	75.6 (49.2–103.0)	0.0 (0.0–0.0)	79.4 (59.6–102.6)
Netherlands	YLDs Rate: 2015	4,838.3 (3,679.3–6,139.8)	682.1 (462.1–973.5)	623.1 (425.9–865.1)	362.2 (236.6–503.3)	92.3 (65.5–125.4)	9.4 (6.5–12.6)	182.3 (113.4–263.9)	0.0 (0.0–0.0)	50.9 (30.9–74.0)	0.0 (0.0–0.0)	67.2 (50.2–86.1)
Netherlands	YLDs Rate: Cumulative change (%)	-3.0 (-6.1 to 0.8)	-5.0 (-19.4 to 17.7)	1.8 (-0.6 to 4.1)	-7.6 (-15.2 to 1.5)	6.6 (-3.3 to 18.7)	8.9 (-7.1 to 26.6)	5.6 (-14.0 to 36.2)	-41.9 (-59.7 to -22.8)	-31.8 (-53.2 to -4.5)	1.3 (-10.7 to 12.6)	-15.2 (-24.0 to -6.4)
Norway	Prevalence Number: 1990	883,451 (872,025–894,570)	215,914 (203,058–228,308)	321,014 (309,375–333,851)	83,027 (60,245–99,457)	151,315 (139,148–163,672)	294 (263–333)	21,621 (18,621–25,446)	0 (0–0)	3,943 (3,226–4,708)	0 (0–0)	10,201 (8,997–11,676)
Norway	Prevalence Number: 2015	1,020,219 (1,001,545–1,039,414)	262,643 (238,342–304,444)	374,856 (361,209–389,183)	79,821 (65,423–96,834)	153,692 (137,182–171,450)	302 (278–331)	25,409 (22,669–28,955)	0 (0–0)	4,561 (3,532–5,712)	0 (0–0)	10,511 (9,142–12,260)
Norway	Prevalence Number: Cumulative change (%)	15.5 (13.0–18.1)	21.9 (8.1–46.9)	16.8 (11.1–22.9)	-2.8 (-15.9 to 28.4)	1.8 (-10.0 to 15.0)	2.9 (-5.9 to 9.7)	18.5 (-3.0 to 42.7)	21.0 (-7.3 to 55.5)	17.0 (-14.2 to 53.1)	13.1 (12.2–13.9)	3.5 (-12.1 to 21.3)
Norway	Prevalence Rate: 1990	78,535.8 (77,495.7–79,563.8)	20,138.9 (18,959.7–21,312.3)	27,667.7 (26,652.0–28,771.1)	7,389.4 (5,321.6–8,918.4)	13,600.3 (12,509.8–14,713.6)	27.8 (24.8–31.5)	1,941.1 (1,672.0–2,286.1)	0.0 (0.0–0.0)	351.0 (287.0–417.3)	0.0 (0.0–0.0)	926.7 (818.8–1,058.7)
Norway	Prevalence Rate: 2015	79,650.4 (78,070.0–81,285.6)	21,605.6 (19,505.2–25,233.6)	28,269.9 (27,261.3–29,347.4)	6,194.5 (5,066.2–7,526.2)	12,155.2 (10,856.4–13,551.3)	25.8 (23.7–28.4)	2,006.2 (1,785.8–2,285.2)	0.0 (0.0–0.0)	357.2 (275.5–447.5)	0.0 (0.0–0.0)	858.4 (750.0–996.7)
Norway	Prevalence Rate: Cumulative change (%)	1.4 (-0.9 to 3.9)	7.5 (-4.8 to 30.4)	2.2 (-2.6 to 7.4)	-15.2 (-26.6 to 12.3)	-10.5 (-20.8 to 1.2)	-6.8 (-14.7 to -0.5)	4.2 (-14.8 to 25.3)	6.0 (-18.8 to 35.2)	3.0 (-24.6 to 34.8)	-0.0 (-0.0 to 0.0)	-7.0 (-20.7 to 8.3)
Norway	YLDs Number: 1990	58,936 (44,851–74,211)	7,201 (5,021–9,820)	6,996 (4,845–9,629)	3,786 (2,372–5,212)	2,287 (1,638–3,047)	48 (33–66)	2,019 (1,266–2,938)	0 (0–0)	1,107 (744–1,506)	0 (0–0)	1,023 (779–1,308)
Norway	YLDs Number: 2015	65,286 (50,064–82,288)	9,096 (6,232–12,836)	8,061 (5,566–11,174)	3,637 (2,382–5,062)	2,387 (1,709–3,168)	50 (34–69)	2,900 (1,572–4,738)	0 (0–0)	764 (465–1,134)	0 (0–0)	935 (711–1,204)
Norway	YLDs Number: Cumulative change (%)	10.8 (7.2–16.4)	26.8 (7.2–66.2)	15.2 (12.2–18.0)	-2.8 (-16.4 to 28.0)	4.5 (-4.2 to 18.8)	3.5 (-16.4 to 25.6)	43.7 (7.3–121.1)	21.3 (-7.3 to 56.1)	-30.1 (-52.6 to -2.4)	16.3 (6.1–30.5)	-8.5 (-17.3 to 0.9)
Norway	YLDs Rate: 1990	5,128.6 (3,899.3–6,483.8)	679.9 (474.0–926.8)	614.0 (428.2–848.2)	337.2 (209.5–466.2)	211.8 (152.0–281.3)	4.6 (3.2–6.3)	181.4 (114.0–264.0)	0.0 (0.0–0.0)	98.7 (66.4–134.2)	0.0 (0.0–0.0)	91.8 (69.9–117.8)
Norway	YLDs Rate: 2015	4,974.3 (3,805.9–6,274.9)	752.3 (513.6–1,063.7)	619.2 (431.1–859.0)	282.4 (184.9–393.1)	192.4 (137.9–255.5)	4.3 (3.0–5.9)	229.2 (124.4–374.2)	0.0 (0.0–0.0)	59.9 (36.3–88.9)	0.0 (0.0–0.0)	73.6 (56.1–94.7)
Norway	YLDs Rate: Cumulative change (%)	-3.0 (-6.4 to 2.4)	11.1 (-6.5 to 46.9)	0.8 (-1.8 to 3.3)	-15.2 (-27.0 to 11.9)	-9.1 (-16.6 to 3.0)	-6.2 (-24.6 to 14.2)	26.4 (-5.5 to 94.2)	6.2 (-18.6 to 35.6)	-38.5 (-58.4 to -14.3)	1.9 (-7.1 to 14.5)	-19.8 (-27.4 to -11.4)
Portugal	Prevalence Number: 1990	2,439,179 (2,406,039–2,473,070)	575,752 (533,909–620,672)	866,433 (836,426–900,759)	306,203 (263,591–360,053)	351,189 (329,150–373,977)	1,143 (992–1,318)	29,646 (26,413–33,557)	16 (10–25)	7,585 (5,924–9,422)	0 (0–0)	29,375 (25,711–34,147)
Portugal	Prevalence Number: 2015	1,754,851 (1,726,545–1,782,399)	478,101 (440,070–514,667)	616,511 (594,140–641,271)	188,787 (153,953–228,212)	220,283 (205,373–235,213)	539 (493–585)	22,226 (19,653–25,119)	2 (1–3)	3,874 (2,986–4,798)	0 (0–0)	18,290 (15,556–21,915)
Portugal	Prevalence Number: Cumulative change (%)	-28.1 (-29.5 to -26.7)	-16.8 (-25.6 to -7.9)	-28.8 (-32.3 to -25.2)	-38.2 (-45.9 to -25.8)	-37.2 (-42.7 to -31.2)	-52.5 (-57.9 to -46.8)	-24.7 (-36.2 to -12.0)	-85.3 (-89.6 to -80.0)	-48.2 (-61.6 to -32.2)	-23.5 (-25.2 to -21.4)	-37.4 (-49.3 to -23.3)
Portugal	Prevalence Rate: 1990	82,666.2 (81,422.0–83,947.8)	21,273.0 (19,607.2–23,051.3)	27,732.8 (26,801.6–28,838.2)	10,143.7 (8,738.9–11,908.3)	12,098.2 (11,347.7–12,877.1)	48.4 (41.7–56.2)	1,022.2 (912.5–1,153.6)	0.8 (0.5–1.1)	258.1 (203.0–318.8)	0.0 (0.0–0.0)	1,116.7 (991.3–1,281.0)
Portugal	Prevalence Rate: 2015	82,640.1 (81,232.9–84,032.8)	23,960.5 (21,830.2–26,123.3)	28,412.5 (27,369.4–29,560.6)	8,622.6 (7,033.0–10,415.6)	10,507.7 (9,797.6–11,223.4)	29.6 (26.8–32.3)	1,058.5 (934.5–1,196.3)	0.1 (0.1–0.2)	183.1 (141.0–226.6)	0.0 (0.0–0.0)	963.3 (830.0–1,138.5)

© 2017 Global Burden of Disease Child and Adolescent Health Collaboration. JAMA Pediatrics.

eTable 3. Prevalent Cases, Rates (per 100,000 population), Years Lived with Disability (YLDs), and Cumulative Percent Change with 95% Uncertainty Interval (UI) for the Top 10 Global Causes of YLDs in Children and Adolescents in 195 Countries and Territories, Aged 0 to 19 Years, Both Sexes, 1990 and 2015. *Note: best viewed by enlarging in browser.*

Location	Measure	All causes	Iron-deficiency anemia	Skin and subcutaneous diseases	Asthma	Hemoglobinopathies and hemolytic anemias	Diarrheal diseases	Congenital anomalies	Protein-energy malnutrition	Epilepsy	Malaria	Neonatal preterm birth complications
Portugal	Prevalence Rate: Cumulative change (%)	-0.0 (-2.2 to 2.1)	12.9 (-0.1 to 26.1)	2.5 (-2.4 to 7.8)	-14.8 (-25.4 to 1.6)	-13.0 (-20.6 to -4.8)	-38.5 (-45.9 to -30.6)	4.0 (-11.8 to 21.4)	-80.1 (-86.3 to -72.3)	-28.0 (-46.8 to -6.5)	0.0 (-0.0 to 0.0)	-13.3 (-28.2 to 3.9)
Portugal	YLDs Number: 1990	161,357 (122,212–204,257)	20,369 (14,266–27,782)	17,865 (12,224–24,943)	13,959 (9,222–19,219)	2,209 (1,528–3,010)	189 (129–258)	3,023 (1,994–4,210)	2 (1–3)	2,055 (1,313–2,882)	0 (0–0)	3,393 (2,632–4,243)
Portugal	YLDs Number: 2015	106,375 (80,822–135,461)	17,877 (12,429–24,295)	12,848 (8,858–17,918)	8,605 (5,627–12,045)	1,485 (1,030–2,005)	89 (62–123)	2,214 (1,421–3,201)	0 (0–0)	633 (384–931)	0 (0–0)	1,634 (1,232–2,065)
Portugal	YLDs Number: Cumulative change (%)	-34.1 (-36.1 to -32.2)	-12.0 (-22.1 to -2.0)	-28.1 (-29.6 to -26.4)	-38.2 (-45.8 to -25.8)	-32.6 (-38.5 to -25.9)	-52.3 (-61.2 to -41.4)	-25.9 (-40.5 to 2.4)	-84.3 (-90.9 to -74.6)	-68.7 (-79.2 to -56.9)	-20.5 (-28.8 to -11.5)	-51.8 (-57.1 to -46.2)
Portugal	YLDs Rate: 1990	5,240.6 (3,967.0–6,624.2)	748.2 (523.9–1,021.0)	587.9 (405.3–822.0)	462.8 (305.1–640.6)	76.2 (52.7–104.4)	8.0 (5.5–11.0)	104.5 (68.7–145.6)	0.1 (0.0–0.2)	70.1 (44.8–98.0)	0.0 (0.0–0.0)	116.2 (90.2–145.0)
Portugal	YLDs Rate: 2015	4,926.7 (3,753.4–6,289.9)	885.4 (612.0–1,213.5)	597.3 (410.7–830.5)	393.2 (258.5–552.5)	70.0 (48.3–94.6)	4.9 (3.4–6.8)	105.6 (67.8–152.2)	0.0 (0.0–0.0)	29.9 (18.1–44.0)	0.0 (0.0–0.0)	77.1 (58.2–97.3)
Portugal	YLDs Rate: Cumulative change (%)	-6.0 (-9.1 to -3.0)	18.7 (3.7–34.0)	1.6 (-0.6 to 3.7)	-14.8 (-25.3 to 1.8)	-7.9 (-16.1 to 1.9)	-38.2 (-50.2 to -24.0)	2.3 (-17.9 to 41.8)	-78.5 (-88.0 to -64.4)	-56.6 (-71.0 to -40.6)	2.3 (-9.6 to 14.7)	-33.6 (-40.9 to -25.8)
Spain	Prevalence Number: 1990	9,652,296 (9,564,760–9,752,736)	3,001,130 (2,846,873–3,177,078)	3,268,237 (3,122,414–3,425,341)	927,318 (795,856–1,062,367)	955,572 (880,493–1,031,202)	2,471 (2,295–2,658)	108,012 (96,693–122,230)	10 (6–14)	25,580 (20,291–31,293)	0 (0–0)	87,771 (77,238–102,955)
Spain	Prevalence Number: 2015	7,879,269 (7,771,220–7,981,137)	2,803,424 (2,694,142–2,918,986)	2,512,784 (2,405,057–2,634,374)	684,631 (560,993–825,946)	704,467 (643,438–769,773)	1,855 (1,696–2,023)	104,059 (92,190–118,429)	4 (2–7)	21,367 (16,653–26,560)	0 (0–0)	65,172 (57,377–75,801)
Spain	Prevalence Number: Cumulative change (%)	-18.4 (-19.7 to -17.1)	-6.5 (-10.9 to -0.7)	-23.1 (-27.7 to -18.4)	-26.2 (-34.0 to -17.7)	-26.1 (-34.5 to -17.7)	-24.9 (-29.4 to -20.7)	-3.2 (-17.9 to 13.5)	-53.0 (-65.6 to -37.1)	-15.5 (-36.4 to 8.6)	-7.0 (-11.8 to -1.5)	-25.2 (-39.0 to -10.4)
Spain	Prevalence Rate: 1990	84,989.1 (84,066.9–86,065.7)	29,476.1 (27,478.6–31,711.3)	26,850.8 (25,727.8–28,123.6)	7,825.4 (6,702.7–9,001.5)	8,632.9 (7,959.7–9,313.2)	27.4 (25.4–29.7)	972.8 (870.5–1,099.5)	0.1 (0.1–0.2)	231.8 (183.7–284.1)	0.0 (0.0–0.0)	895.2 (797.0–1,033.3)
Spain	Prevalence Rate: 2015	85,614.3 (84,484.6–86,686.7)	31,125.7 (29,836.4–32,552.7)	27,526.1 (26,343.1–28,850.7)	7,315.4 (6,009.7–8,790.5)	7,707.4 (7,036.5–8,417.2)	21.4 (19.5–23.4)	1,135.2 (1,006.3–1,292.0)	0.1 (0.0–0.1)	232.7 (181.4–288.7)	0.0 (0.0–0.0)	753.1 (668.7–867.9)
Spain	Prevalence Rate: Cumulative change (%)	0.7 (-1.0 to 2.4)	5.7 (-0.1 to 14.4)	2.6 (-3.3 to 8.6)	-6.5 (-15.6 to 3.8)	-10.5 (-20.6 to -0.5)	-21.9 (-27.1 to -17.5)	17.2 (-0.7 to 37.8)	-50.4 (-64.6 to -32.7)	1.6 (-23.3 to 30.8)	0.0 (-0.0 to 0.0)	-15.4 (-29.4 to 0.3)
Spain	YLDs Number: 1990	621,581 (473,789–789,242)	114,703 (80,742–155,308)	63,758 (43,260–89,325)	42,195 (28,226–57,452)	8,354 (5,964–11,155)	409 (286–548)	11,355 (8,025–15,393)	1 (0–2)	6,939 (4,519–9,600)	0 (0–0)	10,041 (7,776–12,608)
Spain	YLDs Number: 2015	469,123 (356,713–597,598)	110,608 (78,764–148,435)	50,126 (34,294–70,277)	31,212 (20,372–42,897)	6,524 (4,661–8,718)	307 (210–424)	10,884 (6,855–15,699)	0 (0–0)	3,479 (2,120–5,143)	0 (0–0)	6,182 (4,821–7,804)
Spain	YLDs Number: Cumulative change (%)	-24.5 (-26.5 to -22.2)	-3.4 (-10.1 to 5.3)	-21.3 (-23.4 to -19.0)	-26.0 (-33.9 to -17.1)	-21.8 (-27.8 to -15.1)	-24.3 (-39.5 to -7.6)	-3.9 (-25.2 to 31.1)	-52.9 (-65.5 to -37.2)	-49.2 (-65.3 to -29.4)	-3.5 (-10.6 to 5.1)	-38.4 (-45.2 to -31.2)
Spain	YLDs Rate: 1990	5,279.5 (4,008.3–6,735.6)	1,133.7 (792.7–1,547.7)	536.9 (366.8–746.5)	356.3 (238.9–484.7)	77.3 (55.3–103.3)	4.5 (3.1–6.1)	102.4 (72.2–139.2)	0.0 (0.0–0.0)	63.0 (41.5–86.9)	0.0 (0.0–0.0)	89.3 (69.4–112.6)
Spain	YLDs Rate: 2015	5,118.8 (3,897.8–6,524.6)	1,220.3 (866.6–1,639.7)	546.4 (372.3–767.0)	333.5 (217.9–457.8)	71.3 (50.8–95.3)	3.5 (2.4–4.9)	118.7 (74.9–171.4)	0.0 (0.0–0.0)	37.9 (23.1–56.0)	0.0 (0.0–0.0)	67.2 (52.4–84.8)
Spain	YLDs Rate: Cumulative change (%)	-3.0 (-5.6 to 0.2)	8.0 (-1.1 to 19.3)	1.7 (-0.7 to 4.2)	-6.4 (-15.7 to 4.1)	-7.7 (-14.5 to 0.6)	-21.3 (-37.1 to -3.5)	16.3 (-9.4 to 59.0)	-50.2 (-64.4 to -32.5)	-39.1 (-58.5 to -15.2)	2.4 (-4.7 to 11.8)	-24.7 (-32.9 to -16.2)
Sweden	Prevalence Number: 1990	1,657,322 (1,640,618–1,675,054)	418,651 (398,797–438,487)	616,885 (600,736–633,149)	135,031 (119,908–152,392)	269,853 (259,251–281,983)	365 (338–397)	36,559 (31,686–42,341)	0 (0–0)	5,030 (4,002–6,096)	0 (0–0)	19,804 (17,650–22,398)
Sweden	Prevalence Number: 2015	1,769,647 (1,748,176–1,792,928)	497,959 (470,000–525,538)	639,407 (624,630–654,029)	122,621 (100,430–146,312)	249,758 (240,689–259,853)	461 (429–502)	36,885 (32,179–42,951)	0 (0–0)	4,921 (3,980–5,995)	0 (0–0)	19,817 (17,505–22,667)
Sweden	Prevalence Number: Cumulative change (%)	6.8 (5.1–8.4)	19.0 (11.3–27.7)	3.7 (0.5–7.1)	-9.3 (-19.2 to 3.2)	-7.4 (-12.3 to -2.3)	26.4 (19.7–34.3)	1.6 (-16.6 to 23.5)	-37.3 (-52.6 to -18.4)	-1.2 (-21.8 to 22.7)	8.6 (6.7–10.6)	0.4 (-11.8 to 14.3)
Sweden	Prevalence Rate: 1990	78,870.2 (78,051.6–79,760.8)	20,344.8 (19,348.2–21,325.8)	28,764.5 (28,033.9–29,498.9)	6,337.3 (5,623.0–7,143.4)	12,907.0 (12,402.5–13,483.0)	17.7 (16.4–19.2)	1,747.8 (1,515.4–2,024.0)	0.0 (0.0–0.0)	238.6 (189.8–289.1)	0.0 (0.0–0.0)	938.6 (836.0–1,062.8)
Sweden	Prevalence Rate: 2015	81,129.4 (80,139.5–82,192.7)	23,082.2 (21,831.8–24,309.3)	29,281.4 (28,605.4–29,947.8)	5,638.3 (4,606.0–6,735.3)	11,478.4 (11,061.5–11,938.5)	21.1 (19.6–22.9)	1,692.0 (1,475.6–1,970.3)	0.0 (0.0–0.0)	225.5 (182.0–275.3)	0.0 (0.0–0.0)	906.0 (799.8–1,036.6)
Sweden	Prevalence Rate: Cumulative change (%)	2.9 (1.2–4.5)	13.5 (6.3–21.9)	1.8 (-1.3 to 5.1)	-11.1 (-20.9 to 1.3)	-11.0 (-15.8 to -6.1)	19.1 (12.8–26.7)	-2.5 (-20.0 to 18.4)	-38.3 (-53.0 to -20.4)	-4.6 (-24.4 to 18.7)	0.0 (-0.0 to 0.0)	-3.2 (-15.0 to 10.4)
Sweden	YLDs Number: 1990	99,754 (76,026–126,322)	14,298 (10,049–19,354)	14,068 (9,793–19,383)	6,159 (4,182–8,385)	2,961 (2,139–3,921)	60 (42–83)	3,514 (2,266–4,991)	0 (0–0)	1,391 (928–1,924)	0 (0–0)	1,883 (1,403–2,458)
Sweden	YLDs Number: 2015	103,760 (79,352–130,703)	17,905 (12,598–23,923)	14,693 (10,174–20,292)	5,588 (3,656–7,787)	2,142 (1,526–2,865)	76 (53–105)	5,002 (2,660–10,254)	0 (0–0)	803 (504–1,155)	0 (0–0)	1,703 (1,278–2,222)
Sweden	YLDs Number: Cumulative change (%)	4.0 (1.2–9.1)	25.4 (16.2–36.1)	4.4 (2.5–6.4)	-9.2 (-19.3 to 3.4)	-27.7 (-32.3 to -23.4)	26.9 (2.9–52.4)	39.4 (1.5–150.7)	-37.4 (-52.6 to -18.5)	-41.7 (-58.3 to -22.3)	10.4 (-0.9 to 24.1)	-9.5 (-16.9 to -1.6)
Sweden	YLDs Rate: 1990	4,680.3 (3,563.5–5,939.0)	706.1 (497.2–955.9)	665.9 (465.0–915.8)	289.2 (195.3–393.2)	146.1 (105.6–193.6)	2.9 (2.1–4.0)	168.1 (108.3–238.6)	0.0 (0.0–0.0)	66.0 (44.2–91.4)	0.0 (0.0–0.0)	90.1 (67.2–117.7)
Sweden	YLDs Rate: 2015	4,757.1 (3,639.8–5,999.2)	839.8 (592.0–1,121.6)	673.7 (465.7–928.9)	257.0 (168.8–358.7)	101.3 (72.3–134.8)	3.5 (2.4–4.8)	229.5 (122.1–470.6)	0.0 (0.0–0.0)	36.8 (23.2–53.1)	0.0 (0.0–0.0)	78.2 (58.7–102.0)
Sweden	YLDs Rate: Cumulative change (%)	1.7 (-1.2 to 6.5)	19.1 (10.5–29.3)	1.1 (-0.7 to 3.0)	-11.1 (-21.2 to 1.4)	-30.7 (-35.1 to -26.6)	19.7 (-3.0 to 43.8)	33.7 (-2.8 to 140.7)	-38.3 (-53.0 to -20.4)	-43.7 (-59.7 to -25.0)	0.6 (-9.4 to 12.7)	-13.2 (-20.1 to -5.5)
Switzerland	Prevalence Number: 1990	1,310,123 (1,287,083–1,332,420)	347,109 (329,025–369,866)	465,032 (444,213–485,726)	122,319 (103,524–142,609)	250,034 (223,570–288,478)	445 (411–481)	31,765 (27,954–36,473)	1 (1–2)	4,351 (3,359–5,383)	0 (0–0)	14,434 (12,716–16,548)
Switzerland	Prevalence Number: 2015	1,377,492 (1,351,416–1,400,263)	382,617 (358,883–419,528)	499,232 (477,672–522,996)	112,071 (92,234–132,735)	239,047 (209,090–286,569)	468 (429–508)	33,696 (29,225–39,781)	0 (0–0)	3,523 (2,735–4,466)	0 (0–0)	13,714 (12,093–15,820)
Switzerland	Prevalence Number: Cumulative change (%)	5.2 (2.7–7.5)	10.4 (1.9–23.8)	7.4 (1.3–14.2)	-8.1 (-20.3 to 5.7)	-3.9 (-21.1 to 17.6)	5.1 (1.0–9.6)	6.7 (-12.9 to 30.1)	-69.1 (-77.0 to -60.0)	-17.8 (-38.0 to 6.7)	4.7 (4.3–5.1)	-4.6 (-18.0 to 12.1)
Switzerland	Prevalence Rate: 1990	82,872.2 (81,353.9–84,332.2)	22,612.1 (21,443.4–24,100.5)	28,692.3 (27,457.4–29,949.9)	7,626.1 (6,414.5–8,922.4)	15,912.3 (14,223.8–18,346.8)	29.6 (27.3–32.0)	2,020.5 (1,778.9–2,319.9)	0.1 (0.1–0.2)	277.0 (213.5–343.2)	0.0 (0.0–0.0)	934.1 (824.4–1,069.8)
Switzerland	Prevalence Rate: 2015	82,826.0 (81,185.3–84,236.3)	23,672.2 (22,219.9–26,033.1)	29,193.0 (27,966.3–30,559.0)	6,708.9 (5,505.2–7,991.4)	14,457.9 (12,651.7–17,336.5)	29.5 (27.0–32.0)	2,036.7 (1,765.9–2,405.5)	0.0 (0.0–0.0)	213.2 (165.7–269.7)	0.0 (0.0–0.0)	840.3 (742.0–967.6)
Switzerland	Prevalence Rate: Cumulative change (%)	-0.0 (-2.5 to 2.2)	4.9 (-3.4 to 18.0)	1.8 (-4.0 to 8.1)	-11.8 (-23.8 to 1.9)	-8.7 (-25.0 to 11.8)	-0.5 (-4.4 to 3.8)	1.4 (-17.2 to 23.8)	-70.2 (-77.7 to -61.3)	-21.9 (-41.1 to 1.6)	0.0 (-0.0 to 0.0)	-9.6 (-22.2 to 5.9)
Switzerland	YLDs Number: 1990	84,899 (65,034–107,425)	11,994 (8,378–16,526)	10,247 (7,077–14,119)	5,565 (3,698–7,618)	3,124 (2,047–4,521)	73 (50–101)	3,057 (1,794–4,615)	0 (0–0)	1,236 (801–1,713)	0 (0–0)	1,522 (1,144–1,957)
Switzerland	YLDs Number: 2015	85,076 (64,741–107,690)	13,608 (9,442–18,938)	10,933 (7,566–15,217)	5,104 (3,382–7,001)	2,882 (1,880–4,079)	77 (52–103)	3,494 (2,004–5,407)	0 (0–0)	581 (365–845)	0 (0–0)	1,211 (912–1,567)
Switzerland	YLDs Number: Cumulative change (%)	0.2 (-2.5 to 4.0)	13.8 (2.7–34.3)	6.7 (4.1–9.1)	-8.0 (-20.7 to 6.0)	-7.5 (-16.2 to 2.4)	5.7 (-13.9 to 27.8)	16.2 (-9.0 to 69.9)	-69.2 (-77.0 to -60.1)	-52.1 (-68.3 to -33.7)	5.7 (-3.4 to 18.8)	-20.3 (-28.3 to -10.9)
Switzerland	YLDs Rate: 1990	5,272.7 (4,043.1–6,674.3)	789.6 (551.5–1,088.4)	640.9 (443.0–880.9)	347.1 (230.7–475.6)	201.5 (132.6–291.1)	4.9 (3.4–6.8)	194.6 (114.2–293.9)	0.0 (0.0–0.0)	78.8 (51.2–109.2)	0.0 (0.0–0.0)	96.7 (72.8–124.2)
Switzerland	YLDs Rate: 2015	5,024.5 (3,821.9–6,365.3)	850.3 (589.6–1,189.0)	648.9 (451.1–899.3)	305.7 (202.4–420.6)	178.0 (115.3–253.4)	4.9 (3.3–6.5)	211.3 (121.2–327.0)	0.0 (0.0–0.0)	35.2 (22.2–51.2)	0.0 (0.0–0.0)	73.1 (55.2–94.2)
Switzerland	YLDs Rate: Cumulative change (%)	-4.7 (-7.5 to -0.8)	8.0 (-2.8 to 28.2)	1.3 (-1.1 to 3.5)	-11.6 (-24.0 to 2.0)	-11.4 (-19.6 to -1.4)	0.0 (-18.6 to 21.5)	10.4 (-13.5 to 61.2)	-70.3 (-77.8 to -61.5)	-54.5 (-69.8 to -36.9)	1.3 (-7.4 to 14.0)	-24.3 (-31.7 to -15.3)
United Kingdom	Prevalence Number: 1990	12,414,681 (12,365,080–12,466,630)	2,994,988 (2,937,324–3,054,175)	4,288,049 (4,240,233–4,333,942)	2,549,303 (2,188,231–2,914,410)	2,288,909 (2,252,581–2,326,284)	17,975 (17,753–18,231)	166,715 (157,166–177,080)	5 (4–8)	55,200 (48,235–62,697)	0 (0–0)	137,364 (126,666–149,626)
United Kingdom	Prevalence Number: 2015	12,154,453 (12,102,158–12,210,253)	3,070,551 (2,998,335–3,146,784)	4,297,581 (4,251,139–4,345,499)	2,026,380 (1,778,189–2,278,327)	2,037,443 (2,006,845–2,066,247)	15,495 (15,236–15,773)	189,896 (178,350–201,864)	2 (2–4)	55,092 (47,637–63,740)	0 (0–0)	134,800 (124,023–146,084)
United Kingdom	Prevalence Number: Cumulative change (%)	-2.1 (-2.6 to -1.6)	2.5 (-0.5 to 5.6)	0.2 (-1.1 to 1.6)	-20.4 (-23.7 to -17.3)	-11.0 (-12.5 to -9.5)	-13.8 (-14.3 to -13.2)	14.0 (6.6–21.7)	-49.7 (-54.9 to -44.1)	-0.1 (-9.5 to 9.6)	-1.4 (-2.4 to -0.1)	-1.8 (-7.0 to 3.6)
United Kingdom	Prevalence Rate: 1990	83,815.7 (83,471.9–84,177.9)	20,567.6 (20,174.3–20,971.4)	28,516.1 (28,200.5–28,814.3)	17,206.3 (14,745.4–19,708.8)	15,499.7 (15,254.7–15,752.8)	123.4 (121.8–125.1)	1,128.1 (1,063.7–1,198.3)	0.0 (0.0–0.1)	371.4 (325.0–421.2)	0.0 (0.0–0.0)	936.2 (863.8–1,019.0)
United Kingdom	Prevalence Rate: 2015	83,729.7 (83,361.6–84,111.7)	21,525.2 (21,043.7–22,037.0)	29,082.1 (28,774.0–29,396.7)	14,125.3 (12,394.5–15,930.5)	14,019.2 (13,809.3–14,219.1)	104.6 (102.9–106.4)	1,304.9 (1,225.4–1,387.1)	0.0 (0.0–0.0)	377.5 (325.7–437.4)	0.0 (0.0–0.0)	914.9 (841.0–992.3)
United Kingdom	Prevalence Rate: Cumulative change (%)	-0.1 (-0.6 to 0.4)	4.7 (1.6–7.7)	2.0 (0.7–3.3)	-17.8 (-21.1 to -14.6)	-9.5 (-11.1 to -8.0)	-15.2 (-15.7 to -14.7)	15.7 (8.2–23.6)	-49.7 (-54.8 to -44.1)	1.7 (-7.9 to 11.7)	0.0 (-0.0 to 0.0)	-2.2 (-7.3 to 3.1)
United Kingdom	YLDs Number: 1990	820,338 (621,506–1,043,365)	106,908 (76,173–144,141)	97,864 (67,709–135,163)	116,006 (78,602–157,680)	25,376 (18,266–33,886)	2,960 (2,132–3,892)	17,138 (11,860–23,427)	0 (0–1)	15,065 (10,705–19,527)	0 (0–0)	15,129 (11,757–18,922)

© 2017 Global Burden of Disease Child and Adolescent Health Collaboration. JAMA Pediatrics.

eTable 3. Prevalent Cases, Rates (per 100,000 population), Years Lived with Disability (YLDs), and Cumulative Percent Change with 95% Uncertainty Interval (UI) for the Top 10 Global Causes of YLDs in Children and Adolescents in 195 Countries and Territories, Aged 0 to 19 Years, Both Sexes, 1990 and 2015. *Note: best viewed by enlarging in browser.*

Location	Measure	All causes	Iron-deficiency anemia	Skin and subcutaneous diseases	Asthma	Hemoglobinopathies and hemolytic anemias	Diarrheal diseases	Congenital anomalies	Protein-energy malnutrition	Epilepsy	Malaria	Neonatal preterm birth complications
United Kingdom	YLDs Number: 2015	764,556 (577,770–972,101)	110,085 (78,803–148,967)	96,986 (67,125–134,172)	92,268 (62,867–125,547)	21,449 (15,491–28,861)	2,551 (1,849–3,335)	22,129 (14,330–31,606)	0 (0–0)	8,977 (5,950–12,618)	0 (0–0)	12,880 (9,915–16,297)
United Kingdom	YLDs Number: Cumulative change (%)	-6.8 (-8.0 to -5.5)	3.0 (-0.3 to 6.5)	-0.9 (-2.4 to 0.5)	-20.4 (-23.6 to -17.4)	-15.5 (-18.6 to -12.4)	-13.8 (-16.0 to -11.5)	28.7 (13.4–53.4)	-49.7 (-54.9 to -44.2)	-40.6 (-52.1 to -29.0)	-2.0 (-6.9 to 3.4)	-14.9 (-18.6 to -11.3)
United Kingdom	YLDs Rate: 1990	5,490.8 (4,153.5–6,986.7)	742.8 (529.4–1,000.0)	656.4 (455.3–906.3)	783.1 (530.1–1,067.0)	176.1 (127.0–235.0)	20.3 (14.6–26.7)	116.0 (80.3–158.6)	0.0 (0.0–0.0)	101.4 (72.1–131.6)	0.0 (0.0–0.0)	102.4 (79.6–128.1)
United Kingdom	YLDs Rate: 2015	5,240.7 (3,960.8–6,667.6)	788.1 (564.5–1,065.8)	662.2 (458.9–913.8)	643.2 (437.0–871.9)	154.0 (111.4–206.9)	17.2 (12.5–22.5)	152.1 (98.5–217.0)	0.0 (0.0–0.0)	61.5 (40.7–86.9)	0.0 (0.0–0.0)	88.8 (68.5–112.3)
United Kingdom	YLDs Rate: Cumulative change (%)	-4.5 (-5.8 to -3.2)	6.1 (2.8–9.6)	0.8 (-0.6 to 2.3)	-17.8 (-21.1 to -14.7)	-12.6 (-15.8 to -9.4)	-15.3 (-17.5 to -13.0)	30.6 (15.1–55.7)	-49.7 (-54.8 to -44.2)	-39.5 (-51.2 to -27.6)	1.2 (-3.8 to 6.4)	-13.3 (-17.1 to -9.7)
England	Prevalence Number: 1990	10,199,045 (10,153,332–10,248,653)	2,502,581 (2,447,381–2,560,514)	3,530,116 (3,486,273–3,574,183)	2,070,091 (1,779,253–2,374,568)	1,830,046 (1,795,717–1,863,365)	2,234 (2,089–2,399)	135,712 (127,441–145,311)	4 (3–6)	45,679 (39,779–52,000)	0 (0–0)	114,126 (104,743–124,863)
England	Prevalence Number: 2015	10,245,276 (10,196,004–10,299,252)	2,611,898 (2,544,529–2,682,740)	3,622,148 (3,579,410–3,667,915)	1,656,520 (1,448,097–1,876,483)	1,686,539 (1,660,005–1,714,504)	2,620 (2,429–2,816)	159,107 (148,615–171,104)	2 (1–3)	46,642 (40,114–53,906)	0 (0–0)	114,464 (104,837–124,544)
England	Prevalence Number: Cumulative change (%)	0.5 (-0.1 to 1.0)	4.4 (0.8–8.0)	2.6 (1.2–4.1)	-19.9 (-23.2 to -16.9)	-7.8 (-9.6 to -6.0)	17.2 (14.7–19.8)	17.3 (8.7–26.8)	-45.7 (-51.9 to -39.4)	2.2 (-8.7 to 13.1)	1.4 (0.3–2.7)	0.4 (-5.4 to 6.3)
England	Prevalence Rate: 1990	83,426.0 (83,048.3–83,835.9)	20,809.3 (20,356.8–21,281.6)	28,458.4 (28,117.0–28,798.1)	16,951.4 (14,562.0–19,504.0)	15,009.0 (14,729.0–15,283.0)	18.5 (17.3–19.9)	1,112.1 (1,044.3–1,190.6)	0.0 (0.0–0.1)	372.3 (324.4–424.2)	0.0 (0.0–0.0)	938.3 (861.3–1,026.4)
England	Prevalence Rate: 2015	83,390.9 (82,994.4–83,830.5)	21,606.9 (21,088.8–22,172.9)	29,026.6 (28,678.7–29,394.0)	13,670.3 (11,939.9–15,528.9)	13,700.2 (13,483.3–13,924.3)	20.8 (19.3–22.3)	1,290.6 (1,205.6–1,389.0)	0.0 (0.0–0.0)	377.6 (324.0–437.5)	0.0 (0.0–0.0)	912.8 (835.6–994.4)
England	Prevalence Rate: Cumulative change (%)	-0.0 (-0.6 to 0.5)	3.9 (0.4–7.3)	2.0 (0.6–3.5)	-19.3 (-22.6 to -16.3)	-8.7 (-10.5 to -6.9)	12.2 (9.7–14.6)	16.2 (7.6–25.5)	-47.1 (-52.9 to -41.0)	1.6 (-9.4 to 12.2)	0.0 (-0.0 to 0.0)	-2.6 (-8.3 to 3.1)
England	YLDs Number: 1990	668,216 (506,590–849,637)	89,782 (64,138–121,150)	80,146 (55,422–110,703)	94,230 (63,916–128,214)	19,015 (13,687–25,473)	372 (265–498)	13,576 (9,358–18,565)	0 (0–0)	12,489 (8,821–16,405)	0 (0–0)	12,586 (9,740–15,765)
England	YLDs Number: 2015	637,308 (481,403–810,975)	94,004 (67,002–127,300)	81,451 (56,353–112,697)	75,448 (51,098–102,739)	17,033 (12,267–23,044)	435 (308–582)	18,198 (11,685–26,087)	0 (0–0)	7,608 (5,001–10,650)	0 (0–0)	10,982 (8,451–13,933)
England	YLDs Number: Cumulative change (%)	-4.6 (-6.0 to -3.3)	4.7 (0.9–8.6)	1.6 (-0.1 to 3.2)	-19.8 (-23.2 to -16.9)	-10.4 (-14.3 to -6.6)	17.1 (7.1–28.3)	33.6 (17.1–59.4)	-45.8 (-51.9 to -39.5)	-39.2 (-51.2 to -26.8)	0.6 (-5.3 to 6.9)	-12.8 (-16.9 to -8.8)
England	YLDs Rate: 1990	5,423.2 (4,117.3–6,901.8)	755.8 (539.7–1,020.2)	651.5 (451.5–899.3)	771.7 (522.6–1,052.2)	160.2 (115.5–214.5)	3.1 (2.2–4.1)	111.3 (76.7–152.2)	0.0 (0.0–0.0)	101.8 (72.1–133.8)	0.0 (0.0–0.0)	103.2 (79.9–129.2)
England	YLDs Rate: 2015	5,172.5 (3,912.8–6,584.6)	794.3 (567.0–1,076.8)	657.7 (455.7–908.0)	622.7 (421.0–846.9)	144.4 (104.0–194.9)	3.5 (2.4–4.6)	147.6 (94.7–211.6)	0.0 (0.0–0.0)	61.6 (40.5–86.3)	0.0 (0.0–0.0)	89.4 (68.8–113.3)
England	YLDs Rate: Cumulative change (%)	-4.6 (-5.9 to -3.3)	5.1 (1.5–8.9)	0.9 (-0.7 to 2.6)	-19.2 (-22.6 to -16.2)	-9.9 (-13.8 to -6.0)	12.1 (2.3–22.9)	32.2 (15.8–57.8)	-47.1 (-52.9 to -41.1)	-39.6 (-51.6 to -27.3)	1.0 (-4.7 to 6.8)	-13.4 (-17.4 to -9.5)
Northern Ireland	Prevalence Number: 1990	443,855 (439,871–447,880)	101,563 (94,051–109,815)	150,726 (146,313–155,228)	68,846 (57,496–80,452)	93,326 (90,013–96,669)	136 (124–147)	6,495 (5,667–7,458)	0 (0–0)	1,894 (1,492–2,333)	0 (0–0)	4,934 (4,348–5,645)
Northern Ireland	Prevalence Number: 2015	384,837 (380,738–389,193)	95,450 (88,910–104,412)	133,793 (129,846–138,151)	44,887 (37,443–52,360)	71,236 (68,975–73,511)	159 (144–181)	6,424 (5,585–7,547)	0 (0–0)	1,687 (1,340–2,068)	0 (0–0)	4,334 (3,835–4,954)
Northern Ireland	Prevalence Number: Cumulative change (%)	-13.3 (-14.5 to -12.1)	-5.9 (-12.2 to 2.7)	-11.2 (-14.6 to -7.6)	-34.7 (-39.9 to -28.4)	-23.6 (-27.4 to -20.1)	17.2 (9.3–28.8)	-0.5 (-18.5 to 20.5)	-67.8 (-75.7 to -58.1)	-9.7 (-32.7 to 17.2)	-14.3 (-15.3 to -13.1)	-11.7 (-24.0 to 2.5)
Northern Ireland	Prevalence Rate: 1990	84,609.0 (83,844.4–85,383.4)	19,585.5 (18,147.5–21,149.1)	28,454.2 (27,620.8–29,293.7)	13,049.0 (10,888.6–15,263.0)	17,836.1 (17,204.7–18,476.2)	26.6 (24.4–28.8)	1,241.3 (1,083.2–1,425.8)	0.0 (0.0–0.1)	360.8 (284.7–443.8)	0.0 (0.0–0.0)	969.5 (856.4–1,105.8)
Northern Ireland	Prevalence Rate: 2015	85,144.8 (84,220.5–86,115.2)	21,436.6 (20,051.7–23,295.8)	29,062.0 (28,202.6–30,001.3)	9,965.6 (8,321.7–11,616.4)	15,740.6 (15,243.3–16,246.2)	34.6 (31.4–39.3)	1,418.6 (1,230.4–1,667.1)	0.0 (0.0–0.0)	371.4 (294.3–455.8)	0.0 (0.0–0.0)	950.9 (840.4–1,087.8)
Northern Ireland	Prevalence Rate: Cumulative change (%)	0.6 (-0.7 to 2.0)	9.6 (2.3–18.9)	2.2 (-1.8 to 6.2)	-23.5 (-29.6 to -16.3)	-11.7 (-16.1 to -7.6)	29.9 (21.2–42.4)	15.0 (-5.8 to 39.2)	-64.7 (-73.5 to -54.1)	4.3 (-22.1 to 35.0)	-0.0 (-0.0 to 0.0)	-1.5 (-14.8 to 14.5)
Northern Ireland	YLDs Number: 1990	28,203 (21,390–35,735)	3,537 (2,522–4,802)	3,457 (2,395–4,754)	3,130 (2,104–4,307)	1,296 (925–1,743)	22 (15–30)	1,086 (780–1,455)	0 (0–0)	513 (338–712)	0 (0–0)	536 (412–680)
Northern Ireland	YLDs Number: 2015	23,348 (17,819–29,618)	3,401 (2,415–4,672)	3,019 (2,078–4,176)	2,042 (1,340–2,836)	871 (622–1,183)	26 (18–37)	1,167 (822–1,609)	0 (0–0)	274 (174–395)	0 (0–0)	414 (315–532)
Northern Ireland	YLDs Number: Cumulative change (%)	-17.2 (-19.1 to -15.0)	-3.8 (-9.5 to 8.7)	-12.7 (-15.1 to -10.4)	-34.7 (-40.0 to -28.6)	-32.8 (-36.7 to -26.1)	16.9 (-2.8 to 39.7)	7.9 (-10.2 to 27.8)	-67.8 (-75.6 to -58.1)	-45.7 (-62.2 to -26.7)	-13.5 (-23.6 to -0.4)	-22.7 (-29.7 to -15.2)
Northern Ireland	YLDs Rate: 1990	5,342.7 (4,055.9–6,769.4)	684.8 (488.5–930.0)	655.4 (454.1–899.4)	593.5 (398.4–816.6)	249.3 (177.9–335.1)	4.4 (3.1–6.1)	207.8 (149.1–278.4)	0.0 (0.0–0.0)	97.8 (64.4–135.7)	0.0 (0.0–0.0)	102.4 (78.8–130.1)
Northern Ireland	YLDs Rate: 2015	5,134.3 (3,920.6–6,523.0)	777.9 (554.4–1,065.3)	662.2 (457.0–914.5)	453.6 (297.9–633.4)	200.2 (143.0–271.1)	5.7 (4.0–8.0)	258.0 (181.7–356.0)	0.0 (0.0–0.0)	60.5 (38.4–87.1)	0.0 (0.0–0.0)	91.7 (69.7–117.8)
Northern Ireland	YLDs Rate: Cumulative change (%)	-3.9 (-6.1 to -1.4)	13.7 (7.0–27.1)	1.0 (-1.7 to 3.7)	-23.5 (-29.6 to -16.3)	-19.7 (-24.3 to -12.3)	29.6 (7.7–54.8)	24.7 (3.6–47.8)	-64.6 (-73.5 to -54.1)	-37.3 (-56.3 to -15.2)	3.1 (-8.4 to 17.6)	-10.4 (-18.6 to -1.7)
Scotland	Prevalence Number: 1990	1,131,602 (1,121,520–1,141,354)	249,111 (230,762–266,325)	389,321 (377,274–400,054)	249,491 (211,305–290,774)	233,134 (225,235–241,820)	346 (319–375)	15,090 (13,114–17,461)	0 (0–0)	5,032 (4,011–6,131)	0 (0–0)	11,405 (10,173–13,087)
Scotland	Prevalence Number: 2015	942,320 (933,441–951,839)	219,850 (204,495–237,530)	335,816 (325,751–345,688)	186,091 (165,067–209,920)	173,404 (167,966–180,308)	349 (322–384)	14,677 (12,510–17,185)	0 (0–0)	4,249 (3,346–5,241)	0 (0–0)	10,173 (9,046–11,767)
Scotland	Prevalence Number: Cumulative change (%)	-16.7 (-17.8 to -15.6)	-11.6 (-20.7 to -1.1)	-13.7 (-17.2 to -9.9)	-25.1 (-33.4 to -16.9)	-25.6 (-29.2 to -22.0)	0.8 (-3.9 to 7.2)	-2.1 (-20.6 to 19.5)	-69.4 (-76.6 to -60.2)	-14.5 (-35.1 to 10.4)	-17.1 (-18.0 to -16.1)	-10.3 (-24.0 to 5.8)
Scotland	Prevalence Rate: 1990	85,760.0 (84,965.2–86,530.4)	19,404.6 (17,902.7–20,796.1)	28,870.9 (27,977.8–29,636.5)	18,747.2 (15,868.9–21,911.8)	17,774.7 (17,175.1–18,429.9)	27.7 (25.5–30.0)	1,150.7 (1,000.8–1,332.5)	0.1 (0.0–0.1)	380.6 (302.6–463.8)	0.0 (0.0–0.0)	898.1 (803.3–1,025.6)
Scotland	Prevalence Rate: 2015	85,377.9 (84,545.2–86,251.8)	20,547.0 (19,108.7–22,215.9)	29,481.3 (28,603.1–30,313.2)	16,841.4 (14,921.8–19,044.6)	15,776.9 (15,280.3–16,412.9)	32.6 (30.1–35.9)	1,334.3 (1,137.1–1,565.8)	0.0 (0.0–0.0)	382.6 (301.2–472.3)	0.0 (0.0–0.0)	944.5 (842.2–1,089.0)
Scotland	Prevalence Rate: Cumulative change (%)	-0.4 (-1.8 to 0.9)	6.1 (-4.6 to 18.7)	2.1 (-1.8 to 6.5)	-9.8 (-19.8 to 0.3)	-11.2 (-15.5 to -6.9)	17.7 (12.1–25.2)	16.8 (-5.4 to 42.3)	-64.2 (-72.8 to -53.1)	1.7 (-22.7 to 31.3)	0.0 (-0.0 to 0.0)	5.7 (-10.0 to 24.2)
Scotland	YLDs Number: 1990	78,570 (59,839–99,229)	8,688 (6,077–11,839)	9,141 (6,365–12,616)	11,333 (7,558–15,565)	3,182 (2,261–4,235)	57 (39–79)	1,628 (1,102–2,259)	0 (0–0)	1,368 (922–1,899)	0 (0–0)	1,254 (975–1,579)
Scotland	YLDs Number: 2015	63,214 (47,955–80,075)	7,661 (5,396–10,348)	7,772 (5,416–10,674)	8,465 (5,768–11,453)	2,188 (1,558–2,996)	58 (40–79)	1,689 (1,050–2,499)	0 (0–0)	687 (440–988)	0 (0–0)	922 (705–1,165)
Scotland	YLDs Number: Cumulative change (%)	-19.5 (-21.7 to -17.3)	-11.6 (-20.9 to -1.0)	-15.0 (-17.2 to -12.7)	-25.0 (-33.4 to -17.1)	-31.2 (-37.0 to -23.4)	1.2 (-17.2 to 22.6)	3.6 (-14.5 to 29.9)	-69.3 (-76.5 to -60.1)	-49.0 (-64.9 to -30.1)	-17.3 (-29.3 to -3.8)	-26.4 (-32.5 to -19.6)
Scotland	YLDs Rate: 1990	5,856.8 (4,457.6–7,412.1)	684.4 (478.7–934.0)	686.6 (478.8–944.2)	851.9 (567.1–1,170.1)	247.3 (175.4–330.0)	4.6 (3.2–6.4)	124.2 (84.1–172.2)	0.0 (0.0–0.0)	103.6 (69.6–143.5)	0.0 (0.0–0.0)	95.5 (74.3–120.1)
Scotland	YLDs Rate: 2015	5,620.3 (4,261.0–7,119.3)	731.4 (515.4–986.1)	694.6 (484.9–956.2)	766.4 (522.8–1,038.0)	208.0 (148.7–282.6)	5.4 (3.7–7.5)	153.7 (95.4–227.2)	0.0 (0.0–0.0)	61.9 (39.6–89.1)	0.0 (0.0–0.0)	83.9 (64.0–106.1)
Scotland	YLDs Rate: Cumulative change (%)	-4.0 (-6.7 to -1.2)	7.1 (-4.1 to 19.9)	1.1 (-1.5 to 3.9)	-9.7 (-19.9 to 0.2)	-15.8 (-22.9 to -6.6)	18.2 (-3.4 to 43.1)	23.5 (2.0–55.1)	-64.1 (-72.7 to -53.1)	-39.3 (-58.5 to -17.1)	1.6 (-12.9 to 17.3)	-12.1 (-19.2 to -4.2)
Wales	Prevalence Number: 1990	640,178 (633,679–646,688)	141,732 (130,951–153,754)	217,885 (211,504–224,182)	160,875 (139,161–182,552)	132,403 (127,440–137,028)	15,258 (15,192–15,346)	9,417 (8,209–10,855)	0 (0–0)	2,592 (2,038–3,200)	0 (0–0)	6,898 (6,108–7,832)
Wales	Prevalence Number: 2015	582,019 (575,896–588,448)	143,352 (130,815–162,467)	205,822 (199,845–211,844)	138,881 (124,242–155,312)	106,263 (102,573–109,893)	12,366 (12,305–12,448)	9,686 (8,405–11,323)	0 (0–0)	2,513 (1,943–3,083)	0 (0–0)	5,828 (5,093–6,693)
Wales	Prevalence Number: Cumulative change (%)	-9.1 (-10.3 to -7.8)	1.3 (-8.1 to 17.6)	-5.5 (-9.2 to -1.8)	-13.5 (-20.0 to -6.5)	-19.7 (-23.4 to -15.7)	-19.0 (-19.2 to -18.7)	3.5 (-15.2 to 25.9)	-56.3 (-66.5 to -44.6)	-1.9 (-27.5 to 25.5)	-11.3 (-12.4 to -10.3)	-15.2 (-27.3 to -1.5)
Wales	Prevalence Rate: 1990	86,252.9 (85,359.4–87,142.4)	19,454.1 (17,958.7–21,107.9)	28,886.8 (28,029.7–29,724.8)	21,581.7 (18,636.2–24,518.6)	17,898.4 (17,227.5–18,526.7)	2,117.4 (2,108.1–2,129.8)	1,272.7 (1,109.0–1,467.2)	0.0 (0.0–0.0)	348.1 (273.7–429.4)	0.0 (0.0–0.0)	946.1 (838.6–1,072.3)
Wales	Prevalence Rate: 2015	86,350.5 (85,442.0–87,341.0)	21,995.2 (20,081.9–24,957.3)	29,476.1 (28,672.8–30,318.4)	20,643.6 (18,467.8–23,059.9)	15,829.3 (15,288.3–16,368.1)	1,896.9 (1,887.6–1,909.4)	1,441.4 (1,251.0–1,683.6)	0.0 (0.0–0.0)	370.2 (286.0–455.1)	0.0 (0.0–0.0)	885.3 (775.1–1,015.0)
Wales	Prevalence Rate: Cumulative change (%)	0.1 (-1.3 to 1.6)	13.3 (2.6–31.7)	2.1 (-1.8 to 6.2)	-4.1 (-11.3 to 3.9)	-11.5 (-15.5 to -7.1)	-10.4 (-10.7 to -10.2)	14.0 (-6.6 to 38.7)	-51.4 (-62.9 to -37.9)	7.6 (-20.5 to 37.3)	0.0 (-0.0 to 0.0)	-6.1 (-19.3 to 8.8)
Wales	YLDs Number: 1990	45,347 (34,195–57,785)	4,900 (3,405–6,741)	5,119 (3,576–7,052)	7,311 (4,938–9,921)	1,883 (1,327–2,533)	2,507 (1,814–3,307)	847 (585–1,168)	0 (0–0)	694 (466–939)	0 (0–0)	751 (580–948)
Wales	YLDs Number: 2015	40,685 (30,975–51,744)	5,017 (3,415–6,955)	4,742 (3,298–6,501)	6,312 (4,308–8,478)	1,354 (950–1,852)	2,030 (1,475–2,650)	1,073 (688–1,579)	0 (0–0)	406 (257–599)	0 (0–0)	561 (425–719)
Wales	YLDs Number: Cumulative change (%)	-10.3 (-12.8 to -6.8)	2.7 (-8.6 to 28.2)	-7.4 (-9.8 to -5.1)	-13.4 (-20.2 to -6.2)	-28.0 (-34.8 to -17.3)	-19.0 (-21.0 to -16.8)	26.4 (6.0–53.7)	-56.3 (-66.5 to -44.6)	-40.6 (-59.5 to -17.5)	-8.0 (-19.5 to 6.4)	-25.3 (-32.3 to -18.2)

© 2017 Global Burden of Disease Child and Adolescent Health Collaboration. JAMA Pediatrics.

eTable 3. Prevalent Cases, Rates (per 100,000 population), Years Lived with Disability (YLDs), and Cumulative Percent Change with 95% Uncertainty Interval (UI) for the Top 10 Global Causes of YLDs in Children and Adolescents in 195 Countries and Territories, Aged 0 to 19 Years, Both Sexes, 1990 and 2015. *Note: best viewed by enlarging in browser.*

Location	Measure	All causes	Iron-deficiency anemia	Skin and subcutaneous diseases	Asthma	Hemoglobinopathies and hemolytic anemias	Diarrheal diseases	Congenital anomalies	Protein-energy malnutrition	Epilepsy	Malaria	Neonatal preterm birth complications
Wales	YLDs Rate: 1990	6,055.7 (4,564.5–7,724.3)	677.9 (472.0–932.4)	685.2 (479.1–942.8)	981.0 (661.6–1,330.1)	258.9 (182.6–348.7)	348.1 (252.0–459.2)	114.6 (79.1–158.1)	0.0 (0.0–0.0)	93.2 (62.8–126.3)	0.0 (0.0–0.0)	101.5 (78.4–127.8)
Wales	YLDs Rate: 2015	5,939.3 (4,516.4–7,562.6)	785.4 (537.6–1,084.2)	692.3 (481.4–944.3)	938.5 (642.8–1,258.5)	211.9 (148.5–289.7)	311.6 (226.2–406.5)	159.8 (102.4–234.7)	0.0 (0.0–0.0)	60.0 (37.8–88.5)	0.0 (0.0–0.0)	83.5 (63.4–106.9)
Wales	YLDs Rate: Cumulative change (%)	-1.9 (-4.7 to 2.0)	16.2 (3.3–44.4)	1.0 (-1.4 to 3.6)	-4.1 (-11.7 to 4.1)	-18.1 (-26.1 to -6.2)	-10.5 (-12.7 to -8.1)	39.1 (16.7–69.5)	-51.5 (-62.7 to -38.1)	-34.9 (-55.6 to -9.6)	5.6 (-7.4 to 21.1)	-17.7 (-25.4 to -9.8)
Southern Latin America	Prevalence Number: 1990	16,429,823 (16,292,321–16,575,431)	4,877,714 (4,649,048–5,101,264)	5,238,927 (5,118,827–5,369,451)	1,954,984 (1,712,304–2,172,854)	1,328,959 (1,276,936–1,389,376)	23,656 (21,922–25,826)	279,302 (250,637–312,055)	2,253 (1,515–3,193)	79,490 (67,247–92,783)	63 (43–85)	167,337 (150,059–187,091)
Southern Latin America	Prevalence Number: 2015	17,481,603 (17,322,529–17,637,847)	5,289,625 (4,989,320–5,495,184)	5,828,636 (5,683,472–5,962,618)	1,743,887 (1,445,499–2,066,992)	1,305,899 (1,250,977–1,363,572)	14,815 (13,633–16,171)	317,631 (284,490–355,090)	325 (218–474)	81,606 (66,293–97,331)	0 (0–0)	163,368 (146,248–182,402)
Southern Latin America	Prevalence Number: Cumulative change (%)	6.4 (5.1–7.6)	8.6 (-1.8 to 16.0)	11.3 (7.8–14.6)	-10.9 (-20.7 to -1.8)	-1.7 (-7.2 to 4.3)	-37.3 (-42.3 to -32.9)	14.1 (-0.9 to 31.7)	-85.4 (-88.4 to -82.1)	3.2 (-16.1 to 23.8)	-100.0 (-100.0 to -100.0)	-2.1 (-12.6 to 8.5)
Southern Latin America	Prevalence Rate: 1990	86,661.2 (85,941.6–87,425.7)	25,538.1 (24,343.2–26,703.3)	27,749.4 (27,112.7–28,453.4)	10,306.4 (9,030.8–11,440.4)	6,996.9 (6,722.4–7,314.9)	123.3 (114.3–134.6)	1,471.8 (1,320.9–1,643.8)	11.7 (7.8–16.5)	419.6 (354.8–489.5)	0.3 (0.2–0.4)	876.9 (785.8–980.6)
Southern Latin America	Prevalence Rate: 2015	85,920.9 (85,121.4–86,693.4)	26,396.2 (24,870.8–27,443.1)	28,373.6 (27,671.5–29,028.4)	8,558.7 (7,081.2–10,136.2)	6,437.7 (6,166.3–6,720.5)	75.1 (69.0–82.1)	1,564.6 (1,402.1–1,749.7)	1.7 (1.1–2.4)	401.4 (326.3–478.6)	0.0 (0.0–0.0)	817.1 (732.8–912.1)
Southern Latin America	Prevalence Rate: Cumulative change (%)	-0.9 (-2.0 to 0.3)	3.5 (-6.6 to 10.6)	2.3 (-1.0 to 5.3)	-17.0 (-26.1 to -8.6)	-7.9 (-13.1 to -2.4)	-39.1 (-43.8 to -34.7)	6.7 (-7.3 to 23.1)	-85.6 (-88.6 to -82.3)	-3.9 (-21.9 to 15.2)	-100.0 (-100.0 to -100.0)	-6.6 (-16.6 to 3.6)
Southern Latin America	YLDs Number: 1990	1,023,570 (774,266–1,302,550)	182,019 (129,033–249,070)	128,308 (90,459–174,750)	89,029 (60,136–119,423)	12,367 (8,900–16,621)	3,887 (2,809–5,190)	18,820 (13,429–25,356)	274 (158–427)	23,287 (16,474–31,188)	1 (0–2)	20,596 (16,015–25,760)
Southern Latin America	YLDs Number: 2015	1,077,954 (816,464–1,373,424)	200,602 (142,070–268,698)	140,337 (98,679–192,101)	79,492 (52,582–109,569)	12,957 (9,359–17,310)	2,432 (1,738–3,220)	32,320 (20,979–45,656)	39 (22–63)	16,539 (11,302–22,538)	0 (0–0)	17,326 (13,258–22,112)
Southern Latin America	YLDs Number: Cumulative change (%)	5.3 (2.1–8.2)	10.5 (-3.7 to 21.5)	9.3 (7.3–11.1)	-10.8 (-20.7 to -1.7)	5.0 (-5.6 to 13.1)	-37.3 (-44.4 to -29.6)	72.0 (26.3–104.8)	-85.2 (-89.1 to -80.2)	-28.6 (-43.1 to -13.1)	-100.0 (-100.0 to -100.0)	-15.9 (-21.7 to -10.1)
Southern Latin America	YLDs Rate: 1990	5,410.8 (4,093.0–6,881.1)	950.0 (673.7–1,300.5)	677.6 (477.5–923.9)	469.3 (317.1–629.9)	64.5 (46.4–86.7)	20.3 (14.6–27.1)	99.2 (70.8–133.6)	1.4 (0.8–2.2)	122.9 (86.9–164.9)	0.0 (0.0–0.0)	108.5 (84.4–135.7)
Southern Latin America	YLDs Rate: 2015	5,267.2 (3,987.2–6,711.7)	1,002.2 (710.2–1,342.6)	687.4 (483.7–941.8)	390.2 (257.5–540.1)	64.2 (46.3–85.6)	12.3 (8.8–16.3)	159.3 (103.3–224.8)	0.2 (0.1–0.3)	81.4 (55.6–111.1)	0.0 (0.0–0.0)	85.3 (65.2–108.9)
Southern Latin America	YLDs Rate: Cumulative change (%)	-2.6 (-5.8 to 0.0)	5.8 (-8.0 to 16.5)	1.4 (-0.3 to 3.0)	-17.0 (-26.1 to -8.3)	-0.3 (-10.6 to 7.5)	-39.1 (-45.9 to -31.6)	60.9 (18.1–91.6)	-85.4 (-89.3 to -80.4)	-33.5 (-46.9 to -18.8)	-100.0 (-100.0 to -100.0)	-21.4 (-26.7 to -16.0)
Argentina	Prevalence Number: 1990	11,158,545 (11,028,103–11,285,231)	3,569,459 (3,340,283–3,792,352)	3,516,016 (3,404,580–3,628,459)	1,386,654 (1,239,841–1,568,656)	914,626 (868,197–969,512)	18,431 (17,053–20,159)	185,744 (161,958–215,902)	2,060 (1,383–2,939)	34,094 (26,824–41,764)	63 (43–85)	118,191 (103,944–135,934)
Argentina	Prevalence Number: 2015	12,395,807 (12,252,714–12,537,250)	4,033,323 (3,732,598–4,237,550)	4,078,379 (3,951,518–4,200,706)	1,218,092 (998,926–1,446,381)	941,343 (890,353–993,179)	12,376 (11,311–13,606)	224,271 (195,657–258,991)	281 (187–415)	41,581 (31,814–51,815)	0 (0–0)	116,979 (102,321–134,808)
Argentina	Prevalence Number: Cumulative change (%)	11.1 (9.2–12.7)	13.2 (-1.0 to 23.3)	16.0 (11.5–20.8)	-12.2 (-22.5 to 0.0)	3.0 (-4.8 to 10.9)	-32.8 (-38.4 to -27.5)	21.6 (-0.2 to 47.7)	-86.2 (-89.3 to -82.6)	23.5 (-7.9 to 59.5)	-100.0 (-100.0 to -100.0)	-0.6 (-14.3 to 13.7)
Argentina	Prevalence Rate: 1990	87,095.6 (86,088.5–88,079.3)	27,594.2 (25,802.8–29,275.1)	27,641.9 (26,769.3–28,525.0)	10,782.1 (9,631.5–12,190.6)	7,123.3 (6,762.5–7,553.4)	142.6 (131.9–155.9)	1,448.4 (1,263.5–1,683.9)	15.8 (10.6–22.6)	266.9 (209.9–327.0)	0.5 (0.3–0.7)	917.4 (806.1–1,055.9)
Argentina	Prevalence Rate: 2015	86,174.5 (85,174.0–87,161.1)	28,207.0 (26,079.1–29,640.0)	28,253.5 (27,373.0–29,096.2)	8,453.2 (6,933.3–10,049.0)	6,551.4 (6,198.1–6,912.5)	87.3 (79.7–96.0)	1,560.6 (1,361.4–1,802.4)	2.0 (1.3–2.9)	289.1 (221.7–360.2)	0.0 (0.0–0.0)	820.6 (718.4–945.2)
Argentina	Prevalence Rate: Cumulative change (%)	-1.1 (-2.7 to 0.4)	2.4 (-10.4 to 11.5)	2.2 (-1.7 to 6.5)	-21.7 (-30.7 to -10.7)	-7.9 (-14.9 to -0.9)	-38.7 (-43.9 to -34.0)	8.5 (-11.0 to 31.8)	-87.3 (-90.1 to -84.0)	9.7 (-18.0 to 41.9)	-100.0 (-100.0 to -100.0)	-10.2 (-22.5 to 2.8)
Argentina	YLDs Number: 1990	706,158 (534,967–899,016)	135,306 (95,715–186,005)	85,512 (59,954–116,421)	63,133 (42,230–84,943)	8,372 (5,947–11,356)	3,023 (2,179–4,073)	12,767 (9,067–17,233)	250 (143–391)	9,955 (6,671–13,862)	1 (0–2)	15,016 (11,660–18,875)
Argentina	YLDs Number: 2015	771,900 (584,180–981,843)	155,037 (108,958–207,355)	97,861 (68,676–133,839)	55,506 (36,680–77,463)	9,016 (6,478–12,072)	2,029 (1,447–2,711)	24,004 (15,487–34,303)	34 (19–55)	8,429 (5,561–11,805)	0 (0–0)	12,504 (9,514–16,034)
Argentina	YLDs Number: Cumulative change (%)	9.4 (4.8–13.3)	15.1 (-4.1 to 29.3)	14.4 (11.8–16.8)	-12.1 (-22.3 to 0.3)	8.0 (-8.1 to 19.9)	-32.7 (-41.2 to -22.6)	88.5 (33.2–129.6)	-85.9 (-90.2 to -80.7)	-14.0 (-38.8 to 15.7)	-100.0 (-100.0 to -100.0)	-16.7 (-23.7 to -9.6)
Argentina	YLDs Rate: 1990	5,527.9 (4,190.0–7,036.3)	1,038.5 (734.7–1,426.1)	668.9 (468.4–912.4)	490.9 (328.6–660.2)	64.0 (45.5–86.9)	23.4 (16.9–31.5)	99.6 (70.8–134.5)	1.9 (1.1–3.0)	77.9 (52.1–108.3)	0.0 (0.0–0.0)	117.0 (90.8–146.9)
Argentina	YLDs Rate: 2015	5,355.7 (4,053.6–6,815.6)	1,084.1 (761.3–1,450.6)	679.3 (476.6–928.7)	385.2 (254.6–538.6)	62.8 (45.1–84.0)	14.3 (10.2–19.1)	167.0 (107.8–238.8)	0.2 (0.1–0.4)	58.6 (38.7–82.0)	0.0 (0.0–0.0)	87.0 (66.2–111.5)
Argentina	YLDs Rate: Cumulative change (%)	-3.1 (-7.1 to 0.5)	4.9 (-12.8 to 18.0)	1.5 (-0.6 to 3.6)	-21.5 (-30.6 to -10.4)	-1.6 (-16.5 to 9.4)	-38.7 (-46.4 to -29.4)	68.3 (18.9–104.8)	-87.0 (-91.0 to -82.2)	-23.6 (-45.7 to 2.6)	-100.0 (-100.0 to -100.0)	-25.7 (-31.8 to -19.3)
Chile	Prevalence Number: 1990	4,371,982 (4,324,990–4,416,203)	1,014,540 (955,137–1,081,497)	1,422,776 (1,378,297–1,466,928)	450,691 (360,431–531,397)	337,737 (318,309–358,098)	4,342 (4,037–4,692)	78,014 (67,250–90,423)	66 (43–95)	40,245 (31,751–49,170)	0 (0–0)	39,998 (35,745–45,912)
Chile	Prevalence Number: 2015	4,251,417 (4,198,174–4,299,453)	988,941 (928,441–1,054,305)	1,462,430 (1,416,225–1,509,457)	438,939 (359,178–529,248)	299,622 (283,078–316,919)	2,056 (1,940–2,179)	78,359 (68,811–89,286)	25 (16–38)	35,459 (27,552–44,016)	0 (0–0)	38,588 (34,298–43,743)
Chile	Prevalence Number: Cumulative change (%)	-2.8 (-4.3 to -1.2)	-2.4 (-7.4 to 5.3)	2.8 (-1.4 to 7.2)	-1.8 (-22.5 to 14.3)	-11.2 (-18.1 to -3.8)	-52.6 (-56.0 to -49.6)	1.1 (-16.3 to 19.7)	-60.6 (-70.4 to -49.5)	-10.8 (-33.2 to 15.4)	-13.9 (-28.6 to 4.1)	-3.1 (-18.4 to 12.4)
Chile	Prevalence Rate: 1990	86,162.1 (85,245.3–87,046.0)	19,955.2 (18,814.2–21,238.3)	28,035.6 (27,160.3–28,905.3)	8,964.5 (7,159.6–10,576.4)	6,649.2 (6,268.3–7,049.8)	83.5 (77.7–90.2)	1,534.6 (1,323.5–1,779.4)	1.3 (0.8–1.8)	790.7 (623.2–966.4)	0.0 (0.0–0.0)	778.3 (695.2–894.8)
Chile	Prevalence Rate: 2015	85,634.3 (84,493.7–86,677.3)	20,782.0 (19,422.5–22,232.3)	28,737.6 (27,825.2–29,673.5)	8,836.9 (7,220.0–10,648.7)	6,088.8 (5,752.4–6,438.8)	44.5 (41.9–47.2)	1,587.7 (1,394.4–1,810.3)	0.6 (0.4–0.8)	718.7 (560.7–892.3)	0.0 (0.0–0.0)	808.4 (720.4–913.3)
Chile	Prevalence Rate: Cumulative change (%)	-0.6 (-2.2 to 1.0)	4.2 (-1.5 to 12.8)	2.5 (-1.7 to 6.9)	-0.6 (-21.6 to 15.8)	-8.3 (-15.5 to -0.7)	-46.7 (-50.4 to -43.4)	4.2 (-13.7 to 23.4)	-55.3 (-66.5 to -43.3)	-8.0 (-31.2 to 18.6)	-7.8 (-22.5 to 10.7)	4.4 (-12.0 to 20.7)
Chile	YLDs Number: 1990	259,857 (197,262–332,056)	35,835 (25,165–48,970)	35,541 (25,245–48,477)	20,538 (13,315–28,784)	3,334 (2,395–4,489)	718 (503–961)	5,003 (3,555–6,788)	8 (4–13)	11,823 (7,910–16,447)	0 (0–0)	4,436 (3,406–5,566)
Chile	YLDs Number: 2015	254,140 (192,265–322,482)	35,591 (24,991–47,926)	35,595 (25,005–48,642)	20,027 (13,100–27,669)	3,377 (2,413–4,505)	338 (235–456)	6,907 (4,643–9,554)	3 (1–5)	7,181 (4,712–10,014)	0 (0–0)	3,994 (2,973–5,150)
Chile	YLDs Number: Cumulative change (%)	-2.2 (-4.8 to 0.7)	-0.6 (-6.4 to 10.9)	0.1 (-2.2 to 2.3)	-1.8 (-22.5 to 14.2)	1.5 (-7.6 to 12.7)	-52.6 (-60.1 to -44.7)	38.1 (9.8–63.0)	-58.6 (-72.6 to -38.7)	-38.4 (-55.4 to -18.4)	-10.7 (-27.1 to 9.5)	-10.0 (-19.2 to -0.9)
Chile	YLDs Rate: 1990	5,133.5 (3,897.0–6,561.0)	710.1 (498.9–970.4)	700.5 (497.6–955.0)	408.5 (264.5–573.7)	66.7 (48.0–89.8)	13.8 (9.7–18.5)	98.4 (70.0–133.5)	0.2 (0.1–0.3)	232.3 (155.6–323.0)	0.0 (0.0–0.0)	87.4 (67.1–109.6)
Chile	YLDs Rate: 2015	5,024.7 (3,808.4–6,370.3)	748.2 (524.8–1,011.7)	711.0 (500.0–970.5)	403.3 (262.7–557.3)	69.3 (49.6–92.3)	7.3 (5.1–9.9)	140.0 (94.2–193.7)	0.1 (0.0–0.1)	145.6 (95.6–203.1)	0.0 (0.0–0.0)	80.9 (60.3–104.4)
Chile	YLDs Rate: Cumulative change (%)	-2.1 (-4.8 to 0.8)	5.5 (-1.4 to 18.2)	1.5 (-0.7 to 3.5)	-0.6 (-21.5 to 15.9)	4.1 (-5.4 to 15.5)	-46.7 (-55.0 to -37.9)	42.3 (13.2–68.1)	-53.0 (-69.0 to -30.5)	-36.4 (-53.8 to -15.7)	-5.3 (-22.0 to 16.2)	-7.4 (-16.9 to 1.9)
Uruguay	Prevalence Number: 1990	898,805 (886,044–910,638)	293,577 (273,477–327,180)	299,971 (291,105–309,196)	117,580 (97,276–138,085)	76,555 (72,309–81,424)	881 (756–1,031)	15,535 (13,613–18,156)	126 (81–187)	5,149 (4,059–6,260)	0 (0–0)	9,143 (8,076–10,529)
Uruguay	Prevalence Number: 2015	833,804 (821,314–844,893)	267,189 (250,879–286,196)	287,634 (279,260–296,005)	86,797 (71,225–104,028)	64,891 (61,093–68,566)	382 (352–416)	14,990 (13,189–17,236)	17 (10–28)	4,562 (3,548–5,708)	0 (0–0)	7,794 (7,026–8,869)
Uruguay	Prevalence Number: Cumulative change (%)	-7.2 (-9.1 to -5.5)	-8.8 (-18.0 to -0.5)	-4.1 (-7.8 to -0.3)	-26.0 (-36.1 to -17.8)	-15.1 (-21.7 to -8.2)	-56.4 (-61.4 to -51.1)	-2.8 (-19.5 to 15.9)	-85.7 (-90.8 to -79.1)	-10.2 (-33.1 to 16.3)	-14.8 (-28.3 to 2.5)	-14.3 (-27.1 to -0.8)
Uruguay	Prevalence Rate: 1990	83,823.3 (82,601.0–84,957.7)	27,594.7 (25,707.1–30,820.1)	27,702.1 (26,874.4–28,546.9)	10,979.6 (9,072.4–12,911.5)	7,147.7 (6,750.6–7,599.6)	84.6 (72.5–99.1)	1,452.1 (1,271.2–1,697.6)	12.1 (7.8–17.9)	482.4 (380.4–586.6)	0.0 (0.0–0.0)	860.3 (760.0–989.7)
Uruguay	Prevalence Rate: 2015	83,396.5 (82,098.7–84,523.4)	27,361.6 (25,626.3–29,404.4)	28,269.1 (27,445.2–29,078.2)	8,676.3 (7,107.3–10,424.9)	6,526.8 (6,141.4–6,891.0)	40.5 (37.3–44.2)	1,506.2 (1,324.1–1,731.7)	1.9 (1.2–3.1)	458.7 (357.3–571.0)	0.0 (0.0–0.0)	805.0 (727.2–912.5)
Uruguay	Prevalence Rate: Cumulative change (%)	-0.5 (-2.6 to 1.4)	-0.6 (-10.8 to 8.8)	2.1 (-1.8 to 6.2)	-20.8 (-31.6 to -11.9)	-8.6 (-15.6 to -1.2)	-51.9 (-57.4 to -45.9)	4.4 (-13.4 to 24.6)	-83.7 (-89.5 to -76.2)	-3.6 (-28.2 to 24.7)	-7.8 (-22.5 to 10.7)	-5.9 (-19.9 to 8.7)
Uruguay	YLDs Number: 1990	57,522 (43,732–73,015)	10,872 (7,610–15,073)	7,250 (5,113–9,935)	5,354 (3,580–7,331)	660 (469–895)	145 (101–198)	1,049 (742–1,426)	15 (8–24)	1,507 (1,028–2,047)	0 (0–0)	1,142 (885–1,445)
Uruguay	YLDs Number: 2015	51,877 (39,328–65,805)	9,967 (7,055–13,655)	6,875 (4,808–9,386)	3,955 (2,594–5,527)	562 (400–766)	63 (43–86)	1,407 (925–1,997)	2 (1–3)	928 (606–1,289)	0 (0–0)	827 (642–1,065)
Uruguay	YLDs Number: Cumulative change (%)	-9.8 (-12.8 to -7.1)	-8.0 (-20.7 to 3.5)	-5.2 (-7.2 to -3.2)	-26.0 (-36.0 to -17.5)	-14.6 (-23.7 to -4.2)	-56.3 (-64.2 to -47.5)	34.1 (8.3–59.5)	-85.5 (-91.7 to -77.4)	-37.6 (-54.3 to -15.6)	-13.1 (-28.8 to 5.2)	-27.5 (-33.4 to -20.8)
Uruguay	YLDs Rate: 1990	5,326.1 (4,049.1–6,761.6)	1,021.5 (714.6–1,416.7)	674.9 (476.8–922.8)	500.1 (334.7–685.6)	61.5 (43.8–83.4)	14.0 (9.8–19.1)	98.1 (69.3–133.3)	1.5 (0.8–2.4)	141.3 (96.2–192.0)	0.0 (0.0–0.0)	106.8 (82.7–135.2)
Uruguay	YLDs Rate: 2015	5,124.7 (3,894.2–6,514.3)	1,023.8 (725.1–1,410.3)	684.5 (479.9–932.3)	395.5 (259.7–550.3)	57.3 (40.8–78.4)	6.7 (4.7–9.1)	141.5 (93.1–200.5)	0.2 (0.1–0.4)	93.3 (61.2–129.8)	0.0 (0.0–0.0)	83.1 (64.5–107.0)

© 2017 Global Burden of Disease Child and Adolescent Health Collaboration. JAMA Pediatrics.

eTable 3. Prevalent Cases, Rates (per 100,000 population), Years Lived with Disability (YLDs), and Cumulative Percent Change with 95% Uncertainty Interval (UI) for the Top 10 Global Causes of YLDs in Children and Adolescents in 195 Countries and Territories, Aged 0 to 19 Years, Both Sexes, 1990 and 2015. *Note: best viewed by enlarging in browser.*

Location	Measure	All causes	Iron-deficiency anemia	Skin and subcutaneous diseases	Asthma	Hemoglobinopathies and hemolytic anemias	Diarrheal diseases	Congenital anomalies	Protein-energy malnutrition	Epilepsy	Malaria	Neonatal preterm birth complications
Uruguay	YLDs Rate: Cumulative change (%)	-3.8 (-7.1 to -0.7)	0.6 (-13.6 to 13.6)	1.4 (-0.7 to 3.5)	-20.8 (-31.5 to -11.6)	-6.5 (-16.7 to 5.1)	-51.7 (-60.4 to -42.1)	44.2 (16.7–71.3)	-83.5 (-90.6 to -74.2)	-33.0 (-51.0 to -9.3)	-6.0 (-23.2 to 14.0)	-22.1 (-28.5 to -15.1)
Central Europe, Eastern Europe, and Central Asia	Prevalence Number: 1990	120,672,291 (120,145,528–121,253,284)	36,800,846 (35,699,735–38,088,425)	44,920,695 (44,309,927–45,522,264)	8,016,550 (6,874,889–9,180,267)	11,842,995 (11,535,489–12,208,193)	675,015 (622,681–730,407)	2,295,994 (2,132,533–2,483,891)	762,085 (614,514–946,265)	469,085 (411,534–528,159)	2,317 (1,381–3,433)	1,466,425 (1,331,952–1,614,063)
Central Europe, Eastern Europe, and Central Asia	Prevalence Number: 2015	83,229,857 (82,814,336–83,665,343)	24,995,340 (24,123,087–26,011,767)	30,972,492 (30,532,270–31,390,708)	4,034,159 (3,362,107–4,775,527)	7,564,276 (7,373,236–7,783,151)	325,755 (303,068–348,805)	1,567,853 (1,474,076–1,673,521)	430,963 (336,953–554,326)	301,482 (262,992–344,684)	9 (2–33)	1,045,743 (948,805–1,145,086)
Central Europe, Eastern Europe, and Central Asia	Prevalence Number: Cumulative change (%)	-31.0 (-31.4 to -30.6)	-32.0 (-35.3 to -27.8)	-31.0 (-32.1 to -30.0)	-49.7 (-53.9 to -45.5)	-36.1 (-38.2 to -34.0)	-51.7 (-54.4 to -49.1)	-31.6 (-37.2 to -26.4)	-42.5 (-59.5 to -20.2)	-35.6 (-42.9 to -28.1)	-99.6 (-99.9 to -98.4)	-28.6 (-32.7 to -24.3)
Central Europe, Eastern Europe, and Central Asia	Prevalence Rate: 1990	89,168.3 (88,775.9–89,603.7)	27,246.9 (26,417.8–28,218.8)	33,229.8 (32,778.5–33,672.4)	5,886.4 (5,054.1–6,742.2)	8,763.0 (8,535.7–9,033.2)	508.9 (469.6–550.4)	1,699.4 (1,578.5–1,838.6)	578.5 (466.5–718.3)	346.5 (304.1–390.0)	1.7 (1.0–2.6)	1,110.1 (1,008.1–1,220.7)
Central Europe, Eastern Europe, and Central Asia	Prevalence Rate: 2015	88,706.6 (88,274.1–89,147.7)	26,462.7 (25,617.9–27,438.5)	33,446.8 (32,974.4–33,893.7)	4,418.3 (3,674.6–5,237.9)	8,041.7 (7,838.8–8,274.8)	323.6 (302.3–345.1)	1,662.6 (1,562.9–1,773.7)	411.0 (321.4–528.7)	324.5 (283.0–370.9)	0.0 (0.0–0.0)	1,073.1 (971.9–1,177.2)
Central Europe, Eastern Europe, and Central Asia	Prevalence Rate: Cumulative change (%)	-0.5 (-1.1 to 0.0)	-2.8 (-7.4 to 3.0)	0.7 (-0.9 to 2.2)	-25.0 (-31.4 to -18.5)	-8.2 (-11.2 to -5.2)	-36.4 (-39.8 to -32.9)	-2.0 (-10.0 to 5.4)	-27.7 (-49.1 to 0.2)	-6.2 (-16.7 to 4.7)	-99.4 (-99.9 to -97.8)	-3.3 (-8.7 to 2.6)
Central Europe, Eastern Europe, and Central Asia	YLDs Number: 1990	7,733,601 (5,884,403–9,813,640)	1,375,767 (975,708–1,862,353)	933,835 (640,402–1,315,749)	363,859 (247,686–497,905)	144,479 (103,920–194,219)	110,735 (80,430–147,255)	157,053 (107,605–216,058)	95,353 (62,273–134,898)	169,259 (120,308–219,458)	48 (25–81)	178,053 (139,721–224,000)
Central Europe, Eastern Europe, and Central Asia	YLDs Number: 2015	4,908,855 (3,731,932–6,242,762)	925,102 (661,518–1,245,521)	646,982 (445,141–907,804)	183,445 (121,599–252,267)	92,102 (66,904–122,794)	53,541 (38,133–70,917)	131,410 (84,783–184,325)	54,052 (34,200–78,245)	73,908 (52,208–96,691)	0 (0–1)	101,699 (78,997–129,472)
Central Europe, Eastern Europe, and Central Asia	YLDs Number: Cumulative change (%)	-36.5 (-37.8 to -35.2)	-32.7 (-36.2 to -27.3)	-30.7 (-31.3 to -30.1)	-49.6 (-53.8 to -45.3)	-36.2 (-39.3 to -31.9)	-51.6 (-54.4 to -48.6)	-16.6 (-25.9 to -7.0)	-42.4 (-59.5 to -19.8)	-56.3 (-61.8 to -50.3)	-99.2 (-99.8 to -97.1)	-42.9 (-45.2 to -40.4)
Central Europe, Eastern Europe, and Central Asia	YLDs Rate: 1990	5,718.3 (4,351.4–7,255.7)	1,015.0 (719.1–1,375.0)	690.1 (473.0–972.3)	267.2 (182.0–365.5)	106.4 (76.5–143.1)	83.5 (60.7–111.0)	116.3 (79.6–160.0)	72.4 (47.3–102.4)	125.0 (88.9–162.1)	0.0 (0.0–0.1)	131.8 (103.4–165.9)
Central Europe, Eastern Europe, and Central Asia	YLDs Rate: 2015	5,297.7 (4,029.1–6,734.2)	991.4 (709.2–1,336.2)	693.4 (476.4–975.3)	200.9 (132.7–276.6)	99.9 (72.4–133.3)	53.2 (37.9–70.3)	139.3 (89.8–195.6)	51.5 (32.6–74.6)	79.5 (56.2–104.3)	0.0 (0.0–0.0)	107.8 (83.5–137.0)
Central Europe, Eastern Europe, and Central Asia	YLDs Rate: Cumulative change (%)	-7.4 (-9.1 to -5.5)	-2.3 (-7.2 to 5.1)	0.5 (-0.3 to 1.3)	-24.9 (-31.2 to -18.5)	-6.1 (-10.4 to -0.1)	-36.3 (-39.9 to -32.3)	19.5 (6.1–33.3)	-27.6 (-49.1 to 0.8)	-36.3 (-44.3 to -27.6)	-98.9 (-99.8 to -96.0)	-18.3 (-21.6 to -14.7)
Eastern Europe	Prevalence Number: 1990	57,567,274 (57,103,773–58,049,460)	17,572,994 (16,577,637–18,798,901)	21,972,692 (21,459,622–22,452,158)	4,473,427 (3,807,150–5,099,556)	5,182,267 (5,006,920–5,371,944)	231,092 (214,981–249,103)	1,168,182 (1,032,481–1,341,237)	283,434 (173,988–426,604)	169,426 (136,581–204,405)	0 (0–0)	599,067 (527,833–678,479)
Eastern Europe	Prevalence Number: 2015	35,014,905 (34,662,056–35,348,239)	10,661,588 (9,940,922–11,612,735)	13,133,232 (12,783,614–13,451,401)	1,682,897 (1,395,099–1,999,469)	2,945,210 (2,839,520–3,063,942)	109,724 (101,816–117,558)	710,175 (640,102–798,652)	143,535 (73,586–253,419)	74,876 (59,025–91,952)	0 (0–0)	364,503 (323,165–412,833)
Eastern Europe	Prevalence Number: Cumulative change (%)	-39.2 (-39.9 to -38.5)	-39.2 (-44.9 to -31.4)	-40.2 (-42.0 to -38.3)	-62.4 (-66.5 to -58.3)	-43.1 (-45.9 to -40.3)	-52.5 (-55.3 to -50.0)	-38.9 (-47.9 to -29.9)	-45.1 (-77.4 to 8.1)	-55.3 (-66.4 to -43.4)	-34.0 (-36.3 to -31.8)	-39.0 (-45.7 to -31.7)
Eastern Europe	Prevalence Rate: 1990	88,180.0 (87,452.9–88,931.6)	26,990.5 (25,373.4–28,985.8)	33,650.7 (32,868.9–34,385.3)	6,773.9 (5,765.1–7,719.1)	7,963.9 (7,696.4–8,257.2)	368.9 (343.4–397.7)	1,796.6 (1,587.8–2,063.3)	461.2 (283.1–694.2)	258.9 (208.9–312.1)	0.0 (0.0–0.0)	954.7 (842.7–1,076.3)
Eastern Europe	Prevalence Rate: 2015	87,739.5 (86,943.0–88,480.9)	26,359.4 (24,790.9–28,384.6)	33,901.2 (33,007.1–34,727.9)	4,390.4 (3,629.3–5,217.7)	7,331.0 (7,069.0–7,620.0)	244.5 (227.2–261.5)	1,761.5 (1,590.9–1,980.7)	303.8 (155.7–536.4)	188.5 (148.3–230.7)	0.0 (0.0–0.0)	860.4 (760.5–979.2)
Eastern Europe	Prevalence Rate: Cumulative change (%)	-0.5 (-1.6 to 0.6)	-2.1 (-11.0 to 9.8)	0.8 (-2.2 to 3.9)	-35.2 (-42.1 to -28.1)	-7.9 (-12.3 to -3.2)	-33.7 (-37.5 to -30.2)	-1.5 (-15.9 to 13.1)	-28.5 (-70.5 to 40.6)	-26.3 (-44.6 to -6.8)	-0.0 (-1.0 to 1.2)	-9.7 (-19.3 to 1.3)
Eastern Europe	YLDs Number: 1990	3,757,030 (2,861,325–4,754,691)	665,193 (468,597–899,985)	455,490 (311,480–641,565)	202,872 (137,046–278,206)	66,070 (47,061–89,482)	37,980 (27,381–50,402)	77,832 (53,653–107,446)	35,525 (18,773–58,638)	60,831 (42,186–83,053)	0 (0–0)	77,405 (60,127–98,511)
Eastern Europe	YLDs Number: 2015	2,064,354 (1,559,425–2,634,072)	396,743 (280,843–533,421)	276,676 (190,057–387,845)	76,460 (50,224–105,415)	35,684 (25,722–47,295)	18,078 (12,944–24,109)	58,397 (37,526–83,365)	18,042 (8,104–32,838)	17,728 (11,994–24,443)	0 (0–0)	34,663 (26,600–44,953)
Eastern Europe	YLDs Number: Cumulative change (%)	-45.1 (-46.8 to -42.7)	-40.2 (-46.2 to -29.0)	-39.2 (-40.3 to -38.1)	-62.3 (-66.5 to -58.3)	-45.9 (-50.9 to -36.7)	-52.4 (-56.2 to -48.7)	-25.2 (-35.3 to -15.1)	-44.9 (-77.7 to 8.5)	-70.5 (-78.2 to -61.4)	-34.0 (-47.2 to -18.8)	-55.2 (-58.7 to -51.6)
Eastern Europe	YLDs Rate: 1990	5,744.8 (4,372.5–7,269.1)	1,014.8 (714.1–1,375.3)	697.4 (477.2–982.2)	307.2 (207.5–420.9)	100.9 (71.8–136.5)	60.6 (43.7–80.5)	119.7 (82.6–165.3)	57.8 (30.6–95.4)	93.0 (64.3–127.1)	0.0 (0.0–0.0)	119.0 (92.5–151.4)
Eastern Europe	YLDs Rate: 2015	5,312.8 (4,008.3–6,797.6)	992.1 (704.5–1,334.2)	702.6 (482.6–989.5)	199.4 (131.3–275.6)	90.4 (65.3–120.3)	40.3 (28.8–53.6)	144.8 (92.9–206.8)	38.2 (17.2–69.5)	44.6 (30.1–61.7)	0.0 (0.0–0.0)	86.1 (66.3–111.3)
Eastern Europe	YLDs Rate: Cumulative change (%)	-7.5 (-10.2 to -4.1)	-2.0 (-11.5 to 14.6)	0.7 (-0.7 to 2.3)	-35.1 (-42.3 to -28.2)	-10.2 (-18.6 to 3.7)	-33.6 (-38.9 to -28.5)	20.6 (4.2–36.7)	-28.4 (-71.0 to 41.1)	-51.4 (-64.1 to -36.5)	3.0 (-17.8 to 26.1)	-27.6 (-33.3 to -22.0)
Belarus	Prevalence Number: 1990	2,669,279 (2,637,280–2,700,682)	729,850 (685,444–779,687)	1,073,638 (1,040,155–1,105,081)	186,839 (155,648–220,175)	242,117 (230,203–254,245)	10,357 (9,533–11,265)	55,565 (47,471–65,906)	11,031 (4,484–21,111)	8,803 (6,405–11,300)	0 (0–0)	27,832 (24,149–32,148)
Belarus	Prevalence Number: 2015	1,606,264 (1,584,184–1,627,046)	452,910 (420,250–493,652)	655,482 (636,521–674,844)	96,934 (80,226–111,608)	135,274 (128,495–142,179)	3,698 (3,454–3,934)	33,656 (28,647–39,955)	4,659 (1,864–9,239)	4,048 (3,021–5,190)	0 (0–0)	15,048 (13,133–17,480)
Belarus	Prevalence Number: Cumulative change (%)	-39.8 (-40.8 to -38.8)	-37.8 (-43.5 to -29.7)	-38.9 (-41.3 to -36.4)	-47.9 (-54.6 to -38.8)	-44.1 (-48.0 to -40.2)	-64.2 (-66.7 to -61.5)	-38.9 (-51.0 to -24.8)	-46.8 (-86.2 to 22.0)	-52.9 (-68.0 to -34.0)	-38.1 (-39.9 to -35.9)	-45.7 (-53.3 to -37.9)
Belarus	Prevalence Rate: 1990	87,934.5 (86,853.1–88,999.1)	24,152.3 (22,615.2–25,829.6)	35,354.3 (34,255.3–36,388.5)	6,088.4 (5,074.1–7,173.0)	8,002.7 (7,608.8–8,403.4)	355.8 (327.2–386.9)	1,837.8 (1,569.8–2,179.2)	387.3 (157.4–741.1)	288.9 (210.5–371.1)	0.0 (0.0–0.0)	952.3 (829.4–1,094.5)
Belarus	Prevalence Rate: 2015	87,768.4 (86,618.3–88,848.8)	24,772.8 (23,180.7–26,835.8)	35,790.4 (34,755.5–36,850.5)	5,493.4 (4,534.7–6,346.9)	7,369.4 (6,999.9–7,741.0)	187.2 (175.0–199.1)	1,828.1 (1,553.6–2,170.1)	223.4 (89.4–442.9)	222.3 (165.8–285.6)	0.0 (0.0–0.0)	785.4 (682.3–914.7)
Belarus	Prevalence Rate: Cumulative change (%)	-0.2 (-1.8 to 1.5)	2.8 (-6.4 to 14.9)	1.3 (-2.6 to 5.3)	-9.4 (-21.3 to 6.6)	-7.8 (-14.1 to -1.5)	-47.3 (-50.9 to -43.2)	0.3 (-19.4 to 23.6)	-27.3 (-81.2 to 66.5)	-21.3 (-46.7 to 10.7)	-0.0 (-0.0 to 0.0)	-17.2 (-28.7 to -5.1)
Belarus	YLDs Number: 1990	168,732 (128,407–213,962)	26,366 (18,558–36,213)	26,512 (18,442–36,514)	8,487 (5,633–11,543)	2,910 (2,086–3,958)	1,705 (1,226–2,294)	3,975 (2,545–5,761)	1,382 (514–2,801)	3,154 (2,072–4,460)	0 (0–0)	3,572 (2,736–4,560)
Belarus	YLDs Number: 2015	94,472 (71,403–120,544)	16,016 (11,144–21,753)	16,122 (11,274–22,242)	4,406 (2,978–6,111)	1,572 (1,108–2,127)	608 (432–805)	2,743 (1,635–4,276)	586 (207–1,181)	917 (585–1,339)	0 (0–0)	1,406 (1,070–1,818)
Belarus	YLDs Number: Cumulative change (%)	-44.0 (-45.9 to -42.0)	-39.1 (-45.8 to -30.0)	-39.2 (-40.4 to -38.0)	-47.8 (-54.7 to -38.3)	-45.9 (-50.8 to -39.0)	-64.2 (-68.0 to -60.5)	-30.1 (-51.4 to -1.1)	-46.6 (-86.1 to 23.5)	-70.1 (-80.5 to -56.2)	-37.9 (-49.2 to -23.8)	-60.6 (-64.5 to -56.3)
Belarus	YLDs Rate: 1990	5,547.6 (4,222.5–7,039.1)	867.2 (608.6–1,194.7)	872.5 (606.9–1,202.0)	276.6 (183.6–375.8)	95.4 (68.1–129.6)	58.6 (42.0–78.7)	131.5 (84.2–190.7)	48.5 (18.1–98.4)	103.6 (68.0–146.4)	0.0 (0.0–0.0)	118.0 (90.4–150.6)
Belarus	YLDs Rate: 2015	5,214.3 (3,946.2–6,647.9)	896.7 (626.8–1,215.5)	879.4 (613.3–1,211.1)	249.7 (168.8–346.6)	89.2 (63.0–120.6)	30.8 (21.9–40.8)	148.9 (89.0–231.6)	28.1 (10.0–56.6)	50.4 (32.1–73.6)	0.0 (0.0–0.0)	76.7 (58.4–99.1)
Belarus	YLDs Rate: Cumulative change (%)	-6.0 (-9.1 to -2.8)	3.7 (-7.6 to 18.5)	0.8 (-1.3 to 2.6)	-9.2 (-21.1 to 7.4)	-6.3 (-14.6 to 5.0)	-47.3 (-53.0 to -41.8)	14.7 (-20.3 to 62.6)	-27.1 (-81.1 to 68.5)	-50.1 (-67.4 to -26.7)	5.1 (-13.4 to 28.7)	-35.0 (-41.4 to -27.8)
Estonia	Prevalence Number: 1990	392,285 (387,561–396,809)	113,436 (105,862–122,847)	151,615 (147,047–156,158)	29,014 (23,438–34,944)	35,357 (33,567–37,194)	1,215 (1,139–1,299)	8,439 (7,256–9,985)	2,326 (1,012–4,610)	1,270 (955–1,617)	0 (0–0)	4,194 (3,712–4,804)
Estonia	Prevalence Number: 2015	217,922 (214,674–220,785)	63,065 (58,294–68,148)	82,976 (80,427–85,585)	13,028 (10,645–15,694)	18,248 (17,333–19,173)	489 (463–517)	4,753 (4,111–5,577)	786 (294–1,592)	547 (403–709)	0 (0–0)	1,937 (1,685–2,278)
Estonia	Prevalence Number: Cumulative change (%)	-44.4 (-45.4 to -43.5)	-44.3 (-49.9 to -38.7)	-45.3 (-47.4 to -43.0)	-54.8 (-63.6 to -48.0)	-48.3 (-51.7 to -44.8)	-59.7 (-61.7 to -57.7)	-43.2 (-54.7 to -29.5)	-58.0 (-89.7 to 1.5)	-56.0 (-69.2 to -38.6)	-40.4 (-41.7 to -39.0)	-53.6 (-60.2 to -45.6)
Estonia	Prevalence Rate: 1990	86,478.5 (85,409.3–87,487.8)	25,154.7 (23,408.1–27,331.1)	33,317.8 (32,314.5–34,310.5)	6,336.9 (5,118.7–7,632.1)	7,818.1 (7,421.0–8,223.9)	277.3 (259.8–296.6)	1,866.7 (1,605.6–2,209.2)	539.6 (234.8–1,069.7)	280.2 (210.5–356.3)	0.0 (0.0–0.0)	959.7 (852.2–1,094.9)
Estonia	Prevalence Rate: 2015	86,021.2 (84,802.3–87,109.3)	24,413.1 (22,667.6–26,314.8)	33,863.2 (32,808.3–34,936.6)	5,197.5 (4,259.6–6,262.0)	7,184.9 (6,825.6–7,550.7)	181.6 (172.0–191.6)	1,869.6 (1,616.8–2,193.8)	287.3 (107.8–581.8)	218.3 (160.6–282.2)	0.0 (0.0–0.0)	752.3 (652.8–885.6)
Estonia	Prevalence Rate: Cumulative change (%)	-0.5 (-2.1 to 1.1)	-2.8 (-12.6 to 7.1)	1.7 (-2.3 to 5.8)	-17.5 (-33.3 to -5.2)	-8.0 (-14.0 to -1.7)	-34.5 (-37.8 to -31.2)	1.0 (-19.6 to 25.1)	-33.9 (-83.7 to 59.9)	-20.4 (-44.8 to 11.6)	0.0 (-0.0 to 0.0)	-21.3 (-32.7 to -7.9)
Estonia	YLDs Number: 1990	25,428 (19,526–32,151)	4,068 (2,823–5,590)	3,078 (2,085–4,374)	1,316 (868–1,856)	430 (305–579)	199 (142–265)	605 (399–851)	291 (113–583)	456 (305–643)	0 (0–0)	549 (423–693)
Estonia	YLDs Number: 2015	12,216 (9,301–15,570)	2,290 (1,592–3,171)	1,704 (1,155–2,414)	593 (390–823)	219 (154–300)	80 (57–106)	475 (269–829)	99 (33–212)	121 (76–180)	0 (0–0)	179 (136–229)
Estonia	YLDs Number: Cumulative change (%)	-52.0 (-53.8 to -49.9)	-43.6 (-50.2 to -34.7)	-44.6 (-45.8 to -43.3)	-54.7 (-63.7 to -47.8)	-49.1 (-54.4 to -43.1)	-59.6 (-63.3 to -55.4)	-22.1 (-42.6 to 15.6)	-58.0 (-89.7 to 3.3)	-72.7 (-82.1 to -60.6)	-37.4 (-46.8 to -19.5)	-67.4 (-70.7 to -63.7)
Estonia	YLDs Rate: 1990	5,590.4 (4,292.3–7,072.3)	898.8 (624.1–1,238.6)	677.2 (458.4–961.9)	287.6 (189.8–405.6)	94.9 (67.1–128.2)	45.6 (32.5–60.6)	133.9 (88.4–188.5)	67.7 (26.3–135.3)	100.6 (67.4–141.9)	0.0 (0.0–0.0)	121.4 (93.6–153.1)
Estonia	YLDs Rate: 2015	4,941.2 (3,754.9–6,284.3)	881.3 (613.2–1,216.2)	685.4 (463.1–975.4)	236.6 (156.2–328.5)	85.4 (60.2–117.3)	29.9 (21.4–39.6)	186.9 (105.6–326.8)	36.2 (12.2–77.8)	48.4 (30.5–72.0)	0.0 (0.0–0.0)	70.5 (53.4–90.5)
Estonia	YLDs Rate: Cumulative change (%)	-11.6 (-14.8 to -7.9)	-1.7 (-13.7 to 13.9)	1.2 (-0.9 to 3.3)	-17.3 (-33.2 to -4.8)	-9.8 (-19.6 to 0.6)	-34.3 (-40.4 to -27.5)	38.4 (1.8–105.2)	-33.8 (-83.7 to 62.7)	-50.8 (-67.7 to -29.0)	4.9 (-11.1 to 35.8)	-41.9 (-47.9 to -35.5)
Latvia	Prevalence Number: 1990	646,341 (639,838–652,763)	176,061 (163,071–189,248)	246,039 (238,084–254,497)	49,502 (43,581–56,784)	57,835 (55,122–61,064)	1,588 (1,485–1,708)	14,214 (11,983–16,713)	3,251 (1,276–6,455)	1,618 (1,196–2,077)	0 (0–0)	6,777 (5,933–7,802)

© 2017 Global Burden of Disease Child and Adolescent Health Collaboration. JAMA Pediatrics.

eTable 3. Prevalent Cases, Rates (per 100,000 population), Years Lived with Disability (YLDs), and Cumulative Percent Change with 95% Uncertainty Interval (UI) for the Top 10 Global Causes of YLDs in Children and Adolescents in 195 Countries and Territories, Aged 0 to 19 Years, Both Sexes, 1990 and 2015. *Note: best viewed by enlarging in browser.*

Location	Measure	All causes	Iron-deficiency anemia	Skin and subcutaneous diseases	Asthma	Hemoglobinopathies and hemolytic anemias	Diarrheal diseases	Congenital anomalies	Protein-energy malnutrition	Epilepsy	Malaria	Neonatal preterm birth complications
Latvia	Prevalence Number: 2015	335,910 (331,832–339,776)	94,861 (87,664–102,646)	126,874 (122,573–131,186)	20,475 (16,874–24,538)	27,914 (26,565–29,291)	596 (563–628)	7,343 (6,361–8,729)	1,041 (424–2,021)	779 (586–1,010)	0 (0–0)	2,963 (2,575–3,424)
Latvia	Prevalence Number: Cumulative change (%)	-48.0 (-48.8 to -47.3)	-46.0 (-50.5 to -39.2)	-48.4 (-50.5 to -46.2)	-58.6 (-64.3 to -52.2)	-51.7 (-55.1 to -48.4)	-62.4 (-64.3 to -60.6)	-47.8 (-58.2 to -35.9)	-59.4 (-89.6 to -1.2)	-50.8 (-66.5 to -32.1)	-46.7 (-47.3 to -45.9)	-56.1 (-62.8 to -48.9)
Latvia	Prevalence Rate: 1990	87,134.9 (86,244.5–88,001.5)	23,976.4 (22,219.9–25,731.7)	32,994.9 (31,934.7–34,142.6)	6,676.4 (5,880.9–7,658.0)	7,818.5 (7,451.6–8,257.2)	217.0 (202.9–233.1)	1,919.5 (1,618.7–2,256.2)	443.7 (174.2–880.9)	218.0 (161.1–279.9)	0.0 (0.0–0.0)	935.1 (821.1–1,073.2)
Latvia	Prevalence Rate: 2015	87,212.8 (86,166.1–88,216.8)	24,585.9 (22,702.8–26,578.5)	33,324.4 (32,222.9–34,468.7)	5,354.5 (4,401.1–6,416.8)	7,268.4 (6,916.4–7,626.3)	153.3 (144.9–161.3)	1,908.2 (1,654.8–2,266.8)	269.0 (109.5–522.1)	203.0 (153.0–262.9)	0.0 (0.0–0.0)	777.9 (676.7–897.8)
Latvia	Prevalence Rate: Cumulative change (%)	0.1 (-1.4 to 1.5)	2.7 (-5.8 to 15.6)	1.0 (-3.1 to 5.5)	-19.8 (-30.6 to -7.5)	-7.0 (-13.5 to -0.7)	-29.3 (-32.9 to -25.9)	0.4 (-19.5 to 23.4)	-23.1 (-80.3 to 86.9)	-4.8 (-35.2 to 31.3)	0.0 (-0.0 to 0.0)	-16.5 (-29.0 to -3.1)
Latvia	YLDs Number: 1990	40,418 (30,855–51,297)	6,294 (4,457–8,510)	4,773 (3,189–6,817)	2,250 (1,516–3,085)	687 (487–927)	261 (186–348)	1,109 (726–1,553)	407 (144–844)	582 (367–848)	0 (0–0)	849 (656–1,097)
Latvia	YLDs Number: 2015	18,969 (14,455–24,083)	3,383 (2,368–4,679)	2,475 (1,663–3,545)	932 (616–1,298)	330 (233–453)	98 (69–130)	728 (427–1,153)	130 (49–250)	175 (110–251)	0 (0–0)	278 (209–360)
Latvia	YLDs Number: Cumulative change (%)	-53.1 (-54.6 to -51.1)	-46.1 (-51.7 to -35.7)	-48.1 (-49.2 to -47.1)	-58.5 (-64.2 to -51.9)	-51.8 (-57.0 to -45.5)	-62.3 (-65.7 to -58.8)	-34.4 (-49.8 to -6.4)	-59.3 (-89.5 to 1.1)	-69.1 (-79.6 to -56.3)	-42.8 (-51.5 to -25.5)	-67.2 (-70.5 to -63.7)
Latvia	YLDs Rate: 1990	5,444.0 (4,159.2–6,908.9)	861.9 (610.6–1,163.2)	640.9 (429.0–915.4)	303.5 (204.3–415.4)	94.2 (66.9–127.0)	35.8 (25.6–47.6)	149.8 (98.1–209.9)	55.6 (19.7–115.2)	78.4 (49.6–114.4)	0.0 (0.0–0.0)	114.8 (88.7–148.3)
Latvia	YLDs Rate: 2015	4,968.3 (3,787.2–6,302.3)	878.6 (615.7–1,216.1)	647.5 (433.9–929.2)	243.8 (161.3–340.0)	87.0 (61.4–118.9)	25.3 (17.9–33.5)	189.2 (110.7–301.1)	33.7 (12.8–64.6)	45.6 (28.8–65.7)	0.0 (0.0–0.0)	72.3 (54.4–93.7)
Latvia	YLDs Rate: Cumulative change (%)	-8.7 (-11.8 to -5.0)	2.1 (-8.3 to 21.7)	1.0 (-1.0 to 2.9)	-19.6 (-30.6 to -6.6)	-7.4 (-17.3 to 4.5)	-29.2 (-35.4 to -22.7)	26.1 (-3.6 to 79.9)	-22.9 (-80.2 to 91.5)	-40.3 (-60.7 to -15.3)	6.1 (-10.2 to 39.1)	-37.0 (-43.4 to -30.2)
Lithuania	Prevalence Number: 1990	969,521 (959,347–978,646)	273,113 (247,567–297,535)	366,816 (354,946–378,538)	58,254 (50,241–67,576)	86,631 (82,013–91,642)	2,439 (2,290–2,613)	21,204 (18,187–25,061)	5,311 (2,234–10,222)	2,559 (1,897–3,266)	0 (0–0)	9,100 (7,929–10,460)
Lithuania	Prevalence Number: 2015	542,673 (536,327–548,158)	143,379 (132,905–156,715)	217,608 (211,023–224,620)	28,946 (23,902–34,933)	44,285 (42,038–46,530)	946 (893–1,001)	11,660 (10,090–13,786)	1,619 (649–3,270)	1,234 (911–1,578)	0 (0–0)	4,247 (3,656–4,952)
Lithuania	Prevalence Number: Cumulative change (%)	-44.0 (-44.8 to -43.3)	-47.3 (-55.0 to -39.1)	-40.7 (-43.2 to -38.2)	-50.3 (-55.2 to -44.6)	-48.8 (-52.5 to -45.4)	-61.2 (-63.0 to -59.4)	-44.5 (-55.7 to -32.3)	-62.5 (-90.0 to -7.7)	-50.7 (-66.4 to -31.1)	-47.3 (-48.6 to -46.2)	-53.2 (-60.3 to -45.9)
Lithuania	Prevalence Rate: 1990	88,068.0 (87,135.3–88,910.6)	24,995.1 (22,634.7–27,270.2)	33,077.8 (32,000.6–34,127.9)	5,257.9 (4,531.2–6,105.9)	7,893.4 (7,474.4–8,348.1)	228.2 (214.2–244.5)	1,931.7 (1,656.7–2,283.1)	502.7 (211.5–967.6)	231.6 (171.6–295.4)	0.0 (0.0–0.0)	845.7 (738.0–969.9)
Lithuania	Prevalence Rate: 2015	87,882.4 (86,817.6–88,792.9)	24,270.3 (22,515.6–26,533.3)	33,518.0 (32,482.0–34,574.3)	4,726.6 (3,883.2–5,694.8)	7,227.9 (6,862.8–7,580.7)	164.7 (155.2–174.3)	1,898.4 (1,644.0–2,244.6)	281.0 (112.7–567.3)	197.8 (145.8–252.4)	0.0 (0.0–0.0)	711.9 (614.7–826.4)
Lithuania	Prevalence Rate: Cumulative change (%)	-0.2 (-1.6 to 1.2)	-2.5 (-16.6 to 13.2)	1.4 (-3.1 to 5.6)	-10.2 (-19.2 to 0.4)	-8.3 (-14.7 to -2.2)	-27.8 (-31.2 to -24.4)	-0.9 (-20.9 to 20.9)	-31.4 (-81.7 to 69.3)	-12.6 (-40.6 to 22.1)	0.0 (-0.0 to 0.0)	-15.5 (-27.9 to -2.9)
Lithuania	YLDs Number: 1990	58,728 (44,319–74,589)	9,842 (6,932–13,487)	7,179 (4,859–10,212)	2,647 (1,784–3,637)	1,039 (731–1,410)	402 (287–532)	1,616 (1,064–2,282)	670 (258–1,384)	919 (586–1,298)	0 (0–0)	1,056 (809–1,352)
Lithuania	YLDs Number: 2015	31,346 (23,961–39,639)	5,068 (3,571–6,832)	4,183 (2,817–5,974)	1,317 (871–1,842)	487 (347–656)	156 (112–207)	1,241 (741–1,885)	203 (75–402)	275 (171–407)	0 (0–0)	398 (299–514)
Lithuania	YLDs Number: Cumulative change (%)	-46.6 (-48.7 to -44.3)	-48.2 (-55.6 to -38.6)	-41.8 (-43.2 to -40.3)	-50.3 (-55.6 to -44.4)	-53.0 (-58.4 to -45.7)	-61.2 (-64.9 to -57.3)	-23.4 (-39.7 to -1.0)	-62.4 (-90.2 to -7.9)	-69.3 (-80.7 to -54.7)	-47.3 (-58.7 to -32.4)	-62.3 (-66.2 to -58.1)
Lithuania	YLDs Rate: 1990	5,308.8 (4,002.5–6,746.3)	902.9 (636.6–1,237.7)	649.4 (440.3–923.0)	239.0 (161.0–329.4)	95.3 (67.0–129.5)	37.6 (26.9–49.8)	147.3 (97.0–208.1)	63.5 (24.5–131.0)	83.3 (53.0–117.6)	0.0 (0.0–0.0)	96.2 (73.8–123.1)
Lithuania	YLDs Rate: 2015	4,944.5 (3,772.0–6,274.1)	884.8 (623.8–1,193.1)	656.2 (445.3–938.2)	215.2 (142.2–300.3)	84.5 (60.2–113.3)	27.1 (19.5–36.0)	202.3 (120.6–306.3)	35.2 (13.0–69.8)	44.2 (27.4–65.1)	0.0 (0.0–0.0)	65.0 (48.7–84.2)
Lithuania	YLDs Rate: Cumulative change (%)	-6.8 (-10.6 to -2.7)	-1.5 (-15.1 to 16.9)	1.1 (-1.0 to 3.2)	-10.0 (-19.6 to 0.7)	-11.1 (-21.1 to 2.6)	-27.8 (-34.8 to -20.7)	37.0 (8.1–76.7)	-31.2 (-82.0 to 68.9)	-45.6 (-65.7 to -20.0)	3.5 (-18.5 to 32.8)	-32.4 (-39.5 to -25.0)
Moldova	Prevalence Number: 1990	1,364,341 (1,346,242–1,380,775)	407,792 (380,239–454,777)	517,386 (499,261–533,677)	70,905 (59,376–84,074)	130,217 (123,551–136,432)	7,502 (6,904–8,221)	26,277 (22,048–31,666)	13,277 (5,615–25,071)	4,600 (3,407–5,875)	0 (0–0)	15,855 (13,837–18,419)
Moldova	Prevalence Number: 2015	772,216 (761,901–782,090)	230,812 (215,277–255,185)	305,490 (294,801–315,716)	37,642 (31,133–44,859)	68,257 (64,865–71,768)	2,699 (2,461–2,945)	15,240 (13,029–18,307)	2,475 (1,164–4,518)	1,816 (1,316–2,383)	0 (0–0)	8,563 (7,476–10,071)
Moldova	Prevalence Number: Cumulative change (%)	-43.4 (-44.3 to -42.4)	-43.2 (-51.3 to -34.7)	-40.9 (-43.5 to -38.3)	-46.9 (-52.0 to -41.8)	-47.5 (-51.1 to -43.9)	-64.0 (-66.3 to -61.7)	-41.4 (-53.5 to -26.8)	-76.9 (-92.6 to -45.8)	-59.6 (-73.1 to -43.2)	-46.8 (-47.8 to -45.8)	-45.7 (-54.1 to -36.4)
Moldova	Prevalence Rate: 1990	87,655.5 (86,519.6–88,676.5)	25,996.5 (24,340.3–28,895.7)	33,733.0 (32,572.3–34,789.0)	4,576.5 (3,836.3–5,409.9)	8,354.0 (7,928.8–8,751.4)	469.7 (432.9–514.1)	1,685.8 (1,415.1–2,029.6)	821.1 (347.3–1,550.7)	298.8 (221.3–381.6)	0.0 (0.0–0.0)	1,024.5 (894.8–1,188.6)
Moldova	Prevalence Rate: 2015	87,497.3 (86,307.4–88,650.4)	26,722.1 (24,887.9–29,654.1)	33,895.7 (32,704.8–35,025.3)	4,276.7 (3,533.1–5,107.9)	7,780.0 (7,396.3–8,179.3)	321.6 (293.0–351.2)	1,734.3 (1,481.2–2,084.8)	297.7 (140.1–543.6)	204.5 (148.1–268.2)	0.0 (0.0–0.0)	1,005.1 (880.6–1,174.7)
Moldova	Prevalence Rate: Cumulative change (%)	-0.2 (-1.9 to 1.5)	3.1 (-11.6 to 18.4)	0.5 (-3.9 to 5.0)	-6.5 (-15.4 to 3.1)	-6.8 (-13.0 to -0.4)	-31.5 (-36.0 to -27.3)	4.0 (-17.8 to 29.9)	-55.0 (-85.7 to 5.4)	-29.9 (-53.3 to -1.7)	-0.0 (-0.0 to 0.0)	-1.4 (-16.3 to 15.4)
Moldova	YLDs Number: 1990	87,126 (66,391–110,439)	14,951 (10,541–20,614)	11,635 (7,956–16,286)	3,220 (2,135–4,422)	1,533 (1,089–2,094)	1,233 (886–1,630)	2,152 (1,346–3,117)	1,663 (645–3,310)	1,646 (1,081–2,294)	0 (0–0)	2,000 (1,561–2,515)
Moldova	YLDs Number: 2015	46,233 (35,352–58,624)	8,423 (5,924–11,638)	6,731 (4,570–9,450)	1,711 (1,124–2,362)	791 (564–1,081)	444 (316–591)	1,555 (922–2,326)	309 (131–576)	432 (270–627)	0 (0–0)	875 (670–1,112)
Moldova	YLDs Number: Cumulative change (%)	-46.9 (-49.2 to -44.7)	-43.4 (-54.8 to -32.6)	-42.2 (-43.4 to -40.8)	-46.8 (-52.0 to -41.5)	-48.3 (-55.8 to -40.2)	-64.0 (-66.9 to -60.9)	-27.9 (-41.5 to -11.3)	-76.8 (-92.7 to -45.4)	-73.1 (-82.5 to -60.2)	-44.3 (-57.3 to -28.5)	-56.2 (-60.3 to -51.8)
Moldova	YLDs Rate: 1990	5,654.6 (4,307.2–7,160.4)	949.3 (671.1–1,308.0)	752.1 (512.2–1,054.2)	207.8 (137.9–285.4)	97.6 (69.6–133.5)	77.2 (55.6–102.2)	138.0 (86.3–199.9)	102.9 (39.9–204.7)	106.9 (70.0–149.0)	0.0 (0.0–0.0)	128.1 (99.8–161.1)
Moldova	YLDs Rate: 2015	5,184.4 (3,956.3–6,589.3)	987.2 (692.2–1,365.8)	754.8 (513.1–1,058.5)	194.4 (127.9–269.1)	93.0 (66.2–127.3)	52.9 (37.6–70.6)	177.1 (104.9–265.6)	37.2 (15.9–69.3)	48.7 (30.6–70.7)	0.0 (0.0–0.0)	99.8 (76.6–126.6)
Moldova	YLDs Rate: Cumulative change (%)	-8.3 (-12.3 to -4.3)	4.4 (-16.1 to 24.4)	0.4 (-1.5 to 2.4)	-6.3 (-15.6 to 3.4)	-4.5 (-17.9 to 10.4)	-31.4 (-36.8 to -25.7)	28.0 (3.8–57.4)	-55.0 (-85.9 to 6.3)	-53.3 (-69.9 to -30.7)	5.7 (-19.0 to 36.6)	-22.1 (-29.5 to -14.3)
Russia	Prevalence Number: 1990	38,796,274 (38,385,342–39,236,536)	12,716,055 (11,791,403–13,948,055)	14,730,435 (14,261,690–15,196,820)	2,897,141 (2,485,188–3,293,217)	3,454,065 (3,296,540–3,627,044)	162,264 (150,558–175,832)	766,678 (651,124–917,100)	197,387 (95,820–335,394)	111,333 (82,093–141,548)	0 (0–0)	400,518 (347,201–465,843)
Russia	Prevalence Number: 2015	24,552,311 (24,232,008–24,881,139)	8,012,935 (7,326,505–8,980,149)	9,052,668 (8,737,527–9,349,626)	1,063,471 (868,265–1,288,346)	2,045,506 (1,948,754–2,156,469)	80,741 (74,455–86,793)	488,883 (423,177–576,015)	117,996 (49,100–224,425)	48,770 (35,793–63,356)	0 (0–0)	253,114 (221,244–291,702)
Russia	Prevalence Number: Cumulative change (%)	-36.7 (-37.8 to -35.6)	-36.8 (-44.2 to -25.7)	-38.5 (-41.1 to -35.9)	-63.3 (-68.0 to -58.4)	-40.7 (-44.7 to -36.5)	-50.2 (-53.6 to -47.1)	-35.7 (-48.7 to -22.6)	-30.1 (-78.5 to 67.0)	-55.1 (-69.6 to -37.5)	-30.4 (-33.1 to -28.0)	-36.5 (-45.3 to -26.7)
Russia	Prevalence Rate: 1990	88,395.9 (87,439.1–89,414.9)	28,980.9 (26,785.3–31,902.0)	33,682.7 (32,620.0–34,737.0)	6,536.2 (5,612.5–7,424.9)	7,889.0 (7,529.7–8,282.1)	380.7 (353.1–411.9)	1,752.4 (1,488.1–2,095.5)	469.8 (228.0–798.2)	253.4 (187.0–322.3)	0.0 (0.0–0.0)	946.1 (823.5–1,095.7)
Russia	Prevalence Rate: 2015	88,038.8 (87,017.8–89,010.6)	28,261.5 (26,165.6–31,158.8)	33,939.9 (32,799.7–35,046.6)	3,983.9 (3,259.4–4,802.0)	7,263.5 (6,920.9–7,645.5)	251.5 (232.6–269.6)	1,729.9 (1,497.6–2,037.5)	348.2 (144.9–662.2)	174.6 (128.0–227.1)	0.0 (0.0–0.0)	845.2 (734.2–980.6)
Russia	Prevalence Rate: Cumulative change (%)	-0.4 (-1.9 to 1.1)	-2.1 (-13.4 to 13.8)	0.8 (-3.4 to 5.2)	-39.0 (-46.6 to -31.1)	-7.9 (-14.0 to -1.4)	-33.9 (-38.3 to -29.8)	-0.4 (-20.6 to 20.0)	-13.3 (-73.3 to 107.1)	-29.5 (-52.2 to -1.7)	-0.0 (-1.5 to 1.8)	-10.3 (-22.5 to 4.0)
Russia	YLDs Number: 1990	2,572,800 (1,952,579–3,262,382)	496,595 (355,332–675,221)	303,696 (207,975–427,727)	131,274 (89,369–180,828)	43,521 (30,715–58,957)	26,646 (19,124–35,207)	48,792 (33,308–66,423)	24,708 (10,621–45,067)	40,038 (25,706–57,818)	0 (0–0)	52,306 (40,637–66,879)
Russia	YLDs Number: 2015	1,461,094 (1,100,016–1,866,559)	306,959 (213,991–415,449)	190,627 (131,128–267,549)	48,275 (31,470–67,445)	24,524 (17,432–32,936)	13,301 (9,517–17,714)	39,611 (24,912–57,393)	14,841 (5,556–28,782)	10,841 (6,708–15,727)	0 (0–0)	24,127 (18,576–31,236)
Russia	YLDs Number: Cumulative change (%)	-43.2 (-45.7 to -40.0)	-38.0 (-45.7 to -22.6)	-37.2 (-38.6 to -35.6)	-63.2 (-67.9 to -58.3)	-43.4 (-51.0 to -28.8)	-50.0 (-55.0 to -44.9)	-19.1 (-31.8 to -5.1)	-29.8 (-78.4 to 67.4)	-72.2 (-82.0 to -59.2)	-29.5 (-48.8 to -6.6)	-53.9 (-58.3 to -49.4)
Russia	YLDs Rate: 1990	5,867.6 (4,453.9–7,442.6)	1,123.6 (803.2–1,525.9)	692.9 (474.3–975.9)	296.2 (202.0–408.2)	98.7 (69.4–133.6)	62.5 (44.9–82.7)	111.5 (76.1–152.1)	58.8 (25.3–107.2)	91.1 (58.5–131.3)	0.0 (0.0–0.0)	119.4 (92.8–152.9)
Russia	YLDs Rate: 2015	5,425.6 (4,091.7–6,880.9)	1,089.3 (765.6–1,470.5)	698.1 (478.9–981.5)	180.8 (117.7–250.8)	87.3 (62.4–116.5)	41.4 (29.8–55.0)	140.0 (88.2–202.8)	43.8 (16.4–84.9)	38.7 (24.0–56.2)	0.0 (0.0–0.0)	85.5 (65.9–110.4)
Russia	YLDs Rate: Cumulative change (%)	-7.5 (-11.2 to -3.0)	-2.7 (-14.5 to 18.9)	0.8 (-1.2 to 2.9)	-38.9 (-46.5 to -30.9)	-11.1 (-23.0 to 9.6)	-33.7 (-40.2 to -27.0)	25.1 (5.5–46.9)	-13.0 (-73.3 to 107.4)	-56.3 (-72.0 to -35.8)	4.1 (-24.2 to 37.4)	-28.4 (-35.3 to -21.4)
Ukraine	Prevalence Number: 1990	12,729,229 (12,559,055–12,871,751)	3,156,684 (2,778,085–3,480,556)	4,886,760 (4,728,456–5,044,868)	1,181,769 (971,763–1,408,005)	1,176,041 (1,113,716–1,237,387)	45,724 (42,308–49,336)	275,802 (236,403–325,455)	50,849 (20,678–96,220)	39,240 (29,533–49,693)	0 (0–0)	134,788 (115,946–159,198)
Ukraine	Prevalence Number: 2015	6,987,606 (6,887,745–7,077,798)	1,663,623 (1,491,301–1,806,232)	2,692,130 (2,605,745–2,773,399)	422,399 (342,631–504,612)	605,724 (575,458–632,741)	20,550 (18,987–22,049)	148,637 (131,964–169,959)	14,955 (5,704–29,912)	17,680 (13,017–22,915)	0 (0–0)	78,628 (68,975–91,155)
Ukraine	Prevalence Number: Cumulative change (%)	-45.1 (-46.0 to -44.2)	-47.1 (-53.2 to -39.5)	-44.9 (-47.2 to -42.6)	-64.0 (-71.3 to -58.6)	-48.4 (-52.0 to -44.8)	-55.0 (-57.5 to -52.4)	-45.6 (-56.3 to -35.0)	-63.8 (-90.2 to -9.5)	-53.9 (-69.1 to -34.1)	-41.0 (-43.4 to -38.4)	-41.3 (-50.7 to -30.9)

© 2017 Global Burden of Disease Child and Adolescent Health Collaboration. JAMA Pediatrics.

eTable 3. Prevalent Cases, Rates (per 100,000 population), Years Lived with Disability (YLDs), and Cumulative Percent Change with 95% Uncertainty Interval (UI) for the Top 10 Global Causes of YLDs in Children and Adolescents in 195 Countries and Territories, Aged 0 to 19 Years, Both Sexes, 1990 and 2015. *Note: best viewed by enlarging in browser.*

Location	Measure	All causes	Iron-deficiency anemia	Skin and subcutaneous diseases	Asthma	Hemoglobinopathies and hemolytic anemias	Diarrheal diseases	Congenital anomalies	Protein-energy malnutrition	Epilepsy	Malaria	Neonatal preterm birth complications
Ukraine	Prevalence Rate: 1990	87,814.2 (86,580.0–88,842.2)	22,314.8 (19,513.5–24,730.1)	33,274.7 (32,200.9–34,333.0)	7,960.3 (6,538.2–9,491.7)	8,163.6 (7,732.9–8,588.9)	343.3 (317.2–370.6)	1,915.6 (1,641.4–2,261.6)	398.8 (162.2–754.8)	267.8 (201.3–338.6)	0.0 (0.0–0.0)	983.5 (852.6–1,151.6)
Ukraine	Prevalence Rate: 2015	86,857.7 (85,687.4–87,915.4)	20,819.5 (18,968.1–22,363.6)	33,422.5 (32,342.2–34,477.5)	5,465.9 (4,416.2–6,559.4)	7,517.6 (7,146.4–7,849.4)	236.4 (219.1–253.3)	1,837.0 (1,629.8–2,101.0)	162.6 (62.0–325.1)	221.0 (162.3–286.6)	0.0 (0.0–0.0)	937.1 (819.3–1,091.0)
Ukraine	Prevalence Rate: Cumulative change (%)	-1.1 (-2.7 to 0.6)	-6.4 (-17.0 to 5.8)	0.5 (-3.8 to 4.6)	-30.9 (-44.9 to -20.6)	-7.8 (-14.1 to -1.3)	-31.1 (-35.0 to -27.1)	-3.2 (-22.3 to 15.6)	-49.8 (-86.5 to 25.5)	-15.6 (-43.6 to 19.8)	-0.0 (-0.0 to 0.0)	-4.2 (-19.0 to 13.2)
Ukraine	YLDs Number: 1990	803,795 (608,383–1,021,690)	107,074 (73,099–147,952)	98,613 (66,660–139,719)	53,674 (35,449–75,935)	15,948 (11,362–21,644)	7,530 (5,402–10,093)	19,580 (13,449–26,825)	6,400 (2,313–12,843)	14,033 (9,101–19,461)	0 (0–0)	17,070 (13,074–21,947)
Ukraine	YLDs Number: 2015	400,021 (303,643–505,442)	54,601 (38,518–74,339)	54,830 (37,219–77,932)	19,225 (12,632–26,772)	7,759 (5,529–10,407)	3,388 (2,409–4,517)	12,042 (8,352–16,664)	1,872 (664–3,960)	4,965 (3,193–7,028)	0 (0–0)	7,397 (5,648–9,579)
Ukraine	YLDs Number: Cumulative change (%)	-50.2 (-51.6 to -48.9)	-48.8 (-55.0 to -44.0)	-44.4 (-45.6 to -43.0)	-64.0 (-71.3 to -58.2)	-51.3 (-54.4 to -47.9)	-54.9 (-58.9 to -50.3)	-38.2 (-47.5 to -27.6)	-63.8 (-90.2 to -10.5)	-63.7 (-76.1 to -47.8)	-42.7 (-53.1 to -27.8)	-56.6 (-60.6 to -52.7)
Ukraine	YLDs Rate: 1990	5,481.4 (4,147.6–6,971.2)	753.3 (511.7–1,043.0)	675.8 (457.5–957.3)	361.6 (238.9–510.6)	109.9 (78.6–149.0)	56.6 (40.6–75.8)	136.1 (93.5–186.5)	50.2 (18.1–100.7)	95.8 (62.0–132.7)	0.0 (0.0–0.0)	118.6 (90.8–152.2)
Ukraine	YLDs Rate: 2015	5,026.0 (3,814.2–6,373.8)	704.0 (501.4–953.6)	679.3 (460.1–964.7)	248.7 (163.9–347.4)	102.2 (73.1–137.5)	39.0 (27.8–51.9)	148.7 (102.9–206.3)	20.4 (7.2–43.0)	62.0 (39.8–87.7)	0.0 (0.0–0.0)	91.8 (70.0–118.6)
Ukraine	YLDs Rate: Cumulative change (%)	-8.3 (-10.8 to -5.9)	-6.1 (-16.7 to 4.1)	0.5 (-1.5 to 2.7)	-30.8 (-45.0 to -20.0)	-6.8 (-12.9 to -0.8)	-31.0 (-37.1 to -23.9)	9.7 (-6.6 to 28.7)	-49.9 (-86.4 to 24.0)	-33.7 (-56.5 to -4.1)	2.5 (-16.1 to 27.7)	-22.6 (-29.8 to -15.5)
Central Europe	Prevalence Number: 1990	34,720,100 (34,540,871–34,882,715)	9,515,592 (9,115,160–9,964,004)	13,070,365 (12,877,696–13,269,252)	2,078,320 (1,763,755–2,398,899)	3,668,774 (3,437,875–3,956,815)	61,618 (57,250–66,485)	728,267 (676,705–792,136)	171,546 (154,628–189,996)	133,262 (113,576–154,461)	0 (0–0)	370,856 (335,519–410,704)
Central Europe	Prevalence Number: 2015	20,577,466 (20,465,381–20,687,461)	5,438,029 (5,246,889–5,642,515)	7,978,808 (7,871,072–8,097,352)	1,063,290 (879,242–1,261,511)	1,860,190 (1,739,624–2,018,126)	24,565 (23,075–26,190)	426,788 (400,030–455,156)	86,646 (77,118–97,046)	64,416 (53,907–76,506)	0 (0–0)	188,351 (171,241–206,798)
Central Europe	Prevalence Number: Cumulative change (%)	-40.7 (-41.1 to -40.4)	-42.8 (-46.0 to -40.0)	-39.0 (-40.0 to -37.9)	-48.9 (-52.8 to -44.8)	-49.2 (-53.6 to -44.5)	-60.1 (-62.0 to -58.5)	-41.3 (-46.1 to -36.3)	-49.3 (-56.5 to -41.1)	-51.5 (-58.3 to -44.2)	-40.1 (-40.9 to -39.2)	-49.1 (-52.9 to -45.6)
Central Europe	Prevalence Rate: 1990	89,145.0 (88,680.4–89,569.4)	24,926.5 (23,810.5–26,163.4)	32,982.1 (32,495.0–33,476.2)	5,229.2 (4,443.6–6,036.0)	9,460.9 (8,866.9–10,202.6)	173.3 (160.9–187.6)	1,880.2 (1,747.8–2,046.0)	496.4 (447.4–549.8)	340.4 (290.2–394.3)	0.0 (0.0–0.0)	1,004.6 (911.9–1,110.1)
Central Europe	Prevalence Rate: 2015	88,407.9 (87,911.1–88,895.3)	24,068.4 (23,231.9–24,967.7)	33,409.4 (32,947.1–33,899.6)	4,588.6 (3,781.9–5,473.9)	8,037.2 (7,517.9–8,714.7)	110.2 (103.4–117.5)	1,839.5 (1,724.5–1,960.9)	390.6 (347.6–437.4)	276.2 (230.1–328.5)	0.0 (0.0–0.0)	832.5 (757.6–912.7)
Central Europe	Prevalence Rate: Cumulative change (%)	-0.8 (-1.4 to -0.2)	-3.4 (-8.8 to 1.7)	1.3 (-0.4 to 3.0)	-12.3 (-19.2 to -5.2)	-14.9 (-22.2 to -7.0)	-36.4 (-39.5 to -33.8)	-2.0 (-10.1 to 6.3)	-21.0 (-32.2 to -8.3)	-18.6 (-30.0 to -6.3)	0.0 (-0.0 to 0.0)	-17.0 (-22.9 to -11.3)
Central Europe	YLDs Number: 1990	2,099,909 (1,601,314–2,657,004)	334,470 (236,033–457,161)	272,429 (186,137–382,977)	94,530 (64,199–128,742)	36,778 (26,635–49,492)	10,140 (7,293–13,383)	51,604 (34,919–71,012)	21,512 (14,777–29,071)	47,887 (33,858–62,441)	0 (0–0)	46,625 (36,618–59,502)
Central Europe	YLDs Number: 2015	1,161,550 (884,920–1,469,817)	191,996 (135,598–260,527)	165,603 (113,012–232,658)	48,427 (31,953–67,047)	18,791 (13,536–25,200)	4,049 (2,915–5,339)	40,841 (25,828–58,358)	10,900 (7,267–14,906)	14,567 (9,878–19,460)	0 (0–0)	18,275 (14,066–23,306)
Central Europe	YLDs Number: Cumulative change (%)	-44.7 (-45.7 to -43.7)	-42.5 (-46.4 to -39.2)	-39.2 (-39.8 to -38.7)	-48.8 (-52.7 to -44.5)	-48.9 (-51.4 to -46.4)	-60.0 (-62.3 to -57.8)	-21.2 (-31.4 to -11.2)	-49.2 (-56.5 to -41.1)	-69.5 (-75.0 to -63.6)	-40.2 (-44.9 to -34.3)	-60.8 (-63.4 to -58.1)
Central Europe	YLDs Rate: 1990	5,325.2 (4,062.7–6,740.3)	872.7 (617.5–1,192.9)	693.3 (473.5–973.2)	237.9 (161.2–325.8)	93.8 (67.9–126.4)	28.5 (20.6–37.7)	133.3 (90.2–183.3)	62.2 (42.8–84.1)	122.4 (86.7–159.5)	0.0 (0.0–0.0)	120.2 (94.1–153.6)
Central Europe	YLDs Rate: 2015	4,923.3 (3,755.5–6,233.0)	863.3 (609.3–1,170.9)	702.1 (480.1–985.0)	209.0 (137.5–290.2)	83.9 (60.5–112.6)	18.2 (13.1–23.9)	176.1 (111.3–251.5)	49.1 (32.8–67.2)	62.5 (42.4–83.3)	0.0 (0.0–0.0)	78.8 (60.8–100.5)
Central Europe	YLDs Rate: Cumulative change (%)	-7.5 (-9.3 to -5.8)	-0.9 (-8.0 to 5.2)	1.3 (0.4–2.1)	-12.2 (-19.0 to -5.1)	-10.6 (-15.2 to -6.2)	-36.3 (-39.9 to -32.5)	31.6 (14.5–48.2)	-20.8 (-32.3 to -8.2)	-48.8 (-58.0 to -38.8)	2.2 (-6.2 to 12.0)	-34.4 (-38.7 to -29.9)
Albania	Prevalence Number: 1990	1,258,461 (1,244,093–1,271,400)	496,227 (468,568–537,090)	433,805 (420,152–448,849)	66,966 (56,132–78,575)	170,570 (149,396–196,854)	4,276 (3,778–5,009)	23,376 (19,953–27,865)	18,705 (14,231–23,724)	7,506 (5,561–9,589)	0 (0–0)	15,228 (13,046–18,308)
Albania	Prevalence Number: 2015	707,020 (698,516–714,741)	199,395 (192,017–208,157)	274,913 (265,944–283,673)	32,925 (27,457–38,956)	83,924 (73,565–98,832)	1,122 (1,013–1,234)	13,953 (12,087–16,709)	5,647 (4,362–7,262)	3,932 (2,916–5,176)	0 (0–0)	7,226 (6,109–8,786)
Albania	Prevalence Number: Cumulative change (%)	-43.8 (-44.7 to -43.0)	-59.8 (-64.2 to -57.1)	-36.6 (-39.4 to -33.8)	-50.7 (-56.3 to -42.8)	-50.5 (-59.3 to -40.0)	-73.6 (-76.9 to -70.5)	-39.8 (-51.9 to -25.9)	-69.0 (-78.9 to -56.5)	-46.5 (-63.9 to -26.2)	-49.7 (-52.0 to -47.4)	-52.2 (-61.3 to -41.7)
Albania	Prevalence Rate: 1990	90,641.1 (89,634.1–91,548.5)	35,494.9 (33,574.8–38,298.4)	31,527.4 (30,553.8–32,622.2)	4,853.2 (4,066.9–5,697.6)	12,272.7 (10,749.1–14,164.6)	296.0 (262.1–346.2)	1,680.5 (1,434.6–2,003.5)	1,277.3 (971.8–1,620.0)	539.3 (398.0–687.7)	0.0 (0.0–0.0)	1,073.2 (917.4–1,291.2)
Albania	Prevalence Rate: 2015	88,359.9 (87,211.5–89,384.0)	25,853.8 (24,950.4–26,932.9)	31,990.3 (30,942.6–33,001.2)	4,201.2 (3,507.4–4,997.3)	10,538.6 (9,233.3–12,411.1)	157.1 (141.5–173.2)	1,756.8 (1,520.7–2,097.1)	803.0 (620.2–1,032.5)	498.5 (370.4–658.8)	0.0 (0.0–0.0)	943.7 (799.8–1,141.7)
Albania	Prevalence Rate: Cumulative change (%)	-2.5 (-4.0 to -1.1)	-27.1 (-34.7 to -22.6)	1.5 (-2.8 to 5.8)	-13.2 (-22.4 to 0.9)	-13.6 (-29.0 to 4.5)	-46.7 (-53.2 to -40.5)	5.4 (-15.8 to 29.7)	-35.5 (-56.1 to -9.4)	-5.6 (-35.8 to 30.6)	-0.0 (-0.0 to 0.0)	-11.3 (-27.9 to 7.3)
Albania	YLDs Number: 1990	82,269 (62,524–104,295)	19,349 (13,719–26,320)	8,299 (5,573–11,776)	3,041 (2,026–4,196)	2,105 (1,490–2,873)	698 (498–935)	1,399 (931–1,973)	2,334 (1,463–3,372)	2,681 (1,738–3,794)	0 (0–0)	1,340 (1,021–1,713)
Albania	YLDs Number: 2015	42,634 (32,432–53,596)	7,152 (5,120–9,637)	5,104 (3,394–7,342)	1,498 (992–2,063)	993 (712–1,336)	185 (131–248)	860 (564–1,241)	710 (441–1,043)	918 (560–1,352)	0 (0–0)	588 (446–752)
Albania	YLDs Number: Cumulative change (%)	-48.1 (-50.3 to -46.3)	-62.9 (-67.8 to -59.5)	-38.6 (-40.5 to -36.6)	-50.5 (-56.3 to -42.7)	-52.7 (-58.6 to -48.7)	-73.4 (-77.2 to -69.6)	-38.5 (-44.3 to -31.3)	-68.8 (-78.6 to -55.9)	-65.0 (-77.0 to -50.1)	-53.9 (-60.9 to -45.2)	-56.0 (-61.0 to -51.1)
Albania	YLDs Rate: 1990	5,950.4 (4,522.3–7,538.3)	1,386.3 (983.5–1,885.4)	601.0 (402.7–852.3)	220.4 (146.7–303.8)	151.7 (107.4–206.5)	48.3 (34.5–64.7)	100.6 (66.9–141.8)	159.4 (99.9–230.3)	192.7 (124.8–273.6)	0.0 (0.0–0.0)	96.3 (73.6–123.0)
Albania	YLDs Rate: 2015	5,133.9 (3,901.8–6,481.1)	967.8 (694.2–1,304.5)	609.5 (407.0–872.6)	191.3 (127.5–263.8)	132.6 (95.3–178.7)	25.9 (18.4–34.9)	108.5 (71.0–156.1)	101.0 (62.7–148.4)	116.5 (71.5–170.7)	0.0 (0.0–0.0)	74.3 (56.3–95.2)
Albania	YLDs Rate: Cumulative change (%)	-13.7 (-17.0 to -10.7)	-30.0 (-38.7 to -23.8)	1.4 (-0.7 to 3.8)	-12.9 (-22.5 to 0.7)	-12.4 (-22.7 to -5.2)	-46.2 (-54.0 to -38.5)	7.8 (-2.3 to 20.4)	-35.0 (-55.5 to -8.2)	-38.2 (-59.6 to -11.9)	-5.7 (-19.5 to 11.9)	-22.7 (-31.4 to -14.2)
Bosnia and Herzegovina	Prevalence Number: 1990	1,342,340 (1,327,184–1,355,174)	404,698 (382,078–437,237)	488,995 (473,800–502,708)	50,111 (41,174–60,182)	140,259 (118,816–167,432)	3,464 (3,178–3,777)	27,603 (23,806–32,328)	12,587 (9,662–16,124)	9,135 (6,681–11,928)	0 (0–0)	20,500 (17,864–24,143)
Bosnia and Herzegovina	Prevalence Number: 2015	661,440 (654,435–667,447)	184,855 (173,731–201,404)	253,921 (245,202–262,219)	27,590 (22,659–32,920)	59,221 (50,144–71,518)	1,128 (1,023–1,245)	13,169 (11,350–15,650)	2,456 (1,845–3,145)	2,967 (2,200–3,890)	0 (0–0)	7,327 (6,306–8,604)
Bosnia and Herzegovina	Prevalence Number: Cumulative change (%)	-50.7 (-51.4 to -50.1)	-54.2 (-58.5 to -49.6)	-48.1 (-50.3 to -45.9)	-44.8 (-50.5 to -37.7)	-57.4 (-66.7 to -46.9)	-67.4 (-69.6 to -65.4)	-51.9 (-61.5 to -41.3)	-80.0 (-86.6 to -72.0)	-66.7 (-77.5 to -52.9)	-51.9 (-52.4 to -51.5)	-64.0 (-70.0 to -57.7)
Bosnia and Herzegovina	Prevalence Rate: 1990	90,097.4 (89,028.0–90,997.7)	27,649.5 (26,106.4–29,844.5)	32,271.9 (31,265.1–33,178.3)	3,313.0 (2,720.9–3,982.3)	9,464.5 (8,018.1–11,301.0)	254.6 (233.3–278.1)	1,863.0 (1,607.6–2,183.1)	944.7 (725.2–1,210.2)	612.2 (447.6–798.0)	0.0 (0.0–0.0)	1,469.0 (1,285.9–1,715.1)
Bosnia and Herzegovina	Prevalence Rate: 2015	89,474.1 (88,479.9–90,338.1)	26,005.2 (24,349.4–28,461.2)	33,072.3 (31,995.1–34,130.3)	3,739.1 (3,054.5–4,479.7)	8,082.1 (6,836.8–9,746.2)	168.7 (152.9–186.4)	1,793.7 (1,546.7–2,133.2)	375.4 (282.0–480.6)	399.3 (295.9–522.1)	0.0 (0.0–0.0)	1,048.5 (908.5–1,223.3)
Bosnia and Herzegovina	Prevalence Rate: Cumulative change (%)	-0.7 (-2.0 to 0.7)	-5.8 (-15.0 to 4.7)	2.5 (-1.8 to 6.8)	13.1 (1.1–28.0)	-13.8 (-32.7 to 7.5)	-33.7 (-38.3 to -29.5)	-2.9 (-22.3 to 18.6)	-59.2 (-72.8 to -43.0)	-33.1 (-54.9 to -5.4)	0.0 (-0.0 to 0.0)	-28.3 (-39.5 to -16.4)
Bosnia and Herzegovina	YLDs Number: 1990	85,106 (64,786–108,108)	14,797 (10,411–20,381)	10,351 (7,077–14,515)	2,275 (1,504–3,163)	1,284 (907–1,745)	568 (410–759)	1,642 (1,104–2,275)	1,576 (1,000–2,251)	3,259 (2,067–4,663)	0 (0–0)	2,441 (1,905–3,087)
Bosnia and Herzegovina	YLDs Number: 2015	37,240 (28,457–47,157)	6,595 (4,622–8,909)	5,286 (3,577–7,492)	1,256 (823–1,740)	569 (405–765)	186 (133–248)	1,135 (639–1,799)	308 (198–449)	696 (430–1,022)	0 (0–0)	711 (548–902)
Bosnia and Herzegovina	YLDs Number: Cumulative change (%)	-56.2 (-58.4 to -54.1)	-55.4 (-60.8 to -48.6)	-49.0 (-50.1 to -47.8)	-44.6 (-50.8 to -37.3)	-55.6 (-60.1 to -50.6)	-67.2 (-70.6 to -63.7)	-31.0 (-49.9 to -1.0)	-80.0 (-86.6 to -71.8)	-78.0 (-85.8 to -68.4)	-50.8 (-57.4 to -40.4)	-70.8 (-73.8 to -67.7)
Bosnia and Herzegovina	YLDs Rate: 1990	5,682.1 (4,321.9–7,231.0)	1,013.2 (711.3–1,394.1)	688.8 (471.7–969.0)	150.5 (99.5–209.6)	87.2 (61.5–117.9)	41.8 (30.3–55.8)	110.9 (74.6–153.8)	118.3 (75.1–169.0)	218.5 (138.2–312.5)	0.0 (0.0–0.0)	164.7 (128.7–208.4)
Bosnia and Herzegovina	YLDs Rate: 2015	4,933.6 (3,760.1–6,267.3)	947.1 (663.3–1,278.7)	702.3 (476.3–993.9)	170.3 (112.5–237.1)	81.2 (57.9–109.1)	27.8 (19.8–37.2)	154.8 (87.3–244.7)	47.1 (30.4–68.7)	93.7 (58.0–137.6)	0.0 (0.0–0.0)	96.9 (74.6–123.1)
Bosnia and Herzegovina	YLDs Rate: Cumulative change (%)	-13.1 (-17.7 to -8.7)	-6.3 (-18.2 to 8.6)	1.9 (-0.1 to 4.0)	13.5 (0.8–28.9)	-6.7 (-16.4 to 4.5)	-33.3 (-40.2 to -26.2)	39.3 (1.2–99.9)	-59.2 (-72.7 to -42.5)	-55.9 (-71.7 to -36.7)	4.1 (-10.4 to 27.9)	-41.2 (-47.3 to -34.9)
Bulgaria	Prevalence Number: 1990	2,210,643 (2,193,189–2,226,972)	564,185 (522,810–597,850)	831,208 (804,108–858,758)	100,453 (83,956–118,687)	460,375 (434,639–486,253)	4,122 (3,853–4,423)	42,762 (36,891–50,014)	8,449 (6,248–11,041)	8,389 (6,011–11,123)	0 (0–0)	22,753 (19,774–26,749)
Bulgaria	Prevalence Number: 2015	1,113,933 (1,102,674–1,124,035)	304,680 (284,147–322,650)	409,128 (396,107–421,179)	55,576 (46,121–66,474)	195,873 (186,479–205,563)	1,937 (1,784–2,096)	22,217 (19,387–25,623)	4,565 (3,305–6,102)	3,701 (2,720–4,836)	0 (0–0)	11,538 (10,021–13,370)
Bulgaria	Prevalence Number: Cumulative change (%)	-49.6 (-50.2 to -49.1)	-45.9 (-50.8 to -41.0)	-50.8 (-52.8 to -48.7)	-44.5 (-51.7 to -36.9)	-57.4 (-60.4 to -54.5)	-53.0 (-55.3 to -50.9)	-47.6 (-57.8 to -36.6)	-44.3 (-64.1 to -17.8)	-54.7 (-69.4 to -36.2)	-45.2 (-47.0 to -43.4)	-49.0 (-57.1 to -40.3)
Bulgaria	Prevalence Rate: 1990	90,062.8 (89,298.0–90,757.3)	23,473.8 (21,659.8–24,957.9)	32,922.7 (31,849.2–34,006.4)	3,988.7 (3,327.1–4,723.8)	18,861.3 (17,810.3–19,918.4)	187.0 (174.4–200.9)	1,754.9 (1,515.2–2,048.3)	396.5 (293.3–518.2)	337.8 (243.9–446.8)	0.0 (0.0–0.0)	989.9 (865.3–1,155.7)
Bulgaria	Prevalence Rate: 2015	89,631.5 (88,761.7–90,418.3)	24,471.0 (22,980.3–25,828.3)	33,145.9 (32,117.1–34,136.1)	4,519.7 (3,748.4–5,403.3)	15,738.0 (14,984.3–16,517.4)	150.6 (139.0–162.7)	1,784.2 (1,555.9–2,058.6)	347.8 (251.9–465.0)	298.7 (219.5–390.3)	0.0 (0.0–0.0)	922.0 (800.7–1,069.6)

© 2017 Global Burden of Disease Child and Adolescent Health Collaboration. JAMA Pediatrics.

eTable 3. Prevalent Cases, Rates (per 100,000 population), Years Lived with Disability (YLDs), and Cumulative Percent Change with 95% Uncertainty Interval (UI) for the Top 10 Global Causes of YLDs in Children and Adolescents in 195 Countries and Territories, Aged 0 to 19 Years, Both Sexes, 1990 and 2015. *Note: best viewed by enlarging in browser.*

Location	Measure	All causes	Iron-deficiency anemia	Skin and subcutaneous diseases	Asthma	Hemoglobinopathies and hemolytic anemias	Diarrheal diseases	Congenital anomalies	Protein-energy malnutrition	Epilepsy	Malaria	Neonatal preterm birth complications
Bulgaria	Prevalence Rate: Cumulative change (%)	-0.5 (-1.6 to 0.6)	4.4 (-5.1 to 13.9)	0.7 (-3.5 to 5.0)	13.6 (-1.0 to 28.7)	-16.5 (-22.4 to -10.8)	-19.4 (-23.3 to -15.7)	2.5 (-17.4 to 24.1)	-9.5 (-41.7 to 33.4)	-9.3 (-38.8 to 27.9)	0.0 (-0.0 to 0.0)	-6.4 (-20.7 to 9.4)
Bulgaria	YLDs Number: 1990	131,846 (100,607–167,034)	20,242 (14,242–27,673)	17,054 (11,627–24,064)	4,569 (3,065–6,338)	2,520 (1,789–3,421)	679 (489–899)	3,371 (2,258–4,626)	1,065 (642–1,569)	3,012 (1,879–4,335)	0 (0–0)	2,789 (2,149–3,551)
Bulgaria	YLDs Number: 2015	61,279 (46,480–77,625)	11,029 (7,749–14,934)	8,539 (5,808–12,051)	2,530 (1,666–3,549)	1,121 (795–1,512)	319 (229–426)	2,016 (1,344–2,817)	574 (355–854)	846 (526–1,262)	0 (0–0)	1,103 (839–1,417)
Bulgaria	YLDs Number: Cumulative change (%)	-53.5 (-54.8 to -52.2)	-45.4 (-50.5 to -39.9)	-49.9 (-51.0 to -48.7)	-44.5 (-51.9 to -36.3)	-55.5 (-58.3 to -52.6)	-52.9 (-57.7 to -47.8)	-40.2 (-47.5 to -32.1)	-44.3 (-64.3 to -18.0)	-71.1 (-81.5 to -57.5)	-42.9 (-54.1 to -29.2)	-60.4 (-64.2 to -56.4)
Bulgaria	YLDs Rate: 1990	5,261.6 (4,020.1–6,668.2)	841.7 (592.7–1,154.0)	685.0 (466.8–964.9)	181.5 (121.2–252.2)	102.7 (72.9–139.1)	30.9 (22.2–40.9)	138.6 (93.1–190.4)	50.0 (30.1–73.6)	121.4 (75.9–174.9)	0.0 (0.0–0.0)	114.3 (88.1–145.4)
Bulgaria	YLDs Rate: 2015	4,972.3 (3,773.6–6,296.8)	891.0 (626.2–1,202.8)	688.6 (468.8–973.6)	205.7 (135.3–288.6)	91.2 (64.7–123.3)	24.8 (17.8–33.2)	161.9 (108.0–226.1)	43.8 (27.1–65.1)	68.3 (42.5–102.0)	0.0 (0.0–0.0)	88.8 (67.5–113.9)
Bulgaria	YLDs Rate: Cumulative change (%)	-5.5 (-8.1 to -3.0)	6.0 (-4.0 to 17.1)	0.5 (-1.5 to 2.6)	13.6 (-1.3 to 30.3)	-11.2 (-16.8 to -5.1)	-19.4 (-27.3 to -10.8)	16.9 (2.7–32.7)	-9.6 (-42.1 to 33.3)	-42.1 (-63.0 to -14.9)	5.8 (-14.7 to 30.8)	-22.3 (-29.7 to -14.5)
Croatia	Prevalence Number: 1990	1,137,528 (1,127,070–1,148,181)	328,209 (305,145–347,511)	433,201 (419,510–446,815)	63,082 (52,401–74,742)	87,588 (81,222–94,483)	1,566 (1,461–1,685)	18,873 (16,682–21,436)	7,003 (5,223–9,016)	4,487 (3,320–5,735)	0 (0–0)	11,107 (9,721–12,821)
Croatia	Prevalence Number: 2015	775,124 (767,909–782,214)	217,118 (200,962–237,725)	299,214 (290,122–308,366)	43,333 (35,593–51,946)	50,787 (46,840–55,218)	745 (703–792)	14,003 (12,379–16,282)	3,578 (2,599–4,793)	2,562 (1,874–3,294)	0 (0–0)	6,849 (5,955–7,919)
Croatia	Prevalence Number: Cumulative change (%)	-31.9 (-32.7 to -31.0)	-33.8 (-38.0 to -29.4)	-30.9 (-33.7 to -28.0)	-31.2 (-38.4 to -23.5)	-41.9 (-48.0 to -35.4)	-52.4 (-55.3 to -49.5)	-25.4 (-36.8 to -11.2)	-47.5 (-65.4 to -23.6)	-41.7 (-59.6 to -17.4)	-31.4 (-31.9 to -30.8)	-38.1 (-47.3 to -27.6)
Croatia	Prevalence Rate: 1990	88,519.6 (87,659.3–89,364.8)	26,125.0 (24,139.9–27,797.4)	32,975.3 (31,917.4–33,990.0)	4,778.4 (3,966.3–5,657.4)	6,859.9 (6,359.5–7,396.5)	136.2 (126.7–146.9)	1,479.7 (1,309.4–1,680.4)	636.9 (475.0–820.0)	348.0 (257.5–443.1)	0.0 (0.0–0.0)	919.3 (807.5–1,056.2)
Croatia	Prevalence Rate: 2015	88,320.6 (87,469.4–89,171.0)	25,475.5 (23,498.7–28,055.6)	33,248.1 (32,236.8–34,263.1)	4,899.2 (4,012.8–5,900.5)	5,834.1 (5,379.6–6,338.5)	91.6 (86.2–97.5)	1,604.7 (1,419.1–1,866.9)	454.2 (329.9–608.3)	291.2 (213.5–373.8)	0.0 (0.0–0.0)	818.0 (714.4–942.4)
Croatia	Prevalence Rate: Cumulative change (%)	-0.2 (-1.5 to 1.1)	-2.4 (-9.2 to 4.9)	0.9 (-3.3 to 5.1)	2.7 (-8.1 to 14.1)	-14.8 (-23.7 to -5.3)	-32.7 (-36.9 to -28.4)	9.0 (-7.7 to 29.6)	-26.7 (-51.7 to 6.6)	-14.5 (-40.8 to 20.8)	0.0 (-0.0 to 0.0)	-10.7 (-23.3 to 3.4)
Croatia	YLDs Number: 1990	69,071 (52,357–88,043)	12,019 (8,484–16,399)	8,955 (6,115–12,627)	2,871 (1,900–3,995)	1,123 (791–1,534)	257 (182–341)	1,563 (1,078–2,130)	880 (550–1,296)	1,616 (1,083–2,264)	0 (0–0)	1,422 (1,082–1,813)
Croatia	YLDs Number: 2015	44,825 (34,200–56,449)	7,940 (5,536–10,882)	6,176 (4,209–8,699)	1,972 (1,315–2,737)	694 (494–943)	123 (87–165)	1,573 (991–2,267)	449 (278–658)	576 (367–823)	0 (0–0)	683 (513–881)
Croatia	YLDs Number: Cumulative change (%)	-35.1 (-36.6 to -33.4)	-33.9 (-39.1 to -28.3)	-31.0 (-32.5 to -29.5)	-31.1 (-38.7 to -23.4)	-38.1 (-43.1 to -33.4)	-52.0 (-58.3 to -45.3)	0.3 (-18.5 to 21.5)	-47.4 (-66.0 to -23.5)	-63.5 (-76.4 to -46.9)	-30.0 (-36.9 to -19.1)	-51.9 (-56.6 to -47.1)
Croatia	YLDs Rate: 1990	5,291.4 (4,011.7–6,760.1)	953.2 (667.7–1,302.3)	689.6 (469.7–969.5)	217.6 (144.6–300.7)	87.5 (61.7–120.0)	22.4 (15.9–29.7)	122.7 (84.3–167.3)	80.1 (50.0–117.9)	125.5 (83.9–175.5)	0.0 (0.0–0.0)	111.1 (84.7–141.6)
Croatia	YLDs Rate: 2015	5,033.4 (3,840.5–6,345.8)	939.3 (651.9–1,287.6)	695.1 (474.9–979.0)	223.1 (149.0–310.6)	81.6 (58.3–110.9)	15.1 (10.7–20.3)	180.5 (113.4–260.3)	57.1 (35.4–83.5)	65.5 (41.5–94.1)	0.0 (0.0–0.0)	78.2 (58.5–100.8)
Croatia	YLDs Rate: Cumulative change (%)	-4.9 (-7.3 to -2.1)	-1.4 (-9.7 to 7.8)	0.8 (-1.3 to 3.0)	2.8 (-8.7 to 14.6)	-6.6 (-14.3 to 0.3)	-32.3 (-41.2 to -22.7)	46.7 (19.1–77.5)	-26.6 (-52.5 to 6.7)	-46.5 (-65.2 to -22.4)	3.1 (-7.6 to 19.3)	-29.6 (-36.5 to -22.4)
Czech Republic	Prevalence Number: 1990	2,705,981 (2,674,141–2,734,300)	737,617 (689,514–787,116)	1,077,196 (1,042,225–1,110,385)	143,696 (120,338–169,671)	278,261 (235,292–333,892)	2,718 (2,561–2,876)	55,452 (47,606–65,437)	8,453 (6,901–10,082)	10,685 (7,663–14,241)	0 (0–0)	22,138 (19,368–25,605)
Czech Republic	Prevalence Number: 2015	1,742,483 (1,717,606–1,762,110)	510,964 (480,153–538,149)	661,786 (640,228–681,872)	70,218 (57,453–83,898)	156,641 (130,817–191,337)	1,936 (1,817–2,058)	35,981 (31,372–42,392)	8,326 (6,076–11,073)	5,043 (3,592–6,706)	0 (0–0)	14,262 (12,398–16,471)
Czech Republic	Prevalence Number: Cumulative change (%)	-35.6 (-36.6 to -34.6)	-30.6 (-36.3 to -24.4)	-38.5 (-41.1 to -36.0)	-51.1 (-55.6 to -46.5)	-43.1 (-56.2 to -28.0)	-28.7 (-31.3 to -26.1)	-34.5 (-47.5 to -20.1)	-0.2 (-30.8 to 40.0)	-51.5 (-66.8 to -30.6)	-26.5 (-30.3 to -22.3)	-35.2 (-45.9 to -23.5)
Czech Republic	Prevalence Rate: 1990	88,090.1 (86,987.1–89,091.6)	24,945.8 (23,009.3–26,785.5)	33,508.6 (32,453.6–34,529.3)	4,480.4 (3,727.5–5,299.0)	9,122.3 (7,715.4–10,934.5)	102.6 (96.4–108.8)	1,822.5 (1,568.8–2,150.6)	346.2 (282.6–412.9)	341.4 (245.0–453.3)	0.0 (0.0–0.0)	776.6 (683.6–890.2)
Czech Republic	Prevalence Rate: 2015	87,817.5 (86,576.8–88,801.3)	25,621.7 (24,114.5–26,929.6)	33,759.7 (32,647.2–34,764.4)	3,581.3 (2,928.6–4,277.0)	7,897.4 (6,596.0–9,645.5)	94.9 (89.1–100.8)	1,811.4 (1,579.8–2,134.6)	403.6 (294.6–536.7)	254.9 (181.4–338.8)	0.0 (0.0–0.0)	716.1 (622.4–827.4)
Czech Republic	Prevalence Rate: Cumulative change (%)	-0.3 (-1.9 to 1.4)	2.9 (-6.1 to 13.6)	0.8 (-3.5 to 4.9)	-20.0 (-27.3 to -12.7)	-12.5 (-32.6 to 10.6)	-7.5 (-10.8 to -4.0)	0.3 (-19.4 to 22.0)	18.1 (-18.1 to 65.7)	-23.4 (-47.6 to 10.0)	0.0 (-0.0 to 0.0)	-7.4 (-22.1 to 9.0)
Czech Republic	YLDs Number: 1990	163,658 (124,726–207,544)	26,599 (18,872–36,078)	22,188 (15,050–31,462)	6,542 (4,306–9,094)	2,676 (1,883–3,619)	447 (317–602)	4,348 (2,748–6,215)	1,064 (698–1,494)	3,863 (2,412–5,581)	0 (0–0)	2,770 (2,122–3,503)
Czech Republic	YLDs Number: 2015	95,623 (72,821–120,428)	18,582 (13,242–25,220)	14,032 (9,599–19,620)	3,197 (2,103–4,490)	1,527 (1,077–2,057)	319 (226–421)	3,612 (2,082–5,543)	1,046 (632–1,533)	1,109 (679–1,658)	0 (0–0)	1,292 (967–1,650)
Czech Republic	YLDs Number: Cumulative change (%)	-41.6 (-43.3 to -39.5)	-30.0 (-36.3 to -22.6)	-36.7 (-38.2 to -34.9)	-51.1 (-55.9 to -46.2)	-42.9 (-46.9 to -38.1)	-28.4 (-36.7 to -19.1)	-16.8 (-34.4 to 18.7)	-0.2 (-31.7 to 41.8)	-70.5 (-81.2 to -57.2)	-23.6 (-38.7 to -6.4)	-53.3 (-59.2 to -47.0)
Czech Republic	YLDs Rate: 1990	5,140.4 (3,910.1–6,512.4)	898.3 (633.8–1,219.8)	706.6 (481.0–992.4)	204.1 (135.0–283.6)	87.0 (61.4–117.9)	16.9 (12.0–22.8)	143.1 (90.2–204.0)	43.6 (28.6–61.2)	123.6 (77.8–178.6)	0.0 (0.0–0.0)	90.6 (69.7–114.6)
Czech Republic	YLDs Rate: 2015	4,871.9 (3,708.7–6,135.3)	934.8 (664.9–1,268.1)	711.1 (485.9–996.0)	163.1 (107.2–228.7)	77.7 (55.0–104.6)	15.6 (11.1–20.7)	181.8 (104.8–278.9)	50.7 (30.7–74.3)	56.0 (34.2–83.7)	0.0 (0.0–0.0)	65.1 (48.7–83.2)
Czech Republic	YLDs Rate: Cumulative change (%)	-5.2 (-8.0 to -1.9)	4.3 (-5.9 to 16.7)	0.6 (-1.5 to 2.7)	-20.0 (-27.7 to -12.4)	-10.6 (-16.5 to -2.1)	-7.1 (-17.7 to 5.3)	27.2 (0.5–81.3)	18.2 (-19.1 to 67.9)	-53.4 (-70.3 to -32.2)	5.3 (-16.8 to 30.0)	-28.0 (-37.1 to -18.5)
Hungary	Prevalence Number: 1990	2,583,650 (2,556,198–2,606,823)	643,022 (604,619–696,033)	1,042,912 (1,010,977–1,074,285)	141,154 (118,158–165,796)	266,658 (225,834–333,138)	2,351 (2,207–2,494)	64,349 (54,448–78,594)	10,844 (8,250–13,870)	10,893 (7,931–14,372)	0 (0–0)	26,538 (23,339–30,569)
Hungary	Prevalence Number: 2015	1,737,254 (1,718,327–1,754,069)	446,400 (409,595–490,590)	703,449 (681,497–724,389)	75,667 (62,939–89,811)	153,197 (128,407–187,999)	1,661 (1,530–1,811)	49,901 (42,749–60,401)	7,379 (5,432–9,866)	5,115 (3,667–6,807)	0 (0–0)	16,728 (14,807–19,092)
Hungary	Prevalence Number: Cumulative change (%)	-32.8 (-33.6 to -31.9)	-30.5 (-38.0 to -23.0)	-32.5 (-35.2 to -29.7)	-46.3 (-51.2 to -40.7)	-41.9 (-56.2 to -26.9)	-29.3 (-32.9 to -25.1)	-21.6 (-38.2 to -2.4)	-30.3 (-53.9 to -0.2)	-51.9 (-67.6 to -31.8)	-29.4 (-31.4 to -27.4)	-36.6 (-46.2 to -26.3)
Hungary	Prevalence Rate: 1990	88,386.8 (87,373.4–89,277.2)	22,951.5 (21,494.8–25,227.3)	34,274.9 (33,191.8–35,276.5)	4,675.6 (3,906.0–5,496.8)	9,190.9 (7,786.0–11,486.1)	92.8 (87.0–98.7)	2,222.1 (1,878.9–2,710.4)	455.6 (346.6–582.7)	365.9 (264.4–479.9)	0.0 (0.0–0.0)	963.7 (850.2–1,102.2)
Hungary	Prevalence Rate: 2015	88,175.4 (87,134.1–89,093.8)	23,653.3 (21,633.5–26,055.5)	34,634.1 (33,645.0–35,685.6)	3,839.1 (3,184.3–4,581.2)	7,855.4 (6,594.8–9,626.9)	91.4 (84.1–99.8)	2,549.9 (2,186.4–3,090.6)	418.7 (308.3–559.8)	259.4 (186.0–344.4)	0.0 (0.0–0.0)	885.0 (786.3–1,004.8)
Hungary	Prevalence Rate: Cumulative change (%)	-0.2 (-1.7 to 1.2)	3.2 (-9.1 to 14.3)	1.1 (-2.8 to 5.3)	-17.8 (-25.3 to -9.2)	-13.5 (-34.9 to 8.8)	-1.6 (-6.5 to 4.5)	15.9 (-8.5 to 44.2)	-5.9 (-37.7 to 34.8)	-27.4 (-51.2 to 3.1)	0.0 (-0.0 to 0.0)	-7.7 (-21.1 to 6.5)
Hungary	YLDs Number: 1990	160,167 (122,229–202,514)	22,378 (15,783–30,766)	23,500 (16,085–32,531)	6,424 (4,296–8,945)	2,734 (1,942–3,671)	388 (273–517)	5,714 (3,550–8,326)	1,360 (865–1,961)	3,938 (2,449–5,590)	0 (0–0)	3,728 (2,876–4,669)
Hungary	YLDs Number: 2015	101,218 (77,651–127,496)	15,585 (10,887–21,552)	15,891 (10,950–22,039)	3,445 (2,254–4,759)	1,534 (1,064–2,083)	274 (193–371)	4,417 (2,298–7,580)	927 (568–1,383)	1,139 (713–1,669)	0 (0–0)	1,803 (1,387–2,305)
Hungary	YLDs Number: Cumulative change (%)	-36.8 (-38.9 to -34.3)	-30.2 (-39.7 to -22.0)	-32.4 (-33.7 to -30.9)	-46.3 (-51.9 to -40.8)	-43.9 (-49.9 to -38.8)	-29.3 (-37.6 to -20.9)	-23.6 (-40.8 to 14.7)	-30.3 (-53.9 to 1.0)	-70.2 (-80.9 to -56.3)	-27.1 (-41.3 to -9.5)	-51.6 (-56.3 to -46.8)
Hungary	YLDs Rate: 1990	5,322.6 (4,055.2–6,742.9)	796.8 (559.0–1,102.0)	792.3 (545.9–1,091.3)	212.9 (142.7–297.2)	93.4 (66.5–125.7)	15.4 (10.8–20.6)	197.5 (122.7–286.7)	57.2 (36.4–82.4)	132.4 (82.2–188.6)	0.0 (0.0–0.0)	128.3 (99.2–160.4)
Hungary	YLDs Rate: 2015	5,044.4 (3,868.0–6,367.9)	840.0 (586.5–1,160.9)	797.9 (550.3–1,101.0)	174.9 (114.3–242.7)	82.3 (57.1–111.6)	15.1 (10.7–20.4)	225.8 (117.2–388.7)	52.6 (32.3–78.5)	57.8 (36.2–84.8)	0.0 (0.0–0.0)	92.0 (70.5–117.0)
Hungary	YLDs Rate: Cumulative change (%)	-5.2 (-8.9 to -1.2)	5.8 (-10.6 to 18.1)	0.7 (-1.1 to 2.7)	-17.8 (-26.0 to -9.0)	-11.8 (-22.3 to -4.0)	-1.5 (-12.5 to 10.4)	13.0 (-12.6 to 69.2)	-5.8 (-37.8 to 36.4)	-55.1 (-71.1 to -34.0)	4.7 (-16.4 to 29.8)	-28.2 (-35.1 to -21.3)
Macedonia	Prevalence Number: 1990	616,664 (608,214–623,957)	151,566 (140,399–161,324)	228,677 (221,795–235,625)	33,431 (27,483–39,589)	66,372 (56,125–80,901)	3,693 (3,135–4,306)	11,976 (10,165–14,224)	2,314 (1,698–3,048)	2,598 (1,911–3,422)	0 (0–0)	7,054 (6,172–8,304)
Macedonia	Prevalence Number: 2015	430,035 (424,398–435,085)	96,358 (89,837–104,008)	168,257 (163,335–173,466)	23,414 (19,088–28,097)	38,996 (32,871–46,655)	928 (850–1,015)	8,933 (7,738–10,493)	1,352 (1,004–1,754)	1,319 (953–1,779)	0 (0–0)	4,300 (3,705–5,002)
Macedonia	Prevalence Number: Cumulative change (%)	-30.3 (-31.3 to -29.1)	-36.4 (-40.6 to -31.6)	-26.4 (-29.4 to -23.3)	-29.9 (-37.0 to -22.4)	-40.6 (-54.3 to -24.7)	-74.7 (-78.2 to -70.9)	-24.7 (-40.6 to -8.5)	-39.7 (-60.8 to -11.3)	-48.0 (-65.1 to -26.7)	-34.1 (-35.6 to -32.6)	-38.7 (-48.3 to -28.3)
Macedonia	Prevalence Rate: 1990	88,399.2 (87,182.1–89,435.9)	21,897.9 (20,281.0–23,320.2)	32,685.7 (31,702.0–33,679.7)	4,771.8 (3,922.1–5,652.8)	9,530.1 (8,059.0–11,616.5)	543.0 (461.5–632.9)	1,719.6 (1,459.7–2,042.2)	339.8 (249.5–447.6)	372.0 (273.6–489.9)	0.0 (0.0–0.0)	1,030.0 (901.6–1,208.7)
Macedonia	Prevalence Rate: 2015	87,617.2 (86,409.0–88,692.6)	20,504.0 (19,083.5–22,194.2)	33,039.6 (32,063.2–34,095.2)	4,727.1 (3,842.5–5,679.3)	7,998.7 (6,747.0–9,568.2)	208.6 (190.3–228.3)	1,830.9 (1,586.1–2,150.3)	310.5 (230.5–402.8)	265.7 (191.6–356.9)	0.0 (0.0–0.0)	914.2 (792.1–1,057.8)
Macedonia	Prevalence Rate: Cumulative change (%)	-0.9 (-2.5 to 0.8)	-6.3 (-12.5 to 1.0)	1.1 (-2.9 to 5.4)	-0.9 (-11.2 to 10.1)	-15.1 (-34.6 to 7.6)	-61.3 (-66.6 to -55.7)	7.5 (-15.2 to 30.7)	-5.7 (-38.7 to 38.7)	-26.9 (-50.8 to 3.4)	0.0 (-0.0 to 0.0)	-10.8 (-24.6 to 3.7)
Macedonia	YLDs Number: 1990	36,141 (27,560–45,976)	4,985 (3,471–6,870)	4,729 (3,205–6,623)	1,521 (997–2,102)	811 (591–1,083)	608 (428–828)	796 (530–1,107)	289 (180–430)	936 (587–1,330)	0 (0–0)	1,004 (786–1,256)

© 2017 Global Burden of Disease Child and Adolescent Health Collaboration. JAMA Pediatrics.

eTable 3. Prevalent Cases, Rates (per 100,000 population), Years Lived with Disability (YLDs), and Cumulative Percent Change with 95% Uncertainty Interval (UI) for the Top 10 Global Causes of YLDs in Children and Adolescents in 195 Countries and Territories, Aged 0 to 19 Years, Both Sexes, 1990 and 2015. *Note: best viewed by enlarging in browser.*

Location	Measure	All causes	Iron-deficiency anemia	Skin and subcutaneous diseases	Asthma	Hemoglobinopathies and hemolytic anemias	Diarrheal diseases	Congenital anomalies	Protein-energy malnutrition	Epilepsy	Malaria	Neonatal preterm birth complications
Macedonia	YLDs Number: 2015	23,663 (17,912–29,995)	3,209 (2,244–4,411)	3,407 (2,296–4,832)	1,067 (700–1,488)	471 (331–648)	153 (107–203)	656 (423–947)	170 (102–255)	305 (190–448)	0 (0–0)	458 (349–585)
Macedonia	YLDs Number: Cumulative change (%)	-34.5 (-36.0 to -33.1)	-35.6 (-40.4 to -29.4)	-28.0 (-29.6 to -26.5)	-29.7 (-37.2 to -21.7)	-41.9 (-46.4 to -35.5)	-74.7 (-78.3 to -70.6)	-17.6 (-28.5 to -4.6)	-39.4 (-60.6 to -10.5)	-66.6 (-78.7 to -50.5)	-34.3 (-42.4 to -24.9)	-54.3 (-58.9 to -49.4)
Macedonia	YLDs Rate: 1990	5,175.4 (3,944.5–6,583.2)	721.0 (502.7–993.7)	676.6 (458.5–947.5)	217.2 (142.5–300.3)	116.5 (84.9–155.4)	89.5 (63.2–121.8)	114.4 (76.2–159.0)	42.6 (26.5–63.1)	134.0 (84.3–190.4)	0.0 (0.0–0.0)	144.1 (112.8–180.4)
Macedonia	YLDs Rate: 2015	4,699.5 (3,555.9–5,959.3)	688.9 (481.3–950.9)	681.7 (461.9–960.1)	215.7 (141.1–300.7)	98.1 (69.1–134.9)	34.4 (24.3–45.9)	134.7 (86.6–194.3)	39.1 (23.6–58.6)	61.5 (38.4–90.3)	0.0 (0.0–0.0)	94.0 (71.6–119.9)
Macedonia	YLDs Rate: Cumulative change (%)	-9.2 (-11.4 to -7.0)	-4.4 (-11.6 to 5.2)	0.8 (-1.2 to 2.7)	-0.6 (-11.3 to 11.2)	-15.9 (-22.4 to -6.4)	-61.3 (-66.9 to -55.2)	17.7 (2.1–36.1)	-5.2 (-38.3 to 39.9)	-52.9 (-69.8 to -30.7)	0.2 (-12.1 to 14.5)	-34.8 (-41.3 to -27.8)
Montenegro	Prevalence Number: 1990	185,113 (182,720–187,548)	52,361 (48,762–56,719)	69,962 (67,913–72,073)	8,096 (6,579–9,764)	19,824 (16,775–24,339)	358 (325–393)	3,857 (3,351–4,511)	1,279 (955–1,657)	572 (421–754)	0 (0–0)	2,191 (1,924–2,515)
Montenegro	Prevalence Number: 2015	141,350 (139,516–143,057)	39,456 (37,750–41,376)	54,847 (53,029–56,626)	6,613 (5,429–7,923)	12,966 (10,948–15,636)	164 (151–176)	2,932 (2,515–3,417)	490 (380–625)	393 (284–517)	0 (0–0)	1,452 (1,256–1,688)
Montenegro	Prevalence Number: Cumulative change (%)	-23.6 (-25.0 to -22.4)	-24.5 (-31.3 to -18.9)	-21.6 (-24.9 to -18.3)	-18.2 (-24.7 to -10.8)	-33.9 (-49.2 to -15.5)	-54.2 (-57.0 to -51.6)	-23.3 (-39.4 to -6.6)	-60.5 (-73.2 to -44.9)	-29.7 (-52.3 to -1.4)	-25.7 (-26.6 to -24.9)	-33.4 (-43.6 to -22.3)
Montenegro	Prevalence Rate: 1990	88,342.5 (87,169.2–89,543.0)	25,246.8 (23,460.6–27,436.3)	33,075.8 (32,112.5–34,073.8)	3,826.2 (3,105.7–4,617.0)	9,489.2 (8,032.3–11,653.4)	180.2 (163.5–197.4)	1,847.0 (1,604.4–2,159.2)	652.9 (487.4–845.6)	273.1 (200.8–360.7)	0.0 (0.0–0.0)	1,077.2 (946.7–1,232.7)
Montenegro	Prevalence Rate: 2015	88,076.0 (86,896.7–89,194.6)	25,152.5 (24,003.6–26,413.6)	33,329.0 (32,253.1–34,393.1)	4,056.9 (3,336.9–4,859.9)	8,125.3 (6,858.4–9,791.5)	113.0 (103.7–121.9)	1,838.1 (1,575.1–2,139.6)	350.1 (271.2–446.2)	244.0 (176.0–322.7)	0.0 (0.0–0.0)	959.5 (835.4–1,111.3)
Montenegro	Prevalence Rate: Cumulative change (%)	-0.3 (-2.0 to 1.4)	-0.1 (-9.6 to 7.4)	0.8 (-3.4 to 5.0)	6.2 (-2.4 to 15.9)	-13.4 (-33.5 to 10.5)	-37.2 (-41.2 to -33.5)	0.4 (-20.8 to 22.2)	-44.8 (-62.5 to -23.1)	-8.5 (-37.8 to 27.9)	0.0 (-0.0 to 0.0)	-10.6 (-23.6 to 3.9)
Montenegro	YLDs Number: 1990	11,037 (8,368–13,994)	1,900 (1,343–2,602)	1,475 (1,013–2,087)	368 (243–518)	184 (129–252)	59 (42–79)	274 (168–437)	160 (98–231)	206 (132–293)	0 (0–0)	279 (218–355)
Montenegro	YLDs Number: 2015	8,025 (6,110–10,156)	1,427 (1,003–1,937)	1,145 (782–1,618)	301 (198–418)	126 (89–170)	27 (19–35)	255 (135–445)	61 (39–88)	90 (56–134)	0 (0–0)	144 (110–183)
Montenegro	YLDs Number: Cumulative change (%)	-27.3 (-29.7 to -24.9)	-24.7 (-33.9 to -18.2)	-22.4 (-24.1 to -20.8)	-18.1 (-24.7 to -9.9)	-31.4 (-37.3 to -24.8)	-54.2 (-59.6 to -48.3)	-4.5 (-43.9 to 59.3)	-60.5 (-73.2 to -45.0)	-54.8 (-70.8 to -33.6)	-22.6 (-33.6 to -7.6)	-48.1 (-53.1 to -42.9)
Montenegro	YLDs Rate: 1990	5,234.6 (3,964.1–6,635.8)	915.5 (646.3–1,254.3)	700.8 (480.7–990.5)	174.1 (115.0–244.9)	88.4 (61.9–121.2)	29.7 (21.1–40.0)	131.7 (80.6–209.3)	82.0 (50.4–118.0)	98.3 (63.0–140.2)	0.0 (0.0–0.0)	133.8 (104.6–169.9)
Montenegro	YLDs Rate: 2015	4,909.5 (3,736.3–6,212.8)	910.9 (638.5–1,232.1)	704.9 (481.2–992.7)	184.8 (122.1–256.9)	79.5 (56.2–107.6)	18.6 (13.2–24.5)	160.0 (85.0–279.7)	44.0 (27.9–63.0)	56.4 (34.5–83.4)	0.0 (0.0–0.0)	90.6 (69.1–114.7)
Montenegro	YLDs Rate: Cumulative change (%)	-6.2 (-9.5 to -2.9)	-0.3 (-13.0 to 8.3)	0.6 (-1.4 to 2.5)	6.3 (-2.3 to 17.1)	-9.8 (-17.7 to -1.1)	-37.2 (-44.6 to -29.1)	25.0 (-26.5 to 108.3)	-44.9 (-62.6 to -23.1)	-41.1 (-61.9 to -14.1)	4.2 (-10.8 to 24.8)	-32.2 (-38.6 to -25.3)
Poland	Prevalence Number: 1990	11,173,226 (11,046,831–11,288,819)	2,803,731 (2,457,834–3,159,315)	4,123,913 (3,994,502–4,253,872)	831,514 (683,170–967,414)	1,128,792 (951,706–1,357,624)	16,396 (15,180–17,699)	256,922 (222,004–303,080)	48,577 (35,295–63,290)	28,044 (20,719–36,882)	0 (0–0)	122,720 (107,807–141,155)
Poland	Prevalence Number: 2015	6,515,554 (6,427,661–6,599,801)	1,499,704 (1,347,535–1,659,334)	2,548,698 (2,472,641–2,630,941)	367,315 (300,526–441,813)	563,211 (469,945–711,979)	6,578 (6,194–6,964)	130,724 (114,595–152,304)	26,248 (18,729–34,830)	16,202 (11,929–20,998)	0 (0–0)	55,249 (48,919–63,178)
Poland	Prevalence Number: Cumulative change (%)	-41.7 (-42.6 to -40.8)	-46.3 (-53.7 to -40.2)	-38.2 (-40.8 to -35.6)	-55.7 (-62.3 to -49.3)	-49.6 (-61.7 to -36.3)	-59.8 (-62.4 to -57.3)	-48.7 (-58.3 to -37.4)	-44.3 (-64.4 to -17.2)	-40.9 (-59.4 to -17.3)	-42.8 (-44.5 to -41.0)	-54.8 (-61.6 to -47.5)
Poland	Prevalence Rate: 1990	88,876.6 (87,858.4–89,806.1)	22,442.2 (19,553.4–25,436.3)	32,871.4 (31,857.3–33,917.2)	6,485.0 (5,326.7–7,537.0)	9,002.3 (7,588.4–10,825.2)	138.3 (127.9–149.5)	2,051.2 (1,772.2–2,419.7)	420.5 (305.5–547.8)	223.2 (164.8–293.1)	0.0 (0.0–0.0)	1,019.1 (898.4–1,166.3)
Poland	Prevalence Rate: 2015	88,121.1 (86,918.9–89,244.6)	21,216.3 (19,254.6–23,266.5)	33,332.7 (32,348.1–34,404.9)	5,096.8 (4,136.2–6,170.4)	7,664.2 (6,403.0–9,664.4)	89.8 (84.6–95.0)	1,769.7 (1,551.9–2,059.7)	347.3 (247.8–460.9)	218.6 (160.2–283.6)	0.0 (0.0–0.0)	752.3 (666.4–859.8)
Poland	Prevalence Rate: Cumulative change (%)	-0.8 (-2.4 to 0.7)	-5.0 (-17.6 to 5.3)	1.4 (-2.8 to 5.7)	-21.3 (-33.1 to -9.9)	-14.0 (-34.5 to 8.7)	-35.0 (-39.2 to -30.9)	-13.0 (-29.3 to 5.9)	-14.8 (-45.5 to 26.6)	0.2 (-31.0 to 40.2)	0.0 (-0.0 to 0.0)	-25.8 (-36.6 to -14.3)
Poland	YLDs Number: 1990	650,728 (494,327–826,712)	90,185 (60,653–128,253)	85,490 (58,542–120,325)	37,815 (25,438–52,383)	13,386 (9,719–18,030)	2,703 (1,918–3,575)	16,808 (11,785–22,666)	6,086 (3,832–9,098)	10,086 (6,483–14,184)	0 (0–0)	15,536 (12,004–19,972)
Poland	YLDs Number: 2015	355,768 (271,518–448,588)	50,065 (34,021–69,123)	51,948 (35,343–73,150)	16,737 (11,061–23,400)	6,257 (4,393–8,513)	1,085 (774–1,471)	14,556 (9,264–20,904)	3,309 (2,021–4,982)	3,595 (2,236–5,218)	0 (0–0)	5,214 (3,985–6,764)
Poland	YLDs Number: Cumulative change (%)	-45.3 (-47.5 to -43.8)	-44.1 (-55.6 to -38.9)	-39.3 (-40.7 to -38.0)	-55.6 (-62.2 to -49.1)	-53.2 (-59.4 to -47.2)	-59.7 (-64.5 to -54.5)	-13.5 (-33.8 to 6.4)	-44.0 (-64.4 to -17.2)	-63.4 (-76.3 to -45.2)	-44.7 (-54.9 to -31.0)	-66.4 (-70.1 to -62.2)
Poland	YLDs Rate: 1990	5,167.9 (3,924.8–6,566.5)	714.1 (476.3–1,021.8)	680.6 (465.6–957.4)	294.9 (198.0–408.3)	104.1 (75.5–140.3)	22.8 (16.2–30.1)	134.2 (93.9–181.1)	52.7 (33.2–78.8)	80.3 (51.6–112.8)	0.0 (0.0–0.0)	123.8 (95.6–159.4)
Poland	YLDs Rate: 2015	4,747.3 (3,618.1–5,996.6)	730.4 (499.2–1,006.3)	688.9 (470.4–964.3)	232.3 (152.7–326.1)	91.6 (64.6–124.1)	14.8 (10.6–20.0)	197.1 (125.3–284.1)	43.8 (26.7–65.9)	48.5 (30.0–70.2)	0.0 (0.0–0.0)	70.8 (54.3–92.1)
Poland	YLDs Rate: Cumulative change (%)	-8.1 (-11.9 to -5.5)	3.1 (-18.0 to 14.2)	1.2 (-0.8 to 3.1)	-21.1 (-33.0 to -9.5)	-11.9 (-23.7 to 0.1)	-34.9 (-42.5 to -26.7)	46.7 (12.7–79.8)	-14.5 (-45.6 to 26.8)	-38.0 (-59.4 to -7.5)	2.3 (-15.6 to 26.4)	-42.7 (-49.1 to -35.6)
Romania	Prevalence Number: 1990	6,793,392 (6,727,716–6,849,215)	2,078,449 (1,897,574–2,367,035)	2,544,914 (2,468,959–2,630,875)	327,081 (264,995–393,471)	553,731 (521,056–585,332)	15,083 (13,932–16,516)	127,159 (107,516–151,544)	25,637 (21,033–30,919)	29,136 (21,130–38,096)	0 (0–0)	71,175 (61,542–84,748)
Romania	Prevalence Number: 2015	3,678,957 (3,639,265–3,714,576)	1,118,463 (1,054,922–1,197,913)	1,395,928 (1,354,218–1,437,730)	199,000 (162,467–242,153)	266,737 (253,185–283,405)	4,933 (4,567–5,312)	71,615 (62,450–83,304)	13,224 (9,532–17,476)	11,440 (8,192–15,149)	0 (0–0)	34,459 (29,323–40,750)
Romania	Prevalence Number: Cumulative change (%)	-45.8 (-46.6 to -45.2)	-45.9 (-53.9 to -38.1)	-45.1 (-47.5 to -42.9)	-38.9 (-45.7 to -27.4)	-51.8 (-55.1 to -48.0)	-67.3 (-69.2 to -65.6)	-43.1 (-54.4 to -30.9)	-47.7 (-64.5 to -29.2)	-59.6 (-73.9 to -41.3)	-44.2 (-44.7 to -43.6)	-51.3 (-60.1 to -41.9)
Romania	Prevalence Rate: 1990	90,297.9 (89,370.7–91,124.1)	28,625.0 (25,895.2–32,833.2)	32,791.5 (31,783.4–33,912.8)	4,269.9 (3,448.1–5,139.9)	7,405.2 (6,969.2–7,829.5)	226.6 (209.2–248.8)	1,702.8 (1,440.1–2,029.3)	395.0 (324.2–476.5)	380.3 (274.7–496.1)	0.0 (0.0–0.0)	1,016.7 (883.6–1,196.9)
Romania	Prevalence Rate: 2015	89,316.5 (88,311.1–90,205.5)	28,063.2 (26,361.8–30,236.6)	33,211.0 (32,199.5–34,203.3)	4,727.5 (3,861.0–5,746.5)	6,522.5 (6,193.0–6,927.8)	132.7 (122.5–143.3)	1,749.1 (1,525.4–2,034.0)	371.7 (267.9–491.2)	275.0 (197.9–362.4)	0.0 (0.0–0.0)	898.2 (768.8–1,051.7)
Romania	Prevalence Rate: Cumulative change (%)	-1.1 (-2.5 to 0.3)	-1.4 (-17.0 to 14.7)	1.3 (-3.0 to 5.3)	11.2 (-1.4 to 31.6)	-11.8 (-17.9 to -4.8)	-41.4 (-45.0 to -38.3)	3.7 (-16.8 to 26.1)	-4.7 (-35.3 to 29.1)	-25.6 (-51.7 to 8.0)	0.0 (-0.0 to 0.0)	-11.1 (-26.2 to 4.9)
Romania	YLDs Number: 1990	429,821 (326,410–545,319)	76,892 (53,065–108,203)	52,361 (35,452–73,470)	14,865 (9,590–20,665)	4,921 (3,422–6,898)	2,477 (1,778–3,329)	9,009 (6,005–12,450)	3,210 (2,098–4,578)	10,415 (6,520–15,223)	0 (0–0)	8,906 (6,903–11,298)
Romania	YLDs Number: 2015	217,094 (166,087–276,084)	41,350 (29,103–55,807)	28,787 (19,646–40,623)	9,059 (5,973–12,610)	2,553 (1,822–3,450)	810 (578–1,068)	6,353 (4,040–9,207)	1,656 (993–2,450)	2,618 (1,565–3,825)	0 (0–0)	3,328 (2,557–4,248)
Romania	YLDs Number: Cumulative change (%)	-49.5 (-52.0 to -47.3)	-45.6 (-55.6 to -34.3)	-45.0 (-46.0 to -44.0)	-38.8 (-45.9 to -26.9)	-47.8 (-55.3 to -40.5)	-67.3 (-70.7 to -63.6)	-29.5 (-40.8 to -16.7)	-47.7 (-64.3 to -28.4)	-74.1 (-83.9 to -60.9)	-41.7 (-53.5 to -26.6)	-62.6 (-66.4 to -58.6)
Romania	YLDs Rate: 1990	5,620.0 (4,256.8–7,156.3)	1,063.7 (732.4–1,505.0)	684.9 (465.8–957.3)	194.1 (125.5–270.0)	66.3 (45.9–93.0)	37.2 (26.7–50.0)	120.8 (80.7–166.6)	49.5 (32.3–70.5)	136.0 (84.8–198.3)	0.0 (0.0–0.0)	119.2 (92.1–151.0)
Romania	YLDs Rate: 2015	5,204.5 (3,979.3–6,603.2)	1,035.1 (727.2–1,402.7)	691.9 (472.8–974.1)	215.3 (141.7–300.3)	62.8 (44.6–84.8)	21.8 (15.5–28.7)	155.3 (98.7–224.8)	46.6 (27.9–68.9)	63.0 (37.6–91.7)	0.0 (0.0–0.0)	81.1 (62.3–103.3)
Romania	YLDs Rate: Cumulative change (%)	-7.3 (-12.4 to -2.7)	-1.4 (-21.3 to 22.5)	1.0 (-0.9 to 2.9)	11.4 (-1.4 to 32.4)	-4.5 (-19.6 to 10.4)	-41.4 (-47.6 to -34.9)	28.5 (7.9–52.1)	-4.6 (-34.9 to 30.5)	-52.3 (-70.5 to -28.3)	3.3 (-18.2 to 30.5)	-31.9 (-38.8 to -24.7)
Serbia	Prevalence Number: 1990	2,652,449 (2,622,030–2,679,771)	655,998 (610,532–699,990)	1,002,172 (968,378–1,035,889)	214,276 (177,669–253,283)	279,569 (236,357–335,288)	4,823 (4,437–5,225)	53,278 (45,950–63,681)	17,345 (12,694–22,849)	14,939 (10,978–19,036)	0 (0–0)	30,110 (26,252–34,781)
Serbia	Prevalence Number: 2015	1,776,120 (1,755,715–1,793,971)	451,178 (422,795–487,260)	697,252 (675,823–718,678)	104,850 (87,041–125,233)	161,736 (138,108–200,448)	2,055 (1,902–2,228)	36,726 (31,413–43,391)	7,599 (6,072–9,427)	7,649 (5,637–9,881)	0 (0–0)	17,646 (15,117–20,478)
Serbia	Prevalence Number: Cumulative change (%)	-33.0 (-34.0 to -32.1)	-31.1 (-37.0 to -24.1)	-30.4 (-33.4 to -27.5)	-51.0 (-55.1 to -46.6)	-41.5 (-54.5 to -24.9)	-57.4 (-59.9 to -55.1)	-30.5 (-44.4 to -14.9)	-54.7 (-69.2 to -35.4)	-47.5 (-64.6 to -26.7)	-35.8 (-36.8 to -34.8)	-41.1 (-50.6 to -31.6)
Serbia	Prevalence Rate: 1990	88,456.7 (87,435.8–89,380.0)	22,006.9 (20,413.3–23,507.1)	33,071.3 (31,958.9–34,201.8)	7,045.7 (5,845.0–8,323.0)	9,348.1 (7,905.2–11,215.0)	171.3 (157.1–185.7)	1,784.7 (1,538.4–2,132.4)	630.8 (461.7–831.0)	500.8 (367.4–638.7)	0.0 (0.0–0.0)	1,042.7 (912.5–1,201.4)
Serbia	Prevalence Rate: 2015	88,070.4 (87,026.7–88,985.8)	22,974.0 (21,414.3–25,157.0)	33,361.9 (32,294.1–34,377.4)	5,097.3 (4,225.2–6,114.0)	8,062.0 (6,878.6–9,997.6)	113.6 (104.8–123.6)	1,834.1 (1,569.4–2,163.4)	439.5 (351.2–545.2)	380.5 (281.9–491.6)	0.0 (0.0–0.0)	929.1 (802.1–1,069.9)
Serbia	Prevalence Rate: Cumulative change (%)	-0.4 (-2.0 to 1.0)	4.6 (-4.9 to 16.6)	0.9 (-3.4 to 5.3)	-27.6 (-33.4 to -21.0)	-12.8 (-32.1 to 11.9)	-33.6 (-37.7 to -29.9)	3.6 (-17.2 to 27.1)	-28.0 (-51.0 to 2.7)	-22.1 (-47.5 to 8.4)	0.0 (-0.0 to 0.0)	-10.5 (-24.2 to 3.3)
Serbia	YLDs Number: 1990	156,925 (118,587–198,854)	23,026 (16,171–31,420)	21,394 (14,683–30,025)	9,755 (6,524–13,497)	2,939 (2,079–3,951)	795 (564–1,058)	3,495 (2,263–4,995)	2,182 (1,328–3,210)	5,384 (3,413–7,596)	0 (0–0)	3,992 (3,129–5,071)
Serbia	YLDs Number: 2015	100,516 (76,719–127,960)	15,947 (11,449–21,843)	14,646 (9,987–20,671)	4,773 (3,162–6,627)	1,831 (1,309–2,455)	339 (237–458)	2,812 (1,785–4,051)	957 (611–1,364)	1,771 (1,085–2,526)	0 (0–0)	1,843 (1,404–2,355)
Serbia	YLDs Number: Cumulative change (%)	-35.9 (-37.7 to -34.1)	-30.6 (-37.5 to -23.3)	-31.6 (-33.1 to -30.1)	-51.0 (-55.3 to -46.2)	-37.6 (-42.6 to -31.2)	-57.3 (-61.8 to -52.2)	-19.1 (-32.4 to -2.8)	-54.6 (-69.3 to -35.2)	-66.2 (-78.4 to -50.3)	-33.8 (-44.5 to -19.2)	-53.8 (-58.3 to -48.9)

© 2017 Global Burden of Disease Child and Adolescent Health Collaboration. JAMA Pediatrics.

eTable 3. Prevalent Cases, Rates (per 100,000 population), Years Lived with Disability (YLDs), and Cumulative Percent Change with 95% Uncertainty Interval (UI) for the Top 10 Global Causes of YLDs in Children and Adolescents in 195 Countries and Territories, Aged 0 to 19 Years, Both Sexes, 1990 and 2015. *Note: best viewed by enlarging in browser.*

Location	Measure	All causes	Iron-deficiency anemia	Skin and subcutaneous diseases	Asthma	Hemoglobinopathies and hemolytic anemias	Diarrheal diseases	Congenital anomalies	Protein-energy malnutrition	Epilepsy	Malaria	Neonatal preterm birth complications
Serbia	YLDs Rate: 1990	5,180.8 (3,920.9–6,561.5)	767.8 (539.7–1,046.5)	710.1 (487.7–995.4)	320.8 (214.1–442.9)	97.0 (68.6–130.5)	28.3 (20.1–37.5)	117.1 (75.8–167.4)	79.4 (48.3–116.8)	180.6 (114.1–254.2)	0.0 (0.0–0.0)	133.6 (104.9–169.6)
Serbia	YLDs Rate: 2015	4,841.5 (3,697.9–6,152.6)	817.3 (585.9–1,115.6)	714.5 (490.2–1,003.6)	232.1 (154.0–322.3)	91.4 (65.3–123.0)	18.8 (13.2–25.3)	140.6 (89.3–202.0)	55.4 (35.4–78.9)	88.2 (54.1–125.4)	0.0 (0.0–0.0)	91.9 (70.0–117.2)
Serbia	YLDs Rate: Cumulative change (%)	-6.6 (-9.2 to -3.6)	6.7 (-4.2 to 19.6)	0.6 (-1.3 to 2.6)	-27.5 (-33.8 to -20.4)	-5.6 (-13.4 to 4.9)	-33.5 (-40.6 to -25.6)	20.7 (1.0–45.0)	-27.9 (-51.2 to 3.1)	-49.8 (-67.8 to -26.8)	3.9 (-13.8 to 29.1)	-31.1 (-37.9 to -24.1)
Slovakia	Prevalence Number: 1990	1,573,120 (1,553,010–1,591,495)	456,723 (411,752–505,886)	600,125 (581,022–618,241)	76,683 (64,128–90,679)	164,482 (138,904–205,725)	2,191 (2,040–2,358)	31,827 (27,300–37,781)	7,963 (5,852–10,548)	5,289 (3,802–7,047)	0 (0–0)	14,856 (13,013–17,041)
Slovakia	Prevalence Number: 2015	954,266 (940,633–966,763)	262,755 (244,235–278,479)	379,309 (366,144–391,356)	42,920 (34,985–50,986)	85,334 (71,381–103,263)	1,036 (969–1,124)	19,215 (16,600–22,583)	4,221 (3,109–5,548)	3,224 (2,328–4,267)	0 (0–0)	8,253 (7,237–9,527)
Slovakia	Prevalence Number: Cumulative change (%)	-39.3 (-40.4 to -38.3)	-42.2 (-49.7 to -36.1)	-36.8 (-39.6 to -34.1)	-43.9 (-50.5 to -36.6)	-47.5 (-59.6 to -33.4)	-52.7 (-55.4 to -50.0)	-39.2 (-51.1 to -26.9)	-45.4 (-64.3 to -20.6)	-37.5 (-56.2 to -12.3)	-39.5 (-40.9 to -37.9)	-44.2 (-52.5 to -35.6)
Slovakia	Prevalence Rate: 1990	88,502.7 (87,319.1–89,580.3)	26,053.8 (23,265.8–29,192.9)	33,266.0 (32,208.9–34,241.6)	4,223.1 (3,535.3–4,987.1)	9,285.5 (7,834.0–11,621.2)	134.3 (124.9–144.8)	1,799.5 (1,543.3–2,133.8)	504.8 (371.0–668.7)	297.0 (213.5–394.6)	0.0 (0.0–0.0)	878.5 (773.6–1,001.5)
Slovakia	Prevalence Rate: 2015	87,989.6 (86,709.6–89,127.4)	25,129.3 (23,573.5–26,514.0)	33,696.6 (32,521.9–34,768.6)	4,049.5 (3,283.8–4,860.5)	7,916.1 (6,635.6–9,576.3)	97.9 (91.6–106.1)	1,775.6 (1,534.7–2,087.5)	392.2 (288.9–515.5)	295.8 (214.3–390.8)	0.0 (0.0–0.0)	767.6 (673.5–885.8)
Slovakia	Prevalence Rate: Cumulative change (%)	-0.6 (-2.3 to 1.2)	-3.1 (-15.8 to 7.8)	1.3 (-3.1 to 5.6)	-4.0 (-15.9 to 8.8)	-13.8 (-33.8 to 9.3)	-27.1 (-31.2 to -23.0)	-0.6 (-20.3 to 19.5)	-20.0 (-47.7 to 16.3)	2.0 (-28.4 to 44.0)	-0.0 (-0.0 to 0.0)	-12.3 (-24.9 to 0.8)
Slovakia	YLDs Number: 1990	93,123 (71,001–118,534)	16,804 (11,752–23,212)	12,595 (8,597–17,860)	3,489 (2,297–4,868)	1,597 (1,139–2,196)	360 (261–481)	2,331 (1,513–3,301)	1,000 (619–1,501)	1,915 (1,176–2,766)	0 (0–0)	1,843 (1,421–2,365)
Slovakia	YLDs Number: 2015	54,181 (41,160–68,715)	9,193 (6,511–12,654)	7,843 (5,324–11,119)	1,955 (1,278–2,695)	786 (560–1,069)	171 (121–229)	1,839 (1,134–2,700)	532 (320–802)	711 (441–1,031)	0 (0–0)	817 (617–1,049)
Slovakia	YLDs Number: Cumulative change (%)	-41.8 (-43.8 to -40.0)	-45.0 (-53.4 to -38.1)	-37.7 (-39.1 to -36.4)	-43.9 (-50.5 to -36.3)	-50.6 (-56.7 to -45.6)	-52.4 (-58.0 to -45.9)	-21.1 (-33.6 to -5.3)	-45.3 (-64.2 to -19.8)	-61.8 (-75.1 to -44.2)	-39.6 (-51.8 to -21.1)	-55.6 (-59.9 to -51.3)
Slovakia	YLDs Rate: 1990	5,169.4 (3,936.9–6,581.8)	950.5 (663.5–1,318.0)	703.3 (480.8–994.4)	192.2 (126.3–268.3)	88.7 (63.2–121.8)	22.1 (16.0–29.6)	131.9 (85.5–187.2)	63.4 (39.2–95.1)	107.6 (66.2–155.1)	0.0 (0.0–0.0)	103.9 (80.1–133.2)
Slovakia	YLDs Rate: 2015	4,911.5 (3,737.6–6,245.6)	910.9 (647.0–1,247.3)	709.7 (482.5–1,000.6)	184.5 (120.4–255.2)	78.1 (55.9–105.6)	16.2 (11.5–21.7)	170.0 (105.1–249.7)	49.5 (29.8–74.6)	65.3 (40.4–95.1)	0.0 (0.0–0.0)	75.8 (57.4–97.4)
Slovakia	YLDs Rate: Cumulative change (%)	-5.0 (-8.7 to -1.8)	-3.6 (-18.6 to 10.6)	0.9 (-1.1 to 3.0)	-3.8 (-15.3 to 8.9)	-11.7 (-22.5 to -2.0)	-26.6 (-35.1 to -16.4)	28.8 (8.7–54.6)	-19.8 (-47.5 to 17.5)	-37.6 (-59.1 to -8.7)	4.5 (-17.0 to 37.7)	-27.0 (-34.1 to -19.9)
Slovenia	Prevalence Number: 1990	487,528 (481,725–492,420)	142,801 (131,390–157,315)	193,280 (187,320–199,449)	21,771 (18,087–25,704)	52,288 (43,963–64,923)	571 (531–616)	10,828 (9,329–12,694)	2,385 (1,776–3,176)	1,583 (1,125–2,133)	0 (0–0)	4,481 (3,910–5,162)
Slovenia	Prevalence Number: 2015	343,924 (338,860–348,348)	106,698 (94,449–120,152)	132,101 (127,777–136,097)	13,863 (11,272–16,640)	31,560 (26,663–39,360)	336 (314–355)	7,414 (6,498–8,608)	1,555 (1,145–2,053)	865 (613–1,181)	0 (0–0)	3,055 (2,677–3,518)
Slovenia	Prevalence Number: Cumulative change (%)	-29.5 (-30.6 to -28.3)	-25.0 (-37.2 to -14.1)	-31.6 (-34.4 to -28.9)	-36.2 (-43.2 to -29.3)	-39.0 (-52.8 to -21.9)	-41.1 (-44.3 to -37.8)	-31.0 (-43.8 to -16.4)	-32.7 (-56.4 to 0.3)	-43.8 (-63.0 to -20.1)	-24.6 (-27.2 to -22.0)	-31.5 (-41.7 to -18.9)
Slovenia	Prevalence Rate: 1990	86,228.5 (85,128.3–87,188.4)	25,932.2 (23,574.0–28,914.4)	33,369.1 (32,327.2–34,422.1)	3,710.4 (3,085.5–4,383.8)	9,319.9 (7,837.8–11,587.0)	114.9 (106.5–124.7)	1,932.8 (1,666.1–2,265.8)	512.7 (381.7–682.7)	280.5 (199.2–375.1)	0.0 (0.0–0.0)	854.9 (751.8–973.9)
Slovenia	Prevalence Rate: 2015	87,129.2 (85,871.5–88,235.5)	26,905.5 (23,882.0–30,217.6)	33,681.8 (32,586.1–34,707.3)	3,533.7 (2,879.1–4,241.6)	7,993.1 (6,752.6–9,968.7)	83.5 (78.1–88.3)	1,876.3 (1,644.6–2,177.6)	383.8 (282.7–506.9)	219.2 (155.7–299.1)	0.0 (0.0–0.0)	769.2 (673.4–886.4)
Slovenia	Prevalence Rate: Cumulative change (%)	1.0 (-0.6 to 2.8)	4.2 (-13.3 to 19.6)	1.0 (-3.0 to 5.0)	-4.7 (-15.0 to 5.7)	-13.3 (-33.0 to 10.9)	-27.2 (-31.5 to -23.2)	-2.1 (-20.3 to 18.8)	-22.7 (-49.9 to 15.2)	-19.7 (-47.1 to 13.2)	-0.0 (-0.0 to 0.0)	-9.6 (-22.8 to 6.6)
Slovenia	YLDs Number: 1990	30,009 (22,719–38,260)	5,289 (3,694–7,402)	4,032 (2,739–5,688)	989 (649–1,374)	491 (339–683)	94 (66–125)	849 (553–1,207)	299 (187–438)	572 (353–831)	0 (0–0)	569 (432–744)
Slovenia	YLDs Number: 2015	19,478 (14,755–24,702)	3,916 (2,666–5,391)	2,793 (1,910–3,920)	631 (412–878)	323 (228–436)	55 (39–74)	751 (413–1,299)	195 (118–291)	189 (112–283)	0 (0–0)	283 (218–363)
Slovenia	YLDs Number: Cumulative change (%)	-35.1 (-38.7 to -32.0)	-25.5 (-42.0 to -14.9)	-30.7 (-32.2 to -29.1)	-36.1 (-43.3 to -28.9)	-33.8 (-45.3 to -20.0)	-40.8 (-48.8 to -31.7)	-12.3 (-32.8 to 38.4)	-32.6 (-56.7 to 0.8)	-66.0 (-78.7 to -49.7)	-21.2 (-36.2 to -3.4)	-50.1 (-55.4 to -44.2)
Slovenia	YLDs Rate: 1990	5,225.9 (3,949.7–6,682.2)	952.9 (663.5–1,338.2)	705.1 (479.4–994.4)	168.7 (110.5–235.4)	86.4 (59.3–120.7)	18.9 (13.4–25.3)	151.7 (98.7–215.7)	64.3 (40.3–94.2)	101.5 (62.8–147.1)	0.0 (0.0–0.0)	101.2 (76.7–132.0)
Slovenia	YLDs Rate: 2015	4,959.4 (3,755.7–6,290.8)	989.7 (673.2–1,359.0)	709.8 (484.9–995.3)	160.9 (104.9–224.2)	82.3 (58.2–111.2)	13.8 (9.8–18.5)	190.1 (104.6–328.9)	48.2 (29.3–71.9)	47.9 (28.6–71.9)	0.0 (0.0–0.0)	71.7 (55.1–92.0)
Slovenia	YLDs Rate: Cumulative change (%)	-5.0 (-10.9 to -0.6)	4.6 (-20.1 to 19.9)	0.7 (-1.4 to 2.6)	-4.5 (-15.0 to 6.0)	-4.0 (-21.7 to 15.6)	-26.9 (-36.7 to -15.6)	24.2 (-4.7 to 95.8)	-22.6 (-50.1 to 15.8)	-51.4 (-69.3 to -28.2)	6.3 (-14.5 to 30.0)	-28.9 (-36.4 to -20.4)
Central Asia	Prevalence Number: 1990	28,384,916 (28,252,793–28,520,373)	9,712,259 (9,452,727–9,979,044)	9,877,638 (9,730,291–10,033,760)	1,464,802 (1,237,270–1,699,260)	2,991,953 (2,922,258–3,070,945)	382,304 (346,848–419,286)	399,545 (368,323–439,384)	307,104 (228,573–405,763)	166,397 (144,187–190,856)	2,317 (1,381–3,433)	496,501 (447,962–555,047)
Central Asia	Prevalence Number: 2015	27,637,487 (27,485,075–27,791,619)	8,895,722 (8,573,309–9,268,961)	9,860,451 (9,706,968–10,016,701)	1,287,972 (1,074,382–1,525,480)	2,758,875 (2,690,782–2,824,571)	191,465 (177,224–206,313)	430,890 (397,808–473,630)	200,781 (151,808–263,484)	162,189 (139,553–188,354)	9 (2–33)	492,888 (445,161–547,953)
Central Asia	Prevalence Number: Cumulative change (%)	-2.6 (-3.2 to -2.0)	-8.4 (-11.9 to -4.3)	-0.2 (-2.0 to 1.6)	-12.1 (-18.6 to -5.2)	-7.8 (-10.5 to -4.9)	-49.8 (-53.6 to -45.9)	8.1 (-3.2 to 19.5)	-32.6 (-56.4 to -3.7)	-2.1 (-17.5 to 14.1)	-99.6 (-99.9 to -98.4)	-0.6 (-8.5 to 8.0)
Central Asia	Prevalence Rate: 1990	91,020.5 (90,616.8–91,433.7)	30,582.8 (29,836.8–31,327.2)	32,668.5 (32,184.2–33,170.8)	4,819.6 (4,071.1–5,588.9)	9,558.6 (9,335.5–9,809.4)	1,104.4 (1,005.2–1,208.4)	1,270.8 (1,171.1–1,397.1)	859.0 (639.3–1,134.9)	544.9 (472.2–624.5)	7.3 (4.3–10.7)	1,504.0 (1,352.5–1,685.4)
Central Asia	Prevalence Rate: 2015	90,262.5 (89,801.5–90,731.5)	28,647.6 (27,718.7–29,729.2)	32,897.6 (32,382.8–33,413.5)	4,346.7 (3,630.3–5,150.2)	8,982.2 (8,763.6–9,194.3)	571.8 (532.0–614.6)	1,397.7 (1,290.4–1,535.9)	566.8 (428.6–743.8)	541.3 (464.1–629.7)	0.0 (0.0–0.1)	1,523.0 (1,373.8–1,695.1)
Central Asia	Prevalence Rate: Cumulative change (%)	-0.8 (-1.4 to -0.2)	-6.3 (-9.6 to -2.5)	0.7 (-1.1 to 2.5)	-9.8 (-16.8 to -2.7)	-6.0 (-8.8 to -3.2)	-48.1 (-51.9 to -44.2)	10.2 (-1.4 to 21.9)	-32.0 (-56.0 to -2.8)	-0.2 (-16.0 to 16.3)	-99.6 (-99.9 to -98.5)	1.4 (-7.0 to 10.7)
Central Asia	YLDs Number: 1990	1,876,662 (1,423,423–2,391,857)	376,103 (266,683–505,075)	205,915 (140,911–289,318)	66,456 (44,804–90,969)	41,629 (29,876–55,666)	62,614 (45,298–83,770)	27,615 (18,910–38,457)	38,315 (23,994–57,084)	60,540 (42,622–78,616)	48 (25–81)	54,023 (42,254–67,449)
Central Asia	YLDs Number: 2015	1,682,951 (1,279,944–2,140,558)	336,361 (240,170–451,637)	204,701 (139,997–288,655)	58,557 (38,846–80,189)	37,625 (27,054–50,706)	31,413 (22,408–41,504)	32,170 (21,577–45,592)	25,109 (15,796–36,686)	41,611 (29,324–55,761)	0 (0–1)	48,759 (37,835–61,391)
Central Asia	YLDs Number: Cumulative change (%)	-10.3 (-13.5 to -7.3)	-10.6 (-15.1 to -5.0)	-0.6 (-1.6 to 0.3)	-11.9 (-18.7 to -4.9)	-9.6 (-13.6 to -5.3)	-49.8 (-53.6 to -45.6)	16.6 (0.8–39.4)	-32.4 (-56.3 to -3.4)	-31.0 (-42.6 to -18.1)	-99.2 (-99.8 to -97.1)	-9.8 (-14.6 to -5.0)
Central Asia	YLDs Rate: 1990	6,089.0 (4,619.8–7,743.9)	1,183.5 (840.7–1,590.1)	670.8 (459.9–947.2)	218.6 (146.8–299.9)	132.5 (95.2–177.0)	180.8 (131.2–241.6)	87.8 (60.1–122.2)	107.2 (67.1–159.7)	198.1 (140.0–256.7)	0.2 (0.1–0.3)	170.7 (133.6–212.8)
Central Asia	YLDs Rate: 2015	5,565.3 (4,241.8–7,075.9)	1,095.3 (784.0–1,469.6)	675.6 (463.2–959.1)	197.6 (131.1–269.9)	124.3 (89.4–167.2)	93.8 (67.1–123.9)	104.3 (69.9–147.8)	70.9 (44.6–103.6)	138.8 (97.7–185.8)	0.0 (0.0–0.0)	157.6 (122.2–198.8)
Central Asia	YLDs Rate: Cumulative change (%)	-8.6 (-11.4 to -5.9)	-7.4 (-11.6 to -2.3)	0.7 (-0.2 to 1.6)	-9.6 (-16.6 to -2.4)	-6.2 (-10.1 to -2.2)	-48.1 (-51.9 to -43.9)	19.0 (2.7–42.3)	-31.8 (-55.9 to -2.5)	-29.7 (-41.6 to -16.5)	-99.2 (-99.8 to -97.2)	-7.7 (-12.6 to -2.7)
Armenia	Prevalence Number: 1990	1,191,131 (1,177,851–1,204,320)	319,295 (290,410–364,239)	425,488 (413,169–438,206)	52,545 (43,326–62,849)	121,517 (115,042–129,049)	16,140 (14,967–17,616)	18,214 (15,186–22,144)	6,885 (3,232–11,821)	6,697 (5,030–8,584)	314 (66–597)	22,697 (19,718–26,642)
Armenia	Prevalence Number: 2015	642,930 (635,318–649,443)	176,981 (164,233–188,233)	238,003 (230,794–245,463)	27,209 (22,336–32,623)	61,266 (57,423–65,523)	4,336 (3,998–4,720)	10,675 (9,166–12,572)	3,312 (1,745–5,578)	2,833 (2,091–3,674)	0 (0–0)	10,873 (9,519–12,502)
Armenia	Prevalence Number: Cumulative change (%)	-46.0 (-46.8 to -45.3)	-44.2 (-53.3 to -36.5)	-44.0 (-46.2 to -41.7)	-48.2 (-51.4 to -44.7)	-49.5 (-53.6 to -45.6)	-73.1 (-75.4 to -70.9)	-40.7 (-53.1 to -27.0)	-44.1 (-78.6 to 17.3)	-56.6 (-70.6 to -39.2)	-99.0 (-100.0 to -100.0)	-51.8 (-59.2 to -44.1)
Armenia	Prevalence Rate: 1990	89,082.1 (88,141.1–90,033.7)	23,512.0 (21,429.7–26,730.0)	32,675.5 (31,729.2–33,650.5)	3,915.6 (3,228.4–4,655.1)	9,062.0 (8,578.7–9,628.8)	1,151.1 (1,069.4–1,255.4)	1,356.9 (1,131.5–1,650.2)	484.9 (227.7–832.5)	503.2 (378.2–645.5)	23.3 (4.9–44.1)	1,675.2 (1,452.8–1,968.3)
Armenia	Prevalence Rate: 2015	88,855.2 (87,815.2–89,739.7)	24,550.7 (22,844.8–26,078.4)	33,023.4 (32,040.7–34,059.1)	3,796.5 (3,121.6–4,553.9)	8,476.3 (7,943.2–9,063.0)	588.0 (542.5–639.6)	1,474.0 (1,266.0–1,736.6)	442.0 (232.9–744.4)	393.2 (290.3–509.5)	0.0 (0.0–0.0)	1,498.8 (1,312.2–1,723.9)
Armenia	Prevalence Rate: Cumulative change (%)	-0.3 (-1.7 to 1.1)	5.0 (-11.4 to 19.0)	1.1 (-2.7 to 5.3)	-3.0 (-8.8 to 3.3)	-6.4 (-13.9 to 0.9)	-48.8 (-53.3 to -44.8)	9.8 (-13.1 to 35.4)	5.8 (-59.4 to 122.2)	-19.9 (-45.8 to 12.3)	-98.0 (-100.0 to -100.0)	-10.0 (-24.0 to 4.5)
Armenia	YLDs Number: 1990	78,398 (60,420–98,436)	11,789 (8,263–16,474)	8,848 (6,042–12,437)	2,387 (1,577–3,307)	1,820 (1,304–2,486)	2,649 (1,906–3,522)	1,418 (893–2,199)	860 (353–1,579)	2,432 (1,589–3,372)	6 (1–13)	2,754 (2,150–3,460)
Armenia	YLDs Number: 2015	37,213 (28,274–47,028)	6,456 (4,579–8,781)	4,892 (3,345–6,928)	1,238 (829–1,704)	808 (571–1,090)	713 (509–952)	952 (578–1,498)	416 (195–746)	718 (458–1,054)	0 (0–0)	1,143 (885–1,451)
Armenia	YLDs Number: Cumulative change (%)	-52.6 (-55.6 to -49.8)	-44.8 (-55.6 to -37.1)	-44.7 (-45.9 to -43.6)	-48.0 (-51.5 to -44.2)	-55.5 (-60.9 to -51.3)	-73.0 (-75.6 to -70.5)	-31.5 (-54.3 to -0.4)	-43.9 (-78.3 to 19.3)	-69.7 (-80.3 to -56.9)	-98.9 (-100.0 to -100.0)	-58.4 (-61.9 to -54.6)
Armenia	YLDs Rate: 1990	5,935.0 (4,582.4–7,451.2)	861.2 (604.0–1,200.0)	670.4 (456.5–950.6)	177.9 (117.5–246.2)	133.7 (95.8–181.8)	189.0 (135.9–250.9)	105.6 (66.6–163.7)	60.6 (24.9–111.2)	182.7 (119.1–253.5)	0.5 (0.1–1.0)	204.4 (159.4–256.7)
Armenia	YLDs Rate: 2015	5,171.6 (3,932.4–6,539.4)	904.7 (642.4–1,230.1)	677.1 (463.3–960.5)	172.9 (115.6–237.9)	113.9 (80.6–154.1)	96.8 (69.1–129.1)	131.5 (79.9–207.6)	55.5 (26.1–99.7)	99.7 (63.6–146.0)	0.0 (0.0–0.0)	158.0 (122.4–200.4)

© 2017 Global Burden of Disease Child and Adolescent Health Collaboration. JAMA Pediatrics.

eTable 3. Prevalent Cases, Rates (per 100,000 population), Years Lived with Disability (YLDs), and Cumulative Percent Change with 95% Uncertainty Interval (UI) for the Top 10 Global Causes of YLDs in Children and Adolescents in 195 Countries and Territories, Aged 0 to 19 Years, Both Sexes, 1990 and 2015. *Note: best viewed by enlarging in browser.*

Location	Measure	All causes	Iron-deficiency anemia	Skin and subcutaneous diseases	Asthma	Hemoglobinopathies and hemolytic anemias	Diarrheal diseases	Congenital anomalies	Protein-energy malnutrition	Epilepsy	Malaria	Neonatal preterm birth complications
Armenia	YLDs Rate: Cumulative change (%)	-12.9 (-18.3 to -8.0)	5.9 (-14.3 to 20.2)	1.0 (-1.0 to 2.9)	-2.7 (-9.2 to 4.2)	-14.6 (-24.6 to -7.0)	-48.8 (-53.5 to -44.0)	27.0 (-15.2 to 84.7)	6.3 (-58.8 to 125.9)	-44.1 (-63.4 to -20.3)	-98.0 (-100.0 to -100.0)	-22.6 (-29.1 to -15.5)
Azerbaijan	Prevalence Number: 1990	2,795,786 (2,768,058–2,824,825)	873,311 (800,655–942,688)	998,710 (969,000–1,028,884)	120,170 (99,803–142,337)	337,552 (323,351–351,661)	32,833 (30,425–35,587)	31,302 (25,533–39,272)	19,606 (10,116–33,789)	20,002 (14,825–25,699)	332 (96–636)	36,955 (31,594–44,279)
Azerbaijan	Prevalence Number: 2015	2,558,259 (2,527,356–2,582,768)	784,073 (707,864–818,368)	934,256 (904,297–965,287)	111,682 (91,288–133,593)	291,916 (281,437–303,360)	20,023 (18,149–21,910)	36,046 (30,456–43,714)	20,368 (10,197–35,750)	18,293 (13,749–23,413)	0 (0–0)	35,309 (30,506–41,519)
Azerbaijan	Prevalence Number: Cumulative change (%)	-8.5 (-9.7 to -7.2)	-10.0 (-21.1 to 1.4)	-6.4 (-10.2 to -2.6)	-6.9 (-17.5 to 5.1)	-13.5 (-17.9 to -8.6)	-39.0 (-43.2 to -35.2)	16.9 (-11.1 to 48.4)	19.6 (-57.7 to 151.7)	-6.3 (-35.7 to 28.4)	-98.8 (-100.0 to -100.0)	-3.7 (-20.6 to 13.1)
Azerbaijan	Prevalence Rate: 1990	90,250.3 (89,392.0–91,163.3)	27,840.6 (25,646.3–29,942.6)	32,812.8 (31,839.1–33,761.3)	3,936.2 (3,264.1–4,666.1)	10,864.9 (10,403.3–11,317.2)	999.8 (927.3–1,083.0)	1,003.4 (819.0–1,259.9)	586.7 (302.7–1,011.0)	647.6 (479.4–831.8)	10.6 (3.1–20.3)	1,134.6 (965.8–1,372.3)
Azerbaijan	Prevalence Rate: 2015	89,646.3 (88,685.9–90,471.1)	26,928.4 (24,669.2–27,893.9)	33,055.7 (31,994.4–34,107.0)	4,195.4 (3,408.0–5,029.6)	10,174.1 (9,815.0–10,570.1)	614.5 (561.7–667.8)	1,249.6 (1,058.1–1,514.0)	560.2 (280.5–983.2)	649.1 (487.6–833.4)	0.0 (0.0–0.0)	1,134.1 (973.0–1,344.0)
Azerbaijan	Prevalence Rate: Cumulative change (%)	-0.7 (-1.9 to 0.6)	-3.0 (-13.4 to 8.2)	0.8 (-3.4 to 4.9)	6.7 (-5.7 to 20.6)	-6.3 (-11.1 to -1.1)	-38.5 (-42.7 to -34.8)	26.4 (-3.7 to 60.6)	9.9 (-61.1 to 131.4)	2.7 (-29.4 to 40.8)	-98.7 (-100.0 to -100.0)	0.8 (-18.2 to 19.9)
Azerbaijan	YLDs Number: 1990	177,379 (134,156–225,954)	33,467 (23,722–45,542)	20,658 (14,059–29,201)	5,461 (3,648–7,575)	5,532 (3,938–7,422)	5,382 (3,856–7,166)	1,948 (1,297–2,809)	2,450 (1,097–4,506)	7,315 (4,733–10,378)	7 (1–14)	3,889 (2,973–4,913)
Azerbaijan	YLDs Number: 2015	152,356 (115,710–194,921)	28,521 (20,111–38,815)	19,233 (13,164–27,176)	5,083 (3,343–7,019)	4,586 (3,256–6,216)	3,288 (2,341–4,385)	2,303 (1,488–3,388)	2,549 (1,150–4,779)	4,568 (2,972–6,577)	0 (0–0)	3,510 (2,685–4,494)
Azerbaijan	YLDs Number: Cumulative change (%)	-14.1 (-17.8 to -10.3)	-14.4 (-28.2 to -1.6)	-6.9 (-8.8 to -4.9)	-6.8 (-17.8 to 5.8)	-16.9 (-26.9 to -8.4)	-38.9 (-43.6 to -34.2)	18.7 (-0.6 to 47.6)	20.0 (-58.5 to 157.1)	-36.0 (-57.2 to -10.6)	-98.9 (-100.0 to -100.0)	-9.6 (-18.9 to -0.1)
Azerbaijan	YLDs Rate: 1990	5,772.3 (4,367.8–7,347.5)	1,068.6 (756.0–1,452.5)	673.6 (458.3–954.4)	178.9 (120.0–248.1)	178.1 (126.6–238.7)	163.9 (117.3–218.3)	62.5 (41.6–90.2)	73.3 (32.8–134.8)	236.7 (152.9–336.4)	0.2 (0.1–0.5)	124.0 (94.7–156.7)
Azerbaijan	YLDs Rate: 2015	5,428.7 (4,130.3–6,926.5)	1,015.7 (723.3–1,370.2)	676.2 (460.5–957.2)	190.9 (125.8–264.3)	169.1 (120.6–229.4)	100.8 (71.8–134.3)	79.8 (51.7–117.4)	70.1 (31.6–131.4)	162.0 (105.3–234.7)	0.0 (0.0–0.0)	121.2 (92.9–155.1)
Azerbaijan	YLDs Rate: Cumulative change (%)	-5.9 (-9.4 to -2.2)	-4.6 (-17.5 to 8.4)	0.4 (-1.6 to 2.3)	6.9 (-5.9 to 21.0)	-4.9 (-14.6 to 3.8)	-38.4 (-43.1 to -33.9)	28.2 (7.4–59.5)	10.3 (-61.9 to 136.3)	-29.9 (-52.8 to -1.7)	-98.8 (-100.0 to -100.0)	-2.1 (-12.3 to 8.4)
Georgia	Prevalence Number: 1990	1,573,703 (1,558,497–1,588,474)	462,469 (434,833–493,827)	572,476 (554,866–591,337)	101,317 (78,507–121,893)	135,953 (129,383–143,463)	12,870 (11,834–13,939)	24,065 (20,289–29,456)	9,033 (4,487–15,760)	10,292 (7,775–13,107)	320 (69–611)	26,512 (23,370–30,677)
Georgia	Prevalence Number: 2015	836,559 (826,691–845,207)	248,151 (228,552–270,497)	306,786 (296,943–316,799)	47,109 (38,761–55,723)	66,707 (62,914–70,661)	4,830 (4,475–5,250)	14,275 (12,297–16,868)	3,033 (1,540–4,962)	3,481 (2,531–4,486)	0 (0–0)	15,138 (13,212–17,345)
Georgia	Prevalence Number: Cumulative change (%)	-46.8 (-47.6 to -46.1)	-46.3 (-52.4 to -40.8)	-46.4 (-48.6 to -44.2)	-53.0 (-61.8 to -39.0)	-50.9 (-54.4 to -47.1)	-62.4 (-64.9 to -59.9)	-40.1 (-53.2 to -26.0)	-60.9 (-85.8 to -19.0)	-65.4 (-77.1 to -52.2)	-99.4 (-100.0 to -100.0)	-42.6 (-51.6 to -32.3)
Georgia	Prevalence Rate: 1990	89,468.9 (88,607.6–90,315.8)	26,334.8 (24,750.7–28,124.4)	32,584.2 (31,584.5–33,663.9)	5,734.5 (4,442.6–6,892.3)	7,734.1 (7,360.5–8,159.6)	737.2 (677.8–798.3)	1,369.5 (1,154.7–1,676.4)	519.3 (258.0–906.1)	585.5 (442.4–745.3)	18.2 (3.9–34.8)	1,526.5 (1,346.3–1,763.3)
Georgia	Prevalence Rate: 2015	89,024.5 (88,038.7–89,898.4)	26,510.0 (24,637.4–28,620.8)	32,668.2 (31,630.2–33,724.9)	5,195.9 (4,272.7–6,167.6)	7,103.7 (6,698.9–7,520.9)	484.3 (450.5–524.6)	1,514.0 (1,303.4–1,788.6)	286.7 (145.6–469.2)	371.4 (270.8–479.3)	0.0 (0.0–0.0)	1,573.6 (1,369.2–1,807.1)
Georgia	Prevalence Rate: Cumulative change (%)	-0.5 (-1.8 to 0.8)	0.8 (-10.0 to 10.2)	0.3 (-3.9 to 4.5)	-8.4 (-25.5 to 19.3)	-8.1 (-14.5 to -1.2)	-34.2 (-38.6 to -30.0)	11.7 (-12.8 to 37.6)	-35.7 (-76.7 to 33.1)	-35.2 (-57.1 to -10.4)	-98.8 (-100.0 to -100.0)	3.6 (-12.5 to 22.2)
Georgia	YLDs Number: 1990	98,512 (74,617–125,131)	17,186 (12,040–23,503)	11,195 (7,571–16,114)	4,611 (2,957–6,442)	1,528 (1,083–2,082)	2,113 (1,509–2,818)	1,554 (1,062–2,152)	1,130 (500–2,107)	3,775 (2,463–5,304)	6 (1–14)	3,469 (2,740–4,326)
Georgia	YLDs Number: 2015	49,502 (37,761–62,860)	8,947 (6,250–12,337)	6,009 (4,069–8,549)	2,143 (1,422–2,934)	727 (513–992)	793 (566–1,050)	1,093 (730–1,559)	377 (173–651)	881 (561–1,272)	0 (0–0)	1,643 (1,268–2,093)
Georgia	YLDs Number: Cumulative change (%)	-49.7 (-52.1 to -47.7)	-47.8 (-55.2 to -42.1)	-46.3 (-47.4 to -45.2)	-52.9 (-61.8 to -38.9)	-52.3 (-58.1 to -47.5)	-62.4 (-65.3 to -59.3)	-29.6 (-37.3 to -19.3)	-60.9 (-86.0 to -18.4)	-76.1 (-84.4 to -66.3)	-99.4 (-100.0 to -100.0)	-52.6 (-57.1 to -47.7)
Georgia	YLDs Rate: 1990	5,605.9 (4,247.3–7,119.1)	977.1 (685.0–1,338.6)	636.7 (430.5–916.6)	261.0 (167.4–364.6)	86.8 (61.5–118.1)	121.0 (86.5–161.4)	88.5 (60.4–122.5)	65.0 (28.8–121.2)	214.8 (140.1–301.7)	0.4 (0.1–0.8)	197.5 (155.9–246.2)
Georgia	YLDs Rate: 2015	5,319.4 (4,052.5–6,765.2)	983.2 (696.4–1,343.3)	638.4 (431.8–910.3)	236.4 (157.5–324.6)	81.6 (57.8–111.0)	79.5 (56.9–105.2)	115.9 (77.4–165.0)	35.7 (16.4–61.6)	94.0 (59.8–135.7)	0.0 (0.0–0.0)	174.6 (134.8–222.4)
Georgia	YLDs Rate: Cumulative change (%)	-5.0 (-9.3 to -1.6)	0.9 (-12.5 to 11.4)	0.3 (-1.7 to 2.2)	-8.3 (-25.6 to 19.7)	-5.8 (-16.4 to 2.7)	-34.2 (-39.2 to -28.8)	31.2 (16.8–50.0)	-35.7 (-77.0 to 34.2)	-55.2 (-70.6 to -36.9)	-98.8 (-100.0 to -100.0)	-11.6 (-20.0 to -2.3)
Kazakhstan	Prevalence Number: 1990	6,254,738 (6,197,275–6,315,232)	2,544,086 (2,353,258–2,738,473)	2,173,607 (2,102,778–2,241,892)	306,369 (253,842–360,256)	642,944 (607,004–684,932)	57,907 (53,771–62,901)	101,168 (87,332–119,960)	50,300 (26,051–81,908)	29,526 (21,794–37,662)	0 (0–0)	104,574 (90,958–120,954)
Kazakhstan	Prevalence Number: 2015	5,295,929 (5,249,243–5,339,012)	2,136,025 (2,002,993–2,238,852)	1,809,679 (1,748,129–1,868,469)	206,580 (170,218–244,629)	511,802 (482,083–545,771)	27,308 (25,562–29,112)	90,372 (78,071–107,427)	32,925 (17,632–55,143)	23,560 (17,440–29,960)	0 (0–0)	87,798 (77,394–99,996)
Kazakhstan	Prevalence Number: Cumulative change (%)	-15.3 (-16.3 to -14.3)	-15.9 (-21.3 to -6.5)	-16.7 (-20.3 to -13.4)	-32.5 (-38.0 to -26.9)	-20.3 (-26.8 to -14.1)	-52.8 (-56.1 to -49.6)	-9.9 (-27.5 to 11.0)	-25.9 (-71.0 to 46.9)	-18.4 (-44.0 to 13.6)	-7.7 (-25.7 to 15.7)	-15.7 (-27.1 to -3.5)
Kazakhstan	Prevalence Rate: 1990	93,356.1 (92,560.4–94,229.6)	37,616.7 (34,961.8–40,187.2)	33,121.9 (32,050.7–34,164.8)	4,665.5 (3,862.9–5,485.9)	9,587.8 (9,046.1–10,215.8)	805.9 (749.2–873.8)	1,504.0 (1,297.0–1,784.1)	681.9 (353.2–1,110.3)	448.0 (330.2–572.7)	0.0 (0.0–0.0)	1,528.4 (1,327.9–1,772.0)
Kazakhstan	Prevalence Rate: 2015	92,592.4 (91,875.3–93,259.9)	36,384.0 (34,354.6–38,000.3)	33,473.4 (32,355.0–34,510.1)	3,797.2 (3,139.8–4,483.0)	8,930.8 (8,412.2–9,520.0)	415.6 (389.9–441.4)	1,566.5 (1,353.2–1,860.2)	466.6 (249.9–781.5)	429.0 (317.4–548.7)	0.0 (0.0–0.0)	1,439.2 (1,261.4–1,650.9)
Kazakhstan	Prevalence Rate: Cumulative change (%)	-0.8 (-2.0 to 0.3)	-3.1 (-9.2 to 6.8)	1.1 (-3.2 to 5.2)	-18.5 (-25.2 to -11.6)	-6.7 (-14.1 to 0.5)	-48.4 (-51.9 to -45.0)	5.0 (-15.3 to 29.3)	-22.5 (-69.7 to 53.6)	-2.1 (-32.6 to 36.3)	0.1 (-18.6 to 24.2)	-5.4 (-18.6 to 8.9)
Kazakhstan	YLDs Number: 1990	423,150 (320,393–543,417)	104,215 (73,373–141,108)	45,393 (30,999–63,232)	13,854 (9,332–18,917)	8,860 (6,344–11,964)	9,448 (6,857–12,511)	6,545 (4,577–8,846)	6,258 (2,986–11,149)	10,764 (6,885–14,961)	0 (0–0)	12,800 (9,981–16,116)
Kazakhstan	YLDs Number: 2015	318,651 (242,057–407,489)	85,461 (60,526–116,517)	38,357 (26,291–53,679)	9,372 (6,137–12,837)	6,989 (4,936–9,552)	4,465 (3,202–5,862)	5,794 (3,958–8,091)	4,101 (1,955–7,262)	5,795 (3,636–8,212)	0 (0–0)	9,288 (7,120–11,865)
Kazakhstan	YLDs Number: Cumulative change (%)	-24.7 (-27.4 to -21.3)	-17.8 (-24.8 to -5.9)	-15.5 (-17.3 to -13.6)	-32.3 (-37.9 to -26.1)	-20.9 (-28.9 to -11.5)	-52.7 (-56.7 to -48.4)	-11.6 (-20.9 to -2.1)	-25.8 (-70.9 to 48.8)	-44.8 (-62.9 to -22.0)	-8.5 (-28.9 to 18.1)	-27.4 (-34.3 to -20.3)
Kazakhstan	YLDs Rate: 1990	6,381.9 (4,843.3–8,183.1)	1,536.4 (1,086.4–2,081.5)	684.2 (466.1–953.6)	210.9 (142.3–287.0)	131.6 (94.4–177.6)	131.5 (95.3–174.2)	97.3 (68.0–131.4)	84.8 (40.5–151.1)	163.3 (104.2–227.2)	0.0 (0.0–0.0)	189.6 (148.0–238.8)
Kazakhstan	YLDs Rate: 2015	5,744.5 (4,379.3–7,321.8)	1,454.1 (1,030.3–1,973.7)	690.3 (472.7–972.9)	172.2 (113.6–235.4)	121.3 (85.6–164.9)	67.9 (48.8–89.3)	100.3 (68.7–139.9)	58.1 (27.7–102.9)	105.4 (66.2–150.0)	0.0 (0.0–0.0)	160.0 (122.8–204.7)
Kazakhstan	YLDs Rate: Cumulative change (%)	-10.0 (-12.9 to -6.3)	-5.1 (-12.9 to 7.8)	0.9 (-1.2 to 2.9)	-18.3 (-25.1 to -11.0)	-7.6 (-16.4 to 3.1)	-48.2 (-52.6 to -43.7)	3.0 (-7.7 to 13.7)	-22.4 (-69.5 to 55.6)	-33.8 (-55.7 to -6.8)	-0.3 (-21.9 to 27.7)	-15.6 (-23.5 to -7.2)
Kyrgyzstan	Prevalence Number: 1990	1,922,467 (1,904,270–1,936,997)	636,189 (585,522–675,448)	654,656 (633,621–675,436)	82,082 (68,976–96,611)	195,399 (183,442–207,974)	27,330 (23,593–31,348)	26,038 (22,070–31,165)	12,084 (5,891–20,836)	12,416 (9,209–15,692)	391 (94–748)	34,110 (29,683–40,216)
Kyrgyzstan	Prevalence Number: 2015	2,140,270 (2,123,238–2,158,088)	702,976 (674,660–730,701)	735,563 (713,911–756,728)	93,553 (77,118–112,170)	205,092 (193,973–218,038)	16,101 (15,034–17,393)	32,919 (27,998–39,536)	10,351 (6,474–14,975)	12,223 (9,179–15,526)	0 (0–0)	46,627 (40,900–53,929)
Kyrgyzstan	Prevalence Number: Cumulative change (%)	11.3 (10.2–12.5)	10.7 (2.9–20.5)	12.4 (7.9–16.9)	14.2 (0.5–27.5)	5.1 (-2.9 to 13.6)	-40.7 (-47.5 to -33.0)	27.7 (0.8–58.5)	-1.1 (-58.3 to 84.1)	0.8 (-30.1 to 39.2)	-98.9 (-100.0 to -100.0)	37.5 (16.8–61.2)
Kyrgyzstan	Prevalence Rate: 1990	92,570.2 (91,757.2–93,222.3)	30,008.8 (27,765.9–31,644.6)	32,616.3 (31,568.4–33,640.3)	4,092.5 (3,443.2–4,801.8)	9,368.1 (8,784.2–9,968.7)	1,169.8 (1,013.1–1,338.0)	1,242.4 (1,053.0–1,488.9)	501.4 (244.5–864.6)	616.7 (457.5–780.3)	18.4 (4.4–34.7)	1,543.1 (1,340.3–1,835.0)
Kyrgyzstan	Prevalence Rate: 2015	91,939.1 (91,271.4–92,643.2)	29,585.4 (28,471.0–30,618.4)	32,584.6 (31,615.0–33,509.9)	4,183.7 (3,435.0–5,015.4)	8,775.8 (8,304.2–9,332.7)	613.8 (574.7–661.1)	1,402.8 (1,192.9–1,684.4)	371.3 (232.2–537.2)	542.9 (406.6–691.1)	0.0 (0.0–0.0)	1,857.0 (1,620.9–2,163.5)
Kyrgyzstan	Prevalence Rate: Cumulative change (%)	-0.7 (-1.6 to 0.3)	-1.2 (-7.8 to 7.3)	-0.1 (-4.0 to 4.1)	2.4 (-9.9 to 14.2)	-6.2 (-13.3 to 1.3)	-47.2 (-53.1 to -40.6)	14.0 (-10.2 to 41.4)	-14.5 (-63.9 to 59.1)	-9.8 (-37.6 to 24.2)	-99.0 (-100.0 to -100.0)	21.1 (1.7–43.2)
Kyrgyzstan	YLDs Number: 1990	123,535 (94,028–157,344)	24,645 (17,375–33,545)	13,667 (9,323–19,295)	3,720 (2,482–5,060)	2,601 (1,853–3,498)	4,480 (3,152–6,097)	2,047 (1,390–2,843)	1,505 (654–2,754)	4,499 (2,905–6,371)	8 (1–16)	3,967 (3,095–4,979)
Kyrgyzstan	YLDs Number: 2015	126,033 (95,307–160,378)	26,348 (18,722–35,583)	15,372 (10,561–21,755)	4,251 (2,796–5,881)	2,687 (1,915–3,602)	2,646 (1,908–3,512)	2,920 (1,965–4,080)	1,293 (725–2,092)	3,103 (2,021–4,413)	0 (0–0)	4,750 (3,710–5,982)
Kyrgyzstan	YLDs Number: Cumulative change (%)	2.0 (-1.1 to 5.6)	7.2 (-1.2 to 19.1)	12.5 (10.4–14.8)	14.5 (0.0–28.0)	3.5 (-4.6 to 14.3)	-40.6 (-47.8 to -32.5)	43.1 (22.4–67.3)	-0.8 (-58.1 to 85.8)	-29.3 (-52.8 to -0.7)	-98.9 (-100.0 to -100.0)	19.9 (11.3–29.5)
Kyrgyzstan	YLDs Rate: 1990	6,056.0 (4,614.4–7,699.5)	1,163.6 (824.0–1,573.2)	669.9 (456.8–948.5)	185.4 (123.2–251.7)	124.1 (88.7–167.0)	191.7 (135.1–261.0)	97.5 (66.4–135.0)	62.5 (27.2–114.3)	223.3 (144.2–317.1)	0.4 (0.1–0.8)	188.0 (146.6–236.1)
Kyrgyzstan	YLDs Rate: 2015	5,526.1 (4,189.0–7,010.2)	1,124.1 (798.1–1,523.0)	670.9 (457.1–954.5)	190.1 (124.8–261.6)	116.7 (83.2–156.2)	100.8 (72.7–133.9)	124.3 (83.7–174.0)	46.4 (26.0–75.1)	137.8 (89.8–195.7)	0.0 (0.0–0.0)	201.5 (157.2–253.6)
Kyrgyzstan	YLDs Rate: Cumulative change (%)	-8.7 (-11.4 to -5.8)	-3.2 (-10.3 to 7.2)	0.2 (-1.8 to 2.2)	2.7 (-10.1 to 15.3)	-5.8 (-13.1 to 3.8)	-47.1 (-53.2 to -40.2)	27.9 (9.7–49.6)	-14.2 (-63.7 to 60.6)	-36.8 (-57.6 to -11.6)	-99.0 (-100.0 to -100.0)	7.4 (-0.4 to 15.9)
Mongolia	Prevalence Number: 1990	1,008,134 (997,220–1,017,620)	300,309 (287,074–316,800)	357,693 (347,131–369,185)	57,632 (47,773–68,504)	107,854 (101,837–114,649)	9,896 (8,868–11,012)	13,446 (11,245–16,397)	4,731 (2,625–7,571)	4,291 (3,169–5,537)	0 (0–0)	17,099 (14,491–21,039)

© 2017 Global Burden of Disease Child and Adolescent Health Collaboration. JAMA Pediatrics.

eTable 3. Prevalent Cases, Rates (per 100,000 population), Years Lived with Disability (YLDs), and Cumulative Percent Change with 95% Uncertainty Interval (UI) for the Top 10 Global Causes of YLDs in Children and Adolescents in 195 Countries and Territories, Aged 0 to 19 Years, Both Sexes, 1990 and 2015. *Note: best viewed by enlarging in browser.*

Location	Measure	All causes	Iron-deficiency anemia	Skin and subcutaneous diseases	Asthma	Hemoglobinopathies and hemolytic anemias	Diarrheal diseases	Congenital anomalies	Protein-energy malnutrition	Epilepsy	Malaria	Neonatal preterm birth complications
Mongolia	Prevalence Number: 2015	929,868 (918,634–940,303)	250,135 (234,007–264,858)	334,601 (323,689–345,486)	46,438 (38,155–55,500)	93,974 (88,710–100,395)	5,260 (4,868–5,693)	15,240 (13,222–18,211)	2,804 (1,442–4,856)	4,366 (3,277–5,544)	0 (0–0)	15,841 (13,742–18,378)
Mongolia	Prevalence Number: Cumulative change (%)	-7.8 (-9.1 to -6.4)	-16.6 (-25.2 to -7.9)	-6.4 (-10.3 to -2.2)	-19.4 (-25.8 to -13.0)	-12.8 (-19.6 to -5.4)	-46.8 (-50.3 to -43.4)	14.6 (-9.7 to 43.7)	-34.3 (-74.2 to 28.4)	4.2 (-28.9 to 44.1)	-3.7 (-22.2 to 21.5)	-6.4 (-24.6 to 11.3)
Mongolia	Prevalence Rate: 1990	89,595.0 (88,687.5–90,379.4)	26,712.9 (25,601.3–28,124.7)	32,491.3 (31,540.5–33,518.6)	5,175.4 (4,291.6–6,130.4)	9,573.2 (9,042.7–10,178.6)	804.0 (723.2–893.1)	1,183.0 (987.0–1,443.8)	372.2 (206.6–595.6)	387.8 (287.5–501.8)	0.0 (0.0–0.0)	1,442.7 (1,218.8–1,795.2)
Mongolia	Prevalence Rate: 2015	88,674.2 (87,700.4–89,585.8)	23,966.9 (22,565.3–25,236.3)	32,944.1 (31,872.1–34,018.4)	4,544.5 (3,741.1–5,410.8)	8,942.5 (8,452.0–9,552.3)	439.8 (407.8–474.5)	1,434.9 (1,243.9–1,712.9)	222.6 (114.5–385.5)	429.2 (319.7–545.0)	0.0 (0.0–0.0)	1,393.6 (1,203.1–1,627.3)
Mongolia	Prevalence Rate: Cumulative change (%)	-1.0 (-2.3 to 0.3)	-10.2 (-19.1 to -1.8)	1.4 (-2.6 to 5.9)	-12.1 (-19.1 to -5.1)	-6.5 (-13.7 to 1.4)	-45.2 (-48.8 to -41.8)	22.7 (-3.4 to 53.9)	-33.7 (-74.0 to 29.5)	13.3 (-22.7 to 56.8)	0.1 (-18.6 to 25.5)	-2.3 (-22.4 to 17.8)
Mongolia	YLDs Number: 1990	62,514 (47,707–79,047)	11,124 (7,933–15,019)	7,499 (5,148–10,572)	2,618 (1,740–3,626)	1,499 (1,067–2,021)	1,630 (1,171–2,208)	1,198 (716–1,940)	592 (296–1,022)	1,562 (989–2,220)	0 (0–0)	1,579 (1,209–2,000)
Mongolia	YLDs Number: 2015	52,855 (39,983–67,378)	9,130 (6,449–12,462)	7,002 (4,774–9,870)	2,113 (1,377–2,941)	1,374 (976–1,882)	868 (621–1,158)	1,601 (866–2,627)	352 (163–639)	1,099 (694–1,570)	0 (0–0)	1,463 (1,117–1,858)
Mongolia	YLDs Number: Cumulative change (%)	-15.4 (-18.5 to -12.3)	-17.8 (-26.7 to -9.7)	-6.6 (-8.7 to -4.5)	-19.2 (-26.1 to -12.2)	-8.2 (-17.4 to 3.4)	-46.6 (-51.0 to -42.3)	40.1 (-23.5 to 135.6)	-34.1 (-74.4 to 30.2)	-27.8 (-52.1 to 3.2)	-0.6 (-26.6 to 32.6)	-7.1 (-16.6 to 3.2)
Mongolia	YLDs Rate: 1990	5,652.7 (4,321.3–7,146.5)	992.5 (709.7–1,338.2)	673.0 (460.5–951.0)	235.1 (155.9–321.6)	134.4 (96.0–180.8)	132.4 (95.1–179.0)	105.2 (63.0–170.3)	46.6 (23.3–80.5)	141.1 (89.1–199.9)	0.0 (0.0–0.0)	137.9 (105.6–174.7)
Mongolia	YLDs Rate: 2015	5,195.1 (3,937.0–6,618.0)	884.4 (626.9–1,204.8)	677.6 (460.0–961.1)	206.8 (134.6–287.4)	133.9 (95.7–182.5)	72.6 (51.9–96.9)	150.4 (81.5–246.9)	28.0 (13.0–50.8)	107.9 (68.2–154.6)	0.0 (0.0–0.0)	136.8 (104.2–173.7)
Mongolia	YLDs Rate: Cumulative change (%)	-8.1 (-11.3 to -4.9)	-10.7 (-20.1 to -2.2)	0.7 (-1.6 to 2.8)	-12.0 (-19.3 to -4.1)	-0.3 (-10.1 to 10.7)	-45.1 (-49.5 to -40.6)	49.9 (-17.9 to 152.4)	-33.5 (-74.2 to 31.4)	-21.5 (-48.2 to 12.0)	3.9 (-22.3 to 36.6)	-0.7 (-11.0 to 10.4)
Tajikistan	Prevalence Number: 1990	2,576,911 (2,557,142–2,596,098)	868,891 (820,006–929,734)	861,301 (835,290–887,362)	142,338 (117,461–168,542)	336,457 (323,429–349,152)	58,688 (54,838–62,048)	29,219 (24,176–36,058)	41,463 (21,532–68,851)	17,186 (13,060–21,954)	556 (224–961)	42,525 (36,186–51,200)
Tajikistan	Prevalence Number: 2015	3,478,035 (3,442,277–3,513,544)	1,129,926 (1,042,789–1,228,336)	1,211,912 (1,174,902–1,253,064)	168,154 (137,948–202,099)	433,846 (419,093–449,325)	39,216 (36,315–42,975)	48,455 (41,797–57,272)	44,120 (25,486–68,764)	24,747 (18,489–31,418)	6 (1–21)	71,213 (61,550–84,481)
Tajikistan	Prevalence Number: Cumulative change (%)	35.0 (33.3–36.6)	30.1 (20.5–39.4)	40.7 (34.8–46.7)	18.3 (6.9–31.3)	29.0 (22.5–35.9)	-33.2 (-37.6 to -27.9)	68.1 (30.4–110.8)	20.0 (-49.6 to 128.2)	47.2 (-0.3 to 101.9)	-98.0 (-99.8 to -95.1)	68.9 (36.8–102.3)
Tajikistan	Prevalence Rate: 1990	91,618.5 (90,976.1–92,237.9)	29,661.4 (28,123.2–31,576.1)	32,367.2 (31,396.8–33,278.3)	5,250.4 (4,342.6–6,214.6)	11,880.1 (11,427.8–12,326.4)	1,736.2 (1,627.4–1,831.8)	1,018.0 (843.2–1,259.7)	1,159.6 (602.2–1,925.5)	638.7 (484.6–811.5)	19.0 (7.7–32.8)	1,315.8 (1,106.3–1,620.6)
Tajikistan	Prevalence Rate: 2015	90,932.7 (90,059.9–91,823.6)	28,992.6 (26,931.9–31,272.4)	32,386.4 (31,405.1–33,475.4)	4,564.5 (3,736.4–5,495.6)	11,292.5 (10,909.0–11,699.0)	918.6 (853.2–1,002.8)	1,255.9 (1,081.4–1,484.8)	971.8 (561.4–1,514.7)	663.8 (494.7–842.0)	0.1 (0.0–0.5)	1,700.1 (1,458.3–2,036.7)
Tajikistan	Prevalence Rate: Cumulative change (%)	-0.7 (-1.9 to 0.4)	-2.2 (-8.5 to 4.0)	0.1 (-4.1 to 4.4)	-12.9 (-21.2 to -3.6)	-4.9 (-9.7 to 0.2)	-47.1 (-50.4 to -43.2)	25.1 (-3.2 to 57.0)	-5.5 (-60.3 to 79.7)	6.2 (-28.2 to 46.5)	-98.6 (-99.8 to -96.5)	30.6 (3.1–59.1)
Tajikistan	YLDs Number: 1990	168,635 (127,190–216,058)	33,279 (23,409–45,368)	18,261 (12,602–25,606)	6,460 (4,264–8,918)	5,593 (3,980–7,550)	9,590 (6,992–12,596)	1,981 (1,341–2,818)	5,173 (2,470–9,011)	6,233 (4,089–8,752)	11 (3–22)	3,544 (2,667–4,469)
Tajikistan	YLDs Number: 2015	213,917 (162,105–273,472)	42,467 (29,885–57,989)	25,251 (17,399–35,571)	7,646 (5,055–10,654)	6,702 (4,746–9,112)	6,423 (4,628–8,467)	3,449 (2,219–5,091)	5,504 (2,787–9,279)	7,058 (4,613–9,874)	0 (0–0)	5,834 (4,496–7,363)
Tajikistan	YLDs Number: Cumulative change (%)	26.9 (21.7–31.6)	27.7 (14.8–38.3)	38.2 (35.1–41.2)	18.5 (7.1–32.1)	19.9 (10.2–30.2)	-33.0 (-38.0 to -27.2)	75.8 (39.3–133.1)	20.3 (-48.8 to 127.2)	16.2 (-21.6 to 59.5)	-96.9 (-99.6 to -91.5)	65.1 (47.7–83.0)
Tajikistan	YLDs Rate: 1990	6,101.9 (4,615.7–7,790.7)	1,136.8 (800.3–1,539.9)	668.6 (458.4–943.5)	238.2 (157.1–326.6)	195.8 (139.1–265.0)	283.6 (207.5–372.7)	69.1 (46.7–98.5)	144.7 (69.1–252.0)	231.5 (150.8–327.0)	0.4 (0.1–0.8)	121.1 (91.4–153.5)
Tajikistan	YLDs Rate: 2015	5,659.7 (4,291.7–7,225.6)	1,104.1 (776.7–1,500.3)	667.7 (459.3–946.5)	207.5 (136.0–288.9)	176.6 (125.0–240.0)	150.4 (108.4–198.1)	89.3 (57.4–131.8)	121.3 (61.4–204.4)	189.2 (123.6–264.4)	0.0 (0.0–0.0)	150.2 (115.7–189.1)
Tajikistan	YLDs Rate: Cumulative change (%)	-7.2 (-10.5 to -4.2)	-2.8 (-11.5 to 4.2)	-0.1 (-2.1 to 1.9)	-12.8 (-21.2 to -3.2)	-9.7 (-16.4 to -2.5)	-47.0 (-50.8 to -42.5)	30.6 (3.6–73.4)	-5.2 (-59.7 to 79.0)	-16.1 (-43.7 to 15.0)	-97.7 (-99.7 to -93.6)	24.4 (11.3–38.3)
Turkmenistan	Prevalence Number: 1990	1,666,552 (1,649,803–1,683,297)	527,857 (486,187–558,682)	580,411 (562,708–598,324)	122,841 (102,477–145,931)	176,663 (167,105–188,508)	29,690 (26,402–32,297)	19,176 (15,804–23,716)	18,199 (9,180–31,123)	10,165 (7,717–12,900)	33 (11–58)	24,171 (20,404–29,270)
Turkmenistan	Prevalence Number: 2015	1,782,263 (1,764,189–1,797,408)	523,283 (478,531–585,263)	657,783 (637,400–679,754)	97,998 (80,608–117,180)	177,302 (167,712–189,162)	14,161 (13,033–15,314)	25,869 (22,384–30,331)	12,570 (5,964–21,029)	10,863 (8,074–14,149)	3 (0–12)	24,352 (20,682–28,680)
Turkmenistan	Prevalence Number: Cumulative change (%)	6.9 (5.5–8.3)	-0.6 (-12.3 to 15.3)	13.4 (8.9–18.0)	-20.1 (-29.3 to -10.2)	0.5 (-6.8 to 8.6)	-52.2 (-56.3 to -47.7)	36.7 (7.1–70.2)	-20.3 (-71.2 to 71.5)	9.1 (-26.5 to 53.1)	-87.9 (-97.9 to -64.5)	1.7 (-18.7 to 21.8)
Turkmenistan	Prevalence Rate: 1990	90,422.5 (89,572.6–91,278.1)	27,938.9 (25,893.9–29,465.4)	32,665.4 (31,680.2–33,666.1)	6,816.0 (5,708.3–8,079.4)	9,529.9 (9,012.2–10,173.9)	1,408.4 (1,257.1–1,529.7)	1,025.1 (844.7–1,267.1)	833.7 (420.6–1,425.8)	560.3 (426.4–713.1)	1.7 (0.6–3.1)	1,185.5 (992.0–1,454.6)
Turkmenistan	Prevalence Rate: 2015	89,500.8 (88,592.5–90,256.7)	26,233.1 (24,018.7–29,323.4)	33,057.1 (32,034.8–34,160.7)	4,942.1 (4,065.1–5,908.6)	8,897.6 (8,415.6–9,491.7)	703.5 (647.2–760.1)	1,297.6 (1,122.9–1,521.4)	618.5 (293.4–1,034.6)	546.1 (406.1–711.5)	0.2 (0.0–0.6)	1,211.6 (1,028.5–1,428.4)
Turkmenistan	Prevalence Rate: Cumulative change (%)	-1.0 (-2.3 to 0.2)	-5.9 (-16.5 to 8.3)	1.2 (-2.7 to 5.5)	-27.4 (-35.7 to -18.4)	-6.5 (-13.3 to 1.0)	-50.0 (-54.1 to -45.4)	28.3 (0.5–60.3)	-14.4 (-69.1 to 84.2)	-0.5 (-32.6 to 39.5)	-88.3 (-98.0 to -65.7)	3.4 (-19.3 to 24.6)
Turkmenistan	YLDs Number: 1990	106,054 (80,182–134,930)	19,910 (13,919–27,029)	12,116 (8,300–17,047)	5,583 (3,704–7,670)	2,367 (1,685–3,207)	4,867 (3,502–6,518)	1,226 (832–1,717)	2,268 (1,006–4,010)	3,697 (2,428–5,146)	0 (0–1)	2,166 (1,651–2,746)
Turkmenistan	YLDs Number: 2015	106,309 (80,349–135,713)	19,366 (13,517–26,814)	13,487 (9,209–19,109)	4,457 (2,940–6,124)	2,326 (1,648–3,161)	2,324 (1,640–3,091)	1,822 (1,218–2,640)	1,578 (699–2,879)	2,717 (1,701–3,923)	0 (0–0)	2,370 (1,803–3,014)
Turkmenistan	YLDs Number: Cumulative change (%)	0.3 (-4.5 to 5.5)	-2.2 (-17.6 to 17.6)	11.2 (8.6–13.8)	-20.0 (-29.5 to -9.8)	-1.4 (-13.0 to 15.5)	-52.1 (-56.6 to -47.2)	49.4 (25.4–88.5)	-19.9 (-71.4 to 71.1)	-24.9 (-50.6 to 6.4)	-82.0 (-96.6 to -49.6)	9.6 (-0.9 to 20.8)
Turkmenistan	YLDs Rate: 1990	5,838.4 (4,417.5–7,431.7)	1,053.8 (739.6–1,425.7)	670.3 (458.0–947.3)	309.7 (207.3–425.2)	127.3 (90.8–171.7)	230.8 (166.1–308.9)	65.5 (44.6–92.0)	103.9 (46.1–183.7)	203.6 (134.2–283.1)	0.0 (0.0–0.1)	114.3 (86.9–145.1)
Turkmenistan	YLDs Rate: 2015	5,342.7 (4,039.3–6,817.0)	972.4 (679.9–1,343.9)	677.5 (462.6–960.2)	224.8 (148.2–308.8)	116.9 (82.9–158.8)	115.4 (81.5–153.5)	91.4 (61.1–132.4)	77.6 (34.4–141.7)	136.6 (85.5–197.3)	0.0 (0.0–0.0)	118.8 (90.4–151.2)
Turkmenistan	YLDs Rate: Cumulative change (%)	-8.5 (-12.5 to -4.0)	-7.3 (-21.3 to 10.5)	1.1 (-1.0 to 3.2)	-27.3 (-35.9 to -18.2)	-7.9 (-18.2 to 7.3)	-49.9 (-54.4 to -44.8)	40.3 (17.8–76.9)	-13.9 (-69.2 to 83.9)	-31.4 (-55.0 to -2.7)	-82.6 (-96.8 to -50.8)	4.2 (-6.1 to 14.8)
Uzbekistan	Prevalence Number: 1990	9,395,490 (9,296,833–9,485,738)	3,179,847 (3,052,467–3,318,638)	3,253,291 (3,150,416–3,351,711)	479,504 (395,857–569,859)	937,611 (893,139–983,580)	136,945 (118,347–157,793)	136,914 (116,697–165,058)	144,797 (79,606–231,367)	55,817 (41,355–70,762)	369 (88–692)	187,854 (164,515–219,754)
Uzbekistan	Prevalence Number: 2015	9,973,369 (9,842,298–10,091,125)	2,944,169 (2,671,987–3,302,681)	3,631,865 (3,518,038–3,741,388)	489,245 (407,168–582,295)	916,965 (865,874–958,993)	60,225 (54,349–66,172)	157,035 (135,206–187,335)	71,294 (35,657–123,079)	61,818 (46,874–78,087)	0 (0–0)	185,733 (161,943–217,848)
Uzbekistan	Prevalence Number: Cumulative change (%)	6.2 (4.6–7.7)	-7.4 (-15.7 to 3.2)	11.7 (7.0–16.1)	2.5 (-12.6 to 17.3)	-2.1 (-9.0 to 4.5)	-55.7 (-62.4 to -48.5)	15.9 (-8.7 to 44.9)	-45.0 (-78.6 to 9.8)	13.4 (-20.6 to 55.7)	-98.4 (-100.0 to -100.0)	-0.6 (-16.1 to 17.6)
Uzbekistan	Prevalence Rate: 1990	90,033.8 (89,158.6–90,855.0)	29,714.0 (28,738.1–30,925.7)	32,444.0 (31,392.3–33,403.4)	4,804.6 (3,963.9–5,713.3)	8,939.9 (8,515.2–9,382.3)	1,145.3 (993.8–1,314.0)	1,298.7 (1,106.9–1,567.7)	1,164.6 (640.3–1,860.9)	555.6 (410.9–704.2)	3.4 (0.8–6.4)	1,665.9 (1,448.9–1,969.5)
Uzbekistan	Prevalence Rate: 2015	89,078.3 (87,948.9–90,080.6)	26,144.7 (23,951.0–29,103.2)	32,785.0 (31,764.4–33,784.8)	4,470.9 (3,703.3–5,321.5)	8,177.0 (7,723.8–8,551.3)	509.3 (461.6–557.9)	1,396.6 (1,202.8–1,667.7)	579.5 (289.8–1,000.5)	560.6 (424.0–711.9)	0.0 (0.0–0.0)	1,601.3 (1,391.2–1,885.8)
Uzbekistan	Prevalence Rate: Cumulative change (%)	-1.1 (-2.5 to 0.3)	-12.0 (-19.2 to -2.6)	1.1 (-3.1 to 5.1)	-6.5 (-20.8 to 7.0)	-8.5 (-14.9 to -2.3)	-55.2 (-61.7 to -48.2)	8.7 (-14.3 to 35.9)	-44.4 (-78.4 to 11.0)	3.3 (-27.9 to 41.8)	-98.4 (-100.0 to -100.0)	-3.3 (-19.0 to 15.2)
Uzbekistan	YLDs Number: 1990	638,483 (478,296–813,159)	120,484 (85,860–163,148)	68,276 (46,939–95,631)	21,758 (14,446–30,210)	11,825 (8,472–15,981)	22,451 (15,751–30,041)	9,694 (6,368–14,297)	18,074 (8,811–31,528)	20,260 (12,977–27,840)	7 (1–15)	19,852 (15,450–25,092)
Uzbekistan	YLDs Number: 2015	626,111 (474,920–794,250)	109,660 (76,415–149,864)	75,093 (51,187–106,754)	22,250 (14,854–30,585)	11,423 (8,055–15,471)	9,888 (7,033–13,219)	12,232 (7,668–19,355)	8,935 (4,065–16,222)	15,669 (10,203–22,585)	0 (0–0)	18,753 (14,491–23,784)
Uzbekistan	YLDs Number: Cumulative change (%)	-1.8 (-10.9 to 6.4)	-9.0 (-18.8 to 5.8)	9.9 (7.5–12.3)	2.7 (-12.4 to 17.8)	-3.4 (-12.2 to 8.0)	-55.7 (-62.6 to -48.0)	27.7 (-9.5 to 83.7)	-44.8 (-78.5 to 9.6)	-20.8 (-46.6 to 13.0)	-98.4 (-100.0 to -100.0)	-5.5 (-13.3 to 2.4)
Uzbekistan	YLDs Rate: 1990	6,173.7 (4,642.7–7,839.5)	1,127.3 (805.4–1,525.9)	667.7 (458.3–943.3)	217.9 (145.5–302.6)	112.4 (80.2–151.8)	187.7 (132.0–250.6)	91.9 (60.3–135.8)	145.4 (70.9–253.6)	201.6 (129.1–277.6)	0.1 (0.0–0.1)	186.7 (145.2–235.5)
Uzbekistan	YLDs Rate: 2015	5,617.1 (4,263.6–7,128.8)	984.0 (687.9–1,343.3)	674.1 (458.2–960.0)	203.3 (135.7–279.2)	103.6 (73.5–140.2)	83.6 (59.6–111.9)	108.7 (68.3–172.0)	72.6 (33.0–131.9)	142.1 (92.3–205.3)	0.0 (0.0–0.0)	166.5 (129.0–211.2)
Uzbekistan	YLDs Rate: Cumulative change (%)	-8.9 (-16.2 to -2.4)	-12.8 (-20.8 to 0.2)	0.9 (-1.2 to 3.0)	-6.3 (-20.8 to 7.6)	-7.8 (-15.3 to 1.9)	-55.2 (-62.1 to -47.7)	19.8 (-15.4 to 72.2)	-44.2 (-78.2 to 10.8)	-27.9 (-51.6 to 2.9)	-98.5 (-100.0 to -100.0)	-10.8 (-18.3 to -3.2)
Latin America and Caribbean	Prevalence Number: 1990	175,583,561 (175,335,188–175,833,887)	49,819,918 (49,100,023–50,533,613)	63,370,507 (62,979,913–63,772,326)	27,991,212 (23,769,357–32,375,150)	28,502,120 (28,116,538–28,900,469)	2,467,434 (2,313,511–2,630,098)	2,190,729 (2,087,849–2,288,895)	758,993 (712,502–808,862)	857,259 (772,715–950,856)	655,115 (444,643–955,364)	1,751,619 (1,581,883–1,935,902)
Latin America and Caribbean	Prevalence Number: 2015	182,004,058 (181,724,458–182,323,111)	50,978,951 (50,344,837–51,606,718)	68,345,973 (67,939,597–68,790,945)	23,990,262 (20,507,635–27,701,058)	25,477,453 (25,079,456–25,863,867)	1,540,046 (1,450,982–1,637,672)	2,763,503 (2,648,283–2,871,846)	394,588 (365,175–425,983)	766,995 (675,079–873,552)	389,121 (203,870–683,711)	1,848,936 (1,687,155–2,033,634)
Latin America and Caribbean	Prevalence Number: Cumulative change (%)	3.7 (3.5–3.8)	2.3 (0.2–4.2)	7.9 (7.2–8.5)	-14.3 (-17.7 to -10.7)	-10.6 (-12.0 to -9.2)	-37.6 (-39.8 to -35.4)	26.2 (20.4–32.0)	-47.9 (-53.2 to -42.3)	-10.5 (-17.3 to -3.5)	-42.3 (-57.3 to -26.2)	5.6 (1.2–10.0)

© 2017 Global Burden of Disease Child and Adolescent Health Collaboration. JAMA Pediatrics.

eTable 3. Prevalent Cases, Rates (per 100,000 population), Years Lived with Disability (YLDs), and Cumulative Percent Change with 95% Uncertainty Interval (UI) for the Top 10 Global Causes of YLDs in Children and Adolescents in 195 Countries and Territories, Aged 0 to 19 Years, Both Sexes, 1990 and 2015. *Note: best viewed by enlarging in browser.*

Location	Measure	All causes	Iron-deficiency anemia	Skin and subcutaneous diseases	Asthma	Hemoglobinopathies and hemolytic anemias	Diarrheal diseases	Congenital anomalies	Protein-energy malnutrition	Epilepsy	Malaria	Neonatal preterm birth complications
Latin America and Caribbean	Prevalence Rate: 1990	92,350.9 (92,224.2–92,476.3)	26,028.5 (25,661.7–26,394.3)	33,592.4 (33,389.1–33,805.6)	14,634.5 (12,431.2–16,904.7)	14,973.5 (14,770.3–15,183.1)	1,262.0 (1,183.7–1,344.8)	1,149.9 (1,095.8–1,201.3)	385.7 (362.0–411.0)	451.5 (407.3–500.6)	335.3 (228.0–488.4)	909.2 (820.3–1,004.6)
Latin America and Caribbean	Prevalence Rate: 2015	91,214.3 (91,066.6–91,380.5)	25,895.7 (25,567.9–26,231.8)	33,817.3 (33,625.0–34,045.0)	12,039.7 (10,249.8–13,937.5)	12,795.6 (12,593.8–12,989.5)	816.9 (768.8–869.6)	1,390.4 (1,332.3–1,445.1)	213.9 (198.0–231.0)	384.3 (337.7–436.9)	204.2 (106.6–361.5)	956.8 (873.4–1,050.9)
Latin America and Caribbean	Prevalence Rate: Cumulative change (%)	-1.2 (-1.4 to -1.1)	-0.5 (-2.5 to 1.4)	0.7 (0.1–1.3)	-17.7 (-20.9 to -14.5)	-14.5 (-15.9 to -13.2)	-35.3 (-37.5 to -33.1)	21.0 (15.5–26.5)	-44.4 (-50.1 to -38.4)	-14.9 (-21.3 to -8.1)	-40.9 (-56.5 to -24.0)	5.3 (0.9–9.6)
Latin America and Caribbean	YLDs Number: 1990	11,574,712 (8,766,192–14,692,707)	1,884,745 (1,343,753–2,534,783)	1,469,861 (1,018,649–2,043,666)	1,269,780 (852,715–1,748,506)	167,000 (121,306–221,442)	403,510 (292,546–531,863)	184,810 (127,443–254,559)	94,477 (64,996–126,572)	324,865 (235,570–417,207)	19,577 (12,158–28,952)	199,675 (156,942–250,705)
Latin America and Caribbean	YLDs Number: 2015	11,496,105 (8,726,139–14,570,193)	1,937,161 (1,386,878–2,612,676)	1,560,442 (1,079,050–2,180,794)	1,089,632 (724,224–1,496,019)	166,022 (121,394–221,174)	252,010 (181,253–334,230)	264,024 (184,739–363,842)	49,236 (33,824–66,881)	199,216 (142,655–259,107)	12,204 (6,258–21,065)	205,634 (160,780–258,946)
Latin America and Caribbean	YLDs Number: Cumulative change (%)	-0.7 (-1.6 to 0.4)	2.8 (0.3–5.2)	6.1 (5.1–7.2)	-14.2 (-17.6 to -10.5)	-0.6 (-2.7 to 1.7)	-37.5 (-39.7 to -35.3)	43.1 (30.1–56.1)	-47.8 (-53.2 to -42.2)	-38.7 (-44.7 to -32.5)	-38.9 (-54.6 to -18.4)	3.0 (-0.8 to 6.9)
Latin America and Caribbean	YLDs Rate: 1990	6,119.1 (4,633.6–7,765.6)	980.9 (699.4–1,318.7)	774.9 (537.1–1,078.2)	663.8 (445.4–913.1)	87.3 (63.4–115.7)	206.4 (149.6–272.0)	97.0 (66.9–133.6)	48.0 (33.0–64.3)	171.1 (124.3–219.3)	10.0 (6.2–14.8)	104.6 (82.2–131.3)
Latin America and Caribbean	YLDs Rate: 2015	5,701.7 (4,329.1–7,230.8)	986.1 (705.9–1,330.1)	778.9 (538.5–1,086.8)	546.9 (363.5–750.7)	83.5 (61.0–111.2)	133.7 (96.2–177.5)	132.9 (93.0–183.2)	26.7 (18.3–36.3)	99.9 (71.5–130.0)	6.4 (3.3–11.0)	103.6 (80.9–130.4)
Latin America and Caribbean	YLDs Rate: Cumulative change (%)	-6.8 (-7.7 to -5.9)	0.6 (-1.9 to 2.9)	0.5 (-0.1 to 1.1)	-17.6 (-20.8 to -14.5)	-4.4 (-6.5 to -2.2)	-35.2 (-37.4 to -32.9)	37.3 (24.7–49.8)	-44.3 (-50.1 to -38.3)	-41.6 (-47.3 to -35.8)	-37.6 (-54.0 to -16.3)	-1.0 (-4.6 to 2.8)
Central Latin America	Prevalence Number: 1990	77,424,940 (77,258,289–77,589,356)	17,790,564 (17,363,493–18,264,230)	27,170,194 (26,946,699–27,434,158)	10,032,266 (8,376,308–11,764,403)	11,370,379 (11,093,687–11,645,489)	869,343 (806,384–936,444)	1,049,757 (989,441–1,113,469)	409,823 (383,129–440,196)	467,987 (417,932–523,044)	253,744 (166,026–405,033)	495,749 (444,516–549,564)
Central Latin America	Prevalence Number: 2015	83,994,289 (83,785,940–84,207,612)	20,274,661 (19,844,414–20,737,707)	30,778,797 (30,482,157–31,057,823)	9,302,241 (7,778,219–10,923,595)	10,040,751 (9,778,145–10,311,528)	548,915 (511,923–587,723)	1,353,276 (1,285,926–1,425,702)	189,979 (171,043–211,909)	431,697 (373,978–496,706)	144,994 (74,334–252,968)	539,148 (488,975–597,046)
Central Latin America	Prevalence Number: Cumulative change (%)	8.5 (8.2–8.8)	14.0 (9.8–18.0)	13.3 (12.1–14.4)	-7.2 (-11.0 to -3.1)	-11.7 (-14.3 to -9.2)	-36.8 (-39.6 to -34.1)	29.0 (20.9–37.1)	-53.6 (-58.7 to -47.2)	-7.7 (-17.3 to 2.6)	-43.7 (-59.1 to -23.2)	8.8 (3.2–14.3)
Central Latin America	Prevalence Rate: 1990	91,299.2 (91,110.8–91,494.8)	20,817.9 (20,336.4–21,355.6)	32,296.2 (32,027.0–32,606.1)	11,802.0 (9,879.8–13,836.2)	13,381.9 (13,057.3–13,704.4)	985.9 (915.0–1,061.3)	1,234.3 (1,163.4–1,309.2)	461.0 (430.9–495.1)	552.5 (493.7–617.8)	291.3 (191.7–464.3)	572.8 (513.7–635.6)
Central Latin America	Prevalence Rate: 2015	89,808.1 (89,578.9–90,042.2)	21,844.5 (21,365.5–22,364.5)	32,582.1 (32,274.5–32,881.9)	9,966.6 (8,335.4–11,729.2)	10,760.3 (10,478.3–11,049.7)	615.5 (573.3–659.7)	1,452.2 (1,379.9–1,529.7)	216.1 (194.5–241.0)	461.5 (400.6–531.6)	160.5 (81.7–280.9)	591.4 (536.1–655.1)
Central Latin America	Prevalence Rate: Cumulative change (%)	-1.6 (-1.9 to -1.3)	5.0 (1.2–8.6)	0.9 (-0.2 to 1.9)	-15.5 (-18.8 to -12.1)	-19.6 (-22.0 to -17.3)	-37.5 (-40.3 to -34.9)	17.8 (10.3–25.1)	-53.0 (-58.3 to -46.6)	-16.4 (-25.1 to -7.1)	-45.8 (-60.9 to -25.3)	3.3 (-2.1 to 8.6)
Central Latin America	YLDs Number: 1990	4,688,056 (3,555,386–5,934,498)	638,299 (452,791–864,473)	633,194 (438,767–875,518)	456,227 (301,322–630,747)	85,291 (62,114–111,928)	142,880 (103,255–188,078)	93,811 (64,891–127,891)	51,257 (35,498–68,495)	178,025 (127,321–229,646)	7,360 (4,357–11,215)	59,608 (46,606–74,576)
Central Latin America	YLDs Number: 2015	4,890,942 (3,700,293–6,196,946)	755,908 (541,363–1,022,231)	706,785 (489,273–983,600)	423,348 (277,599–582,839)	91,052 (67,124–120,640)	90,295 (64,682–120,032)	130,554 (91,637–177,966)	23,811 (16,174–32,315)	113,023 (80,032–148,182)	4,853 (2,161–9,196)	61,239 (47,764–77,299)
Central Latin America	YLDs Number: Cumulative change (%)	4.3 (3.1–5.7)	18.5 (13.2–23.8)	11.6 (10.5–12.7)	-7.2 (-11.1 to -3.0)	6.8 (3.4–10.8)	-36.8 (-39.6 to -34.0)	39.3 (28.0–51.2)	-53.5 (-58.9 to -47.0)	-36.5 (-43.8 to -28.3)	-35.7 (-58.4 to -3.5)	2.7 (-1.5 to 7.4)
Central Latin America	YLDs Rate: 1990	5,560.1 (4,213.5–7,037.2)	746.2 (529.5–1,010.4)	748.6 (519.2–1,037.3)	536.7 (354.5–743.1)	100.5 (73.2–132.0)	162.0 (117.1–213.3)	110.3 (76.2–150.3)	57.7 (39.9–77.1)	210.1 (150.6–270.9)	8.5 (5.0–12.9)	70.0 (54.7–87.6)
Central Latin America	YLDs Rate: 2015	5,182.4 (3,917.2–6,565.9)	814.1 (583.3–1,099.7)	753.3 (521.3–1,046.1)	453.6 (297.9–623.4)	97.3 (71.8–129.0)	101.3 (72.5–134.6)	140.2 (98.5–191.1)	27.1 (18.4–36.8)	120.9 (85.4–158.6)	5.4 (2.4–10.2)	65.8 (51.3–83.1)
Central Latin America	YLDs Rate: Cumulative change (%)	-6.8 (-7.9 to -5.6)	9.2 (4.4–14.1)	0.6 (-0.1 to 1.4)	-15.5 (-18.8 to -11.8)	-3.2 (-6.2 to 0.4)	-37.5 (-40.3 to -34.8)	27.3 (16.9–38.2)	-53.0 (-58.4 to -46.4)	-42.4 (-49.1 to -35.2)	-38.4 (-60.2 to -7.6)	-6.1 (-10.0 to -1.7)
Colombia	Prevalence Number: 1990	14,925,645 (14,818,967–15,023,119)	2,345,949 (2,020,295–2,701,277)	5,262,000 (5,119,475–5,410,742)	2,116,866 (1,748,795–2,520,889)	3,254,711 (3,052,471–3,435,733)	201,487 (185,393–217,589)	198,164 (167,990–236,892)	43,578 (31,958–56,771)	90,828 (67,783–114,896)	132,687 (97,121–168,903)	100,590 (86,774–118,959)
Colombia	Prevalence Number: 2015	14,755,417 (14,656,648–14,847,165)	2,561,615 (2,300,897–2,775,263)	5,401,767 (5,243,985–5,552,077)	2,262,298 (1,864,048–2,744,499)	2,819,671 (2,667,170–2,975,937)	123,619 (115,620–131,703)	204,113 (171,749–243,375)	20,724 (13,815–29,744)	78,320 (57,252–102,199)	64,888 (30,214–134,317)	90,606 (78,499–105,272)
Colombia	Prevalence Number: Cumulative change (%)	-1.1 (-2.1 to -0.3)	10.2 (-10.6 to 33.4)	2.7 (-1.1 to 6.6)	7.0 (-3.7 to 19.3)	-13.3 (-19.2 to -7.0)	-38.6 (-43.0 to -34.3)	4.1 (-19.2 to 30.0)	-50.9 (-70.9 to -25.0)	-11.8 (-40.4 to 22.5)	-51.8 (-75.4 to -9.8)	-9.5 (-22.4 to 4.8)
Colombia	Prevalence Rate: 1990	93,232.1 (92,579.0–93,822.2)	14,537.2 (12,566.2–16,684.4)	33,226.8 (32,328.2–34,154.6)	13,105.7 (10,839.4–15,565.8)	20,302.5 (19,036.9–21,436.3)	1,214.1 (1,117.8–1,310.8)	1,235.2 (1,048.0–1,476.5)	260.9 (191.3–339.8)	569.1 (424.4–719.8)	798.9 (586.2–1,015.8)	619.5 (533.8–733.8)
Colombia	Prevalence Rate: 2015	93,002.6 (92,348.6–93,594.3)	16,598.1 (14,841.4–18,070.7)	33,511.8 (32,526.2–34,440.3)	14,282.1 (11,762.9–17,387.1)	17,831.5 (16,869.2–18,813.8)	841.4 (785.2–897.5)	1,291.8 (1,088.1–1,538.8)	144.8 (96.5–207.7)	493.3 (361.9–642.9)	430.6 (197.6–898.9)	596.2 (518.4–688.4)
Colombia	Prevalence Rate: Cumulative change (%)	-0.2 (-1.2 to 0.6)	15.2 (-7.0 to 39.2)	0.9 (-2.9 to 4.8)	9.1 (-1.6 to 21.6)	-12.1 (-18.1 to -5.7)	-30.6 (-35.7 to -25.8)	5.6 (-17.9 to 32.0)	-42.7 (-66.1 to -12.5)	-11.3 (-40.1 to 23.3)	-46.9 (-73.1 to 0.5)	-3.2 (-17.1 to 11.7)
Colombia	YLDs Number: 1990	902,301 (683,383–1,145,004)	79,759 (52,594–112,845)	134,293 (94,808–183,693)	96,383 (63,040–131,720)	6,188 (4,274–8,529)	33,141 (23,799–43,693)	15,160 (10,404–21,331)	5,430 (3,288–7,929)	34,291 (22,234–48,399)	4,316 (2,604–6,467)	12,015 (9,285–15,049)
Colombia	YLDs Number: 2015	877,774 (668,597–1,116,644)	89,038 (62,200–122,270)	134,894 (94,423–184,852)	102,995 (68,182–144,049)	6,113 (4,329–8,406)	20,323 (14,604–26,954)	16,521 (11,005–23,212)	2,588 (1,453–4,153)	19,810 (12,684–28,391)	2,209 (883–5,008)	10,317 (7,989–12,988)
Colombia	YLDs Number: Cumulative change (%)	-2.7 (-6.3 to 1.1)	13.5 (-13.8 to 40.4)	0.4 (-1.8 to 2.5)	7.0 (-3.5 to 19.6)	-0.4 (-18.2 to 16.1)	-38.6 (-43.2 to -33.9)	9.0 (-2.6 to 22.4)	-50.9 (-71.5 to -22.4)	-40.9 (-61.1 to -16.0)	-49.2 (-75.7 to -2.3)	-14.0 (-22.0 to -5.6)
Colombia	YLDs Rate: 1990	5,690.2 (4,308.7–7,213.5)	493.1 (326.0–698.3)	840.9 (593.0–1,150.4)	596.6 (390.2–813.2)	38.3 (26.5–52.8)	199.7 (143.5–263.1)	94.5 (64.8–132.9)	32.5 (19.7–47.5)	214.8 (139.0–303.4)	26.0 (15.7–38.9)	74.8 (57.7–93.7)
Colombia	YLDs Rate: 2015	5,453.6 (4,151.2–6,941.0)	574.3 (399.3–791.4)	845.8 (592.5–1,159.6)	650.4 (429.3–910.5)	38.9 (27.5–53.5)	138.4 (99.6–183.6)	104.7 (69.7–147.0)	18.1 (10.1–29.0)	124.8 (80.1–178.7)	14.6 (5.8–33.1)	65.3 (50.6–82.4)
Colombia	YLDs Rate: Cumulative change (%)	-4.1 (-7.7 to -0.5)	18.3 (-10.2 to 46.1)	0.6 (-1.3 to 2.4)	9.1 (-1.5 to 21.9)	2.3 (-16.0 to 19.3)	-30.6 (-35.9 to -25.3)	10.9 (-1.0 to 24.7)	-42.7 (-66.8 to -9.5)	-40.5 (-60.8 to -15.3)	-44.4 (-73.7 to 7.8)	-12.5 (-20.7 to -4.2)
Costa Rica	Prevalence Number: 1990	1,239,545 (1,229,507–1,248,906)	341,731 (320,499–364,281)	421,595 (408,254–434,216)	196,109 (151,622–275,028)	179,103 (168,421–189,689)	11,091 (10,272–11,965)	18,637 (15,902–22,142)	2,709 (1,784–3,908)	6,453 (4,704–8,285)	45 (30–60)	9,597 (8,437–10,926)
Costa Rica	Prevalence Number: 2015	1,339,358 (1,326,689–1,350,293)	358,825 (330,154–394,373)	484,733 (469,995–499,016)	185,868 (154,396–220,892)	165,218 (154,736–175,282)	9,398 (8,804–10,002)	20,367 (17,626–24,211)	1,663 (1,073–2,376)	6,636 (4,865–8,638)	0 (0–0)	8,626 (7,597–9,926)
Costa Rica	Prevalence Number: Cumulative change (%)	8.1 (6.8–9.4)	5.3 (-8.9 to 20.1)	15.0 (10.6–19.4)	-3.3 (-32.2 to 13.9)	-7.6 (-14.9 to 0.0)	-15.2 (-19.1 to -11.0)	10.2 (-10.5 to 34.0)	-34.9 (-66.5 to 6.7)	5.3 (-28.6 to 47.5)	-99.0 (-99.3 to -98.6)	-9.8 (-20.8 to 2.2)
Costa Rica	Prevalence Rate: 1990	91,336.5 (90,639.8–91,996.6)	24,741.1 (23,249.9–26,266.8)	31,783.0 (30,802.6–32,729.5)	14,379.6 (11,169.5–20,180.2)	13,140.3 (12,354.7–13,914.2)	747.5 (693.0–805.1)	1,364.3 (1,163.4–1,620.3)	179.0 (117.9–258.1)	474.2 (346.6–608.8)	3.3 (2.3–4.4)	682.7 (598.7–779.6)
Costa Rica	Prevalence Rate: 2015	90,611.8 (89,712.4–91,383.0)	24,712.5 (22,632.4–27,335.9)	32,184.6 (31,203.3–33,134.6)	12,620.9 (10,457.6–15,072.6)	11,227.5 (10,515.1–11,908.8)	689.6 (645.4–734.9)	1,385.8 (1,201.2–1,644.7)	124.9 (80.6–178.4)	449.4 (330.4–584.0)	0.0 (0.0–0.0)	605.7 (534.8–694.3)
Costa Rica	Prevalence Rate: Cumulative change (%)	-0.8 (-2.0 to 0.4)	0.2 (-13.5 to 14.0)	1.3 (-2.6 to 5.2)	-10.5 (-37.4 to 5.3)	-14.5 (-21.1 to -7.4)	-7.7 (-11.8 to -3.2)	2.4 (-16.8 to 24.5)	-26.1 (-61.9 to 21.2)	-2.9 (-34.2 to 36.2)	-99.1 (-99.3 to -98.7)	-11.0 (-22.1 to 0.7)
Costa Rica	YLDs Number: 1990	74,082 (56,168–94,908)	12,617 (9,013–16,998)	9,444 (6,458–13,090)	8,908 (5,694–13,433)	807 (570–1,101)	1,826 (1,310–2,433)	1,624 (1,106–2,265)	338 (193–531)	2,440 (1,545–3,503)	0 (0–1)	1,207 (935–1,532)
Costa Rica	YLDs Number: 2015	79,686 (60,456–101,486)	13,248 (9,460–18,317)	10,531 (7,191–14,711)	8,456 (5,548–11,643)	811 (572–1,111)	1,547 (1,108–2,079)	2,050 (1,373–2,884)	209 (115–332)	1,674 (1,075–2,411)	0 (0–0)	975 (752–1,223)
Costa Rica	YLDs Number: Cumulative change (%)	7.6 (1.7–12.5)	5.4 (-10.1 to 21.8)	11.4 (8.8–14.0)	-3.3 (-32.3 to 14.1)	0.8 (-12.9 to 14.7)	-15.2 (-20.3 to -9.5)	26.6 (8.0–47.7)	-34.7 (-66.3 to 7.6)	-29.5 (-54.4 to 0.8)	-98.7 (-99.1 to -98.0)	-19.2 (-26.4 to -11.0)
Costa Rica	YLDs Rate: 1990	5,553.2 (4,220.7–7,108.8)	909.3 (649.2–1,226.4)	701.3 (478.2–976.3)	653.0 (418.1–985.7)	59.0 (41.6–80.8)	123.1 (88.3–164.0)	118.8 (80.9–165.9)	22.3 (12.8–35.1)	179.2 (113.6–256.6)	0.1 (0.0–0.1)	88.3 (68.4–112.0)
Costa Rica	YLDs Rate: 2015	5,312.2 (4,023.1–6,769.9)	919.2 (655.7–1,271.7)	707.8 (484.0–987.6)	574.3 (377.0–790.0)	55.8 (39.3–76.4)	113.6 (81.4–152.7)	139.6 (93.8–196.3)	15.7 (8.6–24.9)	113.5 (72.8–163.4)	0.0 (0.0–0.0)	66.4 (51.3–83.3)
Costa Rica	YLDs Rate: Cumulative change (%)	-4.3 (-9.7 to 0.0)	1.4 (-13.2 to 17.4)	0.9 (-1.0 to 2.8)	-10.4 (-37.4 to 5.2)	-5.2 (-18.3 to 8.0)	-7.7 (-13.2 to -1.3)	17.8 (0.7–37.5)	-25.8 (-61.7 to 22.3)	-35.0 (-57.3 to -6.8)	-98.7 (-99.2 to -98.1)	-24.8 (-31.5 to -17.2)
El Salvador	Prevalence Number: 1990	2,473,459 (2,455,873–2,490,532)	750,663 (704,143–809,127)	836,843 (810,347–861,152)	515,043 (427,264–610,572)	374,285 (338,167–407,119)	37,403 (34,129–39,910)	31,866 (26,887–38,416)	6,582 (5,097–8,332)	12,391 (9,018–16,184)	393 (272–523)	20,525 (17,736–24,137)
El Salvador	Prevalence Number: 2015	2,054,991 (2,038,528–2,071,344)	681,824 (657,934–703,422)	764,048 (739,736–785,234)	277,333 (230,096–329,316)	265,299 (238,926–293,038)	11,353 (10,409–12,457)	30,647 (26,521–36,060)	3,383 (2,296–4,788)	9,429 (6,730–12,400)	0 (0–0)	14,562 (12,609–17,278)
El Salvador	Prevalence Number: Cumulative change (%)	-16.9 (-17.7 to -16.1)	-9.0 (-16.0 to -3.4)	-8.7 (-12.3 to -5.1)	-46.0 (-51.7 to -38.8)	-28.9 (-37.5 to -19.8)	-69.6 (-72.2 to -66.5)	-2.9 (-22.9 to 17.7)	-47.5 (-66.9 to -22.3)	-22.0 (-48.6 to 9.6)	-99.9 (-99.9 to -99.9)	-28.6 (-40.1 to -15.3)
El Salvador	Prevalence Rate: 1990	92,077.3 (91,453.8–92,665.4)	28,014.0 (26,471.7–30,001.5)	31,648.1 (30,646.9–32,556.8)	19,008.3 (15,832.6–22,447.9)	13,895.3 (12,548.7–15,121.3)	1,280.2 (1,169.9–1,365.1)	1,178.4 (994.8–1,421.4)	222.5 (172.4–281.4)	463.4 (338.1–604.5)	14.4 (10.1–19.1)	734.0 (633.5–869.0)
El Salvador	Prevalence Rate: 2015	88,384.5 (87,638.4–89,105.6)	29,343.1 (28,195.6–30,487.3)	32,089.7 (31,085.3–32,985.3)	12,068.0 (9,986.2–14,388.3)	11,476.3 (10,336.3–12,680.4)	546.4 (499.7–600.8)	1,331.8 (1,152.9–1,565.0)	168.0 (113.9–237.9)	404.6 (289.7–533.0)	0.0 (0.0–0.0)	670.4 (581.3–789.8)

© 2017 Global Burden of Disease Child and Adolescent Health Collaboration. JAMA Pediatrics.

eTable 3. Prevalent Cases, Rates (per 100,000 population), Years Lived with Disability (YLDs), and Cumulative Percent Change with 95% Uncertainty Interval (UI) for the Top 10 Global Causes of YLDs in Children and Adolescents in 195 Countries and Territories, Aged 0 to 19 Years, Both Sexes, 1990 and 2015. *Note: best viewed by enlarging in browser.*

Location	Measure	All causes	Iron-deficiency anemia	Skin and subcutaneous diseases	Asthma	Hemoglobinopathies and hemolytic anemias	Diarrheal diseases	Congenital anomalies	Protein-energy malnutrition	Epilepsy	Malaria	Neonatal preterm birth complications
El Salvador	Prevalence Rate: Cumulative change (%)	-4.0 (-5.0 to -3.0)	4.9 (-2.9 to 11.1)	1.4 (-2.5 to 5.5)	-36.4 (-43.0 to -27.9)	-17.2 (-27.2 to -6.6)	-57.3 (-61.0 to -52.8)	14.1 (-9.5 to 38.2)	-22.9 (-51.4 to 14.0)	-10.5 (-41.1 to 25.9)	-99.9 (-99.9 to -99.9)	-8.0 (-22.7 to 8.2)
El Salvador	YLDs Number: 1990	167,277 (127,203–211,210)	29,590 (21,160–39,929)	19,640 (13,570–27,094)	23,366 (15,735–31,829)	1,617 (1,133–2,199)	6,140 (4,480–8,111)	2,414 (1,678–3,296)	820 (527–1,193)	4,628 (3,017–6,613)	8 (4–13)	2,280 (1,761–2,880)
El Salvador	YLDs Number: 2015	131,348 (99,554–167,010)	26,778 (18,921–35,978)	17,318 (11,944–24,151)	12,593 (8,289–17,457)	1,379 (984–1,868)	1,868 (1,332–2,494)	2,672 (1,857–3,746)	424 (249–656)	2,400 (1,475–3,405)	0 (0–0)	1,393 (1,072–1,763)
El Salvador	YLDs Number: Cumulative change (%)	-21.5 (-23.9 to -19.1)	-9.3 (-16.9 to -3.1)	-11.9 (-14.1 to -9.8)	-46.0 (-51.8 to -38.9)	-14.4 (-22.5 to -6.9)	-69.5 (-72.4 to -66.2)	10.9 (-2.9 to 28.9)	-47.1 (-67.3 to -20.4)	-46.7 (-65.7 to -23.5)	-99.9 (-99.9 to -99.9)	-38.8 (-45.0 to -31.8)
El Salvador	YLDs Rate: 1990	6,303.0 (4,789.1–7,939.3)	1,100.9 (789.3–1,487.9)	733.9 (506.5–1,016.1)	862.1 (581.0–1,170.7)	60.0 (42.1–81.3)	210.1 (153.5–277.8)	89.2 (62.0–121.8)	27.7 (17.8–40.4)	173.0 (112.5–246.5)	0.3 (0.2–0.5)	84.0 (64.8–106.2)
El Salvador	YLDs Rate: 2015	5,525.8 (4,178.8–7,021.0)	1,159.6 (821.5–1,553.6)	740.7 (512.2–1,029.6)	548.3 (359.8–761.1)	59.9 (42.7–81.2)	90.0 (64.1–120.4)	116.3 (80.9–163.0)	21.1 (12.4–32.6)	103.1 (63.1–146.8)	0.0 (0.0–0.0)	60.7 (46.8–77.0)
El Salvador	YLDs Rate: Cumulative change (%)	-12.3 (-15.0 to -9.8)	5.5 (-2.6 to 13.1)	0.9 (-1.0 to 2.8)	-36.3 (-43.0 to -27.9)	0.1 (-9.3 to 8.3)	-57.1 (-61.2 to -52.5)	30.6 (14.2–51.9)	-22.3 (-52.0 to 16.9)	-38.8 (-60.5 to -12.0)	-99.9 (-99.9 to -99.8)	-27.6 (-34.9 to -19.5)
Guatemala	Prevalence Number: 1990	5,000,860 (4,976,999–5,023,312)	1,535,061 (1,451,585–1,615,748)	1,608,324 (1,558,232–1,658,645)	709,664 (578,134–844,463)	718,733 (657,779–783,651)	104,832 (96,895–111,072)	56,695 (47,046–68,961)	21,220 (15,856–27,738)	25,076 (18,657–31,897)	29,101 (15,726–42,147)	29,127 (24,498–35,214)
Guatemala	Prevalence Number: 2015	7,195,592 (7,140,978–7,246,671)	2,344,808 (2,198,446–2,506,977)	2,521,731 (2,445,059–2,600,648)	748,016 (621,451–889,400)	894,300 (809,187–979,424)	100,740 (91,540–109,867)	100,038 (84,879–119,637)	18,529 (12,514–26,292)	34,474 (25,553–44,723)	36,914 (18,655–55,115)	44,266 (37,950–52,532)
Guatemala	Prevalence Number: Cumulative change (%)	43.9 (42.6–45.1)	53.0 (41.8–70.7)	56.8 (50.6–63.1)	5.7 (-6.1 to 16.5)	24.8 (9.5–41.2)	-3.9 (-11.3 to 2.7)	78.6 (38.3–125.3)	-10.0 (-45.4 to 40.7)	41.0 (-3.8 to 100.4)	26.2 (4.7–43.7)	53.3 (26.4–82.7)
Guatemala	Prevalence Rate: 1990	96,709.4 (96,299.6–97,087.5)	29,838.8 (28,327.2–31,374.5)	32,142.0 (31,182.1–33,174.6)	13,587.7 (11,115.2–16,106.3)	13,896.7 (12,721.7–15,147.5)	1,797.0 (1,665.8–1,902.6)	1,085.8 (901.4–1,319.3)	354.0 (265.0–462.0)	491.3 (365.3–624.9)	511.7 (279.5–733.8)	514.6 (432.0–633.6)
Guatemala	Prevalence Rate: 2015	92,388.8 (91,690.5–93,034.9)	30,087.3 (28,243.9–32,166.7)	32,475.3 (31,487.4–33,485.2)	9,613.3 (7,977.8–11,429.8)	11,475.6 (10,385.3–12,566.4)	1,271.0 (1,155.4–1,385.4)	1,282.8 (1,088.5–1,533.6)	232.4 (157.0–329.7)	443.1 (328.2–574.4)	469.2 (238.0–699.4)	560.9 (480.6–667.2)
Guatemala	Prevalence Rate: Cumulative change (%)	-4.5 (-5.3 to -3.7)	1.0 (-6.2 to 12.3)	1.1 (-3.0 to 5.0)	-29.1 (-36.9 to -22.2)	-17.2 (-27.3 to -6.4)	-29.3 (-34.7 to -24.4)	19.6 (-7.4 to 51.1)	-32.4 (-59.0 to 5.7)	-7.5 (-36.7 to 31.4)	-8.9 (-23.9 to 4.1)	10.1 (-10.4 to 32.2)
Guatemala	YLDs Number: 1990	340,136 (257,636–438,117)	59,749 (42,551–81,431)	39,917 (27,590–55,091)	32,054 (21,366–44,152)	3,380 (2,440–4,651)	17,163 (12,467–22,834)	4,780 (3,269–6,605)	2,627 (1,660–3,797)	9,888 (6,256–14,046)	981 (450–1,639)	2,535 (1,939–3,247)
Guatemala	YLDs Number: 2015	453,161 (343,557–575,328)	90,448 (63,974–122,329)	61,286 (42,084–84,953)	33,936 (22,384–46,404)	5,072 (3,567–6,909)	16,540 (11,801–22,094)	9,598 (6,608–13,391)	2,307 (1,359–3,621)	11,245 (7,185–15,936)	1,312 (567–2,199)	4,040 (3,107–5,113)
Guatemala	YLDs Number: Cumulative change (%)	33.3 (28.2–39.6)	51.7 (39.4–75.2)	53.5 (50.2–56.8)	6.1 (-5.6 to 16.9)	50.5 (33.6–72.7)	-3.6 (-11.5 to 3.7)	102.1 (73.4–135.5)	-9.4 (-44.9 to 41.7)	17.1 (-19.8 to 68.2)	34.2 (1.2–70.2)	59.7 (41.8–77.4)
Guatemala	YLDs Rate: 1990	6,737.9 (5,113.6–8,671.5)	1,152.3 (823.2–1,569.1)	779.8 (539.4–1,079.6)	613.4 (408.7–849.2)	65.7 (47.4–90.3)	294.0 (213.2–391.2)	91.3 (62.3–125.8)	43.8 (27.7–63.4)	193.5 (122.9–276.0)	17.4 (8.0–29.1)	48.0 (36.8–61.6)
Guatemala	YLDs Rate: 2015	5,834.6 (4,422.4–7,406.2)	1,159.8 (820.3–1,568.3)	787.6 (540.3–1,092.1)	436.1 (287.5–596.2)	65.0 (45.8–88.6)	208.7 (148.9–278.7)	123.0 (84.7–171.8)	28.9 (17.1–45.4)	144.5 (92.4–204.7)	16.7 (7.2–28.0)	51.8 (39.8–65.5)
Guatemala	YLDs Rate: Cumulative change (%)	-13.4 (-16.5 to -9.7)	0.8 (-7.1 to 16.0)	1.0 (-0.8 to 2.9)	-28.7 (-36.6 to -22.0)	-0.8 (-11.6 to 13.1)	-29.0 (-34.7 to -23.7)	35.6 (16.2–58.5)	-31.9 (-58.5 to 6.3)	-23.1 (-47.4 to 10.9)	-3.5 (-26.9 to 22.8)	8.1 (-4.2 to 20.3)
Honduras	Prevalence Number: 1990	2,605,567 (2,585,193–2,624,833)	852,953 (788,618–921,944)	890,623 (866,425–914,259)	538,848 (450,278–641,396)	385,775 (349,548–421,917)	50,964 (46,472–56,074)	32,555 (27,590–39,375)	8,327 (6,890–9,910)	17,358 (12,840–21,927)	8,189 (4,277–16,019)	19,757 (17,066–23,367)
Honduras	Prevalence Number: 2015	3,228,885 (3,209,056–3,247,194)	886,650 (858,606–929,942)	1,192,877 (1,158,717–1,225,404)	538,632 (444,234–636,592)	397,130 (359,699–440,570)	39,201 (35,402–43,162)	44,140 (37,776–52,388)	4,818 (3,545–6,403)	18,312 (13,484–23,735)	8,769 (4,897–16,270)	22,913 (19,962–27,056)
Honduras	Prevalence Number: Cumulative change (%)	23.9 (22.8–25.2)	4.2 (-5.2 to 16.5)	34.0 (29.3–39.0)	0.1 (-7.6 to 7.3)	3.2 (-9.7 to 17.8)	-23.0 (-28.8 to -17.2)	37.0 (7.7–70.4)	-41.4 (-59.1 to -18.6)	8.0 (-27.3 to 53.2)	8.9 (-8.2 to 23.7)	16.7 (-2.4 to 37.1)
Honduras	Prevalence Rate: 1990	94,495.0 (93,852.8–95,094.0)	30,572.1 (28,486.9–32,777.8)	33,268.9 (32,392.2–34,139.4)	19,331.3 (16,228.0–22,843.5)	13,945.2 (12,628.7–15,254.3)	1,614.4 (1,475.3–1,771.5)	1,168.5 (987.2–1,416.1)	255.9 (212.0–304.4)	639.1 (472.2–806.4)	272.1 (146.0–525.4)	659.8 (568.4–789.3)
Honduras	Prevalence Rate: 2015	92,237.8 (91,647.5–92,783.2)	25,555.1 (24,726.9–26,947.8)	33,595.3 (32,622.7–34,528.8)	15,465.9 (12,748.3–18,287.2)	11,372.5 (10,297.2–12,617.2)	1,206.6 (1,088.4–1,330.2)	1,267.5 (1,086.3–1,503.3)	151.6 (111.6–201.5)	520.6 (384.0–675.3)	263.2 (145.6–492.0)	683.1 (596.2–804.0)
Honduras	Prevalence Rate: Cumulative change (%)	-2.4 (-3.2 to -1.5)	-16.2 (-23.2 to -6.5)	1.0 (-2.5 to 4.7)	-19.9 (-25.8 to -14.6)	-18.2 (-28.5 to -6.7)	-25.2 (-30.8 to -19.6)	9.6 (-14.0 to 36.5)	-40.1 (-58.1 to -16.7)	-16.6 (-44.1 to 17.9)	-2.4 (-16.9 to 9.7)	4.3 (-13.4 to 22.7)
Honduras	YLDs Number: 1990	180,385 (136,341–229,306)	32,390 (22,727–43,708)	24,230 (17,116–32,613)	24,408 (16,416–33,302)	1,820 (1,294–2,445)	8,344 (6,030–11,245)	2,935 (1,878–4,406)	1,034 (672–1,433)	6,468 (4,167–9,074)	285 (129–589)	2,166 (1,667–2,741)
Honduras	YLDs Number: 2015	205,794 (156,356–261,817)	32,817 (23,533–43,911)	31,363 (22,087–42,748)	24,471 (16,365–34,015)	2,051 (1,441–2,788)	6,433 (4,648–8,625)	4,907 (3,178–7,220)	602 (359–905)	4,661 (2,981–6,695)	303 (141–592)	2,588 (1,991–3,320)
Honduras	YLDs Number: Cumulative change (%)	14.2 (10.0–18.5)	1.7 (-9.2 to 16.9)	29.4 (25.9–32.4)	0.3 (-7.6 to 7.8)	12.9 (1.2–27.5)	-22.8 (-29.2 to -16.5)	71.9 (11.6–140.5)	-41.1 (-59.0 to -17.4)	-26.1 (-52.2 to 6.8)	8.7 (-15.2 to 35.5)	19.7 (7.9–32.3)
Honduras	YLDs Rate: 1990	6,670.7 (5,045.8–8,458.2)	1,154.2 (815.0–1,555.2)	885.2 (626.5–1,195.4)	875.3 (588.4–1,193.0)	65.9 (47.2–87.9)	264.2 (191.0–355.8)	105.0 (67.4–157.1)	31.8 (20.7–44.1)	237.9 (152.2–334.6)	9.6 (4.4–19.6)	77.0 (59.5–97.2)
Honduras	YLDs Rate: 2015	5,816.4 (4,415.7–7,410.6)	946.9 (677.6–1,270.1)	893.1 (629.2–1,212.5)	702.8 (469.7–976.7)	58.6 (41.2–79.4)	198.1 (143.4–266.2)	141.1 (91.3–207.7)	18.9 (11.3–28.5)	132.6 (84.9–190.2)	9.0 (4.2–17.8)	74.5 (57.4–95.5)
Honduras	YLDs Rate: Cumulative change (%)	-12.8 (-15.7 to -9.7)	-17.7 (-25.9 to -5.5)	0.9 (-1.1 to 2.9)	-19.7 (-25.8 to -14.1)	-10.9 (-19.5 to 0.1)	-25.0 (-31.2 to -18.9)	38.1 (-10.4 to 93.8)	-39.7 (-58.0 to -15.5)	-42.8 (-62.7 to -16.8)	-3.9 (-24.1 to 19.5)	-3.2 (-12.6 to 7.3)
Mexico	Prevalence Number: 1990	39,060,175 (38,969,496–39,149,794)	8,146,243 (7,985,500–8,320,036)	13,971,431 (13,855,184–14,087,741)	4,062,134 (3,350,000–4,801,773)	4,650,371 (4,583,584–4,720,364)	330,045 (304,409–360,474)	546,725 (516,046–580,999)	268,291 (245,738–292,953)	252,196 (227,016–280,154)	13,059 (8,676–17,892)	232,860 (208,418–258,711)
Mexico	Prevalence Number: 2015	41,235,070 (41,124,989–41,354,219)	7,731,949 (7,579,517–7,888,277)	15,330,347 (15,201,747–15,448,089)	3,602,964 (3,008,795–4,237,522)	3,678,334 (3,623,212–3,732,504)	146,754 (136,191–158,640)	742,304 (706,103–783,461)	95,931 (87,310–105,735)	215,736 (189,152–245,069)	88 (62–116)	264,320 (239,390–293,219)
Mexico	Prevalence Number: Cumulative change (%)	5.6 (5.2–5.9)	-5.1 (-7.8 to -2.4)	9.7 (8.6–10.8)	-11.2 (-14.8 to -7.8)	-20.9 (-22.2 to -19.6)	-55.5 (-58.7 to -52.8)	35.9 (27.6–44.5)	-64.1 (-68.7 to -59.4)	-14.4 (-22.6 to -5.7)	-99.3 (-99.4 to -99.2)	13.6 (7.5–19.8)
Mexico	Prevalence Rate: 1990	89,107.0 (88,898.6–89,312.0)	18,589.1 (18,221.7–18,984.7)	31,839.4 (31,573.7–32,104.9)	9,300.8 (7,670.6–11,000.3)	10,606.1 (10,454.1–10,765.8)	749.1 (690.8–818.2)	1,246.8 (1,176.8–1,324.9)	608.4 (557.2–664.3)	575.0 (517.5–638.7)	29.8 (19.8–40.8)	528.4 (472.8–587.0)
Mexico	Prevalence Rate: 2015	87,386.8 (87,145.9–87,653.8)	16,626.2 (16,285.8–16,976.0)	32,138.8 (31,863.0–32,383.8)	7,652.0 (6,390.9–9,022.6)	7,824.4 (7,707.4–7,939.9)	327.4 (303.7–354.5)	1,580.1 (1,502.9–1,668.1)	217.2 (197.6–239.4)	458.1 (402.3–520.6)	0.2 (0.1–0.3)	574.9 (520.4–637.2)
Mexico	Prevalence Rate: Cumulative change (%)	-1.9 (-2.2 to -1.6)	-10.5 (-13.1 to -7.9)	0.9 (-0.1 to 1.9)	-17.7 (-20.8 to -14.6)	-26.2 (-27.5 to -25.0)	-56.2 (-59.4 to -53.6)	26.9 (19.1–34.9)	-64.2 (-68.8 to -59.5)	-20.3 (-27.8 to -12.1)	-99.4 (-99.5 to -99.2)	8.9 (3.2–14.7)
Mexico	YLDs Number: 1990	2,251,785 (1,700,729–2,854,017)	277,911 (195,154–376,610)	304,331 (208,744–426,527)	185,185 (121,947–257,440)	63,909 (46,866–83,802)	54,362 (39,055–72,260)	52,953 (36,453–72,682)	33,629 (23,093–45,286)	96,228 (70,149–122,907)	273 (152–431)	28,842 (22,611–36,143)
Mexico	YLDs Number: 2015	2,198,219 (1,667,753–2,789,216)	267,059 (189,639–363,110)	330,535 (227,765–464,438)	164,484 (107,660–229,939)	62,813 (46,226–83,116)	24,257 (17,263–32,020)	74,088 (51,638–102,500)	12,060 (8,344–16,298)	55,969 (39,442–73,600)	1 (1–3)	31,062 (24,028–39,266)
Mexico	YLDs Number: Cumulative change (%)	-2.4 (-3.5 to -1.3)	-3.9 (-7.2 to -0.7)	8.6 (7.2–9.9)	-11.1 (-14.6 to -7.6)	-1.7 (-4.9 to 2.4)	-55.3 (-58.6 to -52.5)	40.1 (27.3–56.6)	-64.0 (-68.7 to -59.1)	-41.8 (-48.3 to -34.6)	-99.3 (-99.4 to -99.1)	7.7 (1.9–14.0)
Mexico	YLDs Rate: 1990	5,133.9 (3,877.2–6,507.6)	635.1 (445.9–860.5)	694.1 (476.2–972.3)	424.0 (279.1–589.8)	146.2 (107.2–191.6)	123.4 (88.6–164.0)	120.8 (83.1–165.8)	76.3 (52.4–102.7)	219.4 (159.9–280.2)	0.6 (0.3–1.0)	65.8 (51.6–82.4)
Mexico	YLDs Rate: 2015	4,611.1 (3,500.9–5,845.7)	574.6 (407.9–782.0)	697.7 (480.8–980.0)	349.4 (228.8–487.2)	133.5 (98.2–176.6)	54.1 (38.6–71.4)	157.8 (110.0–218.4)	27.3 (18.9–36.9)	118.9 (83.9–156.2)	0.0 (0.0–0.0)	66.1 (51.2–83.7)
Mexico	YLDs Rate: Cumulative change (%)	-10.2 (-11.2 to -9.2)	-9.5 (-12.6 to -6.5)	0.5 (-0.7 to 1.7)	-17.5 (-20.7 to -14.4)	-8.7 (-11.6 to -4.8)	-56.1 (-59.3 to -53.3)	30.9 (18.9–46.3)	-64.1 (-68.7 to -59.2)	-45.8 (-51.9 to -39.0)	-99.3 (-99.4 to -99.2)	0.6 (-4.8 to 6.6)
Nicaragua	Prevalence Number: 1990	2,171,643 (2,154,070–2,190,646)	703,974 (657,425–777,361)	736,293 (711,838–759,790)	326,631 (268,550–389,802)	329,393 (298,829–361,199)	41,239 (37,619–44,030)	27,516 (23,329–33,160)	8,774 (6,382–11,962)	14,467 (10,731–18,245)	37,297 (6,550–155,120)	16,270 (14,142–19,148)
Nicaragua	Prevalence Number: 2015	2,153,557 (2,132,673–2,174,542)	637,453 (590,746–683,288)	799,223 (774,431–820,750)	229,085 (185,584–273,798)	278,019 (252,028–305,901)	20,930 (19,067–22,952)	31,188 (26,835–36,847)	3,146 (2,083–4,442)	10,420 (7,582–13,860)	9,116 (5,751–14,796)	15,931 (13,810–18,691)
Nicaragua	Prevalence Number: Cumulative change (%)	-0.8 (-2.1 to 0.3)	-9.3 (-16.9 to -3.9)	8.6 (4.3–12.9)	-29.8 (-37.1 to -21.5)	-15.4 (-25.4 to -4.6)	-49.2 (-53.4 to -45.0)	14.4 (-8.2 to 41.0)	-62.9 (-77.9 to -41.8)	-26.1 (-51.1 to 6.3)	-42.1 (-94.8 to -0.5)	-1.4 (-17.1 to 17.1)
Nicaragua	Prevalence Rate: 1990	92,257.2 (91,556.6–92,991.3)	29,455.6 (27,769.0–32,205.8)	32,146.5 (31,091.5–33,164.1)	13,593.5 (11,202.8–16,146.9)	13,941.5 (12,656.1–15,293.4)	1,566.6 (1,431.2–1,670.7)	1,159.0 (984.8–1,396.8)	327.5 (238.2–446.4)	616.5 (457.5–779.1)	1,444.6 (265.4–5,969.8)	654.3 (565.7–773.5)
Nicaragua	Prevalence Rate: 2015	88,141.3 (87,275.9–89,017.5)	26,217.2 (24,253.3–28,203.4)	32,508.2 (31,484.1–33,378.7)	9,373.7 (7,593.2–11,206.2)	11,405.2 (10,336.3–12,549.0)	893.7 (813.6–980.8)	1,280.8 (1,102.8–1,512.9)	135.5 (89.7–191.4)	426.2 (309.6–566.8)	378.9 (239.5–619.2)	672.3 (584.1–786.3)
Nicaragua	Prevalence Rate: Cumulative change (%)	-4.5 (-5.7 to -3.4)	-10.9 (-18.0 to -5.3)	1.2 (-2.8 to 5.1)	-31.0 (-38.0 to -23.1)	-18.0 (-27.8 to -7.5)	-42.9 (-47.7 to -38.2)	11.6 (-10.6 to 37.3)	-57.2 (-74.5 to -32.9)	-29.1 (-53.1 to 2.3)	-39.6 (-94.3 to 2.2)	3.5 (-13.5 to 22.9)
Nicaragua	YLDs Number: 1990	143,282 (108,930–182,549)	27,157 (19,473–36,477)	18,348 (12,657–25,427)	14,821 (9,715–20,308)	1,540 (1,085–2,071)	6,761 (4,880–8,954)	2,609 (1,783–3,577)	1,091 (668–1,615)	5,411 (3,395–7,703)	317 (162–493)	1,824 (1,405–2,287)

© 2017 Global Burden of Disease Child and Adolescent Health Collaboration. JAMA Pediatrics.

eTable 3. Prevalent Cases, Rates (per 100,000 population), Years Lived with Disability (YLDs), and Cumulative Percent Change with 95% Uncertainty Interval (UI) for the Top 10 Global Causes of YLDs in Children and Adolescents in 195 Countries and Territories, Aged 0 to 19 Years, Both Sexes, 1990 and 2015. *Note: best viewed by enlarging in browser.*

Location	Measure	All causes	Iron-deficiency anemia	Skin and subcutaneous diseases	Asthma	Hemoglobinopathies and hemolytic anemias	Diarrheal diseases	Congenital anomalies	Protein-energy malnutrition	Epilepsy	Malaria	Neonatal preterm birth complications
Nicaragua	YLDs Number: 2015	130,811 (99,306–166,618)	23,889 (16,868–32,671)	19,335 (13,289–26,763)	10,422 (6,866–14,652)	1,448 (1,018–1,966)	3,446 (2,469–4,597)	3,824 (2,615–5,362)	393 (225–622)	2,663 (1,642–3,876)	89 (59–130)	1,632 (1,268–2,060)
Nicaragua	YLDs Number: Cumulative change (%)	-8.7 (-11.8 to -5.9)	-11.9 (-21.3 to -6.4)	5.3 (2.9–7.8)	-29.6 (-37.3 to -21.1)	-5.8 (-15.4 to 3.1)	-49.0 (-53.5 to -44.4)	47.4 (19.6–79.9)	-62.8 (-78.1 to -40.9)	-49.4 (-67.0 to -26.5)	-69.7 (-80.5 to -50.7)	-10.3 (-19.5 to -0.5)
Nicaragua	YLDs Rate: 1990	6,172.9 (4,692.9–7,842.7)	1,126.2 (809.9–1,507.4)	784.0 (540.7–1,088.0)	616.7 (404.1–840.2)	64.5 (45.3–86.8)	256.8 (185.4–339.6)	109.7 (75.1–150.6)	40.7 (24.9–60.3)	230.4 (145.6–326.3)	12.5 (6.4–19.4)	76.5 (58.9–95.9)
Nicaragua	YLDs Rate: 2015	5,325.4 (4,043.3–6,786.2)	982.8 (694.0–1,340.4)	790.2 (543.4–1,095.1)	426.5 (280.8–598.1)	59.4 (41.7–80.9)	147.2 (105.5–196.3)	157.1 (107.6–220.4)	17.0 (9.7–26.8)	108.9 (67.2–158.4)	3.7 (2.4–5.4)	67.1 (52.0–84.7)
Nicaragua	YLDs Rate: Cumulative change (%)	-13.7 (-16.4 to -11.2)	-12.6 (-20.9 to -6.4)	0.8 (-1.2 to 2.8)	-30.7 (-38.0 to -22.7)	-7.6 (-16.8 to 1.3)	-42.6 (-47.7 to -37.5)	44.0 (16.6–75.6)	-57.0 (-74.7 to -31.8)	-51.4 (-68.5 to -29.2)	-68.3 (-79.6 to -49.1)	-12.1 (-21.0 to -2.4)
Panama	Prevalence Number: 1990	1,090,012 (1,083,505–1,095,799)	292,434 (275,282–311,891)	371,842 (360,498–383,340)	202,725 (175,885–237,301)	163,443 (148,641–179,105)	11,270 (10,298–12,307)	14,858 (12,643–17,947)	3,152 (2,394–4,046)	4,935 (3,601–6,334)	2,158 (1,094–3,226)	7,512 (6,568–8,655)
Panama	Prevalence Number: 2015	1,265,164 (1,254,390–1,275,635)	364,919 (341,477–386,810)	462,237 (448,577–475,287)	220,777 (182,310–262,172)	164,759 (149,417–182,196)	13,566 (12,268–14,831)	18,726 (16,078–22,510)	1,829 (1,172–2,615)	6,386 (4,554–8,444)	2,537 (1,131–3,822)	8,145 (7,067–9,405)
Panama	Prevalence Number: Cumulative change (%)	16.1 (14.9–17.1)	24.9 (18.4–33.0)	24.3 (19.2–29.2)	9.0 (-2.8 to 24.9)	1.1 (-11.2 to 14.0)	20.4 (13.3–26.7)	27.2 (1.6–56.9)	-40.4 (-65.8 to -6.5)	32.6 (-12.1 to 86.7)	17.4 (-9.9 to 38.5)	8.9 (-7.2 to 25.0)
Panama	Prevalence Rate: 1990	94,213.6 (93,656.8–94,707.0)	25,211.2 (23,734.5–26,844.1)	32,237.5 (31,252.5–33,231.5)	17,529.5 (15,224.6–20,506.8)	14,118.2 (12,841.4–15,468.4)	957.3 (875.1–1,044.9)	1,283.0 (1,091.7–1,549.5)	266.4 (202.3–341.8)	427.1 (311.1–548.8)	184.5 (93.3–276.0)	643.7 (562.4–742.0)
Panama	Prevalence Rate: 2015	89,660.8 (88,894.0–90,407.3)	25,952.1 (24,273.1–27,532.2)	32,660.2 (31,699.4–33,587.3)	15,687.4 (12,954.2–18,636.7)	11,684.8 (10,596.2–12,919.3)	970.1 (877.0–1,060.7)	1,328.2 (1,140.0–1,595.9)	131.1 (84.0–187.4)	452.2 (322.7–598.4)	181.3 (80.8–273.1)	580.2 (503.7–669.3)
Panama	Prevalence Rate: Cumulative change (%)	-4.8 (-5.8 to -4.0)	3.0 (-2.5 to 9.7)	1.3 (-2.9 to 5.3)	-10.4 (-20.1 to 2.6)	-17.0 (-27.1 to -6.4)	1.4 (-4.7 to 6.6)	4.5 (-16.5 to 28.8)	-49.5 (-71.0 to -20.7)	8.5 (-28.2 to 53.0)	-1.9 (-24.5 to 15.6)	-9.5 (-22.8 to 3.9)
Panama	YLDs Number: 1990	68,118 (51,303–86,861)	10,924 (7,664–14,921)	8,611 (5,973–11,929)	9,216 (6,157–12,652)	674 (459–925)	1,848 (1,342–2,485)	1,412 (964–1,977)	393 (239–565)	1,855 (1,183–2,598)	76 (33–130)	904 (702–1,145)
Panama	YLDs Number: 2015	78,819 (59,599–100,526)	13,669 (9,565–18,764)	10,617 (7,330–14,729)	10,048 (6,725–13,926)	812 (576–1,112)	2,227 (1,590–2,971)	1,920 (1,298–2,665)	227 (132–360)	1,626 (1,017–2,364)	90 (35–157)	954 (738–1,205)
Panama	YLDs Number: Cumulative change (%)	15.7 (12.6–19.2)	25.2 (18.4–35.5)	23.3 (20.9–25.7)	9.2 (-2.4 to 25.0)	20.9 (10.2–35.6)	20.5 (12.4–28.4)	36.6 (17.2–58.7)	-40.5 (-66.4 to -5.8)	-10.0 (-41.2 to 31.3)	18.8 (-11.9 to 53.1)	5.7 (-4.1 to 15.3)
Panama	YLDs Rate: 1990	5,901.2 (4,446.5–7,521.7)	941.9 (661.6–1,285.0)	745.1 (517.3–1,032.7)	796.9 (532.4–1,093.5)	58.2 (39.7–80.0)	157.0 (114.1–211.0)	122.0 (83.3–170.7)	33.3 (20.2–47.8)	160.6 (102.5–224.9)	6.6 (2.9–11.2)	78.1 (60.6–98.9)
Panama	YLDs Rate: 2015	5,577.1 (4,216.4–7,112.3)	974.7 (681.9–1,338.2)	751.7 (519.1–1,042.9)	714.0 (477.9–989.3)	58.0 (41.1–79.3)	159.3 (113.7–212.5)	136.2 (92.1–189.1)	16.3 (9.5–25.8)	115.2 (72.1–167.3)	6.5 (2.5–11.2)	67.7 (52.4–85.5)
Panama	YLDs Rate: Cumulative change (%)	-5.5 (-8.0 to -2.6)	3.5 (-2.2 to 12.0)	0.9 (-1.1 to 2.8)	-10.3 (-19.8 to 2.8)	-0.2 (-9.0 to 11.9)	1.5 (-5.3 to 8.2)	12.2 (-3.7 to 30.5)	-49.5 (-71.5 to -20.1)	-26.4 (-52.0 to 7.6)	-1.0 (-26.3 to 27.7)	-13.2 (-21.2 to -5.2)
Venezuela	Prevalence Number: 1990	8,858,030 (8,798,376–8,910,667)	2,821,553 (2,628,750–3,032,375)	3,071,239 (2,985,538–3,158,582)	1,364,242 (1,114,935–1,630,291)	1,314,563 (1,194,677–1,437,238)	81,007 (74,809–88,086)	122,736 (104,395–149,149)	47,185 (42,107–53,191)	44,281 (31,895–56,802)	30,812 (15,989–46,180)	59,508 (52,182–68,017)
Venezuela	Prevalence Number: 2015	10,766,252 (10,673,875–10,852,251)	4,706,615 (4,432,904–5,070,678)	3,821,829 (3,712,666–3,929,998)	1,237,265 (1,021,228–1,478,206)	1,378,017 (1,250,353–1,508,186)	83,350 (77,020–90,285)	161,749 (139,536–192,242)	39,952 (26,579–57,459)	51,980 (37,967–67,670)	22,678 (12,494–40,185)	69,776 (60,250–79,819)
Venezuela	Prevalence Number: Cumulative change (%)	21.5 (20.4–22.8)	67.2 (49.4–83.5)	24.5 (19.9–29.0)	-9.1 (-19.0 to 0.8)	5.1 (-7.6 to 19.5)	3.0 (-2.8 to 8.8)	33.2 (5.2–64.8)	-15.0 (-44.2 to 25.3)	20.2 (-17.0 to 71.0)	-26.6 (-46.0 to -4.2)	17.7 (2.2–35.8)
Venezuela	Prevalence Rate: 1990	93,209.6 (92,620.0–93,741.6)	29,467.6 (27,627.3–31,385.3)	32,859.3 (31,935.7–33,782.4)	14,291.9 (11,720.8–17,035.1)	13,797.6 (12,543.4–15,082.2)	785.7 (726.2–854.0)	1,284.6 (1,092.7–1,559.8)	450.5 (402.0–507.9)	470.1 (336.6–604.0)	306.6 (160.8–458.4)	606.4 (531.1–695.5)
Venezuela	Prevalence Rate: 2015	93,353.9 (92,541.3–94,112.0)	40,749.3 (38,345.1–43,971.6)	33,023.4 (32,080.4–33,961.0)	10,713.1 (8,837.0–12,808.4)	11,948.7 (10,839.8–13,078.2)	737.9 (681.4–799.6)	1,404.4 (1,211.4–1,669.1)	356.1 (236.9–512.2)	450.2 (328.7–586.3)	198.8 (109.2–353.1)	612.0 (529.3–699.1)
Venezuela	Prevalence Rate: Cumulative change (%)	0.2 (-0.8 to 1.1)	38.5 (24.2–51.8)	0.5 (-3.1 to 4.2)	-24.9 (-32.8 to -17.1)	-13.2 (-23.6 to -1.3)	-6.0 (-11.3 to -0.6)	10.5 (-12.7 to 36.9)	-20.6 (-47.9 to 16.9)	-1.9 (-32.3 to 39.7)	-35.4 (-52.5 to -15.2)	1.3 (-12.1 to 16.9)
Venezuela	YLDs Number: 1990	560,687 (424,683–715,739)	108,198 (76,333–145,640)	74,377 (51,960–102,129)	61,882 (40,771–86,494)	5,352 (3,850–7,174)	13,291 (9,574–17,719)	9,920 (6,899–13,589)	5,893 (4,065–8,103)	16,813 (10,487–23,830)	1,100 (527–1,907)	7,831 (6,067–9,878)
Venezuela	YLDs Number: 2015	735,328 (553,320–941,422)	198,958 (140,980–270,765)	90,902 (63,077–124,594)	55,939 (36,547–77,469)	10,548 (7,550–14,256)	13,650 (9,777–18,281)	14,970 (10,478–20,611)	4,998 (2,888–7,867)	12,970 (8,099–18,538)	845 (400–1,573)	8,274 (6,336–10,484)
Venezuela	YLDs Number: Cumulative change (%)	31.1 (25.8–36.5)	84.5 (59.9–108.5)	22.2 (19.5–24.8)	-9.4 (-19.3 to 0.4)	97.7 (74.5–124.8)	2.8 (-4.7 to 10.2)	51.4 (34.5–72.3)	-14.9 (-44.3 to 26.1)	-20.7 (-47.5 to 16.5)	-22.8 (-47.0 to 7.2)	5.8 (-3.7 to 16.2)
Venezuela	YLDs Rate: 1990	5,972.8 (4,525.0–7,612.1)	1,125.6 (794.8–1,513.5)	785.6 (548.4–1,081.1)	648.1 (427.6–905.6)	56.2 (40.3–75.2)	128.9 (92.8–171.6)	103.8 (72.0–142.0)	56.3 (38.8–77.4)	178.4 (111.2–253.4)	11.0 (5.3–19.1)	81.9 (63.6–103.4)
Venezuela	YLDs Rate: 2015	6,351.9 (4,777.3–8,135.6)	1,721.1 (1,219.2–2,343.0)	787.4 (546.6–1,078.8)	484.4 (316.3–671.9)	91.0 (65.2–123.0)	120.9 (86.6–161.7)	130.0 (91.1–179.0)	44.6 (25.7–70.1)	112.3 (70.1–160.6)	7.4 (3.5–13.8)	71.9 (55.0–91.1)
Venezuela	YLDs Rate: Cumulative change (%)	6.3 (2.1–10.7)	53.4 (33.2–72.3)	0.2 (-1.8 to 2.2)	-25.1 (-33.1 to -17.2)	62.4 (44.3–84.1)	-6.2 (-13.0 to 0.6)	25.7 (11.6–43.2)	-20.5 (-48.0 to 17.8)	-35.3 (-57.2 to -4.9)	-32.7 (-53.7 to -6.9)	-12.2 (-19.9 to -3.6)
Andean Latin America	Prevalence Number: 1990	18,309,004 (18,241,019–18,379,890)	5,865,178 (5,628,262–6,174,677)	6,582,457 (6,445,853–6,717,036)	3,304,815 (2,740,613–3,967,256)	2,137,432 (2,053,213–2,227,538)	363,639 (337,392–387,372)	201,023 (177,956–228,962)	56,479 (46,346–68,458)	89,940 (72,741–107,251)	177,868 (128,411–228,178)	203,565 (174,923–239,024)
Andean Latin America	Prevalence Number: 2015	20,988,527 (20,899,196–21,071,856)	7,087,131 (6,878,093–7,301,228)	7,825,043 (7,678,475–7,966,001)	2,708,694 (2,238,052–3,194,909)	2,196,360 (2,112,746–2,279,924)	242,067 (226,245–257,366)	267,125 (240,413–299,782)	29,637 (23,132–37,511)	86,487 (69,304–105,767)	56,971 (17,515–192,073)	228,882 (198,558–263,101)
Andean Latin America	Prevalence Number: Cumulative change (%)	14.6 (14.0–15.2)	20.9 (14.3–28.3)	18.9 (15.9–21.8)	-17.8 (-28.4 to -8.3)	2.8 (-2.5 to 8.1)	-33.4 (-37.2 to -29.6)	33.5 (13.2–53.5)	-46.8 (-62.0 to -29.0)	-2.9 (-25.4 to 21.4)	-68.9 (-89.7 to -3.5)	12.9 (-1.3 to 26.8)
Andean Latin America	Prevalence Rate: 1990	94,715.3 (94,379.3–95,060.9)	30,293.0 (29,153.7–31,789.4)	34,471.8 (33,781.2–35,164.4)	17,042.0 (14,139.4–20,407.1)	11,045.5 (10,611.8–11,511.2)	1,752.8 (1,627.4–1,865.2)	1,033.7 (915.0–1,177.2)	268.2 (220.1–325.1)	468.4 (379.1–557.0)	878.3 (635.6–1,127.8)	1,003.6 (861.8–1,183.0)
Andean Latin America	Prevalence Rate: 2015	93,924.4 (93,524.4–94,297.1)	31,756.5 (30,817.1–32,714.2)	34,967.1 (34,309.8–35,598.3)	12,148.1 (10,038.9–14,336.5)	9,830.5 (9,456.6–10,204.6)	1,085.3 (1,014.4–1,153.7)	1,195.7 (1,076.1–1,341.9)	132.8 (103.6–168.1)	386.9 (310.1–473.2)	255.7 (78.5–862.3)	1,027.7 (891.6–1,181.0)
Andean Latin America	Prevalence Rate: Cumulative change (%)	-0.8 (-1.3 to -0.4)	4.9 (-0.7 to 11.1)	1.4 (-1.1 to 3.9)	-28.5 (-38.0 to -20.3)	-11.0 (-15.5 to -6.4)	-38.0 (-41.5 to -34.6)	16.2 (-1.5 to 33.7)	-49.8 (-64.1 to -33.0)	-16.6 (-35.8 to 4.3)	-71.7 (-90.6 to -12.3)	2.8 (-10.3 to 15.5)
Andean Latin America	YLDs Number: 1990	1,281,040 (970,383–1,630,310)	224,851 (161,340–302,492)	155,064 (107,000–213,830)	149,472 (100,135–207,653)	11,260 (8,090–15,105)	59,554 (42,953–78,661)	14,048 (9,692–19,461)	6,999 (4,683–9,918)	34,148 (23,730–45,466)	5,363 (3,362–7,576)	20,698 (16,069–26,139)
Andean Latin America	YLDs Number: 2015	1,350,888 (1,020,624–1,713,115)	273,511 (194,665–370,405)	181,745 (125,198–251,611)	122,875 (81,509–169,369)	13,153 (9,456–17,740)	39,559 (28,483–52,779)	24,264 (16,721–33,692)	3,687 (2,396–5,304)	21,852 (14,863–29,642)	1,476 (517–3,439)	21,706 (16,811–27,241)
Andean Latin America	YLDs Number: Cumulative change (%)	5.4 (3.0–7.9)	21.7 (13.5–30.5)	17.2 (15.4–18.8)	-17.6 (-28.4 to -7.9)	16.9 (8.4–26.5)	-33.5 (-37.6 to -29.4)	73.6 (47.8–104.8)	-46.6 (-61.9 to -28.5)	-35.4 (-51.1 to -17.3)	-72.8 (-88.9 to -42.4)	5.0 (-2.5 to 13.1)
Andean Latin America	YLDs Rate: 1990	6,685.5 (5,068.0–8,503.4)	1,158.2 (830.9–1,557.1)	805.1 (557.5–1,113.7)	770.7 (516.8–1,071.9)	58.3 (41.9–78.3)	287.0 (206.7–378.8)	72.2 (49.8–100.0)	33.2 (22.2–47.1)	177.8 (123.6–236.3)	26.8 (16.8–37.8)	105.8 (82.1–133.4)
Andean Latin America	YLDs Rate: 2015	6,042.2 (4,564.9–7,662.0)	1,227.6 (873.9–1,662.2)	812.8 (560.0–1,124.7)	551.1 (365.6–759.9)	59.0 (42.4–79.6)	177.4 (127.7–236.6)	108.6 (74.9–150.8)	16.5 (10.7–23.8)	97.8 (66.5–132.5)	6.6 (2.3–15.5)	97.2 (75.3–122.0)
Andean Latin America	YLDs Rate: Cumulative change (%)	-9.6 (-11.7 to -7.6)	6.1 (-0.9 to 13.3)	0.9 (-0.3 to 2.2)	-28.3 (-37.9 to -19.9)	1.3 (-6.0 to 9.4)	-38.2 (-42.0 to -34.3)	51.2 (28.7–78.4)	-49.6 (-64.0 to -32.6)	-44.5 (-57.9 to -29.0)	-75.5 (-90.0 to -48.0)	-8.1 (-14.6 to -0.9)
Bolivia	Prevalence Number: 1990	3,281,728 (3,255,560–3,308,633)	867,408 (834,648–895,911)	1,191,243 (1,157,673–1,224,711)	399,728 (330,695–476,695)	373,256 (353,429–396,826)	69,860 (65,448–73,764)	29,760 (24,239–37,443)	12,348 (8,657–17,364)	15,725 (11,756–19,879)	1,665 (766–3,411)	43,160 (35,580–53,083)
Bolivia	Prevalence Number: 2015	4,334,436 (4,307,041–4,355,645)	1,630,464 (1,539,132–1,745,325)	1,590,643 (1,542,452–1,637,723)	508,925 (413,128–606,219)	442,153 (419,758–470,714)	67,212 (61,926–73,286)	49,855 (42,039–60,540)	8,228 (5,239–11,873)	16,216 (12,073–21,065)	4,548 (1,107–13,428)	56,020 (47,184–68,176)
Bolivia	Prevalence Number: Cumulative change (%)	32.1 (30.9–33.2)	88.1 (73.4–108.1)	33.6 (28.5–38.8)	27.5 (15.4–40.5)	18.6 (9.6–28.2)	-3.8 (-10.5 to 4.2)	70.1 (28.9–118.9)	-30.5 (-61.9 to 9.5)	5.3 (-27.2 to 47.6)	142.3 (20.6–452.7)	31.0 (8.2–59.1)
Bolivia	Prevalence Rate: 1990	92,269.4 (91,595.8–92,954.3)	24,833.7 (23,929.9–25,600.6)	34,109.5 (33,171.7–35,036.6)	11,211.7 (9,278.8–13,289.8)	10,457.9 (9,903.4–11,117.8)	1,743.7 (1,637.2–1,839.8)	824.2 (670.1–1,040.1)	301.6 (211.5–423.6)	444.9 (331.5–561.7)	43.5 (20.4–88.5)	1,109.7 (911.6–1,378.6)
Bolivia	Prevalence Rate: 2015	94,175.6 (93,583.6–94,634.6)	35,335.6 (33,373.4–37,802.5)	34,581.1 (33,531.0–35,604.6)	11,078.2 (8,992.2–13,197.7)	9,602.4 (9,115.4–10,223.2)	1,449.7 (1,336.5–1,580.1)	1,082.9 (913.2–1,314.9)	176.7 (112.5–255.0)	352.5 (262.5–457.9)	98.6 (24.1–290.8)	1,213.7 (1,022.4–1,477.4)
Bolivia	Prevalence Rate: Cumulative change (%)	2.1 (1.2–2.9)	42.4 (31.7–57.2)	1.4 (-2.4 to 5.3)	-1.1 (-10.3 to 8.8)	-8.1 (-15.1 to -0.6)	-16.8 (-22.6 to -10.0)	33.5 (0.9–71.9)	-38.9 (-66.6 to -3.6)	-19.1 (-44.1 to 13.4)	100.6 (-0.4 to 359.2)	10.5 (-10.3 to 35.1)
Bolivia	YLDs Number: 1990	220,520 (166,738–280,141)	35,078 (25,361–46,825)	28,618 (19,788–39,336)	18,113 (11,777–24,741)	1,796 (1,292–2,415)	11,509 (8,319–15,200)	2,247 (1,474–3,345)	1,537 (927–2,395)	6,184 (3,909–8,645)	51 (19–115)	4,087 (3,078–5,180)
Bolivia	YLDs Number: 2015	301,902 (227,118–383,110)	68,692 (47,988–94,651)	37,404 (25,833–51,866)	23,046 (15,253–31,843)	3,049 (2,155–4,182)	10,905 (7,900–14,531)	4,340 (2,735–6,663)	1,013 (582–1,593)	4,169 (2,666–5,977)	196 (37–617)	5,324 (4,096–6,652)
Bolivia	YLDs Number: Cumulative change (%)	36.9 (32.0–42.7)	96.1 (74.4–126.3)	30.7 (27.8–33.3)	27.4 (15.2–40.6)	70.1 (48.3–96.1)	-5.1 (-12.3 to 3.0)	96.0 (45.9–180.0)	-30.9 (-62.0 to 10.6)	-31.0 (-53.4 to -1.2)	243.6 (46.5–681.1)	30.7 (17.2–46.5)

© 2017 Global Burden of Disease Child and Adolescent Health Collaboration. JAMA Pediatrics.

eTable 3. Prevalent Cases, Rates (per 100,000 population), Years Lived with Disability (YLDs), and Cumulative Percent Change with 95% Uncertainty Interval (UI) for the Top 10 Global Causes of YLDs in Children and Adolescents in 195 Countries and Territories, Aged 0 to 19 Years, Both Sexes, 1990 and 2015. *Note: best viewed by enlarging in browser.*

Location	Measure	All causes	Iron-deficiency anemia	Skin and subcutaneous diseases	Asthma	Hemoglobinopathies and hemolytic anemias	Diarrheal diseases	Congenital anomalies	Protein-energy malnutrition	Epilepsy	Malaria	Neonatal preterm birth complications
Bolivia	YLDs Rate: 1990	6,306.4 (4,779.9–8,022.0)	994.8 (717.7–1,330.1)	807.1 (557.4–1,113.7)	507.9 (331.0–694.3)	50.8 (36.6–68.4)	287.1 (207.5–379.4)	62.2 (40.6–92.6)	37.6 (22.7–58.5)	174.8 (111.0–244.8)	1.4 (0.5–3.1)	111.7 (84.3–141.7)
Bolivia	YLDs Rate: 2015	6,560.1 (4,937.3–8,324.1)	1,488.3 (1,040.5–2,049.4)	812.7 (561.5–1,127.0)	501.7 (332.1–693.2)	66.1 (46.7–90.6)	235.2 (170.4–313.6)	94.3 (59.4–144.7)	21.8 (12.5–34.2)	90.6 (57.9–130.0)	4.3 (0.8–13.4)	115.6 (89.0–144.5)
Bolivia	YLDs Rate: Cumulative change (%)	4.0 (0.4–8.2)	49.8 (33.9–72.4)	0.7 (-1.2 to 2.5)	-1.1 (-10.4 to 8.8)	30.3 (13.7–49.7)	-18.0 (-24.1 to -11.0)	53.9 (14.2–120.2)	-39.2 (-66.6 to -2.6)	-46.9 (-64.1 to -23.8)	180.6 (18.9–534.3)	3.9 (-7.3 to 16.8)
Ecuador	Prevalence Number: 1990	4,826,290 (4,805,975–4,847,360)	1,567,248 (1,493,283–1,655,262)	1,691,537 (1,637,557–1,743,299)	743,409 (613,215–886,153)	571,695 (538,500–602,814)	100,259 (92,089–108,152)	58,178 (49,102–70,506)	15,942 (10,656–22,498)	23,760 (17,777–29,931)	170,126 (122,344–218,542)	52,780 (44,385–63,198)
Ecuador	Prevalence Number: 2015	5,872,315 (5,829,999–5,908,187)	1,881,604 (1,750,677–2,024,434)	2,141,658 (2,075,120–2,207,925)	599,418 (489,630–714,074)	622,934 (588,804–662,266)	61,046 (56,365–65,031)	78,172 (66,865–94,298)	13,407 (8,255–19,857)	23,476 (17,117–30,862)	39,688 (11,466–139,486)	65,723 (56,166–77,870)
Ecuador	Prevalence Number: Cumulative change (%)	21.7 (20.7–22.6)	20.2 (7.8–32.2)	26.6 (21.7–31.8)	-19.2 (-28.5 to -9.3)	9.1 (1.0–18.1)	-39.0 (-43.2 to -34.6)	35.8 (6.6–69.0)	-11.9 (-54.3 to 43.0)	1.1 (-32.3 to 41.7)	-77.3 (-93.0 to -25.6)	25.6 (5.1–48.2)
Ecuador	Prevalence Rate: 1990	96,113.1 (95,734.5–96,504.3)	31,182.8 (29,788.3–32,827.6)	34,106.2 (33,036.2–35,112.7)	14,766.1 (12,171.2–17,563.6)	11,381.3 (10,725.2–12,002.9)	1,862.8 (1,713.2–2,007.7)	1,153.5 (973.9–1,396.5)	291.4 (194.9–411.0)	478.0 (358.3–601.9)	3,232.4 (2,321.4–4,154.9)	1,006.2 (845.6–1,209.7)
Ecuador	Prevalence Rate: 2015	94,788.3 (94,096.1–95,371.9)	30,426.7 (28,307.9–32,755.2)	34,499.0 (33,422.9–35,566.2)	9,693.4 (7,916.1–11,551.2)	10,058.1 (9,506.6–10,693.1)	991.5 (915.2–1,056.3)	1,262.4 (1,079.8–1,522.3)	218.1 (134.3–323.1)	378.6 (275.9–497.5)	643.8 (185.7–2,263.4)	1,065.9 (911.1–1,262.9)
Ecuador	Prevalence Rate: Cumulative change (%)	-1.4 (-2.2 to -0.6)	-2.3 (-12.1 to 7.4)	1.2 (-2.8 to 5.3)	-34.2 (-41.7 to -26.2)	-11.5 (-18.1 to -4.3)	-46.7 (-50.3 to -42.9)	10.6 (-13.1 to 37.6)	-21.6 (-59.3 to 27.2)	-18.9 (-45.5 to 13.4)	-80.6 (-94.0 to -36.5)	6.9 (-11.0 to 26.3)
Ecuador	YLDs Number: 1990	324,824 (245,087–415,074)	59,841 (42,498–80,455)	38,365 (26,475–53,199)	33,657 (22,312–46,408)	2,909 (2,070–3,916)	16,401 (11,799–21,825)	3,992 (2,780–5,534)	1,979 (1,184–3,124)	8,917 (5,654–12,426)	5,113 (3,203–7,211)	5,036 (3,891–6,389)
Ecuador	YLDs Number: 2015	355,194 (269,360–452,528)	70,955 (50,007–96,379)	48,140 (33,296–66,575)	27,217 (17,956–37,456)	3,418 (2,437–4,589)	10,003 (7,209–13,377)	6,767 (4,729–9,343)	1,673 (898–2,705)	5,920 (3,768–8,425)	822 (332–1,595)	6,207 (4,758–7,905)
Ecuador	YLDs Number: Cumulative change (%)	9.4 (5.6–13.1)	18.7 (5.7–31.4)	25.4 (22.9–27.9)	-19.0 (-28.5 to -8.9)	17.7 (4.5–32.3)	-38.9 (-43.4 to -34.2)	70.1 (48.5–97.4)	-11.3 (-54.3 to 45.0)	-31.8 (-56.1 to -2.0)	-83.9 (-92.7 to -71.9)	23.4 (11.7–36.9)
Ecuador	YLDs Rate: 1990	6,526.1 (4,921.4–8,332.1)	1,190.9 (845.1–1,597.4)	767.9 (529.5–1,066.9)	668.4 (443.7–921.1)	58.3 (41.5–78.4)	304.7 (219.6–405.0)	79.1 (55.2–109.7)	36.2 (21.7–57.1)	179.3 (113.6–250.1)	98.4 (61.4–138.4)	99.5 (77.0–126.0)
Ecuador	YLDs Rate: 2015	5,725.7 (4,341.7–7,297.7)	1,149.8 (810.5–1,561.4)	776.4 (537.2–1,073.7)	440.2 (290.1–605.9)	55.4 (39.5–74.4)	162.5 (117.1–217.3)	109.3 (76.4–150.9)	27.2 (14.6–44.0)	95.5 (60.8–135.9)	13.3 (5.4–25.9)	100.3 (76.8–127.8)
Ecuador	YLDs Rate: Cumulative change (%)	-12.3 (-15.2 to -9.3)	-3.4 (-13.9 to 6.9)	1.1 (-0.8 to 3.1)	-34.0 (-41.8 to -26.1)	-4.7 (-15.4 to 6.9)	-46.6 (-50.6 to -42.5)	38.6 (21.0–61.2)	-21.0 (-59.3 to 28.9)	-45.3 (-64.9 to -21.6)	-86.4 (-93.9 to -76.5)	0.9 (-8.5 to 12.1)
Peru	Prevalence Number: 1990	10,200,986 (10,143,445–10,260,165)	3,430,521 (3,211,812–3,737,101)	3,699,676 (3,578,382–3,800,697)	2,161,678 (1,765,527–2,663,122)	1,192,481 (1,126,312–1,265,732)	193,519 (177,772–208,222)	113,084 (95,238–137,095)	28,189 (21,009–36,831)	50,455 (36,601–65,659)	6,076 (4,031–9,249)	107,624 (90,852–131,006)
Peru	Prevalence Number: 2015	10,781,774 (10,715,963–10,847,209)	3,575,061 (3,438,369–3,701,005)	4,092,742 (3,979,298–4,203,038)	1,600,350 (1,317,797–1,892,471)	1,131,271 (1,072,310–1,199,695)	113,808 (106,388–121,702)	139,096 (119,501–164,559)	8,001 (5,715–10,901)	46,795 (33,524–60,713)	12,734 (4,587–37,928)	107,138 (91,186–125,991)
Peru	Prevalence Number: Cumulative change (%)	5.7 (4.8–6.5)	4.4 (-4.1 to 14.1)	10.7 (6.5–15.3)	-25.5 (-39.8 to -14.3)	-5.0 (-12.5 to 2.9)	-41.1 (-46.0 to -36.4)	24.3 (-3.2 to 52.4)	-70.7 (-81.8 to -56.6)	-4.7 (-36.5 to 33.9)	111.8 (-1.3 to 497.5)	0.3 (-17.5 to 19.5)
Peru	Prevalence Rate: 1990	94,906.6 (94,389.1–95,430.6)	31,779.9 (29,874.8–34,494.4)	34,760.1 (33,645.7–35,705.2)	20,053.2 (16,406.0–24,750.3)	11,084.5 (10,468.0–11,765.0)	1,703.6 (1,567.2–1,832.0)	1,047.2 (881.7–1,269.1)	245.2 (182.9–320.3)	471.5 (341.9–612.6)	55.2 (36.6–83.9)	964.2 (812.5–1,180.0)
Peru	Prevalence Rate: 2015	93,356.9 (92,785.9–93,924.3)	31,012.4 (29,833.7–32,095.5)	35,371.2 (34,383.2–36,324.6)	13,893.3 (11,440.5–16,423.2)	9,797.8 (9,287.4–10,390.8)	988.4 (924.2–1,056.9)	1,204.8 (1,035.2–1,425.4)	69.5 (49.6–94.6)	405.0 (290.2–525.6)	110.7 (39.8–330.1)	932.3 (793.8–1,096.8)
Peru	Prevalence Rate: Cumulative change (%)	-1.6 (-2.4 to -0.9)	-2.2 (-9.8 to 6.3)	1.8 (-2.0 to 5.9)	-30.3 (-43.8 to -19.8)	-11.5 (-18.5 to -4.1)	-41.9 (-46.7 to -37.3)	16.3 (-9.4 to 42.4)	-70.8 (-81.8 to -56.7)	-11.8 (-41.2 to 24.2)	102.5 (-5.8 to 472.6)	-2.5 (-20.4 to 16.3)
Peru	YLDs Number: 1990	735,695 (557,472–939,682)	129,931 (92,835–175,024)	88,080 (60,502–121,973)	97,701 (65,127–139,128)	6,555 (4,689–8,763)	31,643 (22,769–41,980)	7,809 (5,371–10,827)	3,482 (2,180–5,189)	19,046 (12,114–26,927)	198 (106–336)	11,573 (8,917–14,661)
Peru	YLDs Number: 2015	693,791 (524,699–879,714)	133,862 (95,080–180,018)	96,200 (66,165–133,620)	72,611 (48,326–99,586)	6,685 (4,806–9,089)	18,651 (13,496–24,739)	13,156 (8,929–18,381)	1,000 (591–1,532)	11,762 (7,548–16,792)	457 (132–1,435)	10,174 (7,854–12,784)
Peru	YLDs Number: Cumulative change (%)	-5.7 (-8.9 to -2.8)	3.2 (-6.8 to 14.9)	9.2 (6.9–11.3)	-25.2 (-39.6 to -14.0)	2.2 (-8.2 to 13.8)	-41.0 (-46.1 to -35.8)	69.5 (41.7–107.1)	-70.4 (-82.0 to -55.2)	-36.6 (-58.8 to -9.5)	134.8 (-3.8 to 586.7)	-11.9 (-20.5 to -1.4)
Peru	YLDs Rate: 1990	6,888.3 (5,218.6–8,789.0)	1,200.2 (859.6–1,617.6)	821.8 (565.1–1,137.8)	906.2 (603.6–1,290.6)	60.8 (43.5–81.3)	278.5 (200.4–369.6)	72.3 (49.6–100.5)	30.3 (19.0–45.1)	177.9 (113.3–252.4)	1.8 (1.0–3.1)	106.7 (82.2–135.4)
Peru	YLDs Rate: 2015	6,003.1 (4,539.4–7,613.3)	1,163.7 (826.5–1,564.7)	832.4 (572.5–1,155.8)	630.4 (419.6–864.6)	58.1 (41.8–78.9)	162.0 (117.2–214.9)	114.0 (77.3–159.2)	8.7 (5.1–13.3)	101.8 (65.3–145.3)	4.0 (1.2–12.5)	88.2 (68.1–110.8)
Peru	YLDs Rate: Cumulative change (%)	-12.8 (-15.8 to -10.3)	-2.9 (-12.0 to 7.6)	1.3 (-0.7 to 3.2)	-30.0 (-43.7 to -19.5)	-4.4 (-13.9 to 6.3)	-41.8 (-46.9 to -36.7)	58.6 (32.4–94.2)	-70.5 (-82.1 to -55.3)	-41.2 (-62.0 to -16.0)	124.2 (-8.2 to 554.8)	-17.2 (-25.2 to -7.3)
Caribbean	Prevalence Number: 1990	14,368,832 (14,321,627–14,413,172)	5,199,610 (5,035,818–5,386,261)	5,346,886 (5,270,059–5,428,032)	2,670,614 (2,291,080–3,098,733)	1,912,450 (1,828,718–2,032,315)	189,184 (176,544–204,396)	183,382 (169,008–200,437)	81,960 (72,194–92,284)	58,043 (49,580–67,244)	73,224 (39,357–127,535)	309,575 (279,480–345,223)
Caribbean	Prevalence Number: 2015	14,098,610 (14,042,012–14,156,503)	4,867,798 (4,721,898–5,009,919)	5,415,326 (5,340,891–5,501,675)	2,437,520 (2,041,563–2,848,575)	1,639,505 (1,572,337–1,723,357)	161,040 (151,104–171,740)	191,664 (176,437–208,421)	61,637 (52,409–72,947)	47,701 (40,063–56,409)	31,611 (19,171–59,617)	298,919 (267,447–336,991)
Caribbean	Prevalence Number: Cumulative change (%)	-1.9 (-2.3 to -1.5)	-6.3 (-10.8 to -2.0)	1.3 (-0.4 to 3.0)	-8.8 (-13.0 to -4.3)	-14.2 (-19.5 to -9.2)	-14.8 (-18.0 to -12.0)	4.7 (-5.6 to 15.5)	-24.4 (-38.6 to -6.3)	-17.5 (-30.6 to -3.3)	-55.3 (-72.9 to -35.3)	-3.3 (-11.7 to 6.8)
Caribbean	Prevalence Rate: 1990	93,516.2 (93,210.6–93,804.9)	33,748.1 (32,715.6–34,914.2)	34,945.0 (34,445.7–35,478.5)	17,481.4 (14,999.1–20,232.8)	12,442.0 (11,897.0–13,223.0)	1,191.8 (1,113.3–1,287.0)	1,191.4 (1,098.0–1,302.6)	509.3 (448.6–573.4)	379.3 (324.0–440.1)	467.2 (251.3–811.3)	1,976.3 (1,782.5–2,206.4)
Caribbean	Prevalence Rate: 2015	91,994.6 (91,615.4–92,379.7)	32,024.6 (31,056.3–32,967.5)	35,027.0 (34,537.9–35,579.6)	15,924.6 (13,346.2–18,664.9)	10,710.5 (10,272.0–11,257.8)	1,089.6 (1,021.7–1,162.7)	1,253.1 (1,153.5–1,362.3)	423.4 (360.0–501.0)	309.5 (260.0–365.2)	210.9 (127.2–400.4)	2,000.2 (1,791.9–2,253.3)
Caribbean	Prevalence Rate: Cumulative change (%)	-1.6 (-2.1 to -1.2)	-5.1 (-9.5 to -0.7)	0.2 (-1.4 to 2.0)	-8.9 (-13.3 to -4.3)	-13.9 (-19.2 to -8.8)	-8.5 (-11.9 to -5.6)	5.4 (-5.0 to 16.2)	-16.4 (-32.1 to 3.6)	-18.1 (-31.2 to -4.0)	-53.3 (-71.7 to -33.1)	1.4 (-7.4 to 12.0)
Caribbean	YLDs Number: 1990	1,047,890 (794,240–1,329,055)	204,320 (146,593–274,448)	134,412 (94,003–185,608)	120,725 (80,866–164,347)	11,700 (8,387–15,723)	30,765 (22,235–40,501)	14,762 (10,357–20,192)	10,130 (7,011–13,997)	22,006 (15,652–28,849)	1,754 (1,021–2,602)	34,656 (27,149–43,300)
Caribbean	YLDs Number: 2015	1,011,612 (768,265–1,280,141)	189,319 (135,076–255,268)	134,395 (93,538–185,682)	110,321 (73,594–151,707)	10,623 (7,643–14,257)	26,205 (18,972–34,367)	17,342 (12,058–23,875)	7,640 (5,192–10,420)	13,827 (9,710–18,475)	545 (361–762)	30,241 (23,457–37,950)
Caribbean	YLDs Number: Cumulative change (%)	-3.4 (-5.7 to -1.1)	-7.2 (-12.7 to -1.7)	-0.0 (-1.1 to 1.0)	-8.6 (-12.9 to -4.1)	-9.2 (-14.1 to -4.7)	-14.8 (-18.2 to -11.5)	17.6 (8.6–29.5)	-24.1 (-39.0 to -6.0)	-36.9 (-48.3 to -24.5)	-67.9 (-75.9 to -56.3)	-12.8 (-16.0 to -9.1)
Caribbean	YLDs Rate: 1990	6,844.1 (5,187.1–8,676.6)	1,332.0 (956.6–1,785.1)	875.8 (612.7–1,210.6)	790.2 (528.2–1,073.6)	76.7 (55.0–102.8)	193.8 (140.0–255.0)	95.9 (67.3–131.2)	62.9 (43.6–87.0)	143.8 (102.3–188.6)	11.2 (6.5–16.6)	224.9 (176.1–280.8)
Caribbean	YLDs Rate: 2015	6,566.2 (4,987.1–8,314.1)	1,248.2 (890.4–1,681.2)	875.2 (610.7–1,206.2)	720.8 (479.8–991.1)	69.8 (50.2–93.5)	177.3 (128.4–232.7)	113.4 (78.9–156.2)	52.5 (35.7–71.6)	89.7 (63.0–120.0)	3.6 (2.4–5.1)	198.3 (153.9–248.8)
Caribbean	YLDs Rate: Cumulative change (%)	-4.0 (-6.2 to -1.7)	-6.2 (-11.7 to -0.6)	-0.1 (-1.0 to 0.8)	-8.8 (-13.1 to -4.2)	-9.0 (-14.0 to -4.5)	-8.5 (-12.1 to -5.0)	18.4 (9.3–30.4)	-16.1 (-32.5 to 3.9)	-37.3 (-48.6 to -24.8)	-66.7 (-74.9 to -54.5)	-11.8 (-15.1 to -8.2)
Antigua and Barbuda	Prevalence Number: 1990	21,038 (20,837–21,241)	7,041 (6,443–7,596)	8,105 (7,841–8,335)	3,218 (2,674–3,777)	3,697 (2,944–5,431)	170 (159–181)	324 (281–380)	80 (52–116)	116 (86–149)	0 (0–0)	424 (373–484)
Antigua and Barbuda	Prevalence Number: 2015	27,369 (27,096–27,637)	8,541 (8,009–9,306)	10,960 (10,632–11,259)	4,133 (3,383–4,930)	4,267 (3,356–6,155)	224 (213–236)	421 (362–501)	71 (49–101)	115 (84–147)	0 (0–0)	505 (444–575)
Antigua and Barbuda	Prevalence Number: Cumulative change (%)	30.1 (28.4–31.7)	21.4 (13.3–30.4)	35.3 (30.0–40.5)	28.8 (10.9–48.0)	18.4 (-22.9 to 70.8)	31.6 (27.0–36.1)	30.7 (4.4–60.6)	-5.7 (-49.3 to 53.4)	1.7 (-31.0 to 42.3)	137.4 (5.0–591.3)	19.7 (4.3–36.8)
Antigua and Barbuda	Prevalence Rate: 1990	90,826.4 (89,968.3–91,708.2)	30,334.9 (27,686.8–32,807.5)	35,406.7 (34,268.5–36,419.0)	13,591.6 (11,300.1–15,880.2)	15,995.9 (12,736.4–23,488.4)	739.8 (693.7–790.3)	1,404.6 (1,217.8–1,643.3)	358.8 (231.7–517.3)	508.1 (374.9–650.0)	0.0 (0.0–0.0)	1,855.8 (1,638.4–2,119.6)
Antigua and Barbuda	Prevalence Rate: 2015	90,513.7 (89,571.7–91,424.9)	28,751.3 (26,886.7–31,518.5)	35,679.8 (34,624.6–36,655.5)	13,730.8 (11,240.9–16,429.0)	14,149.7 (11,128.2–20,418.2)	784.6 (745.1–829.8)	1,398.0 (1,203.0–1,661.7)	258.8 (176.4–365.2)	377.5 (275.9–480.4)	0.0 (0.0–0.0)	1,721.6 (1,520.6–1,953.9)
Antigua and Barbuda	Prevalence Rate: Cumulative change (%)	-0.3 (-1.6 to 0.9)	-5.1 (-11.5 to 1.9)	0.8 (-3.1 to 4.7)	1.3 (-13.0 to 16.5)	-9.3 (-40.8 to 30.9)	6.1 (2.4–9.7)	0.3 (-19.9 to 23.4)	-23.6 (-58.9 to 24.3)	-23.8 (-48.4 to 6.5)	99.3 (-13.5 to 488.7)	-6.9 (-18.7 to 6.1)
Antigua and Barbuda	YLDs Number: 1990	1,420 (1,079–1,793)	268 (189–361)	202 (140–278)	146 (96–200)	22 (16–30)	27 (19–37)	23 (16–32)	10 (5–15)	44 (27–61)	0 (0–0)	54 (42–68)
Antigua and Barbuda	YLDs Number: 2015	1,830 (1,383–2,307)	322 (227–432)	266 (184–371)	187 (122–262)	26 (19–36)	36 (26–48)	38 (26–52)	8 (5–13)	28 (18–40)	0 (0–0)	57 (44–72)
Antigua and Barbuda	YLDs Number: Cumulative change (%)	28.9 (25.2–32.6)	20.6 (10.1–31.9)	31.9 (29.1–34.7)	28.8 (10.7–48.0)	19.5 (10.1–30.2)	31.6 (24.6–38.9)	59.4 (37.0–85.6)	-5.6 (-49.8 to 54.8)	-33.2 (-55.7 to -3.5)	144.9 (-2.4 to 658.6)	5.0 (-1.5 to 12.4)
Antigua and Barbuda	YLDs Rate: 1990	6,161.0 (4,688.4–7,778.9)	1,147.6 (806.4–1,544.2)	874.0 (604.8–1,209.2)	616.9 (409.4–845.1)	97.2 (69.3–129.9)	121.1 (86.1–161.1)	103.5 (73.0–140.9)	44.6 (25.8–69.9)	192.6 (120.4–268.6)	0.0 (0.0–0.0)	235.0 (182.6–297.9)
Antigua and Barbuda	YLDs Rate: 2015	5,984.9 (4,522.0–7,549.3)	1,092.8 (767.5–1,466.0)	878.4 (609.6–1,217.0)	623.6 (405.9–874.7)	90.4 (64.8–120.8)	128.4 (92.5–170.5)	126.4 (87.4–175.3)	32.3 (18.5–50.1)	93.8 (60.4–133.1)	0.0 (0.0–0.0)	189.8 (146.1–239.8)

© 2017 Global Burden of Disease Child and Adolescent Health Collaboration. JAMA Pediatrics.

eTable 3. Prevalent Cases, Rates (per 100,000 population), Years Lived with Disability (YLDs), and Cumulative Percent Change with 95% Uncertainty Interval (UI) for the Top 10 Global Causes of YLDs in Children and Adolescents in 195 Countries and Territories, Aged 0 to 19 Years, Both Sexes, 1990 and 2015. *Note: best viewed by enlarging in browser.*

Location	Measure	All causes	Iron-deficiency anemia	Skin and subcutaneous diseases	Asthma	Hemoglobinopathies and hemolytic anemias	Diarrheal diseases	Congenital anomalies	Protein-energy malnutrition	Epilepsy	Malaria	Neonatal preterm birth complications
Antigua and Barbuda	YLDs Rate: Cumulative change (%)	-2.9 (-5.7 to 0.1)	-4.7 (-13.1 to 4.5)	0.5 (-1.2 to 2.4)	1.3 (-12.9 to 16.8)	-6.9 (-14.2 to 1.4)	6.2 (0.5–12.1)	22.4 (5.1–42.4)	-23.5 (-59.3 to 25.6)	-49.9 (-66.9 to -27.6)	109.2 (-18.4 to 561.9)	-19.2 (-24.2 to -13.6)
The Bahamas	Prevalence Number: 1990	100,441 (99,475–101,279)	33,496 (31,184–35,747)	39,117 (37,825–40,188)	18,121 (15,109–21,425)	11,550 (10,871–12,328)	805 (755–861)	1,521 (1,308–1,820)	403 (263–570)	368 (274–468)	0 (0–0)	2,385 (2,108–2,719)
The Bahamas	Prevalence Number: 2015	100,344 (99,296–101,230)	32,900 (30,746–35,298)	40,095 (38,858–41,220)	15,944 (13,216–18,855)	9,842 (9,231–10,528)	841 (796–891)	1,548 (1,339–1,825)	314 (208–450)	305 (223–395)	0 (0–0)	2,281 (2,011–2,616)
The Bahamas	Prevalence Number: Cumulative change (%)	-0.1 (-1.3 to 1.1)	-1.6 (-9.8 to 11.1)	2.5 (-1.2 to 6.5)	-11.9 (-21.2 to -3.1)	-14.7 (-21.5 to -7.3)	4.5 (1.3–7.8)	2.6 (-17.8 to 25.4)	-17.7 (-53.6 to 36.9)	-15.4 (-42.1 to 19.8)	91.4 (-16.4 to 461.8)	-4.0 (-16.2 to 9.4)
The Bahamas	Prevalence Rate: 1990	90,558.6 (89,696.8–91,311.1)	30,216.8 (28,126.7–32,248.2)	35,358.4 (34,195.6–36,324.3)	16,312.9 (13,613.0–19,292.3)	10,424.7 (9,810.1–11,123.0)	722.7 (677.8–773.3)	1,372.3 (1,179.6–1,641.2)	363.3 (237.4–513.4)	333.2 (248.2–423.3)	0.0 (0.0–0.0)	2,148.5 (1,898.2–2,450.1)
The Bahamas	Prevalence Rate: 2015	90,325.7 (89,379.8–91,134.5)	30,156.2 (28,157.3–32,384.4)	35,631.3 (34,535.9–36,637.4)	14,534.2 (12,009.5–17,261.5)	8,892.3 (8,344.4–9,507.7)	777.5 (735.6–824.7)	1,396.6 (1,207.2–1,646.1)	292.6 (194.1–418.9)	272.8 (199.9–353.5)	0.0 (0.0–0.0)	2,078.5 (1,833.6–2,378.9)
The Bahamas	Prevalence Rate: Cumulative change (%)	-0.3 (-1.5 to 1.0)	0.0 (-8.9 to 12.8)	0.8 (-2.9 to 4.8)	-10.8 (-20.2 to -1.8)	-14.6 (-21.4 to -7.2)	7.6 (4.2–10.9)	2.6 (-17.7 to 25.3)	-14.9 (-52.0 to 41.5)	-16.3 (-42.9 to 18.6)	99.3 (-13.8 to 488.3)	-2.9 (-15.3 to 10.4)
The Bahamas	YLDs Number: 1990	7,076 (5,381–8,993)	1,267 (895–1,732)	966 (666–1,341)	821 (547–1,133)	62 (44–86)	131 (93–174)	120 (82–168)	50 (29–79)	140 (89–194)	0 (0–0)	351 (273–440)
The Bahamas	YLDs Number: 2015	6,866 (5,159–8,662)	1,240 (875–1,701)	976 (676–1,365)	723 (482–993)	58 (41–79)	137 (98–181)	141 (94–196)	39 (22–63)	75 (48–107)	0 (0–0)	284 (221–356)
The Bahamas	YLDs Number: Cumulative change (%)	-3.0 (-6.2 to 0.9)	-1.9 (-13.5 to 16.2)	1.0 (-0.8 to 2.9)	-11.8 (-21.1 to -2.8)	-6.4 (-16.2 to 5.1)	4.4 (-0.8 to 9.8)	17.8 (-0.2 to 38.9)	-17.8 (-54.2 to 38.2)	-44.8 (-63.4 to -21.7)	102.8 (-19.5 to 514.7)	-18.9 (-24.3 to -13.6)
The Bahamas	YLDs Rate: 1990	6,388.3 (4,859.5–8,121.0)	1,146.9 (809.8–1,566.5)	872.4 (601.1–1,211.3)	739.9 (492.7–1,019.8)	57.1 (40.6–78.6)	118.3 (84.0–156.6)	108.8 (74.5–151.7)	45.3 (26.4–71.8)	126.8 (80.8–176.1)	0.0 (0.0–0.0)	316.8 (246.8–397.5)
The Bahamas	YLDs Rate: 2015	6,135.1 (4,613.1–7,748.4)	1,154.1 (814.9–1,581.1)	877.3 (608.1–1,223.4)	659.7 (437.5–906.7)	54.8 (39.2–74.5)	127.1 (91.4–168.3)	127.9 (85.5–177.4)	36.5 (20.9–59.4)	67.6 (43.5–96.6)	0.0 (0.0–0.0)	257.5 (200.1–322.3)
The Bahamas	YLDs Rate: Cumulative change (%)	-4.0 (-7.2 to -0.1)	1.0 (-11.2 to 19.4)	0.6 (-1.1 to 2.3)	-10.7 (-20.3 to -1.4)	-3.8 (-14.2 to 8.0)	7.5 (2.1–13.1)	17.9 (-0.1 to 38.8)	-15.0 (-52.6 to 42.9)	-45.4 (-63.7 to -22.4)	112.6 (-16.4 to 547.2)	-18.7 (-24.0 to -13.2)
Barbados	Prevalence Number: 1990	80,351 (79,584–81,019)	26,289 (24,603–27,817)	30,654 (29,712–31,591)	13,416 (11,851–15,259)	14,332 (11,560–20,017)	545 (506–590)	1,208 (1,032–1,420)	310 (213–444)	365 (269–465)	0 (0–0)	1,637 (1,438–1,897)
Barbados	Prevalence Number: 2015	66,272 (65,609–66,923)	21,290 (19,945–22,652)	25,261 (24,496–25,991)	10,317 (8,732–12,137)	10,598 (8,193–16,257)	435 (405–463)	1,029 (891–1,208)	391 (278–526)	229 (167–293)	0 (0–0)	1,274 (1,129–1,453)
Barbados	Prevalence Number: Cumulative change (%)	-17.5 (-18.5 to -16.5)	-18.9 (-25.7 to -12.1)	-17.6 (-20.7 to -14.4)	-23.1 (-31.5 to -13.7)	-24.3 (-50.3 to 12.7)	-20.2 (-23.2 to -16.9)	-14.1 (-30.5 to 4.5)	32.8 (-22.5 to 106.3)	-35.4 (-56.3 to -9.7)	67.6 (-26.9 to 392.0)	-21.8 (-33.0 to -10.9)
Barbados	Prevalence Rate: 1990	90,698.5 (89,806.1–91,485.3)	30,312.4 (28,266.8–32,100.7)	33,713.6 (32,653.4–34,767.2)	15,213.2 (13,437.6–17,394.1)	16,240.4 (13,097.8–22,675.5)	671.8 (622.3–728.7)	1,372.1 (1,172.8–1,610.1)	394.1 (270.1–563.7)	404.5 (298.6–515.2)	0.0 (0.0–0.0)	1,920.0 (1,693.0–2,218.2)
Barbados	Prevalence Rate: 2015	90,432.2 (89,496.5–91,357.5)	29,510.0 (27,550.4–31,583.2)	33,979.6 (32,939.7–34,974.9)	13,912.7 (11,752.9–16,378.4)	14,506.7 (11,213.9–22,255.0)	646.5 (600.9–690.3)	1,411.2 (1,220.9–1,657.1)	608.3 (432.8–817.2)	309.7 (225.7–396.3)	0.0 (0.0–0.0)	1,811.2 (1,607.4–2,058.4)
Barbados	Prevalence Rate: Cumulative change (%)	-0.3 (-1.6 to 1.0)	-2.5 (-10.9 to 5.9)	0.8 (-3.0 to 4.8)	-8.5 (-18.7 to 2.9)	-8.5 (-40.0 to 36.2)	-3.7 (-7.5 to 0.2)	3.7 (-16.1 to 26.2)	62.7 (-5.1 to 152.8)	-21.4 (-46.7 to 10.1)	99.3 (-13.7 to 488.4)	-5.3 (-18.4 to 7.4)
Barbados	YLDs Number: 1990	5,466 (4,155–6,923)	982 (697–1,329)	653 (445–920)	609 (409–827)	83 (59–114)	89 (64–117)	94 (64–130)	38 (22–61)	138 (90–193)	0 (0–0)	240 (188–301)
Barbados	YLDs Number: 2015	4,380 (3,326–5,573)	808 (567–1,102)	540 (369–759)	468 (313–647)	67 (47–91)	71 (50–94)	93 (61–130)	48 (29–71)	57 (36–81)	0 (0–0)	166 (129–209)
Barbados	YLDs Number: Cumulative change (%)	-19.9 (-22.1 to -17.5)	-17.6 (-24.6 to -9.1)	-17.2 (-18.8 to -15.6)	-23.1 (-31.6 to -13.7)	-19.8 (-28.5 to -11.2)	-20.2 (-25.0 to -15.4)	-1.5 (-14.2 to 13.3)	32.8 (-22.9 to 104.5)	-57.5 (-72.4 to -38.8)	77.0 (-32.4 to 468.2)	-30.8 (-34.8 to -26.5)
Barbados	YLDs Rate: 1990	6,066.9 (4,598.0–7,703.8)	1,150.7 (815.3–1,558.9)	729.0 (500.1–1,019.6)	691.4 (463.7–942.7)	97.7 (69.5–133.2)	110.0 (78.9–145.4)	107.7 (74.1–148.5)	49.3 (28.1–78.5)	153.4 (99.8–215.7)	0.0 (0.0–0.0)	274.3 (215.2–343.8)
Barbados	YLDs Rate: 2015	5,910.1 (4,492.9–7,523.1)	1,114.9 (783.6–1,515.8)	733.3 (502.2–1,026.5)	631.8 (420.7–876.2)	91.5 (64.4–125.2)	105.8 (74.7–140.7)	127.9 (85.1–178.7)	75.8 (46.1–111.2)	77.1 (48.9–109.1)	0.0 (0.0–0.0)	228.5 (177.9–286.8)
Barbados	YLDs Rate: Cumulative change (%)	-2.6 (-5.4 to 0.5)	-3.0 (-11.8 to 7.1)	0.6 (-1.2 to 2.5)	-8.5 (-18.7 to 3.2)	-6.2 (-16.7 to 3.5)	-3.7 (-9.7 to 2.1)	18.9 (3.5–36.9)	62.7 (-5.5 to 150.6)	-48.3 (-66.4 to -25.8)	108.1 (-20.0 to 562.7)	-16.7 (-21.4 to -11.7)
Belize	Prevalence Number: 1990	96,341 (95,644–97,055)	33,217 (31,140–36,877)	34,813 (33,712–35,821)	17,273 (14,376–20,430)	16,139 (13,154–22,010)	1,087 (984–1,191)	1,266 (1,075–1,535)	520 (348–748)	394 (289–503)	133 (92–176)	2,454 (2,165–2,871)
Belize	Prevalence Number: 2015	141,052 (139,628–142,373)	45,839 (42,720–48,919)	54,635 (52,834–56,241)	22,704 (18,815–27,012)	21,960 (17,110–32,514)	1,259 (1,160–1,377)	2,042 (1,757–2,434)	523 (377–693)	487 (361–628)	6 (2–22)	3,160 (2,770–3,678)
Belize	Prevalence Number: Cumulative change (%)	46.4 (44.8–48.0)	38.4 (21.6–52.8)	57.0 (50.8–63.0)	31.9 (15.8–48.1)	39.0 (-8.1 to 105.5)	16.0 (7.9–23.4)	63.0 (28.9–104.0)	6.1 (-37.5 to 71.1)	26.8 (-13.3 to 79.0)	-95.0 (-98.2 to -83.0)	29.4 (9.5–51.3)
Belize	Prevalence Rate: 1990	93,307.4 (92,671.8–93,954.0)	31,557.1 (29,815.1–34,795.8)	34,527.4 (33,462.6–35,512.4)	16,711.3 (13,970.3–19,679.5)	15,579.9 (12,709.0–21,247.1)	946.6 (860.6–1,035.4)	1,218.1 (1,034.4–1,480.0)	435.5 (291.6–625.2)	388.0 (284.7–496.8)	126.0 (88.5–164.9)	2,244.5 (1,971.8–2,638.8)
Belize	Prevalence Rate: 2015	91,267.3 (90,334.3–92,152.3)	29,904.9 (27,769.9–32,021.2)	35,043.4 (33,902.2–36,085.8)	14,729.6 (12,206.6–17,506.8)	14,220.9 (11,075.9–21,051.5)	838.5 (773.0–918.4)	1,323.5 (1,138.3–1,576.9)	354.8 (255.5–469.5)	312.9 (233.1–402.7)	4.3 (1.5–15.5)	2,066.8 (1,814.0–2,403.9)
Belize	Prevalence Rate: Cumulative change (%)	-2.2 (-3.3 to -1.1)	-5.0 (-15.8 to 5.2)	1.5 (-2.5 to 5.4)	-11.6 (-22.1 to -0.7)	-6.8 (-38.4 to 37.9)	-11.3 (-17.4 to -5.8)	9.8 (-13.3 to 37.5)	-14.1 (-49.4 to 38.4)	-17.3 (-43.4 to 17.0)	-96.5 (-98.8 to -88.2)	-7.4 (-22.2 to 8.8)
Belize	YLDs Number: 1990	6,677 (5,041–8,502)	1,271 (889–1,749)	883 (617–1,213)	782 (516–1,083)	97 (68–133)	177 (126–235)	97 (67–136)	64 (38–102)	147 (94–209)	2 (1–4)	291 (227–366)
Belize	YLDs Number: 2015	9,565 (7,238–12,154)	1,745 (1,226–2,349)	1,342 (929–1,859)	1,030 (684–1,428)	139 (98–188)	205 (145–274)	207 (139–291)	65 (39–98)	123 (78–177)	0 (0–0)	339 (263–427)
Belize	YLDs Number: Cumulative change (%)	43.4 (37.5–49.0)	38.0 (18.1–57.2)	51.9 (48.4–55.4)	32.1 (15.9–48.6)	42.9 (25.6–61.3)	16.0 (6.7–25.1)	113.8 (78.9–156.2)	6.2 (-37.6 to 70.4)	-14.2 (-43.6 to 25.8)	-91.6 (-97.6 to -70.3)	16.8 (8.3–25.1)
Belize	YLDs Rate: 1990	6,572.1 (4,971.4–8,369.3)	1,206.7 (848.2–1,650.6)	859.4 (596.9–1,187.6)	757.2 (499.8–1,040.7)	93.8 (66.3–127.7)	154.4 (110.4–204.5)	93.4 (64.4–131.1)	54.2 (32.2–85.9)	144.8 (92.7–204.6)	2.6 (1.5–4.1)	278.6 (218.0–350.9)
Belize	YLDs Rate: 2015	6,150.7 (4,654.0–7,823.9)	1,140.4 (800.4–1,534.8)	867.8 (600.4–1,199.1)	668.5 (443.6–928.1)	90.5 (64.1–122.8)	136.9 (97.1–182.7)	134.2 (90.2–189.0)	44.2 (26.6–66.7)	79.0 (50.1–114.0)	0.1 (0.0–0.5)	220.4 (170.6–277.1)
Belize	YLDs Rate: Cumulative change (%)	-6.4 (-10.0 to -2.8)	-5.1 (-18.1 to 7.9)	0.9 (-0.9 to 2.8)	-11.4 (-22.1 to -0.4)	-3.2 (-14.7 to 9.5)	-11.3 (-18.3 to -4.5)	44.3 (20.9–73.2)	-14.0 (-49.5 to 37.8)	-44.0 (-63.1 to -17.6)	-94.1 (-98.3 to -78.8)	-20.9 (-26.6 to -15.1)
Bermuda	Prevalence Number: 1990	20,162 (19,976–20,333)	6,408 (6,057–6,729)	8,145 (7,898–8,396)	3,364 (2,846–3,919)	3,331 (2,599–4,761)	101 (96–106)	319 (279–370)	50 (33–71)	72 (53–93)	0 (0–0)	298 (261–344)
Bermuda	Prevalence Number: 2015	19,550 (19,359–19,719)	6,114 (5,851–6,417)	8,027 (7,800–8,253)	3,011 (2,474–3,627)	2,872 (2,196–4,363)	93 (89–97)	306 (267–360)	35 (23–49)	56 (40–73)	0 (0–0)	242 (208–283)
Bermuda	Prevalence Number: Cumulative change (%)	-3.0 (-4.2 to -1.9)	-4.5 (-11.6 to 3.1)	-1.4 (-5.1 to 2.4)	-10.3 (-23.2 to 4.5)	-10.9 (-45.5 to 36.8)	-7.9 (-11.0 to -5.0)	-3.6 (-20.5 to 15.4)	-26.4 (-58.4 to 20.1)	-20.0 (-44.9 to 15.9)	84.3 (-18.9 to 443.7)	-18.6 (-30.4 to -4.5)
Bermuda	Prevalence Rate: 1990	90,490.1 (89,573.8–91,317.4)	29,390.6 (27,171.6–31,190.0)	35,988.2 (34,883.3–37,097.4)	14,493.8 (12,241.4–16,930.6)	15,068.6 (11,746.4–21,560.7)	548.4 (520.1–576.6)	1,448.0 (1,266.3–1,682.4)	307.4 (206.8–441.3)	316.7 (234.7–408.1)	0.0 (0.0–0.0)	1,448.4 (1,276.8–1,655.4)
Bermuda	Prevalence Rate: 2015	90,519.7 (89,637.8–91,340.5)	29,869.5 (28,310.8–31,821.8)	36,147.7 (35,127.4–37,197.6)	13,397.1 (11,006.6–16,137.9)	13,426.6 (10,280.9–20,397.5)	548.0 (523.2–574.2)	1,431.4 (1,250.3–1,681.8)	240.6 (161.3–340.1)	256.3 (186.6–335.2)	0.0 (0.0–0.0)	1,245.2 (1,082.0–1,432.2)
Bermuda	Prevalence Rate: Cumulative change (%)	0.0 (-1.2 to 1.3)	1.8 (-7.1 to 12.0)	0.5 (-3.3 to 4.5)	-7.4 (-20.9 to 7.5)	-8.0 (-43.8 to 41.2)	-0.1 (-3.6 to 3.1)	-0.6 (-18.0 to 19.0)	-17.6 (-53.4 to 34.6)	-17.2 (-43.1 to 19.3)	99.3 (-13.6 to 488.5)	-13.8 (-25.4 to -0.4)
Bermuda	YLDs Number: 1990	1,390 (1,048–1,758)	244 (174–327)	196 (136–272)	152 (102–209)	22 (16–29)	16 (11–21)	25 (17–34)	6 (3–9)	28 (18–41)	0 (0–0)	41 (31–53)
Bermuda	YLDs Number: 2015	1,333 (1,014–1,686)	237 (169–320)	191 (132–267)	136 (90–191)	20 (14–28)	15 (10–19)	38 (24–57)	4 (2–6)	14 (8–20)	0 (0–0)	26 (20–34)
Bermuda	YLDs Number: Cumulative change (%)	-4.1 (-6.9 to -1.1)	-2.6 (-11.1 to 7.3)	-2.3 (-4.4 to -0.5)	-10.2 (-23.2 to 4.2)	-7.1 (-15.5 to 1.8)	-8.1 (-13.4 to -2.5)	54.1 (17.8–95.9)	-26.4 (-58.5 to 19.5)	-48.5 (-66.4 to -24.0)	100.5 (-20.3 to 515.2)	-34.3 (-40.1 to -28.1)
Bermuda	YLDs Rate: 1990	6,098.5 (4,592.8–7,732.4)	1,101.0 (782.6–1,492.9)	880.0 (611.2–1,218.2)	657.7 (442.3–900.7)	99.1 (71.2–132.4)	89.9 (64.3–119.1)	114.6 (77.4–158.7)	38.4 (21.2–58.8)	125.0 (81.2–180.0)	0.0 (0.0–0.0)	185.8 (143.3–239.7)
Bermuda	YLDs Rate: 2015	6,020.7 (4,551.8–7,610.2)	1,147.6 (813.2–1,550.6)	883.4 (613.6–1,225.7)	608.3 (402.1–850.8)	96.7 (69.0–131.5)	89.6 (64.9–117.5)	182.2 (114.1–272.2)	30.0 (17.4–47.8)	64.8 (41.1–93.2)	0.0 (0.0–0.0)	125.3 (95.6–161.7)
Bermuda	YLDs Rate: Cumulative change (%)	-1.3 (-4.6 to 2.3)	4.5 (-6.4 to 17.7)	0.4 (-1.6 to 2.3)	-7.3 (-20.8 to 7.7)	-2.3 (-11.5 to 8.8)	-0.2 (-5.9 to 5.9)	59.1 (21.6–102.1)	-17.6 (-53.6 to 33.9)	-46.7 (-65.1 to -21.5)	117.3 (-14.8 to 580.5)	-32.5 (-38.3 to -26.3)
Cuba	Prevalence Number: 1990	3,266,121 (3,239,129–3,289,464)	1,025,065 (950,759–1,116,774)	1,302,047 (1,263,275–1,341,319)	461,726 (382,556–541,685)	317,956 (298,487–339,065)	17,791 (16,770–18,806)	50,780 (43,817–60,185)	11,585 (7,831–16,006)	10,310 (7,657–13,134)	0 (0–0)	63,749 (56,199–73,253)

© 2017 Global Burden of Disease Child and Adolescent Health Collaboration. JAMA Pediatrics.

eTable 3. Prevalent Cases, Rates (per 100,000 population), Years Lived with Disability (YLDs), and Cumulative Percent Change with 95% Uncertainty Interval (UI) for the Top 10 Global Causes of YLDs in Children and Adolescents in 195 Countries and Territories, Aged 0 to 19 Years, Both Sexes, 1990 and 2015. *Note: best viewed by enlarging in browser.*

Location	Measure	All causes	Iron-deficiency anemia	Skin and subcutaneous diseases	Asthma	Hemoglobinopathies and hemolytic anemias	Diarrheal diseases	Congenital anomalies	Protein-energy malnutrition	Epilepsy	Malaria	Neonatal preterm birth complications
Cuba	Prevalence Number: 2015	2,352,041 (2,332,593–2,368,755)	663,984 (612,475–733,740)	943,033 (916,580–968,021)	357,630 (294,328–424,165)	198,157 (186,815–210,030)	11,885 (11,188–12,539)	36,121 (31,460–42,393)	5,850 (4,176–7,781)	6,513 (4,753–8,316)	0 (0–0)	40,841 (35,409–47,572)
Cuba	Prevalence Number: Cumulative change (%)	-28.0 (-28.7 to -27.3)	-35.1 (-41.5 to -26.3)	-27.6 (-30.1 to -24.8)	-22.4 (-28.9 to -12.8)	-37.6 (-42.5 to -32.4)	-33.2 (-35.3 to -30.5)	-28.3 (-42.1 to -14.0)	-47.0 (-68.5 to -17.7)	-35.4 (-55.4 to -9.9)	43.9 (-37.0 to 320.5)	-35.7 (-45.3 to -24.8)
Cuba	Prevalence Rate: 1990	92,311.7 (91,509.9–92,991.7)	29,774.8 (27,803.9–32,499.7)	35,803.1 (34,754.1–36,859.8)	13,343.1 (10,957.5–15,708.6)	9,019.8 (8,471.2–9,611.5)	529.7 (499.2–560.2)	1,439.7 (1,241.1–1,707.0)	346.6 (234.3–478.9)	286.1 (211.6–365.1)	0.0 (0.0–0.0)	1,844.7 (1,628.3–2,115.2)
Cuba	Prevalence Rate: 2015	91,767.6 (90,940.8–92,453.1)	26,323.2 (24,228.2–29,309.0)	36,054.7 (35,053.8–37,027.1)	13,928.9 (11,431.8–16,556.4)	7,757.7 (7,313.7–8,217.5)	505.4 (475.2–534.0)	1,416.2 (1,230.8–1,663.0)	261.3 (186.6–347.5)	251.7 (183.8–322.4)	0.0 (0.0–0.0)	1,685.1 (1,471.4–1,945.4)
Cuba	Prevalence Rate: Cumulative change (%)	-0.6 (-1.7 to 0.5)	-11.4 (-20.2 to 1.3)	0.7 (-3.0 to 4.4)	4.6 (-4.4 to 17.3)	-13.9 (-20.6 to -6.8)	-4.6 (-7.7 to -0.7)	-0.9 (-19.7 to 18.9)	-20.9 (-53.0 to 22.8)	-10.1 (-37.8 to 25.3)	99.3 (-13.7 to 488.3)	-8.3 (-21.5 to 6.2)
Cuba	YLDs Number: 1990	217,337 (166,200–275,846)	37,738 (26,782–51,540)	32,562 (22,833–44,810)	20,938 (13,963–29,167)	1,899 (1,359–2,563)	2,912 (2,091–3,854)	4,003 (2,607–5,687)	1,443 (860–2,253)	3,884 (2,527–5,434)	0 (0–0)	7,507 (5,832–9,587)
Cuba	YLDs Number: 2015	152,403 (115,830–193,246)	24,864 (17,673–33,944)	23,710 (16,520–32,666)	16,235 (10,797–22,345)	1,271 (905–1,731)	1,951 (1,394–2,579)	3,451 (2,122–5,194)	731 (435–1,084)	1,636 (1,066–2,362)	0 (0–0)	4,102 (3,173–5,257)
Cuba	YLDs Number: Cumulative change (%)	-29.9 (-32.0 to -26.9)	-33.9 (-40.9 to -21.9)	-27.2 (-28.5 to -25.8)	-22.3 (-28.7 to -12.9)	-32.9 (-40.8 to -21.2)	-33.0 (-37.0 to -28.7)	-14.0 (-29.3 to 8.7)	-46.9 (-68.5 to -16.5)	-56.9 (-71.0 to -37.8)	45.0 (-45.7 to 375.5)	-45.3 (-49.6 to -41.0)
Cuba	YLDs Rate: 1990	6,046.4 (4,626.8–7,672.3)	1,136.1 (811.4–1,543.2)	917.5 (645.2–1,258.4)	605.2 (401.3–844.6)	57.2 (41.0–77.0)	86.7 (62.1–114.2)	113.6 (73.9–160.5)	43.2 (25.7–67.4)	107.8 (70.2–151.3)	0.0 (0.0–0.0)	213.8 (166.3–273.4)
Cuba	YLDs Rate: 2015	5,837.0 (4,431.6–7,410.6)	990.8 (704.8–1,359.4)	922.3 (644.5–1,265.4)	632.5 (420.8–869.1)	50.1 (35.7–68.4)	83.0 (59.3–110.0)	135.5 (83.2–203.4)	32.7 (19.5–48.4)	63.3 (41.3–91.3)	0.0 (0.0–0.0)	160.8 (124.1–206.1)
Cuba	YLDs Rate: Cumulative change (%)	-3.4 (-6.6 to 0.9)	-12.5 (-21.7 to 4.1)	0.5 (-1.3 to 2.3)	4.8 (-3.9 to 17.5)	-12.2 (-22.2 to 3.2)	-4.2 (-9.9 to 1.9)	19.0 (-2.3 to 50.8)	-20.7 (-52.9 to 24.7)	-40.0 (-59.5 to -13.7)	96.6 (-25.9 to 534.7)	-24.7 (-30.5 to -18.8)
Dominica	Prevalence Number: 1990	27,720 (27,505–27,924)	9,000 (8,452–9,672)	10,351 (10,015–10,638)	4,806 (3,989–5,676)	4,756 (3,835–6,784)	291 (264–316)	413 (358–493)	120 (81–171)	172 (128–225)	0 (0–0)	632 (559–735)
Dominica	Prevalence Number: 2015	21,894 (21,690–22,083)	6,610 (6,240–6,914)	8,638 (8,362–8,901)	3,977 (3,319–4,678)	3,460 (2,713–4,918)	222 (202–242)	334 (287–400)	56 (37–81)	130 (97–167)	0 (0–0)	477 (420–552)
Dominica	Prevalence Number: Cumulative change (%)	-21.0 (-21.9 to -20.1)	-26.4 (-34.2 to -20.8)	-16.5 (-19.7 to -13.1)	-17.1 (-25.2 to -8.6)	-25.6 (-50.1 to 7.9)	-23.7 (-27.4 to -20.3)	-18.4 (-34.7 to -0.2)	-50.6 (-72.1 to -23.5)	-22.4 (-46.8 to 9.0)	50.7 (-33.9 to 338.1)	-24.1 (-34.6 to -12.8)
Dominica	Prevalence Rate: 1990	93,176.4 (92,474.5–93,841.8)	30,195.4 (28,425.2–32,356.8)	34,918.3 (33,804.2–35,897.0)	16,223.8 (13,470.2–19,152.2)	15,979.7 (12,889.5–22,788.1)	951.8 (866.1–1,034.7)	1,388.3 (1,204.1–1,656.5)	387.8 (262.5–551.7)	583.2 (435.2–762.1)	0.0 (0.0–0.0)	2,106.5 (1,862.7–2,451.1)
Dominica	Prevalence Rate: 2015	90,841.4 (89,969.9–91,645.7)	27,851.5 (26,225.4–29,271.1)	35,350.0 (34,208.8–36,430.3)	16,382.6 (13,615.8–19,340.1)	14,405.4 (11,298.0–20,469.0)	999.6 (907.4–1,095.9)	1,394.4 (1,198.8–1,666.3)	268.9 (176.4–384.2)	533.3 (397.2–684.6)	0.0 (0.0–0.0)	2,067.8 (1,828.2–2,382.3)
Dominica	Prevalence Rate: Cumulative change (%)	-2.5 (-3.6 to -1.4)	-7.6 (-17.8 to -0.6)	1.3 (-2.6 to 5.4)	1.2 (-9.0 to 11.5)	-7.9 (-38.1 to 33.7)	5.0 (-0.2 to 9.7)	1.3 (-18.9 to 23.8)	-27.4 (-59.0 to 12.5)	-6.2 (-35.6 to 31.3)	99.3 (-13.7 to 488.3)	-1.4 (-15.0 to 12.8)
Dominica	YLDs Number: 1990	1,926 (1,459–2,450)	340 (239–464)	257 (178–357)	217 (144–297)	27 (19–37)	47 (33–63)	32 (22–44)	14 (8–22)	64 (42–89)	0 (0–0)	76 (58–95)
Dominica	YLDs Number: 2015	1,526 (1,151–1,935)	248 (177–337)	210 (146–291)	180 (117–249)	21 (15–28)	36 (26–48)	29 (20–41)	7 (4–10)	32 (20–47)	0 (0–0)	60 (46–75)
Dominica	YLDs Number: Cumulative change (%)	-20.8 (-24.1 to -18.3)	-26.6 (-37.1 to -19.4)	-18.1 (-19.7 to -16.4)	-17.0 (-25.5 to -8.4)	-23.9 (-33.4 to -14.7)	-23.6 (-28.1 to -19.1)	-8.5 (-21.4 to 5.3)	-50.4 (-71.8 to -22.7)	-47.9 (-65.1 to -26.4)	56.8 (-38.6 to 373.9)	-20.9 (-25.4 to -16.1)
Dominica	YLDs Rate: 1990	6,504.7 (4,922.5–8,271.6)	1,148.3 (809.9–1,560.7)	864.8 (600.5–1,200.4)	735.2 (486.9–1,003.5)	94.9 (67.7–127.5)	155.4 (110.8–206.4)	110.2 (76.0–150.7)	48.1 (27.5–73.5)	217.6 (144.8–301.8)	0.0 (0.0–0.0)	255.6 (197.9–322.1)
Dominica	YLDs Rate: 2015	6,258.0 (4,723.4–7,942.9)	1,044.6 (744.7–1,419.9)	872.2 (605.5–1,204.2)	743.7 (485.3–1,027.8)	88.1 (63.0–118.9)	163.5 (117.2–216.1)	125.1 (86.5–174.1)	33.4 (19.5–51.0)	133.9 (83.9–193.6)	0.0 (0.0–0.0)	251.8 (193.2–317.3)
Dominica	YLDs Rate: Cumulative change (%)	-3.8 (-7.7 to -0.9)	-8.7 (-21.8 to -0.2)	0.9 (-1.0 to 2.8)	1.3 (-8.9 to 11.8)	-6.9 (-18.4 to 3.8)	5.2 (-1.0 to 11.4)	13.8 (-2.1 to 30.9)	-27.2 (-58.6 to 13.5)	-37.0 (-57.6 to -11.4)	102.9 (-22.1 to 515.3)	-1.5 (-6.9 to 4.6)
Dominican Republic	Prevalence Number: 1990	3,262,972 (3,236,888–3,288,894)	1,147,184 (1,052,292–1,226,319)	1,223,034 (1,184,795–1,257,429)	629,245 (530,158–736,765)	413,834 (386,587–441,543)	62,355 (57,977–67,646)	41,129 (34,641–49,602)	11,079 (8,866–13,606)	12,828 (9,592–16,401)	15,926 (8,602–30,452)	76,277 (66,420–88,527)
Dominican Republic	Prevalence Number: 2015	3,743,551 (3,710,419–3,777,142)	1,201,629 (1,091,909–1,291,403)	1,454,585 (1,411,670–1,495,785)	628,304 (521,295–744,341)	405,406 (380,398–428,577)	43,748 (41,270–46,374)	52,080 (44,408–61,982)	10,090 (7,669–12,892)	11,450 (8,287–15,179)	15,532 (8,533–31,844)	79,441 (69,891–91,564)
Dominican Republic	Prevalence Number: Cumulative change (%)	14.7 (13.4–16.1)	5.1 (-8.1 to 19.3)	19.0 (14.1–23.9)	-0.1 (-8.4 to 8.5)	-1.9 (-9.9 to 6.6)	-29.7 (-35.6 to -25.3)	27.9 (0.5–58.8)	-7.5 (-35.5 to 26.1)	-8.7 (-39.3 to 28.3)	1.8 (-51.5 to 51.6)	4.8 (-12.6 to 22.1)
Dominican Republic	Prevalence Rate: 1990	91,435.8 (90,716.6–92,150.0)	31,878.6 (29,377.9–34,005.1)	34,663.4 (33,578.0–35,641.3)	17,513.3 (14,755.6–20,491.3)	11,582.0 (10,826.2–12,357.8)	1,665.9 (1,549.6–1,806.2)	1,148.4 (966.8–1,385.1)	292.1 (233.7–358.7)	361.8 (271.7–462.2)	432.2 (236.6–823.1)	2,080.1 (1,806.2–2,426.2)
Dominican Republic	Prevalence Rate: 2015	90,580.7 (89,777.0–91,395.6)	29,134.1 (26,439.7–31,324.3)	35,150.8 (34,110.8–36,147.0)	15,134.6 (12,549.3–17,915.0)	9,814.6 (9,208.6–10,376.8)	1,074.0 (1,013.2–1,139.8)	1,261.5 (1,075.6–1,501.1)	250.1 (190.1–319.6)	276.8 (200.4–366.7)	377.4 (207.7–776.2)	1,947.2 (1,714.7–2,241.6)
Dominican Republic	Prevalence Rate: Cumulative change (%)	-0.9 (-2.0 to 0.2)	-8.4 (-19.7 to 3.7)	1.4 (-2.7 to 5.6)	-13.5 (-20.7 to -6.0)	-15.1 (-22.0 to -7.8)	-35.4 (-40.8 to -31.4)	10.9 (-12.8 to 37.7)	-13.0 (-39.4 to 18.5)	-21.7 (-48.0 to 9.9)	-9.0 (-56.4 to 37.3)	-5.7 (-21.6 to 9.5)
Dominican Republic	YLDs Number: 1990	236,710 (178,525–300,004)	44,197 (30,761–60,776)	30,645 (21,279–42,299)	28,518 (18,870–39,269)	2,329 (1,650–3,159)	10,178 (7,355–13,376)	3,247 (2,246–4,463)	1,365 (891–1,895)	4,798 (3,087–6,721)	245 (161–346)	9,221 (7,110–11,629)
Dominican Republic	YLDs Number: 2015	252,987 (190,583–322,238)	45,893 (32,360–62,099)	35,969 (25,006–49,666)	28,515 (18,686–39,552)	2,505 (1,762–3,383)	7,142 (5,129–9,426)	4,597 (3,172–6,288)	1,256 (786–1,856)	2,871 (1,788–4,138)	254 (169–354)	9,081 (7,051–11,502)
Dominican Republic	YLDs Number: Cumulative change (%)	6.9 (3.1–11.1)	4.4 (-10.7 to 21.1)	17.4 (14.9–19.5)	0.1 (-8.5 to 8.6)	7.9 (-6.0 to 19.5)	-29.8 (-35.5 to -24.8)	42.1 (23.6–62.6)	-6.5 (-34.8 to 27.6)	-38.6 (-60.6 to -11.9)	5.1 (-17.4 to 34.8)	-1.4 (-8.6 to 6.0)
Dominican Republic	YLDs Rate: 1990	6,672.2 (5,028.7–8,448.2)	1,224.3 (856.3–1,679.5)	860.8 (598.0–1,191.4)	793.7 (525.8–1,091.3)	64.9 (45.9–87.9)	271.9 (196.6–357.6)	90.6 (62.6–124.6)	36.0 (23.5–50.0)	135.3 (86.6–190.0)	6.7 (4.4–9.4)	256.7 (198.1–323.7)
Dominican Republic	YLDs Rate: 2015	6,113.4 (4,605.3–7,786.2)	1,109.8 (782.0–1,500.6)	869.7 (604.7–1,200.7)	686.9 (450.0–953.0)	60.4 (42.5–81.6)	175.4 (126.1–231.6)	111.4 (76.8–152.3)	31.1 (19.5–46.0)	69.4 (43.2–100.0)	6.1 (4.1–8.6)	220.0 (170.9–278.7)
Dominican Republic	YLDs Rate: Cumulative change (%)	-8.4 (-11.5 to -4.8)	-8.9 (-21.8 to 5.4)	1.0 (-0.9 to 2.8)	-13.4 (-20.8 to -5.9)	-6.6 (-18.3 to 3.1)	-35.4 (-40.7 to -30.8)	23.3 (7.3–41.3)	-12.1 (-38.7 to 20.0)	-47.4 (-66.2 to -24.2)	-6.9 (-26.8 to 19.2)	-14.2 (-20.5 to -7.8)
Grenada	Prevalence Number: 1990	45,728 (45,482–45,982)	16,479 (15,143–17,608)	16,034 (15,468–16,525)	6,874 (5,744–8,139)	7,825 (6,233–11,363)	562 (513–621)	641 (549–769)	221 (149–308)	191 (141–245)	0 (0–0)	1,090 (960–1,244)
Grenada	Prevalence Number: 2015	35,473 (35,164–35,747)	11,479 (10,541–12,403)	13,542 (13,091–13,946)	5,495 (4,545–6,559)	5,628 (4,406–8,200)	368 (344–396)	530 (460–622)	110 (72–157)	127 (92–164)	0 (0–0)	765 (674–882)
Grenada	Prevalence Number: Cumulative change (%)	-22.4 (-23.2 to -21.7)	-30.3 (-34.0 to -27.1)	-15.5 (-19.1 to -11.9)	-19.9 (-28.3 to -11.9)	-26.3 (-50.9 to 7.4)	-34.4 (-37.6 to -31.4)	-16.5 (-33.1 to 2.6)	-47.6 (-71.5 to -12.9)	-31.8 (-54.3 to -2.4)	45.8 (-35.9 to 325.9)	-29.5 (-39.6 to -18.0)
Grenada	Prevalence Rate: 1990	96,592.5 (96,119.5–97,093.3)	34,392.5 (31,870.6–36,513.8)	34,727.4 (33,528.1–35,769.7)	14,610.6 (12,268.8–17,269.6)	16,507.8 (13,156.8–23,963.1)	1,066.9 (978.3–1,169.8)	1,348.6 (1,154.5–1,617.0)	396.9 (267.6–552.7)	415.7 (306.6–533.9)	0.0 (0.0–0.0)	2,221.1 (1,952.8–2,546.7)
Grenada	Prevalence Rate: 2015	92,911.9 (92,110.6–93,630.7)	30,205.7 (27,780.0–32,631.1)	35,281.4 (34,099.6–36,346.6)	14,517.5 (11,985.7–17,338.0)	14,753.6 (11,550.0–21,488.6)	969.7 (904.8–1,042.8)	1,391.2 (1,207.1–1,631.6)	288.8 (190.0–411.1)	331.8 (239.7–428.8)	0.0 (0.0–0.0)	2,013.9 (1,775.8–2,321.9)
Grenada	Prevalence Rate: Cumulative change (%)	-3.8 (-4.7 to -2.9)	-12.2 (-16.7 to -7.9)	1.6 (-2.7 to 5.9)	-0.5 (-10.9 to 9.5)	-8.4 (-38.9 to 33.6)	-9.0 (-13.1 to -5.2)	4.1 (-16.6 to 27.9)	-23.5 (-58.4 to 27.0)	-18.2 (-45.0 to 17.1)	99.4 (-13.6 to 491.8)	-8.9 (-22.1 to 6.0)
Grenada	YLDs Number: 1990	3,231 (2,438–4,132)	628 (442–845)	405 (282–558)	310 (203–426)	47 (33–63)	91 (65–122)	46 (32–63)	27 (15–42)	71 (46–101)	0 (0–0)	129 (101–162)
Grenada	YLDs Number: 2015	2,387 (1,797–3,041)	432 (303–586)	333 (230–458)	249 (164–342)	32 (23–44)	60 (42–79)	41 (28–56)	13 (7–21)	31 (19–45)	0 (0–0)	83 (65–105)
Grenada	YLDs Number: Cumulative change (%)	-26.1 (-28.7 to -23.7)	-31.2 (-35.8 to -26.6)	-18.0 (-20.0 to -16.1)	-19.6 (-28.0 to -11.4)	-30.9 (-35.8 to -25.7)	-34.2 (-38.4 to -30.3)	-9.6 (-19.2 to 1.1)	-47.4 (-71.1 to -13.4)	-54.0 (-70.1 to -33.3)	47.3 (-41.5 to 345.0)	-35.6 (-40.0 to -31.1)
Grenada	YLDs Rate: 1990	6,997.2 (5,288.6–8,933.0)	1,319.3 (927.6–1,777.1)	859.4 (596.5–1,181.5)	659.3 (433.2–897.5)	101.7 (72.0–136.7)	173.8 (124.1–231.9)	96.7 (67.0–133.3)	49.2 (28.0–76.1)	154.7 (100.7–219.2)	0.0 (0.0–0.0)	272.0 (212.7–339.2)
Grenada	YLDs Rate: 2015	6,235.6 (4,695.5–7,950.2)	1,148.5 (805.6–1,555.5)	871.5 (603.8–1,199.8)	658.3 (433.9–905.3)	87.6 (62.5–118.1)	158.5 (112.6–209.3)	108.9 (75.7–149.2)	36.0 (20.3–57.0)	83.1 (51.4–119.6)	0.0 (0.0–0.0)	219.5 (170.8–277.9)
Grenada	YLDs Rate: Cumulative change (%)	-10.8 (-14.0 to -8.0)	-12.9 (-18.3 to -7.2)	1.4 (-0.5 to 3.4)	-0.1 (-10.5 to 9.9)	-13.9 (-19.6 to -7.3)	-8.7 (-14.2 to -3.3)	12.9 (0.9–26.3)	-23.2 (-57.8 to 26.3)	-44.7 (-64.0 to -19.7)	98.6 (-23.0 to 508.9)	-19.3 (-24.7 to -13.7)
Guyana	Prevalence Number: 1990	343,454 (340,970–345,892)	139,698 (129,082–147,880)	123,900 (119,976–127,662)	55,241 (46,480–64,252)	62,128 (50,994–85,811)	5,127 (4,737–5,596)	4,158 (3,546–5,068)	5,931 (4,230–7,864)	1,555 (1,146–1,976)	8,536 (4,808–16,140)	9,320 (8,205–10,806)
Guyana	Prevalence Number: 2015	298,017 (295,918–300,070)	96,726 (91,511–100,926)	116,575 (113,219–119,701)	46,226 (38,947–54,046)	48,411 (38,510–68,298)	2,863 (2,612–3,114)	3,980 (3,408–4,765)	2,143 (1,494–2,940)	1,144 (842–1,459)	6,453 (3,694–11,876)	6,903 (6,019–8,079)
Guyana	Prevalence Number: Cumulative change (%)	-13.2 (-14.0 to -12.4)	-30.6 (-37.2 to -24.9)	-5.9 (-9.7 to -2.1)	-16.2 (-23.9 to -7.9)	-20.7 (-44.4 to 12.1)	-44.1 (-47.8 to -40.8)	-3.3 (-24.0 to 18.6)	-62.6 (-77.1 to -44.0)	-24.6 (-49.0 to 6.2)	-23.8 (-34.1 to -11.9)	-25.5 (-37.5 to -11.9)

© 2017 Global Burden of Disease Child and Adolescent Health Collaboration. JAMA Pediatrics.

eTable 3. Prevalent Cases, Rates (per 100,000 population), Years Lived with Disability (YLDs), and Cumulative Percent Change with 95% Uncertainty Interval (UI) for the Top 10 Global Causes of YLDs in Children and Adolescents in 195 Countries and Territories, Aged 0 to 19 Years, Both Sexes, 1990 and 2015. *Note: best viewed by enlarging in browser.*

Location	Measure	All causes	Iron-deficiency anemia	Skin and subcutaneous diseases	Asthma	Hemoglobinopathies and hemolytic anemias	Diarrheal diseases	Congenital anomalies	Protein-energy malnutrition	Epilepsy	Malaria	Neonatal preterm birth complications
Guyana	Prevalence Rate: 1990	95,000.1 (94,353.6–95,611.3)	38,596.4 (36,248.5–40,513.5)	34,557.8 (33,446.7–35,622.3)	15,919.9 (13,446.4–18,482.0)	17,141.4 (14,071.8–23,643.2)	1,254.0 (1,163.4–1,359.8)	1,141.8 (972.1–1,394.4)	1,349.9 (963.2–1,788.7)	437.0 (321.4–555.9)	2,278.1 (1,299.5–4,241.5)	2,417.7 (2,114.8–2,832.5)
Guyana	Prevalence Rate: 2015	93,055.7 (92,355.6–93,730.9)	31,013.3 (29,161.7–32,537.2)	34,867.2 (33,868.1–35,803.2)	14,724.7 (12,289.4–17,339.4)	15,157.9 (12,044.1–21,385.5)	1,018.5 (923.9–1,113.8)	1,251.7 (1,070.9–1,495.8)	804.8 (560.8–1,104.1)	345.4 (252.9–440.1)	2,300.2 (1,273.0–4,369.9)	2,292.1 (2,011.5–2,665.9)
Guyana	Prevalence Rate: Cumulative change (%)	-2.0 (-2.9 to -1.2)	-19.5 (-26.8 to -14.0)	0.9 (-3.0 to 5.1)	-7.4 (-15.9 to 1.2)	-10.0 (-37.0 to 27.3)	-18.8 (-24.3 to -14.1)	10.8 (-13.1 to 36.1)	-38.3 (-62.3 to -7.6)	-19.0 (-45.2 to 13.9)	0.7 (-12.3 to 19.0)	-4.6 (-20.6 to 12.3)
Guyana	YLDs Number: 1990	25,890 (19,591–32,911)	5,457 (3,829–7,353)	3,100 (2,160–4,280)	2,492 (1,651–3,387)	369 (258–496)	833 (606–1,097)	293 (201–403)	736 (445–1,104)	577 (377–817)	33 (21–47)	1,089 (847–1,349)
Guyana	YLDs Number: 2015	21,173 (16,073–26,848)	3,668 (2,610–4,952)	2,793 (1,944–3,902)	2,092 (1,394–2,864)	272 (194–369)	467 (336–616)	389 (264–538)	267 (162–416)	296 (187–426)	25 (16–34)	836 (651–1,043)
Guyana	YLDs Number: Cumulative change (%)	-18.2 (-21.1 to -15.3)	-32.6 (-40.9 to -25.1)	-10.0 (-12.5 to -7.3)	-15.9 (-23.9 to -7.5)	-26.0 (-33.7 to -17.2)	-43.9 (-48.2 to -39.9)	33.3 (12.9–58.6)	-62.4 (-76.9 to -43.0)	-47.4 (-65.8 to -26.2)	-24.5 (-39.7 to -5.2)	-23.2 (-27.8 to -18.2)
Guyana	YLDs Rate: 1990	7,242.6 (5,491.3–9,206.0)	1,535.5 (1,079.0–2,064.8)	858.9 (597.5–1,188.7)	718.2 (476.2–973.7)	103.7 (72.6–140.2)	203.6 (149.0–268.1)	80.4 (55.1–110.7)	167.6 (101.5–251.2)	162.3 (106.1–231.1)	9.2 (6.0–13.1)	297.9 (231.3–369.0)
Guyana	YLDs Rate: 2015	6,435.7 (4,872.0–8,166.9)	1,191.8 (847.1–1,613.2)	865.2 (606.9–1,195.5)	666.9 (444.6–918.5)	87.6 (61.9–118.6)	166.3 (119.3–220.0)	122.6 (83.3–169.7)	100.4 (61.0–156.5)	89.5 (56.4–128.8)	8.7 (5.7–12.1)	266.0 (206.3–331.8)
Guyana	YLDs Rate: Cumulative change (%)	-11.1 (-14.1 to -8.5)	-22.2 (-31.6 to -15.5)	0.7 (-1.2 to 2.5)	-7.0 (-15.6 to 1.8)	-15.4 (-24.0 to -7.0)	-18.3 (-24.7 to -12.7)	53.3 (29.5–82.6)	-37.9 (-62.0 to -6.0)	-43.5 (-63.2 to -20.2)	-4.5 (-23.2 to 19.2)	-10.7 (-16.0 to -5.1)
Haiti	Prevalence Number: 1990	3,632,435 (3,617,152–3,647,835)	1,586,929 (1,509,622–1,720,314)	1,226,652 (1,189,012–1,264,163)	849,604 (712,856–999,158)	451,129 (423,651–479,172)	72,607 (66,726–79,871)	31,165 (25,539–38,508)	35,379 (27,524–44,577)	16,333 (12,062–20,859)	46,090 (23,060–80,453)	80,164 (66,874–98,602)
Haiti	Prevalence Number: 2015	4,399,937 (4,368,042–4,430,441)	1,859,514 (1,792,733–1,934,686)	1,611,781 (1,564,924–1,659,792)	868,767 (718,160–1,022,105)	490,969 (462,761–520,047)	77,658 (71,260–85,764)	50,015 (42,399–61,270)	32,155 (23,718–42,125)	16,886 (12,354–21,744)	8,033 (5,680–12,501)	106,323 (88,746–133,447)
Haiti	Prevalence Number: Cumulative change (%)	21.1 (20.2–22.0)	17.4 (6.3–25.8)	31.4 (26.4–36.8)	2.3 (-5.0 to 10.2)	9.0 (1.1–17.9)	7.0 (1.3–12.7)	62.8 (25.6–106.0)	-7.1 (-38.4 to 32.2)	5.6 (-26.9 to 44.0)	-80.8 (-89.1 to -69.7)	34.1 (5.7–66.8)
Haiti	Prevalence Rate: 1990	97,053.1 (96,686.6–97,421.7)	42,070.4 (40,232.1–45,277.1)	33,977.2 (32,957.6–34,951.2)	22,533.4 (18,989.4–26,435.4)	12,057.8 (11,327.0–12,809.8)	1,709.3 (1,572.7–1,877.6)	820.1 (671.6–1,015.7)	804.3 (625.8–1,012.9)	448.5 (331.1–574.7)	1,098.4 (554.3–1,900.3)	1,926.2 (1,588.5–2,416.6)
Haiti	Prevalence Rate: 2015	92,726.9 (92,052.8–93,370.9)	39,189.2 (37,775.3–40,777.8)	33,982.8 (32,996.8–34,994.5)	18,290.2 (15,121.3–21,512.8)	10,347.4 (9,752.9–10,960.4)	1,637.3 (1,502.2–1,808.2)	1,054.0 (893.5–1,291.3)	678.9 (500.8–889.4)	355.9 (260.4–458.2)	169.3 (119.7–263.5)	2,239.1 (1,868.8–2,810.7)
Haiti	Prevalence Rate: Cumulative change (%)	-4.5 (-5.2 to -3.8)	-6.7 (-14.9 to -0.3)	0.0 (-3.7 to 4.1)	-18.8 (-24.5 to -12.5)	-14.1 (-20.3 to -7.0)	-4.2 (-9.1 to 0.9)	28.5 (0.3–65.2)	-13.7 (-42.8 to 22.8)	-18.9 (-44.0 to 11.1)	-83.1 (-90.3 to -73.5)	17.8 (-8.8 to 47.1)
Haiti	YLDs Number: 1990	298,594 (225,354–381,170)	67,266 (47,261–91,579)	31,565 (22,050–43,344)	38,206 (25,708–52,541)	3,150 (2,234–4,234)	11,736 (8,549–15,664)	2,413 (1,586–3,454)	4,349 (2,798–6,277)	6,364 (4,100–8,988)	1,412 (756–2,167)	6,427 (4,803–8,197)
Haiti	YLDs Number: 2015	358,680 (274,233–455,633)	75,117 (53,275–101,419)	40,426 (28,237–55,765)	39,154 (26,077–53,543)	3,488 (2,476–4,663)	12,585 (9,168–16,656)	4,071 (2,733–5,811)	3,968 (2,466–5,809)	6,093 (3,912–8,484)	235 (142–353)	8,626 (6,379–11,037)
Haiti	YLDs Number: Cumulative change (%)	20.4 (13.2–27.9)	12.2 (-2.3 to 24.7)	28.0 (24.9–31.0)	2.5 (-4.7 to 10.8)	11.1 (-4.1 to 23.9)	7.3 (0.7–13.7)	70.4 (43.1–111.3)	-6.5 (-37.7 to 33.3)	-2.0 (-34.0 to 34.1)	-82.7 (-88.2 to -74.6)	34.6 (21.1–49.3)
Haiti	YLDs Rate: 1990	8,124.9 (6,132.7–10,359.9)	1,766.3 (1,243.3–2,392.4)	850.7 (594.3–1,171.4)	1,012.8 (680.0–1,384.8)	83.6 (59.3–111.8)	276.2 (201.1–368.8)	63.5 (41.5–91.0)	98.9 (63.6–143.0)	174.6 (112.7–246.2)	33.7 (18.1–51.5)	165.8 (123.9–211.9)
Haiti	YLDs Rate: 2015	7,559.2 (5,779.7–9,602.0)	1,582.3 (1,122.4–2,136.5)	852.1 (595.2–1,175.6)	824.3 (549.0–1,127.1)	73.5 (52.2–98.2)	265.3 (193.3–351.2)	85.8 (57.6–122.5)	83.8 (52.1–122.6)	128.4 (82.5–178.8)	5.0 (3.0–7.4)	181.8 (134.4–232.6)
Haiti	YLDs Rate: Cumulative change (%)	-6.8 (-12.0 to -1.4)	-10.1 (-20.9 to -0.4)	0.2 (-1.9 to 2.2)	-18.6 (-24.3 to -12.2)	-11.8 (-23.3 to -2.0)	-3.9 (-9.6 to 1.8)	36.6 (14.3–69.8)	-13.2 (-42.1 to 23.7)	-24.7 (-49.0 to 3.5)	-84.8 (-89.5 to -77.6)	9.9 (-1.3 to 22.0)
Jamaica	Prevalence Number: 1990	1,060,638 (1,056,259–1,064,516)	357,405 (332,808–384,431)	385,636 (372,193–397,515)	179,608 (150,623–212,867)	192,192 (183,197–200,935)	10,523 (9,704–11,422)	14,988 (12,832–17,772)	5,544 (4,442–6,871)	5,382 (3,964–6,884)	0 (0–0)	24,266 (21,423–27,979)
Jamaica	Prevalence Number: 2015	937,351 (934,535–940,166)	291,647 (275,011–307,021)	349,109 (338,193–358,995)	147,726 (121,922–175,127)	148,048 (141,965–154,442)	6,676 (6,126–7,284)	13,849 (11,963–16,249)	3,028 (2,077–4,267)	3,513 (2,619–4,524)	0 (0–0)	20,155 (17,695–23,220)
Jamaica	Prevalence Number: Cumulative change (%)	-11.6 (-12.0 to -11.2)	-18.3 (-22.3 to -10.9)	-9.4 (-13.3 to -5.3)	-17.6 (-27.1 to -7.6)	-22.9 (-27.3 to -18.2)	-36.5 (-41.4 to -32.2)	-6.9 (-25.1 to 13.7)	-44.4 (-64.9 to -18.7)	-33.1 (-55.2 to -6.1)	58.4 (-29.3 to 359.7)	-16.6 (-27.4 to -3.7)
Jamaica	Prevalence Rate: 1990	95,603.9 (95,220.1–95,947.6)	32,032.0 (29,919.7–34,359.5)	34,939.8 (33,732.6–36,020.4)	16,233.6 (13,650.8–19,270.1)	17,316.0 (16,511.8–18,103.4)	922.5 (851.6–999.9)	1,349.5 (1,155.4–1,600.2)	478.5 (383.4–592.9)	488.5 (360.5–625.2)	0.0 (0.0–0.0)	2,172.9 (1,917.2–2,507.8)
Jamaica	Prevalence Rate: 2015	96,765.2 (96,465.3–97,058.3)	30,898.4 (29,161.8–32,556.6)	35,161.1 (34,024.7–36,173.1)	15,594.3 (12,804.9–18,505.1)	15,307.4 (14,681.5–15,960.1)	727.4 (666.5–794.1)	1,432.3 (1,239.9–1,681.5)	332.7 (228.2–469.0)	357.6 (266.4–459.6)	0.0 (0.0–0.0)	2,138.7 (1,880.5–2,455.2)
Jamaica	Prevalence Rate: Cumulative change (%)	1.2 (0.8–1.7)	-3.4 (-8.2 to 5.3)	0.7 (-3.6 to 5.3)	-3.8 (-15.3 to 7.8)	-11.5 (-16.6 to -6.2)	-21.1 (-27.2 to -15.7)	7.0 (-14.0 to 30.6)	-29.2 (-55.3 to 3.5)	-25.0 (-49.7 to 5.3)	99.3 (-13.2 to 488.2)	-1.1 (-13.9 to 13.8)
Jamaica	YLDs Number: 1990	73,514 (55,911–93,600)	13,724 (9,797–18,622)	9,580 (6,671–13,190)	8,130 (5,374–11,131)	1,111 (798–1,505)	1,713 (1,233–2,280)	1,234 (854–1,701)	685 (442–974)	2,008 (1,312–2,811)	0 (0–0)	3,035 (2,360–3,819)
Jamaica	YLDs Number: 2015	64,654 (48,921–82,447)	11,050 (7,911–14,771)	8,457 (5,891–11,685)	6,682 (4,406–9,212)	895 (649–1,186)	1,086 (775–1,444)	1,285 (880–1,777)	375 (224–581)	885 (557–1,298)	0 (0–0)	2,324 (1,794–2,952)
Jamaica	YLDs Number: Cumulative change (%)	-12.1 (-14.8 to -8.9)	-19.3 (-24.7 to -9.8)	-11.7 (-13.8 to -9.8)	-17.6 (-27.2 to -7.6)	-19.2 (-26.8 to -11.0)	-36.6 (-42.0 to -31.4)	4.3 (-10.1 to 20.2)	-44.3 (-65.4 to -17.1)	-54.8 (-71.2 to -33.8)	63.1 (-32.2 to 382.0)	-23.4 (-28.2 to -18.0)
Jamaica	YLDs Rate: 1990	6,656.4 (5,063.8–8,478.0)	1,233.3 (880.6–1,677.9)	863.9 (602.3–1,187.8)	734.8 (485.5–1,001.7)	100.6 (72.2–135.8)	150.2 (108.2–199.3)	111.2 (76.9–153.1)	59.2 (38.2–84.1)	182.2 (119.0–254.9)	0.0 (0.0–0.0)	273.1 (212.3–343.3)
Jamaica	YLDs Rate: 2015	6,600.4 (5,001.4–8,430.0)	1,191.6 (851.8–1,590.6)	867.8 (605.1–1,192.8)	705.5 (462.5–981.5)	95.8 (69.4–126.5)	118.3 (84.4–157.4)	133.0 (91.1–184.2)	41.3 (24.6–63.9)	90.2 (56.8–132.7)	0.0 (0.0–0.0)	241.4 (186.0–306.6)
Jamaica	YLDs Rate: Cumulative change (%)	-0.9 (-4.1 to 2.8)	-3.2 (-9.6 to 8.2)	0.5 (-1.5 to 2.5)	-3.8 (-15.1 to 7.8)	-4.6 (-13.3 to 5.2)	-21.1 (-27.9 to -14.7)	20.0 (3.4–38.5)	-29.1 (-55.9 to 5.5)	-49.3 (-67.6 to -25.8)	105.8 (-16.5 to 517.5)	-11.6 (-17.2 to -5.3)
Puerto Rico	Prevalence Number: 1990	1,179,549 (1,168,033–1,190,574)	386,420 (360,765–398,487)	469,295 (454,948–482,738)	188,876 (158,711–222,177)	209,563 (165,023–307,498)	5,083 (4,856–5,298)	18,807 (16,370–21,926)	3,857 (2,532–5,677)	4,055 (2,985–5,253)	0 (0–0)	20,458 (18,320–23,175)
Puerto Rico	Prevalence Number: 2015	882,546 (873,972–890,544)	271,995 (251,007–297,970)	360,606 (349,283–370,465)	134,076 (111,185–159,487)	135,077 (105,441–193,532)	4,611 (4,402–4,829)	14,353 (12,578–16,679)	2,114 (1,404–3,060)	2,584 (1,910–3,377)	0 (0–0)	14,486 (12,938–16,378)
Puerto Rico	Prevalence Number: Cumulative change (%)	-25.2 (-26.1 to -24.3)	-29.6 (-36.5 to -23.0)	-23.1 (-26.2 to -20.2)	-28.8 (-39.4 to -17.8)	-33.8 (-56.2 to -1.7)	-9.3 (-12.2 to -6.2)	-23.1 (-35.8 to -7.8)	-42.0 (-68.5 to -5.5)	-34.8 (-56.0 to -8.1)	42.2 (-37.1 to 317.4)	-29.0 (-37.6 to -19.6)
Puerto Rico	Prevalence Rate: 1990	90,721.9 (89,818.2–91,597.3)	30,030.5 (27,938.8–31,030.3)	35,644.3 (34,567.3–36,658.9)	14,519.9 (12,172.2–17,126.6)	16,148.4 (12,715.5–23,699.3)	411.5 (393.0–429.5)	1,450.6 (1,263.5–1,691.3)	321.0 (210.7–472.4)	310.2 (228.5–401.5)	0.0 (0.0–0.0)	1,614.1 (1,447.3–1,823.5)
Puerto Rico	Prevalence Rate: 2015	90,489.1 (89,570.3–91,351.8)	28,867.0 (26,442.2–31,851.1)	35,992.0 (34,843.7–37,029.5)	13,796.4 (11,393.0–16,416.8)	13,919.0 (10,865.6–19,932.0)	526.1 (501.4–551.5)	1,479.0 (1,296.1–1,720.0)	252.0 (167.5–364.9)	261.8 (193.4–340.8)	0.0 (0.0–0.0)	1,560.0 (1,397.7–1,755.6)
Puerto Rico	Prevalence Rate: Cumulative change (%)	-0.3 (-1.5 to 1.0)	-3.8 (-13.9 to 5.4)	1.0 (-3.0 to 4.8)	-4.6 (-18.9 to 10.7)	-11.5 (-41.3 to 31.4)	27.9 (23.6–32.2)	2.7 (-14.2 to 23.4)	-16.9 (-54.9 to 35.4)	-13.6 (-41.5 to 21.6)	99.4 (-13.8 to 491.6)	-3.1 (-14.4 to 9.2)
Puerto Rico	YLDs Number: 1990	79,958 (60,606–101,511)	14,533 (10,308–19,627)	11,420 (7,889–15,908)	8,568 (5,690–11,722)	1,287 (920–1,727)	832 (602–1,097)	1,770 (1,229–2,448)	483 (266–767)	1,511 (978–2,180)	0 (0–0)	2,782 (2,177–3,494)
Puerto Rico	YLDs Number: 2015	58,767 (44,470–74,625)	10,261 (7,204–14,054)	8,632 (5,945–12,090)	6,089 (4,035–8,401)	870 (611–1,172)	755 (548–991)	1,551 (1,055–2,131)	263 (150–411)	584 (341–814)	0 (0–0)	1,707 (1,321–2,171)
Puerto Rico	YLDs Number: Cumulative change (%)	-26.5 (-29.0 to -24.1)	-29.3 (-37.5 to -21.7)	-24.4 (-26.0 to -22.9)	-28.7 (-39.3 to -17.3)	-32.3 (-39.0 to -24.6)	-9.2 (-15.1 to -3.5)	-11.9 (-27.5 to 7.5)	-42.0 (-68.7 to -4.1)	-61.6 (-75.8 to -44.0)	45.0 (-42.0 to 336.8)	-38.6 (-43.4 to -33.4)
Puerto Rico	YLDs Rate: 1990	6,087.1 (4,616.2–7,742.0)	1,129.0 (799.7–1,528.4)	875.8 (604.9–1,215.9)	658.8 (437.7–902.1)	99.2 (70.8–133.3)	67.4 (48.8–88.9)	136.6 (94.8–188.7)	40.2 (22.2–63.9)	115.7 (74.9–166.2)	0.0 (0.0–0.0)	214.6 (167.9–269.7)
Puerto Rico	YLDs Rate: 2015	5,907.3 (4,461.3–7,509.2)	1,100.4 (769.4–1,506.5)	879.7 (607.5–1,220.0)	626.8 (414.5–868.4)	92.1 (64.8–124.3)	86.2 (62.8–113.2)	160.1 (108.7–219.6)	31.4 (17.9–49.0)	57.3 (34.9–83.0)	0.0 (0.0–0.0)	176.0 (135.9–223.9)
Puerto Rico	YLDs Rate: Cumulative change (%)	-2.9 (-6.6 to 0.7)	-2.4 (-14.7 to 9.0)	0.4 (-1.5 to 2.3)	-4.5 (-19.0 to 10.9)	-7.0 (-16.7 to 4.1)	28.0 (19.8–35.8)	17.8 (-2.8 to 43.7)	-16.9 (-55.1 to 37.4)	-49.1 (-68.0 to -25.9)	103.5 (-19.9 to 521.3)	-18.0 (-24.3 to -11.0)
Saint Lucia	Prevalence Number: 1990	65,109 (64,817–65,407)	20,159 (19,176–21,157)	23,073 (22,377–23,744)	11,855 (9,835–13,983)	10,899 (8,795–15,648)	643 (583–705)	856 (725–1,020)	365 (245–511)	361 (268–462)	0 (0–0)	1,441 (1,268–1,668)
Saint Lucia	Prevalence Number: 2015	55,324 (54,910–55,669)	16,203 (14,980–17,363)	21,258 (20,616–21,805)	9,665 (8,169–11,377)	8,521 (6,680–12,567)	437 (400–479)	809 (702–947)	206 (145–281)	239 (174–310)	0 (0–0)	1,126 (988–1,302)
Saint Lucia	Prevalence Number: Cumulative change (%)	-15.0 (-15.7 to -14.3)	-19.5 (-26.5 to -12.5)	-7.8 (-11.4 to -4.4)	-18.3 (-25.8 to -9.9)	-20.0 (-47.3 to 17.2)	-31.9 (-36.5 to -27.6)	-4.6 (-23.0 to 15.7)	-40.7 (-64.5 to -8.9)	-32.1 (-54.3 to -3.1)	57.9 (-29.7 to 358.7)	-21.5 (-32.6 to -9.5)
Saint Lucia	Prevalence Rate: 1990	97,253.0 (96,843.3–97,678.2)	30,014.7 (28,586.8–31,418.9)	34,841.6 (33,786.7–35,859.5)	17,820.5 (14,818.6–21,018.2)	16,266.1 (13,127.4–23,353.9)	905.0 (823.8–989.6)	1,275.8 (1,081.1–1,520.4)	499.5 (335.6–698.7)	546.3 (407.0–700.7)	0.0 (0.0–0.0)	2,106.3 (1,848.5–2,443.6)
Saint Lucia	Prevalence Rate: 2015	93,751.2 (93,040.7–94,379.5)	28,103.2 (25,851.9–30,158.6)	35,280.5 (34,222.3–36,217.2)	16,461.8 (13,884.4–19,401.9)	14,484.9 (11,350.3–21,360.4)	804.1 (733.7–883.6)	1,378.2 (1,196.7–1,613.5)	392.5 (277.1–535.5)	398.2 (291.6–514.8)	0.0 (0.0–0.0)	1,996.5 (1,756.9–2,303.4)

© 2017 Global Burden of Disease Child and Adolescent Health Collaboration. JAMA Pediatrics.

eTable 3. Prevalent Cases, Rates (per 100,000 population), Years Lived with Disability (YLDs), and Cumulative Percent Change with 95% Uncertainty Interval (UI) for the Top 10 Global Causes of YLDs in Children and Adolescents in 195 Countries and Territories, Aged 0 to 19 Years, Both Sexes, 1990 and 2015. *Note: best viewed by enlarging in browser.*

Location	Measure	All causes	Iron-deficiency anemia	Skin and subcutaneous diseases	Asthma	Hemoglobinopathies and hemolytic anemias	Diarrheal diseases	Congenital anomalies	Protein-energy malnutrition	Epilepsy	Malaria	Neonatal preterm birth complications
Saint Lucia	Prevalence Rate: Cumulative change (%)	-3.6 (-4.4 to -2.8)	-6.3 (-14.0 to 1.4)	1.3 (-2.6 to 5.2)	-7.5 (-16.0 to 2.0)	-8.9 (-39.9 to 33.4)	-11.1 (-16.9 to -5.5)	9.0 (-12.0 to 32.2)	-17.6 (-50.6 to 26.6)	-25.3 (-49.9 to 6.4)	99.3 (-13.7 to 488.3)	-4.8 (-18.3 to 9.6)
Saint Lucia	YLDs Number: 1990	4,727 (3,566–6,039)	755 (538–1,022)	575 (400–794)	534 (354–738)	62 (45–84)	104 (75–138)	62 (42–85)	45 (26–71)	134 (86–193)	0 (0–0)	179 (140–225)
Saint Lucia	YLDs Number: 2015	3,739 (2,831–4,726)	609 (429–830)	517 (358–714)	438 (292–607)	51 (36–70)	71 (50–95)	77 (52–107)	25 (15–38)	60 (38–85)	0 (0–0)	132 (103–166)
Saint Lucia	YLDs Number: Cumulative change (%)	-20.8 (-24.2 to -17.5)	-19.2 (-26.8 to -11.5)	-10.2 (-12.5 to -8.1)	-17.8 (-25.3 to -9.4)	-18.3 (-26.7 to -8.7)	-31.8 (-37.1 to -26.6)	24.9 (4.4–46.2)	-40.6 (-64.8 to -9.0)	-54.2 (-70.0 to -33.7)	72.3 (-32.4 to 436.5)	-26.2 (-31.0 to -21.2)
Saint Lucia	YLDs Rate: 1990	7,152.2 (5,399.8–9,139.6)	1,129.1 (807.1–1,530.4)	861.3 (597.8–1,188.8)	803.6 (533.4–1,109.7)	94.6 (68.5–126.1)	147.5 (106.0–194.6)	92.4 (62.9–127.1)	62.2 (36.8–98.3)	203.8 (130.7–292.6)	0.0 (0.0–0.0)	266.4 (208.8–334.6)
Saint Lucia	YLDs Rate: 2015	6,245.0 (4,723.9–7,903.9)	1,063.9 (749.6–1,450.7)	872.1 (604.2–1,201.6)	747.0 (498.9–1,038.2)	88.9 (62.5–121.5)	131.4 (92.8–175.9)	131.6 (89.2–182.7)	48.9 (29.1–72.9)	99.8 (64.0–142.6)	0.0 (0.0–0.0)	225.6 (176.4–284.6)
Saint Lucia	YLDs Rate: Cumulative change (%)	-12.6 (-16.1 to -9.2)	-5.7 (-14.4 to 3.1)	1.2 (-0.9 to 3.2)	-6.8 (-15.5 to 2.7)	-6.0 (-15.5 to 4.7)	-10.9 (-17.7 to -4.0)	43.0 (19.4–67.6)	-17.5 (-51.0 to 26.5)	-49.6 (-67.0 to -27.3)	114.6 (-17.8 to 581.3)	-15.3 (-20.7 to -9.5)
Saint Vincent and the Grenadines	Prevalence Number: 1990	50,292 (49,979–50,607)	16,254 (15,177–17,408)	18,417 (17,892–18,961)	8,360 (6,982–9,818)	8,554 (6,868–12,105)	543 (503–586)	622 (528–756)	209 (139–292)	266 (197–341)	0 (0–0)	1,139 (1,005–1,321)
Saint Vincent and the Grenadines	Prevalence Number: 2015	34,287 (34,018–34,570)	10,133 (9,441–10,867)	13,247 (12,831–13,632)	5,627 (4,738–6,628)	5,354 (4,190–7,992)	311 (289–337)	457 (387–549)	90 (59–132)	149 (110–193)	0 (0–0)	722 (630–845)
Saint Vincent and the Grenadines	Prevalence Number: Cumulative change (%)	-31.8 (-32.5 to -31.2)	-37.6 (-41.6 to -33.9)	-28.1 (-30.8 to -25.3)	-32.5 (-39.5 to -25.0)	-35.9 (-57.8 to -3.0)	-42.5 (-46.5 to -39.3)	-25.9 (-42.3 to -7.3)	-54.6 (-75.2 to -28.7)	-42.5 (-60.8 to -17.8)	32.8 (-41.5 to 289.2)	-36.3 (-45.6 to -25.4)
Saint Vincent and the Grenadines	Prevalence Rate: 1990	95,080.2 (94,473.6–95,690.7)	30,746.6 (28,686.4–32,984.6)	34,818.4 (33,825.5–35,848.1)	15,599.8 (13,027.1–18,302.6)	16,189.9 (12,999.7–22,914.6)	1,062.2 (983.7–1,149.0)	1,179.7 (1,001.0–1,433.6)	417.3 (277.0–582.2)	502.4 (372.8–643.6)	0.0 (0.0–0.0)	2,205.6 (1,950.3–2,552.6)
Saint Vincent and the Grenadines	Prevalence Rate: 2015	92,583.0 (91,825.3–93,376.4)	27,927.5 (25,872.4–30,101.4)	35,198.0 (34,067.8–36,212.4)	15,195.7 (12,789.4–17,917.9)	14,510.6 (11,355.8–21,655.6)	903.8 (835.9–981.0)	1,239.8 (1,052.9–1,490.0)	271.5 (177.0–396.5)	396.6 (293.9–515.0)	0.0 (0.0–0.0)	2,042.4 (1,790.5–2,373.2)
Saint Vincent and the Grenadines	Prevalence Rate: Cumulative change (%)	-2.6 (-3.6 to -1.6)	-9.1 (-15.3 to -3.3)	1.1 (-2.8 to 5.0)	-2.3 (-12.4 to 8.3)	-8.2 (-39.7 to 38.9)	-14.8 (-20.8 to -9.8)	6.1 (-17.3 to 32.7)	-31.7 (-62.7 to 7.5)	-19.1 (-45.1 to 15.4)	99.3 (-13.6 to 488.1)	-7.0 (-20.2 to 8.4)
Saint Vincent and the Grenadines	YLDs Number: 1990	3,512 (2,662–4,433)	621 (445–843)	457 (317–631)	378 (248–519)	50 (36–67)	88 (63–117)	41 (28–57)	26 (14–40)	99 (64–140)	0 (0–0)	155 (121–194)
Saint Vincent and the Grenadines	YLDs Number: 2015	2,323 (1,755–2,933)	380 (268–516)	324 (224–450)	255 (170–348)	32 (23–43)	50 (36–67)	40 (27–56)	11 (6–18)	37 (23–55)	0 (0–0)	90 (70–113)
Saint Vincent and the Grenadines	YLDs Number: Cumulative change (%)	-33.8 (-35.7 to -32.1)	-38.7 (-43.9 to -34.2)	-29.1 (-30.5 to -27.8)	-32.4 (-39.6 to -24.7)	-35.5 (-41.1 to -30.2)	-42.5 (-46.7 to -38.6)	-2.0 (-22.0 to 19.6)	-54.5 (-75.3 to -28.6)	-61.1 (-74.9 to -42.1)	36.1 (-46.5 to 319.6)	-41.9 (-45.9 to -37.7)
Saint Vincent and the Grenadines	YLDs Rate: 1990	6,623.9 (5,023.3–8,350.3)	1,165.6 (835.2–1,582.8)	863.7 (599.4–1,191.6)	705.8 (464.1–968.3)	94.4 (67.6–126.6)	173.4 (124.2–229.4)	78.9 (53.6–109.8)	51.9 (29.1–81.6)	187.7 (121.8–265.0)	0.0 (0.0–0.0)	293.8 (230.1–368.0)
Saint Vincent and the Grenadines	YLDs Rate: 2015	6,200.9 (4,685.6–7,839.2)	1,053.2 (744.0–1,429.2)	872.0 (604.8–1,206.2)	688.9 (460.0–937.8)	89.5 (64.1–120.3)	147.6 (106.8–195.6)	110.1 (74.5–154.6)	33.8 (19.3–54.1)	100.1 (63.1–148.2)	0.0 (0.0–0.0)	244.9 (191.5–309.0)
Saint Vincent and the Grenadines	YLDs Rate: Cumulative change (%)	-6.4 (-9.0 to -3.8)	-9.6 (-18.0 to -2.2)	0.9 (-0.9 to 2.7)	-2.2 (-12.3 to 8.8)	-5.1 (-13.5 to 3.1)	-14.8 (-21.1 to -8.8)	40.5 (11.8–71.5)	-31.5 (-62.8 to 7.6)	-45.2 (-64.5 to -18.8)	105.3 (-20.3 to 537.4)	-16.6 (-22.3 to -10.5)
Suriname	Prevalence Number: 1990	167,733 (166,531–168,938)	62,926 (58,847–67,679)	62,137 (60,370–63,827)	32,662 (27,060–38,775)	33,247 (27,721–43,685)	2,027 (1,868–2,195)	2,083 (1,762–2,485)	1,553 (1,054–2,145)	852 (630–1,099)	528 (309–1,014)	3,709 (3,259–4,346)
Suriname	Prevalence Number: 2015	176,959 (175,552–178,420)	56,192 (52,834–59,559)	68,332 (66,229–70,185)	30,747 (25,656–36,135)	30,887 (24,517–43,441)	1,593 (1,451–1,739)	2,283 (1,937–2,744)	909 (636–1,225)	713 (531–914)	590 (295–1,162)	3,690 (3,258–4,265)
Suriname	Prevalence Number: Cumulative change (%)	5.5 (4.4–6.5)	-10.5 (-20.4 to -0.4)	10.0 (5.9–14.2)	-5.6 (-15.5 to 4.8)	-5.8 (-33.9 to 31.8)	-21.4 (-27.3 to -16.2)	10.7 (-12.1 to 38.1)	-38.7 (-63.4 to -6.7)	-14.4 (-41.4 to 19.9)	11.0 (-8.8 to 47.9)	0.0 (-14.4 to 17.4)
Suriname	Prevalence Rate: 1990	92,718.5 (92,093.2–93,374.9)	34,474.1 (32,464.7–36,847.8)	34,780.6 (33,804.4–35,729.7)	18,225.5 (15,138.7–21,607.1)	18,360.0 (15,317.5–24,116.9)	1,040.8 (960.5–1,125.0)	1,147.5 (970.1–1,368.5)	773.0 (525.0–1,067.7)	475.7 (351.0–615.2)	281.1 (165.3–533.6)	1,973.1 (1,729.1–2,322.1)
Suriname	Prevalence Rate: 2015	91,713.9 (90,962.7–92,477.2)	29,374.7 (27,565.6–31,187.6)	35,124.9 (34,042.8–36,100.7)	15,890.2 (13,248.0–18,747.7)	16,029.3 (12,720.9–22,537.2)	859.8 (780.4–940.3)	1,186.6 (1,007.9–1,426.1)	501.8 (351.2–676.1)	367.6 (274.2–471.5)	313.7 (157.4–621.4)	1,962.0 (1,735.6–2,263.6)
Suriname	Prevalence Rate: Cumulative change (%)	-1.1 (-2.1 to -0.1)	-14.6 (-23.8 to -5.2)	1.0 (-2.7 to 4.9)	-12.6 (-21.6 to -3.2)	-11.5 (-37.9 to 23.9)	-17.3 (-23.8 to -11.9)	4.4 (-17.0 to 30.2)	-32.0 (-59.5 to 3.4)	-20.9 (-45.6 to 10.3)	10.6 (-9.4 to 48.9)	0.0 (-14.8 to 17.3)
Suriname	YLDs Number: 1990	12,208 (9,269–15,457)	2,416 (1,697–3,247)	1,552 (1,081–2,136)	1,478 (979–2,050)	178 (127–239)	329 (236–430)	151 (105–206)	192 (113–300)	320 (204–456)	11 (7–17)	454 (356–570)
Suriname	YLDs Number: 2015	12,113 (9,162–15,406)	2,122 (1,513–2,865)	1,681 (1,169–2,322)	1,394 (916–1,933)	172 (122–232)	260 (184–345)	214 (147–296)	113 (67–170)	200 (127–284)	12 (7–18)	454 (354–571)
Suriname	YLDs Number: Cumulative change (%)	-0.7 (-4.9 to 3.2)	-11.8 (-24.5 to 1.5)	8.2 (6.0–10.4)	-5.4 (-15.4 to 5.1)	-3.5 (-16.1 to 6.6)	-21.1 (-27.8 to -14.9)	42.6 (23.4–67.2)	-38.4 (-63.7 to -6.6)	-35.9 (-56.8 to -7.8)	9.3 (-19.8 to 49.9)	0.0 (-6.2 to 6.8)
Suriname	YLDs Rate: 1990	6,804.6 (5,170.1–8,632.4)	1,335.4 (943.3–1,785.6)	861.5 (598.6–1,189.2)	825.1 (544.3–1,139.0)	100.1 (71.5–133.9)	169.4 (121.4–221.0)	83.1 (58.1–113.6)	95.9 (56.6–149.5)	178.7 (113.9–255.0)	6.4 (4.1–9.4)	249.4 (195.9–313.1)
Suriname	YLDs Rate: 2015	6,240.2 (4,722.2–7,933.7)	1,108.2 (788.9–1,497.0)	869.4 (605.5–1,199.1)	720.8 (473.1–1,000.8)	89.5 (63.4–120.7)	140.5 (99.6–186.9)	111.7 (76.5–154.4)	62.5 (37.1–94.0)	103.3 (65.5–146.9)	6.7 (4.1–9.8)	236.4 (184.9–296.6)
Suriname	YLDs Rate: Cumulative change (%)	-8.3 (-11.8 to -4.7)	-16.8 (-28.2 to -4.5)	0.9 (-0.9 to 2.7)	-12.4 (-21.5 to -3.1)	-10.5 (-21.8 to -1.6)	-17.0 (-24.2 to -10.6)	34.7 (16.6–58.0)	-31.7 (-59.7 to 3.5)	-40.7 (-60.2 to -14.5)	6.8 (-21.6 to 46.1)	-5.2 (-11.2 to 1.2)
Trinidad and Tobago	Prevalence Number: 1990	487,027 (482,690–491,090)	160,698 (149,729–171,720)	190,176 (184,273–195,266)	101,576 (85,054–119,186)	88,341 (71,527–127,366)	3,487 (3,280–3,691)	7,087 (6,114–8,369)	2,416 (1,751–3,234)	2,553 (1,875–3,257)	0 (0–0)	10,543 (9,308–12,032)
Trinidad and Tobago	Prevalence Number: 2015	337,203 (333,637–340,330)	105,519 (96,512–115,600)	135,145 (131,060–138,671)	61,842 (51,925–72,880)	54,282 (42,514–78,252)	2,619 (2,434–2,811)	5,059 (4,355–6,009)	1,571 (1,047–2,230)	1,473 (1,109–1,909)	0 (0–0)	6,806 (6,007–7,805)
Trinidad and Tobago	Prevalence Number: Cumulative change (%)	-30.8 (-31.6 to -29.9)	-34.2 (-41.4 to -26.2)	-28.9 (-31.6 to -26.2)	-39.1 (-43.8 to -33.9)	-37.0 (-58.6 to -8.6)	-24.9 (-28.0 to -21.4)	-28.1 (-41.9 to -12.5)	-32.7 (-60.4 to 5.2)	-40.8 (-61.6 to -17.1)	35.1 (-41.2 to 297.8)	-35.2 (-43.6 to -26.1)
Trinidad and Tobago	Prevalence Rate: 1990	91,628.9 (90,815.3–92,387.9)	30,134.4 (28,108.9–32,259.6)	36,128.3 (35,046.3–37,053.1)	18,700.1 (15,696.9–21,919.3)	16,644.7 (13,476.3–23,984.6)	659.3 (620.1–697.8)	1,335.2 (1,152.8–1,577.9)	462.2 (335.1–618.8)	484.3 (357.3–617.7)	0.0 (0.0–0.0)	2,034.3 (1,802.6–2,318.4)
Trinidad and Tobago	Prevalence Rate: 2015	91,167.4 (90,201.9–92,013.7)	28,553.8 (26,101.4–31,271.2)	36,593.6 (35,491.0–37,540.1)	16,628.3 (13,954.1–19,592.9)	14,684.9 (11,500.7–21,167.6)	713.6 (663.2–766.1)	1,368.8 (1,178.8–1,625.7)	428.7 (285.7–608.3)	398.9 (300.6–516.8)	0.0 (0.0–0.0)	1,861.8 (1,644.9–2,132.3)
Trinidad and Tobago	Prevalence Rate: Cumulative change (%)	-0.5 (-1.7 to 0.7)	-5.1 (-15.6 to 6.4)	1.3 (-2.5 to 5.2)	-11.0 (-17.8 to -3.5)	-9.6 (-40.5 to 31.3)	8.3 (3.8–13.3)	3.3 (-16.4 to 25.7)	-4.1 (-43.5 to 49.9)	-15.5 (-45.0 to 18.7)	99.3 (-13.5 to 491.8)	-8.2 (-20.0 to 4.6)
Trinidad and Tobago	YLDs Number: 1990	34,456 (26,093–43,768)	6,159 (4,367–8,326)	5,048 (3,510–6,907)	4,604 (3,030–6,316)	508 (361–681)	569 (410–750)	625 (435–851)	300 (183–445)	966 (621–1,387)	0 (0–0)	1,505 (1,170–1,895)
Trinidad and Tobago	YLDs Number: 2015	23,370 (17,757–29,670)	4,013 (2,800–5,578)	3,536 (2,483–4,831)	2,806 (1,846–3,834)	333 (234–455)	427 (303–562)	480 (323–676)	195 (112–304)	363 (238–530)	0 (0–0)	871 (684–1,099)
Trinidad and Tobago	YLDs Number: Cumulative change (%)	-32.2 (-34.5 to -29.6)	-34.7 (-43.1 to -24.8)	-30.0 (-31.3 to -28.7)	-39.0 (-43.7 to -33.8)	-34.4 (-42.4 to -26.2)	-24.8 (-29.5 to -19.9)	-23.1 (-34.0 to -10.1)	-32.8 (-60.9 to 4.0)	-61.3 (-75.8 to -44.8)	40.8 (-46.0 to 342.7)	-42.1 (-45.6 to -38.7)
Trinidad and Tobago	YLDs Rate: 1990	6,513.3 (4,938.1–8,270.7)	1,141.5 (808.6–1,544.9)	947.3 (659.7–1,301.3)	847.7 (557.8–1,155.1)	94.4 (67.0–126.9)	107.6 (77.7–141.7)	117.9 (82.2–160.2)	57.6 (35.0–85.3)	183.2 (117.8–261.7)	0.0 (0.0–0.0)	283.0 (219.9–355.9)
Trinidad and Tobago	YLDs Rate: 2015	6,326.6 (4,807.5–8,028.3)	1,084.1 (757.1–1,507.3)	954.9 (670.3–1,303.6)	754.5 (496.2–1,030.3)	89.9 (63.4–122.8)	116.5 (82.7–153.2)	130.0 (87.6–182.9)	53.3 (30.8–83.0)	98.4 (64.5–143.7)	0.0 (0.0–0.0)	235.6 (185.1–297.3)
Trinidad and Tobago	YLDs Rate: Cumulative change (%)	-2.8 (-6.2 to 0.8)	-4.8 (-17.2 to 9.5)	0.8 (-1.0 to 2.7)	-10.9 (-17.7 to -3.3)	-4.6 (-16.3 to 7.1)	8.4 (1.7–15.2)	10.5 (-5.3 to 29.2)	-4.1 (-44.2 to 48.3)	-44.7 (-65.5 to -21.2)	109.5 (-20.0 to 563.9)	-16.7 (-21.8 to -11.7)
Virgin Islands, U.S.	Prevalence Number: 1990	38,591 (38,196–38,945)	13,417 (12,680–14,108)	15,076 (14,603–15,494)	6,460 (5,390–7,526)	6,748 (5,426–9,552)	270 (256–285)	619 (536–733)	156 (104–225)	131 (96–169)	0 (0–0)	763 (676–866)
Virgin Islands, U.S.	Prevalence Number: 2015	26,146 (25,890–26,406)	8,314 (7,817–8,824)	10,445 (10,115–10,729)	4,043 (3,297–4,794)	4,181 (3,222–6,260)	176 (167–186)	419 (363–493)	70 (45–100)	80 (57–104)	0 (0–0)	406 (361–461)
Virgin Islands, U.S.	Prevalence Number: Cumulative change (%)	-32.2 (-33.1 to -31.4)	-37.9 (-43.4 to -32.4)	-30.7 (-33.3 to -27.8)	-37.3 (-45.0 to -29.7)	-36.5 (-58.8 to -3.1)	-34.7 (-36.8 to -32.7)	-31.7 (-44.5 to -16.8)	-52.7 (-73.6 to -20.2)	-37.6 (-57.7 to -10.9)	36.7 (-42.0 to 298.9)	-46.6 (-53.1 to -39.6)
Virgin Islands, U.S.	Prevalence Rate: 1990	91,030.3 (90,122.6–91,843.9)	31,552.0 (29,970.3–33,117.0)	35,635.3 (34,522.3–36,630.7)	15,484.9 (12,910.6–18,061.1)	15,891.0 (12,780.3–22,492.6)	609.3 (577.9–641.4)	1,457.5 (1,262.2–1,726.7)	338.6 (226.9–488.0)	311.8 (229.4–401.2)	0.0 (0.0–0.0)	1,776.0 (1,574.2–2,018.7)
Virgin Islands, U.S.	Prevalence Rate: 2015	90,332.1 (89,444.9–91,239.3)	28,882.9 (27,136.5–30,666.0)	35,998.6 (34,860.6–36,977.9)	13,931.5 (11,359.1–16,513.8)	14,462.7 (11,148.5–21,654.8)	621.9 (590.9–656.1)	1,452.6 (1,257.9–1,708.1)	246.3 (161.8–354.9)	276.1 (197.4–360.2)	0.0 (0.0–0.0)	1,433.6 (1,277.6–1,624.6)
Virgin Islands, U.S.	Prevalence Rate: Cumulative change (%)	-0.8 (-2.0 to 0.5)	-8.4 (-16.2 to -0.3)	1.0 (-2.7 to 5.3)	-9.9 (-21.0 to 1.1)	-6.7 (-39.4 to 42.2)	2.1 (-1.0 to 5.2)	0.4 (-18.4 to 22.3)	-23.2 (-57.2 to 29.5)	-9.3 (-38.7 to 29.8)	103.6 (-13.4 to 489.1)	-19.0 (-29.0 to -8.3)
Virgin Islands, U.S.	YLDs Number: 1990	2,597 (1,963–3,304)	503 (357–684)	371 (258–516)	293 (193–405)	41 (29–56)	44 (31–58)	44 (30–60)	19 (11–31)	50 (32–72)	0 (0–0)	101 (79–130)

© 2017 Global Burden of Disease Child and Adolescent Health Collaboration. JAMA Pediatrics.

eTable 3. Prevalent Cases, Rates (per 100,000 population), Years Lived with Disability (YLDs), and Cumulative Percent Change with 95% Uncertainty Interval (UI) for the Top 10 Global Causes of YLDs in Children and Adolescents in 195 Countries and Territories, Aged 0 to 19 Years, Both Sexes, 1990 and 2015. *Note: best viewed by enlarging in browser.*

Location	Measure	All causes	Iron-deficiency anemia	Skin and subcutaneous diseases	Asthma	Hemoglobinopathies and hemolytic anemias	Diarrheal diseases	Congenital anomalies	Protein-energy malnutrition	Epilepsy	Malaria	Neonatal preterm birth complications
Virgin Islands, U.S.	YLDs Number: 2015	1,689 (1,278–2,143)	311 (218–418)	255 (177–354)	183 (119–254)	26 (18–35)	28 (20–38)	48 (31–69)	8 (4–13)	19 (12–28)	0 (0–0)	45 (35–57)
Virgin Islands, U.S.	YLDs Number: Cumulative change (%)	-34.9 (-37.0 to -32.8)	-38.0 (-44.7 to -31.9)	-31.2 (-32.5 to -29.8)	-37.3 (-45.0 to -29.6)	-37.0 (-44.1 to -30.0)	-34.7 (-38.0 to -31.2)	9.5 (-11.7 to 33.5)	-52.6 (-73.9 to -20.4)	-60.1 (-73.9 to -42.3)	39.9 (-45.4 to 338.7)	-55.8 (-59.0 to -52.1)
Virgin Islands, U.S.	YLDs Rate: 1990	6,168.2 (4,664.8–7,832.0)	1,196.0 (852.9–1,621.2)	875.5 (609.1–1,216.3)	702.9 (463.0–970.3)	99.9 (71.8–134.9)	99.6 (71.4–131.7)	104.1 (72.3–141.7)	42.3 (24.9–68.0)	120.6 (76.6–172.2)	0.0 (0.0–0.0)	239.9 (185.8–307.1)
Virgin Islands, U.S.	YLDs Rate: 2015	5,832.3 (4,414.2–7,399.5)	1,083.2 (758.3–1,453.2)	881.7 (611.2–1,222.5)	632.8 (411.6–876.9)	91.4 (64.2–123.6)	101.8 (73.9–134.8)	167.6 (110.0–242.0)	30.8 (17.2–48.0)	68.2 (42.1–98.9)	0.0 (0.0–0.0)	155.7 (121.1–198.2)
Virgin Islands, U.S.	YLDs Rate: Cumulative change (%)	-5.4 (-8.4 to -2.4)	-9.3 (-19.0 to -0.2)	0.7 (-1.2 to 2.6)	-9.8 (-21.0 to 1.3)	-8.4 (-19.0 to 1.5)	2.3 (-3.1 to 7.7)	61.1 (30.0–96.5)	-23.1 (-57.6 to 29.0)	-41.9 (-62.1 to -16.1)	104.8 (-19.6 to 538.0)	-35.0 (-39.7 to -29.8)
Tropical Latin America	Prevalence Number: 1990	65,480,781 (65,351,343–65,601,611)	20,964,563 (20,492,011–21,436,989)	24,270,966 (24,015,570–24,518,646)	11,983,515 (10,184,025–13,792,070)	13,081,858 (12,870,854–13,298,516)	1,045,266 (985,051–1,112,287)	756,565 (704,891–819,597)	210,730 (179,045–249,419)	241,288 (212,493–271,393)	150,278 (90,431–229,624)	742,728 (664,102–824,738)
Tropical Latin America	Prevalence Number: 2015	62,922,628 (62,795,917–63,044,167)	18,749,359 (18,364,698–19,156,972)	24,326,804 (24,084,073–24,563,281)	9,541,805 (8,283,341–10,890,322)	11,600,835 (11,423,386–11,784,394)	588,023 (557,606–622,551)	951,436 (894,189–1,018,085)	113,333 (94,905–135,236)	201,109 (176,617–227,527)	155,544 (91,716–244,808)	781,985 (711,757–859,797)
Tropical Latin America	Prevalence Number: Cumulative change (%)	-3.9 (-4.1 to -3.7)	-10.6 (-13.2 to -8.1)	0.2 (-0.8 to 1.3)	-20.3 (-24.5 to -15.9)	-11.3 (-12.9 to -9.8)	-43.7 (-45.7 to -42.0)	26.0 (16.0–35.7)	-45.6 (-58.1 to -31.3)	-16.5 (-24.4 to -7.8)	3.2 (-4.9 to 10.4)	5.4 (-0.6 to 11.5)
Tropical Latin America	Prevalence Rate: 1990	92,736.8 (92,554.6–92,904.9)	29,589.4 (28,917.9–30,270.9)	34,613.6 (34,253.7–34,958.3)	16,738.6 (14,231.3–19,225.1)	18,523.7 (18,225.9–18,831.7)	1,475.7 (1,390.4–1,569.9)	1,070.9 (997.7–1,160.2)	298.0 (253.2–352.7)	342.3 (301.5–384.2)	207.8 (125.6–317.5)	1,058.8 (946.9–1,175.5)
Tropical Latin America	Prevalence Rate: 2015	92,100.5 (91,903.6–92,286.0)	28,290.9 (27,672.6–28,941.0)	34,887.8 (34,553.4–35,239.9)	13,938.6 (12,057.1–15,916.4)	17,051.0 (16,790.3–17,322.7)	943.2 (891.4–1,001.3)	1,400.8 (1,317.2–1,499.0)	190.1 (159.1–226.8)	296.0 (260.1–335.0)	247.0 (144.6–393.1)	1,201.7 (1,095.9–1,319.8)
Tropical Latin America	Prevalence Rate: Cumulative change (%)	-0.7 (-0.9 to -0.4)	-4.4 (-7.3 to -1.7)	0.8 (-0.2 to 1.9)	-16.6 (-20.6 to -12.6)	-7.9 (-9.5 to -6.4)	-36.1 (-38.3 to -34.0)	31.0 (20.7–41.2)	-35.5 (-50.3 to -18.5)	-13.4 (-21.3 to -4.4)	18.2 (8.8–27.5)	13.6 (7.2–20.0)
Tropical Latin America	YLDs Number: 1990	4,557,724 (3,449,862–5,805,987)	817,273 (587,193–1,102,533)	547,190 (373,733–764,934)	543,355 (362,158–740,971)	58,747 (42,166–78,834)	170,310 (123,161–224,621)	62,188 (42,708–87,025)	26,090 (17,399–36,523)	90,684 (65,651–117,066)	5,098 (2,891–8,041)	84,713 (66,070–106,354)
Tropical Latin America	YLDs Number: 2015	4,242,662 (3,227,496–5,394,068)	718,422 (511,333–960,519)	537,515 (368,082–753,887)	433,087 (291,152–585,207)	51,193 (36,558–68,673)	95,950 (68,836–126,837)	91,862 (62,799–127,772)	14,096 (9,451–20,039)	50,513 (35,596–66,375)	5,329 (3,008–8,303)	92,446 (72,029–116,437)
Tropical Latin America	YLDs Number: Cumulative change (%)	-6.9 (-8.2 to -5.6)	-12.1 (-15.4 to -9.1)	-1.8 (-3.6 to -0.1)	-20.2 (-24.5 to -15.9)	-12.9 (-16.2 to -9.9)	-43.6 (-45.7 to -41.8)	48.1 (30.8–67.0)	-45.4 (-57.8 to -30.9)	-44.2 (-51.1 to -37.3)	4.7 (-3.2 to 12.7)	9.2 (3.4–14.9)
Tropical Latin America	YLDs Rate: 1990	6,486.9 (4,906.9–8,258.6)	1,145.0 (822.1–1,544.7)	776.4 (529.4–1,085.5)	758.9 (506.7–1,033.1)	82.2 (59.1–110.3)	240.4 (173.9–317.0)	88.0 (60.5–123.2)	36.9 (24.6–51.6)	128.6 (93.0–166.1)	7.0 (4.0–11.0)	119.7 (93.4–150.4)
Tropical Latin America	YLDs Rate: 2015	6,105.9 (4,647.3–7,776.3)	1,090.6 (776.0–1,459.2)	781.0 (535.4–1,089.2)	632.8 (425.6–861.0)	76.0 (54.3–101.9)	154.0 (110.6–203.6)	135.4 (92.6–188.4)	23.6 (15.8–33.6)	74.4 (52.4–97.6)	8.4 (4.7–13.2)	136.3 (106.2–171.8)
Tropical Latin America	YLDs Rate: Cumulative change (%)	-5.9 (-7.1 to -4.7)	-4.7 (-8.4 to -1.4)	0.6 (-0.8 to 2.1)	-16.5 (-20.4 to -12.4)	-7.6 (-11.1 to -4.4)	-36.0 (-38.3 to -33.9)	54.3 (36.1–73.9)	-35.2 (-50.0 to -18.0)	-42.1 (-49.1 to -34.9)	20.3 (10.9–29.5)	13.9 (7.7–19.9)
Brazil	Prevalence Number: 1990	63,495,081 (63,364,813–63,617,628)	20,370,700 (19,894,966–20,846,074)	23,506,472 (23,251,875–23,751,863)	11,738,769 (9,974,487–13,505,361)	12,679,624 (12,469,154–12,893,522)	1,019,014 (960,287–1,085,380)	730,226 (678,753–790,873)	208,163 (176,546–246,894)	234,129 (206,256–263,296)	150,263 (90,418–229,604)	715,749 (639,663–794,037)
Brazil	Prevalence Number: 2015	60,443,476 (60,316,114–60,564,538)	17,995,143 (17,617,336–18,390,635)	23,335,146 (23,098,062–23,569,212)	9,279,726 (8,054,668–10,586,584)	11,152,520 (10,971,674–11,337,729)	565,298 (535,802–598,764)	915,565 (859,114–980,897)	109,662 (91,334–131,081)	193,030 (169,288–219,031)	155,533 (91,705–244,794)	751,241 (683,500–825,540)
Brazil	Prevalence Number: Cumulative change (%)	-4.8 (-5.0 to -4.6)	-11.6 (-14.3 to -9.2)	-0.7 (-1.7 to 0.4)	-20.8 (-25.1 to -16.5)	-12.0 (-13.6 to -10.5)	-44.5 (-46.6 to -42.7)	25.6 (15.3–35.6)	-46.7 (-59.2 to -32.3)	-17.4 (-25.4 to -8.6)	3.2 (-4.9 to 10.4)	5.1 (-1.1 to 11.4)
Brazil	Prevalence Rate: 1990	92,762.7 (92,574.1–92,939.9)	29,689.5 (28,983.4–30,389.2)	34,561.8 (34,192.7–34,916.4)	16,908.7 (14,365.3–19,412.6)	18,523.5 (18,216.2–18,836.0)	1,489.9 (1,404.2–1,586.3)	1,066.6 (991.6–1,155.6)	305.3 (259.0–362.0)	342.7 (301.6–385.0)	214.8 (129.8–328.3)	1,055.2 (944.3–1,171.1)
Brazil	Prevalence Rate: 2015	92,117.3 (91,912.7–92,317.0)	28,297.2 (27,659.6–28,965.4)	34,821.4 (34,481.0–35,181.0)	14,111.2 (12,190.8–16,094.5)	17,069.2 (16,792.9–17,354.3)	946.7 (894.9–1,005.7)	1,403.7 (1,317.2–1,504.1)	192.3 (160.2–229.9)	296.1 (259.9–335.7)	257.9 (150.9–410.5)	1,203.7 (1,096.8–1,319.9)
Brazil	Prevalence Rate: Cumulative change (%)	-0.7 (-1.0 to -0.4)	-4.7 (-7.6 to -1.9)	0.8 (-0.3 to 1.9)	-16.5 (-20.5 to -12.4)	-7.8 (-9.5 to -6.2)	-36.4 (-38.8 to -34.4)	31.8 (21.0–42.3)	-36.3 (-51.2 to -19.0)	-13.5 (-21.7 to -4.5)	19.4 (9.8–28.7)	14.2 (7.7–20.9)
Brazil	YLDs Number: 1990	4,428,695 (3,349,935–5,644,641)	795,107 (569,797–1,071,393)	526,653 (359,725–737,539)	532,252 (354,815–726,045)	57,026 (40,911–76,455)	166,007 (119,973–219,127)	60,296 (41,248–84,383)	25,771 (17,171–36,203)	87,990 (63,679–113,635)	5,098 (2,890–8,041)	81,530 (63,434–102,351)
Brazil	YLDs Number: 2015	4,079,217 (3,104,510–5,186,704)	689,946 (491,281–923,561)	511,753 (349,891–719,563)	421,191 (284,037–569,051)	49,176 (35,118–65,941)	92,233 (66,124–121,979)	88,487 (60,547–123,259)	13,639 (9,138–19,388)	48,463 (33,997–63,430)	5,329 (3,007–8,303)	88,979 (69,293–111,993)
Brazil	YLDs Number: Cumulative change (%)	-7.9 (-9.2 to -6.6)	-13.2 (-16.5 to -10.2)	-2.9 (-4.6 to -1.0)	-20.8 (-25.1 to -16.5)	-13.8 (-17.2 to -10.8)	-44.4 (-46.5 to -42.6)	47.2 (29.5–66.5)	-46.5 (-59.0 to -31.6)	-44.9 (-51.7 to -37.7)	4.7 (-3.2 to 12.7)	9.2 (3.1–15.1)
Brazil	YLDs Rate: 1990	6,499.5 (4,917.2–8,277.6)	1,150.4 (824.5–1,549.9)	770.7 (525.8–1,078.3)	766.6 (511.7–1,045.6)	82.4 (59.2–110.3)	242.7 (175.4–320.3)	88.1 (60.2–123.3)	37.8 (25.2–53.1)	128.8 (93.1–166.3)	7.2 (4.1–11.4)	118.9 (92.6–149.3)
Brazil	YLDs Rate: 2015	6,110.1 (4,648.5–7,782.7)	1,091.6 (777.1–1,460.3)	773.7 (530.4–1,081.8)	640.7 (431.4–870.1)	76.1 (54.2–101.9)	154.5 (110.8–204.6)	135.9 (92.9–189.1)	23.9 (16.0–34.0)	74.4 (52.5–97.7)	8.8 (4.9–13.7)	136.6 (106.4–171.9)
Brazil	YLDs Rate: Cumulative change (%)	-6.0 (-7.2 to -4.8)	-5.1 (-8.9 to -1.7)	0.4 (-1.1 to 1.9)	-16.3 (-20.4 to -12.2)	-7.7 (-11.4 to -4.5)	-36.3 (-38.7 to -34.2)	54.7 (36.1–75.1)	-36.0 (-50.9 to -18.1)	-42.2 (-49.3 to -34.9)	21.5 (12.0–30.8)	14.9 (8.4–21.2)
Paraguay	Prevalence Number: 1990	1,985,699 (1,971,300–1,999,968)	593,863 (552,120–638,696)	764,494 (739,245–788,358)	244,745 (198,951–297,462)	402,234 (384,070–420,662)	26,251 (23,377–28,135)	26,338 (22,383–31,613)	2,567 (1,710–3,532)	7,159 (5,291–9,137)	15 (4–28)	26,978 (23,471–31,539)
Paraguay	Prevalence Number: 2015	2,479,152 (2,462,583–2,494,927)	754,216 (697,531–802,106)	991,658 (960,682–1,020,251)	262,079 (215,417–314,340)	448,315 (428,062–470,573)	22,725 (21,088–24,533)	35,871 (30,926–41,948)	3,671 (1,864–6,385)	8,078 (5,926–10,407)	10 (2–19)	30,744 (26,728–35,791)
Paraguay	Prevalence Number: Cumulative change (%)	24.9 (23.7–26.0)	27.2 (16.4–39.2)	29.8 (24.4–35.2)	7.6 (-9.5 to 19.4)	11.5 (4.9–18.6)	-13.2 (-20.5 to -4.4)	37.4 (11.3–69.3)	50.2 (-29.8 to 185.1)	15.7 (-22.3 to 65.9)	-31.8 (-31.9 to -31.9)	14.6 (-2.0 to 33.9)
Paraguay	Prevalence Rate: 1990	92,025.0 (91,422.6–92,625.0)	26,723.8 (25,059.0–28,509.8)	36,200.6 (35,041.5–37,297.0)	11,317.8 (9,228.7–13,683.0)	18,530.2 (17,698.6–19,379.5)	1,064.6 (950.8–1,138.9)	1,208.7 (1,027.5–1,452.4)	101.7 (67.8–139.8)	340.4 (251.1–435.2)	0.7 (0.2–1.3)	1,173.4 (1,017.1–1,382.7)
Paraguay	Prevalence Rate: 2015	91,717.0 (91,087.9–92,314.2)	28,200.4 (26,079.5–29,988.1)	36,445.0 (35,289.7–37,504.1)	9,729.6 (7,982.7–11,698.3)	16,612.5 (15,867.5–17,437.0)	863.0 (800.1–932.3)	1,329.9 (1,146.8–1,553.7)	140.7 (71.4–244.7)	297.0 (218.6–383.0)	0.4 (0.1–0.7)	1,154.4 (1,003.3–1,342.5)
Paraguay	Prevalence Rate: Cumulative change (%)	-0.3 (-1.2 to 0.6)	5.7 (-2.9 to 15.4)	0.7 (-3.4 to 4.8)	-13.7 (-27.7 to -4.3)	-10.3 (-15.6 to -4.6)	-18.8 (-25.5 to -10.7)	11.0 (-10.0 to 36.6)	45.3 (-32.1 to 175.9)	-10.6 (-40.0 to 28.0)	-44.0 (-44.2 to -44.1)	-1.0 (-15.6 to 15.9)
Paraguay	YLDs Number: 1990	129,029 (98,019–164,555)	22,166 (15,318–30,164)	20,536 (14,347–28,287)	11,102 (7,234–15,604)	1,721 (1,205–2,335)	4,302 (3,105–5,765)	1,891 (1,317–2,579)	318 (183–502)	2,693 (1,703–3,776)	0 (0–0)	3,182 (2,491–3,985)
Paraguay	YLDs Number: 2015	163,445 (124,167–207,309)	28,476 (20,340–38,600)	25,762 (17,974–35,601)	11,896 (7,798–16,336)	2,016 (1,439–2,729)	3,716 (2,681–4,909)	3,375 (2,240–4,789)	456 (211–849)	2,049 (1,341–2,952)	0 (0–0)	3,467 (2,672–4,435)
Paraguay	YLDs Number: Cumulative change (%)	26.7 (22.8–30.5)	28.9 (14.4–42.5)	25.4 (22.3–28.1)	7.6 (-9.4 to 19.6)	17.3 (8.3–26.2)	-13.4 (-21.7 to -3.8)	79.2 (46.6–131.0)	51.1 (-30.3 to 189.5)	-21.7 (-48.2 to 14.3)	-31.7 (-31.9 to -31.9)	9.0 (0.7–17.8)
Paraguay	YLDs Rate: 1990	6,120.8 (4,647.6–7,788.6)	993.8 (688.3–1,349.7)	951.1 (661.9–1,311.3)	513.2 (337.5–720.7)	78.5 (55.4–106.6)	174.4 (125.9–233.3)	86.8 (60.3–118.3)	12.6 (7.3–19.9)	127.9 (80.4–178.7)	0.0 (0.0–0.0)	145.5 (114.0–181.9)
Paraguay	YLDs Rate: 2015	6,009.2 (4,564.7–7,626.0)	1,070.5 (764.1–1,451.3)	953.0 (664.3–1,316.1)	441.7 (290.1–606.2)	75.6 (54.1–102.4)	141.1 (101.9–186.4)	125.2 (83.1–177.5)	17.5 (8.1–32.5)	75.4 (49.2–108.5)	0.0 (0.0–0.0)	128.7 (99.1–164.8)
Paraguay	YLDs Rate: Cumulative change (%)	-1.8 (-4.6 to 1.0)	8.0 (-3.5 to 19.1)	0.2 (-1.8 to 2.0)	-13.6 (-27.6 to -4.2)	-3.5 (-10.9 to 3.4)	-18.9 (-26.6 to -9.9)	45.0 (18.6–86.7)	46.2 (-32.5 to 180.0)	-39.4 (-59.9 to -10.9)	-44.0 (-44.2 to -44.1)	-11.6 (-18.2 to -4.6)
Southeast Asia, East Asia, and Oceania	Prevalence Number: 1990	652,848,585 (651,891,934–653,739,208)	172,734,222 (170,403,961–174,950,786)	255,791,585 (253,437,247–258,146,120)	37,952,206 (32,158,063–44,107,383)	140,052,684 (138,732,556–141,489,175)	5,088,695 (4,706,624–5,519,299)	8,507,897 (8,014,054–9,012,070)	4,824,476 (4,273,006–5,509,824)	1,799,512 (1,621,580–1,996,791)	11,845,454 (9,031,561–14,708,209)	6,793,864 (6,140,883–7,505,384)
Southeast Asia, East Asia, and Oceania	Prevalence Number: 2015	508,722,861 (507,650,833–509,852,407)	126,174,820 (124,239,255–127,934,699)	213,068,635 (210,705,581–215,457,185)	28,035,625 (23,518,095–32,888,784)	106,721,501 (105,542,252–107,880,853)	2,805,703 (2,651,475–2,955,147)	9,110,148 (8,655,087–9,624,144)	2,948,521 (2,527,686–3,396,152)	1,552,970 (1,366,256–1,766,802)	7,302,817 (5,814,109–9,319,638)	5,545,902 (4,994,995–6,110,242)
Southeast Asia, East Asia, and Oceania	Prevalence Number: Cumulative change (%)	-22.1 (-22.3 to -21.9)	-26.9 (-28.6 to -25.4)	-16.7 (-17.6 to -15.8)	-26.1 (-28.9 to -22.9)	-23.8 (-24.7 to -22.9)	-44.8 (-47.1 to -42.4)	7.2 (0.5–14.3)	-38.5 (-49.7 to -25.8)	-13.7 (-20.8 to -6.0)	-37.9 (-48.2 to -29.0)	-18.3 (-22.7 to -13.6)
Southeast Asia, East Asia, and Oceania	Prevalence Rate: 1990	94,818.8 (94,679.8–94,947.2)	25,226.8 (24,909.3–25,542.5)	36,960.2 (36,628.9–37,314.1)	5,622.2 (4,764.8–6,528.2)	20,340.7 (20,150.0–20,552.7)	723.4 (670.0–783.6)	1,234.4 (1,162.3–1,307.4)	670.3 (593.7–765.4)	261.4 (235.2–290.1)	1,745.8 (1,332.0–2,177.5)	971.9 (878.3–1,075.8)
Southeast Asia, East Asia, and Oceania	Prevalence Rate: 2015	90,156.8 (89,963.1–90,355.5)	22,505.1 (22,156.9–22,821.8)	37,525.9 (37,121.1–37,949.0)	4,982.8 (4,175.0–5,847.3)	18,933.3 (18,724.3–19,138.2)	502.4 (474.6–529.1)	1,616.1 (1,535.4–1,707.1)	529.7 (454.1–610.1)	275.9 (242.5–313.8)	1,297.2 (1,031.3–1,659.8)	992.2 (894.1–1,093.7)
Southeast Asia, East Asia, and Oceania	Prevalence Rate: Cumulative change (%)	-4.9 (-5.1 to -4.7)	-10.8 (-12.7 to -8.9)	1.5 (0.4–2.7)	-11.4 (-14.7 to -7.5)	-6.9 (-8.0 to -5.9)	-30.5 (-33.3 to -27.5)	31.1 (22.8–39.7)	-20.5 (-35.0 to -4.1)	5.6 (-3.0 to 14.9)	-25.2 (-37.9 to -14.6)	2.1 (-3.3 to 8.1)
Southeast Asia, East Asia, and Oceania	YLDs Number: 1990	40,241,602 (30,401,853–51,699,269)	6,469,139 (4,635,344–8,702,169)	5,412,946 (3,611,904–7,740,326)	1,718,632 (1,149,403–2,354,369)	1,512,474 (1,090,710–2,032,767)	830,541 (603,469–1,100,735)	537,760 (347,661–778,719)	601,289 (408,868–822,429)	704,032 (513,246–900,240)	261,055 (182,796–351,328)	898,448 (714,545–1,109,694)
Southeast Asia, East Asia, and Oceania	YLDs Number: 2015	29,082,285 (22,083,705–37,126,074)	4,663,123 (3,331,264–6,284,280)	4,510,597 (3,003,957–6,426,652)	1,274,260 (843,515–1,742,711)	1,217,157 (876,635–1,628,467)	459,470 (329,810–603,131)	658,318 (420,480–938,444)	368,742 (249,326–501,679)	427,975 (304,879–564,961)	155,249 (110,020–212,894)	666,427 (526,600–823,026)
Southeast Asia, East Asia, and Oceania	YLDs Number: Cumulative change (%)	-27.7 (-29.1 to -26.2)	-27.9 (-30.2 to -25.9)	-16.7 (-17.7 to -15.6)	-25.9 (-28.7 to -22.6)	-19.5 (-21.1 to -17.9)	-44.6 (-47.2 to -42.0)	23.1 (4.1–43.1)	-38.3 (-49.4 to -25.5)	-39.2 (-45.4 to -32.8)	-40.5 (-45.3 to -35.7)	-25.8 (-29.6 to -21.9)

© 2017 Global Burden of Disease Child and Adolescent Health Collaboration. JAMA Pediatrics.

eTable 3. Prevalent Cases, Rates (per 100,000 population), Years Lived with Disability (YLDs), and Cumulative Percent Change with 95% Uncertainty Interval (UI) for the Top 10 Global Causes of YLDs in Children and Adolescents in 195 Countries and Territories, Aged 0 to 19 Years, Both Sexes, 1990 and 2015. *Note: best viewed by enlarging in browser.*

Location	Measure	All causes	Iron-deficiency anemia	Skin and subcutaneous diseases	Asthma	Hemoglobinopathies and hemolytic anemias	Diarrheal diseases	Congenital anomalies	Protein-energy malnutrition	Epilepsy	Malaria	Neonatal preterm birth complications
Southeast Asia, East Asia, and Oceania	YLDs Rate: 1990	5,850.8 (4,420.0–7,528.9)	961.6 (689.7–1,291.9)	783.8 (522.7–1,119.0)	254.6 (170.0–347.8)	225.4 (162.7–303.0)	118.0 (85.9–155.9)	78.0 (50.4–113.0)	83.5 (56.8–114.3)	102.3 (74.4–130.8)	39.1 (27.4–52.6)	130.7 (103.9–161.4)
Southeast Asia, East Asia, and Oceania	YLDs Rate: 2015	5,138.8 (3,902.5–6,565.4)	836.6 (597.8–1,127.0)	796.7 (531.2–1,133.4)	226.5 (149.8–310.0)	218.2 (157.2–291.8)	82.3 (59.1–108.0)	116.8 (74.6–166.5)	66.2 (44.8–90.1)	76.0 (54.1–100.4)	27.8 (19.7–38.1)	118.4 (93.6–146.2)
Southeast Asia, East Asia, and Oceania	YLDs Rate: Cumulative change (%)	-12.1 (-13.9 to -10.3)	-13.0 (-15.7 to -10.6)	1.7 (0.4–2.9)	-11.1 (-14.5 to -7.1)	-3.2 (-5.1 to -1.3)	-30.3 (-33.3 to -27.0)	50.6 (27.3–75.1)	-20.2 (-34.6 to -3.6)	-25.7 (-33.0 to -17.7)	-28.8 (-34.5 to -23.1)	-9.4 (-14.0 to -4.8)
East Asia	Prevalence Number: 1990	440,054,739 (439,298,149–440,835,004)	104,196,128 (102,792,109–105,657,185)	173,267,047 (171,782,653–174,600,977)	21,008,733 (17,492,649–24,759,622)	88,474,123 (87,718,327–89,231,215)	2,174,130 (1,962,389–2,402,784)	5,785,533 (5,393,344–6,169,383)	2,270,166 (1,826,873–2,820,014)	1,051,177 (962,861–1,153,230)	67,462 (46,158–96,378)	3,920,918 (3,542,115–4,328,239)
East Asia	Prevalence Number: 2015	291,154,416 (290,488,509–291,820,257)	70,013,496 (69,046,076–70,962,383)	123,484,074 (122,425,831–124,494,001)	9,882,773 (8,230,783–11,628,655)	60,640,901 (60,176,134–61,126,686)	599,691 (561,214–642,007)	5,838,748 (5,522,666–6,194,102)	1,016,159 (767,333–1,318,925)	706,018 (632,068–791,295)	71,784 (46,298–116,794)	2,584,742 (2,335,326–2,846,889)
East Asia	Prevalence Number: Cumulative change (%)	-33.8 (-34.0 to -33.7)	-32.8 (-34.1 to -31.6)	-28.7 (-29.4 to -28.1)	-53.0 (-55.3 to -50.3)	-31.5 (-32.1 to -30.9)	-72.4 (-74.6 to -70.1)	1.1 (-6.2 to 8.7)	-54.4 (-69.0 to -37.0)	-32.8 (-37.9 to -27.5)	5.2 (-7.8 to 25.1)	-34.0 (-37.9 to -30.1)
East Asia	Prevalence Rate: 1990	94,306.5 (94,143.6–94,467.8)	22,678.1 (22,398.9–22,967.7)	36,701.7 (36,394.7–36,981.5)	4,647.1 (3,861.7–5,470.4)	18,953.6 (18,790.8–19,116.1)	453.4 (409.9–500.1)	1,239.0 (1,155.0–1,321.8)	459.3 (369.7–570.6)	225.1 (205.6–247.9)	14.7 (10.0–20.9)	824.2 (743.8–910.1)
East Asia	Prevalence Rate: 2015	88,314.3 (88,108.9–88,518.8)	21,384.4 (21,088.6–21,671.1)	37,206.4 (36,884.9–37,515.1)	3,008.1 (2,504.0–3,545.7)	18,420.8 (18,279.3–18,568.6)	183.9 (172.1–196.9)	1,773.2 (1,677.0–1,880.7)	311.9 (235.5–404.8)	214.8 (192.2–240.7)	22.0 (14.2–35.9)	791.3 (715.5–871.2)
East Asia	Prevalence Rate: Cumulative change (%)	-6.4 (-6.6 to -6.1)	-5.7 (-7.4 to -4.0)	1.4 (0.4–2.3)	-35.3 (-38.5 to -31.6)	-2.8 (-3.7 to -2.0)	-59.4 (-62.6 to -56.2)	43.4 (33.1–54.1)	-30.8 (-53.0 to -4.4)	-4.6 (-11.8 to 3.0)	48.3 (30.7–78.1)	-3.9 (-9.5 to 1.8)
East Asia	YLDs Number: 1990	25,093,423 (18,954,446–32,323,129)	3,766,536 (2,705,875–5,062,940)	3,537,751 (2,320,820–5,105,232)	953,016 (630,925–1,303,996)	1,176,507 (844,483–1,579,642)	357,184 (257,050–474,231)	351,818 (227,835–516,291)	284,407 (182,505–406,713)	409,351 (300,942–519,867)	1,203 (760–1,738)	505,867 (400,141–627,093)
East Asia	YLDs Number: 2015	15,445,604 (11,691,898–19,779,964)	2,583,236 (1,850,904–3,471,354)	2,497,965 (1,624,815–3,609,263)	449,977 (295,842–622,312)	851,152 (610,538–1,139,047)	98,975 (71,086–131,310)	398,918 (249,841–577,709)	127,719 (79,838–188,689)	186,159 (132,451–244,656)	1,426 (900–2,124)	315,729 (247,400–393,078)
East Asia	YLDs Number: Cumulative change (%)	-38.4 (-39.6 to -37.3)	-31.4 (-32.7 to -30.1)	-29.4 (-30.7 to -28.1)	-52.8 (-55.2 to -50.1)	-27.7 (-29.3 to -26.0)	-72.2 (-74.6 to -69.9)	14.1 (-6.9 to 37.2)	-54.2 (-68.8 to -36.7)	-54.5 (-59.0 to -49.7)	19.2 (-3.5 to 49.2)	-37.6 (-41.9 to -32.8)
East Asia	YLDs Rate: 1990	5,387.5 (4,066.7–6,952.6)	846.2 (608.3–1,135.8)	751.8 (493.6–1,086.3)	210.8 (139.5–290.1)	263.8 (189.3–353.3)	74.5 (53.6–98.8)	75.3 (48.8–110.5)	57.5 (36.9–82.3)	87.6 (64.4–111.5)	0.3 (0.2–0.4)	108.7 (86.0–134.6)
East Asia	YLDs Rate: 2015	4,669.8 (3,534.8–5,982.2)	795.6 (570.3–1,068.2)	754.5 (491.7–1,090.4)	137.0 (90.2–189.6)	261.9 (187.9–350.3)	30.4 (21.8–40.3)	121.2 (75.9–175.4)	39.2 (24.5–57.9)	56.6 (40.3–74.5)	0.4 (0.3–0.7)	96.0 (75.2–119.5)
East Asia	YLDs Rate: Cumulative change (%)	-13.3 (-15.0 to -11.6)	-6.0 (-7.7 to -4.2)	0.3 (-1.4 to 2.2)	-35.0 (-38.3 to -31.3)	-0.7 (-2.9 to 1.6)	-59.2 (-62.6 to -55.9)	61.9 (32.1–94.5)	-30.6 (-52.7 to -4.0)	-35.4 (-41.8 to -28.6)	63.4 (32.7–103.8)	-11.7 (-17.8 to -5.1)
China	Prevalence Number: 1990	426,055,016 (425,301,166–426,793,531)	100,150,257 (98,767,436–101,571,591)	167,610,462 (166,200,940–168,888,903)	20,179,348 (16,778,898–23,796,712)	86,352,020 (85,618,364–87,101,096)	2,115,225 (1,905,508–2,341,723)	5,562,799 (5,170,605–5,943,861)	2,212,311 (1,772,627–2,770,580)	1,017,277 (932,280–1,118,010)	66,242 (44,786–95,545)	3,812,040 (3,441,283–4,208,368)
China	Prevalence Number: 2015	280,171,419 (279,516,546–280,825,930)	66,803,292 (65,849,539–67,751,024)	118,621,007 (117,589,817–119,585,515)	9,343,731 (7,788,753–11,006,560)	58,994,677 (58,556,374–59,462,407)	552,602 (517,270–591,609)	5,652,488 (5,336,822–6,022,803)	973,681 (726,407–1,289,894)	680,725 (608,903–762,428)	71,145 (45,701–116,041)	2,474,579 (2,234,926–2,730,391)
China	Prevalence Number: Cumulative change (%)	-34.2 (-34.4 to -34.1)	-33.3 (-34.5 to -32.0)	-29.2 (-29.9 to -28.6)	-53.7 (-56.1 to -50.9)	-31.7 (-32.3 to -31.1)	-73.8 (-76.0 to -71.6)	1.8 (-5.8 to 9.7)	-55.1 (-69.9 to -37.6)	-33.1 (-38.3 to -27.6)	6.2 (-6.9 to 26.2)	-35.0 (-38.8 to -31.1)
China	Prevalence Rate: 1990	94,363.6 (94,197.3–94,528.2)	22,536.9 (22,253.3–22,829.0)	36,707.8 (36,400.1–36,992.8)	4,619.6 (3,831.7–5,444.6)	19,118.0 (18,955.2–19,284.6)	454.5 (410.3–502.3)	1,230.8 (1,144.1–1,315.1)	460.2 (368.7–576.3)	225.1 (206.0–247.7)	14.9 (10.1–21.3)	826.5 (744.3–912.1)
China	Prevalence Rate: 2015	88,319.9 (88,111.6–88,529.5)	21,196.2 (20,894.8–21,494.6)	37,202.5 (36,874.6–37,513.0)	2,957.6 (2,462.8–3,490.6)	18,622.3 (18,484.3–18,770.2)	175.4 (164.2–187.7)	1,783.7 (1,684.2–1,900.2)	308.8 (230.4–409.1)	215.1 (192.3–241.2)	22.6 (14.5–36.9)	785.9 (710.0–866.8)
China	Prevalence Rate: Cumulative change (%)	-6.4 (-6.7 to -6.2)	-5.9 (-7.6 to -4.2)	1.3 (0.4–2.3)	-36.0 (-39.4 to -32.2)	-2.6 (-3.4 to -1.8)	-61.3 (-64.6 to -58.2)	45.2 (34.3–56.5)	-31.6 (-54.1 to -4.8)	-4.4 (-11.8 to 3.3)	50.1 (32.4–80.0)	-4.9 (-10.4 to 1.0)
China	YLDs Number: 1990	24,275,010 (18,324,039–31,253,800)	3,615,653 (2,597,333–4,863,248)	3,426,965 (2,245,629–4,942,436)	915,378 (605,858–1,255,120)	1,157,227 (831,004–1,554,286)	347,527 (250,139–461,793)	337,583 (217,969–497,328)	277,141 (178,073–396,378)	396,234 (291,274–503,451)	1,178 (741–1,702)	491,905 (389,224–609,526)
China	YLDs Number: 2015	14,821,211 (11,214,063–18,983,149)	2,464,254 (1,764,394–3,312,380)	2,403,908 (1,566,476–3,473,146)	425,445 (279,627–588,960)	835,943 (599,187–1,119,127)	91,224 (65,470–121,015)	385,655 (240,912–558,820)	122,396 (75,633–181,629)	179,597 (127,590–236,128)	1,413 (891–2,107)	303,921 (238,785–378,714)
China	YLDs Number: Cumulative change (%)	-38.9 (-40.1 to -37.8)	-31.8 (-33.2 to -30.4)	-29.9 (-31.2 to -28.5)	-53.5 (-56.0 to -50.7)	-27.8 (-29.4 to -26.1)	-73.7 (-76.0 to -71.4)	15.0 (-6.9 to 39.4)	-55.0 (-69.8 to -37.2)	-54.7 (-59.3 to -49.9)	20.6 (-2.7 to 50.9)	-38.2 (-42.6 to -33.4)
China	YLDs Rate: 1990	5,389.7 (4,066.3–6,954.6)	840.7 (604.2–1,128.8)	752.7 (494.5–1,087.5)	209.5 (138.5–288.7)	268.5 (192.8–359.8)	74.6 (53.7–99.1)	74.6 (48.2–110.0)	57.6 (37.0–82.4)	87.6 (64.4–111.6)	0.3 (0.2–0.4)	109.2 (86.4–135.1)
China	YLDs Rate: 2015	4,662.1 (3,527.6–5,976.9)	788.3 (564.7–1,058.5)	755.3 (492.8–1,091.7)	134.7 (88.7–186.8)	267.2 (191.6–357.5)	28.9 (20.8–38.4)	121.7 (76.0–176.3)	38.8 (24.0–57.6)	56.8 (40.3–74.6)	0.5 (0.3–0.7)	96.0 (75.4–119.6)
China	YLDs Rate: Cumulative change (%)	-13.5 (-15.2 to -11.8)	-6.2 (-8.1 to -4.4)	0.3 (-1.5 to 2.3)	-35.7 (-39.0 to -31.9)	-0.5 (-2.7 to 1.8)	-61.1 (-64.4 to -57.9)	64.2 (32.9–98.9)	-31.4 (-54.0 to -4.3)	-35.2 (-41.9 to -28.3)	65.5 (34.2–106.5)	-12.1 (-18.3 to -5.4)
North Korea	Prevalence Number: 1990	7,019,358 (6,948,720–7,085,355)	2,333,175 (2,188,715–2,495,438)	2,936,832 (2,795,492–3,089,310)	428,262 (355,409–504,546)	1,191,352 (1,111,980–1,312,256)	44,380 (40,722–48,364)	99,906 (83,330–120,793)	57,831 (14,442–144,389)	18,005 (14,459–21,651)	1,220 (777–1,729)	58,716 (50,952–68,317)
North Korea	Prevalence Number: 2015	6,437,277 (6,371,985–6,509,153)	2,040,509 (1,927,043–2,152,289)	2,780,434 (2,650,234–2,922,241)	295,642 (244,710–352,821)	1,057,006 (982,512–1,167,246)	37,814 (34,474–41,165)	102,843 (86,712–123,251)	42,472 (11,677–96,026)	14,644 (11,754–17,725)	639 (434–879)	77,423 (67,339–91,550)
North Korea	Prevalence Number: Cumulative change (%)	-8.3 (-9.5 to -7.1)	-12.4 (-21.3 to -3.9)	-5.3 (-11.5 to 1.0)	-30.9 (-36.3 to -23.9)	-11.1 (-20.8 to -1.0)	-14.7 (-20.0 to -10.9)	4.0 (-19.7 to 29.8)	20.8 (-84.8 to 286.2)	-17.8 (-35.9 to 3.6)	-46.8 (-57.4 to -33.4)	32.6 (12.2–57.6)
North Korea	Prevalence Rate: 1990	90,701.1 (89,731.4–91,554.8)	30,601.9 (28,739.4–32,819.6)	37,078.0 (35,284.2–39,019.4)	5,609.3 (4,648.7–6,658.2)	15,444.1 (14,415.7–17,002.5)	594.6 (544.7–649.1)	1,295.8 (1,081.4–1,570.0)	785.3 (195.7–1,960.8)	234.6 (188.5–282.9)	15.7 (9.9–22.4)	768.1 (667.1–893.3)
North Korea	Prevalence Rate: 2015	88,078.6 (87,150.4–89,102.1)	28,222.9 (26,568.6–29,737.7)	36,992.5 (35,227.8–38,903.4)	4,020.4 (3,317.1–4,822.6)	14,504.1 (13,478.9–16,030.8)	554.2 (504.3–605.0)	1,414.8 (1,194.3–1,693.8)	639.3 (175.7–1,445.3)	200.9 (161.9–243.0)	8.8 (5.9–12.3)	1,102.3 (962.1–1,299.0)
North Korea	Prevalence Rate: Cumulative change (%)	-2.9 (-4.3 to -1.6)	-7.6 (-17.3 to 1.5)	-0.2 (-6.6 to 6.4)	-28.3 (-33.9 to -21.0)	-5.9 (-16.1 to 4.8)	-6.7 (-12.6 to -2.4)	10.3 (-14.7 to 37.9)	33.9 (-83.2 to 328.1)	-13.5 (-32.4 to 9.1)	-42.8 (-54.1 to -28.4)	44.3 (22.2–70.4)
North Korea	YLDs Number: 1990	425,182 (322,086–545,115)	86,390 (61,003–118,875)	58,242 (38,422–84,202)	19,437 (12,834–26,601)	12,223 (8,671–16,625)	7,268 (5,210–9,642)	6,570 (4,193–9,665)	7,263 (1,715–18,813)	6,899 (4,711–9,350)	25 (12–43)	8,353 (6,516–10,455)
North Korea	YLDs Number: 2015	372,830 (281,658–476,522)	76,182 (54,225–102,853)	55,120 (36,112–79,777)	13,445 (8,856–18,862)	10,998 (7,795–14,813)	6,220 (4,450–8,196)	7,059 (4,505–10,723)	5,322 (1,417–12,075)	3,850 (2,576–5,358)	13 (7–21)	8,509 (6,625–10,682)
North Korea	YLDs Number: Cumulative change (%)	-12.3 (-16.7 to -8.4)	-11.5 (-24.8 to -2.0)	-5.4 (-8.2 to -2.5)	-30.8 (-36.8 to -23.5)	-9.7 (-21.6 to -0.7)	-14.4 (-20.8 to -8.2)	9.1 (-17.4 to 46.0)	21.3 (-84.5 to 293.0)	-43.6 (-57.9 to -25.9)	-44.7 (-62.0 to -23.3)	2.1 (-7.0 to 13.0)
North Korea	YLDs Rate: 1990	5,454.6 (4,129.4–7,012.1)	1,165.0 (826.7–1,602.7)	743.3 (490.3–1,069.4)	254.6 (168.3–349.4)	165.4 (117.7–224.9)	97.4 (69.8–129.5)	85.2 (54.4–125.0)	98.6 (23.3–255.5)	89.9 (61.4–121.7)	0.3 (0.2–0.6)	108.9 (84.8–135.9)
North Korea	YLDs Rate: 2015	5,025.6 (3,787.9–6,428.4)	1,062.1 (753.2–1,436.7)	742.9 (488.3–1,069.2)	182.9 (120.8–256.7)	152.6 (108.4–205.6)	91.2 (65.1–120.3)	97.2 (62.0–147.5)	80.1 (21.3–181.8)	52.9 (35.4–73.3)	0.2 (0.1–0.3)	117.4 (91.4–147.1)
North Korea	YLDs Rate: Cumulative change (%)	-7.8 (-12.7 to -3.5)	-8.5 (-22.3 to 1.4)	-0.0 (-3.2 to 3.0)	-28.1 (-34.3 to -20.7)	-7.5 (-19.4 to 1.8)	-6.3 (-13.4 to 0.6)	15.8 (-12.6 to 55.5)	34.5 (-82.8 to 335.8)	-40.6 (-55.6 to -22.0)	-40.8 (-58.5 to -18.8)	7.9 (-1.6 to 19.7)
Taiwan	Prevalence Number: 1990	6,980,366 (6,934,671–7,019,412)	1,712,695 (1,596,855–1,815,561)	2,719,752 (2,635,497–2,807,164)	401,122 (345,789–454,490)	930,750 (900,984–967,591)	14,525 (13,373–15,822)	122,828 (104,515–147,147)	24 (10–50)	15,894 (12,635–19,260)	0 (0–0)	50,160 (43,753–57,652)
Taiwan	Prevalence Number: 2015	4,545,715 (4,508,701–4,583,047)	1,169,694 (1,132,579–1,212,178)	2,082,631 (2,019,612–2,149,937)	243,399 (202,283–286,145)	589,217 (562,144–616,928)	9,274 (8,642–9,947)	83,416 (70,779–99,645)	5 (2–10)	10,648 (8,401–13,150)	0 (0–0)	32,739 (28,165–38,073)
Taiwan	Prevalence Number: Cumulative change (%)	-34.9 (-35.5 to -34.2)	-31.6 (-36.1 to -26.1)	-23.4 (-26.6 to -20.2)	-39.3 (-46.5 to -32.4)	-36.7 (-40.4 to -32.8)	-36.1 (-39.5 to -33.0)	-31.5 (-44.9 to -14.2)	-72.7 (-89.8 to -46.6)	-32.2 (-48.3 to -12.0)	-38.0 (-46.0 to -28.8)	-34.5 (-44.4 to -24.0)
Taiwan	Prevalence Rate: 1990	94,524.7 (93,823.6–95,071.1)	23,284.3 (21,439.3–24,886.8)	35,843.2 (34,737.4–36,981.9)	5,292.5 (4,554.8–5,994.4)	12,638.7 (12,228.7–13,146.5)	223.8 (204.9–245.1)	1,672.4 (1,424.3–2,002.5)	0.4 (0.2–0.8)	219.8 (175.6–266.5)	0.0 (0.0–0.0)	714.0 (626.0–816.2)
Taiwan	Prevalence Rate: 2015	88,120.9 (87,283.4–88,953.8)	24,080.8 (23,040.0–25,025.9)	36,504.3 (35,453.1–37,618.3)	4,741.8 (3,912.9–5,592.7)	11,543.5 (11,013.2–12,077.7)	227.8 (211.2–245.2)	1,640.5 (1,396.9–1,965.0)	0.1 (0.1–0.2)	217.9 (171.5–268.6)	0.0 (0.0–0.0)	684.6 (593.3–790.1)
Taiwan	Prevalence Rate: Cumulative change (%)	-6.8 (-7.8 to -5.7)	3.6 (-3.1 to 14.0)	1.9 (-2.1 to 6.1)	-10.4 (-21.0 to -0.3)	-8.6 (-14.0 to -3.1)	1.9 (-3.9 to 7.1)	-1.0 (-20.3 to 24.1)	-58.0 (-85.6 to -14.1)	0.3 (-23.5 to 29.9)	-3.1 (-14.4 to 9.8)	-3.8 (-17.4 to 10.6)
Taiwan	YLDs Number: 1990	393,230 (298,543–509,805)	64,493 (45,662–87,140)	52,543 (34,879–75,627)	18,200 (12,283–24,823)	7,055 (4,985–9,536)	2,388 (1,675–3,191)	7,664 (4,899–11,195)	3 (1–6)	6,217 (4,188–8,520)	0 (0–0)	5,608 (4,253–7,143)
Taiwan	YLDs Number: 2015	251,562 (190,719–321,492)	42,798 (30,443–57,496)	38,936 (25,592–56,249)	11,086 (7,287–15,338)	4,210 (3,024–5,727)	1,531 (1,095–2,031)	6,203 (3,773–9,432)	0 (0–1)	2,711 (1,780–3,755)	0 (0–0)	3,298 (2,473–4,232)
Taiwan	YLDs Number: Cumulative change (%)	-35.9 (-38.9 to -33.3)	-33.5 (-37.4 to -28.5)	-26.0 (-28.1 to -23.8)	-39.1 (-46.4 to -32.0)	-40.2 (-44.5 to -35.2)	-35.7 (-41.8 to -29.3)	-18.9 (-35.8 to 3.9)	-72.7 (-89.6 to -47.0)	-55.7 (-68.0 to -41.1)	-39.4 (-50.3 to -27.2)	-41.1 (-46.8 to -34.9)
Taiwan	YLDs Rate: 1990	5,181.6 (3,928.5–6,709.5)	862.0 (607.4–1,166.1)	701.9 (466.6–1,007.4)	240.3 (161.6–327.2)	92.8 (65.5–125.4)	36.8 (25.7–49.2)	104.5 (66.8–152.6)	0.0 (0.0–0.1)	86.1 (58.0–117.9)	0.0 (0.0–0.0)	76.4 (57.8–97.3)
Taiwan	YLDs Rate: 2015	4,632.8 (3,500.7–5,951.2)	897.7 (636.2–1,218.9)	713.0 (475.4–1,021.6)	216.1 (140.8–298.6)	85.4 (60.9–115.5)	37.6 (26.9–50.1)	122.3 (74.4–186.1)	0.0 (0.0–0.0)	55.6 (36.6–77.4)	0.0 (0.0–0.0)	64.9 (48.8–84.0)

© 2017 Global Burden of Disease Child and Adolescent Health Collaboration. JAMA Pediatrics.

eTable 3. Prevalent Cases, Rates (per 100,000 population), Years Lived with Disability (YLDs), and Cumulative Percent Change with 95% Uncertainty Interval (UI) for the Top 10 Global Causes of YLDs in Children and Adolescents in 195 Countries and Territories, Aged 0 to 19 Years, Both Sexes, 1990 and 2015. *Note: best viewed by enlarging in browser.*

Location	Measure	All causes	Iron-deficiency anemia	Skin and subcutaneous diseases	Asthma	Hemoglobinopathies and hemolytic anemias	Diarrheal diseases	Congenital anomalies	Protein-energy malnutrition	Epilepsy	Malaria	Neonatal preterm birth complications
Taiwan	YLDs Rate: Cumulative change (%)	-10.5 (-14.3 to -7.0)	4.3 (-2.7 to 14.0)	1.6 (-0.5 to 3.8)	-10.1 (-20.7 to 0.3)	-7.8 (-14.6 to 0.7)	2.4 (-7.3 to 12.5)	17.3 (-7.4 to 50.5)	-58.1 (-85.5 to -14.7)	-34.5 (-52.5 to -12.7)	-0.4 (-18.8 to 21.7)	-14.9 (-23.2 to -6.0)
Southeast Asia	Prevalence Number: 1990	209,459,107 (209,004,566–209,879,568)	67,679,540 (65,808,543–69,451,508)	81,123,399 (79,545,876–82,859,448)	16,580,915 (14,111,443–19,066,711)	50,900,464 (49,894,862–51,994,816)	2,870,280 (2,661,295–3,086,986)	2,680,473 (2,417,899–3,033,138)	2,531,987 (2,238,255–2,874,987)	739,664 (633,801–856,940)	11,350,404 (8,573,316–14,174,814)	2,812,716 (2,519,136–3,156,736)
Southeast Asia	Prevalence Number: 2015	212,766,895 (211,993,998–213,546,970)	54,989,778 (53,308,930–56,529,002)	87,545,667 (85,650,636–89,552,789)	17,619,693 (14,776,975–20,607,332)	45,115,712 (44,176,033–46,159,477)	2,162,600 (2,041,978–2,282,927)	3,206,636 (2,924,208–3,519,916)	1,908,798 (1,606,090–2,248,980)	833,383 (711,746–977,300)	6,782,640 (5,404,722–8,678,385)	2,874,530 (2,567,915–3,213,733)
Southeast Asia	Prevalence Number: Cumulative change (%)	1.6 (1.2–2.0)	-18.7 (-22.5 to -15.1)	7.9 (5.2–10.7)	6.3 (1.0–13.2)	-11.4 (-13.6 to -9.0)	-24.6 (-27.5 to -21.7)	20.0 (6.2–34.3)	-24.1 (-38.8 to -6.4)	12.9 (-1.4 to 28.7)	-39.8 (-50.3 to -30.7)	2.4 (-5.8 to 11.9)
Southeast Asia	Prevalence Rate: 1990	95,899.3 (95,690.9–96,090.6)	30,890.5 (30,047.8–31,691.0)	37,355.0 (36,631.4–38,146.6)	7,577.0 (6,457.0–8,713.7)	23,296.6 (22,836.8–23,797.0)	1,301.4 (1,206.7–1,399.5)	1,226.0 (1,106.0–1,387.2)	1,141.8 (1,009.4–1,296.5)	337.9 (289.8–391.1)	5,166.0 (3,916.0–6,437.2)	1,276.6 (1,143.2–1,434.1)
Southeast Asia	Prevalence Rate: 2015	92,671.0 (92,329.0–93,015.6)	24,110.8 (23,367.5–24,789.4)	37,908.3 (37,090.7–38,767.5)	7,688.9 (6,437.5–8,993.3)	19,665.7 (19,255.8–20,120.4)	953.2 (900.1–1,006.4)	1,398.1 (1,275.1–1,534.2)	846.0 (711.8–996.8)	363.7 (310.7–426.4)	2,957.6 (2,350.1–3,798.0)	1,264.7 (1,129.8–1,414.1)
Southeast Asia	Prevalence Rate: Cumulative change (%)	-3.4 (-3.8 to -3.0)	-21.9 (-25.5 to -18.5)	1.5 (-1.1 to 4.1)	1.5 (-3.4 to 8.0)	-15.6 (-17.7 to -13.4)	-26.7 (-29.5 to -23.9)	14.4 (1.2–28.1)	-25.4 (-39.8 to -8.0)	7.9 (-5.7 to 22.9)	-42.3 (-52.2 to -33.8)	-0.8 (-8.8 to 8.5)
Southeast Asia	YLDs Number: 1990	14,896,106 (11,212,087–19,005,761)	2,666,127 (1,913,689–3,591,381)	1,835,046 (1,244,998–2,580,977)	749,298 (504,405–1,024,840)	331,548 (239,296–444,011)	466,185 (338,632–618,274)	182,999 (118,945–265,510)	314,122 (211,451–426,935)	291,317 (209,144–381,913)	246,606 (172,720–332,071)	387,530 (310,008–478,950)
Southeast Asia	YLDs Number: 2015	13,292,217 (10,090,428–16,882,282)	2,030,377 (1,438,898–2,736,669)	1,954,826 (1,320,484–2,752,717)	800,218 (528,406–1,097,376)	359,444 (257,944–483,331)	353,453 (253,567–464,393)	254,392 (166,305–361,728)	238,088 (161,808–326,905)	236,992 (166,647–316,938)	140,513 (99,578–192,374)	343,313 (272,800–425,511)
Southeast Asia	YLDs Number: Cumulative change (%)	-10.7 (-13.5 to -7.9)	-23.9 (-28.9 to -19.4)	6.5 (5.0–8.0)	6.8 (1.2–13.8)	8.4 (3.8–12.9)	-24.1 (-27.5 to -20.9)	39.8 (19.0–66.4)	-23.7 (-38.2 to -6.2)	-18.4 (-30.5 to -5.5)	-43.0 (-47.8 to -38.0)	-11.4 (-15.9 to -6.8)
Southeast Asia	YLDs Rate: 1990	6,832.4 (5,145.1–8,714.2)	1,212.4 (870.6–1,633.6)	842.5 (570.9–1,188.4)	342.4 (230.7–467.5)	150.9 (108.9–202.2)	211.4 (153.6–280.2)	83.7 (54.4–121.4)	141.7 (96.7–192.5)	133.1 (95.5–174.4)	111.8 (78.3–150.5)	177.0 (141.6–218.7)
Southeast Asia	YLDs Rate: 2015	5,774.4 (4,384.8–7,337.3)	893.1 (633.3–1,204.3)	849.1 (574.3–1,194.2)	349.2 (230.3–479.4)	157.8 (113.3–212.2)	155.8 (111.8–204.7)	110.9 (72.5–157.8)	105.5 (71.7–144.9)	103.4 (72.7–138.3)	61.6 (43.7–84.4)	149.9 (119.1–185.7)
Southeast Asia	YLDs Rate: Cumulative change (%)	-15.4 (-18.1 to -12.8)	-26.4 (-31.1 to -22.1)	0.8 (-0.5 to 2.1)	2.0 (-3.2 to 8.7)	4.6 (0.2–8.9)	-26.2 (-29.5 to -23.1)	33.3 (13.5–58.7)	-25.0 (-39.3 to -7.9)	-22.1 (-33.7 to -9.6)	-44.9 (-49.6 to -40.1)	-15.3 (-19.6 to -10.9)
Cambodia	Prevalence Number: 1990	4,743,617 (4,727,118–4,759,244)	2,194,583 (2,060,809–2,322,996)	1,725,360 (1,643,459–1,809,037)	498,717 (415,193–587,364)	1,965,616 (1,876,603–2,060,181)	92,255 (84,809–99,677)	48,189 (38,078–60,603)	113,328 (82,594–148,354)	20,836 (16,343–25,390)	90,759 (69,028–119,153)	74,454 (62,957–89,457)
Cambodia	Prevalence Number: 2015	6,091,813 (6,058,520–6,125,845)	2,234,328 (2,182,526–2,310,273)	2,422,929 (2,325,919–2,527,962)	557,560 (468,907–653,782)	2,328,790 (2,231,583–2,424,975)	67,333 (62,638–72,789)	81,492 (67,873–99,440)	77,032 (60,971–94,655)	28,319 (22,645–34,415)	69,601 (58,680–81,569)	93,865 (80,357–110,922)
Cambodia	Prevalence Number: Cumulative change (%)	28.4 (27.7–29.3)	1.9 (-3.5 to 9.2)	40.5 (32.3–48.9)	12.0 (3.0–21.9)	18.6 (12.0–25.3)	-27.0 (-31.0 to -22.2)	71.9 (28.8–120.4)	-29.7 (-52.6 to -0.9)	37.6 (5.0–75.3)	-22.2 (-35.4 to -8.3)	27.1 (4.9–48.8)
Cambodia	Prevalence Rate: 1990	97,393.7 (97,109.2–97,668.5)	43,371.9 (41,341.1–45,353.0)	37,626.4 (35,942.0–39,375.9)	10,484.8 (8,817.5–12,278.0)	40,251.1 (38,419.8–42,165.4)	1,595.7 (1,480.7–1,712.1)	970.6 (761.9–1,218.3)	1,722.8 (1,255.6–2,254.8)	409.0 (321.4–496.5)	1,848.8 (1,435.5–2,370.9)	1,305.9 (1,086.5–1,601.2)
Cambodia	Prevalence Rate: 2015	93,595.3 (93,089.7–94,121.8)	34,191.7 (33,404.2–35,326.9)	37,387.2 (35,898.2–38,990.5)	8,626.0 (7,251.1–10,106.0)	35,771.1 (34,279.4–37,250.8)	1,013.2 (943.3–1,094.4)	1,250.8 (1,041.6–1,526.7)	1,136.6 (899.6–1,396.6)	433.7 (346.9–527.0)	1,072.4 (904.5–1,259.9)	1,419.3 (1,214.6–1,679.8)
Cambodia	Prevalence Rate: Cumulative change (%)	-3.9 (-4.4 to -3.3)	-21.1 (-24.4 to -16.3)	-0.6 (-6.2 to 5.0)	-17.6 (-23.6 to -10.9)	-11.1 (-16.0 to -6.0)	-36.5 (-39.8 to -32.9)	31.0 (-2.1 to 67.9)	-31.8 (-54.0 to -3.8)	7.4 (-17.4 to 37.4)	-41.4 (-49.8 to -32.7)	9.8 (-12.0 to 30.4)
Cambodia	YLDs Number: 1990	380,582 (289,763–487,930)	99,904 (71,538–134,031)	41,794 (28,344–58,607)	22,435 (14,968–30,813)	10,473 (7,538–13,975)	14,834 (10,666–19,695)	3,331 (2,090–5,011)	13,923 (8,414–20,695)	8,296 (5,562–11,282)	3,292 (2,139–4,805)	7,716 (5,903–9,736)
Cambodia	YLDs Number: 2015	407,547 (310,276–517,321)	88,320 (63,584–118,913)	55,713 (37,655–79,216)	25,257 (16,665–34,779)	12,248 (8,775–16,574)	10,947 (7,913–14,409)	6,012 (3,701–9,230)	9,571 (6,164–13,455)	8,273 (5,428–11,306)	2,613 (1,755–3,623)	10,417 (8,139–12,887)
Cambodia	YLDs Number: Cumulative change (%)	7.2 (2.9–12.1)	-11.4 (-19.4 to -1.9)	33.3 (28.2–38.2)	12.8 (3.3–22.9)	17.1 (6.6–27.7)	-26.1 (-30.8 to -20.7)	82.5 (47.4–146.9)	-28.9 (-52.2 to 0.1)	1.1 (-24.7 to 32.5)	-19.7 (-32.7 to -7.0)	35.6 (20.3–52.7)
Cambodia	YLDs Rate: 1990	7,922.9 (6,041.3–10,092.1)	1,948.0 (1,404.1–2,615.5)	881.8 (596.7–1,245.3)	471.4 (315.3–643.1)	208.5 (150.9–277.5)	256.5 (185.3–339.5)	67.2 (42.3–101.8)	211.7 (127.9–314.7)	162.7 (110.0–222.3)	67.6 (44.3–97.8)	151.7 (115.8–191.2)
Cambodia	YLDs Rate: 2015	6,273.3 (4,778.4–7,964.1)	1,358.6 (979.3–1,828.5)	858.0 (580.3–1,220.2)	390.7 (258.2–538.1)	189.2 (135.5–256.0)	164.7 (119.1–217.0)	92.3 (56.8–141.8)	141.2 (91.0–198.5)	126.7 (83.3–173.2)	40.5 (27.3–56.1)	159.9 (125.0–197.9)
Cambodia	YLDs Rate: Cumulative change (%)	-20.8 (-23.5 to -17.9)	-30.2 (-35.3 to -24.4)	-2.7 (-5.5 to 0.2)	-17.0 (-23.5 to -10.2)	-9.2 (-16.7 to -2.9)	-35.7 (-39.7 to -31.3)	38.9 (12.0–89.1)	-31.0 (-53.6 to -2.8)	-21.1 (-41.2 to 3.0)	-39.5 (-48.0 to -31.0)	5.8 (-6.5 to 19.3)
Indonesia	Prevalence Number: 1990	81,966,173 (81,611,116–82,297,032)	27,871,890 (26,650,299–29,060,851)	32,110,831 (30,870,575–33,407,417)	5,559,768 (4,632,521–6,504,544)	19,702,967 (18,956,962–20,478,204)	1,315,917 (1,199,447–1,447,081)	908,359 (736,651–1,147,261)	1,010,691 (737,905–1,346,382)	305,427 (243,435–372,106)	2,935,289 (2,689,759–3,224,317)	911,960 (782,258–1,089,320)
Indonesia	Prevalence Number: 2015	86,949,294 (86,319,378–87,518,774)	24,542,382 (23,058,265–25,722,662)	36,134,136 (34,718,310–37,621,160)	6,135,919 (5,076,425–7,264,169)	17,912,505 (17,205,728–18,648,790)	1,164,618 (1,086,675–1,240,711)	1,213,096 (1,013,213–1,444,674)	892,536 (619,642–1,217,119)	358,558 (281,493–439,515)	1,259,436 (1,049,239–1,494,111)	1,095,452 (946,667–1,293,694)
Indonesia	Prevalence Number: Cumulative change (%)	6.1 (5.2–6.8)	-11.8 (-19.8 to -4.1)	12.6 (7.0–18.0)	10.5 (0.6–22.1)	-9.0 (-13.8 to -4.1)	-11.4 (-17.3 to -5.8)	35.7 (1.2–72.5)	-8.6 (-44.6 to 39.2)	18.9 (-10.3 to 53.7)	-57.0 (-63.8 to -49.3)	21.0 (0.8–44.6)
Indonesia	Prevalence Rate: 1990	95,449.5 (95,035.4–95,831.5)	32,508.2 (31,015.1–33,929.1)	37,430.8 (35,985.6–38,920.0)	6,428.1 (5,365.2–7,515.0)	22,950.7 (22,081.9–23,855.7)	1,553.3 (1,414.4–1,710.4)	1,058.6 (858.6–1,337.1)	1,207.7 (881.7–1,608.9)	355.9 (283.6–433.4)	3,410.7 (3,122.4–3,746.6)	1,070.0 (918.3–1,277.2)
Indonesia	Prevalence Rate: 2015	92,260.3 (91,585.9–92,869.3)	26,167.3 (24,623.3–27,419.3)	38,128.8 (36,643.0–39,713.7)	6,537.1 (5,402.1–7,743.4)	19,015.3 (18,263.6–19,797.2)	1,240.9 (1,158.2–1,322.0)	1,288.0 (1,075.7–1,533.4)	948.8 (658.7–1,293.8)	381.2 (298.9–467.7)	1,338.7 (1,113.8–1,590.0)	1,167.9 (1,009.4–1,378.5)
Indonesia	Prevalence Rate: Cumulative change (%)	-3.3 (-4.1 to -2.7)	-19.4 (-26.7 to -12.3)	1.9 (-3.1 to 6.8)	1.8 (-7.1 to 12.3)	-17.1 (-21.5 to -12.6)	-20.0 (-25.4 to -14.9)	23.7 (-7.7 to 57.2)	-18.7 (-50.7 to 23.9)	8.5 (-18.2 to 40.2)	-60.7 (-67.0 to -53.5)	9.9 (-8.3 to 31.3)
Indonesia	YLDs Number: 1990	5,530,971 (4,197,466–7,049,915)	1,103,543 (800,236–1,499,479)	710,269 (475,421–1,013,072)	251,543 (166,841–343,090)	135,934 (98,468–183,175)	213,761 (153,643–285,022)	60,041 (38,177–89,494)	125,322 (78,458–187,955)	121,736 (82,818–165,661)	101,820 (72,198–136,620)	123,974 (96,882–154,053)
Indonesia	YLDs Number: 2015	5,388,988 (4,107,188–6,873,094)	918,665 (643,986–1,250,138)	792,405 (529,470–1,128,389)	278,805 (182,415–384,787)	152,496 (108,556–205,342)	190,332 (137,314–249,664)	93,842 (57,779–144,384)	111,176 (67,210–166,532)	104,129 (68,642–146,080)	43,546 (29,898–60,753)	134,432 (105,953–166,902)
Indonesia	YLDs Number: Cumulative change (%)	-2.5 (-6.5 to 2.8)	-16.7 (-26.9 to -6.3)	11.5 (8.7–14.6)	11.0 (1.0–22.6)	12.2 (3.8–21.4)	-10.8 (-17.3 to -4.3)	58.6 (17.5–121.8)	-8.1 (-44.0 to 39.3)	-13.2 (-37.0 to 15.1)	-57.2 (-64.0 to -49.9)	8.6 (-1.2 to 19.2)
Indonesia	YLDs Rate: 1990	6,442.3 (4,887.1–8,212.9)	1,281.1 (928.4–1,740.8)	827.5 (554.4–1,180.7)	290.8 (192.9–396.2)	157.4 (114.1–212.3)	252.3 (181.3–336.4)	70.0 (44.5–104.3)	149.8 (93.8–224.6)	141.9 (96.4–193.0)	117.5 (83.6–157.7)	144.4 (112.8–179.4)
Indonesia	YLDs Rate: 2015	5,705.5 (4,349.1–7,280.2)	983.5 (689.4–1,336.7)	838.3 (560.5–1,194.9)	297.0 (194.2–410.5)	163.3 (116.2–219.7)	202.8 (146.3–266.0)	99.7 (61.3–153.4)	118.2 (71.5–177.0)	110.7 (72.9–155.0)	46.6 (31.9–65.1)	142.9 (112.6–177.5)
Indonesia	YLDs Rate: Cumulative change (%)	-11.4 (-15.0 to -6.6)	-23.1 (-32.6 to -13.4)	1.3 (-1.2 to 3.9)	2.3 (-6.9 to 12.7)	3.8 (-4.1 to 12.5)	-19.5 (-25.4 to -13.5)	44.5 (7.2–102.4)	-18.2 (-50.2 to 24.0)	-20.9 (-42.6 to 5.1)	-60.3 (-66.7 to -53.5)	-0.9 (-9.8 to 8.8)
Laos	Prevalence Number: 1990	2,222,302 (2,211,914–2,232,493)	849,763 (804,627–885,000)	832,081 (797,067–866,773)	231,082 (192,614–273,091)	599,554 (575,865–623,245)	29,537 (27,402–32,045)	17,820 (13,669–23,786)	40,292 (30,898–50,935)	8,986 (7,135–10,929)	55,734 (47,986–65,520)	26,726 (21,959–32,942)
Laos	Prevalence Number: 2015	2,903,875 (2,882,403–2,924,867)	807,896 (745,385–859,840)	1,161,345 (1,114,468–1,209,451)	316,400 (266,416–372,126)	708,051 (682,142–734,798)	25,478 (23,471–27,914)	30,700 (24,712–38,527)	25,570 (18,619–34,526)	11,820 (9,254–14,368)	85,619 (68,721–108,746)	37,254 (30,570–46,767)
Laos	Prevalence Number: Cumulative change (%)	30.7 (29.5–31.7)	-4.9 (-11.8 to 4.1)	39.6 (32.3–47.7)	37.2 (24.8–49.8)	18.1 (12.4–23.8)	-13.7 (-18.1 to -8.1)	76.3 (26.8–134.1)	-35.0 (-56.4 to -6.7)	33.1 (2.8–71.5)	53.3 (35.2–75.1)	40.9 (11.8–76.8)
Laos	Prevalence Rate: 1990	96,304.2 (95,887.8–96,707.8)	36,010.2 (34,205.3–37,444.7)	37,171.5 (35,656.4–38,766.0)	10,208.0 (8,527.3–12,051.4)	25,911.7 (24,886.5–26,935.9)	1,154.5 (1,073.7–1,249.4)	758.9 (579.8–1,016.7)	1,464.7 (1,123.3–1,851.7)	381.9 (303.6–466.5)	2,477.7 (2,146.5–2,903.4)	1,005.6 (815.5–1,256.0)
Laos	Prevalence Rate: 2015	93,219.0 (92,520.8–93,889.5)	25,889.6 (23,900.6–27,522.4)	37,358.9 (35,866.0–38,914.8)	10,193.7 (8,585.2–11,992.3)	22,727.6 (21,897.0–23,585.8)	809.4 (746.3–886.4)	984.7 (792.7–1,235.3)	805.6 (586.7–1,087.8)	379.1 (296.9–460.8)	2,754.9 (2,209.5–3,502.5)	1,181.1 (968.7–1,485.9)
Laos	Prevalence Rate: Cumulative change (%)	-3.2 (-4.0 to -2.5)	-28.1 (-33.4 to -21.5)	0.6 (-4.6 to 6.4)	0.0 (-9.0 to 9.2)	-12.2 (-16.5 to -8.0)	-29.9 (-33.4 to -25.4)	32.8 (-4.9 to 76.3)	-43.7 (-62.2 to -19.1)	0.5 (-22.4 to 29.7)	11.0 (-2.5 to 27.5)	19.1 (-8.0 to 51.9)
Laos	YLDs Number: 1990	162,715 (123,030–209,476)	34,847 (24,851–46,659)	19,654 (13,349–27,748)	10,428 (6,976–14,352)	4,045 (2,890–5,447)	4,786 (3,471–6,333)	1,391 (853–2,147)	4,990 (3,156–7,331)	3,604 (2,458–4,869)	2,289 (1,552–3,200)	2,545 (1,847–3,365)
Laos	YLDs Number: 2015	186,332 (141,848–238,203)	30,426 (21,288–40,945)	26,651 (17,964–37,828)	14,362 (9,619–19,851)	4,906 (3,472–6,570)	4,161 (2,963–5,533)	2,397 (1,502–3,664)	3,185 (1,953–4,746)	4,297 (2,856–5,857)	3,265 (2,121–4,763)	3,947 (2,978–4,976)
Laos	YLDs Number: Cumulative change (%)	14.6 (9.8–19.5)	-12.7 (-21.4 to -2.7)	35.5 (31.2–39.7)	38.0 (25.0–50.8)	21.3 (10.0–33.9)	-13.1 (-18.6 to -6.5)	74.6 (47.9–123.4)	-34.5 (-56.3 to -5.0)	21.0 (-9.9 to 58.7)	42.3 (24.1–65.0)	56.5 (33.9–81.0)
Laos	YLDs Rate: 1990	7,125.9 (5,388.9–9,158.0)	1,469.0 (1,047.3–1,970.9)	865.3 (588.3–1,220.7)	460.6 (307.1–635.2)	172.3 (123.5–232.3)	187.0 (135.4–247.2)	59.4 (36.4–92.2)	181.4 (114.8–266.6)	153.1 (103.4–206.8)	101.2 (69.1–141.2)	105.5 (76.7–139.8)
Laos	YLDs Rate: 2015	5,988.3 (4,560.5–7,656.4)	979.0 (684.7–1,317.5)	856.8 (577.9–1,216.7)	462.7 (309.6–640.0)	158.3 (112.2–212.0)	132.2 (94.3–175.7)	76.9 (48.2–117.4)	100.4 (61.5–149.5)	137.8 (91.5–188.0)	105.5 (68.7–154.1)	126.5 (95.5–159.6)
Laos	YLDs Rate: Cumulative change (%)	-15.9 (-19.3 to -12.6)	-33.3 (-39.9 to -25.8)	-1.0 (-3.8 to 1.9)	0.6 (-8.4 to 9.6)	-8.1 (-16.7 to 1.5)	-29.3 (-33.7 to -24.0)	31.4 (11.0–68.4)	-43.3 (-62.1 to -17.7)	-8.7 (-31.8 to 20.0)	4.1 (-9.6 to 21.3)	21.1 (3.5–41.0)
Malaysia	Prevalence Number: 1990	8,152,296 (8,106,545–8,193,181)	1,697,967 (1,562,614–1,858,270)	3,101,902 (2,980,847–3,224,677)	566,717 (498,297–648,941)	1,599,794 (1,519,629–1,683,140)	62,071 (56,638–68,227)	129,415 (109,609–152,455)	138,365 (96,322–184,607)	30,583 (23,890–38,018)	484,567 (356,736–616,232)	110,873 (97,685–125,764)

© 2017 Global Burden of Disease Child and Adolescent Health Collaboration. JAMA Pediatrics.

eTable 3. Prevalent Cases, Rates (per 100,000 population), Years Lived with Disability (YLDs), and Cumulative Percent Change with 95% Uncertainty Interval (UI) for the Top 10 Global Causes of YLDs in Children and Adolescents in 195 Countries and Territories, Aged 0 to 19 Years, Both Sexes, 1990 and 2015. *Note: best viewed by enlarging in browser.*

Location	Measure	All causes	Iron-deficiency anemia	Skin and subcutaneous diseases	Asthma	Hemoglobinopathies and hemolytic anemias	Diarrheal diseases	Congenital anomalies	Protein-energy malnutrition	Epilepsy	Malaria	Neonatal preterm birth complications
Malaysia	Prevalence Number: 2015	9,641,608 (9,577,609–9,700,641)	1,329,873 (1,176,656–1,472,824)	3,915,323 (3,776,883–4,061,413)	651,042 (537,928–770,561)	1,367,034 (1,303,044–1,435,367)	50,808 (46,368–55,733)	156,660 (134,452–182,914)	98,906 (69,922–134,088)	39,122 (30,908–49,148)	286,002 (215,346–383,058)	113,626 (99,557–130,331)
Malaysia	Prevalence Number: Cumulative change (%)	18.3 (17.3–19.2)	-21.3 (-35.0 to -8.5)	26.3 (19.7–32.3)	14.9 (2.7–29.5)	-14.5 (-19.9 to -8.9)	-18.1 (-22.7 to -13.8)	22.1 (-1.1 to 49.3)	-25.7 (-53.4 to 14.3)	29.5 (-0.9 to 66.8)	-40.5 (-54.1 to -22.0)	2.9 (-11.5 to 18.2)
Malaysia	Prevalence Rate: 1990	95,624.1 (95,121.2–96,084.3)	19,801.3 (18,286.6–21,608.1)	36,929.2 (35,533.3–38,379.1)	6,728.1 (5,904.9–7,652.4)	18,748.9 (17,813.0–19,722.0)	696.8 (637.6–764.2)	1,513.8 (1,282.1–1,784.4)	1,517.0 (1,056.1–2,024.0)	353.7 (276.2–439.1)	5,536.6 (4,108.9–6,998.2)	1,267.9 (1,114.5–1,441.6)
Malaysia	Prevalence Rate: 2015	94,520.6 (93,829.6–95,147.2)	13,360.0 (11,774.7–14,909.8)	37,520.4 (36,173.6–38,922.9)	6,354.7 (5,245.6–7,551.4)	13,421.3 (12,784.3–14,095.3)	530.7 (483.2–584.6)	1,541.3 (1,321.2–1,798.3)	1,066.9 (754.3–1,446.4)	390.8 (308.0–490.7)	2,828.9 (2,096.4–3,878.2)	1,139.9 (1,000.6–1,304.3)
Malaysia	Prevalence Rate: Cumulative change (%)	-1.2 (-2.0 to -0.4)	-32.2 (-44.0 to -20.8)	1.6 (-3.5 to 6.3)	-5.6 (-16.0 to 5.9)	-28.4 (-32.8 to -23.7)	-23.8 (-28.1 to -19.7)	2.7 (-16.8 to 25.4)	-26.9 (-54.1 to 12.4)	11.9 (-14.7 to 44.2)	-48.6 (-60.6 to -32.7)	-9.7 (-22.4 to 3.6)
Malaysia	YLDs Number: 1990	511,444 (389,105–651,541)	61,233 (42,800–83,690)	69,644 (46,937–97,684)	25,657 (17,357–34,483)	7,405 (5,274–10,092)	10,155 (7,270–13,477)	9,176 (5,665–14,253)	17,288 (10,313–25,740)	11,827 (7,815–16,124)	14,912 (9,850–20,976)	15,040 (11,763–18,809)
Malaysia	YLDs Number: 2015	565,424 (427,306–718,477)	46,687 (32,567–64,521)	85,866 (58,345–120,191)	29,566 (19,701–40,716)	9,123 (6,465–12,399)	8,345 (5,920–11,120)	14,056 (7,973–24,042)	12,448 (7,237–19,100)	10,021 (6,618–13,896)	9,075 (5,918–13,309)	13,242 (10,327–16,821)
Malaysia	YLDs Number: Cumulative change (%)	10.6 (6.2–15.1)	-23.3 (-36.4 to -8.9)	23.2 (19.4–27.2)	15.3 (2.8–29.5)	23.6 (8.0–38.7)	-17.8 (-23.9 to -11.8)	56.9 (-4.5 to 145.9)	-25.5 (-53.8 to 15.0)	-14.0 (-36.9 to 14.0)	-38.8 (-52.0 to -24.0)	-11.9 (-19.3 to -3.9)
Malaysia	YLDs Rate: 1990	6,063.4 (4,614.7–7,718.4)	710.4 (498.8–970.0)	823.0 (553.7–1,154.9)	304.5 (206.7–409.6)	86.5 (61.4–117.8)	114.0 (81.6–151.0)	107.2 (66.2–166.5)	189.5 (113.1–282.2)	136.7 (90.0–186.6)	170.0 (112.3–238.3)	175.8 (137.5–220.0)
Malaysia	YLDs Rate: 2015	5,439.1 (4,101.7–6,918.9)	473.5 (330.7–656.2)	833.1 (566.9–1,165.8)	288.7 (191.9–397.8)	90.5 (64.2–123.4)	87.2 (61.7–116.2)	138.5 (78.6–236.9)	134.3 (78.1–206.0)	100.2 (65.9–139.0)	91.0 (58.8–133.7)	130.3 (101.6–166.0)
Malaysia	YLDs Rate: Cumulative change (%)	-10.3 (-13.7 to -6.7)	-32.9 (-44.5 to -20.2)	1.2 (-1.5 to 3.9)	-5.2 (-15.7 to 6.5)	5.1 (-8.4 to 18.7)	-23.4 (-29.0 to -17.8)	32.3 (-19.2 to 108.0)	-26.7 (-54.5 to 13.1)	-25.6 (-45.1 to -1.8)	-46.3 (-58.3 to -33.2)	-25.8 (-32.0 to -19.1)
Maldives	Prevalence Number: 1990	120,355 (119,396–121,160)	52,468 (49,878–55,516)	45,675 (43,756–47,630)	11,650 (9,613–13,770)	44,086 (42,277–46,087)	1,391 (1,264–1,571)	1,703 (1,433–2,106)	2,528 (1,889–3,219)	547 (428–676)	0 (0–0)	1,569 (1,365–1,828)
Maldives	Prevalence Number: 2015	120,603 (119,492–121,717)	38,777 (35,610–41,097)	49,695 (47,751–51,722)	10,192 (8,432–12,112)	39,974 (38,178–41,953)	799 (725–870)	1,890 (1,607–2,242)	1,683 (1,228–2,221)	448 (348–555)	0 (0–0)	1,314 (1,141–1,541)
Maldives	Prevalence Number: Cumulative change (%)	0.2 (-0.9 to 1.4)	-26.0 (-33.7 to -19.8)	8.9 (2.9–15.3)	-12.4 (-20.9 to -4.5)	-9.3 (-14.5 to -3.8)	-42.4 (-47.7 to -38.6)	12.2 (-12.7 to 37.8)	-31.6 (-55.1 to 0.4)	-17.0 (-37.7 to 7.6)	-5.3 (-16.9 to 7.9)	-15.9 (-28.3 to -2.8)
Maldives	Prevalence Rate: 1990	93,284.6 (92,592.0–93,871.5)	39,139.3 (37,473.4–41,086.5)	37,177.6 (35,729.3–38,723.3)	9,088.9 (7,575.5–10,663.4)	34,037.3 (32,646.3–35,566.1)	930.2 (851.4–1,040.1)	1,307.0 (1,098.6–1,617.1)	1,544.0 (1,153.8–1,965.6)	410.2 (323.8–505.2)	0.0 (0.0–0.0)	1,111.7 (958.9–1,301.1)
Maldives	Prevalence Rate: 2015	91,557.8 (90,720.0–92,404.4)	29,066.8 (26,739.4–30,736.0)	37,865.2 (36,379.8–39,436.3)	7,851.2 (6,495.4–9,347.8)	30,334.3 (28,971.7–31,840.8)	587.6 (534.9–639.6)	1,433.9 (1,220.5–1,700.1)	1,205.6 (879.4–1,591.0)	339.5 (264.0–421.6)	0.0 (0.0–0.0)	976.4 (846.8–1,145.8)
Maldives	Prevalence Rate: Cumulative change (%)	-1.8 (-2.9 to -0.7)	-25.7 (-33.4 to -20.0)	1.9 (-3.5 to 7.6)	-13.6 (-21.3 to -6.3)	-10.8 (-16.0 to -5.5)	-36.7 (-42.3 to -32.7)	10.9 (-13.6 to 36.3)	-19.8 (-47.3 to 17.7)	-16.1 (-36.7 to 8.1)	-1.8 (-13.8 to 11.3)	-11.7 (-25.7 to 2.5)
Maldives	YLDs Number: 1990	10,026 (7,549–12,741)	2,551 (1,827–3,407)	1,070 (728–1,516)	525 (349–725)	630 (453–841)	223 (160–295)	158 (95–251)	310 (193–455)	207 (142–285)	0 (0–0)	207 (163–258)
Maldives	YLDs Number: 2015	8,334 (6,280–10,610)	1,631 (1,150–2,225)	1,130 (767–1,602)	462 (305–640)	587 (418–794)	129 (92–171)	226 (129–364)	207 (128–303)	115 (78–160)	0 (0–0)	139 (109–176)
Maldives	YLDs Number: Cumulative change (%)	-16.8 (-22.1 to -11.9)	-35.8 (-46.1 to -26.0)	5.6 (2.2–9.1)	-12.0 (-20.7 to -4.0)	-6.6 (-18.8 to 3.5)	-41.9 (-47.8 to -37.1)	47.9 (-11.2 to 138.0)	-31.1 (-54.9 to 1.4)	-43.7 (-59.7 to -23.8)	-16.2 (-28.8 to -2.4)	-32.5 (-39.2 to -25.5)
Maldives	YLDs Rate: 1990	7,782.1 (5,884.1–9,852.5)	1,847.3 (1,330.3–2,464.8)	847.5 (571.5–1,196.5)	410.0 (275.6–563.5)	467.9 (339.1–620.8)	149.5 (107.5–197.8)	121.5 (73.2–192.5)	189.5 (118.4–278.1)	155.6 (106.9–214.0)	0.0 (0.0–0.0)	157.9 (124.7–196.8)
Maldives	YLDs Rate: 2015	6,320.3 (4,761.7–8,043.4)	1,227.0 (866.6–1,671.8)	860.7 (584.2–1,219.6)	356.0 (235.3–492.9)	449.2 (321.0–605.1)	95.4 (68.1–126.0)	171.7 (98.3–276.3)	148.7 (91.8–217.5)	87.2 (59.1–122.1)	0.0 (0.0–0.0)	106.3 (82.8–134.7)
Maldives	YLDs Rate: Cumulative change (%)	-18.7 (-23.5 to -14.6)	-33.4 (-43.4 to -24.2)	1.5 (-1.1 to 4.1)	-13.1 (-21.1 to -5.6)	-3.8 (-15.3 to 5.8)	-36.1 (-42.3 to -30.9)	46.6 (-11.9 to 136.2)	-19.2 (-47.1 to 18.9)	-43.1 (-59.2 to -23.2)	-11.2 (-23.6 to 2.7)	-32.6 (-39.3 to -25.7)
Mauritius	Prevalence Number: 1990	378,015 (373,228–382,073)	58,199 (51,745–64,140)	163,339 (156,236–170,820)	35,790 (29,695–41,866)	100,516 (94,102–109,214)	3,297 (3,037–3,593)	6,430 (5,376–7,870)	6,631 (5,016–8,505)	1,781 (1,400–2,203)	0 (0–0)	5,346 (4,711–6,147)
Mauritius	Prevalence Number: 2015	302,873 (298,777–306,306)	28,248 (23,674–33,380)	138,048 (132,434–144,106)	28,242 (23,200–33,401)	69,326 (64,096–76,808)	1,642 (1,504–1,788)	5,341 (4,526–6,419)	2,898 (2,072–3,945)	1,570 (1,248–1,921)	0 (0–0)	3,765 (3,284–4,283)
Mauritius	Prevalence Number: Cumulative change (%)	-19.9 (-21.2 to -18.7)	-51.4 (-58.5 to -42.6)	-15.4 (-20.1 to -10.5)	-21.1 (-26.8 to -15.7)	-30.9 (-37.7 to -23.7)	-50.2 (-53.6 to -47.2)	-16.1 (-34.7 to 4.2)	-55.1 (-71.5 to -32.8)	-10.8 (-31.8 to 14.5)	-22.1 (-33.2 to -9.3)	-29.3 (-38.6 to -19.9)
Mauritius	Prevalence Rate: 1990	88,861.1 (87,732.6–89,823.3)	14,203.8 (12,631.7–15,640.4)	37,875.8 (36,242.5–39,595.4)	8,422.1 (6,983.4–9,872.0)	23,683.3 (22,161.8–25,739.8)	807.5 (742.1–882.3)	1,515.6 (1,268.1–1,854.5)	1,661.4 (1,256.8–2,131.1)	421.1 (330.7–519.5)	0.0 (0.0–0.0)	1,272.1 (1,121.5–1,461.2)
Mauritius	Prevalence Rate: 2015	87,096.2 (85,839.5–88,140.1)	8,987.8 (7,467.8–10,676.7)	38,410.0 (36,839.3–40,078.8)	8,026.9 (6,599.9–9,485.8)	20,128.8 (18,600.4–22,313.3)	551.7 (502.6–604.4)	1,552.2 (1,317.1–1,863.8)	1,074.5 (768.2–1,462.7)	460.9 (366.2–564.3)	0.0 (0.0–0.0)	1,161.2 (1,020.0–1,313.9)
Mauritius	Prevalence Rate: Cumulative change (%)	-2.0 (-3.6 to -0.4)	-36.6 (-46.7 to -25.0)	1.5 (-4.2 to 7.5)	-4.7 (-11.2 to 1.9)	-14.9 (-23.2 to -5.9)	-31.6 (-36.7 to -27.4)	3.5 (-19.4 to 28.5)	-33.6 (-57.9 to -0.6)	10.8 (-15.3 to 42.4)	-2.1 (-15.9 to 14.3)	-8.4 (-20.1 to 3.1)
Mauritius	YLDs Number: 1990	22,810 (17,468–28,757)	1,852 (1,225–2,585)	3,694 (2,502–5,242)	1,631 (1,084–2,250)	1,054 (715–1,474)	539 (386–719)	470 (315–657)	827 (528–1,214)	689 (462–954)	0 (0–0)	864 (679–1,070)
Mauritius	YLDs Number: 2015	17,571 (13,444–22,215)	850 (543–1,225)	3,077 (2,076–4,388)	1,289 (846–1,784)	787 (508–1,124)	269 (192–360)	593 (347–893)	363 (221–539)	406 (271–569)	0 (0–0)	554 (435–699)
Mauritius	YLDs Number: Cumulative change (%)	-22.9 (-25.0 to -20.9)	-54.0 (-64.1 to -45.1)	-16.7 (-18.8 to -14.5)	-21.0 (-26.9 to -15.2)	-25.5 (-32.3 to -17.0)	-50.0 (-54.0 to -46.1)	26.3 (-13.7 to 58.5)	-54.8 (-71.7 to -32.5)	-40.2 (-56.2 to -21.1)	-25.8 (-39.3 to -10.2)	-35.8 (-40.8 to -30.9)
Mauritius	YLDs Rate: 1990	5,317.1 (4,068.8–6,709.5)	453.9 (299.6–634.0)	864.4 (584.4–1,225.7)	384.0 (254.7–527.1)	251.1 (170.3–350.2)	132.2 (94.6–176.8)	111.1 (74.5–155.0)	207.3 (132.3–304.2)	163.0 (109.7–225.2)	0.0 (0.0–0.0)	203.9 (160.4–252.4)
Mauritius	YLDs Rate: 2015	4,910.6 (3,744.3–6,204.7)	268.5 (172.5–389.2)	873.7 (591.0–1,241.1)	366.6 (241.5–508.5)	228.0 (147.8–323.7)	90.8 (64.2–120.8)	173.0 (101.6–260.7)	134.9 (82.2–200.3)	119.4 (80.0–166.8)	0.0 (0.0–0.0)	161.0 (126.3–203.0)
Mauritius	YLDs Rate: Cumulative change (%)	-7.6 (-10.3 to -4.9)	-40.7 (-54.3 to -27.2)	1.1 (-1.5 to 3.7)	-4.5 (-11.6 to 2.2)	-9.5 (-17.9 to 1.9)	-31.3 (-37.1 to -25.9)	56.0 (6.8–96.4)	-33.2 (-58.1 to -0.1)	-25.7 (-45.6 to -2.2)	-7.7 (-24.7 to 11.1)	-21.0 (-27.2 to -15.1)
Myanmar	Prevalence Number: 1990	19,933,226 (19,857,105–20,004,604)	7,046,996 (6,602,500–7,655,076)	7,445,731 (7,138,175–7,761,246)	1,605,701 (1,324,075–1,871,875)	6,304,401 (6,018,555–6,577,826)	297,788 (274,795–327,471)	245,105 (196,122–310,001)	273,131 (218,990–337,479)	61,541 (47,372–76,851)	4,956,002 (3,300,142–6,545,853)	298,851 (250,004–367,246)
Myanmar	Prevalence Number: 2015	18,517,779 (18,416,440–18,631,753)	5,082,991 (4,693,990–5,402,302)	7,603,644 (7,253,142–7,942,306)	1,636,157 (1,359,409–1,921,285)	5,600,377 (5,370,919–5,847,180)	144,060 (136,527–152,405)	290,678 (241,049–355,117)	142,776 (102,368–193,294)	70,484 (54,434–88,706)	3,219,637 (2,460,714–4,318,169)	269,196 (230,988–325,488)
Myanmar	Prevalence Number: Cumulative change (%)	-7.1 (-7.7 to -6.5)	-27.7 (-34.9 to -20.9)	2.2 (-3.3 to 8.0)	2.1 (-5.5 to 10.7)	-11.1 (-15.9 to -6.1)	-51.5 (-55.2 to -48.1)	20.7 (-10.9 to 55.6)	-46.8 (-65.0 to -26.2)	16.0 (-12.2 to 48.3)	-33.8 (-49.3 to -19.2)	-8.9 (-28.3 to 10.4)
Myanmar	Prevalence Rate: 1990	97,797.9 (97,424.4–98,148.0)	34,585.5 (32,408.4–37,573.3)	36,744.8 (35,242.6–38,287.6)	7,799.4 (6,436.1–9,067.7)	30,938.7 (29,540.1–32,280.6)	1,465.9 (1,352.9–1,612.9)	1,202.5 (961.8–1,522.3)	1,352.1 (1,084.1–1,670.7)	302.2 (232.6–377.3)	24,099.0 (16,140.4–31,689.2)	1,472.6 (1,231.6–1,808.8)
Myanmar	Prevalence Rate: 2015	92,573.7 (92,051.4–93,160.3)	25,868.3 (23,768.9–27,592.0)	37,436.6 (35,730.0–39,126.5)	8,080.0 (6,719.6–9,501.2)	28,054.3 (26,909.2–29,297.5)	763.2 (722.5–808.3)	1,458.9 (1,208.7–1,782.7)	802.3 (575.2–1,086.2)	353.0 (272.7–443.2)	16,012.9 (12,125.3–21,729.4)	1,423.2 (1,222.6–1,710.9)
Myanmar	Prevalence Rate: Cumulative change (%)	-5.3 (-6.0 to -4.7)	-25.1 (-33.0 to -17.6)	1.9 (-3.5 to 7.9)	3.7 (-4.1 to 12.5)	-9.3 (-14.1 to -4.1)	-47.8 (-51.8 to -44.2)	23.4 (-8.8 to 59.1)	-39.7 (-60.3 to -16.2)	18.4 (-10.4 to 51.6)	-32.4 (-48.3 to -18.1)	-2.2 (-22.8 to 17.6)
Myanmar	YLDs Number: 1990	1,510,452 (1,142,315–1,921,734)	286,368 (202,730–386,650)	172,726 (117,796–243,142)	72,194 (47,937–98,937)	34,511 (24,730–46,180)	48,117 (35,024–63,471)	21,811 (13,318–33,664)	33,675 (22,216–47,365)	24,117 (16,356–33,517)	49,044 (33,260–70,992)	35,720 (27,277–44,654)
Myanmar	YLDs Number: 2015	1,208,977 (925,216–1,544,241)	191,982 (137,128–260,216)	172,820 (117,150–245,522)	74,149 (48,803–101,282)	32,586 (23,399–44,016)	23,479 (17,062–31,003)	26,460 (16,667–39,580)	17,757 (11,205–26,435)	20,692 (13,648–28,265)	30,683 (21,670–41,695)	32,185 (25,004–39,889)
Myanmar	YLDs Number: Cumulative change (%)	-19.9 (-23.5 to -16.3)	-32.7 (-40.8 to -24.0)	0.0 (-2.8 to 2.8)	2.9 (-5.2 to 11.9)	-5.4 (-14.8 to 4.8)	-51.1 (-55.3 to -47.3)	22.9 (4.0–54.9)	-46.4 (-64.8 to -25.0)	-12.9 (-35.7 to 12.4)	-36.9 (-45.1 to -29.5)	-9.6 (-18.8 to 0.6)
Myanmar	YLDs Rate: 1990	7,420.8 (5,615.1–9,429.5)	1,395.5 (988.0–1,886.0)	849.1 (580.1–1,194.3)	350.7 (232.7–480.5)	168.1 (120.2–225.4)	236.9 (172.4–312.3)	107.0 (65.4–165.2)	166.7 (110.0–234.5)	118.4 (80.5–164.2)	237.7 (161.4–344.6)	175.0 (133.5–218.9)
Myanmar	YLDs Rate: 2015	5,989.9 (4,580.1–7,649.6)	973.6 (691.9–1,323.6)	857.7 (581.1–1,217.7)	366.2 (240.6–503.7)	163.4 (117.0–221.8)	124.4 (90.7–164.6)	132.9 (83.9–198.7)	99.8 (63.0–148.6)	103.7 (68.5–140.7)	152.9 (107.7–208.7)	162.2 (126.4–201.3)
Myanmar	YLDs Rate: Cumulative change (%)	-19.2 (-22.9 to -15.6)	-30.0 (-39.2 to -20.4)	1.0 (-1.7 to 3.6)	4.6 (-3.7 to 13.6)	-2.6 (-12.8 to 8.5)	-47.4 (-51.9 to -43.2)	25.8 (6.5–58.7)	-39.1 (-60.1 to -14.9)	-11.2 (-34.4 to 14.8)	-35.1 (-43.6 to -27.2)	-7.0 (-16.4 to 3.6)
Philippines	Prevalence Number: 1990	31,231,348 (31,159,877–31,306,170)	9,470,186 (8,922,128–10,089,883)	11,723,529 (11,210,709–12,217,380)	3,467,124 (2,881,118–4,025,751)	6,009,504 (5,706,043–6,309,181)	426,887 (397,525–460,373)	411,769 (344,114–502,297)	284,403 (252,811–317,558)	100,668 (79,732–123,424)	1,969,252 (1,307,640–2,622,127)	408,155 (354,417–476,524)
Philippines	Prevalence Number: 2015	40,723,308 (40,510,471–40,912,338)	10,773,951 (10,077,807–11,416,488)	16,016,501 (15,379,874–16,662,136)	4,634,936 (3,898,594–5,408,202)	6,632,186 (6,322,499–6,989,363)	378,469 (355,679–403,515)	606,644 (510,935–723,451)	295,909 (209,856–396,029)	139,408 (108,953–175,041)	1,383,901 (1,089,628–1,772,576)	566,016 (489,153–664,472)
Philippines	Prevalence Number: Cumulative change (%)	30.4 (29.7–31.0)	13.9 (3.7–24.5)	36.7 (29.7–44.2)	34.1 (20.1–57.8)	10.4 (3.4–17.8)	-11.3 (-14.8 to -7.8)	49.0 (15.1–86.7)	4.6 (-26.8 to 41.6)	40.1 (7.9–83.0)	-27.9 (-44.7 to -6.4)	39.4 (18.6–63.5)

© 2017 Global Burden of Disease Child and Adolescent Health Collaboration. JAMA Pediatrics.

eTable 3. Prevalent Cases, Rates (per 100,000 population), Years Lived with Disability (YLDs), and Cumulative Percent Change with 95% Uncertainty Interval (UI) for the Top 10 Global Causes of YLDs in Children and Adolescents in 195 Countries and Territories, Aged 0 to 19 Years, Both Sexes, 1990 and 2015. *Note: best viewed by enlarging in browser.*

Location	Measure	All causes	Iron-deficiency anemia	Skin and subcutaneous diseases	Asthma	Hemoglobinopathies and hemolytic anemias	Diarrheal diseases	Congenital anomalies	Protein-energy malnutrition	Epilepsy	Malaria	Neonatal preterm birth complications
Philippines	Prevalence Rate: 1990	97,997.5 (97,791.6–98,212.8)	29,192.0 (27,603.3–30,962.3)	37,587.3 (35,926.4–39,104.3)	10,963.0 (9,194.3–12,721.0)	18,834.7 (17,885.1–19,772.6)	1,253.0 (1,170.0–1,347.9)	1,285.8 (1,073.1–1,569.3)	797.3 (708.9–890.1)	313.1 (248.0–383.6)	6,056.5 (4,105.6–7,970.2)	1,214.9 (1,053.8–1,424.5)
Philippines	Prevalence Rate: 2015	96,072.3 (95,577.1–96,516.6)	25,403.4 (23,783.6–26,883.3)	37,796.3 (36,301.2–39,319.7)	10,980.5 (9,235.5–12,813.1)	15,642.7 (14,911.8–16,485.8)	886.4 (833.2–944.5)	1,430.5 (1,204.5–1,706.0)	686.5 (486.9–918.8)	328.9 (257.1–413.3)	3,271.7 (2,577.2–4,187.9)	1,326.9 (1,146.2–1,558.3)
Philippines	Prevalence Rate: Cumulative change (%)	-2.0 (-2.5 to -1.5)	-12.9 (-20.3 to -5.1)	0.6 (-4.6 to 6.1)	0.4 (-9.6 to 17.0)	-16.9 (-22.2 to -11.4)	-29.2 (-31.9 to -26.5)	12.5 (-13.1 to 40.9)	-13.4 (-39.4 to 17.2)	6.2 (-18.4 to 38.3)	-44.8 (-56.9 to -29.6)	9.9 (-7.3 to 29.2)
Philippines	YLDs Number: 1990	2,175,889 (1,646,818–2,776,232)	366,397 (256,740–498,966)	271,453 (185,507–380,521)	156,536 (103,521–212,013)	25,100 (17,816–34,247)	69,310 (50,061–92,756)	26,027 (17,314–37,727)	35,271 (23,678–48,011)	38,855 (26,049–52,829)	50,832 (35,066–69,486)	53,261 (41,762–66,225)
Philippines	YLDs Number: 2015	2,549,203 (1,934,422–3,251,331)	396,442 (278,916–542,923)	364,801 (247,609–509,499)	210,214 (139,711–286,948)	29,824 (21,076–39,840)	61,740 (44,354–82,055)	44,893 (29,597–64,673)	36,909 (23,262–54,757)	40,103 (27,112–55,632)	37,520 (26,711–50,200)	67,167 (52,681–83,581)
Philippines	YLDs Number: Cumulative change (%)	17.2 (12.9–21.5)	8.5 (-5.0 to 21.9)	34.3 (30.9–37.9)	34.7 (20.0–59.0)	19.0 (8.2–31.5)	-10.9 (-15.7 to -6.0)	73.3 (49.1–100.3)	5.3 (-26.1 to 42.2)	4.8 (-22.7 to 37.7)	-25.7 (-33.2 to -12.1)	26.3 (16.5–36.6)
Philippines	YLDs Rate: 1990	6,914.7 (5,232.0–8,805.2)	1,122.8 (788.0–1,525.1)	860.3 (587.0–1,205.7)	494.8 (328.1–665.1)	77.8 (55.2–105.8)	203.4 (147.0–272.0)	81.2 (54.1–117.6)	98.9 (66.4–134.6)	120.8 (81.2–163.8)	156.2 (107.9–213.4)	165.1 (129.7–205.0)
Philippines	YLDs Rate: 2015	6,019.6 (4,566.8–7,680.0)	937.7 (659.3–1,283.3)	860.8 (584.0–1,202.8)	498.0 (330.9–679.9)	70.7 (50.0–94.5)	144.6 (103.9–192.0)	105.9 (69.8–152.5)	85.6 (54.0–127.0)	94.6 (63.9–131.2)	89.2 (63.5–119.2)	158.4 (124.2–197.1)
Philippines	YLDs Rate: Cumulative change (%)	-12.9 (-16.0 to -9.8)	-16.3 (-26.1 to -6.3)	0.0 (-2.5 to 2.7)	0.9 (-9.8 to 17.6)	-9.0 (-16.8 to 0.1)	-28.8 (-32.7 to -25.0)	30.9 (12.5–51.7)	-12.8 (-38.8 to 17.5)	-20.5 (-41.4 to 4.3)	-42.6 (-48.2 to -32.5)	-3.9 (-11.3 to 4.0)
Sri Lanka	Prevalence Number: 1990	6,833,089 (6,788,258–6,874,449)	2,400,840 (2,246,143–2,559,101)	2,755,732 (2,665,832–2,848,440)	705,970 (586,212–831,069)	1,717,210 (1,627,260–1,808,911)	73,127 (67,501–81,094)	108,161 (90,832–130,295)	113,996 (96,252–132,677)	24,023 (18,799–29,337)	10,939 (7,506–14,668)	111,453 (98,307–128,734)
Sri Lanka	Prevalence Number: 2015	6,035,154 (5,978,556–6,085,450)	1,765,593 (1,631,297–1,955,452)	2,575,693 (2,491,068–2,658,868)	574,918 (477,890–685,666)	1,291,737 (1,226,765–1,361,731)	31,513 (29,594–33,462)	109,032 (92,385–131,196)	78,378 (57,902–101,297)	23,756 (18,523–29,572)	0 (0–0)	88,124 (77,671–101,406)
Sri Lanka	Prevalence Number: Cumulative change (%)	-11.7 (-12.6 to -10.8)	-26.3 (-34.6 to -17.7)	-6.5 (-10.5 to -2.3)	-18.5 (-26.0 to -11.0)	-24.7 (-29.6 to -19.5)	-56.8 (-61.0 to -53.6)	1.8 (-19.4 to 27.7)	-30.6 (-50.3 to -6.4)	0.1 (-24.1 to 28.2)	-100.0 (-100.0 to -100.0)	-20.6 (-32.2 to -8.0)
Sri Lanka	Prevalence Rate: 1990	93,802.0 (93,163.0–94,386.4)	33,080.7 (30,868.2–35,362.6)	37,710.8 (36,478.6–38,977.1)	9,549.7 (7,939.0–11,248.3)	23,605.0 (22,368.7–24,866.2)	1,043.8 (962.8–1,159.1)	1,488.6 (1,250.3–1,792.9)	1,682.6 (1,420.7–1,958.2)	329.8 (258.6–402.6)	152.1 (103.6–203.8)	1,570.9 (1,386.9–1,810.8)
Sri Lanka	Prevalence Rate: 2015	89,787.9 (88,935.8–90,542.6)	26,383.5 (24,320.7–29,316.5)	38,209.6 (36,948.6–39,435.7)	8,461.8 (7,035.6–10,093.4)	19,251.1 (18,283.9–20,297.7)	478.3 (449.7–508.1)	1,626.0 (1,378.4–1,956.0)	1,234.1 (911.7–1,594.7)	353.8 (275.9–440.1)	0.0 (0.0–0.0)	1,352.3 (1,195.7–1,550.0)
Sri Lanka	Prevalence Rate: Cumulative change (%)	-4.3 (-5.3 to -3.3)	-20.1 (-29.3 to -10.3)	1.4 (-2.9 to 5.9)	-11.3 (-19.5 to -3.1)	-18.4 (-23.7 to -12.8)	-54.1 (-58.7 to -50.6)	10.3 (-12.6 to 38.5)	-25.9 (-47.0 to -0.1)	8.6 (-17.6 to 38.9)	-100.0 (-100.0 to -100.0)	-13.5 (-25.8 to -0.3)
Sri Lanka	YLDs Number: 1990	495,117 (376,963–628,952)	93,082 (66,391–126,046)	63,327 (43,392–89,185)	31,923 (21,012–43,744)	7,632 (5,439–10,354)	11,887 (8,578–15,868)	8,124 (5,336–11,726)	14,193 (9,463–19,577)	9,153 (6,170–12,758)	227 (127–352)	17,530 (13,852–21,979)
Sri Lanka	YLDs Number: 2015	381,014 (287,827–483,101)	66,032 (45,515–91,772)	58,753 (40,163–82,258)	26,106 (17,078–36,079)	6,673 (4,620–9,117)	5,143 (3,650–6,789)	8,297 (5,483–11,787)	9,809 (6,027–14,480)	6,162 (4,080–8,527)	0 (0–0)	10,274 (8,090–12,808)
Sri Lanka	YLDs Number: Cumulative change (%)	-23.0 (-25.9 to -20.3)	-28.9 (-39.3 to -18.7)	-7.2 (-9.2 to -5.3)	-18.1 (-25.5 to -10.1)	-12.4 (-24.5 to -1.7)	-56.6 (-61.2 to -52.8)	2.4 (-9.0 to 14.9)	-30.2 (-50.0 to -5.6)	-31.7 (-50.0 to -10.2)	-100.0 (-100.0 to -100.0)	-41.3 (-46.7 to -35.9)
Sri Lanka	YLDs Rate: 1990	6,779.6 (5,167.5–8,612.3)	1,271.3 (903.3–1,727.3)	867.3 (593.9–1,221.4)	431.9 (284.1–592.4)	104.0 (73.8–141.0)	169.7 (122.4–226.9)	111.9 (73.4–161.3)	209.5 (139.7–289.0)	125.7 (84.8–175.5)	3.2 (1.8–4.9)	241.1 (190.9–302.2)
Sri Lanka	YLDs Rate: 2015	5,653.2 (4,268.7–7,164.9)	980.4 (672.6–1,367.0)	872.2 (596.3–1,222.6)	384.3 (251.6–530.5)	98.7 (68.3–135.0)	78.1 (55.4–103.1)	123.8 (81.8–175.8)	154.5 (94.9–228.0)	91.8 (60.7–127.0)	0.0 (0.0–0.0)	153.1 (120.6–190.8)
Sri Lanka	YLDs Rate: Cumulative change (%)	-16.6 (-19.9 to -13.6)	-22.7 (-34.4 to -10.8)	0.6 (-1.6 to 2.7)	-10.9 (-19.1 to -2.2)	-4.9 (-18.4 to 7.3)	-53.9 (-58.8 to -49.8)	11.0 (-1.4 to 24.6)	-25.6 (-46.7 to 0.8)	-25.9 (-45.8 to -2.7)	-100.0 (-100.0 to -100.0)	-36.4 (-42.3 to -30.5)
Seychelles	Prevalence Number: 1990	31,252 (31,024–31,505)	5,232 (4,721–5,742)	12,560 (12,012–13,097)	2,424 (2,014–2,841)	8,241 (7,591–9,132)	185 (175–198)	521 (438–622)	115 (80–157)	114 (89–142)	0 (0–0)	393 (345–451)
Seychelles	Prevalence Number: 2015	27,571 (27,314–27,819)	4,658 (3,965–5,427)	11,104 (10,684–11,495)	2,091 (1,722–2,461)	6,351 (5,773–7,094)	144 (137–151)	474 (402–565)	86 (57–123)	111 (86–139)	0 (0–0)	345 (303–393)
Seychelles	Prevalence Number: Cumulative change (%)	-11.8 (-12.8 to -10.8)	-10.8 (-24.5 to 6.9)	-11.5 (-16.1 to -6.8)	-13.7 (-21.7 to -4.8)	-22.7 (-32.4 to -12.9)	-22.3 (-25.8 to -19.1)	-8.1 (-27.5 to 14.4)	-22.6 (-55.7 to 19.6)	-1.8 (-24.7 to 25.4)	-11.6 (-22.6 to 1.2)	-11.8 (-23.8 to 0.4)
Seychelles	Prevalence Rate: 1990	94,696.7 (93,988.8–95,475.5)	15,923.0 (14,324.1–17,482.1)	38,089.3 (36,440.0–39,730.4)	7,281.9 (6,052.5–8,534.3)	24,980.4 (23,017.7–27,678.1)	571.3 (538.3–610.9)	1,581.3 (1,330.9–1,887.0)	362.1 (252.0–493.6)	348.2 (270.5–432.3)	0.0 (0.0–0.0)	1,208.3 (1,061.1–1,385.2)
Seychelles	Prevalence Rate: 2015	94,367.8 (93,526.0–95,167.6)	15,735.7 (13,538.7–18,171.8)	38,545.9 (37,055.0–39,915.7)	7,215.3 (5,954.8–8,502.2)	21,713.3 (19,739.7–24,245.4)	471.3 (450.1–493.5)	1,621.4 (1,374.6–1,931.3)	269.8 (180.7–386.3)	379.7 (294.4–474.0)	0.0 (0.0–0.0)	1,166.3 (1,022.3–1,331.2)
Seychelles	Prevalence Rate: Cumulative change (%)	-0.3 (-1.4 to 0.8)	-1.0 (-14.8 to 17.9)	1.3 (-4.0 to 6.8)	-0.8 (-10.1 to 9.3)	-12.9 (-23.8 to -1.8)	-17.4 (-21.2 to -14.0)	3.5 (-18.3 to 28.8)	-22.5 (-55.6 to 19.8)	10.4 (-15.4 to 41.3)	-1.8 (-13.6 to 11.3)	-3.1 (-16.3 to 10.5)
Seychelles	YLDs Number: 1990	1,773 (1,355–2,260)	176 (122–244)	286 (193–406)	110 (72–150)	73 (49–104)	30 (21–40)	35 (23–50)	14 (8–21)	44 (30–61)	0 (0–0)	57 (44–71)
Seychelles	YLDs Number: 2015	1,507 (1,145–1,918)	158 (106–223)	253 (172–360)	95 (62–132)	59 (38–84)	23 (17–31)	35 (22–55)	10 (6–16)	28 (18–40)	0 (0–0)	42 (32–53)
Seychelles	YLDs Number: Cumulative change (%)	-15.0 (-18.1 to -11.3)	-10.0 (-25.3 to 15.0)	-11.6 (-13.9 to -9.3)	-13.6 (-21.9 to -4.6)	-18.5 (-26.4 to -7.2)	-22.2 (-27.3 to -16.4)	2.7 (-20.2 to 40.0)	-22.4 (-55.3 to 20.2)	-35.3 (-52.6 to -15.4)	-13.5 (-27.0 to 2.5)	-26.1 (-31.7 to -19.8)
Seychelles	YLDs Rate: 1990	5,373.7 (4,110.6–6,842.8)	532.7 (370.3–738.5)	868.6 (586.9–1,229.5)	331.6 (217.3–454.1)	219.3 (147.9–312.5)	93.7 (67.5–125.0)	106.3 (70.4–153.4)	45.4 (27.1–68.4)	136.0 (91.2–187.7)	0.0 (0.0–0.0)	172.9 (135.4–215.9)
Seychelles	YLDs Rate: 2015	5,226.2 (3,974.9–6,630.7)	539.3 (363.1–755.3)	872.8 (592.7–1,237.5)	328.5 (217.1–454.9)	206.4 (135.1–290.7)	77.4 (55.8–102.7)	122.1 (75.7–189.5)	33.8 (19.9–52.7)	97.6 (63.9–138.5)	0.0 (0.0–0.0)	144.1 (112.3–181.9)
Seychelles	YLDs Rate: Cumulative change (%)	-2.7 (-6.1 to 1.3)	1.4 (-14.6 to 28.8)	0.5 (-2.2 to 3.0)	-0.8 (-10.2 to 9.5)	-5.7 (-14.4 to 6.7)	-17.3 (-22.9 to -11.3)	15.6 (-10.1 to 57.7)	-22.3 (-55.2 to 20.4)	-27.3 (-46.5 to -4.9)	-2.4 (-17.3 to 14.1)	-16.6 (-23.0 to -9.5)
Thailand	Prevalence Number: 1990	21,845,012 (21,688,897–21,977,407)	5,885,226 (5,648,541–6,120,357)	8,967,302 (8,589,332–9,330,332)	2,092,416 (1,811,714–2,402,977)	5,483,928 (5,268,705–5,684,501)	199,646 (185,474–214,045)	350,223 (291,414–423,681)	165,737 (128,609–209,986)	81,570 (63,625–100,620)	645,984 (363,758–1,011,770)	347,008 (306,711–398,022)
Thailand	Prevalence Number: 2015	14,989,453 (14,851,372–15,104,520)	3,487,966 (3,289,095–3,715,324)	6,408,040 (6,146,658–6,667,568)	1,280,186 (1,043,330–1,535,926)	3,606,952 (3,473,370–3,738,797)	79,587 (73,696–85,985)	267,182 (227,748–320,373)	78,848 (54,667–107,956)	60,362 (46,077–74,437)	251,060 (196,929–329,153)	215,686 (188,903–249,075)
Thailand	Prevalence Number: Cumulative change (%)	-31.4 (-32.1 to -30.7)	-40.7 (-45.2 to -36.1)	-28.5 (-32.3 to -24.8)	-38.8 (-47.3 to -32.1)	-34.2 (-37.6 to -30.4)	-60.1 (-62.6 to -58.1)	-22.8 (-40.2 to -3.2)	-51.2 (-69.3 to -27.9)	-25.1 (-44.0 to -4.2)	-58.2 (-73.9 to -41.6)	-37.6 (-46.8 to -27.4)
Thailand	Prevalence Rate: 1990	93,356.3 (92,622.5–93,948.5)	25,249.3 (24,204.4–26,285.8)	37,602.6 (35,992.5–39,138.4)	8,809.6 (7,641.3–10,136.0)	23,516.7 (22,583.0–24,379.6)	915.8 (847.8–984.1)	1,505.2 (1,254.0–1,820.2)	807.3 (626.4–1,022.8)	355.1 (276.6–437.3)	2,843.9 (1,549.1–4,538.3)	1,544.5 (1,370.2–1,765.5)
Thailand	Prevalence Rate: 2015	91,082.6 (90,227.3–91,817.3)	21,472.7 (20,112.8–23,071.2)	38,158.0 (36,596.8–39,698.9)	7,673.3 (6,242.9–9,231.2)	22,034.4 (21,213.6–22,837.7)	527.1 (485.9–571.7)	1,633.8 (1,393.5–1,958.5)	557.6 (386.6–763.4)	372.0 (284.4–459.7)	1,522.9 (1,185.2–2,009.2)	1,386.3 (1,219.2–1,587.5)
Thailand	Prevalence Rate: Cumulative change (%)	-2.4 (-3.5 to -1.4)	-14.9 (-22.0 to -7.9)	1.5 (-3.8 to 6.9)	-12.9 (-24.8 to -3.2)	-6.3 (-11.1 to -0.9)	-42.4 (-46.2 to -39.3)	9.8 (-14.9 to 37.6)	-29.2 (-55.5 to 4.7)	6.0 (-20.7 to 35.6)	-42.0 (-64.7 to -17.8)	-9.9 (-22.6 to 4.2)
Thailand	YLDs Number: 1990	1,563,666 (1,189,239–1,989,862)	222,053 (159,527–299,168)	200,348 (135,415–278,548)	94,641 (63,493–129,118)	48,566 (34,727–65,418)	32,576 (23,469–42,929)	22,650 (14,792–32,502)	20,734 (13,284–29,986)	31,328 (21,236–42,447)	18,456 (11,126–26,725)	61,689 (49,422–75,262)
Thailand	YLDs Number: 2015	923,064 (698,834–1,172,193)	128,229 (91,861–172,778)	142,105 (96,211–199,701)	58,146 (38,121–82,057)	36,634 (26,273–49,434)	13,049 (9,242–17,338)	20,522 (13,271–29,776)	9,888 (5,868–15,050)	15,587 (10,399–21,863)	7,915 (5,386–11,186)	27,601 (21,711–34,418)
Thailand	YLDs Number: Cumulative change (%)	-41.0 (-42.9 to -39.0)	-42.2 (-47.4 to -37.7)	-29.1 (-30.8 to -27.2)	-38.6 (-47.1 to -31.7)	-24.5 (-31.1 to -18.2)	-59.9 (-63.1 to -56.9)	-8.9 (-22.0 to 9.1)	-51.1 (-69.2 to -26.9)	-49.5 (-63.8 to -33.1)	-56.1 (-65.6 to -42.8)	-55.2 (-59.5 to -50.7)
Thailand	YLDs Rate: 1990	6,631.5 (5,037.9–8,453.1)	954.6 (685.3–1,290.1)	849.0 (575.7–1,180.6)	398.6 (267.3–543.2)	209.7 (149.9–282.4)	149.5 (107.8–197.3)	97.4 (63.7–139.6)	101.0 (64.7–146.0)	136.5 (93.1–185.9)	80.4 (47.7–117.0)	266.3 (213.1–325.3)
Thailand	YLDs Rate: 2015	5,516.5 (4,173.1–7,019.5)	790.1 (564.6–1,069.2)	855.8 (579.6–1,195.5)	348.6 (226.9–492.6)	226.0 (161.9–305.5)	86.5 (61.4–115.1)	125.6 (81.4–182.4)	69.9 (41.5–106.4)	96.1 (64.3–135.0)	48.1 (32.7–68.1)	169.1 (133.1–210.8)
Thailand	YLDs Rate: Cumulative change (%)	-16.8 (-19.8 to -13.8)	-17.2 (-25.1 to -10.5)	0.8 (-1.6 to 3.4)	-12.6 (-24.4 to -2.6)	7.9 (-2.1 to 17.0)	-42.1 (-46.8 to -37.7)	29.6 (11.0–55.0)	-29.0 (-55.2 to 6.1)	-28.5 (-48.7 to -5.5)	-38.6 (-52.5 to -19.2)	-36.5 (-42.4 to -30.0)
Timor-Leste	Prevalence Number: 1990	362,450 (360,301–364,565)	136,620 (127,739–145,503)	137,685 (132,431–142,766)	38,001 (31,758–44,650)	99,397 (91,731–109,006)	5,220 (4,813–5,719)	3,443 (2,694–4,361)	8,644 (6,198–11,428)	1,448 (1,133–1,780)	41,552 (30,599–61,051)	5,085 (4,190–6,146)
Timor-Leste	Prevalence Number: 2015	588,923 (584,325–593,286)	174,970 (164,330–184,002)	232,602 (223,421–241,088)	55,534 (46,098–65,727)	143,554 (130,456–159,823)	6,769 (6,282–7,322)	7,590 (6,240–9,317)	13,308 (9,774–17,521)	2,395 (1,840–2,995)	67,074 (49,377–96,515)	7,710 (6,481–9,225)
Timor-Leste	Prevalence Number: Cumulative change (%)	62.5 (61.0–63.9)	28.2 (19.1–39.9)	69.0 (61.0–77.1)	46.3 (35.6–57.1)	44.8 (26.9–64.4)	29.8 (24.8–35.2)	124.8 (65.9–198.2)	59.5 (0.9–134.5)	67.5 (26.1–116.8)	61.9 (44.4–79.1)	53.0 (23.0–82.1)
Timor-Leste	Prevalence Rate: 1990	94,661.6 (94,131.9–95,169.8)	35,067.7 (33,307.0–36,933.7)	37,267.1 (35,883.2–38,663.0)	10,285.6 (8,620.7–11,985.1)	25,858.1 (23,874.4–28,349.4)	1,161.3 (1,076.8–1,263.9)	876.8 (686.6–1,116.4)	1,726.4 (1,238.1–2,282.7)	364.6 (284.6–448.8)	11,033.3 (8,344.3–15,417.7)	1,101.0 (889.0–1,367.0)
Timor-Leste	Prevalence Rate: 2015	93,321.2 (92,635.8–93,955.2)	27,504.4 (26,070.9–28,672.3)	37,719.9 (36,258.8–39,096.1)	9,100.9 (7,553.5–10,743.1)	22,649.9 (20,591.2–25,214.7)	948.8 (885.5–1,023.0)	1,189.7 (978.2–1,458.8)	1,680.8 (1,234.6–2,212.9)	369.5 (284.8–459.2)	10,769.9 (8,033.3–14,907.2)	1,098.4 (921.3–1,323.9)

© 2017 Global Burden of Disease Child and Adolescent Health Collaboration. JAMA Pediatrics.

eTable 3. Prevalent Cases, Rates (per 100,000 population), Years Lived with Disability (YLDs), and Cumulative Percent Change with 95% Uncertainty Interval (UI) for the Top 10 Global Causes of YLDs in Children and Adolescents in 195 Countries and Territories, Aged 0 to 19 Years, Both Sexes, 1990 and 2015. *Note: best viewed by enlarging in browser.*

Location	Measure	All causes	Iron-deficiency anemia	Skin and subcutaneous diseases	Asthma	Hemoglobinopathies and hemolytic anemias	Diarrheal diseases	Congenital anomalies	Protein-energy malnutrition	Epilepsy	Malaria	Neonatal preterm birth complications
Timor-Leste	Prevalence Rate: Cumulative change (%)	-1.4 (-2.2 to -0.6)	-21.5 (-26.1 to -15.8)	1.3 (-3.5 to 6.0)	-11.5 (-17.8 to -5.0)	-12.2 (-23.0 to -0.3)	-18.2 (-21.1 to -14.9)	38.5 (2.5–84.8)	0.8 (-36.2 to 48.2)	2.6 (-22.6 to 33.2)	-2.2 (-12.7 to 8.4)	1.0 (-22.1 to 23.5)
Timor-Leste	YLDs Number: 1990	25,668 (19,472–33,066)	5,304 (3,776–7,205)	3,282 (2,239–4,603)	1,716 (1,142–2,390)	743 (532–1,006)	848 (612–1,126)	253 (160–384)	1,073 (654–1,593)	582 (398–791)	268 (192–363)	451 (344–574)
Timor-Leste	YLDs Number: 2015	37,659 (28,500–48,221)	6,505 (4,579–8,830)	5,469 (3,720–7,683)	2,519 (1,660–3,470)	1,164 (819–1,581)	1,104 (799–1,472)	567 (355–876)	1,660 (1,027–2,437)	896 (587–1,231)	442 (316–600)	768 (599–956)
Timor-Leste	YLDs Number: Cumulative change (%)	46.8 (41.1–52.8)	22.8 (11.4–39.8)	66.6 (62.6–70.7)	46.9 (35.8–58.5)	56.6 (44.3–70.3)	30.2 (22.9–38.4)	126.6 (85.0–200.9)	60.4 (1.2–136.1)	56.1 (14.5–106.8)	65.1 (52.1–80.9)	71.1 (50.2–95.5)
Timor-Leste	YLDs Rate: 1990	6,808.4 (5,173.5–8,769.0)	1,386.0 (991.7–1,863.2)	873.5 (597.1–1,227.1)	464.5 (308.9–643.2)	197.5 (142.4–265.1)	188.7 (136.7–249.8)	64.7 (40.9–98.0)	214.5 (130.8–318.3)	146.4 (100.4–199.1)	72.1 (52.0–96.9)	112.0 (84.8–142.9)
Timor-Leste	YLDs Rate: 2015	6,065.2 (4,594.4–7,734.1)	1,033.8 (730.3–1,402.0)	875.3 (593.8–1,231.3)	412.8 (272.9–561.7)	186.5 (131.9–252.6)	154.7 (111.9–206.2)	88.8 (55.8–137.3)	209.8 (129.8–307.8)	138.2 (90.0–190.4)	72.4 (52.0–97.5)	119.4 (92.7–148.1)
Timor-Leste	YLDs Rate: Cumulative change (%)	-10.9 (-13.9 to -7.8)	-25.4 (-31.0 to -17.5)	0.2 (-2.1 to 2.5)	-11.1 (-17.6 to -4.3)	-5.6 (-11.9 to 0.5)	-18.0 (-22.4 to -12.9)	39.0 (13.3–86.4)	1.4 (-36.1 to 49.1)	-4.3 (-29.8 to 26.6)	0.4 (-6.5 to 8.7)	7.2 (-6.1 to 23.0)
Vietnam	Prevalence Number: 1990	31,397,844 (31,256,223–31,522,505)	9,931,544 (9,035,318–10,639,557)	12,006,834 (11,493,305–12,531,662)	1,746,661 (1,446,015–2,065,865)	7,206,107 (6,926,856–7,488,137)	359,643 (327,724–392,891)	446,233 (369,557–548,320)	371,188 (319,259–427,559)	101,283 (81,588–123,995)	147,449 (123,302–175,503)	507,541 (444,909–589,957)
Vietnam	Prevalence Number: 2015	25,623,941 (25,369,420–25,876,755)	4,653,884 (4,165,438–5,068,785)	10,772,894 (10,343,789–11,236,023)	1,715,483 (1,406,185–2,035,572)	5,355,734 (5,158,159–5,565,765)	208,926 (191,037–226,710)	432,082 (367,051–518,946)	198,777 (145,939–259,845)	96,045 (77,303–117,689)	152,261 (113,862–212,329)	378,880 (332,139–435,081)
Vietnam	Prevalence Number: Cumulative change (%)	-18.4 (-19.2 to -17.5)	-53.0 (-60.2 to -46.0)	-10.2 (-15.2 to -5.2)	-1.7 (-8.5 to 5.9)	-25.6 (-29.5 to -21.6)	-41.9 (-45.1 to -39.2)	-2.0 (-23.6 to 23.3)	-46.1 (-61.3 to -28.2)	-3.9 (-26.9 to 24.4)	2.9 (-17.5 to 34.9)	-25.0 (-36.5 to -13.0)
Vietnam	Prevalence Rate: 1990	95,865.5 (95,450.3–96,227.7)	29,937.5 (27,332.8–31,996.1)	37,085.1 (35,535.5–38,708.5)	5,372.1 (4,451.9–6,366.9)	21,974.7 (21,121.0–22,832.9)	1,055.5 (964.3–1,151.3)	1,358.6 (1,124.7–1,670.0)	1,058.8 (910.7–1,219.6)	311.3 (251.3–381.6)	451.2 (378.3–535.5)	1,508.7 (1,319.8–1,760.7)
Vietnam	Prevalence Rate: 2015	89,667.1 (88,777.7–90,546.0)	16,250.2 (14,580.1–17,666.4)	37,778.8 (36,282.9–39,396.2)	6,049.4 (4,956.0–7,178.3)	18,748.6 (18,056.2–19,484.4)	720.4 (659.3–781.1)	1,512.0 (1,284.8–1,816.6)	674.7 (495.4–882.0)	336.7 (270.7–412.7)	534.2 (399.8–743.8)	1,315.0 (1,152.7–1,511.4)
Vietnam	Prevalence Rate: Cumulative change (%)	-6.5 (-7.4 to -5.5)	-45.5 (-53.7 to -37.7)	1.9 (-3.8 to 7.7)	12.7 (5.1–21.3)	-14.6 (-19.1 to -10.0)	-31.7 (-35.4 to -28.6)	12.7 (-12.2 to 41.8)	-35.8 (-54.0 to -14.5)	9.5 (-16.6 to 41.9)	18.0 (-5.4 to 54.4)	-12.4 (-26.3 to 1.7)
Vietnam	YLDs Number: 1990	2,487,685 (1,812,244–3,328,372)	385,774 (270,770–532,320)	275,362 (186,432–385,661)	79,097 (51,887–108,742)	54,999 (39,008–75,326)	58,575 (42,201–78,607)	29,315 (18,492–44,076)	46,133 (31,289–63,181)	40,538 (27,009–55,405)	5,186 (3,497–7,256)	68,025 (53,243–85,188)
Vietnam	YLDs Number: 2015	1,600,882 (1,192,728–2,048,463)	152,055 (103,376–210,354)	243,466 (164,141–339,754)	78,287 (51,654–108,278)	71,924 (51,339–96,202)	34,324 (24,118–45,473)	36,186 (20,680–60,016)	24,837 (15,262–36,764)	25,999 (17,456–36,414)	5,282 (3,160–8,465)	42,135 (32,877–52,889)
Vietnam	YLDs Number: Cumulative change (%)	-35.2 (-43.8 to -27.6)	-60.4 (-68.4 to -51.7)	-11.6 (-14.0 to -9.3)	-0.9 (-8.4 to 7.5)	31.3 (16.6–47.6)	-41.4 (-45.4 to -37.8)	25.0 (-15.6 to 89.3)	-45.7 (-61.2 to -27.7)	-35.0 (-52.3 to -14.6)	1.1 (-21.4 to 32.3)	-38.0 (-43.4 to -32.4)
Vietnam	YLDs Rate: 1990	7,534.4 (5,508.3–10,068.8)	1,161.3 (815.3–1,596.6)	845.3 (571.7–1,185.9)	243.3 (159.3–333.9)	166.8 (118.4–227.5)	171.9 (123.8–230.3)	89.2 (56.2–134.3)	131.6 (89.3–180.2)	124.6 (83.0–170.1)	15.9 (10.7–22.2)	206.7 (161.5–259.1)
Vietnam	YLDs Rate: 2015	5,588.2 (4,164.1–7,150.3)	533.6 (362.4–736.1)	853.2 (575.5–1,190.2)	276.1 (181.9–382.2)	256.2 (183.4–342.4)	118.3 (83.1–156.8)	126.6 (72.4–209.7)	84.3 (51.8–124.8)	91.1 (61.2–127.8)	18.7 (11.2–29.9)	147.5 (115.1–185.1)
Vietnam	YLDs Rate: Cumulative change (%)	-25.4 (-34.9 to -17.0)	-53.9 (-62.9 to -44.1)	0.9 (-1.7 to 3.5)	13.6 (5.2–23.2)	54.3 (37.4–72.8)	-31.1 (-35.9 to -26.9)	43.6 (-3.0 to 117.8)	-35.4 (-53.8 to -14.0)	-25.8 (-45.6 to -2.6)	17.0 (-9.0 to 53.4)	-28.6 (-34.8 to -22.2)
Oceania	Prevalence Number: 1990	3,334,744 (3,324,475–3,344,333)	858,555 (817,810–915,070)	1,401,141 (1,365,001–1,435,280)	362,557 (306,519–422,195)	678,097 (653,091–701,486)	44,284 (40,823–47,990)	41,890 (37,041–48,271)	22,322 (17,180–28,554)	8,669 (7,245–10,215)	427,587 (372,809–485,534)	60,230 (52,929–70,223)
Oceania	Prevalence Number: 2015	4,801,545 (4,783,527–4,819,130)	1,171,545 (1,085,717–1,294,509)	2,038,890 (1,986,926–2,089,311)	533,158 (445,699–624,133)	964,886 (928,597–999,423)	43,411 (41,005–46,231)	64,763 (56,959–75,725)	23,563 (17,202–30,914)	13,568 (11,187–16,126)	448,392 (343,522–570,119)	86,629 (75,628–101,558)
Oceania	Prevalence Number: Cumulative change (%)	44.0 (43.3–44.6)	36.6 (26.9–57.1)	45.5 (41.3–50.5)	47.1 (39.5–54.9)	42.3 (35.9–49.0)	-1.9 (-7.8 to 4.2)	55.4 (29.0–85.2)	8.1 (-27.9 to 54.7)	57.3 (30.5–86.0)	4.6 (-12.7 to 21.9)	44.6 (22.3–73.1)
Oceania	Prevalence Rate: 1990	96,163.5 (95,885.0–96,421.1)	24,333.5 (23,241.7–25,809.9)	40,961.2 (39,920.7–41,950.8)	10,566.3 (8,940.1–12,298.8)	19,494.7 (18,774.2–20,171.9)	1,213.3 (1,122.2–1,311.0)	1,201.1 (1,061.5–1,385.1)	583.2 (448.9–746.1)	248.8 (208.3–292.7)	12,468.7 (10,894.5–14,124.8)	1,628.6 (1,425.1–1,908.4)
Oceania	Prevalence Rate: 2015	95,826.5 (95,482.9–96,168.1)	23,282.4 (21,623.1–25,665.3)	40,963.6 (39,923.3–41,968.6)	10,658.9 (8,937.3–12,494.6)	19,230.2 (18,507.9–19,917.4)	852.0 (805.6–906.2)	1,290.0 (1,134.5–1,509.2)	452.8 (330.5–594.0)	270.4 (222.9–321.3)	8,958.5 (6,884.3–11,361.4)	1,688.9 (1,473.6–1,986.0)
Oceania	Prevalence Rate: Cumulative change (%)	-0.4 (-0.8 to 0.1)	-4.2 (-10.8 to 9.7)	0.0 (-2.9 to 3.4)	0.9 (-4.3 to 6.2)	-1.3 (-5.8 to 3.3)	-29.7 (-33.8 to -25.4)	8.0 (-10.5 to 28.7)	-20.5 (-47.0 to 13.7)	9.2 (-9.4 to 29.4)	-28.3 (-40.1 to -16.4)	4.3 (-12.8 to 25.4)
Oceania	YLDs Number: 1990	252,072 (191,597–323,601)	36,474 (25,934–49,543)	40,148 (28,095–55,155)	16,317 (10,892–22,374)	4,418 (3,115–5,956)	7,172 (5,187–9,504)	2,942 (1,933–4,210)	2,758 (1,729–4,053)	3,363 (2,391–4,417)	13,245 (9,329–18,027)	5,049 (3,931–6,304)
Oceania	YLDs Number: 2015	344,463 (262,219–440,004)	49,509 (35,029–66,896)	57,805 (40,258–79,382)	24,064 (16,164–33,178)	6,559 (4,615–8,933)	7,041 (5,084–9,263)	5,006 (3,174–7,612)	2,933 (1,809–4,364)	4,823 (3,379–6,493)	13,309 (9,169–18,545)	7,384 (5,654–9,116)
Oceania	YLDs Number: Cumulative change (%)	36.7 (32.9–42.3)	36.1 (24.1–59.7)	43.9 (41.7–46.1)	47.5 (39.5–55.0)	48.8 (32.5–74.8)	-1.7 (-8.2 to 5.4)	70.7 (42.7–119.0)	8.8 (-27.4 to 55.1)	44.2 (16.3–75.4)	0.5 (-10.2 to 12.9)	46.3 (36.9–57.1)
Oceania	YLDs Rate: 1990	7,348.2 (5,582.4–9,409.7)	1,042.2 (741.0–1,408.0)	1,158.6 (810.7–1,591.0)	475.4 (316.8–650.6)	125.9 (89.1–169.4)	196.4 (142.1–261.0)	84.3 (55.3–120.4)	72.1 (45.2–105.9)	96.4 (68.5–126.2)	385.5 (272.2–523.8)	143.7 (111.8–179.3)
Oceania	YLDs Rate: 2015	6,913.9 (5,265.1–8,821.3)	986.6 (698.2–1,330.7)	1,153.6 (802.8–1,585.3)	481.1 (323.8–661.0)	130.0 (91.6–176.9)	138.2 (99.8–181.8)	99.7 (63.2–151.5)	56.4 (34.8–83.9)	96.1 (67.2–129.5)	265.1 (182.9–368.8)	146.6 (112.2–181.1)
Oceania	YLDs Rate: Cumulative change (%)	-5.9 (-8.5 to -2.1)	-5.1 (-13.3 to 10.9)	-0.4 (-2.0 to 1.0)	1.2 (-4.3 to 6.5)	3.5 (-7.6 to 20.8)	-29.6 (-34.1 to -24.6)	18.6 (-1.0 to 52.2)	-20.0 (-46.6 to 14.0)	0.2 (-19.3 to 21.6)	-31.3 (-38.6 to -22.8)	2.1 (-4.5 to 9.7)
American Samoa	Prevalence Number: 1990	20,979 (20,765–21,174)	5,479 (5,011–5,955)	9,517 (9,211–9,788)	1,917 (1,599–2,247)	3,800 (3,562–4,051)	132 (123–143)	364 (310–434)	128 (81–182)	61 (48–76)	0 (0–0)	353 (314–397)
American Samoa	Prevalence Number: 2015	35,292 (34,897–35,637)	9,445 (8,434–10,207)	15,883 (15,357–16,389)	3,582 (2,945–4,299)	6,106 (5,776–6,505)	210 (199–221)	616 (529–733)	145 (94–209)	128 (102–157)	0 (0–0)	534 (475–611)
American Samoa	Prevalence Number: Cumulative change (%)	68.2 (65.9–70.4)	73.3 (44.9–105.0)	66.9 (60.0–73.7)	87.0 (66.0–110.9)	60.9 (48.0–75.0)	58.8 (49.6–66.5)	70.8 (35.3–112.4)	19.3 (-34.5 to 98.8)	110.0 (58.4–168.3)	82.3 (61.9–104.6)	51.6 (33.0–72.4)
American Samoa	Prevalence Rate: 1990	89,823.0 (88,965.0–90,568.3)	22,346.9 (20,707.5–24,083.5)	41,690.5 (40,350.1–42,853.7)	8,612.7 (7,177.5–10,031.3)	16,060.7 (15,055.0–17,127.6)	504.2 (473.4–543.0)	1,537.8 (1,307.2–1,835.3)	420.1 (264.5–594.5)	261.5 (206.7–323.3)	0.0 (0.0–0.0)	1,352.2 (1,195.0–1,532.1)
American Samoa	Prevalence Rate: 2015	89,656.7 (88,789.3–90,453.0)	22,901.0 (20,598.1–24,704.5)	41,917.7 (40,596.9–43,187.5)	8,932.4 (7,398.5–10,585.5)	15,314.8 (14,491.3–16,312.2)	499.1 (476.0–523.8)	1,550.1 (1,330.6–1,842.8)	330.6 (214.1–476.7)	329.3 (262.4–403.6)	0.0 (0.0–0.0)	1,287.6 (1,139.2–1,475.8)
American Samoa	Prevalence Rate: Cumulative change (%)	-0.2 (-1.4 to 1.0)	2.9 (-12.3 to 21.2)	0.6 (-3.5 to 4.8)	3.9 (-7.0 to 16.4)	-4.5 (-12.3 to 3.8)	-0.9 (-6.0 to 3.1)	1.8 (-19.3 to 26.5)	-16.7 (-54.2 to 38.7)	27.5 (-3.9 to 63.3)	2.1 (-9.6 to 14.3)	-4.4 (-17.1 to 9.6)
American Samoa	YLDs Number: 1990	1,312 (1,005–1,678)	194 (136–265)	273 (189–377)	87 (57–119)	18 (13–24)	21 (15–28)	30 (19–44)	16 (8–25)	24 (16–32)	0 (0–0)	39 (31–49)
American Samoa	YLDs Number: 2015	2,149 (1,643–2,735)	353 (248–480)	464 (324–638)	162 (106–226)	30 (21–42)	34 (24–45)	60 (37–90)	18 (10–28)	33 (22–46)	0 (0–0)	50 (39–64)
American Samoa	YLDs Number: Cumulative change (%)	63.8 (57.2–70.4)	83.1 (49.4–111.4)	69.6 (66.1–72.9)	87.1 (65.6–111.0)	69.8 (46.2–97.4)	59.0 (45.9–71.1)	101.5 (61.1–152.2)	19.5 (-35.3 to 98.9)	41.6 (3.9–89.7)	91.7 (58.0–126.3)	27.3 (17.3–38.1)
American Samoa	YLDs Rate: 1990	5,758.7 (4,411.8–7,351.3)	806.6 (570.3–1,104.8)	1,169.4 (810.3–1,613.7)	391.3 (259.2–531.1)	78.9 (56.3–106.2)	82.5 (58.4–109.6)	127.8 (80.6–186.6)	52.4 (29.0–81.7)	101.9 (69.4–137.8)	0.0 (0.0–0.0)	168.2 (132.3–209.1)
American Samoa	YLDs Rate: 2015	5,620.6 (4,283.5–7,134.7)	831.6 (583.8–1,132.5)	1,170.4 (814.8–1,615.8)	405.9 (267.4–560.9)	73.3 (51.4–99.9)	81.7 (58.7–107.1)	152.6 (94.0–226.8)	41.3 (23.8–65.2)	86.2 (58.7–119.2)	0.0 (0.0–0.0)	126.9 (99.2–160.8)
American Samoa	YLDs Rate: Cumulative change (%)	-2.4 (-5.8 to 1.1)	3.5 (-13.8 to 18.1)	0.1 (-1.7 to 1.7)	3.9 (-7.3 to 16.3)	-6.8 (-18.7 to 8.9)	-0.8 (-8.2 to 6.5)	20.2 (-4.5 to 51.1)	-16.6 (-54.9 to 38.7)	-14.0 (-36.9 to 15.0)	1.2 (-15.0 to 18.9)	-24.5 (-30.6 to -18.0)
Federated States of Micronesia	Prevalence Number: 1990	50,208 (49,812–50,620)	11,235 (10,760–11,806)	21,564 (20,892–22,203)	4,901 (4,103–5,739)	8,663 (8,173–9,162)	441 (413–474)	762 (640–915)	307 (206–442)	142 (114–172)	0 (0–0)	1,019 (888–1,216)
Federated States of Micronesia	Prevalence Number: 2015	45,880 (45,513–46,209)	8,852 (8,220–9,571)	20,631 (20,000–21,238)	4,541 (3,785–5,400)	7,768 (7,331–8,310)	332 (313–351)	735 (633–883)	177 (120–249)	128 (100–159)	0 (0–0)	813 (712–942)
Federated States of Micronesia	Prevalence Number: Cumulative change (%)	-8.6 (-9.6 to -7.7)	-21.2 (-27.0 to -15.1)	-4.3 (-8.2 to -0.5)	-7.2 (-16.5 to 4.4)	-10.2 (-16.7 to -3.0)	-24.7 (-27.5 to -21.9)	-2.5 (-22.6 to 21.9)	-39.1 (-65.3 to -0.0)	-9.0 (-30.8 to 16.5)	-11.0 (-20.9 to -0.1)	-19.7 (-32.8 to -5.7)
Federated States of Micronesia	Prevalence Rate: 1990	93,650.9 (92,976.2–94,360.0)	20,714.7 (19,945.7–21,706.9)	40,936.6 (39,671.5–42,157.7)	9,184.4 (7,684.4–10,768.4)	16,103.3 (15,196.2–17,029.6)	781.7 (733.8–838.8)	1,414.3 (1,186.8–1,698.0)	517.5 (348.3–744.5)	263.5 (211.1–320.0)	0.0 (0.0–0.0)	1,820.5 (1,581.6–2,184.4)
Federated States of Micronesia	Prevalence Rate: 2015	93,082.2 (92,296.4–93,789.6)	18,184.1 (16,846.4–19,724.1)	41,265.7 (39,998.2–42,465.8)	9,228.4 (7,661.6–11,023.8)	15,802.7 (14,915.9–16,901.2)	696.8 (657.2–738.8)	1,498.6 (1,289.5–1,797.6)	385.7 (261.9–542.5)	260.3 (205.3–323.2)	0.0 (0.0–0.0)	1,700.9 (1,493.1–1,964.3)
Federated States of Micronesia	Prevalence Rate: Cumulative change (%)	-0.6 (-1.6 to 0.4)	-12.2 (-18.5 to -5.5)	0.8 (-3.4 to 4.8)	0.6 (-9.2 to 12.9)	-1.8 (-8.9 to 6.1)	-10.8 (-14.1 to -7.6)	7.1 (-14.8 to 33.9)	-21.5 (-55.3 to 28.8)	-0.2 (-24.1 to 27.5)	1.8 (-11.8 to 12.9)	-5.9 (-21.6 to 10.6)
Federated States of Micronesia	YLDs Number: 1990	3,217 (2,442–4,110)	408 (287–553)	622 (434–855)	222 (147–304)	42 (30–57)	72 (51–94)	58 (35–90)	38 (21–60)	54 (36–74)	0 (0–0)	112 (88–139)

© 2017 Global Burden of Disease Child and Adolescent Health Collaboration. JAMA Pediatrics.

eTable 3. Prevalent Cases, Rates (per 100,000 population), Years Lived with Disability (YLDs), and Cumulative Percent Change with 95% Uncertainty Interval (UI) for the Top 10 Global Causes of YLDs in Children and Adolescents in 195 Countries and Territories, Aged 0 to 19 Years, Both Sexes, 1990 and 2015. *Note: best viewed by enlarging in browser.*

Location	Measure	All causes	Iron-deficiency anemia	Skin and subcutaneous diseases	Asthma	Hemoglobinopathies and hemolytic anemias	Diarrheal diseases	Congenital anomalies	Protein-energy malnutrition	Epilepsy	Malaria	Neonatal preterm birth complications
Federated States of Micronesia	YLDs Number: 2015	2,786 (2,124–3,545)	316 (221–429)	571 (398–789)	206 (137–285)	35 (25–48)	54 (39–72)	70 (38–117)	22 (13–34)	35 (23–49)	0 (0–0)	76 (60–96)
Federated States of Micronesia	YLDs Number: Cumulative change (%)	-13.4 (-16.0 to -10.6)	-22.4 (-27.7 to -16.1)	-8.3 (-10.6 to -6.0)	-7.0 (-16.5 to 4.6)	-16.5 (-22.7 to -9.7)	-24.5 (-29.1 to -19.9)	23.4 (-25.4 to 99.5)	-38.7 (-65.4 to 1.4)	-33.1 (-51.1 to -11.3)	-14.3 (-27.4 to -2.0)	-31.8 (-37.7 to -26.0)
Federated States of Micronesia	YLDs Rate: 1990	6,097.4 (4,635.4–7,782.0)	748.2 (528.2–1,016.1)	1,158.0 (808.8–1,594.6)	416.3 (275.4–570.7)	79.1 (56.5–106.7)	127.7 (92.0–167.6)	107.9 (66.5–167.1)	64.6 (36.7–102.3)	100.9 (67.4–138.5)	0.0 (0.0–0.0)	207.6 (163.6–256.8)
Federated States of Micronesia	YLDs Rate: 2015	5,584.0 (4,255.1–7,115.9)	654.6 (457.0–889.7)	1,161.6 (811.6–1,601.1)	419.4 (278.3–581.7)	73.5 (51.7–100.1)	114.2 (82.0–151.3)	142.8 (78.0–239.9)	48.3 (28.4–74.8)	73.1 (47.1–101.3)	0.0 (0.0–0.0)	156.7 (122.6–197.4)
Federated States of Micronesia	YLDs Rate: Cumulative change (%)	-8.4 (-11.2 to -5.5)	-12.4 (-18.3 to -5.3)	0.3 (-1.6 to 2.2)	0.9 (-9.4 to 13.9)	-7.1 (-14.0 to 0.5)	-10.5 (-15.8 to -5.0)	35.8 (-17.9 to 119.4)	-21.1 (-55.5 to 30.6)	-26.6 (-46.3 to -2.1)	-0.7 (-15.8 to 13.9)	-24.5 (-31.1 to -18.3)
Fiji	Prevalence Number: 1990	340,967 (339,160–342,770)	98,595 (91,425–106,039)	146,441 (142,238–150,776)	37,805 (31,992–44,043)	49,543 (46,784–52,419)	2,625 (2,469–2,808)	5,312 (4,486–6,475)	1,925 (1,308–2,682)	1,173 (929–1,438)	0 (0–0)	5,650 (4,946–6,594)
Fiji	Prevalence Number: 2015	318,028 (316,347–319,569)	88,412 (81,786–95,326)	139,047 (135,051–143,169)	35,282 (29,886–41,281)	43,323 (41,003–45,793)	2,596 (2,451–2,757)	5,002 (4,297–5,947)	1,219 (800–1,745)	1,227 (983–1,494)	0 (0–0)	4,970 (4,372–5,780)
Fiji	Prevalence Number: Cumulative change (%)	-6.7 (-7.4 to -6.0)	-10.1 (-22.0 to 0.1)	-5.0 (-8.5 to -1.4)	-6.6 (-14.0 to 1.0)	-12.5 (-18.5 to -6.1)	-1.0 (-4.2 to 2.3)	-4.9 (-24.3 to 17.4)	-33.3 (-63.6 to 6.4)	6.0 (-18.5 to 36.3)	-6.4 (-18.1 to 4.2)	-11.5 (-25.3 to 3.6)
Fiji	Prevalence Rate: 1990	95,450.8 (94,977.9–95,927.8)	26,997.2 (25,145.2–28,914.9)	41,721.6 (40,542.9–42,943.9)	10,734.2 (9,108.8–12,468.8)	13,826.1 (13,051.3–14,631.6)	697.9 (658.6–743.8)	1,480.3 (1,248.9–1,806.6)	481.7 (327.3–670.9)	328.7 (261.5–402.0)	0.0 (0.0–0.0)	1,524.5 (1,331.3–1,787.0)
Fiji	Prevalence Rate: 2015	95,595.2 (95,097.2–96,063.2)	26,552.8 (24,579.7–28,599.0)	41,956.1 (40,749.6–43,200.7)	10,566.1 (8,947.1–12,350.1)	13,023.4 (12,326.9–13,764.6)	778.7 (735.0–826.5)	1,503.5 (1,291.9–1,787.3)	366.1 (240.4–524.1)	369.2 (295.8–449.3)	0.0 (0.0–0.0)	1,505.3 (1,325.1–1,748.9)
Fiji	Prevalence Rate: Cumulative change (%)	0.2 (-0.6 to 0.9)	-1.4 (-14.0 to 9.4)	0.6 (-3.1 to 4.4)	-1.5 (-9.3 to 6.6)	-5.7 (-12.3 to 1.2)	11.6 (8.1–15.3)	2.6 (-18.4 to 26.9)	-20.0 (-56.3 to 27.7)	13.8 (-12.6 to 45.9)	1.7 (-11.8 to 12.9)	-0.6 (-16.4 to 16.8)
Fiji	YLDs Number: 1990	23,428 (17,784–29,895)	3,756 (2,633–5,152)	4,144 (2,891–5,687)	1,707 (1,135–2,359)	194 (137–261)	427 (307–566)	368 (244–520)	239 (141–361)	449 (302–604)	0 (0–0)	607 (479–754)
Fiji	YLDs Number: 2015	20,803 (15,820–26,678)	3,341 (2,339–4,574)	3,860 (2,687–5,352)	1,597 (1,073–2,196)	172 (121–234)	422 (305–556)	358 (239–510)	151 (86–238)	317 (210–437)	0 (0–0)	552 (428–691)
Fiji	YLDs Number: Cumulative change (%)	-11.2 (-14.7 to -8.0)	-10.7 (-25.4 to 1.1)	-6.9 (-8.9 to -4.9)	-6.4 (-13.7 to 1.6)	-11.2 (-23.8 to -0.3)	-1.0 (-6.6 to 4.6)	-2.6 (-13.9 to 10.8)	-33.2 (-63.3 to 6.7)	-28.2 (-47.9 to -3.3)	-7.5 (-21.6 to 7.0)	-9.0 (-15.0 to -2.1)
Fiji	YLDs Rate: 1990	6,657.9 (5,063.2–8,494.4)	1,023.5 (719.5–1,405.8)	1,156.8 (806.3–1,600.0)	484.6 (322.7–666.2)	53.5 (38.0–71.9)	113.5 (81.7–150.2)	102.7 (68.1–145.0)	59.9 (35.4–90.5)	125.8 (84.3–169.5)	0.0 (0.0–0.0)	168.5 (133.2–209.2)
Fiji	YLDs Rate: 2015	6,271.1 (4,771.4–8,036.2)	1,000.5 (701.2–1,369.4)	1,158.8 (806.3–1,606.6)	478.3 (321.9–656.7)	51.7 (36.4–70.6)	126.8 (91.6–166.6)	107.8 (71.9–153.5)	45.5 (26.1–71.5)	95.6 (63.4–131.7)	0.0 (0.0–0.0)	165.9 (128.7–207.4)
Fiji	YLDs Rate: Cumulative change (%)	-5.8 (-9.5 to -2.5)	-1.9 (-17.5 to 10.9)	0.2 (-1.9 to 2.1)	-1.2 (-9.0 to 7.1)	-3.1 (-16.2 to 8.6)	11.8 (5.5–18.0)	5.2 (-7.3 to 19.6)	-19.8 (-56.0 to 28.2)	-22.8 (-44.1 to 3.9)	0.7 (-14.2 to 16.4)	-1.5 (-8.3 to 5.8)
Guam	Prevalence Number: 1990	48,089 (47,713–48,433)	9,879 (9,024–10,710)	21,532 (20,865–22,153)	3,946 (3,264–4,682)	8,360 (7,837–8,920)	193 (184–204)	809 (700–942)	237 (157–336)	117 (90–144)	0 (0–0)	677 (602–768)
Guam	Prevalence Number: 2015	52,055 (51,583–52,509)	11,976 (11,219–12,813)	25,000 (24,217–25,696)	5,210 (4,289–6,186)	8,881 (8,380–9,467)	227 (217–238)	913 (785–1,084)	165 (108–236)	156 (123–195)	0 (0–0)	720 (640–822)
Guam	Prevalence Number: Cumulative change (%)	8.2 (7.0–9.4)	21.4 (13.0–29.3)	16.1 (11.4–20.7)	32.1 (24.6–40.1)	6.4 (-2.5 to 15.7)	17.5 (14.0–21.1)	13.6 (-7.5 to 36.3)	-26.2 (-59.8 to 19.5)	35.8 (4.1–73.0)	12.1 (0.5–26.4)	6.7 (-6.2 to 21.2)
Guam	Prevalence Rate: 1990	92,802.8 (92,117.1–93,400.4)	18,699.0 (17,290.4–20,067.0)	42,058.7 (40,774.2–43,259.1)	7,867.0 (6,508.8–9,312.1)	16,044.8 (15,046.8–17,113.5)	348.1 (331.2–365.8)	1,550.8 (1,341.3–1,806.3)	385.0 (256.6–546.0)	223.8 (173.2–277.2)	0.0 (0.0–0.0)	1,234.6 (1,090.3–1,406.5)
Guam	Prevalence Rate: 2015	89,413.2 (88,565.0–90,221.4)	20,798.9 (19,458.3–22,330.9)	42,507.9 (41,169.0–43,707.9)	8,915.4 (7,344.4–10,603.2)	15,317.0 (14,460.5–16,326.7)	407.2 (387.9–427.2)	1,575.9 (1,354.7–1,872.6)	307.0 (202.1–440.3)	270.9 (212.4–336.6)	0.0 (0.0–0.0)	1,268.5 (1,128.9–1,442.7)
Guam	Prevalence Rate: Cumulative change (%)	-3.7 (-4.7 to -2.7)	11.3 (3.9–18.5)	1.1 (-2.9 to 5.0)	13.4 (7.3–20.2)	-4.4 (-12.3 to 3.8)	17.0 (13.5–20.6)	2.3 (-16.7 to 22.8)	-15.6 (-54.0 to 36.6)	22.6 (-6.1 to 55.8)	2.0 (-9.6 to 14.2)	3.1 (-9.8 to 17.3)
Guam	YLDs Number: 1990	2,832 (2,136–3,621)	347 (242–471)	609 (425–835)	179 (117–245)	37 (26–50)	31 (22–41)	64 (41–93)	29 (17–46)	46 (31–63)	0 (0–0)	74 (58–93)
Guam	YLDs Number: 2015	3,466 (2,615–4,389)	430 (302–579)	684 (473–949)	236 (156–328)	40 (29–54)	37 (26–49)	80 (51–114)	20 (11–32)	39 (26–54)	0 (0–0)	86 (68–108)
Guam	YLDs Number: Cumulative change (%)	22.8 (16.2–33.1)	24.2 (12.7–35.7)	12.3 (9.8–14.8)	32.0 (24.3–40.7)	10.2 (-0.3 to 20.6)	17.6 (9.5–25.9)	24.8 (5.2–47.7)	-26.1 (-60.4 to 20.6)	-12.6 (-36.1 to 14.3)	14.6 (-0.4 to 30.5)	16.0 (7.1–24.6)
Guam	YLDs Rate: 1990	5,560.4 (4,195.9–7,093.8)	670.3 (470.5–905.3)	1,174.2 (815.8–1,614.3)	357.7 (234.4–489.1)	73.8 (52.0–99.6)	57.0 (40.4–75.1)	123.7 (79.2–178.2)	48.1 (27.9–76.0)	88.3 (59.5–121.7)	0.0 (0.0–0.0)	143.3 (112.1–179.7)
Guam	YLDs Rate: 2015	5,897.0 (4,442.0–7,474.2)	748.6 (526.0–1,009.5)	1,178.7 (817.7–1,628.6)	405.3 (268.0–562.5)	70.7 (50.4–94.5)	66.7 (47.6–88.0)	138.7 (89.2–197.1)	38.4 (21.7–60.2)	68.7 (44.9–94.9)	0.0 (0.0–0.0)	149.4 (118.2–186.9)
Guam	YLDs Rate: Cumulative change (%)	6.4 (0.7–15.0)	11.8 (1.6–21.8)	0.4 (-1.6 to 2.1)	13.4 (7.1–20.7)	-4.0 (-13.4 to 5.5)	17.3 (9.2–25.3)	12.6 (-5.0 to 33.3)	-15.4 (-54.7 to 38.0)	-21.0 (-42.1 to 3.4)	2.1 (-11.9 to 17.3)	4.4 (-3.7 to 12.5)
Kiribati	Prevalence Number: 1990	34,466 (34,235–34,673)	8,810 (8,078–9,308)	13,928 (13,498–14,370)	4,395 (3,702–5,163)	6,140 (5,781–6,564)	617 (576–651)	383 (310–483)	463 (322–636)	121 (96–148)	2 (1–4)	665 (571–798)
Kiribati	Prevalence Number: 2015	49,120 (48,867–49,347)	12,446 (11,562–13,386)	20,529 (19,911–21,111)	5,683 (4,781–6,671)	8,506 (7,997–9,076)	499 (468–536)	610 (506–739)	414 (273–575)	156 (122–192)	3 (2–5)	954 (829–1,133)
Kiribati	Prevalence Number: Cumulative change (%)	42.5 (41.5–43.6)	41.4 (29.5–53.8)	47.4 (41.8–53.0)	29.5 (20.8–39.3)	38.7 (27.4–50.1)	-19.1 (-24.0 to -13.2)	61.6 (20.8–112.7)	-7.2 (-45.9 to 44.2)	31.1 (-1.9 to 68.8)	39.3 (25.4–52.6)	45.0 (18.0–76.9)
Kiribati	Prevalence Rate: 1990	96,965.5 (96,437.5–97,442.5)	24,098.7 (22,425.2–25,312.9)	40,652.2 (39,420.6–41,862.6)	12,685.0 (10,744.2–14,853.3)	17,203.1 (16,186.3–18,398.6)	1,519.8 (1,427.0–1,598.6)	1,066.0 (862.3–1,340.7)	1,043.2 (726.6–1,430.8)	335.3 (265.0–410.7)	7.8 (4.9–10.8)	1,632.4 (1,384.4–2,016.0)
Kiribati	Prevalence Rate: 2015	95,950.8 (95,495.4–96,366.0)	24,059.1 (22,429.1–25,834.3)	40,719.3 (39,495.1–41,844.2)	11,235.3 (9,481.9–13,172.9)	16,583.3 (15,598.0–17,684.8)	932.6 (876.8–998.6)	1,187.2 (984.5–1,438.3)	731.1 (482.3–1,015.9)	304.2 (237.3–374.6)	7.7 (4.8–10.8)	1,756.3 (1,516.1–2,110.7)
Kiribati	Prevalence Rate: Cumulative change (%)	-1.0 (-1.7 to -0.4)	-0.1 (-7.9 to 8.0)	0.2 (-3.5 to 4.1)	-11.3 (-17.2 to -5.0)	-3.5 (-11.4 to 4.4)	-38.6 (-42.2 to -34.4)	13.1 (-15.7 to 48.8)	-27.2 (-57.5 to 13.1)	-8.0 (-31.3 to 18.1)	-1.6 (-11.2 to 8.1)	8.9 (-13.7 to 35.2)
Kiribati	YLDs Number: 1990	2,631 (1,978–3,419)	321 (229–436)	407 (285–559)	197 (132–270)	27 (19–37)	100 (73–131)	27 (17–40)	57 (34–89)	45 (31–62)	0 (0–0)	53 (41–67)
Kiribati	YLDs Number: 2015	3,549 (2,664–4,613)	449 (318–618)	585 (410–807)	255 (171–350)	38 (27–51)	80 (58–106)	62 (34–108)	51 (29–80)	41 (27–57)	0 (0–0)	77 (59–97)
Kiribati	YLDs Number: Cumulative change (%)	34.9 (30.1–39.5)	40.3 (26.0–57.1)	43.7 (40.7–46.7)	29.8 (20.9–39.8)	39.0 (28.4–51.9)	-19.1 (-24.8 to -12.4)	131.3 (49.1–279.7)	-6.9 (-46.1 to 46.1)	-7.7 (-33.0 to 22.1)	42.6 (-4.4 to 101.5)	45.2 (32.0–60.7)
Kiribati	YLDs Rate: 1990	7,571.6 (5,701.8–9,835.8)	877.2 (629.4–1,189.7)	1,147.3 (798.7–1,583.6)	568.8 (383.2–777.1)	76.8 (54.7–104.6)	246.1 (179.1–324.5)	75.7 (49.2–111.3)	129.2 (77.2–200.6)	126.4 (85.5–172.8)	0.3 (0.2–0.5)	145.5 (113.4–182.8)
Kiribati	YLDs Rate: 2015	7,022.5 (5,274.0–9,112.4)	877.2 (622.9–1,201.6)	1,145.3 (799.6–1,577.3)	505.2 (339.0–692.5)	75.9 (53.5–102.7)	151.1 (109.2–199.2)	120.8 (66.5–210.1)	90.7 (51.7–142.4)	80.7 (53.3–111.8)	0.3 (0.1–0.5)	149.8 (114.6–188.5)
Kiribati	YLDs Rate: Cumulative change (%)	-7.2 (-10.5 to -4.2)	0.1 (-9.6 to 10.5)	-0.2 (-2.1 to 1.7)	-11.1 (-17.3 to -4.4)	-1.1 (-8.1 to 6.7)	-38.6 (-42.8 to -33.8)	62.0 (4.5–165.9)	-26.9 (-57.6 to 14.7)	-35.2 (-53.2 to -14.2)	1.0 (-30.8 to 40.7)	3.2 (-6.3 to 14.0)
Marshall Islands	Prevalence Number: 1990	24,946 (24,796–25,089)	8,589 (8,127–9,131)	10,270 (9,933–10,580)	2,252 (1,893–2,636)	4,425 (4,153–4,726)	247 (229–264)	358 (299–432)	173 (112–253)	65 (51–80)	0 (0–0)	494 (435–571)
Marshall Islands	Prevalence Number: 2015	32,273 (32,120–32,417)	10,336 (9,524–11,192)	13,657 (13,218–14,076)	3,084 (2,525–3,667)	5,443 (5,130–5,810)	257 (242–274)	481 (404–583)	143 (94–205)	90 (72–110)	0 (0–0)	577 (509–673)
Marshall Islands	Prevalence Number: Cumulative change (%)	29.4 (28.5–30.3)	20.4 (12.6–33.9)	33.0 (27.7–39.1)	37.1 (22.9–52.0)	23.2 (13.1–34.1)	4.3 (0.8–8.3)	35.7 (6.0–71.1)	-11.6 (-53.5 to 53.8)	38.8 (4.8–76.6)	32.0 (16.1–46.8)	17.4 (-0.8 to 39.2)
Marshall Islands	Prevalence Rate: 1990	96,840.7 (96,340.1–97,292.7)	32,908.5 (31,546.2–34,658.6)	40,937.8 (39,613.4–42,150.8)	9,131.8 (7,654.4–10,679.7)	17,125.1 (16,068.4–18,303.8)	858.1 (803.8–914.5)	1,378.7 (1,151.4–1,663.8)	528.7 (344.2–771.9)	253.8 (197.0–307.3)	0.0 (0.0–0.0)	1,712.1 (1,495.7–2,004.9)
Marshall Islands	Prevalence Rate: 2015	96,643.1 (96,207.6–97,043.5)	30,841.1 (28,549.2–33,259.4)	41,217.5 (39,877.2–42,469.6)	9,297.3 (7,630.3–11,051.7)	16,290.7 (15,353.3–17,383.6)	752.5 (708.3–800.1)	1,438.2 (1,209.5–1,743.7)	404.7 (266.3–579.2)	270.7 (215.3–330.5)	0.0 (0.0–0.0)	1,707.5 (1,504.0–1,994.0)
Marshall Islands	Prevalence Rate: Cumulative change (%)	-0.2 (-0.8 to 0.5)	-6.3 (-11.6 to 2.4)	0.7 (-3.2 to 5.2)	1.9 (-8.3 to 12.9)	-4.7 (-12.5 to 3.7)	-12.3 (-15.2 to -9.2)	5.5 (-17.7 to 33.0)	-18.4 (-57.0 to 42.0)	7.9 (-18.3 to 36.5)	1.7 (-11.8 to 13.0)	0.4 (-16.4 to 19.8)
Marshall Islands	YLDs Number: 1990	1,792 (1,351–2,318)	330 (234–448)	296 (207–405)	101 (67–139)	23 (16–31)	40 (29–52)	26 (16–41)	21 (11–33)	25 (17–35)	0 (0–0)	48 (37–60)
Marshall Islands	YLDs Number: 2015	2,278 (1,721–2,930)	395 (278–540)	385 (269–527)	138 (92–193)	28 (19–37)	41 (29–55)	52 (25–103)	17 (10–27)	30 (20–41)	0 (0–0)	56 (43–69)
Marshall Islands	YLDs Number: Cumulative change (%)	27.1 (21.7–32.9)	19.8 (10.6–36.6)	30.1 (27.4–32.7)	37.2 (22.8–52.2)	22.3 (13.5–34.0)	4.4 (-1.6 to 10.2)	102.1 (16.7–275.6)	-11.3 (-53.1 to 54.4)	17.9 (-12.7 to 54.2)	32.0 (12.3–51.3)	16.2 (7.3–25.2)

© 2017 Global Burden of Disease Child and Adolescent Health Collaboration. JAMA Pediatrics.

eTable 3. Prevalent Cases, Rates (per 100,000 population), Years Lived with Disability (YLDs), and Cumulative Percent Change with 95% Uncertainty Interval (UI) for the Top 10 Global Causes of YLDs in Children and Adolescents in 195 Countries and Territories, Aged 0 to 19 Years, Both Sexes, 1990 and 2015. *Note: best viewed by enlarging in browser.*

Location	Measure	All causes	Iron-deficiency anemia	Skin and subcutaneous diseases	Asthma	Hemoglobinopathies and hemolytic anemias	Diarrheal diseases	Congenital anomalies	Protein-energy malnutrition	Epilepsy	Malaria	Neonatal preterm birth complications
Marshall Islands	YLDs Rate: 1990	7,142.1 (5,379.1–9,190.9)	1,279.1 (915.0–1,719.4)	1,152.0 (806.5–1,582.1)	410.9 (275.1–563.7)	90.4 (64.6–122.2)	139.2 (100.9–181.7)	102.0 (63.5–158.7)	65.6 (35.8–102.7)	99.3 (67.9–135.3)	0.0 (0.0–0.0)	184.5 (144.5–230.9)
Marshall Islands	YLDs Rate: 2015	6,874.7 (5,197.0–8,845.9)	1,180.2 (831.5–1,606.5)	1,152.4 (805.3–1,581.2)	418.9 (279.5–582.0)	84.3 (59.8–113.6)	122.2 (87.5–161.4)	158.0 (77.2–310.1)	50.2 (28.7–77.3)	89.8 (61.1–122.7)	0.0 (0.0–0.0)	167.4 (130.8–207.7)
Marshall Islands	YLDs Rate: Cumulative change (%)	-3.7 (-7.8 to 0.5)	-7.8 (-14.1 to 2.8)	0.0 (-1.8 to 2.0)	2.0 (-8.4 to 12.9)	-6.8 (-13.5 to 0.5)	-12.1 (-17.0 to -7.1)	57.3 (-9.0 to 191.8)	-18.1 (-56.6 to 42.4)	-8.2 (-32.0 to 20.3)	0.2 (-14.6 to 15.2)	-9.2 (-16.2 to -2.0)
Northern Mariana Islands	Prevalence Number: 1990	11,931 (11,839–12,019)	2,710 (2,412–3,029)	5,310 (5,152–5,460)	1,102 (915–1,305)	2,087 (1,962–2,218)	66 (60–73)	201 (172–234)	65 (44–90)	30 (23–36)	0 (0–0)	166 (147–188)
Northern Mariana Islands	Prevalence Number: 2015	27,161 (26,903–27,410)	6,814 (6,127–7,593)	12,650 (12,243–13,019)	2,403 (1,975–2,838)	4,663 (4,422–4,931)	132 (124–140)	475 (407–566)	104 (68–149)	78 (61–97)	0 (0–0)	383 (330–444)
Northern Mariana Islands	Prevalence Number: Cumulative change (%)	127.6 (125.1–130.3)	152.3 (118.1–188.9)	138.3 (129.2–148.1)	118.2 (102.7–135.6)	123.7 (105.9–141.4)	98.3 (87.3–110.0)	137.8 (89.1–192.4)	69.0 (-6.2 to 174.5)	163.0 (101.1–235.2)	144.7 (118.8–174.0)	130.8 (98.7–167.6)
Northern Mariana Islands	Prevalence Rate: 1990	93,139.7 (92,501.6–93,764.5)	20,595.5 (18,562.6–22,757.7)	42,196.0 (40,941.5–43,366.6)	8,814.9 (7,303.7–10,412.7)	16,235.0 (15,265.3–17,241.9)	480.7 (438.9–523.8)	1,563.9 (1,333.5–1,816.4)	434.4 (295.9–604.6)	233.2 (184.6–285.0)	0.0 (0.0–0.0)	1,210.3 (1,064.4–1,379.6)
Northern Mariana Islands	Prevalence Rate: 2015	89,604.2 (88,762.4–90,412.1)	22,204.1 (19,918.5–24,762.4)	42,224.7 (40,841.4–43,434.7)	7,832.4 (6,446.5–9,247.0)	15,358.3 (14,554.7–16,237.5)	430.3 (405.4–456.3)	1,565.8 (1,341.4–1,863.1)	343.5 (223.4–491.8)	257.1 (201.4–318.7)	0.0 (0.0–0.0)	1,249.8 (1,077.4–1,452.7)
Northern Mariana Islands	Prevalence Rate: Cumulative change (%)	-3.8 (-4.8 to -2.7)	8.1 (-6.0 to 22.6)	0.1 (-3.7 to 4.1)	-11.1 (-17.3 to -4.0)	-5.3 (-12.8 to 2.2)	-10.4 (-15.0 to -5.5)	1.0 (-19.8 to 24.1)	-16.8 (-53.8 to 35.1)	11.5 (-14.5 to 42.4)	2.0 (-9.6 to 14.1)	3.7 (-11.4 to 20.9)
Northern Mariana Islands	YLDs Number: 1990	727 (549–925)	96 (66–131)	150 (104–206)	50 (32–68)	9 (6–12)	10 (7–14)	14 (9–20)	8 (4–12)	11 (7–16)	0 (0–0)	19 (15–24)
Northern Mariana Islands	YLDs Number: 2015	1,624 (1,232–2,075)	252 (176–346)	357 (249–490)	109 (72–150)	22 (15–30)	21 (15–28)	40 (25–58)	13 (7–21)	20 (13–28)	0 (0–0)	35 (26–44)
Northern Mariana Islands	YLDs Number: Cumulative change (%)	123.4 (114.5–131.2)	163.9 (115.2–200.4)	137.6 (132.8–142.5)	118.5 (102.3–136.0)	138.7 (108.6–170.3)	98.5 (82.3–115.7)	178.5 (128.1–235.8)	69.2 (-6.3 to 175.9)	72.8 (26.5–129.2)	149.2 (113.5–184.6)	81.7 (63.3–102.1)
Northern Mariana Islands	YLDs Rate: 1990	5,770.7 (4,369.0–7,338.0)	739.0 (512.7–1,016.6)	1,177.1 (817.5–1,625.3)	400.1 (262.8–550.3)	73.0 (51.6–99.2)	78.6 (56.0–104.0)	111.8 (72.9–161.1)	54.2 (31.8–83.2)	91.8 (61.6–126.3)	0.0 (0.0–0.0)	149.6 (117.0–189.7)
Northern Mariana Islands	YLDs Rate: 2015	5,395.2 (4,092.2–6,880.9)	811.2 (565.4–1,114.3)	1,177.4 (817.7–1,617.5)	356.2 (235.6–492.5)	70.9 (50.2–96.5)	70.4 (50.6–93.9)	131.9 (84.5–191.1)	42.9 (24.8–70.3)	66.4 (45.2–93.8)	0.0 (0.0–0.0)	115.1 (88.0–146.7)
Northern Mariana Islands	YLDs Rate: Cumulative change (%)	-6.5 (-10.0 to -3.4)	10.3 (-9.6 to 25.4)	0.0 (-1.9 to 2.1)	-11.0 (-17.4 to -3.8)	-2.5 (-14.6 to 10.3)	-10.2 (-17.2 to -2.8)	18.5 (-2.9 to 42.9)	-16.7 (-53.8 to 35.8)	-26.7 (-46.3 to -2.8)	1.3 (-13.5 to 15.9)	-23.0 (-30.9 to -14.2)
Papua New Guinea	Prevalence Number: 1990	2,188,150 (2,179,429–2,196,471)	563,880 (525,652–617,503)	911,926 (880,567–940,877)	245,163 (206,142–286,075)	468,398 (446,205–488,713)	32,776 (29,881–35,781)	24,935 (20,828–30,330)	14,711 (9,954–20,487)	5,360 (4,266–6,505)	333,877 (291,245–377,500)	39,111 (33,184–47,635)
Papua New Guinea	Prevalence Number: 2015	3,517,948 (3,501,119–3,534,051)	858,429 (774,103–976,810)	1,480,898 (1,433,086–1,527,054)	399,438 (333,784–468,763)	739,444 (707,020–771,913)	32,509 (30,614–34,698)	45,026 (37,847–55,262)	17,932 (12,005–24,881)	9,559 (7,589–11,684)	362,472 (280,334–455,388)	63,715 (54,240–77,430)
Papua New Guinea	Prevalence Number: Cumulative change (%)	60.8 (59.8–61.7)	52.6 (37.5–84.5)	62.4 (56.0–69.7)	63.0 (53.1–73.6)	58.0 (48.8–67.2)	-0.6 (-8.2 to 7.1)	82.8 (38.8–131.3)	27.7 (-26.3 to 107.2)	80.4 (38.0–131.4)	8.4 (-9.6 to 26.7)	64.6 (31.0–107.3)
Papua New Guinea	Prevalence Rate: 1990	96,964.0 (96,595.6–97,319.9)	24,629.0 (23,021.5–26,831.4)	40,906.6 (39,509.3–42,197.4)	10,954.4 (9,228.1–12,728.0)	20,700.7 (19,721.3–21,602.6)	1,390.1 (1,271.7–1,514.1)	1,098.7 (916.6–1,337.4)	600.0 (406.1–835.6)	236.3 (187.9–286.7)	14,968.9 (13,079.1–16,923.1)	1,618.2 (1,364.3–1,995.0)
Papua New Guinea	Prevalence Rate: 2015	96,329.9 (95,883.2–96,755.7)	23,421.6 (21,220.7–26,551.4)	40,883.5 (39,557.5–42,153.0)	10,963.0 (9,166.8–12,851.6)	20,214.2 (19,327.3–21,101.3)	871.9 (822.3–929.3)	1,229.7 (1,034.1–1,508.4)	467.8 (313.2–649.0)	261.3 (207.3–319.7)	9,948.3 (7,708.5–12,503.5)	1,692.1 (1,433.9–2,064.7)
Papua New Guinea	Prevalence Rate: Cumulative change (%)	-0.7 (-1.2 to -0.1)	-4.7 (-13.7 to 14.5)	-0.0 (-4.0 to 4.5)	0.1 (-6.0 to 6.6)	-2.3 (-8.0 to 3.4)	-37.2 (-41.8 to -32.4)	13.3 (-13.8 to 43.2)	-18.3 (-52.9 to 32.5)	11.8 (-14.4 to 43.3)	-33.6 (-44.8 to -22.4)	5.8 (-16.9 to 34.3)
Papua New Guinea	YLDs Number: 1990	172,970 (131,330–223,324)	25,217 (17,753–34,523)	26,155 (18,338–35,925)	11,013 (7,298–15,089)	3,465 (2,435–4,701)	5,301 (3,843–7,092)	1,731 (1,121–2,535)	1,813 (1,047–2,763)	2,088 (1,430–2,847)	11,195 (7,845–15,245)	2,947 (2,245–3,732)
Papua New Guinea	YLDs Number: 2015	259,506 (196,774–332,428)	37,681 (26,476–51,922)	42,163 (29,436–58,144)	18,009 (12,130–24,846)	5,559 (3,873–7,629)	5,266 (3,813–6,913)	3,379 (2,078–5,361)	2,232 (1,285–3,494)	3,655 (2,494–4,994)	11,680 (7,988–16,361)	5,150 (3,896–6,464)
Papua New Guinea	YLDs Number: Cumulative change (%)	50.2 (44.6–58.0)	50.1 (32.8–84.2)	61.2 (57.9–64.2)	63.6 (53.0–74.0)	61.0 (40.1–93.7)	-0.5 (-8.7 to 8.7)	97.0 (51.8–176.1)	28.9 (-26.0 to 109.0)	77.4 (31.6–131.4)	4.3 (-8.0 to 18.9)	75.2 (58.3–94.2)
Papua New Guinea	YLDs Rate: 1990	7,735.3 (5,874.0–9,973.1)	1,111.0 (787.7–1,514.3)	1,161.2 (814.1–1,598.8)	492.0 (326.9–672.8)	151.8 (106.8–205.0)	224.7 (162.9–300.1)	76.2 (49.3–111.7)	74.0 (42.7–112.7)	92.0 (63.1–125.6)	500.4 (351.3–681.8)	128.7 (98.1–162.7)
Papua New Guinea	YLDs Rate: 2015	7,159.2 (5,436.5–9,161.1)	1,033.0 (730.2–1,422.8)	1,154.5 (805.3–1,593.5)	494.2 (332.6–678.9)	151.1 (105.4–206.8)	141.2 (102.2–185.6)	92.2 (56.7–146.5)	58.2 (33.5–91.2)	99.9 (68.1–136.5)	319.5 (218.9–448.0)	140.0 (105.9–175.6)
Papua New Guinea	YLDs Rate: Cumulative change (%)	-7.4 (-10.7 to -2.7)	-6.6 (-17.1 to 13.8)	-0.6 (-2.6 to 1.2)	0.5 (-6.1 to 6.9)	-0.1 (-12.7 to 19.2)	-37.0 (-42.2 to -31.4)	22.1 (-5.8 to 71.1)	-17.6 (-52.6 to 33.6)	10.0 (-18.3 to 43.4)	-36.2 (-43.6 to -27.3)	9.1 (-1.7 to 21.0)
Samoa	Prevalence Number: 1990	81,902 (81,115–82,614)	20,827 (19,163–22,202)	36,921 (35,814–37,971)	6,233 (5,212–7,353)	14,570 (13,605–15,608)	626 (583–675)	1,413 (1,202–1,691)	233 (154–329)	225 (179–275)	0 (0–0)	1,632 (1,457–1,854)
Samoa	Prevalence Number: 2015	84,294 (83,523–84,923)	22,051 (20,885–23,309)	38,433 (37,323–39,525)	7,158 (5,894–8,490)	14,431 (13,641–15,374)	652 (610–696)	1,436 (1,207–1,709)	167 (110–242)	246 (195–303)	0 (0–0)	1,467 (1,284–1,693)
Samoa	Prevalence Number: Cumulative change (%)	2.9 (1.7–4.1)	6.1 (-2.4 to 20.6)	4.1 (0.3–8.3)	14.9 (3.8–26.7)	-0.8 (-9.3 to 7.9)	4.3 (0.6–7.5)	2.6 (-19.0 to 28.4)	-24.8 (-56.1 to 20.6)	10.3 (-17.0 to 42.0)	7.1 (-6.7 to 18.9)	-9.8 (-22.9 to 4.5)
Samoa	Prevalence Rate: 1990	90,576.8 (89,729.0–91,340.5)	22,512.3 (20,809.5–23,862.6)	41,040.3 (39,807.8–42,197.0)	7,069.0 (5,908.4–8,329.2)	16,044.9 (14,982.4–17,187.1)	656.0 (613.9–703.7)	1,556.3 (1,323.1–1,862.1)	226.8 (150.6–320.5)	249.3 (197.7–304.4)	0.0 (0.0–0.0)	1,737.6 (1,545.3–1,976.2)
Samoa	Prevalence Rate: 2015	90,381.5 (89,570.7–91,041.2)	23,537.2 (22,300.2–24,863.6)	41,491.2 (40,298.6–42,650.1)	7,617.9 (6,281.1–9,024.2)	15,459.4 (14,614.1–16,466.9)	693.4 (648.9–739.9)	1,538.5 (1,292.7–1,831.6)	177.1 (116.6–256.9)	263.5 (209.1–324.4)	0.0 (0.0–0.0)	1,586.0 (1,388.6–1,829.3)
Samoa	Prevalence Rate: Cumulative change (%)	-0.2 (-1.4 to 0.9)	4.7 (-3.2 to 18.4)	1.1 (-2.6 to 5.2)	7.9 (-2.6 to 18.6)	-3.5 (-11.7 to 5.0)	5.7 (2.1–8.8)	-0.2 (-21.1 to 25.2)	-18.0 (-52.2 to 31.5)	7.0 (-19.4 to 37.6)	1.8 (-11.8 to 13.0)	-8.3 (-21.7 to 6.2)
Samoa	YLDs Number: 1990	5,172 (3,956–6,585)	739 (515–1,004)	1,053 (736–1,443)	283 (187–390)	76 (53–103)	102 (73–134)	109 (66–175)	29 (17–45)	87 (60–119)	0 (0–0)	174 (138–218)
Samoa	YLDs Number: 2015	5,411 (4,138–6,874)	805 (572–1,078)	1,092 (762–1,496)	325 (215–452)	74 (52–100)	106 (75–141)	161 (79–304)	20 (12–33)	90 (59–124)	0 (0–0)	141 (110–178)
Samoa	YLDs Number: Cumulative change (%)	4.7 (1.2–9.6)	9.5 (-1.9 to 31.7)	3.7 (1.7–5.5)	14.9 (3.4–26.9)	-1.5 (-10.5 to 15.7)	4.3 (-1.6 to 10.4)	52.1 (-23.2 to 180.5)	-24.6 (-57.1 to 21.9)	5.0 (-23.7 to 36.3)	8.8 (-6.3 to 22.4)	-19.0 (-25.4 to -12.4)
Samoa	YLDs Rate: 1990	5,759.0 (4,399.1–7,328.5)	810.6 (568.8–1,095.3)	1,162.2 (811.3–1,598.4)	321.3 (211.8–442.3)	85.3 (59.8–116.8)	107.2 (77.2–141.3)	120.0 (73.1–192.5)	28.2 (16.6–44.7)	96.8 (66.5–132.7)	0.0 (0.0–0.0)	192.7 (152.5–240.2)
Samoa	YLDs Rate: 2015	5,830.3 (4,459.7–7,402.0)	853.6 (606.0–1,143.0)	1,167.3 (813.2–1,597.9)	346.0 (228.8–481.7)	79.3 (56.4–107.1)	113.4 (80.6–150.1)	173.3 (85.5–326.4)	22.0 (12.9–35.0)	97.2 (63.9–134.1)	0.0 (0.0–0.0)	151.6 (118.2–190.4)
Samoa	YLDs Rate: Cumulative change (%)	1.3 (-2.0 to 5.9)	5.7 (-4.6 to 25.8)	0.4 (-1.5 to 2.2)	7.8 (-2.9 to 18.8)	-6.8 (-15.3 to 8.6)	5.8 (-0.1 to 11.9)	48.0 (-25.3 to 173.2)	-17.8 (-53.3 to 32.8)	1.9 (-26.0 to 32.5)	0.9 (-13.7 to 13.8)	-21.3 (-27.3 to -14.9)
Solomon Islands	Prevalence Number: 1990	174,764 (173,837–175,663)	42,832 (38,378–47,402)	69,998 (67,816–72,194)	16,539 (13,864–19,369)	42,874 (39,852–45,939)	2,120 (1,983–2,273)	2,516 (2,087–3,097)	1,709 (1,327–2,172)	438 (345–531)	48,279 (39,756–56,914)	3,861 (3,406–4,474)
Solomon Islands	Prevalence Number: 2015	281,774 (280,023–283,431)	69,136 (61,091–75,043)	115,548 (111,716–118,949)	27,999 (22,983–33,235)	62,053 (57,814–66,190)	2,651 (2,501–2,826)	4,345 (3,688–5,178)	1,479 (991–2,078)	774 (597–948)	50,663 (36,664–67,219)	6,004 (5,291–7,008)
Solomon Islands	Prevalence Number: Cumulative change (%)	61.2 (60.0–62.5)	62.1 (39.9–89.9)	65.1 (58.3–72.5)	69.4 (52.1–86.0)	45.0 (32.2–58.6)	25.1 (21.0–29.3)	74.6 (35.5–117.0)	-11.3 (-45.8 to 34.6)	78.8 (37.2–131.8)	4.7 (-16.3 to 28.4)	56.5 (30.1–85.4)
Solomon Islands	Prevalence Rate: 1990	96,249.1 (95,797.2–96,691.6)	22,844.0 (20,746.8–25,062.9)	39,459.8 (38,226.3–40,661.6)	9,383.2 (7,867.3–10,970.6)	23,515.7 (21,857.8–25,195.9)	1,079.0 (1,013.2–1,153.8)	1,375.5 (1,140.2–1,695.0)	786.5 (611.2–999.6)	240.6 (190.2–292.6)	26,711.7 (22,100.2–31,651.7)	1,936.1 (1,693.9–2,278.8)
Solomon Islands	Prevalence Rate: 2015	95,523.6 (94,962.7–96,048.0)	23,052.0 (20,541.3–24,902.3)	39,717.9 (38,418.9–40,871.2)	9,549.9 (7,851.8–11,318.2)	20,989.0 (19,552.1–22,380.8)	868.9 (821.3–926.7)	1,469.4 (1,246.6–1,749.9)	464.2 (311.1–652.1)	262.6 (201.8–320.2)	17,168.3 (12,533.4–22,560.2)	1,976.9 (1,738.0–2,315.9)
Solomon Islands	Prevalence Rate: Cumulative change (%)	-0.8 (-1.5 to -0.0)	1.2 (-11.0 to 16.7)	0.7 (-3.6 to 5.1)	1.8 (-8.5 to 11.9)	-10.6 (-18.5 to -2.2)	-19.4 (-21.9 to -16.9)	8.0 (-16.1 to 34.0)	-39.5 (-63.0 to -8.2)	10.4 (-15.9 to 42.7)	-35.8 (-48.4 to -21.7)	2.9 (-15.9 to 22.7)
Solomon Islands	YLDs Number: 1990	11,755 (8,929–15,022)	1,597 (1,100–2,203)	2,013 (1,413–2,757)	747 (495–1,016)	133 (92–183)	344 (250–458)	176 (114–271)	212 (134–305)	169 (112–230)	700 (497–950)	365 (285–457)
Solomon Islands	YLDs Number: 2015	18,186 (13,896–23,184)	2,553 (1,767–3,473)	3,273 (2,294–4,485)	1,268 (840–1,753)	216 (151–292)	431 (312–570)	351 (209–553)	184 (104–281)	231 (155–325)	738 (527–988)	555 (436–693)
Solomon Islands	YLDs Number: Cumulative change (%)	54.8 (48.6–61.4)	61.0 (32.2–94.3)	62.6 (59.3–65.6)	69.7 (52.6–86.7)	62.9 (36.6–92.2)	25.3 (19.0–31.8)	102.8 (38.1–202.5)	-10.9 (-45.5 to 35.0)	39.0 (2.2–82.1)	5.5 (-4.3 to 13.9)	52.1 (41.6–65.4)
Solomon Islands	YLDs Rate: 1990	6,580.2 (5,006.9–8,412.8)	852.0 (592.1–1,168.7)	1,110.1 (776.2–1,528.4)	424.2 (281.1–573.3)	72.6 (50.8–99.8)	175.3 (126.9–232.6)	96.3 (62.5–147.9)	97.8 (62.1–140.4)	92.8 (62.2–127.1)	388.5 (275.7–525.4)	198.4 (154.6–247.0)
Solomon Islands	YLDs Rate: 2015	6,225.8 (4,766.8–7,916.9)	849.1 (590.2–1,154.4)	1,108.2 (776.4–1,522.3)	432.7 (287.1–596.8)	72.5 (51.0–97.7)	141.4 (102.7–186.6)	118.7 (70.9–187.0)	57.8 (32.7–88.3)	78.5 (53.0–109.8)	247.2 (176.6–330.8)	187.2 (147.0–233.4)

© 2017 Global Burden of Disease Child and Adolescent Health Collaboration. JAMA Pediatrics.

eTable 3. Prevalent Cases, Rates (per 100,000 population), Years Lived with Disability (YLDs), and Cumulative Percent Change with 95% Uncertainty Interval (UI) for the Top 10 Global Causes of YLDs in Children and Adolescents in 195 Countries and Territories, Aged 0 to 19 Years, Both Sexes, 1990 and 2015. *Note: best viewed by enlarging in browser.*

Location	Measure	All causes	Iron-deficiency anemia	Skin and subcutaneous diseases	Asthma	Hemoglobinopathies and hemolytic anemias	Diarrheal diseases	Congenital anomalies	Protein-energy malnutrition	Epilepsy	Malaria	Neonatal preterm birth complications
Solomon Islands	YLDs Rate: Cumulative change (%)	-5.3 (-8.8 to -1.8)	0.2 (-15.7 to 18.2)	-0.2 (-2.1 to 1.7)	2.0 (-8.2 to 12.4)	0.3 (-14.0 to 16.4)	-19.3 (-23.3 to -15.1)	25.5 (-14.5 to 87.2)	-39.3 (-62.9 to -8.0)	-14.1 (-37.2 to 12.7)	-36.3 (-41.8 to -31.8)	-5.6 (-12.0 to 2.6)
Tonga	Prevalence Number: 1990	45,106 (44,660–45,586)	12,363 (11,781–12,866)	20,082 (19,478–20,642)	4,737 (3,895–5,618)	7,908 (7,434–8,437)	361 (340–387)	739 (630–892)	110 (76–151)	111 (87–135)	0 (0–0)	864 (763–980)
Tonga	Prevalence Number: 2015	46,235 (45,829–46,615)	11,996 (11,341–12,962)	21,078 (20,497–21,672)	5,654 (4,656–6,723)	7,646 (7,198–8,165)	355 (334–379)	779 (659–934)	79 (51–111)	128 (99–159)	0 (0–0)	814 (720–933)
Tonga	Prevalence Number: Cumulative change (%)	2.5 (1.2–3.7)	-2.9 (-9.5 to 3.8)	5.0 (1.1–8.8)	19.6 (9.6–32.9)	-3.2 (-10.8 to 5.5)	-1.8 (-4.6 to 1.4)	6.3 (-16.1 to 31.6)	-25.0 (-56.9 to 18.3)	17.4 (-10.7 to 51.7)	3.6 (-9.0 to 15.3)	-5.4 (-18.8 to 9.9)
Tonga	Prevalence Rate: 1990	91,455.5 (90,601.5–92,394.7)	24,735.1 (23,622.1–25,677.5)	41,298.7 (40,072.4–42,412.8)	9,713.6 (7,997.0–11,494.9)	15,961.4 (15,003.5–17,033.8)	691.1 (652.6–736.7)	1,490.6 (1,269.7–1,798.2)	197.3 (137.3–270.8)	223.2 (176.3–273.4)	0.0 (0.0–0.0)	1,686.4 (1,484.3–1,919.2)
Tonga	Prevalence Rate: 2015	90,824.3 (90,038.1–91,570.4)	23,536.6 (22,248.8–25,433.4)	41,492.7 (40,348.9–42,656.0)	11,023.3 (9,095.9–13,092.5)	15,021.2 (14,140.9–16,038.9)	700.5 (659.9–747.0)	1,531.2 (1,295.8–1,837.6)	157.6 (103.0–222.0)	252.8 (194.6–313.1)	0.0 (0.0–0.0)	1,626.9 (1,440.8–1,859.7)
Tonga	Prevalence Rate: Cumulative change (%)	-0.7 (-1.9 to 0.4)	-4.8 (-11.2 to 1.9)	0.5 (-3.2 to 4.1)	13.7 (4.4–25.9)	-5.8 (-13.2 to 2.7)	1.4 (-1.5 to 4.6)	3.7 (-18.3 to 28.5)	-16.6 (-52.1 to 31.5)	14.6 (-12.9 to 48.5)	1.7 (-11.8 to 13.0)	-3.1 (-17.0 to 12.7)
Tonga	YLDs Number: 1990	3,142 (2,317–4,171)	455 (324–615)	582 (405–794)	214 (140–300)	37 (26–50)	59 (42–78)	49 (31–71)	13 (8–21)	42 (28–57)	0 (0–0)	95 (75–119)
Tonga	YLDs Number: 2015	2,955 (2,241–3,754)	449 (312–613)	600 (419–822)	256 (170–351)	37 (26–51)	58 (41–77)	56 (34–87)	9 (5–15)	33 (22–47)	0 (0–0)	86 (67–108)
Tonga	YLDs Number: Cumulative change (%)	-4.9 (-23.1 to 2.7)	-1.4 (-8.1 to 6.6)	3.1 (0.8–5.3)	20.0 (9.4–33.5)	0.5 (-6.1 to 8.5)	-1.5 (-6.9 to 4.6)	16.0 (-14.8 to 59.5)	-24.7 (-56.6 to 20.6)	-19.7 (-41.4 to 7.5)	3.7 (-11.5 to 17.7)	-9.3 (-15.9 to -2.5)
Tonga	YLDs Rate: 1990	6,459.6 (4,769.2–8,572.5)	917.0 (654.9–1,236.0)	1,175.5 (816.5–1,608.6)	439.7 (288.8–616.6)	76.5 (54.2–102.0)	112.7 (81.0–148.6)	98.9 (63.7–144.8)	24.6 (14.8–37.5)	85.3 (57.7–116.3)	0.0 (0.0–0.0)	192.0 (151.0–239.8)
Tonga	YLDs Rate: 2015	5,813.0 (4,407.6–7,383.8)	875.3 (609.9–1,194.8)	1,177.2 (820.3–1,613.7)	500.3 (332.2–686.6)	73.5 (52.0–99.7)	114.6 (82.5–152.7)	110.9 (68.7–171.6)	19.7 (11.3–30.9)	66.0 (44.3–93.9)	0.0 (0.0–0.0)	170.1 (133.0–213.2)
Tonga	YLDs Rate: Cumulative change (%)	-9.0 (-26.5 to -1.9)	-4.6 (-11.0 to 3.5)	0.2 (-1.9 to 2.1)	14.1 (4.3–26.8)	-4.0 (-10.3 to 3.9)	1.7 (-3.7 to 8.0)	13.3 (-16.6 to 55.9)	-16.3 (-51.8 to 34.1)	-21.6 (-42.7 to 4.9)	0.6 (-13.8 to 14.5)	-11.4 (-17.8 to -4.6)
Vanuatu	Prevalence Number: 1990	72,746 (72,133–73,346)	11,828 (10,298–13,783)	31,941 (30,819–32,935)	7,242 (6,039–8,538)	12,342 (11,718–12,996)	964 (904–1,032)	1,082 (900–1,322)	727 (522–989)	198 (155–241)	14,392 (10,998–18,194)	1,531 (1,357–1,774)
Vanuatu	Prevalence Number: 2015	115,142 (114,476–115,879)	14,084 (12,016–16,524)	49,384 (47,686–50,926)	11,558 (9,608–13,809)	17,282 (16,371–18,305)	1,323 (1,246–1,412)	1,708 (1,446–2,032)	706 (474–996)	341 (263–423)	17,098 (11,898–23,880)	2,219 (1,952–2,592)
Vanuatu	Prevalence Number: Cumulative change (%)	58.3 (56.8–59.9)	20.2 (-9.7 to 45.2)	54.7 (48.4–60.8)	59.7 (47.8–72.6)	40.1 (30.1–50.5)	37.3 (33.2–42.0)	59.8 (25.0–101.2)	1.0 (-41.1 to 55.7)	74.4 (32.9–127.6)	18.2 (-3.8 to 50.8)	45.6 (21.9–72.2)
Vanuatu	Prevalence Rate: 1990	91,728.4 (91,019.1–92,427.0)	13,456.5 (11,824.4–15,565.8)	41,322.0 (39,924.8–42,559.6)	9,264.4 (7,779.6–10,865.4)	15,434.1 (14,648.9–16,254.6)	1,127.2 (1,061.0–1,203.4)	1,352.3 (1,124.0–1,654.6)	778.9 (559.5–1,059.8)	248.6 (193.7–302.7)	17,766.0 (13,671.0–22,227.5)	1,775.2 (1,563.1–2,082.0)
Vanuatu	Prevalence Rate: 2015	95,627.8 (95,087.5–96,212.9)	11,368.6 (9,702.6–13,328.3)	41,566.8 (40,147.7–42,863.8)	9,546.9 (7,958.4–11,370.2)	14,325.2 (13,572.0–15,175.1)	1,077.0 (1,015.8–1,149.1)	1,416.3 (1,198.8–1,685.9)	567.2 (381.2–800.3)	283.4 (219.0–351.0)	14,043.5 (9,866.2–19,450.0)	1,803.8 (1,586.1–2,108.6)
Vanuatu	Prevalence Rate: Cumulative change (%)	4.3 (3.3–5.3)	-14.8 (-35.3 to 2.3)	0.6 (-3.3 to 4.6)	3.1 (-4.6 to 11.2)	-7.1 (-13.8 to -0.3)	-4.4 (-7.2 to -1.3)	6.0 (-17.3 to 33.2)	-24.2 (-55.8 to 16.7)	15.4 (-12.3 to 50.1)	-21.3 (-35.7 to 0.9)	2.2 (-15.3 to 21.5)
Vanuatu	YLDs Number: 1990	4,805 (3,641–6,120)	384 (253–556)	941 (655–1,299)	328 (218–450)	36 (25–49)	157 (113–206)	74 (48–113)	90 (53–138)	75 (49–103)	388 (265–526)	149 (116–185)
Vanuatu	YLDs Number: 2015	7,244 (5,479–9,248)	453 (294–676)	1,420 (986–1,947)	524 (346–728)	43 (30–61)	215 (155–283)	129 (79–202)	87 (51–135)	96 (63–134)	362 (256–487)	218 (171–272)
Vanuatu	YLDs Number: Cumulative change (%)	50.8 (43.6–57.0)	20.3 (-25.5 to 58.5)	50.8 (47.6–53.9)	59.6 (46.9–73.0)	22.1 (-2.1 to 45.0)	37.2 (30.5–44.1)	76.2 (23.8–150.4)	1.1 (-40.9 to 55.8)	29.0 (-7.6 to 70.2)	-6.1 (-15.2 to 6.5)	46.6 (35.3–59.1)
Vanuatu	YLDs Rate: 1990	6,149.2 (4,680.4–7,810.1)	434.4 (290.0–624.5)	1,184.0 (821.0–1,637.5)	420.1 (280.1–574.8)	43.4 (30.3–59.3)	183.7 (132.8–240.7)	93.2 (59.9–140.9)	96.9 (57.3–147.8)	95.1 (62.6–129.4)	476.4 (326.7–645.5)	184.7 (144.3–230.0)
Vanuatu	YLDs Rate: 2015	6,061.8 (4,591.4–7,719.6)	364.7 (237.6–544.7)	1,179.3 (819.1–1,621.6)	432.8 (287.4–600.3)	35.6 (24.5–49.5)	175.5 (126.1–230.3)	107.1 (65.8–167.9)	70.6 (41.6–108.5)	80.0 (52.6–111.5)	290.7 (205.7–390.4)	180.4 (141.5–224.6)
Vanuatu	YLDs Rate: Cumulative change (%)	-1.4 (-5.8 to 2.5)	-14.5 (-46.5 to 11.6)	-0.4 (-2.4 to 1.6)	3.1 (-4.9 to 11.5)	-17.6 (-33.2 to -2.2)	-4.4 (-9.0 to 0.2)	17.0 (-17.8 to 65.9)	-24.1 (-55.7 to 16.9)	-14.6 (-39.0 to 13.4)	-38.7 (-44.7 to -30.4)	-2.2 (-9.8 to 6.0)
North Africa and Middle East	Prevalence Number: 1990	159,882,213 (159,294,861–160,491,742)	44,452,470 (43,573,247–45,382,340)	47,720,249 (47,060,826–48,331,317)	13,971,043 (11,869,040–16,184,119)	38,983,149 (38,332,466–39,586,524)	2,525,364 (2,321,241–2,752,609)	1,966,112 (1,840,500–2,096,410)	1,675,033 (1,510,528–1,865,971)	1,035,154 (907,380–1,174,558)	1,417,451 (1,160,150–1,727,222)	1,386,876 (1,215,702–1,578,761)
North Africa and Middle East	Prevalence Number: 2015	203,284,245 (202,557,530–204,108,267)	54,678,673 (53,357,156–55,887,367)	63,541,624 (62,770,977–64,342,473)	15,382,502 (12,861,997–18,097,462)	47,859,607 (46,998,499–48,791,282)	2,159,395 (1,999,095–2,336,191)	2,804,537 (2,654,814–2,957,532)	2,057,860 (1,817,986–2,313,050)	1,161,561 (1,011,458–1,338,128)	2,712,071 (1,988,298–3,621,386)	1,604,151 (1,405,290–1,808,575)
North Africa and Middle East	Prevalence Number: Cumulative change (%)	27.1 (26.6–27.7)	23.0 (19.1–26.6)	33.2 (31.4–34.9)	10.1 (4.4–16.2)	22.8 (20.3–25.3)	-14.5 (-17.8 to -11.4)	42.8 (32.9–52.5)	23.4 (3.3–43.8)	12.3 (3.5–21.0)	90.2 (62.1–123.4)	15.8 (7.9–23.9)
North Africa and Middle East	Prevalence Rate: 1990	90,859.1 (90,538.0–91,189.0)	24,901.3 (24,442.4–25,386.8)	28,005.7 (27,620.6–28,354.5)	7,930.1 (6,754.9–9,148.9)	22,032.4 (21,665.6–22,373.5)	1,291.8 (1,188.5–1,405.8)	1,106.5 (1,035.6–1,180.5)	838.4 (756.0–934.0)	580.8 (511.8–660.8)	796.7 (656.5–959.1)	747.6 (654.9–854.8)
North Africa and Middle East	Prevalence Rate: 2015	90,320.0 (90,006.6–90,679.6)	24,033.2 (23,491.9–24,529.3)	28,677.5 (28,324.8–29,046.0)	6,882.0 (5,771.9–8,079.8)	21,205.1 (20,823.8–21,617.7)	901.5 (835.8–974.7)	1,240.5 (1,174.4–1,308.3)	843.2 (744.9–947.8)	513.7 (446.6–592.0)	1,200.7 (881.7–1,603.0)	689.9 (603.1–778.7)
North Africa and Middle East	Prevalence Rate: Cumulative change (%)	-0.6 (-1.0 to -0.2)	-3.5 (-6.2 to -0.9)	2.4 (1.0–3.8)	-13.3 (-17.5 to -8.7)	-3.7 (-5.7 to -1.8)	-30.2 (-32.9 to -27.8)	12.2 (4.4–20.0)	1.0 (-15.4 to 17.7)	-11.5 (-18.5 to -4.4)	49.8 (27.2–76.6)	-7.6 (-14.2 to -0.9)
North Africa and Middle East	YLDs Number: 1990	11,472,158 (8,741,385–14,534,436)	1,697,773 (1,224,053–2,267,541)	1,111,547 (764,949–1,536,430)	633,195 (420,382–873,065)	404,627 (292,474–540,028)	413,335 (299,417–550,499)	164,683 (110,578–228,592)	208,511 (143,124–286,508)	442,919 (317,613–570,293)	20,969 (14,747–28,343)	176,348 (136,240–224,354)
North Africa and Middle East	YLDs Number: 2015	13,596,683 (10,295,679–17,275,933)	2,051,891 (1,461,504–2,745,788)	1,462,893 (1,002,139–2,033,291)	698,541 (461,353–958,180)	499,378 (359,171–666,666)	353,666 (253,640–470,093)	265,386 (178,770–375,190)	256,463 (172,767–347,454)	377,985 (269,107–491,385)	42,659 (29,541–58,230)	183,712 (141,900–233,768)
North Africa and Middle East	YLDs Number: Cumulative change (%)	18.5 (16.3–20.8)	20.9 (15.6–25.7)	31.6 (30.2–32.9)	10.3 (4.6–16.3)	23.4 (19.8–26.9)	-14.4 (-17.8 to -11.2)	62.0 (36.9–90.6)	23.6 (3.9–43.5)	-14.6 (-22.7 to -6.3)	103.5 (83.1–123.5)	4.2 (-0.9 to 9.5)
North Africa and Middle East	YLDs Rate: 1990	6,622.4 (5,051.5–8,376.5)	944.2 (680.7–1,261.5)	638.5 (439.0–886.2)	359.3 (239.7–493.4)	226.2 (163.6–301.5)	211.4 (153.1–281.4)	92.4 (62.0–128.4)	104.4 (71.6–143.4)	248.3 (178.1–319.3)	11.7 (8.2–15.8)	98.7 (76.4–125.5)
North Africa and Middle East	YLDs Rate: 2015	6,086.5 (4,607.3–7,729.9)	902.5 (643.1–1,208.6)	653.4 (448.4–909.9)	312.5 (206.4–428.3)	221.3 (159.1–295.7)	147.6 (105.9–196.1)	117.3 (79.0–165.8)	105.1 (70.8–142.4)	167.1 (118.9–217.1)	18.9 (13.1–25.7)	81.1 (62.6–103.3)
North Africa and Middle East	YLDs Rate: Cumulative change (%)	-8.1 (-9.7 to -6.4)	-4.4 (-8.2 to -0.8)	2.3 (1.5–3.2)	-13.1 (-17.3 to -8.5)	-2.2 (-4.8 to 0.5)	-30.1 (-32.9 to -27.5)	27.5 (7.7–50.1)	1.2 (-15.0 to 17.5)	-32.7 (-39.0 to -26.1)	61.9 (45.7–77.4)	-17.9 (-21.9 to -13.6)
North Africa and Middle East	Prevalence Number: 1990	159,882,213 (159,294,861–160,491,742)	44,452,470 (43,573,247–45,382,340)	47,720,249 (47,060,826–48,331,317)	13,971,043 (11,869,040–16,184,119)	38,983,149 (38,332,466–39,586,524)	2,525,364 (2,321,241–2,752,609)	1,966,112 (1,840,500–2,096,410)	1,675,033 (1,510,528–1,865,971)	1,035,154 (907,380–1,174,558)	1,417,451 (1,160,150–1,727,222)	1,386,876 (1,215,702–1,578,761)
North Africa and Middle East	Prevalence Number: 2015	203,284,245 (202,557,530–204,108,267)	54,678,673 (53,357,156–55,887,367)	63,541,624 (62,770,977–64,342,473)	15,382,502 (12,861,997–18,097,462)	47,859,607 (46,998,499–48,791,282)	2,159,395 (1,999,095–2,336,191)	2,804,537 (2,654,814–2,957,532)	2,057,860 (1,817,986–2,313,050)	1,161,561 (1,011,458–1,338,128)	2,712,071 (1,988,298–3,621,386)	1,604,151 (1,405,290–1,808,575)
North Africa and Middle East	Prevalence Number: Cumulative change (%)	27.1 (26.6–27.7)	23.0 (19.1–26.6)	33.2 (31.4–34.9)	10.1 (4.4–16.2)	22.8 (20.3–25.3)	-14.5 (-17.8 to -11.4)	42.8 (32.9–52.5)	23.4 (3.3–43.8)	12.3 (3.5–21.0)	90.2 (62.1–123.4)	15.8 (7.9–23.9)
North Africa and Middle East	Prevalence Rate: 1990	90,859.1 (90,538.0–91,189.0)	24,901.3 (24,442.4–25,386.8)	28,005.7 (27,620.6–28,354.5)	7,930.1 (6,754.9–9,148.9)	22,032.4 (21,665.6–22,373.5)	1,291.8 (1,188.5–1,405.8)	1,106.5 (1,035.6–1,180.5)	838.4 (756.0–934.0)	580.8 (511.8–660.8)	796.7 (656.5–959.1)	747.6 (654.9–854.8)
North Africa and Middle East	Prevalence Rate: 2015	90,320.0 (90,006.6–90,679.6)	24,033.2 (23,491.9–24,529.3)	28,677.5 (28,324.8–29,046.0)	6,882.0 (5,771.9–8,079.8)	21,205.1 (20,823.8–21,617.7)	901.5 (835.8–974.7)	1,240.5 (1,174.4–1,308.3)	843.2 (744.9–947.8)	513.7 (446.6–592.0)	1,200.7 (881.7–1,603.0)	689.9 (603.1–778.7)
North Africa and Middle East	Prevalence Rate: Cumulative change (%)	-0.6 (-1.0 to -0.2)	-3.5 (-6.2 to -0.9)	2.4 (1.0–3.8)	-13.3 (-17.5 to -8.7)	-3.7 (-5.7 to -1.8)	-30.2 (-32.9 to -27.8)	12.2 (4.4–20.0)	1.0 (-15.4 to 17.7)	-11.5 (-18.5 to -4.4)	49.8 (27.2–76.6)	-7.6 (-14.2 to -0.9)
North Africa and Middle East	YLDs Number: 1990	11,472,158 (8,741,385–14,534,436)	1,697,773 (1,224,053–2,267,541)	1,111,547 (764,949–1,536,430)	633,195 (420,382–873,065)	404,627 (292,474–540,028)	413,335 (299,417–550,499)	164,683 (110,578–228,592)	208,511 (143,124–286,508)	442,919 (317,613–570,293)	20,969 (14,747–28,343)	176,348 (136,240–224,354)
North Africa and Middle East	YLDs Number: 2015	13,596,683 (10,295,679–17,275,933)	2,051,891 (1,461,504–2,745,788)	1,462,893 (1,002,139–2,033,291)	698,541 (461,353–958,180)	499,378 (359,171–666,666)	353,666 (253,640–470,093)	265,386 (178,770–375,190)	256,463 (172,767–347,454)	377,985 (269,107–491,385)	42,659 (29,541–58,230)	183,712 (141,900–233,768)
North Africa and Middle East	YLDs Number: Cumulative change (%)	18.5 (16.3–20.8)	20.9 (15.6–25.7)	31.6 (30.2–32.9)	10.3 (4.6–16.3)	23.4 (19.8–26.9)	-14.4 (-17.8 to -11.2)	62.0 (36.9–90.6)	23.6 (3.9–43.5)	-14.6 (-22.7 to -6.3)	103.5 (83.1–123.5)	4.2 (-0.9 to 9.5)
North Africa and Middle East	YLDs Rate: 1990	6,622.4 (5,051.5–8,376.5)	944.2 (680.7–1,261.5)	638.5 (439.0–886.2)	359.3 (239.7–493.4)	226.2 (163.6–301.5)	211.4 (153.1–281.4)	92.4 (62.0–128.4)	104.4 (71.6–143.4)	248.3 (178.1–319.3)	11.7 (8.2–15.8)	98.7 (76.4–125.5)
North Africa and Middle East	YLDs Rate: 2015	6,086.5 (4,607.3–7,729.9)	902.5 (643.1–1,208.6)	653.4 (448.4–909.9)	312.5 (206.4–428.3)	221.3 (159.1–295.7)	147.6 (105.9–196.1)	117.3 (79.0–165.8)	105.1 (70.8–142.4)	167.1 (118.9–217.1)	18.9 (13.1–25.7)	81.1 (62.6–103.3)
North Africa and Middle East	YLDs Rate: Cumulative change (%)	-8.1 (-9.7 to -6.4)	-4.4 (-8.2 to -0.8)	2.3 (1.5–3.2)	-13.1 (-17.3 to -8.5)	-2.2 (-4.8 to 0.5)	-30.1 (-32.9 to -27.5)	27.5 (7.7–50.1)	1.2 (-15.0 to 17.5)	-32.7 (-39.0 to -26.1)	61.9 (45.7–77.4)	-17.9 (-21.9 to -13.6)
Afghanistan	Prevalence Number: 1990	6,932,880 (6,877,584–6,990,145)	2,769,147 (2,530,432–2,975,573)	1,877,934 (1,800,499–1,954,815)	668,558 (556,991–794,018)	1,923,831 (1,680,275–2,189,221)	127,647 (114,683–140,475)	52,674 (43,338–64,616)	162,194 (107,406–230,280)	39,647 (32,003–48,178)	51,507 (27,977–80,648)	58,496 (45,962–73,300)

© 2017 Global Burden of Disease Child and Adolescent Health Collaboration. JAMA Pediatrics.

eTable 3. Prevalent Cases, Rates (per 100,000 population), Years Lived with Disability (YLDs), and Cumulative Percent Change with 95% Uncertainty Interval (UI) for the Top 10 Global Causes of YLDs in Children and Adolescents in 195 Countries and Territories, Aged 0 to 19 Years, Both Sexes, 1990 and 2015. *Note: best viewed by enlarging in browser.*

Location	Measure	All causes	Iron-deficiency anemia	Skin and subcutaneous diseases	Asthma	Hemoglobinopathies and hemolytic anemias	Diarrheal diseases	Congenital anomalies	Protein-energy malnutrition	Epilepsy	Malaria	Neonatal preterm birth complications
Afghanistan	Prevalence Number: 2015	17,196,560 (17,052,265–17,341,527)	6,074,080 (5,845,690–6,351,127)	4,972,069 (4,777,731–5,180,592)	1,560,067 (1,300,624–1,834,703)	4,479,724 (3,959,379–5,098,048)	198,294 (181,909–218,219)	163,233 (138,088–198,065)	198,162 (119,718–300,431)	99,296 (78,335–121,164)	101,203 (67,843–139,695)	129,574 (102,511–165,385)
Afghanistan	Prevalence Number: Cumulative change (%)	148.0 (145.4–150.9)	119.8 (104.9–135.9)	164.9 (150.9–180.4)	133.6 (118.5–151.7)	134.0 (97.1–174.1)	55.5 (46.1–68.5)	214.2 (141.1–300.5)	28.8 (-33.1 to 116.2)	152.3 (103.5–209.4)	103.1 (69.1–159.5)	123.6 (79.9–179.9)
Afghanistan	Prevalence Rate: 1990	95,568.9 (94,930.3–96,218.7)	37,762.3 (35,360.0–39,923.7)	27,478.8 (26,354.1–28,627.6)	9,617.2 (8,081.1–11,340.1)	26,387.2 (23,049.6–30,013.3)	1,373.6 (1,241.3–1,506.5)	695.7 (568.2–859.3)	1,654.1 (1,095.2–2,347.6)	545.5 (440.1–667.3)	688.9 (380.8–1,067.5)	670.9 (519.4–851.3)
Afghanistan	Prevalence Rate: 2015	94,409.4 (93,633.9–95,186.5)	33,201.3 (31,944.7–34,645.2)	27,827.9 (26,732.8–29,021.7)	8,536.9 (7,123.4–10,021.8)	24,572.0 (21,715.2–27,965.2)	1,045.5 (959.7–1,149.8)	892.0 (754.3–1,084.1)	1,037.6 (626.9–1,573.1)	545.3 (430.4–666.5)	551.9 (369.5–762.4)	693.3 (547.5–885.9)
Afghanistan	Prevalence Rate: Cumulative change (%)	-1.2 (-2.2 to -0.2)	-12.0 (-16.5 to -7.1)	1.3 (-4.1 to 7.1)	-11.2 (-16.7 to -4.9)	-6.4 (-21.2 to 9.6)	-23.8 (-28.2 to -17.8)	30.1 (-0.5 to 66.5)	-33.8 (-65.6 to 11.0)	0.7 (-18.9 to 23.2)	-17.5 (-30.4 to 3.4)	4.7 (-18.4 to 33.1)
Afghanistan	YLDs Number: 1990	579,802 (436,305–750,903)	112,473 (79,641–150,379)	46,909 (32,422–64,366)	30,020 (19,866–41,559)	17,274 (12,337–22,991)	20,726 (14,841–27,649)	3,882 (2,621–5,536)	20,115 (11,534–31,555)	17,139 (12,006–23,099)	1,362 (807–2,079)	5,241 (3,847–6,835)
Afghanistan	YLDs Number: 2015	1,273,555 (958,545–1,614,303)	239,972 (171,140–325,947)	120,294 (82,883–166,051)	70,525 (46,660–96,091)	40,550 (28,999–54,536)	32,351 (23,286–43,338)	13,007 (8,541–19,174)	24,619 (13,008–39,266)	42,740 (28,480–57,416)	3,181 (1,974–4,747)	12,200 (9,099–15,737)
Afghanistan	YLDs Number: Cumulative change (%)	120.1 (104.8–132.7)	113.9 (94.0–136.2)	156.3 (149.5–163.0)	135.1 (119.7–153.7)	135.4 (113.4–160.3)	56.3 (45.4–69.3)	237.3 (174.9–336.2)	29.3 (-33.1 to 116.8)	151.4 (96.6–213.1)	137.6 (91.9–199.6)	134.0 (104.7–165.0)
Afghanistan	YLDs Rate: 1990	8,205.6 (6,198.9–10,590.3)	1,533.0 (1,095.5–2,053.5)	660.7 (455.1–911.4)	431.5 (284.4–589.8)	236.0 (170.4–313.0)	222.9 (159.7–297.2)	51.2 (34.5–73.4)	205.2 (117.7–321.7)	235.3 (164.8–318.1)	18.4 (11.0–28.0)	67.7 (49.8–88.5)
Afghanistan	YLDs Rate: 2015	7,060.3 (5,317.7–8,952.6)	1,299.9 (926.9–1,764.8)	664.7 (458.9–920.2)	385.8 (255.6–525.6)	219.8 (157.5–296.1)	170.6 (122.8–228.2)	71.0 (46.7–104.4)	128.9 (68.1–205.6)	234.6 (157.0–315.3)	17.2 (10.7–25.8)	66.2 (49.3–85.4)
Afghanistan	YLDs Rate: Cumulative change (%)	-13.8 (-19.4 to -9.3)	-15.1 (-21.3 to -8.0)	0.6 (-1.8 to 3.0)	-10.5 (-16.2 to -3.6)	-6.7 (-14.1 to 1.1)	-23.4 (-28.6 to -17.1)	39.7 (13.4–80.7)	-33.6 (-65.6 to 11.3)	0.5 (-21.1 to 24.7)	-5.2 (-22.0 to 17.8)	-1.7 (-14.1 to 11.8)
Algeria	Prevalence Number: 1990	12,465,624 (12,273,037–12,651,406)	2,968,462 (2,691,567–3,272,181)	3,870,969 (3,715,524–4,020,258)	913,506 (778,399–1,072,253)	1,731,994 (1,649,411–1,813,325)	170,527 (155,007–186,826)	170,533 (145,292–203,887)	131,813 (92,932–179,109)	86,264 (69,527–104,046)	422 (55–834)	106,203 (90,324–127,590)
Algeria	Prevalence Number: 2015	12,428,678 (12,224,102–12,646,803)	2,810,531 (2,608,749–3,153,241)	3,908,256 (3,755,501–4,060,473)	920,561 (749,546–1,109,287)	1,615,036 (1,545,463–1,689,240)	136,237 (125,042–146,876)	180,985 (157,759–209,764)	74,544 (44,668–113,449)	69,564 (55,801–84,683)	1 (1–2)	107,511 (91,398–126,073)
Algeria	Prevalence Number: Cumulative change (%)	-0.3 (-2.3 to 1.8)	-5.0 (-16.7 to 9.1)	1.0 (-4.1 to 6.4)	0.8 (-9.7 to 11.5)	-6.7 (-12.2 to -0.9)	-20.0 (-24.7 to -15.9)	7.0 (-13.3 to 30.8)	-41.2 (-68.4 to -0.8)	-18.8 (-34.4 to -0.7)	91.2 (-99.7 to -96.7)	1.8 (-13.4 to 18.8)
Algeria	Prevalence Rate: 1990	88,342.4 (87,055.5–89,588.6)	20,829.8 (18,969.8–22,853.8)	28,075.9 (26,971.9–29,169.3)	6,467.1 (5,517.3–7,582.8)	12,229.6 (11,646.0–12,800.9)	1,128.2 (1,027.5–1,235.3)	1,200.7 (1,022.0–1,435.3)	858.7 (605.5–1,166.7)	606.8 (490.3–732.5)	3.0 (0.4–5.8)	732.6 (622.0–883.6)
Algeria	Prevalence Rate: 2015	87,684.3 (86,394.0–89,088.8)	19,428.9 (18,137.4–21,590.3)	28,546.4 (27,421.2–29,648.6)	6,648.5 (5,400.5–7,971.7)	11,312.1 (10,829.2–11,830.4)	825.0 (759.0–887.1)	1,262.1 (1,099.3–1,462.1)	431.1 (258.4–656.0)	486.6 (390.8–593.0)	0.0 (0.0–0.0)	705.9 (598.2–833.0)
Algeria	Prevalence Rate: Cumulative change (%)	-0.7 (-2.6 to 1.2)	-6.5 (-16.9 to 5.7)	1.7 (-3.4 to 7.0)	2.8 (-7.6 to 13.9)	-7.4 (-12.9 to -1.7)	-26.8 (-31.1 to -23.0)	6.0 (-14.1 to 29.3)	-47.8 (-72.0 to -12.0)	-19.2 (-35.2 to -1.3)	93.5 (-99.8 to -96.7)	-3.1 (-18.0 to 14.2)
Algeria	YLDs Number: 1990	810,920 (616,130–1,030,898)	108,822 (75,439–149,561)	88,385 (60,887–122,773)	41,558 (27,402–57,162)	24,209 (17,331–32,981)	27,997 (20,093–37,729)	14,990 (9,079–24,491)	16,467 (9,732–24,942)	36,514 (24,994–49,242)	9 (1–18)	14,539 (11,211–18,453)
Algeria	YLDs Number: 2015	735,243 (553,858–931,670)	100,418 (69,489–137,173)	89,478 (61,571–123,520)	41,947 (27,272–58,264)	22,549 (15,871–30,540)	22,429 (16,083–29,820)	21,218 (11,497–37,137)	9,308 (5,005–14,906)	19,812 (13,175–27,222)	0 (0–0)	12,555 (9,481–16,208)
Algeria	YLDs Number: Cumulative change (%)	-9.3 (-12.6 to -5.7)	-7.4 (-22.4 to 12.4)	1.2 (-1.0 to 3.6)	1.0 (-9.7 to 12.1)	-6.7 (-15.9 to 7.6)	-19.8 (-25.5 to -14.3)	47.7 (-21.1 to 148.7)	-41.1 (-68.3 to -1.1)	-45.4 (-57.8 to -30.7)	3.3 (-99.7 to -96.6)	-13.6 (-21.2 to -5.1)
Algeria	YLDs Rate: 1990	5,835.1 (4,434.5–7,415.4)	757.5 (524.1–1,048.0)	630.6 (434.3–876.1)	294.1 (193.8–404.3)	168.5 (120.8–229.2)	185.2 (133.1–249.6)	105.4 (63.6–172.3)	107.3 (63.4–162.5)	256.7 (175.7–346.0)	0.1 (0.0–0.1)	102.1 (78.6–129.7)
Algeria	YLDs Rate: 2015	5,338.3 (4,021.8–6,757.6)	703.1 (487.4–959.4)	637.5 (441.0–884.5)	302.8 (197.8–418.2)	160.2 (113.3–217.6)	135.8 (97.1–180.3)	147.7 (80.1–258.4)	53.8 (29.0–86.2)	138.4 (92.4–190.2)	0.0 (0.0–0.0)	87.4 (66.3–112.6)
Algeria	YLDs Rate: Cumulative change (%)	-8.5 (-11.5 to -5.2)	-6.8 (-20.7 to 10.7)	1.1 (-1.1 to 3.5)	3.0 (-8.0 to 14.3)	-4.8 (-13.5 to 8.2)	-26.6 (-31.7 to -21.6)	46.2 (-22.2 to 146.8)	-47.7 (-71.8 to -12.2)	-45.7 (-58.1 to -31.1)	4.7 (-99.8 to -96.7)	-14.3 (-22.1 to -6.0)
Bahrain	Prevalence Number: 1990	187,449 (185,527–188,999)	46,226 (43,078–50,232)	56,175 (53,917–58,238)	13,255 (10,811–15,622)	104,536 (100,936–108,494)	1,671 (1,556–1,804)	3,092 (2,638–3,666)	2,035 (1,524–2,649)	1,216 (987–1,453)	0 (0–0)	1,618 (1,368–1,919)
Bahrain	Prevalence Number: 2015	343,986 (340,276–347,004)	82,672 (77,902–87,329)	111,331 (107,122–115,202)	24,172 (19,683–28,795)	174,069 (167,746–180,561)	2,026 (1,908–2,146)	5,641 (4,897–6,595)	2,040 (1,222–3,157)	1,888 (1,500–2,340)	0 (0–0)	2,386 (2,031–2,774)
Bahrain	Prevalence Number: Cumulative change (%)	83.5 (80.9–85.9)	79.2 (64.1–96.3)	98.3 (88.6–108.4)	82.9 (60.3–110.5)	66.6 (57.9–75.0)	21.4 (15.9–27.3)	84.2 (47.3–127.4)	3.1 (-44.5 to 65.7)	56.3 (23.0–91.4)	65.3 (25.8–95.0)	48.2 (26.7–72.3)
Bahrain	Prevalence Rate: 1990	92,685.5 (91,835.8–93,342.7)	22,903.3 (21,655.6–24,416.9)	29,251.3 (28,071.9–30,320.7)	6,785.4 (5,553.4–7,980.1)	51,361.9 (49,611.7–53,312.3)	667.8 (624.2–717.9)	1,514.5 (1,293.5–1,806.5)	767.4 (574.5–998.7)	603.1 (489.4–721.3)	0.0 (0.0–0.0)	749.5 (628.1–896.7)
Bahrain	Prevalence Rate: 2015	91,670.4 (90,681.5–92,466.3)	22,104.6 (20,830.7–23,359.3)	29,811.7 (28,700.8–30,854.1)	6,421.3 (5,235.5–7,648.9)	46,389.1 (44,703.9–48,115.2)	538.6 (507.3–570.3)	1,503.3 (1,304.2–1,758.2)	543.9 (325.8–841.7)	503.3 (399.8–622.8)	0.0 (0.0–0.0)	638.0 (543.3–741.4)
Bahrain	Prevalence Rate: Cumulative change (%)	-1.1 (-2.4 to 0.1)	-3.4 (-10.3 to 4.9)	2.0 (-3.0 to 7.1)	-5.2 (-16.5 to 9.1)	-9.6 (-14.4 to -5.1)	-19.3 (-22.7 to -15.5)	0.2 (-20.0 to 23.5)	-27.1 (-60.7 to 17.2)	-16.0 (-34.0 to 3.2)	-3.9 (-23.4 to 10.0)	-14.4 (-27.5 to -0.2)
Bahrain	YLDs Number: 1990	11,603 (8,819–14,636)	1,753 (1,236–2,367)	1,359 (933–1,874)	602 (390–836)	457 (324–617)	275 (195–363)	240 (156–339)	255 (161–374)	528 (366–707)	0 (0–0)	234 (180–300)
Bahrain	YLDs Number: 2015	20,442 (15,495–26,003)	3,169 (2,242–4,324)	2,595 (1,769–3,619)	1,100 (727–1,522)	762 (542–1,030)	333 (239–444)	645 (393–937)	256 (133–422)	540 (361–739)	0 (0–0)	295 (224–378)
Bahrain	YLDs Number: Cumulative change (%)	76.2 (71.0–82.4)	81.1 (62.2–102.9)	90.7 (85.2–96.1)	83.1 (60.4–110.9)	67.0 (45.9–88.7)	21.5 (13.4–30.0)	169.3 (111.8–235.5)	3.3 (-44.0 to 65.7)	3.1 (-22.2 to 32.0)	66.8 (26.9–96.8)	26.4 (15.8–38.0)
Bahrain	YLDs Rate: 1990	6,004.4 (4,571.9–7,561.7)	878.1 (625.4–1,184.2)	684.4 (468.0–955.9)	308.3 (199.7–423.6)	226.9 (162.1–306.4)	109.8 (78.2–145.0)	117.3 (76.0–166.0)	96.2 (60.8–141.0)	261.4 (180.7–350.7)	0.0 (0.0–0.0)	115.3 (88.7–147.9)
Bahrain	YLDs Rate: 2015	5,465.5 (4,140.8–6,950.5)	848.4 (601.3–1,156.6)	692.7 (472.5–966.1)	292.2 (193.6–405.2)	203.1 (144.5–274.7)	88.7 (63.7–118.1)	171.9 (104.9–249.6)	68.3 (35.7–112.7)	144.0 (96.3–197.1)	0.0 (0.0–0.0)	78.7 (59.9–100.7)
Bahrain	YLDs Rate: Cumulative change (%)	-9.0 (-11.4 to -6.0)	-3.3 (-11.4 to 7.3)	1.2 (-1.1 to 3.5)	-5.0 (-16.2 to 9.3)	-10.4 (-20.5 to 0.3)	-19.1 (-24.4 to -13.6)	47.1 (16.0–83.3)	-27.0 (-60.4 to 17.2)	-44.4 (-58.0 to -28.9)	-6.0 (-26.6 to 9.4)	-31.6 (-37.5 to -25.3)
Egypt	Prevalence Number: 1990	25,800,841 (25,521,242–26,063,984)	8,709,231 (8,330,046–9,188,575)	7,468,904 (7,257,535–7,692,274)	2,421,555 (1,998,694–2,875,151)	6,860,420 (6,627,901–7,094,532)	531,361 (483,997–573,899)	274,721 (234,536–331,384)	167,257 (124,951–221,957)	124,204 (101,282–146,899)	128 (36–236)	218,777 (183,713–264,410)
Egypt	Prevalence Number: 2015	34,124,171 (33,665,883–34,527,242)	11,855,191 (10,920,996–12,784,765)	10,358,289 (10,068,835–10,650,034)	2,434,073 (2,019,502–2,902,042)	8,441,643 (8,165,381–8,741,700)	394,916 (358,600–432,480)	442,367 (383,505–521,749)	405,696 (273,352–554,240)	203,556 (164,593–243,789)	0 (0–0)	267,213 (228,816–315,931)
Egypt	Prevalence Number: Cumulative change (%)	32.3 (30.1–34.4)	36.3 (20.0–47.8)	38.7 (33.4–43.6)	0.6 (-6.7 to 8.5)	23.1 (17.5–28.8)	-25.6 (-32.5 to -18.4)	62.5 (30.8–97.1)	150.3 (51.7–278.7)	65.2 (32.4–100.9)	-99.3 (-100.0 to -99.8)	23.0 (2.4–44.8)
Egypt	Prevalence Rate: 1990	91,790.4 (90,912.9–92,643.7)	31,409.1 (30,148.8–32,722.1)	27,553.6 (26,825.2–28,348.5)	8,564.4 (7,076.2–10,153.6)	24,314.8 (23,482.9–25,134.3)	1,636.7 (1,493.7–1,764.6)	963.9 (824.5–1,162.2)	502.0 (375.0–666.2)	438.0 (356.7–519.7)	0.4 (0.1–0.8)	724.3 (604.7–881.4)
Egypt	Prevalence Rate: 2015	90,664.9 (89,548.2–91,649.3)	31,265.3 (29,182.7–33,292.8)	28,209.1 (27,419.1–29,006.2)	6,608.4 (5,485.2–7,858.5)	22,333.6 (21,599.9–23,126.9)	924.5 (843.1–1,007.5)	1,165.4 (1,009.6–1,374.8)	908.5 (612.1–1,241.2)	536.3 (434.4–641.3)	0.0 (0.0–0.0)	660.3 (561.9–783.1)
Egypt	Prevalence Rate: Cumulative change (%)	-1.2 (-2.7 to 0.2)	-0.4 (-10.0 to 6.6)	2.4 (-1.6 to 5.8)	-22.8 (-28.2 to -16.9)	-8.1 (-12.4 to -3.9)	-43.4 (-48.5 to -38.1)	22.0 (-1.7 to 48.0)	86.7 (13.2–182.5)	23.4 (-1.1 to 50.1)	-99.5 (-100.0 to -99.8)	-8.1 (-24.6 to 9.0)
Egypt	YLDs Number: 1990	1,848,269 (1,399,022–2,345,509)	349,360 (250,770–470,031)	178,807 (123,924–245,148)	109,665 (72,693–152,508)	50,354 (36,168–68,325)	87,144 (63,011–115,996)	25,288 (15,922–38,316)	20,884 (12,824–31,026)	53,906 (37,272–71,507)	2 (0–5)	28,478 (21,837–36,040)
Egypt	YLDs Number: 2015	2,220,340 (1,674,542–2,833,395)	459,586 (319,384–620,078)	243,453 (167,486–335,745)	110,473 (73,623–152,425)	65,243 (45,777–88,658)	64,671 (46,326–86,495)	30,946 (20,602–43,668)	50,661 (29,477–78,145)	59,540 (40,201–80,764)	0 (0–0)	31,813 (24,634–40,368)
Egypt	YLDs Number: Cumulative change (%)	20.1 (14.4–25.1)	31.7 (11.3–46.8)	36.1 (33.0–39.1)	0.8 (-6.8 to 9.0)	29.8 (9.2–45.7)	-25.7 (-33.0 to -17.8)	25.6 (-11.1 to 66.8)	150.2 (50.3–277.3)	11.5 (-14.8 to 41.4)	-99.2 (-100.0 to -99.6)	12.0 (0.1–25.5)
Egypt	YLDs Rate: 1990	6,727.7 (5,092.1–8,544.5)	1,250.1 (898.5–1,685.2)	642.0 (443.2–888.2)	387.6 (256.2–537.0)	178.7 (127.8–241.8)	268.3 (194.0–356.7)	88.4 (55.8–133.8)	62.7 (38.5–93.1)	189.8 (131.6–250.7)	0.0 (0.0–0.0)	98.7 (75.7–125.6)
Egypt	YLDs Rate: 2015	5,997.8 (4,536.4–7,630.5)	1,214.5 (852.7–1,637.0)	651.4 (447.3–903.3)	299.8 (199.4–415.1)	172.3 (121.0–232.9)	151.3 (108.8–201.7)	81.4 (54.2–114.7)	113.5 (66.0–175.0)	156.8 (106.1–213.4)	0.0 (0.0–0.0)	83.3 (64.5–105.8)
Egypt	YLDs Rate: Cumulative change (%)	-10.9 (-14.6 to -7.7)	-2.8 (-15.0 to 6.6)	1.5 (-0.7 to 3.5)	-22.6 (-28.4 to -16.3)	-3.4 (-16.1 to 6.8)	-43.5 (-48.9 to -37.7)	-5.5 (-33.3 to 24.9)	86.7 (12.2–181.5)	-16.6 (-36.0 to 5.7)	-99.4 (-100.0 to -99.7)	-15.4 (-24.7 to -5.2)
Iran	Prevalence Number: 1990	27,825,952 (27,541,170–28,076,783)	7,087,180 (6,615,759–7,458,248)	8,120,129 (7,803,313–8,446,937)	2,164,276 (1,831,454–2,538,861)	6,258,044 (6,059,512–6,465,818)	341,884 (308,875–381,008)	374,521 (319,140–447,901)	325,759 (215,456–461,560)	221,790 (178,659–265,462)	3,770 (2,767–4,809)	239,094 (201,536–291,383)
Iran	Prevalence Number: 2015	21,238,454 (21,018,852–21,425,731)	3,753,891 (3,498,124–4,050,004)	6,676,696 (6,435,380–6,944,089)	1,334,585 (1,130,023–1,571,826)	4,476,576 (4,320,609–4,627,262)	168,317 (152,291–186,342)	331,806 (288,530–383,400)	125,795 (77,286–188,761)	159,123 (128,503–192,424)	500 (263–712)	153,535 (130,962–181,066)
Iran	Prevalence Number: Cumulative change (%)	-23.7 (-24.6 to -22.7)	-47.0 (-51.4 to -40.7)	-17.7 (-21.7 to -13.7)	-38.2 (-44.7 to -30.9)	-28.4 (-31.9 to -24.8)	-50.7 (-55.2 to -46.8)	-10.5 (-28.7 to 10.4)	-59.2 (-78.4 to -30.3)	-27.6 (-42.6 to -10.9)	-86.5 (-92.7 to -79.8)	-35.3 (-46.3 to -22.8)

© 2017 Global Burden of Disease Child and Adolescent Health Collaboration. JAMA Pediatrics.

eTable 3. Prevalent Cases, Rates (per 100,000 population), Years Lived with Disability (YLDs), and Cumulative Percent Change with 95% Uncertainty Interval (UI) for the Top 10 Global Causes of YLDs in Children and Adolescents in 195 Countries and Territories, Aged 0 to 19 Years, Both Sexes, 1990 and 2015. *Note: best viewed by enlarging in browser.*

Location	Measure	All causes	Iron-deficiency anemia	Skin and subcutaneous diseases	Asthma	Hemoglobinopathies and hemolytic anemias	Diarrheal diseases	Congenital anomalies	Protein-energy malnutrition	Epilepsy	Malaria	Neonatal preterm birth complications
Iran	Prevalence Rate: 1990	90,053.9 (89,201.1–90,828.0)	22,529.0 (21,178.0–23,609.9)	27,412.3 (26,389.8–28,538.7)	6,953.6 (5,941.6–8,094.6)	20,161.1 (19,517.5–20,831.8)	1,005.9 (911.2–1,118.3)	1,201.7 (1,024.0–1,439.3)	941.4 (622.8–1,333.8)	694.4 (561.5–830.8)	11.9 (8.7–15.1)	749.4 (629.2–914.3)
Iran	Prevalence Rate: 2015	88,457.1 (87,605.9–89,234.3)	15,456.3 (14,440.9–16,624.8)	28,242.0 (27,210.6–29,377.7)	5,588.7 (4,729.2–6,558.6)	18,593.3 (17,943.2–19,220.4)	665.6 (603.0–736.0)	1,376.7 (1,196.7–1,590.6)	489.3 (300.7–734.3)	656.6 (531.9–794.4)	2.0 (1.1–2.9)	628.4 (535.4–742.0)
Iran	Prevalence Rate: Cumulative change (%)	-1.8 (-3.0 to -0.6)	-31.3 (-36.7 to -23.7)	3.1 (-1.7 to 8.1)	-19.5 (-27.3 to -10.8)	-7.7 (-12.2 to -3.1)	-33.7 (-39.7 to -28.5)	15.8 (-7.8 to 42.6)	-45.0 (-70.9 to -6.2)	-4.6 (-24.1 to 17.5)	-82.8 (-90.7 to -74.1)	-15.5 (-30.2 to 1.2)
Iran	YLDs Number: 1990	2,080,019 (1,581,212–2,634,101)	264,642 (186,976–358,853)	175,974 (119,975–245,752)	98,220 (64,159–135,220)	78,702 (56,602–105,383)	55,874 (40,362–74,787)	34,791 (21,002–53,912)	40,500 (23,064–64,752)	93,677 (62,925–122,260)	92 (54–140)	32,472 (24,917–41,775)
Iran	YLDs Number: 2015	1,289,764 (973,339–1,630,287)	124,777 (88,581–174,186)	141,584 (96,224–199,512)	60,779 (40,515–83,873)	63,685 (45,608–86,007)	27,723 (19,658–36,865)	32,582 (19,528–52,221)	15,746 (8,162–25,849)	45,221 (30,746–61,139)	18 (8–30)	18,365 (13,882–23,722)
Iran	YLDs Number: Cumulative change (%)	-38.0 (-41.0 to -35.2)	-52.7 (-58.7 to -44.6)	-19.6 (-21.7 to -17.7)	-38.0 (-44.6 to -30.6)	-19.0 (-24.8 to -11.1)	-50.3 (-55.2 to -45.3)	-2.5 (-42.5 to 48.5)	-58.9 (-78.5 to -30.3)	-51.2 (-63.1 to -37.5)	-79.2 (-88.6 to -68.8)	-43.4 (-50.2 to -36.8)
Iran	YLDs Rate: 1990	6,848.2 (5,213.7–8,652.5)	829.6 (587.6–1,123.3)	580.1 (394.1–816.8)	315.4 (207.6–431.8)	247.2 (177.5–330.7)	164.4 (118.5–220.1)	111.3 (67.2–173.3)	117.0 (66.7–187.1)	293.0 (197.6–381.9)	0.3 (0.2–0.4)	103.7 (79.7–133.0)
Iran	YLDs Rate: 2015	5,428.2 (4,098.4–6,863.3)	517.0 (367.4–721.7)	593.4 (402.4–838.8)	254.5 (169.9–350.6)	266.1 (190.6–359.0)	109.6 (77.9–145.5)	135.0 (80.8–215.9)	61.3 (31.7–100.6)	186.5 (127.1–251.1)	0.1 (0.0–0.1)	76.2 (57.6–98.4)
Iran	YLDs Rate: Cumulative change (%)	-20.7 (-24.5 to -17.3)	-37.6 (-44.9 to -27.6)	2.3 (-0.1 to 4.5)	-19.2 (-27.5 to -10.4)	7.7 (0.4–17.6)	-33.2 (-39.7 to -26.6)	26.2 (-25.2 to 91.4)	-44.7 (-71.1 to -6.2)	-35.7 (-51.4 to -17.8)	-73.2 (-85.5 to -59.5)	-26.4 (-35.1 to -17.8)
Iraq	Prevalence Number: 1990	8,980,992 (8,867,969–9,080,018)	2,385,655 (2,218,478–2,549,232)	2,544,609 (2,468,559–2,628,684)	645,843 (537,366–760,735)	2,508,331 (2,422,024–2,601,386)	118,577 (107,702–130,680)	117,910 (102,160–139,260)	56,583 (45,914–68,849)	43,516 (35,060–52,019)	175 (121–231)	83,275 (70,631–98,398)
Iraq	Prevalence Number: 2015	16,651,363 (16,436,637–16,843,937)	4,223,383 (3,946,748–4,575,461)	4,809,257 (4,677,236–4,942,785)	1,125,283 (928,922–1,334,415)	4,331,336 (4,177,465–4,496,762)	182,267 (162,707–200,765)	237,745 (204,511–281,268)	152,570 (110,155–203,385)	76,732 (60,639–95,022)	0 (0–0)	149,808 (127,985–175,516)
Iraq	Prevalence Number: Cumulative change (%)	85.4 (82.5–88.4)	77.2 (65.4–92.2)	89.1 (81.6–96.6)	74.3 (62.7–86.4)	72.7 (64.6–80.8)	53.8 (45.1–62.7)	103.2 (63.0–149.2)	173.7 (82.7–281.4)	77.4 (43.7–114.9)	-100.0 (-100.0 to -100.0)	81.0 (50.7–112.8)
Iraq	Prevalence Rate: 1990	89,932.0 (88,910.1–90,867.6)	23,265.2 (21,783.6–24,699.5)	26,251.3 (25,438.6–27,126.8)	6,524.0 (5,443.8–7,684.7)	24,986.6 (24,129.1–25,918.6)	1,069.8 (972.4–1,177.7)	1,169.3 (1,012.9–1,381.8)	497.7 (403.9–605.6)	431.9 (349.5–515.6)	1.7 (1.2–2.3)	787.9 (663.9–937.5)
Iraq	Prevalence Rate: 2015	89,422.2 (88,377.2–90,355.2)	21,887.9 (20,588.6–23,482.3)	26,759.9 (26,014.9–27,523.8)	6,140.1 (5,082.8–7,267.3)	23,113.6 (22,293.9–24,001.7)	868.4 (778.4–953.9)	1,264.8 (1,087.3–1,496.4)	701.3 (506.4–934.9)	409.7 (325.1–508.3)	0.0 (0.0–0.0)	752.5 (640.3–886.2)
Iraq	Prevalence Rate: Cumulative change (%)	-0.6 (-2.0 to 0.9)	-5.8 (-11.6 to 1.6)	1.9 (-2.1 to 6.1)	-5.8 (-12.1 to 0.6)	-7.5 (-11.8 to -3.2)	-18.8 (-23.4 to -14.2)	8.2 (-12.9 to 33.7)	40.9 (-4.5 to 99.3)	-4.6 (-22.8 to 15.5)	-100.0 (-100.0 to -100.0)	-4.5 (-20.8 to 14.4)
Iraq	YLDs Number: 1990	671,778 (517,885–842,337)	86,253 (60,907–117,295)	56,701 (39,002–78,709)	29,179 (19,516–40,096)	26,945 (19,193–36,183)	19,422 (13,867–25,988)	11,454 (6,413–19,539)	7,051 (4,673–9,916)	18,618 (12,574–24,794)	3 (2–5)	11,446 (8,771–14,603)
Iraq	YLDs Number: 2015	1,114,381 (849,549–1,419,734)	151,603 (107,099–205,817)	107,159 (73,981–149,683)	51,169 (33,690–69,904)	47,625 (34,170–64,423)	29,934 (21,469–40,028)	28,320 (15,487–49,964)	19,093 (11,534–28,902)	27,936 (18,822–38,542)	0 (0–0)	18,697 (14,305–23,964)
Iraq	YLDs Number: Cumulative change (%)	65.9 (53.8–77.2)	76.0 (62.1–93.5)	89.0 (85.0–93.1)	75.4 (62.5–88.8)	76.7 (67.7–88.3)	54.2 (43.4–65.3)	160.6 (35.9–344.3)	175.0 (80.5–286.8)	51.3 (17.3–89.3)	-100.0 (-100.0 to -100.0)	63.5 (48.3–78.5)
Iraq	YLDs Rate: 1990	6,818.6 (5,277.0–8,543.8)	839.0 (594.0–1,136.7)	574.5 (393.8–801.6)	299.7 (197.8–404.8)	267.4 (190.6–359.0)	175.2 (125.2–234.7)	113.3 (63.4–192.6)	62.0 (41.1–87.2)	184.6 (124.6–245.3)	0.0 (0.0–0.1)	113.3 (86.9–144.3)
Iraq	YLDs Rate: 2015	6,086.8 (4,640.9–7,733.8)	787.3 (558.2–1,061.9)	583.6 (400.3–818.9)	279.1 (184.7–381.3)	254.1 (182.4–342.7)	142.6 (102.4–190.6)	150.3 (82.3–265.4)	87.8 (53.0–132.9)	149.0 (100.1–205.1)	0.0 (0.0–0.0)	99.2 (75.9–126.8)
Iraq	YLDs Rate: Cumulative change (%)	-10.7 (-17.1 to -5.0)	-6.1 (-12.7 to 2.2)	1.6 (-0.6 to 3.9)	-5.3 (-12.2 to 1.7)	-5.0 (-9.7 to 0.7)	-18.6 (-24.2 to -12.8)	39.7 (-27.2 to 137.6)	43.7 (-5.7 to 102.2)	-18.6 (-36.8 to 2.4)	-100.0 (-100.0 to -100.0)	-12.3 (-20.3 to -4.3)
Jordan	Prevalence Number: 1990	1,741,133 (1,723,919–1,756,407)	363,573 (338,488–387,157)	551,994 (530,902–572,720)	152,443 (126,740–181,574)	503,168 (466,063–551,291)	20,041 (18,223–21,938)	27,918 (24,057–32,881)	9,550 (6,132–13,841)	8,171 (6,562–9,940)	0 (0–0)	16,846 (14,263–19,698)
Jordan	Prevalence Number: 2015	3,085,108 (3,052,330–3,112,093)	704,053 (677,581–747,032)	1,006,600 (967,035–1,044,294)	269,665 (218,841–321,459)	818,407 (751,661–910,931)	27,050 (24,769–29,643)	50,691 (44,176–59,538)	12,138 (7,611–18,003)	13,192 (10,480–16,343)	0 (0–0)	26,394 (22,566–30,987)
Jordan	Prevalence Number: Cumulative change (%)	77.2 (75.0–79.3)	93.9 (80.2–108.4)	82.4 (73.3–91.6)	77.1 (61.0–93.6)	63.0 (44.2–83.3)	35.1 (26.0–43.6)	83.1 (48.1–122.8)	35.1 (-31.6 to 128.6)	62.7 (27.7–100.2)	67.6 (33.4–92.3)	57.5 (33.4–83.3)
Jordan	Prevalence Rate: 1990	90,093.9 (89,231.2–90,852.0)	18,683.8 (17,378.3–19,883.5)	29,147.6 (28,010.4–30,245.5)	7,863.1 (6,553.0–9,326.4)	25,955.2 (24,044.5–28,436.2)	993.0 (903.3–1,088.2)	1,438.3 (1,239.6–1,693.8)	470.7 (302.2–682.2)	422.1 (339.0–512.9)	0.0 (0.0–0.0)	845.1 (712.8–990.0)
Jordan	Prevalence Rate: 2015	90,185.6 (89,244.5–90,966.1)	20,460.5 (19,710.6–21,656.2)	29,816.2 (28,649.3–30,916.9)	7,900.8 (6,416.9–9,424.6)	23,858.6 (21,908.7–26,551.5)	752.7 (690.5–824.4)	1,476.5 (1,286.8–1,735.0)	334.0 (209.4–495.4)	384.5 (304.5–477.2)	0.0 (0.0–0.0)	753.1 (643.1–886.3)
Jordan	Prevalence Rate: Cumulative change (%)	0.1 (-1.1 to 1.3)	9.7 (2.2–17.9)	2.3 (-2.8 to 7.6)	0.6 (-8.5 to 10.0)	-7.9 (-18.5 to 3.6)	-24.1 (-29.3 to -19.4)	3.5 (-16.3 to 25.9)	-24.6 (-61.8 to 27.7)	-8.2 (-27.9 to 13.1)	-3.9 (-23.4 to 10.1)	-10.4 (-24.4 to 4.7)
Jordan	YLDs Number: 1990	111,237 (84,716–140,732)	13,293 (9,431–17,917)	13,816 (9,584–18,806)	6,929 (4,614–9,700)	4,209 (3,015–5,662)	3,292 (2,361–4,429)	2,462 (1,526–4,022)	1,195 (685–1,950)	3,450 (2,400–4,603)	0 (0–0)	2,230 (1,697–2,842)
Jordan	YLDs Number: 2015	183,137 (139,194–232,642)	25,770 (18,260–35,014)	24,906 (17,218–34,209)	12,266 (8,119–17,009)	6,512 (4,614–8,732)	4,456 (3,149–5,959)	4,834 (2,940–7,536)	1,524 (812–2,411)	3,752 (2,535–5,250)	0 (0–0)	3,070 (2,325–3,991)
Jordan	YLDs Number: Cumulative change (%)	64.6 (59.8–69.4)	94.1 (77.3–113.2)	80.2 (76.2–84.4)	77.2 (60.2–94.6)	54.8 (45.1–64.8)	35.3 (25.7–45.3)	105.0 (22.1–217.1)	35.4 (-30.9 to 126.8)	9.8 (-18.3 to 40.8)	66.9 (30.5–94.4)	37.7 (25.9–50.7)
Jordan	YLDs Rate: 1990	5,836.9 (4,448.4–7,385.4)	678.1 (481.1–913.0)	719.8 (499.0–983.0)	357.3 (237.2–498.4)	213.3 (152.7–286.8)	163.4 (117.0–219.3)	126.7 (78.6–207.1)	58.9 (33.8–96.1)	178.2 (123.7–237.5)	0.0 (0.0–0.0)	114.7 (87.4–146.5)
Jordan	YLDs Rate: 2015	5,409.8 (4,113.0–6,859.7)	748.2 (529.9–1,014.5)	730.7 (505.6–1,008.3)	359.3 (239.1–497.2)	188.8 (133.9–252.9)	124.0 (87.6–165.6)	140.7 (85.5–219.4)	42.0 (22.4–66.4)	109.3 (73.4–152.8)	0.0 (0.0–0.0)	89.4 (67.8–116.0)
Jordan	YLDs Rate: Cumulative change (%)	-7.3 (-10.0 to -4.7)	10.5 (1.2–21.2)	1.5 (-0.8 to 3.8)	0.7 (-8.8 to 10.6)	-11.4 (-16.8 to -5.5)	-24.0 (-29.4 to -18.3)	15.9 (-31.0 to 79.3)	-24.4 (-61.4 to 26.6)	-38.1 (-53.9 to -20.8)	-3.3 (-24.7 to 12.6)	-22.0 (-28.8 to -14.7)
Kuwait	Prevalence Number: 1990	795,457 (787,227–802,549)	204,816 (196,355–215,482)	272,358 (262,859–281,503)	79,332 (66,297–93,170)	143,941 (134,247–153,324)	3,971 (3,759–4,192)	14,640 (12,822–16,824)	3,609 (2,260–5,274)	4,743 (3,785–5,772)	0 (0–0)	5,104 (4,311–5,994)
Kuwait	Prevalence Number: 2015	927,825 (915,668–938,365)	237,843 (221,025–254,703)	316,548 (305,504–328,402)	84,132 (68,839–100,829)	147,665 (138,075–157,356)	5,491 (5,212–5,801)	16,592 (14,503–19,257)	3,227 (1,912–4,980)	4,976 (3,877–6,180)	0 (0–0)	5,914 (5,062–6,890)
Kuwait	Prevalence Number: Cumulative change (%)	16.6 (14.9–18.3)	16.2 (7.0–24.4)	16.3 (10.6–22.1)	6.1 (-4.0 to 17.4)	2.7 (-6.1 to 11.0)	38.3 (33.0–43.7)	14.1 (-6.6 to 36.3)	-3.8 (-53.0 to 69.9)	5.7 (-16.1 to 30.6)	19.4 (-6.1 to 35.7)	16.4 (0.6–33.3)
Kuwait	Prevalence Rate: 1990	85,892.9 (84,995.9–86,646.1)	22,385.6 (21,406.5–23,573.8)	29,655.3 (28,619.6–30,652.7)	8,415.3 (7,054.9–9,860.9)	15,582.3 (14,527.1–16,596.7)	442.9 (419.3–467.6)	1,584.6 (1,386.1–1,822.5)	409.0 (256.0–597.7)	511.5 (406.5–621.7)	0.0 (0.0–0.0)	570.2 (483.8–667.9)
Kuwait	Prevalence Rate: 2015	85,602.6 (84,548.4–86,490.7)	21,594.1 (20,352.4–22,951.5)	30,253.3 (29,196.7–31,385.6)	7,887.6 (6,438.0–9,442.5)	13,439.1 (12,565.1–14,305.7)	425.2 (404.2–448.5)	1,505.8 (1,316.8–1,753.1)	238.4 (141.2–367.8)	454.4 (354.8–567.8)	0.0 (0.0–0.0)	508.9 (432.8–597.6)
Kuwait	Prevalence Rate: Cumulative change (%)	-0.3 (-1.8 to 1.0)	-3.5 (-10.0 to 2.7)	2.1 (-3.2 to 7.3)	-6.2 (-15.0 to 3.5)	-13.6 (-21.1 to -6.7)	-4.0 (-7.6 to -0.2)	-4.3 (-21.8 to 14.2)	-37.3 (-69.4 to 10.8)	-10.5 (-29.4 to 11.1)	-3.9 (-23.4 to 10.0)	-10.4 (-22.7 to 3.1)
Kuwait	YLDs Number: 1990	52,812 (40,122–67,338)	7,616 (5,430–10,230)	6,413 (4,438–8,878)	3,613 (2,378–4,941)	821 (592–1,097)	654 (471–860)	978 (684–1,314)	454 (245–746)	2,127 (1,453–2,833)	0 (0–0)	739 (556–950)
Kuwait	YLDs Number: 2015	53,107 (40,229–67,453)	8,644 (6,140–11,820)	7,548 (5,222–10,441)	3,837 (2,527–5,315)	839 (600–1,146)	906 (652–1,201)	1,816 (1,138–2,638)	406 (208–679)	1,538 (993–2,158)	0 (0–0)	707 (529–921)
Kuwait	YLDs Number: Cumulative change (%)	0.6 (-5.6 to 5.9)	13.6 (2.8–22.4)	17.7 (15.1–20.5)	6.3 (-4.3 to 17.7)	2.2 (-5.8 to 11.1)	38.5 (29.3–48.3)	85.3 (40.9–126.3)	-3.8 (-53.5 to 70.0)	-27.0 (-46.1 to -6.0)	14.5 (-11.2 to 34.5)	-4.2 (-12.7 to 5.4)
Kuwait	YLDs Rate: 1990	5,737.0 (4,355.0–7,319.0)	825.7 (587.6–1,106.3)	693.1 (480.3–960.9)	383.2 (252.6–523.3)	88.2 (63.2–117.9)	73.0 (52.7–96.0)	105.9 (74.1–142.4)	51.4 (27.9–84.6)	229.4 (156.8–305.4)	0.0 (0.0–0.0)	79.8 (60.1–102.7)
Kuwait	YLDs Rate: 2015	5,069.7 (3,841.1–6,447.9)	793.4 (565.9–1,084.4)	701.2 (482.7–972.6)	359.5 (237.1–494.3)	78.3 (56.2–106.8)	70.1 (50.6–92.8)	164.3 (102.8–238.6)	30.0 (15.4–50.2)	140.2 (89.5–196.6)	0.0 (0.0–0.0)	64.6 (48.3–84.1)
Kuwait	YLDs Rate: Cumulative change (%)	-11.5 (-16.9 to -7.0)	-3.8 (-11.4 to 3.5)	1.2 (-0.9 to 3.4)	-6.1 (-15.0 to 3.6)	-11.2 (-17.2 to -3.7)	-3.9 (-10.4 to 2.7)	54.8 (17.2–89.7)	-37.3 (-69.7 to 10.7)	-38.3 (-54.7 to -20.3)	-4.8 (-26.0 to 10.9)	-18.9 (-26.2 to -10.7)
Lebanon	Prevalence Number: 1990	1,089,460 (1,073,729–1,102,501)	316,769 (298,378–330,671)	362,809 (348,882–377,422)	118,255 (97,969–139,593)	231,348 (223,171–240,751)	14,094 (12,726–15,481)	17,297 (15,011–20,361)	7,256 (4,435–11,231)	6,697 (5,370–8,100)	0 (0–0)	10,133 (8,606–11,917)
Lebanon	Prevalence Number: 2015	1,670,529 (1,649,009–1,688,975)	466,589 (434,022–495,195)	600,691 (577,475–623,804)	162,033 (134,142–193,297)	329,383 (316,369–341,883)	11,126 (10,104–12,147)	27,384 (23,818–31,691)	7,691 (4,550–11,996)	8,310 (6,519–10,221)	0 (0–0)	11,600 (9,918–13,500)
Lebanon	Prevalence Number: Cumulative change (%)	53.3 (50.7–55.9)	47.5 (35.2–62.4)	65.6 (57.5–73.9)	37.5 (18.2–58.0)	42.4 (34.4–49.9)	-21.0 (-24.5 to -17.2)	59.6 (29.5–95.4)	14.9 (-47.6 to 106.9)	25.1 (-2.4 to 55.0)	29.2 (-1.4 to 51.8)	15.1 (-2.5 to 32.8)
Lebanon	Prevalence Rate: 1990	88,982.6 (87,733.1–90,020.4)	25,919.4 (24,506.6–26,987.2)	29,837.7 (28,681.5–31,052.7)	9,700.2 (8,056.0–11,453.2)	18,876.2 (18,204.3–19,640.2)	1,107.0 (1,000.8–1,214.4)	1,409.5 (1,223.5–1,658.5)	563.3 (344.3–871.9)	546.7 (437.8–660.7)	0.0 (0.0–0.0)	821.7 (697.3–967.2)
Lebanon	Prevalence Rate: 2015	88,371.9 (87,125.2–89,490.1)	24,893.9 (22,744.4–26,622.8)	30,402.0 (29,251.8–31,513.2)	8,462.4 (6,996.2–10,095.2)	17,514.8 (16,809.5–18,186.3)	727.0 (657.0–798.6)	1,465.3 (1,277.0–1,693.9)	542.8 (321.2–846.7)	440.5 (346.1–543.5)	0.0 (0.0–0.0)	651.5 (560.9–754.6)

© 2017 Global Burden of Disease Child and Adolescent Health Collaboration. JAMA Pediatrics.

eTable 3. Prevalent Cases, Rates (per 100,000 population), Years Lived with Disability (YLDs), and Cumulative Percent Change with 95% Uncertainty Interval (UI) for the Top 10 Global Causes of YLDs in Children and Adolescents in 195 Countries and Territories, Aged 0 to 19 Years, Both Sexes, 1990 and 2015. *Note: best viewed by enlarging in browser.*

Location	Measure	All causes	Iron-deficiency anemia	Skin and subcutaneous diseases	Asthma	Hemoglobinopathies and hemolytic anemias	Diarrheal diseases	Congenital anomalies	Protein-energy malnutrition	Epilepsy	Malaria	Neonatal preterm birth complications
Lebanon	Prevalence Rate: Cumulative change (%)	-0.7 (-2.5 to 1.1)	-3.8 (-12.9 to 6.7)	1.9 (-3.1 to 6.9)	-12.5 (-24.9 to 0.3)	-7.2 (-12.5 to -2.3)	-34.3 (-37.3 to -31.0)	4.8 (-14.8 to 28.4)	4.5 (-52.3 to 88.1)	-18.8 (-36.2 to 0.3)	-3.9 (-23.4 to 10.0)	-20.3 (-32.2 to -8.6)
Lebanon	YLDs Number: 1990	114,512 (84,290–148,671)	11,868 (8,531–15,852)	9,017 (6,286–12,303)	5,276 (3,519–7,291)	2,062 (1,474–2,777)	2,289 (1,636–3,051)	1,404 (900–2,215)	900 (469–1,505)	2,762 (1,894–3,770)	0 (0–0)	1,426 (1,086–1,838)
Lebanon	YLDs Number: 2015	111,904 (85,192–142,277)	17,431 (12,333–23,721)	14,369 (9,916–19,743)	7,360 (4,836–10,157)	2,843 (2,005–3,864)	1,827 (1,303–2,424)	3,254 (1,701–5,765)	964 (494–1,614)	2,342 (1,553–3,224)	0 (0–0)	1,379 (1,048–1,793)
Lebanon	YLDs Number: Cumulative change (%)	-1.1 (-20.4 to 15.7)	47.0 (33.5–63.0)	59.2 (54.5–64.4)	39.9 (20.2–60.5)	38.1 (25.4–53.5)	-20.2 (-24.9 to -15.0)	139.5 (34.4–305.1)	16.0 (-46.5 to 107.8)	-14.4 (-35.9 to 10.0)	32.6 (-2.8 to 63.8)	-3.2 (-13.2 to 8.0)
Lebanon	YLDs Rate: 1990	9,381.8 (6,919.8–12,161.9)	969.6 (697.6–1,296.7)	736.6 (513.0–1,007.0)	432.8 (289.2–598.9)	168.5 (121.0–226.7)	179.8 (128.6–239.4)	114.4 (73.3–180.4)	69.9 (36.5–116.9)	225.4 (154.1–307.5)	0.0 (0.0–0.0)	116.3 (88.6–150.0)
Lebanon	YLDs Rate: 2015	5,720.5 (4,336.6–7,298.0)	937.7 (660.7–1,285.5)	754.7 (525.0–1,033.3)	384.7 (251.1–529.0)	152.6 (106.8–208.2)	119.5 (85.1–159.4)	174.7 (91.1–308.7)	68.1 (34.9–113.9)	124.3 (82.7–170.9)	0.0 (0.0–0.0)	73.8 (56.2–95.9)
Lebanon	YLDs Rate: Cumulative change (%)	-38.3 (-50.2 to -28.0)	-3.2 (-14.3 to 8.1)	2.4 (0.3–4.9)	-10.9 (-23.6 to 2.4)	-9.4 (-19.3 to 1.6)	-33.5 (-37.4 to -29.4)	58.0 (-11.7 to 168.3)	5.5 (-51.4 to 89.0)	-44.4 (-58.2 to -29.0)	-3.7 (-27.8 to 20.2)	-36.5 (-42.7 to -29.2)
Libya	Prevalence Number: 1990	2,091,347 (2,063,803–2,117,668)	382,890 (330,588–433,852)	669,281 (643,551–696,275)	191,033 (157,979–225,112)	483,798 (460,839–507,408)	20,758 (18,732–22,981)	32,903 (28,570–38,564)	11,774 (7,875–16,917)	8,714 (7,171–10,324)	0 (0–0)	14,677 (12,255–17,723)
Libya	Prevalence Number: 2015	2,107,576 (2,074,097–2,136,729)	437,364 (410,705–487,275)	689,804 (663,822–715,812)	164,986 (131,832–201,249)	473,712 (449,970–498,768)	18,790 (16,719–20,869)	34,833 (30,633–40,060)	12,283 (7,669–18,742)	7,641 (6,213–9,259)	0 (0–0)	14,973 (12,694–17,732)
Libya	Prevalence Number: Cumulative change (%)	0.8 (-1.0 to 2.6)	14.8 (1.4–33.6)	3.1 (-2.1 to 8.2)	-13.5 (-24.8 to -0.7)	-2.0 (-8.2 to 4.5)	-9.5 (-14.3 to -5.2)	6.6 (-12.6 to 27.2)	10.5 (-42.9 to 88.7)	-11.7 (-29.5 to 8.3)	2.8 (-16.9 to 38.4)	2.6 (-13.1 to 20.2)
Libya	Prevalence Rate: 1990	89,325.3 (88,170.2–90,438.1)	16,310.9 (14,090.5–18,433.3)	28,825.1 (27,733.2–29,978.5)	8,105.0 (6,708.3–9,543.0)	20,646.4 (19,661.6–21,655.5)	873.3 (788.0–966.3)	1,403.8 (1,219.1–1,644.9)	494.0 (330.4–709.7)	371.6 (305.7–440.2)	0.0 (0.0–0.0)	627.2 (523.9–757.0)
Libya	Prevalence Rate: 2015	87,915.2 (86,562.7–89,103.4)	18,164.8 (17,093.4–20,154.9)	29,279.6 (28,165.6–30,427.7)	6,845.0 (5,494.0–8,333.2)	19,716.9 (18,724.0–20,756.7)	752.8 (670.5–835.2)	1,448.6 (1,273.8–1,665.3)	487.2 (304.2–743.5)	318.6 (259.0–387.3)	0.0 (0.0–0.0)	621.0 (526.6–735.8)
Libya	Prevalence Rate: Cumulative change (%)	-1.6 (-3.3 to 0.2)	11.9 (-0.9 to 29.2)	1.6 (-3.5 to 6.5)	-15.5 (-26.5 to -3.0)	-4.4 (-10.4 to 1.9)	-13.8 (-18.4 to -9.8)	3.9 (-14.8 to 23.9)	4.5 (-46.0 to 78.4)	-13.6 (-31.2 to 5.8)	-1.9 (-21.0 to 33.0)	-0.4 (-15.6 to 16.7)
Libya	YLDs Number: 1990	130,286 (98,396–164,567)	13,390 (9,138–19,017)	15,867 (10,919–22,043)	8,702 (5,730–11,975)	5,198 (3,702–7,035)	3,417 (2,426–4,529)	2,546 (1,659–3,769)	1,475 (843–2,342)	3,757 (2,585–4,954)	0 (0–0)	2,057 (1,546–2,624)
Libya	YLDs Number: 2015	135,240 (102,348–173,265)	15,858 (11,028–21,891)	16,361 (11,255–22,849)	7,508 (4,770–10,472)	4,581 (3,257–6,258)	3,098 (2,193–4,131)	3,754 (2,151–6,532)	1,540 (792–2,521)	2,172 (1,467–2,961)	0 (0–0)	1,729 (1,297–2,263)
Libya	YLDs Number: Cumulative change (%)	3.8 (-2.0 to 10.7)	19.4 (3.5–41.3)	3.1 (1.0–5.3)	-13.6 (-25.3 to -0.8)	-11.7 (-18.4 to -2.4)	-9.3 (-15.8 to -3.0)	50.8 (-11.4 to 148.3)	10.4 (-43.8 to 87.7)	-41.6 (-55.2 to -25.1)	2.2 (-21.0 to 40.2)	-15.9 (-23.2 to -8.0)
Libya	YLDs Rate: 1990	5,598.2 (4,236.2–7,068.1)	566.9 (387.0–805.5)	678.9 (467.1–942.9)	369.2 (242.8–508.9)	219.3 (156.2–296.7)	143.8 (102.2–190.7)	108.6 (70.8–160.7)	61.9 (35.4–98.3)	160.2 (110.2–211.5)	0.0 (0.0–0.0)	87.7 (66.0–111.8)
Libya	YLDs Rate: 2015	5,703.4 (4,317.8–7,293.7)	654.9 (456.0–899.2)	685.3 (470.1–958.8)	311.5 (199.2–434.1)	188.4 (134.0–257.2)	124.1 (87.8–165.5)	156.0 (89.3–271.3)	61.1 (31.4–100.0)	90.5 (61.4–123.6)	0.0 (0.0–0.0)	71.8 (53.9–93.9)
Libya	YLDs Rate: Cumulative change (%)	1.9 (-3.6 to 8.4)	16.5 (1.3–36.8)	0.9 (-1.2 to 3.1)	-15.5 (-26.7 to -3.1)	-13.9 (-20.4 to -5.3)	-13.6 (-19.8 to -7.6)	47.0 (-13.7 to 141.9)	4.4 (-46.8 to 77.3)	-42.9 (-56.3 to -26.6)	-2.4 (-24.8 to 34.3)	-18.0 (-25.0 to -10.4)
Morocco	Prevalence Number: 1990	11,523,279 (11,396,455–11,633,024)	3,160,754 (3,008,748–3,279,630)	3,641,193 (3,509,981–3,772,863)	850,741 (730,513–992,070)	2,934,740 (2,627,411–3,260,525)	207,090 (190,106–226,273)	138,623 (118,023–168,204)	58,761 (45,008–74,406)	83,585 (67,593–100,497)	55 (15–103)	108,408 (92,191–131,730)
Morocco	Prevalence Number: 2015	11,148,786 (11,017,298–11,260,199)	2,913,845 (2,755,818–3,070,957)	3,650,785 (3,522,710–3,783,500)	646,701 (537,940–775,279)	2,616,828 (2,378,410–2,870,488)	119,250 (110,432–130,679)	156,874 (136,422–183,365)	100,028 (61,433–156,917)	62,177 (49,807–75,813)	0 (0–0)	96,155 (82,137–113,155)
Morocco	Prevalence Number: Cumulative change (%)	-3.2 (-4.7 to -1.8)	-7.8 (-12.2 to -3.9)	0.3 (-4.6 to 5.4)	-24.0 (-31.8 to -14.8)	-10.5 (-22.2 to 2.4)	-42.3 (-47.5 to -37.4)	14.3 (-9.8 to 39.9)	73.9 (0.1–178.4)	-25.0 (-40.1 to -7.7)	-99.6 (-100.0 to -100.0)	-10.7 (-25.6 to 5.2)
Morocco	Prevalence Rate: 1990	90,989.6 (90,024.1–91,832.7)	24,668.2 (23,550.9–25,565.5)	29,225.3 (28,168.8–30,303.7)	6,709.8 (5,756.6–7,782.6)	23,129.8 (20,702.5–25,702.8)	1,566.2 (1,439.0–1,710.6)	1,090.7 (928.8–1,323.4)	441.0 (337.8–558.4)	656.4 (531.0–789.5)	0.4 (0.1–0.8)	841.1 (713.5–1,024.2)
Morocco	Prevalence Rate: 2015	90,524.9 (89,459.2–91,416.2)	23,663.1 (22,428.4–24,875.1)	29,719.3 (28,689.6–30,798.9)	5,312.5 (4,422.7–6,369.3)	21,230.5 (19,297.7–23,289.5)	936.3 (868.2–1,024.1)	1,271.6 (1,105.5–1,486.1)	771.7 (474.0–1,210.6)	503.8 (403.8–616.8)	0.0 (0.0–0.0)	768.0 (655.2–904.8)
Morocco	Prevalence Rate: Cumulative change (%)	-0.5 (-1.9 to 0.9)	-4.1 (-8.5 to -0.3)	1.7 (-3.2 to 6.9)	-20.8 (-28.9 to -11.2)	-7.9 (-20.0 to 5.4)	-40.1 (-45.4 to -35.1)	17.8 (-7.2 to 44.0)	78.8 (2.9–186.1)	-22.6 (-38.2 to -4.8)	-99.6 (-100.0 to -100.0)	-8.0 (-23.5 to 8.5)
Morocco	YLDs Number: 1990	828,503 (631,709–1,044,767)	117,449 (82,394–158,070)	94,906 (65,824–129,437)	38,535 (25,873–52,551)	34,270 (24,315–45,758)	33,934 (24,660–44,889)	11,369 (7,255–16,902)	7,340 (4,545–10,718)	36,573 (25,324–48,985)	1 (0–2)	14,086 (10,827–17,849)
Morocco	YLDs Number: 2015	734,239 (556,770–930,356)	106,493 (75,510–143,085)	93,476 (64,543–127,142)	29,355 (19,451–40,986)	28,861 (20,616–38,429)	19,559 (14,126–25,999)	15,407 (9,691–23,212)	12,462 (6,810–20,365)	21,440 (14,284–29,461)	0 (0–0)	11,554 (8,833–14,653)
Morocco	YLDs Number: Cumulative change (%)	-11.4 (-13.3 to -9.5)	-9.3 (-13.9 to -6.0)	-1.5 (-3.6 to 0.6)	-23.8 (-32.0 to -14.3)	-15.8 (-19.2 to -11.8)	-42.3 (-47.6 to -37.1)	39.2 (-9.7 to 101.0)	74.6 (-1.0 to 178.7)	-40.9 (-54.2 to -26.1)	-99.5 (-100.0 to -100.0)	-17.9 (-25.6 to -9.3)
Morocco	YLDs Rate: 1990	6,597.6 (5,040.8–8,319.8)	910.6 (640.2–1,224.3)	751.9 (520.4–1,027.4)	303.9 (204.8–414.0)	266.6 (188.8–355.1)	256.6 (186.5–339.3)	89.4 (57.0–133.0)	55.1 (34.1–80.4)	287.1 (199.1–384.2)	0.0 (0.0–0.0)	110.5 (85.0–140.0)
Morocco	YLDs Rate: 2015	5,981.2 (4,535.9–7,574.9)	873.0 (619.9–1,170.6)	759.4 (525.3–1,032.4)	241.1 (159.7–336.7)	237.8 (169.8–316.6)	153.5 (111.0–204.1)	124.8 (78.6–187.9)	96.1 (52.5–157.1)	173.7 (115.9–238.8)	0.0 (0.0–0.0)	93.7 (71.7–118.7)
Morocco	YLDs Rate: Cumulative change (%)	-9.3 (-11.3 to -7.4)	-4.1 (-8.8 to -0.6)	1.0 (-1.1 to 3.1)	-20.6 (-29.1 to -10.6)	-10.8 (-14.4 to -6.7)	-40.1 (-45.6 to -34.7)	43.5 (-6.9 to 107.1)	79.5 (1.8–186.5)	-39.0 (-52.8 to -23.8)	-99.5 (-100.0 to -100.0)	-15.1 (-23.1 to -6.0)
Palestine	Prevalence Number: 1990	1,108,625 (1,090,295–1,124,497)	210,148 (193,586–230,751)	335,044 (322,901–348,413)	78,717 (65,276–93,199)	299,631 (259,513–340,841)	12,243 (10,965–13,680)	17,112 (14,567–20,362)	5,593 (3,437–8,584)	7,096 (5,874–8,443)	0 (0–0)	12,384 (10,643–14,299)
Palestine	Prevalence Number: 2015	2,135,962 (2,102,595–2,162,611)	444,998 (394,572–490,034)	671,062 (646,049–699,129)	155,806 (128,641–184,128)	540,732 (469,704–620,407)	16,236 (14,806–17,891)	34,286 (30,067–39,926)	7,110 (4,517–10,758)	11,558 (9,277–14,005)	0 (0–0)	23,250 (20,113–26,816)
Palestine	Prevalence Number: Cumulative change (%)	92.7 (89.1–96.4)	112.6 (77.8–145.0)	100.4 (90.4–110.5)	98.1 (82.7–114.0)	81.5 (50.3–118.1)	32.8 (25.2–39.6)	102.1 (63.1–144.1)	38.0 (-31.1 to 139.3)	64.1 (30.2–100.2)	76.1 (37.8–103.7)	88.6 (62.4–117.2)
Palestine	Prevalence Rate: 1990	88,969.5 (87,641.0–90,128.0)	16,359.9 (15,184.0–17,766.5)	28,179.7 (27,132.0–29,331.6)	6,532.3 (5,475.8–7,729.1)	23,794.1 (20,620.6–27,047.7)	798.2 (718.9–887.7)	1,351.4 (1,150.1–1,607.3)	347.3 (213.4–532.9)	572.7 (472.6–681.6)	0.0 (0.0–0.0)	887.0 (754.5–1,034.0)
Palestine	Prevalence Rate: 2015	88,895.2 (87,585.9–89,961.2)	18,275.8 (16,326.1–20,016.1)	28,439.9 (27,360.3–29,640.4)	6,587.1 (5,452.1–7,792.4)	22,415.0 (19,469.3–25,717.1)	621.1 (567.6–682.7)	1,418.8 (1,244.0–1,652.6)	264.3 (168.0–400.0)	484.0 (387.6–586.9)	0.0 (0.0–0.0)	921.1 (792.4–1,068.9)
Palestine	Prevalence Rate: Cumulative change (%)	-0.1 (-1.9 to 1.7)	12.1 (-4.3 to 28.2)	1.0 (-4.1 to 6.1)	0.9 (-6.8 to 8.6)	-5.3 (-21.5 to 13.8)	-22.1 (-26.4 to -18.2)	5.9 (-14.6 to 27.6)	-17.4 (-58.7 to 43.3)	-14.9 (-32.3 to 3.8)	-3.9 (-23.4 to 10.0)	4.4 (-11.5 to 21.5)
Palestine	YLDs Number: 1990	71,952 (55,112–90,464)	7,174 (5,047–9,892)	7,968 (5,482–10,976)	3,575 (2,348–4,918)	2,966 (2,116–3,971)	2,017 (1,427–2,724)	1,138 (785–1,590)	699 (373–1,128)	2,983 (2,059–3,970)	0 (0–0)	1,522 (1,169–1,948)
Palestine	YLDs Number: 2015	124,763 (94,898–157,857)	15,850 (10,842–22,047)	15,592 (10,618–21,588)	7,100 (4,747–9,775)	5,102 (3,581–6,994)	2,678 (1,892–3,600)	3,239 (2,016–4,891)	891 (479–1,426)	3,300 (2,238–4,513)	0 (0–0)	2,685 (2,053–3,419)
Palestine	YLDs Number: Cumulative change (%)	73.4 (61.5–83.7)	122.0 (76.7–151.0)	95.6 (90.8–100.3)	98.7 (81.9–115.1)	72.3 (49.5–91.2)	33.0 (23.8–41.8)	186.7 (104.5–303.8)	38.3 (-32.5 to 144.8)	11.6 (-15.9 to 41.1)	79.9 (35.5–118.9)	76.5 (61.5–92.2)
Palestine	YLDs Rate: 1990	6,045.4 (4,628.2–7,587.7)	561.1 (393.8–765.8)	649.0 (443.9–900.5)	296.5 (194.6–404.9)	238.1 (169.7–319.0)	131.4 (93.1–177.3)	89.6 (61.5–125.2)	43.4 (23.2–70.0)	240.2 (165.4–320.4)	0.0 (0.0–0.0)	120.3 (92.7–153.9)
Palestine	YLDs Rate: 2015	5,285.4 (4,023.7–6,673.3)	653.9 (450.9–906.5)	652.8 (443.9–908.3)	300.1 (200.5–413.2)	212.6 (149.7–290.1)	102.4 (72.1–137.4)	133.9 (83.3–202.2)	33.1 (17.8–53.0)	138.1 (93.3–188.8)	0.0 (0.0–0.0)	111.2 (84.9–141.6)
Palestine	YLDs Rate: Cumulative change (%)	-12.6 (-18.3 to -7.5)	16.9 (-3.0 to 29.9)	0.6 (-1.7 to 2.9)	1.3 (-7.1 to 9.4)	-10.6 (-20.5 to -1.6)	-21.9 (-27.3 to -16.7)	50.6 (7.9–111.9)	-17.2 (-59.6 to 46.7)	-42.0 (-56.4 to -26.4)	-2.9 (-26.5 to 17.0)	-7.5 (-15.3 to 0.9)
Oman	Prevalence Number: 1990	917,534 (910,596–923,149)	298,939 (278,352–326,076)	258,332 (248,369–268,596)	64,360 (55,046–76,165)	412,647 (396,839–428,899)	10,341 (9,317–11,549)	12,930 (11,164–15,295)	13,543 (10,060–17,650)	3,879 (3,106–4,748)	3 (0–6)	6,550 (5,553–7,791)
Oman	Prevalence Number: 2015	1,066,005 (1,056,729–1,075,220)	279,744 (253,643–307,487)	320,893 (308,924–333,769)	75,668 (61,832–89,757)	431,473 (412,585–451,696)	7,325 (6,722–7,922)	16,383 (14,324–19,251)	10,880 (6,976–15,752)	4,660 (3,691–5,817)	0 (0–0)	6,384 (5,443–7,416)
Oman	Prevalence Number: Cumulative change (%)	16.2 (15.0–17.4)	-6.3 (-16.6 to 5.7)	24.3 (18.1–30.8)	17.8 (4.9–37.0)	4.6 (-1.7 to 11.1)	-29.0 (-34.0 to -24.5)	27.8 (2.8–55.1)	-17.3 (-51.8 to 26.2)	21.3 (-4.8 to 51.7)	-99.4 (-100.0 to -99.7)	-2.1 (-16.0 to 13.1)
Oman	Prevalence Rate: 1990	93,876.0 (93,219.0–94,403.8)	29,753.4 (28,113.0–31,443.2)	28,404.3 (27,368.7–29,562.5)	6,759.1 (5,862.0–7,965.9)	42,003.6 (40,403.4–43,663.0)	851.8 (771.6–946.8)	1,307.7 (1,128.5–1,549.8)	1,062.6 (789.4–1,384.7)	391.3 (311.2–481.5)	0.3 (0.1–0.6)	618.0 (520.0–742.0)
Oman	Prevalence Rate: 2015	92,543.8 (91,774.6–93,275.0)	23,644.4 (21,696.7–25,790.3)	29,030.3 (27,972.8–30,218.4)	6,807.5 (5,579.1–8,082.0)	37,252.0 (35,633.8–39,002.3)	532.1 (490.0–574.2)	1,411.2 (1,232.1–1,659.0)	761.3 (488.1–1,102.3)	403.2 (318.3–504.3)	0.0 (0.0–0.0)	510.2 (431.5–596.5)
Oman	Prevalence Rate: Cumulative change (%)	-1.4 (-2.3 to -0.4)	-20.5 (-27.8 to -11.4)	2.3 (-2.9 to 7.5)	0.8 (-9.8 to 15.2)	-11.3 (-16.7 to -5.8)	-37.4 (-41.7 to -33.6)	8.8 (-12.6 to 32.3)	-26.3 (-57.0 to 12.6)	4.0 (-18.0 to 29.9)	-99.4 (-100.0 to -99.8)	-17.0 (-29.6 to -3.0)
Oman	YLDs Number: 1990	68,231 (51,222–86,674)	11,669 (8,335–15,988)	5,918 (4,083–8,175)	2,905 (1,937–3,993)	1,636 (1,179–2,212)	1,682 (1,185–2,238)	906 (621–1,249)	1,681 (1,011–2,511)	1,654 (1,115–2,243)	0 (0–0)	909 (694–1,172)

© 2017 Global Burden of Disease Child and Adolescent Health Collaboration. JAMA Pediatrics.

eTable 3. Prevalent Cases, Rates (per 100,000 population), Years Lived with Disability (YLDs), and Cumulative Percent Change with 95% Uncertainty Interval (UI) for the Top 10 Global Causes of YLDs in Children and Adolescents in 195 Countries and Territories, Aged 0 to 19 Years, Both Sexes, 1990 and 2015. *Note: best viewed by enlarging in browser.*

Location	Measure	All causes	Iron-deficiency anemia	Skin and subcutaneous diseases	Asthma	Hemoglobinopathies and hemolytic anemias	Diarrheal diseases	Congenital anomalies	Protein-energy malnutrition	Epilepsy	Malaria	Neonatal preterm birth complications
Oman	YLDs Number: 2015	73,078 (54,788–92,584)	10,252 (7,123–13,827)	7,116 (4,878–9,867)	3,424 (2,265–4,706)	1,698 (1,209–2,281)	1,199 (852–1,605)	1,584 (958–2,521)	1,357 (755–2,152)	1,322 (897–1,820)	0 (0–0)	711 (535–915)
Oman	YLDs Number: Cumulative change (%)	7.1 (2.9–11.6)	-11.9 (-24.5 to 2.8)	20.2 (17.0–23.5)	18.1 (4.9–38.1)	3.9 (-6.1 to 13.1)	-28.6 (-34.4 to -22.9)	76.5 (21.2–166.9)	-17.0 (-51.8 to 28.0)	-19.1 (-38.7 to 2.3)	-99.3 (-99.9 to -99.5)	-21.7 (-29.2 to -13.6)
Oman	YLDs Rate: 1990	7,157.8 (5,401.3–9,046.0)	1,139.9 (817.2–1,545.7)	624.1 (429.7–865.4)	305.1 (204.5–418.1)	162.1 (116.9–216.9)	138.5 (98.0–184.2)	91.4 (62.5–125.8)	131.9 (79.4–197.1)	166.7 (112.4–225.9)	0.0 (0.0–0.0)	92.0 (70.3–118.9)
Oman	YLDs Rate: 2015	6,467.9 (4,863.6–8,193.4)	874.7 (612.7–1,176.3)	629.3 (431.1–876.1)	308.0 (204.1–423.1)	148.6 (106.5–198.6)	87.1 (61.9–117.0)	136.1 (82.5–215.8)	95.0 (52.8–156.9)	114.3 (77.3–158.1)	0.0 (0.0–0.0)	61.5 (46.3–79.1)
Oman	YLDs Rate: Cumulative change (%)	-9.6 (-12.8 to -6.3)	-23.2 (-32.5 to -12.0)	0.8 (-1.7 to 3.2)	1.1 (-10.1 to 15.9)	-8.2 (-15.6 to -1.2)	-37.0 (-42.0 to -32.0)	50.5 (3.5–126.4)	-25.9 (-57.0 to 14.1)	-30.6 (-47.3 to -12.2)	-99.4 (-100.0 to -99.6)	-33.1 (-39.4 to -25.8)
Qatar	Prevalence Number: 1990	142,930 (140,360–145,097)	30,626 (27,765–33,762)	45,651 (43,953–47,379)	9,310 (7,697–11,070)	34,816 (29,637–40,327)	1,131 (1,025–1,238)	2,449 (2,124–2,886)	1,145 (674–1,737)	780 (605–963)	0 (0–0)	1,051 (887–1,239)
Qatar	Prevalence Number: 2015	385,572 (379,125–390,803)	87,397 (81,339–93,529)	132,918 (128,061–137,982)	25,212 (20,635–29,895)	83,663 (71,764–97,495)	2,160 (1,983–2,339)	6,605 (5,778–7,671)	1,668 (991–2,577)	1,871 (1,419–2,382)	0 (0–0)	2,349 (1,988–2,760)
Qatar	Prevalence Number: Cumulative change (%)	169.8 (164.0–175.9)	186.2 (161.5–217.6)	191.3 (176.8–205.8)	171.2 (145.1–198.8)	142.0 (96.6–193.1)	91.1 (81.5–102.1)	171.8 (120.0–224.6)	58.6 (-24.7 to 186.4)	142.4 (81.8–209.7)	130.2 (80.1–169.1)	124.3 (93.6–156.9)
Qatar	Prevalence Rate: 1990	87,515.7 (86,076.3–88,701.6)	18,720.0 (17,185.4–20,511.3)	29,397.5 (28,292.6–30,507.4)	5,687.6 (4,715.1–6,690.1)	21,092.0 (17,968.4–24,407.3)	590.0 (536.1–644.6)	1,482.8 (1,285.8–1,742.4)	584.5 (343.9–886.3)	468.0 (363.6–579.1)	0.0 (0.0–0.0)	615.8 (518.4–729.1)
Qatar	Prevalence Rate: 2015	87,137.3 (85,679.8–88,310.7)	19,717.4 (18,367.1–21,096.5)	29,987.2 (28,881.3–31,118.0)	5,748.9 (4,701.5–6,812.4)	18,914.7 (16,219.0–22,038.9)	480.7 (441.9–520.7)	1,491.4 (1,304.6–1,731.1)	371.9 (221.1–574.7)	423.6 (321.1–539.4)	0.0 (0.0–0.0)	522.0 (441.3–614.8)
Qatar	Prevalence Rate: Cumulative change (%)	-0.4 (-2.4 to 1.7)	5.6 (-2.7 to 16.6)	2.1 (-2.8 to 7.1)	1.2 (-8.4 to 11.3)	-9.7 (-26.7 to 9.4)	-18.5 (-22.5 to -13.9)	1.4 (-18.1 to 20.9)	-30.7 (-67.1 to 25.1)	-8.5 (-31.4 to 16.5)	-3.9 (-23.4 to 10.1)	-14.9 (-26.9 to -2.1)
Qatar	YLDs Number: 1990	8,489 (6,471–10,721)	1,052 (725–1,452)	1,102 (751–1,520)	425 (277–597)	236 (168–320)	186 (133–250)	251 (139–420)	143 (75–239)	358 (238–498)	0 (0–0)	159 (120–204)
Qatar	YLDs Number: 2015	22,836 (17,436–28,903)	3,139 (2,208–4,241)	3,054 (2,076–4,282)	1,148 (757–1,590)	513 (366–689)	356 (253–476)	767 (421–1,260)	208 (108–346)	653 (408–927)	0 (0–0)	306 (229–395)
Qatar	YLDs Number: Cumulative change (%)	169.1 (159.2–179.0)	199.8 (169.7–249.4)	176.8 (168.6–185.4)	170.7 (144.6–201.5)	117.1 (99.4–140.4)	91.6 (78.2–106.1)	225.4 (73.7–439.8)	58.9 (-24.5 to 191.8)	85.4 (23.7–159.1)	123.3 (66.1–169.8)	92.9 (74.7–112.0)
Qatar	YLDs Rate: 1990	5,410.8 (4,124.9–6,820.9)	642.9 (445.6–890.8)	684.2 (465.2–953.5)	259.5 (169.6–361.5)	141.0 (99.7–191.2)	97.2 (69.6–130.4)	151.6 (83.9–255.8)	73.3 (38.6–122.2)	214.7 (142.0–296.9)	0.0 (0.0–0.0)	96.5 (72.7–123.7)
Qatar	YLDs Rate: 2015	5,152.2 (3,932.3–6,525.0)	714.4 (503.1–965.4)	691.1 (469.6–970.1)	262.0 (172.9–362.8)	117.4 (83.8–157.8)	79.4 (56.3–106.1)	173.2 (95.2–284.2)	46.6 (24.2–77.2)	147.8 (92.2–210.2)	0.0 (0.0–0.0)	69.3 (52.0–89.4)
Qatar	YLDs Rate: Cumulative change (%)	-4.8 (-8.1 to -1.5)	11.5 (1.2–28.5)	1.0 (-1.4 to 3.1)	1.1 (-8.5 to 12.2)	-16.6 (-22.9 to -8.0)	-18.2 (-23.8 to -12.1)	21.8 (-35.1 to 102.5)	-30.6 (-67.0 to 27.5)	-29.9 (-53.3 to -2.0)	-6.5 (-28.6 to 12.8)	-28.1 (-35.0 to -21.2)
Saudi Arabia	Prevalence Number: 1990	7,348,363 (7,320,522–7,376,320)	1,229,821 (1,206,767–1,253,751)	2,315,992 (2,285,377–2,345,338)	609,967 (510,596–719,436)	3,350,819 (3,303,101–3,401,019)	85,466 (78,170–93,699)	105,925 (96,325–116,179)	52,936 (43,819–63,035)	41,540 (36,520–47,308)	8,154 (5,803–11,108)	57,308 (50,150–65,057)
Saudi Arabia	Prevalence Number: 2015	10,180,693 (10,140,106–10,218,339)	1,237,257 (1,206,260–1,265,123)	3,419,332 (3,378,488–3,461,334)	726,153 (599,347–857,779)	4,586,659 (4,524,560–4,652,075)	67,781 (61,916–74,111)	186,209 (170,268–204,834)	99,096 (82,604–118,809)	50,075 (42,914–58,350)	8,050 (5,406–11,125)	72,287 (62,783–82,326)
Saudi Arabia	Prevalence Number: Cumulative change (%)	38.5 (37.9–39.2)	0.6 (-2.4 to 3.7)	47.6 (45.4–49.9)	19.2 (9.0–29.6)	36.9 (34.3–39.6)	-20.7 (-24.8 to -17.6)	76.2 (59.3–95.2)	89.4 (45.4–139.3)	20.6 (8.6–32.7)	-1.2 (-13.7 to 13.5)	26.3 (17.2–34.9)
Saudi Arabia	Prevalence Rate: 1990	89,580.3 (89,274.6–89,885.1)	15,024.7 (14,760.4–15,299.4)	29,686.9 (29,307.6–30,061.8)	7,415.7 (6,222.7–8,707.6)	40,342.0 (39,770.5–40,954.7)	868.0 (795.8–948.8)	1,269.3 (1,154.1–1,391.4)	519.5 (430.0–618.6)	502.4 (443.0–574.3)	93.9 (66.6–126.8)	642.3 (559.4–732.8)
Saudi Arabia	Prevalence Rate: 2015	89,070.6 (88,726.8–89,395.8)	10,727.9 (10,462.1–10,967.5)	30,294.2 (29,933.7–30,673.0)	6,305.3 (5,207.8–7,436.4)	40,084.7 (39,541.1–40,655.0)	583.3 (533.2–637.7)	1,627.2 (1,487.8–1,790.0)	854.7 (712.4–1,024.7)	437.1 (374.3–509.2)	69.1 (46.5–95.5)	630.7 (547.7–718.3)
Saudi Arabia	Prevalence Rate: Cumulative change (%)	-0.6 (-1.0 to -0.1)	-28.6 (-30.7 to -26.5)	2.0 (0.5–3.5)	-14.9 (-21.6 to -7.6)	-0.6 (-2.5 to 1.3)	-32.8 (-36.3 to -30.2)	28.5 (16.3–42.4)	66.4 (27.8–110.3)	-12.9 (-21.7 to -4.0)	-26.4 (-36.4 to -15.0)	-1.7 (-8.9 to 5.2)
Saudi Arabia	YLDs Number: 1990	438,253 (334,671–555,359)	45,950 (32,913–61,596)	58,678 (40,340–80,404)	27,766 (18,282–38,203)	32,710 (23,521–43,438)	14,120 (10,192–18,870)	10,136 (6,580–15,042)	6,655 (4,410–9,387)	18,063 (12,993–23,002)	133 (95–179)	6,595 (4,961–8,497)
Saudi Arabia	YLDs Number: 2015	582,925 (444,419–735,418)	46,051 (32,680–62,236)	83,614 (57,633–115,501)	33,086 (21,993–45,302)	49,543 (35,798–65,708)	11,205 (7,983–14,959)	25,408 (14,754–39,725)	12,500 (8,268–17,622)	14,542 (10,367–18,964)	179 (112–263)	8,303 (6,230–10,875)
Saudi Arabia	YLDs Number: Cumulative change (%)	33.1 (30.4–36.2)	0.2 (-2.7 to 3.1)	42.4 (39.8–45.1)	19.3 (9.1–30.0)	51.6 (45.9–57.4)	-20.6 (-25.0 to -17.2)	153.1 (74.6–253.1)	90.0 (46.3–140.0)	-19.4 (-28.8 to -9.0)	34.7 (0.2–73.2)	25.8 (17.8–34.9)
Saudi Arabia	YLDs Rate: 1990	5,561.6 (4,235.6–7,020.1)	555.2 (397.3–744.2)	723.7 (497.3–1,000.1)	337.4 (221.9–465.7)	390.5 (280.9–518.3)	143.3 (103.4–191.2)	121.0 (78.5–179.3)	65.3 (43.3–92.1)	218.1 (156.2–279.0)	1.5 (1.1–2.1)	78.7 (59.2–101.1)
Saudi Arabia	YLDs Rate: 2015	5,139.3 (3,913.1–6,487.9)	396.5 (281.4–536.0)	733.9 (505.7–1,017.2)	287.3 (191.1–393.3)	426.6 (308.0–565.6)	96.4 (68.8–128.8)	222.0 (128.8–347.1)	107.8 (71.3–152.0)	126.9 (90.3–165.6)	1.5 (1.0–2.2)	72.5 (54.4–95.0)
Saudi Arabia	YLDs Rate: Cumulative change (%)	-7.6 (-9.4 to -5.5)	-28.6 (-30.7 to -26.6)	1.4 (0.2–2.5)	-14.8 (-21.7 to -7.5)	9.2 (5.5–13.1)	-32.7 (-36.4 to -29.8)	85.3 (28.1–158.0)	67.0 (28.6–110.9)	-41.7 (-48.6 to -34.0)	-1.1 (-26.0 to 25.9)	-8.0 (-14.1 to -1.4)
Sudan	Prevalence Number: 1990	10,611,457 (10,550,210–10,671,721)	3,978,094 (3,503,425–4,282,106)	3,005,923 (2,884,538–3,129,967)	1,188,352 (995,599–1,404,946)	2,096,374 (2,030,272–2,157,864)	207,924 (189,419–229,940)	93,072 (77,364–112,361)	239,029 (185,615–299,985)	58,213 (46,644–70,104)	1,118,308 (910,650–1,367,723)	100,169 (80,669–125,796)
Sudan	Prevalence Number: 2015	19,370,215 (19,249,564–19,474,401)	6,776,962 (6,265,854–7,167,606)	5,748,786 (5,524,609–5,976,527)	1,842,695 (1,534,559–2,202,329)	3,685,315 (3,559,689–3,799,912)	306,254 (276,339–344,152)	211,222 (181,545–247,948)	394,594 (277,382–542,233)	104,311 (83,424–125,668)	1,990,123 (1,439,584–2,672,797)	161,143 (132,217–197,664)
Sudan	Prevalence Number: Cumulative change (%)	82.5 (81.1–84.0)	71.0 (51.4–96.4)	91.3 (82.0–101.3)	55.2 (43.1–69.0)	75.8 (68.8–83.1)	47.3 (39.0–58.2)	129.5 (81.0–188.6)	68.9 (8.2–148.2)	80.8 (44.3–123.5)	77.0 (49.7–108.1)	62.8 (29.7–98.3)
Sudan	Prevalence Rate: 1990	94,627.3 (94,121.9–95,114.1)	34,394.6 (30,572.3–36,901.0)	27,822.4 (26,724.9–28,937.3)	10,690.8 (9,026.6–12,606.9)	18,594.9 (18,009.0–19,138.9)	1,611.5 (1,470.9–1,779.0)	811.2 (671.0–981.8)	1,794.9 (1,394.0–2,252.6)	516.1 (414.2–623.4)	9,986.3 (8,196.2–12,072.5)	791.8 (632.0–1,002.6)
Sudan	Prevalence Rate: 2015	93,643.2 (93,092.3–94,111.4)	32,112.3 (29,821.5–33,721.6)	28,474.3 (27,322.7–29,618.9)	8,959.8 (7,483.0–10,709.2)	17,754.0 (17,151.4–18,307.1)	1,353.1 (1,223.3–1,518.1)	1,012.8 (870.1–1,189.1)	1,701.5 (1,196.2–2,338.0)	502.4 (401.0–604.8)	9,592.9 (6,972.0–12,915.5)	735.6 (601.0–907.0)
Sudan	Prevalence Rate: Cumulative change (%)	-1.0 (-1.8 to -0.3)	-6.3 (-16.4 to 6.6)	2.4 (-2.7 to 7.7)	-16.1 (-22.6 to -8.5)	-4.5 (-8.3 to -0.5)	-16.0 (-20.6 to -9.8)	26.3 (-0.9 to 58.8)	-3.0 (-37.9 to 42.5)	-1.8 (-21.6 to 21.3)	-4.4 (-19.4 to 12.7)	-5.7 (-26.9 to 17.1)
Sudan	YLDs Number: 1990	870,065 (660,753–1,118,111)	167,222 (116,716–228,178)	73,554 (50,993–100,680)	53,571 (35,322–73,660)	29,018 (20,609–39,171)	33,599 (24,409–44,995)	6,729 (4,515–9,575)	29,507 (18,881–42,860)	25,201 (17,114–34,064)	11,987 (8,545–15,982)	9,965 (7,509–12,898)
Sudan	YLDs Number: 2015	1,449,915 (1,094,687–1,844,553)	271,892 (194,342–365,556)	137,790 (95,527–188,855)	83,275 (55,103–114,661)	48,265 (34,388–64,132)	49,730 (35,895–66,752)	17,438 (11,218–26,067)	48,838 (28,536–74,121)	40,479 (27,115–53,902)	19,884 (14,223–26,486)	16,868 (13,007–21,222)
Sudan	YLDs Number: Cumulative change (%)	66.8 (58.1–75.4)	63.6 (37.0–96.6)	87.3 (83.0–91.8)	55.6 (42.9–70.3)	66.5 (44.9–92.8)	48.0 (39.0–58.7)	161.7 (98.1–250.1)	69.7 (7.8–148.4)	62.5 (25.6–106.2)	66.3 (49.2–83.5)	70.0 (48.6–93.1)
Sudan	YLDs Rate: 1990	7,812.8 (5,937.7–10,030.4)	1,428.0 (1,003.7–1,945.1)	665.0 (460.0–912.9)	481.8 (318.7–660.5)	252.9 (179.5–341.0)	260.4 (189.0–348.8)	58.5 (39.1–83.5)	221.6 (141.8–321.9)	223.3 (151.9–302.1)	105.7 (75.3–141.0)	85.9 (64.6–111.2)
Sudan	YLDs Rate: 2015	7,058.9 (5,326.2–8,978.1)	1,280.4 (915.6–1,724.2)	671.4 (465.0–923.1)	404.8 (267.4–556.3)	229.1 (163.3–305.5)	219.7 (158.3–293.7)	83.4 (53.6–124.7)	210.6 (123.1–319.6)	194.9 (130.6–260.2)	95.2 (68.0–126.9)	80.3 (61.9–101.2)
Sudan	YLDs Rate: Cumulative change (%)	-9.6 (-13.8 to -5.4)	-9.8 (-23.9 to 7.2)	0.9 (-1.3 to 3.3)	-15.9 (-22.6 to -8.2)	-9.1 (-20.3 to 4.5)	-15.6 (-20.7 to -9.5)	44.0 (9.2–92.7)	-2.6 (-38.1 to 42.6)	-11.7 (-31.7 to 12.2)	-9.7 (-18.7 to -0.6)	-6.1 (-18.0 to 7.1)
Syria	Prevalence Number: 1990	6,492,477 (6,422,890–6,555,967)	1,523,448 (1,421,164–1,626,310)	1,931,935 (1,852,438–2,011,502)	696,566 (581,515–820,005)	1,537,741 (1,484,004–1,591,522)	78,591 (71,123–86,584)	95,102 (82,967–111,061)	92,279 (69,620–117,430)	23,112 (18,279–28,506)	30 (8–57)	69,862 (59,989–82,302)
Syria	Prevalence Number: 2015	8,147,183 (8,074,866–8,208,595)	1,869,817 (1,807,782–1,948,898)	2,589,711 (2,484,754–2,692,558)	717,414 (600,512–842,463)	1,792,418 (1,729,587–1,860,636)	57,211 (52,515–62,498)	128,416 (111,695–148,614)	94,391 (64,015–131,210)	32,628 (25,475–40,630)	0 (0–0)	68,308 (58,238–80,886)
Syria	Prevalence Number: Cumulative change (%)	25.5 (23.9–27.1)	22.9 (15.2–31.3)	34.1 (27.4–41.3)	3.2 (-5.9 to 13.1)	16.6 (10.9–22.5)	-27.1 (-32.1 to -22.3)	36.0 (10.7–63.9)	5.1 (-36.3 to 60.4)	42.3 (11.6–75.2)	-99.3 (-100.0 to -99.8)	-1.7 (-16.8 to 15.2)
Syria	Prevalence Rate: 1990	90,079.4 (89,154.3–90,913.5)	20,949.5 (19,601.9–22,267.5)	27,629.5 (26,517.5–28,772.3)	9,642.8 (8,096.1–11,285.3)	21,227.1 (20,485.8–21,972.9)	989.8 (897.0–1,089.0)	1,308.4 (1,141.6–1,529.8)	1,144.6 (863.5–1,456.5)	317.9 (250.5–391.4)	0.4 (0.1–0.8)	927.8 (794.4–1,097.6)
Syria	Prevalence Rate: 2015	89,151.6 (88,365.1–89,827.1)	20,569.8 (19,875.7–21,428.8)	28,260.6 (27,110.7–29,385.5)	7,779.5 (6,517.9–9,133.6)	19,647.1 (18,958.4–20,398.4)	648.1 (594.0–707.6)	1,408.6 (1,224.9–1,630.1)	1,078.7 (731.6–1,499.5)	357.1 (278.9–444.3)	0.0 (0.0–0.0)	774.5 (662.9–914.7)
Syria	Prevalence Rate: Cumulative change (%)	-1.0 (-2.2 to 0.2)	-1.7 (-7.2 to 4.3)	2.3 (-2.8 to 7.9)	-19.2 (-26.0 to -11.9)	-7.4 (-12.0 to -2.7)	-34.4 (-38.9 to -30.1)	8.4 (-12.0 to 30.5)	-3.2 (-41.3 to 47.7)	13.2 (-11.0 to 40.0)	-99.5 (-100.0 to -99.8)	-16.1 (-29.1 to -1.9)
Syria	YLDs Number: 1990	416,271 (316,072–528,604)	56,423 (40,061–76,346)	43,679 (29,999–60,473)	31,658 (20,961–43,841)	16,687 (11,935–22,533)	12,908 (9,336–17,343)	5,725 (3,938–7,914)	11,550 (7,259–16,808)	9,868 (6,754–13,494)	0 (0–1)	8,800 (6,748–11,172)
Syria	YLDs Number: 2015	716,850 (506,701–955,444)	68,446 (49,044–92,532)	56,779 (38,786–79,174)	32,624 (21,688–44,876)	19,830 (14,326–26,466)	9,372 (6,728–12,515)	9,633 (6,214–14,405)	11,770 (6,658–18,002)	9,806 (6,525–13,629)	0 (0–0)	7,569 (5,707–9,685)
Syria	YLDs Number: Cumulative change (%)	72.3 (42.0–108.9)	21.5 (14.1–30.0)	29.9 (26.6–33.2)	3.2 (-6.0 to 13.7)	19.0 (11.7–26.5)	-27.3 (-33.3 to -21.6)	69.0 (31.0–126.8)	4.8 (-35.9 to 58.4)	0.4 (-23.3 to 28.5)	-99.3 (-100.0 to -99.6)	-14.0 (-21.9 to -5.4)

© 2017 Global Burden of Disease Child and Adolescent Health Collaboration. JAMA Pediatrics.

eTable 3. Prevalent Cases, Rates (per 100,000 population), Years Lived with Disability (YLDs), and Cumulative Percent Change with 95% Uncertainty Interval (UI) for the Top 10 Global Causes of YLDs in Children and Adolescents in 195 Countries and Territories, Aged 0 to 19 Years, Both Sexes, 1990 and 2015. *Note: best viewed by enlarging in browser.*

Location	Measure	All causes	Iron-deficiency anemia	Skin and subcutaneous diseases	Asthma	Hemoglobinopathies and hemolytic anemias	Diarrheal diseases	Congenital anomalies	Protein-energy malnutrition	Epilepsy	Malaria	Neonatal preterm birth complications
Syria	YLDs Rate: 1990	5,873.2 (4,469.8–7,439.8)	768.4 (546.6–1,042.0)	612.5 (418.9–850.3)	438.1 (292.3–605.9)	226.8 (162.0–306.6)	162.5 (117.5–218.0)	78.7 (54.2–108.7)	143.3 (90.0–208.5)	135.6 (92.4–184.9)	0.0 (0.0–0.0)	120.9 (92.9–153.5)
Syria	YLDs Rate: 2015	7,857.9 (5,546.4–10,483.9)	748.2 (535.5–1,011.6)	620.2 (423.3–865.3)	353.8 (235.3–486.1)	215.8 (155.7–288.7)	106.2 (76.1–142.0)	105.7 (68.2–157.9)	134.5 (76.1–205.8)	107.3 (71.4–149.2)	0.0 (0.0–0.0)	83.0 (62.6–106.1)
Syria	YLDs Rate: Cumulative change (%)	33.9 (10.1–63.3)	-2.5 (-7.7 to 3.8)	1.2 (-1.2 to 3.6)	-19.1 (-26.2 to -11.4)	-4.8 (-10.1 to 0.9)	-34.6 (-39.9 to -29.4)	34.9 (4.7–81.1)	-3.4 (-41.0 to 46.0)	-20.0 (-39.2 to 2.2)	-99.4 (-100.0 to -99.7)	-31.3 (-37.7 to -24.6)
Tunisia	Prevalence Number: 1990	3,486,044 (3,442,749–3,526,630)	705,245 (637,453–749,201)	1,103,648 (1,058,958–1,144,926)	309,362 (265,248–359,958)	900,910 (873,080–929,004)	47,093 (42,662–52,047)	51,949 (44,859–60,832)	24,231 (18,015–31,760)	20,355 (17,001–24,088)	0 (0–0)	32,200 (27,410–38,528)
Tunisia	Prevalence Number: 2015	3,047,593 (3,004,383–3,086,009)	558,510 (521,184–600,925)	1,007,013 (968,912–1,045,935)	239,568 (197,399–282,664)	733,131 (711,212–757,373)	27,790 (25,452–30,430)	49,264 (43,175–57,665)	12,343 (8,055–17,851)	15,061 (12,270–18,042)	0 (0–0)	24,139 (20,634–28,186)
Tunisia	Prevalence Number: Cumulative change (%)	-12.6 (-14.1 to -11.1)	-20.6 (-27.9 to -12.0)	-8.7 (-13.3 to -4.0)	-22.5 (-31.0 to -13.7)	-18.6 (-21.9 to -14.9)	-40.9 (-46.3 to -36.1)	-4.4 (-22.1 to 16.3)	-47.5 (-69.6 to -17.6)	-25.4 (-40.9 to -8.5)	-17.0 (-34.0 to -5.1)	-24.6 (-36.8 to -13.1)
Tunisia	Prevalence Rate: 1990	89,322.1 (88,242.0–90,328.7)	18,043.2 (16,349.7–19,108.1)	28,637.1 (27,478.9–29,737.2)	7,886.5 (6,766.6–9,168.8)	23,046.0 (22,330.1–23,765.3)	1,171.4 (1,062.7–1,293.4)	1,328.0 (1,147.0–1,555.4)	599.6 (445.8–785.9)	518.9 (433.1–612.6)	0.0 (0.0–0.0)	821.2 (698.9–982.9)
Tunisia	Prevalence Rate: 2015	88,495.8 (87,262.3–89,598.3)	16,166.3 (15,141.7–17,323.1)	29,369.3 (28,258.8–30,506.3)	7,070.7 (5,828.4–8,343.3)	21,244.9 (20,602.4–21,949.7)	765.9 (702.3–837.1)	1,426.2 (1,250.7–1,669.5)	332.4 (217.0–480.8)	437.5 (356.6–524.6)	0.0 (0.0–0.0)	682.2 (582.4–799.9)
Tunisia	Prevalence Rate: Cumulative change (%)	-0.9 (-2.6 to 0.7)	-10.2 (-18.1 to -0.4)	2.6 (-2.6 to 7.9)	-10.3 (-20.3 to -0.1)	-7.8 (-11.5 to -3.6)	-34.5 (-40.5 to -29.3)	8.2 (-11.7 to 31.7)	-42.9 (-66.9 to -10.4)	-15.0 (-32.8 to 4.4)	-3.9 (-23.4 to 10.1)	-16.4 (-30.2 to -3.5)
Tunisia	YLDs Number: 1990	225,251 (170,936–285,490)	25,298 (17,710–34,350)	26,783 (18,520–36,631)	14,069 (9,386–19,346)	10,747 (7,654–14,488)	7,743 (5,559–10,401)	4,267 (2,737–6,424)	3,031 (1,894–4,391)	8,556 (6,009–11,393)	0 (0–0)	4,371 (3,370–5,633)
Tunisia	YLDs Number: 2015	178,771 (135,687–226,459)	19,773 (13,932–27,043)	24,065 (16,575–33,105)	10,923 (7,253–15,144)	8,433 (6,034–11,384)	4,580 (3,264–6,053)	5,257 (3,031–8,715)	1,548 (892–2,412)	4,291 (2,917–5,868)	0 (0–0)	2,812 (2,133–3,592)
Tunisia	YLDs Number: Cumulative change (%)	-20.6 (-23.0 to -18.0)	-21.6 (-30.2 to -11.0)	-10.2 (-12.2 to -8.2)	-22.3 (-30.7 to -13.1)	-21.4 (-27.4 to -13.1)	-40.8 (-46.5 to -35.4)	26.4 (-22.6 to 94.4)	-47.3 (-69.2 to -17.6)	-49.5 (-61.4 to -35.5)	-20.3 (-38.7 to -5.5)	-35.6 (-42.3 to -28.3)
Tunisia	YLDs Rate: 1990	5,823.0 (4,422.0–7,379.2)	642.7 (451.0–872.1)	687.9 (475.1–942.4)	358.6 (239.5–491.9)	271.7 (192.9–365.9)	192.6 (138.3–258.6)	109.0 (69.9–163.8)	75.0 (46.9–108.7)	218.0 (153.1–290.4)	0.0 (0.0–0.0)	111.6 (86.0–143.8)
Tunisia	YLDs Rate: 2015	5,222.8 (3,965.9–6,616.6)	579.0 (407.0–790.3)	699.3 (482.6–963.8)	322.4 (214.2–447.3)	249.7 (179.3–337.9)	126.2 (90.1–166.9)	152.1 (87.7–252.1)	41.7 (24.0–65.0)	124.6 (84.8–170.8)	0.0 (0.0–0.0)	81.6 (61.8–104.3)
Tunisia	YLDs Rate: Cumulative change (%)	-10.3 (-12.9 to -7.4)	-9.6 (-19.1 to 2.2)	1.6 (-0.5 to 3.9)	-10.1 (-19.9 to 0.6)	-7.9 (-14.8 to 2.1)	-34.4 (-40.8 to -28.5)	43.2 (-12.1 to 120.3)	-42.6 (-66.4 to -10.4)	-42.4 (-56.1 to -26.5)	-6.3 (-27.8 to 10.7)	-26.8 (-34.3 to -18.6)
Turkey	Prevalence Number: 1990	22,756,677 (22,567,704–22,941,598)	6,598,100 (6,187,874–6,919,986)	7,158,128 (6,937,687–7,374,960)	2,007,710 (1,662,372–2,355,108)	4,792,745 (4,605,140–4,973,037)	366,853 (331,912–407,963)	284,598 (240,199–340,794)	120,773 (83,481–169,721)	206,158 (172,046–242,582)	513 (197–892)	168,598 (142,468–202,044)
Turkey	Prevalence Number: 2015	23,536,604 (23,337,855–23,732,057)	6,311,854 (6,027,489–6,713,369)	8,122,620 (7,895,432–8,358,616)	1,714,951 (1,414,572–2,043,795)	4,796,742 (4,621,863–4,978,607)	187,682 (175,185–201,800)	341,584 (299,383–402,987)	35,952 (21,763–55,912)	159,012 (132,421–188,407)	0 (0–0)	152,123 (129,520–179,506)
Turkey	Prevalence Number: Cumulative change (%)	3.4 (2.3–4.6)	-4.3 (-9.0 to 0.2)	13.5 (9.3–17.9)	-14.3 (-25.3 to 0.6)	0.1 (-5.0 to 5.1)	-48.7 (-53.7 to -44.6)	21.2 (-2.9 to 48.2)	-68.6 (-84.4 to -45.3)	-22.3 (-37.6 to -4.5)	-99.9 (-100.0 to -100.0)	-9.1 (-24.1 to 7.9)
Turkey	Prevalence Rate: 1990	89,863.9 (89,136.9–90,592.8)	25,916.6 (24,318.5–27,172.1)	28,535.3 (27,655.0–29,397.8)	7,864.5 (6,529.3–9,220.2)	18,912.2 (18,171.5–19,626.0)	1,430.5 (1,294.9–1,590.6)	1,122.3 (947.1–1,344.1)	470.1 (325.0–660.6)	812.0 (677.8–954.0)	2.0 (0.8–3.5)	664.8 (561.6–796.8)
Turkey	Prevalence Rate: 2015	88,560.7 (87,767.4–89,334.1)	24,058.7 (22,910.1–25,701.8)	30,096.6 (29,255.8–30,975.0)	6,418.4 (5,286.9–7,654.3)	18,126.0 (17,464.5–18,813.6)	756.1 (704.8–813.6)	1,293.0 (1,133.9–1,523.8)	148.2 (89.7–230.6)	603.5 (501.8–713.5)	0.0 (0.0–0.0)	592.7 (504.5–697.3)
Turkey	Prevalence Rate: Cumulative change (%)	-1.4 (-2.5 to -0.4)	-7.1 (-12.0 to -2.2)	5.5 (1.5–9.6)	-18.1 (-28.5 to -4.0)	-4.1 (-9.0 to 0.7)	-47.0 (-52.2 to -42.8)	16.3 (-6.6 to 42.2)	-66.8 (-83.5 to -42.1)	-25.1 (-39.6 to -8.3)	-99.9 (-100.0 to -100.0)	-10.2 (-24.8 to 6.4)
Turkey	YLDs Number: 1990	1,607,851 (1,222,102–2,034,439)	247,088 (173,712–334,257)	152,705 (104,075–213,931)	91,179 (60,330–125,025)	44,252 (31,379–59,933)	60,243 (43,242–81,071)	20,251 (13,590–29,403)	15,107 (8,686–23,315)	87,139 (60,902–116,829)	10 (3–20)	23,528 (18,051–29,792)
Turkey	YLDs Number: 2015	1,451,248 (1,097,246–1,836,617)	234,251 (165,260–316,130)	167,761 (113,088–238,918)	78,006 (51,634–108,130)	43,992 (31,355–58,883)	30,900 (21,995–41,209)	29,353 (18,461–43,784)	4,506 (2,376–7,556)	45,069 (31,630–61,351)	0 (0–0)	18,760 (14,358–24,083)
Turkey	YLDs Number: Cumulative change (%)	-9.8 (-12.1 to -7.3)	-5.1 (-9.6 to 0.2)	9.7 (6.8–12.7)	-14.1 (-25.1 to 1.1)	-0.4 (-8.2 to 6.2)	-48.6 (-53.8 to -43.7)	46.4 (5.7–102.0)	-68.6 (-84.6 to -45.4)	-47.8 (-60.2 to -33.8)	-99.9 (-100.0 to -100.0)	-20.2 (-28.1 to -11.4)
Turkey	YLDs Rate: 1990	6,376.3 (4,851.2–8,057.7)	964.8 (678.8–1,305.8)	605.5 (412.2–848.8)	357.1 (236.9–490.2)	172.8 (122.4–233.9)	234.9 (168.7–316.0)	79.8 (53.6–115.9)	58.8 (33.8–90.8)	343.1 (239.7–459.8)	0.0 (0.0–0.1)	92.7 (71.0–117.2)
Turkey	YLDs Rate: 2015	5,406.9 (4,085.7–6,843.8)	890.4 (628.9–1,199.6)	627.3 (422.9–892.5)	292.0 (193.7–406.1)	166.0 (118.2–222.9)	124.5 (88.6–166.1)	111.3 (70.0–165.8)	18.6 (9.8–31.2)	171.1 (119.9–232.7)	0.0 (0.0–0.0)	71.0 (54.3–91.0)
Turkey	YLDs Rate: Cumulative change (%)	-15.2 (-17.5 to -12.7)	-7.7 (-12.2 to -2.0)	3.5 (1.1–5.9)	-17.9 (-28.4 to -3.8)	-3.8 (-11.5 to 3.0)	-46.9 (-52.2 to -41.9)	40.8 (1.5–94.4)	-66.7 (-83.7 to -42.2)	-49.6 (-61.6 to -36.1)	-99.9 (-100.0 to -100.0)	-23.3 (-30.9 to -14.8)
United Arab Emirates	Prevalence Number: 1990	600,603 (594,100–606,746)	160,259 (149,929–177,527)	191,814 (184,319–198,877)	55,339 (45,828–65,305)	168,156 (160,870–175,240)	6,367 (5,662–7,116)	10,072 (8,753–11,698)	14,490 (10,196–19,775)	3,918 (3,085–4,797)	6 (1–11)	4,981 (4,172–5,858)
United Arab Emirates	Prevalence Number: 2015	1,464,940 (1,447,787–1,482,147)	349,063 (325,817–369,290)	505,168 (484,267–524,245)	110,911 (90,182–132,453)	369,105 (352,517–384,924)	10,001 (9,074–10,972)	24,813 (21,945–28,459)	22,116 (13,129–33,509)	8,043 (6,346–10,047)	0 (0–0)	11,161 (9,461–13,078)
United Arab Emirates	Prevalence Number: Cumulative change (%)	143.9 (140.3–147.6)	118.1 (99.4–133.5)	163.5 (149.8–177.2)	100.6 (81.4–120.0)	119.6 (106.7–133.5)	57.3 (47.6–67.0)	148.0 (106.1–195.7)	59.2 (-12.4 to 171.1)	107.6 (59.1–163.9)	-98.8 (-100.0 to -99.8)	125.4 (89.0–166.9)
United Arab Emirates	Prevalence Rate: 1990	88,996.2 (88,038.5–89,832.7)	23,460.3 (22,189.7–25,445.9)	29,584.9 (28,382.9–30,679.5)	8,384.3 (6,995.5–9,879.2)	24,662.3 (23,589.2–25,704.9)	790.2 (703.0–880.9)	1,475.0 (1,281.4–1,716.1)	1,761.8 (1,239.8–2,404.2)	582.1 (455.6–711.5)	0.9 (0.3–1.7)	700.3 (583.9–828.5)
United Arab Emirates	Prevalence Rate: 2015	86,805.1 (85,780.7–87,796.2)	20,720.6 (19,396.6–21,837.2)	30,032.4 (28,799.3–31,179.8)	6,695.8 (5,446.1–7,975.1)	21,839.3 (20,857.3–22,775.9)	553.7 (502.5–607.0)	1,464.9 (1,296.5–1,679.5)	1,214.6 (721.1–1,840.3)	477.2 (376.4–596.6)	0.0 (0.0–0.0)	647.7 (548.0–760.1)
United Arab Emirates	Prevalence Rate: Cumulative change (%)	-2.5 (-3.9 to -1.0)	-11.6 (-17.9 to -5.6)	1.6 (-3.7 to 7.0)	-20.1 (-27.7 to -12.6)	-11.4 (-16.6 to -5.7)	-29.8 (-34.1 to -25.5)	-0.0 (-16.7 to 18.9)	-28.1 (-60.4 to 22.4)	-17.1 (-36.2 to 5.8)	-99.5 (-100.0 to -99.9)	-6.9 (-22.5 to 10.5)
United Arab Emirates	YLDs Number: 1990	40,221 (30,387–50,819)	5,899 (4,101–8,025)	4,575 (3,142–6,341)	2,515 (1,659–3,470)	1,038 (728–1,399)	1,044 (731–1,415)	935 (579–1,451)	1,818 (1,058–2,817)	1,838 (1,200–2,511)	0 (0–0)	716 (542–924)
United Arab Emirates	YLDs Number: 2015	90,219 (68,885–113,833)	12,465 (8,895–16,799)	11,714 (7,976–16,338)	5,051 (3,353–6,992)	2,681 (1,916–3,610)	1,646 (1,160–2,196)	3,407 (1,764–6,501)	2,781 (1,475–4,673)	2,373 (1,576–3,315)	0 (0–0)	1,278 (956–1,643)
United Arab Emirates	YLDs Number: Cumulative change (%)	124.4 (114.2–134.9)	111.8 (85.8–131.1)	155.9 (149.5–162.6)	100.9 (81.2–121.7)	158.7 (134.5–182.5)	57.9 (46.5–70.3)	282.0 (91.1–627.3)	59.5 (-12.8 to 169.6)	31.0 (-5.3 to 77.9)	-98.8 (-100.0 to -99.7)	78.7 (61.7–96.9)
United Arab Emirates	YLDs Rate: 1990	6,105.7 (4,624.2–7,693.7)	864.6 (605.0–1,167.2)	686.1 (469.6–958.6)	380.9 (251.6–521.4)	153.2 (108.3–206.8)	129.6 (91.0–175.7)	136.6 (84.5–212.0)	221.0 (128.7–342.6)	272.6 (178.9–372.6)	0.0 (0.0–0.0)	105.5 (79.7–136.1)
United Arab Emirates	YLDs Rate: 2015	5,362.5 (4,091.2–6,763.8)	754.9 (539.3–1,014.5)	694.7 (472.3–971.0)	304.9 (201.9–421.9)	163.5 (117.1–220.0)	91.1 (64.2–121.7)	201.2 (104.3–385.2)	152.8 (81.0–256.6)	140.8 (92.9–197.0)	0.0 (0.0–0.0)	75.7 (56.6–97.2)
United Arab Emirates	YLDs Rate: Cumulative change (%)	-12.2 (-15.7 to -8.3)	-12.5 (-21.0 to -4.8)	1.2 (-1.0 to 3.4)	-19.9 (-27.9 to -11.9)	6.9 (-1.6 to 16.4)	-29.6 (-34.6 to -24.1)	54.4 (-23.0 to 193.7)	-27.9 (-60.6 to 21.8)	-47.6 (-62.1 to -29.5)	-99.5 (-100.0 to -99.9)	-28.1 (-34.9 to -21.1)
Yemen	Prevalence Number: 1990	6,889,455 (6,827,297–6,949,567)	1,297,067 (1,104,290–1,472,597)	1,909,603 (1,833,758–1,986,265)	724,361 (600,580–857,226)	1,682,217 (1,611,720–1,753,384)	150,226 (136,894–164,403)	66,910 (56,613–80,808)	173,416 (143,150–206,576)	40,937 (33,036–49,763)	233,541 (193,775–283,698)	70,324 (57,246–86,618)
Yemen	Prevalence Number: 2015	12,853,620 (12,733,881–12,968,355)	3,157,654 (2,810,211–3,569,422)	3,869,167 (3,709,042–4,021,266)	1,034,616 (855,402–1,221,204)	2,895,220 (2,783,904–3,013,204)	211,456 (194,736–229,217)	155,225 (134,582–182,867)	283,915 (230,930–339,555)	66,893 (54,139–81,383)	609,878 (448,600–850,397)	116,627 (95,920–143,130)
Yemen	Prevalence Number: Cumulative change (%)	86.6 (84.3–88.9)	145.1 (105.4–184.8)	102.7 (92.9–113.2)	43.0 (31.7–54.3)	72.2 (62.9–81.4)	40.9 (34.4–47.2)	134.4 (85.8–186.3)	65.7 (25.0–111.9)	64.7 (33.1–100.9)	159.7 (113.1–218.7)	67.4 (34.3–104.0)
Yemen	Prevalence Rate: 1990	93,523.0 (92,758.2–94,280.3)	14,985.4 (12,917.9–16,901.3)	27,673.3 (26,543.1–28,813.7)	10,043.7 (8,402.1–11,766.5)	22,599.1 (21,651.6–23,557.2)	1,609.1 (1,469.2–1,756.3)	880.3 (741.0–1,070.7)	1,762.8 (1,455.4–2,099.8)	548.9 (443.3–663.7)	3,172.8 (2,657.9–3,777.5)	796.7 (636.2–1,003.6)
Yemen	Prevalence Rate: 2015	92,623.4 (91,781.5–93,431.9)	22,012.9 (19,709.3–24,712.6)	28,430.6 (27,280.5–29,571.5)	7,527.9 (6,236.8–8,891.5)	20,811.7 (20,010.3–21,660.0)	1,420.6 (1,310.0–1,537.4)	1,112.8 (963.9–1,311.0)	1,867.3 (1,518.8–2,233.4)	482.2 (389.3–586.4)	4,386.4 (3,237.2–6,085.1)	802.8 (657.3–989.8)
Yemen	Prevalence Rate: Cumulative change (%)	-1.0 (-2.1 to 0.2)	47.8 (25.4–70.2)	2.8 (-2.4 to 8.1)	-25.0 (-30.5 to -19.2)	-7.9 (-12.8 to -2.9)	-11.7 (-15.7 to -7.7)	27.8 (0.7–56.1)	7.2 (-19.1 to 37.1)	-11.5 (-28.7 to 8.1)	37.5 (13.6–68.9)	2.1 (-21.0 to 27.1)
Yemen	YLDs Number: 1990	479,131 (360,833–608,287)	42,076 (27,162–59,281)	47,770 (32,790–65,070)	32,850 (21,775–45,842)	20,589 (14,743–27,615)	24,509 (17,863–32,668)	4,835 (3,285–6,848)	21,549 (14,318–30,215)	17,939 (12,212–24,218)	7,352 (4,850–10,318)	6,721 (5,063–8,598)
Yemen	YLDs Number: 2015	1,023,105 (763,199–1,328,091)	114,310 (77,941–158,144)	92,925 (63,675–128,584)	46,974 (31,157–64,969)	34,976 (25,009–46,963)	34,419 (24,913–45,298)	13,280 (8,595–19,858)	35,275 (23,116–49,397)	28,787 (19,496–38,764)	19,357 (12,561–27,616)	11,892 (8,984–15,157)
Yemen	YLDs Number: Cumulative change (%)	113.4 (94.3–142.6)	175.4 (113.0–251.5)	94.4 (88.6–100.0)	43.2 (31.5–55.2)	70.0 (54.6–84.0)	40.6 (33.0–48.6)	177.7 (112.3–285.3)	65.7 (24.4–113.0)	62.1 (27.2–100.8)	163.6 (124.4–205.2)	77.8 (54.4–102.3)
Yemen	YLDs Rate: 1990	6,639.0 (5,016.0–8,431.6)	481.6 (312.4–678.7)	664.0 (454.1–919.4)	455.2 (303.6–633.5)	277.6 (198.8–372.0)	262.4 (191.0–349.6)	63.2 (42.8–89.3)	219.1 (145.5–307.2)	240.2 (164.3–322.5)	100.7 (66.6–141.7)	86.9 (65.4–111.6)
Yemen	YLDs Rate: 2015	7,321.4 (5,482.7–9,471.8)	795.5 (546.6–1,098.4)	674.7 (461.7–937.3)	341.8 (225.6–471.0)	251.2 (179.8–336.6)	231.2 (167.5–303.8)	95.1 (61.3–142.2)	232.0 (152.0–324.9)	207.5 (140.3–279.3)	139.3 (90.1–198.5)	84.9 (64.2–108.2)

© 2017 Global Burden of Disease Child and Adolescent Health Collaboration. JAMA Pediatrics.

eTable 3. Prevalent Cases, Rates (per 100,000 population), Years Lived with Disability (YLDs), and Cumulative Percent Change with 95% Uncertainty Interval (UI) for the Top 10 Global Causes of YLDs in Children and Adolescents in 195 Countries and Territories, Aged 0 to 19 Years, Both Sexes, 1990 and 2015. *Note: best viewed by enlarging in browser.*

Location	Measure	All causes	Iron-deficiency anemia	Skin and subcutaneous diseases	Asthma	Hemoglobinopathies and hemolytic anemias	Diarrheal diseases	Congenital anomalies	Protein-energy malnutrition	Epilepsy	Malaria	Neonatal preterm birth complications
Yemen	YLDs Rate: Cumulative change (%)	10.2 (1.1–24.3)	67.2 (31.9–110.6)	1.6 (-0.8 to 3.9)	-24.9 (-30.6 to -19.0)	-9.4 (-17.0 to -2.3)	-11.8 (-16.5 to -7.0)	52.1 (15.8–112.2)	7.2 (-19.5 to 37.8)	-12.8 (-31.1 to 8.2)	38.5 (18.0–60.8)	-1.8 (-14.6 to 12.0)
South Asia	Prevalence Number: 1990	514,149,992 (513,413,060–514,911,357)	225,285,752 (222,731,581–227,586,232)	165,412,603 (163,656,261–167,250,362)	34,028,642 (28,687,596–39,846,519)	175,001,765 (171,309,296–179,832,102)	8,417,569 (7,958,336–8,967,436)	5,026,296 (4,656,220–5,407,655)	13,054,087 (12,613,700–13,487,111)	2,391,734 (2,135,239–2,669,906)	40,892,401 (32,458,597–49,719,619)	3,423,150 (2,937,181–3,954,245)
South Asia	Prevalence Number: 2015	620,847,125 (619,715,557–621,993,772)	248,419,970 (245,510,771–251,455,252)	214,109,490 (211,776,614–216,357,215)	31,947,985 (26,629,175–37,670,277)	199,986,527 (195,933,538–205,973,612)	8,261,636 (7,847,858–8,717,682)	7,813,202 (7,327,466–8,306,806)	10,067,488 (9,582,095–10,574,777)	2,884,194 (2,545,209–3,296,723)	15,450,728 (10,525,502–22,164,492)	4,572,823 (3,994,855–5,201,118)
South Asia	Prevalence Number: Cumulative change (%)	20.8 (20.5–21.0)	10.3 (8.5–12.2)	29.4 (27.8–31.0)	-6.1 (-10.1 to -2.2)	14.3 (10.9–17.7)	-1.8 (-4.8 to 0.7)	55.7 (42.9–68.2)	-22.8 (-27.1 to -18.5)	20.6 (11.3–30.6)	-62.5 (-69.9 to -51.5)	33.8 (25.5–42.6)
South Asia	Prevalence Rate: 1990	95,066.9 (94,938.7–95,200.1)	41,198.2 (40,768.7–41,588.1)	31,445.4 (31,107.1–31,789.7)	6,305.4 (5,324.7–7,384.1)	32,307.6 (31,625.6–33,197.9)	1,462.6 (1,386.2–1,554.9)	922.2 (854.1–992.3)	2,148.9 (2,076.3–2,220.1)	443.8 (396.9–495.2)	7,368.7 (5,849.5–8,990.4)	597.2 (511.3–686.5)
South Asia	Prevalence Rate: 2015	93,527.9 (93,357.2–93,703.9)	37,653.0 (37,193.6–38,125.4)	32,006.7 (31,658.1–32,345.5)	4,797.5 (3,991.4–5,660.5)	30,144.5 (29,534.2–31,045.6)	1,274.3 (1,209.9–1,345.6)	1,179.1 (1,105.6–1,253.1)	1,586.6 (1,510.1–1,666.5)	433.5 (382.7–495.6)	2,344.3 (1,591.3–3,367.2)	699.1 (610.4–796.0)
South Asia	Prevalence Rate: Cumulative change (%)	-1.6 (-1.8 to -1.4)	-8.6 (-10.1 to -7.1)	1.8 (0.5–3.0)	-23.9 (-27.1 to -20.8)	-6.7 (-9.4 to -3.9)	-12.9 (-15.4 to -10.6)	28.1 (17.5–38.3)	-26.1 (-30.2 to -22.0)	-2.3 (-9.8 to 5.4)	-68.4 (-74.8 to -58.5)	17.3 (9.9–25.0)
South Asia	YLDs Number: 1990	41,774,707 (31,649,756–53,262,927)	10,673,247 (7,799,696–14,156,701)	3,614,643 (2,473,864–5,038,203)	1,532,875 (1,020,869–2,126,520)	1,342,130 (983,386–1,766,271)	1,356,063 (983,905–1,782,969)	468,197 (299,053–696,019)	1,604,585 (1,099,426–2,142,415)	902,316 (643,398–1,157,475)	781,113 (556,295–1,042,600)	426,748 (327,954–541,864)
South Asia	YLDs Number: 2015	45,458,863 (34,605,017–57,584,100)	10,764,532 (7,780,667–14,314,503)	4,597,805 (3,133,499–6,458,872)	1,445,214 (949,453–2,000,664)	1,485,374 (1,077,918–1,962,066)	1,338,785 (964,411–1,762,662)	898,954 (564,938–1,341,866)	1,243,899 (856,362–1,654,054)	907,416 (658,419–1,175,103)	328,591 (234,791–443,642)	689,132 (538,074–863,812)
South Asia	YLDs Number: Cumulative change (%)	8.9 (6.7–11.0)	0.8 (-2.3 to 3.7)	27.1 (25.3–29.0)	-5.7 (-9.8 to -2.0)	10.6 (7.3–13.8)	-1.3 (-4.4 to 1.5)	93.7 (54.9–142.5)	-22.4 (-26.7 to -17.9)	0.7 (-8.8 to 10.3)	-57.9 (-60.6 to -54.8)	61.8 (50.5–72.9)
South Asia	YLDs Rate: 1990	7,754.7 (5,876.3–9,867.9)	1,929.1 (1,405.8–2,559.3)	678.0 (463.4–948.7)	284.0 (189.0–392.1)	243.5 (178.1–320.0)	235.6 (170.9–309.5)	85.9 (54.7–127.8)	264.1 (181.0–352.7)	167.4 (119.4–214.5)	141.2 (100.5–188.6)	76.9 (59.0–97.6)
South Asia	YLDs Rate: 2015	6,838.8 (5,204.4–8,665.5)	1,635.2 (1,182.2–2,175.9)	689.9 (471.2–968.4)	217.0 (142.5–300.8)	224.9 (163.1–297.3)	206.5 (148.8–272.0)	135.7 (85.3–202.5)	196.0 (135.0–260.7)	136.4 (99.1–176.7)	49.8 (35.5–67.2)	104.2 (81.4–130.6)
South Asia	YLDs Rate: Cumulative change (%)	-11.8 (-13.4 to -10.3)	-15.3 (-17.8 to -12.9)	1.7 (0.7–2.8)	-23.6 (-26.9 to -20.5)	-7.7 (-10.4 to -5.2)	-12.3 (-15.0 to -9.9)	59.4 (27.1–100.0)	-25.7 (-29.8 to -21.4)	-18.4 (-25.9 to -10.4)	-64.8 (-67.0 to -62.1)	35.8 (26.2–45.4)
South Asia	Prevalence Number: 1990	514,149,992 (513,413,060–514,911,357)	225,285,752 (222,731,581–227,586,232)	165,412,603 (163,656,261–167,250,362)	34,028,642 (28,687,596–39,846,519)	175,001,765 (171,309,296–179,832,102)	8,417,569 (7,958,336–8,967,436)	5,026,296 (4,656,220–5,407,655)	13,054,087 (12,613,700–13,487,111)	2,391,734 (2,135,239–2,669,906)	40,892,401 (32,458,597–49,719,619)	3,423,150 (2,937,181–3,954,245)
South Asia	Prevalence Number: 2015	620,847,125 (619,715,557–621,993,772)	248,419,970 (245,510,771–251,455,252)	214,109,490 (211,776,614–216,357,215)	31,947,985 (26,629,175–37,670,277)	199,986,527 (195,933,538–205,973,612)	8,261,636 (7,847,858–8,717,682)	7,813,202 (7,327,466–8,306,806)	10,067,488 (9,582,095–10,574,777)	2,884,194 (2,545,209–3,296,723)	15,450,728 (10,525,502–22,164,492)	4,572,823 (3,994,855–5,201,118)
South Asia	Prevalence Number: Cumulative change (%)	20.8 (20.5–21.0)	10.3 (8.5–12.2)	29.4 (27.8–31.0)	-6.1 (-10.1 to -2.2)	14.3 (10.9–17.7)	-1.8 (-4.8 to 0.7)	55.7 (42.9–68.2)	-22.8 (-27.1 to -18.5)	20.6 (11.3–30.6)	-62.5 (-69.9 to -51.5)	33.8 (25.5–42.6)
South Asia	Prevalence Rate: 1990	95,066.9 (94,938.7–95,200.1)	41,198.2 (40,768.7–41,588.1)	31,445.4 (31,107.1–31,789.7)	6,305.4 (5,324.7–7,384.1)	32,307.6 (31,625.6–33,197.9)	1,462.6 (1,386.2–1,554.9)	922.2 (854.1–992.3)	2,148.9 (2,076.3–2,220.1)	443.8 (396.9–495.2)	7,368.7 (5,849.5–8,990.4)	597.2 (511.3–686.5)
South Asia	Prevalence Rate: 2015	93,527.9 (93,357.2–93,703.9)	37,653.0 (37,193.6–38,125.4)	32,006.7 (31,658.1–32,345.5)	4,797.5 (3,991.4–5,660.5)	30,144.5 (29,534.2–31,045.6)	1,274.3 (1,209.9–1,345.6)	1,179.1 (1,105.6–1,253.1)	1,586.6 (1,510.1–1,666.5)	433.5 (382.7–495.6)	2,344.3 (1,591.3–3,367.2)	699.1 (610.4–796.0)
South Asia	Prevalence Rate: Cumulative change (%)	-1.6 (-1.8 to -1.4)	-8.6 (-10.1 to -7.1)	1.8 (0.5–3.0)	-23.9 (-27.1 to -20.8)	-6.7 (-9.4 to -3.9)	-12.9 (-15.4 to -10.6)	28.1 (17.5–38.3)	-26.1 (-30.2 to -22.0)	-2.3 (-9.8 to 5.4)	-68.4 (-74.8 to -58.5)	17.3 (9.9–25.0)
South Asia	YLDs Number: 1990	41,774,707 (31,649,756–53,262,927)	10,673,247 (7,799,696–14,156,701)	3,614,643 (2,473,864–5,038,203)	1,532,875 (1,020,869–2,126,520)	1,342,130 (983,386–1,766,271)	1,356,063 (983,905–1,782,969)	468,197 (299,053–696,019)	1,604,585 (1,099,426–2,142,415)	902,316 (643,398–1,157,475)	781,113 (556,295–1,042,600)	426,748 (327,954–541,864)
South Asia	YLDs Number: 2015	45,458,863 (34,605,017–57,584,100)	10,764,532 (7,780,667–14,314,503)	4,597,805 (3,133,499–6,458,872)	1,445,214 (949,453–2,000,664)	1,485,374 (1,077,918–1,962,066)	1,338,785 (964,411–1,762,662)	898,954 (564,938–1,341,866)	1,243,899 (856,362–1,654,054)	907,416 (658,419–1,175,103)	328,591 (234,791–443,642)	689,132 (538,074–863,812)
South Asia	YLDs Number: Cumulative change (%)	8.9 (6.7–11.0)	0.8 (-2.3 to 3.7)	27.1 (25.3–29.0)	-5.7 (-9.8 to -2.0)	10.6 (7.3–13.8)	-1.3 (-4.4 to 1.5)	93.7 (54.9–142.5)	-22.4 (-26.7 to -17.9)	0.7 (-8.8 to 10.3)	-57.9 (-60.6 to -54.8)	61.8 (50.5–72.9)
South Asia	YLDs Rate: 1990	7,754.7 (5,876.3–9,867.9)	1,929.1 (1,405.8–2,559.3)	678.0 (463.4–948.7)	284.0 (189.0–392.1)	243.5 (178.1–320.0)	235.6 (170.9–309.5)	85.9 (54.7–127.8)	264.1 (181.0–352.7)	167.4 (119.4–214.5)	141.2 (100.5–188.6)	76.9 (59.0–97.6)
South Asia	YLDs Rate: 2015	6,838.8 (5,204.4–8,665.5)	1,635.2 (1,182.2–2,175.9)	689.9 (471.2–968.4)	217.0 (142.5–300.8)	224.9 (163.1–297.3)	206.5 (148.8–272.0)	135.7 (85.3–202.5)	196.0 (135.0–260.7)	136.4 (99.1–176.7)	49.8 (35.5–67.2)	104.2 (81.4–130.6)
South Asia	YLDs Rate: Cumulative change (%)	-11.8 (-13.4 to -10.3)	-15.3 (-17.8 to -12.9)	1.7 (0.7–2.8)	-23.6 (-26.9 to -20.5)	-7.7 (-10.4 to -5.2)	-12.3 (-15.0 to -9.9)	59.4 (27.1–100.0)	-25.7 (-29.8 to -21.4)	-18.4 (-25.9 to -10.4)	-64.8 (-67.0 to -62.1)	35.8 (26.2–45.4)
Bangladesh	Prevalence Number: 1990	54,889,048 (54,660,761–55,131,039)	22,113,661 (20,789,523–23,434,609)	18,070,557 (17,314,953–18,942,764)	4,727,306 (3,915,100–5,655,281)	20,536,740 (17,383,139–25,186,269)	741,263 (698,249–789,699)	474,582 (376,016–613,199)	1,207,691 (1,033,711–1,390,144)	581,784 (474,438–687,254)	187,925 (114,945–277,099)	320,517 (263,834–392,610)
Bangladesh	Prevalence Number: 2015	60,589,934 (60,179,128–61,048,481)	17,509,332 (17,004,684–18,095,629)	21,632,619 (20,669,587–22,624,913)	4,424,347 (3,594,516–5,260,489)	21,041,353 (17,716,056–26,142,095)	704,080 (654,585–750,433)	664,196 (544,540–845,674)	858,470 (734,295–998,403)	608,888 (492,573–726,502)	144,519 (96,187–225,824)	374,035 (311,912–458,516)
Bangladesh	Prevalence Number: Cumulative change (%)	10.4 (9.5–11.3)	-20.7 (-26.5 to -13.6)	19.8 (12.9–26.9)	-6.3 (-15.6 to 3.4)	3.5 (-20.6 to 32.1)	-5.0 (-11.5 to 0.5)	42.7 (4.4–94.5)	-28.3 (-41.8 to -13.2)	5.7 (-17.2 to 33.6)	-22.1 (-40.4 to 1.9)	17.7 (-5.6 to 42.8)
Bangladesh	Prevalence Rate: 1990	97,607.6 (97,228.6–98,010.2)	38,922.0 (36,593.9–41,112.2)	33,037.6 (31,670.5–34,592.7)	8,401.5 (6,998.9–10,007.3)	36,482.1 (30,898.5–44,724.7)	1,240.3 (1,170.4–1,319.2)	837.3 (661.9–1,083.3)	1,934.1 (1,655.2–2,226.2)	1,051.4 (858.9–1,239.1)	327.4 (201.1–480.6)	539.0 (442.9–666.4)
Bangladesh	Prevalence Rate: 2015	95,296.6 (94,635.6–96,042.1)	27,937.2 (27,051.4–28,937.0)	33,429.4 (31,951.9–34,969.9)	6,923.4 (5,608.7–8,244.0)	33,142.4 (27,907.3–41,162.0)	1,167.1 (1,084.3–1,246.1)	1,049.0 (860.8–1,336.2)	1,479.8 (1,265.7–1,721.1)	948.4 (768.2–1,133.4)	231.3 (152.8–366.3)	610.1 (511.1–744.4)
Bangladesh	Prevalence Rate: Cumulative change (%)	-2.4 (-3.1 to -1.5)	-28.1 (-33.2 to -21.6)	1.2 (-4.6 to 7.3)	-17.5 (-25.6 to -8.9)	-8.2 (-29.6 to 17.1)	-5.9 (-12.2 to -0.4)	27.7 (-6.7 to 74.0)	-22.9 (-37.4 to -6.6)	-8.9 (-28.6 to 14.9)	-28.5 (-45.9 to -5.8)	14.3 (-9.0 to 38.5)
Bangladesh	YLDs Number: 1990	4,388,665 (3,316,082–5,601,320)	937,452 (670,952–1,267,979)	407,410 (278,060–568,096)	212,475 (140,743–291,438)	130,365 (93,496–176,868)	119,665 (87,498–157,701)	42,933 (27,200–65,316)	148,852 (101,298–207,281)	221,338 (150,269–295,295)	5,987 (3,429–10,020)	38,708 (28,841–50,606)
Bangladesh	YLDs Number: 2015	4,176,176 (3,179,064–5,255,169)	676,845 (480,093–910,578)	478,723 (326,161–664,494)	200,413 (132,626–280,105)	132,347 (95,488–178,353)	114,634 (82,262–152,079)	73,222 (42,677–118,623)	106,798 (70,393–148,618)	224,253 (153,172–298,042)	5,150 (2,819–9,176)	46,615 (35,543–59,687)
Bangladesh	YLDs Number: Cumulative change (%)	-4.7 (-8.9 to -0.6)	-27.6 (-35.4 to -18.4)	17.4 (14.7–20.2)	-5.6 (-15.2 to 4.7)	1.7 (-8.2 to 12.9)	-4.1 (-11.9 to 2.4)	74.1 (23.6–165.9)	-27.7 (-41.5 to -11.2)	2.7 (-21.6 to 31.8)	-14.1 (-32.8 to 8.0)	21.0 (5.4–37.4)
Bangladesh	YLDs Rate: 1990	7,902.4 (5,985.5–10,077.4)	1,635.7 (1,173.1–2,208.7)	734.6 (500.6–1,028.2)	377.5 (250.9–514.7)	228.6 (163.7–308.9)	200.2 (145.9–264.1)	75.7 (47.8–114.8)	238.4 (162.2–332.0)	399.8 (271.0–532.5)	10.4 (6.0–17.2)	67.1 (49.9–87.8)
Bangladesh	YLDs Rate: 2015	6,508.2 (4,946.1–8,206.0)	1,081.2 (768.3–1,455.7)	746.4 (508.2–1,037.7)	313.7 (206.8–436.8)	209.9 (150.9–282.7)	190.1 (136.5–252.1)	115.8 (67.4–187.7)	184.1 (121.3–256.2)	349.5 (238.0–464.3)	8.2 (4.5–14.9)	74.1 (56.5–94.9)
Bangladesh	YLDs Rate: Cumulative change (%)	-17.6 (-21.0 to -14.1)	-33.7 (-40.8 to -25.0)	1.6 (-0.3 to 3.6)	-16.9 (-25.4 to -8.0)	-8.0 (-17.0 to 2.4)	-5.0 (-12.8 to 1.6)	56.2 (10.5–139.1)	-22.2 (-37.0 to -4.5)	-11.4 (-32.9 to 13.6)	-20.9 (-38.5 to -0.3)	11.0 (-3.6 to 26.0)
Bhutan	Prevalence Number: 1990	264,222 (261,760–267,498)	107,624 (104,953–109,913)	91,525 (87,600–95,930)	21,208 (17,500–25,133)	72,283 (54,025–102,279)	5,588 (5,137–6,166)	2,594 (2,049–3,395)	1,644 (1,290–2,099)	1,199 (955–1,450)	6,420 (2,931–13,664)	1,669 (1,374–2,066)
Bhutan	Prevalence Number: 2015	261,071 (258,796–263,696)	100,986 (96,082–105,036)	97,758 (93,608–101,819)	19,388 (16,021–23,114)	66,658 (49,651–92,486)	9,415 (8,866–9,990)	3,041 (2,515–3,701)	1,633 (1,288–2,012)	1,297 (1,035–1,584)	5,666 (2,651–11,940)	1,445 (1,194–1,725)
Bhutan	Prevalence Number: Cumulative change (%)	-1.2 (-2.6 to 0.1)	-6.1 (-11.5 to -0.5)	6.9 (0.6–13.3)	-8.4 (-17.7 to 2.2)	-4.6 (-40.5 to 42.7)	68.8 (54.9–78.9)	19.5 (-13.7 to 53.9)	1.6 (-27.6 to 36.6)	9.2 (-14.8 to 36.6)	-10.1 (-23.9 to 9.8)	-12.7 (-29.8 to 3.9)
Bhutan	Prevalence Rate: 1990	92,640.4 (91,853.8–93,707.2)	38,062.9 (37,171.5–38,709.6)	33,242.5 (31,828.5–34,833.7)	7,380.6 (6,127.1–8,717.2)	25,265.4 (18,899.1–35,728.1)	1,780.8 (1,645.2–1,955.0)	897.1 (707.0–1,177.6)	488.5 (383.5–623.6)	419.1 (334.0–506.4)	2,112.5 (990.5–4,382.7)	545.5 (447.3–678.6)
Bhutan	Prevalence Rate: 2015	92,293.0 (91,462.0–93,255.7)	35,617.9 (33,638.3–37,261.2)	33,920.1 (32,523.5–35,305.7)	6,820.5 (5,622.4–8,134.9)	23,610.4 (17,575.6–32,767.4)	3,530.3 (3,319.3–3,751.3)	1,082.1 (894.5–1,317.3)	651.7 (514.0–802.8)	459.1 (365.9–559.3)	2,058.2 (948.1–4,419.4)	533.8 (443.7–635.0)
Bhutan	Prevalence Rate: Cumulative change (%)	-0.4 (-1.7 to 0.9)	-6.4 (-12.3 to -0.5)	2.1 (-3.9 to 8.2)	-7.5 (-16.7 to 2.9)	-3.4 (-39.7 to 44.5)	98.5 (83.9–109.9)	22.9 (-11.5 to 58.5)	36.5 (-2.8 to 83.3)	10.7 (-13.9 to 38.9)	-2.1 (-15.7 to 18.8)	-1.3 (-20.6 to 17.4)
Bhutan	YLDs Number: 1990	19,070 (14,509–24,276)	4,420 (3,175–5,900)	2,082 (1,424–2,925)	959 (633–1,336)	516 (366–693)	911 (658–1,210)	233 (144–353)	204 (132–296)	454 (311–607)	133 (89–184)	221 (165–286)
Bhutan	YLDs Number: 2015	19,132 (14,565–24,183)	4,083 (2,921–5,490)	2,141 (1,453–2,993)	875 (578–1,219)	464 (333–624)	1,534 (1,107–2,022)	334 (198–546)	203 (131–284)	358 (242–490)	112 (77–153)	198 (150–255)
Bhutan	YLDs Number: Cumulative change (%)	0.4 (-2.4 to 3.1)	-7.6 (-13.6 to -1.3)	2.8 (-0.0 to 5.6)	-8.5 (-18.2 to 2.3)	-9.9 (-17.6 to -2.0)	68.6 (55.1–80.1)	46.5 (1.2–123.0)	1.8 (-28.1 to 38.1)	-20.2 (-40.2 to 4.5)	-14.8 (-28.8 to 4.9)	-9.9 (-21.3 to 2.4)
Bhutan	YLDs Rate: 1990	6,855.0 (5,218.6–8,711.5)	1,561.3 (1,125.5–2,087.1)	741.2 (505.2–1,034.3)	333.7 (221.1–462.8)	181.4 (128.9–243.3)	290.3 (209.6–385.4)	80.5 (49.7–122.1)	60.7 (39.5–88.0)	158.6 (109.1–211.9)	44.5 (30.0–61.3)	74.9 (56.3–97.5)
Bhutan	YLDs Rate: 2015	6,696.0 (5,098.1–8,477.6)	1,441.2 (1,027.4–1,938.2)	750.5 (510.8–1,049.3)	308.2 (203.3–430.1)	164.4 (117.8–220.2)	575.7 (415.5–757.8)	119.3 (70.8–195.4)	81.1 (52.6–113.3)	126.9 (85.7–173.7)	40.4 (27.9–55.1)	71.2 (54.1–91.5)
Bhutan	YLDs Rate: Cumulative change (%)	-2.3 (-5.0 to 0.3)	-7.7 (-14.1 to -1.3)	1.2 (-0.8 to 3.3)	-7.4 (-17.2 to 3.0)	-9.2 (-17.1 to -1.3)	98.7 (84.0–111.4)	51.2 (4.4–129.9)	36.6 (-3.5 to 85.3)	-19.0 (-39.3 to 6.2)	-8.4 (-23.7 to 12.7)	-4.6 (-16.5 to 8.5)
India	Prevalence Number: 1990	397,685,003 (397,195,290–398,189,990)	181,485,531 (179,695,197–183,193,329)	125,446,672 (124,378,589–126,599,362)	24,319,307 (20,418,355–28,464,948)	146,198,677 (145,111,266–147,400,743)	6,284,160 (5,936,858–6,717,524)	3,989,372 (3,696,285–4,305,085)	10,349,343 (10,013,842–10,694,446)	1,459,549 (1,306,543–1,642,410)	35,135,531 (26,974,253–43,672,547)	2,670,437 (2,291,561–3,090,555)

© 2017 Global Burden of Disease Child and Adolescent Health Collaboration. JAMA Pediatrics.

eTable 3. Prevalent Cases, Rates (per 100,000 population), Years Lived with Disability (YLDs), and Cumulative Percent Change with 95% Uncertainty Interval (UI) for the Top 10 Global Causes of YLDs in Children and Adolescents in 195 Countries and Territories, Aged 0 to 19 Years, Both Sexes, 1990 and 2015. *Note: best viewed by enlarging in browser.*

Location	Measure	All causes	Iron-deficiency anemia	Skin and subcutaneous diseases	Asthma	Hemoglobinopathies and hemolytic anemias	Diarrheal diseases	Congenital anomalies	Protein-energy malnutrition	Epilepsy	Malaria	Neonatal preterm birth complications
India	Prevalence Number: 2015	472,123,612 (471,375,744–472,831,985)	196,176,842 (193,955,453–198,283,537)	159,029,380 (157,769,045–160,360,771)	20,628,416 (17,189,869–24,342,521)	167,560,654 (166,168,760–168,848,440)	5,523,875 (5,239,104–5,835,084)	6,215,027 (5,803,671–6,643,046)	7,824,704 (7,438,088–8,224,208)	1,742,883 (1,522,347–1,989,607)	14,530,115 (9,919,897–20,750,281)	3,594,244 (3,146,868–4,076,208)
India	Prevalence Number: Cumulative change (%)	18.7 (18.5–18.9)	8.1 (6.5–9.7)	26.8 (25.5–28.0)	-15.2 (-18.3 to -12.1)	14.6 (13.6–15.6)	-12.1 (-15.2 to -9.5)	56.1 (43.8–68.6)	-24.4 (-28.7 to -19.8)	19.4 (11.3–27.2)	-58.9 (-66.9 to -47.2)	34.9 (25.9–44.3)
India	Prevalence Rate: 1990	95,252.3 (95,137.6–95,367.5)	42,990.4 (42,590.5–43,383.0)	30,820.9 (30,557.5–31,097.6)	5,834.1 (4,905.0–6,810.4)	34,955.5 (34,697.6–35,240.5)	1,427.8 (1,350.9–1,522.8)	949.5 (879.3–1,024.9)	2,240.6 (2,168.0–2,315.2)	349.7 (312.8–393.6)	8,283.0 (6,380.7–10,296.0)	607.8 (521.7–703.6)
India	Prevalence Rate: 2015	94,098.8 (93,948.0–94,240.1)	39,441.4 (38,978.8–39,882.1)	31,362.5 (31,116.8–31,618.1)	4,088.7 (3,406.8–4,819.4)	33,427.2 (33,149.9–33,685.2)	1,136.2 (1,076.2–1,200.6)	1,241.3 (1,159.3–1,326.9)	1,660.6 (1,578.5–1,745.4)	346.3 (302.9–395.2)	2,921.9 (1,988.1–4,186.6)	730.7 (640.2–828.8)
India	Prevalence Rate: Cumulative change (%)	-1.2 (-1.4 to -1.0)	-8.3 (-9.6 to -6.9)	1.8 (0.8–2.7)	-29.9 (-32.5 to -27.5)	-4.4 (-5.2 to -3.6)	-20.4 (-23.0 to -18.1)	31.0 (20.7–41.6)	-25.9 (-30.1 to -21.4)	-1.0 (-7.5 to 5.3)	-64.9 (-72.0 to -54.4)	20.5 (12.3–28.7)
India	YLDs Number: 1990	32,809,677 (24,856,424–41,955,063)	8,867,864 (6,487,175–11,736,220)	2,683,590 (1,817,759–3,764,231)	1,095,271 (725,333–1,521,893)	1,095,350 (800,436–1,440,804)	1,009,518 (731,091–1,331,280)	375,553 (235,464–559,516)	1,269,181 (872,323–1,694,973)	546,348 (393,992–699,460)	736,170 (526,387–987,619)	327,446 (250,823–416,230)
India	YLDs Number: 2015	34,564,580 (26,327,075–43,910,799)	8,702,350 (6,308,584–11,537,105)	3,335,206 (2,255,662–4,718,148)	932,704 (616,264–1,301,430)	1,175,690 (854,402–1,555,701)	893,433 (643,166–1,173,999)	722,875 (449,987–1,074,852)	965,153 (667,662–1,278,991)	494,926 (349,124–642,249)	312,758 (222,859–422,751)	558,153 (436,191–701,084)
India	YLDs Number: Cumulative change (%)	5.4 (3.1–7.6)	-1.9 (-5.3 to 1.3)	24.2 (22.1–26.5)	-14.9 (-18.0 to -11.7)	7.3 (3.8–10.6)	-11.5 (-14.6 to -8.8)	94.2 (52.8–147.4)	-23.9 (-28.1 to -19.3)	-9.4 (-16.7 to -2.1)	-57.5 (-60.1 to -54.2)	70.8 (58.2–83.8)
India	YLDs Rate: 1990	7,872.3 (5,969.6–10,055.0)	2,076.7 (1,517.3–2,749.3)	651.6 (441.2–916.2)	262.7 (174.1–363.2)	257.4 (188.1–338.6)	229.4 (166.1–302.0)	89.4 (56.0–133.3)	274.8 (188.9–366.9)	130.9 (93.9–167.5)	172.9 (123.6–231.9)	76.6 (58.6–97.3)
India	YLDs Rate: 2015	6,881.8 (5,238.5–8,748.0)	1,755.6 (1,273.0–2,327.9)	660.8 (447.4–933.0)	184.9 (121.9–258.4)	236.0 (171.6–312.3)	183.8 (132.4–241.7)	144.4 (89.9–214.8)	204.8 (141.7–271.4)	98.4 (69.3–127.4)	62.7 (44.6–84.8)	111.8 (87.4–140.4)
India	YLDs Rate: Cumulative change (%)	-12.5 (-14.3 to -11.0)	-15.5 (-18.2 to -13.0)	1.4 (0.0–2.8)	-29.7 (-32.2 to -27.2)	-8.3 (-11.3 to -5.6)	-19.9 (-22.6 to -17.5)	63.1 (28.2–107.8)	-25.4 (-29.5 to -20.9)	-24.9 (-31.0 to -18.9)	-63.7 (-65.9 to -60.9)	46.1 (35.4–57.5)
Nepal	Prevalence Number: 1990	9,418,738 (9,354,368–9,492,011)	2,869,072 (2,712,009–3,106,412)	3,285,236 (3,182,832–3,389,700)	809,949 (670,996–960,416)	2,071,394 (1,385,344–3,086,684)	197,421 (182,784–211,255)	77,821 (60,596–102,140)	140,844 (111,569–174,561)	36,891 (29,106–44,706)	18,120 (9,413–31,076)	58,767 (48,491–72,566)
Nepal	Prevalence Number: 2015	11,832,756 (11,743,517–11,941,890)	2,926,988 (2,763,636–3,039,291)	4,534,568 (4,389,190–4,680,912)	977,439 (792,566–1,185,480)	2,453,205 (1,647,017–3,722,568)	251,142 (235,765–267,954)	127,098 (103,920–166,250)	129,930 (103,895–158,208)	52,871 (42,008–64,449)	18,903 (10,260–32,255)	77,591 (63,830–94,412)
Nepal	Prevalence Number: Cumulative change (%)	25.6 (24.3–27.0)	2.2 (-7.7 to 11.2)	38.1 (32.3–43.9)	20.9 (6.3–37.1)	24.2 (-29.6 to 96.6)	27.3 (21.8–33.1)	66.9 (19.9–125.0)	-6.0 (-30.5 to 23.9)	44.9 (11.9–80.8)	5.1 (-7.6 to 17.4)	33.3 (6.6–60.9)
Nepal	Prevalence Rate: 1990	95,802.2 (95,195.6–96,489.9)	28,493.6 (27,131.3–30,562.9)	34,647.3 (33,605.5–35,725.5)	8,248.4 (6,886.5–9,741.7)	20,992.6 (14,039.7–31,279.5)	1,837.2 (1,707.3–1,958.5)	780.8 (606.9–1,023.2)	1,214.3 (961.8–1,505.1)	375.9 (296.4–457.2)	176.8 (92.6–297.2)	543.5 (444.2–681.1)
Nepal	Prevalence Rate: 2015	93,616.3 (92,880.6–94,496.1)	23,810.0 (22,338.0–24,860.1)	35,025.2 (33,908.8–36,177.0)	7,641.9 (6,182.9–9,276.7)	19,472.1 (13,066.3–29,542.4)	2,153.4 (2,018.5–2,303.6)	1,012.2 (827.6–1,323.5)	1,204.8 (963.3–1,467.0)	416.2 (330.8–507.1)	154.4 (83.2–266.3)	653.2 (538.3–791.3)
Nepal	Prevalence Rate: Cumulative change (%)	-2.3 (-3.3 to -1.2)	-16.3 (-24.2 to -8.8)	1.1 (-3.1 to 5.4)	-7.3 (-18.2 to 4.6)	-2.7 (-44.8 to 53.9)	17.3 (12.3–22.2)	32.5 (-4.9 to 78.9)	1.0 (-25.2 to 33.2)	11.9 (-13.5 to 39.1)	-12.6 (-21.8 to -2.7)	21.5 (-4.2 to 46.6)
Nepal	YLDs Number: 1990	661,042 (501,392–840,444)	110,404 (78,402–149,360)	74,503 (51,001–103,207)	36,640 (24,075–51,198)	15,609 (11,164–21,123)	32,123 (23,291–42,504)	7,036 (4,343–10,766)	17,513 (11,048–25,201)	14,098 (9,576–18,904)	559 (255–1,107)	7,064 (5,307–9,101)
Nepal	YLDs Number: 2015	874,657 (660,607–1,118,037)	109,490 (77,973–148,615)	99,389 (67,703–139,555)	44,310 (28,630–61,273)	21,636 (15,426–29,168)	40,950 (29,200–53,956)	13,697 (7,773–23,541)	16,181 (10,648–22,760)	17,579 (11,826–23,894)	590 (274–1,171)	10,005 (7,562–12,725)
Nepal	YLDs Number: Cumulative change (%)	32.4 (24.5–41.1)	-0.6 (-12.7 to 11.2)	33.3 (29.6–37.0)	21.2 (6.3–37.5)	38.9 (24.7–54.3)	27.6 (21.1–34.8)	98.1 (36.0–203.4)	-5.8 (-30.1 to 25.3)	26.3 (-6.7 to 63.9)	6.9 (-13.1 to 29.6)	42.2 (24.3–61.5)
Nepal	YLDs Rate: 1990	6,844.2 (5,201.1–8,671.9)	1,091.8 (777.9–1,465.4)	771.9 (527.5–1,075.3)	373.0 (246.9–519.0)	155.8 (111.1–209.9)	298.8 (215.4–395.5)	70.5 (43.3–108.3)	151.0 (95.3–217.3)	143.5 (96.9–192.9)	5.5 (2.5–10.6)	69.0 (51.9–89.0)
Nepal	YLDs Rate: 2015	6,878.0 (5,185.0–8,811.6)	886.4 (631.9–1,203.3)	776.7 (530.1–1,087.2)	346.5 (223.4–481.1)	171.4 (122.0–231.7)	351.3 (251.5–463.4)	109.2 (61.8–188.7)	150.0 (98.7–211.0)	138.4 (93.4–188.0)	4.8 (2.2–9.7)	80.4 (60.8–102.3)
Nepal	YLDs Rate: Cumulative change (%)	0.5 (-5.5 to 7.2)	-18.6 (-28.8 to -8.7)	0.6 (-1.6 to 2.7)	-7.0 (-18.3 to 5.5)	10.2 (-0.4 to 22.1)	17.6 (12.0–24.0)	57.7 (8.3–142.8)	1.3 (-24.8 to 34.7)	-2.2 (-27.8 to 27.0)	-11.6 (-27.7 to 7.4)	17.1 (2.3–33.0)
Pakistan	Prevalence Number: 1990	51,892,976 (51,484,150–52,329,907)	18,709,860 (17,725,845–19,497,224)	18,518,608 (17,729,001–19,398,099)	4,150,871 (3,473,376–4,893,823)	6,122,667 (5,826,195–6,405,892)	1,189,136 (1,113,257–1,263,272)	481,925 (387,272–615,249)	1,354,564 (1,166,767–1,563,912)	312,309 (249,332–378,174)	5,544,402 (4,894,016–6,264,841)	371,758 (308,915–443,550)
Pakistan	Prevalence Number: 2015	76,039,755 (75,379,645–76,692,103)	31,705,822 (29,898,382–33,931,716)	28,815,164 (27,588,224–30,110,901)	5,898,394 (4,877,824–7,044,053)	8,864,656 (8,429,614–9,290,976)	1,773,122 (1,682,256–1,874,434)	803,838 (648,529–1,018,371)	1,252,750 (1,010,433–1,521,937)	478,254 (385,931–578,605)	751,523 (476,222–1,284,488)	525,507 (436,031–624,341)
Pakistan	Prevalence Number: Cumulative change (%)	46.5 (44.9–48.2)	69.6 (58.2–85.7)	55.7 (46.7–65.4)	42.3 (26.3–56.4)	44.9 (35.2–54.3)	49.2 (42.7–55.9)	69.7 (23.8–125.6)	-6.8 (-28.3 to 18.0)	54.7 (21.1–93.5)	-86.4 (-91.4 to -76.4)	42.2 (18.4–66.8)
Pakistan	Prevalence Rate: 1990	91,286.7 (90,623.4–91,986.8)	33,310.5 (31,789.1–34,589.9)	33,918.0 (32,523.8–35,502.9)	7,350.6 (6,161.9–8,633.9)	10,743.8 (10,221.8–11,238.2)	1,836.2 (1,722.9–1,945.3)	830.8 (667.0–1,063.5)	1,902.3 (1,638.3–2,196.4)	547.4 (436.7–665.3)	8,244.8 (7,290.9–9,344.8)	589.7 (487.4–710.0)
Pakistan	Prevalence Rate: 2015	89,196.1 (88,462.3–89,913.8)	36,798.2 (34,782.1–39,138.8)	34,280.0 (32,840.6–35,835.2)	6,976.6 (5,777.7–8,310.8)	10,369.8 (9,863.2–10,866.0)	1,964.3 (1,866.7–2,073.7)	935.9 (754.5–1,188.1)	1,328.6 (1,071.6–1,614.1)	561.3 (451.8–679.8)	869.3 (554.0–1,461.4)	589.2 (488.3–702.8)
Pakistan	Prevalence Rate: Cumulative change (%)	-2.3 (-3.3 to -1.3)	10.5 (3.1–20.1)	1.1 (-4.7 to 7.4)	-5.0 (-15.7 to 4.4)	-3.4 (-9.9 to 2.8)	7.0 (2.6–11.6)	14.7 (-16.6 to 52.9)	-29.6 (-45.9 to -10.9)	3.6 (-19.0 to 29.8)	-89.5 (-93.3 to -81.9)	0.6 (-17.4 to 19.6)
Pakistan	YLDs Number: 1990	3,896,252 (2,947,891–4,970,622)	753,105 (536,385–1,013,993)	447,056 (306,957–614,185)	187,528 (124,708–256,745)	100,288 (70,556–135,604)	193,844 (141,066–257,164)	42,441 (26,873–64,393)	168,833 (113,741–232,843)	120,076 (80,419–162,756)	38,261 (23,300–55,144)	53,306 (40,334–67,028)
Pakistan	YLDs Number: 2015	5,824,316 (4,437,771–7,376,118)	1,271,762 (903,641–1,729,886)	682,343 (468,719–938,991)	266,909 (174,670–373,130)	155,235 (110,458–209,095)	288,233 (208,672–379,305)	88,823 (52,079–151,289)	155,562 (99,092–215,871)	170,298 (119,298–228,957)	9,979 (6,709–13,650)	74,159 (56,534–94,632)
Pakistan	YLDs Number: Cumulative change (%)	49.5 (44.3–55.6)	68.9 (54.2–86.2)	52.6 (49.3–55.9)	42.5 (25.9–57.1)	55.0 (39.5–72.6)	48.8 (41.2–56.6)	113.1 (43.7–231.0)	-7.2 (-28.7 to 18.1)	44.0 (10.1–85.7)	-72.9 (-80.9 to -60.6)	39.3 (25.0–54.4)
Pakistan	YLDs Rate: 1990	6,979.0 (5,284.7–8,871.0)	1,340.3 (953.5–1,809.9)	799.3 (549.2–1,101.0)	331.9 (221.2–453.6)	176.5 (125.1–238.3)	299.0 (217.6–397.8)	73.0 (46.1–110.5)	237.1 (159.8–327.0)	210.2 (140.3–286.4)	58.9 (36.4–84.1)	89.5 (67.7–113.0)
Pakistan	YLDs Rate: 2015	6,855.6 (5,225.5–8,684.0)	1,479.5 (1,052.2–2,009.8)	805.2 (552.7–1,108.5)	315.7 (207.4–438.4)	181.7 (129.9–244.4)	319.2 (231.1–419.6)	103.3 (60.7–175.6)	165.0 (105.1–229.0)	199.8 (139.7–269.1)	11.6 (7.8–15.9)	85.7 (65.4–109.5)
Pakistan	YLDs Rate: Cumulative change (%)	-1.7 (-5.1 to 1.9)	10.4 (0.4–21.4)	0.7 (-1.3 to 2.9)	-4.8 (-16.2 to 4.7)	3.1 (-7.8 to 14.2)	6.9 (1.4–12.2)	44.0 (-2.4 to 123.9)	-29.9 (-46.2 to -10.8)	-3.5 (-26.2 to 24.6)	-79.6 (-85.4 to -71.1)	-4.1 (-13.9 to 6.4)
Sub-Saharan Africa	Prevalence Number: 1990	266,296,589 (266,054,818–266,524,665)	94,473,579 (92,921,515–95,906,400)	95,483,388 (94,148,253–96,751,971)	21,946,704 (18,544,591–25,744,423)	98,940,391 (97,814,229–100,041,488)	6,745,409 (6,255,742–7,257,266)	2,661,981 (2,419,172–2,955,164)	4,414,609 (4,010,275–4,883,182)	546,947 (472,603–636,754)	80,011,833 (73,172,600–87,482,902)	1,440,510 (1,166,992–1,731,461)
Sub-Saharan Africa	Prevalence Number: 2015	494,607,534 (494,002,063–495,173,077)	153,015,390 (150,566,124–155,227,626)	183,215,777 (180,664,697–185,543,723)	38,031,255 (31,849,088–45,145,603)	173,320,133 (171,429,115–175,332,421)	8,102,380 (7,640,654–8,656,899)	5,789,565 (5,309,800–6,302,914)	6,548,940 (5,980,933–7,200,288)	1,077,077 (920,545–1,259,838)	159,302,462 (138,571,479–182,376,147)	2,719,936 (2,220,393–3,243,068)
Sub-Saharan Africa	Prevalence Number: Cumulative change (%)	85.7 (85.5–86.0)	62.0 (58.0–65.8)	91.9 (89.2–94.7)	73.2 (65.3–82.0)	75.2 (72.9–77.5)	20.2 (16.1–24.4)	118.0 (97.7–140.3)	48.9 (29.8–70.7)	97.5 (72.6–125.7)	99.0 (82.7–116.4)	89.2 (75.6–102.7)
Sub-Saharan Africa	Prevalence Rate: 1990	97,272.4 (97,191.5–97,350.0)	33,211.6 (32,726.4–33,651.3)	36,423.3 (35,930.5–36,880.0)	8,277.4 (7,029.5–9,674.5)	36,011.2 (35,608.0–36,411.5)	2,165.0 (2,016.1–2,321.3)	954.5 (865.7–1,060.4)	1,303.7 (1,184.7–1,442.3)	197.7 (171.2–228.9)	28,727.6 (26,376.0–31,341.2)	452.2 (366.3–545.1)
Sub-Saharan Africa	Prevalence Rate: 2015	96,243.7 (96,131.0–96,348.9)	28,808.1 (28,373.2–29,182.1)	36,818.5 (36,317.1–37,277.3)	7,548.9 (6,323.7–8,923.4)	33,592.4 (33,225.1–33,975.9)	1,462.6 (1,381.8–1,556.4)	1,114.7 (1,022.1–1,213.7)	1,089.9 (995.5–1,198.2)	208.4 (178.1–243.4)	30,876.5 (26,796.3–35,376.2)	482.2 (393.2–574.4)
Sub-Saharan Africa	Prevalence Rate: Cumulative change (%)	-1.1 (-1.2 to -0.9)	-13.3 (-15.2 to -11.4)	1.1 (-0.3 to 2.5)	-8.8 (-12.9 to -4.3)	-6.7 (-7.9 to -5.5)	-32.4 (-34.6 to -30.2)	17.1 (6.0–29.2)	-16.1 (-26.8 to -3.8)	5.7 (-7.6 to 20.7)	7.4 (-1.3 to 16.6)	6.9 (-1.8 to 15.1)
Sub-Saharan Africa	YLDs Number: 1990	21,479,961 (16,084,239–27,681,364)	4,348,308 (3,171,742–5,751,232)	2,032,273 (1,399,430–2,821,582)	987,554 (656,455–1,353,730)	533,567 (388,135–702,175)	1,087,120 (790,188–1,442,113)	196,419 (129,251–290,617)	538,744 (374,371–732,557)	221,266 (157,911–287,324)	1,023,540 (747,806–1,342,628)	77,329 (58,176–99,294)
Sub-Saharan Africa	YLDs Number: 2015	36,858,785 (27,679,514–47,478,817)	6,570,738 (4,765,285–8,729,945)	3,877,618 (2,665,810–5,401,779)	1,717,004 (1,141,026–2,347,190)	922,662 (669,562–1,233,688)	1,312,547 (951,924–1,724,137)	482,258 (327,772–677,574)	806,963 (558,555–1,092,004)	397,452 (278,604–523,796)	1,932,609 (1,410,325–2,545,608)	168,999 (126,057–216,281)
Sub-Saharan Africa	YLDs Number: Cumulative change (%)	71.5 (67.2–77.3)	51.1 (43.9–57.4)	90.7 (89.5–92.1)	73.8 (66.0–82.6)	72.8 (63.6–80.4)	20.8 (16.6–25.2)	147.6 (123.1–176.3)	50.3 (30.9–71.7)	80.1 (55.9–108.6)	88.9 (81.2–96.2)	118.6 (107.0–129.0)
Sub-Saharan Africa	YLDs Rate: 1990	7,938.9 (5,941.6–10,208.3)	1,497.6 (1,090.3–1,976.3)	759.3 (521.8–1,058.0)	372.3 (247.8–506.9)	188.1 (137.1–247.8)	348.8 (253.4–461.0)	70.1 (46.1–104.4)	159.1 (110.5–216.4)	79.9 (57.3–103.6)	363.2 (265.2–474.5)	25.8 (19.3–32.9)
Sub-Saharan Africa	YLDs Rate: 2015	7,267.3 (5,454.4–9,344.9)	1,222.9 (886.7–1,623.0)	766.7 (526.3–1,071.4)	340.7 (225.7–464.7)	174.7 (126.8–233.6)	236.8 (171.7–310.3)	92.5 (62.9–130.1)	134.3 (93.0–181.7)	76.8 (54.0–101.1)	369.7 (270.4–487.8)	31.3 (23.4–40.1)
Sub-Saharan Africa	YLDs Rate: Cumulative change (%)	-8.5 (-10.7 to -5.4)	-18.4 (-21.9 to -15.2)	0.9 (0.4–1.6)	-8.5 (-12.7 to -3.8)	-7.2 (-11.8 to -3.5)	-32.1 (-34.3 to -29.7)	33.3 (19.5–49.1)	-15.3 (-26.2 to -3.3)	-3.6 (-16.7 to 11.4)	1.8 (-2.1 to 5.5)	21.6 (15.0–27.5)
Southern Sub-Saharan Africa	Prevalence Number: 1990	25,653,267 (25,586,301–25,713,814)	7,273,847 (7,083,864–7,471,800)	9,526,534 (9,391,473–9,670,557)	3,535,751 (2,980,005–4,127,907)	6,308,682 (6,182,894–6,441,735)	442,115 (404,534–485,265)	321,923 (289,656–358,720)	251,805 (207,524–302,164)	64,479 (54,557–75,878)	430,128 (343,608–575,415)	156,615 (130,718–185,260)
Southern Sub-Saharan Africa	Prevalence Number: 2015	29,695,592 (29,617,202–29,774,072)	8,012,341 (7,796,478–8,230,525)	11,355,636 (11,195,054–11,517,617)	3,433,792 (2,908,537–4,014,151)	6,406,425 (6,269,692–6,535,846)	396,166 (373,718–419,584)	429,423 (389,800–471,669)	160,371 (133,227–190,813)	66,419 (55,610–77,758)	650,182 (454,497–1,018,639)	192,464 (161,412–225,633)
Southern Sub-Saharan Africa	Prevalence Number: Cumulative change (%)	15.8 (15.3–16.2)	10.2 (4.9–15.5)	19.2 (17.3–21.2)	-2.8 (-6.9 to 1.3)	1.6 (-1.0 to 3.9)	-10.3 (-14.8 to -6.2)	33.8 (19.1–48.7)	-35.4 (-51.0 to -15.6)	3.6 (-14.7 to 23.9)	49.2 (31.1–76.2)	23.1 (13.1–33.1)

© 2017 Global Burden of Disease Child and Adolescent Health Collaboration. JAMA Pediatrics.

eTable 3. Prevalent Cases, Rates (per 100,000 population), Years Lived with Disability (YLDs), and Cumulative Percent Change with 95% Uncertainty Interval (UI) for the Top 10 Global Causes of YLDs in Children and Adolescents in 195 Countries and Territories, Aged 0 to 19 Years, Both Sexes, 1990 and 2015. *Note: best viewed by enlarging in browser.*

Location	Measure	All causes	Iron-deficiency anemia	Skin and subcutaneous diseases	Asthma	Hemoglobinopathies and hemolytic anemias	Diarrheal diseases	Congenital anomalies	Protein-energy malnutrition	Epilepsy	Malaria	Neonatal preterm birth complications
Southern Sub-Saharan Africa	Prevalence Rate: 1990	94,974.4 (94,734.9–95,190.7)	26,571.0 (25,918.2–27,252.9)	35,947.0 (35,442.2–36,463.3)	13,049.4 (11,006.2–15,219.3)	23,299.7 (22,835.6–23,794.8)	1,547.9 (1,419.2–1,696.3)	1,184.2 (1,065.1–1,320.1)	855.9 (705.4–1,027.1)	239.9 (203.0–282.6)	1,570.6 (1,258.2–2,076.1)	559.3 (467.7–661.7)
Southern Sub-Saharan Africa	Prevalence Rate: 2015	94,467.4 (94,218.2–94,712.1)	25,409.5 (24,760.3–26,062.9)	36,284.6 (35,768.3–36,808.0)	11,093.8 (9,393.1–12,984.8)	20,355.7 (19,920.6–20,765.6)	1,220.1 (1,152.8–1,290.6)	1,361.8 (1,235.4–1,495.7)	477.7 (397.0–568.3)	212.2 (177.4–248.4)	2,064.9 (1,443.9–3,199.3)	596.9 (501.0–699.7)
Southern Sub-Saharan Africa	Prevalence Rate: Cumulative change (%)	-0.5 (-0.9 to -0.2)	-4.3 (-8.7 to -0.0)	0.9 (-0.6 to 2.6)	-15.0 (-18.4 to -11.3)	-12.6 (-14.8 to -10.6)	-21.1 (-24.9 to -17.6)	15.3 (2.7–28.2)	-43.4 (-57.1 to -26.1)	-11.0 (-26.7 to 6.4)	29.8 (13.7–53.3)	6.9 (-1.9 to 15.9)
Southern Sub-Saharan Africa	YLDs Number: 1990	1,723,496 (1,306,822–2,192,139)	279,726 (199,794–375,615)	211,413 (146,006–295,051)	160,155 (105,818–219,257)	19,081 (13,725–25,623)	72,245 (52,417–96,300)	24,191 (16,589–34,319)	31,327 (20,316–43,601)	25,791 (18,021–34,191)	10,795 (7,588–14,631)	16,041 (12,030–20,790)
Southern Sub-Saharan Africa	YLDs Number: 2015	2,006,303 (1,526,346–2,540,415)	302,072 (218,834–405,978)	248,647 (171,389–345,796)	155,539 (103,552–211,665)	19,923 (14,305–26,737)	64,622 (46,731–85,128)	42,545 (28,484–59,821)	19,996 (13,161–27,873)	20,611 (14,383–27,395)	13,685 (9,700–18,332)	20,124 (15,089–26,387)
Southern Sub-Saharan Africa	YLDs Number: Cumulative change (%)	16.5 (14.3–18.7)	8.0 (1.7–14.5)	17.6 (16.3–18.8)	-2.8 (-7.0 to 1.4)	4.4 (-1.8 to 10.3)	-10.4 (-15.1 to -6.2)	76.4 (50.6–108.3)	-35.3 (-51.1 to -15.6)	-19.5 (-34.9 to -2.9)	26.9 (17.3–36.2)	25.5 (19.6–31.7)
Southern Sub-Saharan Africa	YLDs Rate: 1990	6,444.5 (4,891.4–8,195.8)	1,015.6 (726.1–1,365.2)	787.9 (542.5–1,100.9)	591.0 (391.9–808.2)	69.4 (49.9–93.2)	252.9 (183.5–337.0)	88.7 (60.9–126.0)	106.5 (69.1–148.2)	95.9 (66.8–127.1)	39.3 (27.7–53.2)	58.3 (43.8–75.4)
Southern Sub-Saharan Africa	YLDs Rate: 2015	6,418.3 (4,882.8–8,126.4)	966.8 (699.7–1,297.6)	792.6 (544.9–1,102.5)	502.5 (334.3–685.9)	64.4 (46.3–86.4)	199.0 (143.9–262.4)	134.7 (90.2–189.5)	59.6 (39.2–83.0)	65.8 (45.9–87.5)	44.1 (31.3–59.0)	63.5 (47.5–83.3)
Southern Sub-Saharan Africa	YLDs Rate: Cumulative change (%)	-0.4 (-2.1 to 1.5)	-4.8 (-10.1 to 0.8)	0.6 (-0.3 to 1.5)	-14.9 (-18.5 to -11.3)	-7.2 (-12.6 to -2.2)	-21.2 (-25.2 to -17.6)	52.3 (30.1–79.8)	-43.3 (-57.2 to -26.0)	-30.9 (-44.0 to -16.7)	12.4 (4.1–20.4)	8.9 (3.7–14.3)
Botswana	Prevalence Number: 1990	726,642 (721,404–730,842)	289,874 (272,448–307,017)	266,495 (256,042–276,489)	93,291 (77,639–110,644)	182,826 (173,900–192,389)	11,956 (11,017–13,152)	10,294 (8,253–12,994)	9,216 (4,941–15,071)	1,797 (1,167–2,495)	21,981 (13,787–37,605)	5,173 (4,218–6,391)
Botswana	Prevalence Number: 2015	883,025 (877,483–888,679)	326,200 (304,459–355,410)	341,186 (328,433–352,889)	107,420 (88,627–127,904)	193,667 (183,054–203,391)	10,951 (10,125–11,837)	13,328 (10,874–16,935)	6,678 (3,581–11,035)	1,940 (1,296–2,727)	31,844 (17,011–63,451)	5,781 (4,718–7,033)
Botswana	Prevalence Number: Cumulative change (%)	21.5 (20.5–22.6)	12.7 (1.2–28.4)	28.1 (21.9–34.5)	15.2 (6.2–23.6)	6.0 (-1.1 to 13.1)	-8.3 (-13.5 to -3.2)	31.6 (-3.2 to 71.6)	-18.3 (-66.9 to 59.2)	13.4 (-34.3 to 76.3)	43.5 (10.5–128.0)	12.5 (-7.0 to 33.0)
Botswana	Prevalence Rate: 1990	94,574.1 (93,941.4–95,070.4)	37,411.2 (35,341.1–39,294.8)	35,709.1 (34,376.7–37,000.8)	12,130.9 (10,134.9–14,405.2)	23,760.2 (22,603.0–24,990.6)	1,449.9 (1,341.1–1,588.5)	1,330.2 (1,067.1–1,680.9)	1,066.9 (572.1–1,744.7)	236.9 (153.4–328.9)	2,748.8 (1,752.4–4,596.5)	647.2 (526.4–802.8)
Botswana	Prevalence Rate: 2015	93,993.3 (93,410.7–94,590.3)	34,551.5 (32,323.1–37,527.6)	36,555.1 (35,191.9–37,821.9)	11,568.1 (9,538.0–13,784.3)	20,594.4 (19,469.1–21,628.9)	1,130.4 (1,047.6–1,220.8)	1,414.9 (1,154.8–1,799.6)	668.5 (358.5–1,104.6)	207.8 (138.8–292.0)	3,352.7 (1,803.9–6,653.3)	602.2 (490.7–734.2)
Botswana	Prevalence Rate: Cumulative change (%)	-0.6 (-1.4 to 0.2)	-7.5 (-16.4 to 4.3)	2.4 (-2.6 to 7.3)	-4.6 (-11.8 to 2.2)	-13.3 (-19.1 to -7.5)	-22.0 (-26.2 to -17.8)	8.1 (-20.5 to 40.9)	-29.4 (-71.4 to 37.6)	-7.9 (-46.7 to 43.8)	20.5 (-7.7 to 94.1)	-6.3 (-23.1 to 11.3)
Botswana	YLDs Number: 1990	51,922 (38,770–66,345)	11,690 (8,241–15,765)	5,962 (4,126–8,241)	4,215 (2,817–5,798)	640 (453–861)	1,947 (1,400–2,581)	837 (485–1,408)	1,148 (545–2,027)	713 (424–1,048)	281 (202–376)	586 (434–759)
Botswana	YLDs Number: 2015	62,273 (46,852–79,465)	12,696 (8,994–17,378)	7,441 (5,161–10,322)	4,858 (3,226–6,713)	685 (477–936)	1,783 (1,275–2,361)	1,462 (734–2,811)	833 (399–1,469)	539 (323–829)	393 (274–537)	651 (477–870)
Botswana	YLDs Number: Cumulative change (%)	20.0 (14.4–26.4)	9.0 (-5.0 to 27.5)	24.7 (21.9–27.5)	15.3 (6.3–24.1)	7.3 (-8.5 to 26.3)	-8.3 (-14.4 to -2.3)	86.2 (-12.8 to 260.2)	-18.0 (-67.6 to 61.9)	-20.5 (-54.9 to 27.0)	40.5 (20.1–71.5)	11.2 (0.2–22.7)
Botswana	YLDs Rate: 1990	6,866.0 (5,134.6–8,765.9)	1,493.6 (1,059.3–2,015.1)	783.0 (538.5–1,088.6)	548.0 (366.2–754.6)	83.0 (58.8–111.2)	238.0 (170.4–312.1)	107.9 (62.9–181.7)	133.0 (63.1–234.8)	94.0 (55.9–138.0)	35.3 (25.5–46.9)	74.9 (55.4–96.6)
Botswana	YLDs Rate: 2015	6,660.6 (5,018.1–8,490.9)	1,350.4 (957.1–1,841.6)	794.3 (549.9–1,101.0)	523.2 (347.6–722.7)	73.3 (51.0–99.9)	184.1 (131.4–243.4)	155.1 (78.0–297.9)	83.5 (40.0–147.1)	57.8 (34.5–89.1)	41.8 (29.2–56.8)	68.9 (50.6–92.2)
Botswana	YLDs Rate: Cumulative change (%)	-2.9 (-7.1 to 1.9)	-9.3 (-20.2 to 4.6)	1.4 (-0.5 to 3.4)	-4.5 (-11.6 to 2.9)	-11.5 (-24.2 to 3.4)	-21.9 (-27.1 to -16.9)	53.2 (-28.2 to 196.1)	-29.1 (-72.0 to 39.9)	-35.4 (-63.2 to 3.1)	18.8 (2.7–42.5)	-7.9 (-16.8 to 1.7)
Lesotho	Prevalence Number: 1990	845,104 (838,128–851,776)	252,247 (235,188–271,807)	302,033 (290,206–313,488)	107,325 (89,040–126,767)	206,735 (194,030–224,629)	17,174 (15,631–19,036)	9,324 (7,191–12,220)	7,012 (3,889–11,087)	1,860 (1,255–2,620)	0 (0–0)	6,043 (4,721–7,929)
Lesotho	Prevalence Number: 2015	968,726 (962,383–974,961)	250,273 (239,308–261,231)	362,465 (347,267–375,706)	135,839 (113,615–161,105)	208,335 (193,740–228,054)	14,862 (13,848–16,114)	10,759 (8,207–14,197)	4,499 (2,533–7,215)	2,468 (1,583–3,614)	0 (0–0)	6,178 (4,698–7,946)
Lesotho	Prevalence Number: Cumulative change (%)	14.6 (13.5–15.8)	-0.6 (-8.3 to 8.4)	20.1 (13.9–26.0)	26.7 (18.0–35.9)	0.9 (-9.0 to 11.5)	-13.4 (-18.1 to -8.9)	18.1 (-18.1 to 68.6)	-28.8 (-69.7 to 41.3)	39.1 (-22.5 to 123.8)	19.4 (-1.7 to 66.4)	3.5 (-22.0 to 32.3)
Lesotho	Prevalence Rate: 1990	95,704.3 (94,957.1–96,413.4)	27,991.7 (26,294.7–30,019.0)	35,086.6 (33,731.9–36,383.6)	12,142.6 (10,078.8–14,290.6)	23,347.8 (21,901.1–25,384.6)	1,823.6 (1,665.9–2,013.6)	1,047.9 (806.1–1,374.4)	714.6 (396.5–1,129.9)	212.9 (142.9–301.2)	0.0 (0.0–0.0)	651.8 (506.9–858.9)
Lesotho	Prevalence Rate: 2015	95,527.4 (94,912.1–96,142.7)	24,734.4 (23,701.1–25,794.7)	35,653.5 (34,148.1–36,955.2)	13,547.9 (11,327.2–16,072.2)	20,540.4 (19,102.5–22,487.5)	1,449.2 (1,351.0–1,570.2)	1,059.2 (807.7–1,398.0)	434.6 (244.8–696.7)	243.5 (156.2–356.8)	0.0 (0.0–0.0)	597.3 (452.5–770.7)
Lesotho	Prevalence Rate: Cumulative change (%)	-0.2 (-1.2 to 0.8)	-11.5 (-18.1 to -4.0)	1.7 (-3.4 to 6.6)	11.7 (4.4–19.6)	-11.9 (-20.6 to -2.7)	-20.4 (-24.7 to -16.5)	3.5 (-28.3 to 48.0)	-32.5 (-71.3 to 33.9)	19.9 (-33.2 to 92.7)	7.8 (-12.0 to 50.9)	-7.1 (-30.9 to 19.7)
Lesotho	YLDs Number: 1990	55,481 (41,960–70,855)	9,563 (6,740–12,911)	6,816 (4,725–9,444)	4,861 (3,226–6,685)	709 (504–947)	2,804 (2,026–3,710)	666 (415–1,029)	869 (414–1,522)	735 (436–1,114)	0 (0–0)	533 (391–688)
Lesotho	YLDs Number: 2015	68,160 (51,710–86,007)	9,179 (6,538–12,405)	7,965 (5,497–10,974)	6,147 (4,082–8,355)	722 (511–985)	2,421 (1,755–3,193)	888 (511–1,453)	558 (277–971)	803 (460–1,232)	0 (0–0)	530 (388–699)
Lesotho	YLDs Number: Cumulative change (%)	23.0 (18.5–27.8)	-3.8 (-14.2 to 8.1)	16.8 (13.9–19.6)	26.6 (18.0–35.8)	2.0 (-8.2 to 11.8)	-13.5 (-19.0 to -7.9)	38.4 (-17.6 to 123.7)	-28.7 (-69.8 to 40.7)	14.9 (-36.7 to 89.9)	17.5 (-4.3 to 62.5)	-0.4 (-12.8 to 12.3)
Lesotho	YLDs Rate: 1990	6,372.8 (4,832.3–8,132.9)	1,055.6 (745.5–1,423.2)	778.4 (538.6–1,081.9)	549.9 (364.8–755.3)	78.8 (55.9–105.2)	297.7 (215.3–392.8)	74.5 (46.5–115.4)	88.6 (42.2–155.1)	84.2 (50.0–127.6)	0.0 (0.0–0.0)	58.8 (43.1–75.9)
Lesotho	YLDs Rate: 2015	6,720.0 (5,096.4–8,474.4)	913.4 (650.1–1,232.9)	785.8 (543.4–1,083.0)	613.2 (406.4–834.2)	72.2 (51.1–98.5)	236.1 (171.2–311.3)	87.4 (50.3–142.9)	53.9 (26.8–93.8)	79.2 (45.4–121.8)	0.0 (0.0–0.0)	52.0 (38.1–68.6)
Lesotho	YLDs Rate: Cumulative change (%)	5.5 (1.8–9.4)	-13.3 (-22.1 to -3.0)	0.9 (-1.2 to 3.1)	11.6 (4.1–19.8)	-8.3 (-17.4 to -0.2)	-20.6 (-25.5 to -15.7)	21.7 (-27.3 to 96.4)	-32.4 (-71.4 to 33.4)	-0.9 (-45.5 to 65.0)	6.2 (-13.0 to 47.7)	-11.3 (-22.5 to 0.1)
Namibia	Prevalence Number: 1990	725,079 (720,365–729,561)	287,145 (272,318–301,751)	270,566 (260,857–280,051)	95,583 (80,282–112,143)	185,220 (175,439–194,604)	16,865 (15,493–18,404)	9,216 (7,214–11,674)	10,395 (6,732–14,496)	1,812 (1,223–2,531)	54,683 (36,566–92,733)	4,842 (3,861–6,064)
Namibia	Prevalence Number: 2015	1,070,227 (1,062,575–1,077,287)	361,602 (347,331–378,383)	416,457 (402,380–430,854)	131,633 (108,562–157,237)	238,087 (225,812–250,239)	17,060 (15,816–18,394)	15,182 (12,220–19,057)	11,171 (7,358–16,005)	2,489 (1,678–3,531)	84,239 (53,629–139,653)	7,121 (5,807–8,826)
Namibia	Prevalence Number: Cumulative change (%)	47.6 (46.3–49.0)	26.0 (17.8–33.7)	54.0 (47.0–61.3)	37.8 (27.0–49.8)	28.7 (19.8–37.4)	1.2 (-3.8 to 6.2)	67.7 (21.7–122.2)	13.7 (-39.8 to 83.0)	43.9 (-18.4 to 127.2)	53.4 (35.7–90.6)	48.4 (20.6–80.5)
Namibia	Prevalence Rate: 1990	93,428.5 (92,850.7–93,958.7)	36,331.1 (34,621.0–38,140.8)	35,774.3 (34,513.5–37,008.0)	12,478.0 (10,493.3–14,599.1)	23,796.1 (22,541.2–24,998.8)	1,993.6 (1,838.9–2,166.6)	1,175.3 (915.1–1,493.8)	1,159.7 (751.0–1,617.0)	237.0 (159.6–329.6)	6,880.3 (4,653.8–11,340.3)	586.2 (464.9–741.1)
Namibia	Prevalence Rate: 2015	92,273.9 (91,628.4–92,860.3)	30,931.9 (29,804.4–32,299.8)	36,280.0 (35,072.7–37,519.3)	11,497.0 (9,473.1–13,713.4)	20,491.4 (19,435.2–21,534.9)	1,406.0 (1,305.3–1,512.8)	1,303.0 (1,046.1–1,637.0)	885.2 (583.1–1,268.1)	215.9 (145.6–306.3)	7,185.0 (4,600.7–11,734.7)	593.4 (482.6–736.2)
Namibia	Prevalence Rate: Cumulative change (%)	-1.2 (-2.1 to -0.4)	-14.8 (-20.1 to -9.8)	1.5 (-3.1 to 6.2)	-7.8 (-15.1 to 0.3)	-13.8 (-19.7 to -8.0)	-29.4 (-32.9 to -26.0)	12.8 (-18.2 to 50.0)	-19.3 (-57.2 to 29.9)	-4.5 (-45.8 to 50.6)	3.8 (-7.8 to 28.2)	2.3 (-17.7 to 25.3)
Namibia	YLDs Number: 1990	52,462 (39,614–66,579)	11,427 (8,108–15,452)	6,034 (4,178–8,324)	4,324 (2,867–5,999)	618 (439–831)	2,741 (1,993–3,668)	678 (413–1,077)	1,288 (725–2,010)	719 (433–1,089)	540 (364–765)	547 (413–718)
Namibia	YLDs Number: 2015	73,996 (55,980–93,561)	13,720 (9,736–18,357)	9,158 (6,298–12,717)	5,962 (3,967–8,207)	771 (538–1,039)	2,783 (2,004–3,665)	1,477 (793–2,720)	1,389 (801–2,167)	710 (419–1,093)	661 (462–905)	807 (600–1,063)
Namibia	YLDs Number: Cumulative change (%)	41.1 (36.6–46.0)	20.2 (10.9–29.6)	51.7 (48.6–54.7)	37.9 (26.8–50.2)	24.7 (11.9–36.8)	1.6 (-4.2 to 7.4)	128.5 (22.0–305.0)	14.4 (-39.7 to 83.9)	3.7 (-42.1 to 65.1)	23.9 (-1.0 to 52.5)	47.7 (33.1–62.8)
Namibia	YLDs Rate: 1990	6,819.6 (5,155.7–8,657.1)	1,441.0 (1,022.6–1,943.9)	785.7 (542.0–1,082.7)	564.4 (375.2–781.6)	78.8 (56.1–106.1)	323.9 (237.1–432.5)	86.2 (52.7–137.1)	143.8 (80.9–224.3)	94.1 (56.6–142.5)	68.2 (46.3–96.0)	68.8 (51.8–90.1)
Namibia	YLDs Rate: 2015	6,419.6 (4,864.4–8,110.3)	1,179.1 (839.5–1,579.4)	793.2 (545.6–1,102.7)	520.7 (345.0–716.9)	66.7 (46.7–90.0)	229.3 (165.6–301.5)	126.6 (68.0–233.1)	110.1 (63.5–171.8)	61.6 (36.4–94.9)	57.0 (40.0–77.9)	68.8 (51.2–90.5)
Namibia	YLDs Rate: Cumulative change (%)	-5.8 (-8.7 to -2.8)	-18.1 (-24.0 to -12.2)	1.0 (-1.0 to 2.9)	-7.7 (-15.1 to 0.6)	-15.2 (-23.8 to -7.3)	-29.2 (-33.1 to -25.1)	54.0 (-17.7 to 173.4)	-18.7 (-57.2 to 30.6)	-31.2 (-61.6 to 9.5)	-15.4 (-32.1 to 3.5)	0.1 (-9.8 to 10.9)
South Africa	Prevalence Number: 1990	17,224,437 (17,179,287–17,270,365)	4,741,798 (4,598,143–4,899,577)	6,454,468 (6,363,176–6,547,419)	2,630,314 (2,209,828–3,076,430)	4,096,550 (4,009,842–4,192,812)	268,139 (245,155–294,250)	214,579 (190,572–244,942)	196,298 (154,349–245,729)	47,387 (39,404–56,280)	156,836 (110,923–235,477)	93,777 (77,989–111,023)
South Africa	Prevalence Number: 2015	18,565,418 (18,505,605–18,623,262)	4,517,368 (4,389,842–4,647,873)	7,183,106 (7,078,951–7,285,726)	2,193,766 (1,875,299–2,545,477)	3,821,673 (3,729,845–3,910,580)	203,257 (191,182–216,342)	283,803 (255,365–314,882)	102,289 (79,668–128,624)	43,023 (35,629–51,135)	138,992 (99,659–207,276)	110,379 (92,826–129,657)
South Africa	Prevalence Number: Cumulative change (%)	7.8 (7.4–8.2)	-4.7 (-9.1 to -0.0)	11.3 (9.3–13.2)	-16.5 (-20.2 to -12.8)	-6.7 (-9.1 to -4.2)	-24.1 (-28.9 to -20.0)	32.8 (15.5–49.4)	-46.8 (-62.5 to -25.7)	-8.5 (-27.1 to 11.3)	-11.2 (-17.0 to -5.4)	17.9 (8.3–27.4)
South Africa	Prevalence Rate: 1990	95,172.9 (94,936.8–95,418.9)	26,026.5 (25,268.9–26,850.5)	36,125.7 (35,619.4–36,632.3)	14,489.6 (12,170.9–16,902.5)	22,599.7 (22,124.2–23,130.6)	1,425.0 (1,304.6–1,561.7)	1,180.4 (1,047.9–1,347.9)	1,023.5 (804.7–1,281.2)	262.5 (218.2–311.8)	855.8 (606.7–1,275.4)	505.5 (421.6–597.4)
South Africa	Prevalence Rate: 2015	94,759.0 (94,458.0–95,054.8)	23,250.7 (22,616.2–23,898.8)	36,461.8 (35,924.8–36,985.5)	11,404.2 (9,729.8–13,277.9)	19,504.0 (19,035.1–19,955.8)	1,023.8 (964.0–1,088.9)	1,447.9 (1,302.5–1,606.3)	506.0 (394.1–636.3)	219.7 (181.6–261.5)	715.7 (511.6–1,065.0)	558.5 (470.4–656.0)

© 2017 Global Burden of Disease Child and Adolescent Health Collaboration. JAMA Pediatrics.

eTable 3. Prevalent Cases, Rates (per 100,000 population), Years Lived with Disability (YLDs), and Cumulative Percent Change with 95% Uncertainty Interval (UI) for the Top 10 Global Causes of YLDs in Children and Adolescents in 195 Countries and Territories, Aged 0 to 19 Years, Both Sexes, 1990 and 2015. *Note: best viewed by enlarging in browser.*

Location	Measure	All causes	Iron-deficiency anemia	Skin and subcutaneous diseases	Asthma	Hemoglobinopathies and hemolytic anemias	Diarrheal diseases	Congenital anomalies	Protein-energy malnutrition	Epilepsy	Malaria	Neonatal preterm birth complications
South Africa	Prevalence Rate: Cumulative change (%)	-0.4 (-0.8 to -0.0)	-10.6 (-14.7 to -6.4)	0.9 (-0.8 to 2.7)	-21.2 (-24.7 to -17.8)	-13.7 (-16.0 to -11.4)	-28.1 (-32.5 to -24.2)	23.2 (7.0–38.5)	-49.5 (-64.4 to -29.5)	-15.6 (-32.8 to 2.8)	-16.3 (-21.5 to -11.0)	10.7 (1.5–19.8)
South Africa	YLDs Number: 1990	1,160,874 (884,204–1,476,786)	182,101 (130,406–246,189)	142,332 (98,429–198,242)	119,165 (78,712–163,605)	11,749 (8,406–15,914)	43,861 (31,883–58,610)	16,344 (11,081–23,020)	24,454 (15,384–34,758)	19,002 (13,265–25,294)	4,923 (3,296–6,920)	10,019 (7,405–13,183)
South Africa	YLDs Number: 2015	1,238,305 (944,891–1,564,306)	166,876 (119,657–224,748)	155,952 (107,272–218,391)	99,394 (66,250–135,455)	10,766 (7,758–14,574)	33,217 (23,952–43,910)	30,663 (20,301–43,727)	12,802 (8,075–18,305)	13,024 (8,966–17,555)	4,313 (2,826–6,195)	12,625 (9,404–16,759)
South Africa	YLDs Number: Cumulative change (%)	6.7 (4.7–9.0)	-8.3 (-13.4 to -3.3)	9.5 (7.9–11.1)	-16.5 (-20.2 to -12.7)	-8.4 (-14.1 to -2.7)	-24.2 (-29.0 to -19.9)	87.7 (60.7–119.2)	-46.6 (-62.2 to -25.8)	-30.9 (-45.4 to -14.4)	-12.4 (-22.6 to -2.5)	26.0 (18.6–34.1)
South Africa	YLDs Rate: 1990	6,457.5 (4,919.0–8,205.2)	992.7 (710.8–1,344.4)	790.1 (544.7–1,105.8)	656.4 (433.4–900.0)	63.8 (45.7–86.4)	233.0 (169.4–311.2)	89.7 (60.8–126.3)	127.5 (80.2–181.2)	105.2 (73.4–140.1)	26.8 (17.9–37.6)	54.7 (40.5–72.0)
South Africa	YLDs Rate: 2015	6,328.5 (4,828.2–7,999.5)	872.6 (626.1–1,175.7)	794.7 (547.1–1,111.1)	516.7 (344.5–706.0)	56.7 (40.9–76.8)	167.3 (120.7–221.1)	156.4 (103.6–223.3)	63.3 (39.9–90.6)	66.5 (45.7–89.6)	22.7 (14.8–32.6)	64.5 (48.0–85.8)
South Africa	YLDs Rate: Cumulative change (%)	-1.9 (-3.7 to -0.1)	-12.1 (-16.8 to -7.3)	0.6 (-0.7 to 1.7)	-21.2 (-24.8 to -17.8)	-11.2 (-16.6 to -5.8)	-28.1 (-32.7 to -24.1)	74.5 (49.2–103.9)	-49.3 (-64.1 to -29.6)	-36.3 (-49.5 to -21.3)	-15.5 (-25.2 to -6.0)	18.0 (10.9–25.6)
Swaziland	Prevalence Number: 1990	494,809 (492,185–497,028)	131,502 (120,610–142,928)	172,873 (166,240–179,401)	73,011 (60,484–86,650)	120,813 (113,092–130,645)	10,317 (9,469–11,267)	5,669 (4,429–7,466)	936 (541–1,511)	1,105 (747–1,567)	18 (10–28)	3,048 (2,412–3,826)
Swaziland	Prevalence Number: 2015	612,053 (608,337–615,526)	157,352 (145,129–177,619)	226,416 (218,357–234,459)	97,312 (80,275–115,065)	130,224 (121,866–140,151)	8,567 (7,987–9,251)	7,192 (5,679–9,112)	927 (522–1,460)	1,532 (1,033–2,115)	16 (2–48)	3,374 (2,674–4,247)
Swaziland	Prevalence Number: Cumulative change (%)	23.7 (22.8–24.6)	19.8 (9.4–33.7)	31.0 (24.7–37.7)	33.4 (23.3–43.8)	8.0 (-2.5 to 18.0)	-16.9 (-20.7 to -12.2)	29.8 (-9.8 to 75.1)	9.0 (-51.5 to 113.9)	45.7 (-15.8 to 125.2)	-5.8 (-83.6 to 171.1)	11.7 (-10.9 to 38.2)
Swaziland	Prevalence Rate: 1990	97,460.2 (97,006.9–97,834.2)	24,641.4 (22,731.7–26,611.1)	35,656.8 (34,350.7–36,947.2)	14,631.9 (12,208.8–17,339.2)	23,708.8 (22,197.9–25,630.6)	1,770.2 (1,634.7–1,921.2)	1,100.0 (860.7–1,451.1)	147.4 (85.4–235.9)	221.9 (149.5–313.3)	3.5 (2.0–5.5)	545.2 (428.1–689.4)
Swaziland	Prevalence Rate: 2015	97,282.0 (96,719.8–97,808.9)	24,791.4 (22,930.1–27,850.9)	36,236.7 (34,948.2–37,525.9)	15,616.3 (12,868.1–18,443.3)	20,678.4 (19,351.0–22,254.8)	1,322.4 (1,232.8–1,426.6)	1,139.7 (899.2–1,444.9)	139.6 (78.6–219.8)	244.6 (164.7–337.8)	2.6 (0.4–7.7)	522.5 (412.8–659.6)
Swaziland	Prevalence Rate: Cumulative change (%)	-0.2 (-0.9 to 0.5)	0.7 (-7.7 to 11.9)	1.7 (-3.1 to 6.7)	6.8 (-0.9 to 14.6)	-12.6 (-21.1 to -4.5)	-25.2 (-28.5 to -21.2)	6.1 (-26.4 to 43.1)	4.2 (-53.7 to 104.9)	15.8 (-32.8 to 79.3)	-22.8 (-86.7 to 128.3)	-3.1 (-24.3 to 21.7)
Swaziland	YLDs Number: 1990	32,584 (24,482–41,797)	4,914 (3,450–6,768)	3,923 (2,722–5,453)	3,301 (2,185–4,601)	368 (259–500)	1,685 (1,212–2,248)	424 (253–673)	114 (58–199)	442 (257–663)	0 (0–0)	240 (177–319)
Swaziland	YLDs Number: 2015	44,042 (33,463–55,769)	5,891 (4,135–8,163)	4,956 (3,423–6,870)	4,394 (2,919–5,987)	427 (295–590)	1,394 (1,002–1,837)	642 (354–1,113)	114 (56–200)	440 (251–667)	0 (0–1)	256 (188–335)
Swaziland	YLDs Number: Cumulative change (%)	35.3 (30.5–40.2)	20.1 (6.9–36.3)	26.2 (23.1–29.3)	33.3 (23.1–43.6)	16.1 (-4.4 to 33.1)	-17.2 (-21.8 to -12.0)	57.9 (-14.9 to 173.3)	10.0 (-51.5 to 114.8)	5.0 (-41.4 to 66.3)	-5.8 (-83.6 to 171.1)	6.7 (-4.4 to 19.0)
Swaziland	YLDs Rate: 1990	6,620.2 (4,989.8–8,463.3)	924.6 (651.3–1,275.4)	785.8 (543.9–1,099.9)	661.2 (438.6–915.0)	71.2 (50.4–97.2)	288.9 (208.6–384.7)	81.7 (48.7–129.3)	18.1 (9.2–31.4)	88.6 (51.7–133.2)	0.1 (0.0–0.1)	45.1 (33.1–60.0)
Swaziland	YLDs Rate: 2015	7,042.8 (5,351.4–8,919.9)	932.2 (656.5–1,283.2)	790.3 (545.4–1,095.5)	705.1 (468.3–962.5)	68.2 (47.1–93.9)	215.2 (154.8–283.1)	101.7 (55.9–176.0)	17.2 (8.5–30.2)	70.3 (40.1–106.5)	0.1 (0.0–0.2)	40.2 (29.7–52.6)
Swaziland	YLDs Rate: Cumulative change (%)	6.4 (2.9–10.1)	1.0 (-9.6 to 13.8)	0.6 (-1.4 to 2.5)	6.8 (-1.1 to 14.8)	-4.1 (-20.8 to 9.9)	-25.4 (-29.5 to -21.1)	29.7 (-29.8 to 123.6)	-5.0 (-53.6 to 104.5)	-16.4 (-53.5 to 32.2)	-22.8 (-86.7 to 128.2)	-10.6 (-20.3 to 0.1)
Zimbabwe	Prevalence Number: 1990	5,637,194 (5,596,160–5,676,933)	1,571,279 (1,449,320–1,713,734)	2,060,095 (1,977,730–2,140,654)	536,224 (444,458–634,499)	1,516,536 (1,443,515–1,592,242)	117,662 (107,377–129,533)	72,838 (58,873–89,955)	27,945 (17,454–42,013)	10,516 (7,048–14,823)	196,609 (175,800–219,739)	43,729 (34,798–54,337)
Zimbabwe	Prevalence Number: 2015	7,596,141 (7,546,375–7,645,049)	2,399,543 (2,233,618–2,539,860)	2,826,003 (2,727,830–2,924,835)	767,820 (636,664–911,954)	1,814,436 (1,729,617–1,904,816)	141,468 (132,399–150,989)	99,156 (79,890–123,322)	34,804 (22,180–51,950)	14,963 (9,786–21,217)	395,089 (279,256–613,355)	59,628 (47,751–74,125)
Zimbabwe	Prevalence Number: Cumulative change (%)	34.8 (33.5–36.1)	53.3 (31.1–73.6)	37.2 (30.5–44.5)	43.3 (31.5–54.7)	19.7 (12.4–28.0)	20.4 (13.3–27.7)	38.3 (0.2–83.5)	33.5 (-33.2 to 123.7)	49.1 (-14.9 to 137.2)	100.1 (52.2–197.8)	37.6 (10.4–69.1)
Zimbabwe	Prevalence Rate: 1990	94,326.5 (93,672.2–94,974.8)	25,078.7 (23,222.3–27,331.0)	35,578.7 (34,211.7–36,916.6)	8,942.9 (7,445.6–10,551.6)	25,238.6 (24,026.5–26,484.9)	1,805.6 (1,656.1–1,981.6)	1,205.7 (972.0–1,492.3)	407.6 (254.9–612.5)	178.0 (118.9–250.5)	3,268.6 (2,931.3–3,619.6)	690.9 (545.1–865.7)
Zimbabwe	Prevalence Rate: 2015	93,778.8 (93,204.8–94,362.7)	28,409.6 (26,575.3–30,026.6)	35,898.1 (34,651.8–37,173.0)	9,585.1 (7,945.2–11,329.8)	22,302.2 (21,249.0–23,409.0)	1,609.4 (1,510.2–1,714.1)	1,211.5 (974.1–1,506.6)	370.6 (236.2–552.9)	187.4 (122.6–265.1)	4,784.2 (3,432.0–7,203.5)	688.7 (547.0–865.3)
Zimbabwe	Prevalence Rate: Cumulative change (%)	-0.6 (-1.5 to 0.3)	13.7 (-2.1 to 28.2)	0.9 (-3.9 to 6.1)	7.3 (-1.3 to 15.5)	-11.6 (-17.0 to -5.5)	-10.7 (-15.8 to -5.6)	2.1 (-26.2 to 36.0)	-2.6 (-51.1 to 63.3)	10.3 (-37.0 to 75.3)	45.9 (11.5–112.7)	0.7 (-20.2 to 25.6)
Zimbabwe	YLDs Number: 1990	370,171 (279,522–472,489)	60,028 (41,964–81,723)	46,344 (32,158–64,481)	24,286 (15,937–33,309)	4,994 (3,557–6,708)	19,205 (13,881–25,781)	5,240 (3,239–8,049)	3,451 (1,870–5,697)	4,177 (2,479–6,323)	5,049 (3,626–6,716)	4,114 (3,029–5,357)
Zimbabwe	YLDs Number: 2015	519,525 (392,716–660,437)	93,707 (66,837–126,866)	63,173 (43,657–87,743)	34,781 (23,003–48,193)	6,549 (4,560–8,776)	23,020 (16,748–30,386)	7,409 (4,597–12,077)	4,297 (2,399–7,120)	5,093 (3,052–7,424)	8,316 (6,013–11,088)	5,255 (3,922–6,787)
Zimbabwe	YLDs Number: Cumulative change (%)	40.5 (33.4–47.5)	57.2 (29.5–88.7)	36.3 (33.6–39.0)	43.4 (31.2–54.9)	31.6 (10.3–51.4)	20.1 (12.1–28.4)	46.5 (-9.1 to 129.9)	33.7 (-33.4 to 125.1)	28.4 (-28.6 to 105.7)	65.0 (47.7–83.6)	28.1 (14.8–42.4)
Zimbabwe	YLDs Rate: 1990	6,295.5 (4,751.0–8,019.5)	953.8 (668.5–1,296.9)	783.0 (542.8–1,093.9)	404.9 (265.2–557.7)	80.7 (57.6–108.6)	294.6 (212.9–393.2)	86.4 (53.4–132.9)	50.3 (27.3–83.1)	70.6 (42.2–107.5)	83.0 (59.8–110.0)	66.7 (49.0–87.0)
Zimbabwe	YLDs Rate: 2015	6,493.5 (4,918.7–8,234.5)	1,105.4 (790.0–1,498.9)	788.0 (541.8–1,096.4)	434.1 (287.0–600.4)	79.0 (55.4–105.7)	261.8 (190.3–345.4)	90.2 (56.2–147.3)	45.8 (25.5–75.8)	63.8 (38.2–93.3)	101.0 (73.2–134.4)	62.7 (46.6–80.9)
Zimbabwe	YLDs Rate: Cumulative change (%)	3.2 (-1.7 to 7.9)	16.6 (-3.0 to 38.1)	0.6 (-1.3 to 2.6)	7.3 (-1.4 to 15.7)	-1.8 (-16.8 to 12.2)	-11.0 (-16.7 to -5.1)	8.1 (-32.8 to 69.3)	-2.4 (-51.4 to 64.1)	-4.9 (-47.6 to 52.1)	21.8 (9.5–34.9)	-5.7 (-15.9 to 5.1)
Western Sub-Saharan Africa	Prevalence Number: 1990	108,272,465 (108,130,865–108,405,419)	41,630,605 (40,634,186–42,617,545)	36,610,757 (35,773,887–37,369,285)	6,708,718 (5,657,935–7,845,091)	47,368,843 (46,544,179–48,180,172)	2,939,948 (2,727,122–3,156,098)	1,056,982 (895,327–1,295,452)	2,134,969 (1,854,444–2,454,040)	194,770 (157,423–238,657)	43,489,671 (39,434,975–47,870,820)	523,448 (414,287–644,000)
Western Sub-Saharan Africa	Prevalence Number: 2015	206,177,716 (205,853,640–206,504,384)	71,758,741 (69,765,362–73,196,454)	71,801,710 (70,175,226–73,300,817)	12,737,815 (10,413,118–15,115,919)	86,474,216 (84,979,763–88,038,296)	3,127,410 (2,958,972–3,335,016)	2,318,812 (2,009,781–2,747,317)	3,389,017 (2,917,343–3,933,314)	435,017 (352,258–540,373)	95,826,414 (82,850,447–110,020,418)	996,271 (797,926–1,212,778)
Western Sub-Saharan Africa	Prevalence Number: Cumulative change (%)	90.4 (90.1–90.8)	72.4 (65.2–78.7)	96.1 (91.0–101.2)	89.7 (79.1–101.2)	82.6 (78.6–86.5)	6.5 (1.9–11.3)	121.5 (78.9–169.3)	59.9 (28.8–96.7)	126.0 (76.0–188.8)	120.4 (97.8–146.6)	91.1 (67.7–115.3)
Western Sub-Saharan Africa	Prevalence Rate: 1990	97,941.7 (97,821.4–98,051.4)	36,425.0 (35,678.9–37,189.6)	34,902.9 (34,133.8–35,614.1)	6,399.5 (5,426.8–7,458.3)	42,741.6 (41,995.3–43,474.6)	2,313.2 (2,153.8–2,479.4)	938.2 (790.7–1,154.1)	1,564.4 (1,358.9–1,798.6)	173.9 (140.7–213.4)	38,651.6 (35,079.9–42,454.7)	404.5 (321.1–498.6)
Western Sub-Saharan Africa	Prevalence Rate: 2015	97,079.0 (96,942.3–97,223.8)	32,759.0 (31,887.5–33,378.8)	35,351.1 (34,575.8–36,104.8)	6,196.5 (5,094.8–7,379.6)	40,608.1 (39,897.7–41,338.2)	1,349.8 (1,280.5–1,433.7)	1,079.2 (933.8–1,281.0)	1,339.5 (1,153.0–1,554.2)	203.7 (164.4–252.4)	44,837.2 (38,561.9–51,561.4)	422.4 (337.9–517.0)
Western Sub-Saharan Africa	Prevalence Rate: Cumulative change (%)	-0.9 (-1.1 to -0.7)	-10.0 (-13.5 to -7.1)	1.3 (-1.2 to 3.8)	-3.2 (-8.5 to 2.5)	-5.0 (-7.1 to -2.9)	-41.6 (-44.0 to -39.1)	15.0 (-6.5 to 41.3)	-13.7 (-30.5 to 6.1)	18.5 (-8.0 to 51.4)	16.0 (4.1–29.9)	5.0 (-9.7 to 20.2)
Western Sub-Saharan Africa	YLDs Number: 1990	8,876,885 (6,660,655–11,363,397)	2,016,370 (1,471,710–2,669,153)	732,725 (503,414–1,029,318)	300,926 (200,101–405,944)	306,223 (224,465–404,705)	472,293 (345,648–625,621)	63,832 (41,708–93,638)	259,956 (174,455–356,836)	79,118 (55,113–107,622)	509,537 (373,426–676,455)	25,013 (18,922–32,189)
Western Sub-Saharan Africa	YLDs Number: 2015	15,184,327 (11,452,317–19,354,908)	3,262,703 (2,378,226–4,335,003)	1,433,936 (981,674–2,018,844)	574,266 (376,446–782,871)	530,793 (386,827–706,606)	505,014 (366,753–659,955)	162,495 (107,542–231,852)	416,459 (281,473–571,839)	167,049 (115,004–227,069)	1,031,751 (752,904–1,368,006)	55,031 (40,784–70,731)
Western Sub-Saharan Africa	YLDs Number: Cumulative change (%)	71.1 (66.1–75.3)	61.9 (49.2–71.9)	95.6 (93.5–97.7)	90.7 (79.9–102.4)	73.3 (59.0–84.9)	7.0 (2.2–12.1)	157.5 (124.2–200.5)	61.4 (30.7–98.8)	113.8 (61.7–177.6)	102.7 (88.2–114.9)	120.2 (101.3–138.2)
Western Sub-Saharan Africa	YLDs Rate: 1990	8,092.5 (6,076.8–10,360.7)	1,722.4 (1,256.9–2,282.4)	684.5 (470.0–969.1)	286.9 (191.1–384.7)	266.5 (195.2–351.8)	371.5 (271.3–491.8)	56.1 (36.3–83.3)	190.5 (127.9–261.4)	70.6 (49.0–95.6)	446.0 (327.0–588.4)	20.0 (15.0–25.6)
Western Sub-Saharan Africa	YLDs Rate: 2015	7,223.8 (5,456.4–9,198.8)	1,462.7 (1,064.2–1,941.1)	693.5 (472.1–982.9)	279.3 (183.8–379.9)	241.5 (175.9–322.7)	217.9 (158.3–285.0)	75.1 (49.8–107.2)	164.6 (111.3–226.1)	78.2 (53.5–106.5)	474.6 (347.2–628.6)	24.1 (17.8–30.9)
Western Sub-Saharan Africa	YLDs Rate: Cumulative change (%)	-10.7 (-13.0 to -8.7)	-15.1 (-21.0 to -10.4)	1.3 (0.2–2.3)	-2.8 (-8.4 to 3.0)	-9.4 (-16.5 to -3.9)	-41.3 (-43.9 to -38.6)	35.4 (17.8–58.2)	-12.9 (-29.5 to 7.2)	12.1 (-14.8 to 45.8)	6.5 (-0.3 to 12.3)	20.9 (9.9–31.4)
Benin	Prevalence Number: 1990	2,764,088 (2,755,846–2,772,671)	769,021 (706,080–809,319)	971,899 (896,679–1,060,944)	171,130 (138,893–203,952)	1,139,024 (1,104,502–1,174,110)	71,252 (66,224–75,557)	27,551 (20,001–37,177)	63,500 (42,235–89,762)	5,218 (3,385–7,328)	1,961,277 (1,802,622–2,129,618)	15,354 (11,444–20,136)
Benin	Prevalence Number: 2015	5,610,229 (5,582,003–5,637,264)	1,609,523 (1,492,713–1,714,122)	2,062,863 (1,910,403–2,241,944)	333,742 (273,566–401,711)	2,241,685 (2,180,793–2,304,628)	67,922 (64,077–71,989)	67,902 (52,280–87,761)	93,519 (63,757–130,206)	12,294 (8,204–17,309)	3,304,320 (2,809,748–3,853,429)	31,479 (24,201–40,578)
Benin	Prevalence Number: Cumulative change (%)	103.0 (101.9–104.1)	109.7 (89.8–135.5)	112.7 (89.5–136.6)	95.2 (82.7–109.8)	96.9 (89.6–104.8)	-4.6 (-10.3 to 1.9)	154.2 (70.1–275.1)	55.6 (-12.9 to 148.9)	147.6 (39.8–297.6)	68.5 (46.1–91.9)	108.2 (56.9–164.3)
Benin	Prevalence Rate: 1990	98,196.8 (97,942.9–98,472.5)	25,787.0 (23,872.6–26,941.0)	36,298.4 (33,647.1–39,492.4)	6,417.8 (5,255.8–7,609.6)	40,290.7 (39,071.4–41,535.2)	2,146.0 (2,001.2–2,268.6)	957.6 (689.1–1,299.1)	1,788.5 (1,191.3–2,526.7)	182.6 (118.6–257.3)	69,093.1 (63,162.9–75,702.0)	459.1 (336.7–617.5)
Benin	Prevalence Rate: 2015	96,999.7 (96,509.1–97,455.2)	27,009.8 (25,189.0–28,643.9)	36,726.3 (34,151.5–39,766.0)	5,934.5 (4,883.3–7,146.8)	38,652.9 (37,604.9–39,731.2)	1,103.7 (1,043.2–1,168.7)	1,164.2 (895.3–1,506.7)	1,412.9 (963.4–1,966.6)	212.6 (141.5–300.3)	57,099.3 (48,449.6–66,795.9)	502.7 (382.2–653.6)
Benin	Prevalence Rate: Cumulative change (%)	-1.2 (-1.7 to -0.7)	4.9 (-4.2 to 17.0)	1.4 (-9.1 to 12.6)	-7.5 (-13.5 to -0.5)	-4.0 (-7.6 to -0.2)	-48.5 (-51.5 to -45.1)	25.6 (-16.7 to 86.0)	-16.5 (-53.3 to 33.4)	22.4 (-31.0 to 96.5)	-17.4 (-28.4 to -5.8)	11.8 (-19.7 to 46.7)
Benin	YLDs Number: 1990	215,326 (163,116–277,845)	34,048 (24,007–45,396)	20,401 (13,932–28,651)	7,691 (5,070–10,552)	8,392 (6,040–11,208)	11,496 (8,385–15,140)	1,613 (1,028–2,419)	7,749 (4,437–11,982)	2,054 (1,183–3,193)	18,995 (13,767–25,222)	690 (502–910)

© 2017 Global Burden of Disease Child and Adolescent Health Collaboration. JAMA Pediatrics.

eTable 3. Prevalent Cases, Rates (per 100,000 population), Years Lived with Disability (YLDs), and Cumulative Percent Change with 95% Uncertainty Interval (UI) for the Top 10 Global Causes of YLDs in Children and Adolescents in 195 Countries and Territories, Aged 0 to 19 Years, Both Sexes, 1990 and 2015. *Note: best viewed by enlarging in browser.*

Location	Measure	All causes	Iron-deficiency anemia	Skin and subcutaneous diseases	Asthma	Hemoglobinopathies and hemolytic anemias	Diarrheal diseases	Congenital anomalies	Protein-energy malnutrition	Epilepsy	Malaria	Neonatal preterm birth complications
Benin	YLDs Number: 2015	387,737 (292,218–493,953)	70,084 (50,077–93,642)	42,941 (29,189–60,632)	15,070 (9,857–20,438)	16,698 (11,875–22,409)	10,995 (8,005–14,347)	4,557 (3,006–6,897)	11,555 (6,711–17,595)	4,200 (2,556–6,484)	32,868 (23,647–43,791)	1,742 (1,279–2,276)
Benin	YLDs Number: Cumulative change (%)	80.2 (71.0–90.2)	106.8 (79.5–143.1)	110.4 (105.5–115.0)	96.1 (81.6–111.6)	99.4 (73.8–123.3)	-4.2 (-10.9 to 2.9)	187.5 (132.1–285.6)	57.4 (-12.6 to 151.9)	116.0 (18.1–243.3)	73.3 (54.9–95.8)	154.1 (113.5–196.8)
Benin	YLDs Rate: 1990	7,754.1 (5,884.7–9,978.2)	1,126.6 (800.1–1,501.2)	747.5 (507.3–1,049.9)	288.3 (189.8–391.7)	284.5 (204.9–379.4)	346.1 (251.5–456.4)	55.4 (35.2–83.3)	218.2 (125.2–337.5)	71.9 (41.8–112.1)	660.3 (479.0–873.5)	21.4 (15.5–28.2)
Benin	YLDs Rate: 2015	6,766.7 (5,106.7–8,618.8)	1,166.8 (832.4–1,560.5)	755.2 (511.9–1,071.2)	267.9 (175.6–362.6)	280.0 (199.1–375.6)	178.6 (129.9–232.1)	77.8 (51.1–117.5)	174.6 (101.4–265.8)	72.6 (44.0–111.6)	559.9 (404.2–744.8)	28.7 (20.9–37.6)
Benin	YLDs Rate: Cumulative change (%)	-12.7 (-16.7 to -8.4)	3.9 (-8.3 to 20.1)	1.0 (-1.2 to 3.2)	-7.0 (-13.6 to 0.3)	-1.4 (-13.3 to 9.6)	-48.3 (-51.8 to -44.6)	43.0 (15.1–90.3)	-15.6 (-53.1 to 35.1)	6.8 (-42.1 to 70.5)	-15.1 (-23.6 to -4.9)	35.3 (11.6–59.2)
Burkina Faso	Prevalence Number: 1990	4,989,115 (4,977,452–5,000,589)	1,568,726 (1,496,099–1,632,498)	1,743,439 (1,605,381–1,903,076)	267,836 (218,044–318,646)	2,979,422 (2,900,309–3,057,672)	173,497 (162,091–182,477)	47,898 (34,254–65,740)	126,687 (89,399–170,112)	8,464 (5,566–12,017)	2,091,149 (1,926,641–2,264,778)	25,600 (19,203–34,476)
Burkina Faso	Prevalence Number: 2015	9,944,539 (9,915,482–9,973,104)	2,810,902 (2,684,955–2,946,810)	3,574,761 (3,300,018–3,910,687)	537,300 (437,629–647,950)	5,653,951 (5,503,226–5,805,115)	181,703 (166,425–202,366)	110,428 (83,899–147,139)	205,383 (138,853–286,550)	19,656 (12,691–27,580)	6,133,875 (5,592,871–6,698,339)	48,233 (36,329–62,846)
Burkina Faso	Prevalence Number: Cumulative change (%)	99.3 (98.6–100.0)	79.4 (66.1–93.6)	105.5 (83.9–128.9)	100.8 (86.7–116.6)	89.8 (82.6–97.2)	4.7 (-3.0 to 14.4)	140.1 (47.8–256.2)	68.5 (0.5–164.0)	143.6 (38.6–291.6)	193.8 (165.1–225.0)	91.5 (46.0–148.0)
Burkina Faso	Prevalence Rate: 1990	98,036.4 (97,816.2–98,252.4)	29,270.6 (28,029.0–30,306.1)	35,948.7 (33,315.8–39,020.4)	5,476.2 (4,489.9–6,514.7)	58,342.0 (56,762.4–59,865.4)	2,956.6 (2,775.5–3,099.4)	920.3 (651.7–1,267.9)	1,998.4 (1,410.7–2,683.1)	165.5 (109.0–234.3)	40,649.3 (37,272.3–44,229.2)	419.7 (309.4–585.8)
Burkina Faso	Prevalence Rate: 2015	97,524.9 (97,240.7–97,809.1)	26,439.7 (25,401.8–27,577.1)	36,408.3 (33,706.1–39,633.1)	5,406.9 (4,424.4–6,499.0)	55,338.6 (53,865.5–56,819.7)	1,643.0 (1,512.0–1,819.4)	1,070.3 (810.0–1,426.9)	1,724.9 (1,166.8–2,405.9)	192.9 (124.5–271.6)	59,347.4 (53,914.3–65,161.9)	426.4 (318.1–563.4)
Burkina Faso	Prevalence Rate: Cumulative change (%)	-0.5 (-0.9 to -0.2)	-9.6 (-15.6 to -3.1)	1.5 (-8.8 to 12.1)	-1.2 (-8.2 to 6.8)	-5.1 (-8.7 to -1.4)	-44.4 (-48.3 to -39.6)	21.3 (-25.8 to 80.6)	-10.3 (-46.5 to 40.5)	22.2 (-30.7 to 96.0)	46.3 (30.8–63.7)	3.8 (-24.4 to 38.9)
Burkina Faso	YLDs Number: 1990	411,441 (309,544–528,076)	72,521 (52,498–97,192)	36,720 (25,141–51,413)	12,033 (7,785–16,470)	19,327 (14,203–25,794)	27,853 (20,276–36,355)	2,777 (1,759–4,180)	15,465 (9,449–23,104)	3,450 (2,078–5,334)	29,057 (21,092–38,343)	903 (653–1,214)
Burkina Faso	YLDs Number: 2015	729,677 (546,794–932,892)	140,175 (98,991–184,300)	74,711 (50,509–104,892)	24,269 (15,818–33,469)	38,810 (28,380–51,312)	29,218 (20,956–38,617)	7,402 (4,813–10,838)	25,072 (14,584–38,115)	6,708 (3,817–10,032)	81,732 (59,246–106,706)	1,993 (1,442–2,610)
Burkina Faso	YLDs Number: Cumulative change (%)	77.5 (66.9–88.4)	94.1 (62.6–124.4)	103.4 (99.1–107.9)	101.9 (86.6–119.1)	101.1 (81.3–118.9)	4.9 (-3.2 to 14.7)	170.3 (125.2–239.1)	68.6 (0.7–165.3)	104.5 (12.9–228.2)	182.1 (150.7–220.0)	122.4 (80.4–167.6)
Burkina Faso	YLDs Rate: 1990	8,113.4 (6,108.2–10,387.4)	1,316.3 (956.0–1,756.6)	743.3 (507.4–1,050.5)	246.0 (159.9–334.5)	360.2 (264.4–479.3)	474.6 (346.0–619.6)	52.7 (33.1–79.8)	244.0 (149.0–364.6)	67.4 (40.6–103.5)	552.8 (402.6–727.6)	15.2 (10.8–20.6)
Burkina Faso	YLDs Rate: 2015	7,142.7 (5,389.1–9,135.9)	1,278.7 (905.4–1,692.3)	749.4 (507.3–1,058.8)	244.2 (159.7–333.5)	363.5 (264.4–480.5)	264.3 (189.7–347.9)	71.2 (46.3–104.3)	210.6 (122.5–320.1)	65.9 (37.6–98.6)	773.4 (562.1–1,008.5)	18.1 (13.0–23.8)
Burkina Faso	YLDs Rate: Cumulative change (%)	-11.9 (-16.7 to -6.8)	-2.5 (-16.9 to 12.1)	0.8 (-1.4 to 3.0)	-0.6 (-8.2 to 7.7)	1.1 (-8.0 to 9.5)	-44.3 (-48.5 to -39.4)	37.2 (14.1–73.4)	-10.2 (-46.3 to 41.1)	2.7 (-43.4 to 65.1)	40.2 (25.9–57.7)	20.4 (-3.7 to 46.0)
Cameroon	Prevalence Number: 1990	6,747,365 (6,723,812–6,770,905)	1,897,820 (1,736,966–2,102,242)	2,380,789 (2,221,519–2,602,965)	413,504 (339,631–490,290)	2,396,584 (2,324,926–2,473,964)	210,623 (192,886–226,287)	75,758 (57,020–97,919)	57,241 (40,569–79,596)	13,846 (9,081–19,721)	2,473,157 (2,258,321–2,724,676)	33,739 (26,121–42,919)
Cameroon	Prevalence Number: 2015	12,014,223 (11,960,525–12,071,910)	3,005,478 (2,834,776–3,136,220)	4,472,490 (4,160,717–4,845,017)	762,031 (623,872–903,202)	4,239,495 (4,096,552–4,378,464)	214,229 (200,190–230,635)	148,335 (112,077–193,909)	96,942 (63,892–141,512)	30,005 (20,098–41,160)	5,492,690 (4,617,057–6,458,836)	65,100 (50,425–84,530)
Cameroon	Prevalence Number: Cumulative change (%)	78.1 (77.1–79.1)	58.9 (44.3–71.7)	88.2 (68.9–108.3)	84.4 (72.6–98.1)	76.9 (69.8–84.2)	1.8 (-3.7 to 8.3)	100.8 (35.5–181.2)	76.5 (0.7–181.2)	127.5 (27.7–253.6)	122.3 (88.4–160.1)	94.9 (52.8–144.3)
Cameroon	Prevalence Rate: 1990	98,842.2 (98,554.3–99,131.6)	25,700.6 (23,763.3–28,200.8)	36,785.8 (34,546.6–39,886.6)	6,447.0 (5,326.8–7,601.5)	35,002.6 (33,958.3–36,144.6)	2,618.2 (2,408.8–2,802.8)	1,091.9 (818.4–1,411.1)	660.3 (468.2–918.1)	199.6 (131.4–283.7)	37,724.7 (34,354.6–41,731.1)	424.3 (322.7–551.7)
Cameroon	Prevalence Rate: 2015	96,384.7 (95,979.7–96,831.8)	23,184.3 (21,918.0–24,083.3)	36,977.6 (34,545.4–39,976.8)	6,284.7 (5,165.5–7,424.0)	33,917.6 (32,774.3–35,028.1)	1,598.3 (1,497.8–1,715.6)	1,178.6 (886.9–1,543.1)	672.7 (443.8–981.4)	239.3 (159.9–328.2)	44,159.0 (36,918.3–52,111.5)	480.0 (368.9–631.4)
Cameroon	Prevalence Rate: Cumulative change (%)	-2.5 (-3.0 to -2.0)	-9.6 (-16.8 to -3.1)	0.7 (-8.8 to 10.8)	-2.5 (-8.8 to 4.7)	-3.1 (-7.0 to 0.9)	-38.9 (-42.0 to -35.3)	10.8 (-25.7 to 55.5)	6.2 (-39.4 to 69.1)	25.9 (-29.4 to 96.5)	17.2 (-1.1 to 37.0)	14.7 (-13.4 to 46.9)
Cameroon	YLDs Number: 1990	513,120 (386,009–659,307)	79,116 (55,349–110,216)	50,616 (34,918–70,717)	18,587 (12,289–25,118)	16,125 (11,597–21,821)	34,085 (24,580–45,267)	4,490 (2,847–6,581)	6,987 (4,169–10,654)	5,559 (3,273–8,443)	29,193 (21,085–38,798)	1,796 (1,314–2,301)
Cameroon	YLDs Number: 2015	817,346 (619,574–1,039,202)	116,653 (83,438–158,776)	94,150 (64,781–132,349)	34,436 (22,541–46,981)	26,362 (18,925–34,950)	34,867 (25,197–45,958)	10,280 (6,605–15,458)	11,992 (6,978–19,127)	10,863 (6,445–16,085)	60,150 (43,585–79,706)	3,823 (2,812–4,964)
Cameroon	YLDs Number: Cumulative change (%)	59.4 (52.5–65.9)	48.5 (24.9–67.5)	85.9 (81.5–89.6)	85.4 (72.6–99.5)	64.1 (46.7–82.6)	2.4 (-3.8 to 9.4)	131.9 (83.2–206.8)	78.7 (1.4–185.6)	105.8 (16.4–228.3)	106.4 (89.7–123.7)	113.7 (83.0–146.1)
Cameroon	YLDs Rate: 1990	7,678.7 (5,812.1–9,817.2)	1,054.6 (746.1–1,456.4)	761.9 (525.4–1,079.6)	289.7 (192.0–391.1)	226.8 (163.2–305.8)	423.4 (305.6–561.0)	64.2 (40.6–93.9)	80.6 (48.1–122.9)	80.1 (46.7–120.7)	430.9 (312.4–571.9)	23.5 (17.1–30.2)
Cameroon	YLDs Rate: 2015	6,654.4 (5,050.6–8,460.5)	893.5 (638.2–1,213.4)	766.6 (527.5–1,083.1)	283.9 (185.7–384.1)	207.0 (149.1–274.7)	260.0 (187.5–342.5)	81.2 (52.3–122.8)	83.2 (48.4–132.8)	86.6 (51.2–128.1)	478.1 (346.8–633.5)	29.0 (21.3–37.8)
Cameroon	YLDs Rate: Cumulative change (%)	-13.3 (-16.6 to -10.2)	-14.8 (-26.8 to -5.1)	0.6 (-1.7 to 2.6)	-1.9 (-8.8 to 5.6)	-8.5 (-17.0 to 0.6)	-38.5 (-42.1 to -34.4)	28.3 (1.9–69.3)	7.5 (-38.9 to 71.8)	13.9 (-35.3 to 82.3)	11.1 (3.1–19.4)	24.0 (5.3–43.1)
Cape Verde	Prevalence Number: 1990	178,315 (176,492–180,109)	77,529 (73,664–82,264)	68,183 (63,307–74,251)	11,353 (9,340–13,500)	42,417 (40,329–44,619)	4,910 (4,408–5,448)	2,830 (2,233–3,583)	1,950 (1,358–2,668)	489 (303–710)	443 (99–854)	1,832 (1,548–2,213)
Cape Verde	Prevalence Number: 2015	189,833 (187,744–191,826)	70,856 (66,993–75,777)	79,239 (74,079–85,796)	11,036 (8,856–13,149)	43,148 (41,172–45,402)	2,219 (2,051–2,374)	3,229 (2,618–4,105)	1,570 (954–2,370)	517 (341–742)	0 (0–0)	1,433 (1,185–1,760)
Cape Verde	Prevalence Number: Cumulative change (%)	6.5 (4.9–7.9)	-8.5 (-17.1 to -0.9)	16.5 (4.5–28.8)	-2.7 (-11.5 to 5.8)	1.8 (-5.0 to 8.2)	-54.7 (-58.3 to -51.3)	16.0 (-15.1 to 54.6)	-15.8 (-54.5 to 43.2)	12.0 (-33.8 to 78.8)	1,273.6 (-100.0 to -100.0)	-21.2 (-35.5 to -5.8)
Cape Verde	Prevalence Rate: 1990	91,881.9 (90,983.2–92,743.0)	37,881.4 (36,152.4–39,844.4)	36,828.8 (34,389.4–39,954.4)	6,206.2 (5,136.2–7,402.1)	21,727.5 (20,658.8–22,854.0)	2,134.3 (1,928.4–2,355.7)	1,443.0 (1,143.0–1,827.1)	792.7 (552.4–1,084.5)	249.4 (157.0–365.2)	219.8 (50.7–426.4)	852.8 (715.8–1,044.0)
Cape Verde	Prevalence Rate: 2015	90,949.2 (89,937.2–91,931.1)	34,336.9 (32,434.5–36,796.8)	37,585.0 (35,065.4–40,815.8)	5,277.5 (4,230.8–6,289.8)	20,709.9 (19,762.1–21,791.9)	1,079.6 (996.2–1,155.9)	1,550.3 (1,258.9–1,970.3)	774.2 (470.6–1,168.7)	247.9 (163.6–356.2)	0.0 (0.0–0.0)	695.5 (575.7–852.2)
Cape Verde	Prevalence Rate: Cumulative change (%)	-1.0 (-2.4 to 0.3)	-9.3 (-17.1 to -1.5)	2.2 (-7.7 to 12.6)	-14.9 (-22.8 to -7.2)	-4.6 (-10.9 to 1.4)	-49.3 (-53.1 to -45.8)	9.3 (-20.0 to 46.0)	2.0 (-44.9 to 73.6)	5.3 (-38.3 to 68.6)	1,229.9 (-100.0 to -100.0)	-17.8 (-33.1 to -1.2)
Cape Verde	YLDs Number: 1990	13,257 (10,034–16,949)	3,329 (2,340–4,505)	1,431 (970–2,008)	513 (335–701)	286 (200–389)	795 (576–1,060)	188 (123–276)	239 (142–362)	193 (109–307)	9 (1–18)	171 (129–220)
Cape Verde	YLDs Number: 2015	13,012 (9,894–16,576)	2,915 (2,071–4,019)	1,614 (1,078–2,291)	500 (326–686)	275 (196–379)	361 (259–478)	294 (179–474)	193 (107–310)	146 (85–226)	0 (0–0)	140 (106–179)
Cape Verde	YLDs Number: Cumulative change (%)	-1.8 (-7.4 to 3.9)	-12.0 (-27.2 to 3.4)	12.7 (9.2–16.0)	-2.4 (-11.8 to 6.7)	-3.7 (-17.1 to 12.0)	-54.5 (-58.4 to -51.1)	59.3 (5.8–140.2)	-15.2 (-54.0 to 45.4)	-19.5 (-54.4 to 29.5)	346.5 (-100.0 to -100.0)	-18.0 (-26.0 to -9.2)
Cape Verde	YLDs Rate: 1990	6,869.6 (5,215.8–8,777.7)	1,601.5 (1,130.2–2,156.2)	757.0 (508.7–1,074.4)	280.4 (183.9–383.1)	142.3 (100.2–193.1)	345.8 (251.1–459.9)	95.6 (62.8–140.7)	97.5 (58.0–147.3)	98.7 (55.6–156.4)	4.6 (0.9–9.1)	85.3 (64.8–110.0)
Cape Verde	YLDs Rate: 2015	6,214.5 (4,719.8–7,931.1)	1,424.9 (1,012.1–1,964.2)	769.7 (515.2–1,091.0)	239.2 (155.8–328.7)	134.6 (96.2–185.6)	175.7 (126.2–232.9)	141.7 (86.3–227.3)	95.6 (52.9–152.9)	70.4 (40.9–108.0)	0.0 (0.0–0.0)	67.6 (51.3–86.5)
Cape Verde	YLDs Rate: Cumulative change (%)	-9.5 (-14.1 to -4.6)	-10.7 (-24.8 to 4.6)	1.7 (-0.5 to 3.9)	-14.6 (-22.9 to -6.3)	-5.1 (-17.9 to 10.0)	-49.1 (-53.2 to -45.5)	50.6 (-0.1 to 127.3)	2.8 (-44.2 to 76.3)	-24.3 (-57.4 to 21.8)	311.2 (-100.0 to -100.0)	-20.7 (-28.3 to -12.4)
Chad	Prevalence Number: 1990	3,291,896 (3,276,791–3,307,854)	1,588,082 (1,526,826–1,651,894)	1,164,810 (1,073,853–1,278,379)	190,735 (156,280–226,874)	981,983 (931,904–1,038,205)	98,947 (93,384–104,208)	31,503 (22,667–43,929)	83,983 (55,352–119,583)	5,499 (3,439–8,139)	713,241 (596,877–869,927)	16,700 (12,396–22,113)
Chad	Prevalence Number: 2015	7,944,294 (7,899,231–7,987,397)	3,604,396 (3,433,705–3,739,426)	2,883,736 (2,662,190–3,155,910)	515,816 (427,546–607,271)	2,242,497 (2,124,916–2,368,026)	172,542 (162,101–185,629)	83,436 (62,044–118,566)	188,565 (127,735–260,487)	18,885 (11,924–27,914)	1,938,579 (1,420,047–2,604,298)	36,885 (27,798–47,358)
Chad	Prevalence Number: Cumulative change (%)	141.3 (139.7–142.9)	127.1 (113.7–141.8)	148.2 (120.6–177.2)	170.7 (149.2–194.4)	128.6 (113.1–144.6)	74.5 (64.3–88.6)	175.0 (70.2–312.2)	137.1 (32.6–280.9)	262.9 (99.6–469.6)	170.8 (120.7–227.1)	124.1 (66.9–180.9)
Chad	Prevalence Rate: 1990	96,396.3 (95,979.9–96,846.2)	43,956.8 (42,449.7–45,531.3)	36,141.3 (33,471.1–39,474.6)	5,955.9 (4,938.6–7,062.6)	28,638.8 (27,174.3–30,277.6)	2,440.5 (2,308.5–2,565.0)	899.1 (642.5–1,263.8)	1,872.5 (1,236.2–2,663.5)	156.1 (98.6–229.1)	20,384.0 (17,090.7–24,782.8)	396.2 (287.6–538.8)
Chad	Prevalence Rate: 2015	96,133.5 (95,633.7–96,634.3)	41,574.7 (39,745.9–43,048.7)	36,469.8 (33,840.9–39,655.2)	6,473.0 (5,369.9–7,603.2)	27,050.7 (25,617.8–28,558.9)	1,887.9 (1,781.1–2,023.2)	993.9 (735.8–1,422.0)	1,864.9 (1,263.0–2,576.1)	224.1 (141.7–332.1)	22,815.4 (17,056.0–30,268.1)	390.2 (287.9–509.6)
Chad	Prevalence Rate: Cumulative change (%)	-0.3 (-0.9 to 0.3)	-5.4 (-10.4 to 0.3)	1.1 (-9.5 to 12.2)	8.8 (-0.1 to 18.2)	-5.5 (-11.9 to 1.1)	-22.6 (-26.8 to -16.8)	15.0 (-29.2 to 73.9)	5.2 (-41.1 to 68.7)	51.7 (-16.9 to 139.9)	11.6 (-8.3 to 33.9)	0.6 (-29.8 to 30.8)
Chad	YLDs Number: 1990	302,060 (227,632–386,463)	94,286 (68,657–124,392)	24,563 (16,767–34,230)	8,559 (5,679–11,617)	6,874 (5,010–9,125)	15,699 (11,370–20,602)	1,772 (1,107–2,728)	10,085 (5,947–15,981)	2,243 (1,261–3,490)	7,212 (5,309–9,625)	616 (455–812)
Chad	YLDs Number: 2015	688,462 (516,936–880,103)	207,519 (151,689–272,782)	60,435 (40,909–84,851)	23,194 (15,385–31,947)	15,244 (11,164–20,036)	27,499 (20,039–36,130)	5,139 (3,332–7,603)	22,850 (12,972–35,327)	7,720 (4,356–12,086)	18,212 (13,392–24,142)	1,534 (1,123–2,018)
Chad	YLDs Number: Cumulative change (%)	128.0 (117.1–140.0)	120.5 (94.7–148.1)	146.0 (140.4–151.5)	171.3 (147.9–195.8)	122.1 (96.2–142.3)	75.3 (63.7–89.6)	193.5 (154.0–252.0)	138.5 (32.7–283.3)	264.9 (99.6–490.7)	153.1 (126.9–183.0)	150.5 (108.0–196.3)

© 2017 Global Burden of Disease Child and Adolescent Health Collaboration. JAMA Pediatrics.

eTable 3. Prevalent Cases, Rates (per 100,000 population), Years Lived with Disability (YLDs), and Cumulative Percent Change with 95% Uncertainty Interval (UI) for the Top 10 Global Causes of YLDs in Children and Adolescents in 195 Countries and Territories, Aged 0 to 19 Years, Both Sexes, 1990 and 2015. *Note: best viewed by enlarging in browser.*

Location	Measure	All causes	Iron-deficiency anemia	Skin and subcutaneous diseases	Asthma	Hemoglobinopathies and hemolytic anemias	Diarrheal diseases	Congenital anomalies	Protein-energy malnutrition	Epilepsy	Malaria	Neonatal preterm birth complications
Chad	YLDs Rate: 1990	8,667.2 (6,553.0–11,097.3)	2,443.1 (1,786.5–3,236.5)	746.1 (508.5–1,053.0)	267.3 (177.1–364.6)	188.0 (137.3–249.5)	387.6 (281.2–507.9)	50.1 (30.8–77.4)	224.9 (132.6–356.4)	63.8 (36.0–100.2)	203.5 (151.3–269.8)	15.0 (10.9–19.9)
Chad	YLDs Rate: 2015	8,212.3 (6,189.1–10,442.6)	2,283.5 (1,669.2–3,003.2)	751.3 (505.2–1,059.2)	291.1 (194.6–399.5)	174.1 (127.4–229.9)	301.2 (219.6–396.1)	60.7 (39.3–90.0)	226.0 (128.3–349.2)	91.7 (51.9–144.3)	211.7 (156.4–279.9)	16.6 (12.0–22.1)
Chad	YLDs Rate: Cumulative change (%)	-5.2 (-9.4 to -0.9)	-6.4 (-16.9 to 4.6)	0.7 (-1.6 to 2.9)	9.0 (-0.4 to 19.0)	-7.3 (-17.7 to 0.8)	-22.2 (-27.2 to -16.3)	22.7 (6.6–47.4)	5.8 (-41.1 to 69.9)	52.5 (-17.8 to 146.9)	4.2 (-6.0 to 15.3)	12.1 (-9.3 to 33.7)
Cote d'Ivoire	Prevalence Number: 1990	6,543,669 (6,530,045–6,557,495)	2,137,659 (2,018,786–2,200,556)	2,512,822 (2,415,531–2,606,506)	439,416 (362,497–524,642)	2,533,175 (2,418,870–2,644,786)	183,455 (167,283–200,913)	68,184 (49,603–91,537)	97,304 (69,119–128,974)	18,553 (12,132–26,295)	3,368,697 (3,024,456–3,732,795)	31,614 (24,034–41,492)
Cote d'Ivoire	Prevalence Number: 2015	11,788,888 (11,753,453–11,828,477)	3,449,824 (3,281,285–3,570,157)	4,687,868 (4,517,069–4,864,210)	771,869 (634,070–920,943)	4,433,788 (4,255,624–4,628,145)	207,734 (194,482–223,408)	135,157 (102,914–177,033)	133,880 (88,503–186,520)	38,386 (25,049–55,344)	7,118,610 (6,411,241–7,826,486)	62,671 (48,307–80,420)
Cote d'Ivoire	Prevalence Number: Cumulative change (%)	80.2 (79.6–80.8)	61.5 (52.0–74.1)	86.6 (77.9–96.5)	75.8 (63.3–87.9)	75.1 (65.5–86.2)	13.4 (6.2–21.2)	104.7 (34.7–196.5)	42.5 (-15.9 to 116.3)	116.5 (23.6–240.0)	111.7 (93.2–132.5)	101.1 (54.8–156.5)
Cote d'Ivoire	Prevalence Rate: 1990	98,611.2 (98,421.6–98,801.8)	30,954.7 (29,349.9–31,759.7)	39,500.2 (37,988.8–40,886.7)	7,033.6 (5,861.1–8,341.2)	38,078.6 (36,353.6–39,761.4)	2,412.4 (2,206.3–2,630.3)	1,012.1 (732.3–1,358.9)	1,190.2 (845.5–1,576.8)	273.5 (179.2–385.5)	50,622.6 (45,512.0–56,245.1)	412.9 (309.9–553.1)
Cote d'Ivoire	Prevalence Rate: 2015	97,287.7 (97,008.4–97,600.0)	27,538.6 (26,318.7–28,402.8)	39,659.4 (38,222.7–41,125.6)	6,568.8 (5,407.0–7,815.6)	36,510.1 (35,050.2–38,108.8)	1,604.6 (1,506.7–1,716.1)	1,106.0 (840.9–1,452.5)	964.1 (637.6–1,342.7)	312.8 (203.8–449.2)	58,767.8 (53,205.4–64,820.6)	472.6 (358.4–614.8)
Cote d'Ivoire	Prevalence Rate: Cumulative change (%)	-1.3 (-1.7 to -1.0)	-11.0 (-15.4 to -4.7)	0.4 (-4.1 to 5.6)	-6.5 (-13.4 to 0.1)	-4.1 (-9.4 to 2.0)	-33.4 (-37.5 to -29.1)	12.9 (-25.8 to 64.3)	-16.1 (-50.5 to 27.2)	19.7 (-31.5 to 88.4)	16.3 (6.1–27.9)	16.7 (-13.7 to 52.8)
Cote d'Ivoire	YLDs Number: 1990	543,522 (407,330–700,244)	96,354 (69,269–128,712)	60,261 (41,955–82,164)	19,687 (12,829–26,837)	16,006 (11,538–21,277)	29,512 (21,152–39,104)	4,050 (2,640–5,923)	11,878 (7,333–17,640)	7,490 (4,305–11,528)	43,105 (31,278–56,682)	1,694 (1,226–2,234)
Cote d'Ivoire	YLDs Number: 2015	882,947 (669,470–1,127,900)	151,034 (108,358–203,267)	111,763 (77,833–153,256)	34,762 (22,627–47,215)	27,319 (19,553–36,399)	33,578 (24,270–44,033)	9,018 (5,692–13,078)	16,425 (9,843–25,408)	14,360 (8,431–22,229)	86,361 (62,936–115,207)	3,668 (2,653–4,776)
Cote d'Ivoire	YLDs Number: Cumulative change (%)	62.6 (55.9–69.7)	57.1 (42.5–72.4)	85.4 (81.5–89.1)	76.8 (63.4–89.9)	71.0 (53.0–89.2)	14.0 (6.2–22.4)	125.1 (86.3–187.9)	43.3 (-14.8 to 121.1)	101.1 (14.4–221.8)	100.7 (82.4–117.1)	117.6 (85.7–152.8)
Cote d'Ivoire	YLDs Rate: 1990	8,314.9 (6,231.0–10,708.1)	1,369.9 (984.6–1,831.4)	926.1 (645.9–1,274.5)	315.0 (206.2–427.9)	233.1 (168.4–309.1)	387.9 (279.4–513.5)	59.6 (38.8–87.4)	145.3 (89.6–215.9)	110.3 (63.6–168.3)	633.2 (460.8–831.4)	23.0 (16.5–30.5)
Cote d'Ivoire	YLDs Rate: 2015	7,333.5 (5,556.8–9,344.9)	1,193.2 (855.6–1,603.2)	933.4 (651.3–1,289.8)	295.8 (193.4–401.2)	219.9 (157.3–293.1)	259.3 (187.8–340.0)	73.4 (46.4–106.9)	118.3 (70.9–183.0)	117.0 (68.6–180.9)	702.2 (512.2–938.9)	28.7 (20.8–37.6)
Cote d'Ivoire	YLDs Rate: Cumulative change (%)	-11.7 (-15.3 to -8.2)	-12.8 (-20.0 to -4.9)	0.8 (-1.2 to 2.7)	-6.0 (-13.3 to 1.2)	-5.5 (-14.9 to 3.8)	-33.0 (-37.5 to -28.3)	24.6 (3.3–60.1)	-15.6 (-49.8 to 29.8)	11.2 (-37.0 to 77.7)	11.1 (1.4–19.4)	25.4 (6.6–46.2)
The Gambia	Prevalence Number: 1990	487,750 (484,606–490,887)	184,652 (170,955–194,382)	178,313 (165,099–194,246)	28,130 (22,864–33,805)	164,234 (157,740–170,692)	16,689 (15,225–18,027)	6,528 (5,059–8,697)	10,402 (6,838–14,899)	750 (502–1,043)	91,483 (82,780–101,315)	3,980 (3,194–4,969)
The Gambia	Prevalence Number: 2015	1,071,252 (1,063,521–1,078,449)	348,915 (326,978–373,715)	398,931 (370,310–433,116)	59,991 (48,007–73,144)	344,349 (330,387–357,666)	21,313 (20,021–22,914)	16,928 (13,785–21,194)	16,939 (11,975–22,585)	1,857 (1,221–2,632)	254,194 (204,483–306,298)	9,507 (7,817–11,823)
The Gambia	Prevalence Number: Cumulative change (%)	119.6 (117.7–121.6)	89.3 (72.6–108.8)	124.2 (100.2–149.3)	113.3 (96.2–130.9)	109.8 (98.9–120.9)	27.9 (20.9–36.6)	165.2 (90.3–263.2)	72.4 (2.0–170.7)	159.1 (49.1–305.5)	178.1 (129.1–231.3)	141.2 (93.4–196.9)
The Gambia	Prevalence Rate: 1990	94,582.6 (94,026.2–95,147.2)	33,616.2 (31,732.1–35,122.5)	36,639.8 (34,151.5–39,760.7)	5,866.6 (4,822.5–6,990.3)	31,677.0 (30,421.7–32,924.5)	2,664.7 (2,448.8–2,866.6)	1,236.1 (952.3–1,655.3)	1,519.4 (999.5–2,174.6)	144.7 (96.8–201.3)	17,614.3 (15,738.6–19,784.7)	642.7 (507.4–822.9)
The Gambia	Prevalence Rate: 2015	94,235.7 (93,612.7–94,831.7)	28,772.0 (27,108.7–30,692.4)	36,751.4 (34,291.6–39,735.1)	5,537.9 (4,476.3–6,738.7)	30,144.6 (28,919.3–31,309.3)	1,662.5 (1,569.3–1,777.6)	1,470.7 (1,197.5–1,839.5)	1,186.0 (838.5–1,580.8)	164.2 (108.0–233.0)	21,946.8 (17,678.9–26,568.2)	740.0 (603.4–930.6)
The Gambia	Prevalence Rate: Cumulative change (%)	-0.4 (-1.2 to 0.5)	-14.3 (-20.9 to -6.5)	0.5 (-9.6 to 11.0)	-5.6 (-13.3 to 2.5)	-4.8 (-9.8 to 0.3)	-37.5 (-40.7 to -33.6)	21.8 (-13.2 to 67.8)	-17.4 (-51.1 to 29.7)	18.8 (-31.5 to 85.1)	24.7 (2.9–48.9)	16.7 (-10.2 to 47.2)
The Gambia	YLDs Number: 1990	37,419 (28,257–47,813)	8,211 (5,849–10,925)	3,752 (2,550–5,265)	1,268 (820–1,734)	1,050 (756–1,395)	2,692 (1,940–3,592)	433 (285–636)	1,275 (717–1,993)	295 (182–444)	2,051 (1,492–2,747)	272 (198–351)
The Gambia	YLDs Number: 2015	76,686 (57,816–98,792)	15,340 (10,808–21,101)	8,369 (5,655–11,771)	2,713 (1,742–3,765)	2,187 (1,549–2,970)	3,448 (2,521–4,521)	1,360 (888–2,082)	2,086 (1,259–3,177)	610 (368–936)	5,514 (3,940–7,429)	728 (538–942)
The Gambia	YLDs Number: Cumulative change (%)	105.0 (95.0–115.8)	87.5 (59.1–118.8)	123.0 (118.0–128.2)	113.8 (95.3–132.0)	108.4 (88.2–134.0)	28.2 (20.5–37.8)	220.0 (136.7–361.0)	73.3 (2.8–172.6)	117.0 (23.8–258.8)	169.0 (141.7–199.6)	168.1 (135.7–203.7)
The Gambia	YLDs Rate: 1990	7,275.8 (5,501.7–9,235.1)	1,460.5 (1,042.7–1,949.2)	753.3 (509.8–1,069.4)	264.6 (171.3–362.7)	195.5 (141.5–259.5)	429.9 (310.5–571.0)	81.0 (53.2–119.6)	186.3 (104.9–290.9)	56.8 (34.9–85.6)	390.0 (284.8–517.7)	47.9 (34.8–61.5)
The Gambia	YLDs Rate: 2015	6,792.2 (5,133.9–8,727.8)	1,240.9 (876.3–1,702.4)	756.0 (510.8–1,069.6)	250.5 (161.5–346.7)	184.5 (131.3–250.0)	268.9 (196.9–352.9)	117.6 (76.8–179.8)	146.1 (88.2–222.5)	54.0 (32.4–82.6)	469.9 (338.9–631.7)	60.9 (45.2–78.6)
The Gambia	YLDs Rate: Cumulative change (%)	-6.6 (-10.3 to -2.7)	-14.9 (-25.8 to -2.4)	0.4 (-1.8 to 2.6)	-5.3 (-13.4 to 2.7)	-5.6 (-14.1 to 4.3)	-37.4 (-41.0 to -33.0)	47.9 (9.6–113.2)	-16.9 (-50.7 to 30.5)	-0.5 (-43.4 to 63.3)	20.5 (10.1–32.9)	27.6 (12.0–45.7)
Ghana	Prevalence Number: 1990	7,748,863 (7,707,181–7,785,305)	2,743,411 (2,658,239–2,827,582)	2,483,736 (2,382,125–2,588,536)	372,277 (307,204–445,267)	2,672,900 (2,564,858–2,775,064)	242,385 (222,459–259,028)	87,860 (67,658–112,727)	119,357 (95,665–148,545)	12,188 (7,861–17,558)	3,082,813 (2,707,778–3,493,094)	52,935 (41,567–67,399)
Ghana	Prevalence Number: 2015	12,715,122 (12,634,538–12,798,001)	3,481,743 (3,349,713–3,738,815)	4,253,103 (4,072,511–4,429,213)	599,132 (480,979–717,314)	4,088,491 (3,924,157–4,240,809)	191,280 (178,340–204,176)	175,098 (137,796–225,542)	97,654 (72,320–128,228)	24,672 (16,268–35,448)	5,474,517 (4,527,027–6,466,640)	82,368 (65,526–103,518)
Ghana	Prevalence Number: Cumulative change (%)	64.1 (62.8–65.4)	26.9 (20.5–33.6)	71.3 (61.7–81.1)	61.1 (48.6–74.0)	53.0 (44.8–60.8)	-21.0 (-25.6 to -16.3)	104.3 (42.1–182.6)	-16.7 (-42.8 to 15.0)	114.0 (17.9–249.6)	77.5 (57.4–100.2)	57.5 (22.5–95.8)
Ghana	Prevalence Rate: 1990	96,896.2 (96,386.2–97,341.2)	32,929.4 (31,953.4–33,947.5)	32,210.6 (30,938.8–33,506.1)	4,836.3 (4,012.7–5,773.9)	33,329.1 (31,970.0–34,603.2)	2,726.1 (2,508.7–2,905.5)	1,086.3 (834.7–1,398.7)	1,274.6 (1,022.0–1,586.0)	152.2 (98.0–219.4)	38,007.3 (33,647.6–42,829.9)	596.5 (462.7–774.7)
Ghana	Prevalence Rate: 2015	94,971.4 (94,400.6–95,566.8)	25,501.3 (24,642.5–27,241.8)	32,763.7 (31,448.2–34,030.3)	4,615.3 (3,734.6–5,533.2)	30,455.2 (29,236.9–31,585.3)	1,334.2 (1,247.7–1,418.0)	1,298.4 (1,020.5–1,672.2)	633.0 (468.8–831.2)	183.5 (120.9–263.9)	40,431.8 (33,593.5–47,916.0)	572.6 (453.1–722.2)
Ghana	Prevalence Rate: Cumulative change (%)	-2.0 (-2.7 to -1.2)	-22.5 (-26.1 to -18.8)	1.8 (-3.6 to 7.3)	-4.5 (-11.8 to 3.1)	-8.6 (-13.5 to -3.9)	-51.0 (-53.8 to -48.1)	22.6 (-14.9 to 70.2)	-49.4 (-65.3 to -30.2)	27.5 (-29.6 to 108.2)	6.3 (-6.1 to 20.7)	-2.6 (-26.3 to 23.3)
Ghana	YLDs Number: 1990	609,897 (460,060–782,297)	130,132 (95,099–174,175)	41,315 (27,381–58,885)	16,752 (10,885–22,741)	14,170 (10,252–18,825)	38,958 (28,328–51,091)	5,462 (3,514–7,969)	14,545 (9,570–20,795)	4,834 (2,853–7,472)	42,457 (30,893–56,515)	3,621 (2,673–4,689)
Ghana	YLDs Number: 2015	807,278 (609,284–1,030,638)	139,366 (98,425–187,841)	70,707 (46,933–102,055)	27,131 (17,688–36,916)	19,302 (13,904–25,895)	31,172 (22,375–41,034)	13,911 (8,377–22,675)	12,106 (7,466–17,727)	7,396 (4,362–11,295)	70,583 (50,303–94,482)	6,611 (4,845–8,601)
Ghana	YLDs Number: Cumulative change (%)	32.4 (28.2–36.5)	7.0 (-2.3 to 16.1)	71.1 (67.3–74.8)	62.0 (49.1–77.4)	36.3 (18.4–47.2)	-19.9 (-24.8 to -14.8)	159.0 (81.1–296.3)	-15.4 (-41.9 to 17.6)	61.6 (-10.4 to 166.8)	66.2 (55.2–76.5)	83.2 (56.9–109.9)
Ghana	YLDs Rate: 1990	7,642.4 (5,764.1–9,796.1)	1,529.5 (1,117.4–2,054.0)	531.1 (352.0–757.2)	217.6 (142.3–297.6)	171.3 (123.7–227.8)	438.2 (319.3–576.1)	67.2 (43.2–98.4)	155.3 (102.2–222.1)	60.4 (35.6–93.3)	518.0 (376.1–689.0)	42.7 (31.5–55.5)
Ghana	YLDs Rate: 2015	6,129.3 (4,633.8–7,803.6)	1,021.3 (724.3–1,374.8)	540.1 (356.5–780.0)	208.9 (136.3–285.1)	142.2 (102.5–189.9)	217.3 (155.7–286.6)	102.7 (61.9–167.0)	78.5 (48.4–114.9)	55.0 (32.3–83.9)	520.6 (371.9–695.4)	47.6 (35.0–62.0)
Ghana	YLDs Rate: Cumulative change (%)	-19.8 (-22.1 to -17.4)	-33.3 (-38.5 to -28.2)	1.7 (-0.6 to 3.9)	-3.9 (-11.7 to 5.0)	-17.0 (-28.0 to -10.4)	-50.4 (-53.3 to -47.2)	55.4 (9.4–137.5)	-48.7 (-64.7 to -28.7)	-3.8 (-46.7 to 59.0)	0.5 (-6.0 to 6.5)	12.0 (-4.3 to 28.5)
Guinea	Prevalence Number: 1990	3,124,780 (3,109,832–3,138,267)	983,685 (864,137–1,085,746)	1,169,601 (1,088,959–1,263,928)	241,268 (198,628–287,226)	1,026,691 (988,331–1,070,597)	104,988 (97,130–114,497)	29,106 (20,542–40,070)	58,382 (41,331–78,322)	5,655 (3,642–8,116)	913,645 (778,873–1,078,960)	14,654 (10,815–19,644)
Guinea	Prevalence Number: 2015	6,453,779 (6,426,240–6,480,283)	1,877,833 (1,807,015–1,961,751)	2,475,975 (2,297,399–2,681,653)	458,870 (377,615–548,238)	2,079,580 (1,988,199–2,162,150)	109,478 (102,612–116,451)	68,542 (50,500–92,395)	88,021 (61,364–120,131)	13,135 (8,425–19,125)	2,214,421 (1,897,217–2,552,607)	32,330 (24,370–43,036)
Guinea	Prevalence Number: Cumulative change (%)	106.5 (105.4–107.7)	91.8 (71.5–118.3)	112.1 (91.1–134.1)	90.3 (76.5–105.9)	102.7 (91.2–114.1)	4.5 (-3.3 to 12.7)	145.3 (50.2–272.2)	56.6 (-4.6 to 136.7)	142.4 (38.4–279.9)	143.4 (110.8–175.3)	124.2 (68.7–191.9)
Guinea	Prevalence Rate: 1990	97,274.3 (96,849.8–97,657.2)	28,489.3 (25,362.0–31,126.9)	38,179.1 (35,631.8–40,988.7)	7,918.3 (6,526.6–9,406.2)	31,774.6 (30,591.7–33,127.0)	2,811.4 (2,615.9–3,043.5)	883.7 (616.9–1,222.9)	1,424.6 (1,010.3–1,911.4)	171.0 (109.8–244.2)	28,382.5 (24,086.9–33,686.9)	370.4 (264.5–516.0)
Guinea	Prevalence Rate: 2015	96,865.1 (96,462.3–97,248.4)	27,040.1 (26,069.7–28,238.6)	38,186.6 (35,532.1–41,150.9)	7,069.9 (5,838.1–8,427.2)	31,113.8 (29,740.1–32,356.5)	1,534.3 (1,442.0–1,629.5)	1,018.7 (747.5–1,376.5)	1,151.7 (803.8–1,571.6)	195.8 (126.0–284.3)	33,098.3 (28,208.7–38,414.8)	442.0 (331.1–592.0)
Guinea	Prevalence Rate: Cumulative change (%)	-0.4 (-0.9 to 0.1)	-4.8 (-13.9 to 7.3)	0.2 (-9.4 to 9.8)	-10.7 (-16.9 to -3.6)	-2.0 (-7.6 to 3.5)	-45.4 (-49.1 to -41.3)	20.3 (-26.8 to 83.1)	-16.1 (-48.8 to 26.7)	19.5 (-32.1 to 86.8)	17.1 (1.6–32.4)	22.2 (-12.8 to 65.3)
Guinea	YLDs Number: 1990	262,670 (196,430–337,013)	46,697 (31,634–64,335)	28,498 (19,908–39,140)	10,823 (7,135–14,903)	8,385 (5,922–11,305)	16,844 (12,150–22,315)	1,645 (1,019–2,521)	7,071 (4,373–10,708)	2,310 (1,319–3,546)	18,931 (13,453–25,272)	595 (433–779)
Guinea	YLDs Number: 2015	490,039 (366,701–624,447)	84,009 (60,995–112,365)	59,813 (41,374–81,931)	20,650 (13,633–28,106)	15,608 (11,090–21,013)	17,645 (12,715–23,315)	4,298 (2,701–6,394)	10,770 (6,654–15,973)	5,230 (3,044–8,267)	43,146 (31,547–56,714)	1,484 (1,066–1,947)
Guinea	YLDs Number: Cumulative change (%)	86.8 (74.2–96.7)	82.2 (40.7–119.0)	109.9 (104.9–114.5)	91.0 (75.7–107.7)	87.1 (58.1–115.6)	5.0 (-3.3 to 14.2)	164.5 (122.2–228.7)	58.4 (-3.5 to 142.3)	137.0 (32.4–275.9)	128.9 (97.3–154.8)	150.9 (108.7–198.6)
Guinea	YLDs Rate: 1990	8,201.5 (6,151.9–10,510.3)	1,306.4 (891.4–1,786.0)	908.5 (631.3–1,259.2)	355.1 (235.0–484.9)	247.1 (175.9–329.2)	451.0 (325.8–596.0)	49.4 (30.5–76.6)	172.5 (106.5–261.1)	69.8 (40.1–106.8)	575.0 (409.1–768.3)	15.3 (11.0–20.1)
Guinea	YLDs Rate: 2015	7,395.3 (5,533.1–9,413.6)	1,191.8 (868.5–1,590.4)	908.3 (625.0–1,242.5)	318.1 (209.8–433.0)	226.6 (161.0–305.3)	247.3 (178.5–326.8)	63.5 (40.0–94.7)	140.9 (87.1–209.0)	77.9 (45.4–122.9)	633.4 (463.9–832.3)	20.8 (14.9–27.4)

© 2017 Global Burden of Disease Child and Adolescent Health Collaboration. JAMA Pediatrics.

eTable 3. Prevalent Cases, Rates (per 100,000 population), Years Lived with Disability (YLDs), and Cumulative Percent Change with 95% Uncertainty Interval (UI) for the Top 10 Global Causes of YLDs in Children and Adolescents in 195 Countries and Territories, Aged 0 to 19 Years, Both Sexes, 1990 and 2015. *Note: best viewed by enlarging in browser.*

Location	Measure	All causes	Iron-deficiency anemia	Skin and subcutaneous diseases	Asthma	Hemoglobinopathies and hemolytic anemias	Diarrheal diseases	Congenital anomalies	Protein-energy malnutrition	Epilepsy	Malaria	Neonatal preterm birth complications
Guinea	YLDs Rate: Cumulative change (%)	-9.8 (-15.0 to -5.6)	-7.8 (-27.5 to 8.8)	-0.0 (-2.2 to 2.1)	-10.4 (-17.3 to -2.8)	-7.9 (-20.7 to 4.3)	-45.1 (-49.2 to -40.6)	30.4 (9.7–62.8)	-15.1 (-48.2 to 29.9)	16.9 (-34.7 to 85.3)	10.5 (-3.2 to 21.9)	37.3 (11.2–65.6)
Guinea-Bissau	Prevalence Number: 1990	551,476 (547,740–554,729)	201,568 (190,977–211,779)	196,502 (181,344–214,962)	35,638 (28,939–42,671)	155,192 (144,564–165,446)	16,702 (15,533–17,750)	5,363 (3,771–7,489)	9,596 (6,148–13,684)	1,013 (636–1,481)	69,816 (60,034–81,776)	2,688 (1,978–3,623)
Guinea-Bissau	Prevalence Number: 2015	893,298 (885,426–901,183)	310,626 (292,857–325,164)	335,291 (310,647–363,712)	62,530 (51,310–74,690)	241,174 (225,469–257,685)	15,194 (14,052–16,887)	9,581 (7,089–13,127)	8,977 (6,016–12,530)	2,444 (1,550–3,700)	119,983 (95,417–146,632)	4,563 (3,436–6,064)
Guinea-Bissau	Prevalence Number: Cumulative change (%)	62.0 (60.3–63.6)	54.2 (47.4–63.5)	71.0 (52.8–90.0)	75.6 (64.2–88.9)	55.6 (42.0–70.4)	-9.0 (-15.0 to -0.8)	85.9 (17.2–182.1)	-0.4 (-46.7 to 62.0)	153.5 (42.2–313.7)	71.9 (45.6–95.4)	72.2 (29.1–121.0)
Guinea-Bissau	Prevalence Rate: 1990	96,168.2 (95,570.6–96,705.8)	32,961.2 (31,475.8–34,422.7)	36,126.9 (33,618.6–39,277.8)	6,582.6 (5,405.2–7,845.4)	26,915.4 (25,069.8–28,713.6)	2,452.5 (2,286.0–2,601.0)	913.5 (640.4–1,278.4)	1,314.5 (844.8–1,874.5)	172.7 (108.2–251.9)	12,052.7 (10,308.3–14,124.2)	391.1 (282.9–540.8)
Guinea-Bissau	Prevalence Rate: 2015	94,414.3 (93,586.8–95,222.2)	31,495.2 (29,877.0–32,934.2)	36,547.3 (33,951.6–39,512.5)	6,807.8 (5,593.3–8,110.8)	25,384.2 (23,724.9–27,126.9)	1,477.6 (1,371.8–1,632.4)	998.8 (735.9–1,374.8)	806.7 (539.9–1,125.1)	255.0 (161.7–387.5)	12,497.0 (9,988.0–15,234.5)	431.6 (321.0–581.6)
Guinea-Bissau	Prevalence Rate: Cumulative change (%)	-1.8 (-2.8 to -0.8)	-4.4 (-8.1 to 1.2)	1.4 (-9.1 to 11.7)	3.5 (-3.5 to 11.3)	-5.5 (-13.9 to 3.4)	-39.7 (-43.5 to -34.6)	13.9 (-28.9 to 73.5)	-34.7 (-65.0 to 6.0)	55.2 (-12.8 to 153.6)	3.8 (-11.6 to 17.9)	12.6 (-19.6 to 50.1)
Guinea-Bissau	YLDs Number: 1990	42,599 (32,110–55,017)	9,199 (6,511–12,390)	4,159 (2,834–5,812)	1,606 (1,059–2,174)	848 (608–1,133)	2,690 (1,935–3,550)	303 (193–461)	1,169 (665–1,888)	414 (232–636)	2,419 (1,734–3,220)	107 (78–139)
Guinea-Bissau	YLDs Number: 2015	65,948 (49,864–84,050)	13,607 (9,825–18,253)	6,997 (4,771–9,789)	2,822 (1,851–3,841)	1,289 (927–1,727)	2,457 (1,771–3,244)	593 (382–867)	1,098 (653–1,648)	962 (539–1,532)	3,879 (2,854–5,110)	194 (140–254)
Guinea-Bissau	YLDs Number: Cumulative change (%)	54.9 (50.0–61.5)	48.4 (36.4–67.2)	68.2 (64.3–71.8)	75.9 (63.4–90.0)	52.2 (40.4–69.3)	-8.6 (-15.1 to -0.4)	98.0 (71.7–138.5)	0.3 (-46.6 to 65.2)	145.1 (33.4–300.4)	60.7 (48.1–78.3)	82.4 (51.1–115.8)
Guinea-Bissau	YLDs Rate: 1990	7,459.7 (5,625.2–9,586.7)	1,463.4 (1,042.0–1,963.9)	748.2 (506.5–1,054.7)	296.6 (195.1–400.2)	140.4 (101.3–187.6)	395.1 (284.1–520.4)	51.2 (32.4–78.6)	160.2 (91.1–258.6)	70.6 (39.8–108.6)	411.7 (296.0–545.7)	15.8 (11.4–20.9)
Guinea-Bissau	YLDs Rate: 2015	7,013.9 (5,309.5–8,943.7)	1,362.0 (986.5–1,824.8)	753.0 (512.1–1,061.7)	307.3 (202.7–418.4)	132.2 (95.0–176.7)	238.9 (171.9–315.0)	61.5 (39.7–90.2)	98.7 (58.7–148.2)	100.4 (55.8–159.9)	401.6 (295.8–526.9)	18.8 (13.5–24.8)
Guinea-Bissau	YLDs Rate: Cumulative change (%)	-5.9 (-8.5 to -2.7)	-6.7 (-13.4 to 3.9)	0.6 (-1.5 to 2.7)	3.7 (-3.4 to 11.7)	-5.7 (-12.6 to 3.9)	-39.5 (-43.7 to -34.4)	21.7 (5.6–47.0)	-34.2 (-64.9 to 8.4)	50.1 (-17.6 to 144.9)	-2.3 (-9.6 to 7.8)	19.8 (-2.5 to 43.9)
Liberia	Prevalence Number: 1990	1,182,242 (1,178,620–1,185,716)	372,196 (350,048–392,171)	413,970 (382,163–452,479)	74,159 (60,644–88,554)	437,933 (422,430–453,485)	39,124 (36,525–41,594)	10,912 (7,648–15,216)	16,881 (10,913–24,150)	3,428 (2,286–4,813)	533,437 (450,152–618,559)	5,726 (4,162–7,790)
Liberia	Prevalence Number: 2015	2,329,290 (2,321,056–2,337,307)	692,554 (644,486–741,730)	846,288 (783,755–924,278)	130,862 (105,901–156,065)	821,105 (793,530–852,339)	44,152 (41,503–47,488)	26,200 (19,461–34,718)	20,765 (14,428–28,028)	6,621 (4,231–9,562)	900,943 (744,187–1,071,609)	13,692 (10,451–17,695)
Liberia	Prevalence Number: Cumulative change (%)	97.0 (96.1–97.9)	86.1 (75.0–97.7)	104.9 (83.3–128.0)	76.8 (60.4–94.1)	87.6 (79.2–95.9)	12.9 (6.9–20.4)	150.2 (56.7–268.2)	30.5 (-26.7 to 109.2)	102.1 (15.1–222.4)	69.1 (46.7–92.1)	143.6 (83.0–213.0)
Liberia	Prevalence Rate: 1990	98,496.4 (98,222.3–98,755.7)	28,568.8 (27,097.5–29,944.2)	36,292.1 (33,696.1–39,322.6)	6,421.2 (5,261.7–7,623.0)	36,344.4 (35,038.2–37,641.6)	2,723.4 (2,553.5–2,887.9)	891.9 (619.6–1,245.6)	1,093.6 (708.9–1,564.0)	274.5 (182.3–384.6)	43,295.4 (36,547.8–50,429.7)	408.1 (291.2–567.7)
Liberia	Prevalence Rate: 2015	97,727.2 (97,398.9–98,053.3)	27,891.3 (26,103.9–29,743.5)	36,533.4 (33,940.8–39,696.9)	5,582.8 (4,529.4–6,650.7)	34,370.0 (33,210.0–35,689.0)	1,751.1 (1,646.0–1,876.9)	1,091.3 (809.9–1,447.8)	774.2 (538.0–1,044.9)	275.5 (176.7–395.8)	37,585.8 (30,864.2–44,783.7)	533.1 (403.9–696.4)
Liberia	Prevalence Rate: Cumulative change (%)	-0.8 (-1.2 to -0.4)	-2.3 (-7.7 to 3.2)	0.8 (-9.1 to 11.3)	-12.9 (-20.7 to -4.6)	-5.4 (-9.6 to -1.2)	-35.7 (-38.9 to -31.6)	27.7 (-20.2 to 88.6)	-24.9 (-57.8 to 20.6)	5.0 (-40.2 to 67.1)	-13.1 (-24.0 to -2.3)	33.9 (-4.3 to 76.7)
Liberia	YLDs Number: 1990	110,251 (82,102–141,954)	18,678 (13,049–25,170)	8,657 (5,897–12,092)	3,317 (2,175–4,536)	3,793 (2,691–5,073)	6,259 (4,502–8,256)	611 (383–929)	2,039 (1,172–3,215)	1,377 (808–2,099)	7,108 (5,141–9,508)	230 (169–302)
Liberia	YLDs Number: 2015	183,917 (138,433–234,950)	33,181 (23,478–44,681)	17,554 (11,908–24,855)	5,882 (3,850–8,045)	7,077 (5,031–9,479)	7,103 (5,148–9,395)	1,713 (1,097–2,552)	2,539 (1,504–3,793)	2,346 (1,370–3,539)	11,681 (8,397–15,551)	681 (490–895)
Liberia	YLDs Number: Cumulative change (%)	67.0 (57.5–76.1)	77.9 (58.6–104.2)	102.7 (97.7–107.2)	77.6 (60.3–95.5)	86.7 (71.9–104.8)	13.5 (6.6–21.7)	185.4 (131.9–272.1)	32.3 (-26.0 to 111.8)	79.0 (-0.2 to 200.1)	64.6 (52.3–77.3)	198.3 (143.2–256.0)
Liberia	YLDs Rate: 1990	9,287.2 (6,918.1–11,974.8)	1,383.4 (975.9–1,859.5)	743.1 (504.0–1,046.2)	287.1 (190.1–389.6)	300.2 (215.2–400.6)	435.5 (314.6–574.2)	49.5 (30.7–76.1)	132.1 (76.0–208.3)	110.2 (64.9–165.8)	567.5 (411.3–763.9)	16.5 (12.0–22.0)
Liberia	YLDs Rate: 2015	7,777.3 (5,853.0–9,942.5)	1,311.1 (927.1–1,765.4)	748.5 (508.3–1,058.3)	250.9 (163.5–343.6)	287.3 (204.7–383.4)	281.7 (204.7–370.9)	71.1 (45.5–105.9)	94.7 (56.1–141.4)	97.6 (57.0–148.2)	479.1 (344.2–637.3)	27.4 (19.7–36.2)
Liberia	YLDs Rate: Cumulative change (%)	-16.1 (-21.2 to -11.7)	-5.1 (-14.4 to 8.3)	0.7 (-1.6 to 2.9)	-12.5 (-20.6 to -3.9)	-4.2 (-11.3 to 3.8)	-35.3 (-39.2 to -30.9)	46.3 (18.2–91.3)	-23.8 (-57.4 to 21.8)	-7.0 (-48.1 to 54.7)	-15.5 (-21.4 to -9.5)	67.0 (34.9–101.7)
Mali	Prevalence Number: 1990	4,749,551 (4,732,023–4,767,146)	1,984,934 (1,842,817–2,114,791)	1,669,342 (1,545,037–1,836,480)	205,584 (167,960–243,061)	1,305,749 (1,262,269–1,351,880)	139,152 (126,617–152,616)	45,017 (32,535–60,709)	125,345 (89,308–165,989)	5,996 (4,025–8,454)	1,958,308 (1,784,905–2,146,115)	23,717 (17,359–31,702)
Mali	Prevalence Number: 2015	9,720,282 (9,659,048–9,779,479)	4,051,066 (3,913,779–4,191,431)	3,495,169 (3,238,323–3,807,692)	424,802 (345,513–506,726)	2,546,168 (2,468,288–2,632,824)	147,631 (139,379–156,520)	102,351 (74,622–140,732)	198,500 (134,538–273,364)	13,984 (9,333–19,871)	4,956,712 (4,142,218–5,785,353)	48,952 (36,869–64,387)
Mali	Prevalence Number: Cumulative change (%)	104.7 (103.2–106.0)	104.4 (92.4–118.1)	109.8 (87.1–133.4)	106.7 (94.3–119.2)	95.1 (87.0–103.7)	6.3 (-1.0 to 14.4)	135.6 (50.2–251.4)	64.2 (-2.4 to 151.4)	144.0 (41.8–272.6)	153.2 (114.8–190.1)	109.8 (57.4–170.7)
Mali	Prevalence Rate: 1990	97,314.6 (96,988.2–97,635.3)	39,726.3 (37,213.6–41,903.8)	35,758.6 (33,144.1–39,079.2)	4,530.4 (3,698.7–5,385.4)	26,668.1 (25,780.4–27,631.6)	2,456.5 (2,249.8–2,686.4)	900.8 (645.5–1,222.9)	2,054.8 (1,469.8–2,721.5)	122.7 (82.4–173.2)	39,261.9 (35,640.0–43,210.8)	402.8 (287.9–559.6)
Mali	Prevalence Rate: 2015	95,633.8 (95,047.8–96,231.1)	38,590.4 (37,301.2–39,806.8)	36,001.2 (33,549.1–38,996.4)	4,425.6 (3,630.5–5,297.4)	24,975.1 (24,214.2–25,821.9)	1,319.8 (1,251.2–1,396.5)	991.2 (719.9–1,363.1)	1,612.8 (1,093.5–2,219.8)	137.9 (91.6–196.4)	47,909.4 (39,809.4–56,565.1)	424.3 (314.5–564.2)
Mali	Prevalence Rate: Cumulative change (%)	-1.7 (-2.4 to -1.1)	-2.8 (-7.8 to 3.1)	0.9 (-9.4 to 11.4)	-2.3 (-8.3 to 3.8)	-6.3 (-10.2 to -2.2)	-46.2 (-49.7 to -42.2)	14.2 (-27.8 to 70.8)	-18.6 (-51.6 to 24.6)	17.5 (-31.8 to 80.4)	22.0 (3.8–40.1)	7.8 (-23.6 to 44.6)
Mali	YLDs Number: 1990	392,037 (293,014–505,704)	92,294 (64,521–126,483)	31,521 (21,078–45,351)	9,208 (6,090–12,546)	8,209 (5,808–11,118)	22,378 (16,023–29,928)	2,557 (1,611–3,861)	15,277 (9,229–23,290)	2,468 (1,467–3,749)	21,709 (15,467–29,108)	975 (720–1,292)
Mali	YLDs Number: 2015	730,573 (548,178–941,171)	194,218 (139,651–259,153)	65,514 (44,000–93,779)	19,149 (12,606–26,659)	17,095 (12,457–23,025)	23,782 (17,253–31,297)	6,445 (4,099–9,249)	24,279 (14,286–37,333)	5,653 (3,290–8,755)	56,086 (40,694–74,174)	2,246 (1,642–2,946)
Mali	YLDs Number: Cumulative change (%)	86.6 (78.2–95.3)	111.4 (85.3–136.1)	107.8 (103.5–112.2)	108.0 (94.1–122.8)	109.1 (86.6–134.5)	6.5 (-1.4 to 15.3)	154.9 (122.0–210.2)	65.1 (-3.6 to 152.9)	140.3 (37.6–280.3)	159.4 (132.9–186.3)	131.4 (92.3–172.6)
Mali	YLDs Rate: 1990	8,102.4 (6,084.2–10,389.7)	1,818.7 (1,282.5–2,464.5)	663.9 (443.9–960.0)	202.8 (134.6–276.3)	164.6 (116.7–222.4)	394.9 (282.7–525.5)	50.6 (31.6–77.3)	250.4 (151.3–381.8)	50.5 (29.8–76.6)	430.8 (309.4–573.6)	16.8 (12.2–22.4)
Mali	YLDs Rate: 2015	7,221.4 (5,419.2–9,248.9)	1,806.5 (1,297.3–2,408.8)	663.1 (441.7–958.9)	199.5 (130.9–277.0)	161.7 (117.9–217.7)	212.6 (154.2–280.0)	61.9 (39.4–88.8)	197.3 (116.2–303.3)	55.7 (32.5–86.4)	528.9 (384.2–701.1)	19.9 (14.5–26.3)
Mali	YLDs Rate: Cumulative change (%)	-10.8 (-14.4 to -7.0)	-0.4 (-11.0 to 9.5)	-0.2 (-2.2 to 2.0)	-1.6 (-8.3 to 5.4)	-1.5 (-10.8 to 8.7)	-46.1 (-49.9 to -41.8)	23.9 (8.0–49.9)	-18.2 (-52.1 to 25.2)	15.8 (-34.3 to 82.5)	23.2 (12.6–34.6)	19.3 (-2.9 to 43.2)
Mauritania	Prevalence Number: 1990	1,073,469 (1,067,009–1,080,141)	557,249 (525,565–583,519)	398,211 (367,592–432,931)	78,417 (64,888–92,813)	297,340 (278,633–316,367)	32,292 (30,142–34,836)	13,812 (10,685–17,789)	23,789 (17,484–30,595)	1,904 (1,220–2,768)	59,829 (46,469–77,465)	6,908 (5,460–8,733)
Mauritania	Prevalence Number: 2015	1,950,015 (1,937,325–1,963,005)	913,558 (878,066–949,568)	744,911 (690,597–813,366)	141,322 (113,409–170,101)	512,649 (479,788–547,562)	33,452 (31,254–35,706)	28,840 (23,051–37,060)	27,453 (17,878–39,062)	4,370 (2,806–6,508)	128,695 (97,256–169,855)	12,572 (10,147–15,611)
Mauritania	Prevalence Number: Cumulative change (%)	81.7 (80.3–83.2)	64.1 (53.6–75.3)	87.4 (68.9–108.2)	80.3 (64.0–98.5)	72.6 (58.1–86.9)	3.7 (-3.8 to 11.7)	113.8 (50.6–189.1)	19.0 (-29.9 to 87.0)	141.5 (33.0–287.4)	115.4 (92.6–147.4)	84.1 (46.1–127.4)
Mauritania	Prevalence Rate: 1990	95,535.4 (94,988.2–96,091.6)	48,587.4 (46,046.3–50,708.3)	36,732.0 (34,070.7–39,675.7)	7,294.3 (6,074.8–8,593.4)	26,392.6 (24,719.3–28,079.4)	2,556.1 (2,393.9–2,744.8)	1,213.2 (935.3–1,566.9)	1,762.9 (1,296.2–2,266.7)	168.2 (107.1–244.1)	5,224.3 (4,075.3–6,729.5)	550.7 (430.5–702.5)
Mauritania	Prevalence Rate: 2015	94,425.9 (93,826.8–95,039.3)	43,296.8 (41,796.3–44,830.5)	37,020.0 (34,382.3–40,287.7)	6,994.6 (5,637.7–8,447.3)	24,767.5 (23,181.5–26,442.7)	1,519.2 (1,423.0–1,618.4)	1,386.4 (1,108.5–1,785.4)	1,169.4 (761.7–1,663.8)	209.5 (134.5–312.4)	6,135.1 (4,669.5–8,064.0)	572.3 (460.1–714.3)
Mauritania	Prevalence Rate: Cumulative change (%)	-1.2 (-1.9 to -0.4)	-10.8 (-15.8 to -5.3)	1.0 (-8.5 to 11.6)	-4.1 (-13.0 to 5.9)	-6.0 (-13.9 to 1.7)	-40.5 (-44.6 to -36.2)	17.0 (-17.5 to 58.9)	-31.6 (-59.7 to 7.4)	31.1 (-28.0 to 111.2)	17.6 (5.5–34.7)	5.4 (-18.2 to 32.3)
Mauritania	YLDs Number: 1990	92,846 (70,067–119,260)	26,785 (19,202–35,680)	8,283 (5,612–11,644)	3,517 (2,314–4,837)	1,977 (1,428–2,617)	5,183 (3,775–6,831)	882 (578–1,251)	2,908 (1,764–4,243)	781 (456–1,208)	459 (326–631)	550 (406–709)
Mauritania	YLDs Number: 2015	150,850 (114,929–194,693)	41,631 (30,122–56,003)	15,449 (10,487–21,842)	6,368 (4,185–8,694)	3,229 (2,353–4,329)	5,402 (3,892–7,124)	2,192 (1,420–3,290)	3,363 (1,914–5,276)	1,684 (974–2,657)	857 (654–1,110)	1,092 (804–1,411)
Mauritania	YLDs Number: Cumulative change (%)	62.6 (55.9–71.7)	55.8 (39.5–81.9)	86.5 (82.6–90.3)	81.1 (64.5–99.6)	63.5 (51.2–86.1)	4.3 (-3.4 to 13.1)	151.8 (94.3–248.1)	19.7 (-29.7 to 88.8)	127.5 (24.5–272.2)	89.3 (52.7–135.8)	99.1 (74.0–127.1)
Mauritania	YLDs Rate: 1990	8,293.8 (6,268.4–10,639.9)	2,302.9 (1,659.2–3,070.5)	752.9 (509.3–1,065.0)	327.1 (215.2–449.6)	172.9 (125.5–229.5)	410.2 (298.4–539.6)	76.9 (50.5–109.4)	215.6 (130.8–314.4)	69.0 (40.3–107.0)	41.3 (29.7–56.0)	46.2 (34.1–59.6)
Mauritania	YLDs Rate: 2015	7,335.6 (5,590.8–9,427.7)	1,948.6 (1,414.9–2,630.9)	759.2 (512.8–1,075.2)	315.1 (207.2–426.8)	152.9 (111.6–205.1)	245.3 (176.7–323.9)	105.1 (68.0–158.4)	143.3 (81.5–224.8)	80.8 (46.5–126.9)	41.6 (32.0–53.7)	51.4 (37.8–66.4)
Mauritania	YLDs Rate: Cumulative change (%)	-11.5 (-14.7 to -7.2)	-15.2 (-23.4 to -2.8)	0.8 (-1.2 to 2.9)	-3.6 (-12.7 to 6.4)	-11.4 (-17.6 to -0.5)	-40.1 (-44.5 to -35.4)	38.4 (7.0–90.0)	-31.2 (-59.6 to 8.5)	23.6 (-32.3 to 102.1)	2.1 (-16.5 to 25.0)	11.6 (-2.8 to 27.5)
Niger	Prevalence Number: 1990	4,374,264 (4,358,263–4,387,610)	1,614,824 (1,533,438–1,693,503)	1,509,505 (1,421,851–1,595,737)	282,151 (231,443–336,309)	1,654,444 (1,585,563–1,725,140)	135,717 (124,895–146,326)	39,611 (28,599–53,564)	151,612 (120,434–186,981)	7,532 (4,764–11,125)	875,719 (746,923–1,040,100)	22,529 (16,762–29,780)

© 2017 Global Burden of Disease Child and Adolescent Health Collaboration. JAMA Pediatrics.

eTable 3. Prevalent Cases, Rates (per 100,000 population), Years Lived with Disability (YLDs), and Cumulative Percent Change with 95% Uncertainty Interval (UI) for the Top 10 Global Causes of YLDs in Children and Adolescents in 195 Countries and Territories, Aged 0 to 19 Years, Both Sexes, 1990 and 2015. *Note: best viewed by enlarging in browser.*

Location	Measure	All causes	Iron-deficiency anemia	Skin and subcutaneous diseases	Asthma	Hemoglobinopathies and hemolytic anemias	Diarrheal diseases	Congenital anomalies	Protein-energy malnutrition	Epilepsy	Malaria	Neonatal preterm birth complications
Niger	Prevalence Number: 2015	11,457,886 (11,407,437–11,511,545)	4,062,393 (3,896,809–4,175,371)	4,029,592 (3,807,478–4,246,672)	654,900 (531,364–785,629)	4,188,637 (4,013,364–4,363,684)	232,900 (219,062–250,914)	126,933 (96,320–181,945)	313,352 (229,264–406,361)	22,276 (14,196–32,131)	2,777,488 (2,143,488–3,516,128)	65,049 (49,281–84,164)
Niger	Prevalence Number: Cumulative change (%)	161.9 (160.6–163.4)	151.9 (132.3–168.9)	167.2 (148.7–187.6)	132.2 (115.6–150.5)	153.3 (139.4–166.6)	71.8 (60.4–84.3)	231.9 (116.5–399.7)	110.2 (42.4–190.9)	211.0 (78.3–387.4)	216.9 (166.2–274.2)	192.8 (124.6–269.7)
Niger	Prevalence Rate: 1990	96,871.9 (96,559.0–97,160.3)	33,195.0 (31,680.2–34,723.8)	35,598.9 (33,597.8–37,566.9)	6,656.1 (5,489.0–7,854.1)	36,471.4 (34,945.5–38,024.2)	2,469.5 (2,286.0–2,649.9)	844.4 (599.2–1,155.4)	2,514.5 (1,999.2–3,101.6)	162.9 (103.3–240.3)	19,327.9 (16,305.6–23,034.7)	383.3 (277.0–528.5)
Niger	Prevalence Rate: 2015	95,548.8 (95,150.3–95,983.1)	32,243.9 (31,254.0–32,964.3)	35,706.1 (33,809.4–37,489.2)	5,755.3 (4,678.8–6,888.1)	34,805.8 (33,336.6–36,261.9)	1,698.9 (1,607.9–1,817.2)	1,035.5 (781.1–1,493.2)	2,007.6 (1,468.8–2,603.7)	183.2 (116.4–267.4)	22,400.0 (17,429.9–28,338.0)	449.4 (335.8–592.2)
Niger	Prevalence Rate: Cumulative change (%)	-1.4 (-1.8 to -0.8)	-2.8 (-9.2 to 2.8)	0.4 (-6.2 to 7.8)	-13.5 (-19.8 to -6.9)	-4.5 (-9.8 to 0.6)	-31.1 (-35.5 to -26.4)	27.3 (-18.2 to 93.4)	-18.8 (-44.9 to 12.3)	18.3 (-32.0 to 85.5)	15.8 (-1.1 to 34.6)	19.9 (-13.6 to 59.2)
Niger	YLDs Number: 1990	344,435 (257,609–440,986)	73,630 (52,511–98,515)	32,091 (21,732–44,803)	12,704 (8,404–17,258)	12,329 (8,964–16,456)	21,804 (15,835–29,027)	2,290 (1,436–3,617)	18,409 (11,837–26,496)	3,108 (1,761–4,887)	14,752 (10,681–19,546)	713 (523–954)
Niger	YLDs Number: 2015	803,356 (604,678–1,034,407)	172,540 (123,022–230,917)	86,016 (58,731–120,518)	29,631 (19,352–40,275)	29,028 (20,781–38,867)	37,676 (27,344–49,882)	7,963 (5,221–11,873)	38,618 (23,380–56,691)	8,977 (4,923–13,955)	42,766 (30,781–57,123)	2,179 (1,619–2,844)
Niger	YLDs Number: Cumulative change (%)	133.3 (124.8–141.8)	134.5 (112.7–155.7)	168.0 (162.7–173.5)	133.3 (114.8–153.5)	135.4 (114.7–155.9)	73.0 (60.7–86.5)	253.5 (198.3–338.8)	113.3 (44.2–196.3)	205.0 (72.9–386.9)	190.3 (163.3–216.1)	208.4 (144.6–273.8)
Niger	YLDs Rate: 1990	7,578.0 (5,682.8–9,689.4)	1,464.7 (1,056.0–1,939.6)	740.5 (500.1–1,031.3)	299.7 (198.3–406.0)	256.6 (186.8–341.3)	396.8 (288.4–525.1)	47.8 (29.4–76.4)	305.2 (196.5–439.6)	67.3 (38.1–105.7)	318.5 (231.9–419.6)	12.2 (8.7–16.5)
Niger	YLDs Rate: 2015	6,760.4 (5,091.6–8,679.6)	1,334.5 (954.5–1,786.7)	743.7 (504.5–1,047.6)	260.3 (168.9–354.6)	231.1 (165.6–309.1)	274.8 (198.4–362.4)	64.2 (41.7–95.3)	247.5 (149.8–363.2)	73.8 (40.9–116.0)	340.9 (246.7–454.8)	15.6 (11.5–20.6)
Niger	YLDs Rate: Cumulative change (%)	-10.8 (-13.6 to -8.0)	-8.9 (-16.8 to -1.6)	0.4 (-1.6 to 2.4)	-13.1 (-20.0 to -5.8)	-9.9 (-17.6 to -2.7)	-30.7 (-35.5 to -25.5)	37.0 (15.6–70.5)	-17.6 (-44.2 to 14.3)	15.9 (-34.6 to 85.0)	7.2 (-1.8 to 15.7)	29.7 (-0.3 to 62.4)
Nigeria	Prevalence Number: 1990	52,090,685 (51,974,981–52,200,935)	21,867,318 (20,954,263–22,833,436)	16,710,588 (16,150,988–17,275,311)	3,364,970 (2,884,566–3,931,454)	26,539,968 (25,817,384–27,270,130)	1,202,593 (1,102,950–1,322,101)	473,934 (346,254–664,717)	1,086,717 (812,133–1,382,861)	88,067 (57,724–123,359)	22,959,977 (20,372,558–25,720,568)	218,762 (164,660–286,315)
Nigeria	Prevalence Number: 2015	97,334,338 (97,087,701–97,597,226)	36,161,519 (34,186,862–37,558,417)	31,920,285 (30,824,859–33,023,456)	6,394,581 (5,249,314–7,623,190)	47,487,418 (46,154,489–48,849,074)	1,209,645 (1,144,271–1,290,524)	1,021,994 (777,726–1,346,871)	1,732,929 (1,290,452–2,269,130)	193,754 (127,497–278,885)	50,774,056 (42,777,821–59,690,680)	390,480 (302,250–505,037)
Nigeria	Prevalence Number: Cumulative change (%)	86.9 (86.3–87.5)	65.5 (53.1–77.2)	91.1 (82.7–99.6)	90.0 (72.8–110.0)	79.0 (72.4–85.4)	0.8 (-5.8 to 6.9)	123.9 (40.7–236.0)	63.8 (8.5–137.0)	131.7 (29.9–274.3)	121.6 (88.7–159.1)	81.2 (37.7–132.8)
Nigeria	Prevalence Rate: 1990	98,508.0 (98,309.6–98,698.2)	40,629.0 (39,175.3–42,204.9)	33,508.0 (32,445.3–34,606.3)	6,705.4 (5,750.5–7,799.1)	50,096.7 (48,738.8–51,480.4)	1,995.3 (1,836.8–2,182.2)	879.7 (639.2–1,237.7)	1,684.5 (1,259.5–2,142.7)	164.9 (107.8–231.8)	42,245.0 (37,481.4–47,310.2)	355.4 (261.4–474.1)
Nigeria	Prevalence Rate: 2015	98,235.4 (97,999.8–98,491.3)	35,664.5 (33,819.1–36,916.7)	34,062.5 (32,914.6–35,191.1)	6,656.4 (5,457.6–7,933.5)	47,826.1 (46,486.1–49,191.6)	1,114.7 (1,056.9–1,184.8)	1,018.7 (773.3–1,346.5)	1,454.8 (1,083.4–1,904.5)	194.7 (127.8–280.8)	51,010.3 (42,803.6–60,277.7)	354.5 (269.9–464.8)
Nigeria	Prevalence Rate: Cumulative change (%)	-0.3 (-0.6 to 0.0)	-12.2 (-18.3 to -6.7)	1.7 (-2.8 to 6.1)	-0.8 (-9.7 to 9.4)	-4.5 (-8.0 to -1.0)	-44.0 (-47.5 to -40.8)	20.4 (-25.1 to 81.4)	-11.3 (-41.2 to 28.4)	24.4 (-30.2 to 102.3)	21.0 (2.4–42.7)	1.8 (-26.0 to 35.5)
Nigeria	YLDs Number: 1990	4,304,230 (3,223,391–5,519,302)	1,070,556 (771,769–1,423,707)	315,779 (216,294–446,108)	150,721 (100,598–203,453)	169,257 (123,174–223,550)	193,138 (139,636–256,725)	29,274 (18,536–44,287)	132,523 (82,201–193,695)	35,983 (21,098–54,450)	230,392 (166,905–308,439)	9,634 (7,085–12,700)
Nigeria	YLDs Number: 2015	7,295,045 (5,500,875–9,314,959)	1,637,446 (1,179,545–2,182,957)	600,370 (408,361–848,358)	287,944 (188,397–394,737)	281,212 (198,751–373,051)	195,174 (142,169–255,784)	73,116 (47,256–108,974)	213,288 (135,725–317,404)	79,180 (46,230–122,422)	451,270 (329,144–597,597)	21,297 (15,265–27,488)
Nigeria	YLDs Number: Cumulative change (%)	69.6 (59.7–77.3)	53.3 (32.1–70.3)	90.1 (86.1–94.1)	91.0 (73.1–111.7)	66.4 (40.6–86.5)	1.2 (-5.9 to 8.8)	153.8 (106.3–229.4)	65.6 (9.2–140.4)	132.7 (24.0–291.4)	96.7 (68.9–121.6)	122.2 (84.6–160.5)
Nigeria	YLDs Rate: 1990	8,228.6 (6,188.3–10,532.2)	1,947.7 (1,411.7–2,583.8)	620.6 (422.5–878.2)	300.2 (200.1–404.6)	309.6 (226.8–407.9)	320.4 (232.6–424.8)	53.9 (33.9–81.7)	205.4 (127.5–299.9)	67.3 (39.6–101.6)	418.1 (304.0–556.6)	15.9 (11.5–21.1)
Nigeria	YLDs Rate: 2015	7,474.4 (5,643.7–9,534.5)	1,587.9 (1,141.6–2,114.9)	628.0 (425.3–890.6)	299.6 (196.1–410.2)	274.5 (194.7–365.1)	179.8 (131.0–235.4)	72.3 (46.8–107.7)	179.1 (113.9–266.5)	79.5 (46.5–122.5)	445.6 (326.3–589.7)	19.8 (14.2–25.6)
Nigeria	YLDs Rate: Cumulative change (%)	-9.1 (-13.7 to -5.6)	-18.3 (-28.3 to -10.2)	1.2 (-0.9 to 3.3)	-0.2 (-9.4 to 10.3)	-11.2 (-24.7 to -1.8)	-43.8 (-47.6 to -39.7)	36.5 (11.6–77.6)	-10.3 (-40.8 to 30.2)	24.9 (-33.2 to 110.7)	6.9 (-7.0 to 19.0)	25.4 (2.3–48.1)
Sao Tome and Principe	Prevalence Number: 1990	64,236 (63,926–64,546)	19,036 (18,102–20,070)	23,695 (21,888–25,982)	4,782 (3,918–5,700)	21,692 (20,463–22,937)	1,829 (1,676–1,974)	839 (661–1,063)	541 (366–768)	121 (77–180)	7,514 (6,124–9,122)	421 (337–532)
Sao Tome and Principe	Prevalence Number: 2015	98,369 (97,877–98,916)	27,778 (26,209–29,199)	36,813 (34,200–40,134)	6,356 (5,188–7,572)	31,798 (29,963–33,689)	1,784 (1,661–1,913)	1,491 (1,204–1,884)	1,179 (780–1,697)	204 (128–299)	11,567 (9,422–13,837)	672 (544–827)
Sao Tome and Principe	Prevalence Number: Cumulative change (%)	53.1 (52.1–54.1)	46.0 (37.1–54.9)	55.7 (39.2–72.9)	33.0 (23.4–42.8)	46.7 (35.7–59.5)	-2.4 (-7.7 to 3.4)	81.7 (28.2–146.1)	129.6 (24.8–270.0)	76.8 (-1.4 to 178.9)	54.3 (40.4–70.0)	61.4 (28.7–96.3)
Sao Tome and Principe	Prevalence Rate: 1990	96,890.9 (96,455.8–97,324.3)	27,949.3 (26,738.6–29,344.2)	36,804.1 (34,117.4–40,141.6)	7,343.4 (6,065.9–8,735.8)	32,644.6 (30,795.3–34,518.0)	2,527.1 (2,323.2–2,720.7)	1,255.5 (988.0–1,591.6)	718.6 (487.3–1,020.4)	182.3 (115.5–271.9)	11,187.9 (9,139.5–13,573.2)	587.3 (466.7–751.9)
Sao Tome and Principe	Prevalence Rate: 2015	96,559.2 (96,093.6–97,065.0)	26,763.1 (25,233.2–28,071.4)	37,151.4 (34,598.4–40,404.3)	6,383.7 (5,235.1–7,592.0)	31,156.2 (29,357.8–33,007.0)	1,650.4 (1,541.5–1,763.3)	1,455.4 (1,174.8–1,843.6)	1,026.2 (678.8–1,476.2)	200.6 (127.2–294.6)	11,179.9 (9,144.8–13,352.6)	626.1 (505.4–774.4)
Sao Tome and Principe	Prevalence Rate: Cumulative change (%)	-0.3 (-1.0 to 0.3)	-4.2 (-9.5 to 1.2)	1.1 (-9.2 to 11.8)	-13.0 (-19.2 to -6.8)	-4.5 (-11.7 to 3.8)	-34.6 (-38.1 to -30.8)	18.6 (-16.5 to 60.8)	50.4 (-18.2 to 142.2)	15.7 (-35.4 to 82.6)	0.2 (-9.0 to 10.2)	8.0 (-15.4 to 32.6)
Sao Tome and Principe	YLDs Number: 1990	4,662 (3,539–5,983)	776 (553–1,046)	492 (334–693)	215 (142–295)	118 (85–157)	297 (214–395)	55 (36–79)	66 (38–104)	48 (27–75)	276 (191–376)	30 (22–38)
Sao Tome and Principe	YLDs Number: 2015	6,883 (5,154–8,907)	1,087 (775–1,481)	764 (517–1,078)	287 (189–393)	165 (118–224)	290 (208–385)	131 (77–220)	146 (83–224)	57 (32–90)	397 (281–533)	54 (40–71)
Sao Tome and Principe	YLDs Number: Cumulative change (%)	47.5 (40.5–57.7)	40.4 (25.6–52.5)	55.1 (51.7–58.6)	33.3 (23.0–43.7)	40.3 (26.1–52.9)	-2.2 (-8.1 to 4.2)	141.4 (61.7–297.1)	132.3 (27.1–274.6)	26.3 (-31.3 to 103.7)	43.8 (33.1–54.9)	79.9 (55.7–104.6)
Sao Tome and Principe	YLDs Rate: 1990	7,100.7 (5,378.9–9,133.3)	1,121.5 (799.8–1,503.8)	755.6 (511.9–1,066.7)	331.5 (220.3–452.9)	173.1 (124.6–229.7)	410.3 (296.4–547.1)	82.7 (54.0–118.2)	88.1 (51.6–138.8)	72.0 (41.0–114.2)	407.2 (283.2–553.8)	44.0 (33.1–56.3)
Sao Tome and Principe	YLDs Rate: 2015	6,860.3 (5,141.7–8,882.2)	1,038.6 (741.3–1,411.8)	761.8 (514.8–1,081.8)	288.8 (189.6–395.3)	159.7 (114.2–216.6)	268.2 (192.4–355.3)	128.4 (75.7–214.2)	127.3 (72.6–195.0)	56.4 (32.1–88.7)	381.4 (270.5–511.7)	52.3 (38.5–68.2)
Sao Tome and Principe	YLDs Rate: Cumulative change (%)	-3.5 (-7.9 to 3.4)	-7.2 (-15.9 to 0.1)	0.8 (-1.4 to 3.0)	-12.8 (-19.5 to -6.2)	-7.7 (-16.9 to 0.2)	-34.6 (-38.4 to -30.3)	57.9 (6.4–159.2)	52.1 (-16.7 to 145.4)	-17.3 (-55.3 to 33.0)	-6.2 (-13.0 to 0.9)	19.2 (3.1–35.7)
Senegal	Prevalence Number: 1990	4,133,160 (4,114,409–4,151,674)	1,857,379 (1,798,816–1,917,001)	1,517,808 (1,407,377–1,654,696)	251,451 (205,952–298,038)	1,326,247 (1,280,823–1,374,235)	141,219 (131,129–148,271)	48,558 (37,076–63,143)	50,135 (40,256–61,856)	7,953 (5,239–11,390)	572,252 (515,044–642,701)	23,864 (18,387–31,176)
Senegal	Prevalence Number: 2015	7,703,698 (7,654,742–7,752,116)	3,326,882 (3,164,453–3,513,921)	2,893,173 (2,682,209–3,183,922)	410,341 (330,849–495,704)	2,498,631 (2,395,155–2,601,710)	151,225 (142,245–160,677)	111,157 (89,099–141,292)	83,899 (66,537–103,856)	16,373 (10,723–23,327)	1,238,086 (1,005,609–1,516,699)	49,681 (39,967–61,233)
Senegal	Prevalence Number: Cumulative change (%)	86.4 (84.9–87.8)	79.2 (67.9–91.3)	91.0 (70.3–111.9)	63.2 (50.4–76.9)	88.5 (78.1–98.5)	7.2 (1.5–13.7)	134.7 (64.7–218.0)	70.2 (21.1–127.0)	115.7 (23.5–230.8)	116.4 (82.4–154.5)	111.5 (61.2–164.5)
Senegal	Prevalence Rate: 1990	95,356.4 (94,949.7–95,766.1)	41,104.2 (39,906.6–42,331.3)	36,496.4 (33,940.5–39,653.6)	6,060.0 (4,967.2–7,174.1)	30,485.5 (29,442.0–31,583.7)	2,864.5 (2,669.0–3,005.9)	1,101.1 (834.9–1,434.3)	954.4 (767.6–1,177.1)	181.7 (119.6–260.1)	13,286.7 (11,895.5–15,024.3)	481.7 (363.5–643.2)
Senegal	Prevalence Rate: 2015	94,424.2 (93,849.0–94,978.0)	39,432.5 (37,708.4–41,353.8)	36,863.3 (34,315.9–40,335.9)	5,232.2 (4,261.4–6,309.8)	30,534.2 (29,262.5–31,791.8)	1,679.6 (1,586.4–1,777.9)	1,346.7 (1,080.0–1,710.6)	855.8 (678.9–1,059.4)	200.7 (131.3–287.3)	15,044.8 (12,285.9–18,357.9)	550.2 (439.2–684.6)
Senegal	Prevalence Rate: Cumulative change (%)	-1.0 (-1.7 to -0.3)	-4.0 (-9.1 to 1.8)	1.2 (-9.2 to 11.5)	-13.6 (-20.4 to -6.2)	0.2 (-5.3 to 5.6)	-41.3 (-44.3 to -37.9)	25.5 (-12.3 to 70.4)	-8.8 (-35.1 to 21.5)	15.8 (-34.0 to 77.5)	13.3 (-3.8 to 33.0)	16.6 (-14.6 to 49.9)
Senegal	YLDs Number: 1990	329,801 (247,958–420,030)	91,348 (65,829–121,125)	31,748 (21,713–44,545)	11,321 (7,539–15,355)	7,238 (5,254–9,545)	22,686 (16,480–30,124)	3,000 (1,954–4,321)	6,062 (4,012–8,559)	3,257 (1,934–4,936)	9,749 (7,102–12,815)	1,354 (990–1,807)
Senegal	YLDs Number: 2015	553,798 (419,166–712,377)	151,011 (107,441–204,924)	60,297 (40,858–84,695)	18,554 (12,054–25,265)	12,313 (8,650–16,714)	24,475 (17,800–32,230)	8,465 (5,550–12,514)	10,310 (6,681–14,698)	5,606 (3,212–8,675)	16,960 (12,519–22,513)	3,380 (2,524–4,400)
Senegal	YLDs Number: Cumulative change (%)	68.0 (60.5–77.2)	65.7 (45.1–90.8)	89.9 (85.8–93.8)	63.8 (49.9–78.6)	70.3 (51.3–92.9)	8.0 (1.6–15.1)	186.0 (122.8–285.1)	72.8 (23.6–129.6)	80.6 (0.1–189.1)	74.4 (57.0–94.9)	151.1 (111.8–189.8)
Senegal	YLDs Rate: 1990	7,584.0 (5,725.3–9,640.6)	1,967.6 (1,419.9–2,606.0)	750.8 (511.4–1,060.5)	272.8 (180.9–368.9)	159.8 (115.8–210.3)	460.2 (334.6–611.0)	67.4 (43.9–98.0)	115.4 (76.5–162.9)	74.4 (44.0–112.2)	221.4 (162.0–289.0)	28.6 (20.8–38.2)
Senegal	YLDs Rate: 2015	6,828.2 (5,179.4–8,741.6)	1,759.2 (1,246.1–2,376.8)	755.7 (511.5–1,073.2)	236.6 (154.4–322.1)	146.3 (103.5–199.1)	271.8 (197.4–357.8)	102.1 (66.9–151.9)	105.2 (68.2–149.9)	68.7 (39.3–105.3)	204.5 (151.5–269.5)	39.4 (29.3–51.4)
Senegal	YLDs Rate: Cumulative change (%)	-9.9 (-13.4 to -5.7)	-10.4 (-20.0 to 1.8)	0.6 (-1.5 to 2.8)	-13.3 (-20.8 to -5.5)	-8.4 (-18.4 to 2.4)	-40.9 (-44.2 to -37.0)	53.5 (19.0–106.4)	-7.4 (-33.8 to 22.8)	-3.2 (-46.5 to 54.8)	-7.4 (-15.9 to 2.5)	38.8 (15.6–61.6)
Sierra Leone	Prevalence Number: 1990	2,094,599 (2,089,416–2,100,222)	650,203 (575,613–722,475)	730,089 (675,279–794,480)	129,276 (104,792–154,292)	900,229 (862,596–939,374)	56,017 (51,592–59,962)	17,976 (12,638–24,063)	28,424 (24,484–33,314)	3,814 (2,397–5,518)	835,586 (756,974–919,470)	9,281 (6,896–12,357)
Sierra Leone	Prevalence Number: 2015	3,290,917 (3,275,752–3,304,874)	880,018 (805,996–935,866)	1,218,706 (1,127,324–1,328,356)	211,510 (174,047–253,955)	1,412,465 (1,353,990–1,474,741)	53,984 (50,975–57,456)	33,142 (24,933–43,325)	44,142 (33,964–54,914)	7,278 (4,617–10,299)	1,358,689 (1,182,444–1,539,244)	14,939 (11,073–19,892)
Sierra Leone	Prevalence Number: Cumulative change (%)	57.1 (56.3–57.9)	35.9 (20.0–52.4)	67.3 (49.6–84.9)	63.8 (53.8–75.1)	57.0 (48.4–66.0)	-3.5 (-8.7 to 2.6)	92.2 (21.4–194.7)	56.7 (15.0–105.9)	100.7 (14.6–211.6)	62.7 (46.3–78.7)	63.4 (23.0–113.6)

© 2017 Global Burden of Disease Child and Adolescent Health Collaboration. JAMA Pediatrics.

eTable 3. Prevalent Cases, Rates (per 100,000 population), Years Lived with Disability (YLDs), and Cumulative Percent Change with 95% Uncertainty Interval (UI) for the Top 10 Global Causes of YLDs in Children and Adolescents in 195 Countries and Territories, Aged 0 to 19 Years, Both Sexes, 1990 and 2015. *Note: best viewed by enlarging in browser.*

Location	Measure	All causes	Iron-deficiency anemia	Skin and subcutaneous diseases	Asthma	Hemoglobinopathies and hemolytic anemias	Diarrheal diseases	Congenital anomalies	Protein-energy malnutrition	Epilepsy	Malaria	Neonatal preterm birth complications
Sierra Leone	Prevalence Rate: 1990	98,794.0 (98,556.9–99,041.9)	28,195.7 (25,237.4–31,000.2)	36,155.2 (33,642.3–39,097.6)	6,398.8 (5,221.7–7,608.9)	42,333.9 (40,562.1–44,161.7)	2,269.3 (2,096.1–2,423.6)	830.4 (579.3–1,117.9)	1,079.8 (932.0–1,262.2)	173.9 (110.2–251.3)	39,314.5 (35,363.2–43,664.2)	369.6 (267.4–506.2)
Sierra Leone	Prevalence Rate: 2015	95,827.7 (95,408.7–96,223.5)	24,884.3 (22,901.1–26,425.9)	36,466.9 (33,848.6–39,597.5)	6,289.7 (5,189.2–7,522.7)	41,033.8 (39,331.2–42,843.5)	1,486.8 (1,407.1–1,579.0)	955.9 (717.1–1,255.3)	1,146.6 (882.3–1,426.5)	210.1 (133.6–296.9)	39,479.5 (34,258.7–44,801.9)	404.3 (297.0–542.1)
Sierra Leone	Prevalence Rate: Cumulative change (%)	-3.0 (-3.5 to -2.5)	-11.5 (-21.0 to -1.8)	1.0 (-9.0 to 11.1)	-1.6 (-7.5 to 4.9)	-3.0 (-8.3 to 2.6)	-34.4 (-37.9 to -30.4)	20.2 (-24.6 to 85.1)	7.1 (-20.8 to 40.5)	27.0 (-27.6 to 97.8)	0.5 (-9.7 to 10.6)	11.7 (-19.9 to 49.6)
Sierra Leone	YLDs Number: 1990	188,580 (140,765–245,228)	43,718 (30,038–59,514)	15,269 (10,421–21,311)	5,791 (3,822–7,844)	6,978 (5,047–9,265)	8,871 (6,490–11,654)	953 (592–1,463)	3,388 (2,283–4,669)	1,518 (865–2,322)	17,407 (12,469–23,063)	337 (251–439)
Sierra Leone	YLDs Number: 2015	230,422 (173,383–297,011)	37,153 (25,925–50,221)	25,533 (17,455–35,885)	9,557 (6,348–12,904)	8,641 (6,137–11,488)	8,751 (6,296–11,607)	1,990 (1,296–2,993)	5,447 (3,443–7,753)	2,392 (1,399–3,625)	24,012 (17,205–32,299)	588 (430–773)
Sierra Leone	YLDs Number: Cumulative change (%)	22.5 (13.3–31.3)	-14.1 (-32.9 to 5.4)	67.2 (63.5–70.8)	65.2 (54.5–77.0)	24.3 (5.0–41.9)	-1.3 (-7.3 to 5.9)	115.2 (44.7–202.8)	61.9 (19.0–112.5)	65.9 (-7.9 to 163.7)	38.2 (21.9–53.1)	75.5 (44.8–109.7)
Sierra Leone	YLDs Rate: 1990	8,777.7 (6,552.8–11,368.1)	1,794.8 (1,242.1–2,426.1)	742.9 (502.8–1,044.3)	286.7 (190.0–389.5)	308.2 (223.3–409.4)	359.5 (262.3–471.2)	43.5 (26.8–67.9)	128.7 (86.5–176.4)	69.3 (39.7–105.7)	792.4 (572.9–1,048.5)	13.4 (9.9–17.8)
Sierra Leone	YLDs Rate: 2015	6,766.5 (5,087.8–8,706.5)	1,036.1 (727.9–1,402.3)	755.0 (514.4–1,067.5)	284.2 (188.6–382.5)	245.8 (175.1–328.4)	241.0 (173.3–318.8)	57.1 (37.0–85.7)	141.5 (89.5–201.4)	69.0 (40.5–104.0)	687.6 (493.6–922.8)	16.1 (11.7–21.2)
Sierra Leone	YLDs Rate: Cumulative change (%)	-22.7 (-28.0 to -17.6)	-41.7 (-53.9 to -29.5)	1.6 (-0.5 to 3.7)	-0.8 (-7.0 to 6.1)	-20.0 (-31.6 to -9.7)	-33.0 (-36.9 to -28.3)	35.4 (-9.4 to 90.0)	10.7 (-18.3 to 44.9)	4.8 (-41.9 to 66.5)	-13.1 (-22.5 to -4.5)	20.5 (-2.7 to 45.7)
Togo	Prevalence Number: 1990	2,080,775 (2,070,846–2,090,296)	554,517 (510,876–584,299)	766,670 (711,860–832,890)	146,486 (119,916–177,355)	792,669 (760,304–830,318)	68,501 (63,077–72,708)	23,714 (18,169–31,163)	23,086 (16,000–32,107)	4,265 (2,901–5,996)	920,467 (781,549–1,076,383)	13,126 (10,145–17,261)
Togo	Prevalence Number: 2015	3,666,456 (3,645,477–3,686,076)	1,072,544 (1,001,629–1,161,921)	1,392,106 (1,296,725–1,508,682)	250,749 (203,477–303,742)	1,366,761 (1,312,942–1,422,558)	69,004 (64,504–74,059)	48,048 (37,510–61,449)	35,328 (23,832–50,082)	8,296 (5,494–11,646)	1,628,534 (1,344,961–1,922,688)	25,652 (20,013–32,762)
Togo	Prevalence Number: Cumulative change (%)	76.2 (74.9–77.4)	93.7 (76.9–112.8)	81.9 (63.9–100.2)	71.4 (55.8–86.2)	72.5 (63.1–82.5)	0.8 (-4.5 to 7.7)	107.8 (42.5–188.4)	59.6 (-7.4 to 147.5)	103.9 (14.8–218.2)	77.0 (60.5–93.0)	98.0 (51.3–147.6)
Togo	Prevalence Rate: 1990	96,821.8 (96,378.9–97,243.5)	24,003.1 (22,277.5–25,148.7)	37,117.0 (34,680.0–40,084.5)	7,160.7 (5,868.2–8,588.6)	36,741.8 (35,223.4–38,492.3)	2,810.4 (2,599.4–2,976.1)	1,085.6 (829.2–1,432.3)	889.8 (616.6–1,234.0)	196.0 (134.1–277.1)	42,033.6 (35,802.7–49,142.9)	535.0 (408.8–716.0)
Togo	Prevalence Rate: 2015	95,592.4 (95,066.2–96,090.6)	26,491.5 (24,800.9–28,581.5)	37,445.4 (34,992.5–40,434.1)	6,728.1 (5,500.2–8,129.3)	35,545.1 (34,138.3–36,991.4)	1,680.8 (1,575.6–1,798.2)	1,241.0 (967.7–1,589.0)	803.2 (541.9–1,138.5)	216.3 (143.3–302.4)	41,940.8 (34,669.3–49,523.6)	615.4 (476.3–800.3)
Togo	Prevalence Rate: Cumulative change (%)	-1.3 (-2.0 to -0.6)	10.5 (1.6–20.6)	1.1 (-8.4 to 10.9)	-5.9 (-14.8 to 2.2)	-3.2 (-8.5 to 2.4)	-40.2 (-43.2 to -36.3)	17.3 (-20.1 to 63.7)	-5.9 (-45.4 to 45.8)	15.7 (-35.0 to 80.5)	-0.2 (-9.7 to 8.9)	17.0 (-13.7 to 49.8)
Togo	YLDs Number: 1990	158,546 (119,001–202,089)	24,646 (17,389–33,391)	17,144 (11,878–23,864)	6,597 (4,335–9,062)	4,845 (3,488–6,433)	11,038 (7,990–14,521)	1,467 (955–2,137)	2,807 (1,643–4,285)	1,724 (1,045–2,629)	14,237 (10,311–19,024)	718 (520–948)
Togo	YLDs Number: 2015	270,266 (203,601–344,946)	53,712 (37,822–73,779)	30,923 (21,324–43,144)	11,335 (7,385–15,498)	8,927 (6,435–11,969)	11,111 (8,081–14,604)	3,616 (2,379–5,327)	4,311 (2,508–6,617)	2,947 (1,754–4,525)	25,265 (18,137–33,334)	1,588 (1,172–2,077)
Togo	YLDs Number: Cumulative change (%)	70.6 (61.6–81.2)	119.0 (82.7–161.0)	80.3 (76.4–84.3)	71.9 (56.2–87.1)	84.6 (60.5–106.6)	0.7 (-5.2 to 8.1)	150.0 (96.0–240.1)	60.2 (-8.6 to 149.8)	80.1 (0.3–188.9)	77.8 (61.2–96.5)	122.4 (92.4–154.3)
Togo	YLDs Rate: 1990	7,413.5 (5,568.6–9,424.5)	1,043.4 (736.1–1,411.4)	812.9 (559.7–1,135.5)	322.4 (212.6–439.0)	216.4 (155.6–287.2)	452.9 (327.9–596.1)	66.6 (43.3–96.9)	108.2 (63.3–165.1)	79.2 (47.9–121.0)	641.1 (466.4–855.7)	30.5 (22.1–40.3)
Togo	YLDs Rate: 2015	7,037.5 (5,307.8–8,954.2)	1,293.8 (912.6–1,777.3)	818.1 (558.6–1,144.6)	304.1 (198.7–414.1)	223.7 (161.7–300.1)	270.8 (196.4–357.0)	92.9 (60.9–136.9)	98.0 (57.0–150.5)	76.9 (46.0–117.8)	638.4 (459.6–843.5)	39.5 (29.1–51.5)
Togo	YLDs Rate: Cumulative change (%)	-5.0 (-9.6 to 0.4)	24.5 (5.0–47.5)	0.6 (-1.5 to 2.8)	-5.6 (-14.6 to 2.6)	3.6 (-8.9 to 15.0)	-40.2 (-43.6 to -36.0)	41.8 (11.5–91.7)	-5.5 (-46.0 to 47.1)	2.3 (-42.7 to 64.1)	-0.3 (-8.6 to 9.3)	30.0 (11.8–49.4)
Eastern Sub-Saharan Africa	Prevalence Number: 1990	103,257,407 (103,111,492–103,402,166)	36,138,810 (35,139,332–37,047,239)	38,465,382 (37,682,654–39,218,230)	8,666,809 (7,297,576–10,164,292)	33,420,297 (32,937,499–33,913,899)	2,649,335 (2,461,081–2,851,972)	986,146 (888,555–1,098,855)	1,563,864 (1,292,322–1,879,470)	202,201 (168,656–240,928)	25,511,152 (23,510,789–27,663,544)	599,277 (485,488–726,927)
Eastern Sub-Saharan Africa	Prevalence Number: 2015	195,800,822 (195,396,300–196,197,011)	55,706,419 (54,641,663–57,040,772)	76,085,688 (74,579,296–77,589,373)	14,829,604 (12,386,578–17,533,553)	56,946,975 (56,056,363–57,874,611)	3,235,815 (3,035,530–3,459,104)	2,311,022 (2,106,150–2,544,223)	2,290,920 (1,960,152–2,642,818)	393,064 (321,781–468,176)	40,319,184 (35,601,184–45,491,643)	1,143,654 (946,214–1,363,102)
Eastern Sub-Saharan Africa	Prevalence Number: Cumulative change (%)	89.6 (89.2–90.0)	54.2 (49.2–60.3)	97.8 (93.6–102.3)	71.1 (62.1–80.6)	70.4 (67.5–73.6)	22.2 (18.2–26.3)	135.0 (111.2–161.4)	48.4 (16.7–86.6)	95.3 (64.7–129.2)	57.9 (46.8–69.8)	91.5 (74.5–109.9)
Eastern Sub-Saharan Africa	Prevalence Rate: 1990	96,937.9 (96,814.7–97,063.7)	32,162.9 (31,391.5–32,884.1)	37,580.4 (36,848.3–38,300.8)	8,367.8 (7,091.7–9,775.8)	31,269.0 (30,815.7–31,730.6)	2,207.6 (2,060.7–2,363.7)	906.4 (815.7–1,011.9)	1,170.7 (967.8–1,406.4)	185.7 (155.1–218.6)	23,414.2 (21,694.9–25,272.5)	474.1 (383.5–577.2)
Eastern Sub-Saharan Africa	Prevalence Rate: 2015	95,264.3 (95,077.5–95,448.1)	26,096.2 (25,638.9–26,672.8)	37,921.1 (37,217.3–38,676.0)	7,311.2 (6,106.0–8,599.5)	27,597.4 (27,165.9–28,049.2)	1,487.6 (1,399.8–1,584.6)	1,113.4 (1,013.5–1,227.2)	974.1 (833.2–1,123.6)	189.0 (156.1–224.4)	19,621.0 (17,382.8–22,122.4)	512.8 (425.3–609.7)
Eastern Sub-Saharan Africa	Prevalence Rate: Cumulative change (%)	-1.7 (-1.9 to -1.5)	-18.8 (-21.2 to -16.0)	0.9 (-1.1 to 3.1)	-12.6 (-17.1 to -7.9)	-11.7 (-13.3 to -10.1)	-32.6 (-34.5 to -30.5)	23.2 (10.5–37.3)	-15.7 (-33.7 to 5.9)	2.2 (-13.6 to 19.9)	-16.3 (-21.9 to -10.1)	8.6 (-2.5 to 20.0)
Eastern Sub-Saharan Africa	YLDs Number: 1990	8,210,296 (6,174,676–10,543,990)	1,636,323 (1,183,083–2,185,469)	812,131 (556,187–1,131,485)	390,402 (259,661–535,397)	156,546 (113,960–207,967)	427,335 (309,297–568,002)	84,068 (53,793–125,984)	190,629 (125,812–265,846)	81,515 (57,646–108,038)	345,357 (251,586–453,923)	29,696 (22,079–38,423)
Eastern Sub-Saharan Africa	YLDs Number: 2015	13,844,433 (10,378,598–17,947,385)	2,250,727 (1,620,051–2,982,003)	1,589,604 (1,089,025–2,229,783)	670,667 (442,766–924,452)	268,953 (193,583–357,975)	525,699 (378,067–691,066)	212,384 (142,561–302,854)	283,413 (191,031–384,288)	145,305 (101,760–195,973)	564,410 (410,218–750,792)	76,371 (56,351–98,172)
Eastern Sub-Saharan Africa	YLDs Number: Cumulative change (%)	68.6 (64.7–73.2)	37.6 (29.3–45.9)	95.6 (93.7–97.5)	71.8 (62.7–81.1)	71.7 (63.7–79.9)	23.1 (18.8–27.6)	155.5 (127.4–189.0)	50.4 (17.9–89.8)	79.0 (48.9–111.8)	63.5 (56.6–70.3)	157.6 (134.8–178.1)
Eastern Sub-Saharan Africa	YLDs Rate: 1990	7,796.2 (5,881.6–10,016.8)	1,425.4 (1,023.5–1,904.5)	778.8 (532.1–1,091.9)	376.8 (251.2–514.3)	141.9 (103.6–188.3)	355.9 (258.3–471.0)	77.1 (49.1–116.4)	142.7 (94.2–198.9)	74.8 (52.9–98.6)	314.7 (229.7–413.6)	25.2 (18.8–32.6)
Eastern Sub-Saharan Africa	YLDs Rate: 2015	6,824.0 (5,122.0–8,831.0)	1,048.0 (753.3–1,390.8)	782.6 (536.8–1,102.0)	330.6 (220.1–452.8)	128.3 (92.2–170.8)	241.6 (174.1–318.5)	102.1 (68.5–146.1)	120.5 (81.2–163.4)	69.8 (49.1–93.8)	271.3 (197.6–360.0)	35.8 (26.4–46.0)
Eastern Sub-Saharan Africa	YLDs Rate: Cumulative change (%)	-12.5 (-14.4 to -10.3)	-26.5 (-30.5 to -22.5)	0.5 (-0.3 to 1.3)	-12.3 (-16.8 to -7.6)	-9.6 (-13.6 to -5.6)	-32.1 (-34.3 to -29.8)	34.0 (18.8–52.6)	-14.6 (-33.0 to 7.8)	-6.2 (-22.0 to 10.8)	-13.8 (-17.2 to -10.4)	42.1 (28.9–53.6)
Burundi	Prevalence Number: 1990	3,092,145 (3,068,942–3,111,404)	572,646 (511,822–635,139)	1,066,046 (1,004,122–1,141,822)	307,290 (254,118–363,821)	931,130 (882,686–981,636)	84,848 (77,326–92,142)	28,671 (21,822–37,842)	43,110 (31,255–57,227)	6,077 (3,776–8,822)	246,114 (210,200–295,414)	18,053 (13,794–22,969)
Burundi	Prevalence Number: 2015	5,667,192 (5,608,089–5,722,876)	915,143 (802,687–1,033,291)	2,095,923 (1,972,858–2,235,039)	463,578 (380,804–550,556)	1,557,493 (1,485,230–1,625,706)	130,675 (120,318–146,159)	65,570 (51,650–84,763)	61,855 (37,733–93,962)	11,050 (6,998–16,325)	604,465 (503,540–710,639)	39,853 (30,996–51,450)
Burundi	Prevalence Number: Cumulative change (%)	83.3 (81.1–85.5)	60.4 (33.3–88.7)	96.9 (80.5–113.8)	51.0 (39.3–64.5)	67.4 (57.0–78.8)	54.1 (44.9–64.2)	134.1 (60.7–225.5)	48.2 (-15.2 to 139.1)	90.7 (7.6–211.3)	147.8 (95.2–201.9)	123.2 (77.8–178.4)
Burundi	Prevalence Rate: 1990	96,737.6 (96,040.6–97,339.0)	14,581.1 (13,038.8–16,010.4)	35,574.9 (33,690.8–37,858.8)	10,091.8 (8,436.5–11,868.3)	28,912.1 (27,387.6–30,476.8)	2,236.2 (2,059.1–2,414.4)	869.2 (655.9–1,152.4)	988.1 (716.6–1,310.0)	186.9 (118.9–268.0)	7,485.6 (6,498.5–8,873.7)	448.5 (337.5–587.3)
Burundi	Prevalence Rate: 2015	92,242.0 (91,277.7–93,127.0)	12,749.6 (11,330.0–14,218.0)	35,898.0 (33,886.8–38,023.0)	7,803.5 (6,451.3–9,244.7)	25,167.2 (23,988.4–26,270.9)	1,870.5 (1,736.1–2,067.1)	1,043.1 (819.1–1,351.8)	765.4 (467.9–1,162.2)	172.8 (109.7–256.1)	9,643.1 (8,078.0–11,301.4)	540.7 (415.9–710.6)
Burundi	Prevalence Rate: Cumulative change (%)	-4.6 (-5.8 to -3.6)	-12.3 (-25.9 to 2.0)	1.0 (-6.6 to 8.8)	-22.6 (-28.7 to -15.5)	-12.9 (-18.3 to -6.9)	-16.3 (-20.9 to -11.6)	23.0 (-16.0 to 71.7)	-20.0 (-54.2 to 29.1)	-3.1 (-45.4 to 58.4)	29.8 (3.5–57.4)	22.6 (-7.8 to 59.2)
Burundi	YLDs Number: 1990	206,950 (155,727–263,546)	21,092 (14,657–28,992)	22,915 (15,684–31,976)	13,913 (9,233–19,186)	3,333 (2,346–4,501)	13,807 (9,973–18,306)	2,448 (1,552–3,699)	5,301 (3,275–8,065)	2,476 (1,383–3,897)	7,480 (5,323–10,231)	744 (532–990)
Burundi	YLDs Number: 2015	340,354 (256,744–434,269)	31,666 (21,407–43,716)	44,802 (30,798–63,098)	21,098 (13,958–29,256)	5,320 (3,721–7,256)	21,373 (15,372–28,005)	5,816 (3,765–8,634)	7,669 (4,160–12,491)	3,796 (2,171–6,096)	21,499 (14,585–30,127)	2,056 (1,434–2,756)
Burundi	YLDs Number: Cumulative change (%)	64.7 (55.6–72.9)	51.2 (14.2–88.9)	95.5 (91.3–99.6)	51.7 (39.5–65.7)	59.6 (45.3–72.7)	54.9 (44.5–65.9)	139.9 (107.7–186.1)	49.5 (-15.3 to 140.9)	61.6 (-9.7 to 163.0)	188.5 (131.8–247.5)	177.7 (130.2–225.6)
Burundi	YLDs Rate: 1990	6,705.7 (5,051.4–8,508.7)	537.6 (376.0–740.1)	739.4 (502.4–1,044.2)	456.5 (302.5–626.5)	99.6 (70.1–133.7)	363.5 (263.7–478.5)	74.0 (46.7–112.4)	121.5 (75.0–185.0)	75.9 (42.9–117.9)	230.1 (164.4–313.4)	19.7 (14.0–26.3)
Burundi	YLDs Rate: 2015	5,685.0 (4,294.2–7,233.3)	439.2 (299.3–607.5)	746.4 (509.9–1,055.0)	354.9 (233.9–492.1)	84.5 (59.1–115.4)	305.8 (219.0–401.8)	92.1 (59.4–136.9)	94.9 (51.5–154.4)	59.3 (34.2–94.3)	345.1 (236.0–482.7)	30.5 (21.3–40.8)
Burundi	YLDs Rate: Cumulative change (%)	-15.1 (-19.7 to -11.1)	-17.8 (-36.6 to 0.8)	0.9 (-1.2 to 3.0)	-22.2 (-28.4 to -15.2)	-15.1 (-22.1 to -8.5)	-15.8 (-21.0 to -10.4)	25.8 (9.6–49.5)	-19.3 (-54.2 to 29.9)	-17.7 (-54.1 to 36.2)	50.4 (20.7–79.7)	56.0 (27.1–84.6)
Comoros	Prevalence Number: 1990	228,248 (227,179–229,458)	72,604 (66,758–76,841)	80,923 (76,295–86,470)	23,935 (19,902–28,345)	58,068 (54,773–61,395)	4,938 (4,473–5,514)	2,439 (1,956–3,149)	2,451 (1,649–3,502)	528 (335–761)	61,450 (51,276–71,632)	1,310 (1,048–1,655)
Comoros	Prevalence Number: 2015	388,641 (386,906–390,548)	109,170 (101,318–117,009)	142,596 (134,651–151,251)	32,686 (25,986–39,930)	83,569 (78,641–88,407)	6,896 (6,443–7,380)	4,954 (4,046–6,187)	6,151 (3,757–9,286)	722 (450–1,046)	103,999 (87,182–121,073)	2,485 (2,016–3,053)
Comoros	Prevalence Number: Cumulative change (%)	70.3 (69.1–71.4)	50.6 (37.8–63.8)	76.4 (62.2–91.5)	36.8 (18.4–58.9)	44.1 (33.3–54.6)	39.9 (29.5–49.2)	106.8 (49.9–169.2)	165.1 (35.9–353.6)	44.0 (-19.6 to 127.4)	69.4 (56.4–82.2)	91.8 (51.5–132.9)
Comoros	Prevalence Rate: 1990	97,134.7 (96,716.5–97,589.0)	29,504.9 (27,382.3–31,051.7)	35,985.6 (33,994.1–38,359.7)	10,417.3 (8,693.0–12,292.4)	24,653.2 (23,243.8–26,073.9)	1,888.2 (1,725.3–2,095.7)	1,024.0 (817.7–1,323.5)	851.3 (573.2–1,216.3)	219.0 (139.7–312.0)	25,953.7 (21,840.0–30,221.5)	494.2 (392.3–633.8)
Comoros	Prevalence Rate: 2015	96,504.6 (96,091.3–96,983.6)	26,034.8 (24,329.8–27,729.1)	36,379.4 (34,407.1–38,510.1)	8,269.2 (6,618.3–10,069.6)	20,704.0 (19,479.7–21,898.6)	1,611.4 (1,512.1–1,719.5)	1,221.2 (996.4–1,523.6)	1,320.0 (806.3–1,992.0)	177.3 (110.5–255.8)	25,705.0 (21,597.8–29,892.0)	573.4 (461.0–713.9)

© 2017 Global Burden of Disease Child and Adolescent Health Collaboration. JAMA Pediatrics.

eTable 3. Prevalent Cases, Rates (per 100,000 population), Years Lived with Disability (YLDs), and Cumulative Percent Change with 95% Uncertainty Interval (UI) for the Top 10 Global Causes of YLDs in Children and Adolescents in 195 Countries and Territories, Aged 0 to 19 Years, Both Sexes, 1990 and 2015. *Note: best viewed by enlarging in browser.*

Location	Measure	All causes	Iron-deficiency anemia	Skin and subcutaneous diseases	Asthma	Hemoglobinopathies and hemolytic anemias	Diarrheal diseases	Congenital anomalies	Protein-energy malnutrition	Epilepsy	Malaria	Neonatal preterm birth complications
Comoros	Prevalence Rate: Cumulative change (%)	-0.6 (-1.3 to -0.0)	-11.7 (-18.3 to -4.7)	1.2 (-6.6 to 9.4)	-20.5 (-31.0 to -8.1)	-15.9 (-22.2 to -9.8)	-14.5 (-20.4 to -9.3)	21.5 (-12.6 to 59.0)	63.7 (-15.9 to 180.1)	-14.8 (-52.4 to 34.3)	-0.9 (-8.6 to 6.8)	17.6 (-9.7 to 45.2)
Comoros	YLDs Number: 1990	16,780 (12,685–21,510)	3,060 (2,169–4,231)	1,705 (1,172–2,378)	1,079 (713–1,490)	166 (117–226)	798 (578–1,067)	206 (133–304)	300 (174–473)	211 (120–318)	1,359 (984–1,842)	103 (76–136)
Comoros	YLDs Number: 2015	26,508 (19,887–34,008)	4,450 (3,101–6,039)	2,972 (2,025–4,185)	1,479 (959–2,072)	246 (170–335)	1,117 (805–1,474)	463 (302–672)	759 (411–1,239)	247 (138–379)	2,182 (1,560–2,928)	198 (146–260)
Comoros	YLDs Number: Cumulative change (%)	58.0 (50.4–65.1)	46.0 (21.7–68.2)	74.2 (70.1–78.4)	37.2 (18.9–59.1)	48.9 (22.7–68.1)	40.2 (30.0–50.9)	128.0 (84.3–199.3)	167.9 (36.4–353.9)	23.6 (-30.6 to 98.8)	60.7 (46.8–74.7)	93.1 (67.7–119.5)
Comoros	YLDs Rate: 1990	7,246.2 (5,501.7–9,262.2)	1,221.0 (867.0–1,688.8)	742.0 (504.7–1,042.3)	469.8 (310.6–647.3)	68.3 (48.8–92.6)	305.3 (221.1–405.5)	86.4 (55.8–127.6)	104.4 (60.7–164.4)	87.4 (49.7–131.7)	564.4 (410.5–762.8)	41.6 (30.5–54.9)
Comoros	YLDs Rate: 2015	6,627.5 (4,966.8–8,491.8)	1,051.3 (737.2–1,421.5)	747.9 (508.3–1,056.2)	374.1 (244.0–524.2)	59.5 (41.5–80.8)	261.1 (188.1–345.5)	114.2 (74.4–166.4)	163.0 (88.5–266.0)	60.7 (33.8–93.3)	533.8 (382.9–714.8)	48.2 (35.6–63.2)
Comoros	YLDs Rate: Cumulative change (%)	-8.5 (-12.3 to -5.0)	-13.6 (-26.4 to -1.4)	0.8 (-1.4 to 3.1)	-20.2 (-30.9 to -7.7)	-12.6 (-26.9 to -2.1)	-14.3 (-20.2 to -8.1)	34.2 (8.9–76.3)	65.5 (-15.7 to 180.5)	-26.8 (-58.7 to 17.2)	-5.4 (-12.5 to 2.1)	16.2 (0.7–32.3)
Djibouti	Prevalence Number: 1990	296,139 (293,317–298,977)	110,216 (104,699–114,147)	110,362 (104,414–117,520)	29,288 (24,373–34,496)	93,446 (84,354–105,187)	6,014 (5,427–6,753)	3,381 (2,698–4,265)	5,108 (4,294–6,042)	708 (434–1,046)	473 (249–842)	1,401 (1,113–1,793)
Djibouti	Prevalence Number: 2015	362,550 (358,654–366,487)	134,846 (128,674–143,583)	139,482 (131,872–147,845)	30,547 (25,339–36,069)	101,154 (90,188–115,980)	5,716 (5,318–6,178)	4,715 (3,886–5,793)	9,421 (5,684–14,012)	799 (498–1,209)	441 (264–693)	1,764 (1,416–2,208)
Djibouti	Prevalence Number: Cumulative change (%)	22.4 (20.8–24.0)	22.4 (15.3–30.7)	26.5 (16.9–35.9)	4.4 (-3.4 to 12.8)	8.7 (-7.9 to 26.8)	-4.7 (-12.1 to 2.1)	42.1 (4.2–89.7)	86.3 (8.3–182.9)	19.1 (-34.0 to 90.9)	-1.0 (-43.4 to 51.4)	27.1 (0.8–55.1)
Djibouti	Prevalence Rate: 1990	95,323.7 (94,405.2–96,246.6)	34,484.3 (32,815.1–35,731.5)	36,441.1 (34,553.9–38,747.8)	9,537.9 (7,965.4–11,222.1)	29,986.5 (27,069.7–33,746.3)	1,799.4 (1,628.0–2,010.8)	1,074.1 (855.7–1,359.5)	1,442.8 (1,212.3–1,706.1)	223.0 (138.4–328.3)	148.9 (79.5–265.5)	406.1 (318.9–525.3)
Djibouti	Prevalence Rate: 2015	95,086.7 (94,059.2–96,125.8)	35,138.3 (33,577.2–37,367.2)	36,742.9 (34,749.4–38,936.6)	8,060.1 (6,686.4–9,520.9)	26,514.5 (23,639.1–30,400.0)	1,476.8 (1,375.0–1,596.0)	1,234.4 (1,016.7–1,517.0)	2,380.2 (1,436.3–3,539.9)	208.5 (130.0–315.7)	115.2 (69.3–180.7)	455.4 (364.8–571.1)
Djibouti	Prevalence Rate: Cumulative change (%)	-0.2 (-1.5 to 1.1)	1.9 (-4.0 to 8.6)	0.9 (-6.5 to 8.4)	-15.4 (-21.7 to -8.7)	-11.2 (-24.8 to 3.6)	-17.8 (-23.9 to -11.9)	17.1 (-14.3 to 56.6)	66.7 (-3.1 to 152.8)	-1.4 (-45.1 to 57.5)	-18.0 (-52.9 to 25.3)	13.5 (-11.6 to 39.8)
Djibouti	YLDs Number: 1990	21,929 (16,365–28,100)	4,508 (3,207–6,042)	2,294 (1,567–3,235)	1,323 (876–1,803)	383 (273–512)	974 (703–1,321)	298 (196–434)	629 (416–862)	281 (157–438)	17 (9–30)	97 (71–126)
Djibouti	YLDs Number: 2015	27,814 (20,483–36,204)	5,519 (3,907–7,406)	2,861 (1,947–4,045)	1,379 (926–1,879)	439 (312–596)	925 (665–1,225)	461 (298–684)	1,164 (583–1,864)	296 (168–467)	17 (10–28)	139 (103–185)
Djibouti	YLDs Number: Cumulative change (%)	26.8 (19.7–37.6)	22.5 (11.7–35.2)	24.7 (22.0–27.4)	4.3 (-3.8 to 12.8)	14.5 (3.8–29.6)	-4.9 (-12.8 to 2.5)	56.6 (23.9–110.6)	87.3 (8.7–187.7)	11.7 (-38.3 to 86.3)	7.0 (-30.5 to 48.5)	43.8 (26.2–62.5)
Djibouti	YLDs Rate: 1990	7,106.9 (5,315.0–9,126.5)	1,393.0 (990.9–1,867.5)	749.6 (511.8–1,057.2)	431.0 (286.7–584.6)	120.5 (85.3–161.0)	291.6 (210.0–394.2)	94.6 (62.0–136.7)	177.7 (117.4–243.6)	88.4 (49.4–137.0)	5.4 (2.9–9.6)	29.8 (21.8–38.9)
Djibouti	YLDs Rate: 2015	7,301.2 (5,377.5–9,500.0)	1,437.7 (1,019.5–1,932.4)	752.1 (511.6–1,064.9)	364.0 (244.5–495.9)	115.0 (81.7–156.2)	239.0 (171.5–316.5)	120.7 (77.9–179.1)	294.2 (147.3–471.1)	77.2 (44.1–121.9)	4.6 (2.6–7.5)	36.3 (26.9–48.2)
Djibouti	YLDs Rate: Cumulative change (%)	2.7 (-2.9 to 11.4)	3.3 (-5.1 to 13.3)	0.3 (-1.7 to 2.3)	-15.5 (-21.9 to -8.7)	-4.6 (-13.5 to 7.3)	-17.9 (-24.6 to -11.6)	29.4 (2.5–74.6)	67.5 (-2.7 to 157.1)	-7.5 (-49.0 to 53.4)	-11.4 (-41.7 to 22.8)	22.2 (6.9–38.1)
Eritrea	Prevalence Number: 1990	1,738,487 (1,724,745–1,750,927)	778,684 (734,487–821,086)	625,246 (586,058–668,214)	155,374 (128,791–183,108)	546,214 (489,900–620,663)	44,240 (40,026–48,375)	17,646 (13,822–22,562)	41,544 (27,070–58,699)	3,154 (1,964–4,635)	32,480 (20,683–49,016)	11,105 (8,654–14,231)
Eritrea	Prevalence Number: 2015	2,624,953 (2,603,122–2,645,216)	1,047,496 (990,532–1,085,993)	970,091 (913,224–1,034,975)	206,154 (170,112–244,693)	738,404 (648,842–848,468)	42,139 (39,265–45,822)	32,639 (26,500–39,587)	44,952 (24,692–71,653)	5,109 (3,289–7,414)	53,382 (29,226–83,531)	16,385 (13,214–20,352)
Eritrea	Prevalence Number: Cumulative change (%)	51.0 (49.5–52.5)	34.7 (25.6–43.6)	55.4 (42.9–68.6)	32.8 (23.6–42.5)	35.7 (15.2–59.1)	-4.6 (-10.7 to 2.1)	88.8 (39.2–150.5)	14.2 (-45.9 to 101.8)	71.0 (-2.9 to 174.0)	62.5 (31.1–96.5)	49.3 (18.9–85.4)
Eritrea	Prevalence Rate: 1990	96,077.9 (95,346.5–96,753.5)	41,910.5 (39,822.2–43,910.2)	35,895.8 (33,785.9–38,180.1)	8,755.9 (7,286.1–10,327.4)	30,103.6 (27,003.0–34,212.5)	2,205.0 (2,008.3–2,396.1)	958.2 (748.1–1,230.5)	1,888.8 (1,231.4–2,669.3)	171.8 (107.5–249.0)	1,698.4 (1,098.9–2,508.3)	536.3 (414.3–699.1)
Eritrea	Prevalence Rate: 2015	94,871.8 (94,122.0–95,587.2)	36,995.1 (35,245.9–38,262.1)	36,150.6 (34,143.5–38,404.1)	7,489.0 (6,219.2–8,814.2)	26,617.6 (23,394.5–30,587.0)	1,444.6 (1,351.5–1,564.3)	1,168.5 (950.7–1,421.4)	1,429.0 (785.0–2,277.6)	183.3 (118.6–264.7)	1,817.7 (1,014.2–2,819.7)	554.9 (445.9–694.8)
Eritrea	Prevalence Rate: Cumulative change (%)	-1.3 (-2.2 to -0.3)	-11.7 (-16.9 to -6.6)	0.8 (-6.7 to 9.0)	-14.4 (-20.3 to -8.3)	-11.2 (-24.6 to 4.1)	-34.4 (-38.4 to -29.9)	24.5 (-8.4 to 66.0)	-20.2 (-62.2 to 40.9)	12.6 (-36.4 to 80.8)	5.8 (-14.3 to 27.1)	5.0 (-19.3 to 33.0)
Eritrea	YLDs Number: 1990	142,022 (106,079–183,170)	34,768 (24,900–46,664)	13,249 (9,025–18,694)	6,986 (4,678–9,555)	2,550 (1,834–3,422)	7,125 (5,177–9,407)	1,458 (939–2,212)	5,072 (2,949–7,849)	1,296 (732–2,021)	1,333 (763–2,095)	606 (441–781)
Eritrea	YLDs Number: 2015	197,358 (147,966–255,214)	44,503 (31,737–60,064)	20,375 (13,886–28,534)	9,298 (6,111–12,743)	3,469 (2,430–4,697)	6,819 (4,966–8,993)	2,978 (1,959–4,369)	5,536 (2,698–9,498)	2,027 (1,170–3,146)	2,242 (1,159–3,584)	1,112 (811–1,445)
Eritrea	YLDs Number: Cumulative change (%)	39.0 (32.0–46.3)	28.4 (13.0–43.0)	53.8 (50.5–57.0)	33.2 (23.6–43.1)	36.4 (17.6–59.3)	-4.3 (-11.1 to 3.4)	107.2 (70.9–166.4)	15.3 (-45.7 to 106.4)	65.7 (-8.4 to 169.1)	67.3 (39.2–101.0)	84.0 (59.7–110.1)
Eritrea	YLDs Rate: 1990	7,931.7 (5,925.1–10,213.3)	1,842.3 (1,319.9–2,465.8)	745.6 (509.2–1,052.8)	393.5 (263.9–534.9)	138.0 (99.3–185.7)	355.0 (258.6–467.6)	79.0 (50.7–120.1)	230.6 (134.5–356.4)	70.6 (40.0–109.2)	70.3 (40.7–109.2)	31.1 (22.5–40.3)
Eritrea	YLDs Rate: 2015	7,250.7 (5,454.4–9,330.6)	1,546.8 (1,106.9–2,084.2)	746.1 (508.5–1,049.8)	337.6 (223.3–463.1)	122.3 (85.6–165.6)	233.7 (170.3–307.9)	106.5 (69.9–156.6)	176.0 (85.8–301.8)	72.7 (42.2–111.6)	76.8 (40.3–122.4)	38.8 (28.3–50.3)
Eritrea	YLDs Rate: Cumulative change (%)	-8.5 (-12.7 to -4.0)	-15.9 (-25.1 to -7.6)	0.1 (-2.1 to 2.1)	-14.1 (-20.2 to -7.9)	-11.2 (-22.4 to 2.3)	-34.1 (-38.5 to -29.1)	36.7 (12.9–75.9)	-19.4 (-62.0 to 43.7)	9.2 (-39.6 to 76.7)	8.6 (-9.7 to 30.0)	25.2 (8.7–43.1)
Ethiopia	Prevalence Number: 1990	25,968,016 (25,861,244–26,083,295)	9,664,264 (8,730,828–10,440,381)	11,166,138 (10,632,609–11,671,860)	1,915,886 (1,591,274–2,270,914)	6,488,554 (6,205,253–6,785,189)	636,838 (579,292–701,278)	228,077 (171,398–303,905)	535,533 (281,028–836,344)	43,412 (28,952–60,341)	1,341,012 (1,128,333–1,589,917)	151,812 (114,245–203,227)
Ethiopia	Prevalence Number: 2015	50,334,438 (50,048,201–50,627,254)	10,586,351 (9,849,084–11,882,901)	22,705,850 (21,750,906–23,665,816)	3,294,967 (2,716,490–3,895,615)	10,300,102 (9,789,546–10,790,952)	733,770 (686,032–786,797)	564,862 (447,333–730,908)	660,414 (424,669–933,523)	78,736 (51,249–111,817)	2,377,965 (1,814,787–3,301,135)	269,054 (213,121–344,670)
Ethiopia	Prevalence Number: Cumulative change (%)	93.8 (92.5–95.1)	9.9 (-2.8 to 28.1)	103.5 (91.5–116.6)	72.4 (56.9–94.7)	58.8 (49.1–69.2)	15.4 (7.9–23.9)	153.8 (75.4–244.7)	36.2 (-32.9 to 147.1)	90.0 (8.0–199.5)	77.3 (41.0–136.7)	80.5 (36.5–128.8)
Ethiopia	Prevalence Rate: 1990	96,549.1 (96,171.0–96,950.4)	32,946.8 (29,951.1–35,349.6)	42,812.2 (40,953.2–44,609.6)	7,339.3 (6,143.7–8,677.9)	23,993.7 (22,945.9–25,097.9)	2,101.8 (1,929.9–2,298.3)	824.6 (614.1–1,107.6)	1,569.4 (824.2–2,448.8)	159.6 (106.9–220.2)	4,927.5 (4,137.1–5,781.8)	463.3 (342.9–636.2)
Ethiopia	Prevalence Rate: 2015	95,106.7 (94,565.7–95,642.7)	19,473.6 (18,125.2–21,803.6)	43,144.4 (41,350.5–44,967.0)	6,254.1 (5,156.3–7,388.9)	19,430.9 (18,467.8–20,352.5)	1,358.0 (1,270.2–1,455.9)	1,063.0 (841.1–1,376.0)	1,187.6 (763.7–1,678.6)	148.4 (96.6–210.6)	4,464.7 (3,422.9–6,199.3)	491.1 (387.5–630.4)
Ethiopia	Prevalence Rate: Cumulative change (%)	-1.5 (-2.2 to -0.9)	-40.7 (-47.3 to -31.4)	0.8 (-4.9 to 6.9)	-14.6 (-21.9 to -3.3)	-19.0 (-24.0 to -13.7)	-35.3 (-39.0 to -31.1)	32.3 (-8.8 to 80.9)	-16.5 (-58.8 to 51.5)	-2.6 (-44.6 to 53.2)	-9.4 (-27.9 to 21.2)	8.6 (-22.4 to 40.5)
Ethiopia	YLDs Number: 1990	2,092,635 (1,572,841–2,701,179)	456,122 (310,420–621,006)	226,274 (155,518–316,890)	86,393 (57,315–119,276)	37,414 (26,015–50,501)	102,364 (73,765–136,843)	19,314 (11,840–29,574)	65,049 (32,356–110,295)	17,844 (10,765–26,278)	35,463 (25,931–46,669)	7,271 (5,240–9,601)
Ethiopia	YLDs Number: 2015	3,356,758 (2,505,168–4,361,394)	393,003 (274,695–533,225)	454,865 (310,676–641,965)	149,331 (98,466–204,258)	66,193 (46,388–89,140)	119,468 (86,736–158,366)	50,870 (33,310–74,722)	82,088 (45,432–125,690)	32,856 (18,986–50,636)	72,852 (50,534–97,763)	17,817 (12,824–23,372)
Ethiopia	YLDs Number: Cumulative change (%)	60.6 (49.3–73.4)	-13.0 (-29.9 to 10.2)	100.9 (96.5–105.1)	73.3 (57.4–96.5)	77.7 (52.3–107.7)	16.9 (8.6–26.3)	167.5 (126.7–224.2)	39.0 (-31.6 to 151.1)	92.6 (7.4–207.9)	105.7 (69.5–138.3)	147.0 (102.3–192.6)
Ethiopia	YLDs Rate: 1990	7,777.3 (5,848.9–10,021.9)	1,507.3 (1,031.7–2,040.2)	857.3 (585.3–1,204.7)	330.9 (219.5–455.4)	131.6 (92.1–177.3)	337.9 (244.2–450.7)	69.8 (42.5–107.8)	190.6 (95.0–323.3)	65.6 (39.9–96.8)	130.0 (95.6–170.2)	23.7 (17.0–31.3)
Ethiopia	YLDs Rate: 2015	6,369.6 (4,750.1–8,271.9)	722.1 (505.0–976.9)	862.2 (588.6–1,217.7)	283.4 (187.2–386.8)	123.9 (87.0–166.8)	221.1 (160.2–292.8)	95.7 (62.5–140.3)	147.6 (81.7–226.0)	61.9 (35.8–95.4)	137.0 (95.1–183.5)	33.1 (23.8–43.5)
Ethiopia	YLDs Rate: Cumulative change (%)	-18.0 (-23.3 to -12.2)	-51.7 (-60.7 to -39.5)	0.5 (-1.3 to 2.4)	-14.2 (-21.8 to -2.3)	-5.5 (-17.9 to 9.2)	-34.5 (-38.8 to -29.6)	39.5 (17.4–70.3)	-14.7 (-58.0 to 54.1)	-1.3 (-45.1 to 57.9)	5.5 (-13.0 to 21.9)	41.3 (14.1–68.3)
Kenya	Prevalence Number: 1990	13,356,016 (13,341,145–13,370,826)	4,718,924 (4,669,225–4,764,356)	4,933,012 (4,892,984–4,976,630)	1,005,407 (843,669–1,181,869)	5,872,656 (5,834,954–5,910,212)	268,342 (247,863–293,474)	184,201 (171,393–194,603)	151,507 (140,413–163,422)	18,602 (16,304–21,049)	1,385,904 (1,252,864–1,502,293)	95,894 (81,355–112,299)
Kenya	Prevalence Number: 2015	22,685,394 (22,658,247–22,712,536)	8,087,359 (8,006,164–8,171,680)	8,588,854 (8,526,532–8,659,691)	1,419,345 (1,179,905–1,691,058)	9,066,348 (9,010,540–9,116,006)	328,562 (308,181–351,963)	370,264 (349,954–389,532)	218,593 (198,701–238,868)	34,803 (30,297–40,101)	1,733,333 (1,536,382–1,941,965)	191,211 (163,825–220,325)
Kenya	Prevalence Number: Cumulative change (%)	69.9 (69.6–70.1)	71.4 (69.0–73.9)	74.1 (72.5–75.7)	41.2 (34.2–48.9)	54.4 (53.4–55.4)	22.5 (17.9–26.7)	101.2 (89.8–112.9)	44.6 (28.4–61.1)	87.3 (69.7–106.6)	25.0 (17.7–32.9)	99.6 (90.0–109.3)
Kenya	Prevalence Rate: 1990	95,309.7 (95,204.5–95,414.1)	32,578.6 (32,280.1–32,858.6)	36,534.5 (36,254.6–36,838.0)	7,338.1 (6,157.2–8,604.4)	41,759.0 (41,487.9–42,024.3)	1,749.4 (1,623.9–1,900.6)	1,299.0 (1,208.3–1,373.0)	896.7 (830.9–967.0)	132.1 (116.4–149.2)	9,844.4 (8,928.7–10,652.8)	616.8 (523.7–721.0)
Kenya	Prevalence Rate: 2015	94,085.1 (93,978.5–94,196.2)	32,645.7 (32,334.5–32,961.1)	36,655.0 (36,394.0–36,950.8)	5,988.2 (4,990.2–7,103.2)	37,494.0 (37,260.8–37,698.8)	1,290.7 (1,213.6–1,378.5)	1,524.6 (1,439.5–1,604.5)	788.3 (716.7–861.3)	144.5 (126.1–166.1)	7,087.0 (6,304.4–7,937.4)	743.4 (639.1–854.3)
Kenya	Prevalence Rate: Cumulative change (%)	-1.3 (-1.4 to -1.1)	0.2 (-1.1 to 1.6)	0.3 (-0.6 to 1.2)	-18.4 (-22.2 to -14.3)	-10.2 (-10.8 to -9.6)	-26.2 (-28.8 to -23.8)	17.5 (10.8–24.3)	-11.9 (-21.8 to -1.8)	9.4 (-0.9 to 20.5)	-28.0 (-32.2 to -23.5)	20.7 (14.4–27.0)
Kenya	YLDs Number: 1990	1,060,779 (787,792–1,387,319)	218,791 (158,305–288,560)	115,502 (79,274–160,534)	45,362 (30,185–62,136)	30,910 (22,507–40,724)	43,355 (31,385–57,190)	16,907 (11,148–23,957)	18,599 (12,781–24,779)	7,413 (5,422–9,573)	36,382 (26,250–48,522)	6,901 (5,171–8,890)

© 2017 Global Burden of Disease Child and Adolescent Health Collaboration. JAMA Pediatrics.

eTable 3. Prevalent Cases, Rates (per 100,000 population), Years Lived with Disability (YLDs), and Cumulative Percent Change with 95% Uncertainty Interval (UI) for the Top 10 Global Causes of YLDs in Children and Adolescents in 195 Countries and Territories, Aged 0 to 19 Years, Both Sexes, 1990 and 2015. *Note: best viewed by enlarging in browser.*

Location	Measure	All causes	Iron-deficiency anemia	Skin and subcutaneous diseases	Asthma	Hemoglobinopathies and hemolytic anemias	Diarrheal diseases	Congenital anomalies	Protein-energy malnutrition	Epilepsy	Malaria	Neonatal preterm birth complications
Kenya	YLDs Number: 2015	1,800,705 (1,343,248–2,361,303)	361,546 (261,865–478,534)	198,348 (135,834–278,037)	64,136 (42,326–88,398)	50,350 (36,569–66,702)	53,191 (38,370–69,997)	36,845 (24,818–52,677)	26,951 (18,407–36,813)	11,745 (8,397–15,231)	43,027 (31,044–57,559)	18,939 (14,327–24,483)
Kenya	YLDs Number: Cumulative change (%)	69.7 (64.9–77.2)	65.2 (61.3–69.5)	71.7 (69.7–73.9)	41.4 (34.4–49.2)	62.9 (59.0–66.6)	22.8 (18.0–27.0)	119.1 (96.3–151.3)	45.1 (28.6–61.6)	58.5 (42.8–76.7)	18.3 (12.2–25.7)	174.6 (160.2–190.3)
Kenya	YLDs Rate: 1990	7,701.7 (5,719.3–10,079.3)	1,482.7 (1,071.9–1,956.1)	836.8 (574.7–1,171.0)	330.9 (219.3–450.4)	215.1 (156.9–283.5)	282.5 (205.2–370.9)	119.1 (78.4–169.2)	110.1 (75.6–146.7)	52.6 (38.5–67.9)	254.0 (183.3–338.2)	47.3 (35.4–60.9)
Kenya	YLDs Rate: 2015	7,572.4 (5,641.7–9,934.9)	1,438.5 (1,042.1–1,905.3)	832.2 (568.7–1,169.2)	270.5 (179.4–369.9)	203.9 (148.1–270.2)	208.9 (151.1–275.0)	151.5 (102.2–216.4)	97.2 (66.4–132.7)	48.7 (35.0–63.4)	173.5 (125.0–232.2)	76.9 (58.0–99.1)
Kenya	YLDs Rate: Cumulative change (%)	-1.7 (-4.6 to 2.7)	-3.0 (-5.1 to -0.8)	-0.5 (-1.7 to 0.7)	-18.3 (-22.1 to -14.1)	-5.2 (-7.3 to -3.2)	-26.0 (-28.6 to -23.7)	27.9 (14.5–46.6)	-11.6 (-21.7 to -1.5)	-7.3 (-16.5 to 3.3)	-31.7 (-35.1 to -27.5)	62.6 (53.7–72.2)
Madagascar	Prevalence Number: 1990	6,446,506 (6,435,979–6,455,668)	2,404,989 (2,313,766–2,506,891)	2,112,132 (2,024,831–2,198,264)	777,129 (642,649–915,886)	2,251,179 (2,163,983–2,344,953)	170,498 (158,286–181,534)	71,094 (54,857–91,524)	128,668 (93,221–172,604)	12,294 (8,009–17,465)	3,193,283 (2,889,863–3,520,511)	32,931 (25,588–42,551)
Madagascar	Prevalence Number: 2015	12,438,907 (12,400,139–12,473,132)	4,076,688 (3,767,652–4,376,201)	4,270,955 (4,109,307–4,438,957)	1,254,203 (1,046,142–1,488,159)	3,859,381 (3,708,686–4,006,842)	210,841 (197,154–226,833)	163,444 (130,218–211,750)	292,898 (168,371–463,058)	22,544 (14,264–32,999)	4,040,894 (3,398,985–4,854,884)	68,423 (55,074–85,573)
Madagascar	Prevalence Number: Cumulative change (%)	93.0 (92.3–93.6)	69.7 (52.9–85.6)	102.3 (91.7–114.4)	61.6 (48.1–76.1)	71.5 (63.1–80.4)	23.7 (17.3–30.5)	134.9 (68.5–218.4)	135.6 (23.7–291.4)	92.0 (7.9–206.2)	26.8 (6.1–53.0)	110.5 (64.3–156.8)
Madagascar	Prevalence Rate: 1990	99,617.5 (99,489.5–99,728.7)	35,036.3 (33,874.2–36,407.1)	34,052.0 (32,645.4–35,417.6)	12,499.8 (10,397.7–14,725.7)	34,720.8 (33,396.0–36,173.6)	2,327.4 (2,172.7–2,472.7)	1,081.8 (829.9–1,396.7)	1,599.9 (1,160.1–2,144.9)	184.6 (119.3–262.1)	49,675.6 (45,353.8–54,177.2)	431.2 (328.8–565.5)
Madagascar	Prevalence Rate: 2015	97,584.6 (97,300.6–97,840.9)	31,138.8 (28,917.3–33,339.0)	34,271.0 (32,941.2–35,606.2)	9,979.7 (8,331.6–11,774.4)	30,220.6 (29,037.7–31,373.5)	1,581.9 (1,482.0–1,697.9)	1,274.2 (1,013.2–1,653.5)	2,068.6 (1,189.5–3,270.2)	175.3 (111.5–255.3)	32,218.5 (27,092.8–38,814.4)	500.7 (399.2–632.8)
Madagascar	Prevalence Rate: Cumulative change (%)	-2.0 (-2.3 to -1.7)	-11.0 (-19.7 to -3.2)	0.7 (-4.4 to 6.4)	-20.1 (-26.8 to -13.1)	-12.9 (-17.1 to -8.4)	-32.0 (-35.3 to -28.5)	20.4 (-14.3 to 63.9)	33.8 (-29.7 to 122.2)	-0.7 (-44.5 to 59.2)	-35.1 (-45.2 to -22.2)	18.1 (-11.3 to 47.6)
Madagascar	YLDs Number: 1990	558,933 (418,201–723,287)	113,714 (81,268–152,846)	46,084 (31,662–63,887)	34,834 (23,354–47,365)	9,526 (6,892–12,638)	27,280 (19,706–36,017)	6,797 (4,275–10,439)	15,573 (9,609–23,113)	4,917 (2,775–7,529)	21,497 (15,489–28,274)	1,773 (1,287–2,378)
Madagascar	YLDs Number: 2015	925,015 (691,800–1,188,737)	164,592 (117,097–224,657)	92,502 (63,525–129,453)	56,619 (37,393–78,087)	16,062 (11,476–21,738)	34,129 (24,502–45,126)	16,596 (10,764–24,595)	36,092 (19,235–58,968)	8,925 (4,960–14,417)	26,213 (19,339–34,668)	4,599 (3,370–6,067)
Madagascar	YLDs Number: Cumulative change (%)	65.6 (56.2–75.4)	45.3 (21.1–69.4)	100.6 (96.5–104.8)	62.8 (49.0–77.4)	69.0 (43.2–94.4)	25.1 (18.0–32.8)	147.1 (112.6–198.6)	140.1 (25.9–296.3)	90.6 (7.0–211.6)	22.2 (9.2–34.1)	160.8 (122.7–199.7)
Madagascar	YLDs Rate: 1990	8,666.7 (6,464.9–11,225.1)	1,612.3 (1,156.1–2,154.6)	729.2 (499.0–1,015.4)	560.2 (376.6–763.4)	141.2 (102.4–187.7)	372.4 (269.1–489.5)	103.5 (65.0–160.0)	193.6 (120.1–287.1)	73.8 (41.5–113.3)	330.4 (238.7–434.3)	25.1 (18.1–33.6)
Madagascar	YLDs Rate: 2015	7,304.5 (5,475.6–9,376.1)	1,248.3 (891.2–1,694.3)	733.9 (505.0–1,029.5)	450.5 (297.8–617.9)	123.9 (88.6–167.7)	256.0 (183.6–337.8)	129.3 (83.4–191.8)	254.9 (135.9–416.3)	69.3 (38.9–112.4)	206.5 (152.7–272.6)	35.1 (25.7–46.4)
Madagascar	YLDs Rate: Cumulative change (%)	-15.7 (-20.2 to -10.9)	-22.3 (-34.7 to -10.3)	0.6 (-1.5 to 2.6)	-19.5 (-26.3 to -12.2)	-12.1 (-24.9 to -0.0)	-31.2 (-35.1 to -27.3)	26.6 (8.7–53.1)	36.3 (-28.4 to 125.1)	-1.4 (-44.8 to 61.9)	-37.4 (-43.5 to -31.7)	40.8 (19.0–61.8)
Malawi	Prevalence Number: 1990	5,105,607 (5,080,132–5,127,178)	1,312,474 (1,204,912–1,394,398)	1,805,502 (1,703,498–1,922,780)	372,412 (309,117–439,753)	1,483,132 (1,412,605–1,557,086)	169,716 (161,176–178,127)	43,251 (32,168–59,462)	49,561 (33,184–68,142)	8,963 (5,696–13,241)	2,208,255 (1,841,620–2,548,509)	29,687 (22,035–39,358)
Malawi	Prevalence Number: 2015	9,111,559 (9,052,228–9,168,219)	2,505,667 (2,330,127–2,648,986)	3,368,341 (3,176,041–3,595,468)	678,912 (560,136–811,026)	2,363,745 (2,254,062–2,468,547)	175,986 (159,221–201,807)	98,355 (78,100–126,719)	69,726 (48,155–96,594)	16,942 (10,638–25,023)	3,611,708 (3,042,949–4,234,265)	53,332 (41,053–68,585)
Malawi	Prevalence Number: Cumulative change (%)	78.5 (77.1–79.8)	91.3 (73.8–110.5)	86.8 (71.5–102.4)	82.5 (65.0–103.6)	59.5 (49.5–69.9)	3.7 (-5.3 to 18.0)	134.6 (56.8–224.9)	47.7 (-14.2 to 136.9)	98.9 (13.9–216.6)	64.1 (44.4–86.1)	82.4 (41.9–129.1)
Malawi	Prevalence Rate: 1990	96,865.8 (96,428.5–97,236.5)	24,685.5 (23,011.3–26,024.7)	35,681.2 (33,666.8–37,882.0)	7,257.6 (6,050.4–8,524.3)	28,008.8 (26,676.5–29,404.6)	2,817.1 (2,680.8–2,955.6)	797.9 (591.9–1,104.4)	746.4 (500.1–1,024.9)	163.6 (104.1–240.7)	41,799.8 (35,075.7–48,350.2)	458.3 (338.4–627.6)
Malawi	Prevalence Rate: 2015	94,443.5 (93,842.8–95,016.2)	24,840.3 (23,237.6–26,176.4)	36,053.1 (34,002.3–38,342.7)	7,116.5 (5,902.0–8,450.4)	24,424.5 (23,291.0–25,504.4)	1,699.5 (1,547.1–1,932.8)	1,006.9 (798.0–1,298.0)	621.8 (430.0–861.1)	171.4 (107.2–252.5)	37,368.7 (31,306.2–43,925.9)	498.0 (380.0–651.1)
Malawi	Prevalence Rate: Cumulative change (%)	-2.5 (-3.2 to -1.8)	0.8 (-7.8 to 9.3)	1.1 (-6.8 to 9.0)	-1.9 (-11.1 to 9.1)	-12.7 (-18.1 to -7.0)	-39.7 (-44.6 to -31.9)	30.4 (-13.5 to 81.4)	-12.6 (-49.1 to 40.6)	10.2 (-36.9 to 75.2)	-10.4 (-20.8 to 1.0)	11.0 (-18.1 to 44.8)
Malawi	YLDs Number: 1990	375,584 (282,691–490,191)	51,765 (36,601–70,102)	38,285 (26,231–53,631)	16,795 (11,019–23,162)	4,395 (3,090–5,967)	27,660 (20,034–36,478)	3,710 (2,300–5,670)	6,084 (3,573–9,330)	3,535 (2,018–5,611)	28,612 (20,502–38,096)	1,068 (766–1,423)
Malawi	YLDs Number: 2015	620,097 (467,459–798,273)	98,854 (70,049–133,462)	70,805 (48,157–99,805)	30,761 (20,224–42,770)	8,418 (5,970–11,341)	28,642 (20,450–38,202)	8,800 (5,666–12,967)	8,599 (5,084–13,131)	5,368 (3,084–8,223)	48,338 (34,915–64,498)	2,492 (1,789–3,342)
Malawi	YLDs Number: Cumulative change (%)	65.3 (56.1–73.7)	91.4 (69.8–114.7)	84.9 (81.1–88.5)	83.3 (64.8–104.3)	92.0 (71.8–115.4)	3.6 (-6.1 to 17.6)	140.8 (107.8–190.3)	48.3 (-14.5 to 136.3)	60.2 (-7.8 to 160.1)	69.3 (55.8–84.8)	135.2 (90.9–179.2)
Malawi	YLDs Rate: 1990	7,319.2 (5,501.5–9,552.1)	977.3 (691.5–1,322.7)	741.7 (507.1–1,047.1)	327.1 (215.0–449.9)	83.0 (58.9–112.6)	458.5 (330.1–605.2)	68.3 (42.0–105.6)	91.6 (53.8–140.3)	64.4 (36.3–101.4)	539.4 (387.3–716.3)	17.3 (12.2–23.2)
Malawi	YLDs Rate: 2015	6,510.9 (4,909.3–8,366.7)	968.9 (685.8–1,304.0)	745.4 (508.7–1,054.6)	322.3 (213.8–449.6)	84.8 (60.1–114.8)	276.5 (198.6–367.5)	89.9 (57.8–132.4)	76.7 (45.3–117.1)	54.3 (31.1–82.8)	492.3 (357.4–656.7)	24.3 (17.4–32.6)
Malawi	YLDs Rate: Cumulative change (%)	-10.9 (-15.8 to -6.4)	-0.7 (-11.5 to 10.1)	0.5 (-1.6 to 2.5)	-1.4 (-11.0 to 9.5)	2.5 (-8.2 to 13.8)	-39.7 (-45.0 to -32.1)	33.8 (14.8–61.8)	-12.2 (-49.2 to 39.6)	-11.0 (-49.1 to 44.6)	-8.6 (-15.2 to -0.7)	41.6 (12.4–69.7)
Mozambique	Prevalence Number: 1990	7,718,548 (7,698,192–7,737,586)	2,581,658 (2,428,924–2,685,381)	2,626,775 (2,511,288–2,734,399)	559,429 (461,938–662,432)	2,171,806 (2,063,497–2,280,568)	161,958 (146,324–180,296)	61,902 (46,097–82,838)	113,658 (70,044–175,189)	20,856 (13,381–30,805)	2,781,744 (2,431,996–3,138,755)	45,549 (33,555–60,164)
Mozambique	Prevalence Number: 2015	15,256,157 (15,202,425–15,307,253)	4,682,253 (4,476,332–4,906,799)	5,373,253 (5,148,656–5,598,228)	1,176,042 (970,348–1,396,578)	3,744,292 (3,552,273–3,935,574)	204,767 (192,503–217,959)	161,292 (129,176–209,513)	126,388 (74,208–194,433)	49,787 (30,867–72,484)	5,664,829 (4,689,828–6,595,453)	81,909 (63,687–103,613)
Mozambique	Prevalence Number: Cumulative change (%)	97.7 (96.9–98.5)	81.6 (68.5–99.0)	104.7 (93.4–115.9)	110.5 (92.9–131.2)	72.5 (61.7–84.4)	26.7 (17.1–35.4)	168.2 (83.8–274.2)	20.9 (-44.8 to 123.6)	151.2 (36.9–303.7)	103.7 (82.2–126.8)	83.1 (38.5–131.0)
Mozambique	Prevalence Rate: 1990	98,062.5 (97,826.0–98,279.9)	31,293.2 (29,611.6–32,354.1)	34,687.3 (33,210.7–36,123.9)	7,236.4 (5,983.6–8,553.3)	27,509.3 (26,143.3–28,889.1)	1,858.6 (1,691.7–2,056.2)	769.9 (570.4–1,032.2)	1,196.7 (737.5–1,843.6)	257.0 (164.7–377.5)	35,186.4 (30,953.4–39,627.1)	489.8 (355.4–668.2)
Mozambique	Prevalence Rate: 2015	97,007.6 (96,697.9–97,319.5)	28,679.7 (27,506.5–29,987.3)	35,308.1 (33,812.8–36,752.9)	7,602.8 (6,266.6–9,012.9)	23,736.2 (22,514.8–24,958.4)	1,224.3 (1,153.1–1,296.1)	1,013.6 (812.0–1,321.5)	688.7 (404.4–1,059.1)	310.8 (192.7–451.3)	35,765.9 (29,607.8–41,533.8)	469.7 (364.5–602.9)
Mozambique	Prevalence Rate: Cumulative change (%)	-1.1 (-1.4 to -0.7)	-8.3 (-13.9 to -0.1)	1.8 (-3.8 to 7.4)	5.2 (-3.3 to 15.1)	-13.6 (-19.1 to -7.7)	-34.0 (-38.8 to -29.6)	35.7 (-7.7 to 90.3)	-37.4 (-71.4 to 15.7)	27.2 (-30.8 to 104.7)	1.7 (-9.3 to 13.9)	-1.8 (-28.9 to 27.4)
Mozambique	YLDs Number: 1990	649,927 (487,119–856,141)	114,587 (82,387–154,285)	54,587 (37,257–75,861)	25,142 (16,644–34,737)	6,541 (4,635–8,788)	26,064 (18,750–34,661)	5,310 (3,248–8,323)	13,878 (7,558–22,846)	8,523 (4,988–13,285)	46,098 (33,205–60,955)	1,661 (1,177–2,258)
Mozambique	YLDs Number: 2015	1,137,316 (849,562–1,475,325)	197,010 (139,613–263,536)	110,614 (75,355–154,426)	53,036 (34,446–72,425)	10,901 (7,664–14,706)	33,159 (24,017–43,355)	14,743 (9,629–21,620)	15,621 (8,049–26,427)	17,825 (9,770–28,339)	88,016 (63,067–116,715)	4,016 (2,869–5,286)
Mozambique	YLDs Number: Cumulative change (%)	75.5 (61.2–88.3)	72.4 (52.7–98.5)	102.6 (98.1–107.0)	111.3 (93.7–132.7)	67.2 (43.2–93.9)	27.5 (17.0–37.2)	183.3 (136.5–253.9)	22.1 (-44.4 to 126.9)	120.2 (17.4–254.4)	91.1 (78.8–106.5)	144.5 (96.8–196.4)
Mozambique	YLDs Rate: 1990	8,357.4 (6,273.4–10,982.2)	1,362.9 (980.5–1,831.4)	709.2 (482.1–991.3)	325.1 (216.8–448.9)	79.3 (56.2–106.3)	299.0 (215.2–397.5)	66.0 (40.3–104.3)	146.1 (79.7–240.5)	105.0 (61.7–163.2)	569.4 (410.4–750.4)	18.7 (13.2–25.6)
Mozambique	YLDs Rate: 2015	7,330.5 (5,492.5–9,497.5)	1,194.3 (849.0–1,602.1)	716.2 (486.4–1,001.5)	342.8 (223.2–470.5)	66.6 (46.8–89.9)	198.2 (143.2–258.5)	92.4 (60.3–136.1)	85.1 (43.9–144.0)	111.2 (60.8–176.5)	547.2 (393.3–726.0)	24.1 (17.2–31.8)
Mozambique	YLDs Rate: Cumulative change (%)	-12.0 (-19.3 to -5.8)	-12.2 (-21.1 to -0.5)	1.0 (-1.2 to 3.2)	5.6 (-3.2 to 16.0)	-15.7 (-26.8 to -2.8)	-33.6 (-38.9 to -28.6)	42.9 (19.1–79.1)	-36.8 (-71.2 to 17.8)	11.5 (-40.7 to 80.0)	-3.8 (-9.5 to 3.3)	30.5 (3.7–59.6)
Rwanda	Prevalence Number: 1990	4,171,719 (4,143,721–4,200,027)	1,361,294 (1,214,243–1,484,088)	1,430,415 (1,340,972–1,525,897)	515,703 (425,494–615,221)	886,805 (844,575–928,987)	108,264 (98,249–118,124)	39,841 (30,551–51,356)	48,265 (36,697–62,467)	8,818 (5,695–12,530)	299,876 (224,850–414,153)	23,252 (17,890–30,256)
Rwanda	Prevalence Number: 2015	5,697,944 (5,651,910–5,739,847)	1,586,329 (1,509,072–1,655,263)	2,065,103 (1,945,975–2,195,564)	595,607 (494,193–712,741)	1,182,263 (1,121,740–1,238,027)	92,605 (87,206–98,657)	64,790 (51,860–83,427)	25,774 (15,651–38,509)	11,456 (7,161–16,536)	179,375 (157,444–203,424)	30,609 (23,819–39,908)
Rwanda	Prevalence Number: Cumulative change (%)	36.6 (35.3–37.9)	16.8 (9.2–27.6)	44.5 (33.3–56.6)	15.7 (4.2–27.1)	33.4 (24.2–42.6)	-14.3 (-19.7 to -8.3)	66.4 (17.9–130.6)	-45.1 (-69.6 to -14.0)	35.6 (-22.0 to 117.4)	-38.6 (-54.5 to -23.9)	33.4 (4.8–66.5)
Rwanda	Prevalence Rate: 1990	96,219.3 (95,621.3–96,842.8)	29,973.8 (27,218.2–31,979.4)	34,514.8 (32,511.1–36,692.6)	12,137.0 (10,073.3–14,440.7)	20,372.4 (19,410.3–21,335.8)	2,186.3 (2,002.1–2,367.7)	900.0 (690.2–1,163.9)	878.4 (668.6–1,135.5)	199.1 (129.8–282.3)	6,643.7 (5,088.2–8,959.1)	462.9 (348.9–618.2)
Rwanda	Prevalence Rate: 2015	94,910.3 (94,153.8–95,592.2)	25,764.6 (24,521.4–26,834.6)	35,054.9 (33,091.4–37,208.3)	9,907.0 (8,231.1–11,853.1)	19,657.1 (18,650.2–20,598.0)	1,492.0 (1,406.8–1,585.6)	1,073.1 (859.9–1,382.6)	395.9 (240.4–591.7)	188.7 (119.2–270.9)	2,966.2 (2,613.0–3,351.0)	489.4 (380.3–641.5)
Rwanda	Prevalence Rate: Cumulative change (%)	-1.4 (-2.3 to -0.4)	-13.9 (-18.4 to -7.0)	1.7 (-5.8 to 9.6)	-18.3 (-26.3 to -10.6)	-3.4 (-10.1 to 3.2)	-31.7 (-35.6 to -27.2)	22.1 (-13.9 to 69.8)	-53.7 (-74.4 to -27.5)	-1.1 (-42.6 to 57.2)	-54.3 (-65.2 to -43.9)	7.6 (-18.2 to 37.1)
Rwanda	YLDs Number: 1990	297,036 (221,643–382,062)	53,174 (36,509–72,254)	26,395 (18,011–37,053)	23,311 (15,335–32,115)	3,376 (2,336–4,526)	17,596 (12,718–23,623)	3,356 (2,112–5,093)	5,917 (3,821–8,787)	3,596 (2,107–5,457)	6,412 (4,565–8,773)	1,126 (798–1,504)
Rwanda	YLDs Number: 2015	365,658 (272,253–472,452)	59,791 (41,956–80,161)	37,633 (25,517–53,091)	26,999 (17,821–37,245)	4,335 (2,998–5,876)	15,115 (10,921–19,968)	5,790 (3,743–8,526)	3,197 (1,728–5,230)	3,611 (2,033–5,838)	4,418 (3,267–5,763)	1,855 (1,308–2,462)
Rwanda	YLDs Number: Cumulative change (%)	23.1 (17.8–30.7)	13.0 (2.6–28.9)	42.4 (38.9–46.0)	16.1 (4.6–27.7)	28.9 (11.0–50.7)	-14.0 (-19.7 to -7.5)	74.6 (47.3–117.4)	-44.5 (-69.6 to -12.3)	4.9 (-41.0 to 67.5)	-30.4 (-41.3 to -19.7)	66.0 (36.6–97.9)

© 2017 Global Burden of Disease Child and Adolescent Health Collaboration. JAMA Pediatrics.

eTable 3. Prevalent Cases, Rates (per 100,000 population), Years Lived with Disability (YLDs), and Cumulative Percent Change with 95% Uncertainty Interval (UI) for the Top 10 Global Causes of YLDs in Children and Adolescents in 195 Countries and Territories, Aged 0 to 19 Years, Both Sexes, 1990 and 2015. *Note: best viewed by enlarging in browser.*

Location	Measure	All causes	Iron-deficiency anemia	Skin and subcutaneous diseases	Asthma	Hemoglobinopathies and hemolytic anemias	Diarrheal diseases	Congenital anomalies	Protein-energy malnutrition	Epilepsy	Malaria	Neonatal preterm birth complications
Rwanda	YLDs Rate: 1990	7,020.1 (5,241.7–9,030.0)	1,159.1 (799.4–1,563.0)	623.8 (422.3–879.9)	548.3 (361.6–749.3)	76.3 (53.3–102.7)	355.0 (256.4–473.3)	75.8 (47.4–115.2)	107.7 (69.6–160.1)	81.1 (47.9–122.9)	143.2 (102.3–194.7)	23.4 (16.5–31.4)
Rwanda	YLDs Rate: 2015	6,166.1 (4,598.9–7,971.5)	963.5 (676.9–1,289.5)	633.7 (429.2–898.6)	449.0 (295.1–614.8)	71.0 (49.1–95.9)	243.4 (175.6–321.4)	95.8 (61.9–141.3)	49.1 (26.6–80.3)	59.5 (33.7–95.3)	72.8 (54.0–95.0)	30.1 (21.2–40.0)
Rwanda	YLDs Rate: Cumulative change (%)	-12.2 (-15.8 to -7.0)	-16.6 (-22.9 to -7.1)	1.5 (-0.7 to 3.8)	-18.0 (-26.0 to -10.0)	-6.8 (-18.3 to 7.4)	-31.3 (-35.7 to -26.5)	28.0 (7.8–60.3)	-53.2 (-74.3 to -26.0)	-23.5 (-57.3 to 22.6)	-48.7 (-56.2 to -41.6)	30.0 (5.9–56.0)
Somalia	Prevalence Number: 1990	3,427,497 (3,411,904–3,443,871)	1,437,246 (1,332,844–1,531,746)	1,196,613 (1,131,580–1,269,949)	317,818 (262,530–373,581)	909,795 (860,199–963,522)	71,679 (65,593–78,208)	30,611 (23,293–40,605)	70,833 (41,370–111,280)	7,544 (4,575–11,240)	298,851 (242,580–369,567)	19,403 (14,625–25,753)
Somalia	Prevalence Number: 2015	6,079,542 (6,049,506–6,108,002)	2,665,103 (2,556,634–2,800,651)	2,163,511 (2,041,113–2,315,963)	480,277 (392,254–572,082)	1,374,167 (1,300,872–1,446,684)	106,914 (102,160–112,235)	60,452 (47,125–78,973)	119,054 (66,299–188,706)	12,239 (7,786–18,271)	691,058 (520,152–897,151)	35,853 (27,274–46,722)
Somalia	Prevalence Number: Cumulative change (%)	77.4 (76.2–78.5)	85.8 (71.5–102.0)	81.0 (66.7–96.5)	51.2 (39.9–62.7)	51.2 (40.5–62.3)	49.5 (38.0–60.9)	102.4 (38.0–179.2)	82.8 (-13.5 to 236.6)	70.9 (-3.5 to 176.8)	130.9 (94.2–170.6)	87.7 (42.5–137.9)
Somalia	Prevalence Rate: 1990	97,017.7 (96,613.9–97,432.7)	39,162.7 (36,847.9–41,347.4)	35,669.3 (33,860.4–37,790.9)	9,391.2 (7,813.8–11,014.6)	25,610.3 (24,225.1–27,122.3)	1,739.2 (1,606.5–1,885.1)	839.9 (633.7–1,126.4)	1,483.9 (866.8–2,323.4)	202.4 (124.2–302.6)	8,319.2 (6,855.2–10,064.4)	442.9 (329.3–604.4)
Somalia	Prevalence Rate: 2015	96,843.0 (96,401.6–97,259.9)	41,502.5 (40,148.7–43,355.4)	35,819.0 (33,865.3–38,152.4)	7,859.6 (6,438.1–9,351.1)	21,809.8 (20,645.8–22,968.3)	1,564.1 (1,496.2–1,636.6)	946.1 (735.6–1,241.9)	1,542.2 (858.7–2,442.5)	190.2 (120.9–284.4)	10,694.9 (8,174.9–13,722.8)	492.0 (370.6–653.1)
Somalia	Prevalence Rate: Cumulative change (%)	-0.2 (-0.8 to 0.4)	6.1 (-1.1 to 14.9)	0.5 (-7.0 to 8.9)	-16.3 (-22.2 to -9.9)	-14.8 (-20.8 to -8.4)	-9.9 (-16.1 to -3.9)	15.6 (-21.4 to 60.1)	12.9 (-46.5 to 107.5)	-1.1 (-44.0 to 59.4)	28.4 (9.6–48.7)	13.7 (-18.7 to 50.8)
Somalia	YLDs Number: 1990	279,295 (207,926–360,658)	63,652 (44,820–85,599)	25,537 (17,572–35,476)	14,272 (9,519–19,595)	2,959 (2,055–4,038)	11,563 (8,336–15,441)	2,587 (1,624–3,989)	8,650 (4,374–14,281)	3,099 (1,737–4,857)	5,613 (4,051–7,435)	740 (538–982)
Somalia	YLDs Number: 2015	494,084 (368,088–648,122)	121,911 (87,561–163,493)	45,639 (31,323–63,888)	21,572 (14,268–29,412)	5,420 (3,779–7,497)	17,229 (12,587–22,538)	5,270 (3,372–7,831)	14,641 (7,298–25,552)	5,037 (2,844–8,079)	11,671 (8,501–15,546)	1,463 (1,039–1,956)
Somalia	YLDs Number: Cumulative change (%)	77.0 (67.1–87.5)	92.2 (71.0–116.5)	78.7 (74.8–82.6)	51.2 (39.2–63.3)	84.1 (50.0–121.8)	49.4 (37.2–62.3)	105.5 (84.5–134.4)	83.9 (-13.6 to 237.2)	71.9 (-3.0 to 177.1)	108.5 (85.8–134.7)	99.0 (63.0–136.6)
Somalia	YLDs Rate: 1990	8,108.3 (6,038.5–10,459.3)	1,737.8 (1,233.6–2,336.3)	740.3 (506.4–1,031.1)	421.4 (282.9–574.9)	81.1 (57.1–110.6)	280.2 (202.1–373.5)	70.9 (44.0–109.7)	181.2 (91.8–298.9)	83.0 (45.9–129.3)	157.6 (114.5–208.9)	17.9 (13.0–23.9)
Somalia	YLDs Rate: 2015	8,021.4 (6,015.1–10,481.0)	1,896.6 (1,376.6–2,524.8)	740.4 (505.4–1,039.6)	352.8 (233.9–477.3)	84.4 (59.2–116.5)	251.8 (183.7–330.5)	82.3 (52.8–122.5)	189.7 (94.7–330.6)	78.2 (44.8–124.3)	180.6 (132.3–238.8)	21.3 (15.1–28.7)
Somalia	YLDs Rate: Cumulative change (%)	-1.0 (-6.3 to 4.8)	9.4 (-0.7 to 22.2)	-0.0 (-2.1 to 2.2)	-16.2 (-22.4 to -9.5)	4.5 (-15.2 to 24.7)	-9.9 (-16.6 to -2.9)	17.2 (5.2–34.0)	13.6 (-46.5 to 108.7)	-0.5 (-43.5 to 60.8)	14.8 (3.5–27.8)	20.0 (-3.7 to 44.0)
South Sudan	Prevalence Number: 1990	3,084,942 (3,069,058–3,100,038)	1,224,788 (1,140,729–1,296,069)	1,099,197 (1,034,984–1,171,456)	322,591 (266,946–384,828)	992,072 (892,040–1,119,634)	84,963 (77,631–93,462)	26,967 (20,163–36,052)	86,910 (48,629–133,491)	6,972 (4,506–10,304)	674,337 (518,787–825,277)	13,402 (10,212–17,172)
South Sudan	Prevalence Number: 2015	6,219,245 (6,187,584–6,249,846)	2,305,833 (2,185,280–2,427,130)	2,288,089 (2,165,148–2,442,692)	555,004 (458,400–662,222)	1,775,392 (1,584,012–2,022,110)	133,755 (124,540–143,568)	62,786 (48,422–81,152)	176,519 (117,068–250,509)	12,682 (8,001–18,293)	1,099,280 (825,185–1,451,839)	27,910 (21,551–35,919)
South Sudan	Prevalence Number: Cumulative change (%)	101.6 (100.3–102.8)	88.6 (72.3–104.4)	108.4 (92.2–125.6)	72.3 (58.7–86.6)	79.6 (52.5–111.2)	57.6 (48.0–66.8)	139.5 (63.2–234.1)	123.7 (13.0–283.2)	90.4 (11.8–201.0)	63.4 (27.7–95.7)	110.4 (65.7–161.3)
South Sudan	Prevalence Rate: 1990	96,619.8 (96,163.7–97,034.9)	36,638.3 (34,585.8–38,374.6)	35,990.6 (33,980.0–38,221.1)	10,492.5 (8,712.4–12,443.1)	30,931.1 (27,823.4–34,903.8)	2,299.8 (2,118.7–2,511.8)	821.5 (608.4–1,105.6)	2,068.0 (1,158.6–3,173.8)	206.1 (133.0–302.9)	20,284.0 (15,929.9–24,541.0)	350.6 (262.6–469.8)
South Sudan	Prevalence Rate: 2015	95,971.6 (95,508.5–96,426.4)	34,789.8 (33,065.5–36,502.9)	36,114.3 (34,211.6–38,452.0)	8,656.4 (7,165.8–10,294.7)	27,332.8 (24,398.8–31,134.2)	1,980.1 (1,849.1–2,121.4)	958.9 (738.2–1,240.9)	2,436.9 (1,616.0–3,458.0)	192.3 (122.2–276.5)	16,684.3 (12,597.2–21,883.8)	395.0 (304.2–513.3)
South Sudan	Prevalence Rate: Cumulative change (%)	-0.7 (-1.3 to -0.1)	-4.9 (-12.4 to 2.3)	0.4 (-7.0 to 8.1)	-17.4 (-23.7 to -10.6)	-11.3 (-24.7 to 4.3)	-13.8 (-18.6 to -9.2)	20.2 (-18.5 to 68.2)	29.7 (-34.5 to 122.1)	-2.4 (-42.9 to 53.2)	-17.7 (-35.2 to -1.9)	14.3 (-13.7 to 44.3)
South Sudan	YLDs Number: 1990	266,777 (200,125–342,197)	52,339 (37,474–69,962)	23,091 (15,961–32,280)	14,480 (9,547–19,900)	4,049 (2,885–5,454)	13,658 (9,843–18,108)	2,227 (1,382–3,465)	10,659 (5,255–17,785)	2,857 (1,718–4,402)	7,424 (5,319–9,886)	600 (439–784)
South Sudan	YLDs Number: 2015	510,993 (383,794–653,417)	96,235 (68,172–128,545)	47,579 (32,555–66,545)	24,971 (16,593–34,216)	7,625 (5,394–10,262)	21,519 (15,484–28,253)	5,416 (3,405–8,085)	21,717 (12,775–33,518)	5,217 (3,002–8,223)	11,417 (8,206–15,298)	1,431 (1,024–1,871)
South Sudan	YLDs Number: Cumulative change (%)	91.6 (81.1–102.6)	84.5 (57.5–112.9)	106.0 (101.6–110.4)	72.7 (59.1–87.9)	88.8 (62.1–115.9)	57.8 (47.7–67.5)	145.6 (115.9–183.4)	125.5 (13.1–287.6)	91.7 (10.8–208.3)	54.4 (33.9–75.3)	139.4 (100.1–180.5)
South Sudan	YLDs Rate: 1990	8,481.4 (6,370.2–10,820.9)	1,544.0 (1,102.3–2,071.9)	739.3 (511.3–1,038.3)	470.7 (313.2–644.6)	123.1 (87.8–164.9)	369.4 (267.6–488.4)	67.8 (41.5–105.7)	253.6 (125.0–423.2)	84.3 (50.6–129.2)	224.3 (162.0–298.3)	16.5 (12.0–21.6)
South Sudan	YLDs Rate: 2015	7,942.2 (5,988.4–10,157.7)	1,436.2 (1,015.0–1,920.9)	743.1 (506.3–1,046.0)	389.4 (259.1–532.3)	115.4 (81.7–155.2)	318.5 (229.1–419.7)	82.6 (51.9–123.6)	299.8 (176.5–462.7)	79.1 (45.6–124.2)	173.0 (125.0–231.9)	21.0 (15.1–27.6)
South Sudan	YLDs Rate: Cumulative change (%)	-6.3 (-11.1 to -1.5)	-6.8 (-19.1 to 5.7)	0.5 (-1.5 to 2.5)	-17.2 (-23.6 to -10.3)	-6.2 (-17.9 to 5.7)	-13.7 (-18.8 to -8.8)	23.2 (8.3–42.5)	30.7 (-34.5 to 124.7)	-1.6 (-43.3 to 56.5)	-22.7 (-32.0 to -13.1)	28.3 (6.1–51.0)
Tanzania	Prevalence Number: 1990	14,055,387 (14,022,145–14,086,663)	5,751,133 (5,464,796–6,050,104)	5,069,751 (4,886,409–5,251,416)	1,149,446 (954,306–1,365,158)	4,924,179 (4,732,830–5,101,227)	330,051 (303,849–357,995)	116,156 (88,411–150,449)	182,902 (127,085–249,852)	32,206 (21,532–45,047)	6,378,353 (6,101,751–6,654,182)	70,825 (56,770–90,376)
Tanzania	Prevalence Number: 2015	27,999,090 (27,858,278–28,136,964)	10,027,019 (9,774,328–10,430,657)	10,527,562 (10,130,085–10,931,236)	2,213,907 (1,829,858–2,637,768)	9,133,726 (8,815,885–9,460,319)	470,486 (440,176–503,504)	301,812 (244,708–372,728)	234,163 (137,924–360,776)	65,808 (44,328–93,212)	8,360,501 (6,980,827–9,927,797)	151,864 (121,613–190,908)
Tanzania	Prevalence Number: Cumulative change (%)	99.2 (98.2–100.3)	74.5 (65.6–83.3)	107.7 (97.7–118.2)	92.9 (74.2–113.3)	85.6 (76.6–95.1)	42.7 (35.3–50.9)	166.4 (88.7–259.1)	33.4 (-32.7 to 122.8)	115.0 (21.7–242.5)	31.1 (9.6–56.2)	117.0 (67.9–165.5)
Tanzania	Prevalence Rate: 1990	97,596.5 (97,385.0–97,790.7)	39,196.1 (37,464.0–40,971.4)	36,728.6 (35,489.2–37,998.4)	8,161.0 (6,811.4–9,630.5)	34,137.5 (32,818.4–35,362.1)	2,048.5 (1,897.6–2,207.1)	790.6 (597.6–1,025.2)	1,035.7 (720.2–1,414.0)	219.4 (147.0–307.3)	42,110.2 (40,282.6–43,919.9)	421.1 (332.5–548.2)
Tanzania	Prevalence Rate: 2015	95,123.8 (94,694.9–95,533.6)	33,175.0 (32,393.6–34,367.7)	37,153.7 (35,782.0–38,505.0)	7,626.0 (6,343.3–9,068.3)	30,875.8 (29,809.4–31,973.7)	1,483.6 (1,394.9–1,581.2)	1,009.2 (818.4–1,250.2)	662.9 (390.8–1,021.1)	219.9 (148.1–309.3)	29,494.3 (24,676.9–35,054.6)	464.2 (369.9–591.3)
Tanzania	Prevalence Rate: Cumulative change (%)	-2.5 (-3.0 to -2.1)	-15.3 (-19.1 to -11.5)	1.2 (-3.4 to 6.1)	-6.4 (-15.5 to 3.4)	-9.5 (-13.9 to -4.9)	-27.5 (-31.1 to -23.5)	31.0 (-7.8 to 77.4)	-33.3 (-66.3 to 11.3)	5.5 (-40.6 to 67.5)	-29.9 (-41.3 to -16.4)	12.0 (-16.8 to 40.4)
Tanzania	YLDs Number: 1990	1,135,551 (849,410–1,461,503)	272,821 (194,302–365,026)	106,917 (73,761–149,850)	51,686 (34,448–70,685)	24,608 (17,572–33,269)	53,079 (38,647–70,029)	8,271 (5,408–12,048)	22,266 (13,062–33,339)	12,733 (7,549–19,266)	69,304 (49,070–93,239)	3,448 (2,491–4,553)
Tanzania	YLDs Number: 2015	1,965,749 (1,478,580–2,536,207)	404,353 (289,415–536,467)	221,107 (152,170–310,553)	100,012 (66,189–138,929)	41,095 (29,129–55,432)	76,449 (54,978–101,932)	25,578 (16,820–37,370)	28,969 (14,577–46,131)	24,007 (14,429–36,119)	86,141 (62,469–114,300)	10,225 (7,374–13,477)
Tanzania	YLDs Number: Cumulative change (%)	73.2 (64.1–81.9)	48.6 (28.3–63.8)	106.7 (102.0–111.1)	93.8 (74.5–114.7)	67.2 (51.2–82.9)	44.1 (35.4–54.2)	214.6 (143.1–324.3)	35.6 (-31.7 to 127.0)	99.1 (13.5–223.6)	24.7 (9.7–36.5)	198.0 (152.1–246.3)
Tanzania	YLDs Rate: 1990	7,965.2 (5,959.3–10,254.3)	1,819.3 (1,298.1–2,443.7)	759.2 (523.0–1,061.7)	366.8 (245.9–499.9)	167.7 (120.0–225.1)	329.3 (239.6–433.9)	55.9 (36.5–81.5)	126.1 (74.0–188.8)	86.7 (51.6–130.4)	454.3 (323.3–607.0)	21.9 (15.8–29.0)
Tanzania	YLDs Rate: 2015	6,814.2 (5,126.2–8,768.7)	1,326.6 (949.3–1,761.2)	764.3 (524.3–1,077.5)	344.3 (227.9–478.9)	137.2 (96.7–185.1)	240.8 (173.3–319.4)	85.2 (55.7–124.1)	82.0 (41.3–130.6)	80.2 (48.4–121.1)	296.9 (215.4–395.4)	33.0 (23.7–43.5)
Tanzania	YLDs Rate: Cumulative change (%)	-14.4 (-18.4 to -10.6)	-27.0 (-35.2 to -20.7)	0.6 (-1.7 to 2.8)	-6.0 (-15.3 to 3.9)	-18.1 (-25.1 to -10.6)	-26.8 (-31.1 to -22.0)	55.0 (19.7–108.2)	-32.2 (-65.8 to 13.2)	-2.3 (-44.3 to 59.1)	-34.5 (-41.3 to -28.9)	51.4 (27.3–76.9)
Uganda	Prevalence Number: 1990	9,846,903 (9,812,164–9,882,160)	2,889,375 (2,681,314–3,022,525)	3,474,598 (3,271,160–3,707,828)	851,470 (703,375–1,010,343)	3,887,163 (3,768,234–4,003,247)	355,451 (326,715–385,611)	89,535 (68,162–119,214)	69,211 (45,605–99,140)	22,220 (14,904–32,141)	3,355,441 (3,137,624–3,577,543)	61,926 (48,160–79,800)
Uganda	Prevalence Number: 2015	21,926,816 (21,806,684–22,036,017)	4,696,731 (4,402,054–5,145,808)	8,088,022 (7,597,901–8,631,932)	1,787,376 (1,492,256–2,131,667)	8,292,248 (8,031,596–8,551,811)	418,335 (384,167–463,880)	251,289 (199,092–318,470)	168,442 (100,886–254,569)	51,583 (34,203–72,916)	9,447,652 (8,615,020–10,307,072)	127,768 (100,175–162,156)
Uganda	Prevalence Number: Cumulative change (%)	122.7 (121.3–124.0)	62.7 (52.6–74.0)	133.0 (115.3–152.8)	110.3 (91.6–129.0)	113.4 (104.3–122.8)	17.8 (9.9–26.7)	187.9 (97.6–292.8)	157.3 (32.7–316.6)	143.9 (36.9–287.7)	181.7 (160.5–203.5)	108.3 (65.0–157.1)
Uganda	Prevalence Rate: 1990	96,695.5 (96,375.3–97,009.2)	26,783.0 (25,169.5–27,831.2)	35,837.9 (33,890.7–38,042.3)	8,644.3 (7,179.8–10,235.4)	38,001.3 (36,828.1–39,152.7)	3,066.6 (2,843.0–3,305.0)	858.1 (652.3–1,149.3)	526.8 (348.7–753.2)	213.7 (142.9–308.4)	32,643.4 (30,469.0–34,901.2)	492.7 (375.2–642.0)
Uganda	Prevalence Rate: 2015	94,783.6 (94,300.9–95,217.9)	19,499.7 (18,310.5–21,199.9)	36,351.6 (34,211.3–38,613.6)	7,880.8 (6,593.3–9,376.5)	35,703.6 (34,585.5–36,813.6)	1,654.5 (1,527.4–1,817.9)	1,070.7 (845.6–1,359.7)	599.5 (359.6–905.6)	219.5 (145.5–311.2)	40,388.7 (37,130.3–43,793.7)	489.4 (380.9–630.9)
Uganda	Prevalence Rate: Cumulative change (%)	-2.0 (-2.5 to -1.4)	-27.2 (-31.6 to -22.6)	1.5 (-5.7 to 9.8)	-8.7 (-16.8 to -0.8)	-6.0 (-10.0 to -1.9)	-46.0 (-49.3 to -42.3)	28.1 (-12.2 to 76.1)	20.2 (-37.8 to 94.9)	7.9 (-39.8 to 72.0)	23.8 (14.6–33.3)	0.7 (-23.9 to 28.4)
Uganda	YLDs Number: 1990	756,162 (571,063–966,020)	118,832 (83,961–158,460)	74,208 (50,949–104,468)	38,400 (25,291–53,103)	16,667 (11,927–22,240)	57,567 (41,615–76,822)	7,544 (4,650–11,420)	8,459 (4,756–13,270)	8,856 (5,198–13,294)	54,504 (39,224–72,518)	2,602 (1,876–3,421)
Uganda	YLDs Number: 2015	1,445,962 (1,096,671–1,844,777)	175,808 (125,175–239,354)	170,332 (116,738–238,865)	80,992 (53,888–111,945)	31,643 (22,735–42,687)	68,248 (48,873–90,259)	22,656 (14,401–33,450)	20,950 (11,032–34,649)	18,195 (10,481–27,923)	127,133 (91,039–169,863)	7,537 (5,473–9,950)
Uganda	YLDs Number: Cumulative change (%)	91.3 (84.0–98.3)	48.0 (34.0–64.2)	129.5 (124.9–134.4)	111.2 (92.0–129.8)	90.0 (73.0–108.6)	18.6 (10.4–28.5)	205.2 (152.7–281.7)	161.5 (35.8–329.8)	116.2 (19.3–250.8)	133.4 (117.6–148.5)	191.3 (145.0–241.8)
Uganda	YLDs Rate: 1990	7,583.0 (5,738.2–9,692.5)	1,088.1 (773.3–1,453.2)	747.6 (511.7–1,056.9)	389.6 (256.9–536.2)	158.5 (114.2–211.4)	496.2 (360.1–666.1)	72.2 (44.1–110.0)	64.4 (36.4–101.2)	85.1 (49.8–127.8)	523.0 (376.7–695.2)	22.4 (16.1–29.5)
Uganda	YLDs Rate: 2015	6,416.4 (4,874.3–8,162.9)	727.2 (519.9–990.5)	750.0 (511.4–1,056.0)	357.0 (238.3–490.6)	134.3 (96.3–180.5)	269.7 (192.5–355.6)	96.3 (61.5–142.5)	74.6 (39.2–123.4)	77.3 (44.5–119.9)	540.7 (386.7–719.9)	30.7 (22.2–40.6)

© 2017 Global Burden of Disease Child and Adolescent Health Collaboration. JAMA Pediatrics.

eTable 3. Prevalent Cases, Rates (per 100,000 population), Years Lived with Disability (YLDs), and Cumulative Percent Change with 95% Uncertainty Interval (UI) for the Top 10 Global Causes of YLDs in Children and Adolescents in 195 Countries and Territories, Aged 0 to 19 Years, Both Sexes, 1990 and 2015. *Note: best viewed by enlarging in browser.*

Location	Measure	All causes	Iron-deficiency anemia	Skin and subcutaneous diseases	Asthma	Hemoglobinopathies and hemolytic anemias	Diarrheal diseases	Congenital anomalies	Protein-energy malnutrition	Epilepsy	Malaria	Neonatal preterm birth complications
Uganda	YLDs Rate: Cumulative change (%)	-15.4 (-18.5 to -12.4)	-33.2 (-39.0 to -26.8)	0.3 (-1.7 to 2.3)	-8.3 (-16.7 to -0.3)	-15.2 (-22.4 to -7.4)	-45.6 (-49.1 to -41.5)	35.6 (12.4–69.8)	22.2 (-36.5 to 100.5)	-4.3 (-47.2 to 54.5)	3.5 (-3.4 to 9.9)	37.9 (14.8–62.1)
Zambia	Prevalence Number: 1990	4,667,513 (4,653,221–4,680,056)	1,239,753 (1,117,157–1,311,346)	1,648,627 (1,552,311–1,758,662)	359,099 (297,298–423,669)	1,906,755 (1,833,473–1,981,017)	150,156 (139,618–158,806)	41,856 (32,216–56,686)	33,789 (21,783–49,358)	9,737 (6,539–13,675)	3,240,262 (2,811,868–3,671,471)	22,416 (17,188–28,892)
Zambia	Prevalence Number: 2015	8,889,513 (8,847,922–8,927,393)	2,247,159 (1,983,606–2,419,530)	3,251,601 (3,069,366–3,462,645)	631,823 (526,090–749,498)	3,340,260 (3,224,548–3,453,278)	172,456 (161,458–183,737)	102,395 (82,222–130,726)	75,256 (48,662–109,937)	18,561 (12,337–26,536)	2,325,585 (2,034,079–2,699,839)	44,582 (35,178–56,291)
Zambia	Prevalence Number: Cumulative change (%)	90.5 (89.5–91.5)	81.8 (57.0–110.6)	97.5 (82.0–114.3)	76.1 (63.0–89.9)	75.3 (67.0–83.4)	14.9 (9.5–20.9)	151.2 (70.5–243.3)	136.0 (25.3–288.3)	99.5 (16.2–214.4)	-28.1 (-36.6 to -18.4)	101.4 (56.6–153.8)
Zambia	Prevalence Rate: 1990	98,493.0 (98,230.1–98,751.6)	23,829.7 (21,563.3–25,194.1)	36,288.3 (34,293.9–38,589.0)	7,774.1 (6,487.0–9,145.3)	40,128.3 (38,577.0–41,705.2)	2,838.6 (2,653.8–2,993.0)	865.1 (661.6–1,178.0)	580.6 (376.0–845.6)	199.2 (133.8–279.1)	66,213.8 (57,667.9–75,169.4)	401.9 (304.3–532.1)
Zambia	Prevalence Rate: 2015	96,519.2 (96,117.0–96,893.0)	22,404.7 (19,973.9–24,005.6)	36,639.2 (34,611.3–38,874.6)	7,014.7 (5,828.0–8,315.3)	36,117.2 (34,849.8–37,325.7)	1,744.7 (1,640.8–1,853.2)	1,095.9 (877.4–1,403.0)	686.2 (444.2–1,003.2)	199.4 (133.2–287.1)	25,389.5 (22,265.1–29,321.8)	434.6 (338.8–551.3)
Zambia	Prevalence Rate: Cumulative change (%)	-2.0 (-2.5 to -1.5)	-5.7 (-18.0 to 8.3)	1.1 (-6.4 to 9.3)	-9.7 (-16.2 to -2.7)	-10.0 (-14.2 to -5.8)	-38.5 (-41.2 to -35.5)	30.2 (-12.1 to 79.0)	25.2 (-33.5 to 105.5)	4.7 (-38.7 to 64.7)	-61.5 (-66.4 to -56.2)	10.0 (-18.3 to 42.5)
Zambia	YLDs Number: 1990	345,662 (261,525–441,508)	56,242 (40,173–75,453)	34,657 (23,640–48,953)	16,216 (10,653–22,799)	9,582 (6,835–12,705)	24,217 (17,568–31,736)	3,585 (2,264–5,409)	4,089 (2,243–6,546)	3,830 (2,257–5,726)	23,671 (17,238–31,320)	933 (674–1,230)
Zambia	YLDs Number: 2015	621,652 (465,699–798,296)	90,134 (62,021–124,700)	68,195 (46,530–96,278)	28,562 (19,085–38,944)	17,264 (11,500–23,830)	27,999 (20,281–37,202)	9,967 (6,391–14,834)	9,291 (5,297–14,961)	6,058 (3,515–9,371)	18,888 (13,620–25,373)	2,441 (1,761–3,239)
Zambia	YLDs Number: Cumulative change (%)	79.9 (70.6–90.3)	60.9 (27.2–95.4)	96.8 (92.5–101.0)	76.3 (62.7–90.7)	80.3 (33.4–107.6)	15.7 (9.3–22.1)	182.5 (129.5–267.4)	141.1 (25.6–296.3)	65.8 (-5.4 to 163.9)	-20.1 (-27.8 to -11.4)	163.1 (119.3–207.5)
Zambia	YLDs Rate: 1990	7,315.6 (5,530.7–9,354.2)	1,050.1 (746.4–1,409.2)	749.0 (509.7–1,061.7)	351.0 (231.4–491.5)	193.8 (138.4–257.9)	457.8 (332.7–600.5)	73.9 (46.4–111.8)	70.3 (38.5–112.5)	78.4 (46.4–117.0)	482.8 (352.1–637.3)	17.7 (12.7–23.3)
Zambia	YLDs Rate: 2015	6,856.6 (5,154.8–8,820.5)	889.9 (615.5–1,231.0)	754.2 (513.7–1,068.2)	317.0 (212.6–432.3)	181.2 (121.4–250.7)	283.1 (204.9–375.0)	106.2 (68.0–157.8)	84.7 (48.3–136.5)	65.0 (37.8–100.8)	202.4 (146.3–271.4)	24.9 (18.0–33.0)
Zambia	YLDs Rate: Cumulative change (%)	-6.3 (-10.7 to -1.3)	-14.9 (-31.6 to 2.7)	0.7 (-1.5 to 2.9)	-9.6 (-16.5 to -2.4)	-6.4 (-30.2 to 6.8)	-38.1 (-41.4 to -34.8)	46.3 (18.7–90.4)	27.9 (-33.3 to 110.0)	-13.0 (-50.3 to 37.8)	-58.0 (-61.7 to -53.9)	42.3 (17.7–68.0)
Central Sub-Saharan Africa	Prevalence Number: 1990	29,113,448 (29,052,544–29,168,112)	9,430,315 (8,989,164–9,830,132)	10,880,713 (10,541,335–11,226,730)	3,035,425 (2,514,485–3,629,211)	11,842,568 (11,518,290–12,159,278)	714,010 (655,220–774,676)	296,929 (241,857–369,207)	463,969 (378,911–561,296)	85,496 (65,752–109,388)	10,580,880 (9,450,181–11,890,064)	161,168 (124,067–208,072)
Central Sub-Saharan Africa	Prevalence Number: 2015	62,933,394 (62,781,329–63,074,476)	17,537,885 (16,454,143–18,635,544)	23,972,738 (23,248,753–24,736,181)	7,030,042 (5,704,097–8,403,058)	23,492,513 (22,904,968–24,050,478)	1,342,987 (1,253,221–1,440,574)	730,306 (610,501–870,868)	708,630 (583,534–854,587)	182,575 (136,836–237,735)	22,506,678 (18,651,657–26,651,837)	387,546 (297,821–494,000)
Central Sub-Saharan Africa	Prevalence Number: Cumulative change (%)	116.2 (115.5–116.8)	86.2 (71.5–102.6)	120.4 (111.3–128.8)	131.8 (109.6–156.0)	98.4 (92.5–104.8)	88.3 (77.1–100.9)	149.2 (90.2–210.6)	55.0 (17.5–100.7)	118.1 (50.0–204.6)	112.4 (91.8–130.0)	142.2 (97.7–187.4)
Central Sub-Saharan Africa	Prevalence Rate: 1990	97,976.9 (97,804.0–98,137.9)	30,885.9 (29,817.0–31,917.5)	38,311.0 (37,198.9–39,475.3)	10,558.3 (8,815.3–12,546.4)	39,708.2 (38,621.9–40,755.5)	2,018.5 (1,865.5–2,177.8)	974.8 (788.5–1,218.0)	1,187.5 (970.2–1,437.0)	288.5 (221.3–367.6)	36,026.9 (32,049.6–40,736.4)	446.0 (341.4–577.4)
Central Sub-Saharan Africa	Prevalence Rate: 2015	97,393.3 (97,179.7–97,595.1)	26,367.6 (24,939.2–27,764.5)	38,407.3 (37,280.0–39,590.9)	11,074.4 (9,072.2–13,294.1)	36,235.3 (35,333.5–37,094.3)	1,851.4 (1,735.0–1,977.2)	1,113.9 (930.2–1,332.1)	895.8 (737.9–1,079.8)	284.3 (213.2–369.4)	35,096.8 (29,140.3–41,710.2)	525.4 (401.5–679.6)
Central Sub-Saharan Africa	Prevalence Rate: Cumulative change (%)	-0.6 (-0.8 to -0.3)	-14.6 (-20.4 to -8.4)	0.3 (-3.6 to 3.9)	5.0 (-5.1 to 15.8)	-8.7 (-11.5 to -5.8)	-8.2 (-13.4 to -2.6)	15.8 (-12.1 to 44.9)	-23.4 (-42.0 to -0.9)	0.6 (-30.7 to 40.7)	-2.7 (-12.2 to 5.4)	19.0 (-6.3 to 45.0)
Central Sub-Saharan Africa	YLDs Number: 1990	2,669,283 (1,977,397–3,518,600)	415,887 (299,932–561,847)	276,001 (194,322–376,250)	136,070 (89,815–189,472)	51,715 (37,093–69,058)	115,245 (83,077–152,305)	24,326 (15,687–37,067)	56,831 (37,127–80,980)	34,841 (23,345–48,462)	157,848 (113,126–209,354)	6,578 (4,899–8,571)
Central Sub-Saharan Africa	YLDs Number: 2015	5,823,721 (4,078,777–8,112,207)	755,235 (537,445–1,012,113)	605,429 (426,834–826,501)	316,530 (207,247–443,537)	102,992 (73,888–137,092)	217,210 (158,030–288,430)	64,833 (42,882–95,306)	87,093 (57,948–121,949)	64,486 (41,261–92,983)	322,762 (233,879–427,233)	17,470 (12,683–22,604)
Central Sub-Saharan Africa	YLDs Number: Cumulative change (%)	116.7 (93.4–156.2)	81.9 (59.3–113.5)	119.3 (116.4–122.2)	132.8 (110.4–156.5)	99.3 (80.1–128.2)	88.7 (77.0–101.4)	169.4 (134.6–216.2)	55.8 (18.0–102.4)	88.9 (27.7–169.9)	104.6 (90.6–126.2)	166.2 (133.7–199.3)
Central Sub-Saharan Africa	YLDs Rate: 1990	9,267.1 (6,854.3–12,237.8)	1,338.5 (965.0–1,804.6)	940.9 (661.0–1,293.5)	472.9 (312.7–651.7)	169.4 (122.1–225.7)	325.4 (235.1–429.6)	79.5 (50.8–121.9)	145.5 (95.0–207.5)	117.3 (78.9–163.4)	528.4 (381.5–699.8)	19.0 (14.0–24.8)
Central Sub-Saharan Africa	YLDs Rate: 2015	9,255.0 (6,455.5–12,922.3)	1,114.2 (796.8–1,488.4)	944.0 (663.4–1,291.3)	498.4 (328.1–691.4)	154.4 (110.4–206.4)	299.2 (217.9–397.1)	98.4 (65.0–145.3)	110.1 (73.2–154.1)	100.3 (63.6–144.6)	493.9 (359.0–653.6)	24.9 (18.0–32.2)
Central Sub-Saharan Africa	YLDs Rate: Cumulative change (%)	-0.8 (-11.5 to 17.8)	-16.7 (-25.7 to -4.2)	0.3 (-1.0 to 1.6)	5.4 (-4.7 to 16.3)	-8.8 (-16.6 to 2.7)	-8.0 (-13.3 to -2.2)	25.3 (9.3–46.7)	-23.1 (-41.7 to 0.0)	-12.8 (-40.9 to 24.3)	-6.5 (-12.0 to 2.5)	31.3 (13.9–49.2)
Angola	Prevalence Number: 1990	6,355,813 (6,324,742–6,386,460)	1,389,308 (1,302,617–1,438,324)	2,418,656 (2,319,120–2,519,338)	820,426 (679,182–982,068)	2,403,186 (2,309,481–2,503,558)	181,187 (164,504–200,218)	63,169 (47,384–83,535)	81,032 (58,307–108,765)	22,388 (14,952–31,164)	2,516,258 (2,049,718–3,009,952)	31,838 (23,814–41,210)
Angola	Prevalence Number: 2015	14,110,686 (14,048,311–14,172,953)	1,976,035 (1,870,838–2,053,962)	5,555,034 (5,326,480–5,776,771)	1,746,236 (1,429,384–2,095,879)	4,837,537 (4,644,412–5,020,555)	274,825 (256,207–297,236)	165,414 (130,786–205,399)	123,561 (87,655–163,503)	45,397 (29,543–64,843)	5,174,513 (4,210,786–6,223,888)	68,697 (53,223–88,234)
Angola	Prevalence Number: Cumulative change (%)	122.0 (120.7–123.4)	42.4 (31.6–53.4)	129.8 (117.1–142.2)	113.0 (99.2–127.9)	101.4 (91.4–112.0)	51.9 (43.0–61.1)	169.9 (81.8–280.6)	57.7 (-6.3 to 134.2)	112.2 (23.4–244.5)	106.4 (78.7–134.9)	118.7 (68.9–172.6)
Angola	Prevalence Rate: 1990	97,115.3 (96,737.1–97,499.9)	23,146.5 (21,924.9–23,834.5)	38,722.7 (37,203.6–40,222.6)	12,978.7 (10,791.3–15,455.8)	36,518.1 (35,114.9–38,026.4)	2,260.3 (2,067.5–2,479.8)	932.7 (695.4–1,240.7)	908.1 (654.3–1,220.6)	342.0 (228.4–475.9)	38,734.0 (31,722.3–46,404.9)	391.4 (283.8–532.2)
Angola	Prevalence Rate: 2015	95,908.4 (95,548.0–96,271.0)	14,455.9 (13,662.8–15,072.9)	39,166.7 (37,643.4–40,634.4)	12,106.4 (9,951.0–14,466.4)	32,655.1 (31,342.6–33,876.5)	1,637.9 (1,538.7–1,762.2)	1,100.7 (867.5–1,370.6)	657.2 (466.4–869.6)	307.6 (200.6–439.7)	35,968.3 (29,066.2–43,342.5)	404.0 (310.2–525.7)
Angola	Prevalence Rate: Cumulative change (%)	-1.2 (-1.7 to -0.7)	-37.5 (-41.9 to -33.5)	1.2 (-4.0 to 6.3)	-6.7 (-12.5 to -0.4)	-10.5 (-14.9 to -5.8)	-27.4 (-31.4 to -23.4)	21.8 (-18.6 to 72.3)	-25.1 (-55.6 to 10.6)	-5.9 (-45.4 to 52.6)	-6.9 (-18.3 to 5.5)	5.2 (-23.0 to 36.5)
Angola	YLDs Number: 1990	527,579 (388,077–696,640)	59,425 (42,887–79,022)	62,675 (44,245–85,736)	36,892 (24,433–50,591)	8,254 (5,625–11,288)	29,588 (21,283–39,325)	5,139 (3,213–7,869)	9,996 (5,939–15,400)	9,219 (5,702–13,628)	28,118 (19,655–37,920)	1,217 (905–1,610)
Angola	YLDs Number: 2015	1,108,937 (787,023–1,588,231)	81,223 (58,232–108,150)	143,085 (100,967–196,001)	78,933 (52,601–109,641)	16,101 (10,664–22,130)	45,016 (32,261–59,617)	14,939 (9,501–22,407)	15,425 (9,353–22,979)	15,575 (8,968–23,743)	61,323 (44,352–82,263)	3,075 (2,204–4,027)
Angola	YLDs Number: Cumulative change (%)	109.7 (86.8–152.3)	36.8 (27.8–48.1)	128.2 (124.0–132.7)	114.1 (100.0–129.7)	96.6 (43.3–149.1)	52.4 (42.9–62.5)	194.8 (145.7–270.0)	60.0 (-5.1 to 136.7)	76.7 (0.0–190.6)	119.5 (91.3–159.4)	153.8 (110.4–200.2)
Angola	YLDs Rate: 1990	8,469.4 (6,249.2–11,220.3)	982.5 (713.9–1,311.4)	967.8 (679.9–1,332.7)	582.9 (387.9–796.3)	126.2 (87.7–170.9)	368.3 (267.7–491.0)	75.4 (47.0–116.4)	112.0 (66.6–172.8)	140.4 (86.8–206.1)	430.4 (306.7–576.9)	15.4 (11.3–20.6)
Angola	YLDs Rate: 2015	7,879.1 (5,593.5–11,321.2)	583.7 (418.4–778.1)	976.9 (688.6–1,344.3)	546.8 (364.6–754.1)	108.0 (71.9–148.7)	267.7 (192.8–354.3)	98.7 (62.6–148.2)	82.0 (49.8–122.3)	105.3 (59.9–160.1)	421.3 (306.2–559.1)	18.8 (13.5–24.9)
Angola	YLDs Rate: Cumulative change (%)	-7.2 (-17.5 to 12.2)	-40.6 (-44.4 to -36.4)	0.9 (-0.9 to 2.8)	-6.2 (-11.9 to 0.2)	-14.0 (-37.1 to 3.2)	-27.2 (-31.6 to -22.8)	33.0 (11.4–66.9)	-24.1 (-54.9 to 12.1)	-21.6 (-55.6 to 27.8)	-1.7 (-13.1 to 12.4)	23.4 (0.2–47.6)
Central African Republic	Prevalence Number: 1990	1,525,870 (1,522,266–1,529,242)	555,382 (536,253–572,927)	576,075 (552,953–599,054)	168,000 (138,001–200,908)	619,181 (597,208–642,556)	43,020 (39,613–46,694)	14,284 (10,650–19,297)	20,768 (15,477–27,016)	4,762 (3,208–6,727)	601,916 (517,116–708,050)	8,523 (6,270–11,449)
Central African Republic	Prevalence Number: 2015	2,388,173 (2,381,199–2,394,628)	817,577 (785,793–843,899)	927,629 (887,935–963,252)	262,948 (216,549–315,141)	887,388 (855,705–918,960)	43,727 (40,538–47,738)	23,935 (18,267–30,824)	27,212 (19,898–36,330)	6,886 (4,400–9,780)	1,018,666 (802,387–1,231,158)	14,180 (10,308–19,086)
Central African Republic	Prevalence Number: Cumulative change (%)	56.5 (55.9–57.0)	47.3 (40.2–54.6)	61.1 (53.0–69.6)	56.8 (42.6–71.3)	43.4 (36.7–50.1)	1.7 (-3.1 to 8.1)	72.3 (12.3–144.0)	34.9 (-11.8 to 103.4)	51.2 (-14.4 to 135.6)	69.0 (48.4–88.3)	68.8 (25.2–123.7)
Central African Republic	Prevalence Rate: 1990	97,944.4 (97,727.2–98,154.1)	33,602.0 (32,598.2–34,531.8)	38,289.4 (36,883.9–39,674.0)	11,021.5 (9,108.3–13,137.9)	39,636.5 (38,236.2–41,117.2)	2,438.8 (2,253.1–2,634.3)	900.5 (668.2–1,221.1)	1,094.4 (816.1–1,423.3)	307.0 (207.7–435.4)	38,974.7 (33,493.2–45,680.2)	472.6 (342.4–657.1)
Central African Republic	Prevalence Rate: 2015	97,512.3 (97,238.2–97,769.3)	32,380.9 (31,187.1–33,377.0)	38,440.3 (36,842.3–39,883.6)	10,896.0 (8,987.0–13,031.4)	36,181.8 (34,886.2–37,463.4)	1,694.4 (1,574.7–1,844.3)	969.5 (739.1–1,250.2)	1,006.8 (736.9–1,344.1)	282.3 (180.4–401.4)	41,746.6 (32,924.6–50,602.2)	535.0 (386.3–726.5)
Central African Republic	Prevalence Rate: Cumulative change (%)	-0.4 (-0.8 to -0.1)	-3.6 (-7.8 to 0.7)	0.4 (-4.4 to 5.5)	-1.0 (-9.6 to 7.8)	-8.7 (-13.0 to -4.4)	-30.5 (-33.7 to -26.4)	10.8 (-28.1 to 58.0)	-5.3 (-38.0 to 42.8)	-3.8 (-45.7 to 50.0)	7.0 (-6.6 to 19.2)	15.5 (-18.4 to 57.3)
Central African Republic	YLDs Number: 1990	141,560 (106,385–183,405)	27,623 (19,890–37,118)	14,779 (10,394–20,247)	7,521 (4,938–10,393)	3,008 (2,164–4,034)	6,871 (5,029–9,149)	1,165 (729–1,804)	2,512 (1,554–3,737)	1,909 (1,154–2,857)	7,966 (5,776–10,487)	322 (233–430)
Central African Republic	YLDs Number: 2015	207,920 (156,281–268,204)	39,809 (28,712–53,137)	23,408 (16,541–32,110)	11,811 (7,729–16,200)	4,225 (3,020–5,665)	7,006 (5,120–9,211)	2,036 (1,296–3,036)	3,305 (2,029–4,908)	2,752 (1,619–4,164)	11,808 (8,589–15,469)	552 (397–729)
Central African Republic	YLDs Number: Cumulative change (%)	46.9 (39.7–54.0)	44.5 (26.4–58.7)	58.4 (55.0–61.4)	57.3 (43.0–72.3)	40.6 (26.1–56.8)	2.0 (-3.7 to 9.2)	76.0 (57.1–104.7)	35.5 (-11.5 to 103.1)	50.8 (-17.6 to 137.6)	48.4 (39.1–57.6)	72.8 (41.6–108.8)
Central African Republic	YLDs Rate: 1990	9,135.6 (6,865.3–11,796.2)	1,619.5 (1,172.3–2,171.5)	958.4 (671.2–1,322.0)	493.3 (326.2–679.6)	183.9 (132.5–246.2)	389.6 (285.8–519.3)	73.4 (45.5–114.2)	132.4 (81.9–197.0)	123.1 (74.5–185.3)	503.3 (365.4–663.1)	18.3 (13.1–24.7)
Central African Republic	YLDs Rate: 2015	8,490.2 (6,379.6–10,950.8)	1,552.9 (1,122.4–2,072.8)	961.1 (677.6–1,323.2)	489.5 (320.1–671.0)	168.4 (120.9–226.0)	271.5 (198.4–355.9)	82.4 (52.4–122.9)	122.3 (75.1–181.6)	112.8 (66.6–171.2)	479.6 (348.9–628.1)	21.4 (15.3–28.3)
Central African Republic	YLDs Rate: Cumulative change (%)	-7.0 (-11.5 to -2.5)	-3.9 (-14.8 to 4.8)	0.3 (-1.6 to 2.1)	-0.6 (-9.3 to 8.6)	-8.4 (-17.0 to 1.3)	-30.3 (-34.0 to -25.6)	13.1 (1.0–31.7)	-4.9 (-37.8 to 42.2)	-4.1 (-47.1 to 51.7)	-4.6 (-10.3 to 1.1)	17.5 (-5.0 to 43.4)
Congo	Prevalence Number: 1990	1,293,350 (1,290,411–1,296,264)	429,400 (409,218–457,585)	503,735 (484,162–521,757)	123,796 (102,226–147,501)	477,868 (457,475–497,704)	26,133 (23,672–29,148)	16,093 (12,916–20,414)	15,900 (11,234–22,443)	3,965 (2,540–5,700)	739,252 (636,210–852,565)	6,935 (5,443–8,801)

© 2017 Global Burden of Disease Child and Adolescent Health Collaboration. JAMA Pediatrics.

eTable 3. Prevalent Cases, Rates (per 100,000 population), Years Lived with Disability (YLDs), and Cumulative Percent Change with 95% Uncertainty Interval (UI) for the Top 10 Global Causes of YLDs in Children and Adolescents in 195 Countries and Territories, Aged 0 to 19 Years, Both Sexes, 1990 and 2015. *Note: best viewed by enlarging in browser.*

Location	Measure	All causes	Iron-deficiency anemia	Skin and subcutaneous diseases	Asthma	Hemoglobinopathies and hemolytic anemias	Diarrheal diseases	Congenital anomalies	Protein-energy malnutrition	Epilepsy	Malaria	Neonatal preterm birth complications
Congo	Prevalence Number: 2015	2,394,682 (2,387,792–2,401,438)	792,966 (771,929–818,137)	939,295 (905,688–975,878)	226,186 (186,590–271,102)	794,388 (761,735–826,776)	39,322 (36,421–42,727)	32,649 (26,737–40,984)	21,079 (14,935–27,705)	7,115 (4,825–10,081)	904,310 (749,747–1,101,372)	13,724 (10,827–17,168)
Congo	Prevalence Number: Cumulative change (%)	85.2 (84.5–85.8)	84.8 (75.5–93.1)	86.6 (77.3–96.1)	83.0 (69.7–97.5)	66.3 (57.4–75.3)	50.7 (40.9–60.1)	106.8 (50.4–179.9)	38.2 (-16.8 to 109.1)	89.1 (6.4–207.0)	22.4 (8.2–37.2)	100.0 (58.7–150.6)
Congo	Prevalence Rate: 1990	98,813.3 (98,602.7–99,020.3)	31,724.4 (30,436.1–33,511.5)	39,543.4 (38,093.1–40,895.0)	9,647.4 (7,987.1–11,483.8)	36,445.8 (34,885.3–37,951.6)	1,806.8 (1,645.3–2,005.6)	1,218.1 (978.3–1,548.7)	1,037.5 (732.9–1,464.4)	304.0 (195.4–433.7)	56,593.8 (48,944.2–65,034.6)	482.8 (374.5–620.2)
Congo	Prevalence Rate: 2015	98,198.2 (97,944.3–98,450.7)	31,165.3 (30,412.7–31,998.5)	39,769.3 (38,435.9–41,222.9)	9,425.0 (7,805.5–11,205.9)	32,485.1 (31,168.5–33,826.3)	1,464.2 (1,361.4–1,583.3)	1,325.1 (1,083.2–1,663.3)	723.3 (512.6–950.5)	292.6 (199.2–415.3)	37,702.3 (31,365.6–45,642.2)	513.3 (401.4–646.2)
Congo	Prevalence Rate: Cumulative change (%)	-0.6 (-0.9 to -0.3)	-1.7 (-6.1 to 2.2)	0.6 (-4.2 to 5.5)	-2.2 (-9.2 to 5.2)	-10.8 (-15.7 to -6.0)	-18.8 (-23.8 to -13.9)	10.9 (-19.5 to 50.4)	-27.3 (-56.2 to 9.9)	1.5 (-43.0 to 64.6)	-33.4 (-41.3 to -25.4)	7.7 (-16.3 to 37.6)
Congo	YLDs Number: 1990	105,592 (79,737–135,411)	18,789 (13,460–24,998)	13,141 (9,267–17,755)	5,561 (3,638–7,717)	1,756 (1,253–2,327)	4,209 (3,034–5,660)	1,368 (888–2,025)	1,950 (1,175–2,952)	1,565 (934–2,434)	5,697 (4,136–7,491)	427 (314–552)
Congo	YLDs Number: 2015	192,876 (141,000–257,327)	32,746 (23,640–43,263)	24,686 (17,375–33,311)	10,184 (6,634–14,049)	3,048 (2,152–4,071)	6,367 (4,573–8,448)	3,138 (1,927–4,756)	2,595 (1,542–3,783)	2,178 (1,276–3,272)	6,856 (5,007–9,055)	909 (667–1,192)
Congo	YLDs Number: Cumulative change (%)	82.3 (69.3–107.2)	74.7 (57.2–88.3)	87.8 (84.5–91.3)	83.4 (69.8–98.1)	73.7 (51.4–89.9)	51.5 (40.2–62.2)	132.6 (71.4–232.1)	39.0 (-16.7 to 112.4)	46.7 (-18.5 to 145.1)	20.5 (12.8–30.0)	113.2 (84.9–144.3)
Congo	YLDs Rate: 1990	8,159.1 (6,175.9–10,453.2)	1,374.5 (982.9–1,822.1)	1,011.1 (711.4–1,372.7)	433.3 (282.9–593.6)	130.6 (93.8–173.0)	290.9 (210.5–391.1)	103.2 (67.0–152.9)	127.2 (76.8–192.6)	119.9 (71.4–185.4)	431.3 (313.6–567.0)	31.0 (22.8–40.1)
Congo	YLDs Rate: 2015	8,067.6 (5,909.8–10,797.3)	1,280.0 (925.7–1,696.2)	1,018.6 (715.3–1,393.5)	424.2 (277.4–580.0)	121.0 (85.7–161.6)	236.9 (170.3–315.0)	126.9 (78.4–191.5)	89.0 (53.0–129.8)	89.5 (53.0–134.2)	280.1 (205.2–369.3)	35.6 (26.1–46.6)
Congo	YLDs Rate: Cumulative change (%)	-1.3 (-8.3 to 12.5)	-6.7 (-15.0 to -0.3)	0.7 (-1.1 to 2.5)	-1.9 (-9.0 to 5.8)	-7.3 (-19.2 to 1.2)	-18.4 (-24.3 to -12.8)	24.8 (-7.6 to 76.7)	-26.9 (-56.2 to 11.6)	-21.3 (-56.3 to 31.4)	-35.0 (-38.8 to -30.4)	15.2 (-0.6 to 32.2)
Democratic Republic of the Congo	Prevalence Number: 1990	19,287,802 (19,241,817–19,333,241)	6,843,379 (6,419,138–7,224,370)	7,128,142 (6,815,663–7,449,004)	1,843,177 (1,515,460–2,216,624)	8,092,923 (7,816,169–8,368,162)	449,741 (413,069–488,979)	195,614 (149,458–262,333)	340,720 (259,024–437,299)	52,225 (34,705–73,025)	6,495,395 (5,874,777–7,203,438)	110,543 (82,972–145,052)
Democratic Republic of the Congo	Prevalence Number: 2015	42,830,338 (42,699,692–42,952,068)	13,606,341 (12,527,813–14,692,969)	16,060,335 (15,391,544–16,742,973)	4,640,649 (3,682,456–5,628,374)	16,552,562 (16,010,779–17,050,278)	968,153 (893,930–1,044,893)	492,554 (383,516–621,996)	530,919 (411,293–679,767)	119,493 (80,780–169,102)	14,904,985 (12,247,484–17,717,330)	285,588 (212,447–377,574)
Democratic Republic of the Congo	Prevalence Number: Cumulative change (%)	122.1 (121.2–122.8)	99.2 (77.7–122.5)	125.4 (112.2–137.8)	152.3 (117.6–191.7)	104.6 (96.1–113.1)	115.6 (99.7–133.4)	158.3 (74.5–253.3)	60.1 (9.6–128.5)	140.4 (32.9–285.2)	129.3 (97.4–158.2)	161.7 (101.3–230.1)
Democratic Republic of the Congo	Prevalence Rate: 1990	98,213.4 (98,005.1–98,415.7)	33,356.5 (31,822.0–34,855.2)	38,030.4 (36,534.1–39,656.8)	9,710.9 (8,023.5–11,562.0)	41,075.0 (39,662.6–42,473.6)	1,925.7 (1,779.9–2,082.4)	972.1 (736.7–1,305.9)	1,317.8 (1,003.1–1,690.7)	266.8 (178.4–375.0)	33,525.2 (30,033.9–37,594.2)	460.5 (338.6–619.3)
Democratic Republic of the Congo	Prevalence Rate: 2015	97,844.1 (97,565.5–98,098.9)	29,900.9 (27,784.0–31,923.9)	38,011.3 (36,522.8–39,532.3)	10,783.9 (8,571.7–13,014.2)	37,717.2 (36,488.6–38,839.4)	1,971.3 (1,830.1–2,117.3)	1,110.2 (862.6–1,404.4)	996.2 (772.2–1,275.1)	275.6 (186.2–391.6)	34,168.1 (28,076.9–40,684.5)	570.5 (422.4–766.6)
Democratic Republic of the Congo	Prevalence Rate: Cumulative change (%)	-0.4 (-0.7 to -0.1)	-10.3 (-18.5 to -1.5)	0.0 (-5.5 to 5.3)	11.3 (-4.0 to 28.2)	-8.1 (-11.9 to -4.3)	2.5 (-4.7 to 10.4)	17.3 (-21.3 to 60.9)	-22.3 (-46.7 to 10.8)	8.5 (-39.8 to 74.7)	1.9 (-12.3 to 14.6)	26.3 (-8.4 to 66.2)
Democratic Republic of the Congo	YLDs Number: 1990	1,842,469 (1,355,612–2,441,905)	301,210 (213,521–405,953)	179,170 (126,844–244,536)	82,500 (54,203–115,051)	37,829 (27,344–50,749)	72,320 (52,022–95,898)	15,998 (10,106–24,463)	41,690 (26,314–61,783)	21,281 (12,555–31,587)	113,292 (81,442–150,832)	4,377 (3,193–5,793)
Democratic Republic of the Congo	YLDs Number: 2015	4,216,098 (2,887,013–5,917,447)	587,729 (414,185–799,657)	402,413 (283,375–551,272)	208,664 (133,866–294,911)	78,091 (55,994–104,790)	156,070 (112,597–208,072)	43,164 (27,806–64,463)	65,043 (41,607–92,788)	42,790 (24,774–63,961)	236,968 (170,077–313,806)	12,520 (9,016–16,497)
Democratic Republic of the Congo	YLDs Number: Cumulative change (%)	127.1 (95.8–175.4)	95.9 (63.5–139.5)	124.6 (120.4–128.8)	153.4 (118.2–192.9)	106.7 (83.5–146.7)	116.2 (98.3–135.0)	173.2 (129.3–237.1)	60.5 (10.3–128.1)	111.3 (16.6–248.6)	109.4 (91.8–139.7)	187.6 (139.5–239.2)
Democratic Republic of the Congo	YLDs Rate: 1990	9,671.4 (7,116.3–12,867.9)	1,442.9 (1,038.2–1,946.1)	925.2 (651.0–1,266.9)	434.3 (284.0–601.2)	187.1 (135.5–249.6)	309.4 (223.5–409.6)	79.1 (49.7–122.1)	161.3 (101.9–239.3)	108.5 (63.7–162.1)	573.7 (414.0–762.1)	19.0 (13.8–25.0)
Democratic Republic of the Congo	YLDs Rate: 2015	9,873.5 (6,730.2–13,896.4)	1,267.4 (899.9–1,710.7)	927.1 (651.2–1,272.2)	484.7 (314.0–678.1)	172.5 (124.3–232.8)	317.6 (229.9–423.1)	96.9 (62.5–144.9)	122.1 (78.0–174.0)	98.6 (57.4–147.0)	532.8 (385.0–706.7)	26.3 (18.8–34.7)
Democratic Republic of the Congo	YLDs Rate: Cumulative change (%)	1.3 (-12.6 to 23.5)	-12.0 (-24.7 to 5.4)	0.2 (-1.6 to 2.1)	11.8 (-3.4 to 29.2)	-7.8 (-16.9 to 7.7)	2.8 (-5.3 to 11.2)	24.1 (4.8–53.1)	-22.1 (-46.4 to 10.7)	-4.5 (-47.6 to 57.7)	-7.1 (-13.6 to 4.7)	39.4 (14.8–65.9)
Equatorial Guinea	Prevalence Number: 1990	171,752 (171,342–172,165)	51,979 (47,517–55,874)	62,813 (60,222–65,480)	17,110 (14,122–20,567)	72,223 (69,436–75,041)	5,011 (4,520–5,581)	1,692 (1,287–2,253)	1,967 (1,387–2,610)	516 (334–727)	73,501 (68,383–79,303)	1,092 (814–1,410)
Equatorial Guinea	Prevalence Number: 2015	406,547 (405,349–407,762)	83,401 (73,598–93,552)	160,711 (154,385–166,825)	54,310 (44,904–64,555)	155,407 (149,276–161,031)	6,165 (5,672–6,685)	4,441 (3,477–5,844)	1,890 (1,306–2,552)	1,195 (780–1,660)	191,214 (150,795–228,050)	1,562 (1,181–2,078)
Equatorial Guinea	Prevalence Number: Cumulative change (%)	136.7 (135.8–137.6)	60.7 (45.2–80.9)	156.0 (142.9–170.5)	218.1 (189.3–246.5)	115.3 (104.4–126.0)	23.3 (14.5–31.2)	169.4 (84.5–273.1)	-0.5 (-39.8 to 51.0)	142.3 (39.7–282.9)	159.9 (114.4–202.6)	44.8 (13.0–87.6)
Equatorial Guinea	Prevalence Rate: 1990	98,412.0 (98,247.3–98,588.5)	27,314.6 (25,329.7–29,149.3)	38,514.6 (37,070.3–40,037.9)	10,586.8 (8,766.5–12,631.3)	41,189.3 (39,593.9–42,769.5)	2,167.5 (1,976.5–2,384.7)	934.9 (701.9–1,261.5)	743.8 (527.6–986.5)	299.7 (194.8–422.5)	43,880.6 (40,893.9–47,333.6)	463.2 (335.8–612.0)
Equatorial Guinea	Prevalence Rate: 2015	98,024.1 (97,756.3–98,289.6)	19,212.1 (17,166.6–21,358.4)	39,870.8 (38,325.2–41,317.6)	13,279.6 (11,030.0–15,745.7)	37,384.4 (35,903.0–38,738.5)	1,365.9 (1,262.2–1,475.5)	1,058.9 (827.5–1,398.4)	387.8 (268.3–523.5)	287.5 (187.5–399.7)	46,271.1 (36,632.5–55,264.6)	343.6 (258.0–465.4)
Equatorial Guinea	Prevalence Rate: Cumulative change (%)	-0.4 (-0.7 to -0.1)	-29.6 (-35.7 to -21.5)	3.6 (-1.5 to 8.9)	25.6 (15.4–35.8)	-9.2 (-13.7 to -4.7)	-36.9 (-40.8 to -33.2)	16.5 (-20.8 to 62.5)	-46.0 (-67.2 to -18.4)	0.3 (-41.8 to 57.6)	5.3 (-13.9 to 23.2)	-24.4 (-44.6 to -0.0)
Equatorial Guinea	YLDs Number: 1990	13,263 (10,007–17,099)	2,090 (1,493–2,829)	1,649 (1,163–2,263)	769 (513–1,068)	299 (215–403)	810 (585–1,093)	140 (88–213)	240 (147–360)	208 (125–321)	960 (693–1,281)	42 (31–55)
Equatorial Guinea	YLDs Number: 2015	29,184 (21,977–37,752)	3,134 (2,153–4,318)	4,051 (2,836–5,553)	2,452 (1,635–3,355)	653 (456–893)	1,001 (720–1,317)	398 (251–590)	233 (140–357)	418 (243–637)	2,645 (1,917–3,562)	77 (55–103)
Equatorial Guinea	YLDs Number: Cumulative change (%)	120.0 (111.4–130.7)	50.2 (31.9–77.2)	145.4 (138.9–151.5)	219.2 (190.1–248.0)	118.6 (87.3–152.4)	23.7 (14.6–32.8)	187.4 (132.3–279.4)	0.6 (-37.9 to 52.3)	110.3 (19.5–229.2)	175.8 (154.3–201.1)	84.3 (50.8–119.6)
Equatorial Guinea	YLDs Rate: 1990	7,852.4 (5,929.1–10,077.4)	1,100.6 (789.5–1,484.9)	967.2 (678.9–1,333.1)	475.9 (317.1–652.5)	166.7 (120.2–223.0)	350.4 (253.8–471.2)	77.2 (47.5–117.8)	90.8 (55.9–136.2)	120.6 (73.2–185.4)	565.0 (411.1–750.9)	19.0 (13.9–25.2)
Equatorial Guinea	YLDs Rate: 2015	7,157.6 (5,390.5–9,245.4)	720.6 (500.6–989.2)	985.2 (688.3–1,353.6)	599.5 (399.4–818.2)	154.6 (108.3–211.0)	221.8 (159.4–292.2)	94.4 (59.5–140.3)	48.0 (28.9–73.0)	100.5 (58.1–153.0)	634.3 (460.1–852.7)	17.5 (12.6–23.3)
Equatorial Guinea	YLDs Rate: Cumulative change (%)	-8.9 (-12.1 to -4.6)	-34.5 (-41.6 to -23.9)	1.8 (-0.2 to 3.7)	26.1 (15.5–36.6)	-7.1 (-20.6 to 5.0)	-36.6 (-40.8 to -32.4)	24.5 (0.4–63.7)	-45.4 (-66.2 to -17.5)	-12.8 (-50.6 to 37.5)	12.3 (4.3–21.1)	-7.5 (-24.9 to 11.4)
Gabon	Prevalence Number: 1990	478,859 (477,532–480,134)	160,864 (143,412–174,982)	191,288 (183,209–199,198)	62,913 (51,757–74,785)	177,185 (170,233–184,637)	8,916 (8,140–9,774)	6,074 (4,913–7,613)	3,580 (2,464–4,856)	1,638 (1,073–2,304)	154,556 (135,930–173,915)	2,235 (1,757–2,797)
Gabon	Prevalence Number: 2015	802,965 (800,653–804,852)	261,563 (248,532–272,311)	329,732 (316,669–342,154)	99,711 (82,743–119,268)	265,229 (253,118–277,697)	10,793 (10,007–11,635)	11,310 (9,256–14,232)	3,966 (2,828–5,318)	2,487 (1,594–3,561)	312,988 (242,699–382,832)	3,793 (3,037–4,664)
Gabon	Prevalence Number: Cumulative change (%)	67.7 (67.1–68.3)	63.2 (47.4–82.3)	72.5 (63.3–82.0)	58.7 (46.5–71.4)	49.8 (41.3–58.2)	21.2 (14.3–27.9)	89.4 (39.9–147.9)	15.7 (-29.9 to 76.9)	59.1 (-10.1 to 153.1)	102.1 (70.1–133.2)	71.3 (36.4–109.8)
Gabon	Prevalence Rate: 1990	98,368.1 (98,130.3–98,603.8)	32,415.6 (29,394.5–34,887.3)	40,650.2 (39,043.7–42,176.0)	13,175.1 (10,909.4–15,665.7)	36,314.4 (34,886.5–37,828.6)	1,615.6 (1,484.2–1,761.4)	1,234.0 (996.2–1,545.8)	603.7 (415.5–819.3)	336.6 (220.7–473.0)	31,731.2 (28,104.7–35,432.8)	418.0 (326.9–528.1)
Gabon	Prevalence Rate: 2015	98,307.3 (98,043.5–98,524.3)	31,674.5 (30,262.1–32,897.4)	41,056.1 (39,473.8–42,541.0)	12,351.3 (10,258.3–14,790.2)	32,431.7 (30,938.9–33,959.9)	1,246.5 (1,158.6–1,341.3)	1,378.3 (1,127.2–1,735.4)	435.4 (310.4–583.7)	304.6 (195.8–436.7)	38,289.8 (29,755.6–46,863.1)	443.9 (355.0–548.9)
Gabon	Prevalence Rate: Cumulative change (%)	-0.1 (-0.4 to 0.3)	-2.0 (-10.1 to 8.0)	1.0 (-4.1 to 6.3)	-6.1 (-13.4 to 1.3)	-10.6 (-15.7 to -5.6)	-22.8 (-26.9 to -18.8)	13.6 (-16.1 to 49.0)	-24.7 (-54.4 to 15.2)	-5.2 (-46.0 to 50.4)	20.4 (1.2–39.8)	7.4 (-16.2 to 32.8)
Gabon	YLDs Number: 1990	38,817 (28,753–50,396)	6,748 (4,711–9,115)	4,585 (3,212–6,267)	2,825 (1,852–3,869)	566 (400–763)	1,445 (1,028–1,947)	512 (326–748)	441 (264–668)	657 (379–986)	1,812 (1,283–2,388)	191 (141–251)
Gabon	YLDs Number: 2015	68,704 (48,936–96,139)	10,590 (7,567–14,297)	7,784 (5,461–10,691)	4,483 (2,932–6,167)	872 (606–1,189)	1,748 (1,263–2,322)	1,156 (682–1,819)	489 (295–726)	770 (451–1,182)	3,159 (2,267–4,260)	335 (245–444)
Gabon	YLDs Number: Cumulative change (%)	76.3 (58.8–116.4)	57.8 (39.7–83.7)	69.7 (66.4–73.2)	58.9 (46.3–71.7)	55.0 (22.2–83.2)	21.2 (13.4–29.0)	130.8 (52.2–243.4)	16.0 (-30.2 to 79.1)	23.2 (-31.3 to 97.3)	75.0 (53.9–100.9)	75.4 (53.3–97.0)
Gabon	YLDs Rate: 1990	8,170.7 (6,060.1–10,574.4)	1,356.1 (951.9–1,819.9)	951.1 (666.5–1,296.3)	591.3 (387.5–805.5)	114.2 (81.0–153.2)	261.6 (186.5–350.2)	103.7 (65.9–152.0)	74.4 (44.7–112.7)	135.0 (78.4–202.2)	366.3 (260.0–482.2)	37.6 (27.6–49.3)
Gabon	YLDs Rate: 2015	8,538.0 (6,080.3–11,922.5)	1,285.0 (920.2–1,726.6)	957.7 (670.5–1,315.7)	555.2 (366.1–762.8)	106.1 (73.8–144.2)	201.9 (146.2–267.8)	140.7 (82.9–221.4)	53.7 (32.5–79.7)	94.3 (55.1–144.9)	385.6 (276.9–518.7)	40.3 (29.5–53.5)
Gabon	YLDs Rate: Cumulative change (%)	4.0 (-6.2 to 27.9)	-4.9 (-14.9 to 8.6)	0.7 (-1.0 to 2.6)	-6.0 (-13.4 to 1.3)	-6.6 (-25.8 to 8.9)	-22.7 (-27.6 to -18.0)	38.8 (-7.9 to 106.8)	-24.5 (-54.5 to 16.6)	-26.5 (-59.1 to 17.5)	5.6 (-6.2 to 19.2)	7.4 (-5.9 to 20.9)

© 2017 Global Burden of Disease Child and Adolescent Health Collaboration. JAMA Pediatrics.

eTable 4. Prevalent cases, Rates (per 100,000 population), Years Lived with Disability (YLDs), and Cumulative Percent Change with 95% Uncertainty Interval (UI) for the Top 10 Global Causes of YLDsin Children and Adolescents in 195 Countries and Territories, Aged Under 5 Years, Both Sexes, 1990 and 2015. *Note: best viewed by enlarging in browser.*

Location	Measure	All causes	Iron-deficiency anemia	Skin and subcutaneous diseases	Protein-energy malnutrition	Diarrheal diseases	Hemoglobinopathies and hemolytic anemias	Asthma	Neonatal preterm birth complications	Malaria	Neonatal encephalopathy due to birth asphyxia and trauma	Other neonatal disorders
Global	Prevalence Number: 1990	558,401,064 (557,259,861–559,563,336)	230,096,585 (225,947,988–234,141,002)	144,288,869 (142,952,534–145,687,059)	25,434,622 (24,530,444–26,389,977)	18,796,263 (17,168,214–20,603,354)	152,595,623 (151,154,301–154,359,412)	22,761,865 (17,664,624–28,614,107)	10,194,803 (9,136,197–11,306,571)	42,014,665 (34,107,691–50,439,478)	1,980,605 (1,517,568–2,534,960)	-
Global	Prevalence Number: 2015	576,893,302 (575,312,689–578,250,120)	242,217,647 (237,764,009–246,698,237)	152,511,901 (150,982,435–154,078,171)	22,413,471 (21,488,336–23,373,697)	14,993,809 (13,885,572–16,255,769)	160,389,399 (158,967,210–162,177,623)	20,379,618 (15,755,567–25,714,433)	10,039,322 (8,937,793–11,252,128)	49,069,604 (41,193,009–58,100,392)	2,681,494 (2,077,797–3,374,978)	-
Global	Prevalence Number: Cumulative change (%	3.3 (3.0–3.6)	5.3 (2.5–8.1)	5.7 (4.6–6.7)	-11.8 (-16.7 to -6.8)	-20.2 (-22.2 to -18.1)	5.1 (4.0–6.2)	-10.5 (-15.1 to -5.5)	-1.5 (-3.9 to 1.0)	17.0 (9.0–24.2)	35.6 (31.0–40.4)	-
Global	Prevalence Rate: 1990	87,507.3 (87,328.7–87,689.6)	35,966.4 (35,313.0–36,600.7)	22,658.1 (22,448.1–22,877.4)	3,980.0 (3,838.7–4,129.4)	2,941.4 (2,686.5–3,224.1)	23,896.9 (23,671.2–24,173.2)	3,586.0 (2,782.9–4,508.0)	1,574.9 (1,411.6–1,747.0)	6,604.5 (5,359.5–7,930.8)	308.0 (235.5–394.9)	-
Global	Prevalence Rate: 2015	85,919.5 (85,683.8–86,121.6)	36,052.0 (35,389.4–36,719.9)	22,725.3 (22,497.3–22,958.8)	3,337.0 (3,199.3–3,480.0)	2,231.8 (2,066.9–2,419.7)	23,883.4 (23,671.6–24,149.7)	3,039.1 (2,349.5–3,834.6)	1,490.0 (1,326.6–1,669.6)	7,312.4 (6,137.6–8,659.0)	398.7 (308.8–501.8)	-
Global	Prevalence Rate: Cumulative change (%	-1.8 (-2.1 to -1.6)	0.3 (-2.4 to 3.0)	0.3 (-0.7 to 1.2)	-16.1 (-20.7 to -11.3)	-24.1 (-26.0 to -22.1)	-0.1 (-1.1 to 1.0)	-15.2 (-19.6 to -10.5)	-5.4 (-7.7 to -3.0)	11.0 (3.3–17.8)	29.6 (25.2–34.3)	-
Global	YLDs Number: 1990	35,189,627 (26,448,377–45,685,636)	9,508,258 (6,920,421–12,660,596)	3,806,434 (2,639,934–5,176,715)	3,136,175 (2,183,363–4,210,118)	3,052,547 (2,214,951–4,051,661)	1,117,166 (803,178–1,488,878)	1,035,477 (680,033–1,498,417)	721,248 (565,137–908,896)	564,184 (398,626–766,055)	398,154 (269,700–548,906)	137,624 (92,683–189,253)
Global	YLDs Number: 2015	33,322,646 (24,871,475–43,068,753)	9,388,301 (6,777,883–12,604,370)	4,034,981 (2,798,122–5,482,606)	2,775,002 (1,922,630–3,691,538)	2,442,575 (1,764,664–3,238,703)	1,116,265 (801,227–1,495,853)	928,885 (603,571–1,344,504)	641,521 (497,412–815,910)	614,083 (435,507–826,334)	528,363 (365,594–725,755)	155,385 (107,382–208,679)
Global	YLDs Number: Cumulative change (%)	-5.3 (-8.5 to -2.6)	-1.3 (-5.7 to 3.1)	6.1 (4.5–7.6)	-11.5 (-16.4 to -6.3)	-20.0 (-22.1 to -17.9)	-0.1 (-4.9 to 4.7)	-10.3 (-14.9 to -5.4)	-11.1 (-14.1 to -8.1)	8.9 (-0.6 to 18.1)	33.0 (27.9–38.5)	14.5 (-10.2 to 43.1)
Global	YLDs Rate: 1990	5,508.0 (4,140.6–7,150.6)	1,484.4 (1,080.5–1,977.2)	598.3 (415.0–813.6)	490.8 (341.7–658.8)	477.7 (346.5–634.4)	174.3 (125.3–232.4)	163.1 (107.1–236.1)	112.7 (88.3–142.0)	88.6 (62.6–120.3)	62.2 (42.1–85.8)	21.5 (14.5–29.6)
Global	YLDs Rate: 2015	4,961.4 (3,703.0–6,412.4)	1,396.9 (1,008.3–1,875.3)	601.4 (417.0–817.1)	413.2 (286.2–549.6)	363.6 (262.6–482.1)	166.1 (119.2–222.6)	138.5 (90.0–200.5)	95.5 (74.0–121.5)	91.5 (64.9–123.1)	78.7 (54.4–108.0)	23.1 (16.0–31.1)
Global	YLDs Rate: Cumulative change (%)	-9.9 (-12.9 to -7.4)	-5.9 (-10.2 to -1.7)	0.6 (-0.9 to 2.0)	-15.8 (-20.5 to -10.9)	-23.9 (-25.9 to -21.9)	-4.8 (-9.3 to -0.1)	-15.1 (-19.4 to -10.5)	-15.3 (-18.1 to -12.4)	3.4 (-5.7 to 12.0)	26.6 (21.8–32.0)	9.1 (-14.4 to 36.2)
High SDI	Prevalence Number: 1990	53,716,748 (53,189,030–54,228,990)	17,985,924 (16,696,793–19,628,772)	13,872,961 (13,666,708–14,068,145)	388,070 (277,053–538,470)	254,547 (234,620–277,321)	10,008,158 (9,879,519–10,149,959)	2,505,228 (1,978,033–3,076,824)	1,487,896 (1,402,954–1,566,033)	349 (165–621)	101,140 (80,575–125,681)	-
High SDI	Prevalence Number: 2015	48,316,086 (47,881,007–48,762,502)	16,697,292 (15,766,734–17,774,530)	12,517,557 (12,336,408–12,674,616)	233,608 (159,870–342,071)	151,831 (140,300–163,813)	8,219,270 (8,096,867–8,344,406)	2,347,013 (2,057,622–2,666,254)	1,294,446 (1,213,617–1,373,695)	64 (38–121)	87,292 (70,626–106,679)	-
High SDI	Prevalence Number: Cumulative change (%	-10.1 (-11.1 to -9.0)	-6.9 (-15.5 to 4.1)	-9.8 (-11.1 to -8.3)	-37.2 (-64.1 to 1.1)	-40.3 (-43.4 to -37.7)	-17.9 (-19.1 to -16.6)	-5.6 (-15.0 to 5.6)	-13.0 (-14.9 to -11.1)	-79.7 (-90.8 to -63.6)	-13.6 (-16.4 to -10.7)	-
High SDI	Prevalence Rate: 1990	70,355.9 (69,669.8–71,024.3)	23,706.4 (22,019.1–25,850.2)	18,114.8 (17,845.8–18,368.8)	509.2 (363.5–706.5)	334.8 (308.8–364.8)	13,127.5 (12,959.4–13,313.4)	3,256.2 (2,571.0–3,999.1)	1,993.7 (1,881.0–2,096.5)	0.5 (0.2–0.8)	133.6 (106.7–166.1)	-
High SDI	Prevalence Rate: 2015	69,168.2 (68,544.5–69,808.2)	23,965.8 (22,629.9–25,506.1)	17,898.2 (17,639.6–18,122.8)	334.8 (229.1–490.2)	217.7 (201.3–234.9)	11,773.5 (11,598.3–11,952.8)	3,349.6 (2,936.6–3,805.2)	1,870.7 (1,753.7–1,985.3)	0.1 (0.1–0.2)	125.4 (101.5–153.2)	-
High SDI	Prevalence Rate: Cumulative change (%	-1.7 (-2.9 to -0.5)	1.3 (-7.9 to 13.3)	-1.2 (-2.7 to 0.4)	-31.4 (-60.8 to 10.4)	-34.9 (-38.3 to -32.1)	-10.3 (-11.6 to -9.0)	3.7 (-6.7 to 15.9)	-6.2 (-8.2 to -4.1)	-77.8 (-89.9 to -60.1)	-6.0 (-9.0 to -2.8)	-
High SDI	YLDs Number: 1990	2,119,124 (1,594,925–2,753,142)	500,721 (340,940–692,695)	417,510 (293,842–558,089)	48,624 (29,306–75,220)	42,044 (30,020–55,717)	50,045 (34,038–68,799)	115,469 (75,965–160,905)	80,854 (61,941–103,792)	7 (2–13)	19,877 (13,709–26,878)	15,977 (12,239–20,482)
High SDI	YLDs Number: 2015	1,841,490 (1,382,073–2,376,718)	466,894 (326,126–637,099)	379,403 (268,224–507,138)	29,383 (16,939–46,336)	25,128 (17,953–33,440)	45,110 (31,692–62,097)	108,173 (73,567–146,549)	55,106 (41,663–71,737)	1 (0–2)	16,389 (11,475–22,349)	20,991 (15,624–27,167)
High SDI	YLDs Number: Cumulative change (%)	-13.1 (-17.5 to -8.0)	-6.3 (-18.9 to 10.8)	-9.1 (-9.9 to -8.3)	-37.0 (-63.8 to 1.7)	-40.2 (-43.9 to -36.5)	-9.4 (-21.8 to 5.1)	-5.6 (-15.1 to 6.0)	-31.9 (-35.0 to -28.9)	-81.7 (-93.6 to -62.5)	-17.4 (-22.8 to -11.4)	31.4 (17.6–44.4)
High SDI	YLDs Rate: 1990	2,778.8 (2,091.7–3,610.1)	661.6 (451.0–914.0)	544.2 (383.0–727.3)	63.8 (38.4–98.7)	55.3 (39.5–73.3)	66.1 (45.0–90.8)	150.1 (98.7–209.1)	106.0 (81.2–136.2)	0.0 (0.0–0.0)	26.1 (18.0–35.2)	21.0 (16.1–26.9)
High SDI	YLDs Rate: 2015	2,637.6 (1,979.6–3,404.2)	670.9 (468.5–915.1)	542.1 (383.3–724.6)	42.1 (24.3–66.4)	36.0 (25.8–47.9)	64.8 (45.6–89.2)	154.4 (105.0–209.2)	78.9 (59.7–102.8)	0.0 (0.0–0.0)	23.5 (16.4–32.0)	30.1 (22.4–38.9)
High SDI	YLDs Rate: Cumulative change (%)	-5.0 (-9.8 to 0.5)	1.9 (-11.8 to 20.5)	-0.4 (-1.2 to 0.6)	-31.2 (-60.5 to 11.1)	-34.8 (-38.8 to -30.8)	-1.4 (-14.8 to 14.2)	3.7 (-6.7 to 16.4)	-25.6 (-29.0 to -22.3)	-80.0 (-93.0 to -58.9)	-9.7 (-15.7 to -3.2)	43.5 (28.4–57.6)
High-middle SDI	Prevalence Number: 1990	119,138,060 (118,624,294–119,693,638)	39,753,092 (38,404,139–41,094,567)	32,118,242 (31,728,680–32,572,403)	3,296,111 (2,977,032–3,686,325)	2,900,188 (2,651,541–3,192,765)	23,652,118 (23,445,888–23,868,954)	5,136,656 (4,008,935–6,466,228)	2,434,683 (2,216,446–2,655,150)	977,959 (563,372–1,537,998)	277,448 (215,083–351,274)	-
High-middle SDI	Prevalence Number: 2015	98,606,932 (98,153,806–99,038,922)	33,750,762 (32,687,558–34,949,074)	27,414,118 (27,086,933–27,752,449)	1,994,856 (1,833,900–2,208,290)	1,621,399 (1,480,443–1,774,398)	19,579,313 (19,406,668–19,749,209)	3,660,319 (2,782,894–4,659,657)	1,881,774 (1,711,985–2,063,882)	494,163 (250,538–928,447)	269,673 (213,233–340,186)	-
High-middle SDI	Prevalence Number: Cumulative change (%	-17.2 (-17.7 to -16.8)	-15.1 (-18.8 to -11.0)	-14.6 (-15.7 to -13.5)	-39.2 (-47.2 to -30.8)	-44.1 (-47.5 to -41.0)	-17.2 (-18.1 to -16.4)	-28.8 (-33.3 to -23.8)	-22.7 (-24.7 to -20.6)	-50.3 (-63.7 to -31.3)	-2.6 (-5.9 to 0.6)	-
High-middle SDI	Prevalence Rate: 1990	85,272.6 (84,904.5–85,670.2)	28,508.5 (27,545.6–29,470.3)	22,966.1 (22,687.1–23,288.7)	2,361.1 (2,132.6–2,640.6)	2,078.8 (1,900.9–2,288.5)	16,936.6 (16,789.2–17,091.9)	3,666.1 (2,861.2–4,615.1)	1,755.9 (1,598.9–1,914.6)	698.2 (402.4–1,097.9)	199.2 (154.6–252.1)	-
High-middle SDI	Prevalence Rate: 2015	82,135.2 (81,757.2–82,491.2)	28,248.9 (27,365.7–29,239.6)	22,782.9 (22,511.4–23,063.9)	1,665.4 (1,531.0–1,843.4)	1,356.3 (1,238.7–1,484.1)	16,330.0 (16,185.9–16,471.1)	3,028.6 (2,302.6–3,855.5)	1,598.0 (1,454.1–1,752.4)	409.5 (208.0–768.7)	226.3 (178.9–285.1)	-
High-middle SDI	Prevalence Rate: Cumulative change (%	-3.7 (-4.2 to -3.1)	-0.9 (-5.2 to 3.8)	-0.8 (-2.0 to 0.6)	-29.2 (-38.5 to -19.3)	-34.7 (-38.7 to -31.2)	-3.6 (-4.6 to -2.6)	-17.4 (-22.7 to -11.6)	-9.0 (-11.4 to -6.5)	-42.3 (-57.9 to -20.2)	13.8 (10.0–17.6)	-
High-middle SDI	YLDs Number: 1990	6,174,296 (4,629,069–8,001,667)	1,383,780 (978,509–1,883,843)	866,499 (595,367–1,184,179)	426,310 (293,607–587,761)	494,026 (355,375–658,235)	153,685 (108,942–207,433)	244,184 (159,363–352,425)	196,293 (154,087–247,181)	20,387 (13,797–28,342)	59,871 (40,965–82,209)	35,947 (26,200–47,643)
High-middle SDI	YLDs Number: 2015	4,530,242 (3,376,121–5,838,326)	1,145,591 (813,003–1,536,374)	739,769 (508,010–1,009,102)	257,358 (174,926–346,866)	275,553 (196,627–367,194)	121,976 (86,657–166,295)	173,373 (111,681–251,614)	126,129 (97,841–161,523)	10,359 (6,411–15,146)	56,659 (39,414–78,165)	29,021 (21,660–37,367)
High-middle SDI	YLDs Number: Cumulative change (%)	-26.6 (-29.1 to -24.2)	-17.2 (-22.8 to -11.3)	-14.6 (-15.9 to -13.4)	-39.4 (-47.5 to -31.1)	-44.2 (-47.7 to -41.1)	-20.6 (-26.7 to -13.8)	-29.0 (-33.7 to -24.1)	-35.8 (-38.5 to -33.0)	-49.1 (-60.4 to -35.4)	-5.1 (-9.4 to -0.2)	-18.3 (-35.7 to 0.8)
High-middle SDI	YLDs Rate: 1990	4,422.2 (3,315.3–5,730.6)	993.1 (702.6–1,352.1)	619.3 (425.4–846.3)	305.4 (210.3–421.0)	354.1 (254.7–471.8)	110.3 (78.2–148.9)	174.3 (113.7–251.5)	140.6 (110.4–177.1)	14.6 (9.9–20.2)	42.9 (29.3–58.9)	25.8 (18.8–34.1)
High-middle SDI	YLDs Rate: 2015	3,780.0 (2,817.4–4,871.6)	960.8 (682.1–1,289.3)	614.1 (421.5–837.6)	214.8 (146.0–289.6)	230.5 (164.6–307.0)	102.4 (72.8–139.6)	143.5 (92.4–208.2)	105.2 (81.6–134.7)	8.6 (5.3–12.6)	47.3 (32.9–65.2)	24.2 (18.1–31.2)
High-middle SDI	YLDs Rate: Cumulative change (%)	-14.5 (-17.4 to -11.7)	-3.2 (-9.7 to 3.6)	-0.8 (-2.3 to 0.6)	-29.4 (-38.8 to -19.7)	-34.9 (-39.0 to -31.2)	-7.1 (-14.3 to 0.7)	-17.7 (-23.2 to -12.0)	-25.2 (-28.4 to -21.9)	-40.9 (-54.0 to -25.0)	10.5 (5.6–16.2)	-4.8 (-25.1 to 17.4)
Middle SDI	Prevalence Number: 1990	180,095,452 (179,420,602–180,736,070)	67,895,686 (65,680,925–70,160,746)	48,136,616 (47,342,425–48,952,183)	7,390,385 (6,885,192–7,966,637)	5,646,939 (5,112,657–6,255,348)	46,496,226 (46,107,187–46,899,980)	6,915,844 (5,364,228–8,749,034)	3,457,415 (3,081,412–3,847,452)	3,422,389 (2,164,109–5,032,214)	445,986 (339,808–569,395)	-
Middle SDI	Prevalence Number: 2015	153,820,255 (153,001,367–154,644,804)	58,580,449 (56,169,504–60,667,250)	42,627,003 (41,883,528–43,416,707)	5,308,951 (4,879,376–5,781,784)	3,538,981 (3,249,174–3,838,913)	38,343,806 (37,982,376–38,700,252)	4,940,431 (3,788,910–6,304,467)	3,100,404 (2,770,286–3,459,859)	1,984,279 (1,195,254–3,121,876)	465,684 (361,840–591,758)	-
Middle SDI	Prevalence Number: Cumulative change (%	-14.6 (-15.1 to -14.1)	-13.7 (-19.1 to -8.7)	-11.4 (-13.1 to -9.8)	-28.0 (-35.9 to -19.7)	-37.3 (-40.4 to -34.3)	-17.5 (-18.4 to -16.7)	-28.6 (-32.5 to -24.7)	-10.3 (-13.0 to -7.3)	-42.3 (-53.8 to -30.2)	4.6 (0.3–9.5)	-

© 2017 Global Burden of Disease Child and Adolescent Health Collaboration. JAMA Pediatrics.

eTable 4. Prevalent cases, Rates (per 100,000 population), Years Lived with Disability (YLDs), and Cumulative Percent Change with 95% Uncertainty Interval (UI) for the Top 10 Global Causes of YLDsin Children and Adolescents in 195 Countries and Territories, Aged Under 5 Years, Both Sexes, 1990 and 2015. *Note: best viewed by enlarging in browser.*

Location	Measure	All causes	Iron-deficiency anemia	Skin and subcutaneous diseases	Protein-energy malnutrition	Diarrheal diseases	Hemoglobinopathies and hemolytic anemias	Asthma	Neonatal preterm birth complications	Malaria	Neonatal encephalopathy due to birth asphyxia and trauma	Other neonatal disorders
Middle SDI	Prevalence Rate: 1990	89,371.3 (89,035.9–89,691.7)	33,621.5 (32,518.3–34,742.9)	23,921.5 (23,526.0–24,327.5)	3,663.6 (3,413.2–3,949.2)	2,798.4 (2,533.1–3,100.2)	23,062.2 (22,868.9–23,262.5)	3,446.3 (2,673.1–4,359.8)	1,696.5 (1,512.9–1,888.0)	1,704.2 (1,077.0–2,506.6)	220.3 (167.6–281.4)	-
Middle SDI	Prevalence Rate: 2015	85,754.3 (85,297.5–86,214.1)	32,648.6 (31,303.8–33,813.4)	23,768.1 (23,353.5–24,208.5)	2,959.3 (2,719.9–3,222.9)	1,972.4 (1,810.9–2,139.7)	21,374.7 (21,173.2–21,573.4)	2,755.8 (2,113.5–3,516.7)	1,725.8 (1,541.9–1,925.7)	1,106.7 (666.5–1,741.2)	259.5 (201.6–329.7)	-
Middle SDI	Prevalence Rate: Cumulative change (%	-4.0 (-4.6 to -3.5)	-2.8 (-9.0 to 2.8)	-0.6 (-2.5 to 1.2)	-19.1 (-27.9 to -9.7)	-29.4 (-33.0 to -26.1)	-7.3 (-8.2 to -6.4)	-20.1 (-24.4 to -15.7)	1.7 (-1.4 to 5.2)	-35.3 (-48.3 to -21.8)	18.0 (13.2–23.6)	-
Middle SDI	YLDs Number: 1990	10,376,773 (7,755,872–13,497,953)	2,583,739 (1,868,887–3,474,788)	1,202,865 (820,958–1,655,536)	890,132 (617,843–1,196,693)	896,641 (650,258–1,193,020)	324,405 (230,911–437,313)	306,350 (199,232–445,386)	257,605 (202,177–324,925)	67,715 (46,709–94,503)	93,226 (63,242–128,225)	36,091 (24,288–51,457)
Middle SDI	YLDs Number: 2015	7,989,323 (5,962,383–10,335,475)	2,078,929 (1,479,965–2,818,162)	1,076,528 (732,758–1,479,853)	645,150 (442,532–872,871)	565,920 (405,233–750,038)	268,265 (190,418–367,194)	220,329 (142,533–318,813)	210,171 (163,451–266,173)	34,859 (23,049–50,144)	95,500 (66,138–130,471)	32,841 (22,873–44,321)
Middle SDI	YLDs Number: Cumulative change (%)	-23.0 (-26.7 to -19.7)	-19.5 (-26.6 to -13.3)	-10.5 (-11.9 to -9.0)	-27.4 (-35.1 to -18.9)	-36.8 (-40.0 to -33.6)	-17.3 (-24.9 to -9.9)	-28.1 (-31.9 to -24.1)	-18.4 (-22.3 to -14.1)	-48.5 (-56.7 to -39.8)	2.7 (-3.0 to 9.3)	-6.5 (-34.2 to 25.1)
Middle SDI	YLDs Rate: 1990	5,144.3 (3,844.7–6,691.5)	1,278.1 (924.6–1,718.9)	598.1 (408.2–823.4)	441.3 (306.3–593.3)	444.3 (322.1–591.3)	160.4 (114.1–216.1)	152.7 (99.3–221.9)	127.6 (100.1–161.0)	33.7 (23.3–47.0)	46.2 (31.4–63.6)	17.9 (12.0–25.5)
Middle SDI	YLDs Rate: 2015	4,453.5 (3,323.6–5,761.4)	1,158.5 (824.8–1,570.5)	600.3 (408.6–825.2)	359.6 (246.7–486.6)	315.4 (225.8–418.0)	149.5 (106.1–204.6)	122.9 (79.5–177.8)	117.2 (91.1–148.4)	19.4 (12.9–28.0)	53.2 (36.9–72.7)	18.3 (12.8–24.7)
Middle SDI	YLDs Rate: Cumulative change (%)	-13.4 (-17.6 to -9.7)	-9.4 (-17.3 to -2.3)	0.4 (-1.2 to 2.0)	-18.3 (-27.1 to -8.9)	-29.0 (-32.5 to -25.4)	-6.8 (-15.3 to 1.5)	-19.5 (-23.8 to -15.1)	-8.2 (-12.6 to -3.4)	-42.3 (-51.5 to -32.6)	15.5 (9.1–22.9)	5.2 (-25.9 to 40.8)
Low-middle SDI	Prevalence Number: 1990	159,000,053 (158,569,527–159,456,866)	80,330,006 (77,722,092–82,849,773)	37,622,477 (36,911,971–38,300,280)	11,816,946 (11,312,148–12,318,324)	7,073,531 (6,471,405–7,760,201)	56,582,240 (55,443,033–58,098,921)	6,284,450 (4,856,156–8,003,859)	2,220,570 (1,893,657–2,575,178)	26,451,727 (21,916,936–31,168,042)	815,453 (612,524–1,052,132)	-
Low-middle SDI	Prevalence Number: 2015	191,658,368 (190,922,633–192,365,361)	93,383,868 (90,118,021–96,586,575)	46,742,613 (45,890,683–47,679,724)	10,882,417 (10,230,936–11,614,803)	6,417,092 (5,967,765–6,936,616)	66,713,669 (65,613,143–68,248,247)	6,259,100 (4,729,717–8,058,236)	2,667,285 (2,282,080–3,076,981)	24,676,044 (21,514,566–28,633,784)	1,192,031 (911,703–1,524,457)	-
Low-middle SDI	Prevalence Number: Cumulative change (%	20.5 (20.0–21.0)	16.3 (10.9–21.8)	24.3 (21.8–26.8)	-7.8 (-14.4 to -0.2)	-9.2 (-12.0 to -6.4)	17.9 (14.8–20.9)	-0.5 (-6.1 to 5.4)	20.2 (15.8–24.7)	-6.4 (-13.7 to 2.4)	46.5 (39.0–55.4)	-
Low-middle SDI	Prevalence Rate: 1990	92,821.0 (92,568.1–93,087.2)	46,707.3 (45,170.4–48,178.9)	22,080.2 (21,663.8–22,478.0)	6,877.7 (6,583.9–7,169.5)	4,122.1 (3,768.9–4,523.5)	32,992.2 (32,328.7–33,876.5)	3,716.0 (2,871.5–4,732.7)	1,252.2 (1,069.8–1,450.9)	15,567.3 (12,886.2–18,370.4)	467.3 (351.5–604.4)	-
Low-middle SDI	Prevalence Rate: 2015	90,789.2 (90,439.2–91,123.3)	44,183.3 (42,633.9–45,706.5)	22,173.6 (21,769.0–22,619.0)	5,151.0 (4,842.8–5,497.6)	3,035.8 (2,823.0–3,282.8)	31,592.5 (31,071.2–32,319.5)	2,975.1 (2,248.1–3,830.2)	1,252.5 (1,071.9–1,444.0)	11,707.5 (10,203.2–13,586.5)	562.2 (430.0–719.2)	-
Low-middle SDI	Prevalence Rate: Cumulative change (%	-2.2 (-2.6 to -1.8)	-5.4 (-9.8 to -0.8)	0.4 (-1.5 to 2.5)	-25.1 (-30.4 to -18.9)	-26.3 (-28.6 to -24.0)	-4.2 (-6.7 to -1.8)	-20.0 (-24.6 to -15.3)	0.1 (-3.6 to 3.9)	-24.6 (-30.4 to -17.5)	20.6 (14.5–27.9)	-
Low-middle SDI	YLDs Number: 1990	12,701,528 (9,469,120–16,475,724)	3,872,325 (2,846,748–5,128,125)	994,709 (688,909–1,341,557)	1,453,441 (1,001,071–1,943,968)	1,143,665 (831,839–1,519,796)	489,022 (355,523–645,851)	283,844 (183,496–409,700)	162,649 (124,639–207,508)	310,145 (216,162–423,812)	165,777 (110,071–231,084)	31,033 (14,430–52,852)
Low-middle SDI	YLDs Number: 2015	13,088,285 (9,765,667–16,893,837)	3,968,110 (2,872,151–5,287,107)	1,233,510 (853,963–1,669,007)	1,346,349 (924,532–1,794,740)	1,043,968 (754,898–1,384,144)	515,571 (370,449–692,053)	284,300 (179,950–418,680)	203,522 (157,223–258,451)	273,091 (191,719–372,722)	240,836 (164,700–330,617)	41,467 (24,803–64,493)
Low-middle SDI	YLDs Number: Cumulative change (%)	3.2 (-3.1 to 8.3)	2.5 (-5.4 to 11.0)	24.0 (22.3–25.6)	-7.3 (-14.0 to 0.1)	-8.7 (-11.8 to -5.7)	5.4 (-3.0 to 13.8)	0.1 (-6.0 to 6.3)	25.3 (17.5–32.9)	-11.7 (-24.7 to 1.5)	45.7 (37.4–55.2)	46.4 (-19.0 to 136.5)
Low-middle SDI	YLDs Rate: 1990	7,395.5 (5,512.7–9,593.9)	2,245.1 (1,649.3–2,977.9)	584.9 (405.5–789.0)	846.0 (582.7–1,131.6)	666.5 (484.7–885.8)	283.3 (206.0–374.5)	167.8 (108.5–242.3)	93.9 (71.8–119.5)	182.1 (126.9–249.1)	96.2 (63.8–134.2)	17.9 (8.3–30.5)
Low-middle SDI	YLDs Rate: 2015	6,195.1 (4,621.9–7,995.6)	1,875.5 (1,356.9–2,498.8)	585.5 (405.3–792.1)	637.3 (437.6–849.5)	493.9 (357.1–654.8)	243.6 (175.1–327.0)	135.1 (85.5–199.0)	96.2 (74.4–122.2)	129.5 (90.8–176.6)	113.9 (77.9–156.4)	19.6 (11.7–30.5)
Low-middle SDI	YLDs Rate: Cumulative change (%)	-16.1 (-21.3 to -11.9)	-16.5 (-22.9 to -9.3)	0.1 (-1.3 to 1.4)	-24.6 (-30.1 to -18.6)	-25.9 (-28.4 to -23.5)	-14.0 (-21.0 to -7.0)	-19.6 (-24.4 to -14.5)	2.7 (-3.9 to 9.0)	-28.7 (-39.2 to -18.0)	18.9 (12.0–26.6)	19.9 (-33.6 to 93.6)
Low SDI	Prevalence Number: 1990	46,386,148 (46,259,052–46,511,510)	24,295,797 (23,206,646–25,332,682)	12,511,105 (12,170,124–12,874,741)	2,603,573 (2,311,464–2,931,045)	2,952,344 (2,689,754–3,213,890)	16,129,017 (15,913,975–16,365,094)	1,887,629 (1,464,978–2,391,684)	580,649 (455,672–716,850)	11,212,947 (9,194,135–13,374,347)	341,223 (258,655–437,148)	-
Low SDI	Prevalence Number: 2015	84,346,325 (84,031,819–84,672,571)	39,864,928 (38,267,527–41,570,804)	23,172,589 (22,521,573–23,823,852)	4,036,645 (3,649,313–4,455,850)	3,282,599 (2,999,214–3,619,580)	27,676,295 (27,290,236–28,083,006)	3,143,538 (2,353,480–4,051,467)	1,090,202 (864,658–1,336,618)	21,963,632 (17,977,533–26,190,570)	667,665 (508,836–852,795)	-
Low SDI	Prevalence Number: Cumulative change (%	81.8 (81.2–82.6)	64.2 (54.6–74.6)	85.2 (79.7–91.0)	55.9 (33.2–81.6)	11.2 (5.7–17.3)	71.6 (68.8–74.1)	66.4 (54.1–78.2)	88.0 (79.6–96.6)	96.0 (82.6–109.4)	95.9 (84.9–106.9)	-
Low SDI	Prevalence Rate: 1990	94,632.1 (94,372.3–94,884.9)	49,157.9 (46,871.9–51,317.2)	25,902.3 (25,190.3–26,657.1)	5,276.6 (4,682.2–5,939.2)	6,012.2 (5,475.6–6,549.6)	32,834.8 (32,396.8–33,314.2)	3,967.5 (3,079.1–5,026.9)	1,084.8 (855.6–1,335.6)	23,212.0 (18,994.9–27,803.1)	658.9 (498.5–842.3)	-
Low SDI	Prevalence Rate: 2015	92,894.1 (92,545.4–93,254.2)	43,696.4 (41,910.2–45,595.6)	25,680.4 (24,963.7–26,405.1)	4,432.7 (4,007.6–4,893.6)	3,601.3 (3,286.8–3,973.1)	30,446.1 (30,020.9–30,893.1)	3,506.4 (2,625.2–4,519.2)	1,155.9 (917.3–1,417.0)	24,341.5 (19,911.1–29,033.5)	719.3 (546.6–921.1)	-
Low SDI	Prevalence Rate: Cumulative change (%	-1.8 (-2.2 to -1.4)	-11.0 (-16.4 to -5.3)	-0.8 (-3.8 to 2.2)	-15.5 (-27.8 to -1.6)	-40.1 (-43.0 to -36.8)	-7.3 (-8.8 to -5.9)	-11.7 (-18.2 to -5.4)	6.7 (1.8–11.7)	4.9 (-2.2 to 12.1)	9.3 (3.2–15.7)	-
Low SDI	YLDs Number: 1990	3,800,240 (2,822,167–4,905,442)	1,163,566 (836,943–1,548,188)	322,281 (226,220–434,398)	316,624 (218,403–432,354)	474,497 (342,367–632,206)	99,661 (71,786–131,994)	84,710 (55,588–122,646)	23,231 (17,290–30,253)	165,691 (117,526–223,081)	59,258 (38,800–83,570)	18,442 (8,936–33,083)
Low SDI	YLDs Number: 2015	5,857,039 (4,360,806–7,617,588)	1,724,854 (1,248,529–2,310,269)	603,199 (423,205–813,920)	495,800 (338,511–664,472)	530,667 (385,755–709,825)	165,004 (120,110–221,241)	141,834 (90,547–206,452)	46,057 (34,219–59,386)	295,623 (208,580–399,050)	118,809 (79,546–165,703)	30,914 (15,841–53,012)
Low SDI	YLDs Number: Cumulative change (%)	54.1 (46.4–61.8)	48.4 (34.2–63.8)	87.2 (85.1–89.4)	57.3 (34.5–83.0)	11.9 (6.4–18.2)	65.7 (52.0–80.3)	67.3 (55.3–79.6)	98.4 (86.3–110.5)	78.7 (62.6–97.7)	100.9 (88.0–113.3)	80.3 (-4.4 to 202.5)
Low SDI	YLDs Rate: 1990	7,719.6 (5,726.8–9,964.5)	2,338.0 (1,682.5–3,117.0)	670.8 (471.1–904.7)	641.7 (442.5–876.1)	966.4 (696.6–1,289.0)	199.9 (143.9–265.6)	178.0 (116.8–257.8)	45.0 (33.5–58.7)	340.5 (241.5–458.8)	118.2 (77.1–166.4)	36.4 (17.6–65.5)
Low SDI	YLDs Rate: 2015	6,436.6 (4,791.7–8,373.7)	1,884.9 (1,364.6–2,526.5)	670.1 (470.0–904.0)	544.5 (371.8–729.6)	582.2 (423.7–778.6)	180.3 (131.4–241.8)	158.2 (101.0–230.3)	49.9 (37.0–64.4)	326.6 (230.4–440.8)	129.8 (86.4–181.2)	33.7 (17.3–57.8)
Low SDI	YLDs Rate: Cumulative change (%)	-16.6 (-20.9 to -12.4)	-19.3 (-27.2 to -10.7)	-0.1 (-1.2 to 1.1)	-14.7 (-27.2 to -0.9)	-39.7 (-42.6 to -36.3)	-9.8 (-17.4 to -1.6)	-11.2 (-17.6 to -4.7)	11.0 (4.1–17.7)	-3.9 (-12.7 to 6.4)	10.1 (2.8–17.2)	-0.5 (-47.1 to 66.5)
High-income	Prevalence Number: 1990	40,746,426 (40,443,435–41,058,850)	15,172,107 (14,587,193–15,775,218)	9,800,272 (9,688,206–9,914,029)	2,303 (1,548–3,261)	57,954 (54,135–62,506)	8,403,709 (8,305,001–8,503,489)	2,353,614 (1,892,658–2,862,553)	1,253,315 (1,188,817–1,314,643)	219 (101–378)	50,976 (40,746–62,981)	-
High-income	Prevalence Number: 2015	39,582,199 (39,252,200–39,904,028)	15,586,263 (14,997,216–16,190,774)	9,540,592 (9,438,874–9,650,570)	350 (236–510)	44,385 (41,065–48,176)	7,083,701 (6,983,587–7,185,847)	2,367,143 (2,083,458–2,667,348)	1,137,424 (1,068,665–1,203,763)	62 (37–119)	49,315 (39,949–60,467)	-
High-income	Prevalence Number: Cumulative change (%	-2.9 (-3.7 to -1.9)	2.8 (-3.1 to 8.6)	-2.6 (-3.8 to -1.5)	-84.7 (-87.7 to -81.3)	-23.4 (-26.3 to -20.8)	-15.7 (-16.9 to -14.5)	1.3 (-8.5 to 11.9)	-9.3 (-11.1 to -7.4)	-69.0 (-84.9 to -45.3)	-3.2 (-5.3 to -1.0)	-
High-income	Prevalence Rate: 1990	67,244.2 (66,744.7–67,759.3)	25,088.0 (24,124.5–26,082.4)	16,154.4 (15,969.9–16,341.9)	3.8 (2.6–5.4)	95.9 (89.6–103.4)	13,874.0 (13,711.1–14,038.6)	3,873.8 (3,115.2–4,711.5)	2,084.4 (1,977.0–2,185.9)	0.4 (0.2–0.6)	84.4 (67.5–104.1)	-
High-income	Prevalence Rate: 2015	67,263.1 (66,702.2–67,810.7)	26,563.3 (25,568.3–27,591.1)	16,184.3 (16,012.2–16,370.7)	0.6 (0.4–0.9)	75.7 (70.0–82.1)	12,045.0 (11,874.9–12,218.7)	4,006.0 (3,525.9–4,514.1)	1,956.9 (1,839.4–2,070.9)	0.1 (0.1–0.2)	84.2 (68.2–103.2)	-
High-income	Prevalence Rate: Cumulative change (%	0.0 (-0.9 to 1.0)	5.9 (-0.1 to 11.9)	0.2 (-1.0 to 1.4)	-84.2 (-87.4 to -80.7)	-21.0 (-24.0 to -18.3)	-13.2 (-14.4 to -11.9)	4.1 (-5.9 to 15.0)	-6.1 (-8.1 to -4.2)	-68.1 (-84.4 to -43.8)	-0.1 (-2.3 to 2.1)	-
High-income	YLDs Number: 1990	1,594,241 (1,191,231–2,076,470)	439,276 (307,313–609,340)	316,381 (225,718–419,096)	280 (160–438)	9,590 (6,905–12,728)	38,671 (27,153–53,118)	108,527 (71,667–151,886)	60,808 (46,385–78,639)	4 (1–8)	9,709 (6,664–13,113)	11,160 (8,553–14,246)
High-income	YLDs Number: 2015	1,553,945 (1,170,480–2,019,803)	463,011 (326,389–634,433)	306,623 (219,166–408,452)	42 (24–67)	7,344 (5,254–9,872)	38,243 (26,804–52,507)	109,060 (74,330–147,762)	45,861 (34,584–60,108)	1 (0–2)	9,072 (6,328–12,462)	16,561 (12,223–21,373)

© 2017 Global Burden of Disease Child and Adolescent Health Collaboration. JAMA Pediatrics.

eTable 4. Prevalent cases, Rates (per 100,000 population), Years Lived with Disability (YLDs), and Cumulative Percent Change with 95% Uncertainty Interval (UI) for the Top 10 Global Causes of YLDsin Children and Adolescents in 195 Countries and Territories, Aged Under 5 Years, Both Sexes, 1990 and 2015. *Note: best viewed by enlarging in browser.*

Location	Measure	All causes	Iron-deficiency anemia	Skin and subcutaneous diseases	Protein-energy malnutrition	Diarrheal diseases	Hemoglobinopathies and hemolytic anemias	Asthma	Neonatal preterm birth complications	Malaria	Neonatal encephalopathy due to birth asphyxia and trauma	Other neonatal disorders
High-income	YLDs Number: Cumulative change (%)	-2.5 (-6.1 to 1.0)	5.6 (-3.9 to 15.5)	-3.1 (-3.9 to -2.2)	-84.5 (-88.5 to -79.4)	-23.4 (-27.8 to -19.1)	-0.9 (-11.3 to 7.9)	1.2 (-8.6 to 12.1)	-24.7 (-27.7 to -21.6)	-72.8 (-89.0 to -46.5)	-6.4 (-11.3 to -1.2)	48.4 (33.7–63.0)
High-income	YLDs Rate: 1990	2,631.8 (1,966.3–3,428.3)	727.0 (508.6–1,008.6)	521.2 (371.8–690.4)	0.5 (0.3–0.7)	15.9 (11.4–21.0)	64.0 (44.9–87.9)	178.6 (118.0–250.0)	100.4 (76.6–129.8)	0.0 (0.0–0.0)	16.0 (11.0–21.7)	18.4 (14.1–23.5)
High-income	YLDs Rate: 2015	2,642.0 (1,989.9–3,434.4)	790.1 (556.6–1,082.2)	519.6 (371.4–692.2)	0.1 (0.0–0.1)	12.5 (9.0–16.8)	65.3 (45.8–89.6)	184.6 (125.8–250.1)	78.0 (58.8–102.2)	0.0 (0.0–0.0)	15.4 (10.8–21.2)	28.2 (20.8–36.3)
High-income	YLDs Rate: Cumulative change (%)	0.4 (-3.3 to 4.0)	8.9 (-0.9 to 19.0)	-0.3 (-1.2 to 0.6)	-84.0 (-88.2 to -78.8)	-21.0 (-25.5 to -16.6)	2.2 (-8.5 to 11.3)	4.1 (-6.0 to 15.2)	-22.4 (-25.6 to -19.3)	-72.0 (-88.6 to -44.9)	-3.6 (-8.7 to 1.7)	52.8 (37.7–67.9)
High-income North America	Prevalence Number: 1990	14,656,036 (14,532,862–14,781,109)	5,999,364 (5,695,063–6,292,996)	3,110,002 (3,083,009–3,134,958)	69 (47–98)	2,754 (2,535–2,986)	4,388,502 (4,338,622–4,438,794)	715,132 (545,182–900,836)	582,151 (557,098–606,413)	0 (0–0)	20,456 (16,512–24,979)	-
High-income North America	Prevalence Number: 2015	14,971,540 (14,816,343–15,125,908)	6,040,335 (5,744,453–6,389,478)	3,273,449 (3,244,787–3,301,083)	39 (25–56)	2,899 (2,614–3,216)	3,567,821 (3,527,439–3,611,313)	1,225,647 (1,155,512–1,291,459)	554,461 (519,820–587,190)	0 (0–0)	20,966 (17,442–24,949)	-
High-income North America	Prevalence Number: Cumulative change (%	2.2 (1.0–3.4)	0.8 (-6.0 to 7.8)	5.3 (4.3–6.3)	-43.8 (-53.8 to -33.6)	5.2 (0.6–10.1)	-18.7 (-19.7 to -17.7)	75.2 (37.9–121.9)	-4.8 (-8.5 to -0.9)	4.5 (4.3–4.6)	2.7 (-1.5 to 7.0)	-
High-income North America	Prevalence Rate: 1990	69,383.9 (68,800.4–69,975.9)	28,365.3 (26,918.1–29,759.2)	14,736.6 (14,608.6–14,855.0)	0.3 (0.2–0.5)	13.0 (12.0–14.1)	20,773.1 (20,536.9–21,011.1)	3,392.9 (2,586.6–4,273.9)	2,740.3 (2,622.2–2,854.9)	0.0 (0.0–0.0)	96.7 (78.0–118.0)	-
High-income North America	Prevalence Rate: 2015	68,138.2 (67,432.6–68,839.3)	27,561.7 (26,217.9–29,146.0)	14,873.0 (14,742.6–14,998.2)	0.2 (0.1–0.3)	13.3 (12.0–14.7)	16,243.5 (16,059.9–16,441.5)	5,556.8 (5,238.8–5,855.2)	2,551.0 (2,391.7–2,702.5)	0.0 (0.0–0.0)	95.8 (79.8–114.0)	-
High-income North America	Prevalence Rate: Cumulative change (%	-1.8 (-2.9 to -0.6)	-2.7 (-9.2 to 4.0)	0.9 (-0.0 to 1.9)	-45.0 (-54.8 to -35.0)	1.9 (-2.6 to 6.7)	-21.8 (-22.8 to -20.9)	67.4 (31.8–112.0)	-6.9 (-10.6 to -3.1)	0.1 (0.0–0.1)	-0.7 (-4.8 to 3.5)	-
High-income North America	YLDs Number: 1990	576,549 (436,137–752,829)	171,159 (120,724–236,489)	117,962 (84,858–155,702)	8 (5–13)	461 (330–625)	15,322 (10,881–20,929)	32,957 (20,888–47,624)	28,592 (21,937–36,960)	0 (0–0)	3,855 (2,692–5,238)	7,110 (5,416–9,157)
High-income North America	YLDs Number: 2015	602,887 (457,287–785,889)	171,092 (121,021–236,248)	123,718 (89,002–163,861)	4 (2–7)	484 (341–658)	15,240 (10,686–21,060)	56,473 (38,790–75,877)	22,171 (16,592–28,917)	0 (0–0)	3,819 (2,707–5,150)	7,934 (5,820–10,314)
High-income North America	YLDs Number: Cumulative change (%)	4.6 (0.3–9.4)	0.2 (-10.4 to 10.7)	4.9 (3.8–6.0)	-44.0 (-54.0 to -33.8)	5.1 (-5.5 to 16.2)	-0.4 (-10.6 to 10.0)	75.1 (37.9–122.4)	-22.5 (-26.4 to -18.7)	3.1 (-7.0 to 14.3)	-0.6 (-8.7 to 7.9)	11.7 (-2.4 to 26.9)
High-income North America	YLDs Rate: 1990	2,729.0 (2,064.5–3,563.1)	808.7 (570.6–1,117.8)	559.2 (402.3–738.1)	0.0 (0.0–0.1)	2.2 (1.6–3.0)	72.4 (51.4–98.9)	156.4 (99.1–225.9)	135.3 (103.9–175.0)	0.0 (0.0–0.0)	18.3 (12.7–24.8)	33.7 (25.6–43.3)
High-income North America	YLDs Rate: 2015	2,744.6 (2,082.4–3,577.7)	781.7 (552.6–1,079.0)	561.6 (404.0–743.9)	0.0 (0.0–0.0)	2.2 (1.6–3.0)	69.6 (48.8–96.2)	256.0 (175.9–344.0)	100.9 (75.5–131.7)	0.0 (0.0–0.0)	17.4 (12.3–23.4)	36.1 (26.5–47.0)
High-income North America	YLDs Rate: Cumulative change (%)	0.6 (-3.5 to 5.2)	-3.1 (-13.3 to 7.1)	0.4 (-0.6 to 1.5)	-45.2 (-55.0 to -35.3)	1.8 (-8.5 to 12.6)	-3.6 (-13.5 to 6.3)	67.4 (31.8–112.6)	-25.5 (-29.3 to -21.8)	-1.2 (-10.8 to 9.5)	-4.4 (-12.2 to 3.8)	7.5 (-6.1 to 22.1)
Canada	Prevalence Number: 1990	1,267,059 (1,221,004–1,318,868)	429,590 (318,357–551,515)	346,700 (330,167–364,908)	4 (2–6)	366 (339–395)	287,821 (273,550–302,672)	83,955 (65,325–106,648)	44,355 (41,064–48,506)	0 (0–0)	2,026 (1,633–2,479)	-
Canada	Prevalence Number: 2015	1,245,310 (1,189,854–1,300,325)	425,191 (282,418–578,241)	355,170 (337,755–374,220)	2 (1–4)	453 (395–516)	208,553 (196,987–220,200)	80,756 (60,672–104,353)	43,669 (40,410–47,572)	0 (0–0)	2,058 (1,650–2,527)	-
Canada	Prevalence Number: Cumulative change (%	-1.7 (-7.3 to 3.8)	1.1 (-30.8 to 46.3)	2.5 (-4.1 to 9.2)	-35.0 (-58.5 to -5.0)	23.7 (12.2–37.0)	-27.5 (-32.7 to -22.2)	-3.6 (-17.6 to 12.5)	-1.4 (-9.5 to 7.7)	1.6 (1.6–1.7)	1.7 (-4.5 to 8.2)	-
Canada	Prevalence Rate: 1990	67,345.0 (64,895.4–70,096.2)	22,819.2 (16,906.8–29,293.1)	18,435.4 (17,556.4–19,403.7)	0.2 (0.1–0.3)	19.5 (18.0–21.0)	15,295.3 (14,536.8–16,084.6)	4,466.5 (3,475.3–5,673.7)	2,350.4 (2,176.2–2,570.7)	0.0 (0.0–0.0)	107.6 (86.7–131.7)	-
Canada	Prevalence Rate: 2015	65,209.3 (62,315.4–68,083.2)	22,289.3 (14,830.7–30,290.0)	18,583.5 (17,673.3–19,580.7)	0.1 (0.1–0.2)	23.8 (20.8–27.1)	10,923.9 (10,318.2–11,533.9)	4,222.5 (3,172.4–5,456.3)	2,297.8 (2,126.6–2,503.1)	0.0 (0.0–0.0)	108.0 (86.6–132.6)	-
Canada	Prevalence Rate: Cumulative change (%	-3.1 (-8.6 to 2.3)	-0.2 (-31.7 to 44.3)	0.9 (-5.7 to 7.5)	-35.4 (-58.8 to -5.6)	22.3 (10.9–35.4)	-28.5 (-33.7 to -23.4)	-5.3 (-19.1 to 10.5)	-2.1 (-10.1 to 6.9)	-0.0 (-0.0 to 0.0)	0.4 (-5.7 to 6.9)	-
Canada	YLDs Number: 1990	49,599 (36,751–65,134)	11,000 (6,390–17,252)	15,806 (11,375–21,019)	0 (0–0)	61 (38–89)	627 (373–975)	3,867 (2,472–5,570)	1,830 (1,369–2,390)	0 (0–0)	378 (246–537)	321 (224–446)
Canada	YLDs Number: 2015	49,289 (35,938–65,585)	10,783 (5,733–18,253)	16,117 (11,714–21,427)	0 (0–0)	75 (47–109)	636 (348–1,037)	3,719 (2,326–5,403)	1,627 (1,200–2,141)	0 (0–0)	372 (245–522)	670 (452–962)
Canada	YLDs Number: Cumulative change (%)	-0.3 (-13.3 to 16.4)	2.6 (-45.1 to 77.8)	2.0 (-1.5 to 5.9)	-34.9 (-58.5 to -4.6)	25.8 (-17.7 to 80.6)	5.8 (-41.1 to 76.7)	-3.6 (-18.5 to 13.2)	-10.9 (-22.1 to 1.6)	2.5 (-41.9 to 80.5)	0.0 (-24.0 to 29.8)	112.2 (45.8–196.2)
Canada	YLDs Rate: 1990	2,636.5 (1,953.6–3,462.8)	584.3 (339.4–916.3)	840.7 (605.0–1,118.0)	0.0 (0.0–0.0)	3.3 (2.1–4.8)	33.3 (19.8–51.8)	205.7 (131.5–296.4)	97.3 (72.8–127.0)	0.0 (0.0–0.0)	20.1 (13.1–28.6)	17.1 (11.9–23.7)
Canada	YLDs Rate: 2015	2,580.7 (1,881.7–3,434.0)	565.5 (301.0–956.3)	843.0 (612.7–1,120.8)	0.0 (0.0–0.0)	3.9 (2.5–5.7)	33.4 (18.3–54.3)	194.5 (121.7–282.5)	85.3 (62.9–112.2)	0.0 (0.0–0.0)	19.5 (12.8–27.4)	35.1 (23.7–50.4)
Canada	YLDs Rate: Cumulative change (%)	-1.8 (-14.6 to 14.6)	1.4 (-45.8 to 75.4)	0.3 (-3.2 to 4.1)	-35.3 (-58.8 to -5.2)	24.3 (-18.6 to 78.4)	4.5 (-41.8 to 74.4)	-5.2 (-19.9 to 11.2)	-12.2 (-23.2 to 0.2)	0.8 (-42.9 to 77.7)	-1.4 (-25.1 to 27.9)	109.2 (43.7–192.0)
Greenland	Prevalence Number: 1990	7,306 (7,016–7,557)	2,680 (2,252–3,164)	1,813 (1,700–1,933)	0 (0–0)	1 (1–2)	1,730 (1,650–1,809)	381 (280–492)	334 (304–367)	0 (0–0)	11 (9–14)	-
Greenland	Prevalence Number: 2015	5,141 (4,942–5,316)	1,782 (1,500–2,033)	1,363 (1,278–1,460)	0 (0–0)	1 (1–1)	952 (906–999)	274 (203–354)	221 (201–243)	0 (0–0)	8 (6–10)	-
Greenland	Prevalence Number: Cumulative change (%	-29.6 (-33.1 to -26.0)	-32.7 (-45.9 to -15.0)	-24.7 (-31.5 to -18.2)	-67.3 (-85.0 to -40.5)	-26.7 (-30.2 to -22.8)	-44.9 (-48.5 to -41.3)	-28.1 (-33.8 to -22.5)	-33.8 (-39.5 to -28.2)	-25.7 (-26.1 to -25.3)	-28.3 (-34.6 to -21.4)	-
Greenland	Prevalence Rate: 1990	68,051.8 (65,335.4–70,406.5)	24,662.5 (20,645.6–29,216.0)	17,042.6 (15,977.2–18,169.4)	0.1 (0.0–0.2)	17.9 (16.6–19.3)	16,089.2 (15,353.0–16,821.7)	3,614.0 (2,661.3–4,670.1)	2,980.7 (2,711.3–3,280.1)	0.0 (0.0–0.0)	104.6 (82.4–131.1)	-
Greenland	Prevalence Rate: 2015	65,287.6 (62,778.0–67,487.6)	22,719.0 (19,167.2–25,899.9)	17,269.8 (16,205.0–18,493.7)	0.0 (0.0–0.1)	18.4 (16.9–20.0)	12,096.3 (11,519.7–12,694.4)	3,465.7 (2,576.4–4,478.6)	2,842.6 (2,592.3–3,127.2)	0.0 (0.0–0.0)	104.0 (84.2–128.0)	-
Greenland	Prevalence Rate: Cumulative change (%	-4.0 (-8.8 to 0.9)	-6.8 (-25.5 to 17.8)	1.5 (-7.6 to 10.3)	-53.1 (-78.4 to -15.6)	3.0 (-1.9 to 8.5)	-24.8 (-29.7 to -19.8)	-4.0 (-11.7 to 3.4)	-4.5 (-12.8 to 3.8)	0.0 (-0.0 to 0.0)	-0.2 (-9.2 to 9.3)	-
Greenland	YLDs Number: 1990	297 (219–386)	71 (44–105)	78 (55–106)	0 (0–0)	0 (0–0)	5 (3–7)	17 (10–25)	20 (14–25)	0 (0–0)	2 (1–3)	3 (2–5)
Greenland	YLDs Number: 2015	202 (149–260)	46 (30–65)	58 (41–79)	0 (0–0)	0 (0–0)	3 (2–4)	12 (7–18)	10 (8–14)	0 (0–0)	1 (0–2)	1 (1–3)
Greenland	YLDs Number: Cumulative change (%)	-31.7 (-37.9 to -24.2)	-33.3 (-52.2 to -6.8)	-25.2 (-28.0 to -22.4)	-67.5 (-85.1 to -40.5)	-25.0 (-45.1 to -0.2)	-30.5 (-49.6 to -0.6)	-28.0 (-35.1 to -20.5)	-45.7 (-51.4 to -39.7)	-25.3 (-50.7 to 2.1)	-29.0 (-45.6 to -6.2)	-41.6 (-68.5 to -7.6)
Greenland	YLDs Rate: 1990	2,766.4 (2,041.8–3,604.8)	652.6 (408.3–960.7)	743.0 (526.9–1,002.2)	0.0 (0.0–0.0)	3.0 (1.9–4.3)	47.8 (30.4–71.2)	166.5 (103.6–245.4)	185.8 (138.0–239.7)	0.0 (0.0–0.0)	20.3 (13.1–29.3)	33.8 (18.8–55.7)
Greenland	YLDs Rate: 2015	2,571.5 (1,902.0–3,307.0)	591.5 (384.0–830.3)	745.5 (529.1–1,009.8)	0.0 (0.0–0.0)	3.1 (2.1–4.5)	45.1 (29.0–63.3)	159.8 (99.6–234.3)	137.9 (103.5–183.2)	0.0 (0.0–0.0)	19.4 (12.7–27.8)	25.3 (15.8–39.0)
Greenland	YLDs Rate: Cumulative change (%)	-6.8 (-15.3 to 3.2)	-6.9 (-33.4 to 29.3)	0.4 (-3.5 to 4.1)	-53.3 (-78.5 to -15.8)	5.5 (-23.0 to 40.8)	-3.0 (-29.9 to 38.5)	-3.9 (-13.4 to 6.1)	-25.7 (-33.5 to -17.3)	0.8 (-33.8 to 37.3)	-3.0 (-25.8 to 28.5)	-20.0 (-56.8 to 26.6)
United States	Prevalence Number: 1990	13,374,443 (13,260,268–13,486,385)	5,564,141 (5,283,723–5,836,609)	2,759,954 (2,743,694–2,778,151)	65 (44–92)	2,384 (2,193–2,593)	4,096,773 (4,049,073–4,141,798)	630,442 (476,603–796,777)	537,176 (514,566–559,501)	0 (0–0)	18,408 (14,889–22,390)	-
United States	Prevalence Number: 2015	13,716,207 (13,578,050–13,863,324)	5,611,403 (5,330,272–5,925,579)	2,915,844 (2,896,813–2,935,497)	36 (23–52)	2,443 (2,209–2,696)	3,357,156 (3,320,304–3,396,013)	1,144,213 (1,082,310–1,203,219)	510,394 (479,109–540,894)	0 (0–0)	18,892 (15,776–22,439)	-

© 2017 Global Burden of Disease Child and Adolescent Health Collaboration. JAMA Pediatrics.

eTable 4. Prevalent cases, Rates (per 100,000 population), Years Lived with Disability (YLDs), and Cumulative Percent Change with 95% Uncertainty Interval (UI) for the Top 10 Global Causes of YLDsin Children and Adolescents in 195 Countries and Territories, Aged Under 5 Years, Both Sexes, 1990 and 2015. *Note: best viewed by enlarging in browser.*

Location	Measure	All causes	Iron-deficiency anemia	Skin and subcutaneous diseases	Protein-energy malnutrition	Diarrheal diseases	Hemoglobinopathies and hemolytic anemias	Asthma	Neonatal preterm birth complications	Malaria	Neonatal encephalopathy due to birth asphyxia and trauma	Other neonatal disorders
United States	Prevalence Number: Cumulative change (%	2.6 (1.3–3.8)	0.9 (-6.3 to 8.1)	5.6 (4.9–6.4)	-44.2 (-54.3 to -33.9)	2.4 (-1.6 to 6.6)	-18.1 (-19.0 to -17.1)	86.0 (43.9–138.5)	-5.0 (-9.1 to -0.9)	4.7 (4.6–4.9)	2.9 (-1.9 to 7.7)	-
United States	Prevalence Rate: 1990	69,583.5 (68,987.5–70,167.7)	28,909.7 (27,447.1–30,331.9)	14,372.8 (14,288.1–14,467.6)	0.3 (0.2–0.5)	12.4 (11.4–13.5)	21,311.8 (21,063.6–21,546.0)	3,287.5 (2,485.3–4,154.9)	2,778.3 (2,661.1–2,894.1)	0.0 (0.0–0.0)	95.6 (77.2–116.3)	-
United States	Prevalence Rate: 2015	68,419.7 (67,728.5–69,152.8)	28,067.3 (26,673.1–29,631.0)	14,519.5 (14,424.8–14,617.3)	0.2 (0.1–0.3)	12.3 (11.1–13.5)	16,751.9 (16,568.1–16,945.7)	5,684.4 (5,376.8–5,977.5)	2,575.2 (2,417.4–2,729.5)	0.0 (0.0–0.0)	94.6 (79.1–112.4)	-
United States	Prevalence Rate: Cumulative change (%	-1.7 (-2.9 to -0.5)	-2.8 (-9.7 to 4.1)	1.0 (0.3–1.7)	-45.6 (-55.4 to -35.5)	-1.0 (-4.9 to 3.0)	-21.4 (-22.3 to -20.4)	77.2 (37.1–127.2)	-7.3 (-11.3 to -3.3)	0.0 (-0.0 to 0.0)	-0.8 (-5.4 to 3.9)	-
United States	YLDs Number: 1990	526,368 (398,662–686,502)	160,003 (113,082–221,356)	102,019 (73,344–135,378)	8 (4–12)	399 (284–541)	14,682 (10,414–20,187)	29,055 (18,379–42,295)	26,727 (20,495–34,396)	0 (0–0)	3,473 (2,439–4,718)	6,781 (5,179–8,721)
United States	YLDs Number: 2015	553,198 (417,479–720,665)	160,207 (112,485–220,989)	107,501 (77,211–142,775)	4 (2–7)	408 (286–550)	14,595 (10,256–20,188)	52,723 (36,371–71,233)	20,526 (15,363–26,811)	0 (0–0)	3,444 (2,445–4,651)	7,259 (5,343–9,433)
United States	YLDs Number: Cumulative change (%)	5.1 (0.4–10.2)	0.3 (-10.0 to 11.4)	5.4 (4.2–6.5)	-44.4 (-54.4 to -34.2)	2.5 (-7.2 to 12.2)	-0.4 (-10.6 to 10.4)	85.9 (44.0–138.5)	-23.3 (-27.4 to -19.2)	3.4 (-6.6 to 14.8)	-0.5 (-8.9 to 8.5)	7.2 (-7.2 to 22.0)
United States	YLDs Rate: 1990	2,737.9 (2,073.6–3,571.1)	830.8 (587.0–1,149.5)	531.5 (382.1–705.3)	0.0 (0.0–0.1)	2.1 (1.5–2.8)	76.2 (54.1–104.8)	151.5 (95.8–220.6)	139.0 (106.6–178.9)	0.0 (0.0–0.0)	18.1 (12.7–24.5)	35.3 (26.9–45.4)
United States	YLDs Rate: 2015	2,760.5 (2,083.0–3,596.6)	802.5 (563.4–1,106.9)	534.8 (384.1–710.4)	0.0 (0.0–0.0)	2.0 (1.4–2.8)	73.1 (51.4–101.1)	261.9 (180.7–353.9)	102.4 (76.7–133.8)	0.0 (0.0–0.0)	17.2 (12.2–23.2)	36.2 (26.7–47.1)
United States	YLDs Rate: Cumulative change (%)	0.9 (-3.6 to 5.7)	-3.2 (-13.2 to 7.5)	0.6 (-0.5 to 1.7)	-45.8 (-55.5 to -35.8)	-1.0 (-10.4 to 8.3)	-3.9 (-13.8 to 6.6)	77.1 (37.2–127.2)	-26.4 (-30.4 to -22.5)	-1.2 (-10.8 to 9.7)	-4.5 (-12.6 to 4.0)	2.8 (-10.9 to 17.1)
Australasia	Prevalence Number: 1990	894,473 (849,127–941,090)	287,699 (221,649–388,157)	288,839 (275,789–301,870)	3 (2–5)	825 (750–909)	62,980 (57,602–68,984)	89,478 (71,309–109,199)	27,085 (25,106–29,275)	0 (0–0)	1,887 (1,540–2,282)	-
Australasia	Prevalence Number: 2015	1,096,780 (1,041,830–1,146,696)	419,347 (306,314–533,459)	360,363 (343,988–377,976)	2 (1–4)	1,160 (1,026–1,298)	69,179 (62,698–75,644)	71,658 (54,183–90,785)	30,812 (28,520–33,288)	0 (0–0)	1,821 (1,481–2,226)	-
Australasia	Prevalence Number: Cumulative change (%	22.7 (14.4–31.1)	50.8 (-5.4 to 118.6)	24.8 (17.8–32.3)	-21.6 (-46.5 to 11.6)	40.7 (32.0–51.3)	10.2 (-3.7 to 23.9)	-20.0 (-29.9 to -9.2)	13.9 (6.5–21.4)	23.7 (23.6–23.8)	-3.4 (-9.7 to 2.5)	-
Australasia	Prevalence Rate: 1990	59,492.7 (56,466.8–62,599.0)	19,091.7 (14,700.7–25,798.5)	19,227.9 (18,360.6–20,094.8)	0.2 (0.1–0.3)	54.7 (49.8–60.3)	4,186.4 (3,828.7–4,585.3)	5,967.0 (4,755.4–7,282.2)	1,788.6 (1,657.7–1,934.1)	0.0 (0.0–0.0)	125.2 (102.2–151.4)	-
Australasia	Prevalence Rate: 2015	59,076.4 (56,119.7–61,764.4)	22,601.4 (16,514.9–28,741.8)	19,403.8 (18,522.1–20,351.8)	0.1 (0.1–0.2)	62.6 (55.3–70.0)	3,726.8 (3,377.8–4,075.0)	3,857.0 (2,916.4–4,886.4)	1,662.8 (1,539.2–1,796.5)	0.0 (0.0–0.0)	98.2 (79.8–120.0)	-
Australasia	Prevalence Rate: Cumulative change (%	-0.6 (-7.4 to 6.2)	22.5 (-23.2 to 77.5)	1.0 (-4.8 to 7.0)	-36.0 (-56.4 to -8.9)	14.3 (7.3–22.9)	-10.7 (-22.0 to 0.4)	-35.4 (-43.5 to -26.7)	-7.0 (-13.1 to -0.7)	-0.0 (-0.0 to 0.0)	-21.5 (-26.6 to -16.7)	-
Australasia	YLDs Number: 1990	35,738 (26,404–47,283)	7,247 (4,388–12,244)	8,254 (5,816–11,128)	0 (0–0)	137 (93–188)	467 (280–729)	4,134 (2,683–5,811)	1,432 (1,054–1,895)	0 (0–0)	354 (239–493)	136 (96–186)
Australasia	YLDs Number: 2015	44,034 (31,687–57,964)	10,950 (6,009–17,609)	10,245 (7,272–13,801)	0 (0–0)	192 (128–270)	623 (367–962)	3,306 (2,092–4,715)	1,401 (1,034–1,848)	0 (0–0)	334 (228–474)	648 (444–911)
Australasia	YLDs Number: Cumulative change (%)	23.7 (3.4–46.3)	61.3 (-16.7 to 180.8)	24.2 (19.4–28.8)	-21.5 (-46.6 to 12.0)	41.5 (8.8–79.8)	39.4 (-17.7 to 115.1)	-20.0 (-30.8 to -8.6)	-2.0 (-13.0 to 10.0)	34.1 (-24.9 to 104.1)	-5.0 (-24.5 to 17.4)	382.5 (246.8–549.3)
Australasia	YLDs Rate: 1990	2,377.2 (1,755.8–3,144.4)	480.6 (290.8–814.5)	549.9 (387.5–741.4)	0.0 (0.0–0.0)	9.1 (6.2–12.5)	31.0 (18.6–48.5)	275.7 (179.0–387.5)	95.2 (70.1–126.0)	0.0 (0.0–0.0)	23.6 (15.9–32.9)	9.1 (6.4–12.4)
Australasia	YLDs Rate: 2015	2,372.0 (1,706.9–3,121.9)	590.4 (324.2–949.1)	551.6 (391.5–743.0)	0.0 (0.0–0.0)	10.4 (6.9–14.6)	33.6 (19.8–51.9)	177.9 (112.7–253.8)	75.5 (55.7–99.6)	0.0 (0.0–0.0)	18.0 (12.3–25.5)	35.0 (23.9–49.1)
Australasia	YLDs Rate: Cumulative change (%)	0.2 (-16.3 to 18.4)	31.2 (-32.4 to 128.1)	0.3 (-3.6 to 4.1)	-35.9 (-56.4 to -8.6)	15.0 (-11.7 to 46.1)	13.4 (-33.3 to 74.9)	-35.4 (-44.2 to -26.3)	-20.6 (-29.5 to -10.9)	8.4 (-39.3 to 65.0)	-23.0 (-38.8 to -4.9)	291.0 (181.1–426.1)
Australia	Prevalence Number: 1990	738,077 (695,087–784,134)	246,601 (183,160–351,126)	236,625 (224,235–249,192)	3 (2–4)	670 (607–744)	53,204 (47,973–59,029)	74,272 (59,297–90,590)	21,679 (20,042–23,538)	0 (0–0)	1,297 (1,060–1,579)	-
Australia	Prevalence Number: 2015	917,846 (864,782–968,085)	363,355 (249,633–474,722)	301,349 (285,558–317,600)	2 (1–3)	975 (855–1,092)	55,905 (49,544–62,283)	59,845 (45,039–76,275)	25,375 (23,452–27,603)	0 (0–0)	1,170 (947–1,434)	-
Australia	Prevalence Number: Cumulative change (%	24.5 (14.8–34.8)	54.0 (-10.9 to 128.5)	27.5 (19.3–36.6)	-19.6 (-46.0 to 16.5)	45.6 (34.8–58.6)	5.5 (-10.4 to 21.1)	-19.4 (-30.8 to -7.3)	17.2 (8.5–26.6)	26.2 (26.1–26.3)	-9.7 (-17.7 to -2.1)	-
Australia	Prevalence Rate: 1990	59,979.9 (56,477.9–63,731.1)	20,007.5 (14,850.8–28,524.7)	19,241.9 (18,234.4–20,264.1)	0.3 (0.2–0.4)	54.4 (49.3–60.4)	4,321.7 (3,896.8–4,794.7)	6,048.0 (4,828.5–7,376.7)	1,752.1 (1,619.4–1,902.5)	0.0 (0.0–0.0)	105.3 (86.0–128.1)	-
Australia	Prevalence Rate: 2015	59,150.4 (55,729.0–62,390.5)	23,408.6 (16,080.6–30,595.0)	19,423.3 (18,405.4–20,470.7)	0.2 (0.1–0.3)	62.9 (55.1–70.3)	3,602.5 (3,192.3–4,013.7)	3,859.0 (2,904.2–4,918.4)	1,632.8 (1,509.1–1,776.4)	0.0 (0.0–0.0)	75.4 (61.1–92.4)	-
Australia	Prevalence Rate: Cumulative change (%	-1.2 (-8.9 to 6.9)	22.3 (-29.3 to 81.4)	1.0 (-5.5 to 8.3)	-36.0 (-57.1 to -7.4)	15.6 (7.0–25.9)	-16.3 (-28.9 to -3.9)	-36.2 (-45.2 to -26.6)	-6.7 (-13.6 to 0.9)	-0.0 (-0.0 to 0.0)	-28.3 (-34.6 to -22.3)	-
Australia	YLDs Number: 1990	29,401 (21,626–39,286)	6,222 (3,699–10,943)	6,769 (4,772–9,134)	0 (0–0)	111 (74–157)	319 (186–552)	3,431 (2,229–4,834)	1,161 (856–1,550)	0 (0–0)	244 (160–348)	115 (79–160)
Australia	YLDs Number: 2015	37,064 (26,420–49,112)	9,551 (4,899–15,923)	8,566 (6,069–11,518)	0 (0–0)	161 (105–228)	445 (226–731)	2,761 (1,739–3,944)	1,156 (849–1,528)	0 (0–0)	215 (139–309)	565 (381–803)
Australia	YLDs Number: Cumulative change (%)	26.8 (2.6–54.5)	66.8 (-21.8 to 207.7)	26.6 (20.4–32.5)	-19.4 (-45.9 to 16.8)	46.9 (7.7–93.0)	50.5 (-25.3 to 160.1)	-19.4 (-31.4 to -6.2)	-0.1 (-13.4 to 14.3)	39.2 (-30.2 to 129.6)	-10.8 (-35.2 to 19.0)	399.3 (240.9–594.3)
Australia	YLDs Rate: 1990	2,389.5 (1,757.3–3,192.3)	504.6 (299.4–889.7)	550.9 (388.4–743.5)	0.0 (0.0–0.1)	9.0 (6.0–12.8)	25.9 (15.1–44.8)	279.4 (181.5–393.6)	94.4 (69.6–126.0)	0.0 (0.0–0.0)	19.9 (13.0–28.3)	9.4 (6.4–13.1)
Australia	YLDs Rate: 2015	2,388.7 (1,702.5–3,165.2)	615.3 (315.6–1,025.8)	552.3 (391.3–742.6)	0.0 (0.0–0.0)	10.4 (6.8–14.7)	28.7 (14.6–47.1)	178.0 (112.2–254.3)	74.6 (54.7–98.5)	0.0 (0.0–0.0)	13.9 (9.0–19.9)	36.4 (24.6–51.8)
Australia	YLDs Rate: Cumulative change (%)	0.6 (-18.7 to 22.5)	32.6 (-38.0 to 144.6)	0.3 (-4.6 to 5.0)	-35.9 (-57.0 to -7.1)	16.7 (-14.5 to 53.4)	19.6 (-40.8 to 106.7)	-36.2 (-45.7 to -25.7)	-20.8 (-31.3 to -9.3)	10.3 (-44.7 to 82.1)	-29.3 (-48.7 to -5.6)	296.1 (170.5–450.9)
New Zealand	Prevalence Number: 1990	156,395 (147,199–165,785)	41,098 (24,998–58,228)	52,213 (49,323–55,154)	0 (0–0)	154 (138–171)	9,776 (8,466–10,945)	15,205 (11,782–18,750)	5,406 (4,946–5,901)	0 (0–0)	589 (479–716)	-
New Zealand	Prevalence Number: 2015	178,933 (169,175–188,350)	55,991 (44,066–68,877)	59,013 (55,621–62,257)	0 (0–0)	184 (165–208)	13,273 (12,146–14,338)	11,813 (8,575–15,439)	5,436 (4,977–5,949)	0 (0–0)	651 (528–799)	-
New Zealand	Prevalence Number: Cumulative change (%	14.5 (6.4–23.6)	43.4 (-0.8 to 112.9)	13.1 (4.4–21.6)	-46.0 (-68.3 to -11.3)	19.6 (13.7–26.8)	36.6 (18.8–60.3)	-22.3 (-35.0 to -7.7)	0.7 (-6.9 to 8.6)	12.5 (12.1–12.9)	10.6 (3.7–17.9)	-
New Zealand	Prevalence Rate: 1990	57,291.3 (53,925.0–60,730.0)	14,956.1 (9,097.5–21,199.3)	19,164.7 (18,101.6–20,245.1)	0.1 (0.1–0.1)	56.3 (50.6–62.6)	3,575.6 (3,096.6–4,002.0)	5,600.7 (4,340.0–6,906.8)	1,951.9 (1,785.1–2,133.1)	0.0 (0.0–0.0)	214.9 (174.5–261.4)	-
New Zealand	Prevalence Rate: 2015	58,705.7 (55,524.3–61,787.7)	18,514.6 (14,570.1–22,780.5)	19,304.9 (18,197.8–20,368.6)	0.0 (0.0–0.1)	60.9 (54.8–68.6)	4,362.0 (3,991.0–4,714.0)	3,846.8 (2,792.4–5,027.3)	1,819.3 (1,666.7–1,989.1)	0.0 (0.0–0.0)	215.4 (174.7–263.9)	-
New Zealand	Prevalence Rate: Cumulative change (%	2.6 (-4.7 to 10.7)	30.3 (-9.9 to 94.2)	0.8 (-6.9 to 8.4)	-49.9 (-70.7 to -17.8)	8.3 (3.0–15.0)	22.7 (6.7–44.1)	-31.3 (-42.6 to -18.4)	-6.7 (-13.8 to 0.5)	-0.0 (-0.0 to 0.0)	0.3 (-6.0 to 7.1)	-
New Zealand	YLDs Number: 1990	6,336 (4,559–8,339)	1,024 (452–1,777)	1,484 (1,056–1,991)	0 (0–0)	25 (17–35)	148 (69–256)	703 (454–1,001)	270 (200–357)	0 (0–0)	109 (74–151)	20 (14–29)
New Zealand	YLDs Number: 2015	6,970 (5,134–9,041)	1,399 (828–2,098)	1,678 (1,185–2,270)	0 (0–0)	30 (20–43)	177 (106–267)	544 (333–810)	244 (178–325)	0 (0–0)	119 (80–168)	83 (55–119)
New Zealand	YLDs Number: Cumulative change (%)	10.4 (-2.7 to 24.9)	54.0 (-17.9 to 188.5)	13.1 (8.6–17.7)	-45.9 (-68.3 to -10.8)	21.7 (-9.6 to 56.7)	30.9 (-22.4 to 140.2)	-22.5 (-35.5 to -7.1)	-9.5 (-19.1 to 1.0)	25.8 (-18.2 to 93.1)	9.3 (-7.5 to 29.5)	305.6 (173.9–466.6)
New Zealand	YLDs Rate: 1990	2,321.1 (1,672.3–3,054.3)	371.9 (164.5–645.0)	545.7 (388.4–731.9)	0.0 (0.0–0.0)	9.4 (6.2–13.0)	54.1 (25.1–93.5)	259.0 (167.3–368.7)	99.1 (73.2–130.7)	0.0 (0.0–0.0)	40.1 (27.4–55.5)	7.7 (5.3–10.8)

© 2017 Global Burden of Disease Child and Adolescent Health Collaboration. JAMA Pediatrics.

eTable 4. Prevalent cases, Rates (per 100,000 population), Years Lived with Disability (YLDs), and Cumulative Percent Change with 95% Uncertainty Interval (UI) for the Top 10 Global Causes of YLDsin Children and Adolescents in 195 Countries and Territories, Aged Under 5 Years, Both Sexes, 1990 and 2015. *Note: best viewed by enlarging in browser.*

Location	Measure	All causes	Iron-deficiency anemia	Skin and subcutaneous diseases	Protein-energy malnutrition	Diarrheal diseases	Hemoglobinopathies and hemolytic anemias	Asthma	Neonatal preterm birth complications	Malaria	Neonatal encephalopathy due to birth asphyxia and trauma	Other neonatal disorders
New Zealand	YLDs Rate: 2015	2,287.9 (1,685.7–2,968.7)	464.2 (275.2–694.6)	548.0 (386.8–741.6)	0.0 (0.0–0.0)	10.2 (6.8–14.3)	58.6 (35.3–88.5)	177.4 (108.6–263.8)	80.4 (58.5–106.8)	0.0 (0.0–0.0)	39.2 (26.5–55.4)	27.4 (18.3–39.3)
New Zealand	YLDs Rate: Cumulative change (%)	-1.1 (-12.7 to 11.8)	40.6 (-24.5 to 162.9)	0.4 (-3.6 to 4.5)	-49.8 (-70.8 to -17.2)	10.3 (-18.1 to 42.1)	19.3 (-29.3 to 118.9)	-31.5 (-43.0 to -17.9)	-18.8 (-27.4 to -9.5)	12.0 (-27.4 to 72.0)	-2.0 (-17.0 to 16.0)	264.0 (145.9–408.0)
High-income Asia Pacific	Prevalence Number: 1990	6,450,297 (6,343,637–6,554,216)	503,636 (473,801–536,545)	1,997,192 (1,953,703–2,040,705)	15 (10–23)	7,222 (6,586–8,047)	367,940 (354,599–380,932)	463,651 (368,863–567,695)	162,271 (150,869–173,907)	194 (87–346)	9,514 (7,687–11,557)	-
High-income Asia Pacific	Prevalence Number: 2015	4,792,041 (4,713,901–4,868,786)	333,263 (310,230–357,474)	1,518,590 (1,488,218–1,550,854)	11 (7–17)	3,894 (3,583–4,250)	269,008 (259,664–278,610)	185,287 (138,794–237,624)	109,490 (101,705–117,167)	62 (37–119)	7,204 (5,761–8,842)	-
High-income Asia Pacific	Prevalence Number: Cumulative change (%	-25.7 (-27.1 to -24.4)	-33.7 (-39.6 to -27.4)	-24.0 (-25.7 to -22.1)	-25.3 (-45.4 to -4.9)	-46.0 (-48.5 to -44.0)	-26.9 (-29.8 to -23.9)	-60.1 (-65.0 to -55.3)	-32.5 (-35.1 to -29.9)	-64.5 (-83.1 to -37.0)	-24.3 (-27.4 to -21.2)	-
High-income Asia Pacific	Prevalence Rate: 1990	62,159.5 (61,141.7–63,152.8)	4,968.9 (4,676.5–5,287.3)	19,212.0 (18,794.1–19,630.8)	0.2 (0.1–0.2)	70.2 (64.2–78.2)	3,562.7 (3,433.5–3,688.6)	4,436.7 (3,529.7–5,432.3)	1,617.7 (1,503.6–1,731.5)	1.9 (0.8–3.3)	92.9 (75.1–112.5)	-
High-income Asia Pacific	Prevalence Rate: 2015	61,556.8 (60,553.7–62,537.9)	4,346.7 (4,051.1–4,656.4)	19,489.3 (19,100.3–19,903.1)	0.2 (0.1–0.2)	50.3 (46.3–54.9)	3,466.2 (3,346.3–3,589.6)	2,370.4 (1,775.6–3,039.9)	1,435.5 (1,333.9–1,536.1)	0.8 (0.5–1.5)	93.3 (74.7–114.5)	-
High-income Asia Pacific	Prevalence Rate: Cumulative change (%	-1.0 (-2.8 to 0.8)	-12.4 (-20.1 to -4.2)	1.5 (-0.9 to 3.9)	-1.9 (-28.3 to 24.9)	-28.3 (-31.5 to -25.6)	-2.7 (-6.6 to 1.3)	-46.6 (-53.2 to -40.2)	-11.2 (-14.6 to -7.9)	-52.6 (-77.4 to -15.9)	0.4 (-3.7 to 4.5)	-
High-income Asia Pacific	YLDs Number: 1990	211,212 (158,914–273,718)	10,709 (7,295–15,030)	67,597 (47,926–90,267)	1 (1–3)	1,205 (856–1,638)	3,628 (2,519–4,957)	21,438 (14,066–30,005)	6,367 (4,835–8,218)	3 (1–7)	1,817 (1,243–2,456)	1,502 (1,131–1,944)
High-income Asia Pacific	YLDs Number: 2015	144,020 (109,690–185,487)	6,760 (4,531–9,565)	51,068 (36,163–68,428)	1 (0–2)	650 (463–878)	2,794 (1,948–3,797)	8,567 (5,385–12,526)	3,688 (2,723–4,866)	1 (0–2)	1,313 (892–1,823)	2,073 (1,476–2,721)
High-income Asia Pacific	YLDs Number: Cumulative change (%)	-31.8 (-33.8 to -29.6)	-36.7 (-44.3 to -28.9)	-24.4 (-26.4 to -22.6)	-25.5 (-45.6 to -5.3)	-45.9 (-51.7 to -39.5)	-22.9 (-29.4 to -16.1)	-60.1 (-64.8 to -55.1)	-42.1 (-47.1 to -36.7)	-68.5 (-87.8 to -37.5)	-27.7 (-35.7 to -19.2)	38.1 (16.7–59.9)
High-income Asia Pacific	YLDs Rate: 1990	2,036.7 (1,532.0–2,639.1)	106.3 (72.4–149.2)	648.5 (459.5–866.0)	0.0 (0.0–0.0)	11.7 (8.3–15.9)	35.5 (24.6–48.4)	205.1 (134.6–287.1)	61.6 (46.8–79.6)	0.0 (0.0–0.1)	17.6 (12.0–23.8)	14.6 (11.0–18.8)
High-income Asia Pacific	YLDs Rate: 2015	1,851.2 (1,409.7–2,382.9)	88.5 (59.4–125.3)	654.3 (463.3–876.8)	0.0 (0.0–0.0)	8.4 (6.0–11.3)	36.2 (25.3–49.2)	109.6 (68.9–160.2)	47.5 (35.1–62.7)	0.0 (0.0–0.0)	16.9 (11.5–23.5)	26.7 (19.0–35.1)
High-income Asia Pacific	YLDs Rate: Cumulative change (%)	-9.1 (-11.7 to -6.1)	-16.5 (-26.5 to -6.2)	0.9 (-1.7 to 3.4)	-2.2 (-28.5 to 24.5)	-28.1 (-35.8 to -19.8)	2.4 (-6.1 to 11.2)	-46.6 (-52.9 to -40.0)	-22.9 (-29.5 to -15.8)	-58.0 (-83.7 to -16.6)	-3.7 (-14.3 to 7.4)	83.8 (55.3–112.7)
Brunei	Prevalence Number: 1990	23,723 (22,897–24,517)	2,505 (1,961–3,149)	7,117 (6,714–7,575)	0 (0–0)	26 (24–30)	1,866 (1,722–2,024)	1,779 (1,321–2,331)	540 (489–598)	67 (33–158)	34 (27–41)	-
Brunei	Prevalence Number: 2015	20,661 (19,816–21,484)	1,489 (993–1,946)	6,379 (6,015–6,756)	0 (0–0)	19 (17–21)	1,243 (1,137–1,357)	1,408 (1,036–1,840)	471 (427–521)	54 (31–105)	30 (24–36)	-
Brunei	Prevalence Number: Cumulative change (%	-12.9 (-17.1 to -8.6)	-39.8 (-60.9 to -16.8)	-10.3 (-17.0 to -3.6)	-25.0 (-59.8 to 20.0)	-28.6 (-32.8 to -24.7)	-33.2 (-40.3 to -25.5)	-20.8 (-27.5 to -13.9)	-12.6 (-20.0 to -5.1)	-15.2 (-38.2 to 5.1)	-11.9 (-17.1 to -6.6)	-
Brunei	Prevalence Rate: 1990	62,834.3 (60,617.8–64,966.7)	6,523.3 (5,085.2–8,235.7)	18,877.3 (17,810.2–20,091.3)	0.2 (0.1–0.3)	70.4 (63.0–79.7)	4,924.1 (4,540.9–5,340.2)	4,745.6 (3,523.4–6,217.8)	1,387.8 (1,254.7–1,537.4)	178.9 (88.7–422.4)	89.7 (72.5–109.3)	-
Brunei	Prevalence Rate: 2015	61,347.8 (58,849.3–63,768.5)	4,491.7 (3,007.8–5,857.5)	18,923.1 (17,844.6–20,040.3)	0.1 (0.1–0.2)	57.1 (51.5–63.6)	3,704.7 (3,387.6–4,041.8)	4,165.0 (3,063.3–5,440.7)	1,430.1 (1,294.6–1,581.1)	160.3 (92.5–313.0)	90.4 (73.7–109.4)	-
Brunei	Prevalence Rate: Cumulative change (%	-2.3 (-7.1 to 2.4)	-30.4 (-54.1 to -4.7)	0.3 (-7.1 to 7.9)	-13.1 (-53.6 to 39.3)	-18.8 (-23.6 to -14.2)	-24.6 (-32.6 to -15.9)	-12.2 (-19.7 to -4.6)	3.2 (-5.6 to 12.0)	-5.3 (-31.1 to 17.5)	1.0 (-5.2 to 7.0)	-
Brunei	YLDs Number: 1990	841 (624–1,076)	53 (31–80)	230 (164–309)	0 (0–0)	4 (2–6)	6 (3–9)	82 (50–121)	21 (15–28)	1 (0–3)	6 (4–9)	7 (2–16)
Brunei	YLDs Number: 2015	704 (526–896)	30 (15–49)	208 (147–277)	0 (0–0)	3 (2–4)	3 (1–5)	65 (39–97)	18 (13–24)	0 (0–2)	5 (3–7)	7 (3–14)
Brunei	YLDs Number: Cumulative change (%)	-16.2 (-23.8 to -8.5)	-40.9 (-69.1 to -12.0)	-9.9 (-13.2 to -6.3)	-24.9 (-59.7 to 20.3)	-27.5 (-45.3 to -5.9)	-42.7 (-68.5 to -15.3)	-20.6 (-28.3 to -12.7)	-10.4 (-23.0 to 4.4)	-18.2 (-62.1 to 36.1)	-13.1 (-35.5 to 13.6)	38.7 (-64.1 to 236.1)
Brunei	YLDs Rate: 1990	2,226.0 (1,652.0–2,848.2)	138.1 (81.5–209.3)	613.8 (436.9–822.4)	0.0 (0.0–0.0)	11.7 (7.8–16.5)	16.6 (9.9–25.3)	219.2 (135.7–323.9)	55.9 (40.7–74.8)	3.4 (1.2–8.4)	17.0 (11.1–24.2)	20.1 (6.6–44.0)
Brunei	YLDs Rate: 2015	2,092.5 (1,565.1–2,661.4)	92.8 (48.6–150.4)	616.0 (438.1–823.5)	0.0 (0.0–0.0)	9.5 (6.3–13.5)	10.8 (5.7–17.6)	192.6 (118.2–288.3)	56.3 (41.2–74.2)	2.8 (1.0–6.1)	16.4 (10.5–23.4)	22.9 (9.8–41.7)
Brunei	YLDs Rate: Cumulative change (%)	-5.9 (-14.4 to 2.7)	-31.2 (-63.4 to 1.5)	0.4 (-3.3 to 4.4)	-13.0 (-53.4 to 39.6)	-17.4 (-37.7 to 7.0)	-33.7 (-62.9 to -3.1)	-12.0 (-20.6 to -3.2)	1.2 (-13.1 to 17.8)	-8.5 (-57.5 to 51.9)	-1.9 (-27.2 to 28.5)	56.8 (-59.3 to 280.2)
Japan	Prevalence Number: 1990	4,306,261 (4,260,606–4,347,962)	461,421 (433,098–492,881)	1,359,674 (1,341,615–1,377,785)	7 (5–10)	4,233 (3,868–4,649)	245,645 (240,474–250,673)	319,791 (244,628–401,238)	94,385 (88,035–100,582)	0 (0–0)	6,104 (4,937–7,464)	-
Japan	Prevalence Number: 2015	3,331,022 (3,291,230–3,368,138)	306,574 (283,961–330,266)	1,065,926 (1,052,222–1,079,399)	11 (7–16)	2,863 (2,614–3,149)	187,836 (183,798–192,052)	118,450 (89,448–153,653)	70,426 (65,841–74,997)	0 (0–0)	4,901 (3,918–6,035)	-
Japan	Prevalence Number: Cumulative change (%	-22.6 (-23.6 to -21.7)	-33.5 (-39.5 to -26.9)	-21.6 (-22.8 to -20.4)	46.6 (15.4–79.8)	-32.4 (-33.4 to -31.4)	-23.5 (-25.3 to -21.7)	-62.9 (-67.6 to -57.6)	-25.4 (-27.0 to -23.7)	-16.4 (-35.5 to 13.9)	-19.8 (-23.3 to -16.5)	-
Japan	Prevalence Rate: 1990	63,700.4 (63,035.6–64,318.2)	7,033.5 (6,606.7–7,513.8)	20,050.4 (19,785.1–20,316.3)	0.1 (0.1–0.2)	63.3 (57.9–69.4)	3,656.3 (3,579.2–3,731.3)	4,684.6 (3,583.6–5,877.7)	1,458.8 (1,360.9–1,554.4)	0.0 (0.0–0.0)	91.9 (74.4–112.3)	-
Japan	Prevalence Rate: 2015	63,114.6 (62,364.5–63,813.4)	5,909.6 (5,478.0–6,365.2)	20,167.7 (19,908.8–20,422.4)	0.2 (0.1–0.3)	54.6 (49.9–60.0)	3,572.0 (3,495.1–3,651.8)	2,232.9 (1,686.2–2,896.5)	1,367.1 (1,277.7–1,454.7)	0.0 (0.0–0.0)	93.8 (74.9–115.3)	-
Japan	Prevalence Rate: Cumulative change (%	-0.9 (-2.1 to 0.3)	-15.9 (-23.3 to -7.7)	0.6 (-0.9 to 2.1)	84.6 (45.3–126.2)	-13.7 (-15.0 to -12.4)	-2.3 (-4.6 to 0.1)	-52.3 (-58.3 to -45.4)	-6.3 (-8.3 to -4.3)	7.3 (-17.2 to 46.2)	2.0 (-2.6 to 6.1)	-
Japan	YLDs Number: 1990	142,967 (107,122–185,576)	9,737 (6,595–13,668)	47,757 (33,730–63,954)	0 (0–1)	705 (500–966)	3,471 (2,409–4,743)	14,785 (9,519–21,130)	3,602 (2,707–4,723)	0 (0–0)	1,150 (788–1,567)	1,080 (823–1,390)
Japan	YLDs Number: 2015	102,488 (77,867–131,988)	6,147 (4,122–8,679)	37,205 (26,394–50,075)	1 (0–2)	477 (338–647)	2,699 (1,882–3,673)	5,473 (3,471–8,002)	2,376 (1,756–3,137)	0 (0–0)	893 (596–1,235)	1,558 (1,118–2,050)
Japan	YLDs Number: Cumulative change (%)	-28.3 (-30.6 to -25.7)	-36.7 (-44.9 to -28.5)	-22.1 (-24.6 to -19.8)	46.3 (15.2–79.2)	-32.2 (-38.3 to -25.5)	-22.1 (-28.9 to -15.0)	-62.9 (-67.6 to -57.5)	-34.0 (-39.4 to -28.5)	-16.0 (-37.0 to 15.5)	-22.4 (-29.5 to -14.4)	44.4 (23.8–66.6)
Japan	YLDs Rate: 1990	2,116.5 (1,587.1–2,748.0)	149.5 (101.6–210.0)	701.8 (495.4–939.4)	0.0 (0.0–0.0)	10.6 (7.5–14.5)	52.2 (36.2–71.2)	216.6 (139.5–309.5)	53.6 (40.2–70.2)	0.0 (0.0–0.0)	17.1 (11.7–23.3)	16.1 (12.3–20.7)
Japan	YLDs Rate: 2015	1,943.4 (1,476.8–2,503.6)	119.1 (79.9–168.0)	702.6 (498.3–945.5)	0.0 (0.0–0.0)	9.1 (6.5–12.3)	51.7 (36.0–70.3)	103.2 (65.4–150.8)	45.2 (33.4–59.6)	0.0 (0.0–0.0)	17.0 (11.3–23.5)	29.6 (21.3–39.0)
Japan	YLDs Rate: Cumulative change (%)	-8.1 (-11.1 to -4.8)	-20.2 (-30.4 to -10.0)	0.1 (-3.1 to 3.0)	84.1 (45.0–125.3)	-13.6 (-21.2 to -5.0)	-0.9 (-9.4 to 7.9)	-52.2 (-58.3 to -45.3)	-15.7 (-22.5 to -8.6)	7.7 (-19.2 to 48.3)	-0.8 (-9.9 to 9.3)	84.4 (58.1–112.8)
Singapore	Prevalence Number: 1990	139,052 (134,129–143,890)	14,673 (9,913–21,963)	39,847 (37,526–42,259)	0 (0–0)	220 (190–260)	10,439 (9,630–11,285)	9,911 (7,904–11,927)	3,348 (3,064–3,658)	5 (2–10)	207 (168–254)	-
Singapore	Prevalence Number: 2015	113,607 (109,378–118,041)	7,112 (4,387–10,728)	34,508 (32,665–36,496)	0 (0–0)	82 (73–91)	8,041 (7,488–8,713)	4,639 (3,493–5,936)	2,209 (2,000–2,449)	4 (2–9)	163 (129–205)	-
Singapore	Prevalence Number: Cumulative change (%	-18.3 (-22.2 to -14.2)	-48.5 (-74.3 to -6.2)	-13.3 (-20.0 to -6.2)	-73.7 (-82.8 to -61.2)	-62.3 (-68.0 to -56.9)	-22.8 (-30.8 to -14.2)	-53.2 (-60.8 to -44.4)	-33.9 (-39.2 to -27.6)	-10.4 (-33.8 to 13.6)	-21.1 (-27.7 to -14.9)	-
Singapore	Prevalence Rate: 1990	61,432.7 (59,228.6–63,595.0)	6,331.0 (4,251.0–9,649.3)	17,636.1 (16,602.9–18,711.3)	0.0 (0.0–0.1)	96.1 (82.8–113.7)	4,582.0 (4,229.5–4,956.2)	4,425.9 (3,529.8–5,325.9)	1,410.8 (1,289.5–1,544.7)	2.3 (1.3–4.7)	90.0 (72.8–110.4)	-
Singapore	Prevalence Rate: 2015	59,076.0 (56,875.5–61,377.9)	3,766.4 (2,328.8–5,673.4)	17,931.8 (16,972.9–18,965.4)	0.0 (0.0–0.0)	43.1 (38.7–47.9)	4,194.3 (3,904.8–4,543.9)	2,401.9 (1,808.7–3,073.2)	1,173.1 (1,062.6–1,300.7)	2.4 (1.3–4.8)	85.9 (67.9–107.5)	-
Singapore	Prevalence Rate: Cumulative change (%	-3.8 (-8.5 to 1.1)	-36.7 (-69.0 to 14.5)	1.8 (-6.1 to 10.1)	-67.8 (-79.0 to -52.5)	-54.8 (-61.6 to -48.3)	-8.3 (-17.8 to 1.9)	-45.7 (-54.6 to -35.5)	-16.7 (-23.5 to -8.7)	4.8 (-22.7 to 33.3)	-4.5 (-12.4 to 3.1)	-

© 2017 Global Burden of Disease Child and Adolescent Health Collaboration. JAMA Pediatrics.

eTable 4. Prevalent cases, Rates (per 100,000 population), Years Lived with Disability (YLDs), and Cumulative Percent Change with 95% Uncertainty Interval (UI) for the Top 10 Global Causes of YLDsin Children and Adolescents in 195 Countries and Territories, Aged Under 5 Years, Both Sexes, 1990 and 2015. *Note: best viewed by enlarging in browser.*

Location	Measure	All causes	Iron-deficiency anemia	Skin and subcutaneous diseases	Protein-energy malnutrition	Diarrheal diseases	Hemoglobinopathies and hemolytic anemias	Asthma	Neonatal preterm birth complications	Malaria	Neonatal encephalopathy due to birth asphyxia and trauma	Other neonatal disorders
Singapore	YLDs Number: 1990	4,359 (3,243–5,587)	304 (163–552)	1,173 (826–1,580)	0 (0–0)	36 (24–51)	44 (24–78)	458 (297–653)	134 (99–179)	0 (0–0)	39 (25–56)	19 (12–28)
Singapore	YLDs Number: 2015	3,217 (2,421–4,136)	149 (71–292)	1,014 (715–1,367)	0 (0–0)	13 (8–19)	20 (10–39)	215 (134–316)	83 (60–110)	0 (0–0)	30 (18–43)	66 (42–96)
Singapore	YLDs Number: Cumulative change (%)	-26.1 (-31.8 to -20.5)	-46.8 (-76.7 to 9.5)	-13.6 (-16.9 to -9.9)	-73.7 (-82.9 to -61.3)	-62.0 (-73.3 to -48.3)	-49.0 (-77.4 to 9.7)	-53.0 (-61.3 to -44.2)	-37.5 (-48.8 to -25.1)	-6.4 (-68.1 to 107.0)	-22.6 (-43.3 to 1.1)	249.0 (122.9–423.1)
Singapore	YLDs Rate: 1990	1,923.5 (1,430.4–2,465.6)	130.7 (69.5–241.5)	521.6 (367.4–701.9)	0.0 (0.0–0.0)	16.0 (10.7–22.5)	19.1 (10.5–34.2)	204.8 (133.1–291.7)	58.9 (43.7–78.7)	0.0 (0.0–0.1)	17.3 (11.2–25.0)	8.6 (5.5–12.5)
Singapore	YLDs Rate: 2015	1,674.7 (1,260.2–2,155.4)	79.6 (38.0–156.0)	526.3 (371.1–709.7)	0.0 (0.0–0.0)	7.2 (4.6–10.0)	11.0 (5.4–20.8)	111.4 (69.8–163.8)	43.5 (31.5–57.7)	0.0 (0.0–0.1)	15.7 (9.8–22.6)	34.5 (22.4–50.1)
Singapore	YLDs Rate: Cumulative change (%)	-12.8 (-19.6 to -6.1)	-33.8 (-71.7 to 35.8)	0.9 (-2.9 to 5.1)	-67.7 (-79.1 to -52.5)	-54.4 (-68.1 to -37.8)	-37.4 (-72.7 to 32.9)	-45.6 (-55.2 to -35.3)	-25.8 (-39.2 to -11.1)	9.9 (-62.6 to 144.4)	-8.1 (-32.8 to 20.4)	315.2 (165.7–522.0)
South Korea	Prevalence Number: 1990	1,981,259 (1,896,792–2,056,950)	25,036 (18,162–35,862)	590,553 (556,218–625,655)	8 (4–13)	2,741 (2,458–3,130)	109,988 (99,688–120,129)	132,169 (107,453–157,963)	63,997 (57,869–70,254)	122 (28–246)	3,167 (2,544–3,861)	-
South Korea	Prevalence Number: 2015	1,326,750 (1,269,648–1,384,366)	18,085 (14,287–22,354)	411,776 (387,588–436,484)	0 (0–0)	929 (842–1,031)	71,886 (64,894–79,183)	60,788 (44,061–79,859)	36,383 (32,962–40,238)	3 (1–6)	2,108 (1,683–2,583)	-
South Korea	Prevalence Number: Cumulative change (%	-33.0 (-36.9 to -29.1)	-24.0 (-54.1 to 7.8)	-30.2 (-35.6 to -24.6)	-92.7 (-95.7 to -88.6)	-66.0 (-70.2 to -62.1)	-34.4 (-43.0 to -25.6)	-54.1 (-62.3 to -46.1)	-43.1 (-48.4 to -37.3)	6,710.6 (-98.3 to -89.9)	-33.4 (-38.5 to -28.5)	-
South Korea	Prevalence Rate: 1990	59,128.2 (56,614.5–61,378.8)	762.1 (555.6–1,087.7)	17,619.2 (16,597.8–18,667.4)	0.2 (0.1–0.4)	82.3 (73.9–93.9)	3,291.6 (2,983.1–3,594.6)	3,930.6 (3,195.6–4,697.7)	1,947.9 (1,761.3–2,136.6)	3.6 (0.8–7.4)	95.3 (76.6–116.1)	-
South Korea	Prevalence Rate: 2015	58,179.8 (55,678.9–60,703.0)	805.5 (638.0–993.2)	18,054.2 (16,993.8–19,137.9)	0.0 (0.0–0.0)	40.9 (37.1–45.4)	3,157.6 (2,850.8–3,477.9)	2,660.3 (1,928.3–3,495.0)	1,613.5 (1,462.5–1,782.3)	0.2 (0.1–0.3)	92.9 (74.2–113.9)	-
South Korea	Prevalence Rate: Cumulative change (%	-1.6 (-7.3 to 4.1)	11.0 (-32.6 to 57.0)	2.6 (-5.3 to 10.8)	-89.4 (-93.8 to -83.4)	-50.2 (-56.3 to -44.5)	-3.8 (-16.3 to 9.1)	-32.4 (-44.6 to -20.6)	-17.0 (-24.7 to -8.7)	9,911.1 (-97.4 to -85.1)	-2.5 (-10.0 to 4.7)	-
South Korea	YLDs Number: 1990	63,043 (47,573–81,216)	613 (392–913)	18,435 (12,990–24,549)	1 (0–1)	458 (308–649)	106 (65–166)	6,112 (4,019–8,706)	2,608 (1,976–3,376)	2 (0–5)	621 (397–861)	394 (258–560)
South Korea	YLDs Number: 2015	37,610 (28,681–48,408)	432 (290–619)	12,640 (8,912–16,887)	0 (0–0)	155 (101–225)	70 (45–102)	2,813 (1,782–4,187)	1,209 (881–1,609)	0 (0–0)	383 (247–552)	441 (272–662)
South Korea	YLDs Number: Cumulative change (%)	-40.3 (-42.8 to -37.7)	-27.8 (-49.8 to -7.8)	-31.4 (-34.1 to -28.7)	-92.7 (-95.8 to -88.6)	-65.5 (-75.1 to -54.3)	-32.0 (-54.7 to -7.8)	-54.0 (-62.6 to -45.5)	-53.5 (-60.9 to -45.3)	10,788.1 (-98.3 to -89.9)	-37.3 (-53.6 to -18.4)	14.2 (-27.2 to 66.2)
South Korea	YLDs Rate: 1990	1,882.1 (1,420.1–2,424.4)	18.8 (12.0–27.9)	549.2 (387.1–731.7)	0.0 (0.0–0.1)	13.8 (9.3–19.5)	3.2 (2.0–5.1)	181.8 (119.5–258.9)	78.1 (59.2–101.0)	0.1 (0.0–0.2)	18.6 (11.9–25.8)	11.8 (7.7–16.8)
South Korea	YLDs Rate: 2015	1,649.7 (1,258.1–2,123.4)	19.3 (13.0–27.7)	553.7 (390.4–739.8)	0.0 (0.0–0.0)	6.9 (4.5–9.9)	3.1 (2.0–4.6)	123.1 (78.0–183.3)	53.1 (38.7–70.7)	0.0 (0.0–0.0)	16.9 (10.9–24.3)	19.4 (12.0–29.1)
South Korea	YLDs Rate: Cumulative change (%)	-12.3 (-15.9 to -8.5)	5.5 (-26.2 to 34.6)	0.9 (-3.1 to 4.9)	-89.4 (-93.8 to -83.4)	-49.5 (-63.5 to -33.2)	-0.6 (-33.5 to 34.2)	-32.3 (-45.0 to -19.7)	-31.8 (-42.6 to -19.8)	15,867.9 (-97.4 to -85.1)	-7.9 (-31.8 to 19.7)	67.7 (6.8–143.9)
Western Europe	Prevalence Number: 1990	14,884,104 (14,692,965–15,077,821)	6,546,243 (6,093,973–6,983,638)	3,517,930 (3,434,784–3,603,127)	47 (31–68)	29,268 (27,708–30,929)	3,222,746 (3,144,079–3,298,334)	814,282 (667,888–984,218)	402,617 (377,085–427,810)	0 (0–0)	13,892 (10,736–17,671)	-
Western Europe	Prevalence Number: 2015	14,815,520 (14,608,978–15,045,796)	6,629,154 (6,198,477–7,138,953)	3,477,384 (3,398,212–3,559,162)	26 (17–39)	26,119 (24,573–27,872)	2,830,009 (2,752,397–2,911,687)	667,724 (535,662–809,801)	367,365 (346,251–388,887)	0 (0–0)	14,132 (11,018–17,874)	-
Western Europe	Prevalence Number: Cumulative change (%	-0.5 (-2.2 to 1.5)	1.5 (-8.6 to 11.9)	-1.1 (-3.6 to 1.4)	-43.1 (-56.7 to -29.8)	-10.8 (-12.3 to -9.3)	-12.2 (-14.8 to -9.4)	-18.0 (-22.2 to -13.5)	-8.7 (-11.4 to -6.2)	-1.3 (-1.6 to -0.9)	1.8 (-0.6 to 4.0)	-
Western Europe	Prevalence Rate: 1990	66,025.1 (65,180.6–66,880.4)	29,127.6 (27,129.6–31,072.8)	15,567.0 (15,199.6–15,943.6)	0.2 (0.1–0.3)	130.2 (123.3–137.6)	14,301.4 (13,952.5–14,636.3)	3,593.0 (2,947.0–4,342.8)	1,811.6 (1,697.4–1,922.8)	0.0 (0.0–0.0)	61.9 (47.8–78.7)	-
Western Europe	Prevalence Rate: 2015	67,012.2 (66,088.5–68,050.8)	30,069.1 (28,120.5–32,373.9)	15,695.2 (15,338.1–16,063.6)	0.1 (0.1–0.2)	118.5 (111.5–126.5)	12,806.5 (12,456.1–13,175.6)	3,005.9 (2,411.4–3,645.4)	1,684.4 (1,587.5–1,782.8)	0.0 (0.0–0.0)	64.2 (50.1–81.2)	-
Western Europe	Prevalence Rate: Cumulative change (%	1.5 (-0.3 to 3.5)	3.4 (-6.8 to 14.0)	0.8 (-1.7 to 3.5)	-42.1 (-55.9 to -28.4)	-9.0 (-10.6 to -7.5)	-10.4 (-13.1 to -7.6)	-16.4 (-20.7 to -11.8)	-7.0 (-9.7 to -4.4)	0.7 (0.3–1.0)	3.8 (1.3–6.1)	-
Western Europe	YLDs Number: 1990	597,743 (444,022–785,325)	191,527 (131,108–271,175)	94,941 (67,439–127,085)	5 (3–9)	4,841 (3,501–6,388)	16,655 (11,186–23,760)	37,545 (24,779–53,005)	19,004 (14,412–24,605)	0 (0–0)	2,686 (1,799–3,751)	1,739 (1,316–2,261)
Western Europe	YLDs Number: 2015	578,145 (428,414–757,639)	199,385 (138,601–278,152)	93,521 (66,487–125,454)	3 (1–5)	4,325 (3,115–5,791)	16,429 (11,411–22,919)	30,777 (20,243–43,285)	14,365 (10,829–18,793)	0 (0–0)	2,647 (1,759–3,681)	4,939 (3,604–6,436)
Western Europe	YLDs Number: Cumulative change (%)	-3.2 (-9.2 to 2.9)	4.7 (-11.0 to 21.4)	-1.5 (-3.0 to 0.0)	-42.5 (-57.6 to -25.5)	-10.7 (-15.4 to -5.4)	-0.6 (-21.4 to 16.3)	-18.0 (-22.5 to -13.3)	-24.4 (-29.1 to -20.1)	1.3 (-9.5 to 12.7)	-1.3 (-9.9 to 8.2)	184.2 (143.0–224.6)
Western Europe	YLDs Rate: 1990	2,652.8 (1,970.5–3,486.1)	853.5 (584.2–1,208.6)	419.5 (298.0–561.5)	0.0 (0.0–0.0)	21.5 (15.6–28.4)	74.2 (49.9–105.8)	165.7 (109.3–233.9)	84.3 (64.0–109.2)	0.0 (0.0–0.0)	11.9 (8.0–16.6)	7.7 (5.8–10.0)
Western Europe	YLDs Rate: 2015	2,616.8 (1,939.0–3,428.7)	905.7 (630.0–1,264.6)	421.5 (299.6–565.5)	0.0 (0.0–0.0)	19.6 (14.1–26.3)	74.6 (51.8–104.1)	138.6 (91.1–194.9)	65.0 (49.0–85.0)	0.0 (0.0–0.0)	12.0 (8.0–16.7)	22.4 (16.3–29.1)
Western Europe	YLDs Rate: Cumulative change (%)	-1.3 (-7.4 to 4.9)	6.7 (-9.2 to 23.7)	0.5 (-1.0 to 2.0)	-41.4 (-56.8 to -24.0)	-8.9 (-13.7 to -3.5)	1.3 (-19.7 to 18.4)	-16.4 (-20.9 to -11.5)	-22.9 (-27.7 to -18.5)	3.3 (-7.7 to 15.0)	0.6 (-8.0 to 10.2)	189.8 (147.8–231.1)
Andorra	Prevalence Number: 1990	2,123 (2,032–2,205)	927 (704–1,176)	483 (448–518)	0 (0–0)	2 (2–2)	471 (418–548)	103 (80–128)	39 (35–43)	0 (0–0)	1 (0–1)	-
Andorra	Prevalence Number: 2015	2,726 (2,614–2,826)	1,189 (975–1,440)	635 (587–680)	0 (0–0)	2 (2–2)	562 (484–689)	115 (85–146)	46 (41–52)	0 (0–0)	1 (1–2)	-
Andorra	Prevalence Number: Cumulative change (%	28.5 (21.8–35.2)	30.1 (2.1–60.6)	31.7 (19.2–45.6)	-50.9 (-92.3 to 39.6)	4.4 (-3.5 to 11.2)	20.0 (-1.9 to 48.9)	11.2 (-3.3 to 25.8)	17.3 (6.8–30.7)	31.7 (30.7–32.7)	27.8 (17.2–37.9)	-
Andorra	Prevalence Rate: 1990	71,260.2 (68,169.8–74,014.9)	30,988.4 (23,551.1–39,406.5)	16,250.2 (15,091.8–17,443.6)	0.1 (0.0–0.3)	83.6 (74.3–92.9)	15,810.8 (14,037.1–18,379.0)	3,500.0 (2,727.2–4,354.1)	1,300.5 (1,163.0–1,450.1)	0.0 (0.0–0.0)	47.1 (33.2–64.2)	-
Andorra	Prevalence Rate: 2015	70,630.1 (67,745.3–73,129.3)	31,271.9 (25,918.2–37,623.8)	16,295.9 (15,085.9–17,450.4)	0.0 (0.0–0.1)	68.8 (60.9–77.2)	14,612.2 (12,584.2–17,895.2)	2,920.5 (2,175.8–3,711.0)	1,277.4 (1,140.9–1,435.7)	0.0 (0.0–0.0)	47.7 (33.1–65.5)	-
Andorra	Prevalence Rate: Cumulative change (%	-0.8 (-5.9 to 4.3)	2.4 (-19.4 to 26.5)	0.5 (-9.1 to 11.0)	-59.5 (-93.6 to 15.5)	-17.6 (-24.0 to -12.3)	-7.1 (-24.0 to 15.3)	-16.5 (-27.4 to -5.5)	-1.6 (-10.4 to 8.8)	0.0 (-0.0 to 0.0)	1.4 (-7.0 to 9.5)	-
Andorra	YLDs Number: 1990	82 (59–112)	27 (16–42)	13 (9–17)	0 (0–0)	0 (0–0)	3 (1–4)	4 (3–6)	1 (1–2)	0 (0–0)	0 (0–0)	0 (0–0)
Andorra	YLDs Number: 2015	102 (74–137)	34 (21–50)	17 (12–23)	0 (0–0)	0 (0–0)	3 (2–5)	5 (3–7)	1 (1–2)	0 (0–0)	0 (0–0)	0 (0–1)
Andorra	YLDs Number: Cumulative change (%)	24.8 (7.8–42.0)	28.7 (-7.8 to 65.3)	32.4 (26.8–38.5)	-51.0 (-92.3 to 39.5)	5.4 (-18.6 to 33.4)	29.0 (-6.6 to 66.9)	11.5 (-4.4 to 29.2)	0.5 (-16.3 to 18.0)	32.1 (-1.7 to 83.3)	29.5 (-16.1 to 86.7)	153.3 (-50.6 to 588.9)
Andorra	YLDs Rate: 1990	2,778.6 (1,986.3–3,771.3)	921.5 (539.0–1,401.7)	450.0 (316.0–605.6)	0.0 (0.0–0.0)	13.8 (9.4–19.4)	104.3 (59.4–159.5)	161.3 (103.3–230.8)	58.0 (41.9–77.2)	0.0 (0.0–0.0)	9.0 (5.0–14.1)	7.8 (2.4–16.9)
Andorra	YLDs Rate: 2015	2,679.4 (1,926.6–3,594.5)	918.7 (583.9–1,337.5)	450.4 (319.7–608.0)	0.0 (0.0–0.0)	11.4 (7.6–15.9)	103.8 (65.1–152.2)	134.7 (84.1–195.7)	44.9 (32.2–60.7)	0.0 (0.0–0.0)	8.8 (4.8–14.2)	11.3 (3.6–26.3)
Andorra	YLDs Rate: Cumulative change (%)	-3.1 (-16.4 to 10.2)	2.4 (-26.2 to 30.2)	0.1 (-4.1 to 4.8)	-59.6 (-93.7 to 15.1)	-16.8 (-35.6 to 4.5)	2.5 (-25.6 to 31.9)	-16.3 (-28.2 to -3.0)	-22.2 (-35.1 to -8.7)	0.6 (-25.2 to 39.5)	0.2 (-34.8 to 44.8)	96.1 (-61.6 to 433.9)
Austria	Prevalence Number: 1990	308,237 (295,992–320,919)	137,758 (114,080–164,455)	69,313 (64,540–74,450)	0 (0–1)	297 (281–315)	61,811 (56,630–67,172)	13,187 (10,542–16,167)	9,366 (8,917–9,959)	0 (0–0)	19 (13–25)	-

© 2017 Global Burden of Disease Child and Adolescent Health Collaboration. JAMA Pediatrics.

eTable 4. Prevalent cases, Rates (per 100,000 population), Years Lived with Disability (YLDs), and Cumulative Percent Change with 95% Uncertainty Interval (UI) for the Top 10 Global Causes of YLDsin Children and Adolescents in 195 Countries and Territories, Aged Under 5 Years, Both Sexes, 1990 and 2015. Note: best viewed by enlarging in browser.

Location	Measure	All causes	Iron-deficiency anemia	Skin and subcutaneous diseases	Protein-energy malnutrition	Diarrheal diseases	Hemoglobinopathies and hemolytic anemias	Asthma	Neonatal preterm birth complications	Malaria	Neonatal encephalopathy due to birth asphyxia and trauma	Other neonatal disorders
Austria	Prevalence Number: 2015	274,246 (262,332–286,214)	108,370 (84,976–138,578)	64,705 (60,123–69,392)	0 (0–0)	378 (344–418)	50,136 (44,781–56,059)	11,823 (8,968–14,937)	7,547 (6,853–8,298)	0 (0–0)	21 (15–27)	-
Austria	Prevalence Number: Cumulative change (%	-11.0 (-15.7 to -5.8)	-20.4 (-42.3 to 3.0)	-6.5 (-15.2 to 2.9)	-27.8 (-58.7 to 18.6)	27.1 (18.0–37.2)	-18.7 (-28.7 to -7.3)	-10.2 (-23.9 to 6.2)	-19.3 (-27.3 to -10.9)	-7.8 (-7.8 to -7.8)	11.0 (2.2–26.0)	-
Austria	Prevalence Rate: 1990	71,139.7 (68,314.2–74,065.8)	31,800.6 (26,337.5–37,959.0)	15,994.7 (14,893.2–17,180.1)	0.2 (0.1–0.3)	68.8 (64.9–72.8)	14,266.3 (13,070.6–15,503.4)	3,042.7 (2,432.4–3,730.2)	2,163.7 (2,060.1–2,300.3)	0.0 (0.0–0.0)	4.4 (3.2–5.8)	-
Austria	Prevalence Rate: 2015	68,603.5 (65,623.8–71,599.3)	27,090.0 (21,234.3–34,653.5)	16,192.4 (15,045.8–17,364.4)	0.1 (0.1–0.2)	94.6 (86.1–104.7)	12,540.2 (11,200.7–14,021.6)	2,960.7 (2,245.7–3,740.4)	1,882.5 (1,709.2–2,070.4)	0.0 (0.0–0.0)	5.3 (3.9–7.0)	-
Austria	Prevalence Rate: Cumulative change (%	-3.5 (-8.7 to 2.1)	-13.8 (-37.6 to 11.5)	1.4 (-8.0 to 11.6)	-22.1 (-55.4 to 27.9)	37.6 (27.8–48.6)	-11.9 (-22.8 to 0.5)	-2.5 (-17.4 to 15.3)	-12.9 (-21.5 to -3.8)	-0.0 (-0.0 to 0.0)	20.1 (10.6–36.3)	-
Austria	YLDs Number: 1990	12,156 (8,853–16,072)	4,100 (2,653–6,023)	1,924 (1,373–2,577)	0 (0–0)	49 (33–68)	324 (210–469)	607 (397–865)	398 (297–517)	0 (0–0)	3 (2–5)	25 (17–34)
Austria	YLDs Number: 2015	10,130 (7,320–13,468)	3,031 (1,794–4,788)	1,784 (1,263–2,402)	0 (0–0)	62 (42–86)	228 (134–352)	546 (344–799)	287 (210–378)	0 (0–0)	3 (2–5)	67 (44–96)
Austria	YLDs Number: Cumulative change (%)	-16.4 (-28.9 to -3.8)	-24.5 (-53.2 to 6.1)	-7.3 (-11.5 to -3.1)	-27.6 (-58.7 to 19.1)	29.2 (-0.4 to 63.6)	-28.1 (-55.5 to 2.1)	-9.8 (-24.0 to 8.7)	-27.7 (-37.3 to -16.6)	-10.9 (-43.1 to 27.2)	5.5 (-3.5 to 24.4)	174.0 (86.3–276.1)
Austria	YLDs Rate: 1990	2,805.9 (2,043.6–3,709.5)	946.6 (612.7–1,390.5)	444.2 (316.9–594.8)	0.0 (0.0–0.0)	11.3 (7.8–15.8)	74.9 (48.7–108.5)	140.2 (91.6–199.6)	91.9 (68.6–119.4)	0.0 (0.0–0.0)	0.9 (0.5–1.2)	5.8 (4.1–8.0)
Austria	YLDs Rate: 2015	2,533.7 (1,831.1–3,367.7)	757.6 (448.4–1,197.2)	446.7 (316.4–601.4)	0.0 (0.0–0.0)	15.7 (10.6–21.5)	57.1 (33.7–88.2)	136.9 (86.3–200.1)	71.9 (52.7–94.6)	0.0 (0.0–0.0)	1.0 (0.6–1.4)	16.9 (11.2–24.1)
Austria	YLDs Rate: Cumulative change (%)	-9.4 (-22.9 to 4.2)	-18.3 (-49.4 to 14.8)	0.6 (-3.9 to 5.1)	-22.0 (-55.5 to 28.5)	39.9 (7.8–77.2)	-22.2 (-51.8 to 10.4)	-2.1 (-17.5 to 17.9)	-21.6 (-32.0 to -9.6)	-3.3 (-38.3 to 38.0)	14.3 (4.6–34.8)	196.9 (101.9–307.6)
Belgium	Prevalence Number: 1990	368,362 (351,831–384,725)	155,058 (121,954–195,851)	93,030 (86,827–99,841)	0 (0–0)	502 (442–562)	75,033 (68,575–82,349)	18,984 (15,406–23,119)	9,027 (8,535–9,676)	0 (0–0)	495 (393–612)	-
Belgium	Prevalence Number: 2015	412,270 (390,340–432,782)	179,970 (127,571–222,346)	102,704 (95,850–109,753)	0 (0–1)	563 (498–632)	72,839 (63,777–82,756)	14,369 (11,075–17,918)	10,668 (9,770–11,659)	0 (0–0)	537 (427–662)	-
Belgium	Prevalence Number: Cumulative change (%	12.0 (4.3–19.6)	18.2 (-21.7 to 58.6)	10.6 (0.7–20.6)	83.3 (11.0–185.6)	12.0 (6.6–17.6)	-2.7 (-17.0 to 12.4)	-24.3 (-34.1 to -13.6)	18.3 (7.2–30.6)	9.4 (9.4–9.4)	8.7 (1.1–16.5)	-
Belgium	Prevalence Rate: 1990	62,610.9 (59,801.4–65,395.4)	26,387.7 (20,778.2–33,308.6)	15,796.8 (14,744.5–16,953.5)	0.1 (0.0–0.1)	85.6 (75.3–95.7)	12,754.8 (11,657.0–13,998.5)	3,220.2 (2,613.3–3,921.8)	1,542.9 (1,459.2–1,653.2)	0.0 (0.0–0.0)	84.3 (67.1–104.4)	-
Belgium	Prevalence Rate: 2015	64,045.5 (60,647.8–67,224.0)	28,003.2 (19,879.5–34,563.6)	15,937.9 (14,874.2–17,031.6)	0.1 (0.1–0.2)	87.6 (77.5–98.3)	11,316.7 (9,909.0–12,856.8)	2,227.6 (1,716.9–2,777.6)	1,666.6 (1,526.7–1,821.0)	0.0 (0.0–0.0)	83.6 (66.5–103.1)	-
Belgium	Prevalence Rate: Cumulative change (%	2.4 (-4.6 to 9.3)	8.0 (-28.3 to 45.0)	1.1 (-8.0 to 10.2)	67.6 (1.4–160.9)	2.4 (-2.6 to 7.5)	-11.0 (-24.2 to 2.7)	-30.8 (-39.8 to -21.0)	8.1 (-1.9 to 19.3)	-0.0 (-0.0 to 0.0)	-0.6 (-7.6 to 6.5)	-
Belgium	YLDs Number: 1990	14,952 (10,639–19,855)	4,195 (2,419–6,572)	2,538 (1,803–3,402)	0 (0–0)	83 (56–115)	220 (128–340)	876 (568–1,230)	389 (290–501)	0 (0–0)	96 (61–139)	48 (32–67)
Belgium	YLDs Number: 2015	16,265 (11,626–21,790)	5,225 (2,788–8,070)	2,787 (1,980–3,737)	0 (0–0)	93 (63–128)	261 (138–395)	663 (423–940)	397 (295–526)	0 (0–0)	99 (65–142)	178 (115–250)
Belgium	YLDs Number: Cumulative change (%)	9.3 (-11.2 to 27.2)	30.8 (-36.4 to 100.3)	9.8 (4.9–14.8)	83.4 (10.8–185.7)	12.1 (-11.6 to 39.2)	24.1 (-37.7 to 88.5)	-24.1 (-35.1 to -11.8)	2.3 (-13.5 to 18.8)	14.1 (-43.3 to 68.5)	4.9 (-22.8 to 37.8)	275.7 (153.2–428.8)
Belgium	YLDs Rate: 1990	2,541.7 (1,808.8–3,375.3)	714.5 (412.8–1,118.8)	430.7 (306.1–577.4)	0.0 (0.0–0.0)	14.3 (9.6–19.6)	37.6 (21.9–57.9)	148.6 (96.4–208.7)	66.2 (49.3–85.2)	0.0 (0.0–0.0)	16.4 (10.4–23.6)	8.3 (5.5–11.5)
Belgium	YLDs Rate: 2015	2,527.9 (1,807.7–3,386.0)	813.9 (435.1–1,257.1)	432.3 (307.1–579.7)	0.0 (0.0–0.0)	14.5 (9.9–19.9)	40.8 (21.6–61.5)	102.8 (65.7–145.9)	61.7 (45.9–81.8)	0.0 (0.0–0.0)	15.4 (10.1–22.1)	27.7 (17.9–39.0)
Belgium	YLDs Rate: Cumulative change (%)	-0.0 (-18.8 to 16.4)	19.6 (-41.8 to 83.3)	0.4 (-4.2 to 4.9)	67.7 (1.2–161.2)	2.5 (-19.1 to 27.2)	13.5 (-43.0 to 72.3)	-30.7 (-40.7 to -19.4)	-6.5 (-20.9 to 8.5)	4.3 (-48.1 to 54.0)	-4.2 (-29.4 to 25.9)	243.3 (131.4–383.3)
Cyprus	Prevalence Number: 1990	42,323 (40,353–44,280)	15,095 (11,262–20,993)	9,238 (8,584–9,906)	0 (0–0)	61 (55–71)	13,816 (13,179–14,455)	2,236 (1,762–2,740)	1,222 (1,097–1,355)	0 (0–0)	35 (24–48)	-
Cyprus	Prevalence Number: 2015	26,278 (25,339–27,193)	10,085 (8,629–12,311)	5,802 (5,405–6,222)	0 (0–0)	31 (28–35)	8,304 (7,925–8,714)	1,047 (803–1,318)	729 (658–810)	0 (0–0)	21 (14–29)	-
Cyprus	Prevalence Number: Cumulative change (%	-37.9 (-41.2 to -34.3)	-31.7 (-45.9 to -15.4)	-37.1 (-43.0 to -30.7)	-83.3 (-92.5 to -70.2)	-49.3 (-53.3 to -46.1)	-39.8 (-43.6 to -36.1)	-53.2 (-57.6 to -48.1)	-40.2 (-45.4 to -35.3)	-37.6 (-37.8 to -37.5)	-39.3 (-43.5 to -34.9)	-
Cyprus	Prevalence Rate: 1990	72,441.5 (69,084.4–75,773.2)	25,995.9 (19,450.5–36,056.4)	15,753.0 (14,637.5–16,891.3)	0.3 (0.1–0.5)	106.8 (95.1–122.9)	23,670.9 (22,576.8–24,767.1)	3,794.7 (2,991.5–4,651.4)	2,141.6 (1,924.3–2,373.3)	0.0 (0.0–0.0)	60.5 (41.7–83.1)	-
Cyprus	Prevalence Rate: 2015	72,494.0 (69,914.7–74,986.3)	28,146.5 (24,078.7–34,105.3)	15,887.6 (14,810.1–17,039.3)	0.1 (0.0–0.1)	87.7 (79.0–98.3)	22,950.4 (21,904.9–24,077.7)	2,841.7 (2,180.1–3,577.4)	2,107.1 (1,901.0–2,334.3)	0.0 (0.0–0.0)	59.7 (40.6–82.3)	-
Cyprus	Prevalence Rate: Cumulative change (%	0.1 (-5.2 to 5.9)	10.7 (-12.6 to 36.8)	1.0 (-8.4 to 11.3)	-72.3 (-87.7 to -50.6)	-17.7 (-24.2 to -12.6)	-3.0 (-9.0 to 3.0)	-25.1 (-32.3 to -17.1)	-1.5 (-9.9 to 6.6)	0.0 (-0.0 to 0.0)	-1.2 (-8.1 to 5.9)	-
Cyprus	YLDs Number: 1990	1,589 (1,124–2,153)	427 (238–757)	258 (183–345)	0 (0–0)	10 (7–14)	73 (42–128)	103 (66–144)	70 (52–91)	0 (0–0)	6 (3–10)	33 (11–71)
Cyprus	YLDs Number: 2015	962 (707–1,276)	290 (189–431)	161 (114–216)	0 (0–0)	5 (3–7)	47 (30–71)	48 (30–69)	38 (28–51)	0 (0–0)	3 (2–6)	21 (9–42)
Cyprus	YLDs Number: Cumulative change (%)	-39.1 (-47.0 to -31.3)	-28.5 (-51.3 to 0.1)	-37.3 (-40.1 to -34.5)	-83.3 (-92.5 to -70.2)	-48.9 (-58.9 to -37.4)	-32.1 (-52.9 to -6.0)	-53.1 (-58.2 to -47.8)	-44.4 (-51.2 to -37.1)	-33.7 (-52.0 to -6.0)	-40.5 (-58.7 to -17.4)	-11.9 (-76.3 to 127.6)
Cyprus	YLDs Rate: 1990	2,724.8 (1,930.0–3,689.0)	738.6 (413.5–1,300.1)	439.2 (312.2–587.3)	0.0 (0.0–0.1)	17.7 (12.2–25.2)	127.6 (72.6–221.1)	174.9 (112.6–244.8)	120.3 (89.9–156.1)	0.0 (0.0–0.0)	11.6 (6.7–17.9)	57.8 (19.5–123.0)
Cyprus	YLDs Rate: 2015	2,664.3 (1,957.5–3,526.1)	815.0 (533.7–1,214.2)	440.8 (312.2–591.8)	0.0 (0.0–0.0)	14.6 (10.0–20.3)	134.4 (86.8–199.7)	131.1 (83.2–188.1)	107.7 (79.1–142.0)	0.0 (0.0–0.0)	11.0 (6.2–17.2)	60.4 (25.4–116.3)
Cyprus	YLDs Rate: Cumulative change (%)	-1.6 (-14.6 to 11.2)	16.3 (-20.9 to 63.0)	0.4 (-4.0 to 4.9)	-72.4 (-87.7 to -50.6)	-17.1 (-33.1 to 1.2)	10.4 (-23.5 to 52.8)	-25.0 (-33.2 to -16.5)	-10.4 (-21.3 to 1.5)	6.4 (-22.8 to 50.5)	-4.1 (-33.3 to 33.0)	42.0 (-61.8 to 267.3)
Denmark	Prevalence Number: 1990	201,162 (191,789–210,584)	81,780 (62,119–104,613)	50,790 (47,588–54,302)	0 (0–1)	231 (204–260)	43,789 (38,284–52,318)	9,440 (7,316–11,627)	5,109 (4,738–5,551)	0 (0–0)	159 (112–217)	-
Denmark	Prevalence Number: 2015	206,641 (195,952–217,040)	83,099 (63,404–115,244)	53,790 (50,441–57,697)	0 (0–0)	267 (239–302)	41,403 (35,504–50,959)	7,474 (5,733–9,420)	4,738 (4,350–5,160)	0 (0–0)	163 (113–224)	-
Denmark	Prevalence Number: Cumulative change (%	2.8 (-3.8 to 10.0)	4.1 (-31.9 to 38.8)	6.1 (-3.1 to 15.4)	-62.2 (-79.7 to -36.1)	15.7 (7.6–24.8)	-4.7 (-24.4 to 21.0)	-20.7 (-31.1 to -8.5)	-7.1 (-14.7 to 1.2)	4.5 (4.0–5.0)	2.2 (-5.3 to 9.3)	-
Denmark	Prevalence Rate: 1990	70,170.7 (66,890.0–73,485.8)	28,335.1 (21,480.1–36,312.1)	17,807.2 (16,685.4–19,038.1)	0.2 (0.1–0.4)	80.3 (70.8–90.2)	15,247.3 (13,327.4–18,218.7)	3,329.4 (2,580.3–4,100.8)	1,726.1 (1,600.9–1,876.3)	0.0 (0.0–0.0)	55.1 (38.7–75.2)	-
Denmark	Prevalence Rate: 2015	69,623.1 (66,017.0–73,133.0)	28,119.7 (21,494.3–38,926.3)	18,072.9 (16,950.3–19,387.0)	0.1 (0.0–0.1)	90.6 (80.9–102.3)	13,961.1 (11,971.8–17,180.8)	2,504.1 (1,920.8–3,156.2)	1,623.5 (1,492.1–1,765.2)	0.0 (0.0–0.0)	55.3 (38.5–76.2)	-
Denmark	Prevalence Rate: Cumulative change (%	-0.7 (-7.1 to 6.3)	1.8 (-33.4 to 36.6)	1.6 (-7.1 to 10.6)	-61.9 (-79.4 to -35.5)	12.9 (5.0–21.9)	-7.8 (-26.8 to 17.2)	-24.6 (-34.5 to -13.0)	-5.8 (-13.6 to 2.6)	0.0 (-0.0 to 0.0)	0.5 (-6.9 to 7.4)	-
Denmark	YLDs Number: 1990	7,782 (5,572–10,491)	2,293 (1,297–3,624)	1,645 (1,169–2,200)	0 (0–0)	38 (25–53)	269 (155–425)	434 (275–612)	225 (165–296)	0 (0–0)	29 (16–47)	30 (20–45)
Denmark	YLDs Number: 2015	7,777 (5,486–10,483)	2,392 (1,352–3,980)	1,753 (1,253–2,356)	0 (0–0)	44 (30–62)	276 (157–468)	344 (220–500)	172 (123–230)	0 (0–0)	30 (16–47)	24 (15–35)
Denmark	YLDs Number: Cumulative change (%)	0.5 (-22.5 to 18.4)	9.5 (-50.3 to 75.4)	6.6 (1.2–12.4)	-62.1 (-79.7 to -35.8)	16.1 (-10.8 to 47.3)	7.6 (-50.4 to 68.4)	-20.6 (-31.7 to -7.2)	-23.4 (-35.1 to -10.8)	10.5 (-46.6 to 103.3)	3.1 (-32.5 to 45.2)	-17.9 (-48.2 to 20.8)

© 2017 Global Burden of Disease Child and Adolescent Health Collaboration. JAMA Pediatrics.

eTable 4. Prevalent cases, Rates (per 100,000 population), Years Lived with Disability (YLDs), and Cumulative Percent Change with 95% Uncertainty Interval (UI) for the Top 10 Global Causes of YLDsin Children and Adolescents in 195 Countries and Territories, Aged Under 5 Years, Both Sexes, 1990 and 2015. *Note: best viewed by enlarging in browser.*

Location	Measure	All causes	Iron-deficiency anemia	Skin and subcutaneous diseases	Protein-energy malnutrition	Diarrheal diseases	Hemoglobinopathies and hemolytic anemias	Asthma	Neonatal preterm birth complications	Malaria	Neonatal encephalopathy due to birth asphyxia and trauma	Other neonatal disorders
Denmark	YLDs Rate: 1990	2,712.9 (1,940.0–3,659.2)	792.7 (442.7–1,253.3)	578.7 (411.4–773.8)	0.0 (0.0–0.1)	13.4 (9.0–18.7)	93.0 (53.3–147.7)	153.4 (97.2–216.0)	78.7 (57.5–103.4)	0.0 (0.0–0.0)	10.4 (5.8–16.4)	10.8 (7.1–15.8)
Denmark	YLDs Rate: 2015	2,623.1 (1,849.5–3,533.5)	811.6 (459.1–1,344.5)	588.1 (420.3–790.3)	0.0 (0.0–0.0)	15.0 (10.2–21.1)	93.6 (53.6–158.3)	115.4 (73.8–167.5)	58.2 (41.8–77.5)	0.0 (0.0–0.0)	10.1 (5.6–16.0)	8.3 (5.4–12.1)
Denmark	YLDs Rate: Cumulative change (%)	-2.8 (-24.9 to 14.7)	7.7 (-51.1 to 73.5)	1.7 (-3.5 to 7.1)	-61.7 (-79.4 to -35.4)	13.4 (-12.9 to 44.1)	5.8 (-51.0 to 67.3)	-24.5 (-35.2 to -11.8)	-25.8 (-37.1 to -13.5)	5.9 (-48.7 to 95.9)	-0.2 (-34.8 to 40.6)	-20.5 (-49.8 to 17.0)
Finland	Prevalence Number: 1990	213,295 (206,040–221,617)	100,583 (81,843–122,826)	54,481 (51,084–58,340)	0 (0–0)	278 (249–310)	46,659 (41,189–55,794)	17,258 (13,327–21,734)	5,813 (5,371–6,334)	0 (0–0)	189 (134–257)	-
Finland	Prevalence Number: 2015	199,238 (190,761–207,729)	84,384 (65,799–103,289)	51,011 (47,871–54,605)	0 (0–0)	206 (190–223)	40,007 (34,131–50,014)	14,962 (11,164–19,019)	4,819 (4,416–5,275)	0 (0–0)	175 (121–243)	-
Finland	Prevalence Number: Cumulative change (%	-6.6 (-11.4 to -1.7)	-15.5 (-34.5 to 4.1)	-6.2 (-14.3 to 1.9)	-52.6 (-74.2 to -22.2)	-25.7 (-30.0 to -21.5)	-13.6 (-31.4 to 8.4)	-13.0 (-28.2 to 2.9)	-17.0 (-24.6 to -9.0)	-6.9 (-6.9 to -6.8)	-7.9 (-14.7 to -1.6)	-
Finland	Prevalence Rate: 1990	67,989.5 (65,675.5–70,641.4)	32,084.4 (26,111.2–39,168.8)	17,353.0 (16,270.6–18,582.3)	0.1 (0.0–0.1)	88.8 (79.6–99.1)	14,875.2 (13,130.8–17,787.0)	5,493.1 (4,241.8–6,917.6)	1,860.6 (1,719.3–2,026.5)	0.0 (0.0–0.0)	60.6 (43.0–82.2)	-
Finland	Prevalence Rate: 2015	68,265.4 (65,372.3–71,173.5)	28,973.4 (22,641.9–35,439.4)	17,449.5 (16,375.4–18,680.1)	0.0 (0.0–0.0)	71.0 (65.5–76.7)	13,714.0 (11,701.3–17,145.0)	5,109.4 (3,812.4–6,494.8)	1,668.3 (1,529.4–1,825.1)	0.0 (0.0–0.0)	60.3 (41.9–83.4)	-
Finland	Prevalence Rate: Cumulative change (%	0.4 (-4.7 to 5.6)	-9.1 (-29.5 to 12.0)	0.7 (-7.9 to 9.4)	-48.8 (-72.2 to -15.9)	-20.0 (-24.6 to -15.5)	-7.1 (-26.2 to 16.6)	-6.6 (-22.9 to 10.4)	-10.2 (-18.4 to -1.7)	-0.0 (-0.0 to 0.0)	-0.8 (-8.2 to 6.1)	-
Finland	YLDs Number: 1990	9,102 (6,522–12,118)	2,900 (1,763–4,359)	1,708 (1,225–2,310)	0 (0–0)	46 (31–64)	335 (205–493)	793 (505–1,137)	265 (196–348)	0 (0–0)	35 (20–56)	86 (55–126)
Finland	YLDs Number: 2015	7,935 (5,641–10,578)	2,372 (1,394–3,642)	1,599 (1,144–2,152)	0 (0–0)	34 (22–47)	275 (160–419)	689 (433–1,001)	170 (123–225)	0 (0–0)	32 (18–52)	81 (53–120)
Finland	YLDs Number: Cumulative change (%)	-12.6 (-22.7 to -2.4)	-17.0 (-40.2 to 11.4)	-6.3 (-10.6 to -2.1)	-52.7 (-74.4 to -22.2)	-25.5 (-43.9 to -6.3)	-16.6 (-41.7 to 12.6)	-12.8 (-28.3 to 4.3)	-35.3 (-45.0 to -24.3)	-9.1 (-34.3 to 23.7)	-8.7 (-38.7 to 28.1)	-2.2 (-39.4 to 45.5)
Finland	YLDs Rate: 1990	2,901.7 (2,079.0–3,863.6)	925.6 (562.7–1,391.9)	543.8 (390.1–735.5)	0.0 (0.0–0.0)	14.8 (10.1–20.6)	106.9 (65.4–157.3)	252.7 (160.9–362.0)	84.5 (62.5–111.1)	0.0 (0.0–0.0)	11.5 (6.6–18.0)	27.7 (17.7–40.3)
Finland	YLDs Rate: 2015	2,719.5 (1,933.3–3,626.6)	815.6 (479.7–1,249.4)	546.7 (391.0–735.4)	0.0 (0.0–0.0)	11.7 (7.9–16.3)	94.7 (55.4–144.1)	235.4 (148.1–342.0)	58.6 (42.4–77.2)	0.0 (0.0–0.0)	11.0 (6.2–17.9)	28.0 (18.3–41.4)
Finland	YLDs Rate: Cumulative change (%)	-6.1 (-16.9 to 4.9)	-10.6 (-35.5 to 19.8)	0.6 (-4.0 to 5.1)	-48.8 (-72.3 to -15.9)	-19.8 (-39.5 to 0.7)	-10.2 (-37.1 to 21.2)	-6.4 (-23.1 to 11.9)	-30.4 (-40.8 to -18.7)	-2.4 (-29.4 to 32.9)	-1.8 (-34.0 to 37.8)	5.1 (-34.9 to 56.5)
France	Prevalence Number: 1990	2,331,138 (2,230,879–2,443,845)	938,398 (752,556–1,159,326)	575,890 (535,287–623,650)	8 (5–11)	4,864 (4,446–5,324)	407,749 (385,397–434,658)	118,918 (95,894–148,636)	68,026 (62,285–74,079)	0 (0–0)	2,027 (1,543–2,615)	-
France	Prevalence Number: 2015	2,473,095 (2,385,560–2,572,229)	1,054,434 (883,258–1,256,654)	599,921 (558,138–647,087)	5 (3–9)	4,509 (4,107–4,939)	365,769 (346,491–389,266)	101,917 (77,809–128,391)	67,282 (61,321–73,751)	0 (0–0)	2,123 (1,624–2,724)	-
France	Prevalence Number: Cumulative change (%	6.2 (-0.2 to 12.4)	14.6 (-13.7 to 49.1)	4.4 (-5.6 to 15.1)	-27.3 (-56.4 to 10.7)	-7.3 (-10.8 to -3.6)	-10.2 (-17.1 to -2.9)	-14.1 (-26.3 to 1.1)	-1.0 (-9.8 to 8.1)	4.0 (3.9–4.0)	4.8 (-2.0 to 12.1)	-
France	Prevalence Rate: 1990	61,863.2 (59,230.1–64,858.0)	25,009.7 (20,066.9–30,869.6)	15,236.2 (14,163.4–16,500.1)	0.2 (0.1–0.3)	129.7 (118.6–141.9)	10,826.5 (10,234.1–11,541.3)	3,134.3 (2,527.5–3,917.6)	1,839.1 (1,684.5–2,001.6)	0.0 (0.0–0.0)	54.1 (41.2–69.8)	-
France	Prevalence Rate: 2015	63,036.7 (60,805.3–65,542.1)	26,951.8 (22,623.7–32,114.6)	15,262.8 (14,198.7–16,462.3)	0.1 (0.1–0.2)	115.2 (105.1–126.2)	9,327.7 (8,835.6–9,927.8)	2,586.5 (1,974.6–3,258.3)	1,736.8 (1,584.1–1,902.9)	0.0 (0.0–0.0)	54.3 (41.5–69.8)	-
France	Prevalence Rate: Cumulative change (%	2.0 (-4.1 to 8.0)	9.9 (-17.3 to 42.6)	0.4 (-9.2 to 10.7)	-30.6 (-58.4 to 5.7)	-11.1 (-14.4 to -7.5)	-13.7 (-20.4 to -6.8)	-17.3 (-29.0 to -2.7)	-5.4 (-13.8 to 3.1)	-0.0 (-0.0 to 0.0)	0.5 (-6.1 to 7.5)	-
France	YLDs Number: 1990	88,761 (65,041–118,813)	25,238 (14,844–39,306)	14,659 (10,296–19,788)	1 (0–1)	804 (554–1,087)	1,583 (919–2,493)	5,488 (3,614–7,927)	2,935 (2,168–3,905)	0 (0–0)	381 (223–584)	289 (199–393)
France	YLDs Number: 2015	91,613 (66,879–121,779)	28,747 (18,656–42,993)	15,270 (10,739–20,541)	0 (0–1)	749 (521–1,016)	1,762 (1,156–2,584)	4,706 (2,997–6,738)	2,382 (1,721–3,186)	0 (0–0)	392 (225–588)	1,344 (859–1,942)
France	YLDs Number: Cumulative change (%)	3.8 (-12.3 to 23.1)	19.5 (-27.8 to 87.7)	4.2 (-0.5 to 9.3)	-27.3 (-56.5 to 10.8)	-6.0 (-23.1 to 13.9)	16.9 (-28.4 to 75.6)	-14.0 (-27.9 to 2.2)	-18.6 (-30.1 to -5.8)	9.8 (-22.7 to 54.6)	5.6 (-30.2 to 50.6)	370.2 (214.3–554.6)
France	YLDs Rate: 1990	2,356.6 (1,726.4–3,156.6)	674.2 (396.2–1,049.4)	387.1 (271.9–522.5)	0.0 (0.0–0.0)	21.4 (14.8–29.0)	42.3 (24.6–66.5)	144.7 (95.3–208.9)	77.9 (57.6–103.6)	0.0 (0.0–0.0)	10.1 (5.9–15.5)	7.7 (5.3–10.5)
France	YLDs Rate: 2015	2,336.4 (1,704.6–3,105.8)	735.9 (479.6–1,100.4)	388.0 (272.9–522.1)	0.0 (0.0–0.0)	19.2 (13.3–26.0)	45.1 (29.6–66.1)	119.4 (76.1–171.0)	60.7 (43.9–81.2)	0.0 (0.0–0.0)	10.0 (5.8–15.0)	34.3 (21.9–49.5)
France	YLDs Rate: Cumulative change (%)	-0.3 (-15.8 to 18.5)	14.5 (-30.9 to 80.5)	0.3 (-4.2 to 5.1)	-30.6 (-58.4 to 5.8)	-9.9 (-26.2 to 9.1)	12.0 (-31.3 to 68.8)	-17.2 (-30.6 to -1.6)	-21.9 (-32.9 to -9.6)	5.6 (-25.6 to 48.6)	1.3 (-32.9 to 44.1)	351.6 (201.9–528.1)
Germany	Prevalence Number: 1990	2,707,494 (2,614,624–2,808,312)	1,295,247 (1,069,663–1,570,974)	688,606 (648,753–726,623)	2 (1–4)	3,338 (3,081–3,603)	472,577 (425,709–523,193)	146,075 (115,422–179,112)	73,106 (66,807–80,028)	0 (0–0)	2,337 (1,612–3,212)	-
Germany	Prevalence Number: 2015	2,209,829 (2,102,033–2,351,970)	1,070,345 (856,967–1,449,539)	553,120 (519,986–584,624)	1 (1–2)	3,022 (2,763–3,295)	328,922 (283,410–383,103)	77,395 (59,892–96,158)	55,253 (50,526–60,443)	0 (0–0)	1,801 (1,272–2,432)	-
Germany	Prevalence Number: Cumulative change (%	-18.3 (-23.4 to -12.5)	-16.3 (-42.3 to 11.2)	-19.6 (-25.5 to -13.4)	-33.0 (-60.6 to 5.1)	-9.4 (-14.0 to -4.3)	-30.2 (-41.6 to -18.0)	-47.0 (-53.1 to -39.5)	-24.3 (-31.0 to -17.2)	-20.3 (-20.5 to -20.2)	-22.7 (-28.8 to -16.3)	-
Germany	Prevalence Rate: 1990	64,613.4 (62,398.4–67,023.1)	31,032.0 (25,617.4–37,619.5)	16,380.9 (15,434.1–17,284.6)	0.1 (0.0–0.1)	79.9 (73.8–86.2)	11,281.6 (10,162.5–12,490.4)	3,461.7 (2,735.3–4,244.6)	1,776.5 (1,624.2–1,943.1)	0.0 (0.0–0.0)	56.1 (38.6–77.0)	-
Germany	Prevalence Rate: 2015	65,899.0 (62,684.7–70,137.6)	31,920.0 (25,557.6–43,227.1)	16,493.5 (15,505.5–17,432.9)	0.1 (0.0–0.1)	90.1 (82.4–98.3)	9,808.8 (8,451.6–11,424.6)	2,307.9 (1,786.0–2,867.4)	1,647.9 (1,506.9–1,802.7)	0.0 (0.0–0.0)	53.7 (38.0–72.5)	-
Germany	Prevalence Rate: Cumulative change (%	2.0 (-4.3 to 9.4)	4.2 (-28.0 to 38.5)	0.8 (-6.6 to 8.5)	-17.9 (-51.8 to 28.8)	12.8 (7.3–19.1)	-12.8 (-27.0 to 2.4)	-33.3 (-41.0 to -23.8)	-7.1 (-15.2 to 1.6)	0.0 (-0.0 to 0.0)	-4.0 (-11.4 to 3.8)	-
Germany	YLDs Number: 1990	109,843 (79,252–146,690)	36,884 (22,771–55,778)	18,590 (13,336–24,767)	0 (0–0)	550 (364–777)	1,557 (988–2,292)	6,732 (4,338–9,580)	3,363 (2,433–4,440)	0 (0–0)	446 (248–708)	276 (178–395)
Germany	YLDs Number: 2015	88,450 (62,780–124,916)	32,091 (17,952–55,974)	14,870 (10,655–19,828)	0 (0–0)	501 (348–696)	1,281 (731–2,168)	3,569 (2,275–5,044)	2,045 (1,477–2,749)	0 (0–0)	329 (178–513)	367 (239–528)
Germany	YLDs Number: Cumulative change (%)	-19.1 (-36.0 to -1.3)	-10.3 (-51.3 to 36.0)	-20.0 (-23.7 to -16.2)	-33.0 (-60.6 to 5.3)	-7.2 (-31.1 to 24.0)	-15.3 (-52.0 to 29.8)	-46.9 (-54.0 to -38.6)	-39.0 (-47.9 to -28.8)	-12.1 (-41.4 to 22.7)	-23.7 (-50.9 to 10.5)	37.1 (-10.1 to 99.5)
Germany	YLDs Rate: 1990	2,622.9 (1,891.6–3,502.4)	885.8 (546.2–1,339.4)	441.3 (316.5–588.1)	0.0 (0.0–0.0)	13.2 (8.7–18.6)	37.4 (23.7–55.0)	159.6 (102.8–227.0)	80.3 (58.1–106.0)	0.0 (0.0–0.0)	10.7 (5.9–16.9)	6.6 (4.3–9.4)
Germany	YLDs Rate: 2015	2,637.7 (1,872.2–3,725.2)	957.1 (535.4–1,669.2)	443.4 (317.7–591.3)	0.0 (0.0–0.0)	14.9 (10.4–20.8)	38.2 (21.8–64.7)	106.5 (67.9–150.4)	61.0 (44.0–82.0)	0.0 (0.0–0.0)	9.8 (5.3–15.3)	11.0 (7.1–15.8)
Germany	YLDs Rate: Cumulative change (%)	1.1 (-20.1 to 23.2)	11.4 (-39.4 to 69.2)	0.5 (-4.1 to 5.3)	-17.9 (-51.8 to 29.0)	15.5 (-14.0 to 53.8)	5.2 (-40.4 to 61.1)	-33.1 (-42.1 to -22.7)	-23.7 (-34.8 to -11.1)	10.3 (-26.5 to 53.8)	-4.7 (-38.6 to 37.9)	71.2 (12.3–149.1)
Greece	Prevalence Number: 1990	359,232 (348,633–370,291)	158,677 (132,805–192,872)	79,383 (73,965–85,716)	1 (0–2)	273 (255–291)	92,157 (88,005–96,272)	13,935 (10,752–17,106)	10,378 (9,295–11,580)	0 (0–0)	306 (214–416)	-
Greece	Prevalence Number: 2015	323,851 (305,926–342,438)	126,031 (90,729–175,701)	73,155 (67,734–79,480)	0 (0–0)	200 (189–213)	66,171 (62,908–69,428)	13,500 (10,357–16,874)	8,667 (7,779–9,724)	0 (0–0)	280 (200–377)	-
Greece	Prevalence Number: Cumulative change (%	-9.8 (-15.3 to -4.5)	-20.0 (-45.8 to 13.8)	-7.7 (-16.9 to 1.7)	-92.5 (-95.2 to -89.0)	-26.5 (-29.6 to -23.6)	-28.1 (-32.6 to -23.1)	-3.0 (-13.4 to 7.2)	-16.4 (-24.2 to -7.9)	-8.3 (-8.4 to -8.2)	-8.2 (-15.4 to -1.0)	-
Greece	Prevalence Rate: 1990	65,646.8 (63,771.9–67,651.4)	29,226.8 (24,523.4–35,417.0)	14,414.7 (13,431.8–15,562.2)	0.3 (0.2–0.5)	50.6 (47.3–53.9)	16,848.9 (16,090.8–17,605.8)	2,509.3 (1,936.1–3,080.2)	1,970.1 (1,764.6–2,195.5)	0.0 (0.0–0.0)	56.8 (39.8–76.9)	-
Greece	Prevalence Rate: 2015	64,751.3 (61,178.6–68,430.3)	25,602.7 (18,629.2–35,420.4)	14,504.3 (13,429.4–15,756.7)	0.0 (0.0–0.0)	40.9 (38.5–43.4)	13,247.2 (12,594.4–13,900.9)	2,646.0 (2,030.0–3,307.3)	1,831.4 (1,643.4–2,053.6)	0.0 (0.0–0.0)	57.2 (40.7–77.1)	-

© 2017 Global Burden of Disease Child and Adolescent Health Collaboration. JAMA Pediatrics.

eTable 4. Prevalent cases, Rates (per 100,000 population), Years Lived with Disability (YLDs), and Cumulative Percent Change with 95% Uncertainty Interval (UI) for the Top 10 Global Causes of YLDsin Children and Adolescents in 195 Countries and Territories, Aged Under 5 Years, Both Sexes, 1990 and 2015. *Note: best viewed by enlarging in browser.*

Location	Measure	All causes	Iron-deficiency anemia	Skin and subcutaneous diseases	Protein-energy malnutrition	Diarrheal diseases	Hemoglobinopathies and hemolytic anemias	Asthma	Neonatal preterm birth complications	Malaria	Neonatal encephalopathy due to birth asphyxia and trauma	Other neonatal disorders
Greece	Prevalence Rate: Cumulative change (%	-1.3 (-7.2 to 4.4)	-11.8 (-39.6 to 24.9)	0.8 (-9.3 to 11.0)	-91.7 (-94.7 to -87.7)	-19.1 (-22.5 to -16.0)	-21.3 (-26.2 to -15.9)	5.6 (-5.8 to 16.7)	-6.9 (-15.4 to 2.1)	0.0 (-0.0 to 0.0)	1.0 (-7.1 to 9.4)	-
Greece	YLDs Number: 1990	13,622 (9,903–18,167)	4,467 (2,834–6,700)	1,837 (1,274–2,487)	0 (0–0)	45 (30–63)	492 (314–725)	643 (410–912)	545 (410–706)	0 (0–0)	60 (34–95)	12 (8–16)
Greece	YLDs Number: 2015	11,042 (7,721–15,281)	3,555 (1,868–6,033)	1,691 (1,180–2,270)	0 (0–0)	33 (22–46)	374 (197–639)	623 (400–883)	355 (261–464)	0 (0–0)	53 (29–82)	27 (18–40)
Greece	YLDs Number: Cumulative change (%)	-18.7 (-32.8 to 1.3)	-18.8 (-51.0 to 32.4)	-7.9 (-11.9 to -3.4)	-92.5 (-95.2 to -89.0)	-25.5 (-44.8 to -2.7)	-22.6 (-54.3 to 27.8)	-2.8 (-15.1 to 10.6)	-34.7 (-43.7 to -24.9)	-11.9 (-47.6 to 31.4)	-7.8 (-37.3 to 29.5)	131.4 (54.4–227.1)
Greece	YLDs Rate: 1990	2,494.3 (1,813.0–3,324.2)	826.6 (527.5–1,234.7)	332.5 (230.8–450.6)	0.0 (0.0–0.1)	8.4 (5.6–11.7)	91.2 (58.2–133.8)	115.8 (74.0–164.2)	99.7 (75.2–129.2)	0.0 (0.0–0.0)	11.0 (6.3–17.4)	2.2 (1.5–3.1)
Greece	YLDs Rate: 2015	2,218.1 (1,552.0–3,073.2)	728.2 (390.2–1,231.4)	333.8 (232.8–448.2)	0.0 (0.0–0.0)	6.8 (4.5–9.4)	76.7 (40.9–130.1)	122.3 (78.6–173.1)	71.0 (52.3–92.7)	0.0 (0.0–0.0)	10.8 (6.0–16.6)	5.5 (3.7–8.2)
Greece	YLDs Rate: Cumulative change (%)	-10.8 (-26.1 to 11.1)	-10.2 (-45.0 to 45.6)	0.5 (-3.9 to 5.2)	-91.7 (-94.8 to -87.7)	-18.1 (-39.0 to 6.6)	-14.3 (-48.7 to 40.3)	5.8 (-7.6 to 20.4)	-28.6 (-38.4 to -17.8)	-3.8 (-42.7 to 43.1)	0.8 (-31.1 to 41.0)	153.2 (69.2–257.3)
Iceland	Prevalence Number: 1990	14,789 (14,094–15,413)	5,975 (5,127–7,399)	3,096 (2,861–3,346)	0 (0–0)	14 (12–16)	3,132 (2,749–3,716)	741 (576–915)	362 (325–403)	0 (0–0)	10 (7–12)	-
Iceland	Prevalence Number: 2015	15,537 (14,831–16,137)	7,224 (6,121–8,183)	3,244 (3,006–3,523)	0 (0–0)	13 (11–14)	3,036 (2,626–3,691)	639 (488–808)	340 (304–382)	0 (0–0)	10 (7–13)	-
Iceland	Prevalence Number: Cumulative change (%	5.1 (-1.3 to 11.7)	22.6 (-14.9 to 49.4)	5.0 (-5.4 to 16.2)	-57.2 (-73.6 to -33.1)	-8.4 (-12.5 to -3.8)	-2.4 (-21.5 to 19.9)	-13.5 (-26.2 to 0.2)	-5.8 (-14.4 to 3.4)	4.2 (4.0–4.5)	4.0 (-4.2 to 12.1)	-
Iceland	Prevalence Rate: 1990	69,079.0 (65,831.8–71,993.3)	27,889.3 (23,925.2–34,533.7)	14,466.8 (13,372.7–15,639.4)	0.1 (0.0–0.1)	67.8 (59.9–75.7)	14,631.1 (12,840.1–17,355.0)	3,469.0 (2,696.7–4,278.9)	1,685.6 (1,514.0–1,880.1)	0.0 (0.0–0.0)	47.2 (36.2–60.1)	-
Iceland	Prevalence Rate: 2015	69,953.6 (66,804.4–72,657.6)	32,664.4 (27,755.4–36,929.8)	14,568.3 (13,503.1–15,818.6)	0.0 (0.0–0.1)	60.1 (53.6–67.5)	13,686.3 (11,834.8–16,635.0)	2,859.8 (2,184.7–3,612.3)	1,566.1 (1,399.6–1,756.8)	0.0 (0.0–0.0)	47.7 (36.0–61.1)	-
Iceland	Prevalence Rate: Cumulative change (%	1.3 (-4.9 to 7.6)	18.8 (-17.6 to 44.4)	0.9 (-9.1 to 11.7)	-57.8 (-74.0 to -33.9)	-11.2 (-15.2 to -6.7)	-5.8 (-24.3 to 15.7)	-17.3 (-29.4 to -4.2)	-6.9 (-15.4 to 2.0)	0.0 (-0.0 to 0.0)	1.1 (-6.8 to 9.1)	-
Iceland	YLDs Number: 1990	526 (383–703)	170 (109–259)	69 (48–94)	0 (0–0)	2 (1–3)	18 (11–27)	34 (21–48)	16 (12–21)	0 (0–0)	1 (1–2)	0 (0–0)
Iceland	YLDs Number: 2015	565 (409–747)	214 (137–300)	73 (50–98)	0 (0–0)	2 (1–3)	23 (15–32)	29 (18–42)	12 (9–16)	0 (0–0)	2 (1–3)	2 (1–3)
Iceland	YLDs Number: Cumulative change (%)	8.0 (-15.9 to 22.7)	30.3 (-28.8 to 76.2)	4.7 (0.3–9.9)	-57.2 (-73.6 to -32.8)	-6.2 (-30.2 to 21.6)	33.4 (-26.5 to 77.4)	-13.8 (-27.7 to 1.5)	-22.4 (-33.8 to -10.0)	11.0 (-20.6 to 48.5)	4.6 (-30.3 to 49.0)	240.0 (114.5–407.1)
Iceland	YLDs Rate: 1990	2,459.3 (1,789.5–3,286.4)	795.2 (512.5–1,212.1)	326.6 (225.8–440.2)	0.0 (0.0–0.0)	11.1 (7.5–15.6)	84.2 (54.2–128.5)	160.2 (102.5–225.0)	75.2 (56.1–98.8)	0.0 (0.0–0.0)	9.1 (5.6–13.8)	2.9 (1.9–4.2)
Iceland	YLDs Rate: 2015	2,551.6 (1,846.7–3,368.1)	974.3 (623.2–1,364.7)	327.8 (228.5–439.7)	0.0 (0.0–0.0)	10.0 (6.5–14.2)	105.2 (68.9–146.7)	131.6 (84.1–190.3)	56.2 (40.9–75.0)	0.0 (0.0–0.0)	9.0 (5.4–14.0)	9.2 (5.6–13.7)
Iceland	YLDs Rate: Cumulative change (%)	4.3 (-18.8 to 18.3)	26.6 (-31.0 to 70.2)	0.4 (-3.8 to 5.4)	-57.8 (-74.1 to -33.7)	-9.0 (-32.1 to 17.9)	29.5 (-28.8 to 72.0)	-17.5 (-30.9 to -2.9)	-25.1 (-36.1 to -13.2)	6.5 (-23.8 to 42.4)	0.9 (-32.6 to 43.5)	227.9 (107.0–389.3)
Ireland	Prevalence Number: 1990	185,718 (177,940–193,341)	73,192 (49,720–92,543)	47,312 (44,286–50,641)	0 (0–0)	186 (169–208)	42,313 (37,176–50,267)	13,485 (10,480–16,644)	4,971 (4,557–5,450)	0 (0–0)	209 (166–261)	-
Ireland	Prevalence Number: 2015	240,950 (230,091–252,621)	104,771 (80,319–132,320)	61,250 (57,424–65,481)	0 (0–0)	273 (240–306)	49,670 (43,149–60,659)	12,403 (9,547–15,582)	5,345 (4,907–5,843)	0 (0–0)	266 (211–330)	-
Ireland	Prevalence Number: Cumulative change (%	29.8 (21.9–38.4)	51.0 (-4.9 to 140.9)	29.6 (18.4–41.7)	-58.3 (-74.8 to -33.5)	46.3 (36.0–59.0)	18.1 (-4.0 to 45.0)	-7.8 (-19.2 to 4.7)	7.7 (-1.9 to 18.5)	26.9 (26.5–27.2)	27.7 (19.2–36.2)	-
Ireland	Prevalence Rate: 1990	66,796.9 (64,065.7–69,541.6)	26,754.3 (18,538.3–33,880.0)	16,831.9 (15,762.4–18,012.8)	0.1 (0.1–0.1)	68.1 (61.8–75.6)	15,239.2 (13,387.9–18,103.4)	4,747.8 (3,690.0–5,860.2)	1,897.0 (1,740.2–2,073.7)	0.0 (0.0–0.0)	76.6 (61.0–95.3)	-
Ireland	Prevalence Rate: 2015	67,850.2 (64,812.6–71,140.7)	29,696.7 (22,791.0–37,361.4)	17,160.1 (16,091.6–18,340.2)	0.0 (0.0–0.0)	77.5 (68.3–86.7)	14,003.2 (12,170.4–17,097.3)	3,456.2 (2,660.4–4,341.9)	1,554.2 (1,429.5–1,694.8)	0.0 (0.0–0.0)	75.9 (60.1–94.1)	-
Ireland	Prevalence Rate: Cumulative change (%	1.6 (-4.5 to 8.3)	16.7 (-25.7 to 82.0)	2.1 (-6.8 to 11.6)	-67.8 (-80.6 to -48.4)	13.9 (6.0–23.8)	-7.5 (-24.8 to 13.5)	-27.1 (-36.1 to -17.1)	-17.9 (-25.0 to -10.0)	-0.0 (-0.0 to 0.0)	-0.8 (-7.5 to 5.9)	-
Ireland	YLDs Number: 1990	7,631 (5,495–10,071)	2,086 (995–3,247)	1,465 (1,049–1,967)	0 (0–0)	30 (20–43)	246 (122–378)	621 (399–889)	217 (158–283)	0 (0–0)	39 (25–57)	14 (9–19)
Ireland	YLDs Number: 2015	9,475 (6,788–12,760)	2,986 (1,762–4,550)	1,862 (1,331–2,506)	0 (0–0)	45 (30–62)	342 (200–514)	571 (364–812)	178 (128–238)	0 (0–0)	48 (30–71)	48 (32–68)
Ireland	YLDs Number: Cumulative change (%)	25.6 (-1.9 to 70.6)	61.8 (-27.4 to 290.9)	27.2 (21.5–33.1)	-58.2 (-74.8 to -33.3)	48.1 (12.7–92.7)	54.4 (-20.8 to 273.0)	-7.9 (-19.5 to 6.1)	-17.9 (-31.3 to -4.9)	44.8 (-26.8 to 217.4)	24.6 (-12.6 to 71.0)	248.9 (135.6–386.8)
Ireland	YLDs Rate: 1990	2,751.7 (1,982.0–3,625.6)	769.8 (373.2–1,201.9)	518.2 (371.2–695.4)	0.0 (0.0–0.0)	11.2 (7.5–15.8)	90.9 (45.8–138.1)	218.7 (140.6–313.2)	78.3 (57.2–102.0)	0.0 (0.0–0.0)	14.3 (9.1–20.9)	5.1 (3.5–7.1)
Ireland	YLDs Rate: 2015	2,671.5 (1,911.8–3,589.5)	849.7 (504.7–1,290.4)	520.3 (372.2–700.1)	0.0 (0.0–0.0)	12.8 (8.7–17.6)	97.5 (57.3–146.2)	159.1 (101.7–226.3)	50.2 (36.4–67.2)	0.0 (0.0–0.0)	13.7 (8.7–20.1)	13.6 (9.1–19.4)
Ireland	YLDs Rate: Cumulative change (%)	-1.8 (-23.5 to 33.4)	24.2 (-44.0 to 194.9)	0.4 (-4.0 to 5.1)	-67.8 (-80.6 to -47.8)	15.3 (-12.1 to 49.5)	18.6 (-38.2 to 181.2)	-27.1 (-36.3 to -16.0)	-35.7 (-46.1 to -25.6)	13.6 (-42.1 to 147.5)	-2.5 (-31.2 to 33.1)	173.1 (84.4–280.9)
Israel	Prevalence Number: 1990	341,002 (327,679–353,002)	178,470 (136,037–210,813)	75,114 (69,851–80,358)	2 (1–4)	428 (385–483)	75,346 (71,985–78,976)	18,752 (14,721–23,122)	9,452 (8,460–10,561)	0 (0–0)	272 (190–370)	-
Israel	Prevalence Number: 2015	578,369 (559,806–596,403)	292,078 (245,980–337,885)	129,151 (119,985–138,670)	3 (2–5)	627 (562–708)	116,858 (110,898–122,180)	23,085 (17,715–28,662)	14,341 (12,796–15,995)	0 (0–0)	457 (322–617)	-
Israel	Prevalence Number: Cumulative change (%	69.7 (62.0–77.6)	65.7 (27.9–100.0)	72.2 (56.7–88.5)	35.6 (-8.6 to 90.9)	46.7 (38.9–54.1)	55.2 (45.8–65.1)	23.4 (7.7–40.4)	52.0 (39.5–66.5)	70.5 (70.4–70.6)	68.4 (57.4–80.4)	-
Israel	Prevalence Rate: 1990	71,030.6 (68,227.8–73,531.5)	37,122.6 (28,311.7–43,858.6)	15,670.6 (14,571.2–16,761.7)	0.6 (0.4–0.9)	88.9 (80.1–100.5)	15,690.4 (14,992.4–16,446.0)	3,919.8 (3,077.1–4,833.2)	1,950.5 (1,745.8–2,182.4)	0.0 (0.0–0.0)	56.5 (39.5–76.9)	-
Israel	Prevalence Rate: 2015	70,776.3 (68,500.1–72,982.3)	35,730.6 (30,091.5–41,333.7)	15,808.2 (14,686.3–16,972.7)	0.5 (0.3–0.7)	76.8 (68.8–86.7)	14,299.5 (13,570.2–14,950.9)	2,826.9 (2,169.3–3,509.8)	1,751.7 (1,563.0–1,953.7)	0.0 (0.0–0.0)	56.0 (39.5–75.6)	-
Israel	Prevalence Rate: Cumulative change (%	-0.3 (-4.8 to 4.4)	-2.6 (-24.7 to 17.9)	1.0 (-8.0 to 10.6)	-19.7 (-45.9 to 13.0)	-13.6 (-18.2 to -9.3)	-8.8 (-14.3 to -3.0)	-27.7 (-36.9 to -17.8)	-10.1 (-17.4 to -1.4)	-0.0 (-0.0 to 0.0)	-0.8 (-7.2 to 6.2)	-
Israel	YLDs Number: 1990	15,027 (10,756–20,196)	5,588 (3,209–8,323)	2,052 (1,454–2,735)	0 (0–0)	70 (49–96)	313 (176–463)	862 (553–1,231)	444 (332–583)	0 (0–0)	51 (28–79)	56 (37–80)
Israel	YLDs Number: 2015	23,456 (17,194–31,284)	8,963 (5,676–13,097)	3,519 (2,484–4,735)	0 (0–0)	103 (69–147)	498 (320–712)	1,060 (667–1,503)	527 (387–706)	0 (0–0)	83 (48–130)	256 (164–363)
Israel	YLDs Number: Cumulative change (%)	56.8 (29.6–76.5)	65.0 (4.6–121.9)	71.5 (64.2–79.6)	35.3 (-8.8 to 90.5)	47.8 (16.0–84.8)	63.9 (5.5–122.8)	23.4 (6.1–41.4)	19.0 (2.7–37.2)	73.0 (32.9–118.8)	67.5 (15.7–133.6)	362.3 (205.6–556.3)
Israel	YLDs Rate: 1990	3,129.0 (2,239.2–4,205.2)	1,161.2 (666.9–1,727.4)	428.6 (303.8–571.2)	0.1 (0.0–0.1)	14.7 (10.2–20.1)	65.1 (36.7–96.4)	180.4 (115.8–257.3)	92.6 (69.2–121.6)	0.0 (0.0–0.0)	10.7 (6.0–16.5)	11.8 (7.8–16.8)
Israel	YLDs Rate: 2015	2,870.1 (2,103.7–3,828.0)	1,096.3 (694.5–1,602.6)	430.9 (304.2–579.7)	0.1 (0.0–0.1)	12.7 (8.5–18.1)	61.0 (39.2–87.1)	129.9 (81.7–184.1)	64.6 (47.5–86.4)	0.0 (0.0–0.0)	10.3 (5.9–16.0)	31.4 (20.1–44.5)
Israel	YLDs Rate: Cumulative change (%)	-7.9 (-23.8 to 3.6)	-2.9 (-38.4 to 30.6)	0.6 (-3.7 to 5.3)	-19.9 (-46.0 to 12.8)	-13.0 (-31.7 to 8.9)	-3.5 (-37.9 to 31.1)	-27.7 (-37.8 to -17.2)	-30.0 (-39.7 to -19.4)	1.5 (-22.1 to 28.4)	-1.6 (-32.0 to 37.5)	171.7 (79.6–285.8)
Italy	Prevalence Number: 1990	1,969,694 (1,886,542–2,053,353)	871,893 (666,678–1,108,907)	385,582 (363,789–406,229)	2 (1–3)	1,859 (1,675–2,054)	743,184 (707,973–771,321)	59,282 (48,731–70,228)	48,998 (45,057–53,312)	0 (0–0)	852 (658–1,081)	-

© 2017 Global Burden of Disease Child and Adolescent Health Collaboration. JAMA Pediatrics.

eTable 4. Prevalent cases, Rates (per 100,000 population), Years Lived with Disability (YLDs), and Cumulative Percent Change with 95% Uncertainty Interval (UI) for the Top 10 Global Causes of YLDsin Children and Adolescents in 195 Countries and Territories, Aged Under 5 Years, Both Sexes, 1990 and 2015. *Note: best viewed by enlarging in browser.*

Location	Measure	All causes	Iron-deficiency anemia	Skin and subcutaneous diseases	Protein-energy malnutrition	Diarrheal diseases	Hemoglobinopathies and hemolytic anemias	Asthma	Neonatal preterm birth complications	Malaria	Neonatal encephalopathy due to birth asphyxia and trauma	Other neonatal disorders
Italy	Prevalence Number: 2015	1,785,650<br>(1,704,861–1,859,459)	738,936<br>(547,972–953,611)	355,158<br>(336,775–372,224)	5<br>(3–8)	1,652<br>(1,467–1,851)	632,547<br>(603,355–660,863)	44,577<br>(34,436–56,920)	40,122<br>(37,521–43,321)	0<br>(0–0)	739<br>(548–975)	-
Italy	Prevalence Number: Cumulative change (%	-9.3<br>(-14.7 to -3.8)	-12.8<br>(-47.4 to 19.1)	-7.8<br>(-14.1 to -1.2)	125.2<br>(43.5–234.8)	-11.1<br>(-16.0 to -5.9)	-14.8<br>(-20.1 to -9.0)	-24.8<br>(-38.1 to -11.0)	-18.0<br>(-24.9 to -10.9)	-8.4<br>(-8.5 to -8.3)	-13.5<br>(-22.0 to -3.7)	-
Italy	Prevalence Rate: 1990	69,715.0<br>(66,787.0–72,667.2)	30,959.2<br>(23,736.7–39,303.9)	13,599.1<br>(12,832.1–14,325.4)	0.1<br>(0.1–0.1)	66.1<br>(59.5–72.9)	26,312.7<br>(25,066.0–27,309.5)	2,082.9<br>(1,712.2–2,467.4)	1,767.5<br>(1,626.2–1,921.7)	0.0<br>(0.0–0.0)	30.4<br>(23.4–38.5)	-
Italy	Prevalence Rate: 2015	69,102.7<br>(66,007.4–71,931.1)	28,739.1<br>(21,361.4–36,980.9)	13,680.4<br>(12,973.9–14,338.8)	0.2<br>(0.1–0.3)	64.4<br>(57.2–72.1)	24,495.1<br>(23,367.6–25,588.8)	1,707.9<br>(1,319.4–2,180.8)	1,598.5<br>(1,497.2–1,724.9)	0.0<br>(0.0–0.0)	28.9<br>(21.4–38.1)	-
Italy	Prevalence Rate: Cumulative change (%	-0.8<br>(-6.7 to 5.1)	-4.5<br>(-42.1 to 30.1)	0.7<br>(-6.2 to 7.8)	149.6<br>(59.1–271.6)	-2.6<br>(-7.8 to 3.2)	-6.8<br>(-12.6 to -0.5)	-18.0<br>(-32.5 to -2.9)	-9.4<br>(-16.9 to -1.7)	-0.0<br>(-0.0 to 0.0)	-5.1<br>(-14.3 to 5.6)	-
Italy	YLDs Number: 1990	75,969<br>(53,254–105,633)	25,816<br>(14,937–41,231)	10,071<br>(7,226–13,555)	0<br>(0–0)	308<br>(206–425)	5,284<br>(3,115–8,304)	2,737<br>(1,795–3,806)	2,593<br>(1,955–3,415)	0<br>(0–0)	165<br>(93–256)	225<br>(158–307)
Italy	YLDs Number: 2015	63,965<br>(45,593–86,803)	21,717<br>(12,295–34,295)	9,217<br>(6,565–12,424)	0<br>(0–1)	273<br>(181–390)	4,411<br>(2,553–6,800)	2,056<br>(1,308–3,002)	1,550<br>(1,155–2,035)	0<br>(0–0)	140<br>(76–224)	715<br>(475–1,003)
Italy	YLDs Number: Cumulative change (%)	-14.6<br>(-40.7 to 4.1)	-9.8<br>(-62.8 to 37.1)	-8.4<br>(-12.9 to -3.7)	126.2<br>(44.3–236.3)	-10.2<br>(-33.7 to 17.3)	-11.1<br>(-60.8 to 33.8)	-24.7<br>(-38.7 to -8.6)	-40.0<br>(-49.1 to -29.6)	-4.1<br>(-57.9 to 48.2)	-12.6<br>(-48.2 to 34.9)	222.6<br>(126.5–343.5)
Italy	YLDs Rate: 1990	2,690.8<br>(1,885.4–3,738.6)	918.3<br>(533.0–1,461.8)	354.5<br>(254.3–477.0)	0.0<br>(0.0–0.0)	11.0<br>(7.3–15.1)	188.0<br>(111.0–294.7)	96.2<br>(63.1–133.7)	91.8<br>(69.3–120.9)	0.0<br>(0.0–0.0)	5.9<br>(3.3–9.1)	8.0<br>(5.6–10.9)
Italy	YLDs Rate: 2015	2,478.4<br>(1,771.1–3,353.8)	846.7<br>(481.4–1,331.9)	354.0<br>(252.1–477.1)	0.0<br>(0.0–0.0)	10.6<br>(7.0–15.2)	172.1<br>(100.1–264.0)	78.8<br>(50.1–115.0)	60.0<br>(44.7–78.7)	0.0<br>(0.0–0.0)	5.4<br>(2.9–8.7)	27.7<br>(18.4–38.8)
Italy	YLDs Rate: Cumulative change (%)	-6.6<br>(-34.9 to 13.9)	-1.2<br>(-58.7 to 49.7)	-0.1<br>(-4.9 to 5.0)	150.7<br>(59.7–274.0)	-1.6<br>(-27.2 to 28.2)	-2.7<br>(-56.5 to 46.1)	-17.9<br>(-33.2 to -0.3)	-34.4<br>(-44.3 to -23.1)	4.7<br>(-53.9 to 61.6)	-4.5<br>(-43.2 to 47.2)	252.8<br>(147.5–385.3)
Luxembourg	Prevalence Number: 1990	15,458<br>(14,703–16,247)	5,820<br>(4,375–8,096)	3,554<br>(3,301–3,809)	0<br>(0–0)	20<br>(18–23)	3,421<br>(3,027–4,085)	1,031<br>(818–1,251)	358<br>(329–393)	0<br>(0–0)	18<br>(14–22)	-
Luxembourg	Prevalence Number: 2015	21,227<br>(20,375–22,079)	8,964<br>(7,599–10,667)	4,919<br>(4,555–5,287)	0<br>(0–0)	26<br>(24–29)	4,331<br>(3,722–5,314)	1,084<br>(852–1,352)	421<br>(386–463)	0<br>(0–0)	27<br>(21–34)	-
Luxembourg	Prevalence Number: Cumulative change (%	37.4<br>(29.4–45.6)	57.5<br>(20.0–99.7)	38.6<br>(26.2–52.1)	-47.6<br>(-66.8 to -23.4)	26.7<br>(21.0–33.3)	27.5<br>(2.4–59.7)	5.3<br>(-7.7 to 20.2)	17.9<br>(8.0–29.1)	36.6<br>(36.5–36.6)	52.1<br>(38.2–64.9)	-
Luxembourg	Prevalence Rate: 1990	69,648.6<br>(66,244.3–73,181.0)	26,041.7<br>(19,500.0–36,270.6)	16,074.2<br>(14,934.2–17,232.5)	0.3<br>(0.2–0.4)	93.5<br>(84.6–103.9)	15,396.1<br>(13,623.4–18,388.9)	4,687.1<br>(3,721.9–5,689.0)	1,573.7<br>(1,446.0–1,730.4)	0.0<br>(0.0–0.0)	81.5<br>(64.4–101.9)	-
Luxembourg	Prevalence Rate: 2015	70,059.5<br>(67,232.7–72,897.7)	29,419.2<br>(24,826.9–35,072.0)	16,289.5<br>(15,082.9–17,510.4)	0.1<br>(0.1–0.2)	86.8<br>(79.0–96.8)	14,280.4<br>(12,273.5–17,526.6)	3,605.4<br>(2,834.8–4,496.9)	1,361.1<br>(1,246.3–1,496.8)	0.0<br>(0.0–0.0)	90.8<br>(71.0–113.5)	-
Luxembourg	Prevalence Rate: Cumulative change (%	0.7<br>(-5.2 to 6.6)	15.5<br>(-11.9 to 47.0)	1.5<br>(-7.7 to 11.4)	-61.6<br>(-75.6 to -43.9)	-7.1<br>(-11.3 to -2.3)	-6.6<br>(-25.0 to 17.1)	-22.9<br>(-32.5 to -12.0)	-13.4<br>(-20.8 to -5.0)	-0.0<br>(-0.0 to 0.0)	11.6<br>(1.4–20.9)	-
Luxembourg	YLDs Number: 1990	601<br>(424–822)	165<br>(87–283)	98<br>(70–133)	0<br>(0–0)	3<br>(2–4)	18<br>(10–30)	47<br>(30–67)	14<br>(10–19)	0<br>(0–0)	3<br>(2–4)	5<br>(3–7)
Luxembourg	YLDs Number: 2015	812<br>(593–1,078)	262<br>(166–388)	135<br>(96–181)	0<br>(0–0)	4<br>(2–6)	30<br>(19–43)	50<br>(32–72)	14<br>(10–18)	0<br>(0–0)	5<br>(3–7)	14<br>(9–21)
Luxembourg	YLDs Number: Cumulative change (%)	36.1<br>(16.3–54.6)	68.1<br>(6.3–139.3)	37.5<br>(30.9–43.9)	-47.6<br>(-66.8 to -23.3)	28.0<br>(1.9–56.3)	66.7<br>(15.7–133.3)	5.4<br>(-8.8 to 21.3)	-5.6<br>(-20.9 to 11.7)	48.0<br>(2.2–117.6)	50.2<br>(11.0–97.0)	188.9<br>(88.4–314.7)
Luxembourg	YLDs Rate: 1990	2,704.0<br>(1,908.0–3,701.2)	736.7<br>(391.1–1,266.7)	448.1<br>(320.5–604.5)	0.0<br>(0.0–0.1)	15.4<br>(10.5–21.2)	84.4<br>(46.8–138.3)	216.1<br>(139.3–305.5)	67.2<br>(48.3–88.6)	0.0<br>(0.0–0.0)	15.7<br>(10.2–22.3)	22.9<br>(15.3–32.6)
Luxembourg	YLDs Rate: 2015	2,678.9<br>(1,952.9–3,553.8)	859.3<br>(542.9–1,276.4)	450.9<br>(319.5–603.4)	0.0<br>(0.0–0.0)	14.4<br>(9.8–19.8)	98.7<br>(63.8–142.5)	166.3<br>(108.4–241.0)	46.3<br>(33.2–62.2)	0.0<br>(0.0–0.0)	17.0<br>(10.8–24.6)	47.3<br>(30.8–69.9)
Luxembourg	YLDs Rate: Cumulative change (%)	-0.3<br>(-14.6 to 13.3)	23.4<br>(-21.9 to 76.5)	0.6<br>(-4.2 to 5.4)	-61.6<br>(-75.6 to -43.7)	-6.1<br>(-25.5 to 14.8)	22.4<br>(-15.2 to 72.2)	-22.9<br>(-33.3 to -11.2)	-30.8<br>(-42.0 to -18.1)	8.4<br>(-25.2 to 59.5)	10.1<br>(-18.9 to 44.5)	111.7<br>(38.1–204.2)
Malta	Prevalence Number: 1990	20,133<br>(19,263–20,951)	8,114<br>(6,567–9,875)	4,355<br>(4,063–4,706)	0<br>(0–0)	19<br>(17–22)	4,657<br>(4,065–5,436)	1,051<br>(826–1,288)	575<br>(531–627)	0<br>(0–0)	27<br>(22–34)	-
Malta	Prevalence Number: 2015	13,065<br>(12,470–13,605)	5,889<br>(4,989–7,302)	2,845<br>(2,638–3,070)	0<br>(0–0)	14<br>(12–16)	2,759<br>(2,371–3,335)	653<br>(494–823)	342<br>(319–370)	0<br>(0–0)	18<br>(15–22)	-
Malta	Prevalence Number: Cumulative change (%	-35.1<br>(-38.7 to -31.3)	-26.5<br>(-42.1 to -4.3)	-34.6<br>(-40.9 to -27.8)	-67.2<br>(-79.4 to -49.2)	-28.6<br>(-33.4 to -22.5)	-40.4<br>(-52.3 to -26.4)	-37.7<br>(-48.4 to -26.0)	-40.3<br>(-45.3 to -35.3)	-35.8<br>(-35.9 to -35.8)	-33.0<br>(-38.3 to -27.4)	-
Malta	Prevalence Rate: 1990	70,620.2<br>(67,573.1–73,483.5)	28,569.9<br>(23,164.8–34,756.5)	15,244.5<br>(14,221.6–16,471.4)	0.3<br>(0.2–0.4)	69.6<br>(62.8–77.9)	16,346.2<br>(14,269.9–19,076.8)	3,671.3<br>(2,884.9–4,497.1)	2,044.8<br>(1,889.3–2,230.3)	0.0<br>(0.0–0.0)	98.3<br>(79.0–121.0)	-
Malta	Prevalence Rate: 2015	71,257.5<br>(68,011.6–74,203.9)	32,142.3<br>(27,236.5–39,848.6)	15,509.7<br>(14,383.2–16,739.3)	0.1<br>(0.1–0.2)	77.0<br>(69.0–88.4)	15,051.6<br>(12,937.2–18,193.6)	3,558.4<br>(2,692.4–4,487.9)	1,873.9<br>(1,750.0–2,024.2)	0.0<br>(0.0–0.0)	101.8<br>(83.0–122.5)	-
Malta	Prevalence Rate: Cumulative change (%	1.0<br>(-4.7 to 6.7)	13.9<br>(-10.2 to 48.1)	1.9<br>(-7.9 to 12.5)	-49.5<br>(-68.3 to -21.5)	10.7<br>(3.2–20.1)	-7.3<br>(-25.8 to 14.4)	-2.8<br>(-19.4 to 15.5)	-8.2<br>(-15.8 to -0.6)	-0.0<br>(-0.0 to 0.0)	3.8<br>(-4.4 to 12.4)	-
Malta	YLDs Number: 1990	774<br>(566–1,031)	233<br>(144–360)	116<br>(82–155)	0<br>(0–0)	3<br>(2–4)	26<br>(16–41)	48<br>(31–69)	29<br>(21–38)	0<br>(0–0)	5<br>(3–7)	1<br>(0–1)
Malta	YLDs Number: 2015	518<br>(383–691)	173<br>(108–268)	74<br>(53–99)	0<br>(0–0)	2<br>(1–3)	19<br>(12–29)	30<br>(19–43)	16<br>(11–21)	0<br>(0–0)	3<br>(2–4)	2<br>(1–3)
Malta	YLDs Number: Cumulative change (%)	-32.7<br>(-40.8 to -18.1)	-24.0<br>(-46.7 to 19.4)	-35.6<br>(-38.5 to -32.5)	-67.2<br>(-79.4 to -49.0)	-27.4<br>(-44.5 to -7.7)	-24.4<br>(-46.9 to 20.5)	-37.8<br>(-49.3 to -24.3)	-44.6<br>(-51.7 to -36.9)	-29.9<br>(-49.6 to 5.6)	-34.5<br>(-51.9 to -15.9)	123.2<br>(51.3–214.9)
Malta	YLDs Rate: 1990	2,718.0<br>(1,989.4–3,620.5)	823.9<br>(513.2–1,269.4)	407.2<br>(286.8–544.4)	0.0<br>(0.0–0.1)	11.5<br>(7.9–15.8)	93.3<br>(57.3–144.8)	169.4<br>(108.4–242.3)	103.8<br>(76.7–136.0)	0.0<br>(0.0–0.0)	18.4<br>(12.0–26.1)	4.1<br>(2.8–5.8)
Malta	YLDs Rate: 2015	2,830.7<br>(2,093.1–3,772.9)	945.3<br>(591.9–1,466.5)	408.6<br>(289.3–544.6)	0.0<br>(0.0–0.0)	12.8<br>(8.5–17.9)	105.9<br>(67.1–159.4)	163.7<br>(103.7–236.5)	89.1<br>(65.3–116.9)	0.0<br>(0.0–0.0)	18.4<br>(12.0–26.3)	13.8<br>(9.0–19.9)
Malta	YLDs Rate: Cumulative change (%)	4.5<br>(-8.0 to 27.1)	17.6<br>(-17.3 to 84.1)	0.4<br>(-4.0 to 5.3)	-49.5<br>(-68.3 to -21.3)	12.6<br>(-13.9 to 42.7)	17.0<br>(-17.7 to 86.0)	-2.9<br>(-20.8 to 18.2)	-13.9<br>(-24.9 to -2.0)	9.2<br>(-21.5 to 64.5)	1.8<br>(-25.2 to 30.7)	247.0<br>(135.1–389.3)
Netherlands	Prevalence Number: 1990	606,764<br>(581,315–638,818)	258,034<br>(190,189–344,494)	144,647<br>(134,889–155,876)	2<br>(1–3)	1,273<br>(1,189–1,358)	112,802<br>(101,908–124,806)	34,571<br>(26,641–43,520)	16,040<br>(14,737–17,465)	0<br>(0–0)	424<br>(340–521)	-
Netherlands	Prevalence Number: 2015	593,867<br>(556,949–633,492)	276,595<br>(185,055–390,224)	142,063<br>(132,757–151,858)	1<br>(0–1)	1,325<br>(1,231–1,428)	96,930<br>(84,458–110,704)	30,456<br>(23,474–38,257)	14,313<br>(13,309–15,453)	0<br>(0–0)	400<br>(318–498)	-
Netherlands	Prevalence Number: Cumulative change (%	-2.1<br>(-9.2 to 5.7)	8.2<br>(-24.3 to 53.6)	-1.6<br>(-10.2 to 8.0)	-47.7<br>(-67.8 to -23.5)	4.1<br>(-0.4 to 8.6)	-13.7<br>(-27.7 to 1.8)	-11.8<br>(-20.7 to -1.2)	-10.6<br>(-18.3 to -2.6)	-3.5<br>(-3.7 to -3.3)	-5.7<br>(-11.6 to 0.4)	-
Netherlands	Prevalence Rate: 1990	65,810.8<br>(63,035.0–69,285.0)	27,937.9<br>(20,582.1–37,356.9)	15,707.4<br>(14,648.2–16,927.9)	0.2<br>(0.1–0.3)	137.9<br>(128.8–147.1)	12,231.8<br>(11,050.6–13,533.7)	3,760.1<br>(2,897.6–4,733.4)	1,726.9<br>(1,586.2–1,881.3)	0.0<br>(0.0–0.0)	45.9<br>(36.9–56.5)	-
Netherlands	Prevalence Rate: 2015	67,044.8<br>(62,879.1–71,508.1)	31,303.4<br>(20,992.4–44,114.4)	16,008.2<br>(14,961.0–17,112.3)	0.1<br>(0.1–0.2)	150.0<br>(139.4–161.7)	10,947.7<br>(9,538.9–12,502.3)	3,424.1<br>(2,639.1–4,301.1)	1,634.7<br>(1,520.7–1,764.7)	0.0<br>(0.0–0.0)	45.4<br>(36.2–56.4)	-
Netherlands	Prevalence Rate: Cumulative change (%	1.9<br>(-5.5 to 10.0)	13.1<br>(-20.7 to 60.3)	2.1<br>(-6.8 to 12.1)	-44.4<br>(-65.8 to -18.8)	8.8<br>(4.1–13.5)	-10.2<br>(-24.7 to 6.0)	-8.8<br>(-18.1 to 2.1)	-5.2<br>(-13.3 to 3.3)	0.0<br>(-0.0 to 0.0)	-1.2<br>(-7.4 to 5.3)	-
Netherlands	YLDs Number: 1990	23,528<br>(16,761–31,736)	7,363<br>(4,004–11,465)	3,861<br>(2,731–5,177)	0<br>(0–0)	210<br>(147–283)	352<br>(191–548)	1,595<br>(1,025–2,297)	721<br>(537–958)	0<br>(0–0)	79<br>(49–119)	71<br>(48–98)
Netherlands	YLDs Number: 2015	23,548<br>(16,463–33,341)	8,244<br>(4,090–14,597)	3,777<br>(2,672–5,065)	0<br>(0–0)	220<br>(150–300)	383<br>(193–685)	1,403<br>(898–2,013)	580<br>(424–768)	0<br>(0–0)	72<br>(43–114)	250<br>(168–364)
Netherlands	YLDs Number: Cumulative change (%)	0.4<br>(-17.0 to 28.1)	15.0<br>(-32.6 to 93.3)	-2.1<br>(-6.7 to 2.4)	-47.8<br>(-67.9 to -23.4)	5.1<br>(-13.4 to 27.1)	11.5<br>(-36.0 to 82.0)	-11.8<br>(-21.6 to -0.6)	-19.3<br>(-31.1 to -6.5)	1.1<br>(-35.9 to 38.0)	-6.4<br>(-39.0 to 38.0)	258.1<br>(144.0–396.6)

© 2017 Global Burden of Disease Child and Adolescent Health Collaboration. JAMA Pediatrics.

eTable 4. Prevalent cases, Rates (per 100,000 population), Years Lived with Disability (YLDs), and Cumulative Percent Change with 95% Uncertainty Interval (UI) for the Top 10 Global Causes of YLDsin Children and Adolescents in 195 Countries and Territories, Aged Under 5 Years, Both Sexes, 1990 and 2015. *Note: best viewed by enlarging in browser.*

Location	Measure	All causes	Iron-deficiency anemia	Skin and subcutaneous diseases	Protein-energy malnutrition	Diarrheal diseases	Hemoglobinopathies and hemolytic anemias	Asthma	Neonatal preterm birth complications	Malaria	Neonatal encephalopathy due to birth asphyxia and trauma	Other neonatal disorders
Netherlands	YLDs Rate: 1990	2,551.2 (1,818.5–3,443.8)	796.6 (433.0–1,243.0)	419.7 (296.9–562.7)	0.0 (0.0–0.0)	22.8 (16.0–30.7)	38.2 (20.7–59.4)	173.5 (111.5–249.9)	78.3 (58.3–103.9)	0.0 (0.0–0.0)	8.7 (5.3–13.0)	7.7 (5.3–10.7)
Netherlands	YLDs Rate: 2015	2,660.1 (1,859.8–3,765.2)	934.2 (464.6–1,652.6)	425.2 (300.8–570.1)	0.0 (0.0–0.0)	24.9 (17.1–34.0)	43.4 (21.9–77.5)	157.8 (101.0–226.3)	65.5 (47.9–86.8)	0.0 (0.0–0.0)	8.2 (4.9–12.9)	28.3 (19.0–41.1)
Netherlands	YLDs Rate: Cumulative change (%)	4.6 (-13.5 to 33.4)	20.4 (-29.2 to 101.8)	1.4 (-3.3 to 6.1)	-44.4 (-65.9 to -18.6)	9.8 (-9.4 to 32.6)	16.8 (-32.9 to 89.8)	-8.9 (-19.0 to 2.7)	-16.0 (-28.2 to -2.7)	4.9 (-33.4 to 43.1)	-2.5 (-36.4 to 43.8)	273.0 (154.0–417.3)
Norway	Prevalence Number: 1990	173,090 (167,026–178,752)	78,011 (65,894–89,411)	43,314 (40,411–46,252)	0 (0–0)	219 (189–260)	37,676 (34,684–40,733)	9,848 (7,597–12,088)	4,909 (4,514–5,314)	0 (0–0)	85 (67–107)	-
Norway	Prevalence Number: 2015	194,750 (182,253–209,076)	94,209 (75,103–134,307)	48,056 (44,956–51,343)	0 (0–0)	218 (196–247)	36,825 (32,942–40,962)	8,608 (6,594–10,663)	4,900 (4,495–5,358)	0 (0–0)	99 (78–126)	-
Norway	Prevalence Number: Cumulative change (%	12.6 (4.7–22.1)	21.8 (-5.4 to 87.9)	11.1 (2.0–21.8)	-3.7 (-41.3 to 52.2)	0.0 (-11.3 to 9.0)	-2.1 (-13.5 to 10.8)	-12.3 (-23.7 to 2.8)	-0.1 (-7.6 to 8.5)	9.1 (8.9–9.3)	16.4 (7.6–24.5)	-
Norway	Prevalence Rate: 1990	62,753.0 (60,566.2–64,801.6)	28,178.4 (23,813.2–32,333.4)	15,755.2 (14,698.4–16,827.5)	0.0 (0.0–0.1)	79.5 (68.6–94.5)	13,657.5 (12,571.7–14,764.2)	3,595.3 (2,773.5–4,412.8)	1,750.9 (1,609.0–1,897.3)	0.0 (0.0–0.0)	31.0 (24.2–38.9)	-
Norway	Prevalence Rate: 2015	64,999.1 (60,827.7–69,780.6)	31,442.3 (25,066.0–44,825.8)	16,039.2 (15,004.5–17,136.4)	0.0 (0.0–0.0)	73.0 (65.6–82.5)	12,290.5 (10,994.9–13,671.5)	2,873.3 (2,201.1–3,559.5)	1,635.2 (1,499.9–1,788.1)	0.0 (0.0–0.0)	33.3 (26.1–42.2)	-
Norway	Prevalence Rate: Cumulative change (%	3.6 (-3.6 to 12.4)	12.6 (-12.5 to 73.4)	2.0 (-6.4 to 11.8)	-10.2 (-45.1 to 41.3)	-7.8 (-18.2 to 0.5)	-9.8 (-20.4 to 2.1)	-19.8 (-30.3 to -6.0)	-6.5 (-13.5 to 1.6)	-0.0 (-0.0 to 0.0)	7.7 (-0.3 to 15.3)	-
Norway	YLDs Number: 1990	7,194 (5,247–9,509)	2,185 (1,409–3,168)	1,197 (855–1,600)	0 (0–0)	36 (24–50)	219 (139–318)	454 (287–652)	230 (169–307)	0 (0–0)	15 (9–24)	45 (30–62)
Norway	YLDs Number: 2015	8,000 (5,622–11,297)	2,811 (1,629–5,077)	1,304 (920–1,747)	0 (0–0)	36 (24–50)	259 (153–467)	396 (255–563)	195 (140–261)	0 (0–0)	18 (10–27)	57 (39–81)
Norway	YLDs Number: Cumulative change (%)	11.6 (-5.5 to 53.0)	32.4 (-14.4 to 160.1)	9.0 (3.6–14.3)	-3.6 (-41.5 to 52.4)	0.9 (-23.4 to 28.8)	21.6 (-20.5 to 145.9)	-12.4 (-24.7 to 2.6)	-15.0 (-27.1 to -2.1)	14.9 (-15.1 to 67.8)	19.2 (-31.8 to 83.8)	29.8 (-10.2 to 80.8)
Norway	YLDs Rate: 1990	2,607.5 (1,901.7–3,447.3)	787.9 (507.8–1,142.1)	436.3 (311.6–583.1)	0.0 (0.0–0.0)	13.2 (8.8–18.3)	79.3 (50.4–114.6)	165.9 (104.9–238.1)	83.7 (61.5–111.6)	0.0 (0.0–0.0)	5.7 (3.3–8.9)	16.4 (11.1–22.8)
Norway	YLDs Rate: 2015	2,670.3 (1,876.6–3,770.4)	938.5 (543.8–1,694.8)	435.3 (307.1–583.2)	0.0 (0.0–0.0)	12.1 (8.1–16.8)	86.7 (51.1–156.2)	132.2 (85.2–188.2)	65.4 (46.9–87.3)	0.0 (0.0–0.0)	6.0 (3.4–9.3)	19.2 (13.1–27.2)
Norway	YLDs Rate: Cumulative change (%)	2.8 (-12.9 to 40.7)	22.5 (-20.6 to 140.2)	-0.2 (-5.2 to 4.7)	-10.2 (-45.2 to 42.4)	-6.9 (-29.3 to 18.8)	12.6 (-26.3 to 127.1)	-19.9 (-31.2 to -6.2)	-21.7 (-32.9 to -9.9)	5.4 (-22.0 to 54.0)	9.8 (-37.2 to 69.3)	19.5 (-17.3 to 66.5)
Portugal	Prevalence Number: 1990	414,229 (398,334–430,045)	206,010 (174,071–251,417)	91,935 (85,315–98,786)	14 (9–22)	890 (741–1,059)	72,093 (66,838–77,466)	28,073 (22,714–33,825)	12,493 (11,469–13,650)	0 (0–0)	364 (255–498)	-
Portugal	Prevalence Number: 2015	311,397 (295,662–325,829)	173,338 (139,958–212,079)	69,552 (64,733–74,698)	2 (1–3)	361 (316–405)	47,486 (43,674–51,215)	16,632 (12,700–21,132)	8,359 (7,554–9,238)	0 (0–0)	276 (187–382)	-
Portugal	Prevalence Number: Cumulative change (%	-24.8 (-29.1 to -20.2)	-15.1 (-35.0 to 5.5)	-24.2 (-30.9 to -16.6)	-85.3 (-90.2 to -78.9)	-59.1 (-65.1 to -52.6)	-34.0 (-40.8 to -26.8)	-40.8 (-49.6 to -32.3)	-33.0 (-39.3 to -26.6)	-25.1 (-25.1 to -25.0)	-24.2 (-29.9 to -17.7)	-
Portugal	Prevalence Rate: 1990	69,828.6 (67,183.4–72,458.2)	34,982.0 (29,606.0–42,469.8)	15,397.6 (14,286.0–16,536.5)	2.6 (1.6–4.0)	151.7 (126.8–180.4)	12,169.2 (11,284.4–13,076.3)	4,664.4 (3,774.0–5,620.0)	2,194.3 (2,016.5–2,392.4)	0.0 (0.0–0.0)	62.3 (43.5–85.1)	-
Portugal	Prevalence Rate: 2015	70,190.1 (66,716.9–73,398.9)	39,404.1 (32,063.0–47,856.0)	15,551.7 (14,471.9–16,697.0)	0.5 (0.3–0.8)	82.3 (72.0–92.2)	10,717.6 (9,857.8–11,556.0)	3,684.6 (2,813.4–4,681.4)	1,980.8 (1,792.0–2,184.9)	0.0 (0.0–0.0)	63.4 (43.2–87.6)	-
Portugal	Prevalence Rate: Cumulative change (%	0.6 (-5.1 to 6.6)	13.6 (-12.8 to 40.2)	1.2 (-7.7 to 11.4)	-80.1 (-86.8 to -71.4)	-45.4 (-53.4 to -36.7)	-11.8 (-20.8 to -2.1)	-21.0 (-32.8 to -9.7)	-9.6 (-17.7 to -1.3)	0.0 (-0.0 to 0.0)	1.7 (-6.2 to 10.5)	-
Portugal	YLDs Number: 1990	17,553 (12,812–23,450)	6,067 (3,832–9,241)	2,463 (1,745–3,314)	1 (0–3)	146 (98–206)	252 (158–382)	1,291 (850–1,816)	623 (467–805)	0 (0–0)	71 (42–110)	32 (22–45)
Portugal	YLDs Number: 2015	13,095 (9,217–17,791)	5,612 (3,362–8,394)	1,850 (1,315–2,490)	0 (0–0)	59 (40–83)	233 (141–341)	765 (488–1,096)	298 (219–397)	0 (0–0)	52 (27–82)	163 (107–233)
Portugal	YLDs Number: Cumulative change (%)	-25.2 (-36.5 to -13.9)	-5.6 (-35.1 to 23.3)	-24.9 (-28.3 to -21.2)	-83.8 (-91.5 to -71.7)	-59.0 (-68.8 to -47.4)	-5.2 (-35.5 to 26.9)	-40.7 (-49.9 to -31.7)	-52.1 (-59.2 to -44.2)	-18.7 (-50.1 to 13.2)	-25.5 (-48.6 to 2.8)	416.0 (250.0–621.5)
Portugal	YLDs Rate: 1990	2,963.4 (2,160.6–3,947.2)	1,034.5 (655.0–1,568.1)	411.0 (291.1–553.2)	0.3 (0.1–0.6)	25.0 (16.8–35.3)	43.0 (27.2–64.7)	214.6 (141.4–301.8)	105.2 (79.0–136.2)	0.0 (0.0–0.0)	12.1 (7.2–18.6)	5.5 (3.8–7.6)
Portugal	YLDs Rate: 2015	2,961.9 (2,086.0–4,023.0)	1,283.9 (781.0–1,914.7)	411.8 (292.7–554.2)	0.1 (0.0–0.1)	13.5 (9.2–18.9)	53.3 (32.6–77.4)	169.6 (108.3–242.8)	67.2 (49.5–89.6)	0.0 (0.0–0.0)	11.9 (6.3–18.7)	37.0 (24.3–52.7)
Portugal	YLDs Rate: Cumulative change (%)	0.2 (-14.5 to 14.8)	26.5 (-11.1 to 64.0)	0.3 (-4.4 to 5.1)	-78.1 (-88.6 to -61.3)	-45.2 (-58.2 to -30.1)	26.9 (-12.1 to 68.0)	-21.0 (-33.2 to -8.9)	-36.0 (-45.4 to -25.6)	8.6 (-33.1 to 51.0)	-0.6 (-30.8 to 36.5)	589.4 (368.7–866.0)
Spain	Prevalence Number: 1990	1,486,780 (1,433,963–1,553,878)	1,001,011 (826,413–1,215,567)	311,410 (288,123–337,795)	6 (4–10)	1,564 (1,408–1,742)	190,300 (174,820–206,125)	69,960 (55,239–85,401)	36,911 (33,999–40,121)	0 (0–0)	933 (706–1,225)	-
Spain	Prevalence Number: 2015	1,621,457 (1,575,516–1,665,944)	1,136,191 (1,051,410–1,252,501)	322,743 (299,065–350,187)	2 (1–4)	1,214 (1,060–1,385)	178,480 (163,473–194,051)	77,030 (60,105–95,142)	32,166 (30,399–34,389)	0 (0–0)	946 (719–1,214)	-
Spain	Prevalence Number: Cumulative change (%	9.1 (3.8–14.2)	14.9 (-5.4 to 48.3)	3.8 (-6.5 to 15.0)	-56.6 (-72.5 to -35.4)	-22.3 (-29.6 to -16.2)	-6.0 (-16.4 to 4.8)	10.3 (-2.1 to 26.2)	-12.6 (-20.9 to -4.0)	2.6 (2.6–2.6)	1.5 (-5.9 to 9.1)	-
Spain	Prevalence Rate: 1990	70,057.7 (67,613.6–73,179.7)	47,367.2 (39,319.8–57,250.7)	14,575.1 (13,483.8–15,804.5)	0.3 (0.2–0.5)	74.5 (67.2–83.0)	8,975.2 (8,248.9–9,713.9)	3,245.9 (2,562.9–3,962.4)	1,815.9 (1,674.8–1,971.0)	0.0 (0.0–0.0)	44.6 (33.7–58.4)	-
Spain	Prevalence Rate: 2015	74,360.0 (72,280.2–76,386.2)	52,290.6 (48,432.7–57,599.6)	14,721.1 (13,639.6–15,970.6)	0.1 (0.1–0.2)	56.2 (49.2–64.1)	8,198.1 (7,506.8–8,910.5)	3,484.5 (2,718.9–4,303.8)	1,538.5 (1,457.8–1,640.7)	0.0 (0.0–0.0)	44.0 (33.4–56.5)	-
Spain	Prevalence Rate: Cumulative change (%	6.2 (1.1–11.1)	11.7 (-7.5 to 43.1)	1.2 (-8.9 to 12.0)	-57.7 (-73.3 to -37.1)	-24.5 (-31.4 to -18.6)	-8.5 (-18.6 to 2.0)	7.6 (-4.6 to 23.1)	-15.1 (-22.8 to -7.0)	-0.0 (-0.0 to 0.0)	-1.2 (-8.4 to 6.4)	-
Spain	YLDs Number: 1990	73,188 (51,622–99,793)	35,328 (22,169–51,618)	7,395 (5,170–9,829)	0 (0–1)	257 (176–350)	1,464 (935–2,150)	3,207 (2,063–4,511)	1,654 (1,279–2,130)	0 (0–0)	197 (121–294)	158 (110–215)
Spain	YLDs Number: 2015	79,635 (57,499–106,372)	42,038 (29,172–58,275)	7,605 (5,328–10,255)	0 (0–0)	200 (131–281)	1,728 (1,176–2,421)	3,525 (2,296–5,053)	1,330 (990–1,724)	0 (0–0)	202 (122–298)	725 (465–1,029)
Spain	YLDs Number: Cumulative change (%)	9.8 (-6.6 to 34.3)	22.6 (-8.2 to 74.8)	2.9 (-1.8 to 7.7)	-56.5 (-72.4 to -35.3)	-21.4 (-41.9 to 2.2)	21.5 (-8.9 to 75.1)	10.2 (-3.3 to 28.1)	-19.3 (-31.5 to -5.0)	10.0 (-12.2 to 57.8)	5.3 (-29.1 to 47.2)	365.2 (222.4–539.7)
Spain	YLDs Rate: 1990	3,454.3 (2,439.2–4,694.4)	1,677.2 (1,063.0–2,442.9)	344.8 (240.8–458.3)	0.0 (0.0–0.1)	12.3 (8.4–16.7)	69.5 (44.7–101.6)	148.8 (95.7–209.3)	77.9 (60.3–100.3)	0.0 (0.0–0.0)	9.3 (5.7–13.9)	7.4 (5.2–10.1)
Spain	YLDs Rate: 2015	3,660.0 (2,645.7–4,899.8)	1,941.3 (1,347.8–2,695.4)	345.7 (242.0–466.5)	0.0 (0.0–0.0)	9.3 (6.1–13.0)	79.8 (54.4–111.9)	159.5 (103.9–228.6)	61.0 (45.4–79.0)	0.0 (0.0–0.0)	9.3 (5.6–13.7)	33.3 (21.3–47.2)
Spain	YLDs Rate: Cumulative change (%)	6.8 (-8.8 to 30.1)	19.0 (-9.9 to 67.2)	0.3 (-4.3 to 4.9)	-57.7 (-73.3 to -37.0)	-23.7 (-43.1 to -0.9)	18.0 (-10.9 to 67.4)	7.5 (-5.8 to 24.9)	-21.4 (-33.3 to -7.6)	7.2 (-14.3 to 53.0)	2.4 (-30.6 to 43.0)	352.9 (214.5–522.4)
Sweden	Prevalence Number: 1990	339,739 (329,061–349,388)	145,309 (118,844–167,328)	91,079 (87,101–94,934)	0 (0–0)	253 (226–286)	70,240 (67,554–73,554)	8,513 (6,743–10,913)	9,930 (9,218–10,700)	0 (0–0)	253 (203–315)	-
Sweden	Prevalence Number: 2015	374,421 (361,791–386,751)	149,095 (124,776–175,069)	97,520 (93,410–101,468)	0 (0–0)	330 (299–368)	65,496 (62,886–68,251)	7,631 (5,259–10,343)	9,930 (9,147–10,809)	0 (0–0)	296 (236–370)	-
Sweden	Prevalence Number: Cumulative change (%	10.2 (5.6–15.1)	3.6 (-16.5 to 29.1)	7.1 (1.4–13.4)	-37.2 (-59.2 to -8.4)	30.6 (21.0–42.7)	-6.7 (-12.0 to -1.3)	-10.5 (-26.6 to 9.7)	0.1 (-6.1 to 6.8)	6.6 (6.4–6.8)	17.0 (9.7–23.4)	-
Sweden	Prevalence Rate: 1990	62,359.2 (60,386.9–64,129.8)	26,504.6 (21,581.6–30,538.0)	16,803.1 (16,068.5–17,512.7)	0.1 (0.0–0.1)	46.4 (41.5–52.5)	12,886.2 (12,393.5–13,496.3)	1,579.0 (1,250.8–2,024.1)	1,776.7 (1,648.0–1,915.8)	0.0 (0.0–0.0)	46.2 (37.1–57.5)	-
Sweden	Prevalence Rate: 2015	64,864.7 (62,676.4–66,998.0)	25,803.9 (21,598.2–30,297.3)	16,905.1 (16,192.2–17,589.7)	0.0 (0.0–0.1)	57.2 (51.9–63.9)	11,344.5 (10,892.5–11,821.8)	1,324.0 (912.6–1,794.6)	1,713.4 (1,578.0–1,865.3)	0.0 (0.0–0.0)	51.3 (41.0–64.2)	-

© 2017 Global Burden of Disease Child and Adolescent Health Collaboration. JAMA Pediatrics.

eTable 4. Prevalent cases, Rates (per 100,000 population), Years Lived with Disability (YLDs), and Cumulative Percent Change with 95% Uncertainty Interval (UI) for the Top 10 Global Causes of YLDsin Children and Adolescents in 195 Countries and Territories, Aged Under 5 Years, Both Sexes, 1990 and 2015. *Note: best viewed by enlarging in browser.*

Location	Measure	All causes	Iron-deficiency anemia	Skin and subcutaneous diseases	Protein-energy malnutrition	Diarrheal diseases	Hemoglobinopathies and hemolytic anemias	Asthma	Neonatal preterm birth complications	Malaria	Neonatal encephalopathy due to birth asphyxia and trauma	Other neonatal disorders
Sweden	Prevalence Rate: Cumulative change (%	4.0 (-0.4 to 8.7)	-1.6 (-20.8 to 23.0)	0.7 (-4.7 to 6.5)	-39.1 (-60.3 to -11.5)	23.5 (14.4–35.1)	-11.9 (-17.0 to -6.8)	-16.2 (-31.4 to 2.6)	-3.5 (-9.6 to 3.0)	0.0 (-0.0 to 0.0)	11.2 (4.4–17.2)	-
Sweden	YLDs Number: 1990	12,880 (9,475–16,928)	4,044 (2,569–5,809)	2,657 (1,881–3,542)	0 (0–0)	42 (28–59)	307 (194–439)	393 (255–574)	472 (346–630)	0 (0–0)	47 (30–68)	49 (34–68)
Sweden	YLDs Number: 2015	13,535 (9,793–17,654)	4,035 (2,534–5,818)	2,847 (2,007–3,844)	0 (0–0)	54 (36–75)	293 (184–417)	352 (213–532)	431 (314–583)	0 (0–0)	53 (33–77)	181 (121–256)
Sweden	YLDs Number: Cumulative change (%)	5.4 (-6.8 to 20.2)	1.4 (-25.9 to 39.5)	7.2 (3.4–11.0)	-37.3 (-59.3 to -8.4)	31.9 (-0.9 to 68.6)	-2.9 (-29.8 to 34.9)	-10.2 (-27.4 to 11.3)	-8.5 (-18.8 to 3.7)	9.5 (-24.4 to 67.2)	15.7 (-17.0 to 52.4)	273.1 (171.2–395.4)
Sweden	YLDs Rate: 1990	2,362.4 (1,737.4–3,108.6)	735.5 (464.9–1,058.2)	491.7 (348.2–655.6)	0.0 (0.0–0.0)	7.7 (5.1–11.0)	55.8 (35.2–80.0)	72.9 (47.3–106.5)	86.7 (63.7–115.7)	0.0 (0.0–0.0)	8.6 (5.6–12.6)	9.0 (6.3–12.6)
Sweden	YLDs Rate: 2015	2,344.5 (1,696.5–3,057.4)	698.2 (438.4–1,007.2)	493.9 (348.1–666.7)	0.0 (0.0–0.0)	9.5 (6.4–13.1)	50.8 (31.9–72.2)	61.2 (37.0–92.4)	74.8 (54.5–101.0)	0.0 (0.0–0.0)	9.3 (5.9–13.5)	31.4 (21.1–44.4)
Sweden	YLDs Rate: Cumulative change (%)	-0.5 (-12.0 to 13.6)	-3.4 (-29.8 to 33.5)	0.4 (-3.1 to 4.0)	-39.2 (-60.4 to -11.4)	24.8 (-6.6 to 60.2)	-7.5 (-33.3 to 29.3)	-16.0 (-32.1 to 4.1)	-13.6 (-23.4 to -2.1)	2.9 (-29.1 to 57.4)	9.2 (-21.9 to 44.2)	252.2 (155.9–367.9)
Switzerland	Prevalence Number: 1990	273,103 (261,723–283,098)	112,613 (97,087–133,464)	63,577 (59,129–68,446)	0 (0–0)	309 (278–345)	61,036 (54,207–71,497)	10,139 (7,890–12,646)	6,939 (6,284–7,686)	0 (0–0)	185 (148–231)	-
Switzerland	Prevalence Number: 2015	291,299 (276,972–306,366)	130,349 (107,087–173,434)	67,807 (63,416–72,698)	0 (0–0)	338 (300–376)	59,132 (51,443–71,605)	11,438 (8,684–14,536)	6,903 (6,252–7,615)	0 (0–0)	191 (153–238)	-
Switzerland	Prevalence Number: Cumulative change (%	6.7 (0.6–13.5)	17.1 (-9.1 to 63.8)	6.8 (-2.4 to 17.6)	-54.5 (-73.0 to -31.0)	9.4 (3.7–15.8)	-2.6 (-20.8 to 20.1)	13.4 (-6.4 to 36.1)	-0.4 (-8.4 to 8.8)	6.0 (6.0–6.1)	3.3 (-4.0 to 9.8)	-
Switzerland	Prevalence Rate: 1990	70,374.9 (67,438.3–72,959.8)	28,963.0 (24,974.1–34,336.3)	16,402.6 (15,256.2–17,658.1)	0.2 (0.1–0.2)	79.5 (71.5–88.7)	15,723.0 (13,964.1–18,418.3)	2,620.2 (2,039.0–3,268.0)	1,774.8 (1,607.5–1,966.4)	0.0 (0.0–0.0)	47.7 (38.3–59.6)	-
Switzerland	Prevalence Rate: 2015	70,750.6 (67,262.4–74,418.5)	31,578.9 (25,891.7–42,073.7)	16,497.8 (15,427.1–17,690.7)	0.1 (0.0–0.1)	81.9 (72.8–91.2)	14,353.8 (12,488.9–17,383.2)	2,789.8 (2,118.1–3,545.5)	1,656.5 (1,499.5–1,827.9)	0.0 (0.0–0.0)	46.2 (37.1–57.6)	-
Switzerland	Prevalence Rate: Cumulative change (%	0.6 (-5.2 to 7.0)	10.3 (-14.3 to 54.5)	0.7 (-7.9 to 10.9)	-57.4 (-74.6 to -35.2)	3.0 (-2.3 to 9.1)	-8.2 (-25.4 to 13.1)	7.0 (-11.6 to 28.4)	-6.5 (-14.0 to 2.1)	-0.0 (-0.0 to 0.0)	-2.8 (-9.7 to 3.3)	-
Switzerland	YLDs Number: 1990	10,473 (7,752–13,763)	3,203 (2,060–4,650)	1,791 (1,264–2,400)	0 (0–0)	51 (34–72)	361 (231–522)	467 (297–663)	332 (245–441)	0 (0–0)	35 (21–52)	61 (39–88)
Switzerland	YLDs Number: 2015	11,282 (8,191–15,556)	3,900 (2,390–6,454)	1,903 (1,360–2,549)	0 (0–0)	55 (36–76)	441 (261–732)	526 (336–768)	267 (196–358)	0 (0–0)	35 (21–52)	73 (48–104)
Switzerland	YLDs Number: Cumulative change (%)	8.1 (-7.6 to 37.8)	25.5 (-19.0 to 110.0)	6.3 (1.4–11.0)	-54.4 (-72.9 to -30.9)	10.3 (-15.7 to 40.3)	24.8 (-14.1 to 103.9)	13.5 (-6.6 to 37.9)	-19.2 (-31.2 to -6.5)	12.0 (-19.9 to 63.8)	1.0 (-35.4 to 47.8)	23.5 (-19.8 to 79.4)
Switzerland	YLDs Rate: 1990	2,697.5 (1,996.6–3,546.5)	823.1 (528.6–1,195.4)	462.6 (326.7–619.9)	0.0 (0.0–0.0)	13.2 (8.9–18.5)	92.8 (59.4–134.4)	120.7 (76.8–171.5)	85.7 (63.3–113.9)	0.0 (0.0–0.0)	9.2 (5.6–13.6)	15.8 (10.3–22.7)
Switzerland	YLDs Rate: 2015	2,738.6 (1,990.7–3,778.3)	943.8 (578.2–1,565.8)	463.7 (331.5–621.1)	0.0 (0.0–0.0)	13.5 (8.9–18.5)	106.7 (63.3–177.7)	128.4 (82.0–187.5)	65.0 (47.7–87.0)	0.0 (0.0–0.0)	8.5 (5.2–12.6)	17.8 (11.8–25.3)
Switzerland	YLDs Rate: Cumulative change (%)	1.9 (-12.9 to 30.0)	18.2 (-23.7 to 98.2)	0.3 (-4.3 to 4.8)	-57.3 (-74.6 to -35.2)	3.9 (-20.7 to 32.2)	17.6 (-19.2 to 92.4)	7.1 (-11.8 to 30.2)	-23.9 (-35.2 to -11.9)	5.6 (-24.4 to 54.6)	-4.8 (-39.1 to 39.5)	16.4 (-24.4 to 69.1)
United Kingdom	Prevalence Number: 1990	2,495,821 (2,465,858–2,526,110)	711,852 (653,688–767,411)	628,354 (616,083–639,729)	2 (1–4)	12,347 (12,133–12,604)	589,341 (580,132–599,512)	217,914 (174,054–269,807)	68,172 (63,977–72,469)	0 (0–0)	4,668 (3,822–5,670)	-
United Kingdom	Prevalence Number: 2015	2,630,215 (2,594,618–2,664,833)	786,810 (724,556–850,689)	664,681 (652,412–677,187)	1 (1–2)	10,513 (10,265–10,796)	559,429 (550,938–567,597)	190,199 (156,767–229,451)	69,741 (65,504–74,135)	0 (0–0)	5,261 (4,319–6,337)	-
United Kingdom	Prevalence Number: Cumulative change (%	5.4 (3.8–7.0)	10.8 (-1.3 to 23.6)	5.8 (3.6–8.1)	-46.5 (-55.9 to -36.3)	-14.9 (-15.6 to -14.0)	-5.1 (-6.9 to -3.4)	-12.5 (-17.7 to -7.3)	2.3 (-0.6 to 5.1)	4.8 (4.8–4.8)	12.7 (9.8–15.4)	-
United Kingdom	Prevalence Rate: 1990	65,192.5 (64,410.3–65,983.3)	18,616.3 (17,098.4–20,068.5)	16,404.3 (16,084.0–16,701.2)	0.1 (0.1–0.1)	322.7 (317.1–329.4)	15,396.0 (15,155.4–15,661.5)	5,686.2 (4,541.8–7,040.3)	1,785.8 (1,675.9–1,898.3)	0.0 (0.0–0.0)	122.1 (100.0–148.2)	-
United Kingdom	Prevalence Rate: 2015	65,573.1 (64,687.9–66,434.8)	19,646.1 (18,095.0–21,241.7)	16,559.4 (16,253.9–16,870.8)	0.0 (0.0–0.1)	262.4 (256.2–269.5)	13,950.1 (13,738.2–14,153.8)	4,734.9 (3,902.6–5,712.0)	1,746.1 (1,640.2–1,856.0)	0.0 (0.0–0.0)	131.4 (107.9–158.2)	-
United Kingdom	Prevalence Rate: Cumulative change (%	0.6 (-0.9 to 2.2)	5.8 (-5.7 to 18.0)	1.0 (-1.2 to 3.1)	-48.9 (-57.8 to -39.1)	-18.7 (-19.4 to -17.9)	-9.4 (-11.1 to -7.8)	-16.5 (-21.5 to -11.5)	-2.2 (-4.9 to 0.4)	0.0 (-0.0 to 0.0)	7.6 (4.9–10.2)	-
United Kingdom	YLDs Number: 1990	93,919 (69,959–120,971)	18,547 (12,434–25,919)	18,435 (13,002–24,815)	0 (0–0)	2,044 (1,475–2,700)	2,913 (1,933–4,061)	10,063 (6,567–14,117)	3,439 (2,611–4,420)	0 (0–0)	907 (644–1,212)	213 (160–272)
United Kingdom	YLDs Number: 2015	95,379 (71,399–124,391)	20,476 (14,072–28,996)	19,316 (13,691–25,970)	0 (0–0)	1,738 (1,264–2,283)	3,234 (2,183–4,585)	8,781 (5,800–12,255)	3,095 (2,332–4,029)	0 (0–0)	981 (685–1,331)	328 (245–423)
United Kingdom	YLDs Number: Cumulative change (%)	1.6 (-3.5 to 6.8)	10.9 (-6.4 to 30.4)	4.8 (2.6–7.1)	-46.5 (-55.9 to -36.3)	-14.9 (-17.5 to -12.1)	12.0 (-9.9 to 40.9)	-12.5 (-18.0 to -6.7)	-10.0 (-15.2 to -5.0)	6.6 (-12.7 to 25.6)	8.2 (-0.7 to 17.7)	54.8 (34.1–78.5)
United Kingdom	YLDs Rate: 1990	2,453.5 (1,827.5–3,160.3)	485.3 (325.4–678.1)	481.2 (339.4–647.7)	0.0 (0.0–0.0)	53.4 (38.6–70.6)	76.2 (50.6–106.2)	262.6 (171.4–368.4)	89.8 (68.2–115.5)	0.0 (0.0–0.0)	23.7 (16.8–31.7)	5.6 (4.2–7.1)
United Kingdom	YLDs Rate: 2015	2,378.3 (1,780.3–3,101.4)	511.6 (351.7–724.4)	481.0 (341.0–646.7)	0.0 (0.0–0.0)	43.4 (31.6–57.0)	80.8 (54.6–114.5)	218.6 (144.4–305.1)	77.2 (58.2–100.5)	0.0 (0.0–0.0)	24.5 (17.1–33.2)	8.2 (6.1–10.6)
United Kingdom	YLDs Rate: Cumulative change (%)	-3.1 (-7.9 to 1.9)	5.9 (-10.6 to 24.5)	-0.0 (-2.1 to 2.2)	-48.9 (-57.9 to -39.2)	-18.8 (-21.2 to -16.1)	6.9 (-13.9 to 34.5)	-16.5 (-21.7 to -11.0)	-14.1 (-19.1 to -9.3)	1.7 (-16.7 to 19.8)	3.3 (-5.2 to 12.3)	47.8 (28.0–70.4)
England	Prevalence Number: 1990	2,053,236 (2,025,112–2,081,515)	586,479 (532,942–641,431)	519,272 (508,125–529,789)	2 (1–3)	1,591 (1,452–1,753)	475,242 (466,125–484,514)	178,835 (142,438–220,735)	56,900 (53,184–60,766)	0 (0–0)	3,938 (3,232–4,780)	-
England	Prevalence Number: 2015	2,232,329 (2,198,092–2,266,668)	649,857 (593,129–711,471)	565,820 (554,354–578,772)	1 (0–1)	1,810 (1,635–2,003)	468,424 (460,043–476,444)	158,221 (129,408–191,573)	59,492 (55,674–63,510)	0 (0–0)	4,546 (3,751–5,474)	-
England	Prevalence Number: Cumulative change (%	8.7 (6.9–10.7)	11.2 (-2.4 to 26.1)	9.0 (6.5–11.6)	-40.8 (-52.1 to -28.0)	13.7 (10.4–17.1)	-1.4 (-3.5 to 0.7)	-11.3 (-16.9 to -5.9)	4.6 (1.3–7.7)	7.9 (7.9–7.9)	15.5 (12.2–18.3)	-
England	Prevalence Rate: 1990	64,547.9 (63,663.7–65,436.5)	18,431.5 (16,749.8–20,158.8)	16,326.0 (15,975.9–16,656.7)	0.1 (0.0–0.1)	50.0 (45.6–55.1)	14,939.6 (14,653.1–15,231.1)	5,624.1 (4,479.5–6,941.8)	1,787.0 (1,670.3–1,908.5)	0.0 (0.0–0.0)	123.8 (101.6–150.2)	-
England	Prevalence Rate: 2015	65,078.0 (64,080.1–66,078.9)	18,954.3 (17,301.3–20,749.9)	16,491.3 (16,157.2–16,868.8)	0.0 (0.0–0.1)	52.8 (47.7–58.4)	13,656.6 (13,412.3–13,890.4)	4,610.7 (3,771.1–5,582.6)	1,736.3 (1,624.9–1,853.7)	0.0 (0.0–0.0)	132.6 (109.4–159.6)	-
England	Prevalence Rate: Cumulative change (%	0.8 (-0.9 to 2.6)	3.2 (-9.4 to 17.1)	1.0 (-1.3 to 3.5)	-44.9 (-55.5 to -33.1)	5.5 (2.4–8.6)	-8.6 (-10.5 to -6.6)	-17.8 (-23.0 to -12.8)	-2.8 (-5.9 to 0.1)	0.0 (-0.0 to 0.0)	7.2 (4.1–9.8)	-
England	YLDs Number: 1990	75,809 (56,431–97,745)	15,237 (10,106–21,534)	15,126 (10,692–20,301)	0 (0–0)	264 (184–363)	2,265 (1,465–3,189)	8,259 (5,394–11,628)	2,879 (2,188–3,714)	0 (0–0)	765 (542–1,022)	166 (125–214)
England	YLDs Number: 2015	78,810 (58,798–102,890)	16,688 (11,363–23,594)	16,343 (11,582–21,996)	0 (0–0)	300 (211–404)	2,565 (1,721–3,655)	7,307 (4,792–10,190)	2,667 (2,001–3,474)	0 (0–0)	847 (588–1,151)	256 (191–331)
England	YLDs Number: Cumulative change (%)	4.0 (-1.6 to 9.7)	10.2 (-9.0 to 31.7)	8.0 (5.6–10.6)	-40.8 (-52.2 to -27.9)	13.9 (1.4–26.7)	14.8 (-11.0 to 50.2)	-11.3 (-17.1 to -5.3)	-7.4 (-13.2 to -1.8)	8.3 (-13.6 to 31.1)	10.9 (0.8–21.6)	54.2 (30.4–80.6)
England	YLDs Rate: 1990	2,383.2 (1,774.1–3,072.9)	478.9 (317.7–676.7)	475.6 (336.2–638.4)	0.0 (0.0–0.0)	8.3 (5.8–11.4)	71.2 (46.0–100.2)	259.7 (169.6–365.7)	90.5 (68.8–116.8)	0.0 (0.0–0.0)	24.1 (17.1–32.1)	5.2 (4.0–6.7)
England	YLDs Rate: 2015	2,297.7 (1,714.3–2,999.7)	486.9 (331.6–688.4)	476.3 (337.5–641.0)	0.0 (0.0–0.0)	8.8 (6.2–11.8)	74.8 (50.2–106.6)	213.0 (139.6–297.0)	77.8 (58.3–101.3)	0.0 (0.0–0.0)	24.7 (17.2–33.6)	7.5 (5.6–9.7)
England	YLDs Rate: Cumulative change (%)	-3.6 (-8.7 to 1.8)	2.3 (-15.5 to 22.2)	0.1 (-2.1 to 2.5)	-45.0 (-55.6 to -33.0)	5.6 (-5.9 to 17.6)	6.6 (-17.4 to 39.4)	-17.8 (-23.1 to -12.2)	-14.1 (-19.5 to -8.9)	0.4 (-19.9 to 21.6)	2.8 (-6.5 to 12.7)	43.0 (20.9–67.5)
Northern Ireland	Prevalence Number: 1990	91,236 (89,221–93,111)	24,492 (18,502–29,832)	22,380 (21,338–23,384)	0 (0–0)	98 (87–109)	23,654 (22,790–24,502)	5,353 (4,083–6,790)	2,381 (2,197–2,581)	0 (0–0)	151 (122–188)	-

© 2017 Global Burden of Disease Child and Adolescent Health Collaboration. JAMA Pediatrics.

eTable 4. Prevalent cases, Rates (per 100,000 population), Years Lived with Disability (YLDs), and Cumulative Percent Change with 95% Uncertainty Interval (UI) for the Top 10 Global Causes of YLDsin Children and Adolescents in 195 Countries and Territories, Aged Under 5 Years, Both Sexes, 1990 and 2015. *Note: best viewed by enlarging in browser.*

Location	Measure	All causes	Iron-deficiency anemia	Skin and subcutaneous diseases	Protein-energy malnutrition	Diarrheal diseases	Hemoglobinopathies and hemolytic anemias	Asthma	Neonatal preterm birth complications	Malaria	Neonatal encephalopathy due to birth asphyxia and trauma	Other neonatal disorders
Northern Ireland	Prevalence Number: 2015	88,010 (84,787–90,826)	29,643 (22,128–39,199)	20,761 (19,755–21,755)	0 (0–0)	121 (107–144)	19,386 (18,732–20,028)	3,510 (2,628–4,499)	2,208 (2,031–2,412)	0 (0–0)	154 (124–187)	-
Northern Ireland	Prevalence Number: Cumulative change (%	-3.5 (-7.1 to 0.1)	22.5 (-8.5 to 73.0)	-7.2 (-12.9 to -0.9)	-67.5 (-80.0 to -51.6)	24.3 (13.1–40.3)	-18.0 (-21.9 to -14.1)	-34.2 (-43.1 to -24.8)	-7.1 (-13.7 to 0.7)	-8.3 (-8.6 to -8.1)	1.9 (-4.1 to 8.4)	-
Northern Ireland	Prevalence Rate: 1990	67,726.5 (66,236.9–69,098.2)	18,482.1 (14,093.0–22,432.0)	16,507.3 (15,741.7–17,242.8)	0.1 (0.1–0.2)	73.5 (65.6–82.1)	17,597.0 (16,950.0–18,226.5)	3,917.7 (2,988.2–4,969.4)	1,843.7 (1,701.8–1,999.2)	0.0 (0.0–0.0)	114.2 (92.2–141.8)	-
Northern Ireland	Prevalence Rate: 2015	70,825.4 (68,258.7–73,091.0)	23,946.9 (17,927.8–31,585.4)	16,676.4 (15,870.3–17,474.1)	0.0 (0.0–0.1)	98.4 (86.9–116.7)	15,612.3 (15,085.3–16,128.5)	2,813.6 (2,106.1–3,606.1)	1,800.1 (1,656.1–1,966.0)	0.0 (0.0–0.0)	124.7 (100.6–152.0)	-
Northern Ireland	Prevalence Rate: Cumulative change (%	4.6 (0.7–8.5)	31.0 (-1.6 to 84.5)	1.1 (-5.2 to 7.9)	-65.9 (-79.0 to -49.0)	33.9 (21.9–51.1)	-11.2 (-15.5 to -7.0)	-28.0 (-37.7 to -17.6)	-2.2 (-9.0 to 5.7)	0.0 (-0.0 to 0.0)	9.3 (2.8–16.4)	-
Northern Ireland	YLDs Number: 1990	3,254 (2,373–4,186)	627 (372–931)	663 (471–890)	0 (0–0)	16 (11–22)	124 (73–184)	246 (158–358)	125 (93–163)	0 (0–0)	29 (19–40)	5 (3–7)
Northern Ireland	YLDs Number: 2015	3,203 (2,306–4,289)	834 (486–1,365)	606 (428–811)	0 (0–0)	20 (13–28)	147 (85–244)	161 (101–236)	101 (75–134)	0 (0–0)	28 (18–41)	10 (6–14)
Northern Ireland	YLDs Number: Cumulative change (%)	-1.5 (-10.3 to 23.5)	36.3 (0.7–153.6)	-8.6 (-13.3 to -4.0)	-67.5 (-80.0 to -51.4)	24.2 (0.9–52.1)	21.3 (-13.5 to 124.8)	-34.3 (-44.2 to -23.5)	-18.8 (-29.1 to -7.7)	5.6 (-31.3 to 65.2)	-1.2 (-28.5 to 31.3)	95.1 (31.0–175.0)
Northern Ireland	YLDs Rate: 1990	2,420.9 (1,764.2–3,115.2)	476.0 (284.7–700.0)	487.3 (346.2–653.7)	0.0 (0.0–0.0)	12.2 (8.3–16.9)	94.3 (55.9–139.2)	180.5 (116.3–262.4)	93.0 (69.3–121.5)	0.0 (0.0–0.0)	21.7 (14.4–30.4)	4.0 (2.7–5.5)
Northern Ireland	YLDs Rate: 2015	2,580.0 (1,858.9–3,450.0)	675.6 (394.7–1,099.6)	486.7 (343.6–651.3)	0.0 (0.0–0.0)	16.2 (10.9–23.1)	119.0 (69.6–196.7)	129.5 (81.6–189.3)	81.6 (60.5–108.3)	0.0 (0.0–0.0)	22.9 (14.8–33.4)	8.2 (5.4–11.8)
Northern Ireland	YLDs Rate: Cumulative change (%)	6.7 (-2.8 to 33.5)	45.1 (7.6–168.3)	-0.1 (-5.3 to 4.9)	-65.8 (-79.0 to -48.9)	33.8 (8.8–63.0)	29.2 (-7.2 to 137.8)	-28.1 (-39.0 to -16.3)	-12.1 (-23.0 to -0.2)	15.0 (-24.9 to 79.9)	7.0 (-22.5 to 41.7)	111.2 (41.9–197.5)
Scotland	Prevalence Number: 1990	217,711 (211,030–224,076)	62,109 (48,891–77,099)	55,095 (52,397–57,735)	0 (0–0)	247 (221–274)	57,056 (54,969–59,263)	19,930 (15,305–25,615)	5,543 (5,234–5,912)	0 (0–0)	368 (298–455)	-
Scotland	Prevalence Number: 2015	185,143 (179,809–190,846)	59,987 (45,676–78,771)	48,607 (46,363–51,018)	0 (0–0)	256 (229–291)	44,336 (42,796–46,115)	16,011 (13,041–19,740)	5,238 (4,945–5,621)	0 (0–0)	352 (286–436)	-
Scotland	Prevalence Number: Cumulative change (%	-14.9 (-18.4 to -11.2)	-0.8 (-36.6 to 41.0)	-11.7 (-17.1 to -6.0)	-69.5 (-80.1 to -54.7)	3.6 (-3.0 to 12.5)	-22.3 (-26.2 to -18.2)	-19.0 (-30.8 to -5.1)	-5.4 (-12.5 to 2.1)	-12.6 (-12.6 to -12.6)	-4.4 (-10.0 to 1.4)	-
Scotland	Prevalence Rate: 1990	66,967.3 (64,869.7–68,926.7)	19,299.3 (15,174.8–23,964.3)	16,877.5 (16,050.6–17,684.4)	0.1 (0.1–0.2)	76.6 (68.6–84.9)	17,566.7 (16,925.3–18,245.8)	6,078.4 (4,668.0–7,812.2)	1,746.7 (1,651.7–1,860.9)	0.0 (0.0–0.0)	114.4 (92.8–141.3)	-
Scotland	Prevalence Rate: 2015	65,184.9 (63,316.1–67,188.7)	21,279.0 (16,212.1–27,856.0)	17,042.3 (16,255.9–17,883.9)	0.0 (0.0–0.1)	90.8 (81.4–103.1)	15,626.6 (15,082.7–16,252.9)	5,588.4 (4,551.7–6,889.8)	1,893.0 (1,789.6–2,026.5)	0.0 (0.0–0.0)	125.2 (101.8–154.7)	-
Scotland	Prevalence Rate: Cumulative change (%	-2.6 (-6.7 to 1.7)	13.1 (-27.5 to 60.8)	1.0 (-5.1 to 7.6)	-64.9 (-77.3 to -47.6)	18.6 (11.2–29.0)	-11.0 (-15.5 to -6.4)	-7.3 (-20.8 to 8.7)	8.5 (0.6–16.8)	-0.0 (-0.0 to 0.0)	9.5 (3.1–16.2)	-
Scotland	YLDs Number: 1990	8,282 (6,038–10,875)	1,656 (979–2,587)	1,684 (1,200–2,274)	0 (0–0)	41 (27–57)	322 (195–506)	922 (589–1,307)	269 (203–350)	0 (0–0)	72 (49–101)	30 (21–40)
Scotland	YLDs Number: 2015	7,127 (5,229–9,428)	1,604 (950–2,572)	1,476 (1,050–1,974)	0 (0–0)	42 (28–60)	287 (170–463)	739 (486–1,054)	204 (152–269)	0 (0–0)	66 (43–95)	49 (33–70)
Scotland	YLDs Number: Cumulative change (%)	-13.7 (-25.6 to -0.4)	1.5 (-41.7 to 59.1)	-12.3 (-17.1 to -7.4)	-69.4 (-80.2 to -54.4)	4.7 (-19.0 to 33.1)	-6.5 (-46.7 to 49.3)	-19.0 (-31.1 to -4.3)	-23.9 (-33.7 to -13.7)	-3.3 (-51.1 to 52.4)	-7.5 (-28.4 to 17.3)	67.3 (14.3–131.6)
Scotland	YLDs Rate: 1990	2,550.4 (1,857.5–3,344.4)	516.9 (306.0–804.6)	514.8 (366.8–695.1)	0.0 (0.0–0.0)	12.8 (8.5–17.8)	100.5 (60.7–157.6)	281.2 (179.7–398.8)	82.9 (62.6–107.7)	0.0 (0.0–0.0)	22.3 (15.3–31.3)	9.3 (6.5–12.6)
Scotland	YLDs Rate: 2015	2,510.3 (1,842.3–3,322.3)	571.1 (338.6–911.1)	516.4 (367.4–690.3)	0.0 (0.0–0.0)	15.1 (10.2–21.4)	102.2 (60.6–164.4)	258.3 (169.9–368.0)	72.0 (53.8–94.8)	0.0 (0.0–0.0)	23.4 (15.4–33.4)	17.6 (11.9–25.0)
Scotland	YLDs Rate: Cumulative change (%)	-1.3 (-14.8 to 14.0)	15.7 (-33.3 to 81.4)	0.3 (-5.1 to 6.0)	-64.8 (-77.3 to -47.4)	20.0 (-6.6 to 51.8)	6.6 (-38.9 to 70.1)	-7.3 (-21.1 to 9.5)	-12.9 (-24.1 to -1.2)	10.6 (-43.8 to 74.1)	5.9 (-17.9 to 34.3)	91.5 (31.0–165.1)
Wales	Prevalence Number: 1990	133,636 (129,731–137,626)	38,771 (30,249–49,587)	31,606 (30,041–33,145)	0 (0–0)	10,409 (10,344–10,497)	33,387 (32,058–34,667)	13,796 (11,149–16,850)	3,347 (3,079–3,637)	0 (0–0)	209 (167–260)	-
Wales	Prevalence Number: 2015	124,731 (120,347–129,368)	47,323 (35,082–66,099)	29,493 (27,980–31,029)	0 (0–0)	8,324 (8,263–8,408)	27,282 (26,272–28,335)	12,454 (10,571–14,579)	2,802 (2,546–3,096)	0 (0–0)	207 (166–255)	-
Wales	Prevalence Number: Cumulative change (%	-6.6 (-10.8 to -2.4)	25.0 (-16.0 to 85.2)	-6.6 (-12.5 to 0.2)	-52.0 (-70.9 to -27.8)	-20.0 (-20.4 to -19.7)	-18.2 (-22.4 to -14.0)	-9.2 (-20.8 to 3.3)	-16.2 (-24.4 to -8.4)	-7.7 (-7.8 to -7.5)	-1.0 (-7.5 to 6.1)	-
Wales	Prevalence Rate: 1990	71,213.6 (69,134.8–73,335.8)	20,699.7 (16,159.3–26,469.9)	16,827.0 (15,994.5–17,647.1)	0.1 (0.0–0.1)	5,554.2 (5,519.4–5,601.1)	17,796.1 (17,087.7–18,477.7)	7,338.7 (5,930.7–8,963.4)	1,792.7 (1,650.1–1,948.6)	0.0 (0.0–0.0)	112.0 (89.7–138.8)	-
Wales	Prevalence Rate: 2015	72,285.3 (69,760.2–74,945.6)	27,577.2 (20,556.4–38,333.7)	17,021.4 (16,153.9–17,908.9)	0.0 (0.0–0.0)	4,862.3 (4,827.1–4,911.1)	15,830.7 (15,247.1–16,439.8)	7,154.2 (6,072.2–8,374.8)	1,666.1 (1,514.1–1,836.4)	0.0 (0.0–0.0)	121.5 (97.3–149.3)	-
Wales	Prevalence Rate: Cumulative change (%	1.5 (-3.0 to 6.1)	36.4 (-7.8 to 101.5)	1.2 (-5.2 to 8.6)	-46.5 (-67.6 to -19.7)	-12.5 (-12.8 to -12.1)	-11.0 (-15.5 to -6.3)	-1.9 (-14.4 to 11.6)	-7.0 (-15.9 to 1.6)	-0.0 (-0.0 to 0.0)	8.5 (1.3–16.3)	-
Wales	YLDs Number: 1990	6,573 (4,825–8,506)	1,026 (588–1,645)	961 (683–1,302)	0 (0–0)	1,721 (1,247–2,271)	200 (117–321)	635 (409–892)	165 (122–216)	0 (0–0)	40 (26–56)	10 (7–14)
Wales	YLDs Number: 2015	6,238 (4,532–8,273)	1,348 (759–2,276)	890 (634–1,194)	0 (0–0)	1,374 (999–1,804)	234 (130–394)	572 (383–792)	122 (90–162)	0 (0–0)	38 (24–53)	12 (8–17)
Wales	YLDs Number: Cumulative change (%)	-4.9 (-17.4 to 13.0)	39.6 (-25.8 to 164.1)	-7.3 (-12.2 to -2.3)	-51.9 (-70.9 to -27.8)	-20.1 (-22.5 to -17.7)	23.9 (-35.1 to 130.9)	-9.3 (-21.4 to 3.9)	-25.5 (-35.7 to -14.7)	12.6 (-33.0 to 72.8)	-3.6 (-26.4 to 22.9)	21.9 (-14.8 to 68.2)
Wales	YLDs Rate: 1990	3,504.4 (2,572.3–4,535.6)	548.2 (314.7–878.9)	511.4 (363.5–693.3)	0.0 (0.0–0.0)	918.7 (665.7–1,212.0)	107.3 (62.9–171.5)	338.0 (218.0–474.6)	88.0 (65.2–115.2)	0.0 (0.0–0.0)	21.4 (14.4–29.9)	5.6 (3.9–7.8)
Wales	YLDs Rate: 2015	3,621.8 (2,633.7–4,797.0)	787.4 (447.1–1,322.7)	512.4 (365.3–687.4)	0.0 (0.0–0.0)	803.1 (583.6–1,053.9)	137.0 (76.7–228.9)	328.7 (220.4–455.1)	71.2 (52.3–94.0)	0.0 (0.0–0.0)	22.2 (14.4–31.3)	7.3 (5.0–10.1)
Wales	YLDs Rate: Cumulative change (%)	3.6 (-10.0 to 23.0)	52.5 (-18.8 to 187.0)	0.3 (-5.1 to 5.7)	-46.5 (-67.6 to -19.4)	-12.6 (-15.1 to -10.0)	35.3 (-29.0 to 150.9)	-2.1 (-15.1 to 12.2)	-18.9 (-30.0 to -7.3)	21.9 (-27.4 to 86.9)	4.9 (-19.8 to 33.7)	32.7 (-7.2 to 83.0)
Southern Latin America	Prevalence Number: 1990	3,861,515 (3,779,734–3,941,053)	1,835,163 (1,611,640–2,039,008)	886,306 (846,115–928,467)	2,166 (1,450–3,072)	17,884 (16,197–19,968)	361,539 (345,796–379,166)	271,069 (225,979–320,924)	79,189 (72,683–85,752)	24 (12–37)	5,225 (4,111–6,612)	-
Southern Latin America	Prevalence Number: 2015	3,906,317 (3,815,875–3,986,771)	2,164,162 (1,878,935–2,369,955)	910,804 (867,970–956,661)	269 (178–396)	10,311 (9,175–11,649)	347,683 (330,733–363,958)	216,825 (167,703–270,239)	75,294 (69,290–82,135)	0 (0–0)	5,189 (4,110–6,502)	-
Southern Latin America	Prevalence Number: Cumulative change (%	1.2 (-1.9 to 4.0)	18.7 (-7.1 to 39.1)	2.8 (-2.7 to 8.7)	-87.4 (-90.3 to -84.1)	-42.3 (-48.2 to -36.6)	-3.8 (-10.1 to 2.6)	-20.1 (-31.6 to -8.1)	-4.8 (-10.9 to 1.0)	-100.0 (-100.0 to -100.0)	-0.5 (-6.6 to 4.6)	-
Southern Latin America	Prevalence Rate: 1990	76,123.7 (74,508.3–77,693.2)	36,152.1 (31,741.0–40,173.8)	17,481.5 (16,688.5–18,313.6)	42.5 (28.5–60.3)	352.1 (318.9–393.1)	7,125.6 (6,815.5–7,472.9)	5,350.7 (4,460.6–6,334.7)	1,556.0 (1,428.0–1,684.8)	0.5 (0.2–0.7)	102.9 (81.0–130.2)	-
Southern Latin America	Prevalence Rate: 2015	76,146.0 (74,382.8–77,712.8)	42,234.4 (36,678.7–46,247.8)	17,733.6 (16,899.5–18,627.5)	5.3 (3.5–7.8)	201.5 (179.3–227.6)	6,780.5 (6,450.0–7,097.1)	4,215.1 (3,260.1–5,253.4)	1,478.5 (1,360.9–1,611.5)	0.0 (0.0–0.0)	101.4 (80.4–127.1)	-
Southern Latin America	Prevalence Rate: Cumulative change (%	0.0 (-3.0 to 2.8)	17.6 (-8.0 to 37.8)	1.5 (-3.9 to 7.3)	-87.5 (-90.3 to -84.1)	-42.7 (-48.6 to -37.1)	-4.8 (-11.1 to 1.5)	-21.3 (-32.6 to -9.5)	-4.9 (-10.9 to 1.0)	-100.0 (-100.0 to -100.0)	-1.3 (-7.2 to 3.9)	-
Southern Latin America	YLDs Number: 1990	172,997 (127,009–228,361)	58,633 (39,145–83,988)	27,625 (19,576–36,839)	263 (151–411)	2,944 (2,120–3,939)	2,595 (1,748–3,686)	12,451 (8,376–17,393)	5,412 (4,082–6,959)	0 (0–0)	994 (651–1,390)	670 (466–910)
Southern Latin America	YLDs Number: 2015	184,857 (134,057–242,200)	74,823 (50,214–102,842)	28,069 (19,953–37,280)	32 (17–53)	1,692 (1,182–2,293)	3,155 (2,101–4,330)	9,934 (6,418–14,281)	4,232 (3,145–5,547)	0 (0–0)	957 (635–1,358)	964 (683–1,298)
Southern Latin America	YLDs Number: Cumulative change (%)	7.2 (-9.6 to 21.3)	30.0 (-11.6 to 69.7)	1.6 (-1.5 to 4.6)	-87.3 (-91.3 to -82.4)	-42.4 (-50.3 to -33.9)	23.8 (-22.2 to 59.8)	-20.3 (-32.0 to -8.2)	-21.8 (-29.5 to -13.5)	-100.0 (-100.0 to -100.0)	-2.9 (-21.9 to 19.0)	46.2 (8.4–93.2)

© 2017 Global Burden of Disease Child and Adolescent Health Collaboration. JAMA Pediatrics.

eTable 4. Prevalent cases, Rates (per 100,000 population), Years Lived with Disability (YLDs), and Cumulative Percent Change with 95% Uncertainty Interval (UI) for the Top 10 Global Causes of YLDsin Children and Adolescents in 195 Countries and Territories, Aged Under 5 Years, Both Sexes, 1990 and 2015. *Table best viewed by enlarging in browser.*

Location	Measure	All causes	Iron-deficiency anemia	Skin and subcutaneous diseases	Protein-energy malnutrition	Diarrheal diseases	Hemoglobinopathies and hemolytic anemias	Asthma	Neonatal preterm birth complications	Malaria	Neonatal encephalopathy due to birth asphyxia and trauma	Other neonatal disorders
Southern Latin America	YLDs Rate: 1990	3,409.5 (2,503.4–4,500.1)	1,154.6 (770.7–1,654.7)	545.1 (386.3–726.8)	5.2 (3.0–8.1)	58.0 (41.8–77.6)	51.1 (34.4–72.6)	245.8 (165.3–343.3)	106.7 (80.5–137.1)	0.0 (0.0–0.0)	19.6 (12.8–27.4)	13.2 (9.2–17.9)
Southern Latin America	YLDs Rate: 2015	3,605.2 (2,614.7–4,723.9)	1,461.4 (980.2–2,009.2)	546.1 (388.2–725.2)	0.6 (0.3–1.0)	33.1 (23.1–44.8)	61.6 (41.0–84.6)	193.1 (124.8–277.6)	82.6 (61.4–108.2)	0.0 (0.0–0.0)	18.7 (12.4–26.5)	18.8 (13.3–25.3)
Southern Latin America	YLDs Rate: Cumulative change (%)	6.1 (-10.6 to 20.1)	29.0 (-12.3 to 68.3)	0.2 (-2.9 to 3.2)	-87.3 (-91.3 to -82.4)	-42.8 (-50.7 to -34.4)	22.8 (-22.9 to 58.4)	-21.5 (-33.0 to -9.6)	-22.6 (-30.2 to -14.4)	-100.0 (-100.0 to -100.0)	-4.0 (-22.7 to 17.8)	44.7 (7.3–91.2)
Argentina	Prevalence Number: 1990	2,623,372 (2,547,107–2,695,865)	1,354,042 (1,150,402–1,552,995)	592,762 (556,914–632,113)	1,983 (1,324–2,844)	13,990 (12,663–15,681)	248,481 (234,240–265,050)	196,830 (165,241–237,733)	54,982 (49,794–60,291)	24 (12–37)	3,540 (2,769–4,447)	-
Argentina	Prevalence Number: 2015	2,863,637 (2,780,802–2,938,058)	1,697,717 (1,412,575–1,889,967)	657,085 (619,148–698,469)	235 (153–347)	8,743 (7,718–9,965)	257,246 (241,728–272,448)	157,060 (120,978–197,809)	55,074 (49,976–60,471)	0 (0–0)	3,788 (2,991–4,775)	-
Argentina	Prevalence Number: Cumulative change (%	9.2 (5.0–13.3)	26.8 (-8.0 to 54.7)	11.0 (2.8–19.7)	-88.0 (-91.0 to -84.5)	-37.4 (-44.4 to -30.8)	3.7 (-5.2 to 12.1)	-20.1 (-31.3 to -4.7)	0.3 (-7.7 to 8.8)	-100.0 (-100.0 to -100.0)	7.2 (-0.7 to 14.7)	-
Argentina	Prevalence Rate: 1990	76,964.5 (74,727.1–79,093.4)	39,691.6 (33,708.1–45,544.4)	17,404.8 (16,352.7–18,560.1)	57.8 (38.6–82.9)	409.7 (370.7–459.1)	7,287.9 (6,869.8–7,773.9)	5,785.8 (4,857.3–6,988.2)	1,605.5 (1,453.8–1,759.6)	0.7 (0.4–1.1)	103.7 (81.1–130.3)	-
Argentina	Prevalence Rate: 2015	77,074.1 (74,846.4–79,072.3)	45,728.4 (38,054.6–50,904.2)	17,669.1 (16,649.7–18,780.6)	6.4 (4.1–9.4)	235.7 (208.1–268.7)	6,926.0 (6,508.9–7,335.5)	4,218.3 (3,249.2–5,312.7)	1,490.8 (1,352.7–1,636.4)	0.0 (0.0–0.0)	102.2 (80.7–128.7)	-
Argentina	Prevalence Rate: Cumulative change (%	0.2 (-3.7 to 3.9)	16.5 (-15.5 to 42.1)	1.6 (-5.9 to 9.7)	-88.9 (-91.6 to -85.6)	-42.4 (-48.8 to -36.3)	-4.8 (-13.0 to 2.9)	-27.0 (-37.3 to -13.0)	-7.0 (-14.5 to 0.9)	-100.0 (-100.0 to -100.0)	-1.2 (-8.5 to 5.7)	-
Argentina	YLDs Number: 1990	123,101 (89,643–164,242)	44,568 (28,772–65,132)	18,343 (12,980–24,473)	240 (136–376)	2,299 (1,638–3,095)	1,952 (1,285–2,821)	9,035 (5,997–12,660)	3,946 (2,989–5,084)	0 (0–0)	670 (426–953)	558 (376–766)
Argentina	YLDs Number: 2015	141,844 (101,206–187,101)	60,730 (40,044–84,615)	20,119 (14,275–26,670)	28 (15–46)	1,433 (987–1,962)	2,519 (1,590–3,487)	7,188 (4,607–10,237)	3,125 (2,284–4,097)	0 (0–0)	697 (448–1,003)	694 (465–968)
Argentina	YLDs Number: Cumulative change (%)	15.9 (-8.2 to 35.8)	40.5 (-15.5 to 96.8)	9.7 (4.9–14.0)	-87.8 (-92.1 to -82.6)	-37.5 (-47.1 to -26.8)	33.0 (-28.3 to 83.2)	-20.3 (-32.9 to -4.1)	-20.7 (-30.3 to -10.0)	-100.0 (-100.0 to -100.0)	5.5 (-21.0 to 37.9)	27.3 (-11.9 to 78.9)
Argentina	YLDs Rate: 1990	3,610.3 (2,628.9–4,816.4)	1,305.7 (842.8–1,907.3)	538.9 (381.3–719.1)	7.0 (4.0–11.0)	67.3 (48.0–90.7)	57.2 (37.6–82.7)	265.6 (176.3–372.2)	115.7 (87.7–149.1)	0.0 (0.0–0.0)	19.7 (12.5–28.0)	16.4 (11.0–22.5)
Argentina	YLDs Rate: 2015	3,819.1 (2,725.1–5,037.6)	1,636.8 (1,079.6–2,279.2)	540.7 (383.6–716.8)	0.8 (0.4–1.3)	38.7 (26.6–52.9)	67.9 (42.9–94.0)	193.1 (123.7–275.0)	84.2 (61.5–110.3)	0.0 (0.0–0.0)	18.8 (12.1–27.0)	18.7 (12.5–26.1)
Argentina	YLDs Rate: Cumulative change (%)	6.4 (-15.7 to 24.7)	29.3 (-22.3 to 80.9)	0.4 (-4.1 to 4.3)	-88.7 (-92.7 to -83.9)	-42.5 (-51.3 to -32.6)	22.3 (-34.0 to 68.8)	-27.2 (-38.7 to -12.4)	-27.2 (-36.0 to -17.3)	-100.0 (-100.0 to -100.0)	-3.2 (-27.5 to 26.6)	16.9 (-19.1 to 64.3)
Chile	Prevalence Number: 1990	1,042,876 (1,014,894–1,068,381)	385,245 (323,134–446,081)	245,514 (231,255–258,554)	58 (37–86)	3,169 (2,870–3,519)	93,475 (87,668–99,506)	59,894 (43,354–76,291)	20,075 (18,409–21,941)	0 (0–0)	1,410 (1,110–1,806)	-
Chile	Prevalence Number: 2015	869,154 (839,817–901,565)	378,306 (312,772–443,797)	210,119 (198,344–222,457)	17 (11–26)	1,298 (1,198–1,407)	74,309 (70,027–79,191)	49,350 (37,254–63,286)	16,698 (15,346–18,201)	0 (0–0)	1,159 (914–1,457)	-
Chile	Prevalence Number: Cumulative change (%	-16.6 (-20.1 to -12.8)	-1.3 (-16.1 to 18.8)	-14.3 (-20.7 to -7.7)	-69.5 (-78.5 to -59.0)	-58.9 (-62.7 to -55.4)	-20.4 (-26.7 to -13.3)	-16.2 (-41.4 to 4.2)	-16.7 (-23.8 to -9.5)	-25.0 (-40.2 to -5.8)	-17.7 (-23.2 to -11.2)	-
Chile	Prevalence Rate: 1990	74,938.2 (72,927.5–76,769.6)	27,694.6 (23,235.2–32,064.7)	17,638.0 (16,613.6–18,574.7)	4.2 (2.7–6.2)	227.9 (206.4–253.0)	6,717.6 (6,300.4–7,151.1)	4,302.0 (3,114.0–5,479.8)	1,444.3 (1,324.6–1,578.7)	0.0 (0.0–0.0)	101.4 (79.9–129.9)	-
Chile	Prevalence Rate: 2015	74,227.9 (71,723.0–76,988.2)	32,393.4 (26,854.6–38,018.1)	17,913.7 (16,911.2–18,967.5)	1.5 (1.0–2.3)	111.5 (102.8–120.7)	6,351.8 (5,985.9–6,768.5)	4,197.2 (3,168.4–5,382.5)	1,442.3 (1,325.8–1,570.1)	0.0 (0.0–0.0)	99.4 (78.5–125.2)	-
Chile	Prevalence Rate: Cumulative change (%	-0.9 (-5.0 to 3.7)	17.6 (-0.1 to 41.5)	1.7 (-5.9 to 9.5)	-63.5 (-74.2 to -50.9)	-51.0 (-55.5 to -46.7)	-5.3 (-12.8 to 3.1)	-0.7 (-30.6 to 23.3)	0.0 (-8.5 to 8.6)	-11.1 (-29.1 to 11.8)	-1.7 (-8.4 to 6.0)	-
Chile	YLDs Number: 1990	41,175 (30,315–53,601)	11,132 (7,325–15,870)	7,764 (5,583–10,376)	7 (3–12)	524 (366–707)	512 (338–723)	2,756 (1,718–4,108)	1,182 (882–1,546)	0 (0–0)	271 (173–398)	74 (49–105)
Chile	YLDs Number: 2015	35,575 (25,935–46,501)	11,340 (7,072–16,213)	6,579 (4,682–8,834)	2 (1–3)	214 (150–292)	514 (329–738)	2,267 (1,447–3,300)	917 (677–1,206)	0 (0–0)	215 (137–313)	214 (144–302)
Chile	YLDs Number: Cumulative change (%)	-13.6 (-21.4 to -2.8)	2.9 (-21.6 to 36.7)	-15.3 (-18.9 to -12.0)	-67.8 (-80.2 to -49.5)	-58.9 (-66.8 to -50.7)	1.4 (-19.3 to 35.6)	-16.4 (-41.8 to 4.5)	-22.3 (-33.2 to -10.8)	-17.2 (-43.6 to 18.0)	-19.4 (-41.1 to 3.6)	194.1 (96.2–315.5)
Chile	YLDs Rate: 1990	2,959.2 (2,178.7–3,852.1)	800.5 (526.8–1,141.1)	557.8 (401.1–745.4)	0.5 (0.2–0.9)	37.7 (26.3–50.8)	36.8 (24.3–52.0)	198.0 (123.4–295.1)	85.0 (63.4–111.1)	0.0 (0.0–0.0)	19.5 (12.4–28.6)	5.4 (3.6–7.6)
Chile	YLDs Rate: 2015	3,040.6 (2,215.9–3,975.4)	972.4 (606.9–1,390.6)	560.3 (398.6–752.5)	0.2 (0.1–0.3)	18.4 (12.9–25.2)	44.2 (28.2–63.3)	192.8 (123.1–280.7)	78.4 (57.9–103.1)	0.0 (0.0–0.0)	18.4 (11.8–26.8)	18.3 (12.3–25.9)
Chile	YLDs Rate: Cumulative change (%)	2.8 (-6.5 to 15.7)	22.7 (-6.3 to 63.1)	0.5 (-3.8 to 4.3)	-61.3 (-76.3 to -39.5)	-50.9 (-60.4 to -41.2)	20.9 (-3.7 to 61.8)	-1.0 (-31.0 to 23.8)	-7.6 (-20.6 to 6.0)	-1.8 (-33.0 to 40.0)	-4.2 (-29.9 to 23.2)	249.9 (133.3–394.3)
Uruguay	Prevalence Number: 1990	195,166 (186,441–203,783)	95,827 (78,493–127,394)	48,007 (45,237–50,922)	124 (80–185)	723 (595–870)	19,572 (18,358–20,876)	14,337 (11,256–17,686)	4,129 (3,767–4,532)	0 (0–0)	274 (215–346)	-
Uruguay	Prevalence Number: 2015	173,400 (165,537–181,321)	88,070 (70,966–108,302)	43,569 (41,185–46,186)	16 (9–27)	268 (240–303)	16,115 (15,142–17,084)	10,407 (8,072–13,326)	3,518 (3,328–3,758)	0 (0–0)	241 (191–303)	-
Uruguay	Prevalence Number: Cumulative change (%	-11.1 (-16.3 to -5.7)	-6.6 (-29.9 to 18.5)	-9.2 (-15.9 to -2.5)	-86.3 (-91.3 to -79.9)	-62.6 (-67.6 to -56.9)	-17.5 (-24.3 to -10.3)	-27.4 (-35.8 to -18.5)	-14.6 (-22.5 to -6.2)	-20.2 (-36.3 to 0.3)	-11.9 (-17.9 to -5.4)	-
Uruguay	Prevalence Rate: 1990	71,646.0 (68,439.4–74,821.8)	35,129.5 (28,757.7–46,763.3)	17,640.5 (16,621.6–18,712.7)	45.4 (29.1–67.4)	264.8 (218.0–318.7)	7,182.2 (6,736.5–7,661.0)	5,275.3 (4,141.9–6,507.8)	1,507.3 (1,375.0–1,654.1)	0.0 (0.0–0.0)	100.7 (78.7–127.1)	-
Uruguay	Prevalence Rate: 2015	71,201.2 (67,982.8–74,439.5)	36,287.4 (29,282.1–44,577.0)	17,849.4 (16,874.2–18,919.2)	7.0 (4.1–11.3)	111.0 (99.4–125.2)	6,623.9 (6,224.1–7,020.8)	4,251.3 (3,297.6–5,443.7)	1,464.5 (1,386.4–1,563.7)	0.0 (0.0–0.0)	99.7 (79.0–125.4)	-
Uruguay	Prevalence Rate: Cumulative change (%	-0.6 (-6.3 to 5.5)	4.9 (-21.3 to 33.1)	1.3 (-6.2 to 8.7)	-84.2 (-90.0 to -76.9)	-57.7 (-63.5 to -51.3)	-7.6 (-15.2 to 0.5)	-19.4 (-28.7 to -9.5)	-2.6 (-11.6 to 6.9)	-11.1 (-29.1 to 11.8)	-0.7 (-7.5 to 6.6)	-
Uruguay	YLDs Number: 1990	8,716 (6,230–11,757)	2,930 (1,809–4,677)	1,516 (1,073–2,027)	15 (8–24)	119 (81–167)	130 (80–204)	659 (430–950)	282 (212–364)	0 (0–0)	52 (33–75)	36 (24–51)
Uruguay	YLDs Number: 2015	7,431 (5,397–9,944)	2,749 (1,718–4,163)	1,369 (965–1,833)	2 (0–3)	44 (30–61)	121 (77–177)	478 (303–698)	189 (141–250)	0 (0–0)	44 (28–63)	55 (37–76)
Uruguay	YLDs Number: Cumulative change (%)	-14.3 (-29.2 to 0.9)	-2.6 (-38.9 to 38.1)	-9.7 (-13.3 to -5.9)	-86.1 (-92.4 to -78.2)	-62.5 (-70.3 to -53.9)	-4.0 (-37.1 to 36.8)	-27.4 (-36.3 to -17.7)	-32.7 (-41.8 to -22.8)	-17.2 (-44.5 to 13.9)	-13.5 (-35.1 to 12.6)	54.0 (5.5–113.1)
Uruguay	YLDs Rate: 1990	3,198.4 (2,287.2–4,312.5)	1,073.4 (662.3–1,717.2)	557.4 (394.8–745.6)	5.5 (3.1–8.9)	43.8 (29.9–61.4)	47.8 (29.5–74.8)	242.6 (158.4–349.8)	103.8 (78.2–133.6)	0.0 (0.0–0.0)	19.2 (12.4–27.6)	13.6 (9.2–18.9)
Uruguay	YLDs Rate: 2015	3,055.4 (2,222.4–4,087.0)	1,135.5 (712.7–1,716.0)	560.1 (394.9–750.4)	0.9 (0.4–1.6)	18.3 (12.6–25.5)	50.0 (32.0–73.1)	195.4 (124.0–285.2)	78.0 (58.1–103.0)	0.0 (0.0–0.0)	18.3 (11.6–26.3)	22.8 (15.3–31.6)
Uruguay	YLDs Rate: Cumulative change (%)	-4.0 (-20.7 to 13.0)	9.8 (-31.2 to 55.5)	0.5 (-3.5 to 4.7)	-84.1 (-91.3 to -74.9)	-57.7 (-66.5 to -47.9)	8.2 (-29.2 to 53.9)	-19.4 (-29.3 to -8.6)	-24.6 (-34.8 to -13.5)	-7.7 (-38.1 to 26.9)	-3.2 (-27.2 to 26.0)	72.5 (18.1–138.9)
Central Europe, Eastern Europe, and Central Asia	Prevalence Number: 1990	27,535,489 (27,135,219–27,981,084)	10,280,838 (9,071,548–11,788,375)	7,510,001 (7,331,116–7,690,663)	761,952 (614,342–946,147)	519,891 (468,922–574,956)	3,005,884 (2,919,085–3,103,235)	642,075 (479,037–802,913)	702,319 (644,672–760,216)	978 (459–1,600)	100,650 (79,505–126,569)	-
Central Europe, Eastern Europe, and Central Asia	Prevalence Number: 2015	21,637,587 (21,317,943–21,973,859)	7,875,052 (7,052,881–8,894,238)	5,935,177 (5,769,608–6,084,580)	430,919 (336,921–554,280)	233,860 (212,622–256,222)	2,188,219 (2,127,214–2,257,792)	373,951 (270,245–491,919)	566,790 (519,080–614,230)	5 (1–19)	79,216 (63,014–98,460)	-
Central Europe, Eastern Europe, and Central Asia	Prevalence Number: Cumulative change (%	-21.4 (-23.0 to -19.8)	-22.8 (-34.9 to -5.5)	-21.0 (-23.2 to -18.7)	-42.5 (-59.5 to -20.2)	-55.0 (-58.3 to -51.6)	-27.2 (-29.9 to -24.4)	-41.7 (-52.5 to -32.7)	-19.3 (-21.9 to -16.6)	-99.4 (-99.9 to -97.7)	-21.2 (-23.8 to -18.3)	-
Central Europe, Eastern Europe, and Central Asia	Prevalence Rate: 1990	78,971.9 (77,835.0–80,245.4)	29,743.2 (26,307.6–34,036.0)	21,456.3 (20,944.3–21,973.0)	2,194.9 (1,769.6–2,725.5)	1,502.3 (1,356.5–1,661.0)	8,647.3 (8,397.1–8,926.2)	1,820.4 (1,358.2–2,276.4)	2,085.8 (1,916.5–2,258.3)	2.8 (1.3–4.6)	292.8 (232.0–367.7)	-

© 2017 Global Burden of Disease Child and Adolescent Health Collaboration. JAMA Pediatrics.

eTable 4. Prevalent cases, Rates (per 100,000 population), Years Lived with Disability (YLDs), and Cumulative Percent Change with 95% Uncertainty Interval (UI) for the Top 10 Global Causes of YLDsin Children and Adolescents in 195 Countries and Territories, Aged Under 5 Years, Both Sexes, 1990 and 2015. *Note: best viewed by enlarging in browser.*

Location	Measure	All causes	Iron-deficiency anemia	Skin and subcutaneous diseases	Protein-energy malnutrition	Diarrheal diseases	Hemoglobinopathies and hemolytic anemias	Asthma	Neonatal preterm birth complications	Malaria	Neonatal encephalopathy due to birth asphyxia and trauma	Other neonatal disorders
Central Europe, Eastern Europe, and Central Asia	Prevalence Rate: 2015	78,245.4 (77,092.7–79,463.6)	28,524.4 (25,551.6–32,209.9)	21,448.1 (20,850.2–21,988.1)	1,559.5 (1,219.3–2,006.0)	846.9 (769.9–927.9)	7,917.5 (7,696.8–8,169.2)	1,349.6 (975.4–1,775.4)	2,062.3 (1,888.5–2,235.2)	0.0 (0.0–0.1)	287.2 (228.5–356.8)	-
Central Europe, Eastern Europe, and Central Asia	Prevalence Rate: Cumulative change (%	-0.9 (-2.8 to 1.1)	-3.4 (-18.3 to 18.2)	-0.0 (-2.9 to 2.8)	-27.7 (-49.1 to 0.2)	-43.6 (-47.8 to -39.4)	-8.4 (-11.9 to -5.0)	-25.7 (-39.6 to -14.3)	-1.1 (-4.2 to 2.0)	-99.2 (-99.9 to -97.1)	-1.8 (-5.0 to 1.7)	-
Central Europe, Eastern Europe, and Central Asia	YLDs Number: 1990	1,332,005 (993,589–1,724,633)	307,466 (205,140–431,921)	192,172 (133,473–259,691)	95,336 (62,267–134,884)	85,518 (61,739–114,463)	25,459 (16,767–36,246)	29,437 (18,637–42,309)	48,279 (37,170–61,338)	20 (8–37)	19,957 (13,684–27,552)	12,183 (8,971–15,823)
Central Europe, Eastern Europe, and Central Asia	YLDs Number: 2015	938,633 (689,656–1,204,553)	231,341 (157,245–324,100)	151,674 (104,625–206,046)	54,046 (34,196–78,239)	38,572 (27,543–51,488)	18,909 (12,844–26,390)	17,186 (10,673–25,445)	30,826 (23,374–39,943)	0 (0–0)	15,026 (10,422–20,822)	10,819 (7,807–14,180)
Central Europe, Eastern Europe, and Central Asia	YLDs Number: Cumulative change (%)	-29.4 (-35.7 to -21.5)	-23.8 (-39.7 to 3.4)	-21.1 (-22.2 to -19.9)	-42.3 (-59.5 to -19.7)	-54.9 (-58.2 to -51.3)	-24.4 (-41.7 to 4.5)	-41.5 (-52.4 to -32.9)	-36.2 (-39.3 to -32.8)	-99.2 (-99.9 to -96.8)	-24.6 (-29.7 to -19.0)	-10.5 (-29.3 to 11.8)
Central Europe, Eastern Europe, and Central Asia	YLDs Rate: 1990	3,834.9 (2,862.4–4,962.2)	892.7 (597.4–1,253.2)	547.9 (380.4–740.5)	274.6 (179.4–388.5)	247.1 (178.5–330.4)	73.9 (48.7–104.9)	83.5 (52.8–120.0)	139.1 (107.1–176.7)	0.1 (0.0–0.1)	57.4 (39.4–79.2)	35.1 (25.9–45.6)
Central Europe, Eastern Europe, and Central Asia	YLDs Rate: 2015	3,396.6 (2,495.8–4,358.3)	838.5 (570.0–1,174.2)	547.9 (377.9–744.4)	195.6 (123.8–283.1)	139.7 (99.8–186.5)	68.5 (46.6–95.6)	62.0 (38.5–91.8)	111.6 (84.6–144.6)	0.0 (0.0–0.0)	54.4 (37.7–75.3)	39.2 (28.3–51.3)
Central Europe, Eastern Europe, and Central Asia	YLDs Rate: Cumulative change (%)	-11.3 (-19.2 to -1.3)	-4.9 (-24.5 to 28.8)	0.0 (-1.4 to 1.5)	-27.6 (-49.1 to 0.8)	-43.4 (-47.6 to -39.0)	-5.7 (-27.0 to 30.1)	-25.6 (-39.4 to -14.6)	-19.8 (-23.8 to -15.6)	-98.9 (-99.8 to -96.0)	-5.1 (-11.5 to 1.8)	12.4 (-11.2 to 40.3)
Eastern Europe	Prevalence Number: 1990	12,243,281 (11,882,623–12,653,252)	4,051,312 (2,929,237–5,554,690)	3,578,995 (3,422,276–3,728,276)	283,347 (173,927–426,498)	168,273 (152,933–185,496)	1,250,880 (1,194,644–1,310,309)	322,447 (236,741–416,242)	260,532 (236,080–284,942)	0 (0–0)	46,823 (36,823–59,001)	-
Eastern Europe	Prevalence Number: 2015	9,277,913 (9,012,018–9,576,375)	3,081,602 (2,384,150–4,056,008)	2,716,439 (2,591,378–2,828,626)	143,519 (73,572–253,406)	80,343 (73,215–87,885)	884,943 (842,927–931,927)	147,829 (106,388–194,776)	198,036 (180,004–217,543)	0 (0–0)	35,313 (28,376–43,559)	-
Eastern Europe	Prevalence Number: Cumulative change (%	-24.2 (-27.3 to -21.0)	-20.6 (-46.1 to 26.6)	-24.1 (-28.0 to -19.9)	-45.0 (-77.3 to 8.1)	-52.2 (-55.8 to -49.0)	-29.2 (-33.8 to -24.4)	-53.7 (-66.3 to -43.8)	-23.9 (-28.8 to -18.5)	-24.3 (-24.7 to -23.9)	-24.4 (-29.0 to -19.5)	-
Eastern Europe	Prevalence Rate: 1990	75,101.4 (72,911.4–77,613.8)	25,238.3 (18,391.1–34,424.9)	21,822.1 (20,870.1–22,728.3)	1,749.8 (1,074.1–2,633.7)	1,043.9 (950.8–1,149.9)	7,712.1 (7,366.4–8,071.8)	1,942.0 (1,425.8–2,506.9)	1,685.6 (1,530.2–1,842.6)	0.0 (0.0–0.0)	293.6 (232.1–369.7)	-
Eastern Europe	Prevalence Rate: 2015	74,484.0 (72,348.4–76,879.7)	24,761.4 (19,164.0–32,578.1)	21,800.0 (20,796.0–22,700.1)	1,152.7 (590.9–2,035.3)	645.3 (588.0–705.8)	7,106.5 (6,769.1–7,483.7)	1,185.7 (853.3–1,562.2)	1,594.6 (1,449.6–1,751.4)	0.0 (0.0–0.0)	283.9 (228.2–350.2)	-
Eastern Europe	Prevalence Rate: Cumulative change (%	-0.8 (-4.9 to 3.4)	2.3 (-30.1 to 63.2)	-0.1 (-5.2 to 5.4)	-28.5 (-70.5 to 40.7)	-38.1 (-42.8 to -34.0)	-7.8 (-13.8 to -1.5)	-38.3 (-55.1 to -25.1)	-5.3 (-11.1 to 1.2)	-0.0 (-0.0 to 0.0)	-3.1 (-8.9 to 2.8)	-
Eastern Europe	YLDs Number: 1990	544,698 (399,492–716,970)	110,186 (60,378–186,756)	91,061 (63,501–123,627)	35,514 (18,767–58,629)	27,789 (19,937–37,277)	10,614 (5,762–18,052)	14,802 (9,087–21,995)	19,244 (14,728–24,961)	0 (0–0)	9,361 (6,296–13,016)	4,761 (3,466–6,159)
Eastern Europe	YLDs Number: 2015	378,131 (270,928–494,495)	83,064 (48,729–137,490)	69,058 (47,551–94,106)	18,040 (8,102–32,836)	13,302 (9,451–17,883)	6,856 (3,977–11,197)	6,799 (4,169–10,257)	10,417 (7,806–13,654)	0 (0–0)	6,691 (4,588–9,191)	4,951 (3,415–6,647)
Eastern Europe	YLDs Number: Cumulative change (%)	-30.2 (-41.6 to -12.2)	-17.7 (-53.0 to 77.4)	-24.2 (-26.1 to -22.2)	-44.9 (-77.7 to 8.5)	-52.1 (-56.7 to -47.5)	-28.8 (-61.0 to 52.0)	-53.6 (-66.0 to -43.2)	-45.8 (-51.6 to -39.9)	-17.0 (-49.7 to 34.7)	-28.2 (-37.5 to -17.6)	4.5 (-18.3 to 31.0)
Eastern Europe	YLDs Rate: 1990	3,358.4 (2,462.5–4,418.0)	690.0 (381.0–1,158.5)	553.3 (385.6–751.4)	219.3 (115.9–362.1)	172.4 (123.7–231.0)	66.4 (36.3–112.1)	89.2 (54.7–132.5)	118.6 (90.6–153.5)	0.0 (0.0–0.0)	57.7 (38.9–80.2)	29.4 (21.4–38.0)
Eastern Europe	YLDs Rate: 2015	3,036.6 (2,175.6–3,970.7)	667.7 (391.7–1,104.6)	554.1 (381.5–755.1)	144.9 (65.1–263.7)	106.7 (75.9–143.6)	55.1 (32.0–90.0)	54.5 (33.4–82.3)	83.7 (62.7–109.7)	0.0 (0.0–0.0)	53.7 (36.8–73.8)	39.8 (27.4–53.4)
Eastern Europe	YLDs Rate: Cumulative change (%)	-9.1 (-23.8 to 14.3)	5.4 (-39.2 to 126.7)	0.1 (-2.5 to 2.8)	-28.4 (-71.0 to 41.1)	-38.0 (-43.8 to -32.0)	-8.8 (-49.5 to 94.9)	-38.2 (-54.8 to -24.4)	-29.4 (-36.9 to -21.8)	9.4 (-33.4 to 77.3)	-6.4 (-18.6 to 7.4)	36.0 (6.1–70.6)
Belarus	Prevalence Number: 1990	576,963 (554,291–598,065)	184,472 (140,147–233,867)	180,774 (171,427–190,571)	11,024 (4,477–21,105)	7,491 (6,729–8,353)	58,587 (55,122–61,909)	11,540 (8,334–15,120)	12,287 (11,023–13,586)	0 (0–0)	2,181 (1,736–2,707)	-
Belarus	Prevalence Number: 2015	418,919 (402,969–434,319)	146,671 (117,024–183,513)	131,510 (124,461–138,464)	4,659 (1,863–9,239)	2,727 (2,501–2,948)	39,639 (37,335–42,001)	8,318 (5,854–10,990)	8,130 (7,331–8,980)	0 (0–0)	1,527 (1,233–1,875)	-
Belarus	Prevalence Number: Cumulative change (%	-27.4 (-31.0 to -23.6)	-18.8 (-42.3 to 10.4)	-27.2 (-32.4 to -22.4)	-46.7 (-86.2 to 22.1)	-63.5 (-66.6 to -60.0)	-32.3 (-37.5 to -26.6)	-27.6 (-40.5 to -12.0)	-33.8 (-39.1 to -28.6)	-27.7 (-28.0 to -27.4)	-29.8 (-34.7 to -24.6)	-
Belarus	Prevalence Rate: 1990	76,424.6 (73,359.5–79,184.0)	24,838.9 (18,927.8–31,280.2)	23,808.7 (22,582.8–25,095.5)	1,468.8 (596.5–2,811.3)	1,001.6 (900.1–1,115.9)	7,797.0 (7,341.2–8,239.4)	1,505.7 (1,087.5–1,972.9)	1,703.1 (1,531.5–1,880.9)	0.0 (0.0–0.0)	294.3 (234.5–365.0)	-
Belarus	Prevalence Rate: 2015	76,172.9 (73,272.9–78,972.0)	26,685.0 (21,297.7–33,385.4)	23,906.5 (22,625.4–25,170.5)	847.5 (339.0–1,680.4)	496.1 (455.1–536.4)	7,209.0 (6,790.2–7,638.7)	1,511.7 (1,064.1–1,997.4)	1,480.8 (1,335.4–1,635.7)	0.0 (0.0–0.0)	278.0 (224.4–341.2)	-
Belarus	Prevalence Rate: Cumulative change (%	-0.3 (-5.3 to 4.9)	9.7 (-22.4 to 47.7)	0.5 (-6.8 to 7.1)	-27.3 (-81.2 to 66.7)	-50.4 (-54.6 to -45.5)	-7.4 (-14.6 to 0.3)	0.8 (-17.1 to 22.6)	-13.0 (-19.8 to -6.2)	-0.0 (-0.0 to 0.0)	-5.3 (-11.8 to 1.6)	-
Belarus	YLDs Number: 1990	24,903 (18,291–32,426)	5,064 (3,020–7,966)	5,535 (3,864–7,462)	1,381 (513–2,800)	1,239 (879–1,672)	404 (240–625)	529 (325–788)	872 (645–1,141)	0 (0–0)	437 (295–603)	336 (113–688)
Belarus	YLDs Number: 2015	16,501 (11,747–22,016)	4,072 (2,403–6,312)	4,003 (2,818–5,397)	586 (207–1,181)	450 (317–601)	299 (173–465)	382 (228–576)	401 (294–530)	0 (0–0)	289 (201–402)	196 (65–430)
Belarus	YLDs Number: Cumulative change (%)	-33.6 (-42.4 to -24.3)	-16.4 (-47.0 to 22.4)	-27.7 (-30.4 to -25.0)	-46.6 (-86.1 to 23.6)	-63.5 (-67.8 to -58.8)	-23.2 (-51.5 to 13.6)	-27.3 (-41.1 to -10.4)	-53.8 (-60.0 to -47.2)	-19.0 (-49.7 to 28.4)	-33.3 (-44.4 to -21.0)	-20.1 (-84.1 to 117.6)
Belarus	YLDs Rate: 1990	3,314.3 (2,435.0–4,320.5)	686.8 (409.9–1,072.0)	726.4 (507.0–979.0)	184.0 (68.4–373.1)	165.7 (117.7–223.8)	54.9 (32.4–84.1)	69.1 (42.5–102.9)	116.0 (86.0–151.8)	0.0 (0.0–0.0)	58.2 (39.2–80.2)	44.8 (15.1–91.8)
Belarus	YLDs Rate: 2015	3,001.0 (2,136.4–4,003.9)	741.1 (437.7–1,148.5)	727.7 (512.2–981.1)	106.7 (37.8–214.9)	82.0 (57.7–109.4)	54.6 (31.6–84.8)	69.6 (41.5–104.8)	73.1 (53.6–96.5)	0.0 (0.0–0.0)	52.7 (36.6–73.2)	35.7 (11.9–78.3)
Belarus	YLDs Rate: Cumulative change (%)	-9.3 (-21.3 to 3.5)	12.0 (-28.9 to 64.8)	0.2 (-3.6 to 3.8)	-27.0 (-81.1 to 68.7)	-50.4 (-56.1 to -44.0)	3.0 (-34.8 to 52.3)	1.3 (-18.0 to 24.8)	-36.9 (-45.3 to -27.9)	11.8 (-30.6 to 76.6)	-8.7 (-23.9 to 7.8)	9.1 (-78.3 to 197.2)
Estonia	Prevalence Number: 1990	86,799 (83,633–89,900)	30,362 (22,546–40,811)	24,789 (23,346–26,212)	2,324 (1,011–4,609)	865 (793–943)	8,709 (8,200–9,206)	1,919 (1,347–2,466)	1,868 (1,708–2,026)	0 (0–0)	323 (254–411)	-
Estonia	Prevalence Number: 2015	54,115 (51,785–56,401)	18,617 (13,130–23,190)	15,757 (14,892–16,615)	786 (294–1,592)	341 (318–365)	5,076 (4,800–5,372)	1,134 (802–1,516)	1,013 (911–1,118)	0 (0–0)	194 (155–244)	-
Estonia	Prevalence Number: Cumulative change (%	-37.6 (-40.8 to -34.1)	-37.1 (-55.8 to -10.6)	-36.4 (-41.1 to -31.7)	-58.0 (-89.7 to 1.7)	-60.5 (-63.0 to -58.2)	-41.6 (-45.9 to -37.1)	-40.4 (-54.2 to -27.7)	-45.7 (-50.1 to -40.6)	-37.0 (-37.2 to -36.9)	-39.7 (-44.5 to -34.5)	-
Estonia	Prevalence Rate: 1990	75,854.4 (73,090.9–78,507.7)	26,858.3 (20,012.7–35,952.5)	21,565.0 (20,316.7–22,805.8)	2,046.7 (890.4–4,058.3)	764.0 (700.3–833.2)	7,651.5 (7,204.5–8,090.3)	1,649.9 (1,158.1–2,120.5)	1,721.3 (1,574.9–1,862.6)	0.0 (0.0–0.0)	289.2 (227.9–366.1)	-
Estonia	Prevalence Rate: 2015	74,797.5 (71,584.0–77,924.2)	25,907.1 (18,345.7–32,242.2)	21,734.0 (20,545.6–22,920.2)	1,090.3 (408.8–2,207.7)	473.9 (442.4–507.5)	7,034.6 (6,653.1–7,446.0)	1,556.6 (1,101.2–2,080.2)	1,437.1 (1,293.3–1,581.9)	0.0 (0.0–0.0)	272.1 (217.5–341.5)	-
Estonia	Prevalence Rate: Cumulative change (%	-1.3 (-6.3 to 4.2)	-1.2 (-29.9 to 40.3)	0.9 (-6.6 to 8.3)	-33.9 (-83.7 to 60.1)	-37.9 (-41.8 to -34.3)	-8.0 (-14.6 to -0.8)	-4.9 (-27.0 to 15.4)	-16.4 (-23.0 to -8.9)	0.0 (-0.0 to 0.0)	-5.7 (-13.0 to 2.5)	-
Estonia	YLDs Number: 1990	3,806 (2,766–5,052)	820 (449–1,368)	615 (425–837)	291 (113–583)	143 (102–191)	64 (35–109)	88 (55–130)	133 (100–173)	0 (0–0)	64 (43–92)	167 (111–240)
Estonia	YLDs Number: 2015	1,954 (1,385–2,615)	504 (253–790)	389 (269–530)	98 (33–212)	56 (39–75)	36 (18–56)	52 (32–79)	47 (35–62)	0 (0–0)	36 (24–51)	20 (13–29)
Estonia	YLDs Number: Cumulative change (%)	-48.4 (-56.9 to -37.4)	-34.7 (-61.9 to 25.5)	-36.8 (-39.2 to -34.4)	-58.0 (-89.7 to 3.4)	-60.4 (-64.8 to -55.9)	-39.5 (-65.6 to 18.6)	-40.2 (-55.0 to -26.1)	-64.3 (-69.5 to -59.1)	-31.2 (-59.6 to 27.7)	-42.9 (-53.1 to -32.0)	-87.6 (-92.1 to -82.2)
Estonia	YLDs Rate: 1990	3,343.0 (2,431.7–4,438.2)	728.4 (403.4–1,209.5)	534.2 (369.0–726.6)	256.9 (99.7–513.4)	126.3 (90.3–169.6)	57.7 (31.3–96.7)	75.8 (47.6–112.0)	117.5 (88.5–152.2)	0.0 (0.0–0.0)	57.0 (38.6–81.2)	147.7 (97.8–211.8)
Estonia	YLDs Rate: 2015	2,707.7 (1,920.3–3,624.5)	703.9 (356.2–1,098.5)	536.4 (371.9–730.6)	137.3 (46.3–295.3)	78.5 (55.0–105.3)	51.3 (26.0–79.2)	71.7 (43.9–108.5)	66.0 (48.8–87.1)	0.0 (0.0–0.0)	51.0 (34.4–71.8)	28.1 (18.4–40.7)
Estonia	YLDs Rate: Cumulative change (%)	-18.7 (-32.1 to -1.4)	2.4 (-39.9 to 94.5)	0.4 (-3.4 to 4.2)	-33.8 (-83.7 to 62.9)	-37.7 (-44.5 to -30.7)	-5.2 (-45.8 to 82.9)	-4.6 (-28.2 to 18.0)	-43.7 (-51.9 to -35.6)	9.2 (-35.6 to 101.7)	-9.9 (-26.0 to 7.0)	-80.4 (-87.6 to -71.9)

© 2017 Global Burden of Disease Child and Adolescent Health Collaboration. JAMA Pediatrics.

eTable 4. Prevalent cases, Rates (per 100,000 population), Years Lived with Disability (YLDs), and Cumulative Percent Change with 95% Uncertainty Interval (UI) for the Top 10 Global Causes of YLDsin Children and Adolescents in 195 Countries and Territories, Aged Under 5 Years, Both Sexes, 1990 and 2015. *Note: best viewed by enlarging in browser.*

Location	Measure	All causes	Iron-deficiency anemia	Skin and subcutaneous diseases	Protein-energy malnutrition	Diarrheal diseases	Hemoglobinopathies and hemolytic anemias	Asthma	Neonatal preterm birth complications	Malaria	Neonatal encephalopathy due to birth asphyxia and trauma	Other neonatal disorders
Latvia	Prevalence Number: 1990	143,874 (139,915–148,164)	47,232 (37,166–57,517)	40,981 (38,402–43,546)	3,250 (1,276–6,454)	1,130 (1,034–1,248)	14,748 (13,910–15,672)	3,277 (2,454–4,181)	3,069 (2,785–3,378)	0 (0–0)	554 (441–701)	-
Latvia	Prevalence Number: 2015	76,736 (73,673–79,743)	27,000 (20,499–34,350)	21,644 (20,251–22,861)	1,041 (423–2,021)	407 (377–435)	7,264 (6,836–7,647)	1,628 (1,142–2,207)	1,443 (1,310–1,588)	0 (0–0)	281 (225–349)	-
Latvia	Prevalence Number: Cumulative change (%	-46.7 (-49.0 to -44.2)	-42.0 (-57.3 to -19.7)	-47.1 (-51.1 to -43.0)	-59.3 (-89.6 to -1.2)	-63.9 (-66.4 to -61.6)	-50.7 (-54.6 to -46.9)	-50.3 (-59.9 to -39.9)	-52.9 (-57.1 to -48.5)	-47.5 (-47.6 to -47.4)	-49.1 (-53.3 to -45.3)	-
Latvia	Prevalence Rate: 1990	74,145.3 (72,135.8–76,329.4)	24,811.7 (19,696.1–29,944.6)	21,004.3 (19,684.4–22,320.3)	1,683.4 (660.8–3,342.2)	588.1 (538.6–648.3)	7,633.4 (7,203.3–8,111.0)	1,662.1 (1,244.4–2,120.2)	1,653.2 (1,500.7–1,815.9)	0.0 (0.0–0.0)	290.9 (231.9–366.4)	-
Latvia	Prevalence Rate: 2015	74,946.4 (71,987.3–77,869.0)	26,579.9 (20,251.9–33,747.9)	21,088.9 (19,734.3–22,275.0)	1,020.8 (415.5–1,981.2)	400.3 (371.6–427.6)	7,113.2 (6,694.1–7,488.4)	1,577.3 (1,106.0–2,137.2)	1,449.3 (1,316.2–1,592.4)	0.0 (0.0–0.0)	278.2 (223.1–344.6)	-
Latvia	Prevalence Rate: Cumulative change (%	1.1 (-3.3 to 5.8)	8.6 (-19.8 to 49.8)	0.5 (-7.1 to 8.3)	-23.1 (-80.3 to 87.0)	-31.8 (-36.5 to -27.6)	-6.7 (-14.0 to 0.5)	-5.0 (-23.4 to 14.7)	-12.2 (-19.8 to -4.2)	0.0 (-0.0 to 0.0)	-4.1 (-12.2 to 3.1)	-
Latvia	YLDs Number: 1990	6,099 (4,479–8,043)	1,316 (815–1,948)	961 (649–1,320)	407 (144–844)	187 (132–251)	102 (62–151)	150 (97–220)	220 (167–290)	0 (0–0)	110 (75–156)	190 (127–273)
Latvia	YLDs Number: 2015	2,874 (2,065–3,847)	730 (422–1,183)	507 (345–692)	130 (49–250)	67 (47–91)	53 (30–85)	74 (45–113)	70 (51–94)	0 (0–0)	53 (35–75)	33 (22–47)
Latvia	YLDs Number: Cumulative change (%)	-52.7 (-59.4 to -43.4)	-42.9 (-65.0 to 8.1)	-47.3 (-48.7 to -45.8)	-59.2 (-89.5 to 1.1)	-63.8 (-67.7 to -59.9)	-45.9 (-66.5 to 1.2)	-50.2 (-61.1 to -38.7)	-67.9 (-72.1 to -63.6)	-39.4 (-62.1 to 10.6)	-51.6 (-60.4 to -41.6)	-81.8 (-87.9 to -74.1)
Latvia	YLDs Rate: 1990	3,163.6 (2,320.0–4,162.2)	697.3 (434.1–1,031.1)	491.8 (332.0–675.5)	211.1 (74.6–437.1)	97.3 (69.0–130.8)	54.2 (33.8–80.0)	76.5 (49.5–111.7)	114.0 (86.4–150.3)	0.0 (0.0–0.0)	57.1 (38.9–80.8)	98.7 (65.7–141.3)
Latvia	YLDs Rate: 2015	2,815.3 (2,024.2–3,767.2)	721.0 (418.2–1,164.2)	493.3 (335.9–673.4)	127.8 (48.4–245.2)	66.3 (46.6–89.6)	53.1 (30.3–84.2)	72.6 (44.1–109.9)	69.2 (50.4–92.2)	0.0 (0.0–0.0)	52.0 (34.8–73.7)	33.1 (22.1–46.9)
Latvia	YLDs Rate: Cumulative change (%)	-10.8 (-23.4 to 6.9)	6.3 (-34.3 to 99.0)	0.3 (-2.5 to 3.0)	-22.9 (-80.2 to 91.6)	-31.7 (-39.1 to -24.4)	0.6 (-37.2 to 86.6)	-4.9 (-25.7 to 17.0)	-39.3 (-47.1 to -31.1)	15.2 (-28.0 to 109.3)	-8.3 (-24.9 to 10.2)	-65.5 (-77.0 to -51.0)
Lithuania	Prevalence Number: 1990	209,920 (202,937–216,785)	69,765 (50,836–93,475)	59,300 (55,690–62,989)	5,310 (2,234–10,221)	1,735 (1,598–1,897)	21,327 (20,076–22,699)	3,652 (2,646–4,855)	4,208 (3,805–4,633)	0 (0–0)	807 (646–1,011)	-
Lithuania	Prevalence Number: 2015	114,777 (110,507–119,116)	38,647 (28,917–50,824)	32,530 (30,567–34,473)	1,619 (649–3,270)	644 (597–693)	10,768 (10,126–11,364)	2,151 (1,534–2,897)	2,004 (1,808–2,215)	0 (0–0)	417 (332–517)	-
Lithuania	Prevalence Number: Cumulative change (%	-45.3 (-47.9 to -42.7)	-42.1 (-66.9 to -12.0)	-45.1 (-49.3 to -40.8)	-62.5 (-90.0 to -7.6)	-62.8 (-65.0 to -60.7)	-49.4 (-53.6 to -45.4)	-40.9 (-49.5 to -29.0)	-52.3 (-56.5 to -48.2)	-45.5 (-45.5 to -45.5)	-48.2 (-51.7 to -44.4)	-
Lithuania	Prevalence Rate: 1990	75,189.8 (72,698.3–77,633.1)	25,144.3 (18,444.8–33,544.2)	21,199.0 (19,909.6–22,513.9)	1,907.3 (802.4–3,671.5)	624.3 (575.0–682.2)	7,655.3 (7,207.8–8,146.1)	1,299.4 (941.4–1,727.4)	1,539.6 (1,392.6–1,692.5)	0.0 (0.0–0.0)	291.7 (233.2–365.4)	-
Lithuania	Prevalence Rate: 2015	75,391.0 (72,600.6–78,237.4)	25,576.9 (19,186.5–33,563.4)	21,325.4 (20,043.7–22,596.8)	1,066.2 (427.6–2,152.9)	425.0 (394.1–456.8)	7,087.4 (6,665.7–7,479.8)	1,404.2 (1,001.7–1,890.9)	1,343.2 (1,211.0–1,482.5)	0.0 (0.0–0.0)	276.5 (220.3–342.3)	-
Lithuania	Prevalence Rate: Cumulative change (%	0.3 (-4.3 to 5.0)	6.2 (-38.7 to 60.8)	0.7 (-6.9 to 8.5)	-31.3 (-81.7 to 69.3)	-31.9 (-35.9 to -28.0)	-7.3 (-14.8 to 0.2)	8.4 (-7.4 to 30.3)	-12.7 (-20.3 to -5.2)	0.0 (-0.0 to 0.0)	-5.0 (-11.6 to 2.0)	-
Lithuania	YLDs Number: 1990	8,561 (6,228–11,451)	1,902 (1,082–2,990)	1,405 (952–1,918)	670 (258–1,383)	287 (204–383)	148 (85–230)	167 (102–256)	259 (192–337)	0 (0–0)	159 (107–221)	70 (47–99)
Lithuania	YLDs Number: 2015	4,188 (3,020–5,475)	1,082 (624–1,653)	769 (526–1,053)	203 (75–402)	106 (76–143)	78 (45–118)	99 (59–147)	93 (69–122)	0 (0–0)	80 (54–112)	32 (21–46)
Lithuania	YLDs Number: Cumulative change (%)	-50.6 (-60.4 to -40.1)	-38.5 (-69.9 to -2.0)	-45.2 (-47.3 to -43.2)	-62.4 (-90.2 to -7.9)	-62.8 (-66.9 to -58.4)	-43.3 (-71.8 to -9.9)	-40.6 (-51.1 to -27.8)	-63.9 (-69.0 to -58.2)	-40.6 (-73.8 to 10.8)	-49.7 (-57.8 to -41.0)	-52.6 (-69.6 to -31.7)
Lithuania	YLDs Rate: 1990	3,073.4 (2,236.0–4,107.5)	687.0 (392.5–1,076.1)	502.0 (340.1–685.0)	240.9 (93.0–497.0)	103.6 (73.5–138.2)	53.7 (31.0–83.0)	59.7 (36.5–91.2)	93.0 (69.2–120.9)	0.0 (0.0–0.0)	57.4 (38.7–79.3)	25.5 (17.1–35.6)
Lithuania	YLDs Rate: 2015	2,759.0 (1,990.7–3,610.2)	718.4 (415.8–1,100.1)	504.1 (344.6–689.5)	133.7 (49.5–264.7)	70.4 (50.2–94.4)	52.0 (30.3–78.7)	64.7 (39.0–96.2)	61.3 (45.5–80.6)	0.0 (0.0–0.0)	52.7 (35.6–73.9)	21.6 (14.5–30.7)
Lithuania	YLDs Rate: Cumulative change (%)	-9.4 (-27.2 to 9.8)	12.8 (-44.3 to 78.8)	0.4 (-3.3 to 4.1)	-31.1 (-82.0 to 68.9)	-31.9 (-39.2 to -23.9)	4.0 (-47.7 to 64.5)	8.9 (-10.3 to 32.5)	-33.9 (-43.2 to -23.6)	8.9 (-51.8 to 103.0)	-7.7 (-22.7 to 8.1)	-13.1 (-44.2 to 25.1)
Moldova	Prevalence Number: 1990	327,788 (315,794–339,946)	116,349 (88,338–159,925)	93,448 (87,579–98,881)	13,276 (5,614–25,071)	5,880 (5,298–6,595)	34,883 (32,884–36,804)	5,889 (4,300–7,720)	7,824 (7,060–8,641)	0 (0–0)	1,268 (981–1,595)	-
Moldova	Prevalence Number: 2015	168,133 (161,670–175,573)	67,315 (52,616–91,113)	47,666 (44,632–50,488)	2,475 (1,164–4,518)	1,988 (1,769–2,227)	16,957 (16,034–17,982)	2,928 (2,091–3,920)	4,269 (3,868–4,764)	0 (0–0)	641 (511–784)	-
Moldova	Prevalence Number: Cumulative change (%	-48.7 (-51.4 to -45.8)	-40.1 (-63.7 to -5.1)	-48.9 (-53.1 to -44.8)	-76.9 (-92.6 to -45.8)	-66.2 (-68.8 to -63.5)	-51.3 (-54.8 to -47.5)	-50.2 (-57.7 to -41.8)	-45.4 (-50.2 to -40.4)	-48.9 (-49.0 to -48.8)	-49.2 (-53.6 to -45.0)	-
Moldova	Prevalence Rate: 1990	76,517.6 (73,742.2–79,330.3)	27,472.7 (20,923.9–37,488.9)	21,713.5 (20,343.5–22,971.0)	3,115.7 (1,317.8–5,884.4)	1,392.6 (1,256.1–1,559.9)	8,115.7 (7,705.6–8,625.4)	1,355.7 (990.0–1,777.2)	1,907.7 (1,720.9–2,101.8)	0.0 (0.0–0.0)	301.2 (233.0–377.7)	-
Moldova	Prevalence Rate: 2015	76,538.5 (73,611.1–79,901.9)	30,831.2 (24,185.8–41,612.9)	21,643.3 (20,267.8–22,922.0)	1,129.8 (531.7–2,062.6)	909.6 (809.5–1,018.2)	7,735.4 (7,316.4–8,203.0)	1,323.1 (944.8–1,771.3)	1,992.3 (1,806.2–2,220.8)	0.0 (0.0–0.0)	294.6 (235.1–360.5)	-
Moldova	Prevalence Rate: Cumulative change (%	0.1 (-5.2 to 5.8)	16.1 (-29.0 to 83.2)	-0.2 (-8.3 to 7.9)	-55.0 (-85.7 to 5.4)	-34.6 (-39.8 to -29.5)	-5.3 (-12.1 to 2.2)	-2.2 (-17.0 to 14.3)	4.6 (-4.3 to 13.7)	-0.0 (-0.0 to 0.0)	-1.8 (-10.1 to 6.4)	-
Moldova	YLDs Number: 1990	15,438 (11,155–20,491)	3,197 (1,820–5,420)	2,465 (1,705–3,393)	1,663 (645–3,310)	969 (692–1,288)	238 (134–403)	269 (167–398)	560 (429–711)	0 (0–0)	254 (173–350)	57 (36–88)
Moldova	YLDs Number: 2015	6,947 (5,010–9,430)	1,953 (1,144–3,396)	1,259 (870–1,722)	309 (131–576)	328 (231–445)	143 (85–248)	134 (83–201)	215 (161–277)	0 (0–0)	122 (83–167)	78 (52–112)
Moldova	YLDs Number: Cumulative change (%)	-54.7 (-64.7 to -41.6)	-34.4 (-73.4 to 27.3)	-48.9 (-50.8 to -47.0)	-76.8 (-92.7 to -45.4)	-66.1 (-69.4 to -62.5)	-35.1 (-72.7 to 25.8)	-50.0 (-58.3 to -40.1)	-61.4 (-66.3 to -56.1)	-41.0 (-70.4 to -2.5)	-51.3 (-59.8 to -41.7)	42.3 (-9.6 to 115.3)
Moldova	YLDs Rate: 1990	3,621.8 (2,613.3–4,803.6)	758.5 (434.0–1,280.7)	571.3 (395.2–786.7)	390.3 (151.5–776.9)	229.7 (164.4–305.1)	56.7 (32.1–94.9)	62.1 (38.6–91.6)	131.3 (100.6–166.8)	0.0 (0.0–0.0)	59.7 (40.6–82.0)	13.5 (8.7–20.8)
Moldova	YLDs Rate: 2015	3,170.1 (2,286.1–4,300.0)	896.7 (526.4–1,551.9)	570.8 (394.5–781.3)	141.2 (60.2–263.1)	150.3 (105.8–203.9)	66.1 (39.4–113.5)	60.8 (37.6–91.1)	98.5 (73.6–126.6)	0.0 (0.0–0.0)	56.0 (38.2–76.6)	35.9 (23.8–51.5)
Moldova	YLDs Rate: Cumulative change (%)	-11.8 (-31.2 to 13.5)	26.8 (-47.7 to 144.3)	-0.0 (-3.7 to 3.8)	-55.0 (-85.9 to 6.3)	-34.5 (-40.7 to -27.7)	25.3 (-46.5 to 141.6)	-1.9 (-18.1 to 17.6)	-24.9 (-34.3 to -14.8)	15.4 (-42.0 to 89.8)	-5.3 (-21.7 to 13.3)	176.7 (75.9–318.3)
Russia	Prevalence Number: 1990	8,293,866 (7,960,022–8,667,191)	2,633,084 (1,570,642–4,207,550)	2,453,098 (2,311,409–2,584,894)	197,362 (95,798–335,368)	119,824 (108,792–132,711)	839,469 (784,917–895,054)	225,117 (163,312–300,176)	174,149 (156,264–193,224)	0 (0–0)	31,934 (25,056–40,305)	-
Russia	Prevalence Number: 2015	6,633,309 (6,376,690–6,910,775)	2,208,494 (1,556,982–3,175,303)	1,948,960 (1,838,935–2,051,471)	117,986 (49,093–224,418)	59,272 (53,760–64,999)	627,422 (589,240–669,488)	93,352 (65,703–123,773)	138,468 (125,075–153,775)	0 (0–0)	25,267 (20,407–31,285)	-
Russia	Prevalence Number: Cumulative change (%	-20.0 (-24.7 to -15.1)	-6.9 (-48.1 to 80.4)	-20.5 (-26.2 to -14.3)	-30.1 (-78.5 to 67.0)	-50.5 (-54.8 to -46.4)	-25.1 (-31.8 to -18.0)	-57.9 (-71.5 to -46.2)	-20.4 (-27.4 to -12.8)	-20.8 (-21.3 to -20.3)	-20.7 (-26.6 to -13.8)	-
Russia	Prevalence Rate: 1990	74,368.4 (71,362.5–77,735.2)	24,022.0 (14,483.1–38,081.1)	21,847.2 (20,586.7–23,014.1)	1,782.5 (865.0–3,028.6)	1,087.1 (988.8–1,201.7)	7,568.7 (7,082.6–8,061.3)	1,977.9 (1,434.9–2,637.4)	1,654.5 (1,489.5–1,831.2)	0.0 (0.0–0.0)	293.4 (231.0–370.5)	-
Russia	Prevalence Rate: 2015	74,270.9 (71,400.3–77,375.7)	24,725.6 (17,429.5–35,547.7)	21,821.4 (20,589.4–22,969.1)	1,321.0 (549.7–2,512.6)	663.6 (601.9–727.7)	7,024.7 (6,597.2–7,495.6)	1,045.5 (735.8–1,386.1)	1,549.4 (1,399.5–1,720.7)	0.0 (0.0–0.0)	282.8 (228.4–350.2)	-
Russia	Prevalence Rate: Cumulative change (%	-0.1 (-5.9 to 6.0)	13.8 (-35.9 to 122.4)	-0.0 (-7.1 to 7.8)	-13.3 (-73.3 to 107.1)	-38.9 (-44.2 to -34.0)	-7.0 (-15.3 to 1.8)	-46.3 (-63.6 to -31.4)	-6.2 (-14.3 to 2.5)	0.0 (-0.0 to 0.0)	-3.3 (-10.6 to 4.9)	-
Russia	YLDs Number: 1990	374,355 (267,449–504,696)	70,710 (31,268–139,853)	61,996 (42,795–84,244)	24,705 (10,619–45,063)	19,781 (14,133–26,489)	7,407 (3,292–14,484)	10,337 (6,326–15,572)	13,193 (9,972–17,044)	0 (0–0)	6,426 (4,306–8,998)	2,869 (1,982–3,890)
Russia	YLDs Number: 2015	278,907 (195,953–373,998)	59,169 (30,535–110,381)	49,210 (33,901–66,694)	14,840 (5,555–28,780)	9,812 (6,919–13,216)	5,000 (2,586–9,155)	4,291 (2,566–6,513)	7,420 (5,501–9,759)	0 (0–0)	4,791 (3,237–6,609)	3,738 (2,496–5,162)

© 2017 Global Burden of Disease Child and Adolescent Health Collaboration. JAMA Pediatrics.

eTable 4. Prevalent cases, Rates (per 100,000 population), Years Lived with Disability (YLDs), and Cumulative Percent Change with 95% Uncertainty Interval (UI) for the Top 10 Global Causes of YLDsin Children and Adolescents in 195 Countries and Territories, Aged Under 5 Years, Both Sexes, 1990 and 2015. *Note: best viewed by enlarging in browser.*

Location	Measure	All causes	Iron-deficiency anemia	Skin and subcutaneous diseases	Protein-energy malnutrition	Diarrheal diseases	Hemoglobinopathies and hemolytic anemias	Asthma	Neonatal preterm birth complications	Malaria	Neonatal encephalopathy due to birth asphyxia and trauma	Other neonatal disorders
Russia	YLDs Number: Cumulative change (%)	-24.6 (-40.7 to 1.8)	3.4 (-57.1 to 207.8)	-20.6 (-23.4 to -17.7)	-29.8 (-78.4 to 67.4)	-50.3 (-55.8 to -44.1)	-17.3 (-65.8 to 151.6)	-57.8 (-71.4 to -45.6)	-43.7 (-50.8 to -35.8)	-6.8 (-55.7 to 80.9)	-24.8 (-37.9 to -9.2)	31.8 (-4.3 to 76.6)
Russia	YLDs Rate: 1990	3,374.6 (2,412.5–4,555.7)	648.6 (290.5–1,265.5)	550.1 (379.7–747.4)	223.1 (96.0–406.9)	179.5 (128.3–240.3)	67.8 (30.6–131.0)	90.8 (55.6–136.8)	118.9 (89.9–153.7)	0.0 (0.0–0.0)	57.9 (38.7–81.0)	25.9 (17.9–35.2)
Russia	YLDs Rate: 2015	3,122.8 (2,194.2–4,187.4)	662.5 (342.0–1,235.7)	551.0 (379.6–746.8)	166.2 (62.2–322.2)	109.9 (77.5–148.0)	56.0 (29.0–102.5)	48.1 (28.7–72.9)	83.1 (61.6–109.3)	0.0 (0.0–0.0)	53.7 (36.2–74.0)	41.9 (28.0–57.8)
Russia	YLDs Rate: Cumulative change (%)	-6.3 (-26.3 to 26.7)	25.3 (-47.4 to 272.8)	0.2 (-3.4 to 3.9)	-13.0 (-73.3 to 107.5)	-38.7 (-45.4 to -31.1)	0.4 (-57.8 to 204.9)	-46.2 (-63.5 to -30.6)	-30.0 (-38.9 to -20.4)	17.2 (-44.2 to 127.8)	-6.5 (-22.8 to 12.6)	63.5 (18.8–118.8)
Ukraine	Prevalence Number: 1990	2,604,068 (2,497,493–2,700,467)	970,044 (650,061–1,289,298)	726,601 (685,818–769,303)	50,797 (20,625–96,157)	31,346 (28,147–34,664)	273,152 (255,050–290,522)	71,050 (50,969–91,229)	57,123 (51,557–63,501)	0 (0–0)	9,752 (7,577–12,231)	-
Ukraine	Prevalence Number: 2015	1,811,921 (1,743,377–1,876,242)	574,855 (397,143–715,542)	518,369 (485,414–549,572)	14,950 (5,698–29,907)	14,962 (13,559–16,407)	177,814 (166,445–188,660)	38,314 (26,975–51,889)	42,706 (38,574–47,381)	0 (0–0)	6,982 (5,521–8,684)	-
Ukraine	Prevalence Number: Cumulative change (%	-30.4 (-33.8 to -26.6)	-39.2 (-58.2 to -15.1)	-28.6 (-33.9 to -23.0)	-63.8 (-90.2 to -9.1)	-52.2 (-55.8 to -48.5)	-34.8 (-40.2 to -29.4)	-45.6 (-60.9 to -32.7)	-25.1 (-31.3 to -17.8)	-28.5 (-28.7 to -28.3)	-28.3 (-33.0 to -23.0)	-
Ukraine	Prevalence Rate: 1990	77,083.0 (73,978.9–79,925.2)	29,027.8 (19,624.5–38,258.5)	21,416.0 (20,214.9–22,672.7)	1,512.2 (614.1–2,862.8)	937.0 (841.6–1,034.7)	8,118.9 (7,581.0–8,637.0)	2,073.5 (1,487.4–2,662.3)	1,765.9 (1,595.0–1,959.4)	0.0 (0.0–0.0)	293.9 (228.4–368.3)	-
Ukraine	Prevalence Rate: 2015	74,620.3 (71,831.1–77,253.2)	23,773.8 (16,444.7–29,558.6)	21,321.0 (19,967.7–22,603.7)	616.6 (235.0–1,233.5)	617.5 (559.7–677.1)	7,332.0 (6,863.1–7,778.4)	1,571.7 (1,106.6–2,128.6)	1,781.0 (1,609.3–1,976.0)	0.0 (0.0–0.0)	289.0 (228.8–359.3)	-
Ukraine	Prevalence Rate: Cumulative change (%	-3.1 (-7.8 to 2.0)	-16.1 (-41.7 to 15.2)	-0.3 (-7.8 to 7.5)	-49.8 (-86.5 to 25.9)	-34.0 (-39.0 to -28.8)	-9.6 (-17.0 to -2.0)	-23.5 (-45.1 to -5.5)	1.0 (-7.1 to 10.6)	-0.0 (-0.0 to 0.0)	-1.5 (-7.9 to 5.6)	-
Ukraine	YLDs Number: 1990	111,533 (80,891–148,493)	27,174 (14,477–44,094)	18,080 (12,448–24,616)	6,394 (2,309–12,837)	5,180 (3,689–7,006)	2,247 (1,224–3,643)	3,257 (1,999–4,845)	4,005 (3,049–5,217)	0 (0–0)	1,907 (1,275–2,660)	1,068 (753–1,449)
Ukraine	YLDs Number: 2015	66,757 (49,201–86,687)	15,552 (9,111–23,327)	12,919 (8,792–17,767)	1,871 (664–3,959)	2,479 (1,742–3,358)	1,242 (732–1,861)	1,764 (1,078–2,717)	2,167 (1,573–2,856)	0 (0–0)	1,316 (878–1,836)	851 (572–1,178)
Ukraine	YLDs Number: Cumulative change (%)	-39.8 (-47.9 to -31.4)	-39.7 (-64.9 to -10.8)	-28.6 (-31.6 to -25.3)	-63.8 (-90.2 to -10.4)	-52.0 (-57.4 to -46.4)	-41.9 (-64.2 to -15.0)	-45.3 (-61.1 to -30.7)	-45.8 (-53.2 to -37.8)	-24.2 (-59.0 to 23.9)	-30.5 (-42.9 to -16.6)	-19.4 (-41.3 to 6.4)
Ukraine	YLDs Rate: 1990	3,316.4 (2,415.7–4,402.6)	816.3 (438.7–1,311.3)	531.5 (365.8–724.5)	190.3 (68.8–382.0)	154.8 (110.4–209.1)	67.5 (37.2–108.7)	95.1 (58.3–141.4)	119.1 (90.6–155.0)	0.0 (0.0–0.0)	56.7 (37.9–78.9)	31.8 (22.5–43.1)
Ukraine	YLDs Rate: 2015	2,752.8 (2,031.1–3,573.3)	644.4 (378.5–966.3)	531.0 (361.4–730.4)	77.2 (27.4–163.3)	102.3 (71.9–138.6)	51.5 (30.4–77.1)	72.4 (44.2–111.5)	89.4 (64.9–117.8)	0.0 (0.0–0.0)	54.3 (36.2–75.6)	35.1 (23.6–48.6)
Ukraine	YLDs Rate: Cumulative change (%)	-16.6 (-27.6 to -5.1)	-17.1 (-51.5 to 20.4)	-0.1 (-4.3 to 4.4)	-49.8 (-86.4 to 24.2)	-33.8 (-41.2 to -26.0)	-20.1 (-50.5 to 15.0)	-23.1 (-45.3 to -2.6)	-24.8 (-35.2 to -13.8)	5.7 (-42.6 to 73.1)	-3.6 (-20.7 to 15.5)	11.6 (-18.7 to 47.2)
Central Europe	Prevalence Number: 1990	7,343,538 (7,241,980–7,441,732)	2,827,315 (2,488,381–3,195,699)	1,967,608 (1,912,146–2,024,330)	171,537 (154,619–189,988)	45,341 (41,395–50,138)	861,225 (805,441–931,878)	163,311 (122,236–205,342)	161,025 (147,992–175,097)	0 (0–0)	23,944 (19,059–29,780)	-
Central Europe	Prevalence Number: 2015	4,619,604 (4,556,693–4,683,749)	1,671,226 (1,494,377–1,857,718)	1,267,427 (1,230,336–1,304,204)	86,639 (77,113–97,039)	17,216 (15,800–18,764)	469,172 (437,127–510,507)	88,039 (62,992–115,963)	90,678 (83,492–98,654)	0 (0–0)	14,558 (11,732–17,779)	-
Central Europe	Prevalence Number: Cumulative change (%	-37.1 (-38.2 to -36.0)	-40.6 (-49.9 to -31.3)	-35.6 (-37.7 to -33.4)	-49.3 (-56.5 to -41.1)	-62.0 (-64.3 to -59.9)	-45.4 (-50.3 to -40.2)	-46.1 (-54.3 to -38.8)	-43.7 (-46.1 to -41.4)	-36.1 (-36.3 to -36.0)	-39.1 (-41.7 to -36.7)	-
Central Europe	Prevalence Rate: 1990	80,185.9 (79,082.7–81,244.8)	31,184.1 (27,495.1–35,199.3)	21,402.6 (20,799.0–22,015.0)	1,883.6 (1,697.6–2,086.3)	501.2 (458.0–553.5)	9,441.7 (8,831.6–10,215.9)	1,760.8 (1,317.9–2,214.0)	1,832.6 (1,684.5–1,992.5)	0.0 (0.0–0.0)	265.8 (212.1–329.8)	-
Central Europe	Prevalence Rate: 2015	78,773.9 (77,694.4–79,862.3)	28,695.8 (25,704.5–31,871.0)	21,566.6 (20,932.5–22,190.5)	1,482.0 (1,319.0–1,659.8)	295.3 (271.1–321.8)	8,020.0 (7,472.4–8,726.1)	1,490.8 (1,066.7–1,963.6)	1,584.0 (1,458.4–1,723.7)	0.0 (0.0–0.0)	250.6 (202.1–305.7)	-
Central Europe	Prevalence Rate: Cumulative change (%	-1.8 (-3.5 to -0.0)	-7.5 (-21.8 to 6.8)	0.8 (-2.6 to 4.2)	-21.0 (-32.2 to -8.3)	-41.0 (-44.6 to -37.9)	-14.9 (-22.5 to -6.8)	-15.4 (-28.3 to -3.9)	-13.5 (-17.1 to -10.2)	-0.0 (-0.0 to 0.0)	-5.6 (-9.5 to -1.8)	-
Central Europe	YLDs Number: 1990	320,468 (236,303–419,763)	83,676 (54,798–121,714)	51,250 (35,496–69,103)	21,511 (14,776–29,070)	7,479 (5,383–10,021)	5,100 (3,403–7,318)	7,495 (4,726–10,824)	10,581 (8,058–13,587)	0 (0–0)	4,775 (3,320–6,490)	4,449 (3,264–5,794)
Central Europe	YLDs Number: 2015	171,744 (128,426–223,455)	48,089 (32,604–67,524)	32,981 (22,816–44,559)	10,899 (7,267–14,904)	2,844 (2,018–3,796)	2,880 (1,942–4,017)	4,051 (2,494–6,010)	4,255 (3,230–5,509)	0 (0–0)	2,769 (1,936–3,781)	2,493 (1,832–3,228)
Central Europe	YLDs Number: Cumulative change (%)	-46.3 (-51.0 to -41.8)	-41.7 (-56.1 to -27.2)	-35.6 (-36.7 to -34.6)	-49.2 (-56.5 to -41.1)	-61.9 (-64.6 to -59.2)	-42.9 (-55.4 to -31.4)	-46.0 (-54.6 to -37.9)	-59.8 (-63.1 to -56.7)	-35.3 (-49.0 to -19.3)	-41.9 (-47.2 to -36.5)	-43.5 (-54.4 to -30.9)
Central Europe	YLDs Rate: 1990	3,514.2 (2,590.6–4,603.0)	926.4 (608.5–1,343.3)	556.0 (385.1–750.0)	236.2 (162.3–319.2)	82.7 (59.5–110.8)	56.5 (37.8–81.0)	80.8 (51.0–116.7)	116.0 (88.3–149.1)	0.0 (0.0–0.0)	52.3 (36.4–71.1)	48.8 (35.8–63.6)
Central Europe	YLDs Rate: 2015	2,936.8 (2,195.9–3,823.1)	828.2 (561.9–1,161.4)	560.4 (387.6–757.3)	186.4 (124.3–255.0)	48.8 (34.7–65.0)	49.6 (33.4–69.1)	68.6 (42.2–101.8)	72.7 (55.2–94.1)	0.0 (0.0–0.0)	47.3 (33.1–64.6)	42.6 (31.3–55.3)
Central Europe	YLDs Rate: Cumulative change (%)	-16.3 (-23.5 to -9.3)	-9.3 (-31.6 to 13.0)	0.8 (-0.9 to 2.5)	-20.8 (-32.3 to -8.2)	-41.0 (-45.0 to -36.7)	-11.2 (-30.4 to 6.5)	-15.1 (-28.8 to -2.4)	-37.3 (-42.4 to -32.5)	1.3 (-20.1 to 26.2)	-9.4 (-17.6 to -1.1)	-11.9 (-28.9 to 7.5)
Albania	Prevalence Number: 1990	326,400 (318,846–333,617)	170,298 (147,289–208,315)	76,578 (71,635–81,610)	18,703 (14,230–23,722)	3,396 (2,907–4,129)	47,141 (41,247–54,344)	8,716 (6,709–10,990)	8,860 (7,728–10,205)	0 (0–0)	1,015 (807–1,265)	-
Albania	Prevalence Number: 2015	143,919 (139,628–148,029)	40,287 (35,029–46,903)	36,848 (34,600–39,087)	5,647 (4,361–7,262)	811 (704–914)	18,715 (16,380–22,427)	3,010 (2,260–3,910)	3,882 (3,339–4,481)	0 (0–0)	476 (386–586)	-
Albania	Prevalence Number: Cumulative change (%	-55.9 (-57.4 to -54.3)	-76.2 (-81.3 to -72.6)	-51.8 (-55.8 to -47.6)	-69.0 (-78.9 to -56.5)	-75.9 (-79.7 to -72.3)	-60.0 (-67.3 to -51.3)	-65.5 (-70.4 to -60.6)	-56.1 (-60.1 to -51.6)	-52.3 (-52.5 to -52.2)	-52.9 (-56.5 to -48.6)	-
Albania	Prevalence Rate: 1990	84,555.7 (82,602.2–86,424.6)	44,131.0 (38,178.8–53,975.0)	19,832.9 (18,552.9–21,135.9)	4,846.4 (3,687.3–6,147.0)	880.2 (753.6–1,070.0)	12,213.7 (10,686.6–14,080.5)	2,256.4 (1,736.8–2,845.3)	2,300.6 (2,006.8–2,649.9)	0.0 (0.0–0.0)	263.3 (209.3–328.1)	-
Albania	Prevalence Rate: 2015	77,887.1 (75,556.9–80,105.2)	21,588.1 (18,752.7–25,143.6)	19,981.4 (18,756.8–21,195.6)	3,046.9 (2,353.3–3,918.2)	436.8 (378.6–492.3)	10,104.1 (8,844.0–12,106.3)	1,641.7 (1,232.8–2,132.7)	2,042.5 (1,756.7–2,358.3)	0.0 (0.0–0.0)	255.4 (206.9–313.9)	-
Albania	Prevalence Rate: Cumulative change (%	-7.9 (-11.0 to -4.6)	-50.7 (-61.4 to -43.3)	0.9 (-7.5 to 9.6)	-35.5 (-56.1 to -9.4)	-50.0 (-57.8 to -42.6)	-16.7 (-31.8 to 1.6)	-27.2 (-37.7 to -16.9)	-11.1 (-19.2 to -1.9)	-0.0 (-0.0 to 0.0)	-2.8 (-10.0 to 6.1)	-
Albania	YLDs Number: 1990	17,906 (13,132–23,328)	5,834 (3,754–8,572)	1,766 (1,193–2,414)	2,334 (1,463–3,371)	555 (388–765)	496 (322–719)	397 (255–576)	379 (287–487)	0 (0–0)	196 (134–270)	367 (254–520)
Albania	YLDs Number: 2015	5,456 (4,067–7,086)	1,062 (680–1,585)	851 (574–1,164)	710 (441–1,043)	134 (93–182)	137 (88–203)	138 (86–204)	134 (99–175)	0 (0–0)	89 (61–121)	338 (210–500)
Albania	YLDs Number: Cumulative change (%)	-69.4 (-73.4 to -66.5)	-81.6 (-87.0 to -76.1)	-51.8 (-53.6 to -50.0)	-68.8 (-78.6 to -55.9)	-75.7 (-79.9 to -71.4)	-71.9 (-79.9 to -63.8)	-65.0 (-70.7 to -59.1)	-64.6 (-69.6 to -59.3)	-61.9 (-71.7 to -53.5)	-54.1 (-62.1 to -45.2)	-5.2 (-42.9 to 43.3)
Albania	YLDs Rate: 1990	4,639.9 (3,402.7–6,045.4)	1,512.1 (973.0–2,221.1)	457.6 (309.1–625.3)	605.0 (379.2–873.7)	143.9 (100.7–198.3)	128.8 (83.5–186.6)	103.0 (66.1–149.2)	98.3 (74.5–126.2)	0.0 (0.0–0.0)	50.9 (34.7–70.2)	95.1 (66.0–134.8)
Albania	YLDs Rate: 2015	2,943.0 (2,195.8–3,818.8)	567.1 (364.2–844.9)	462.3 (311.7–631.8)	383.3 (238.0–563.2)	72.1 (50.6–98.3)	73.7 (47.4–108.7)	75.7 (47.3–111.3)	72.4 (54.0–94.6)	0.0 (0.0–0.0)	48.3 (33.3–65.7)	182.5 (113.8–270.3)
Albania	YLDs Rate: Cumulative change (%)	-36.3 (-44.5 to -30.1)	-62.0 (-73.1 to -50.7)	1.0 (-2.7 to 4.8)	-35.0 (-55.5 to -8.2)	-49.5 (-58.3 to -40.6)	-42.1 (-58.6 to -25.5)	-26.4 (-38.2 to -13.8)	-26.1 (-36.8 to -15.2)	-20.1 (-40.9 to -2.7)	-4.3 (-21.1 to 14.5)	97.4 (18.7–198.4)
Bosnia and Herzegovina	Prevalence Number: 1990	285,211 (275,781–294,065)	104,653 (84,017–133,346)	74,774 (70,004–79,519)	12,587 (9,662–16,124)	2,626 (2,352–2,937)	32,533 (27,405–39,123)	3,977 (2,895–5,208)	9,334 (8,342–10,481)	0 (0–0)	1,009 (805–1,266)	-
Bosnia and Herzegovina	Prevalence Number: 2015	136,971 (132,220–141,445)	50,393 (38,554–63,602)	37,034 (34,700–39,207)	2,456 (1,845–3,145)	843 (743–955)	13,684 (11,430–16,655)	2,242 (1,572–3,042)	3,429 (3,038–3,848)	0 (0–0)	448 (363–550)	-
Bosnia and Herzegovina	Prevalence Number: Cumulative change (%	-52.0 (-54.0 to -49.8)	-51.1 (-64.7 to -35.4)	-50.4 (-54.1 to -46.4)	-80.0 (-86.6 to -72.0)	-67.9 (-70.7 to -65.4)	-57.5 (-67.1 to -46.5)	-43.5 (-52.2 to -31.8)	-63.2 (-66.5 to -59.6)	-51.4 (-51.5 to -51.2)	-55.4 (-59.4 to -51.8)	-
Bosnia and Herzegovina	Prevalence Rate: 1990	80,668.6 (78,060.8–83,142.8)	30,111.6 (24,287.5–38,082.1)	21,007.1 (19,667.6–22,337.5)	3,584.8 (2,752.0–4,592.3)	756.6 (677.6–845.8)	9,249.2 (7,798.9–11,111.3)	1,103.4 (803.2–1,445.1)	2,811.6 (2,509.5–3,151.6)	0.0 (0.0–0.0)	291.9 (233.8–365.6)	-

© 2017 Global Burden of Disease Child and Adolescent Health Collaboration. JAMA Pediatrics.

eTable 4. Prevalent cases, Rates (per 100,000 population), Years Lived with Disability (YLDs), and Cumulative Percent Change with 95% Uncertainty Interval (UI) for the Top 10 Global Causes of YLDsin Children and Adolescents in 195 Countries and Territories, Aged Under 5 Years, Both Sexes, 1990 and 2015. *Note: best viewed by enlarging in browser.*

Location	Measure	All causes	Iron-deficiency anemia	Skin and subcutaneous diseases	Protein-energy malnutrition	Diarrheal diseases	Hemoglobinopathies and hemolytic anemias	Asthma	Neonatal preterm birth complications	Malaria	Neonatal encephalopathy due to birth asphyxia and trauma	Other neonatal disorders
Bosnia and Herzegovina	Prevalence Rate: 2015	79,163.6 (76,432.3–81,748.0)	29,356.8 (22,512.7–36,961.8)	21,348.5 (20,003.3–22,598.0)	1,424.5 (1,070.0–1,823.9)	490.8 (433.2–555.7)	7,929.8 (6,624.9–9,648.6)	1,285.6 (901.3–1,744.6)	2,037.1 (1,805.1–2,283.8)	0.0 (0.0–0.0)	261.9 (212.1–320.8)	-
Bosnia and Herzegovina	Prevalence Rate: Cumulative change (%	-1.8 (-6.0 to 2.5)	-1.2 (-28.1 to 30.0)	1.7 (-5.9 to 9.9)	-59.2 (-72.8 to -43.0)	-35.1 (-40.8 to -30.1)	-13.4 (-32.9 to 9.0)	16.8 (-1.1 to 41.0)	-27.4 (-33.9 to -20.6)	0.0 (-0.0 to 0.0)	-10.0 (-17.9 to -2.8)	-
Bosnia and Herzegovina	YLDs Number: 1990	15,707 (11,370–20,774)	2,981 (1,818–4,857)	1,978 (1,364–2,685)	1,576 (1,000–2,251)	432 (308–579)	177 (108–288)	182 (113–266)	565 (429–732)	0 (0–0)	202 (139–277)	83 (28–187)
Bosnia and Herzegovina	YLDs Number: 2015	5,165 (3,744–6,779)	1,421 (833–2,310)	971 (673–1,315)	308 (198–449)	139 (97–186)	86 (49–139)	103 (62–155)	156 (116–203)	0 (0–0)	86 (59–119)	34 (13–69)
Bosnia and Herzegovina	YLDs Number: Cumulative change (%)	-66.9 (-72.7 to -60.2)	-50.8 (-71.6 to -23.8)	-50.9 (-52.6 to -49.0)	-80.0 (-86.6 to -71.8)	-67.7 (-71.6 to -63.6)	-49.8 (-71.1 to -21.3)	-43.1 (-53.7 to -29.2)	-72.4 (-75.9 to -68.6)	-47.3 (-67.7 to -10.5)	-56.8 (-64.4 to -48.1)	-43.7 (-87.2 to 34.7)
Bosnia and Herzegovina	YLDs Rate: 1990	4,461.7 (3,232.8–5,898.0)	864.2 (532.0–1,395.2)	553.8 (382.0–751.8)	448.8 (284.8–641.3)	124.6 (89.1–166.4)	51.6 (31.7–82.6)	50.5 (31.5–73.9)	160.7 (121.7–208.2)	0.0 (0.0–0.0)	57.6 (39.5–78.8)	23.9 (8.1–53.3)
Bosnia and Herzegovina	YLDs Rate: 2015	2,994.9 (2,170.2–3,930.0)	830.7 (487.8–1,346.9)	559.1 (387.6–756.4)	178.7 (115.3–260.6)	81.2 (57.0–108.8)	50.5 (29.1–81.0)	59.2 (35.9–89.0)	90.4 (67.3–118.0)	0.0 (0.0–0.0)	50.3 (34.3–69.4)	19.8 (7.8–40.0)
Bosnia and Herzegovina	YLDs Rate: Cumulative change (%)	-32.5 (-44.3 to -19.0)	-0.9 (-41.8 to 52.3)	1.0 (-2.5 to 4.8)	-59.2 (-72.7 to -42.5)	-34.8 (-42.7 to -26.6)	0.8 (-41.2 to 57.2)	17.7 (-4.4 to 46.4)	-43.7 (-51.0 to -36.1)	8.2 (-33.6 to 83.1)	-11.9 (-27.4 to 5.6)	14.7 (-74.0 to 174.5)
Bulgaria	Prevalence Number: 1990	450,443 (438,160–461,685)	126,630 (95,226–158,655)	120,883 (113,433–127,991)	8,448 (6,248–11,041)	2,936 (2,680–3,239)	104,697 (98,759–110,702)	6,290 (4,676–8,230)	9,865 (8,779–11,085)	0 (0–0)	1,472 (1,167–1,819)	-
Bulgaria	Prevalence Number: 2015	274,181 (265,506–282,209)	86,475 (66,715–105,654)	74,625 (70,232–78,676)	4,564 (3,305–6,102)	1,426 (1,279–1,576)	53,884 (51,254–56,548)	5,297 (3,729–7,235)	5,927 (5,259–6,695)	0 (0–0)	878 (703–1,084)	-
Bulgaria	Prevalence Number: Cumulative change (%	-39.1 (-41.4 to -36.9)	-29.8 (-53.4 to -3.1)	-38.2 (-42.6 to -33.4)	-44.3 (-64.1 to -17.8)	-51.4 (-54.6 to -48.5)	-48.5 (-52.2 to -44.7)	-15.4 (-32.7 to 4.0)	-39.8 (-45.3 to -33.9)	-38.8 (-39.0 to -38.7)	-40.2 (-43.9 to -36.2)	-
Bulgaria	Prevalence Rate: 1990	79,738.7 (77,556.9–81,724.5)	22,795.3 (17,273.0–28,398.7)	21,294.2 (19,995.9–22,550.0)	1,504.7 (1,112.8–1,966.5)	526.1 (480.8–579.6)	18,607.1 (17,547.7–19,674.0)	1,096.8 (815.3–1,434.8)	1,834.7 (1,635.0–2,059.3)	0.0 (0.0–0.0)	265.7 (211.0–328.5)	-
Bulgaria	Prevalence Rate: 2015	78,998.0 (76,515.9–81,304.2)	25,090.1 (19,373.8–30,539.1)	21,454.2 (20,192.6–22,617.4)	1,319.9 (955.6–1,764.6)	414.0 (371.4–457.3)	15,562.2 (14,803.8–16,331.4)	1,514.9 (1,066.5–2,069.0)	1,754.1 (1,556.9–1,979.0)	0.0 (0.0–0.0)	255.9 (204.8–316.2)	-
Bulgaria	Prevalence Rate: Cumulative change (%	-0.9 (-4.5 to 2.7)	13.0 (-24.3 to 54.4)	0.9 (-6.4 to 8.7)	-9.5 (-41.7 to 33.4)	-21.3 (-26.4 to -16.6)	-16.3 (-22.3 to -10.2)	38.7 (10.3–70.5)	-4.2 (-12.8 to 4.9)	-0.0 (-0.0 to 0.0)	-3.6 (-9.7 to 3.0)	-
Bulgaria	YLDs Number: 1990	17,240 (12,798–22,408)	3,291 (1,936–5,048)	3,137 (2,158–4,217)	1,065 (642–1,569)	487 (348–651)	261 (155–400)	289 (182–426)	616 (467–799)	0 (0–0)	295 (198–401)	611 (422–830)
Bulgaria	YLDs Number: 2015	9,751 (7,123–12,876)	2,290 (1,288–3,563)	1,923 (1,327–2,606)	574 (355–854)	236 (167–315)	164 (92–255)	243 (145–373)	290 (212–378)	0 (0–0)	167 (113–232)	93 (63–129)
Bulgaria	YLDs Number: Cumulative change (%)	-43.3 (-52.7 to -36.4)	-27.3 (-66.6 to 4.0)	-38.6 (-41.1 to -36.3)	-44.3 (-64.3 to -18.0)	-51.4 (-57.1 to -45.4)	-34.1 (-70.3 to -6.2)	-15.4 (-34.3 to 6.3)	-52.9 (-58.4 to -47.0)	-27.7 (-68.1 to 16.8)	-43.1 (-53.4 to -31.1)	-84.3 (-89.1 to -77.8)
Bulgaria	YLDs Rate: 1990	3,068.4 (2,276.3–3,991.3)	596.0 (356.0–913.9)	551.0 (379.0–740.2)	189.8 (113.4–279.3)	87.3 (62.4–116.7)	47.4 (28.4–72.6)	50.5 (31.8–74.3)	109.6 (83.1–142.0)	0.0 (0.0–0.0)	52.5 (35.3–71.5)	108.8 (75.3–147.8)
Bulgaria	YLDs Rate: 2015	2,816.6 (2,059.4–3,719.7)	666.3 (377.7–1,039.2)	552.2 (381.0–748.6)	166.1 (102.9–246.9)	68.6 (48.6–91.6)	48.0 (27.2–74.6)	69.8 (41.5–106.9)	83.8 (61.5–109.4)	0.0 (0.0–0.0)	48.3 (32.8–66.9)	27.1 (18.5–37.5)
Bulgaria	YLDs Rate: Cumulative change (%)	-8.0 (-23.1 to 3.3)	16.6 (-45.4 to 65.4)	0.3 (-3.6 to 4.2)	-9.6 (-42.1 to 33.2)	-21.3 (-30.4 to -11.6)	5.5 (-51.5 to 49.0)	38.8 (7.7–74.3)	-23.4 (-32.3 to -13.9)	17.9 (-47.7 to 89.6)	-7.5 (-24.1 to 11.7)	-74.6 (-82.3 to -64.0)
Croatia	Prevalence Number: 1990	229,390 (222,497–235,965)	83,555 (62,591–100,760)	63,114 (59,369–66,817)	7,003 (5,223–9,016)	1,106 (1,008–1,220)	19,493 (17,958–21,213)	4,091 (2,957–5,335)	4,457 (3,992–4,958)	0 (0–0)	752 (600–943)	-
Croatia	Prevalence Number: 2015	162,847 (157,766–168,110)	60,846 (43,565–83,147)	45,082 (42,295–47,865)	3,578 (2,599–4,793)	499 (460–544)	11,964 (10,951–13,097)	3,269 (2,309–4,416)	3,051 (2,727–3,405)	0 (0–0)	526 (419–649)	-
Croatia	Prevalence Number: Cumulative change (%	-29.0 (-31.8 to -26.0)	-26.5 (-46.0 to -0.6)	-28.5 (-33.9 to -22.4)	-47.5 (-65.4 to -23.6)	-54.8 (-58.5 to -50.9)	-38.5 (-45.5 to -30.8)	-19.9 (-32.6 to -5.0)	-31.4 (-37.6 to -25.2)	-28.8 (-28.9 to -28.6)	-29.9 (-35.2 to -24.6)	-
Croatia	Prevalence Rate: 1990	78,648.4 (76,246.3–80,885.5)	29,108.3 (21,929.2–34,861.5)	21,537.6 (20,259.1–22,805.8)	2,416.8 (1,802.7–3,111.6)	383.4 (349.7–422.5)	6,721.7 (6,193.1–7,308.0)	1,381.2 (998.6–1,801.4)	1,602.6 (1,437.8–1,782.5)	0.0 (0.0–0.0)	262.9 (210.4–329.7)	-
Croatia	Prevalence Rate: 2015	78,125.3 (75,683.7–80,609.9)	29,461.5 (21,224.0–40,138.5)	21,571.6 (20,241.3–22,899.6)	1,723.4 (1,251.7–2,308.3)	241.1 (222.4–263.4)	5,760.0 (5,273.7–6,303.0)	1,554.9 (1,098.4–2,100.6)	1,507.0 (1,347.3–1,680.4)	0.0 (0.0–0.0)	255.3 (203.5–314.3)	-
Croatia	Prevalence Rate: Cumulative change (%	-0.6 (-4.5 to 3.5)	2.2 (-24.6 to 37.7)	0.3 (-7.3 to 8.8)	-26.7 (-51.7 to 6.6)	-37.0 (-42.1 to -31.6)	-14.1 (-23.9 to -3.4)	12.8 (-5.0 to 33.9)	-5.8 (-14.1 to 2.7)	0.0 (-0.0 to 0.0)	-2.7 (-10.0 to 4.7)	-
Croatia	YLDs Number: 1990	9,866 (7,134–13,011)	2,358 (1,250–3,595)	1,639 (1,128–2,220)	880 (550–1,296)	182 (127–247)	143 (76–212)	188 (116–282)	302 (224–398)	0 (0–0)	149 (101–212)	181 (127–248)
Croatia	YLDs Number: 2015	6,737 (4,819–8,938)	1,765 (944–2,929)	1,171 (814–1,583)	449 (278–658)	82 (58–111)	107 (58–176)	150 (90–225)	149 (109–198)	0 (0–0)	100 (66–139)	105 (72–147)
Croatia	YLDs Number: Cumulative change (%)	-31.6 (-40.8 to -20.0)	-23.2 (-48.7 to 15.6)	-28.5 (-31.8 to -25.4)	-47.4 (-66.0 to -23.5)	-54.6 (-61.8 to -46.6)	-23.0 (-48.5 to 15.7)	-19.7 (-35.1 to -1.7)	-50.3 (-57.8 to -41.6)	-25.8 (-50.6 to 6.2)	-32.4 (-46.4 to -17.5)	-40.8 (-58.7 to -19.4)
Croatia	YLDs Rate: 1990	3,404.0 (2,456.8–4,501.0)	827.4 (439.9–1,261.4)	557.7 (383.6–755.1)	303.9 (189.9–447.3)	63.3 (44.4–85.5)	50.4 (27.4–74.7)	63.6 (39.5–95.3)	104.1 (77.1–137.3)	0.0 (0.0–0.0)	51.5 (35.0–73.2)	62.7 (44.0–85.6)
Croatia	YLDs Rate: 2015	3,244.2 (2,320.7–4,300.9)	858.2 (463.0–1,413.8)	559.3 (388.8–756.7)	216.6 (134.3–316.9)	39.9 (28.1–53.5)	52.3 (28.8–85.5)	71.6 (43.0–107.3)	72.0 (52.9–95.5)	0.0 (0.0–0.0)	48.2 (32.2–67.2)	50.7 (34.9–70.8)
Croatia	YLDs Rate: Cumulative change (%)	-4.5 (-17.4 to 11.7)	6.4 (-28.8 to 59.4)	0.3 (-4.2 to 4.8)	-26.6 (-52.5 to 6.7)	-36.7 (-46.6 to -25.9)	6.6 (-28.4 to 59.7)	13.2 (-8.5 to 38.5)	-30.5 (-41.0 to -18.6)	4.0 (-30.5 to 49.0)	-5.6 (-25.1 to 15.0)	-17.4 (-42.4 to 12.3)
Czech Republic	Prevalence Number: 1990	506,146 (490,670–521,578)	180,915 (129,931–222,414)	140,199 (131,513–148,340)	8,453 (6,901–10,081)	1,637 (1,500–1,762)	57,753 (48,537–69,573)	8,278 (5,964–11,047)	8,807 (8,027–9,642)	0 (0–0)	1,589 (1,279–1,964)	-
Czech Republic	Prevalence Number: 2015	425,591 (411,063–438,072)	160,641 (132,948–187,461)	118,893 (111,613–126,066)	8,325 (6,076–11,072)	1,259 (1,159–1,366)	42,391 (35,292–52,164)	5,507 (3,805–7,400)	7,168 (6,522–7,847)	0 (0–0)	1,313 (1,020–1,630)	-
Czech Republic	Prevalence Number: Cumulative change (%	-15.9 (-19.1 to -12.6)	-7.8 (-34.0 to 33.0)	-15.1 (-21.9 to -8.3)	-0.2 (-30.8 to 40.0)	-23.0 (-26.7 to -19.1)	-25.8 (-43.0 to -6.4)	-33.3 (-44.4 to -21.7)	-18.5 (-25.9 to -10.5)	-15.5 (-15.6 to -15.5)	-17.3 (-23.8 to -11.0)	-
Czech Republic	Prevalence Rate: 1990	78,428.4 (76,058.0–80,812.2)	28,221.8 (20,374.5–34,697.1)	21,682.2 (20,341.1–22,940.7)	1,313.7 (1,072.4–1,566.7)	254.7 (233.5–274.1)	8,969.2 (7,539.7–10,801.6)	1,274.4 (918.2–1,700.8)	1,393.6 (1,271.0–1,524.7)	0.0 (0.0–0.0)	248.3 (200.2–306.5)	-
Czech Republic	Prevalence Rate: 2015	78,064.8 (75,405.2–80,350.0)	29,627.4 (24,549.4–34,580.9)	21,771.4 (20,437.2–23,087.1)	1,531.4 (1,117.7–2,036.7)	232.1 (213.7–251.7)	7,793.2 (6,489.2–9,588.3)	1,004.1 (693.9–1,349.3)	1,342.5 (1,223.1–1,468.8)	0.0 (0.0–0.0)	242.9 (188.7–301.4)	-
Czech Republic	Prevalence Rate: Cumulative change (%	-0.4 (-4.3 to 3.5)	8.8 (-21.9 to 55.6)	0.5 (-7.5 to 8.6)	18.1 (-18.1 to 65.7)	-8.8 (-13.1 to -4.2)	-12.2 (-32.5 to 10.8)	-21.0 (-34.2 to -7.3)	-3.5 (-12.2 to 5.7)	0.0 (-0.0 to 0.0)	-2.2 (-9.9 to 5.4)	-
Czech Republic	YLDs Number: 1990	19,791 (14,346–25,926)	5,131 (2,951–7,679)	3,639 (2,505–4,922)	1,064 (698–1,494)	270 (191–367)	319 (184–478)	380 (233–570)	536 (399–698)	0 (0–0)	323 (220–450)	67 (47–91)
Czech Republic	YLDs Number: 2015	15,249 (11,340–20,232)	4,543 (2,786–6,795)	3,078 (2,126–4,152)	1,046 (632–1,533)	208 (145–278)	271 (166–403)	253 (148–384)	322 (239–425)	0 (0–0)	257 (174–360)	241 (168–336)
Czech Republic	YLDs Number: Cumulative change (%)	-22.5 (-34.1 to -8.0)	-6.3 (-43.4 to 63.1)	-15.4 (-18.3 to -12.1)	-0.2 (-31.7 to 41.8)	-22.6 (-33.9 to -10.3)	-10.4 (-46.0 to 56.1)	-33.3 (-45.7 to -19.8)	-39.6 (-49.8 to -29.0)	1.6 (-52.5 to 123.9)	-19.9 (-33.0 to -4.1)	261.8 (153.2–392.9)
Czech Republic	YLDs Rate: 1990	3,074.6 (2,232.4–4,028.0)	802.6 (466.1–1,198.8)	562.0 (387.0–759.8)	165.4 (108.6–232.3)	42.0 (29.8–57.1)	50.0 (29.1–74.6)	58.7 (36.0–87.9)	83.3 (62.0–108.5)	0.0 (0.0–0.0)	50.2 (34.3–69.9)	10.5 (7.4–14.2)
Czech Republic	YLDs Rate: 2015	2,803.6 (2,082.7–3,719.5)	840.0 (515.2–1,256.8)	563.0 (388.8–759.2)	192.5 (116.4–282.1)	38.3 (26.8–51.3)	50.1 (30.9–74.8)	46.2 (27.1–70.0)	59.3 (44.0–78.2)	0.0 (0.0–0.0)	47.3 (32.1–66.2)	44.4 (31.0–61.9)
Czech Republic	YLDs Rate: Cumulative change (%)	-8.3 (-22.1 to 8.6)	10.5 (-33.3 to 89.2)	0.2 (-3.3 to 4.1)	18.2 (-19.1 to 67.8)	-8.4 (-21.6 to 6.1)	5.7 (-36.2 to 81.2)	-21.0 (-35.7 to -5.0)	-28.5 (-40.5 to -15.9)	20.1 (-43.5 to 163.1)	-5.2 (-20.8 to 13.4)	328.1 (199.8–482.6)

© 2017 Global Burden of Disease Child and Adolescent Health Collaboration. JAMA Pediatrics.

eTable 4. Prevalent cases, Rates (per 100,000 population), Years Lived with Disability (YLDs), and Cumulative Percent Change with 95% Uncertainty Interval (UI) for the Top 10 Global Causes of YLDsin Children and Adolescents in 195 Countries and Territories, Aged Under 5 Years, Both Sexes, 1990 and 2015. *Note: best viewed by enlarging in browser.*

Location	Measure	All causes	Iron-deficiency anemia	Skin and subcutaneous diseases	Protein-energy malnutrition	Diarrheal diseases	Hemoglobinopathies and hemolytic anemias	Asthma	Neonatal preterm birth complications	Malaria	Neonatal encephalopathy due to birth asphyxia and trauma	Other neonatal disorders
Hungary	Prevalence Number: 1990	491,088 (476,404–505,808)	173,858 (141,618–233,143)	143,277 (135,606–150,797)	10,844 (8,250–13,870)	1,542 (1,411–1,674)	57,260 (48,415–71,654)	9,461 (7,078–12,274)	10,207 (9,261–11,204)	0 (0–0)	1,618 (1,283–2,040)	-
Hungary	Prevalence Number: 2015	361,425 (349,711–373,381)	129,327 (95,967–164,182)	106,875 (101,014–112,169)	7,379 (5,432–9,865)	1,121 (994–1,268)	36,140 (30,364–43,875)	5,691 (4,137–7,496)	7,214 (6,625–7,822)	0 (0–0)	1,134 (907–1,408)	-
Hungary	Prevalence Number: Cumulative change (%	-26.4 (-29.4 to -23.2)	-23.9 (-50.1 to 0.6)	-25.3 (-30.5 to -20.1)	-30.3 (-53.9 to -0.2)	-27.3 (-32.0 to -21.6)	-36.2 (-51.8 to -19.2)	-39.7 (-49.1 to -29.8)	-29.2 (-35.5 to -23.0)	-25.9 (-25.9 to -25.9)	-29.7 (-34.9 to -23.8)	-
Hungary	Prevalence Rate: 1990	78,144.1 (75,808.2–80,475.6)	27,786.1 (22,651.6–37,239.3)	22,764.7 (21,546.9–23,955.6)	1,728.8 (1,315.3–2,211.2)	246.3 (225.3–267.2)	9,124.5 (7,715.9–11,418.0)	1,499.0 (1,121.5–1,944.8)	1,645.0 (1,493.0–1,804.2)	0.0 (0.0–0.0)	258.9 (205.4–326.1)	-
Hungary	Prevalence Rate: 2015	77,636.1 (75,120.7–80,191.9)	27,908.4 (20,786.2–35,361.2)	22,920.1 (21,663.4–24,057.4)	1,588.9 (1,169.7–2,124.3)	241.9 (214.7–273.6)	7,777.5 (6,537.4–9,444.4)	1,216.4 (884.3–1,602.2)	1,575.7 (1,447.5–1,705.2)	0.0 (0.0–0.0)	245.4 (196.5–304.4)	-
Hungary	Prevalence Rate: Cumulative change (%	-0.6 (-4.8 to 3.7)	2.7 (-32.5 to 35.6)	0.8 (-6.1 to 7.8)	-5.9 (-37.7 to 34.8)	-1.8 (-8.2 to 6.0)	-13.8 (-34.9 to 9.1)	-18.6 (-31.3 to -5.3)	-4.0 (-12.6 to 4.2)	-0.0 (-0.0 to 0.0)	-4.9 (-12.1 to 3.1)	-
Hungary	YLDs Number: 1990	22,239 (16,412–29,239)	4,816 (2,947–8,001)	4,135 (2,894–5,563)	1,360 (865–1,961)	255 (179–340)	301 (185–500)	435 (272–630)	751 (571–959)	0 (0–0)	325 (221–458)	40 (29–54)
Hungary	YLDs Number: 2015	14,374 (10,452–18,769)	3,543 (2,065–5,395)	3,075 (2,156–4,137)	927 (568–1,383)	185 (129–254)	214 (124–328)	261 (159–387)	391 (290–518)	0 (0–0)	216 (147–301)	305 (206–421)
Hungary	YLDs Number: Cumulative change (%)	-35.0 (-48.0 to -23.3)	-22.3 (-62.0 to 14.0)	-25.6 (-27.6 to -23.8)	-30.3 (-53.9 to 1.0)	-27.3 (-36.2 to -17.5)	-24.8 (-62.9 to 9.4)	-39.7 (-50.7 to -28.9)	-47.9 (-54.2 to -41.5)	-16.2 (-67.0 to 43.2)	-33.0 (-45.0 to -20.5)	662.4 (438.5–925.0)
Hungary	YLDs Rate: 1990	3,544.3 (2,615.1–4,659.6)	771.3 (471.9–1,282.2)	656.4 (459.4–883.2)	216.9 (138.0–312.6)	40.8 (28.8–54.4)	48.3 (29.7–80.1)	69.0 (43.2–99.9)	119.7 (90.9–152.8)	0.0 (0.0–0.0)	51.9 (35.2–73.0)	6.5 (4.6–8.6)
Hungary	YLDs Rate: 2015	3,092.6 (2,251.5–4,037.6)	766.0 (448.4–1,165.2)	658.6 (461.8–886.6)	199.7 (122.4–297.9)	40.1 (27.8–54.9)	46.4 (27.2–70.9)	55.9 (34.1–82.7)	84.3 (62.6–111.5)	0.0 (0.0–0.0)	46.7 (31.7–64.9)	65.7 (44.4–90.7)
Hungary	YLDs Rate: Cumulative change (%)	-12.2 (-29.8 to 3.4)	4.9 (-48.6 to 53.6)	0.3 (-2.2 to 2.8)	-5.8 (-37.8 to 36.4)	-1.7 (-13.8 to 11.4)	1.5 (-49.6 to 47.7)	-18.7 (-33.6 to -4.1)	-29.6 (-38.0 to -21.1)	13.1 (-55.3 to 93.2)	-9.5 (-25.6 to 7.4)	929.6 (627.4–1,284.2)
Macedonia	Prevalence Number: 1990	144,545 (140,314–147,913)	54,378 (44,793–63,807)	38,230 (35,811–40,471)	2,314 (1,698–3,048)	3,196 (2,654–3,816)	17,184 (14,512–21,160)	2,608 (1,912–3,481)	3,164 (2,813–3,585)	0 (0–0)	478 (373–599)	-
Macedonia	Prevalence Number: 2015	89,428 (86,538–92,086)	29,889 (24,354–35,979)	24,353 (22,868–25,854)	1,352 (1,004–1,754)	695 (618–781)	9,117 (7,656–10,930)	1,760 (1,230–2,411)	1,985 (1,748–2,227)	0 (0–0)	294 (237–362)	-
Macedonia	Prevalence Number: Cumulative change (%	-38.1 (-40.6 to -35.8)	-44.6 (-55.4 to -32.4)	-36.2 (-41.5 to -30.8)	-39.7 (-60.8 to -11.3)	-78.1 (-81.5 to -74.3)	-46.3 (-58.7 to -31.6)	-32.4 (-42.8 to -21.1)	-37.2 (-43.2 to -31.0)	-36.5 (-36.7 to -36.4)	-38.2 (-43.5 to -32.2)	-
Macedonia	Prevalence Rate: 1990	80,256.3 (77,925.9–82,121.9)	30,455.6 (25,248.3–35,579.1)	21,164.7 (19,828.3–22,398.4)	1,289.6 (946.6–1,698.5)	1,792.1 (1,492.0–2,137.9)	9,567.7 (8,080.0–11,779.2)	1,435.1 (1,051.9–1,915.1)	1,805.3 (1,605.0–2,045.0)	0.0 (0.0–0.0)	268.6 (209.0–336.7)	-
Macedonia	Prevalence Rate: 2015	77,887.1 (75,370.5–80,196.5)	26,055.0 (21,239.6–31,352.8)	21,204.8 (19,911.4–22,511.4)	1,178.2 (874.9–1,528.7)	606.3 (539.1–680.8)	7,943.1 (6,670.4–9,523.3)	1,531.8 (1,070.9–2,098.9)	1,734.2 (1,526.9–1,945.8)	0.0 (0.0–0.0)	257.0 (207.5–316.2)	-
Macedonia	Prevalence Rate: Cumulative change (%	-2.9 (-6.8 to 0.7)	-13.9 (-30.5 to 5.2)	0.3 (-8.1 to 8.8)	-5.7 (-38.7 to 38.7)	-65.9 (-71.3 to -60.0)	-15.9 (-35.4 to 7.0)	6.9 (-9.5 to 24.7)	-3.8 (-12.9 to 5.7)	0.0 (-0.0 to 0.0)	-3.9 (-12.2 to 5.3)	-
Macedonia	YLDs Number: 1990	6,179 (4,584–8,005)	1,607 (1,052–2,316)	983 (681–1,326)	289 (180–430)	527 (366–724)	102 (67–147)	119 (74–177)	252 (192–321)	0 (0–0)	96 (65–135)	132 (83–198)
Macedonia	YLDs Number: 2015	3,154 (2,323–4,183)	818 (514–1,240)	627 (435–844)	170 (102–255)	115 (81–155)	51 (31–76)	81 (49–122)	101 (75–133)	0 (0–0)	55 (37–79)	36 (24–53)
Macedonia	YLDs Number: Cumulative change (%)	-49.0 (-53.8 to -43.7)	-48.8 (-61.2 to -33.3)	-36.3 (-38.7 to -33.7)	-39.4 (-60.6 to -10.5)	-78.0 (-81.7 to -73.9)	-49.7 (-62.4 to -34.8)	-32.1 (-44.0 to -18.6)	-59.5 (-65.2 to -53.4)	-37.0 (-57.4 to -11.6)	-41.3 (-51.9 to -29.2)	-70.7 (-81.7 to -54.7)
Macedonia	YLDs Rate: 1990	3,445.3 (2,557.9–4,457.7)	904.1 (591.2–1,294.4)	543.7 (376.4–733.1)	161.5 (100.7–239.6)	295.9 (205.5–405.4)	57.6 (38.2–82.5)	65.8 (41.3–97.6)	140.4 (107.4–178.8)	0.0 (0.0–0.0)	53.5 (36.3–75.1)	73.7 (46.8–110.7)
Macedonia	YLDs Rate: 2015	2,748.6 (2,024.2–3,645.6)	714.1 (448.8–1,082.3)	545.9 (379.3–735.1)	148.5 (89.4–222.2)	100.3 (70.9–135.9)	44.7 (27.8–66.7)	70.6 (42.9–106.8)	88.8 (65.8–115.9)	0.0 (0.0–0.0)	48.7 (32.8–69.1)	32.2 (21.0–46.7)
Macedonia	YLDs Rate: Cumulative change (%)	-20.2 (-27.8 to -12.0)	-20.6 (-39.7 to 3.4)	0.4 (-3.5 to 4.4)	-5.2 (-38.3 to 39.9)	-65.8 (-71.6 to -59.5)	-22.0 (-41.6 to 1.1)	7.4 (-11.4 to 28.7)	-36.7 (-45.5 to -27.1)	-0.8 (-32.9 to 38.4)	-8.2 (-24.6 to 10.7)	-54.2 (-71.3 to -29.2)
Montenegro	Prevalence Number: 1990	40,933 (39,620–42,184)	14,033 (11,180–18,412)	11,166 (10,520–11,841)	1,279 (955–1,657)	271 (239–303)	4,789 (4,035–5,894)	619 (435–829)	961 (863–1,076)	0 (0–0)	135 (109–170)	-
Montenegro	Prevalence Number: 2015	29,097 (28,192–29,954)	10,083 (8,570–11,652)	7,963 (7,460–8,416)	490 (380–625)	117 (105–129)	2,946 (2,478–3,584)	505 (358–679)	654 (580–738)	0 (0–0)	94 (75–115)	-
Montenegro	Prevalence Number: Cumulative change (%	-28.9 (-31.9 to -25.9)	-26.5 (-47.9 to -8.3)	-28.6 (-34.3 to -22.9)	-60.5 (-73.2 to -44.9)	-56.5 (-59.9 to -53.3)	-37.8 (-52.4 to -19.8)	-18.2 (-28.9 to -6.5)	-31.8 (-38.0 to -25.1)	-28.3 (-28.4 to -28.3)	-30.6 (-36.0 to -25.6)	-
Montenegro	Prevalence Rate: 1990	79,001.9 (76,487.3–81,406.5)	27,284.2 (21,784.2–35,644.8)	21,502.1 (20,262.5–22,800.2)	2,477.6 (1,849.5–3,208.8)	527.8 (465.3–589.2)	9,267.7 (7,806.5–11,404.8)	1,186.6 (833.4–1,588.4)	1,901.3 (1,706.0–2,125.2)	0.0 (0.0–0.0)	264.8 (212.7–332.3)	-
Montenegro	Prevalence Rate: 2015	78,430.1 (76,007.4–80,738.3)	27,442.6 (23,384.1–31,689.5)	21,406.3 (20,055.5–22,621.4)	1,328.5 (1,029.1–1,693.2)	320.8 (288.0–353.1)	7,967.9 (6,702.4–9,689.6)	1,349.0 (957.1–1,815.8)	1,821.1 (1,612.2–2,047.6)	0.0 (0.0–0.0)	256.9 (207.3–315.0)	-
Montenegro	Prevalence Rate: Cumulative change (%	-0.7 (-4.8 to 3.5)	2.8 (-26.6 to 28.1)	-0.3 (-8.2 to 7.6)	-44.8 (-62.5 to -23.1)	-39.1 (-43.8 to -34.6)	-13.0 (-33.5 to 12.1)	14.1 (-0.8 to 30.4)	-4.1 (-12.4 to 5.1)	0.0 (-0.0 to 0.0)	-2.8 (-10.4 to 4.3)	-
Montenegro	YLDs Number: 1990	1,724 (1,256–2,274)	390 (234–626)	290 (200–392)	160 (98–231)	44 (31–60)	24 (14–38)	28 (16–43)	66 (50–85)	0 (0–0)	26 (18–36)	19 (6–41)
Montenegro	YLDs Number: 2015	1,060 (776–1,381)	276 (181–395)	208 (144–282)	61 (39–88)	19 (13–26)	16 (10–24)	23 (13–34)	31 (23–40)	0 (0–0)	18 (12–25)	8 (3–18)
Montenegro	YLDs Number: Cumulative change (%)	-38.3 (-47.7 to -31.1)	-26.1 (-58.6 to 1.4)	-28.3 (-31.1 to -25.5)	-60.5 (-73.2 to -45.0)	-56.4 (-62.5 to -49.7)	-28.6 (-58.9 to 0.6)	-17.7 (-30.3 to -2.8)	-53.0 (-59.3 to -45.7)	-22.3 (-49.4 to 15.6)	-32.1 (-44.5 to -18.9)	-40.5 (-85.8 to 58.5)
Montenegro	YLDs Rate: 1990	3,336.8 (2,430.6–4,408.0)	761.2 (456.2–1,215.0)	559.0 (386.0–755.2)	311.1 (191.4–447.6)	87.2 (61.6–117.4)	47.7 (28.2–75.5)	54.5 (31.9–83.1)	128.6 (98.5–165.3)	0.0 (0.0–0.0)	51.9 (35.6–71.0)	37.6 (13.0–80.1)
Montenegro	YLDs Rate: 2015	2,867.7 (2,100.4–3,733.5)	755.0 (496.2–1,080.8)	559.2 (387.5–758.4)	166.8 (105.9–239.0)	53.1 (37.3–70.9)	45.7 (29.8–66.4)	62.1 (37.0–92.6)	84.1 (62.3–109.6)	0.0 (0.0–0.0)	48.8 (33.8–67.6)	23.4 (9.1–49.7)
Montenegro	YLDs Rate: Cumulative change (%)	-13.8 (-26.8 to -3.8)	3.4 (-41.5 to 41.0)	0.1 (-3.9 to 3.9)	-44.9 (-62.6 to -23.1)	-38.9 (-47.4 to -29.6)	-0.1 (-42.0 to 39.8)	14.8 (-2.8 to 35.5)	-34.4 (-43.1 to -24.4)	8.5 (-29.3 to 61.6)	-5.2 (-22.4 to 13.1)	-16.8 (-80.2 to 121.6)
Poland	Prevalence Number: 1990	2,474,289 (2,403,712–2,533,502)	946,062 (673,545–1,224,829)	655,205 (614,399–696,084)	48,575 (35,294–63,288)	11,955 (10,779–13,208)	276,920 (233,120–332,312)	66,759 (47,609–86,666)	52,553 (47,968–57,568)	0 (0–0)	8,086 (6,407–10,161)	-
Poland	Prevalence Number: 2015	1,578,939 (1,526,813–1,626,335)	512,700 (364,598–662,237)	430,003 (404,523–456,064)	26,246 (18,728–34,828)	4,611 (4,253–4,970)	151,604 (125,007–192,442)	32,841 (22,846–44,192)	27,771 (25,487–30,393)	0 (0–0)	4,912 (3,928–6,040)	-
Poland	Prevalence Number: Cumulative change (%	-36.2 (-38.6 to -33.8)	-44.9 (-62.2 to -32.0)	-34.3 (-39.6 to -28.7)	-44.3 (-64.4 to -17.2)	-61.3 (-64.7 to -58.2)	-44.7 (-58.2 to -29.5)	-50.3 (-64.8 to -38.6)	-47.1 (-51.7 to -42.4)	-35.0 (-35.2 to -34.9)	-39.1 (-44.0 to -34.1)	-
Poland	Prevalence Rate: 1990	80,680.2 (78,361.5–82,612.0)	31,148.7 (22,201.5–40,167.8)	21,281.8 (19,959.5–22,614.8)	1,595.5 (1,159.2–2,078.6)	396.0 (358.3–437.4)	9,076.0 (7,640.8–10,885.2)	2,145.4 (1,530.0–2,785.1)	1,799.8 (1,642.7–1,969.5)	0.0 (0.0–0.0)	269.0 (213.0–337.1)	-
Poland	Prevalence Rate: 2015	79,002.8 (76,387.5–81,368.1)	25,904.0 (18,391.6–33,321.0)	21,463.0 (20,198.1–22,762.9)	1,317.9 (940.4–1,748.8)	232.1 (214.1–250.0)	7,608.2 (6,277.4–9,652.4)	1,630.1 (1,134.0–2,193.4)	1,428.5 (1,311.5–1,562.1)	0.0 (0.0–0.0)	248.5 (199.2–305.1)	-
Poland	Prevalence Rate: Cumulative change (%	-2.1 (-5.8 to 1.6)	-15.4 (-42.1 to 4.5)	1.0 (-7.2 to 9.4)	-14.8 (-45.5 to 26.6)	-41.3 (-46.2 to -36.6)	-15.3 (-36.0 to 7.9)	-23.2 (-45.6 to -5.2)	-20.5 (-27.4 to -13.6)	-0.0 (-0.0 to 0.0)	-7.4 (-14.8 to 0.3)	-
Poland	YLDs Number: 1990	101,497 (72,804–138,151)	26,615 (14,290–43,295)	16,817 (11,572–22,775)	6,086 (3,832–9,097)	1,975 (1,387–2,645)	1,687 (910–2,716)	3,064 (1,880–4,574)	3,641 (2,712–4,711)	0 (0–0)	1,596 (1,067–2,200)	384 (248–565)
Poland	YLDs Number: 2015	54,048 (38,838–72,418)	14,437 (7,670–23,059)	10,988 (7,597–14,933)	3,308 (2,021–4,982)	762 (541–1,038)	885 (474–1,400)	1,512 (907–2,315)	1,300 (954–1,708)	0 (0–0)	920 (623–1,280)	234 (159–331)

© 2017 Global Burden of Disease Child and Adolescent Health Collaboration. JAMA Pediatrics.

eTable 4. Prevalent cases, Rates (per 100,000 population), Years Lived with Disability (YLDs), and Cumulative Percent Change with 95% Uncertainty Interval (UI) for the Top 10 Global Causes of YLDsin Children and Adolescents in 195 Countries and Territories, Aged Under 5 Years, Both Sexes, 1990 and 2015. *Note: best viewed by enlarging in browser.*

Location	Measure	All causes	Iron-deficiency anemia	Skin and subcutaneous diseases	Protein-energy malnutrition	Diarrheal diseases	Hemoglobinopathies and hemolytic anemias	Asthma	Neonatal preterm birth complications	Malaria	Neonatal encephalopathy due to birth asphyxia and trauma	Other neonatal disorders
Poland	YLDs Number: Cumulative change (%)	-46.5 (-57.4 to -40.9)	-43.6 (-74.5 to -28.7)	-34.6 (-37.0 to -32.1)	-44.0 (-64.4 to -17.2)	-61.3 (-66.6 to -55.6)	-45.3 (-75.8 to -28.1)	-50.1 (-64.8 to -37.6)	-64.2 (-69.6 to -58.5)	-31.2 (-65.3 to 11.0)	-41.9 (-52.5 to -29.3)	-36.7 (-59.2 to -8.8)
Poland	YLDs Rate: 1990	3,323.1 (2,382.4–4,519.8)	878.8 (474.9–1,423.9)	544.6 (374.5–737.4)	199.9 (125.8–298.8)	65.4 (46.1–87.6)	55.7 (30.3–89.3)	98.5 (60.4–147.0)	119.4 (88.9–154.5)	0.0 (0.0–0.0)	52.3 (35.1–72.1)	12.6 (8.2–18.6)
Poland	YLDs Rate: 2015	2,714.2 (1,950.8–3,639.9)	732.5 (390.4–1,168.4)	547.6 (378.3–744.6)	166.1 (101.4–250.2)	38.4 (27.2–52.3)	44.9 (24.3–71.1)	75.1 (45.0–114.9)	65.2 (48.0–85.6)	0.0 (0.0–0.0)	46.2 (31.3–64.2)	11.8 (8.0–16.6)
Poland	YLDs Rate: Cumulative change (%)	-17.9 (-34.8 to -9.4)	-13.4 (-60.8 to 8.9)	0.6 (-3.0 to 4.4)	-14.5 (-45.6 to 26.8)	-41.1 (-49.1 to -32.7)	-16.1 (-62.8 to 9.8)	-22.9 (-45.6 to -3.5)	-45.2 (-53.4 to -36.7)	5.9 (-46.5 to 70.5)	-11.1 (-27.2 to 7.9)	-3.3 (-37.6 to 39.4)
Romania	Prevalence Number: 1990	1,400,890 (1,352,735–1,454,531)	661,497 (477,936–935,333)	369,206 (347,340–389,854)	25,634 (21,029–30,916)	11,218 (10,120–12,582)	128,578 (119,198–138,753)	26,638 (19,010–35,946)	31,539 (27,709–35,656)	0 (0–0)	4,520 (3,603–5,642)	-
Romania	Prevalence Number: 2015	760,382 (738,484–783,073)	373,474 (307,973–452,122)	201,839 (189,453–213,795)	13,220 (9,531–17,469)	3,459 (3,111–3,814)	62,879 (59,550–66,605)	15,458 (10,996–20,786)	16,116 (14,080–18,359)	0 (0–0)	2,370 (1,915–2,887)	-
Romania	Prevalence Number: Cumulative change (%	-45.7 (-48.2 to -43.0)	-40.6 (-64.4 to -6.9)	-45.3 (-49.3 to -41.0)	-47.7 (-64.6 to -29.2)	-69.1 (-71.6 to -66.9)	-51.0 (-55.5 to -46.3)	-41.7 (-50.1 to -32.0)	-48.8 (-53.8 to -43.5)	-45.4 (-45.5 to -45.3)	-47.4 (-51.1 to -43.4)	-
Romania	Prevalence Rate: 1990	81,423.2 (78,632.5–84,500.2)	38,898.2 (28,237.0–54,517.1)	21,337.2 (20,073.8–22,524.5)	1,499.0 (1,230.1–1,807.9)	664.3 (600.7–743.8)	7,507.5 (6,965.2–8,089.2)	1,522.5 (1,086.5–2,054.5)	1,934.1 (1,700.0–2,183.4)	0.0 (0.0–0.0)	268.1 (213.4–333.5)	-
Romania	Prevalence Rate: 2015	80,736.7 (78,426.9–83,155.1)	39,997.0 (33,043.6–48,278.1)	21,343.1 (20,037.5–22,604.5)	1,410.1 (1,016.5–1,863.3)	371.3 (334.1–408.8)	6,700.3 (6,344.8–7,097.0)	1,621.2 (1,153.2–2,179.9)	1,784.2 (1,558.9–2,031.7)	0.0 (0.0–0.0)	255.7 (207.2–312.1)	-
Romania	Prevalence Rate: Cumulative change (%	-0.8 (-5.4 to 4.2)	7.9 (-34.5 to 68.3)	0.1 (-7.3 to 7.8)	-4.7 (-35.3 to 29.1)	-44.0 (-48.6 to -40.1)	-10.6 (-18.7 to -2.1)	6.9 (-8.5 to 24.8)	-7.6 (-16.3 to 1.5)	0.0 (-0.0 to 0.0)	-4.4 (-11.2 to 3.0)	-
Romania	YLDs Number: 1990	68,840 (48,932–95,419)	22,169 (11,664–39,239)	9,586 (6,684–12,955)	3,209 (2,097–4,578)	1,847 (1,311–2,512)	985 (521–1,765)	1,218 (736–1,829)	2,015 (1,518–2,597)	0 (0–0)	904 (616–1,263)	2,235 (1,474–3,167)
Romania	YLDs Number: 2015	33,417 (24,216–44,168)	12,026 (7,475–17,859)	5,241 (3,599–7,089)	1,656 (993–2,449)	568 (399–765)	540 (343–799)	709 (429–1,054)	717 (531–937)	0 (0–0)	451 (309–618)	955 (632–1,341)
Romania	YLDs Number: Cumulative change (%)	-50.6 (-63.4 to -33.5)	-38.3 (-72.7 to 35.2)	-45.3 (-47.3 to -43.2)	-47.7 (-64.3 to -28.4)	-69.2 (-73.0 to -65.1)	-38.2 (-71.7 to 24.4)	-41.4 (-51.2 to -29.9)	-64.3 (-69.3 to -58.8)	-42.8 (-68.5 to -2.4)	-49.6 (-59.0 to -39.2)	-55.8 (-71.2 to -35.3)
Romania	YLDs Rate: 1990	4,026.3 (2,868.8–5,567.3)	1,310.6 (700.0–2,294.4)	552.2 (385.3–746.4)	187.7 (122.7–267.6)	109.4 (77.7–148.7)	58.3 (31.5–102.8)	69.6 (42.1–104.6)	117.7 (88.6–151.6)	0.0 (0.0–0.0)	52.7 (35.9–73.8)	130.7 (86.2–184.9)
Romania	YLDs Rate: 2015	3,565.5 (2,579.7–4,703.2)	1,294.7 (804.6–1,919.5)	552.9 (379.5–747.9)	176.6 (106.0–261.6)	61.0 (43.0–82.1)	58.1 (37.0–85.7)	74.4 (45.1–110.6)	76.4 (56.5–99.7)	0.0 (0.0–0.0)	48.1 (32.9–65.8)	101.9 (67.5–143.0)
Romania	YLDs Rate: Cumulative change (%)	-9.8 (-33.0 to 21.1)	11.7 (-49.8 to 141.0)	0.1 (-3.6 to 3.9)	-4.6 (-35.0 to 30.5)	-44.1 (-51.1 to -36.9)	11.7 (-48.0 to 122.8)	7.5 (-10.4 to 28.6)	-35.0 (-43.9 to -25.1)	4.7 (-42.2 to 77.5)	-8.1 (-24.9 to 10.7)	-19.5 (-47.5 to 18.0)
Serbia	Prevalence Number: 1990	569,315 (551,070–583,874)	149,712 (113,410–182,567)	157,787 (148,314–167,776)	17,343 (12,692–22,849)	3,492 (3,127–3,875)	65,307 (54,898–78,732)	18,369 (13,486–23,884)	13,127 (11,740–14,747)	0 (0–0)	1,907 (1,495–2,377)	-
Serbia	Prevalence Number: 2015	354,397 (343,761–363,383)	102,057 (76,309–129,762)	98,969 (93,269–104,668)	7,598 (6,071–9,427)	1,389 (1,248–1,559)	35,286 (29,816–44,176)	7,379 (5,351–9,592)	7,971 (7,066–9,014)	0 (0–0)	1,159 (942–1,414)	-
Serbia	Prevalence Number: Cumulative change (%	-37.7 (-40.0 to -35.4)	-29.9 (-51.5 to 6.8)	-37.2 (-41.9 to -32.5)	-54.7 (-69.2 to -35.4)	-60.2 (-63.6 to -57.1)	-45.3 (-57.4 to -29.6)	-59.7 (-65.2 to -52.7)	-39.2 (-44.9 to -33.2)	-37.4 (-37.4 to -37.3)	-39.0 (-43.7 to -33.9)	-
Serbia	Prevalence Rate: 1990	78,214.3 (75,717.9–80,210.4)	20,770.6 (15,749.0–25,348.2)	21,628.8 (20,333.3–22,997.3)	2,393.7 (1,751.8–3,153.5)	483.7 (433.7–535.9)	9,004.4 (7,572.0–10,856.1)	2,502.2 (1,837.1–3,253.5)	1,857.6 (1,656.7–2,083.8)	0.0 (0.0–0.0)	265.4 (208.1–330.3)	-
Serbia	Prevalence Rate: 2015	77,575.4 (75,249.5–79,541.3)	22,453.5 (16,830.0–28,482.8)	21,638.4 (20,391.8–22,879.5)	1,667.7 (1,332.6–2,068.9)	305.5 (274.2–342.8)	7,740.1 (6,542.9–9,690.2)	1,607.9 (1,166.0–2,090.1)	1,776.2 (1,574.2–2,007.9)	0.0 (0.0–0.0)	255.7 (207.8–311.3)	-
Serbia	Prevalence Rate: Cumulative change (%	-0.8 (-4.5 to 2.9)	11.2 (-22.8 to 68.2)	0.2 (-7.4 to 7.6)	-28.0 (-51.0 to 2.7)	-36.8 (-42.3 to -31.9)	-13.1 (-32.2 to 11.9)	-35.5 (-44.3 to -24.3)	-4.2 (-13.0 to 5.1)	-0.0 (-0.0 to 0.0)	-3.3 (-10.9 to 4.8)	-
Serbia	YLDs Number: 1990	21,886 (16,217–28,806)	3,725 (1,942–5,604)	4,219 (2,927–5,696)	2,182 (1,328–3,210)	577 (405–781)	306 (165–458)	845 (533–1,247)	936 (717–1,212)	0 (0–0)	382 (261–530)	180 (77–438)
Serbia	YLDs Number: 2015	12,008 (8,796–15,714)	2,615 (1,454–4,467)	2,644 (1,843–3,573)	957 (611–1,364)	230 (162–317)	207 (117–345)	339 (209–505)	391 (293–513)	0 (0–0)	223 (154–311)	77 (51–109)
Serbia	YLDs Number: Cumulative change (%)	-45.0 (-51.9 to -33.4)	-24.4 (-58.9 to 53.1)	-37.3 (-39.7 to -34.9)	-54.6 (-69.3 to -35.2)	-60.0 (-65.4 to -54.0)	-27.4 (-59.4 to 41.4)	-59.6 (-65.8 to -52.3)	-58.2 (-64.0 to -52.5)	-28.8 (-60.9 to 35.0)	-41.0 (-50.8 to -29.6)	-45.2 (-83.0 to 0.5)
Serbia	YLDs Rate: 1990	3,015.5 (2,232.3–3,968.5)	518.5 (271.1–778.2)	577.2 (400.4–779.7)	301.2 (183.3–443.2)	80.0 (56.2–108.3)	42.6 (23.1–63.6)	115.2 (72.7–169.9)	129.1 (99.1–167.1)	0.0 (0.0–0.0)	52.7 (36.1–73.0)	24.9 (10.7–60.5)
Serbia	YLDs Rate: 2015	2,632.7 (1,930.1–3,444.8)	576.2 (321.9–980.9)	577.5 (402.7–780.1)	210.2 (134.3–299.6)	50.7 (35.7–69.7)	45.6 (26.1–75.8)	74.0 (45.6–110.1)	85.8 (64.4–112.6)	0.0 (0.0–0.0)	49.1 (33.8–68.2)	17.0 (11.3–24.1)
Serbia	YLDs Rate: Cumulative change (%)	-12.4 (-23.4 to 6.0)	19.6 (-34.6 to 141.8)	0.1 (-3.6 to 3.9)	-27.9 (-51.2 to 3.1)	-36.5 (-45.0 to -26.9)	14.9 (-35.2 to 123.4)	-35.5 (-45.3 to -23.7)	-33.5 (-42.7 to -24.5)	13.6 (-37.5 to 115.8)	-6.2 (-21.7 to 11.9)	-12.9 (-73.0 to 59.7)
Slovakia	Prevalence Number: 1990	332,173 (320,395–343,084)	126,372 (84,483–181,577)	90,190 (84,571–95,180)	7,962 (5,851–10,547)	1,578 (1,436–1,741)	38,288 (32,236–48,085)	6,235 (4,605–8,034)	6,387 (5,822–6,986)	0 (0–0)	1,046 (833–1,318)	-
Slovakia	Prevalence Number: 2015	221,605 (213,719–229,301)	79,962 (62,697–94,696)	61,590 (58,174–65,175)	4,221 (3,109–5,548)	744 (683–833)	22,045 (18,424–26,775)	3,869 (2,767–5,199)	4,001 (3,628–4,415)	0 (0–0)	680 (548–851)	-
Slovakia	Prevalence Number: Cumulative change (%	-33.3 (-36.5 to -30.1)	-32.9 (-60.8 to 7.4)	-31.6 (-36.8 to -26.2)	-45.4 (-64.3 to -20.6)	-52.8 (-56.3 to -49.3)	-41.7 (-55.4 to -26.2)	-37.7 (-50.0 to -24.9)	-37.3 (-42.8 to -31.8)	-32.3 (-32.5 to -32.1)	-34.8 (-39.3 to -30.2)	-
Slovakia	Prevalence Rate: 1990	79,505.6 (76,722.9–82,098.5)	30,523.1 (20,642.7–43,624.5)	21,519.7 (20,180.9–22,710.3)	1,915.4 (1,407.7–2,537.4)	381.3 (347.5–420.3)	9,197.9 (7,745.2–11,547.1)	1,476.5 (1,090.4–1,902.3)	1,584.4 (1,444.8–1,731.9)	0.0 (0.0–0.0)	254.1 (202.1–319.3)	-
Slovakia	Prevalence Rate: 2015	78,028.0 (75,253.2–80,744.4)	28,234.7 (22,149.0–33,450.2)	21,668.5 (20,467.9–22,930.4)	1,488.3 (1,096.3–1,956.2)	262.8 (241.2–294.3)	7,770.4 (6,494.7–9,436.9)	1,358.7 (971.8–1,825.8)	1,422.1 (1,290.3–1,568.3)	0.0 (0.0–0.0)	240.7 (194.0–300.8)	-
Slovakia	Prevalence Rate: Cumulative change (%	-1.8 (-6.5 to 2.8)	-2.1 (-42.5 to 54.6)	0.8 (-6.8 to 8.8)	-20.0 (-47.7 to 16.3)	-31.0 (-36.0 to -25.9)	-14.5 (-34.6 to 8.3)	-7.6 (-25.8 to 11.3)	-10.1 (-17.9 to -2.2)	-0.0 (-0.0 to 0.0)	-5.1 (-11.9 to 1.5)	-
Slovakia	YLDs Number: 1990	13,196 (9,299–18,031)	3,726 (1,654–6,429)	2,354 (1,624–3,192)	1,000 (619–1,501)	260 (187–351)	230 (102–393)	286 (180–426)	401 (304–528)	0 (0–0)	211 (144–299)	132 (87–189)
Slovakia	YLDs Number: 2015	7,907 (5,831–10,587)	2,236 (1,309–3,327)	1,598 (1,108–2,157)	532 (320–802)	123 (86–166)	133 (77–201)	178 (107–263)	198 (147–262)	0 (0–0)	129 (88–178)	34 (22–50)
Slovakia	YLDs Number: Cumulative change (%)	-39.3 (-55.2 to -27.9)	-32.5 (-73.1 to 8.9)	-32.1 (-34.5 to -29.5)	-45.3 (-64.2 to -19.8)	-52.5 (-59.0 to -45.2)	-34.8 (-73.7 to 6.6)	-37.4 (-50.7 to -22.7)	-50.6 (-57.7 to -43.3)	-18.3 (-62.2 to 73.6)	-38.5 (-49.4 to -26.1)	-73.2 (-83.3 to -58.2)
Slovakia	YLDs Rate: 1990	3,169.2 (2,236.7–4,330.8)	902.1 (407.0–1,545.5)	560.4 (386.4–759.6)	240.7 (148.9–360.8)	63.0 (45.3–84.8)	55.8 (25.2–94.9)	67.8 (42.8–100.9)	96.5 (73.1–126.8)	0.0 (0.0–0.0)	50.8 (34.5–71.8)	31.8 (21.2–45.6)
Slovakia	YLDs Rate: 2015	2,787.3 (2,055.3–3,732.6)	790.7 (463.6–1,178.0)	562.1 (389.8–758.8)	187.7 (113.2–283.0)	43.6 (30.4–58.9)	47.4 (27.5–71.4)	62.6 (37.9–92.5)	69.8 (52.0–92.6)	0.0 (0.0–0.0)	45.6 (31.2–63.0)	12.0 (7.9–17.7)
Slovakia	YLDs Rate: Cumulative change (%)	-11.0 (-34.2 to 5.6)	-2.0 (-60.4 to 56.5)	0.3 (-3.3 to 4.1)	-19.8 (-47.5 to 17.5)	-30.6 (-40.0 to -20.0)	-5.4 (-61.3 to 53.6)	-7.1 (-26.9 to 14.6)	-27.4 (-37.9 to -16.8)	20.4 (-44.2 to 155.8)	-9.7 (-25.7 to 8.3)	-60.8 (-75.5 to -38.7)
Slovenia	Prevalence Number: 1990	92,709 (89,130–96,290)	35,345 (26,054–49,058)	26,993 (25,336–28,597)	2,385 (1,776–3,176)	381 (343–425)	11,275 (9,357–14,245)	1,265 (927–1,653)	1,758 (1,596–1,930)	0 (0–0)	311 (250–389)	-
Slovenia	Prevalence Number: 2015	80,816 (77,023–84,107)	35,084 (24,131–47,594)	23,346 (21,863–24,573)	1,555 (1,145–2,053)	235 (216–253)	8,509 (7,183–10,535)	1,206 (846–1,621)	1,503 (1,378–1,636)	0 (0–0)	268 (212–333)	-
Slovenia	Prevalence Number: Cumulative change (%	-12.8 (-17.6 to -7.9)	3.2 (-40.6 to 52.2)	-13.4 (-20.2 to -6.4)	-32.7 (-56.4 to 0.3)	-38.2 (-42.7 to -33.6)	-23.6 (-41.2 to -1.9)	-4.4 (-21.2 to 13.2)	-14.4 (-21.8 to -5.9)	-14.2 (-14.7 to -13.8)	-13.8 (-21.3 to -7.7)	-
Slovenia	Prevalence Rate: 1990	75,133.4 (72,217.6–78,028.3)	29,155.8 (21,663.0–40,173.3)	21,734.9 (20,398.6–23,015.3)	1,945.6 (1,448.4–2,590.5)	311.6 (281.0–347.8)	9,181.1 (7,626.4–11,591.9)	1,006.4 (737.6–1,315.4)	1,504.8 (1,370.3–1,646.0)	0.0 (0.0–0.0)	258.1 (207.6–320.6)	-

© 2017 Global Burden of Disease Child and Adolescent Health Collaboration. JAMA Pediatrics.

eTable 4. Prevalent cases, Rates (per 100,000 population), Years Lived with Disability (YLDs), and Cumulative Percent Change with 95% Uncertainty Interval (UI) for the Top 10 Global Causes of YLDsin Children and Adolescents in 195 Countries and Territories, Aged Under 5 Years, Both Sexes, 1990 and 2015. *Note: best viewed by enlarging in browser.*

Location	Measure	All causes	Iron-deficiency anemia	Skin and subcutaneous diseases	Protein-energy malnutrition	Diarrheal diseases	Hemoglobinopathies and hemolytic anemias	Asthma	Neonatal preterm birth complications	Malaria	Neonatal encephalopathy due to birth asphyxia and trauma	Other neonatal disorders
Slovenia	Prevalence Rate: 2015	75,639.1 (72,088.3–78,718.7)	32,876.1 (22,629.0–44,570.0)	21,838.3 (20,451.9–22,984.7)	1,456.4 (1,072.7–1,923.4)	220.4 (202.8–237.5)	7,968.2 (6,725.4–9,864.6)	1,127.7 (790.7–1,514.9)	1,414.1 (1,297.0–1,539.7)	0.0 (0.0–0.0)	251.7 (199.3–312.7)	-
Slovenia	Prevalence Rate: Cumulative change (%	0.7 (-4.8 to 6.4)	17.0 (-32.0 to 69.7)	0.6 (-7.3 to 8.8)	-22.7 (-49.9 to 15.2)	-29.1 (-34.3 to -24.0)	-12.2 (-32.3 to 12.8)	12.3 (-7.4 to 32.9)	-5.9 (-13.6 to 3.3)	-0.0 (-0.0 to 0.0)	-2.3 (-10.8 to 4.5)	-
Slovenia	YLDs Number: 1990	4,391 (3,089–6,124)	1,029 (561–1,829)	700 (486–948)	299 (187–438)	62 (44–85)	62 (34–109)	58 (35–87)	115 (85–153)	0 (0–0)	64 (43–89)	13 (8–18)
Slovenia	YLDs Number: 2015	3,414 (2,378–4,647)	1,052 (533–1,752)	601 (414–816)	195 (118–291)	38 (27–51)	63 (32–105)	55 (33–83)	70 (52–91)	0 (0–0)	51 (34–71)	28 (18–40)
Slovenia	YLDs Number: Cumulative change (%)	-21.1 (-43.2 to -1.6)	10.5 (-58.0 to 70.3)	-14.1 (-17.3 to -10.8)	-32.6 (-56.7 to 0.8)	-37.9 (-47.6 to -27.6)	10.9 (-57.4 to 76.3)	-4.1 (-23.6 to 17.0)	-38.8 (-48.1 to -29.0)	-0.2 (-51.7 to 56.9)	-18.8 (-33.1 to -3.8)	124.2 (49.8–211.4)
Slovenia	YLDs Rate: 1990	3,582.1 (2,520.9–5,008.0)	856.3 (467.7–1,508.5)	561.9 (390.4–760.5)	244.2 (152.9–357.4)	51.5 (36.2–69.5)	52.1 (28.4–90.8)	46.2 (28.2–69.5)	93.5 (69.1–124.6)	0.0 (0.0–0.0)	52.0 (35.3–72.8)	10.7 (7.3–14.8)
Slovenia	YLDs Rate: 2015	3,196.9 (2,227.4–4,353.3)	986.6 (500.7–1,641.2)	562.6 (387.7–763.2)	183.1 (111.2–272.7)	36.4 (25.8–48.5)	59.9 (30.5–98.7)	51.8 (31.4–78.1)	65.7 (48.8–86.0)	0.0 (0.0–0.0)	48.3 (32.6–67.2)	26.8 (17.4–37.6)
Slovenia	YLDs Rate: Cumulative change (%)	-9.5 (-34.6 to 13.0)	24.0 (-52.1 to 90.4)	0.1 (-3.6 to 4.0)	-22.6 (-50.1 to 15.8)	-28.8 (-39.8 to -16.9)	24.5 (-51.4 to 96.0)	12.6 (-10.2 to 37.4)	-29.5 (-40.2 to -18.2)	16.0 (-43.7 to 82.8)	-6.4 (-22.8 to 10.7)	157.8 (72.5–257.5)
Central Asia	Prevalence Number: 1990	7,948,669 (7,859,015–8,034,250)	3,402,210 (3,145,244–3,672,295)	1,963,397 (1,904,654–2,020,232)	307,067 (228,551–405,737)	306,275 (271,155–343,331)	893,778 (870,795–917,681)	156,316 (119,227–200,094)	280,761 (254,776–306,749)	978 (459–1,600)	29,882 (22,998–38,092)	-
Central Asia	Prevalence Number: 2015	7,740,069 (7,630,178–7,848,959)	3,122,223 (2,806,601–3,477,970)	1,951,310 (1,891,214–2,009,539)	200,761 (151,798–263,469)	136,300 (122,867–150,694)	834,103 (809,948–856,404)	138,082 (100,686–181,461)	278,075 (253,540–303,949)	5 (1–19)	29,343 (22,875–36,850)	-
Central Asia	Prevalence Number: Cumulative change (%	-2.6 (-4.2 to -1.0)	-8.0 (-18.8 to 4.8)	-0.6 (-4.1 to 2.8)	-32.6 (-56.4 to -3.7)	-55.4 (-59.8 to -50.8)	-6.7 (-9.9 to -3.5)	-11.8 (-21.8 to -1.9)	-0.9 (-5.3 to 3.7)	-99.4 (-99.9 to -97.7)	-1.7 (-6.2 to 3.3)	-
Central Asia	Prevalence Rate: 1990	84,427.8 (83,475.6–85,337.9)	36,084.2 (33,351.9–38,967.8)	20,871.8 (20,247.7–21,476.6)	3,259.2 (2,425.8–4,306.5)	3,249.2 (2,876.0–3,642.7)	9,488.7 (9,244.3–9,742.5)	1,664.3 (1,269.4–2,130.4)	2,961.6 (2,686.9–3,234.1)	10.4 (4.9–17.0)	316.5 (243.6–403.6)	-
Central Asia	Prevalence Rate: 2015	82,916.7 (81,739.4–84,083.1)	33,446.2 (30,065.0–37,257.5)	20,903.2 (20,259.4–21,527.1)	2,150.7 (1,626.2–2,822.5)	1,460.1 (1,316.2–1,614.3)	8,935.4 (8,676.6–9,174.3)	1,479.4 (1,078.7–1,944.1)	2,978.3 (2,715.5–3,255.5)	0.1 (0.0–0.2)	314.3 (245.0–394.7)	-
Central Asia	Prevalence Rate: Cumulative change (%	-1.8 (-3.3 to -0.2)	-7.1 (-18.0 to 5.9)	0.2 (-3.3 to 3.6)	-32.0 (-56.0 to -2.8)	-55.0 (-59.4 to -50.3)	-5.8 (-9.1 to -2.6)	-11.2 (-21.4 to -1.3)	0.6 (-3.9 to 5.3)	-99.4 (-99.9 to -97.7)	-0.5 (-5.1 to 4.6)	-
Central Asia	YLDs Number: 1990	466,838 (345,029–608,884)	113,603 (79,816–155,766)	49,860 (34,593–67,483)	38,310 (23,991–57,075)	50,249 (35,837–67,332)	9,745 (6,893–13,326)	7,139 (4,574–10,410)	18,454 (14,231–23,451)	20 (8–37)	5,820 (3,924–8,092)	2,972 (1,725–4,790)
Central Asia	YLDs Number: 2015	388,757 (286,936–505,290)	100,186 (67,966–139,540)	49,634 (34,362–66,981)	25,106 (15,794–36,683)	22,424 (15,882–29,874)	9,171 (6,331–12,838)	6,335 (3,957–9,384)	16,152 (12,288–20,709)	0 (0–0)	5,566 (3,757–7,744)	3,374 (2,083–5,351)
Central Asia	YLDs Number: Cumulative change (%)	-16.4 (-27.9 to -4.8)	-11.5 (-26.4 to 8.0)	-0.4 (-2.2 to 1.3)	-32.4 (-56.3 to -3.4)	-55.3 (-59.8 to -50.6)	-5.6 (-20.1 to 13.4)	-11.3 (-22.3 to -0.9)	-12.5 (-17.6 to -7.1)	-99.2 (-99.9 to -96.8)	-4.1 (-12.2 to 4.7)	22.9 (-37.5 to 114.1)
Central Asia	YLDs Rate: 1990	4,954.3 (3,662.1–6,463.1)	1,203.9 (846.1–1,650.5)	530.3 (367.9–717.6)	406.6 (254.6–605.8)	533.1 (380.2–714.3)	103.3 (73.1–141.3)	76.0 (48.7–110.8)	195.8 (151.0–248.8)	0.2 (0.1–0.4)	61.8 (41.6–85.9)	31.5 (18.3–50.8)
Central Asia	YLDs Rate: 2015	4,164.6 (3,073.8–5,412.9)	1,073.2 (728.0–1,494.8)	531.7 (368.1–717.6)	269.0 (169.2–393.0)	240.2 (170.1–320.0)	98.2 (67.8–137.5)	67.9 (42.4–100.5)	173.0 (131.6–221.9)	0.0 (0.0–0.0)	59.6 (40.3–83.0)	36.2 (22.3–57.3)
Central Asia	YLDs Rate: Cumulative change (%)	-15.7 (-27.2 to -3.9)	-10.5 (-25.6 to 9.2)	0.3 (-1.5 to 2.1)	-31.8 (-55.9 to -2.5)	-54.8 (-59.4 to -50.1)	-4.5 (-19.3 to 14.7)	-10.8 (-21.8 to -0.3)	-11.6 (-16.8 to -6.2)	-99.2 (-99.9 to -96.8)	-3.2 (-11.4 to 5.7)	24.1 (-36.8 to 116.2)
Armenia	Prevalence Number: 1990	303,161 (294,315–312,387)	96,952 (69,363–132,878)	78,372 (74,008–83,043)	6,879 (3,228–11,813)	12,011 (10,854–13,478)	33,291 (31,185–35,528)	5,716 (4,093–7,603)	11,615 (10,499–12,972)	126 (24–259)	1,237 (941–1,594)	-
Armenia	Prevalence Number: 2015	158,498 (153,373–163,487)	51,041 (40,809–62,475)	41,345 (38,934–43,781)	3,312 (1,744–5,578)	2,896 (2,581–3,259)	16,193 (15,130–17,353)	2,819 (2,009–3,785)	5,498 (4,970–6,107)	0 (0–0)	616 (484–770)	-
Armenia	Prevalence Number: Cumulative change (%	-47.7 (-49.9 to -45.5)	-44.3 (-67.1 to -12.6)	-47.2 (-51.1 to -43.1)	-44.1 (-78.5 to 17.4)	-75.8 (-78.5 to -73.2)	-51.3 (-55.7 to -46.8)	-50.6 (-55.4 to -46.0)	-52.6 (-56.7 to -48.4)	-99.0 (-100.0 to -100.0)	-50.0 (-54.4 to -45.1)	-
Armenia	Prevalence Rate: 1990	80,808.2 (78,443.1–83,273.7)	26,010.1 (18,679.2–35,518.5)	20,842.3 (19,684.4–22,082.7)	1,838.4 (862.7–3,156.8)	3,216.5 (2,905.8–3,608.1)	8,890.5 (8,325.7–9,486.7)	1,513.3 (1,083.6–2,012.9)	3,160.2 (2,859.4–3,526.1)	33.5 (6.6–69.1)	332.6 (253.1–428.5)	-
Armenia	Prevalence Rate: 2015	80,079.9 (77,501.2–82,607.9)	25,973.1 (20,843.9–31,755.7)	20,844.8 (19,632.3–22,070.0)	1,677.2 (883.5–2,824.5)	1,470.9 (1,311.9–1,654.6)	8,197.0 (7,659.4–8,784.4)	1,415.4 (1,008.8–1,900.1)	2,832.3 (2,562.1–3,145.0)	0.0 (0.0–0.0)	313.8 (247.0–391.8)	-
Armenia	Prevalence Rate: Cumulative change (%	-0.9 (-5.1 to 3.4)	5.5 (-37.3 to 65.2)	0.1 (-7.2 to 7.8)	6.0 (-59.3 to 122.5)	-54.2 (-59.3 to -49.1)	-7.7 (-15.9 to 0.8)	-6.3 (-15.4 to 2.5)	-10.2 (-18.0 to -2.4)	-98.1 (-100.0 to -100.0)	-5.2 (-13.6 to 4.0)	-
Armenia	YLDs Number: 1990	16,720 (12,376–21,809)	2,871 (1,499–4,901)	1,992 (1,383–2,697)	859 (353–1,578)	1,978 (1,412–2,649)	285 (153–479)	261 (160–392)	852 (645–1,088)	2 (0–5)	245 (160–354)	283 (85–611)
Armenia	YLDs Number: 2015	6,899 (5,010–9,041)	1,485 (893–2,246)	1,051 (719–1,430)	416 (195–746)	479 (338–642)	147 (89–221)	129 (78–196)	325 (245–421)	0 (0–0)	118 (78–168)	143 (56–285)
Armenia	YLDs Number: Cumulative change (%)	-58.5 (-66.6 to -49.7)	-42.0 (-73.7 to 5.0)	-47.2 (-49.3 to -45.1)	-43.8 (-78.3 to 19.4)	-75.7 (-78.8 to -72.7)	-42.6 (-73.6 to -1.0)	-50.3 (-56.3 to -43.4)	-61.8 (-66.1 to -57.1)	-98.9 (-100.0 to -100.0)	-51.3 (-59.7 to -41.6)	-29.4 (-84.5 to 82.3)
Armenia	YLDs Rate: 1990	4,466.8 (3,309.4–5,833.9)	772.5 (404.5–1,312.1)	529.3 (367.4–716.7)	229.7 (94.3–421.9)	529.7 (377.9–708.7)	76.7 (41.3–129.6)	69.2 (42.5–103.9)	227.9 (172.6–290.9)	0.7 (0.1–1.6)	65.7 (42.7–94.9)	75.9 (23.0–163.6)
Armenia	YLDs Rate: 2015	3,496.2 (2,540.3–4,584.5)	758.6 (456.6–1,145.7)	529.6 (362.5–720.6)	210.8 (99.2–378.2)	243.2 (172.2–326.2)	75.1 (45.7–112.9)	65.0 (39.6–98.7)	164.8 (124.2–213.6)	0.0 (0.0–0.0)	60.0 (39.8–85.5)	72.7 (28.5–144.6)
Armenia	YLDs Rate: Cumulative change (%)	-21.2 (-36.6 to -4.4)	9.9 (-49.5 to 99.7)	0.1 (-3.9 to 4.1)	6.5 (-58.8 to 126.2)	-54.0 (-59.8 to -48.3)	8.7 (-49.4 to 88.1)	-5.8 (-17.1 to 7.3)	-27.6 (-35.8 to -18.9)	-97.9 (-100.0 to -100.0)	-7.7 (-23.6 to 10.5)	33.8 (-70.7 to 245.3)
Azerbaijan	Prevalence Number: 1990	730,824 (709,610–750,682)	294,000 (219,747–369,711)	182,937 (172,208–193,237)	19,599 (10,108–33,781)	24,677 (22,398–27,258)	96,075 (91,686–100,614)	11,459 (8,300–14,976)	21,233 (18,703–23,875)	133 (33–281)	2,629 (1,983–3,388)	-
Azerbaijan	Prevalence Number: 2015	790,023 (767,529–808,774)	324,568 (260,791–362,034)	200,970 (188,539–213,814)	20,365 (10,195–35,747)	14,836 (13,015–16,761)	98,067 (94,167–102,236)	14,268 (10,185–19,382)	21,083 (18,621–23,747)	0 (0–0)	2,849 (2,202–3,616)	-
Azerbaijan	Prevalence Number: Cumulative change (%	8.1 (4.4–12.1)	13.6 (-17.8 to 58.0)	10.0 (1.5–19.3)	19.6 (-57.7 to 151.9)	-39.9 (-45.3 to -34.6)	2.1 (-3.7 to 8.3)	24.9 (2.8–51.2)	-0.5 (-10.2 to 9.1)	-98.6 (-100.0 to -100.0)	8.8 (-3.4 to 21.6)	-
Azerbaijan	Prevalence Rate: 1990	83,289.0 (80,860.4–85,557.7)	33,238.8 (24,710.8–41,903.5)	20,935.7 (19,697.8–22,115.2)	2,225.4 (1,147.8–3,835.6)	2,790.6 (2,529.3–3,086.4)	10,922.6 (10,421.5–11,440.4)	1,321.5 (957.2–1,727.1)	2,336.1 (2,055.3–2,631.6)	15.3 (3.8–32.3)	295.2 (222.6–381.7)	-
Azerbaijan	Prevalence Rate: 2015	82,347.9 (80,016.4–84,297.5)	33,895.2 (27,232.8–37,790.3)	20,926.0 (19,634.5–22,263.3)	2,125.4 (1,064.0–3,730.8)	1,551.4 (1,361.1–1,752.1)	10,230.2 (9,823.7–10,664.8)	1,482.7 (1,058.4–2,014.0)	2,219.4 (1,959.8–2,498.7)	0.0 (0.0–0.0)	298.3 (230.6–378.3)	-
Azerbaijan	Prevalence Rate: Cumulative change (%	-1.1 (-4.6 to 2.5)	5.0 (-24.2 to 46.3)	0.1 (-7.6 to 8.6)	9.9 (-61.1 to 131.6)	-44.4 (-49.5 to -39.5)	-6.3 (-11.7 to -0.6)	12.5 (-7.4 to 36.3)	-4.8 (-14.3 to 4.4)	-98.7 (-100.0 to -100.0)	1.5 (-9.8 to 13.2)	-
Azerbaijan	YLDs Number: 1990	37,233 (26,752–49,308)	9,479 (5,208–14,675)	4,635 (3,199–6,294)	2,450 (1,097–4,505)	4,054 (2,893–5,438)	1,078 (596–1,665)	524 (321–784)	1,326 (1,004–1,697)	2 (0–6)	511 (332–736)	56 (18–121)
Azerbaijan	YLDs Number: 2015	36,303 (26,580–48,407)	10,046 (6,135–14,435)	5,078 (3,493–6,892)	2,549 (1,149–4,778)	2,445 (1,724–3,273)	1,234 (772–1,761)	655 (398–978)	1,310 (968–1,709)	0 (0–0)	544 (364–775)	63 (21–151)
Azerbaijan	YLDs Number: Cumulative change (%)	-1.8 (-17.7 to 16.8)	13.0 (-34.9 to 85.9)	9.6 (5.5–13.6)	20.0 (-58.5 to 157.3)	-39.7 (-45.9 to -33.9)	22.0 (-24.6 to 96.9)	25.5 (1.5–56.0)	-1.0 (-13.0 to 11.2)	-98.7 (-100.0 to -100.0)	7.2 (-11.9 to 29.0)	53.9 (-67.0 to 296.9)
Azerbaijan	YLDs Rate: 1990	4,222.4 (3,031.1–5,591.0)	1,067.3 (582.8–1,650.9)	531.6 (366.8–721.6)	278.2 (124.5–511.6)	458.6 (327.2–614.7)	121.5 (66.8–187.7)	60.5 (37.1–90.5)	149.9 (113.4–192.0)	0.3 (0.0–0.7)	58.1 (37.8–83.7)	6.4 (2.1–13.7)
Azerbaijan	YLDs Rate: 2015	3,788.4 (2,773.9–5,052.2)	1,050.1 (641.9–1,508.9)	528.5 (363.5–717.4)	266.0 (120.0–498.8)	255.7 (180.3–342.3)	129.1 (80.8–184.0)	68.1 (41.4–101.7)	136.8 (101.1–178.4)	0.0 (0.0–0.0)	56.8 (38.0–80.9)	6.7 (2.2–15.8)
Azerbaijan	YLDs Rate: Cumulative change (%)	-9.6 (-24.3 to 7.6)	5.1 (-39.6 to 73.8)	-0.6 (-4.2 to 3.1)	10.3 (-61.9 to 136.4)	-44.2 (-49.9 to -39.0)	13.4 (-30.1 to 83.8)	13.1 (-8.6 to 40.6)	-8.6 (-19.8 to 2.9)	-98.9 (-100.0 to -100.0)	-1.4 (-19.0 to 18.5)	42.2 (-69.5 to 266.7)

© 2017 Global Burden of Disease Child and Adolescent Health Collaboration. JAMA Pediatrics.

eTable 4. Prevalent cases, Rates (per 100,000 population), Years Lived with Disability (YLDs), and Cumulative Percent Change with 95% Uncertainty Interval (UI) for the Top 10 Global Causes of YLDsin Children and Adolescents in 195 Countries and Territories, Aged Under 5 Years, Both Sexes, 1990 and 2015. *Note: best viewed by enlarging in browser.*

Location	Measure	All causes	Iron-deficiency anemia	Skin and subcutaneous diseases	Protein-energy malnutrition	Diarrheal diseases	Hemoglobinopathies and hemolytic anemias	Asthma	Neonatal preterm birth complications	Malaria	Neonatal encephalopathy due to birth asphyxia and trauma	Other neonatal disorders
Georgia	Prevalence Number: 1990	376,269 (366,190–386,692)	142,158 (110,898–171,382)	94,673 (89,040–100,606)	9,032 (4,486–15,759)	9,193 (8,224–10,208)	35,055 (33,291–36,963)	12,687 (10,017–15,951)	12,266 (11,133–13,586)	120 (23–253)	1,453 (1,118–1,856)	-
Georgia	Prevalence Number: 2015	224,934 (217,764–232,288)	83,340 (64,835–107,035)	57,453 (53,876–61,333)	3,032 (1,539–4,962)	3,373 (3,040–3,780)	19,475 (18,295–20,818)	5,975 (4,242–7,948)	7,868 (7,073–8,691)	0 (0–0)	880 (693–1,105)	-
Georgia	Prevalence Number: Cumulative change (%	-40.2 (-42.7 to -37.8)	-40.1 (-61.1 to -15.8)	-39.2 (-44.3 to -34.3)	-60.9 (-85.8 to -19.0)	-63.2 (-66.6 to -59.9)	-44.4 (-48.7 to -39.5)	-52.7 (-63.3 to -39.9)	-35.8 (-41.3 to -28.9)	-99.3 (-100.0 to -100.0)	-39.2 (-44.0 to -34.0)	-
Georgia	Prevalence Rate: 1990	81,861.1 (79,672.4–84,122.1)	31,138.5 (24,391.2–37,468.8)	20,544.1 (19,319.9–21,832.7)	1,970.6 (978.9–3,438.3)	2,009.5 (1,798.0–2,229.9)	7,643.5 (7,260.0–8,058.6)	2,738.1 (2,162.0–3,442.5)	2,728.8 (2,477.2–3,017.9)	26.2 (5.0–55.0)	319.2 (245.9–407.3)	-
Georgia	Prevalence Rate: 2015	80,380.9 (77,853.0–82,966.4)	30,029.5 (23,471.2–38,436.4)	20,468.8 (19,196.0–21,843.1)	1,088.0 (552.4–1,780.4)	1,215.9 (1,095.8–1,362.6)	6,981.1 (6,558.8–7,461.2)	2,113.9 (1,500.9–2,811.8)	2,900.6 (2,609.1–3,198.8)	0.0 (0.0–0.0)	318.8 (251.7–399.2)	-
Georgia	Prevalence Rate: Cumulative change (%	-1.8 (-5.8 to 2.1)	-1.6 (-35.5 to 38.2)	-0.2 (-8.5 to 7.9)	-35.7 (-76.7 to 33.1)	-39.4 (-44.9 to -33.9)	-8.6 (-15.7 to -0.5)	-22.5 (-39.9 to -1.5)	6.5 (-2.7 to 17.5)	-98.9 (-100.0 to -100.0)	0.2 (-7.9 to 9.0)	-
[truncated: 649,445 more chars]
